# Extreme Wildlife Declines and Concurrent Increase in Livestock Numbers in Kenya: What Are the Causes?

**DOI:** 10.1371/journal.pone.0163249

**Published:** 2016-09-27

**Authors:** Joseph O. Ogutu, Hans-Peter Piepho, Mohamed Y. Said, Gordon O. Ojwang, Lucy W. Njino, Shem C. Kifugo, Patrick W. Wargute

**Affiliations:** 1 University of Hohenheim, Institute for Crop Science-340, 70599, Stuttgart, Germany; 2 International Livestock Research Institute, P.O. Box 30709–00100, Nairobi, Kenya; 3 Directorate of Resource Surveys and Remote Sensing, P.O. Box 47146–00100, Nairobi, Kenya; 4 Kenya Market Trust, 14 Riverside, Cavendish Block 3rd Floor, Suite B, Riverside Drive P.O. Box 44817–00100, Nairobi, Kenya; 5 Center for Sustainable Drylands Ecosystems and Societies, University of Nairobi, P.O. Box 30197, 00100, Nairobi, Kenya; 6 Northern Rangelands Trust, Private Bag, Isiolo, 60300, Kenya; Embrapa, BRAZIL

## Abstract

There is growing evidence of escalating wildlife losses worldwide. Extreme wildlife losses have recently been documented for large parts of Africa, including western, Central and Eastern Africa. Here, we report extreme declines in wildlife and contemporaneous increase in livestock numbers in Kenya rangelands between 1977 and 2016. Our analysis uses systematic aerial monitoring survey data collected in rangelands that collectively cover 88% of Kenya’s land surface. Our results show that wildlife numbers declined on average by 68% between 1977 and 2016. The magnitude of decline varied among species but was most extreme (72–88%) and now severely threatens the population viability and persistence of warthog, lesser kudu, Thomson’s gazelle, eland, oryx, topi, hartebeest, impala, Grevy’s zebra and waterbuck in Kenya’s rangelands. The declines were widespread and occurred in most of the 21 rangeland counties. Likewise to wildlife, cattle numbers decreased (25.2%) but numbers of sheep and goats (76.3%), camels (13.1%) and donkeys (6.7%) evidently increased in the same period. As a result, livestock biomass was 8.1 times greater than that of wildlife in 2011–2013 compared to 3.5 times in 1977–1980. Most of Kenya’s wildlife (*ca*. 30%) occurred in Narok County alone. The proportion of the total “national” wildlife population found in each county increased between 1977 and 2016 substantially only in Taita Taveta and Laikipia but marginally in Garissa and Wajir counties, largely reflecting greater wildlife losses elsewhere. The declines raise very grave concerns about the future of wildlife, the effectiveness of wildlife conservation policies, strategies and practices in Kenya. Causes of the wildlife declines include exponential human population growth, increasing livestock numbers, declining rainfall and a striking rise in temperatures but the fundamental cause seems to be policy, institutional and market failures. Accordingly, we thoroughly evaluate wildlife conservation policy in Kenya. We suggest policy, institutional and management interventions likely to succeed in reducing the declines and restoring rangeland health, most notably through strengthening and investing in community and private wildlife conservancies in the rangelands.

## Introduction

There is mounting evidence of widespread and catastrophic recent declines in the numbers and range of many wildlife populations worldwide [1] especially in Africa [2,3]. The magnitude and extent of these declines as well as their suggested underlying drivers vary widely regionally. The extreme declines in wildlife numbers in Africa have become widely recognized and documented in recent years for South Africa [4,5], West Africa [6–9], Central Africa [10,11] and East Africa [12–22]. The declines occur both inside and outside protected areas and have been variously attributed to rapid human population growth, land use and cover changes, land fragmentation, infrastructural development, poaching for trophy and bushmeat, climate change and variability, outbreaks of infectious diseases, proliferation of firearms, weak law enforcement, poor governance, competition with livestock for space, water and pasture, poverty and inequality [3,8–11,16,17,21–24]. Rapid human population growth is driving wildlife population declines in Africa through its influence on expansion of agriculture, settlements and development of infrastructure. Deterioration in wildlife and livestock habitats caused by major land use and cover changes is exacerbated by climate change and variability, piling enormous pressures on pastoralism, ranching and wildlife conservation in African rangelands and protected areas [19,20,25].

**Fig 1:**
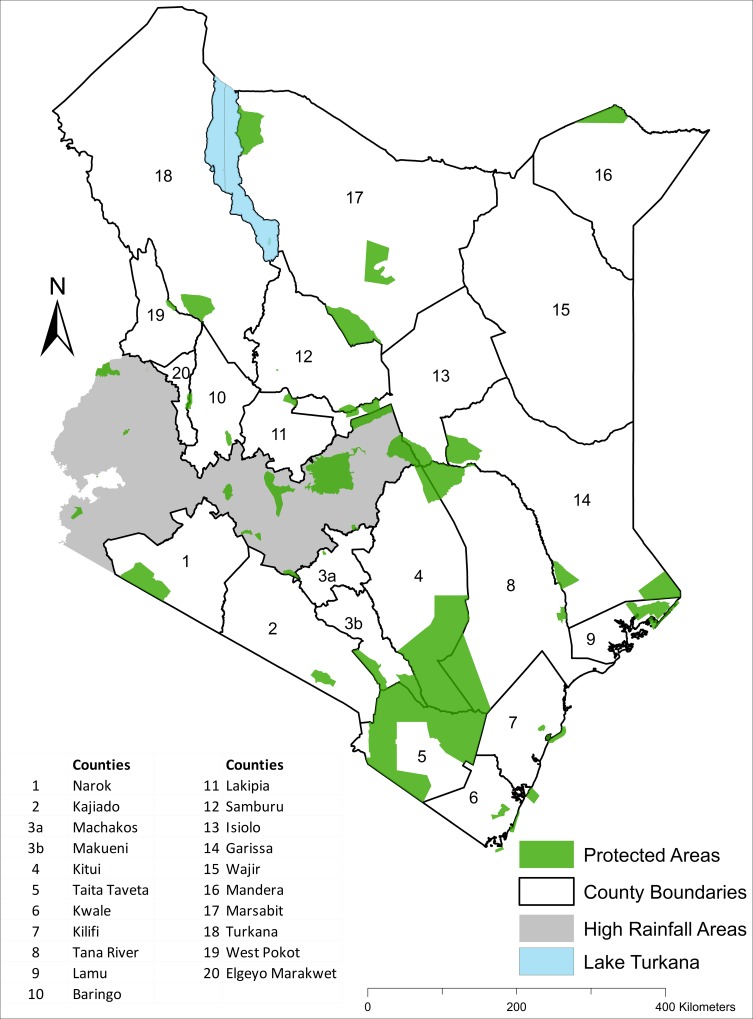
Map of Kenya showing the 21 rangeland counties in which the Directorate of Resource Surveys and Remote Sensing (DRSRS) conducts aerial surveys. **Note that Machakos and Makueni Counties are treated as one unit during the surveys**. Protected national parks and reserves are shaded green.

Rangelands cover about 512586.8 km^2^, representing 88% of the 582,646 km^2^ land surface of Kenya (Fig 1). They are hot, semiarid or arid with highly variable rainfall, often averaging less than 600 mm per year and thus are drought-prone and less suitable for sustainable crop production. The rangelands are currently home to 32.6% of the Kenyan population (12,582,028 of 38,610,097 people in 2009), principally pastoral communities and are crucially important for extensive livestock production and wildlife conservation in Kenya. More than half of the Kenyan livestock populations are found on these rangelands. The livestock are raised mainly for meat and milk. Over 70% of the protected wildlife reserves and parks occur in the rangelands. Also, most (about 65–70%) of the national terrestrial wildlife populations occur in the human-modified rangelands outside the protected areas [21,26]. About 10–12% of Kenya is officially designated for biodiversity conservation, with protected wildlife areas covering only 8% (over 60 parks and reserves and numerous sanctuaries and conservancies), and the rest consisting of forests, water catchments and private sanctuaries [27,28]. Tourism based on wildlife viewing and photography ranks among the leading industries in Kenya, contributing about 13.7% of the gross domestic product and over 10% of the national formal sector employment. For example, in 2011 wildlife-based safaris contributed about US$ 1.16 billion to the national revenue of Kenya [29].

Considerable effort and resources have been invested in monitoring wildlife, livestock and their environment in Kenya’s rangelands since 1977. However, relatively little effort and resources have been invested in analyzing and interpreting the status and trends in wildlife and livestock numbers or their environmental and anthropogenic drivers. The very few studies that have analyzed wildlife and livestock population status and trends in Kenya’s rangelands have considered either only changes in numbers of single species [30], changes in decadal averages of numbers of individual species [12,26], changes in aggregated numbers of all species [21] or meta-analyses of population trends [13]. As a result, we still have relatively little understanding of the status and trends in numbers of individual livestock and wildlife species within particular rangeland counties, as well as nationally. Here, we update and extend the earlier analyses of livestock and wildlife population status and trends throughout all of Kenya’s rangelands and within individual rangeland counties that covered 1977–1997 [12,13, 21,26,30] to cover 1977–2016. Our analysis addresses seven objectives. First, we quantify population status and trends in numbers of individual wildlife and livestock species within each county as well as the population status and trends in numbers aggregated across all the 21 rangeland counties. Second, we quantify trends in the aggregated biomass of all the wildlife and livestock species across all the 21 rangeland counties. Third, we calculate the proportionate distribution of the biomass of each wildlife and livestock species among the 21 counties during 1977–1980 and 2011–2016. Fourth, we analyze temporal changes in human population size, rainfall, minimum and maximum temperatures as proxies for anthropogenic and environmental changes. Fifth, we relate wildlife population density to human population density, total livestock biomass density, percentage area of each county under protection, average annual rainfall, minimum and maximum temperatures. Sixth, we use the quantitative evidence provided by the trends and their relationships with the anthropogenic and environmental covariates, as well as county and interspecific distinctions in changes in numbers of individual species during the 40-year monitoring period spanning 1977–2016 to infer the success of pastoral livestock production, wildlife management, conservation policies and practices in the Kenyan rangelands.

Lastly, and perhaps most importantly, we discuss and interpret the trends relative to the existing policies, institutions and markets for wildlife, with a strong focus on provisions of the Wildlife Conservation and Management Act passed by the Kenya Parliament in 2013 to infer important policy gaps. The Act devolves wildlife conservation and management rights, opportunities and responsibilities to county governments, land owners and land managers where wildlife occurs outside public conservation areas and sanctuaries. In so doing, the Act aims to rectify long-standing legal, policy, administrative and law enforcement deficiencies that had hitherto undermined the effectiveness of conservation and management of wildlife on public, community and private lands in Kenya. Specifically, we consider the data needed to realize the potential, and monitor the effectiveness of the Act. We examine if the Act has the potential to mark a turning point in the declining wildlife trends and highlight its provisions with the greatest potential to turn the declining trends around and how effectively it addresses the root causes of the declines. Our assessment thus examines the status of wildlife now, compared to what it used to be in the early part of the monitoring period, attempts to identify the most promising areas for population recovery and restoration. We also explore the levels of wildlife populations these areas might be restored to using the monitoring data. The monitoring data also have huge potential to contribute to spatially explicit modeling, for example, of factors causing wildlife decline or, conversely, of factors that in combination are conducive to recovery. The data are also vital for planning at the landscape and regional levels. Nonetheless, for brevity we focus here only on spatial analyses at the county and ‘national’ levels and relegate finer-scale analyses of the dynamics of the distribution of wildlife and livestock abundance within counties in relation to environmental and anthropogenic correlates to future analyses.

## Methods

The Directorate of Resource Surveys and Remote Sensing of Kenya (DRSRS), and its predecessors, the Kenya Rangelands Ecological Monitoring Unit (KREMU: 1976–1986) and the Department of Resource Surveys and Remote Sensing (1986–2013), started monitoring the population size and spatial distribution of wildlife and livestock in Kenya’s rangelands using aerial sample surveys in 1977. The same sampling procedure has been used since monitoring began in 1977. A total of 361 surveys were conducted between 1977 and 2016 (S1 Data). Systematic reconnaissance flights are used to obtain information on animal numbers and distribution. The survey flights follow systematic, east-west oriented and parallel flight lines or transects spaced 5 km and, occasionally 10 km apart, following the Universal Transverse Mercator (UTM) coordinate system [31]. North-south transects are used in areas where the terrain makes east-west flying at low altitude too dangerous to undertake. The flights are carried out using high-winged aircraft (Cessna 186 or Partenavia 68) equipped with Global Navigation Systems, a Global Positioning System, Internal Communication System and radar altimeters for accurate navigation and mapping of animal distribution. Each transect is divided into equal sample intervals, generally 5 km long. The typical sampling unit is thus 5 km × 5 km but 5 km × 2.5 km (*n* = 30 surveys) or 5 km × 10 km (*n* = 26) were used in some surveys. The total number of sampling units used per survey vary with the area of the county (23267.7 ± 17821.8 km^2^, range 2801–75972 km^2^) and during 1977–2016 averaged (812.5 ± 408.4, range 198–2372 units, *n* = 249 surveys). Animals are counted only within observation strips defined on the ground by two parallel rods (streamers) attached to the wing struts of the aircraft. The crew consists of a pilot, two rear-seat observers who count and record animals sighted within the strip width and one front-seat observer who records land use and cover attributes and other environmental variables. Prior to the actual survey the aircraft is calibrated to the defined survey strip width [31]. The average strip width was 285.0 ± 37.6 m (*n* = 257 surveys, range 224–490 m) and the average flying height was 75–122 m above ground level. The flight speed varies with terrain features but never falls below 190 km /hour. The average coverage or sampling intensity was 5.7 ± 2.3% (*n* = 246 surveys, range 1.85–11.9%; S2 Data). The technical details of each survey, including the start and end dates, are also summarized in S2 Data.

Observations were recorded on audio tapes. A 35 mm analogue camera was used to take oblique photos of groups of more than 10 animals for later correction of the visual estimates from 1970s to 1990s. The photos were captured on film rolls, which were then projected onto a large screen for accurate counting. From 1990s onwards, 35 mm digital cameras were used. The digital photos are downloaded and animals counted on large computer or digital television screens. Animals in earlier photos were counted with the aid of binocular microscopes or data projectors. Jolly’s method 2 [32] for transects of unequal lengths is used to estimate population size and its standard error [30]. Because they are hard to reliably distinguish during the aerial surveys, sheep (*Ovis aries*) and goats (*Capra hircus*) are lumped together as shoats. Animals smaller than Thomson’s gazelle (*Gazella thomsoni*, *ca*. 15 kg) are too small to count reliably with this technique and hence are omitted. Also omitted are large herbivore species that were too few to reliably model trends in their numbers. The accuracy of population estimates derived from the aerial sample surveys has been tested repeatedly empirically and ranges between 71 and 83% or higher [26,33,34].

### Estimation of animal population size using Jolly’s Method 2

The total animal population size, its variance and standard error are calculated using Jolly’s method 2 for aerial transects of unequal lengths [32] as follows. The total population size is estimated as $\hat{Y}=Z\hat{R}$ with variance $Var(\hat{Y})=\frac{N(N-n)}{n}(s_{y}^{2}-2\hat{R}s_{zy}+{\hat{R}}^{2}s_{z}^{2})$ and standard deviation $SE(\hat{Y})=\surd \left(Var(\hat{Y})\right)$. *Z* is the area of the census zone (e.g. county) and $\hat{R}=\frac{\sum y}{\sum z}$ is the sample population density calculated as the total number of all animals counted in each sampling unit *y* divided by the area of each sampling unit *z* summed over all the units included in the survey sample. *N* is the population of all the sampling units in the census zone whereas *n* is the number of sampling units included in the survey sample (S2 Data). $s_{y}^{2}$ is the sample variance of the number of animals counted in all the sampled units while $s_{z}^{2}$ is the variance of the area of all the sampling units included in the survey sample. *s*_*zy*_ is the covariance between the number of animals counted and the area of each sampling unit.

### Anthropogenic and environmental changes

We used the following six variables (covariates) as proxies for anthropogenic and environmental changes affecting wildlife population dynamics. 1) The proportion of each county that is protected for wildlife conservation, considering only official state parks and reserves (S6 and S7 Datas). 2) Human population size for each county over the period 1962–2009 (S8 Data). National human population censuses were conducted in Kenya in 1962, 1969, 1979, 1989, 1999 and 2009 [35–39]. Population size for a target year without a census ($P_{t_{2}}$) was estimated from that for a year with a census ($P_{t_{1}}$) using $P_{t_{2}}=P_{t_{1}}e^{rt}$, where *r* = *ln*(*p*_2_/*p*_1_)/*t* is the average annual population growth rate between the two reference years and *t* = *t*_2_ − *t*_1_ years [40–41]. 3) The total biomass of sheep, goats, camels, donkeys and cattle per km^2^ (livestock biomass density). 4) Total annual (January-December) rainfall for each county spanning 1960–2014 (S9 Data). 5) Annual (January-December) average maximum and minimum temperatures for each county for 1960–2013 (S10 Data). The rainfall and temperature data were extracted from the Geospatial Climate (GeoCLIM) software tool developed through a partnership between The Planning for Resilience in East Africa through Policy, Adaptation, Research, and Economic Development (USAID PREPARED) project and The Famine Early Warning Systems Network (FEWS NET). GeoCLIM is a gridded national and regional (East African Community) climate data set tool that interpolates time-series grids of precipitation and temperature values from station observations and associated satellite imagery, elevation data, and other spatially continuous fields. The GeoCLIM tool is also able to identify anomalies and quantify their occurrence frequency and temporal trend [42]. Further particulars of GeoCLIM can be found at (http://chg-wiki.geog.ucsb.edu/wiki/GeoCLIM).

### Ethics Statement

All the aerial monitoring surveys were conducted by the Directorate of Resource Surveys and Remote Sensing (DRSRS) of Kenya and its predecessors—The Department of Resource Surveys and Remote Sensing (DRSRS: 1986–2013) and the Kenya Rangelands Ecological Monitoring Programme (KREMU: 1976–1986). DRSRS is currently part of the ministry of mining of the Government of Kenya. The mission of DRSRS is to promote sustainable development of geo-spatial information databases while up-holding efficiency in its dissemination for purposes of alleviating poverty and supporting sustainable development. DRSRS is officially mandated to collect, store, analyse, update and disseminate geo-spatial information on natural resources to facilitate informed decision-making for sustainable management of these resources with the major aim of alleviating poverty and environmental management. DRSRS collects data on the numbers and distributions of livestock and wildlife and associated anthropogenic and environmental / ecological attributes in all the Kenya Rangelands (spanning 88% of Kenya’s land surface); inventorizes, maps and monitors the vegetation and habitats of livestock and wildlife in Kenya; and undertakes land cover and use assessment, mapping and monitoring, among other activities, since 1974. The rangelands, and hence the aerial surveys, cover national parks, game reserves, sanctuaries, game ranches, private lands, communal lands and other types of land uses with wildlife or livestock. The aerial surveys are carried out from 75–122 m above ground and cover all species weighing at least 15 kg.

### Statistical models and analyses

#### Modeling temporal trends in animal numbers

The area of each county covered by the surveys did not vary over time. Hence we modeled trends in the estimated population size for all the wildlife species in each county simultaneously using a flexible multivariate semiparametric generalized linear mixed model (SGLMM). Livestock trends were likewise, but separately modeled. Separate models were fit to the resident and migratory populations of wildebeest (*Connochaetes taurinus*) and Burchell’s zebra (*Equus quagga burchelli*) in Narok County, corresponding to the wet (November-June) and dry (July-October) season counts, respectively. The migratory herds move seasonally between Kenya and Tanzania and occupy Narok County from July to at least October of each year.

The SGLMM model assumes that the population size estimates follow a negative binomial error distribution, that the variance of the counts is a quadratic function of the mean and a log link function. The logarithm of the overall mean population estimate for each species in each county was calculated and used as an offset to adjust for interspecific differences in population size in the same county. The model allows for trend patterns specific to each species as well as trend patterns common to all the species in each county, curvilinear trends, many zero counts and irregularly spaced surveys.

The SGLMM model consists of both parametric and non-parametric components. The parametric component can be represented relatively easily, unlike the non-parametric component that can be very challenging to model properly. Here, we model the non-parametric component by noting that spline smoothing and mixed modeling tackle equivalent minimization problems and produce the same solutions. A noteworthy difference is that unlike the solutions of spline coefficients in the classical framework, which are fixed effects, the solutions of the spline coefficients within the mixed modeling setting are solutions of random effects. As a result, standard errors of the predicted counts take the variation in spline coefficients associated with treating the coefficients as random effects in mixed models into account [43]. A key advantage of using the mixed model formulation of spline smoothing is that the smoothing parameter is computed ‘automatically’ as a function of the covariance parameter estimates produced by the mixed model. The semi-parametric model is also very flexible and can accommodate irregularly spaced counts and non-normal counts with many zeroes.

The negative binomial distribution (NB) of animal counts (*Y*) can be given by

$$P(Y=y)=\frac{\Gamma (y+k)}{y!\Gamma (k)}{\left(\frac{\mu }{\mu +k}\right)}^{y}{\left(\frac{k}{\mu +k}\right)}^{k}.$$ (1)

The mean of *Y* is given by *μ* = *E*(*Y*) and its variance by Var(*Y*) = *μ*(1 + *μ*/*k*). *k* is a shape or dispersion parameter and quantifies the amount of overdispersion [44].

Let **z** ≡ (***x***,*u*) be a covariate vector with ***x*** = (*x*_1_,…,*x*_*p*_,*x*_*p*+1_,…,*x*_*q*_), a 1 × (*p* + *q*) covariate vector and *u* be a continuous independent variable. Further, denote the expectation of *Y* with *u*(***z***) = *E*(*Y*|***z***). Then (1) can be recast in exponential form by

$$P(Y=y;z)=ylog\left\{\frac{\mu (z)}{\mu (z)+k}\right\}-klog(\mu (k)+k)+klogk+log\left\{\frac{\Gamma (y+k)}{\Gamma (k)}\right\}-log(y!).$$ (2)

This formulation shows that the NB model belongs to the exponential family of distributions if *k* is known. The variance of the NB we used is a quadratic function of its mean. Together with the parameter *k*, this accounts for overdispersion in the animal counts better than the Poisson distribution that is typically used with count data and assumes that the mean and variance of the counts are equal.

Next, we model the temporal trends in the animal counts by letting *u* be an unspecified smooth function of time and the fixed effects in ***x*** to be linear.

Assuming a log link function, because the canonical link log[*μ*(***z***)/{*μ*(***z***) + *k*}] implied by (1) is challenging to work with because it is always negative, the expected counts given the covariates is then specified as

$$log\{\mu (z)\}=x\beta +s_{1}(u_{1})+s_{2}(u_{2})+1.{log}_{e}(average\;pop\;size\;for\;each\;species)$$ (3)

***β*** = (*β*_1_,…,*β*_*p*_,*β*_*p*+1_,…,*β*_*q*_)^*T*^ is a 1 × (*p* + *q*)-parameter vector for the *p* + *q* -covariate vector ***x*** = (*x*_1_,…,*x*_*p*_,*x*_*p*+1_…*x*_*q*_), *s*_1_(⦁) and *s*_2_(⦁) are unspecified smooth functions that capture the effects of *u*_1_ and *u*_2_ and the offset log(average pop size for each species) has a slope coefficient equal to unity by construction. The unspecified smooth functions *s*_1_(⦁) and *s*_2_(⦁) are approximated by penalized *B*-spline basis functions as

$$s_{1}(u_{1})=\sum_{l=1}^{L}b_{l}B_{l}(u_{1})=Z_{1}u_{1}$$

$$s_{2}(u_{2})=\sum_{m=1}^{M}c_{m}B_{m}(u_{2})=Z_{2}u_{2}$$ (4)

where *b*_*l*_ and *c*_*m*_ are the penalized cubic B-spline coefficients to be estimated. The details of computation and mathematical properties of *B*-splines can be found in [45].

If we let ${\tilde{U}}_{s}$ represent the (*n* × *K*) matrix of *B*-splines of degree *d* and ***Q***_*r*_ the (*K* − *r* × *K*) matrix of *r*-th order difference penalty, then the (*n* × *K* − *r*) matrix $Z_{s}=[Z_{s_{1}},Z_{s_{2}}]$ used to fit the mixed model specified by Eqs (3) and (4) equals

$$Z_{s}={\tilde{U}}_{s}{\left({Q_{r}^{T}Q}_{r}\right)}^{-}Q_{r}^{T}$$ (5)

The total number of *B*-spline knots used to specify ${\tilde{U}}_{s}$ equals the number *m* of equally spaced interior knots plus *d* knots placed at the starting date and *max*{1,*d*} knots placed at the ending date of the surveys. The total number of columns in the *B*-spline basis is thus *K* = *m* + *d* + 1. The number of variables (columns) representing the penalized *B*-spline (called *P*-spline) random effect of time trend common to all the species is given by $d_{s_{1}}=(K-r)=(m+d+1-r)=20+3+1-3=21$. Here, the number of interior knots is *m* = 20, the degree of the B-spline basis is *d* = 3 and the order of differences of the spline coefficients is *r* = 3.

To clarify the rest of the notation used in (4), we use the 12 resident wildlife species counted in Narok County from 1977 to 2014. The number of all observations up to 2014 is *n* = (*n*_1_ + *n*_2_) × *p* = (34 + 11) × 12 = 540, where *n*_1_ = 34 is the number of surveys, *n*_2_ = 11 is the number of years with missing surveys and *p* = 12 is the number of resident wildlife species. Furthermore, the number of coefficients to be estimated for the species × time interaction term *q* = 12. Accordingly, the full design matrix of fixed effects ***x*** has dimension *n* × (*p* + *q*) = [540 × (12 + 12)] whereas the vector of fixed effect parameters ***β*** has dimension 1 × (*p* + *q*) = 1 × 24.

Let $d_{s_{1}}=21$ (assuming 20 knots) denote the number of variables representing the random P-spline effect of time trend common to all species. The number of variables representing the random P-spline effect for the time trend specific to each species (species × time interaction) is then calculated as $d_{s_{2}}=d_{s_{1}}\times p=21\times 12=252$. It follows that the dimension of the design matrix for the random effect ***Z***_1_ in (4) is $n\times d_{s_{1}}=540\times 21$ and that of the design matrix for the random effect ***Z***_2_ in (4) is $n\times d_{s_{2}}=540\times 252$. The full design matrix of random effects **Z** =[***Z***_1_,***Z***_2_] therefore has dimension $n\times (d_{s_{1}}+d_{s_{2}})=540\times (21+252).$ The vector of parameters of random effects ***u***_1_ in (4) has dimension $1\times d_{s_{1}}=1\times 21$ whereas ***u***_2_ has dimension $1\times d_{s_{2}}=1\times 252$. Analogously, the full vector of parameters of random effects ***u*** = (***u***_1_,***u***_2_)^*T*^ has dimension $[1\times (d_{s_{1}}+d_{s_{2}})]=1\times 273$. The random effects $u_{1}∼i.i.d.N(0,\sigma_{u_{1}}^{2})$ whereas $u_{2}∼i.i.d.N(0,\sigma_{u_{2}}^{2})$.

The only fixed effect in the mixed model is the main effect of animal species to enable direct estimation of different average population sizes (intercepts) for the different species in each county. The non-parametric part of the model consists of two continuous random effects, each with a penalized spline variance-covariance structure. The first random spline effect fits a penalized cubic B-spline (P-spline, [46]) with a third-order difference penalty to random spline coefficients common to all the species and thus models the time trend common to all the species. The second random spline effect similarly fits a penalized cubic B-spline with random spline coefficients specific to each species and hence models the time trend specific to each species. Both the random spline effects had 20 equally spaced interior knots placed on the running date (February 1977,…, January 2016 for Narok County) plus 3 evenly spaced exterior knots placed both at the start date and end date of the censuses. [45] describes the computation and properties of B-splines. The specific smoothers we used derive from the automatic smoothers described in [43]. For some counties the model with the P-spline smoother variance-covariance structure did not converge, so we used the radial basis spline smoother and constructed the spline knots based on the vertices of a k-d tree [47,48] with a bucket size of 10 to 100 observations. The k-d tree is a tree data structure that facilitates efficient determination of a prescribed number of nearest neighbors of a point. The full model specified by (3) and (4) therefore contains three variance components to be estimated, corresponding to the random spline time trend common to all species $\sigma_{u_{1}}^{2}$, random spline effects for the time trend specific to each species $\sigma_{u_{2}}^{2}$ and the scale parameter for the negative binomial distribution *k*. The full trend model was fitted by the residual penalized quasi-likelihood (pseudo-likelihood) method [49] in the SAS GLIMMIX procedure [50]. The fitted model was examined graphically for potential outliers, resulting in the exclusion of 22 observations (S3 Data) before final model fitting. An annotated SAS (version 9.4, GLIMMIX version 14.1) code used to fit the full model is provided in S1 File.

The model was used to predict expected counts for all years between 1977 and 2013 in which surveys were not conducted in each county. Predicted population size estimates and their 95% confidence limits for each species in each county were assigned to June for years without surveys between 1977 and 2013. After model fitting the population estimates for years with multiple surveys were averaged to obtain one estimate for each species per year. Population estimates for each species in each year were then summed across all the 21 rangeland counties to obtain an approximate “national” population size estimate for the species. Trends in the time series of the “national” population estimates for all the species were then simultaneously smoothed using the SGLMM model, separately for wildlife and livestock species. The predicted population estimates for each species were averaged separately over 1978–1980, 1994–1997 and 2011–2013 to reduce the influence of stochastic sampling variation on the estimated population sizes. The change in population size of each species between 1977–1980 and 1994–1997 and between 1977–80 and 2011–2013 (2011–2016 for Narok, Laikipia, Kilifi and Kwale, 2011–2014 for Kajiado, Marsabit, Tana River and Taita Taveta counties and 2011–2015 for Samburu, Isiolo, Machakos, Kitui Counties) was calculated as a percentage of the averaged population size in 1977–1980. The aggregate biomasses of wildlife and livestock were calculated using the unit weights in [51] and used to characterize temporal variation in biomass and proportionate distribution of biomass across counties.

#### Relating wildlife population size to anthropogenic and environmental change

The population size of each wildlife species was related to each of the six covariates using univariate regression models and to all the six covariates simultaneously using multiple regression models. For the univariate models, the population size of each wildlife species was related to each of the six covariates using a generalized linear mixed model with a negative binomial error distribution and a log link function. The logarithm of the total area of each county was used as an offset to obtain numerical population densities. The corrected Akaike Information Criterion (AICc) [52] was used to choose between the linear and quadratic models for each covariate, separately for each species. The model fitting procedure automatically computes the dispersion (scale) parameter of the negative binomial model and allows for potential overdispersion and serial autocorrelation in population size. The models were fitted in the SAS GLIMMIX procedure. Careful graphical inspection of the fitted models for human population density and livestock biomass density showed that the 95% confidence bands were too wide for both covariates. As a result, we used a nonlinear model assuming a constant variance to relate population density for each species to each of the two covariates using the SAS NLIN procedure [50]. We similarly related the total wildlife biomass density to each of the six covariates using the NLIN procedure.

A generalized linear model assuming a negative binomial error distribution and a log link function and using the logarithm of county area as an offset to calculate numerical population densities was also used to select the subset of the six covariates most strongly correlated with the density of each of the 18 wildlife species. The six covariates considered were human population density, total livestock biomass density, proportion of each county under protection, total annual rainfall, average annual minimum and maximum temperatures, their quadratic terms and all possible interactions. All the six main effects were internally centered and scaled (standardized) but parameter estimates and related statistics are reported on the original scale. The forward selection method was used to select the covariates most strongly correlated with wildlife population density. This selection method starts with no covariate effect in the model and adds covariate effects sequentially. Backward covariate elimination and stepwise selection methods produced essentially identical results. At each step of the selection method covariate effects were chosen and added to the model using the Akaike, corrected Akaike and Schwarz Bayesian information criteria. Selection of effects was subject to the strong hierarchy requirement, meaning that for any interaction term to be included in the model, all the main effects that are contained in the interaction term must also be present in the model. For example, in order for the interaction term livestock biomass density × human population density to enter the model, the main effects: livestock biomass density and human population density must also be in the model. Similarly, neither livestock biomass density nor human population density can leave the model while the interaction term livestock biomass density × human population density is still in the model. Model selection was carried out using the SAS GENSELECT procedure [50].

## Results

**Fig 2:**
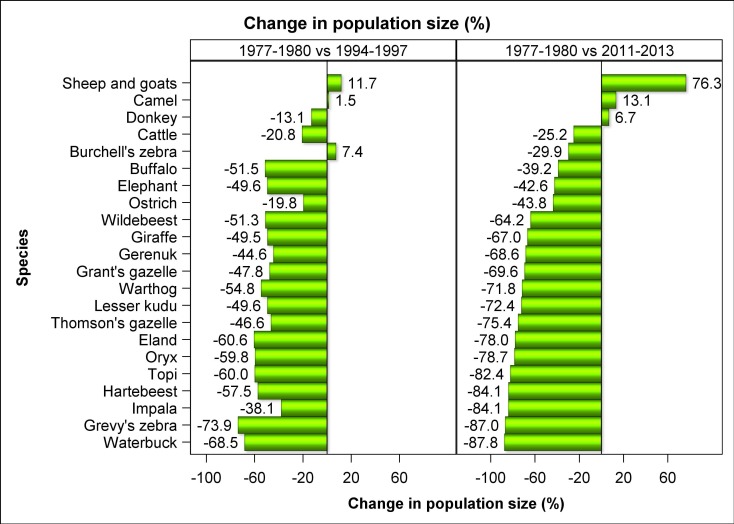
Percentage changes in numbers of each livestock and wildlife species aggregated across all the 21 rangeland counties of Kenya between 1977–1980 and 1994–1997 and between 1977–1980 and 2011–2013.

**Fig 3:**
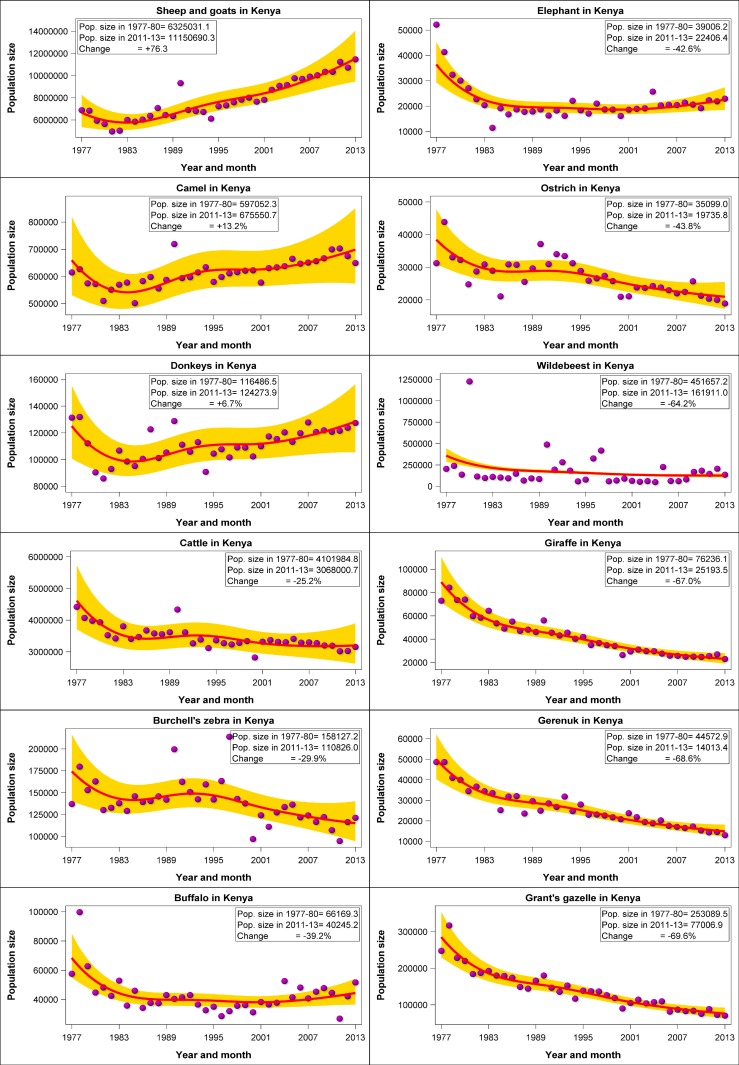
Trends in sheep and goats, camels, donkeys, cattle, Burchell’s zebra, buffalo, elephant, ostrich, wildebeest, giraffe, gerenuk and Grant’s gazelle numbers in the 21 Kenyan rangeland counties (“national” trends) between 1977 and 2016. Note that the data points do not refer to actual counts but to the sum of the counts in all the counties for the same year. If no survey was done in a county in a given year, then the missing count was predicted by the trend model for the county.

**Fig 4:**
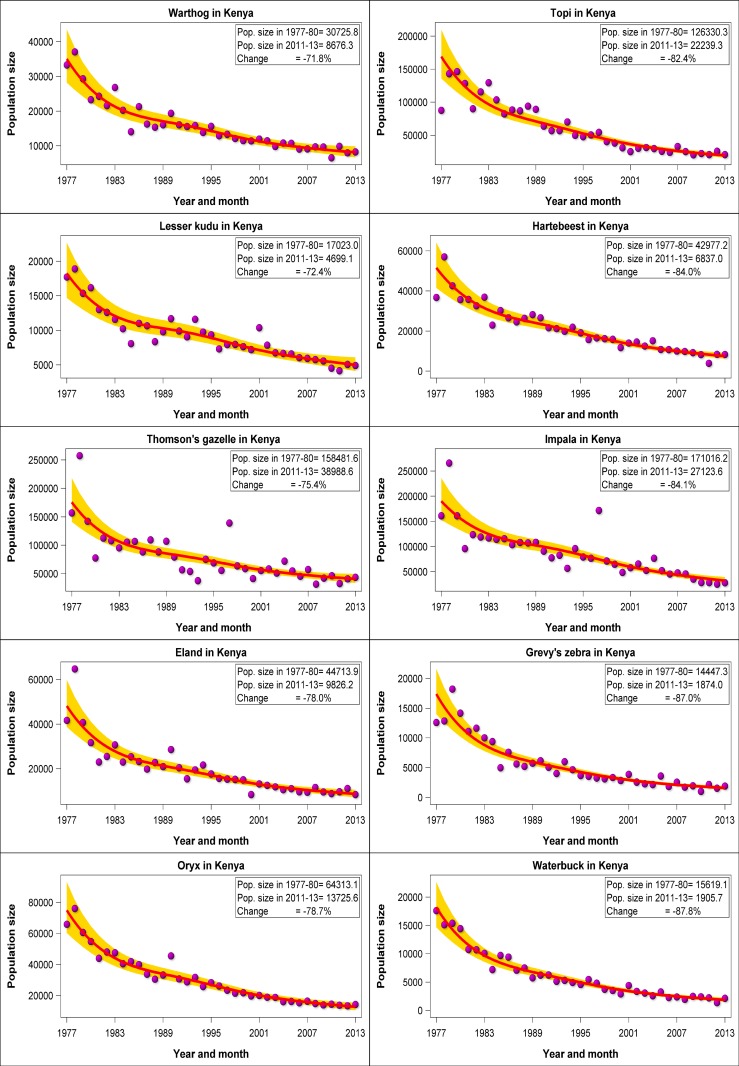
Trends in warthog, lesser kudu, Thomson’s gazelle, eland, oryx, topi, hartebeest, impala, Grevy’s zebra and waterbuck numbers in the 21 Kenyan rangeland counties (“national” trends) between 1977 and 2016. Note that the data points do not refer to actual counts but to the sum of the counts in all the counties for the same year. If no survey was done in a county in a given year, then the missing count was predicted by the trend model for the county.

The most salient features of the trends were a striking increase in numbers of sheep and goats and camels and concurrent extreme declines in numbers of 14 of the 18 common wildlife species throughout Kenya’s rangelands between 1977 and 2016 (Fig 2). The numbers of sheep and goats aggregated across all the 21 rangeland counties (‘national’ trend) increased markedly by 76.3%, followed by 13.1% for camels (*Camelus dromedarius*) and 6.7% for donkeys (*Equus asinus*) while the number of cattle (*Bos indicus*) dropped by 25.2%. In sharp contrast to the increasing trends or moderate declines in livestock numbers, the aggregated numbers of the common wildlife species declined precipitously, and for certain species catastrophically, in the same period in the Kenyan rangelands. The declines were pervasive and extreme despite the contrasting feeding and foraging guilds, body sizes, gut morphology and spatial distribution of the different species among the 21 counties with widely varying rainfall patterns. The rates of decline between 1977 and 2016 varied markedly among species but averaged 68.1% (1.7% per year) across all the 18 wildlife species. The declines were particularly extreme (72–88%) for warthog (*Pharcoerus africanus*), lesser kudu (*Tragelaphus imbermbis*), Thomson’s gazelle, eland (*Taurotragus oryx*), oryx (*Oryx gazelle beisa*), topi (*Damaliscus lunatus korrigum*), hartebeest (*Alcelaphus buselaphus*), impala (*Aepyceros melampus*), Grevy’s zebra (*Equus grevyi*) and waterbuck (*Kobus ellipsiprymnus*); severe (60–70%) for wildebeest, giraffe (*Giraffa cemelopardalis*), gerenuk (*Litocranius walleri*) and Grant’s gazelle (*Gazella granti*); and moderate (30–50%) for Burchell’s zebra, buffalo (*Syncerus caffer*), elephant (*Loxodonta africana*) and ostrich (*Struthio camelus*). The declines reduced the populations of many species, most notably of warthog (30726 in 1977–1980 vs 8676 in 2011–2013), lesser kudu (17023 vs 4699), Thomson’s gazelle (158452 vs 38989), eland (447145 vs 9826), oryx (64313 vs 13726), topi (126330 22239), hartebeest (42977 vs 6837), impala (171016 vs 27124), Grevy’s zebra (14447 vs 1874) and waterbuck (15619 vs 1906), to levels that now critically threaten their future population viability or persistence in the rangelands unless urgent, decisive, drastic and sustained remedial steps are taken to restore their depleted populations (Figs 3 and 4). The numerical population estimates from all the aerial surveys in the 21 counties between 1977 and 2016 and the corresponding smoothed population estimates and the associated 95% confidence limits are provided in S4 Data. The estimated average population size and the proportion of the total population of each species in each county in 1977–1980 and 2011–2016 and the percentage changes in the population size and proportion between the two periods are summarized in S1 Table.

On average, wildlife numbers declined by 48.7% between 1977–1980 and 1994–1997 compared to 68.1% between 1977–1980 and 2011–2016, implying approximately 20% wildlife losses between 1994–1997 and 2011–2016 (Fig 2). Except for buffalo and elephant, numbers of all the wildlife species had declined much more severely by 2011–2016 relative to 1994–1997. Livestock showed the converse pattern with numbers of all livestock species increasing distinctly between 1994–1997 and 2011–2016 except for cattle whose numbers continued to decline (Fig 2).

**Fig 5:**
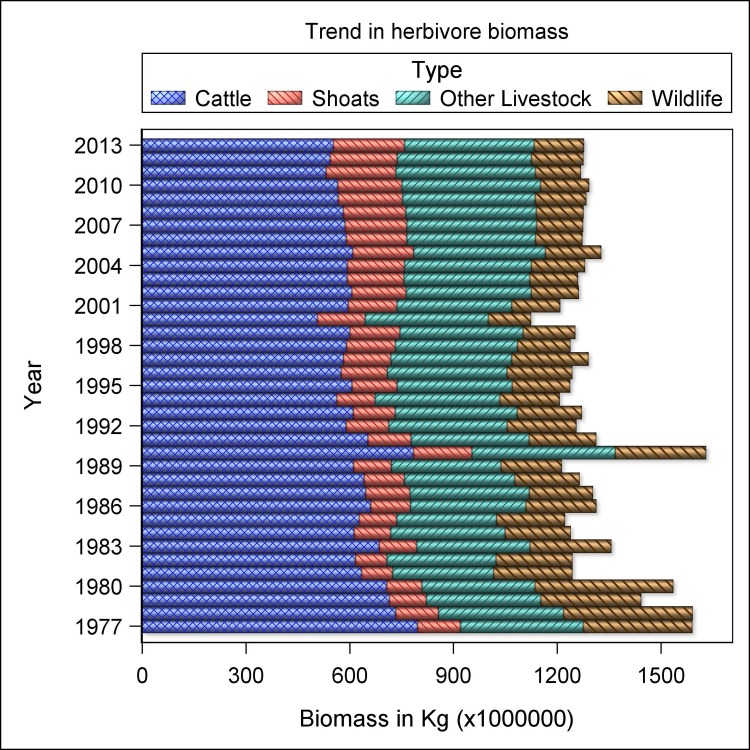
Trends in wildlife and livestock biomass aggregated across all the 21 rangeland counties of Kenya between 1977–1980 and 2011–2013. Shoats denote sheep and goats while other livestock denotes camels plus donkeys.

The aggregate biomass of livestock and wildlife decreased differentially between 1977–1980 and 2011–2013. The magnitude of the decrease was 12.9 times greater for wildlife (from 345.0 × 10^6^ to 140.5× 10^6^ kg, i.e., -59.30%) than for livestock (from 1195.7 to 1140.7 × 10^6^ kg, i.e., -4.6%). As a consequence of the differential rates of decline, the contribution of wildlife to the total herbivore biomass dropped by half from 22.4% in 1977–1980 to 11.0% in 2011–2013. This resulted in livestock biomass becoming 8.1-fold larger than wildlife biomass in 2011–2013 compared to 3.5-fold in 1977–1980, implying that livestock were evidently replacing wildlife (Fig 5). The fact that livestock biomass decreased rather than increased between 1977 and 2013, despite the contemporaneous marked increase in numbers of sheep and goats, camels and donkeys, imply long-term range degradation or loss especially for cattle and that the expansions in the numbers of the four livestock species were occurring, at least in part, at the expense of the shrinking cattle numbers (Fig 5).

**Fig 6:**
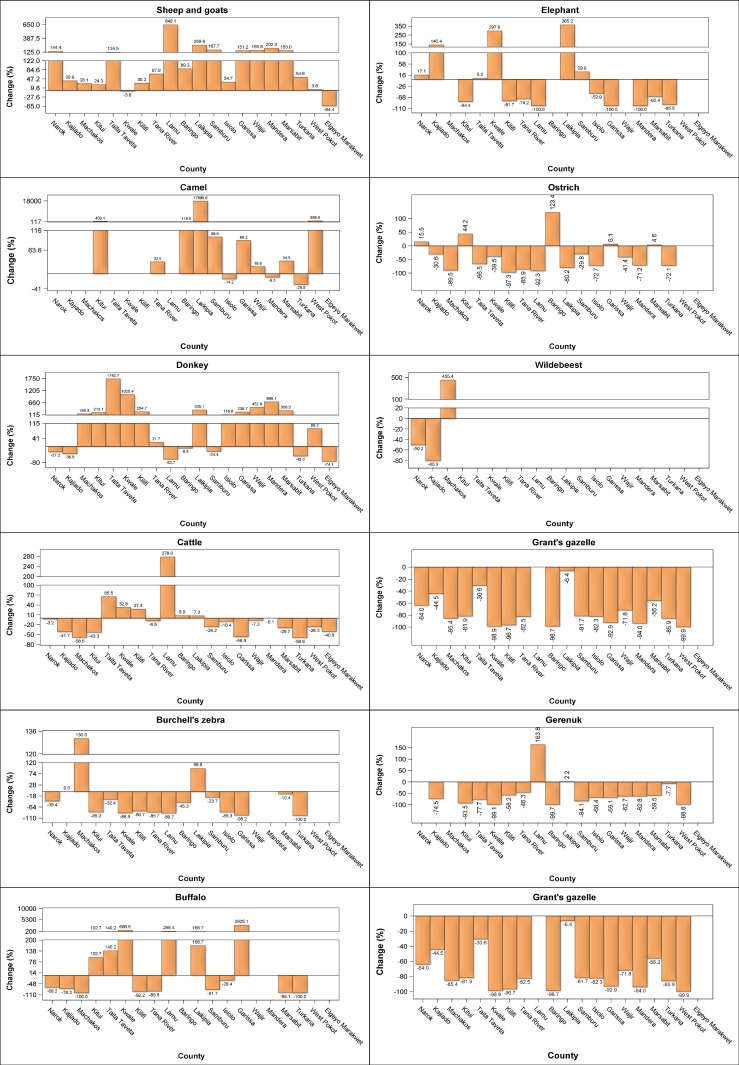
Percentage changes in numbers of sheep and goats, camels, donkeys, cattle, Burchell’s zebra, buffalo, elephant, ostrich, wildebeest, giraffe, gerenuk, Grant’s gazelle, warthog, Lesser kudu, Thomson’s gazelle and eland in each of the 21 rangeland counties between 1977–1980 and 2011–2016.

**Fig 7:**
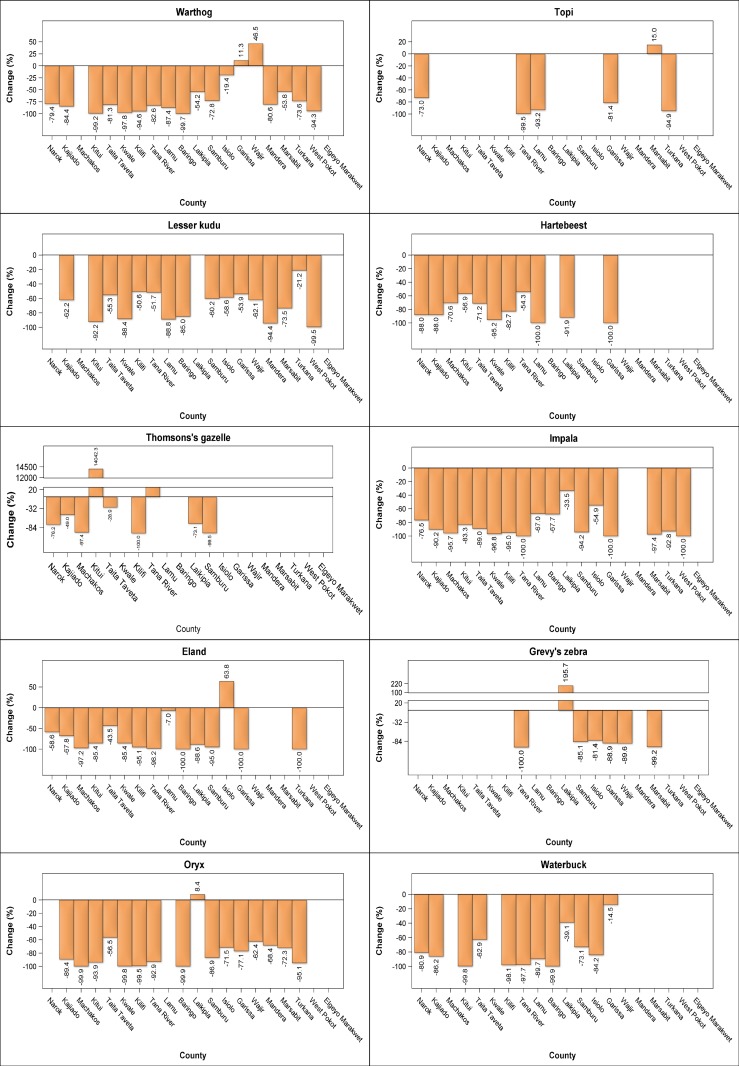
Percentage changes in numbers of warthog, lesser kudu, Thomson’s gazelle, eland, oryx, topi, hartebeest, impala, Grevy’s zebra and waterbuck in each of the 21 rangeland counties between 1977–1980 and 2011–2016.

There were considerable interspecific differences in the trends shown by individual livestock species across counties. The substantial increase in numbers of sheep and goats, camel and donkeys occurred in most of the counties. The numbers of sheep and goats increased most spectacularly (124.5–648.1%) in 8 counties (Narok, Taita Taveta, Lamu, Laikipia, Samburu, Garissa, Wajir, Mandera and Marsabit), moderately (3.8–89.3%) in 10 counties but decreased marginally (3.8–64.4%) in Kwale and Elgeyo Marakwet counties (Figs 6 and 7, S2–S21, S32 Figs). The population of camels also increased many-fold (450–17896%) in Kitui, Laikipia and West Pokot counties and, to a lesser extent (89–119%), in Baringo, Garissa and Samburu counties, signifying increasing and widespread adoption of camels in these counties. Minor decreases were apparent in camel numbers in Turkana, Mandera and Isiolo counties (Figs 6 and 7, S5, S9, S11–S20, S32 Figs). The number of donkeys also increased substantially in 12 counties but decreased noticeably in 6 others (Figs 6 and 7, S2–S21, S32 Figs). By contrast, cattle numbers increased only in Taita Taveta, Kwale, Kilifi, Lamu, Baringo and Laikipia counties but decreased in all the other 16 counties (Figs 6 and 7, S2–S21, S32 Figs).

In stark contrast to the widespread and substantial increase in numbers of sheep and goats, camel and donkeys, the numbers of the common wildlife species declined severely in most or all of the counties (Figs 6 and 7, S2–S21, S32 Figs). The wildlife species that declined most extremely in all the counties comprised giraffe, lesser kudu, hartebeest, impala and waterbuck. The other 13 wildlife species also declined severely in most counties but increased in at least one county (Figs 6 and 7, S2–S21, S32 Figs).

**Fig 8:**
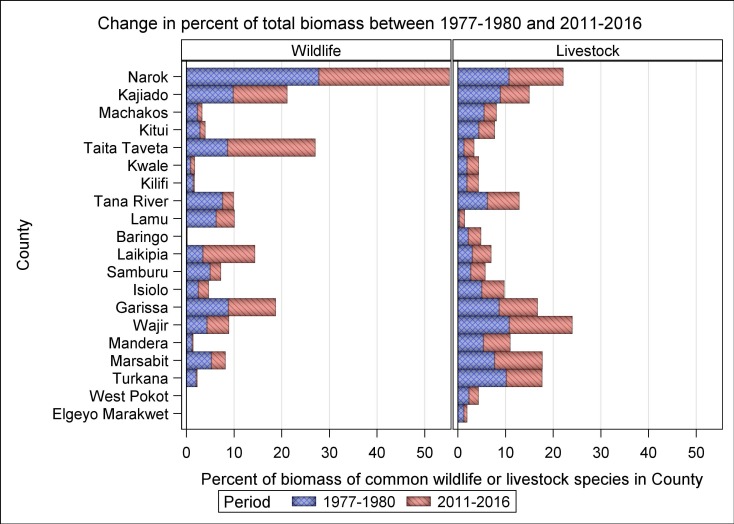
The distribution of the proportion of the total giraffe biomass among the 21 rangeland counties of Kenya in 1977–1980 and 2011–2016.

**Fig 9:**
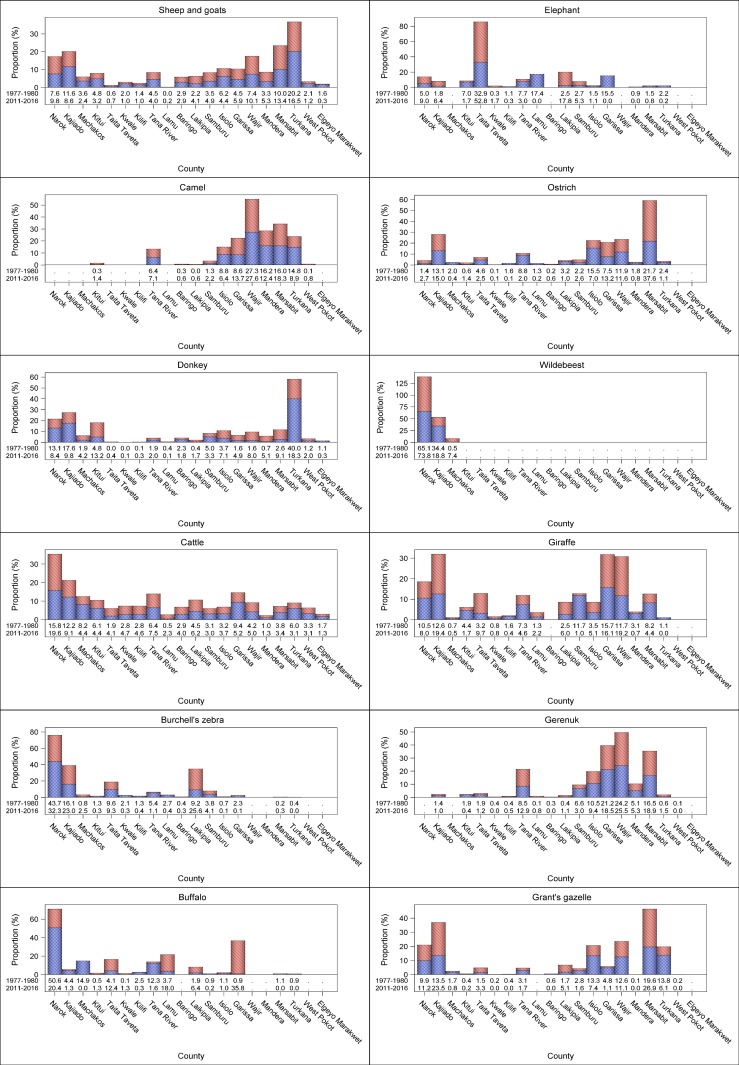
The distribution of the proportion of the total biomass of sheep and goats, camels, donkeys, cattle, Burchell’s zebra, buffalo, elephant, ostrich, wildebeest, giraffe, gerenuk and Grant’s gazelle among the 21 rangeland counties of Kenya during 1977–80 and 2011–2016.

**Fig 10:**
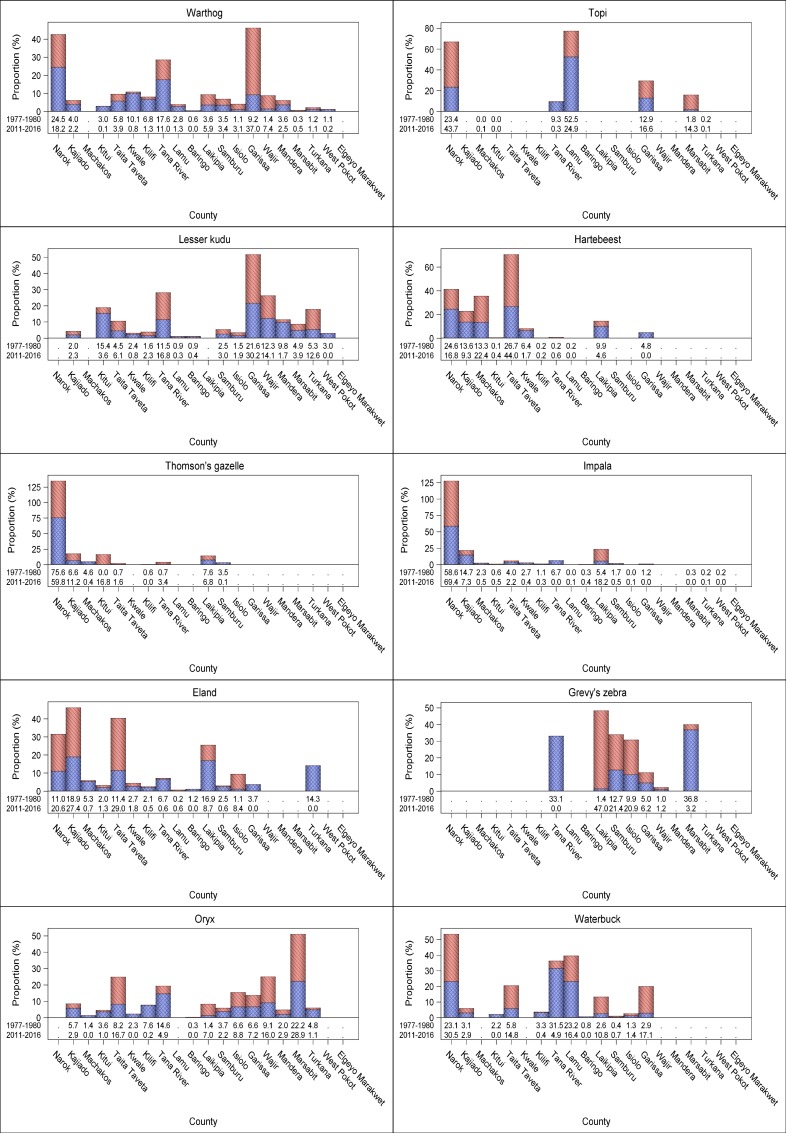
The distribution of the proportion of the total biomass of warthog, lesser kudu, Thomson’s gazelle, eland, oryx, topi, hartebeest, impala, Grevy’s zebra and waterbuck among the 21 rangeland counties of Kenya during 1977–80 and 2011–2016.

The proportions covered by protected areas vary considerably among the 21 rangeland counties of Kenya, with important implications for wildlife conservation (Fig 1, S6 and S7 Datas). While more than half of some counties are protected for wildlife conservation, much smaller proportions of some counties are devoted to wildlife conservation (Fig 1, S6 and S7 Datas). This variation may be expected to be reflected in the distribution of herbivore population size and hence biomass among the counties. The contrasting rates of decline or increase in livestock (e.g. 4,101,984.8 cattle in 1977–1980 vs 3,068,000.7 in 2011–2013) and wildlife numbers across counties were associated with major changes in the proportional distribution of livestock or wildlife biomass among counties in 2011–2016 compared with 1977–1980 (Fig 8). Moreover, within each of the latter two periods, the proportional distribution of livestock and wildlife species biomass varied markedly across counties (Figs 9 and 10). The biomass of livestock appeared more evenly distributed across counties than that of wildlife. Even so, livestock were relatively more abundant in Narok, Kajiado, Garissa, Wajir, Marsabit and Turkana than in the other counties in both 1977–1980 and 2011–2016. Wildlife were also relatively more abundant in the same counties as livestock were, even though Narok held a far greater proportion of Kenya’s wildlife biomass than any of the other counties in both 1977–1980 (27.8% vs. 0.03 to 9.8% for the other counties) and 2011–2016 (27.4% vs. 0.0 to 18.4%) (Fig 8). Moreover, there were several notable differences among counties in the magnitude and direction of changes in the proportional distribution of livestock and wildlife biomass over time. Specifically, the proportion of total livestock biomass found in Kajiado, Machakos and Turkana decreased whereas the fraction found in Narok, Wajir and Marsabit increased in the same period (Fig 8). In contrast to livestock, the proportion of total national wildlife biomass increased noticeably only in Taita Taveta (by 9.8%) and Laikipia (7.5%) counties but decreased evidently in Tana River (5.3%), Lamu (2.4%), Samburu (2.7%) and Turkana (1.8%, Fig 8).

Noteworthy changes were also evident in the proportional distributions of the livestock and wildlife species’ biomass within counties. Marked declines in the proportion of total livestock biomass were noted for donkeys in Kajiado and Turkana whereas increases in the proportions of the total population biomass were recorded for sheep and goats in Narok, Wajir and Marsabit and for cattle in Narok (Figs 9 and 10). Among individual wildlife species, marked increases or decreases in the proportion of the total population biomass were recorded for several counties, for example topi in Narok and Grant’s gazelle and eland in Kajiado. Given the massive overall declines in numbers of most of the wildlife species, such increases primarily highlight differential rates of decline of the same species across counties (Figs 9 and 10).

### Temporal anthropogenic and environmental changes

The total human population size in all the 21 rangeland counties grew 4.8-fold (383%) from 1962 (2,604,900 people) to 2009 (12,582,028 people). Although human population size grew exponentially in each of the 21 rangeland counties during 1962–2009, the population growth rate varied markedly across the counties. As a result, the population size per county in 2009 was, on average, 469% (range: 162%–1259%) larger than in 1962 (S22 Fig). The two key climatic components, rainfall and temperature, also fluctuated widely among counties and over time. Both the minimum and maximum temperatures increased significantly in all the 21 rangeland counts except for Narok and Kajiado where the increase in the maximum temperature did not reach statistical significance (S4 Table). The striking temperature rise was also evident in all the other 26 counties of Kenya, reflecting a more general pattern of regional warming (S4 Table). The pattern of inter-annual variation in rainfall provided strong evidence of quasi-periodic oscillation in the annual rainfall component and a general decline in rainfall, or an initial decline in rainfall followed by an upward trend (Narok, Baringo, Laikipia, Turkana, West Pokot and Elgeyo Marakwet counties) between 1960 and 2014 (S23 Fig). Although substantial, the decline in annual rainfall over time was not statistically significant (S4 Table). The annual average maximum temperature ranged between 24.3 and 33.2°C and increased persistently by 0.7 to 1.9°C between 1960 and 2013 in all the 21 counties (S24 Fig). The average annual minimum temperature ranged between 10.6 and 24.0°C and increased by 0.6°C to 1.7°C between 1960 and 2013. Notably, minimum temperatures initially decreased up to the 1970s before increasing steadily in 10 of the 21 counties located mostly in Southeastern Kenya. The counties that experienced the largest increase in both minimum and maximum temperatures were, in increasing order, Machakos, Marsabit, Samburu, Baringo, Elgeyo Marakwet, Turkana and West Pokot (S25 Fig).

### Relationships between wildlife population size, anthropogenic and environmental changes- univariate patterns

Wildlife population density was generally nonlinearly related to human population density, livestock biomass density, percentage of each county under protection, rainfall, or maximum or minimum temperature (S5 and S6 Tables, S26–S31 Figs). Wildlife numbers declined at high human population density. Gerenuk, lesser kudu and oryx avoided people so that their densities peaked at zero human population density. The density of all the other 15 wildlife species increased to a peak (= −*β*/2*γ*, where *β* and *γ* are the linear and quadratic slopes in a given covariate in S6 Table, respectively) at human population densities ranging between 3 and 34 people / km^2^ and then dropped to zero at larger human population densities (S6 Table, S26 Fig). Wildlife population density either decreased with increasing livestock biomass density, with a peak at zero livestock biomass density, indicating avoidance of livestock (elephant, lesser kudu, oryx, topi and waterbuck), or, initially increased up to a peak and then declined with further increase in livestock biomass density. The location of the peak in density in the latter case varied with wildlife species and occurred at livestock biomass densities ranging between 2000 and 8000 kg/km^2^ (S5 and S6 Tables, S27 Fig.).

**Fig 11:**
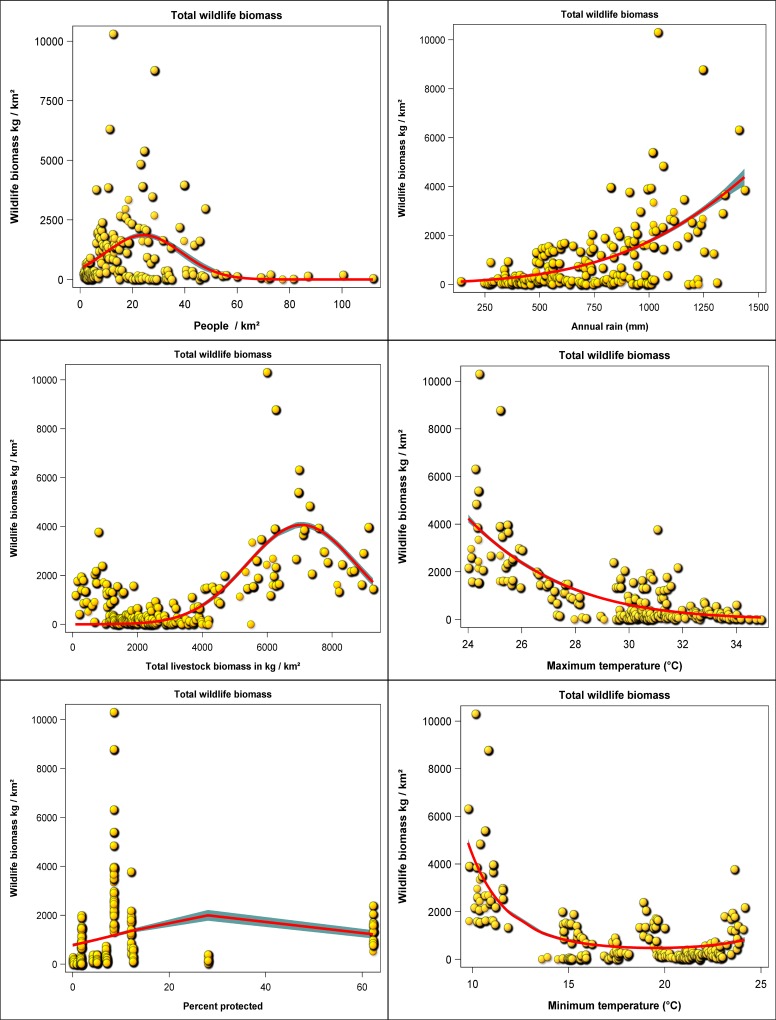
The relationship between total wildlife biomass (kg/km^2^) and human population density (people /km^2^), total livestock biomass (kg/km^2^), percentage of each county under protection (%), total annual rainfall (mm), annual average maximum and minimum temperatures (deg C).

Wildlife density increased with the percentage of a county under protection, most notably when only counties with up to 20% of their areas under protection are considered. Counties with larger percentages of their area under protection were relatively few and deviated somewhat from this general pattern for certain species (S5 and S6 Tables, S28 Fig). Wildlife population density increased with rainfall except for two species (ostrich and gerenuk) whose densities decreased with rainfall and six species (elephant, ostrich, lesser kudu, eland, oryx and Grevy’s zebra) whose densities increased with rainfall up to a peak at intermediate levels of rainfall and then decreased at higher rainfall values (S5 and S6 Tables, S29 Fig). In contrast, high maximum and minimum temperatures were associated with lower densities of all but five wildlife species (gerenuk, lesser kudu, oryx, hartebeest and Grevy’s zebra) which occurred at higher densities in areas of intermediate or high temperatures. The densities of the latter five species also appeared to decline at the higher end of temperature values. Two minor departures from this pattern were shown by topi and waterbuck that occurred at high densities where temperatures were high in Lamu, near water bodies (S5 and S6 Tables, S30 and S31 Figs). The aggregate wildlife biomass also increased to a peak at intermediate values and then declined with further increase in human population or total livestock biomass density. In contrast, wildlife biomass increased with increasing rainfall or percent of protected area in a county (notably for values < 20%) but decreased with rising minimum or maximum temperatures (S6 Table, Fig 11).

### Relationships between wildlife population size, anthropogenic and environmental changes- multivariate patterns

Population density for each of the 18 wildlife species was significantly influenced by changes in at least one of the six covariates. Nevertheless, the specific suite of covariates most strongly correlated with population density varied with the species of wildlife as follows (S7 and S8 Tables). Human population density significantly influenced population trends of only 10 of the 18 wildlife species (Burchell's zebra, buffalo, elephant, ostrich, giraffe, gerenuk, Grant's gazelle, Thomson's gazelle, oryx and waterbuck). Further, the proportion of each county under protection was strongly correlated with the population trends of 8 wildlife species (Burchell's zebra, buffalo, elephant, gerenuk, eland, impala, hartebeest and waterbuck). Quite, surprisingly, however, livestock biomass density only significantly affected the population trend of Grant’s gazelle. Lastly, rainfall (buffalo, elephant, ostrich, giraffe, gerenuk, Grant's gazelle, warthog, topi and waterbuck), maximum temperature (Burchell's zebra, buffalo, ostrich, giraffe, gerenuk, oryx, waterbuck, Grant's gazelle, Thomson's gazelle, eland, impala, hartebeest) and minimum temperature (Burchell's zebra, buffalo, ostrich, giraffe, gerenuk, oryx, waterbuck, wildebeest, lesser kudu, topi, Grevy's zebra) strongly and significantly influenced the population density of 9, 10 and 11 wildlife species, respectively (S7 and S8 Tables).

## Discussion

We analysed the status and trends in the population of pastoral livestock and wildlife species in the Kenya rangelands using aerial sample survey monitoring data collected by the Directorate of Resource Surveys and Remote Sensing of Kenya during 1977–2016. We found substantial increases in numbers of sheep and goats, camels and donkeys but a moderate decline in numbers of cattle. The results also showed a disturbing loss of wildlife in the same period averaging 68.1% (or 1.7% per year). The magnitude of the declines varied considerably among species and counties. The declines were most pronounced (64–88%) for, and therefore severely threaten the continued population viability and persistence of wildebeest, giraffe, gerenuk, Grant’s gazelle, warthog, lesser kudu, Thomson’s gazelle, eland, oryx, topi, hartebeest, impala, Grevy’s zebra and waterbuck. The gravity of the declines is underscored by the facts that already by 2013, 7 species of large mammals had been classified as critically endangered, including Ader’s duiker (*Cephalophus adersi*), the hirola or Hunter’s hartebeest (*Beatragus hunteri*), roan (*Hippotragus equinus*) and sable (*Hippotragus niger*) antelopes, 19 species of mammals were rated as endangered, whereas 37 species of mammals were classified as vulnerable in Kenya [53].

Our analysis updates and broadens four earlier ones, the two most recent of which covered the period 1977–1997. Our results show that the declines reported by the earlier analyses have worsened such that populations of several species are now severely threatened in Kenya. The substantial but less severe declines in wildlife numbers documented for the rangelands by the earlier analyses are as follows. [12] reported that between 1977 and 1994 wildlife declined in the Kenya rangelands by 33%. [26] found wide fluctuations but little change in cattle numbers, a 10–14% decline in numbers of sheep, goats and donkeys, a 12% increase in camel numbers and a 40–60% decline in numbers of all the common wildlife species but wildebeest and ostrich during 1977–1994. Lastly, [26] estimated that over 70% of the wildlife in the Kenya rangelands occurred in non-protected pastoral areas. [21] concluded that wildlife declined by 38% in 17 rangeland counties and by an average of 41% in five premier protected areas of Kenya between 1977 and 1997. In contrast, our results based on all the available aerial survey data sets for 1977–2016 show that, on average, wildlife numbers declined by 48.7% between 1977–1980 and 1994–1997 and, hence, that wildlife numbers declined by a further 20% between 1994–1997 and 2011–2013.

Severe wildlife declines and range contraction have also been recently documented based on analyses of the DRSRS data for several individual rangeland counties, comprising Kajiado [54], Kwale, Kilifi and Lamu [55], Tana River [56], Laikipia [57], Garissa [58] and Marsabit [59]. Extreme Kenyan-wide declines in numbers and range of Hunter’s hartebeest [60,61], Grevy’s zebra [62,63], Ader’s duiker [64], roan antelope [65] and sable antelope [66] have also been recently reported. Several other studies have also reported devastating declines or wide fluctuations in wildlife numbers in particular regions of Kenya, including the Masai Mara [16,17,67–73], Lake Nakuru National Park [18], Nairobi National Park and Kajiado County [19,20,74,75], Kisumu Impala Sanctuary [76], Laikipia County [77] and Ruma National Park [65]. More precisely, [67] reported that numbers of non-migratory wildlife declined by 58% in the Masai Mara National Reserve and at a similar rate in the adjoining pastoral lands between 1977 and 1997. [70] and [68] found that numbers of resident wildebeest had declined in Masai Mara by between 75% and 81% during 1977–1997. [17] later estimated an average rate of decline of about 67% for all the common wildlife species in the Masai Mara between 1977 and 2009. [21] reported that wildlife numbers declined by 63% in Tsavo East and Tsavo West National Parks between 1977 and 1997 and by 78% in Meru National Park between 1977 and 2000.

Our analysis showed that six species of wildlife declined by 63–89% in Taita Taveta County (home to Tsavo National Park) during 1977–2014, highlighting a very disturbing loss of wildlife within the confines of Kenya’s largest and heavily guarded Tsavo National Park. The status of wildlife and livestock in the rangelands now (2011–2016), compared to what they used to be in the early part of the monitoring period (1977–1980), provide a strong justification for continued monitoring of wildlife and livestock numbers and the conditions of their ecosystems as regularly as possible to acquire accurate data. Such data are necessary for at least three reasons. *i*) Early detection of changes of conservation concern. *ii*) Assessing the performance of wildlife and livestock populations. *iii*) Realizing the goals and monitoring the effectiveness of the wildlife Act 2013. Similarly important should be regular monitoring and assessment of poaching and poisoning of wildlife (mainly large carnivores) and availability of small fire arms often used to illegally kill wildlife. Note that poaching still makes a significant but unknown contribution to the declines even though the Kenya Government banned trophy hunting in 1977 to stem large-scale poaching attributed to weak regulation and law enforcement (S9 Table). Our results are also useful in selecting the most promising areas for population recovery and restoration. Specifically, the earliest abundance and distribution data provide invaluable ‘reference’ or ‘baseline’ material to inform recovery strategies. They can be used to determine the levels of wildlife populations the different areas might be restored to. The potential for restoring wildlife in the rangelands is highlighted by the increase in numbers of some wildlife species where wildlife conservancies have been recently established, notably in the Narok, Kajiado, Laikipia and Nakuru Counties of Kenya [78,79,80]. However, where drastic and virtually permanent changes have occurred, for example, in large parts of the Athi-Kaputiei ecosystem in Kajiado County [19], it would be politically, socially and economically too costly to restore wildlife populations.

### Causes of wildlife declines and concurrent increase in livestock numbers

The extreme and widespread wildlife losses are troubling given the enormous resources that have been invested in wildlife management, conservation and protection in Kenya. An important question then is: what causes the relentless, pervasive and catastrophic declines in wildlife numbers and the contemporaneous increase in numbers of sheep, goats, camels and donkeys in the rangelands? Many processes have been proposed to explain the trends. Nevertheless, analyses such as ours relating the trends to their putative underlying causes are rare because of the paucity of monitoring data on population trends and the associated covariates [3,10,11,81]. Here, we discuss the likely causes suggested by our results and earlier studies. The declines suggest the primary involvement of several factors beyond natural climatic or environmental variation, including human activities, policies, institutions, etc. This is because virtually all the common wildlife species declined regardless of the contrasting climatic conditions (semi-arid, arid or very arid) and human population densities prevailing in the counties, or functional groupings of the species based on body size (small, medium, large), gut morphology (ruminant versus non-ruminants) or feeding style (pure grazer, pure browser or mixed grazer-browser).

The first cause of the declines is rapid human population growth and its ramifying effects on the rangelands. For all the wildlife species, an increase in human population density beyond a certain threshold level was associated with a decline in density. This raises the question why did the density of certain wildlife species peak at low but not zero human population density levels? Similarly, why did the density of some livestock species peak at intermediate densities of livestock? Although we do not have the pertinent data to directly address these questions, we speculate that the most likely explanation for both patterns relate to the creation and maintenance of functional resource heterogeneity in the rangelands by the activities of people and their livestock and not the spatial scale of the analyses. For example, livestock create functional heterogeneity in savannas through the concentration of excreta in temporary overnight corrals. These livestock-induced nutrient hotspots (glades) can persist as grazing lawns for decades to centuries and act as sources of above-maintenance levels of forage nutrients for pregnant and lactating wild herbivores [82]. Besides the nutritional benefits, livestock-derived grazing lawns of short grasses can reduce the risk of predation on wild herbivores [82]. In addition, it is possible that wildlife, people and their livestock are selecting the same best places in the rangeland landscapes. Thus, the fact that only Grant's gazelles were apparently significantly influenced by livestock density, may suggest an interaction between human population density and livestock density such that when human population density has been selected into a model, then livestock will tends to be excluded from the model.

Hence, the documented exponential human population growth can be expected to accelerate wildlife losses. Kenya’s human population grew nearly five-fold from 8.1 million in 1960 to 44.4 million in 2013 and at an average annual rate of 2.9% in 2013 [83]. The rapid population increase is also occurring in the rangelands and is forecasted to continue in the coming years [84]. For example, Kenya’s pastoral population increased 4.8-fold between 1962 and 2009 and was projected to double between 1990 and 2015 [84]. The second cause is rangeland degradation and fragmentation. Associated with the rising population pressures in the pastoral regions are browning trends in vegetation condition, signalling progressive rangeland degradation or loss [84–87]. Such rangeland habitat degradation, fragmentation and loss are usually attributed to land use and cover changes. The latter changes are linked to unregulated expansion of settlements and agriculture into the rangelands [88,89], intensification of land use and persistent grazing. The changes are also linked to unsuitable rangeland management, unregulated wood harvesting for firewood and charcoal trade, uncontrolled livestock stocking levels leading to overgrazing, unregulated spread of settlements, including urban centres and infrastructural development [19,20,26,90,91].

The third cause of the wildlife losses is climate change and variability, manifested by declining rainfall and striking rise in the minimum maximum temperatures, which jointly amplify the effects of land use and cover changes and other factors on wildlife populations and their habitats. The declining rainfall and rising temperature are likely partly responsible for the vegetation browning trend. There has also been an overall reduction in both the long (March-May) and short (November-December) rainy seasons, an increase in spatial and temporal variability of rainfall and increased frequency of droughts in East Africa in recent years [92–94]. Both livestock and wildlife alike suffer mass mortality due to starvation and heightened depredation during recurrent severe droughts [20,95–97]. The trend towards increasing frequency and intensity of droughts and aridification and rising temperatures is apparently negatively affecting tall grasses favoured by cattle more than short grasses favoured by sheep and goats and browse favoured by goats and camels, consistent with the expectation that the relative biomass of cattle should be greater in wetter areas and that of sheep and goats in arid areas [98]. Thus, the change in herd structure from cattle to camels, sheep and goats by the pastoralists is probably an adaptation to rangeland degradation linked to intensification of land use and sendentarization of the formerly semi-nomadic pastoralists [99–101]. The drying up of the rangelands further accelerates the adoption of camels, sheep and goats by the pastoralists because they are less vulnerable to droughts and suffer relatively less mortality due to starvation and dehydration during droughts than do cattle. Camels are hardy and better adapted to life in the arid and semi-arid rangelands than are cattle because they are less water dependent and are browsers and thus less influenced by rainfall fluctuations than grazers or mixed feeders. Camels also increase the flexibility of managing cows [97,102,103]. Moreover, cattle take much longer to recover from droughts because of their longer gestation and maturity times than do sheep and goats which are also capable of multiple births and have high market demand [26]. High livestock mortality during droughts is associated with frequent incidences of cattle rustling in the rangelands. Thus, shoats are also thought to be getting increasingly preferred to cattle because they cannot move over long distances and hence are less likely to be stolen than are cattle. The increase in camel numbers in the rangelands is also partly due to strong promotion by the government and Non-Governmental Organizations (NGOs) in recent times [26]. Since high livestock population density is associated with reduced wildlife population density, the growing number of livestock is the fourth contributor to the wildlife losses. Counties with larger proportions of their areas under wildlife conservation tend to have higher wildlife population density, hence the area of a county designated for conservation is the fifth correlate of the wildlife losses.

The sixth cause of the tragic wildlife losses in the Kenyan rangelands are major policy, institutional and market failures [104,105]. For centuries East African rangelands were sparsely populated by pastoralists who moved around seasonally with their livestock tracking changes in rainfall to obtain forage for their livestock [106]. But changes in government land policies and rapid population growth progressively discourage pastoralism and promote privatization of land tenure, land subdivision, sedentarization, cultivation and diversification of livelihood options, resulting in intensification of land use, habitat degradation, fragmentation and loss [16,20,107,108]. Agricultural development policies that promote farming in the wetter margins of the rangelands exacerbate the destruction of wildlife habitats and exclusion of wildlife [104,105,109]. As a result, wildlife and livestock ranges are contracting, their seasonal mobility is becoming constrained and wildlife are being displaced or excluded from the pastoral lands and becoming increasingly confined to the few protected areas (Fig 1). The lack of meaningful economic benefits to poor pastoral landowners who bear the costs of supporting wildlife on their lands without ownership or use rights over wildlife, or compensation for lost economic opportunities, damage to private property, injury or deaths further aggravate the declines [110,111]. Most of the wildlife revenue accrues to the government or to the tourism industry. The absence of a government wildlife management agency in the rangelands since the Kenya Wildlife Service (KWS), the official body mandated to manage and protect wildlife, focuses primarily on the protected areas, also contributes to the declines (S9 Table).

The seventh cause of the declines is escalating human-wildlife and land use conflicts and poaching associated with the increasing human population size and expansion of settlements and cultivation into the rangelands [23,89,112–115]. Conflicts arise when wildlife damage crops [116], water works and fences, injure or kill livestock and people, or transfer diseases to livestock. Conflicts also arise when people kill wildlife, encroach onto or destroy wildlife habitats. Poaching, persistent illegal livestock incursions into protected areas, deepening human-wildlife conflicts, competition between wildlife and livestock for forage and between livestock, people and wildlife for water and space, restricted or blocked wildlife access to water, or seasonal dispersal and migratory movements accelerate the declines [7,16,117]. Wildlife are harassed and displaced by livestock, people, vehicles and dogs in pastoral lands. Wildlife almost invariably lose the conflicts with humans, become displaced by development and land uses incompatible with conservation and increasingly confined to the few protected areas. The protected areas are generally isolated and too small to supply the year-round requirements, or ensure the long-term viability of their current wildlife populations without seasonal or year-round access to the neighbouring rangelands [77,118]. The resilience and persistence of wildlife species in the non-protected rangelands will thus depend most strongly upon their susceptibility to human disturbance, proneness to displacement by livestock and humans, preference as a source of bush meat, the size and degree of isolation of the landscapes [77].

The extreme declines raise very grave concerns about wildlife management, conservation policies and practices in Kenya. They provide compelling evidence of the need for a far reaching, far-sighted and urgent review of the implementation of the wildlife management and conservation policies, strategies and practices in the protected areas and unprotected rangelands. The trends show, in particular, that the current model of wildlife conservation focused on state parks and reserves that jointly cover a mere 8% of the land area of Kenya yet having no adequately funded official institutions in charge of conserving and managing wildlife on the private and communal rangelands that span about 88% of Kenya and support 65–70% of Kenya’s wildlife has clearly and understandably failed to protect Kenya’s wildlife.

Given the inadequacy of the national parks and reserves to sustain their contemporary populations of the large mammals and the diminishing opportunities for expanding existing parks and reserves, it is imperative to invest in effectively conserving wildlife and their habitats in the privately or communally owned or used pastoral rangelands [119]. Much of the biodiversity in the rangelands will continue to be lost at even faster rates unless active and effective conservation programs are urgently instituted [77] because of the escalating land use and cover changes, population and other pressures in the rangelands and impinging on protected area boundaries. Policies that integrate national, private and community conservation initiatives are thus more likely to be successful in sustaining wildlife populations and extensive pastoralism at ecosystem, landscape and regional scales.

A juxtaposition of wildlife trends in Kenya and South Africa (S2 and S3 Tables) further reinforces the argument that the real underlying cause of wildlife declines in Kenya is policy, institutional and market failures. In the same period that wildlife declined in Kenya, wildlife in several or most southern African countries have increased—but only where opportunities, rights and responsibilities for wildlife conservation have been fully devolved to private landholders and communities. This devolution was based on two bold policies: 1) devolving landholding /proprietorship to landholders or communities and 2) maximizing the value of wildlife by allowing a full range of uses unrestricted by various bans and minimizing the cost of and conflicts with wildlife, with the big contrast with Kenya being that hunting is allowed outside parks. Most of the problems in Kenya therefore emanate from a bigger overall problem which can be succinctly summarized as follows. Kenya is flipping very rapidly from being in an “empty world” to being in a “full world” but the institutions for managing wildlife, and indeed wildlife range, in a full world have not correspondingly evolved [120]. Consequently, centralized and/or open access property regimes put in place by colonials and reinforced by post-colonial governments are the order of the day–the status quo benefits the elites in the system [108,121,122], creating resentment among local communities.

In this tragedy of the commons, which has become a common tragedy in the Kenya rangelands, individually owned and hardy species (such as domestic goats, sheep, donkeys and camels) replace wildlife which is not owned and therefore needs to be managed collectively and at scale. Numbers of the individually owned livestock species are also not regulated. They individually owned and hard livestock species also replace cattle for the ecological reasons suggested above. In other words, the livestock species that are owned replace the wildlife species that are not owned. As well, hardy livestock species replace the less hardy livestock species. The replacement of wildlife by livestock is exacerbated because restrictions on the use of wildlife are made easy because they are owned by the state and not by the landholders or users in the rangelands, and this underprices wildlife. The net result is that the competitive advantages of multi-species wildlife systems with multiple values (tourism, hunting, biodiversity, ecological adaptability, ecosystem services) are replaced by individually owned commodity production systems in the form of cattle, shoats, donkeys and camels. Wildlife typically earns far less on the rangelands than it should, and most of what it earns is captured by the elites [123]. The solution should therefore entail creation of new ownership systems for wildlife that (1) maximise their value to land holders or communities (and eliminates arbitrary bans, crippling bureaucracy, etc.) and (2) ensure that wildlife benefit gets to the people who live with and support wildlife on their lands–the price proprietorship hypothesis [124]. Various such systems are being tested in southern Africa, and they need to cope with the challenges of retaining local equitable benefit sharing by scaling down, while also building ecologies and economies of scale–i.e., private ownership coupled with conservancies, Community Based Natural Resource Management (CBRM) coupled with micro-participation.

### What should be done to stop the wildlife declines?

Since policy, institutional and market failures are at the heart of wildlife declines in Kenya, we examine important gaps in the current wildlife conservation and management policy which need to be addressed to stem the wildlife losses. To be successful, efforts aiming to slow down or halt the declines and restore the depleted wildlife populations and the degraded rangelands must address the twin crux issues: what is wildlife beneficial for and who mainly benefits? Such efforts must also account for the possibility that large areas of East Africa will inevitably pass over to more lucrative activities, as has happened, for example, in South Africa, which no longer has any counterpart of subsistence pastoralism. Counteracting this progression will require that some pastoral lands retaining wildlife should be buffered against such changes to ensure that they deliver the multiple benefits that they provide sustainably. This demands a far-sighted land-use plan to secure wildlife habitats from the impacts of the rapidly expanding human and livestock populations. Such a plan would benefit from incorporating the biosphere concept of a protected core area enlarged by a multi-use buffer zone with compatible activities.

As the future role of wildlife has become a leading issue globally it is not surprising that different countries are following different routes in search for solutions, including (1) laissez-faire as traditionally prevalent in Kenya, (2) multiple economic uses including hunting, as in Tanzania and earlier in Botswana, (3) devolvement of full financial control to local communities, as in Namibia, (4) fenced protected areas as tourist attractions or living museums, as in South Africa, (5) private ownership in fenced ranches or conservancies, as in South Africa, and (6) transfrontier protected areas, consisting of a mosaic of wildlands and settlements. Despite the diversity of these approaches, the basic issues confronting all countries with wildlife are primarily those of land ownership and devolvement of financial benefits. A crucial need is thus for part of the benefits of protected areas and conservancies to filter down to impoverished neighbours.

Although East Africa still supports the richest herds of wildlife on earth, our analysis shows that the future of Kenyan wildlife is in serious jeopardy without urgent, far-reaching and far-sighted changes to their current conservation and management. The new Act [53] therefore not only restores some badly needed hope but also recognizes that for much of Kenya, environmental imperatives have progressed far beyond ‘conservation’ to ‘recovery’ and ‘restoration’. However, for the current wildlife Act to mark a significant turning point in wildlife trends in Kenya several additional steps, including the following would have to be taken. 1) Careful planning and regulation and effective implementation of the provisions of the wildlife Act are needed to minimize adverse impacts of many large development projects on wildlife conservation and livestock production in the rangelands. These include developments of major infrastructure, urban expansion, exploration and mining activities, expansion of cultivation to the wetter rangeland margins, including irrigation schemes in ecologically sensitive and important wildlife habitats and land fragmentation in the rangelands. Planning, regulation and effective law enforcement should consider the following at the very least. 2) Zoning and demarcating development, wildlife conservation and livestock areas and effectively managing and protecting wildlife and their habitats, including dispersal and migratory routes. 3) Vigorously implementing effective and coherent land use policies and legislations to avert or minimize human-wildlife conflicts, habitat degradation, fragmentation and loss by planning and regulating the spread of settlements, fences, cultivation, and annexation of water resources in the rangelands for farms, towns and other uses to the detriment of wildlife. 4) Promoting compatible land uses in the same landscape would also help reduce human-wildlife conflicts and negative attitudes towards wildlife [125,126]. 5) Wildlife conservation and management, legislation, regulations and policies should be harmonized with those governing pastoralism, water use, forest and environmental protection, land use and disposal of toxic wastes in the rangelands and the activities in these sectors more tightly coordinated.

One of the hallmarks of the new Wildlife Conservation and Management Act 2013 is that it promotes private and community conservation and transition from open-access to private property regimes. It thus provides a framework within which communities can be empowered to use, manage and receive expanded economic benefits from wildlife [122,126]. Greater benefits enhance the importance of wildlife as a component of livelihoods and development, help pay the costs of conservation and reduce human-wildlife conflicts. Yet, widespread poverty and inequality still deny many landowners the opportunity to benefit from wildlife. This reduces interest and investment in conservation because, understandably, attitudes of people towards conservation on private or communal lands are often shaped by the amount and distribution of financial benefits from supporting wildlife on their lands [127]. Communities getting no benefits from wildlife and having little say in national policy, as most pastoralists are, are more likely to be more intolerant to wildlife.

Although initially started by individuals and communities in a policy vacuum, wildlife conservancies have had some tangible success in Kenya, associated with direct economic benefits to poor landowner households, poverty alleviation, rising land values and increasing wildlife numbers within the conservancies [80, 128]. As a result, conservancies are fast emerging as the centrepiece of natural resource conservation on the rangelands and broader development institutions for championing community development projects around the conservancies and ensuring sustainability through land use planning, managing wildlife, livestock, rangelands, and forests, trading in conservation beef, organic products or carbon—because traditional institutions have collapsed in the pastoral lands. Community conservation in conservancies is also important in complementing limited capacity and skills of state agencies and dwindling state resources for conservation in the wake of mounting conservation challenges [24].

### Important wildlife policy gaps that should be addressed to stop the declines

Here, we highlight some root causes of wildlife declines that are not adequately addressed by the current wildlife conservation policy and hence need to be urgently addressed (S9 Table). It is crucial to regulate livestock stocking levels to limit the number of livestock that can be reared on the available rangelands in conservancies, or ranches to minimize rangeland degradation through overgrazing. Reducing livestock stocking levels is also important to ensuring economic viability and sustainability of wildlife conservation on the human and livestock dominated pastoral lands. High livestock stocking levels are associated with declines in large mammalian species richness, abundance and distribution [129]. Regulating livestock stocking levels will also help ensure that pastoralists do not regularly move increasingly large livestock herds to conservancies, parks and reserves, as currently happens [130]. As most ordinary pastoralists still earn more from livestock than wildlife, it is crucial to maintain some balance between conservancies and livestock, make and enforce rules that control livestock grazing in conservancies. These measures will ensure that communities benefit from wildlife without necessarily having to sacrifice all their current major livelihood—livestock. However, policies that can guide the development of models for optimally integrating livestock and wildlife in conservancies to ensure economically viable conservancies on pastoral lands rather than completely separating pastoral livestock and wildlife, especially in areas with low tourism potential, are still lacking. Although there are some benefits to be gained by not completely separating wildlife from livestock in conservancies, including mutually beneficial long-term modifications of rangeland habitats [82,131], livestock grazing and herd size in conservancies should be regulated and monitored. This is especially important because a major problem for conservancies currently is that some pastoral land owners benefitting from conservancies use their incomes to buy more livestock that then compete with wildlife and degrade rangeland habitats. Equally important to regulate and monitor to stem widespread destruction of woodland habitats is clear felling of woodlands for charcoal trade, fuel wood, fencing, and construction materials in pastoral lands.

Conservancies are critical in creating more space for conserving biodiversity and ecological services outside state protected areas and buffering protected areas from growing human impacts pressing on their edges. However, the necessary regulations, access rights, arrangements for fair and transparent benefit sharing with communities living in wildlife areas [132] and other incentives necessary for their success are still lacking. There is thus a need to build community capacity in wildlife conservation, management and protection, conservation planning, effective leadership, security operations, conservation business enterprises, technical and negotiation skills, access to information, democratic and effective collective or collaborative action [126,129,133,134]. Without such skills local communities cannot meaningfully be involved in the management, tourism development and control of tourism resources in conservancies [135], or in ensuring that the benefits of conservancies outweigh the costs. Yet the participation and support of pastoral land owners is critical to the success of conservancies because they have to vacate their lands for conservancies, refrain from erecting fences and other developments. Wildlife conservation policy should also recognize that wildlife is not just a Kenyan heritage but a global heritage, conferring upon Kenya both global and local responsibilities that need funding for conservation and habitat restoration.

Lastly, there is a need to strengthen collaborative natural resource conservation and management partnerships between governmental agencies, conservation organizations, the private sector and pastoral communities, enhance community participation, and encourage and support investment initiatives that enhance socio-economic development and wildlife revenue flowing to communities. Wildlife policy should embrace a strong paradigm shift away from the past and current bureaucratic uncertainty, crippling restrictions on use, and extracting most wildlife revenues from community areas. Wildlife policy should also do away with state nationalization, monopolization and centralization of wildlife and grant local communities responsibility and authority over local conservation decisions within a wider and carefully crafted framework of accountability, regulation and governance [109,126,136,137].

## Supporting Information

S1 Data The distribution of the 361 aerial surveys carried out by the Directorate of Resource Surveys and Remote Sensing (DRSRS) between 1977 and 2016 across the 21 rangeland counties of Kenya. For completeness, surveys covering sections of counties conducted in Masai Mara Ecosystem, Athi-Kaputiei Ecosystem, Tsavo National Park, Tsavo Ecosystem, Taita Hills, Gala Ranch, and Northern Kenya are also provided. The Kitui 2011 survey represents merged 2011 and 2012 surveys. The Taita Hills Survey of 1981 covered parts of Tana Delta, Lamu, Garissa and Malindi regions. The Northern Kenya Survey of 1981 focused on Grevy's zebra in Baragoi, Marti, and South Horr regions. The Tsavo Ecosystem survey for 1986 covered only Tsavo East National Park.
(XLSX)

S2 Data The dates on which aerial surveys were carried out by the Directorate of Resource Surveys and Remote Sensing (DRSRS) between 1977 and 2016, length of sampling units in km, distance between consecutive transects in km, population of sampling units, number of sampled units, number of transects flown, sampled strip width in meters, percentage of target area sampled (sampling fraction) and target area in km^2^. (XLSX)

S3 Data Population estimates for individual species that were excluded from the trend models but included in plots of trend patterns because they were considered as outliers. The actual calendar year and month of survey represented by the survey code can be found in S2 Data.
(DOCX)

S4 Data The last date on which aerial surveys were carried out between 1977 and 2016, taken as the date of survey in the trend models, the total area of each county in km^2^, the actual number of animals of each species counted, the population size of each species and its associated standard error estimated by Jolly’s method 2 and the corresponding population size estimate derived from the multivariate semiparametric generalized linear mixed model for trends and its pointwise lower and upper 95% confidence limits. Population estimates for Kwale and Kilifi 1983 were not included in model fitting because the survey covered only 0.81% of each of the two counties.
(XLSX)

S5 Data The aerial survey team at the Directorate of Resource Surveys and Remote Sensing between 1977 and 2016, including the Rear Seat Observers (RSO), Front Seat Observers (FSO), pilots, directors, senior biologists and data technicians. (XLSX)

S6 Data The total area of each county (km^2^), the total area of game parks and reserves in each county in km^2^ and as a percentage of the total area of the county. (XLSX)

S7 Data Some private, communal or state owned wildlife conservancies, conservation areas, ranches and sanctuaries located within the 21 rangeland counties. These conservation entities were not used in the model relating animal counts to covariates because their start dates were either too recent or unknown or their actual areal sizes were unknown. For some of the entities it is unclear whether they are being currently actively managed for biodiversity conservation.
(XLSX)

S8 Data The total area (km^2^) and human population size in each of Kenya’s 47 counties between 1962 and 2009 based on decadal censuses and interpolation. **The censuses were carried out by the Kenya National Bureau of Statistics**. Population density is calculated as the number of people / km^2^.
(XLSX)

S9 Data The total annual rainfall averaged across all the 5 km × 5 km grid cells in each of Kenya’s 47 counties for 1960–2014. The rainfall measurements were derived from the GeoCLIM software tool developed by the USGS FEWS NET for the USAID PREAPRED project which blends station rainfall data with satellite rainfall data.
(XLSX)

S10 Data Average monthly maximum and minimum temperature data for all the 47 counties of Kenya. The temperature measurements were derived from the GeoCLIM software too developed by the USGS FEWS NET for the USAID PREAPRED project (USAID) which blends station temperature data with satellite temperature data. Annual averages of the maximum and minimum temperatures were used in the model relating animal counts to covariates.
(XLSX)

S1 Fig Pictures of 17 of the 18 studied wildlife species (Burchell’s zebra, buffalo, elephant, ostrich, wildebeest, Masai giraffe, reticulated giraffe, gerenuk, Grant’s gazelle, warthog, Lesser kudu, Thomson’s gazelle, eland, oryx, topi, hartebeest, impala, Grevy’s zebra, and waterbuck. Photo Credit: Reto Buehler took all the photos except the photos of Thomson’s gazelle, Grant’s gazelle and hartebeest that were taken by Niels Mogensen.
(PDF)

S2 Fig Temporal trend in the population size (dark magenta filled circle) of each of the common livestock (sheep and goats, donkey and cattle) and wildlife (Burchell’s zebra, buffalo, elephant, ostrich, wildebeest, giraffe, Grant’s gazelle, warthog, Thomson’s gazelle, eland, topi, hartebeest, impala and waterbuck) species in Narok County between 1977 and 2016. The solid red line is the fitted trend curve and the shaded chartreuse band is the pointwise 95% confidence band. The estimated average population size in 1977–1980 and 2011–2016 and the percentage change in population size between the two periods are provided in the inset.
(PDF)

S3 Fig Temporal trend in the population size (dark magenta filled circle) of each of the common livestock (sheep and goats, donkey and cattle) and wildlife (Burchell’s zebra, buffalo, elephant, ostrich, wildebeest, giraffe, gerenuk, Grant’s gazelle, warthog, Lesser kudu, Thomson’s gazelle, eland, oryx, topi, hartebeest, impala, and waterbuck) species in Kajiado County between 1977 and 2014. The solid red line is the fitted trend curve and the shaded chartreuse band is the pointwise 95% confidence band. The estimated average population size in 1977–1980 and 2011–2014 and the percentage change in population size between the two periods are provided in the inset.
(PDF)

S4 Fig Temporal trend in the population size (dark magenta filled circle) of each of the common livestock (sheep and goats, donkey and cattle) and wildlife (Burchell’s zebra, buffalo, ostrich, wildebeest, giraffe, Grant’s gazelle, Thomson’s gazelle, eland, oryx, hartebeest, and impala) species in Machakos and Makueni Counties combined between 1977 and 2015. The solid red line is the fitted trend curve and the shaded chartreuse band is the pointwise 95% confidence band. The estimated average population size in 1977–1980 and 2011–2015 and the percentage change in population size between the two periods are provided in the inset.
(PDF)

S5 Fig Temporal trend in the population size (dark magenta filled circle) of each of the common livestock (sheep and goats, camel, donkey and cattle) and wildlife (Burchell’s zebra, buffalo, elephant, ostrich, giraffe, gerenuk, Grant’s gazelle, warthog, Lesser kudu, Thomson’s gazelle, eland, oryx, topi, hartebeest, impala, and waterbuck) species in Kitui County between 1977 and 2015. The solid red line is the fitted trend curve and the shaded chartreuse band is the pointwise 95% confidence band. The estimated average population size in 1977–1980 and 2011–2015 and the percentage change in population size between the two periods are provided in the inset.
(PDF)

S6 Fig Temporal trend in the population size of each of the common livestock (sheep and goats, donkey and cattle) and wildlife (Burchell’s zebra, buffalo, elephant, ostrich, giraffe, gerenuk, Grant’s gazelle, warthog, Lesser kudu, Thomson’s gazelle, eland, oryx, topi, hartebeest, impala and waterbuck) species in Taita Taveta County between 1977 and 2014. The solid red line is the fitted trend curve and the shaded chartreuse band is the pointwise 95% confidence band. The estimated average population size in 1977–1980 and 2011–2014 and the percentage change in population size between the two periods are provided in the inset.
(PDF)

S7 Fig Temporal trend in the population size (dark magenta filled circle) of each of the common livestock (sheep and goats, donkey and cattle) and wildlife (Burchell’s zebra, buffalo, elephant, ostrich, giraffe, gerenuk, Grant’s gazelle, warthog, Lesser kudu, eland, oryx, hartebeest and impala) species in Kwale County between 1977 and 2013. The solid red line is the fitted trend curve and the shaded chartreuse band is the pointwise 95% confidence band. The estimated average population size in 1977–1980 and 2011–2013 and the percentage change in population size between the two periods are provided in the inset.
(PDF)

S8 Fig Temporal trend in the population size (dark magenta filled circle) of each of the common livestock (sheep and goats, donkey and cattle) and wildlife (Burchell’s zebra, buffalo, elephant, ostrich, giraffe, gerenuk, Grant’s gazelle, warthog, Lesser kudu, Thomson’s gazelle, eland, oryx, hartebeest, impala and waterbuck) species in Kilifi County between 1977 and 2013. The solid red line is the fitted trend curve and the shaded chartreuse band is the pointwise 95% confidence band. The estimated average population size in 1977–1980 and 2011–2013 and the percentage change in population size between the two periods are provided in the inset.
(PDF)

S9 Fig Temporal trend in the population size (dark magenta filled circle) of each of the common livestock (sheep and goats, camel, donkey and cattle) and wildlife (Burchell’s zebra, buffalo, elephant, ostrich, giraffe, gerenuk, Grant’s gazelle, warthog, Lesser kudu, Thomson’s gazelle, eland, oryx, topi, hartebeest, impala, Grevy’s zebra and waterbuck) species in Tana River County between 1977 and 2014. The solid red line is the fitted trend curve and the shaded chartreuse band is the pointwise 95% confidence band. The estimated average population size in 1977–1980 and 2011–2014 and the percentage change in population size between the two periods are provided in the inset.
(PDF)

S10 Fig Temporal trend in the population size (dark magenta filled circle) of each of the common livestock (sheep and goats, donkey and cattle) and wildlife (Burchell’s zebra, buffalo, elephant, ostrich, giraffe, gerenuk, warthog, Lesser kudu, eland, topi, impala and Waterbuck) species in Lamu County between 1977 and 2013. The solid red line is the fitted trend curve and the shaded chartreuse band is the pointwise 95% confidence band. The estimated average population size in 1977–1980 and 2011–2013 and the percentage change in population size between the two periods are provided in the inset.
(PDF)

S11 Fig Temporal trend in the population size (dark magenta filled circle) of each of the common livestock (sheep and goats, camel, donkey and cattle) and wildlife (Burchell’s zebra, ostrich, gerenuk, Grant’s gazelle, warthog, Lesser kudu, eland, oryx, impala and waterbuck) species in Baringo County between 1977 and 2013. The solid red line is the fitted trend curve and the shaded chartreuse band is the pointwise 95% confidence band. The estimated average population size in 1977–1980 and 2011–2013 and the percentage change in population size between the two periods are provided in the inset.
(PDF)

S12 Fig Temporal trend in the population size (dark magenta filled circle) of each of the common livestock (sheep and goats, camel, donkey, cattle) and wildlife (Burchell’s zebra, buffalo, elephant, ostrich, giraffe, gerenuk, Grant’s gazelle, warthog, Lesser kudu, Thomson’s gazelle, eland, oryx, impala, Grevy’s zebra and waterbuck) species in Laikipia County between 1977 and 2016. The solid red line is the fitted trend curve and the shaded chartreuse band is the pointwise 95% confidence band. The estimated average population size in 1977–1980 and 2011–2016 and the percentage change in population size between the two periods are provided in the inset.
(PDF)

S13 Fig Temporal trend in the population size (dark magenta filled circle) of each of the common livestock (sheep and goats, camel, donkey and cattle) and wildlife (Burchell’s zebra, buffalo, elephant, ostrich, giraffe, gerenuk, Grant’s gazelle, warthog, Lesser kudu, Thomson’s gazelle, eland, oryx, impala, Grevy’s zebra and Waterbuck) species in Samburu County between 1977 and 2015. The solid red line is the fitted trend curve and the shaded chartreuse band is the pointwise 95% confidence band. The estimated average population size in 1977–1980 and 2011–2015 and the percentage change in population size between the two periods are provided in the inset.
(PDF)

S14 Fig Temporal trend in the population size (dark magenta filled circle) of each of the common livestock (sheep and goats, camel, donkey and cattle) and wildlife (Burchell’s zebra, buffalo, elephant, ostrich, giraffe, gerenuk, Grant’s gazelle, warthog, Lesser kudu, eland, oryx, impala, Grevy’s zebra and waterbuck) species in Isiolo County between 1977 and 2015. The solid red line is the fitted trend curve and the shaded chartreuse band is the pointwise 95% confidence band. The estimated average population size in 1977–1980 and 2011–2015 and the percentage change in population size between the two periods are provided in the inset.
(PDF)

S15 Fig Temporal trend in the population size (dark magenta filled circle) of each of the common livestock (sheep and goats, camel, donkey and cattle) and wildlife (Burchell’s zebra, buffalo, elephant, ostrich, giraffe, gerenuk, Grant’s gazelle, warthog, Lesser kudu, eland, oryx, topi, impala, Grevy’s zebra and waterbuck) species in Garissa County between 1977 and 2013. The solid red line is the fitted trend curve and the shaded chartreuse band is the pointwise 95% confidence band. The estimated average population size in 1977–1980 and 2011–2013 and the percentage change in population size between the two periods are provided in the inset.
(PDF)

S16 Fig Temporal trend in the population size (dark magenta filled circle) of each of the common livestock (sheep and goats, camel, donkey and cattle) and wildlife (ostrich, giraffe, gerenuk, Grant’s gazelle, warthog, Lesser kudu, oryx, and Grevy’s zebra) species in Wajir County between 1977 and 2013. The solid red line is the fitted trend curve and the shaded chartreuse band is the pointwise 95% confidence band. The estimated average population size in 1977–1980 and 2011–2013 and the percentage change in population size between the two periods are provided in the inset.
(PDF)

S17 Fig Temporal trend in the population size (dark magenta filled circle) of each of the common livestock (sheep and goats, camel, donkey and cattle) and wildlife (Burchell’s zebra, buffalo, elephant, ostrich, giraffe, gerenuk, Grant’s gazelle, warthog, Lesser kudu, and Oryx) species in Mandera County between 1977 and 2013. The solid red line is the fitted trend curve and the shaded chartreuse band is the pointwise 95% confidence band. The estimated average population size in 1977–1980 and 2011–2013 and the percentage change in population size between the two periods are provided in the inset.
(PDF)

S18 Fig Temporal trend in the population size (dark magenta filled circle) of each of the common livestock (sheep and goats, camel, donkey and cattle) and wildlife (Burchell’s zebra, buffalo, elephant, ostrich, giraffe, gerenuk, Grant’s gazelle, warthog, Lesser kudu, eland, oryx, topi, impala and Grevy’s zebra) species in Marsabit County between 1977 and 2014. The solid red line is the fitted trend curve and the shaded chartreuse band is the pointwise 95% confidence band. The estimated average population size in 1977–1980 and 2011–2014 and the percentage change in population size between the two periods are provided in the inset.
(PDF)

S19 Fig Temporal trend in the population size (dark magenta filled circle) of each of the common livestock (sheep and goats, camel, donkey and cattle) and wildlife (Burchell’s zebra, buffalo, elephant, ostrich, giraffe, gerenuk, Grant’s gazelle, warthog, lesser kudu, eland, oryx, topi, and impala) species in Turkana County between 1977 and 2013. The solid red line is the fitted trend curve and the shaded chartreuse band is the pointwise 95% confidence band. The estimated average population size in 1977–1980 and 2011–2013 and the percentage change in population size between the two periods are provided in the inset.
(PDF)

S20 Fig Temporal trend in the population size (dark magenta filled circle) of each of the common livestock (sheep and goats, camel, donkey and cattle) and wildlife (gerenuk, Grant’s gazelle, warthog, Lesser kudu and impala) species in West Pokot County between 1977 and 2013. The solid red line is the fitted trend curve and the shaded chartreuse band is the pointwise 95% confidence band. The estimated average population size in 1977–1980 and 2011–2013 and the percentage change in population size between the two periods are provided in the inset.
(PDF)

S21 Fig Temporal trend in the population size (dark magenta filled circle) of each of the common livestock (sheep and goats, donkey and cattle) species in Elgeyo Marakwet County between 1977 and 2013. The solid red line is the fitted trend curve and the shaded chartreuse band is the pointwise 95% confidence band. The estimated average population size in 1977–1980 and 2011–2013 and the percentage change in population size between the two periods are provided in the inset. Numbers of all the 18 wildlife species were too few to model trends
(PDF)

S22 Fig Temporal trend in total human population size in each of the 21 rangeland counties of Kenya between 1962 and 2009. Numeric data labels refer to population sizes from decadal censuses were conducted in 1962, 1969, 1979, 1989, 1999 and 2009. Population sizes for the remaining years were obtained using interpolation based on a formula developed by Kenya National Bureau of statistics.
(PDF)

S23 Fig Temporal trends in total annual (January-December) rainfall (mm) in each of the 21 rangeland counties between 1960 and 2014. The filled goldenrod circles denote the observations, the solid magenta curve the fitted trend curve whereas the cadet blue band the pointwise 95% confidence band.
(PDF)

S24 Fig Temporal trends in annual (January-December) average maximum temperature (°C) in each of the 21 rangeland counties between 1960 and 2013. The filled goldenrod circles denote the observations, the solid magenta curve the fitted trend curve whereas the forest green band the pointwise 95% confidence band.
(PDF)

S25 Fig Temporal trends in annual (January-December) average minimum temperature (°C) in each of the 21 rangeland counties between 1960 and 2013. The filled goldenrod circles denote the observations, the solid blue curve the fitted trend curve whereas the chartreuse band the pointwise 95% confidence band.
(PDF)

S26 Fig Relationships between the population density (number/km^2^) of each of the 18 wildlife species and human population density (people /km^2^) in each county during each year of survey. The filled circles are the observations, the solid lines are the quadratic regression lines while the shaded bands are the 95% pointwise confidence bands.
(PDF)

S27 Fig Relationships between the population density (number/km^2^) of each of the 18 wildlife species and the total livestock biomass (kg /km^2^) in each county during each year of survey. The filled circles are the observations, the solid lines are the quadratic regression lines while the shaded bands are the 95% pointwise confidence bands.
(PDF)

S28 Fig Relationships between the population density (number/km^2^) of each of the 18 wildlife species and the percentage of each county under protection (state parks and reserves). The filled circles are the observations, the solid lines are the quadratic regression lines while the shaded bands are the 95% pointwise confidence bands.
(PDF)

S29 Fig Relationships between the population density (number/km^2^) of each of the 18 wildlife species and the total annual rainfall (mm) for each county during each year of survey. The filled circles are the observations, the solid lines are the quadratic regression lines while the shaded bands are the 95% pointwise confidence bands.
(PDF)

S30 Fig Relationships between the population density (number/km^2^) of each of the 18 wildlife species and the annual average maximum temperature (deg C) for each county during each year of survey. The filled circles are the observations, the solid lines are the quadratic regression lines while the shaded bands are the 95% pointwise confidence bands.
(PDF)

S31 Fig Relationships between the population density (number/km^2^) of each of the 18 wildlife species and the annual average minimum temperature (deg C) for each county during each year of survey. The filled circles are the observations, the solid lines are the quadratic regression lines while the shaded bands are the 95% pointwise confidence bands.
(PDF)

S32 Fig Maps showing the distribution of the percentage changes between 1977–1980 and 2011–2016 in numbers of all the livestock and wildlife species across the 21 rangeland counties. (PDF)

S1 File An annotated SAS (version 9.4) GLIMMIX procedures (SAS/STATversion 14.1) codes used to fit the trend model simultaneously to all the species in each County. Separate models were used for wildlife and livestock. The SAS GENSELECT, GLIMMIX and NLIN procedures were used to select and fit models to the covariates most strongly correlated with wildlife population size.
(DOCX)

S1 Table The estimated average population size and proportion of the total rangeland population of each species in each of the 21 rangeland counties in 1977–1980 and 2011–2013. Also shown are the percentage changes in both population size and population proportion between 1977–1980 and 2011–2013 for the common livestock (*n* = 4) and wildlife (*n* = 18) species for each county of the 21 rangeland counties.
(XLSX)

S2 Table Summary of wildlife population trends in Kenya reported by earlier studies. (DOCX)

S3 Table Indicators of wildlife population increase and concurrent declines in livestock numbers in Namibia, Zimbabwe, South Africa and Zambia but substantial increase in livestock numbers and decline in wildlife numbers in Kenya over the same period. (DOCX)

S4 Table Results of statistical tests of significance of the increase in minimum and maximum temperatures and decline in rainfall in all the 47 counties of Kenya during 1960–2014. (XLSX)

S5 Table Selection of the best model (between linear or quadratic) relating the total wildlife biomass (kg/km^2^) and population density (number/km^2^) of each of the 18 wildlife species to human population density (people /km^2^), total livestock biomass (kg/km^2^), total annual rainfall (mm), annual average maximum temperature (°C), annual average minimum temperature (°C) and percentage of each county under protection (parks and reserves). (XLSX)

S6 Table The estimated coefficients of the linear and quadratic regression models relating the total wildlife biomass (kg/km^2^) and population density (number/km^2^) of each of the 18 wildlife species to each of the 6 covariates, the associated standard error and t-test of the null hypothesis that each of the coefficients is equal to zero. (XLSX)

S7 Table Selection of the covariates most strongly related to the population density for each of the 18 wildlife species based on the change in Akaike and Schwarz Bayesian Information Criterion criteria. (XLSX)

S8 Table Regression coefficients of the covariates in the selected best models relating the total wildlife biomass or the population density of each of the 18 wildlife species to the six covariates and their interactions and tests of significance of the coefficients. (XLSX)

S9 Table Summary of the key wildlife policies, institutions and markets in Kenya from 1977 to 2016. Policy, institutional and market failures that have contributed to catastrophic wildlife population declines in Kenya between 1977 and 2016 are highlighted.
(DOCX)
