# Supplementary material for: Extreme Wildlife Declines and Concurrent Increase in Livestock Numbers in Kenya: What Are the Causes?
Source: PLoS One. 2016 Sep 27;11(9):e0163249. doi: 10.1371/journal.pone.0163249 (PMC5039022; doi:10.1371/journal.pone.0163249)
Supplement: S3 Data — The actual calendar year and month of survey represented by the survey code can be found in S2 Data. (DOCX) [file pone.0163249.s003.docx]

**S3 Data**. Population estimates that were excluded from the trend models but included in plots of trend patterns for individual species because they were considered as outliers. The actual calendar year and month of survey represented by the survey code can be found in S2 Data.

| **No** | **County** | **Survey Code** | **Species** |
| --- | --- | --- | --- |
|  | No population estimates available because species was not sighted at all | | |
| 1 | Samburu | 7803, 10M1 | Thomson’s gazelle |
| 2 | Samburu | 8005 | Thomson’s gazelle, Waterbuck |
| 3 | Samburu | 1501 | Buffalo, Thomson’s gazelle, Waterbuck |
|  | Population estimates apparently much higher than expected by the overall trend. Digital records agreed with records on the original paper sheets, suggesting observer errors | | |
| 4 | Narok | 9703 | Impala, Thomson’s gazelle |
| 5 | Narok | 1403 | Burchell’s zebra |
| 6 | Kajiado | 9004 | Elephant |
| 7 | Kajiado | 9104 | Gerenuk |
| 8 | Kajiado | 1106 | Warthog |
| 9 | Taita Taveta | 9104 | Buffalo |
| 10 | Kwale | 8904 | Buffalo |
| 11 | Tana River | 8904 | Grevy’s zebra |
| 12 | Lamu | 8904 | Impala |
| 13 | Laikipia | 9004 | Donkey, Oryx, |
| 14 | Laikpia | 9205 | Elephant |
| 15 | Samburu | 7803, 8005, 85M1, 9303 | Impala |
| 16 | Samburu | 9303 | Camel, Burchell’s zebra |
| 17 | Samburu | 9404 | Elephant |
| 18 | Wajir | 1990M3 | Cattle |
| 19 | Wajir | 9501 | Warthog |
| 20 | Marsabit | 90M1 | Burchell’s zebra |
