# Supplementary figures and images for: Extreme Wildlife Declines and Concurrent Increase in Livestock Numbers in Kenya: What Are the Causes?
Source: PLoS One. 2016 Sep 27;11(9):e0163249. doi: 10.1371/journal.pone.0163249 (PMC5039022; doi:10.1371/journal.pone.0163249)

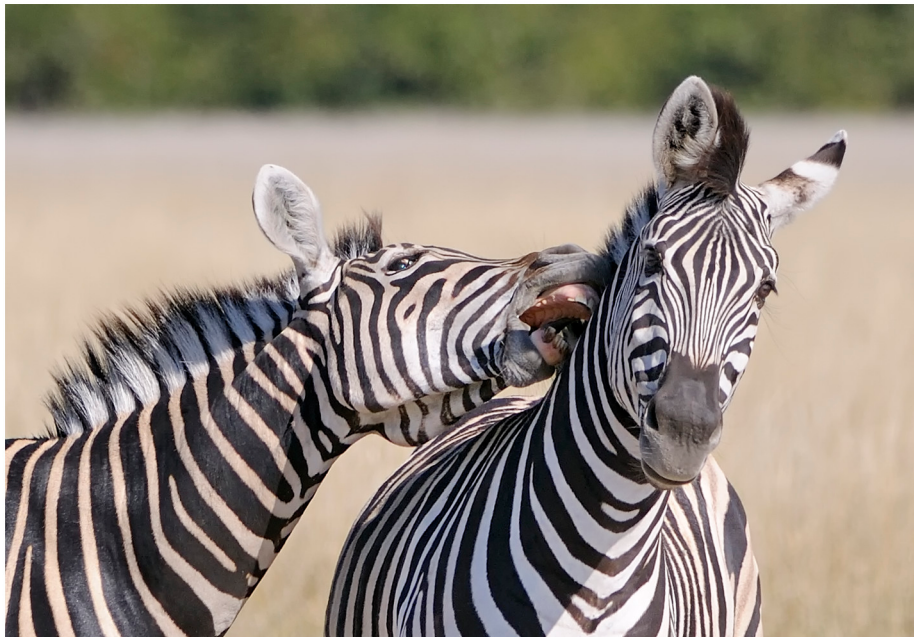

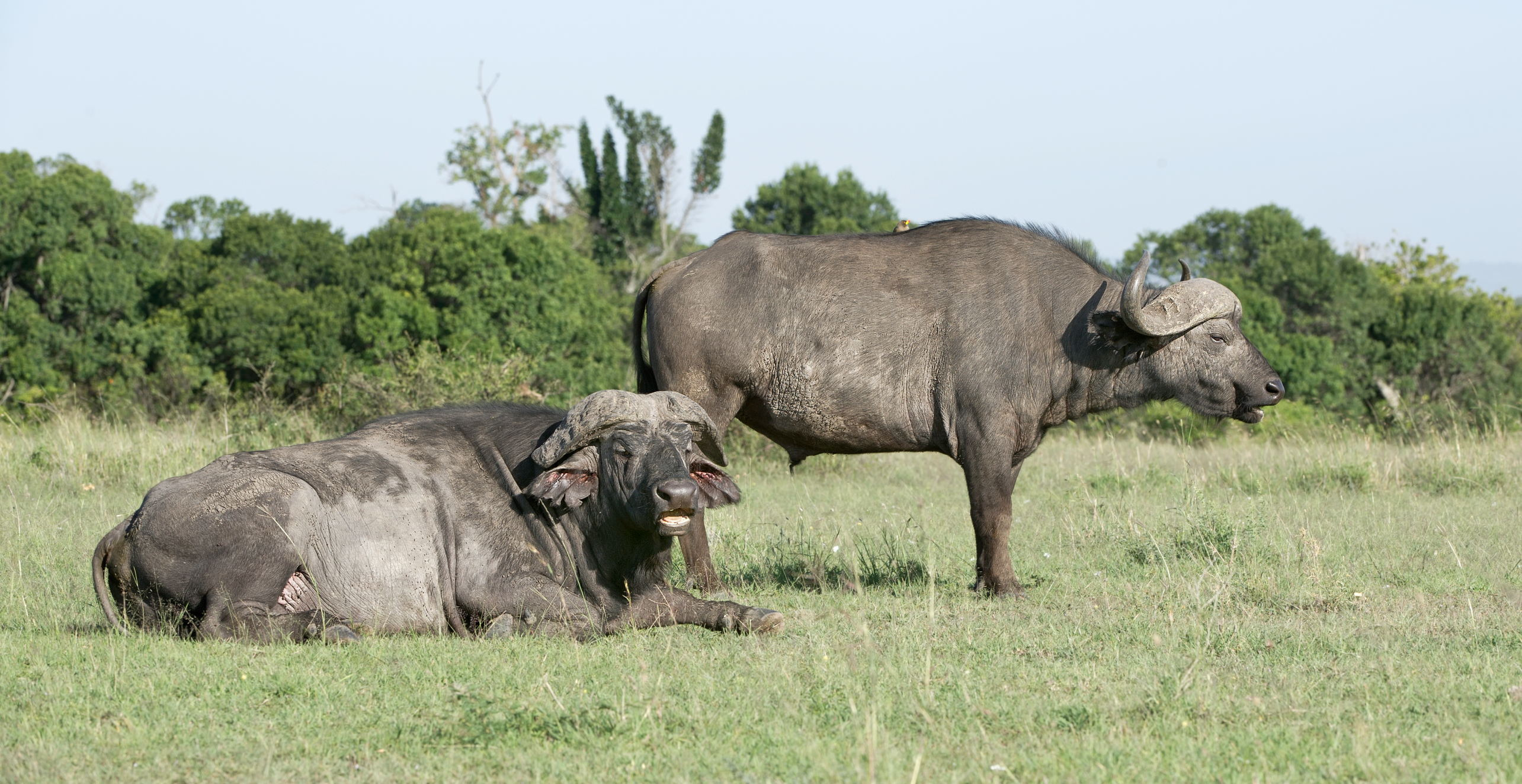

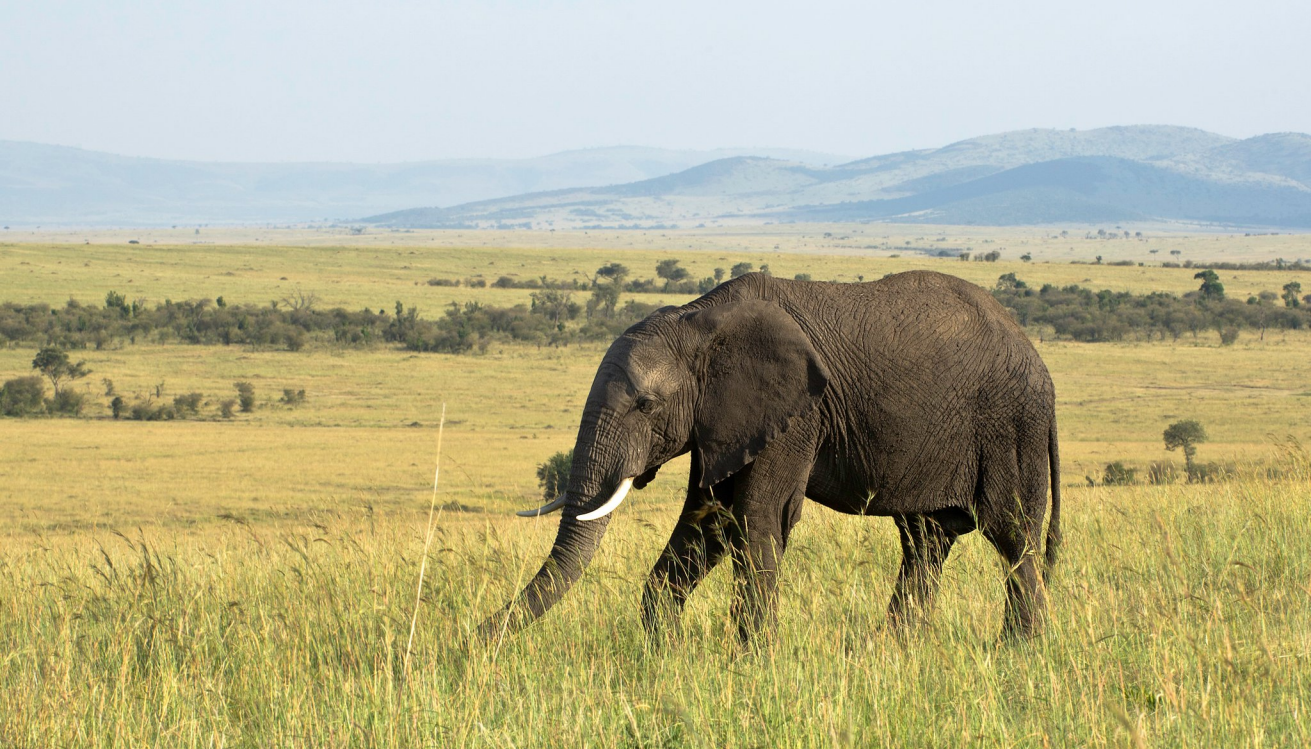

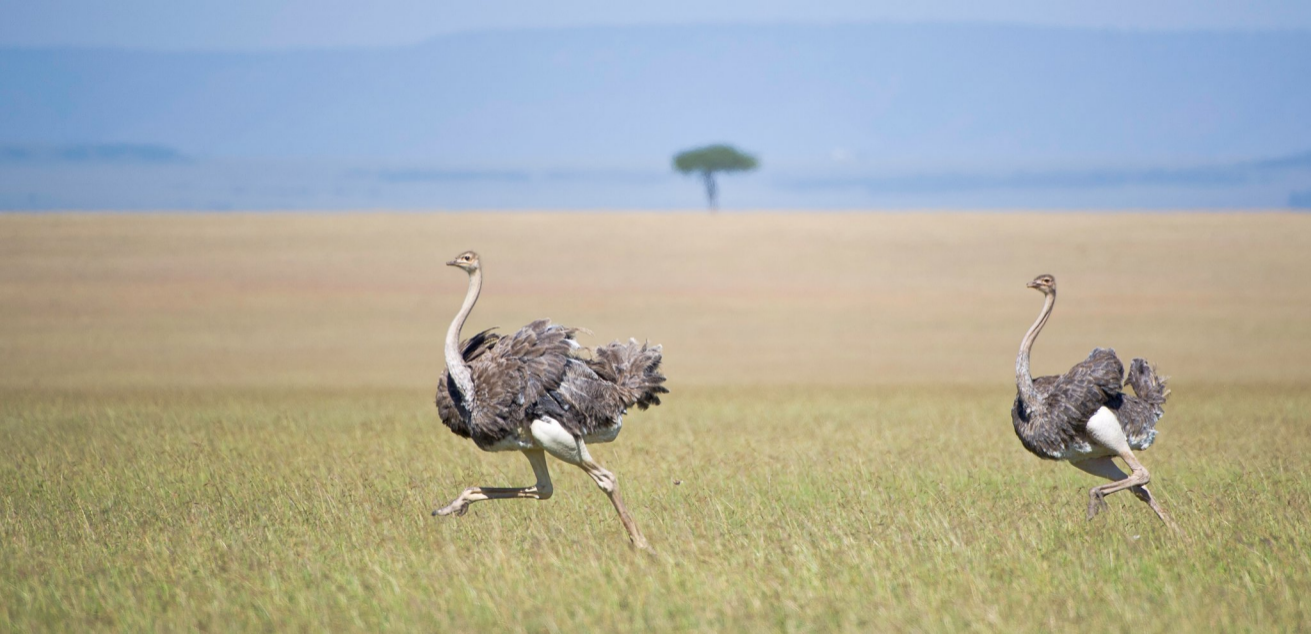

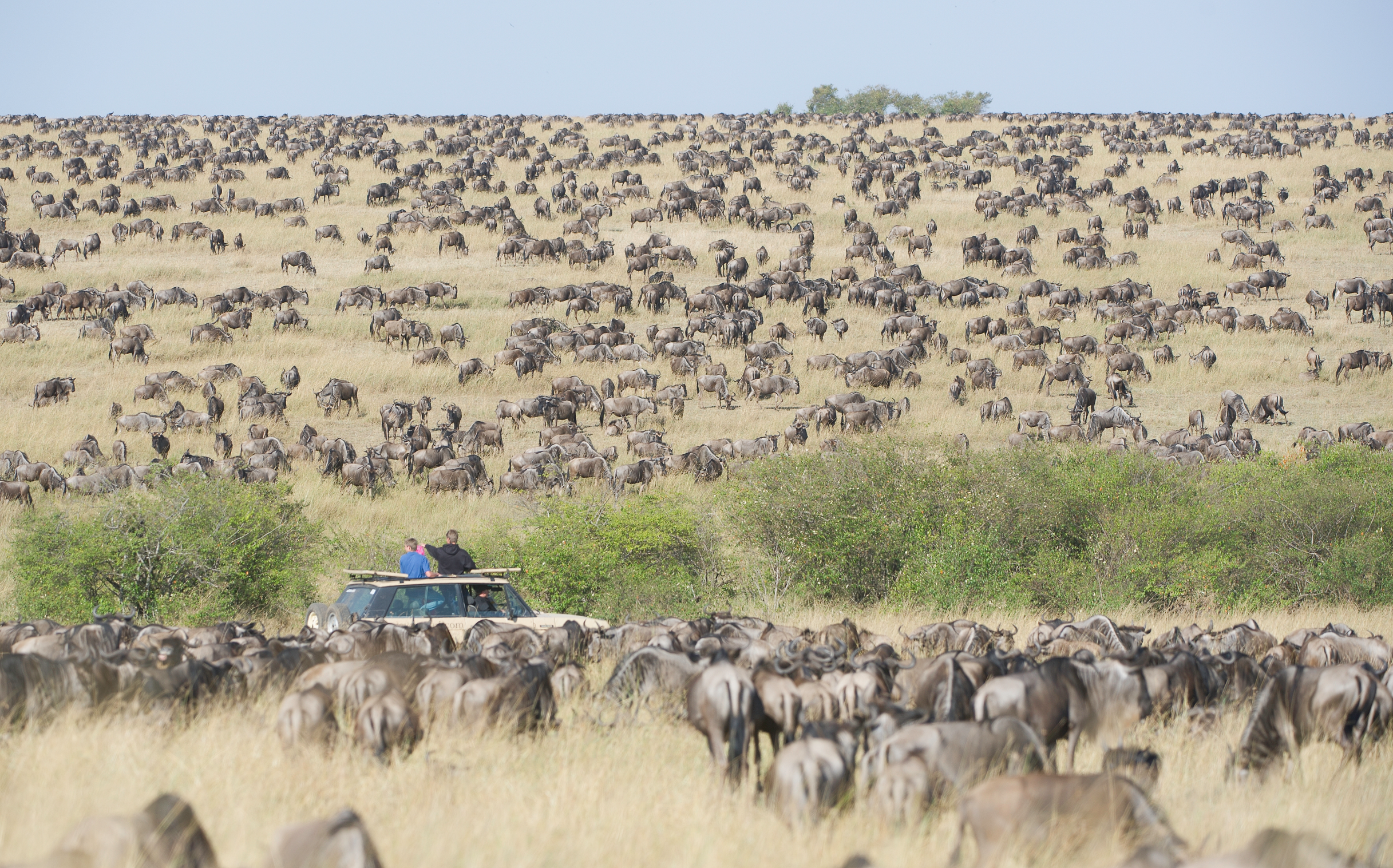

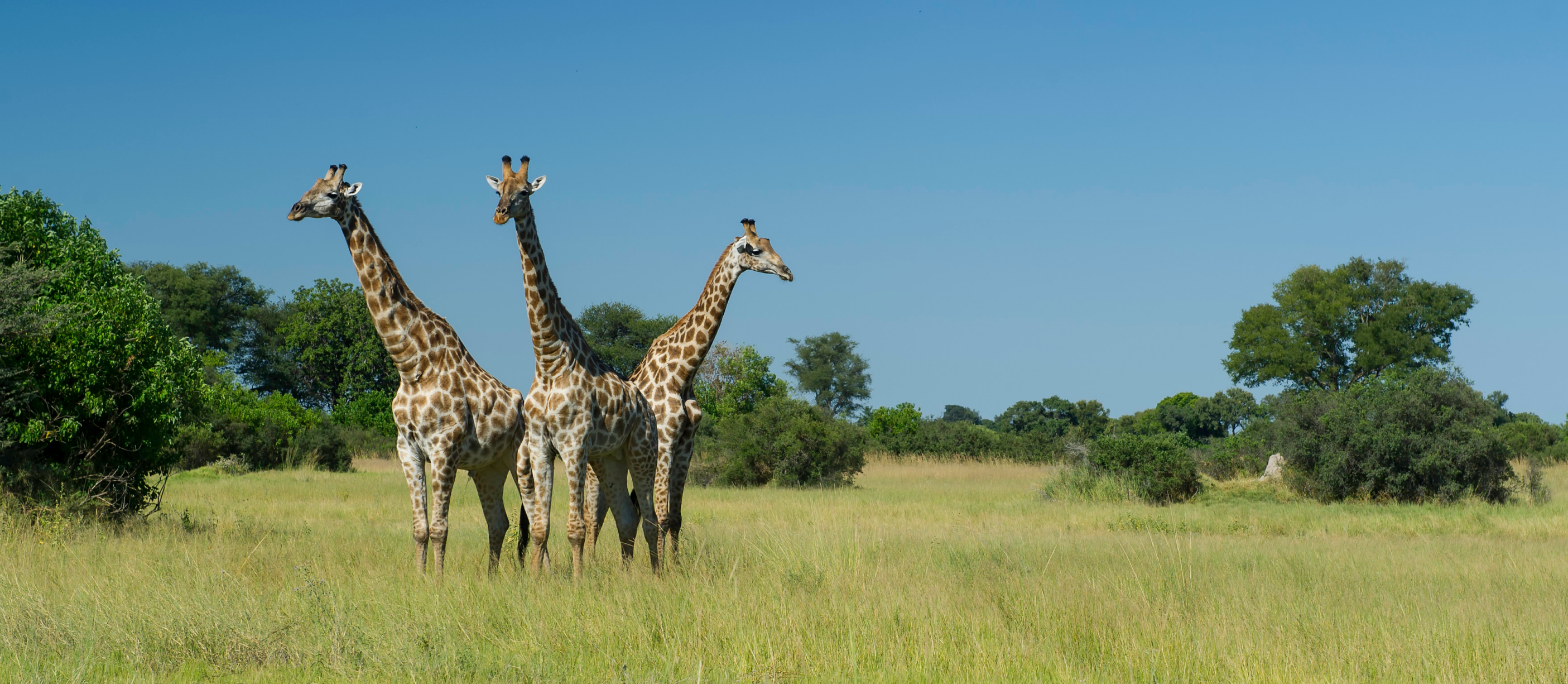

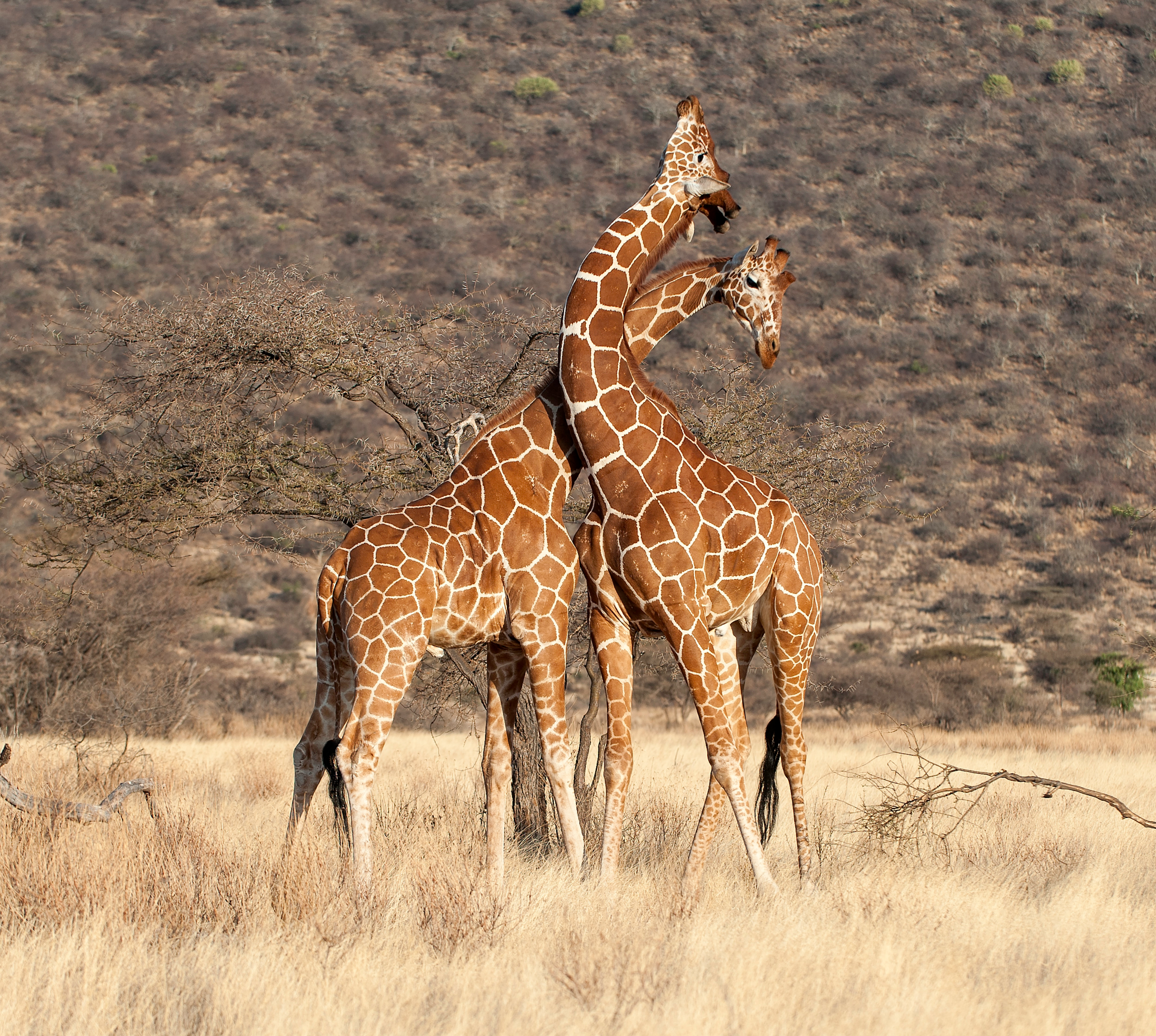

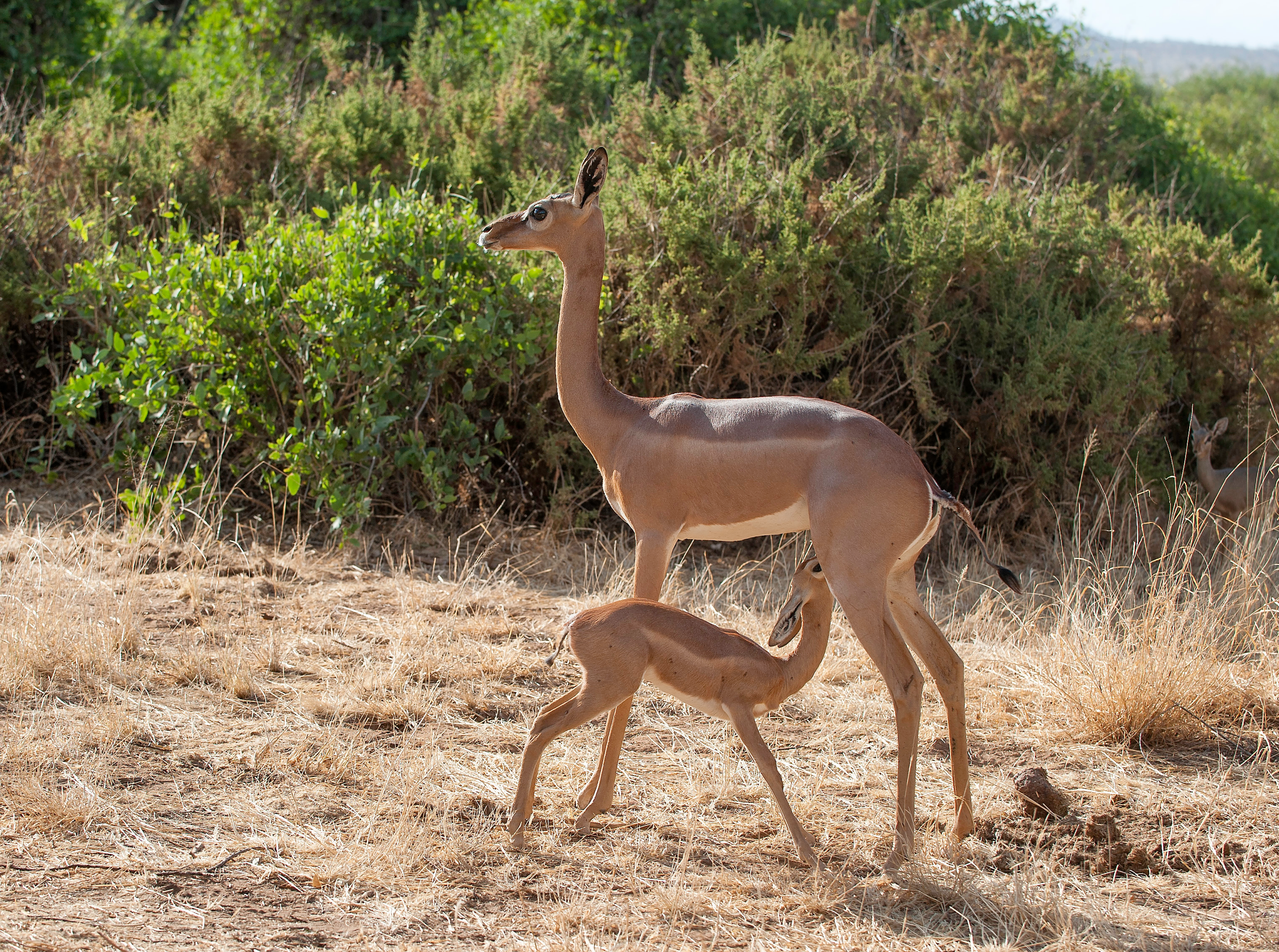

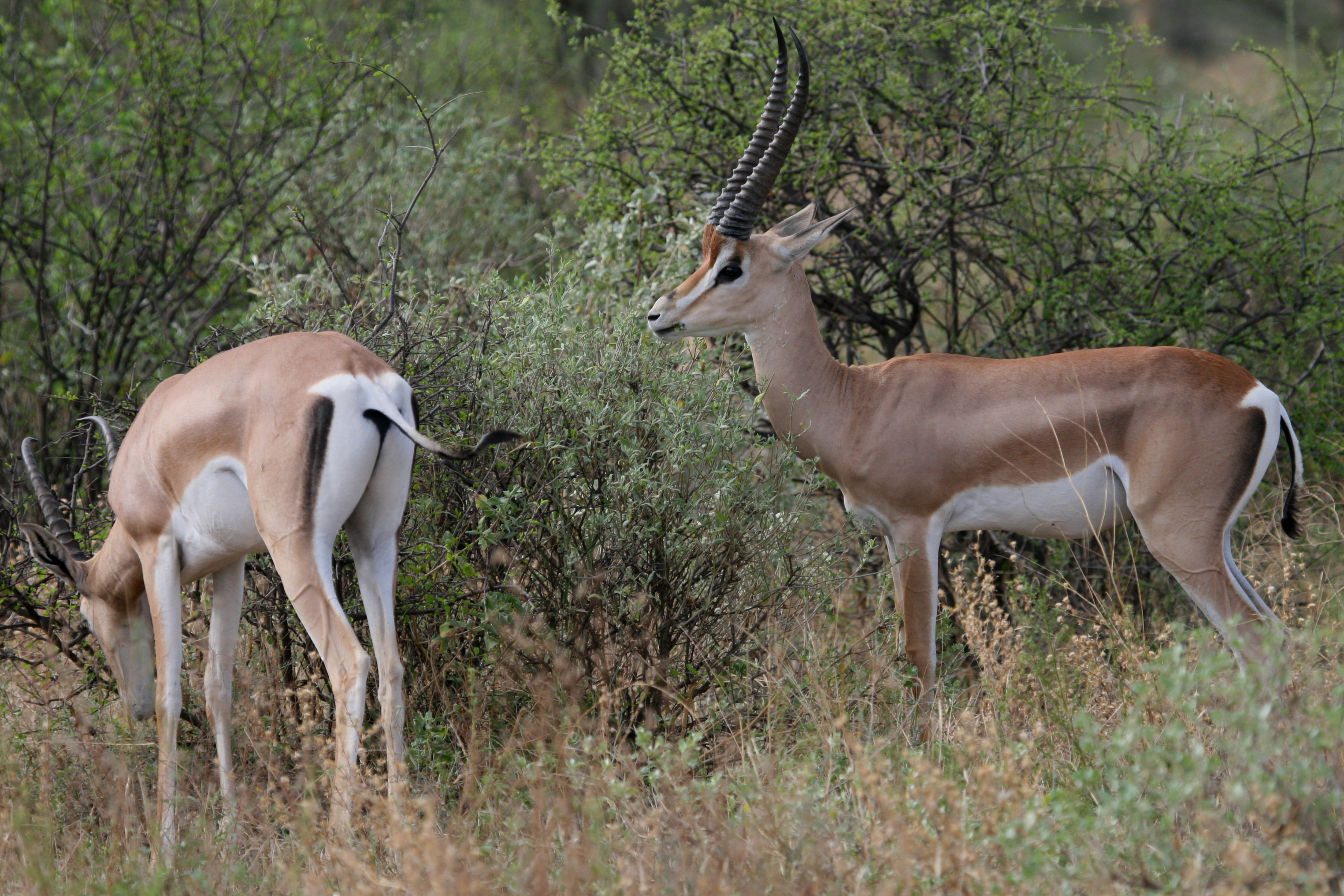

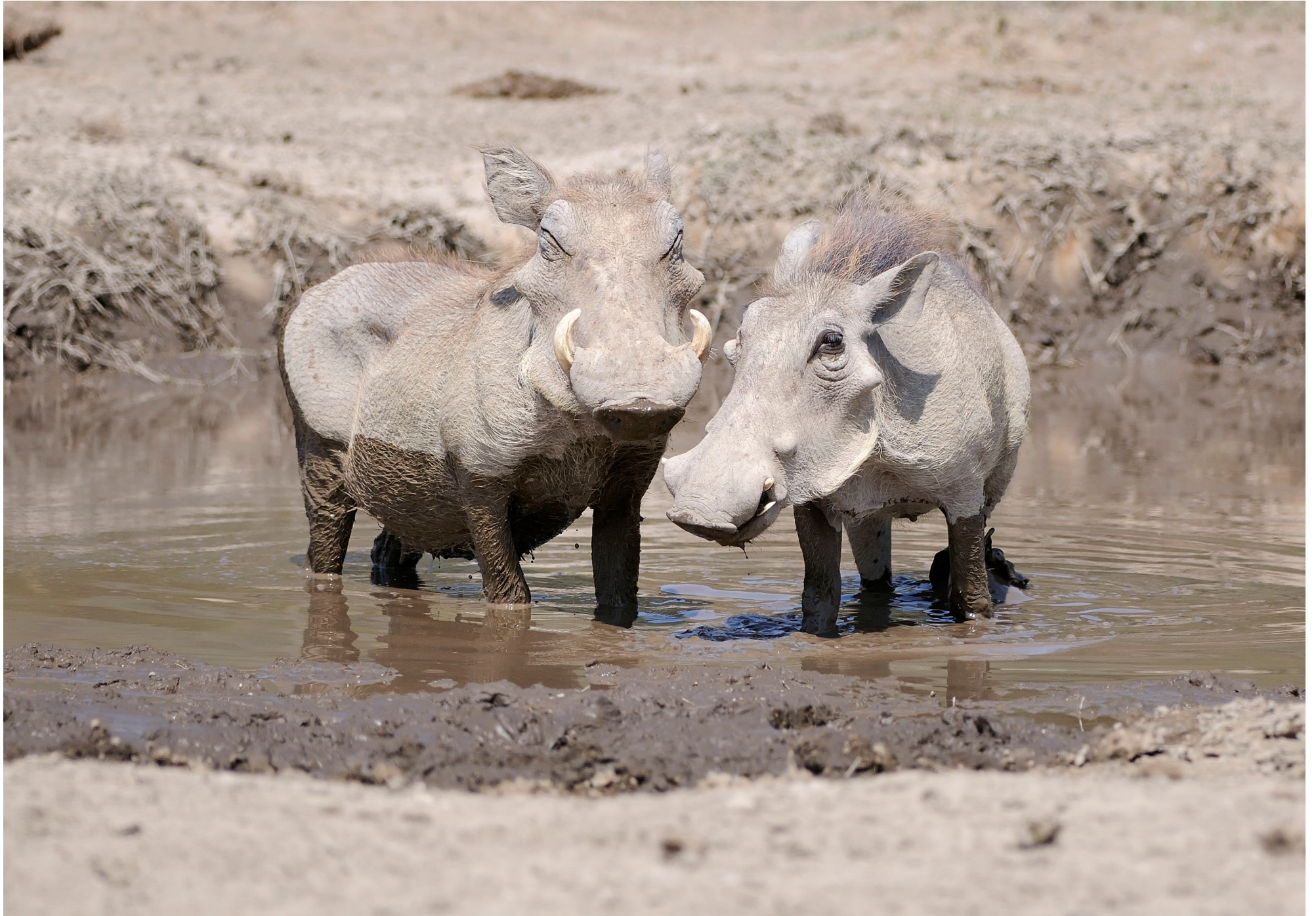

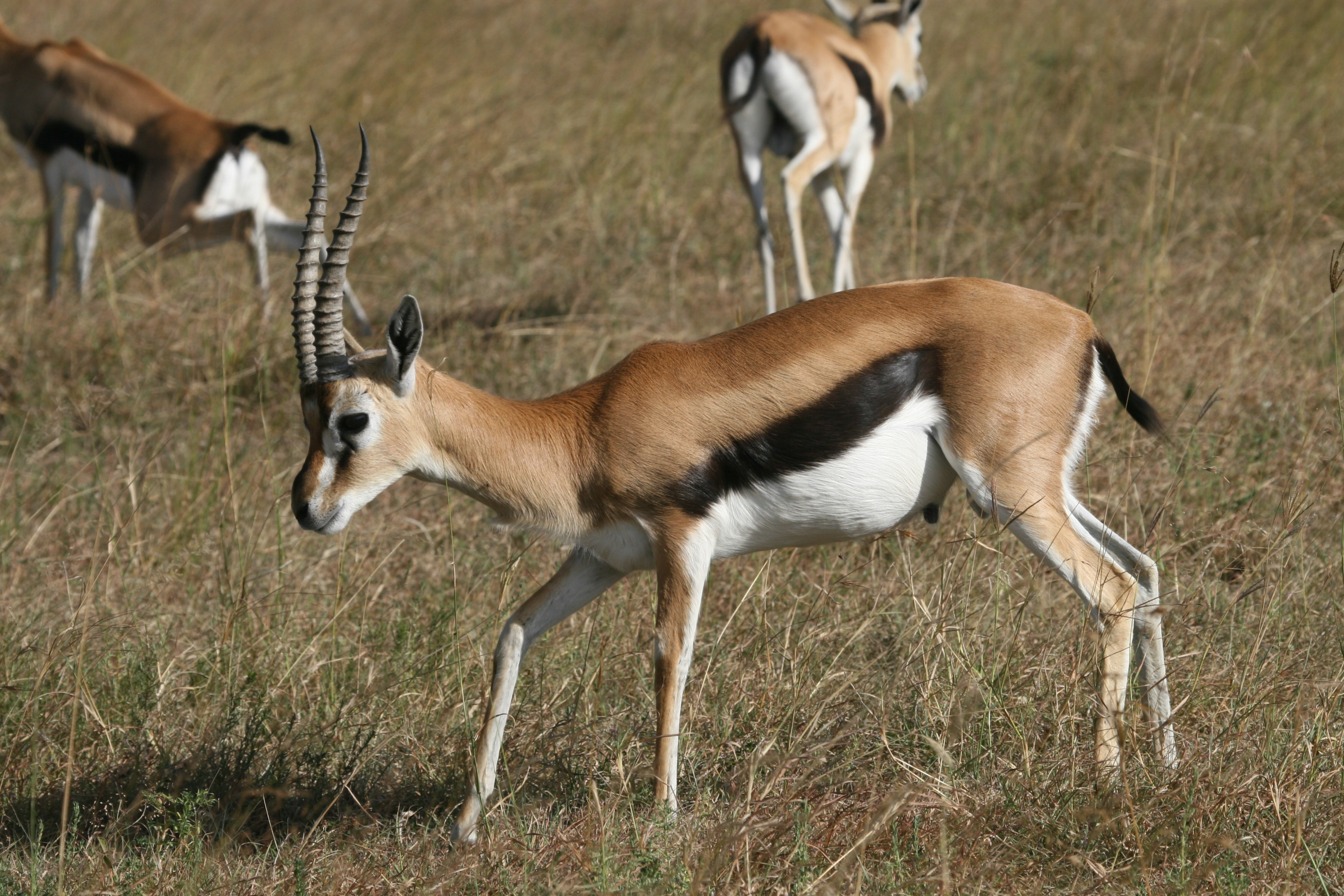

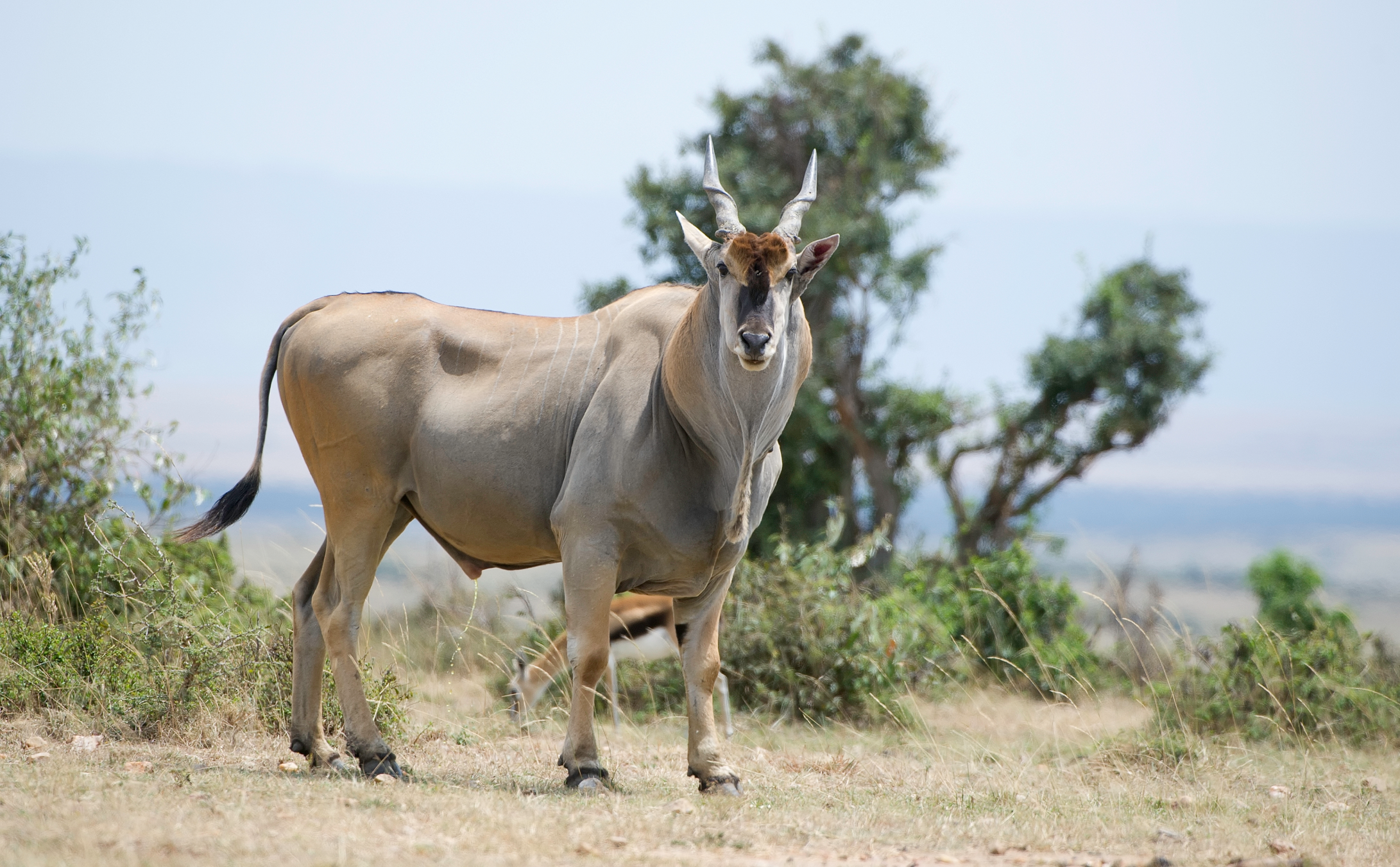

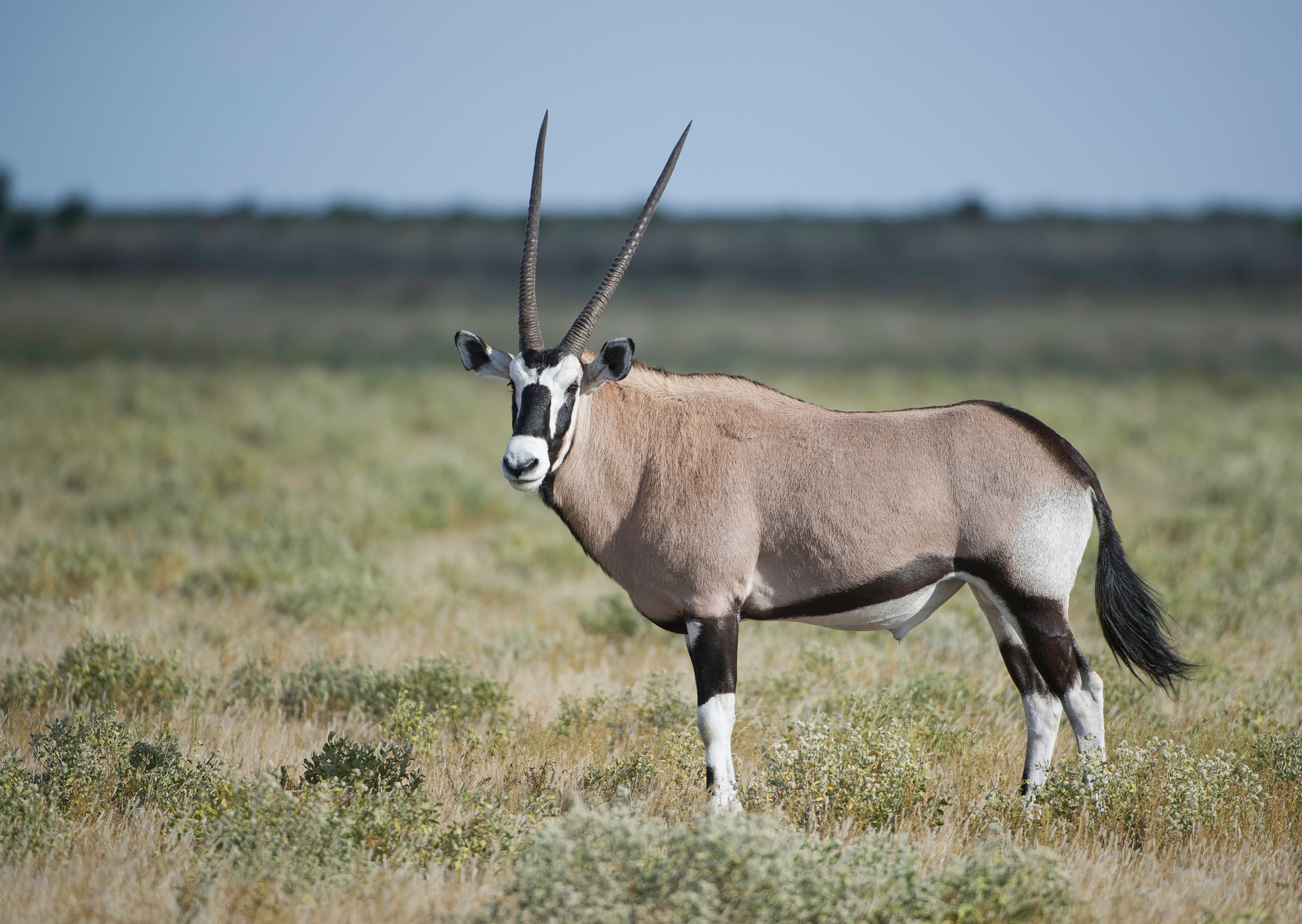

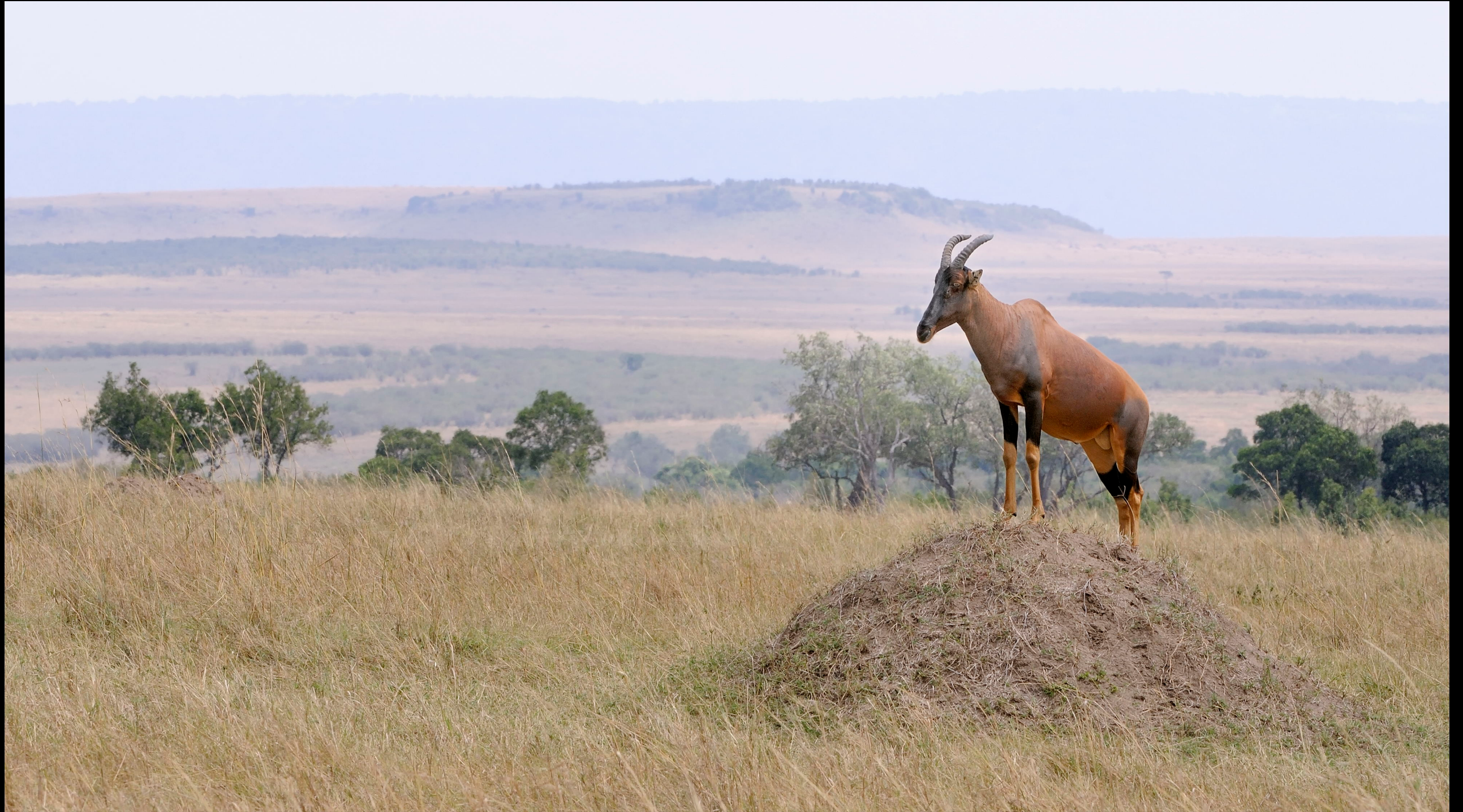

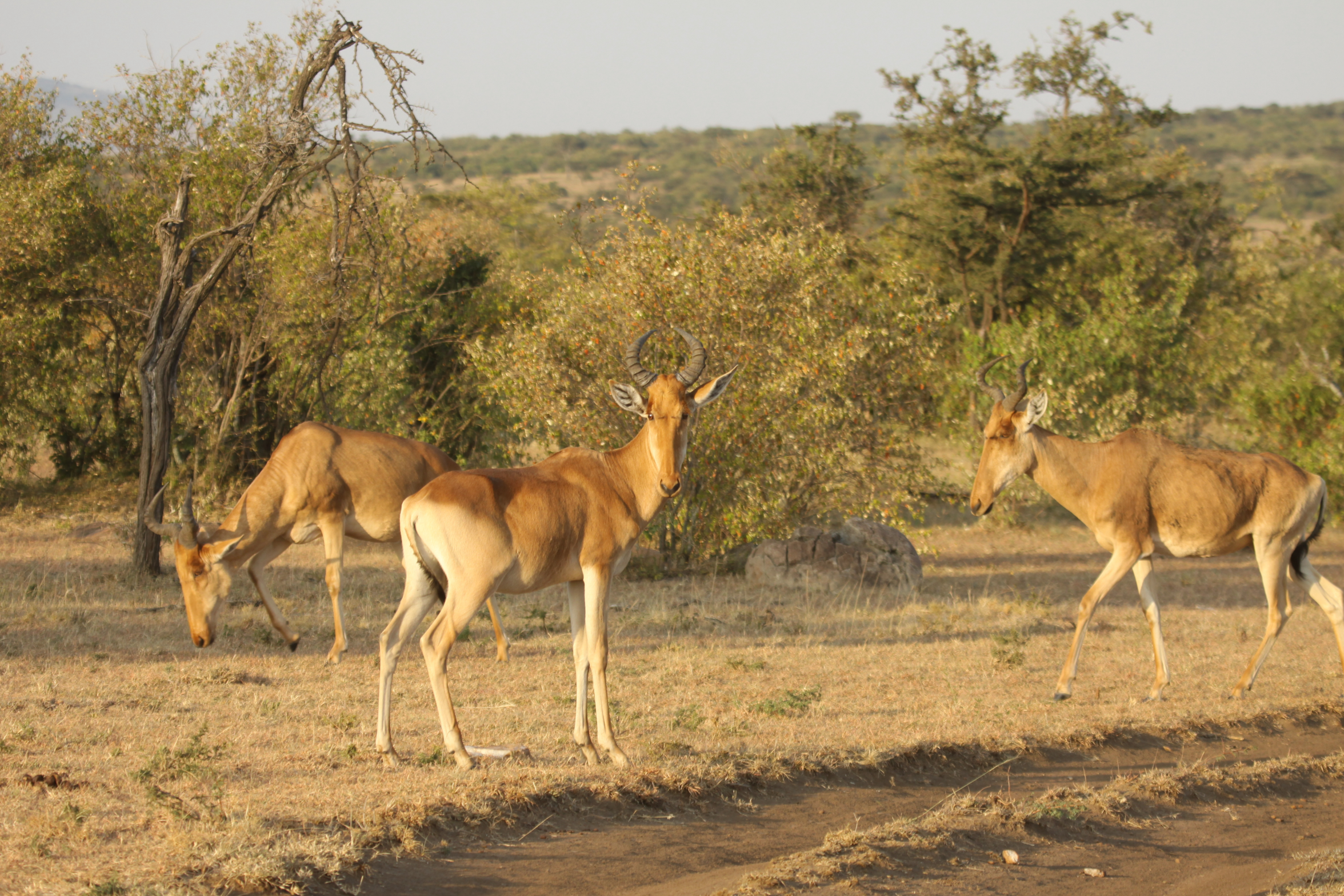

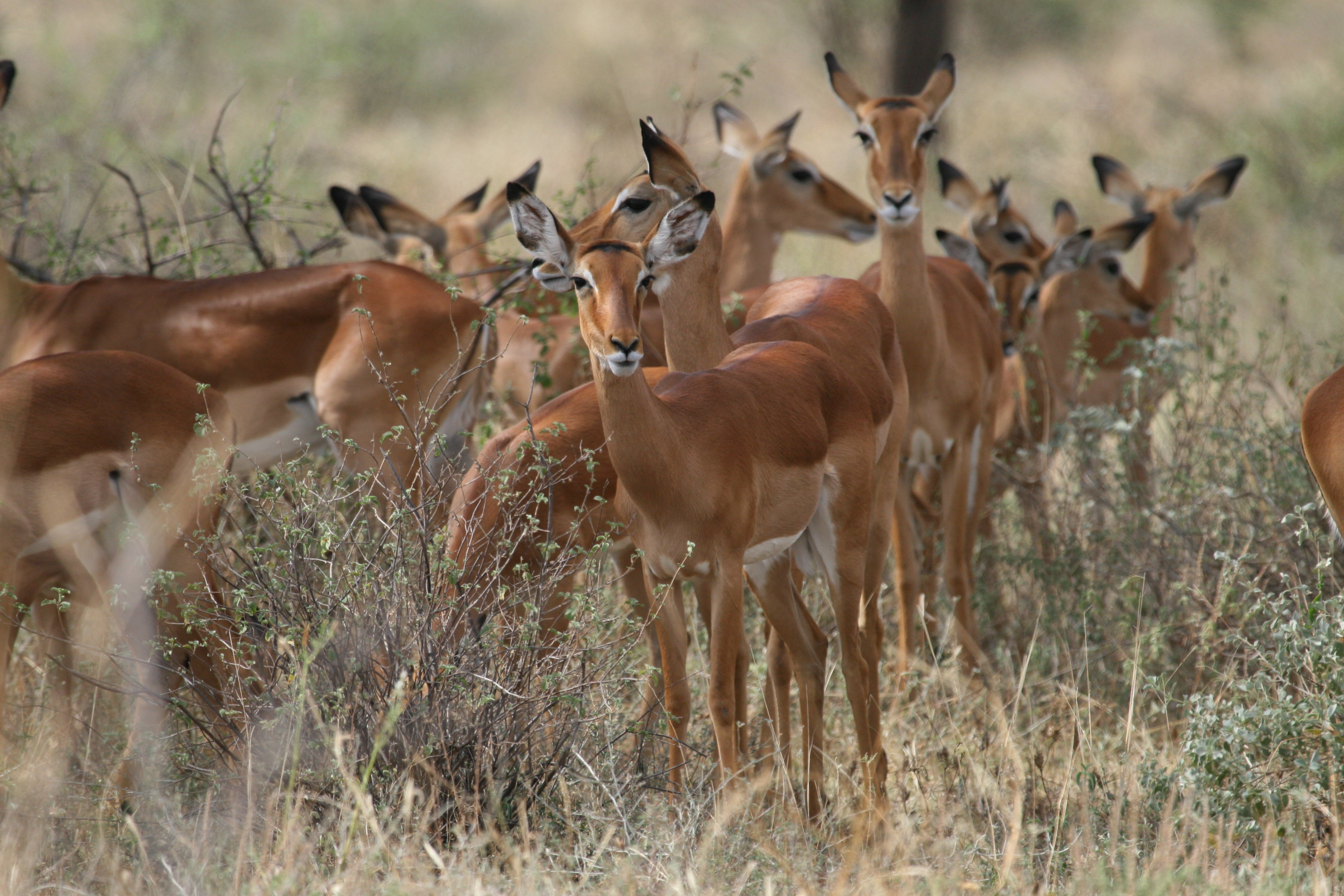

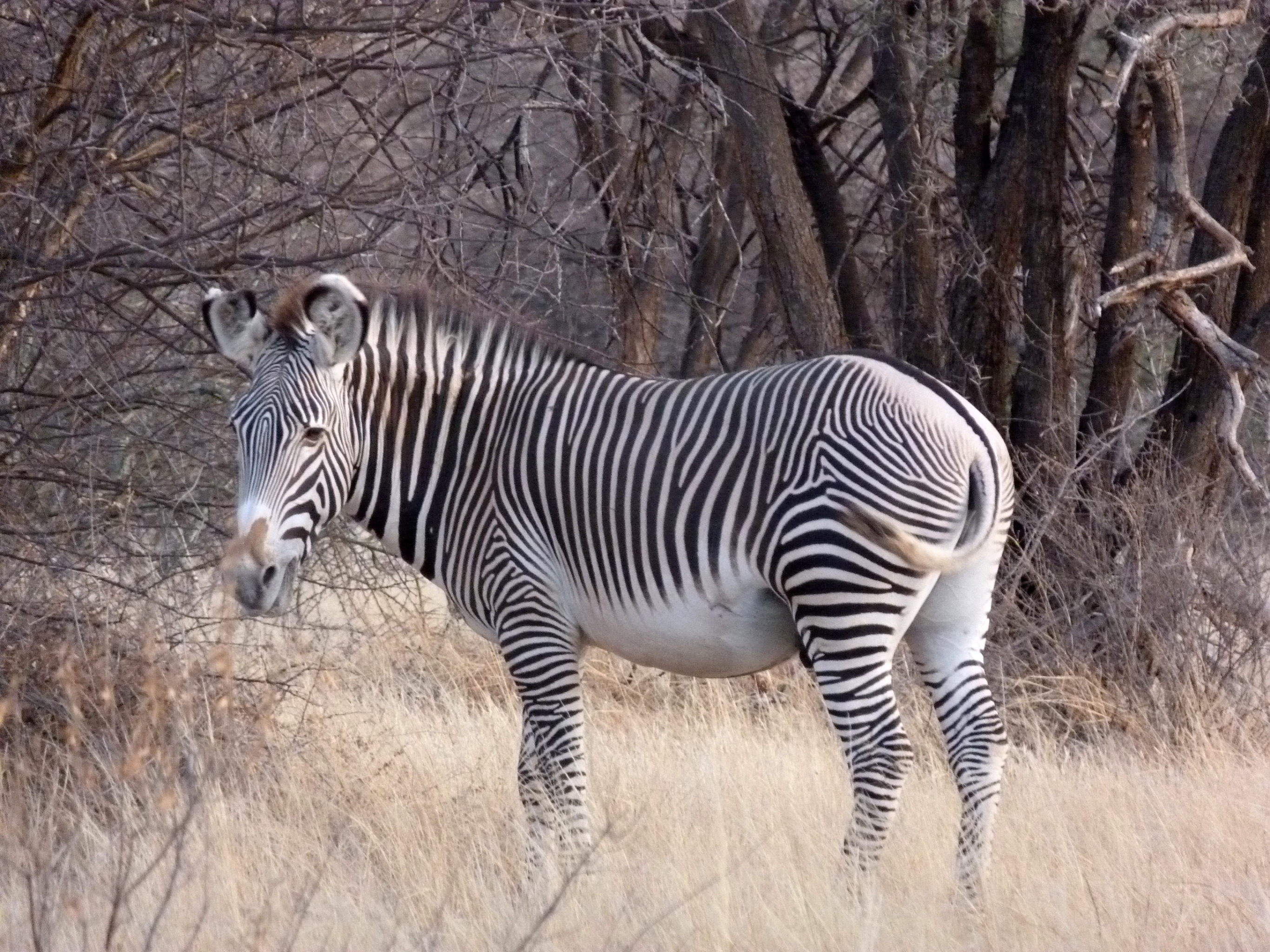

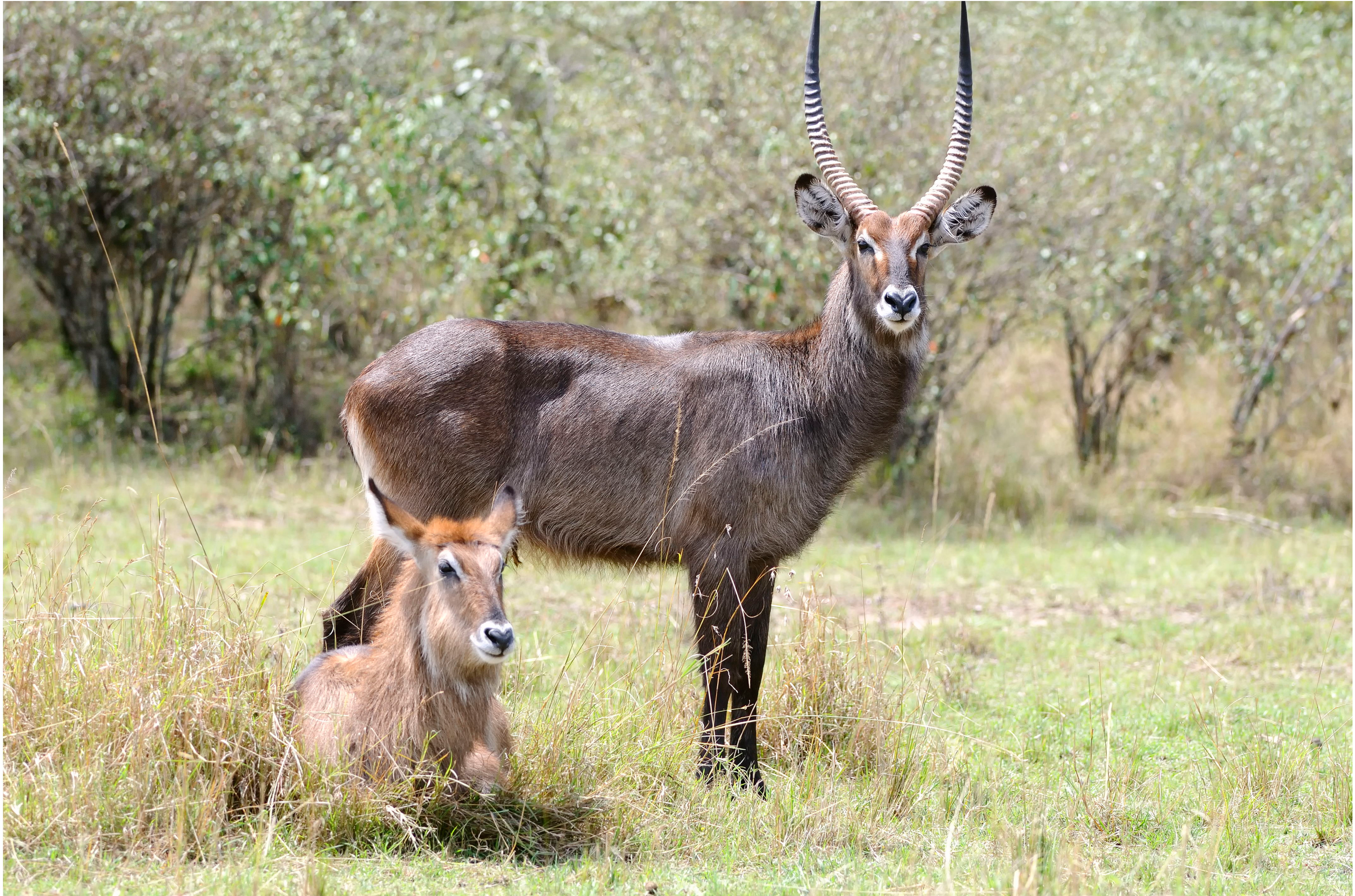

Supplement: S1 Fig — Photo Credit: Reto Buehler took all the photos except the photos of Thomson’s gazelle, Grant’s gazelle and hartebeest that were taken by Niels Mogensen. (PDF) [file pone.0163249.s011.pdf]

## Sheep and goats in Elgeyo Marakwet

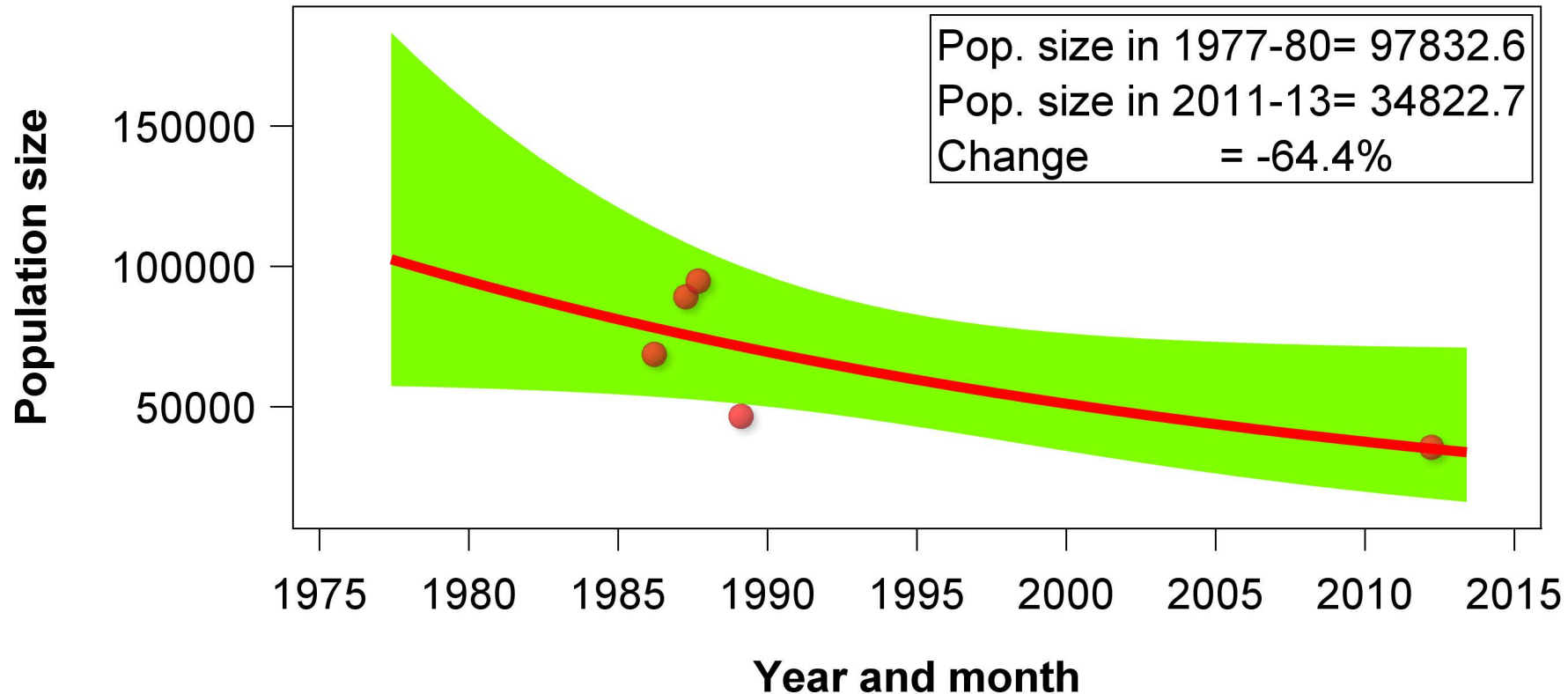

## Donkeys in Elgeyo Marakwet

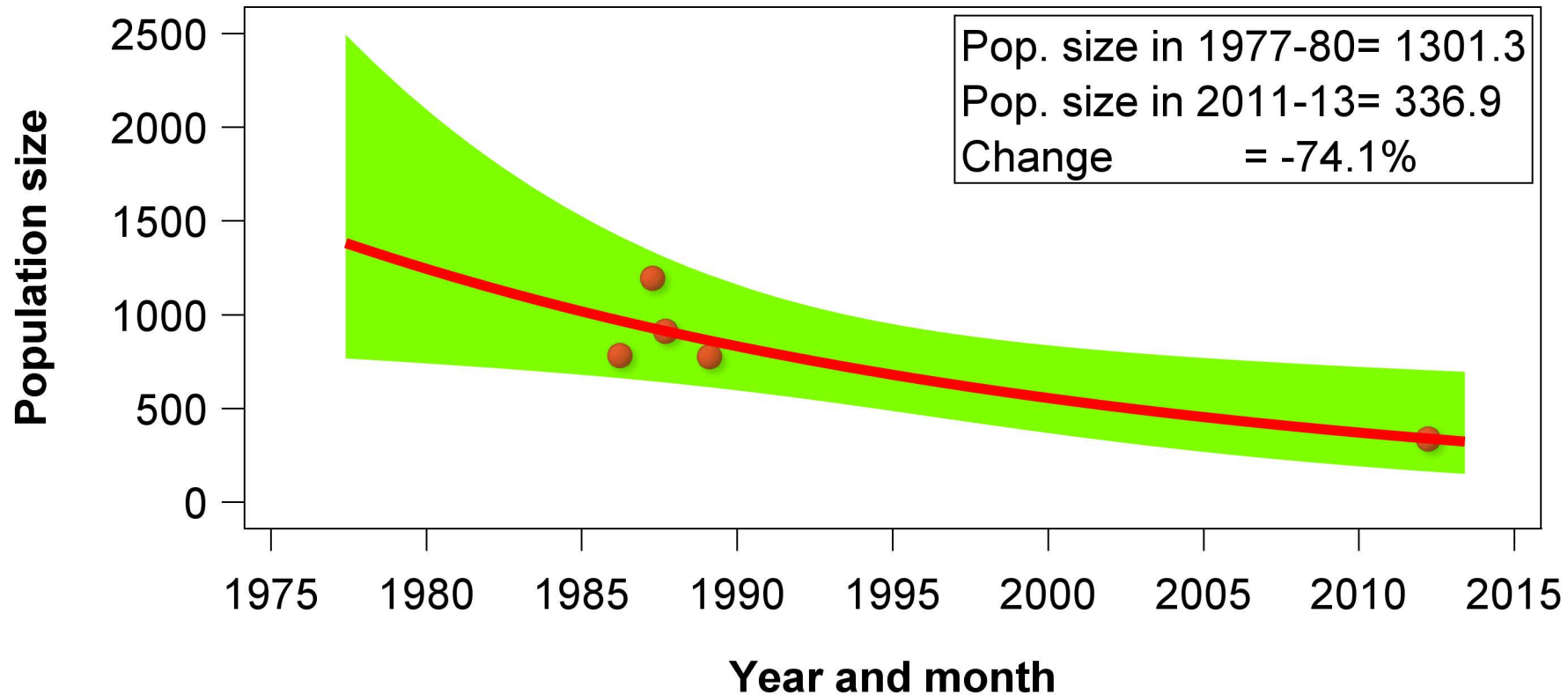

## Cattle in Elgeyo Marakwet

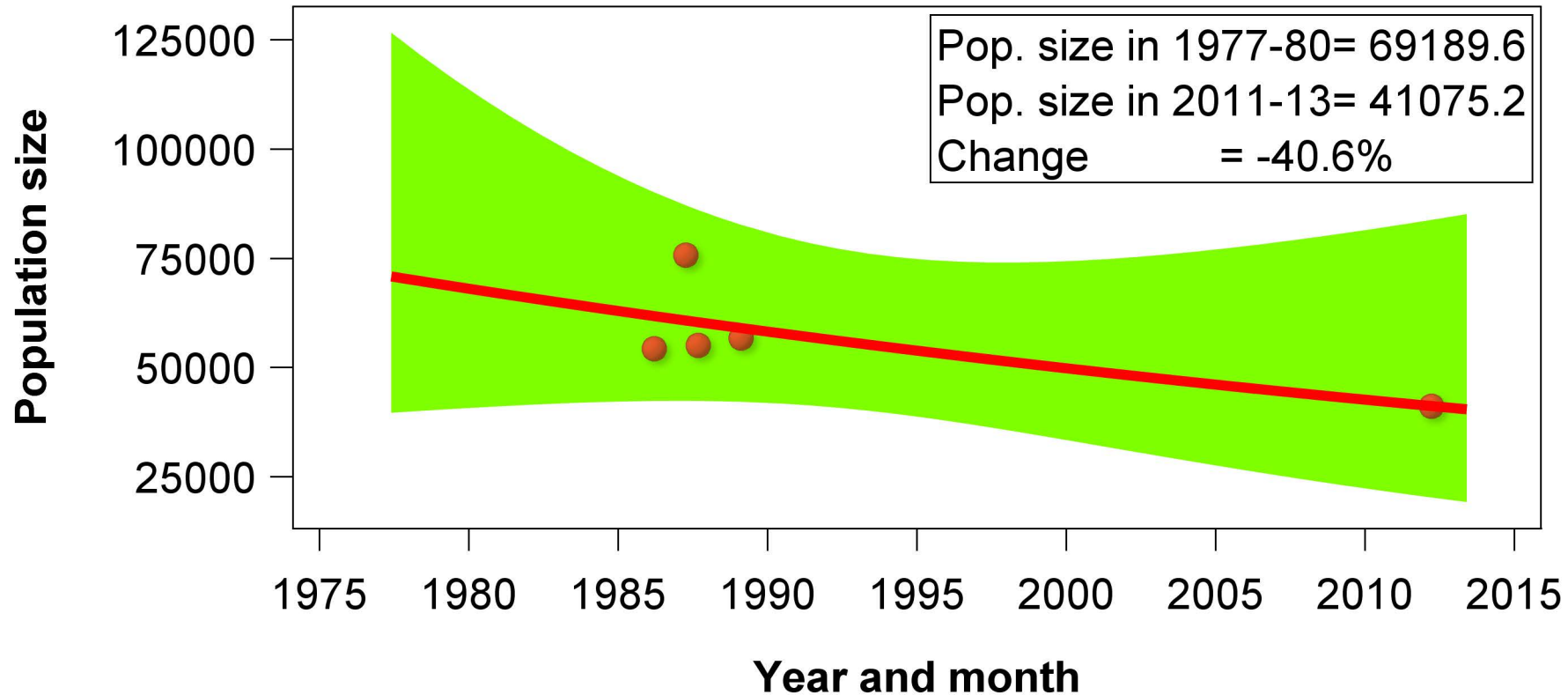

Supplement: S21 Fig — The solid red line is the fitted trend curve and the shaded chartreuse band is the pointwise 95% confidence band. The estimated average population size in 1977–1980 and 2011–2013 and the percentage change in population size between the two periods are provided in the inset. Numbers of all the 18 wildlife species were too few to model trends (PDF) [file pone.0163249.s031.pdf]
