# Supplementary material for: Extreme Wildlife Declines and Concurrent Increase in Livestock Numbers in Kenya: What Are the Causes?
Source: PLoS One. 2016 Sep 27;11(9):e0163249. doi: 10.1371/journal.pone.0163249 (PMC5039022; doi:10.1371/journal.pone.0163249)
Supplement: S2 Fig — The solid red line is the fitted trend curve and the shaded chartreuse band is the pointwise 95% confidence band. The estimated average population size in 1977–1980 and 2011–2016 and the percentage change in population size between the two periods are provided in the inset. (PDF) [file pone.0163249.s012.pdf]

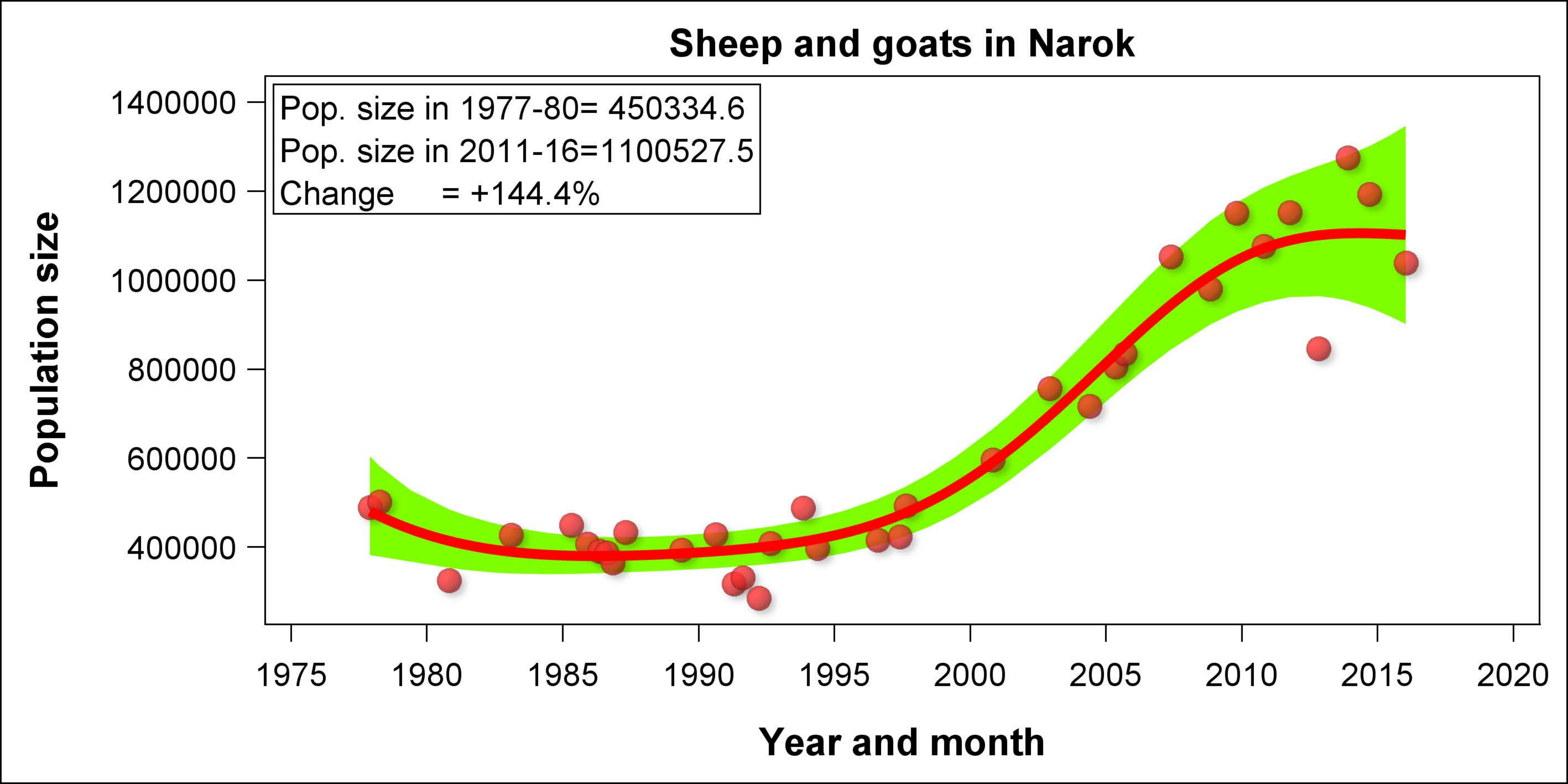

Sheep and goats in Narok
Pop. size in 1977-80= 450334.6
Pop. size in 2011-16=1100527.5
Change = +144.4%
Population size
1400000
1200000
1000000
800000
600000
400000
1975
1980
1985
1990
1995
2000
2005
2010
2015
2020
Year and month

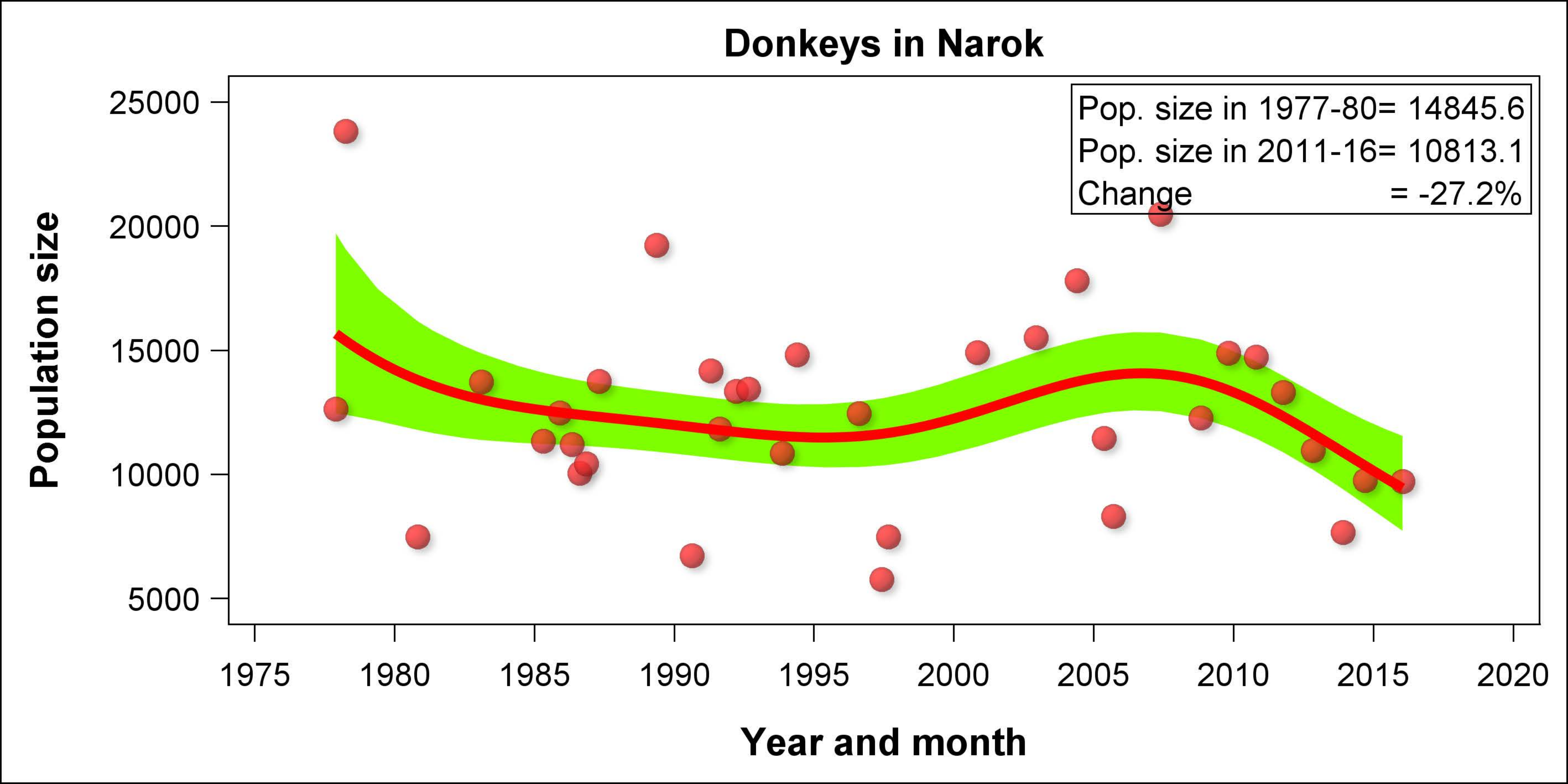
Donkeys in Narok
Pop. size in 1977-80= 14845.6
Pop. size in 2011-16= 10813.1
Change = -27.2%
Population size
25000
20000
15000
10000
5000
1975
1980
1985
1990
1995
2000
2005
2010
2015
2020
Year and month

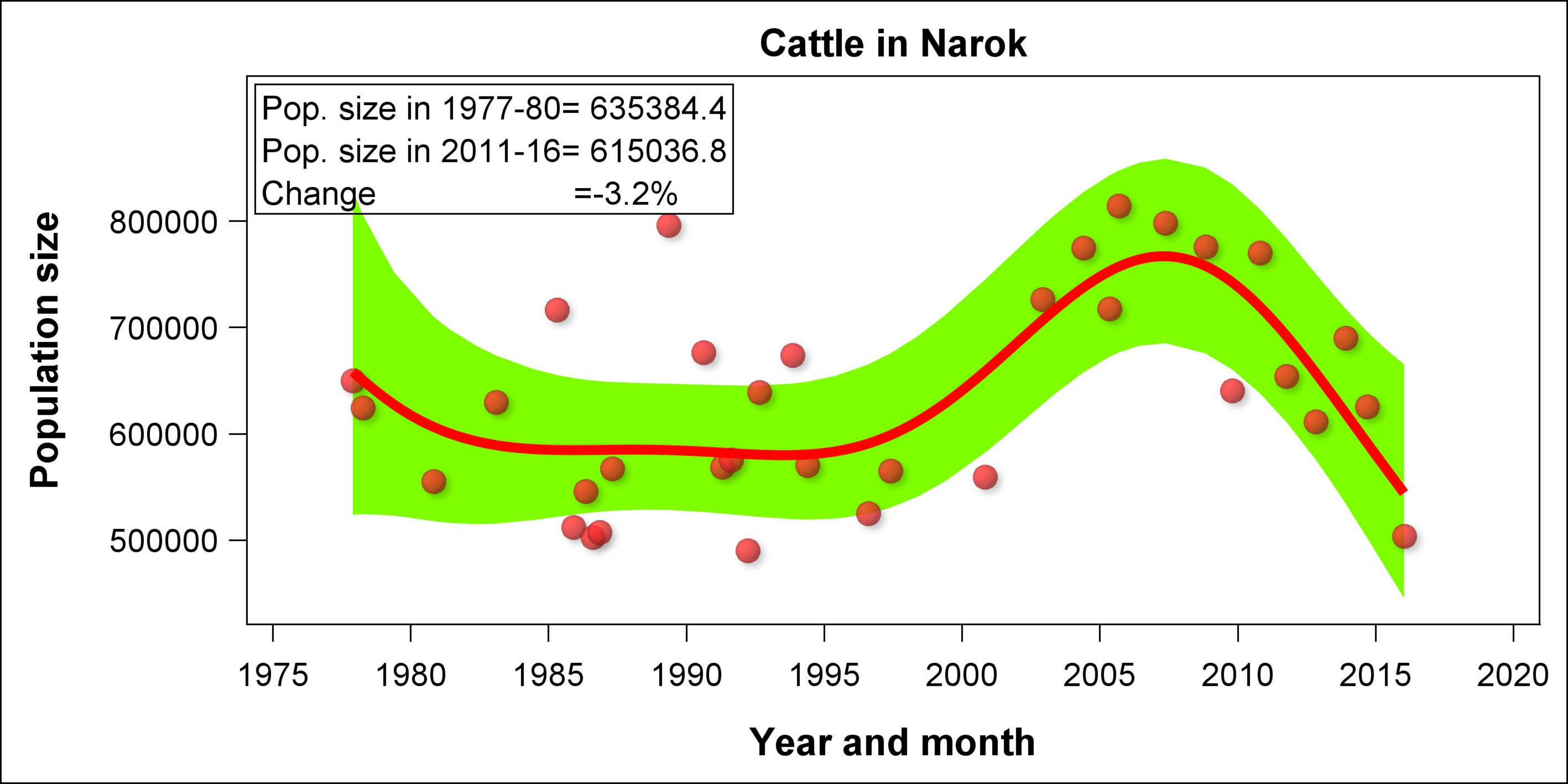

Cattle in Narok
Pop. size in 1977-80= 635384.4
Pop. size in 2011-16= 615036.8
Change =-3.2%
Population size
800000
700000
600000
500000
1975
1980
1985
1990
1995
2000
2005
2010
2015
2020
Year and month

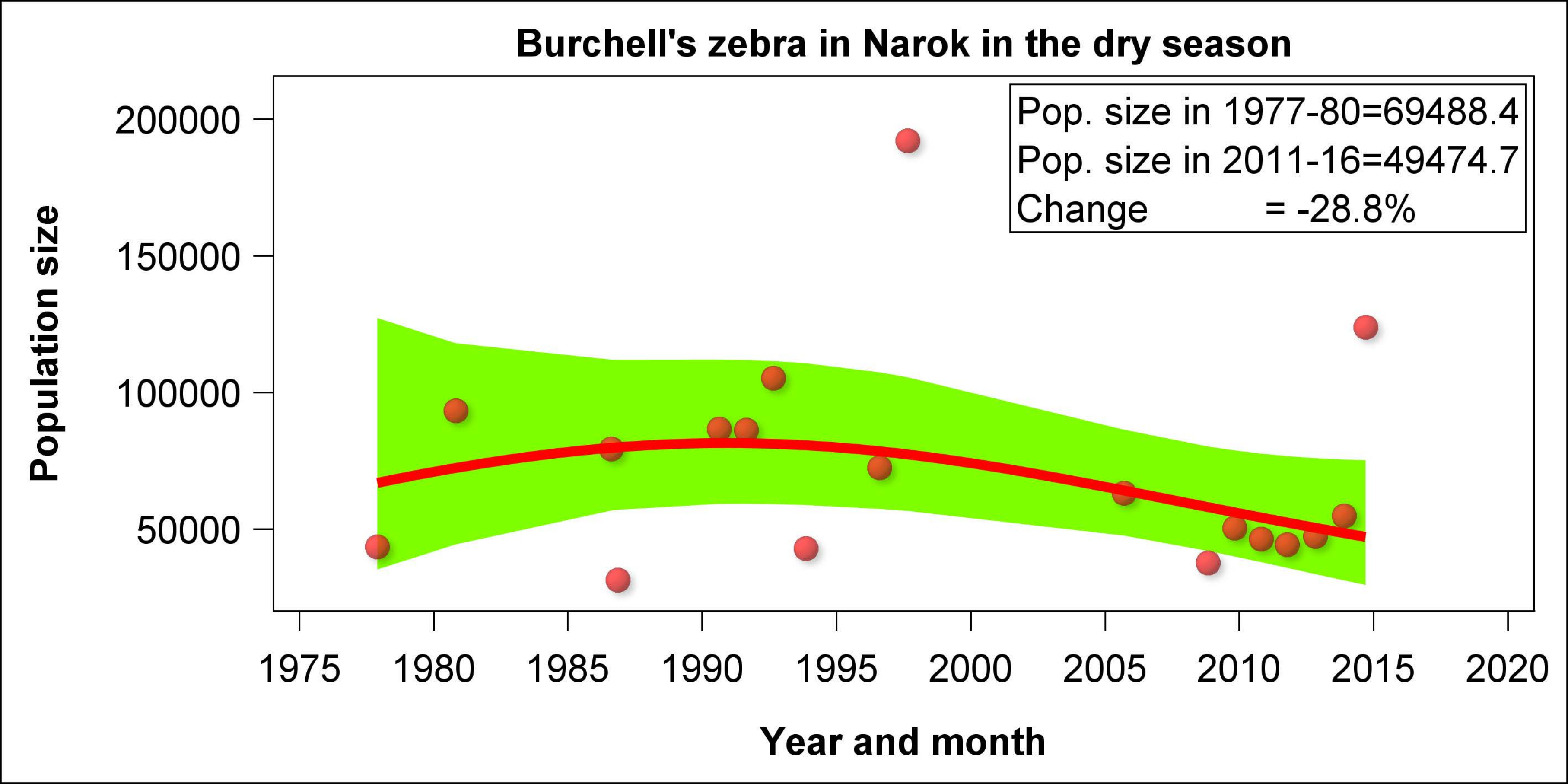
Burchell's zebra in Narok in the dry season
Pop. size in 1977-80=69488.4
Pop. size in 2011-16=49474.7
Change = -28.8%
Population size
200000
150000
100000
50000
1975
1980
1985
1990
1995
2000
2005
2010
2015
2020
Year and month

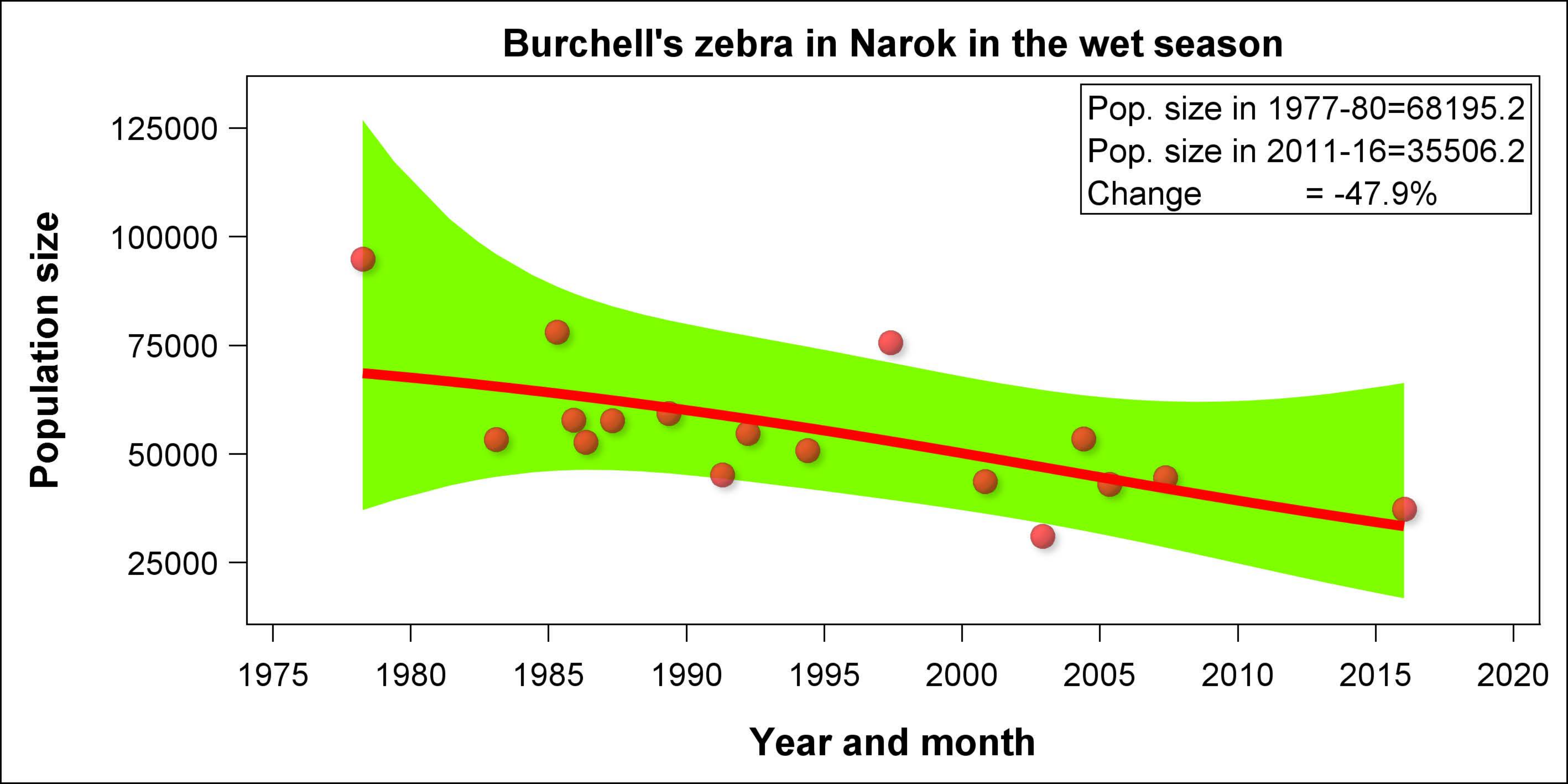

Burchell's zebra in Narok in the wet season
Pop. size in 1977-80=68195.2
Pop. size in 2011-16=35506.2
Change = -47.9%
Population size
125000
100000
75000
50000
25000
1975
1980
1985
1990
1995
2000
2005
2010
2015
2020
Year and month

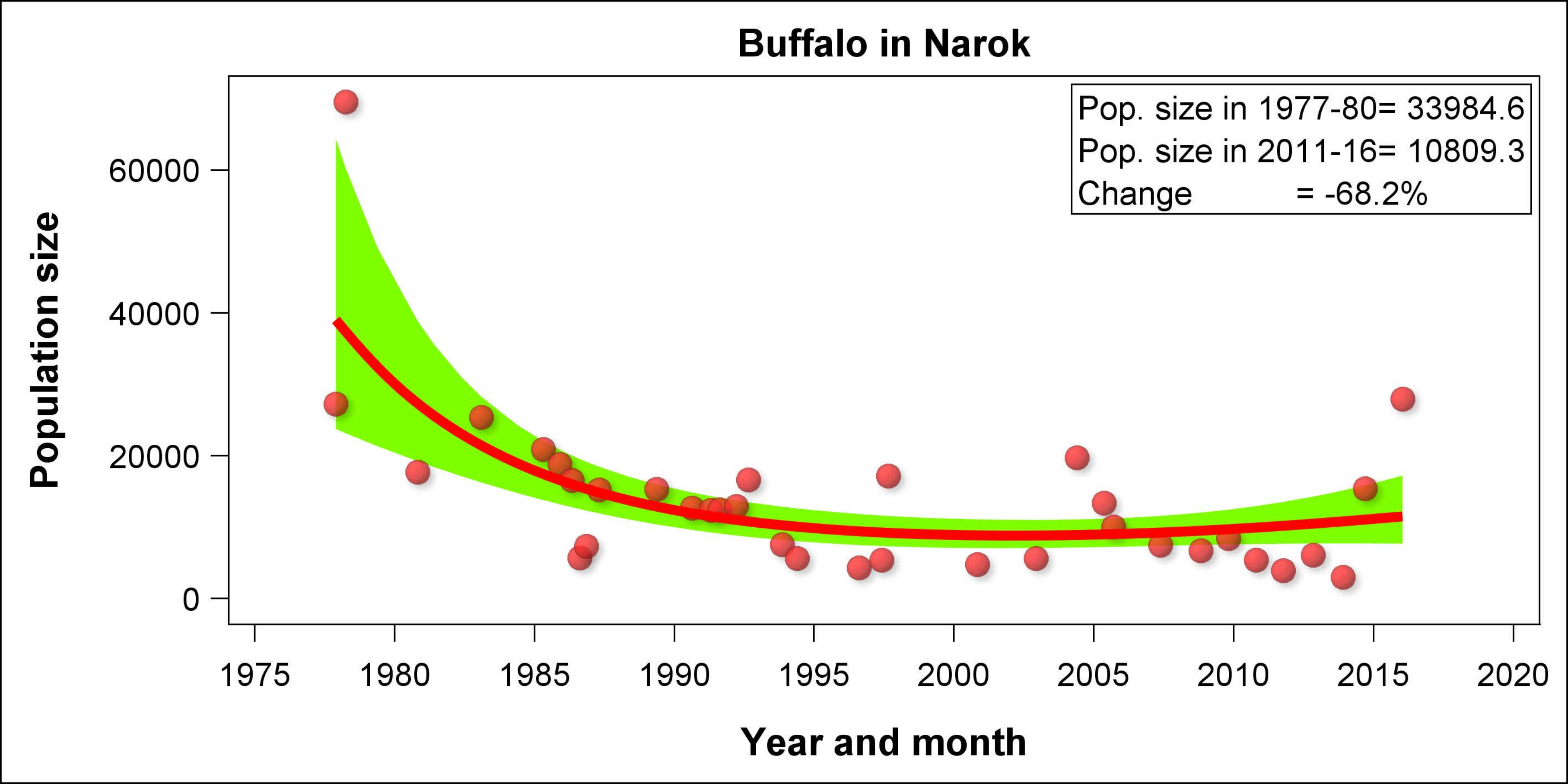
Buffalo in Narok
Pop. size in 1977-80= 33984.6
Pop. size in 2011-16= 10809.3
Change = -68.2%
Population size
0
20000
40000
60000
1975
1980
1985
1990
1995
2000
2005
2010
2015
2020
Year and month

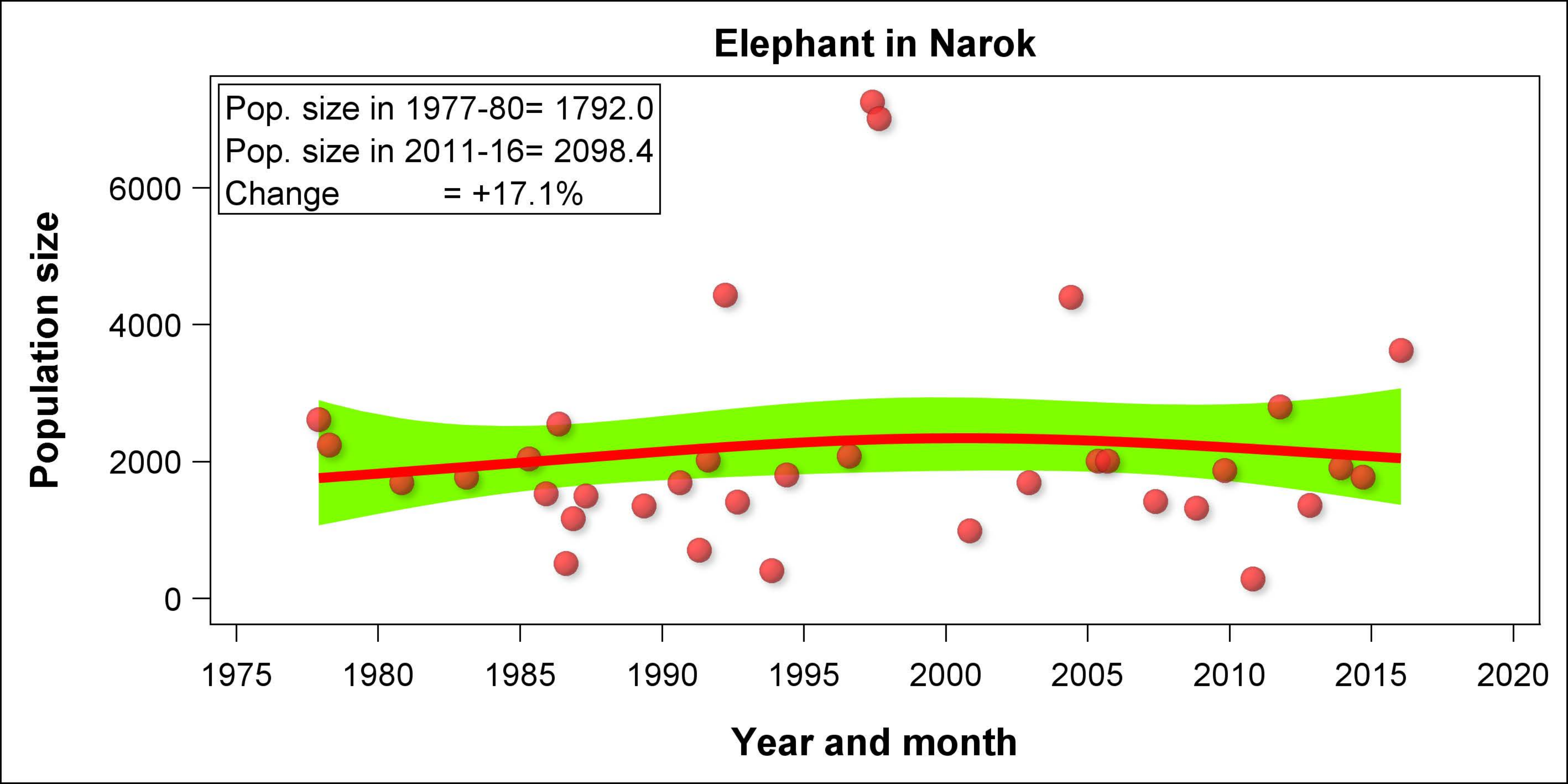
Elephant in Narok
Pop. size in 1977-80= 1792.0
Pop. size in 2011-16= 2098.4
Change = +17.1%
Population size
0
2000
4000
6000
1975
1980
1985
1990
1995
2000
2005
2010
2015
2020
Year and month

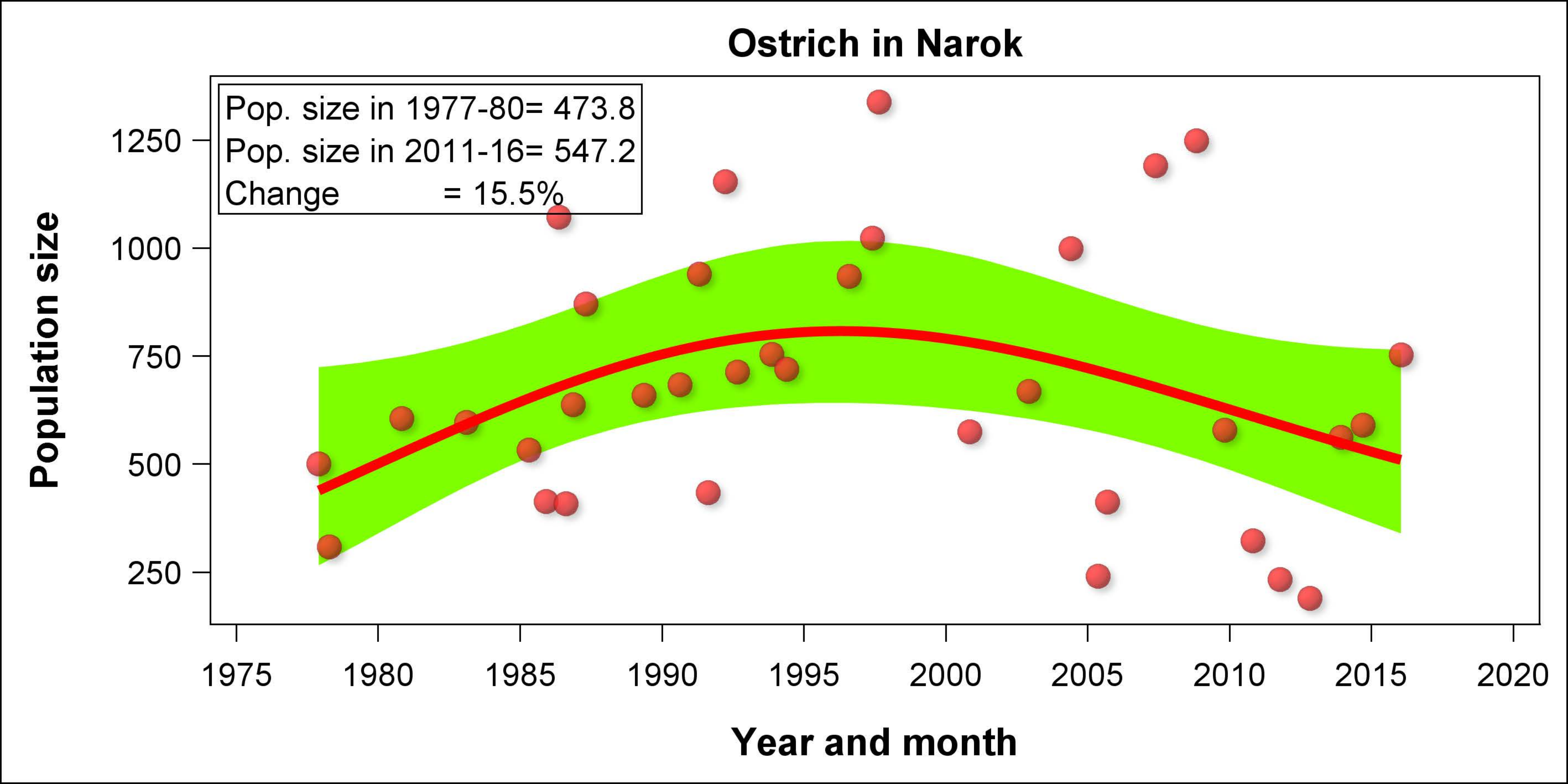

Ostrich in Narok
Pop. size in 1977-80= 473.8
Pop. size in 2011-16= 547.2
Change = 15.5%
Population size
1250
1000
750
500
250
1975
1980
1985
1990
1995
2000
2005
2010
2015
2020
Year and month

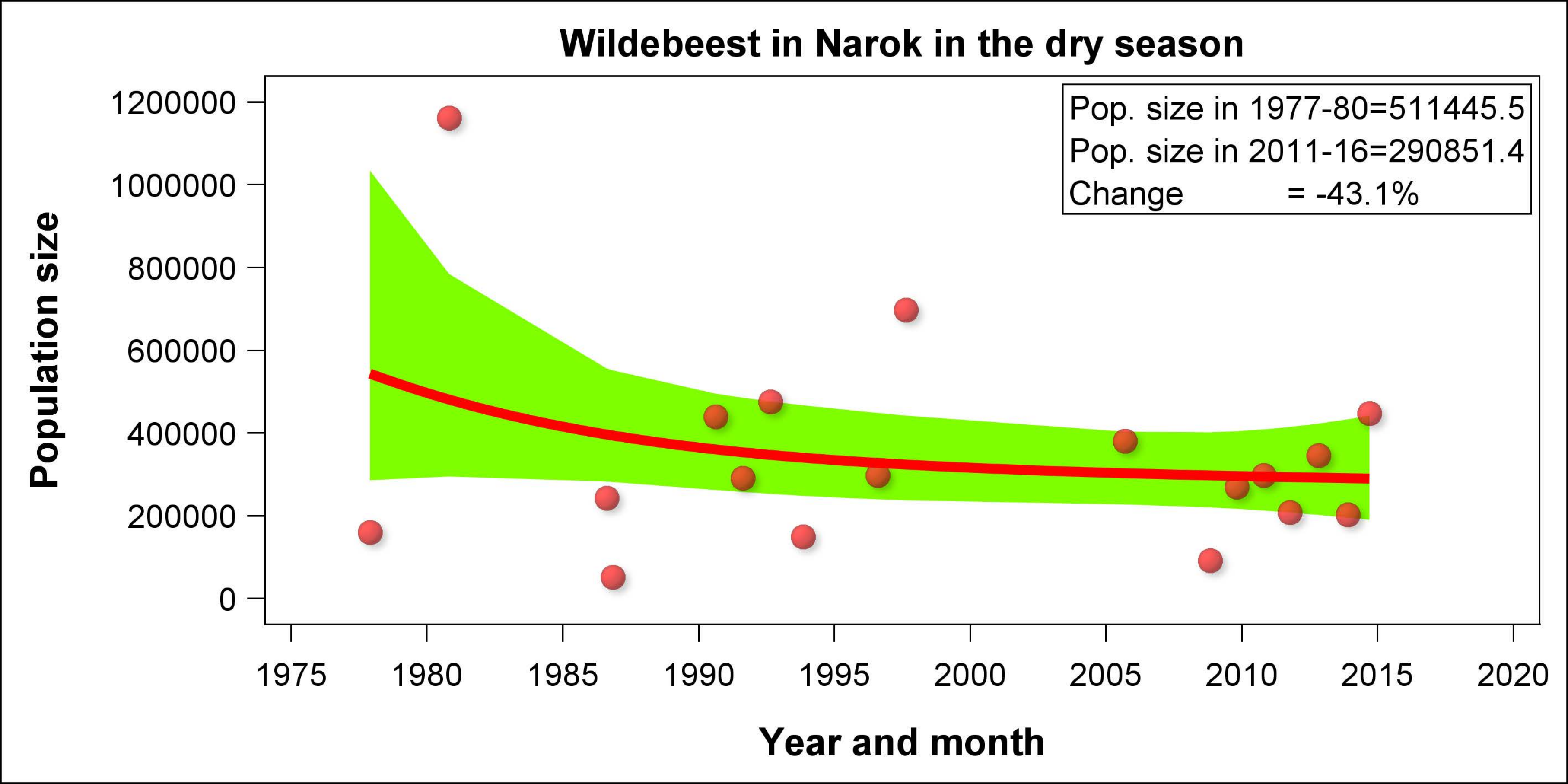
Wildebeest in Narok in the dry season
Pop. size in 1977-80=511445.5
Pop. size in 2011-16=290851.4
Change = -43.1%
Population size
1200000
1000000
800000
600000
400000
200000
0
1975
1980
1985
1990
1995
2000
2005
2010
2015
2020
Year and month

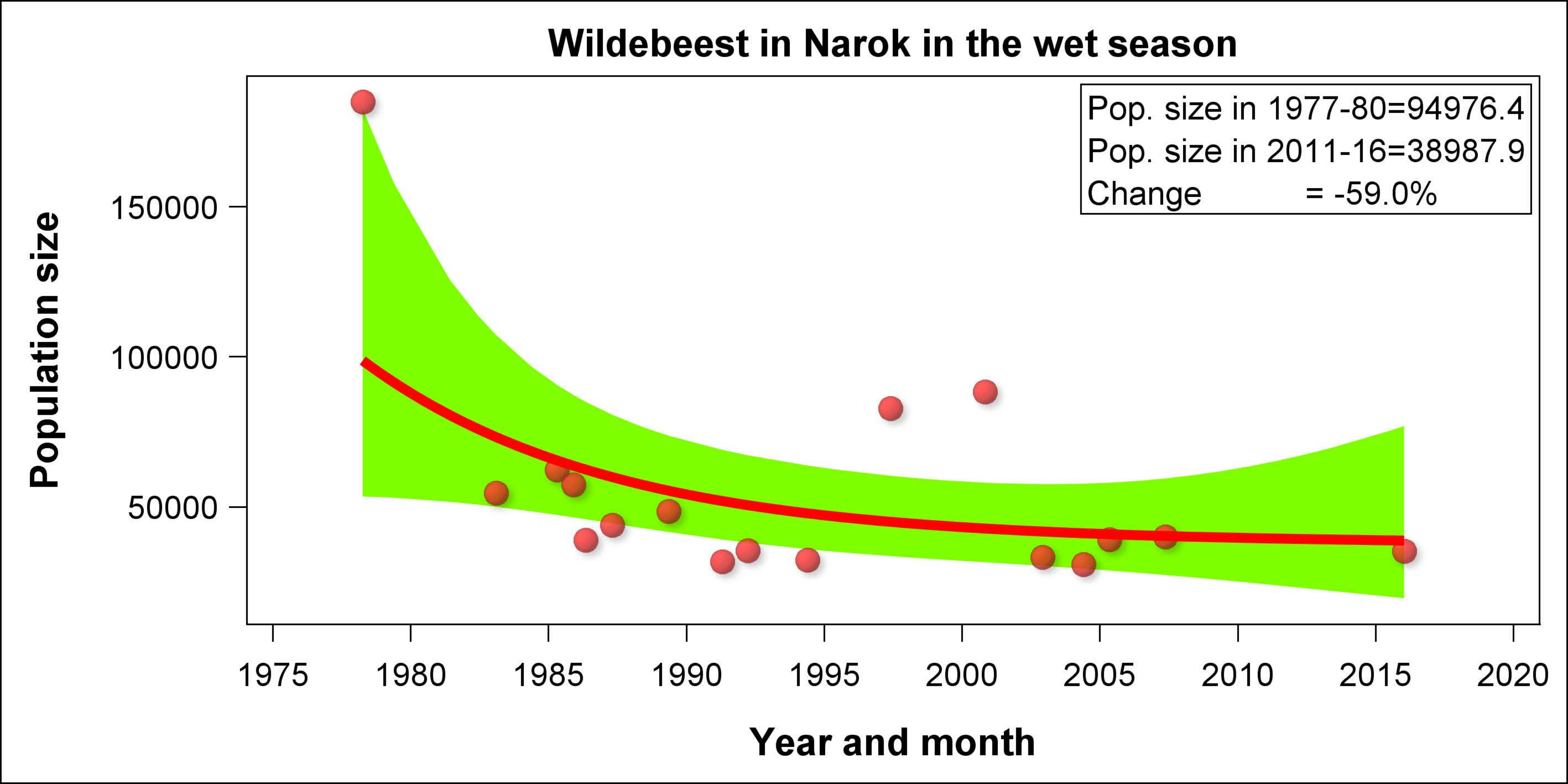
Wildebeest in Narok in the wet season
Pop. size in 1977-80=94976.4
Pop. size in 2011-16=38987.9
Change = -59.0%
Population size
150000
100000
50000
1975
1980
1985
1990
1995
2000
2005
2010
2015
2020
Year and month

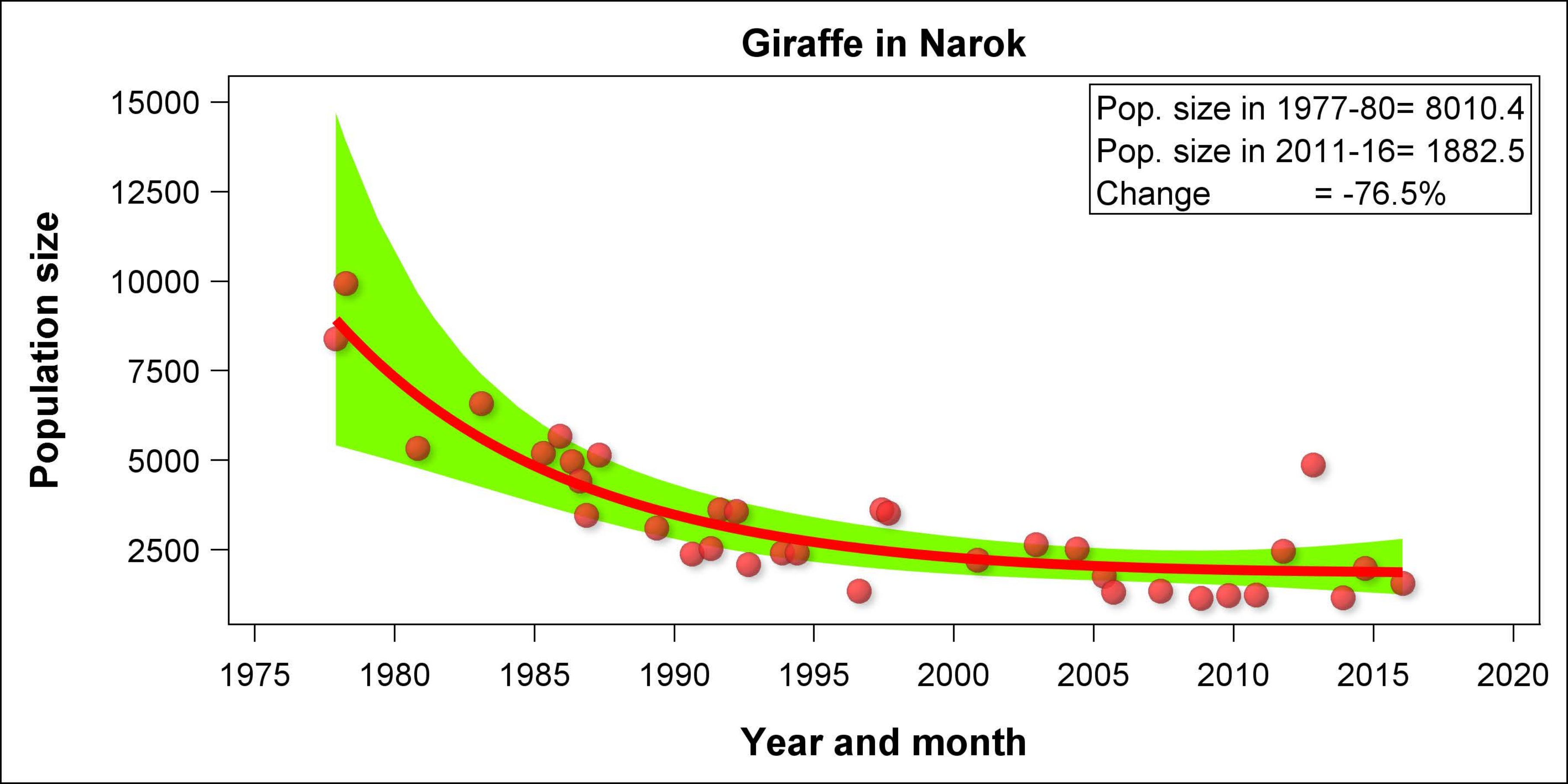
Giraffe in Narok
Pop. size in 1977-80= 8010.4
Pop. size in 2011-16= 1882.5
Change = -76.5%
Population size
15000
12500
10000
7500
5000
2500
1975
1980
1985
1990
1995
2000
2005
2010
2015
2020
Year and month

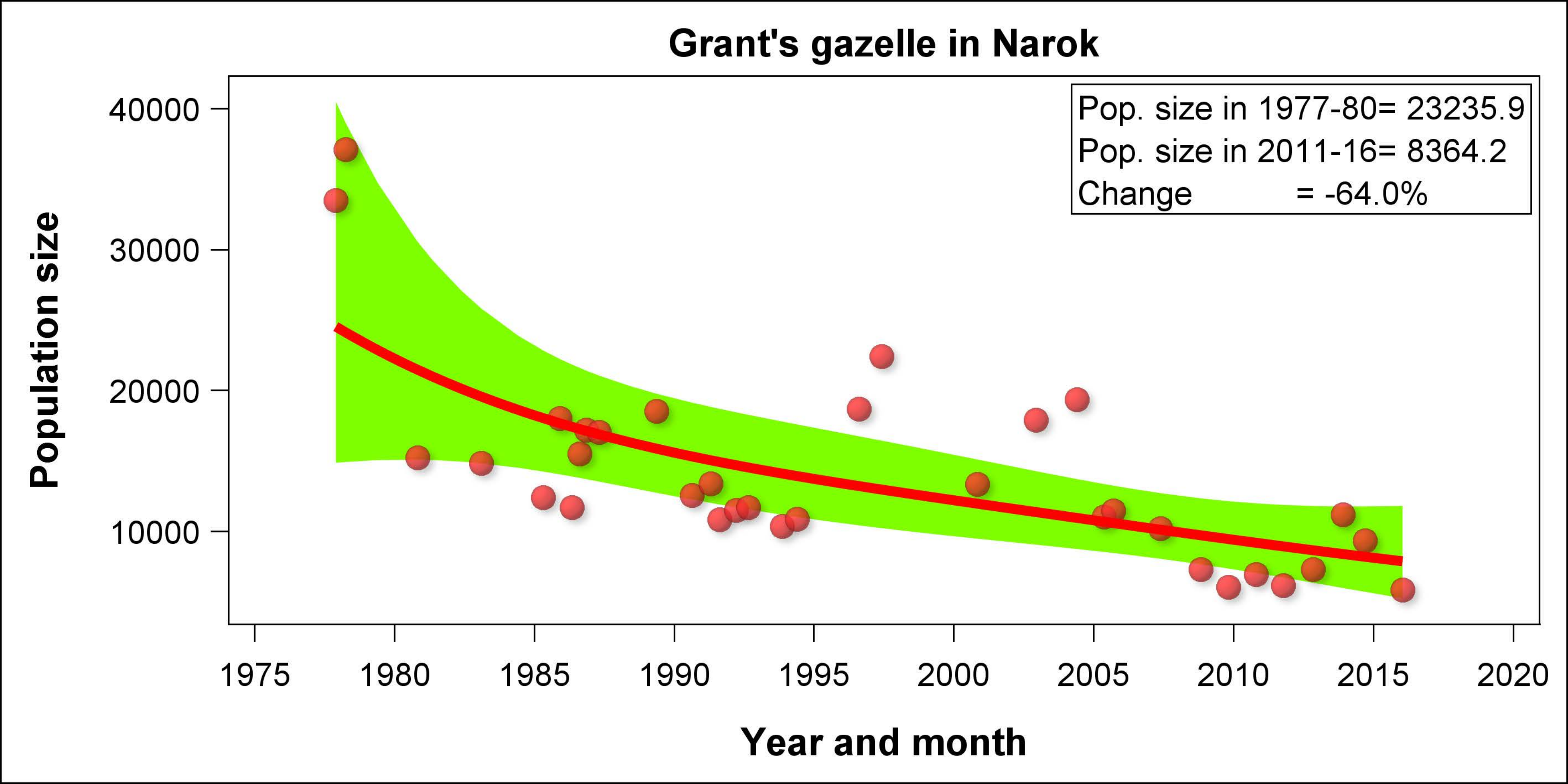

Grant's gazelle in Narok
Pop. size in 1977-80= 23235.9
Pop. size in 2011-16= 8364.2
Change = -64.0%
40000
30000
20000
10000
Population size
1975
1980
1985
1990
1995
2000
2005
2010
2015
2020
Year and month

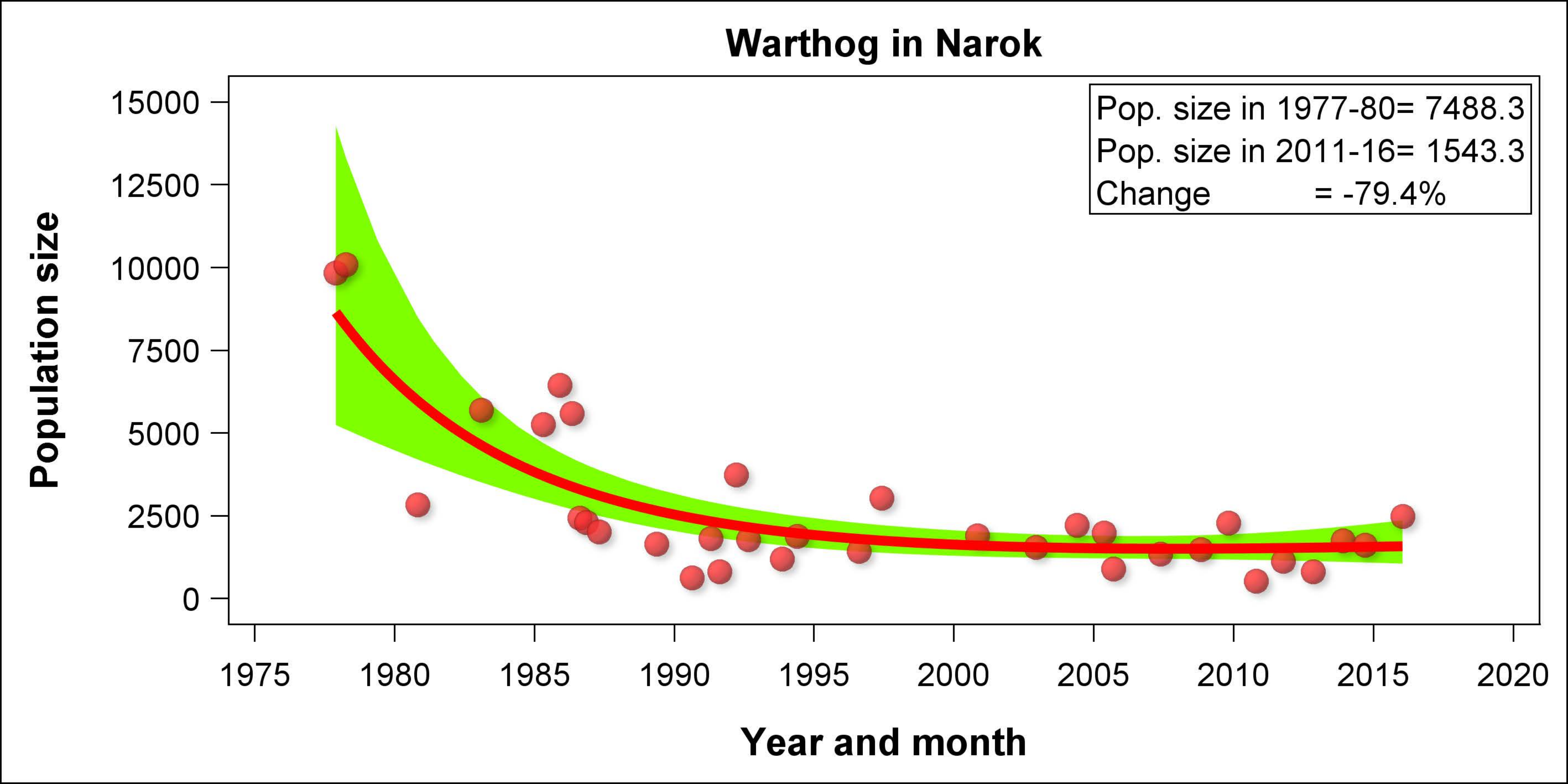

Warthog in Narok
Pop. size in 1977-80= 7488.3
Pop. size in 2011-16= 1543.3
Change = -79.4%
Population size
15000
12500
10000
7500
5000
2500
0
1975
1980
1985
1990
1995
2000
2005
2010
2015
2020
Year and month

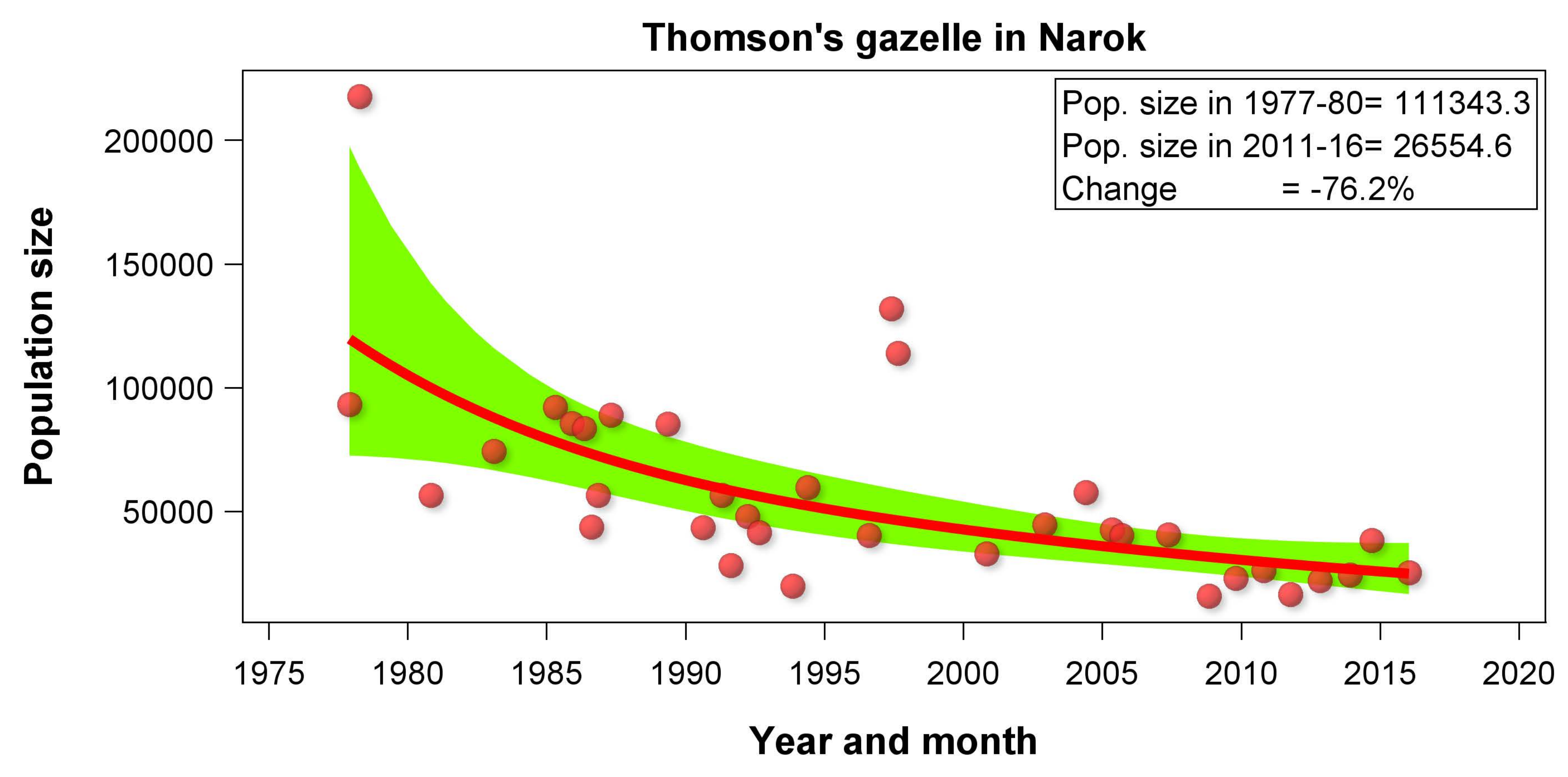

Thomson's gazelle in Narok
Pop. size in 1977-80= 111343.3
Pop. size in 2011-16= 26554.6
Change = -76.2%
Population size
200000
150000
100000
50000
1975
1980
1985
1990
1995
2000
2005
2010
2015
2020
Year and month

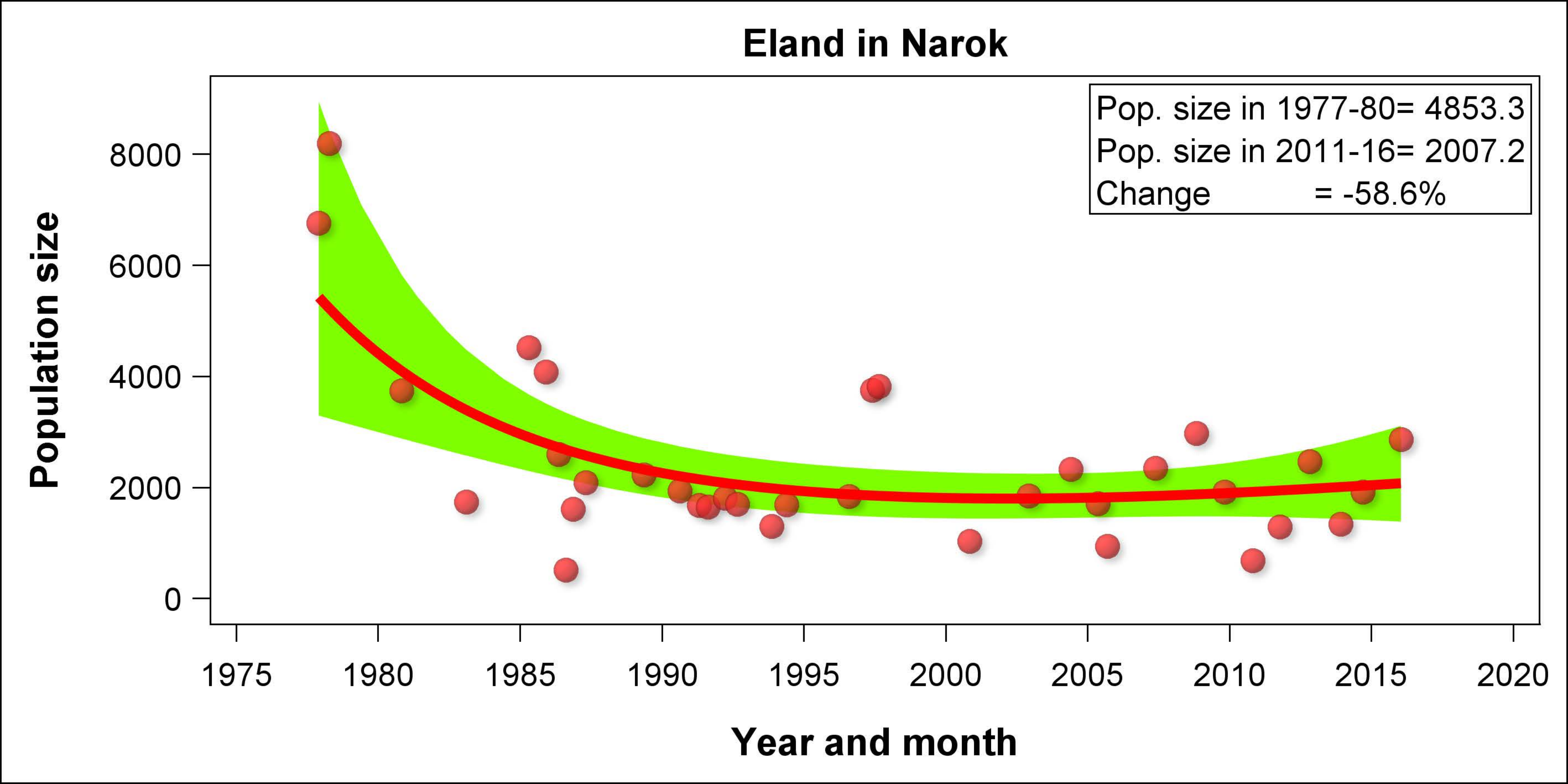
Eland in Narok
Pop. size in 1977-80= 4853.3
Pop. size in 2011-16= 2007.2
Change = -58.6%
Population size
0
2000
4000
6000
8000
1975
1980
1985
1990
1995
2000
2005
2010
2015
2020
Year and month

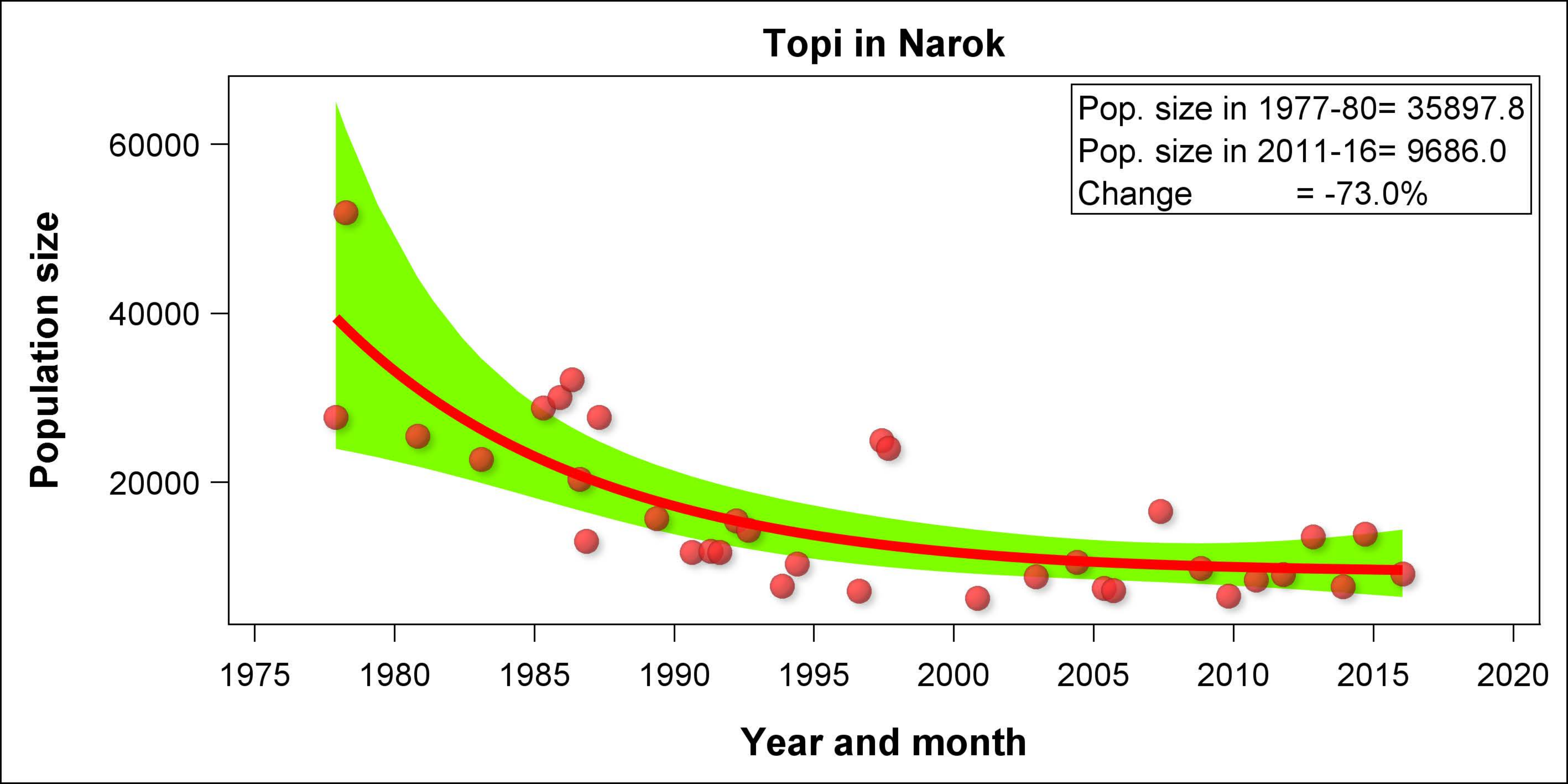
Topi in Narok
Pop. size in 1977-80= 35897.8
Pop. size in 2011-16= 9686.0
Change = -73.0%
Population size
60000
40000
20000
1975
1980
1985
1990
1995
2000
2005
2010
2015
2020
Year and month

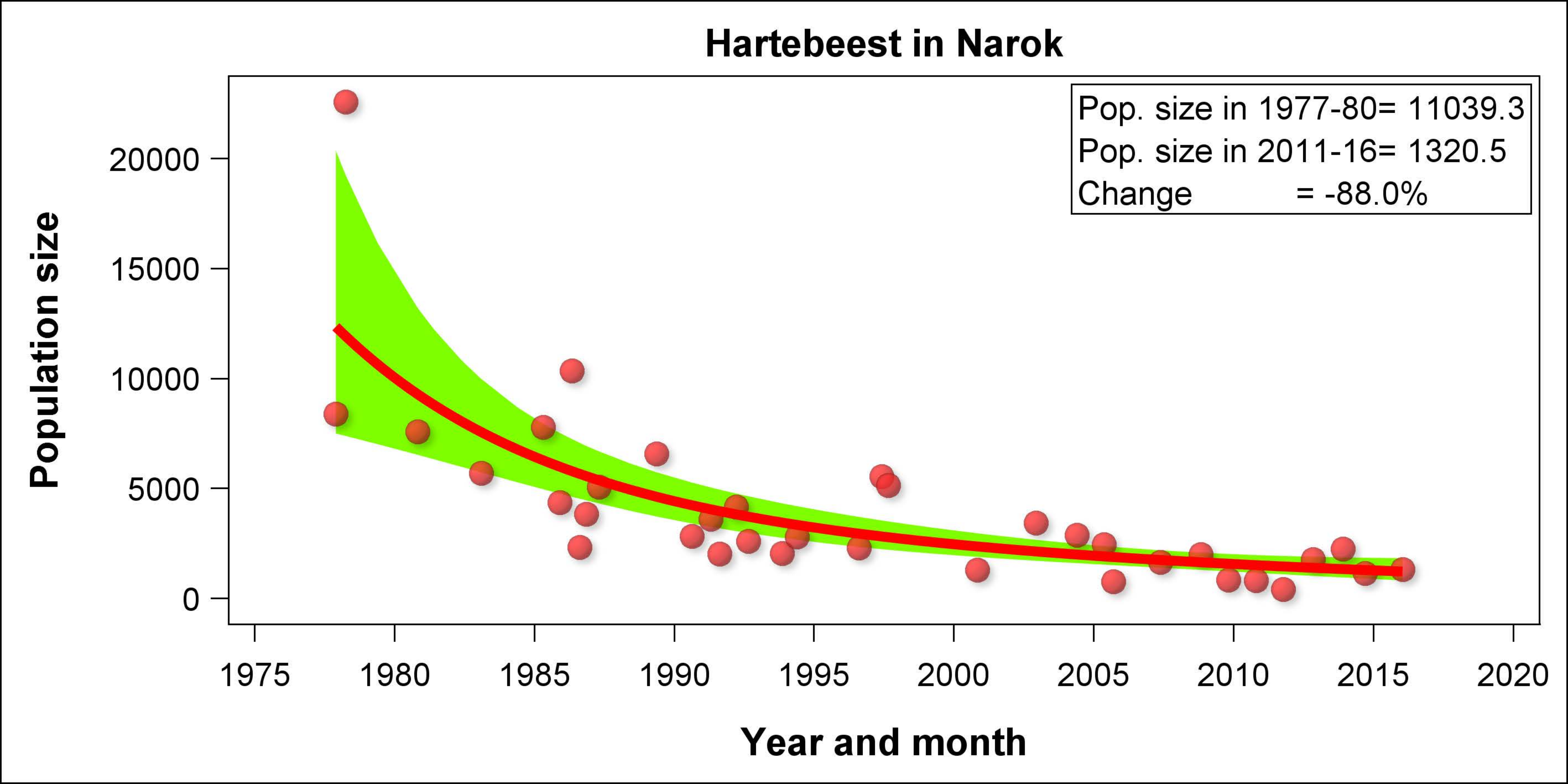

Hartebeest in Narok
Pop. size in 1977-80= 11039.3
Pop. size in 2011-16= 1320.5
Change = -88.0%
Population size
20000
15000
10000
5000
0
1975
1980
1985
1990
1995
2000
2005
2010
2015
2020
Year and month

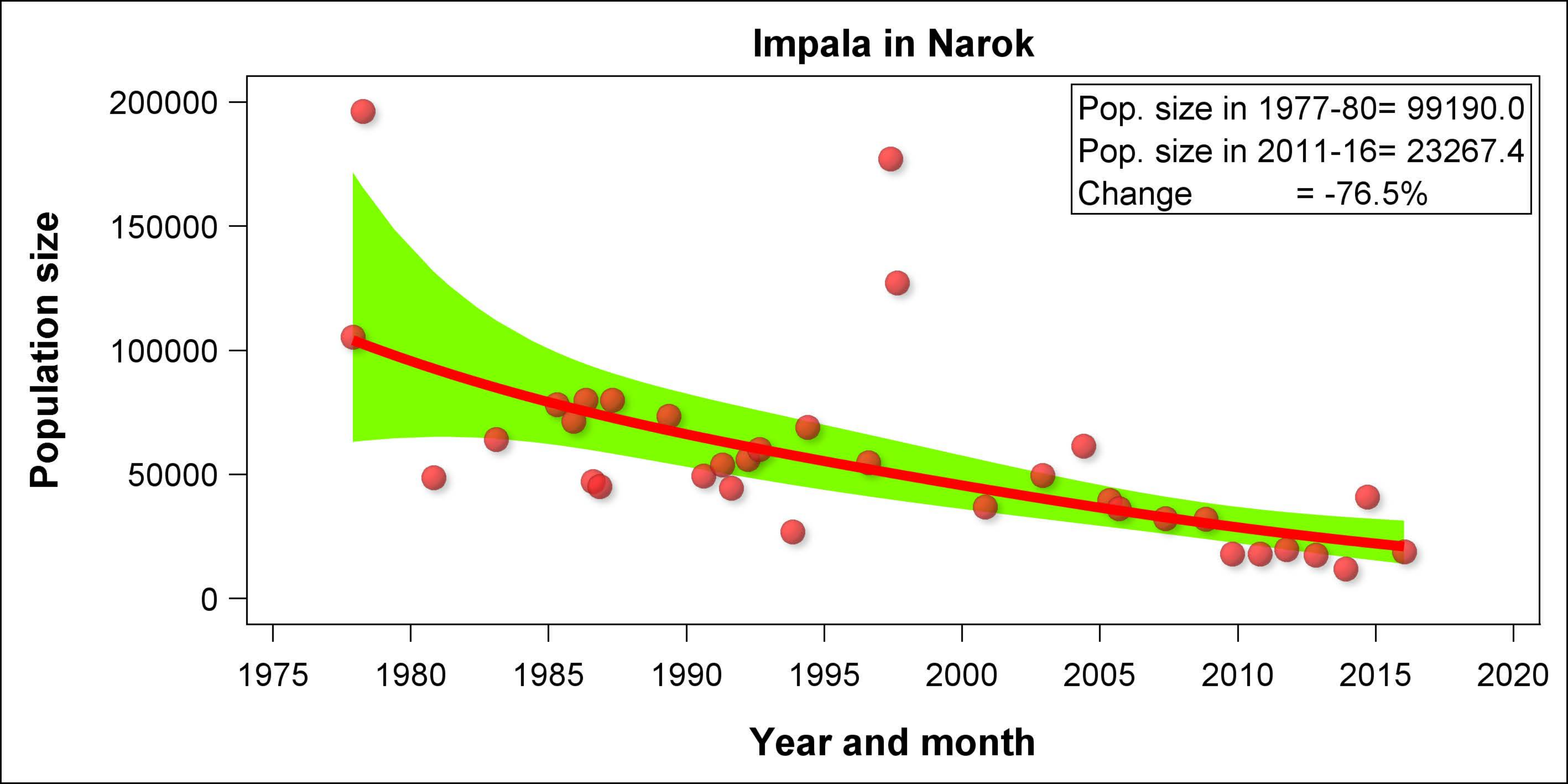

Impala in Narok
Pop. size in 1977-80= 99190.0
Pop. size in 2011-16= 23267.4
Change = -76.5%
Population size
200000
150000
100000
50000
0
1975
1980
1985
1990
1995
2000
2005
2010
2015
2020
Year and month

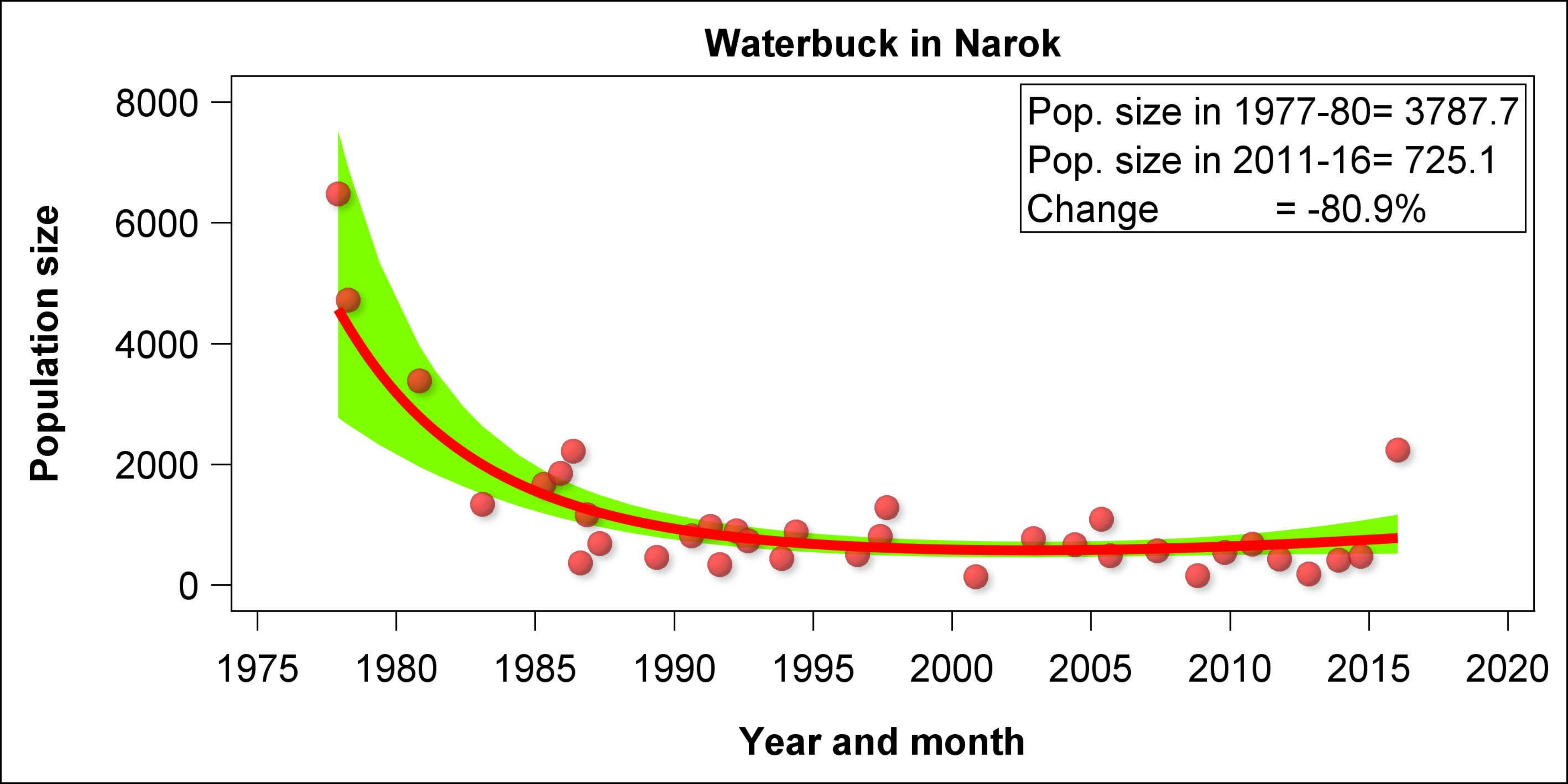
Waterbuck in Narok
Pop. size in 1977-80= 3787.7
Pop. size in 2011-16= 725.1
Change = -80.9%
Population size
0
2000
4000
6000
8000
1975
1980
1985
1990
1995
2000
2005
2010
2015
2020
Year and month
