# Supplementary material for: Extreme Wildlife Declines and Concurrent Increase in Livestock Numbers in Kenya: What Are the Causes?
Source: PLoS One. 2016 Sep 27;11(9):e0163249. doi: 10.1371/journal.pone.0163249 (PMC5039022; doi:10.1371/journal.pone.0163249)
Supplement: S3 Fig — The solid red line is the fitted trend curve and the shaded chartreuse band is the pointwise 95% confidence band. The estimated average population size in 1977–1980 and 2011–2014 and the percentage change in population size between the two periods are provided in the inset. (PDF) [file pone.0163249.s013.pdf]

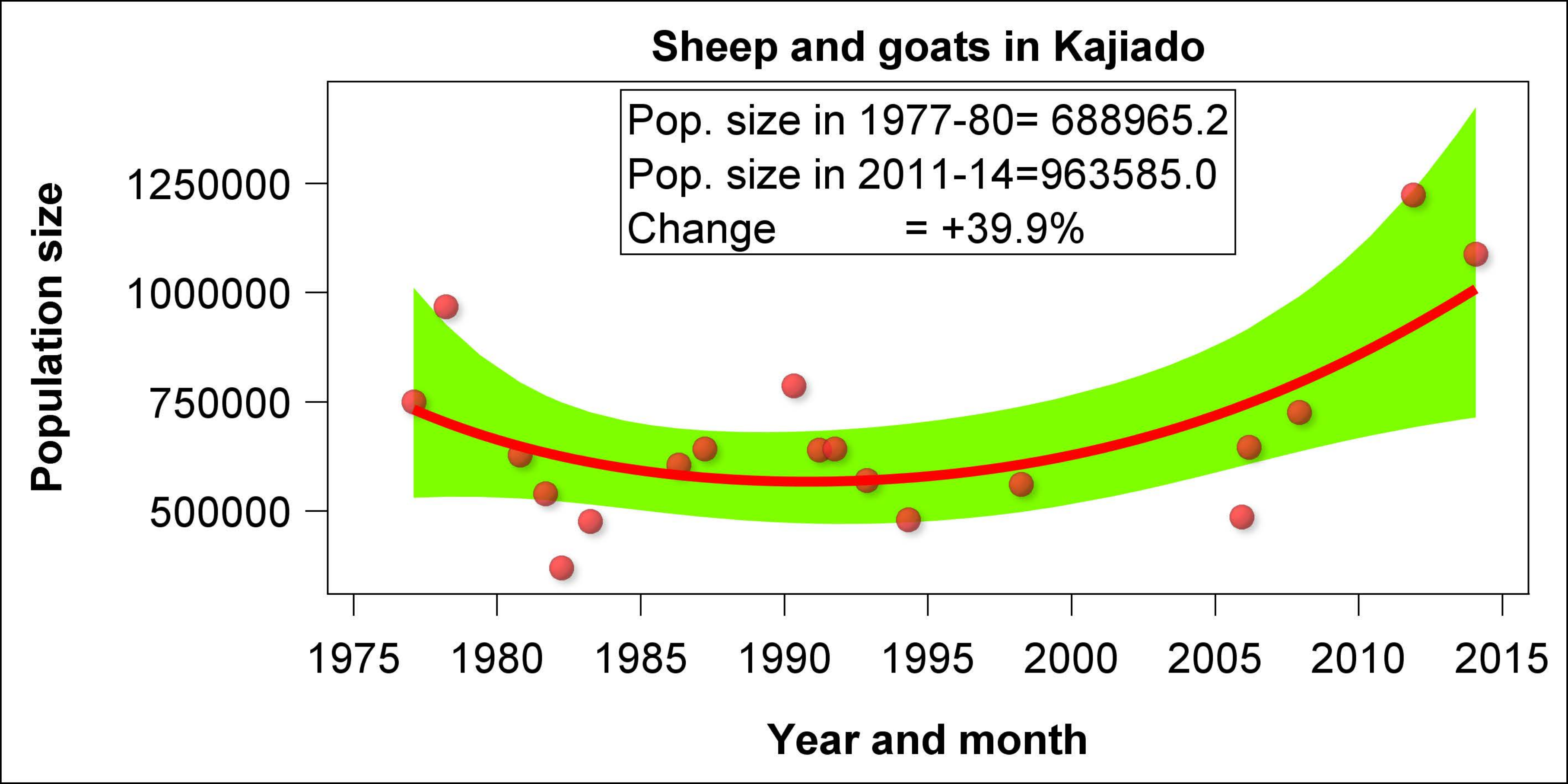
Sheep and goats in Kajiado
Pop. size in 1977-80= 688965.2
Pop. size in 2011-14=963585.0
Change = +39.9%
Population size
1250000
1000000
750000
500000
1975
1980
1985
1990
1995
2000
2005
2010
2015
Year and month

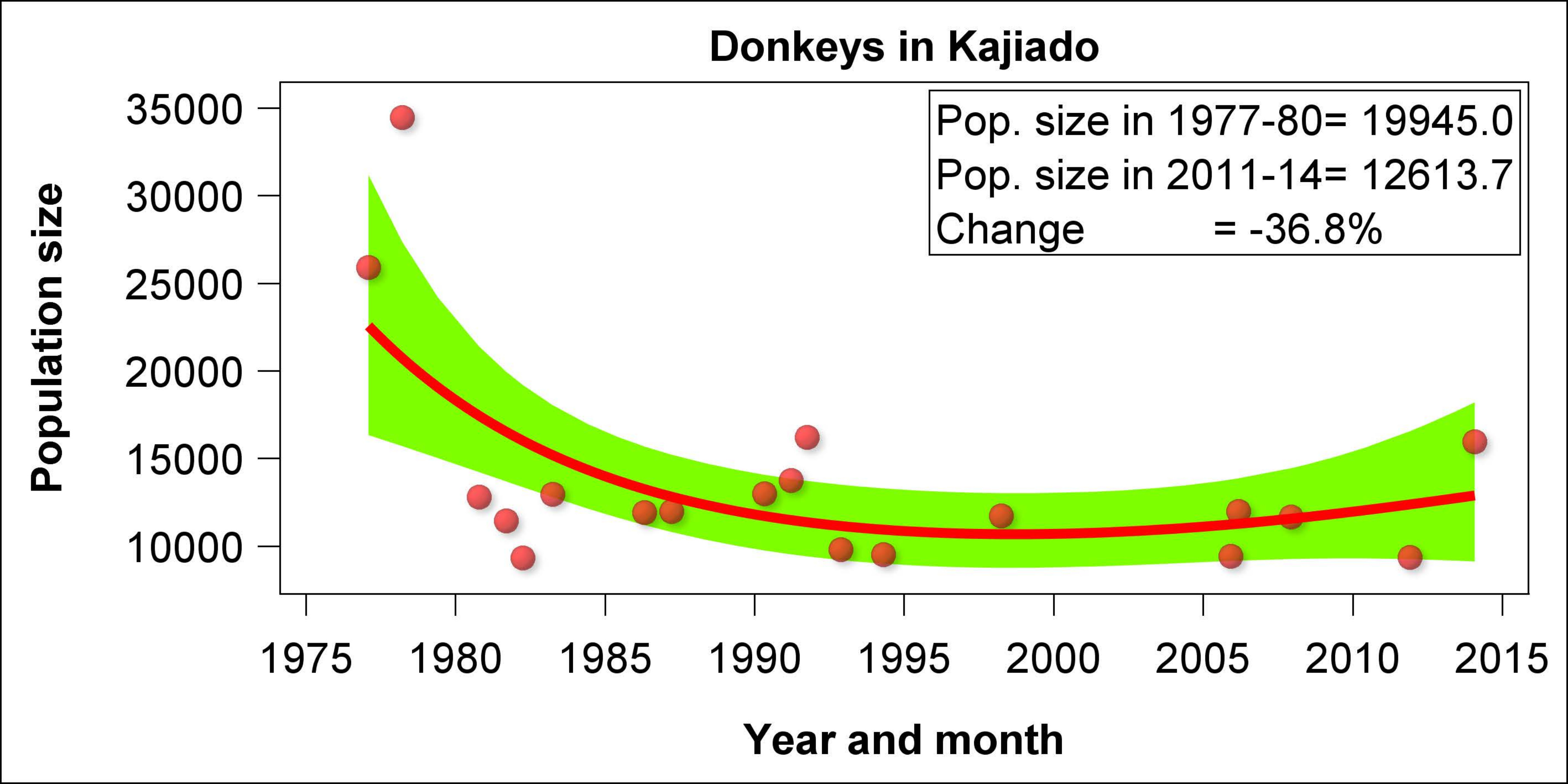
Donkeys in Kajiado
Pop. size in 1977-80= 19945.0
Pop. size in 2011-14= 12613.7
Change = -36.8%
Population size
35000
30000
25000
20000
15000
10000
1975
1980
1985
1990
1995
2000
2005
2010
2015
Year and month

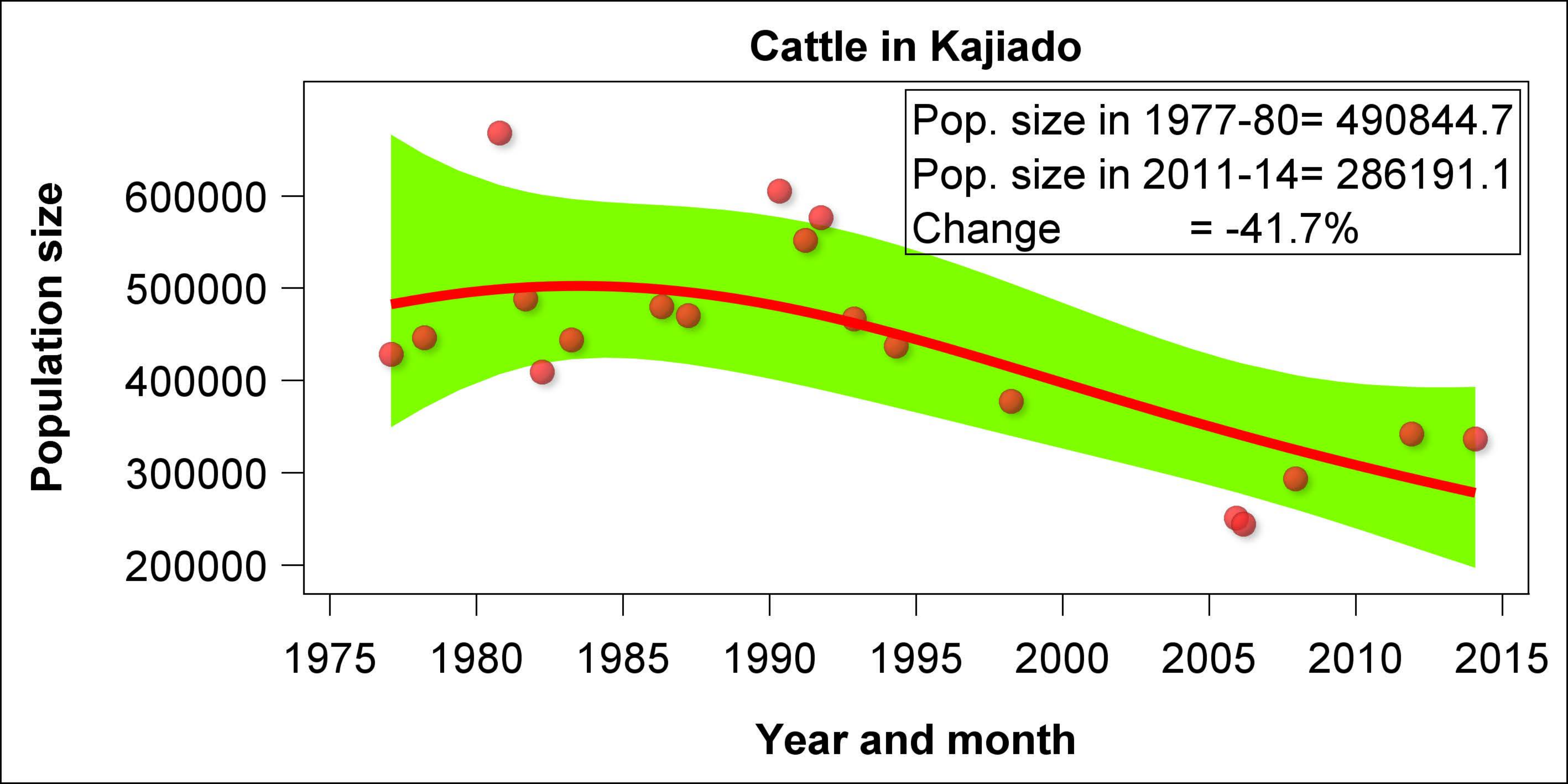

Cattle in Kajiado
Pop. size in 1977-80= 490844.7
Pop. size in 2011-14= 286191.1
Change = -41.7%
Population size
600000
500000
400000
300000
200000
1975
1980
1985
1990
1995
2000
2005
2010
2015
Year and month

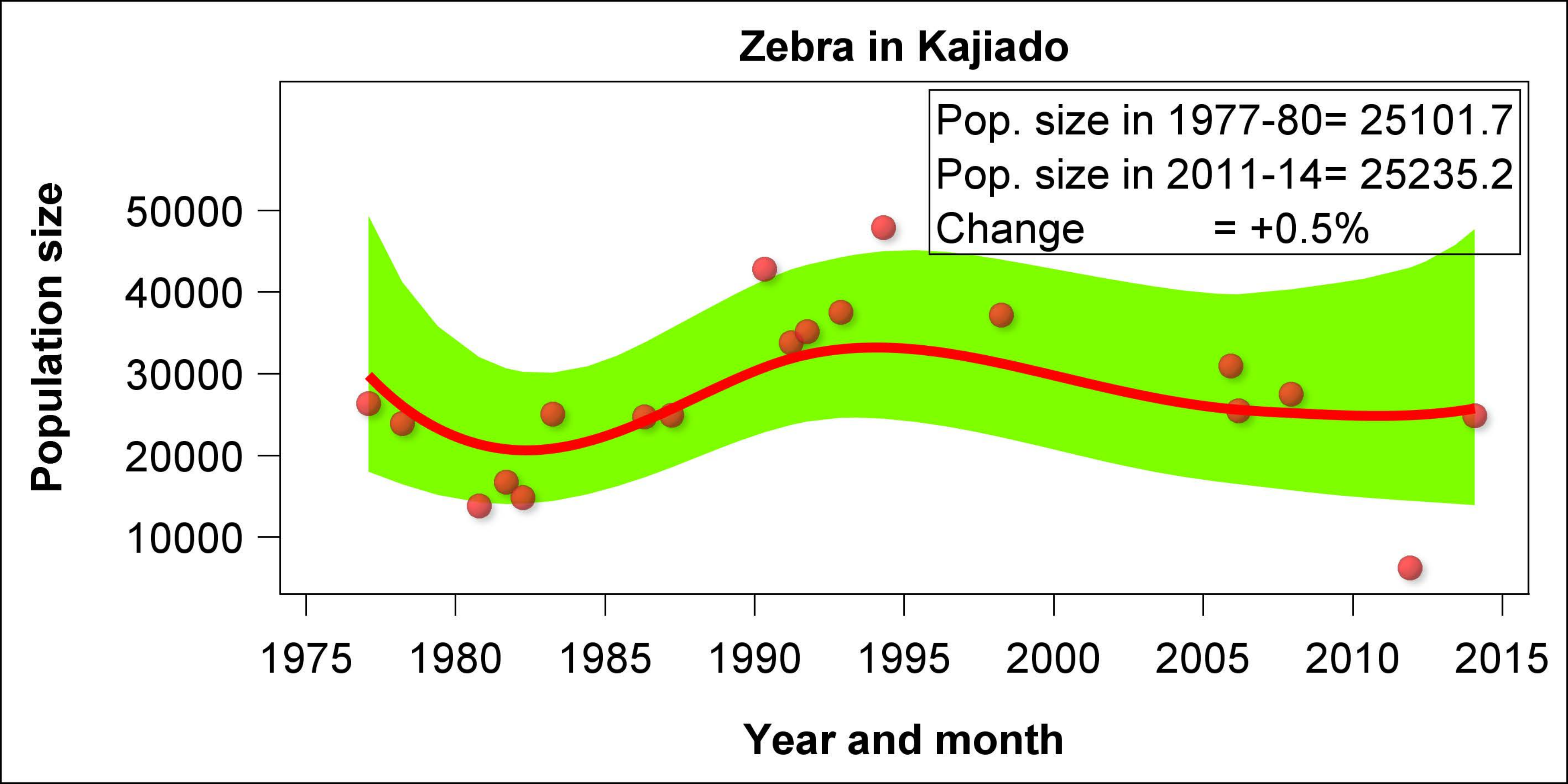

Zebra in Kajiado
Pop. size in 1977-80= 25101.7
Pop. size in 2011-14= 25235.2
Change = +0.5%
Population size
50000
40000
30000
20000
10000
1975
1980
1985
1990
1995
2000
2005
2010
2015
Year and month

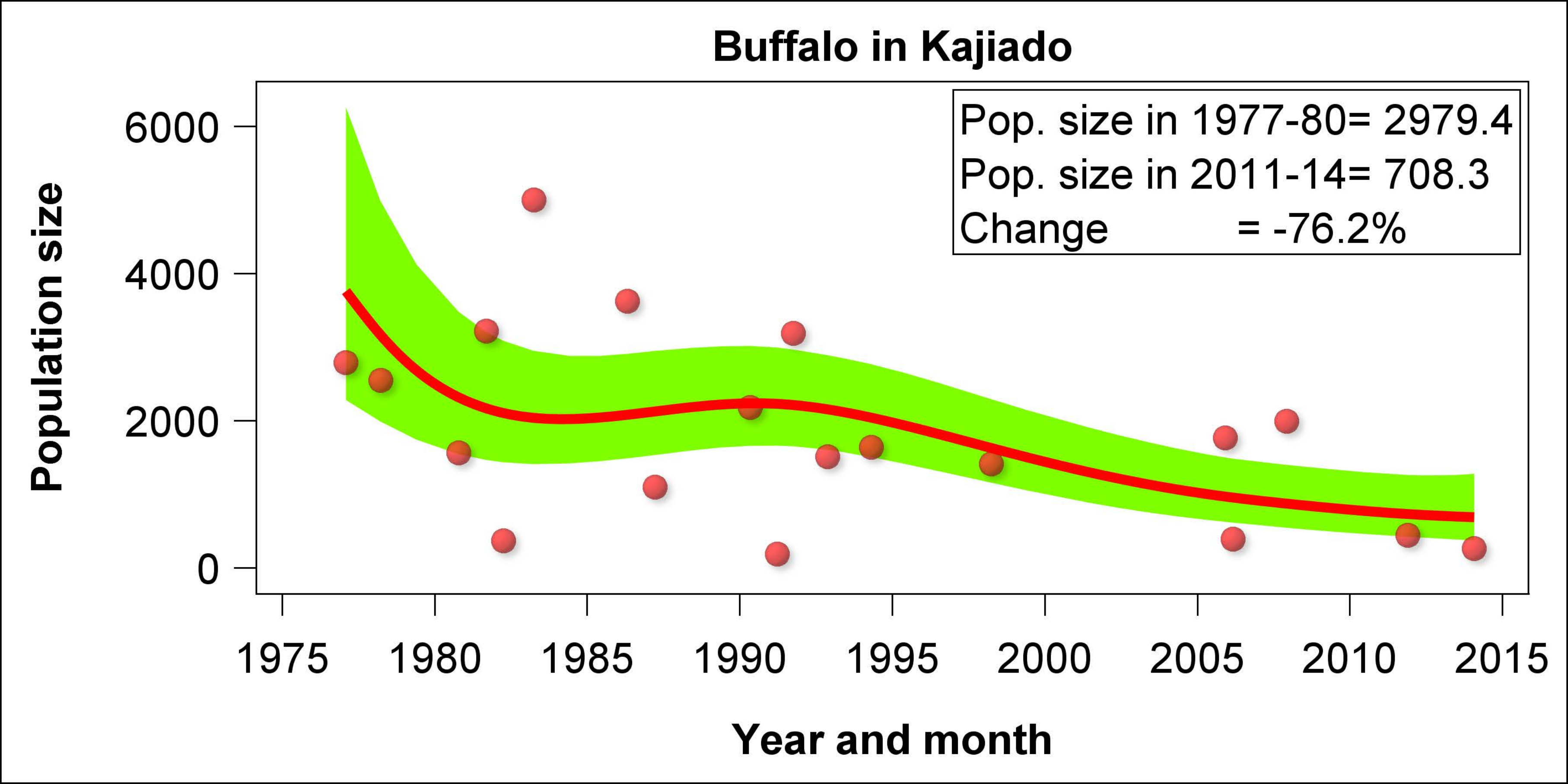

Buffalo in Kajiado
Pop. size in 1977-80= 2979.4
Pop. size in 2011-14= 708.3
Change = -76.2%
6000
4000
2000
0
Population size
1975
1980
1985
1990
1995
2000
2005
2010
2015
Year and month

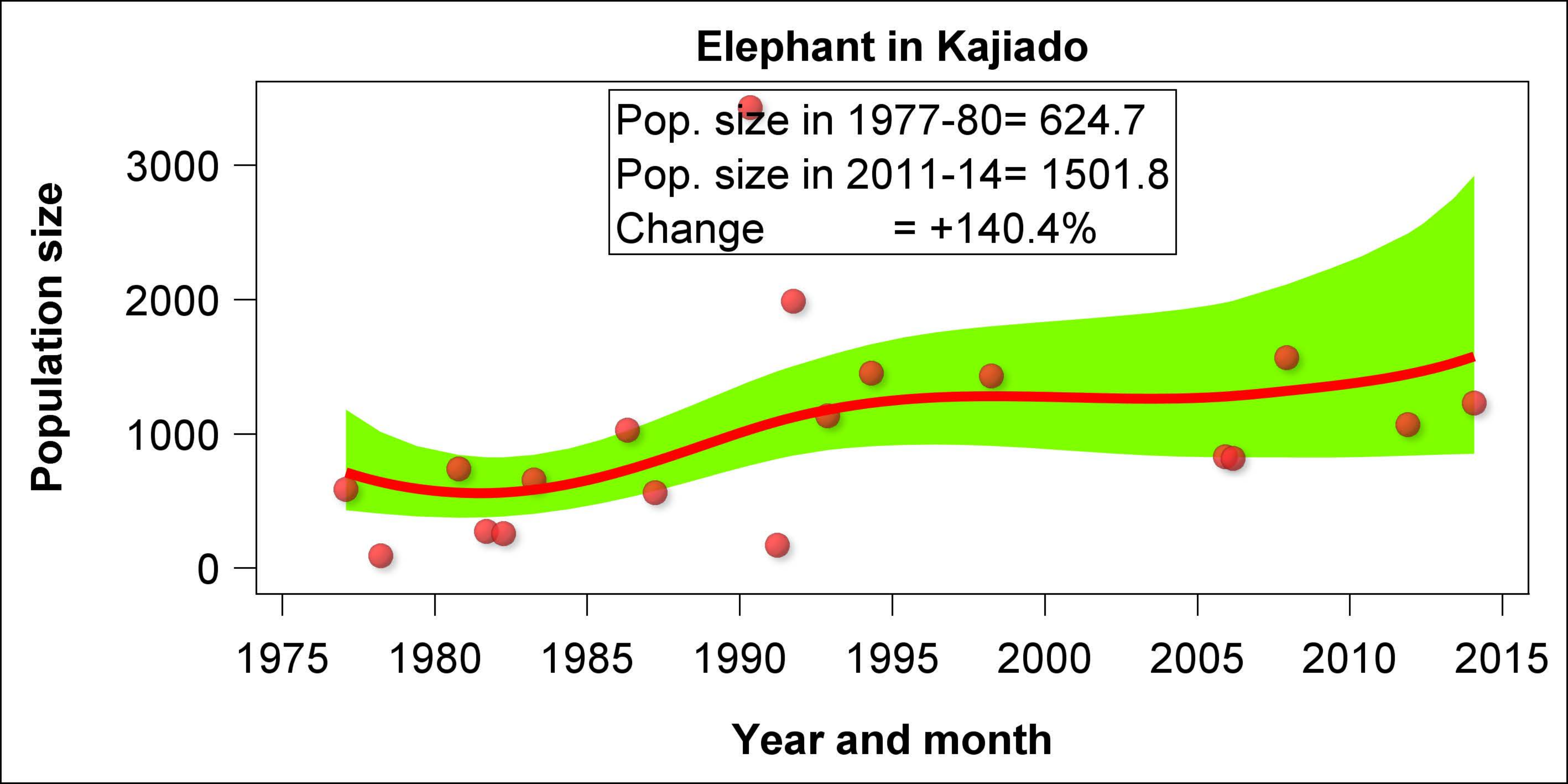
Elephant in Kajiado
Pop. size in 1977-80= 624.7
Pop. size in 2011-14= 1501.8
Change = +140.4%
Population size
0
1000
2000
3000
1975
1980
1985
1990
1995
2000
2005
2010
2015
Year and month

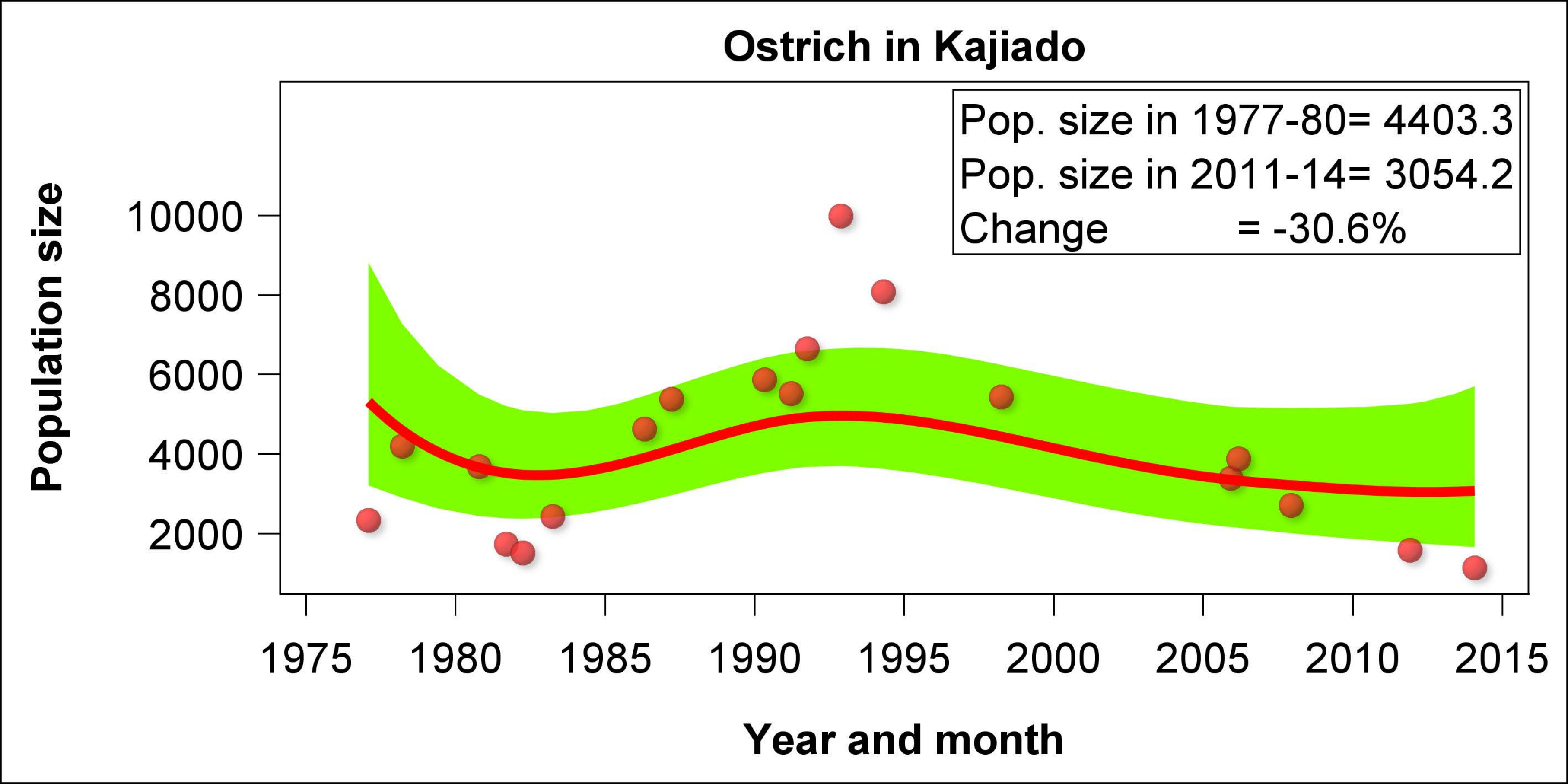

Ostrich in Kajiado
Pop. size in 1977-80= 4403.3
Pop. size in 2011-14= 3054.2
Change = -30.6%
Population size
10000
8000
6000
4000
2000
1975
1980
1985
1990
1995
2000
2005
2010
2015
Year and month

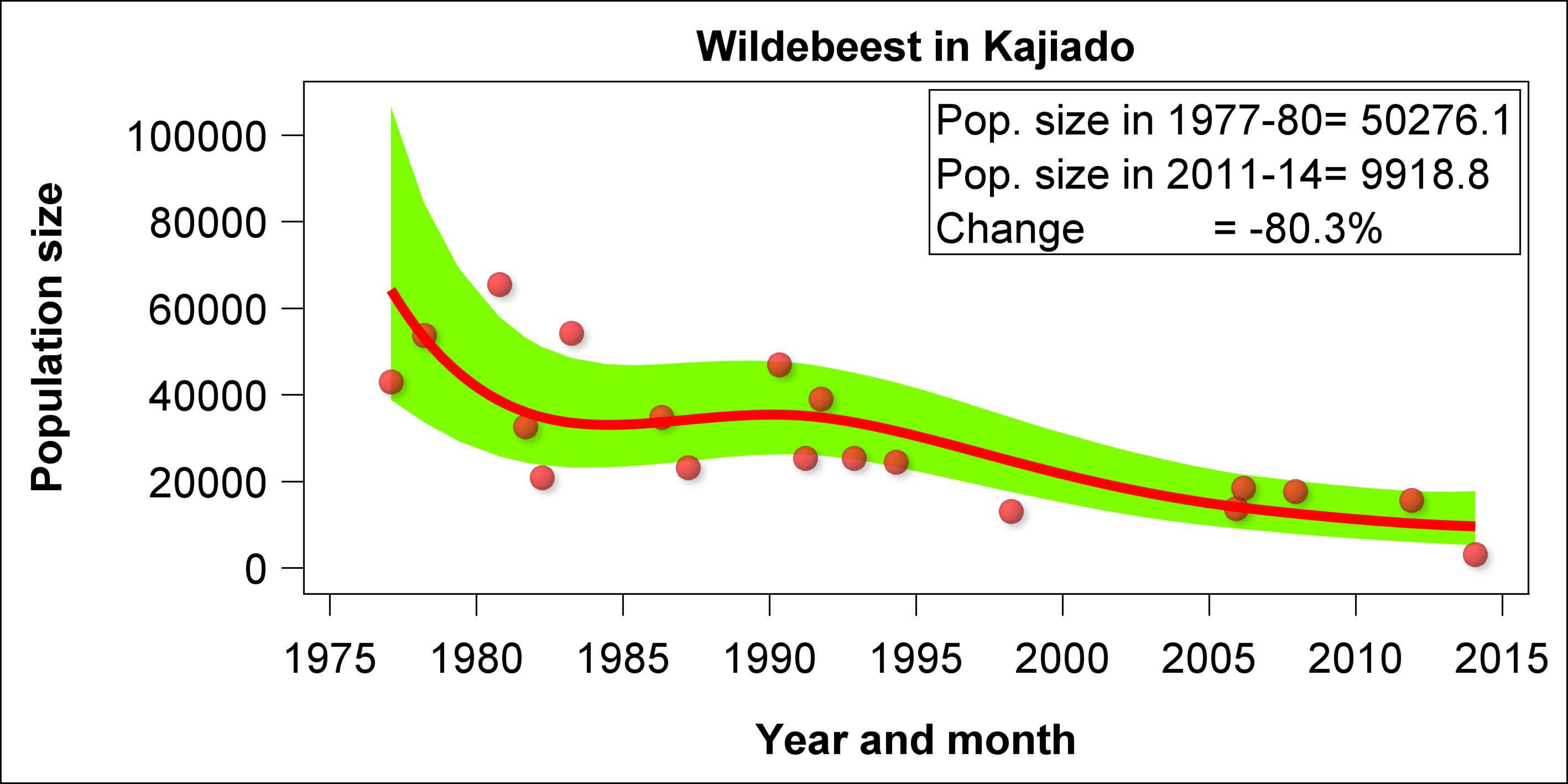

Wildebeest in Kajiado
Pop. size in 1977-80= 50276.1
Pop. size in 2011-14= 9918.8
Change = -80.3%
Population size
100000
80000
60000
40000
20000
0
1975
1980
1985
1990
1995
2000
2005
2010
2015
Year and month

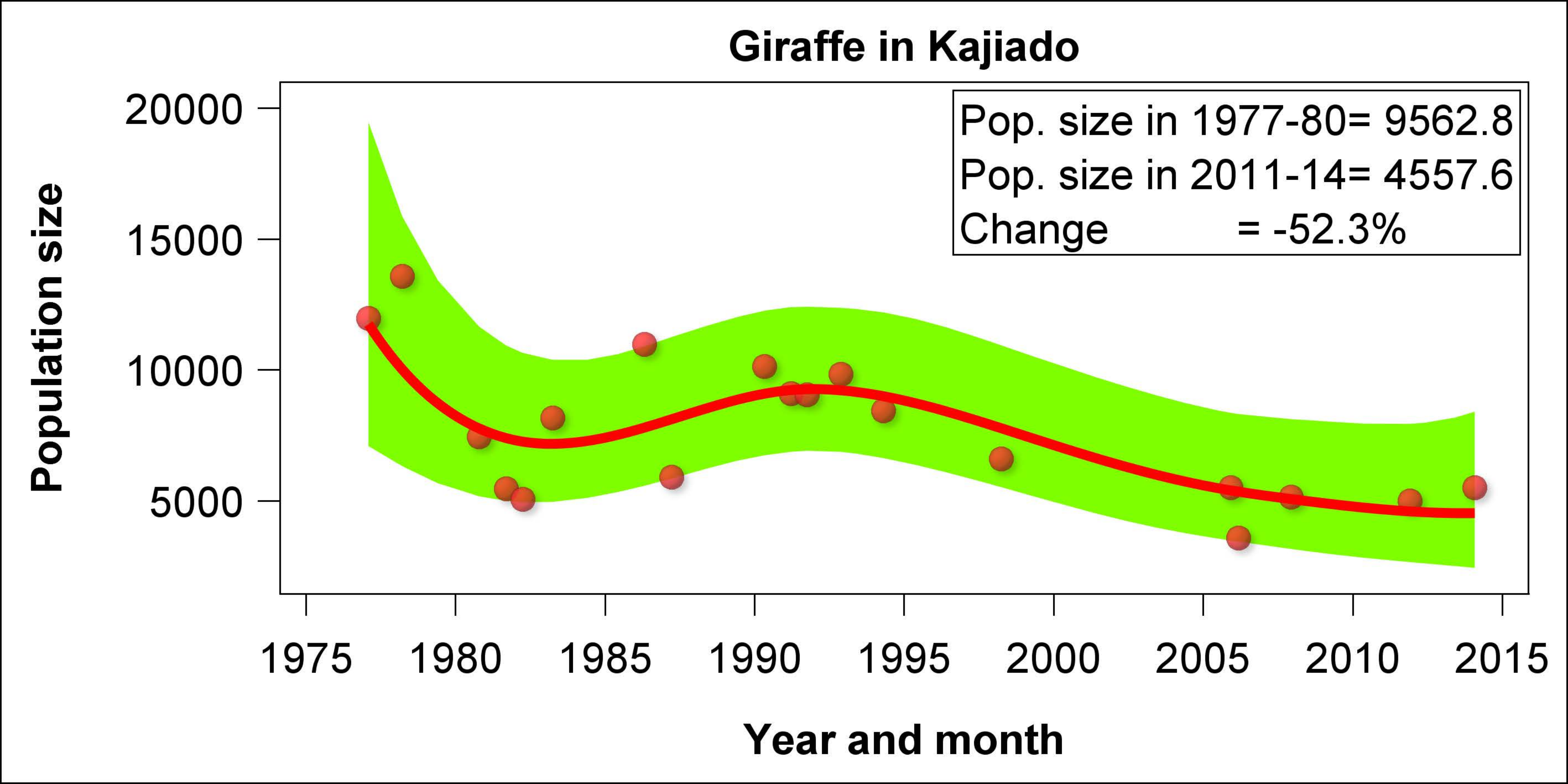
Giraffe in Kajiado
Pop. size in 1977-80= 9562.8
Pop. size in 2011-14= 4557.6
Change = -52.3%
Population size
20000
15000
10000
5000
1975
1980
1985
1990
1995
2000
2005
2010
2015
Year and month

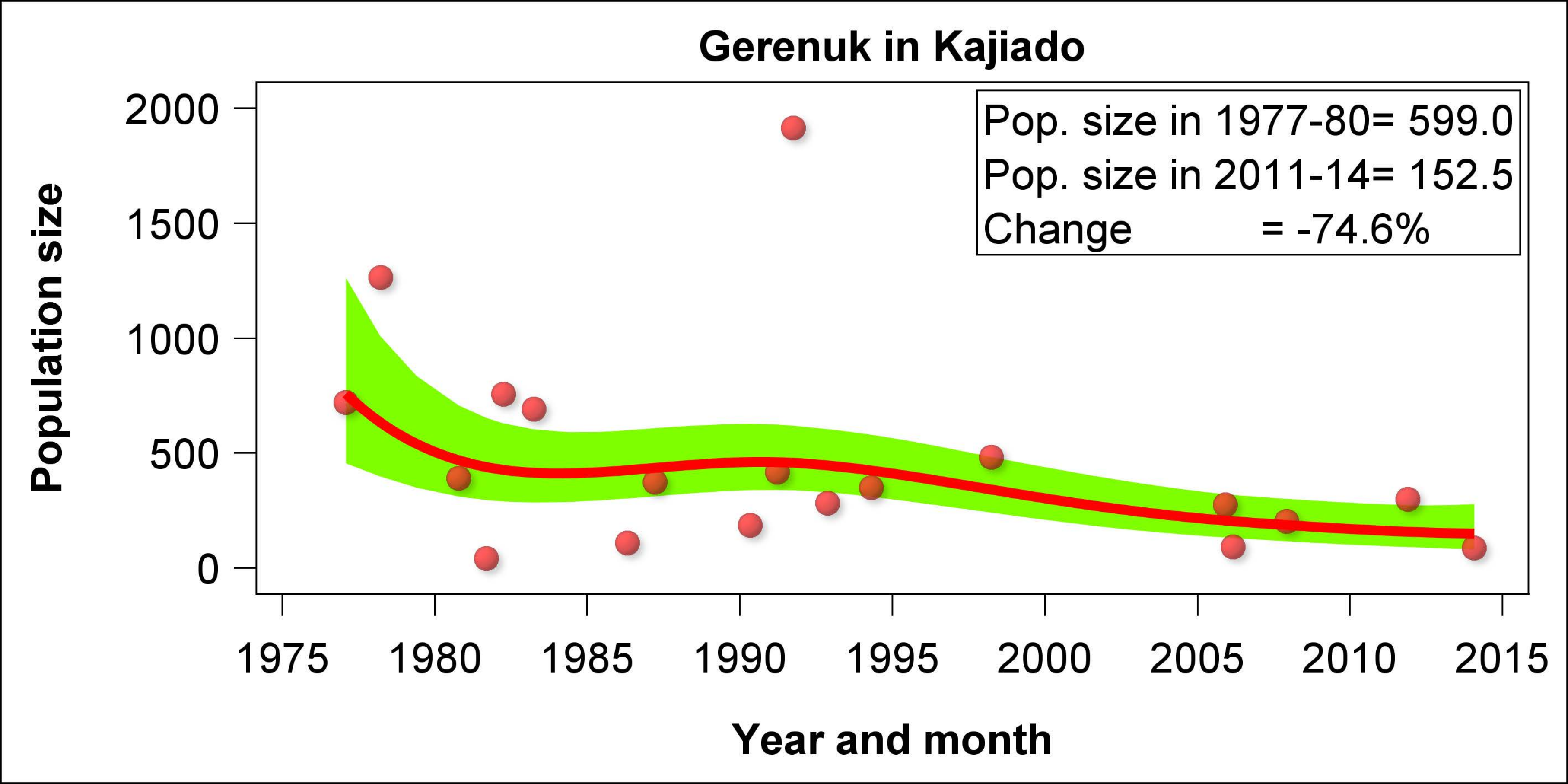
Gerenuk in Kajiado
Pop. size in 1977-80= 599.0
Pop. size in 2011-14= 152.5
Change = -74.6%
Population size
2000
1500
1000
500
0
1975
1980
1985
1990
1995
2000
2005
2010
2015
Year and month

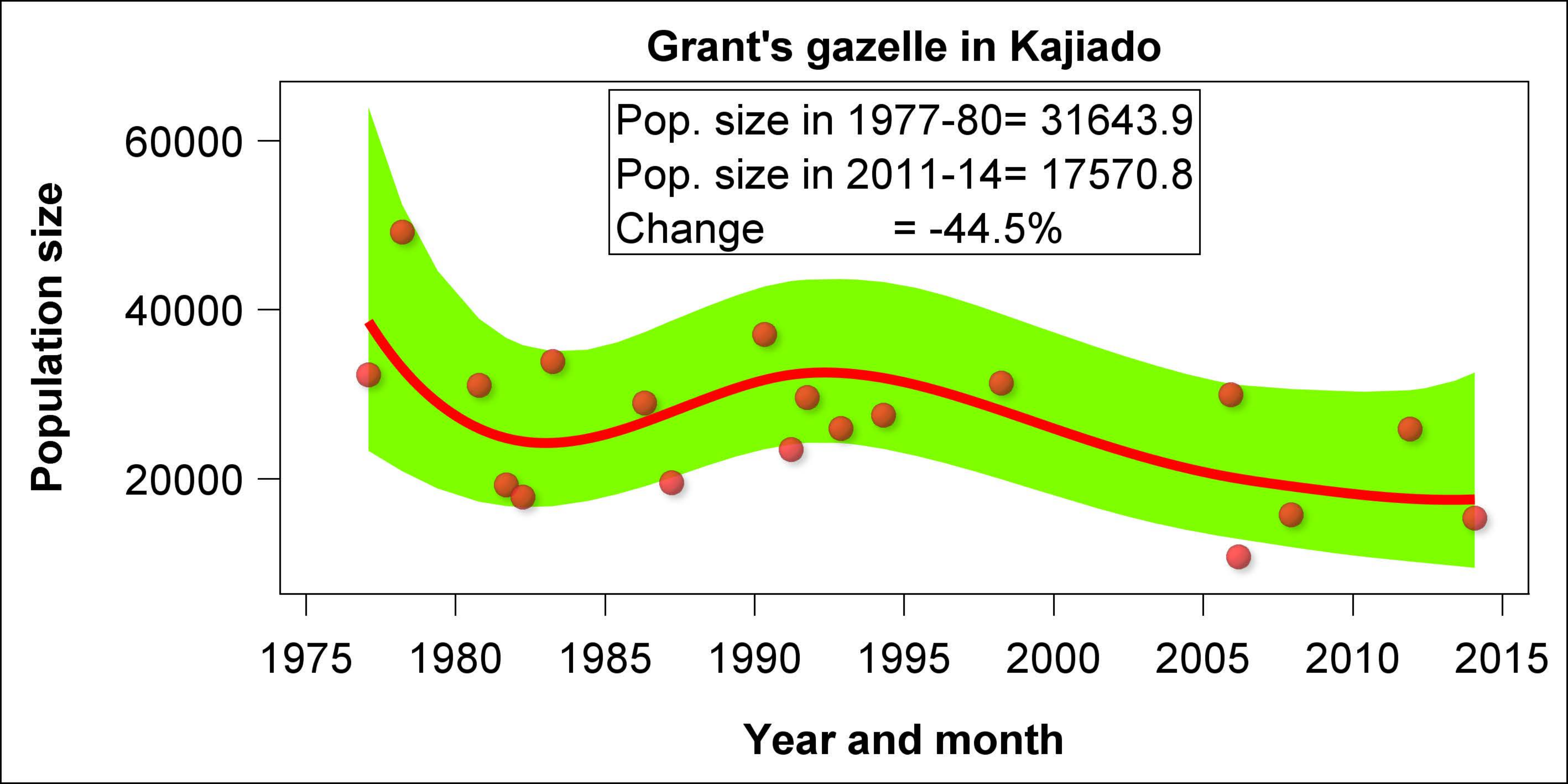

Grant's gazelle in Kajiado
Pop. size in 1977-80= 31643.9
Pop. size in 2011-14= 17570.8
Change = -44.5%
Population size
60000
40000
20000
1975
1980
1985
1990
1995
2000
2005
2010
2015
Year and month

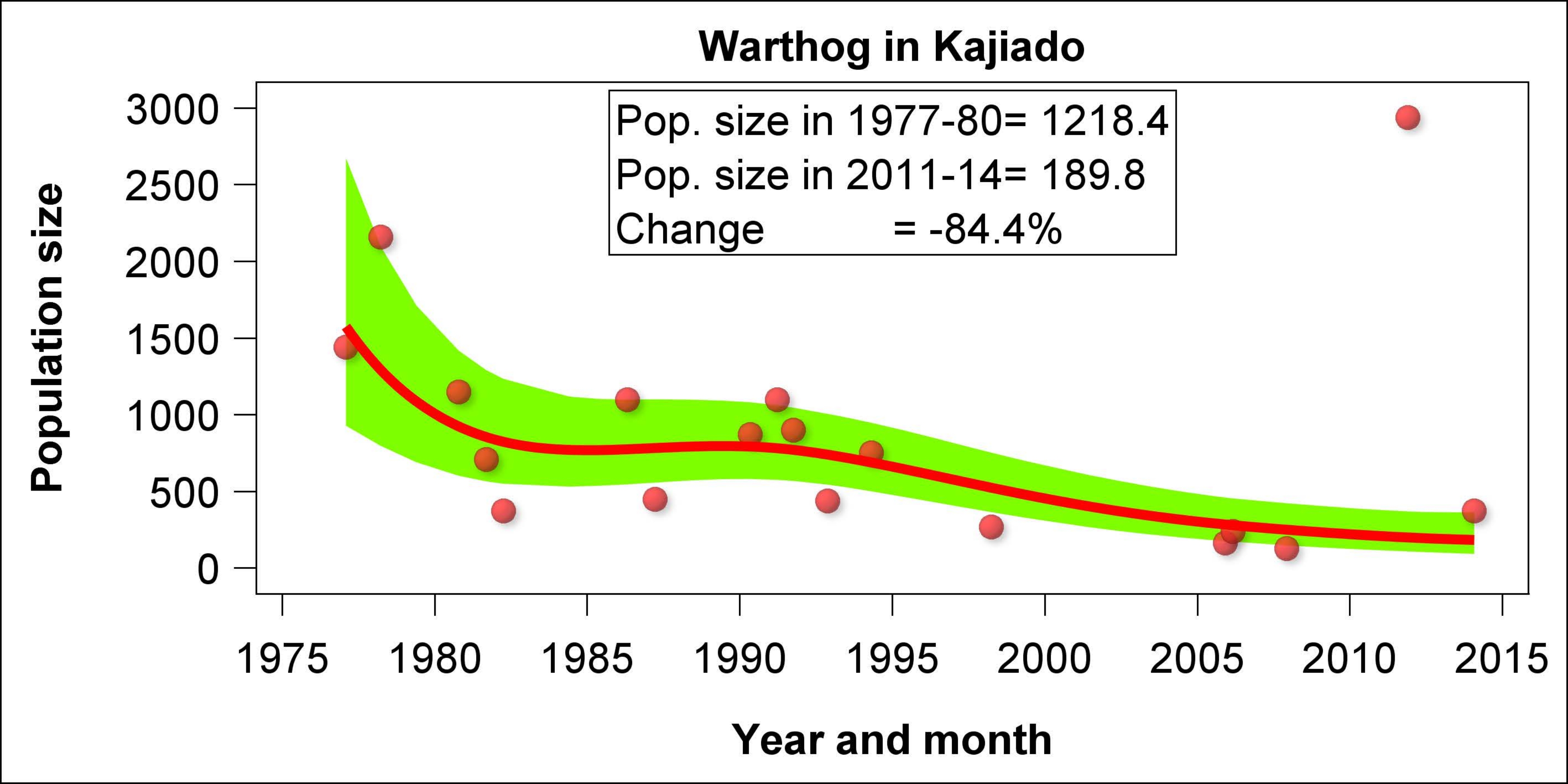
Warthog in Kajiado
Pop. size in 1977-80= 1218.4
Pop. size in 2011-14= 189.8
Change = -84.4%
Population size
0
500
1000
1500
2000
2500
3000
1975
1980
1985
1990
1995
2000
2005
2010
2015
Year and month

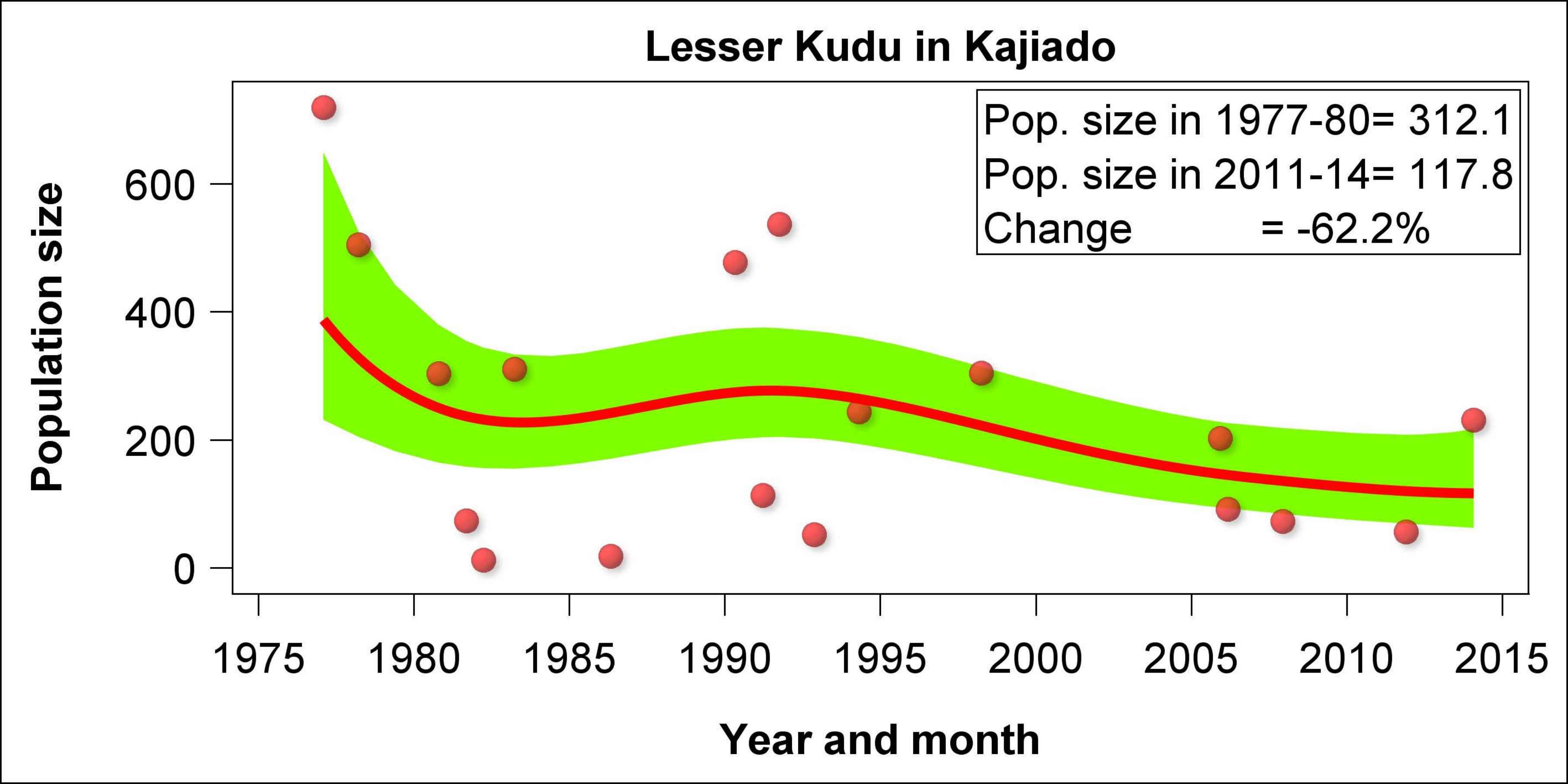
Lesser Kudu in Kajiado
Pop. size in 1977-80= 312.1
Pop. size in 2011-14= 117.8
Change = -62.2%
Population size
600
400
200
0
1975
1980
1985
1990
1995
2000
2005
2010
2015
Year and month

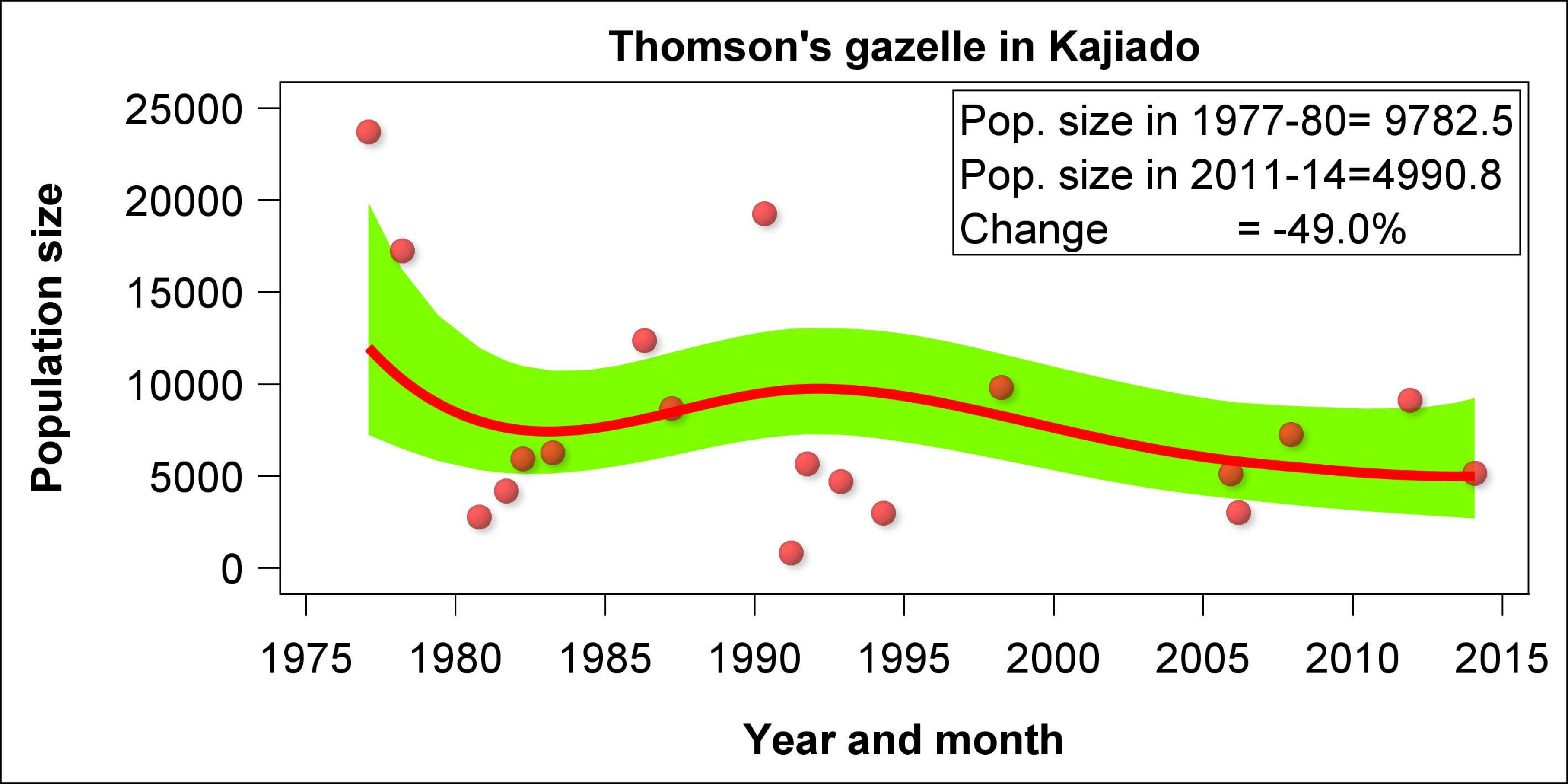
Thomson's gazelle in Kajiado
Pop. size in 1977-80= 9782.5
Pop. size in 2011-14=4990.8
Change = -49.0%
Population size
25000
20000
15000
10000
5000
0
1975
1980
1985
1990
1995
2000
2005
2010
2015
Year and month

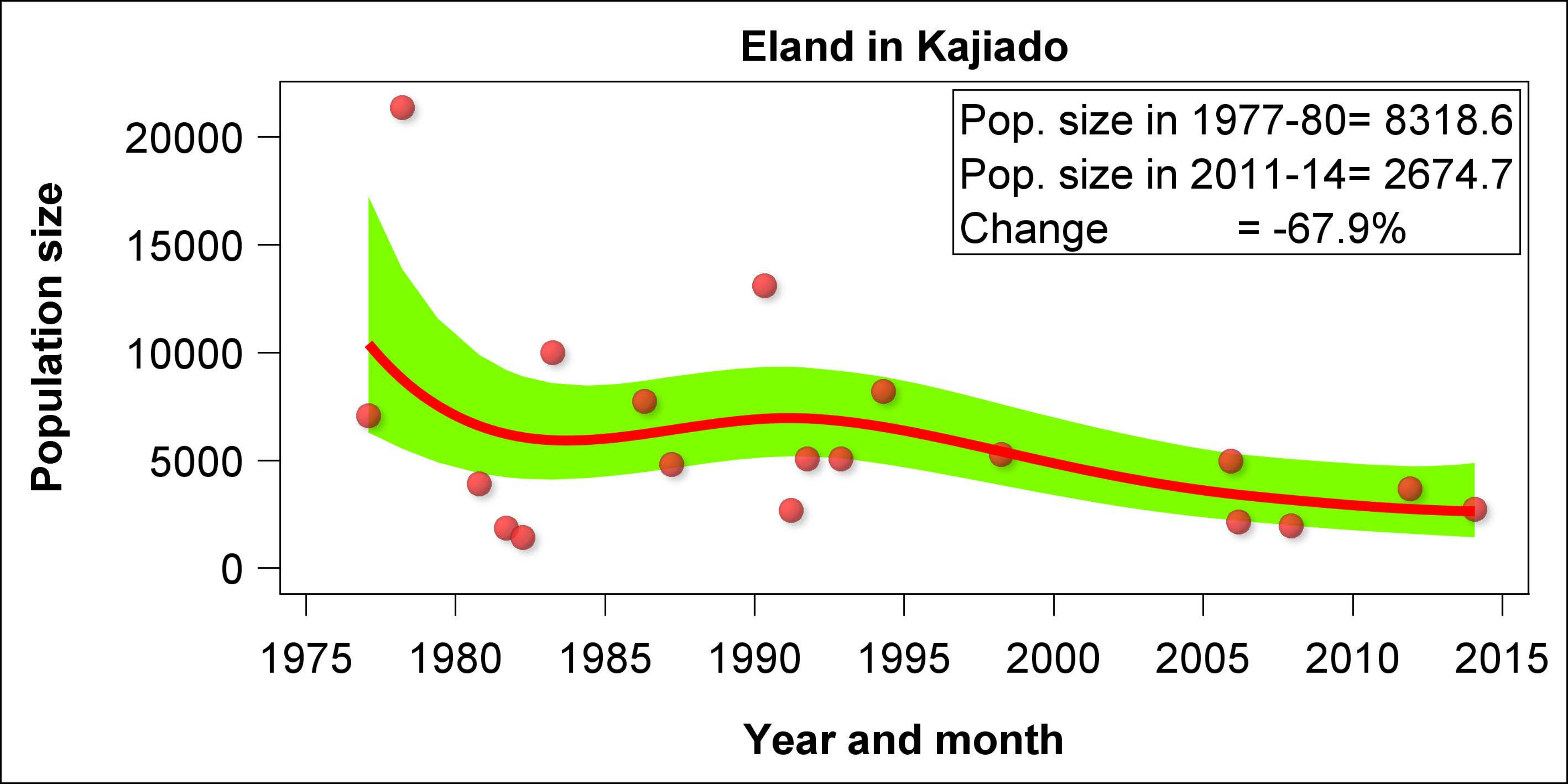

Eland in Kajiado
Pop. size in 1977-80= 8318.6
Pop. size in 2011-14= 2674.7
Change = -67.9%
Population size
20000
15000
10000
5000
0
1975
1980
1985
1990
1995
2000
2005
2010
2015
Year and month

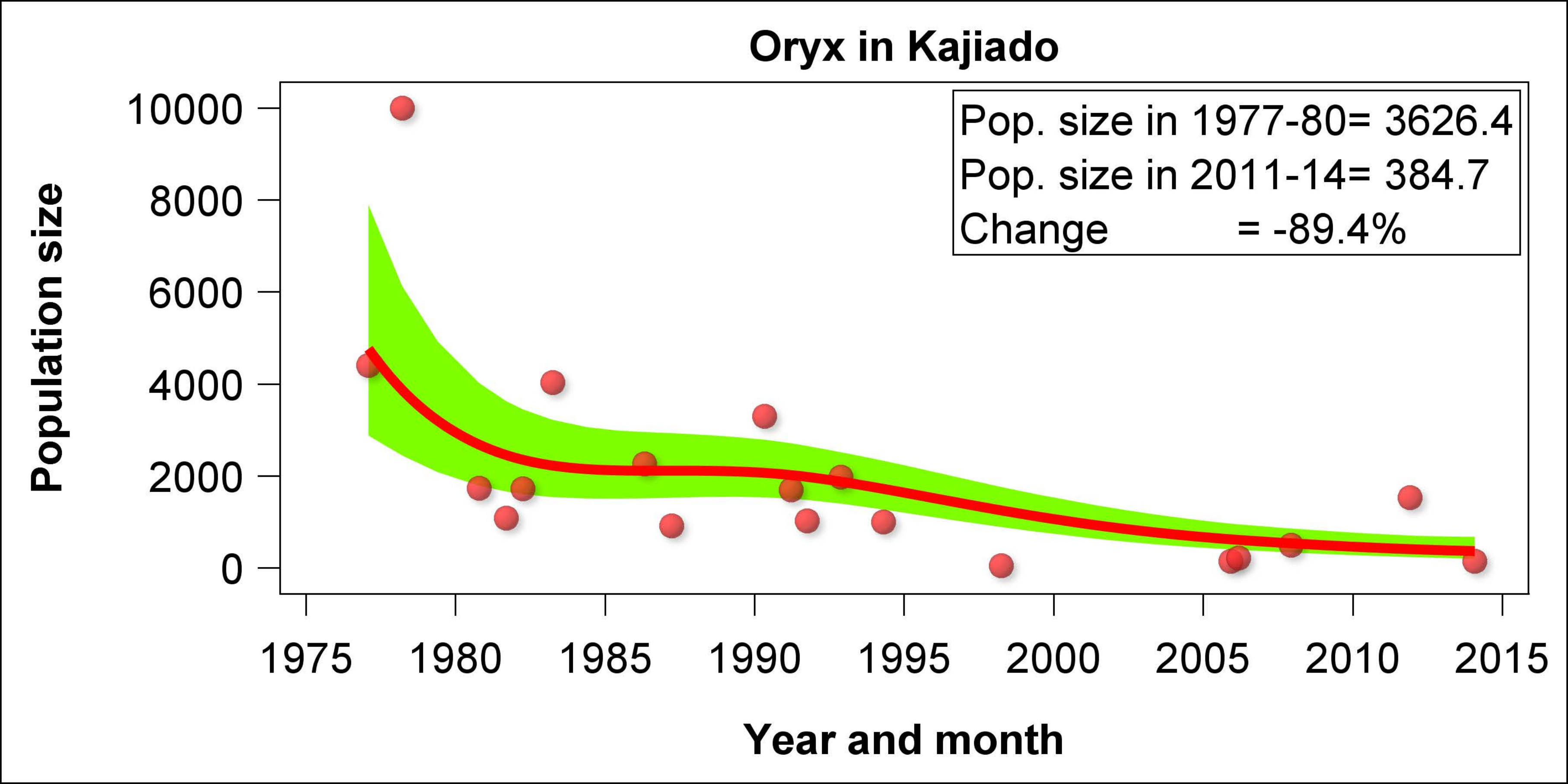

Oryx in Kajiado
Pop. size in 1977-80= 3626.4
Pop. size in 2011-14= 384.7
Change = -89.4%
Population size
10000
8000
6000
4000
2000
0
1975
1980
1985
1990
1995
2000
2005
2010
2015
Year and month

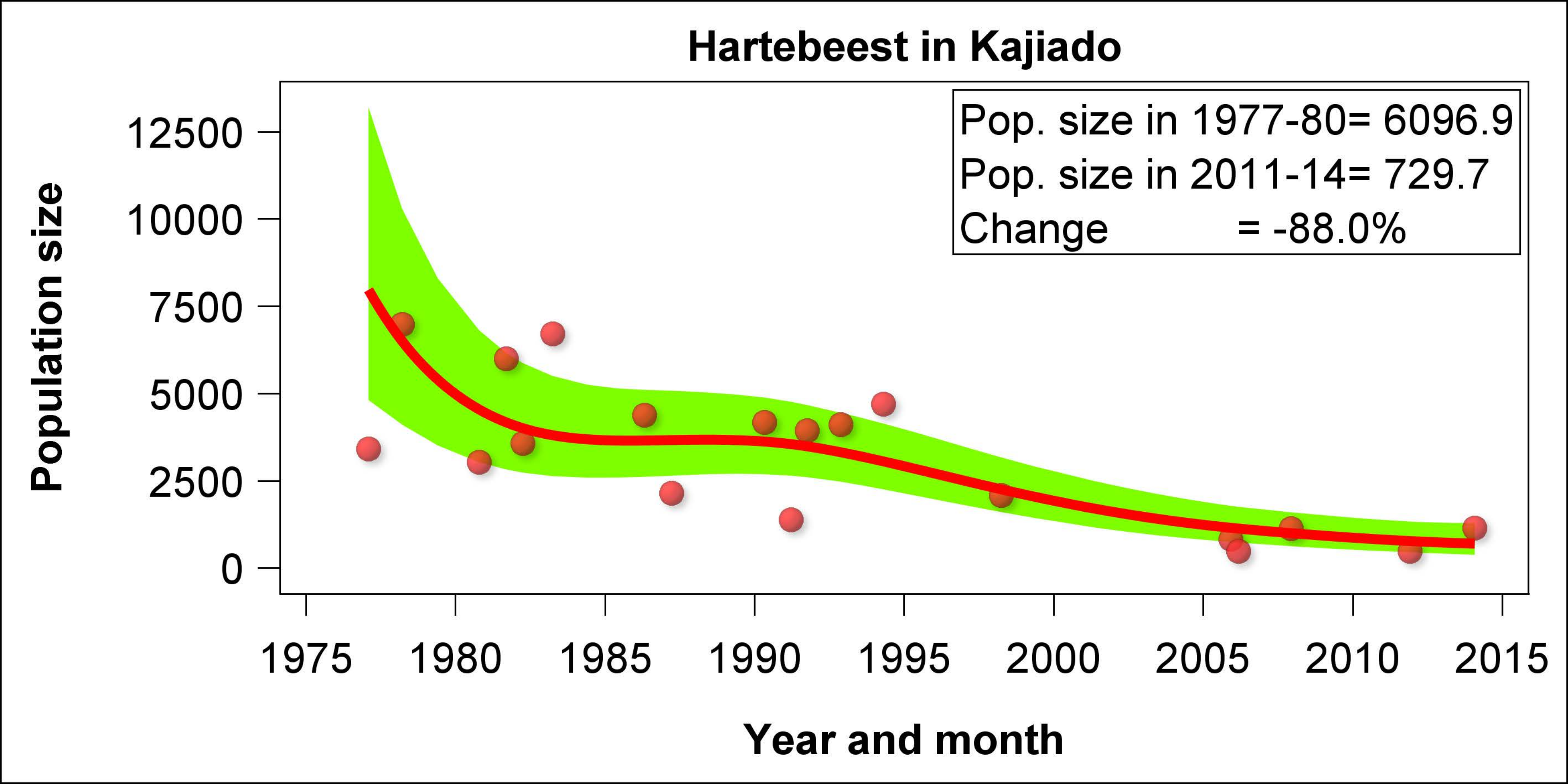
Hartebeest in Kajiado
Pop. size in 1977-80= 6096.9
Pop. size in 2011-14= 729.7
Change = -88.0%
Population size
12500
10000
7500
5000
2500
0
1975
1980
1985
1990
1995
2000
2005
2010
2015
Year and month

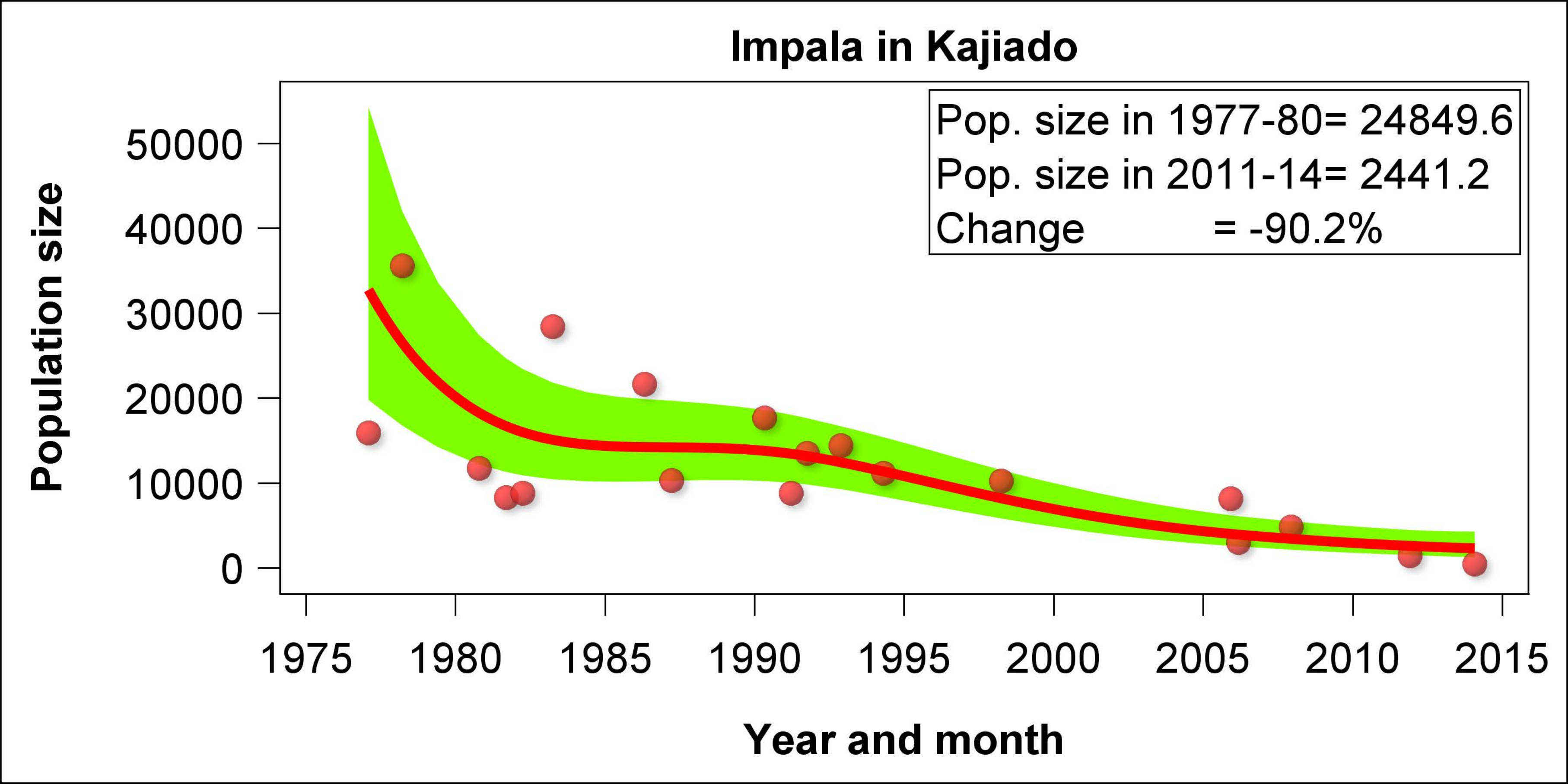

Impala in Kajiado
Pop. size in 1977-80= 24849.6
Pop. size in 2011-14= 2441.2
Change = -90.2%
Population size
50000
40000
30000
20000
10000
0
1975
1980
1985
1990
1995
2000
2005
2010
2015
Year and month

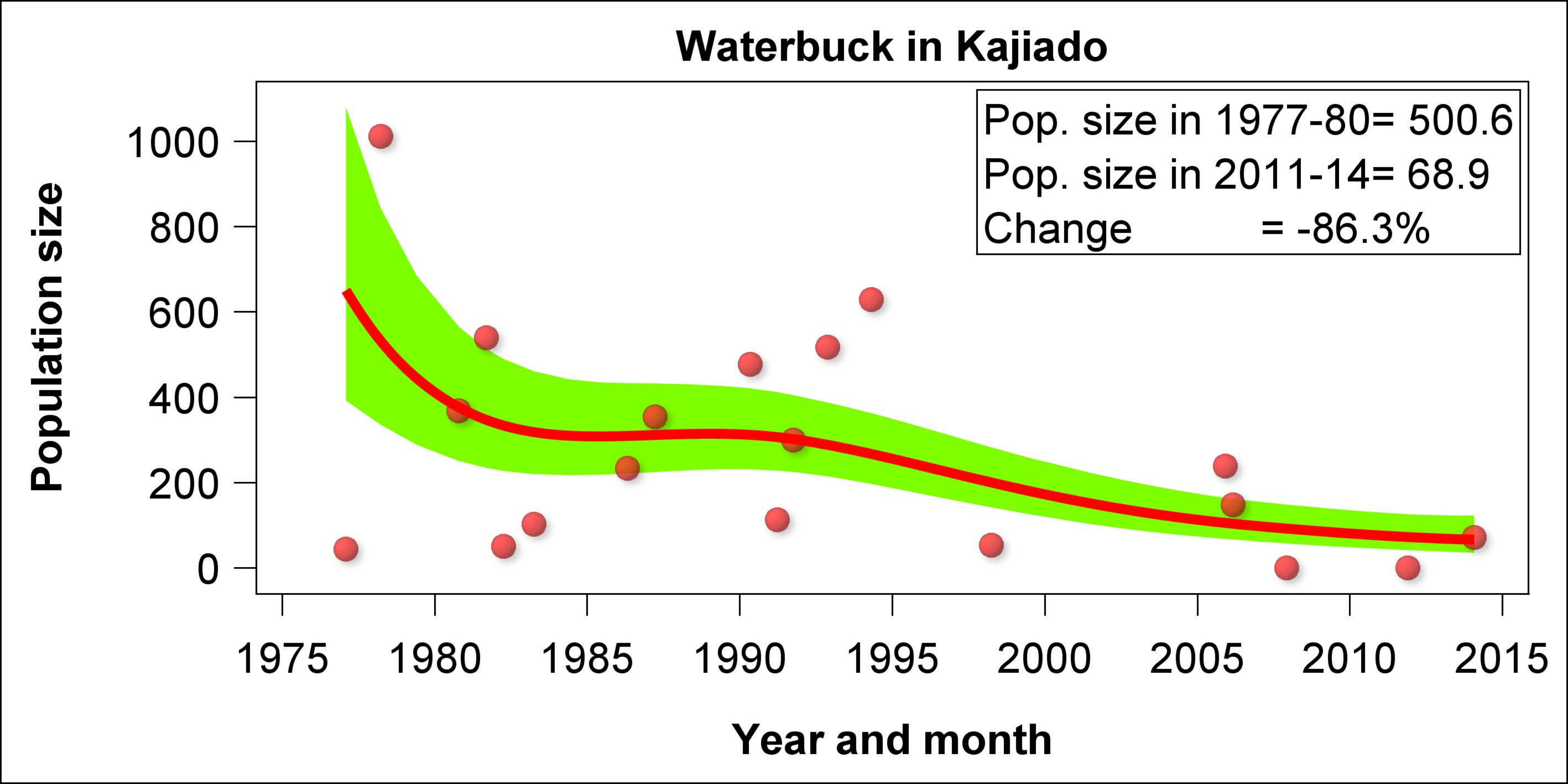
Waterbuck in Kajiado
Pop. size in 1977-80= 500.6
Pop. size in 2011-14= 68.9
Change = -86.3%
Population size
0
200
400
600
800
1000
1975
1980
1985
1990
1995
2000
2005
2010
2015
Year and month
