# Supplementary material for: Extreme Wildlife Declines and Concurrent Increase in Livestock Numbers in Kenya: What Are the Causes?
Source: PLoS One. 2016 Sep 27;11(9):e0163249. doi: 10.1371/journal.pone.0163249 (PMC5039022; doi:10.1371/journal.pone.0163249)
Supplement: S4 Fig — The solid red line is the fitted trend curve and the shaded chartreuse band is the pointwise 95% confidence band. The estimated average population size in 1977–1980 and 2011–2015 and the percentage change in population size between the two periods are provided in the inset. (PDF) [file pone.0163249.s014.pdf]

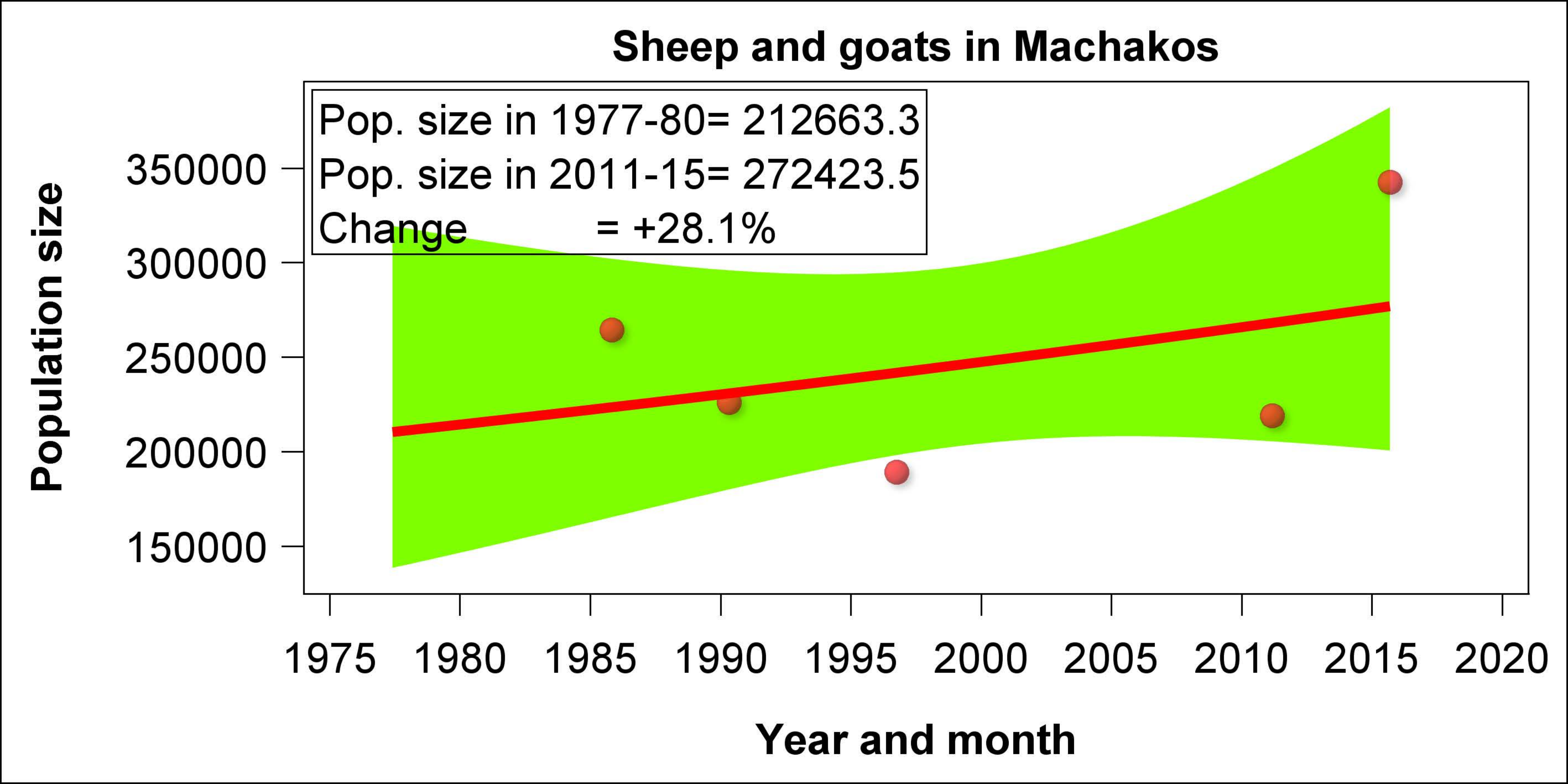
Sheep and goats in Machakos
Pop. size in 1977-80= 212663.3
Pop. size in 2011-15= 272423.5
Change = +28.1%
Population size
350000
300000
250000
200000
150000
1975
1980
1985
1990
1995
2000
2005
2010
2015
2020
Year and month

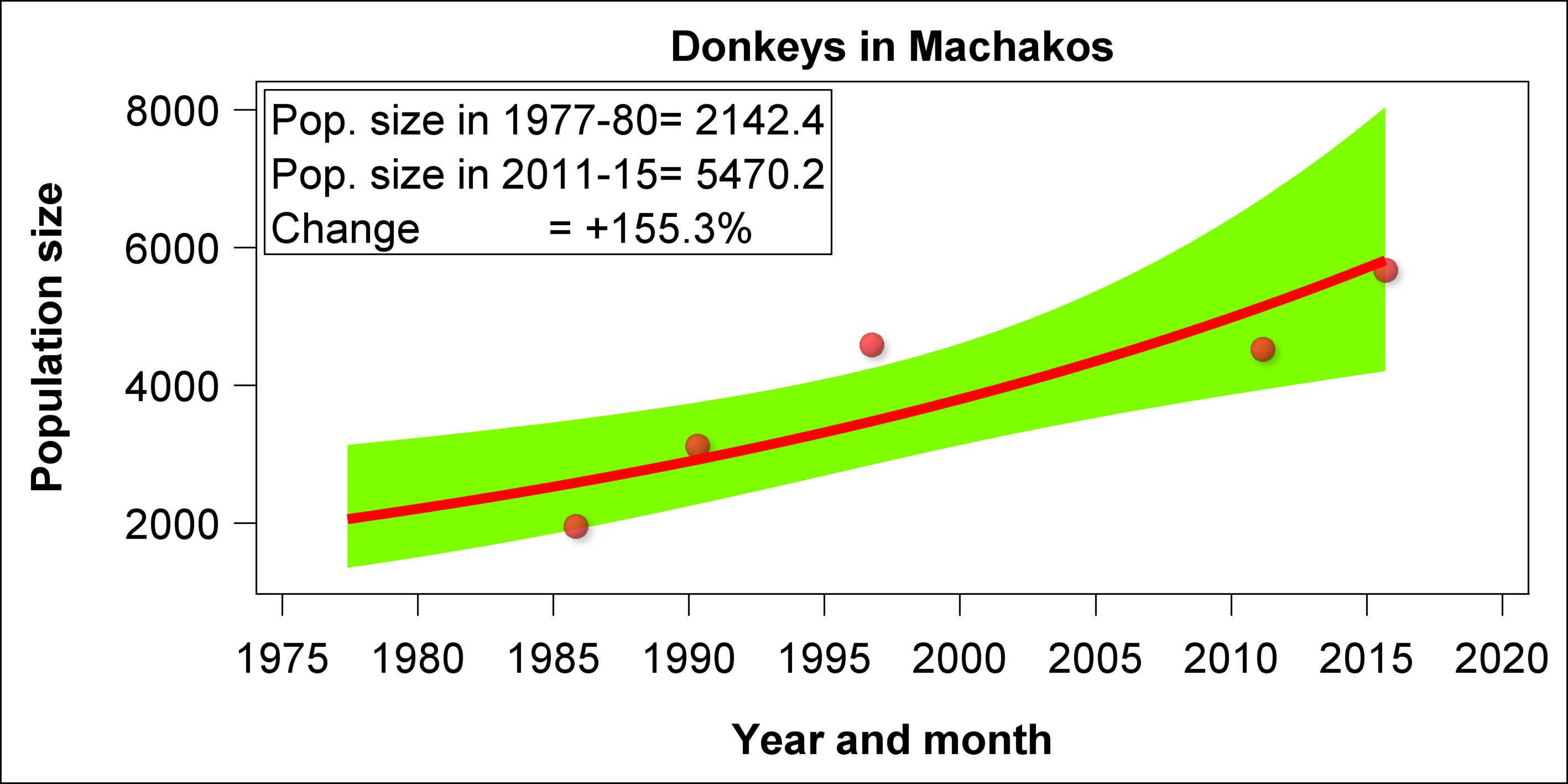
Donkeys in Machakos
Pop. size in 1977-80= 2142.4
Pop. size in 2011-15= 5470.2
Change = +155.3%
8000
6000
4000
2000
Population size
1975
1980
1985
1990
1995
2000
2005
2010
2015
2020
Year and month

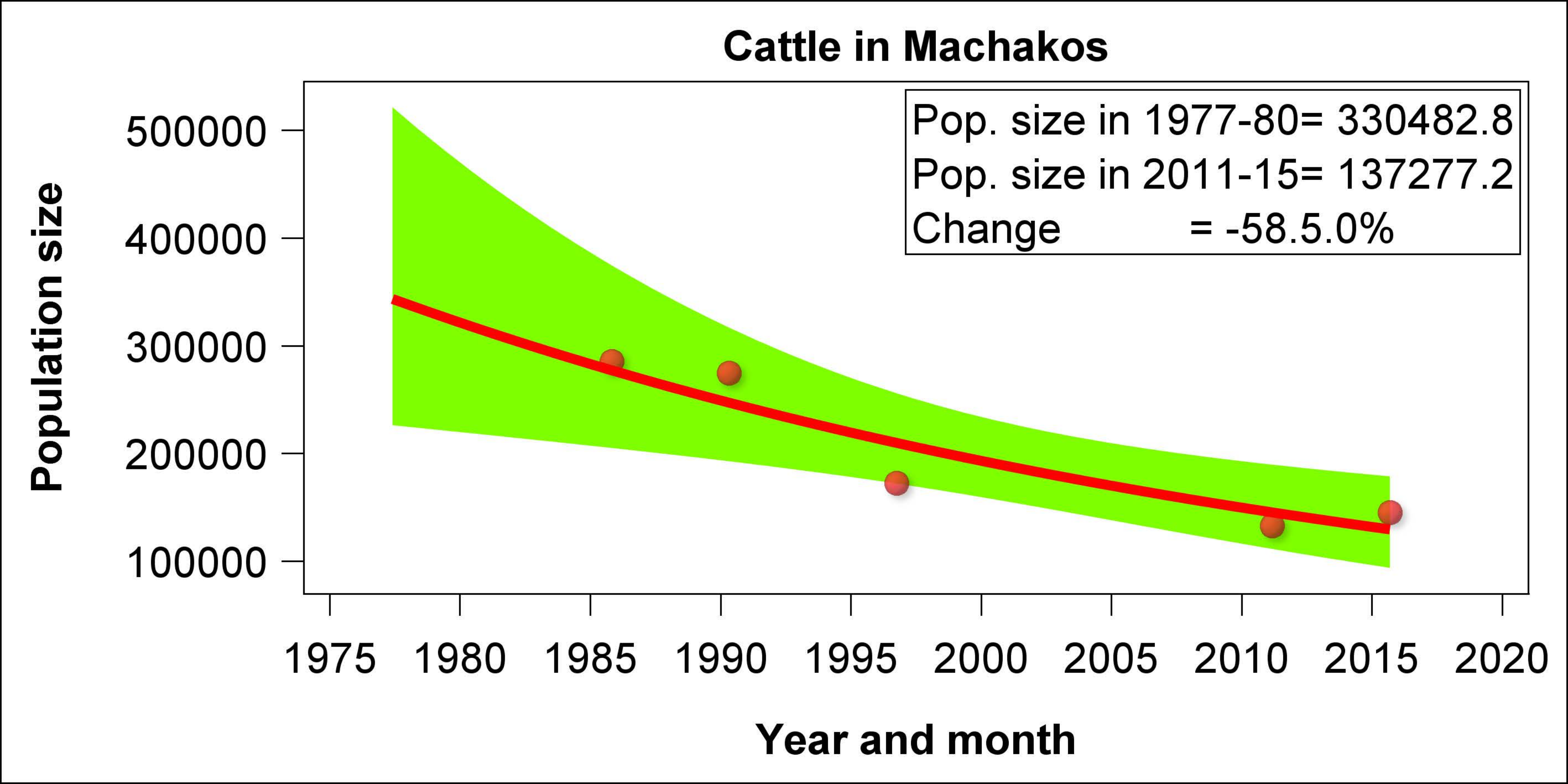
Cattle in Machakos
Pop. size in 1977-80= 330482.8
Pop. size in 2011-15= 137277.2
Change = -58.5.0%
500000
400000
300000
200000
100000
Population size
1975
1980
1985
1990
1995
2000
2005
2010
2015
2020
Year and month

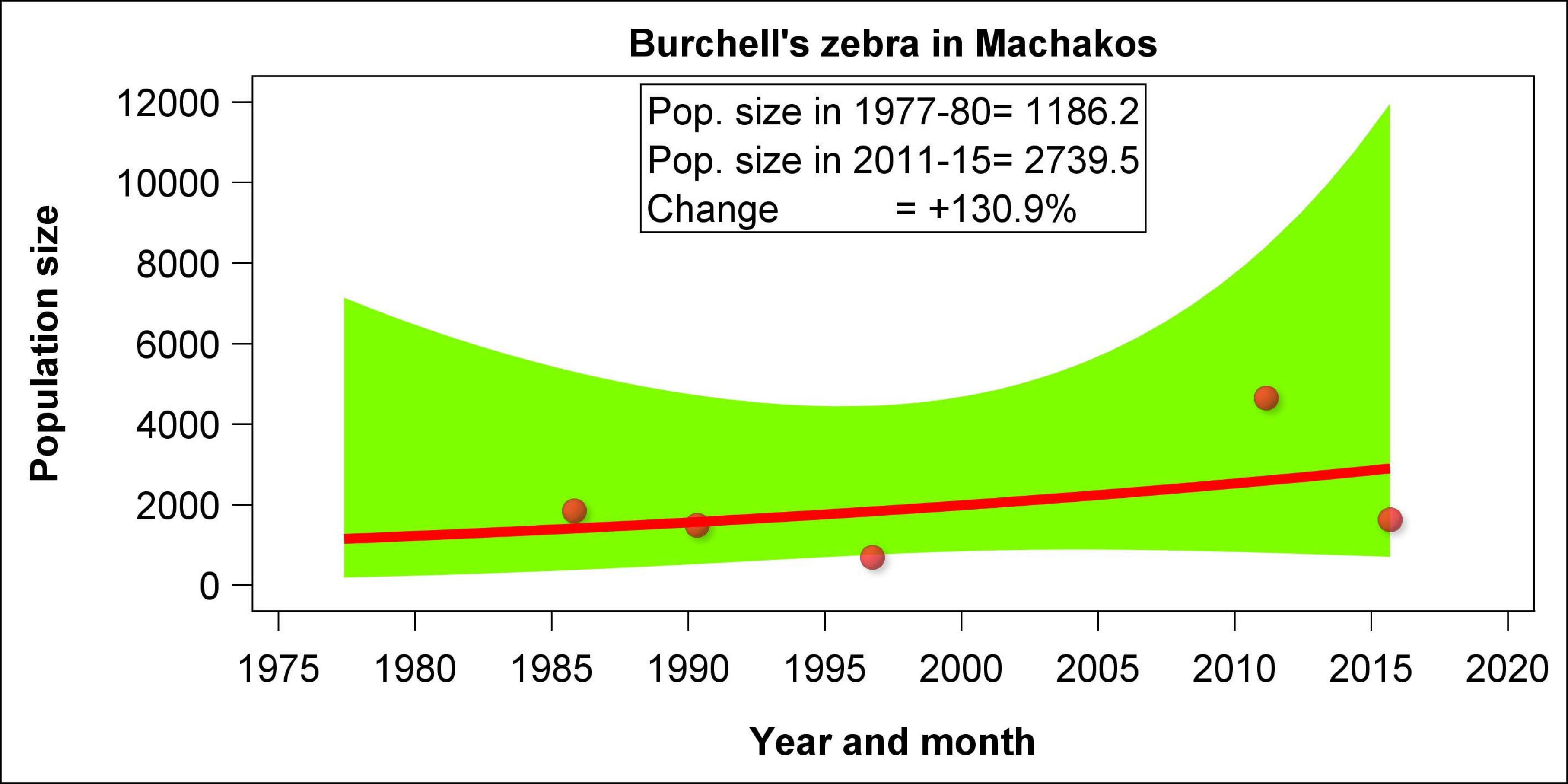

Burchell's zebra in Machakos
Pop. size in 1977-80= 1186.2
Pop. size in 2011-15= 2739.5
Change = +130.9%
Population size
12000
10000
8000
6000
4000
2000
0
1975
1980
1985
1990
1995
2000
2005
2010
2015
2020
Year and month

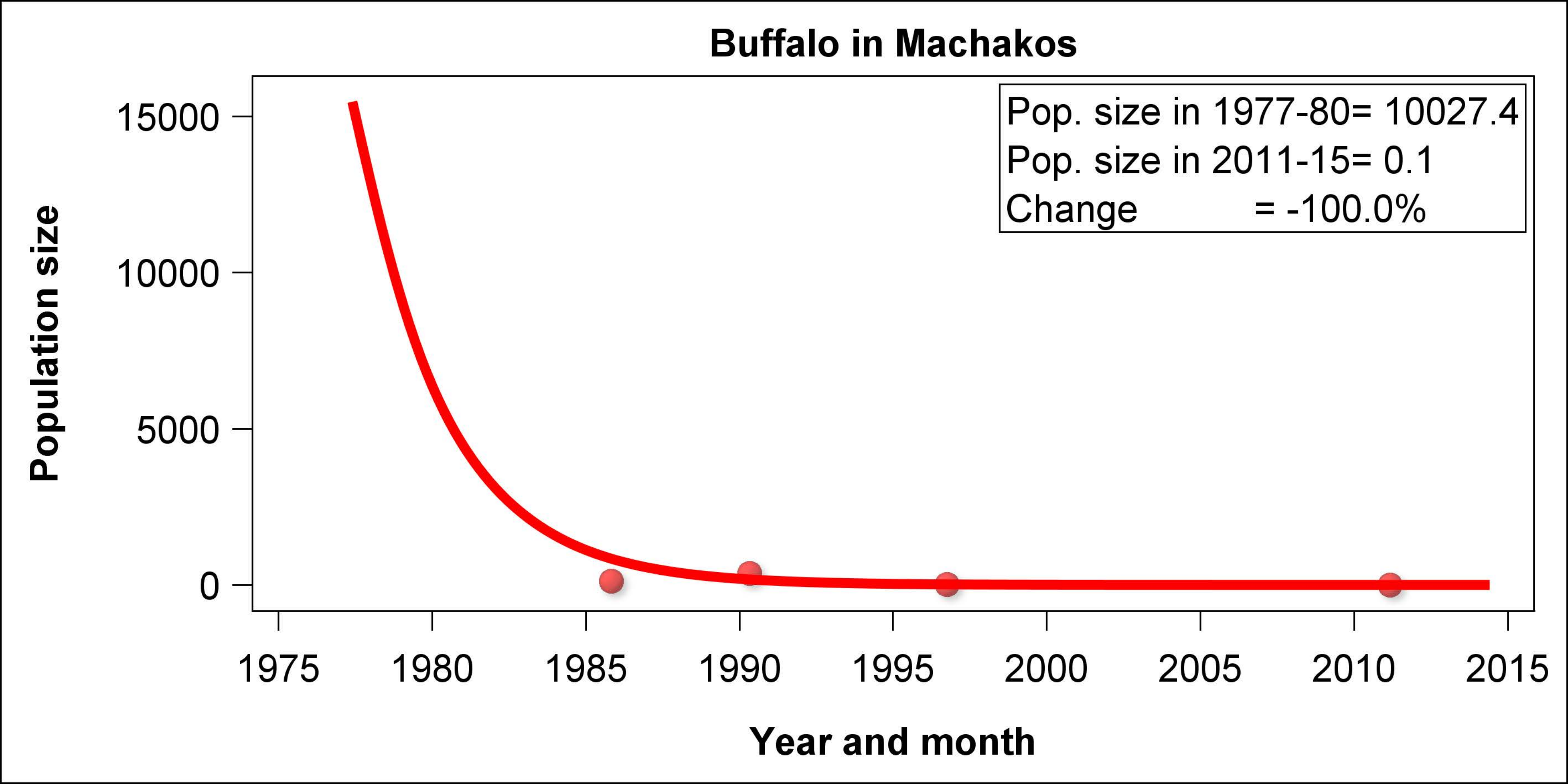
Buffalo in Machakos
Pop. size in 1977-80= 10027.4
Pop. size in 2011-15= 0.1
Change = -100.0%
Population size
15000
10000
5000
0
1975
1980
1985
1990
1995
2000
2005
2010
2015
Year and month

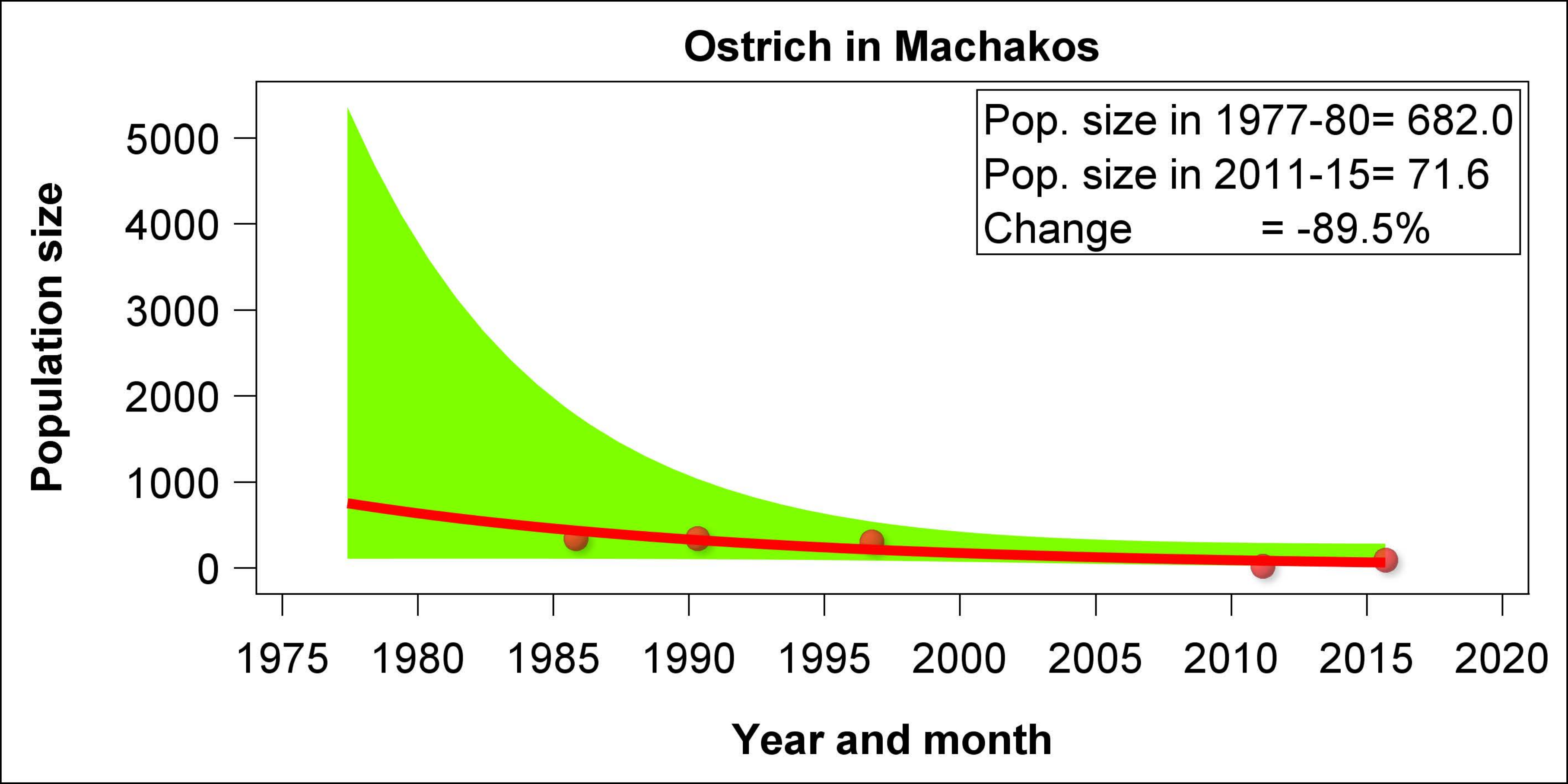

Ostrich in Machakos
Pop. size in 1977-80= 682.0
Pop. size in 2011-15= 71.6
Change = -89.5%
Population size
5000
4000
3000
2000
1000
0
1975
1980
1985
1990
1995
2000
2005
2010
2015
2020
Year and month

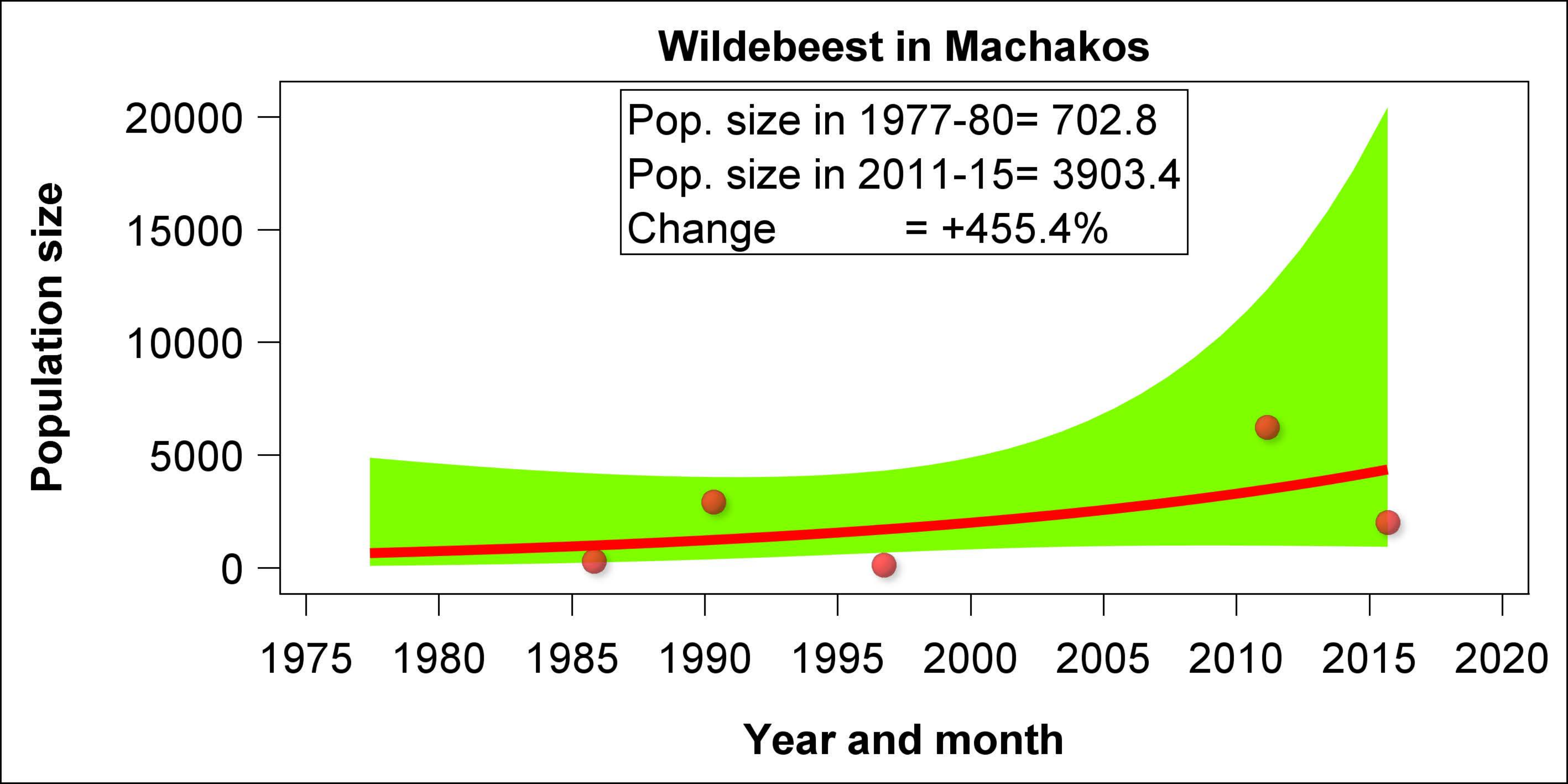

Wildebeest in Machakos
Pop. size in 1977-80= 702.8
Pop. size in 2011-15= 3903.4
Change = +455.4%
Population size
20000
15000
10000
5000
0
1975
1980
1985
1990
1995
2000
2005
2010
2015
2020
Year and month

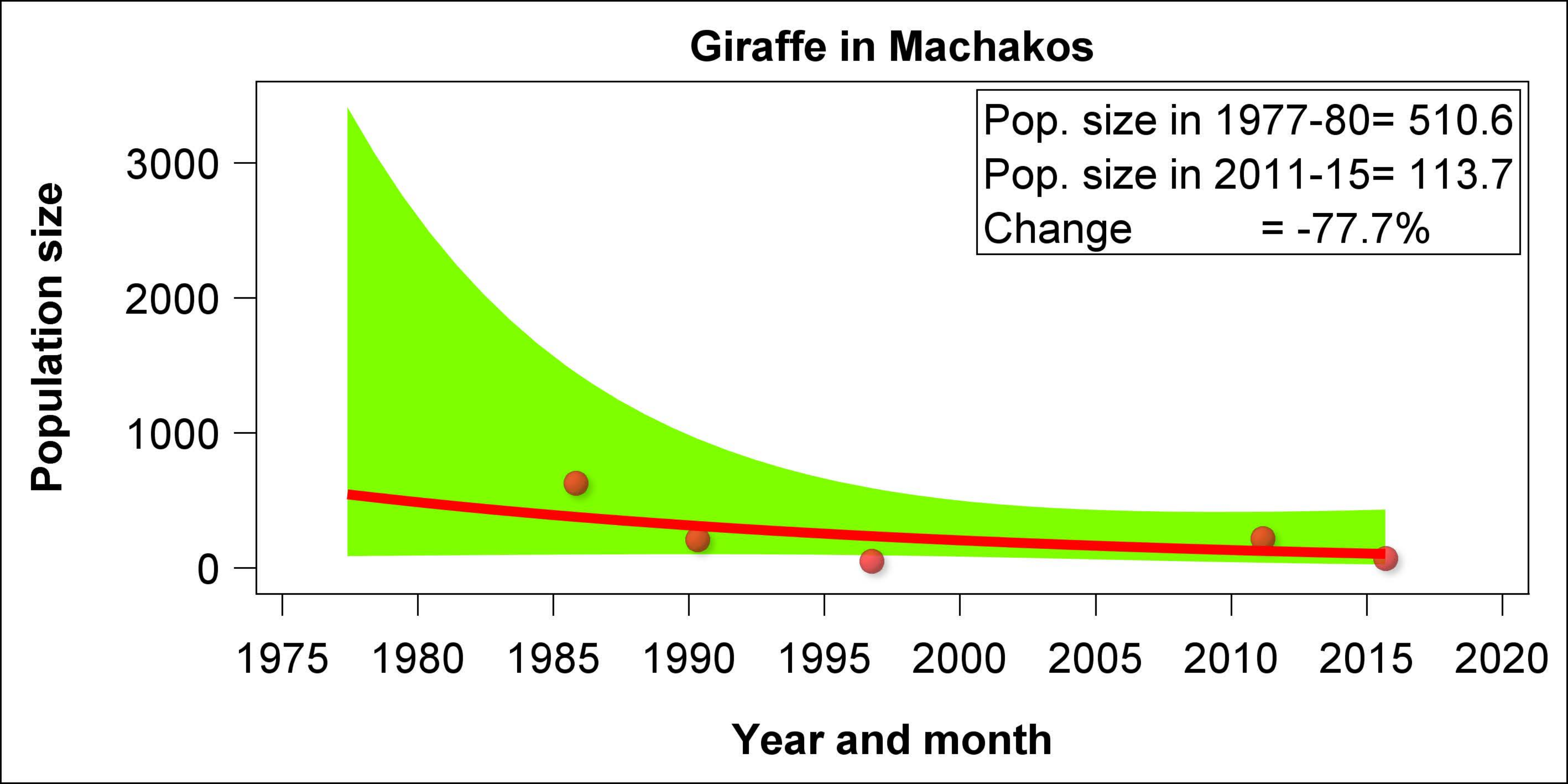
Giraffe in Machakos
Pop. size in 1977-80= 510.6
Pop. size in 2011-15= 113.7
Change = -77.7%
Population size
3000
2000
1000
0
1975
1980
1985
1990
1995
2000
2005
2010
2015
2020
Year and month

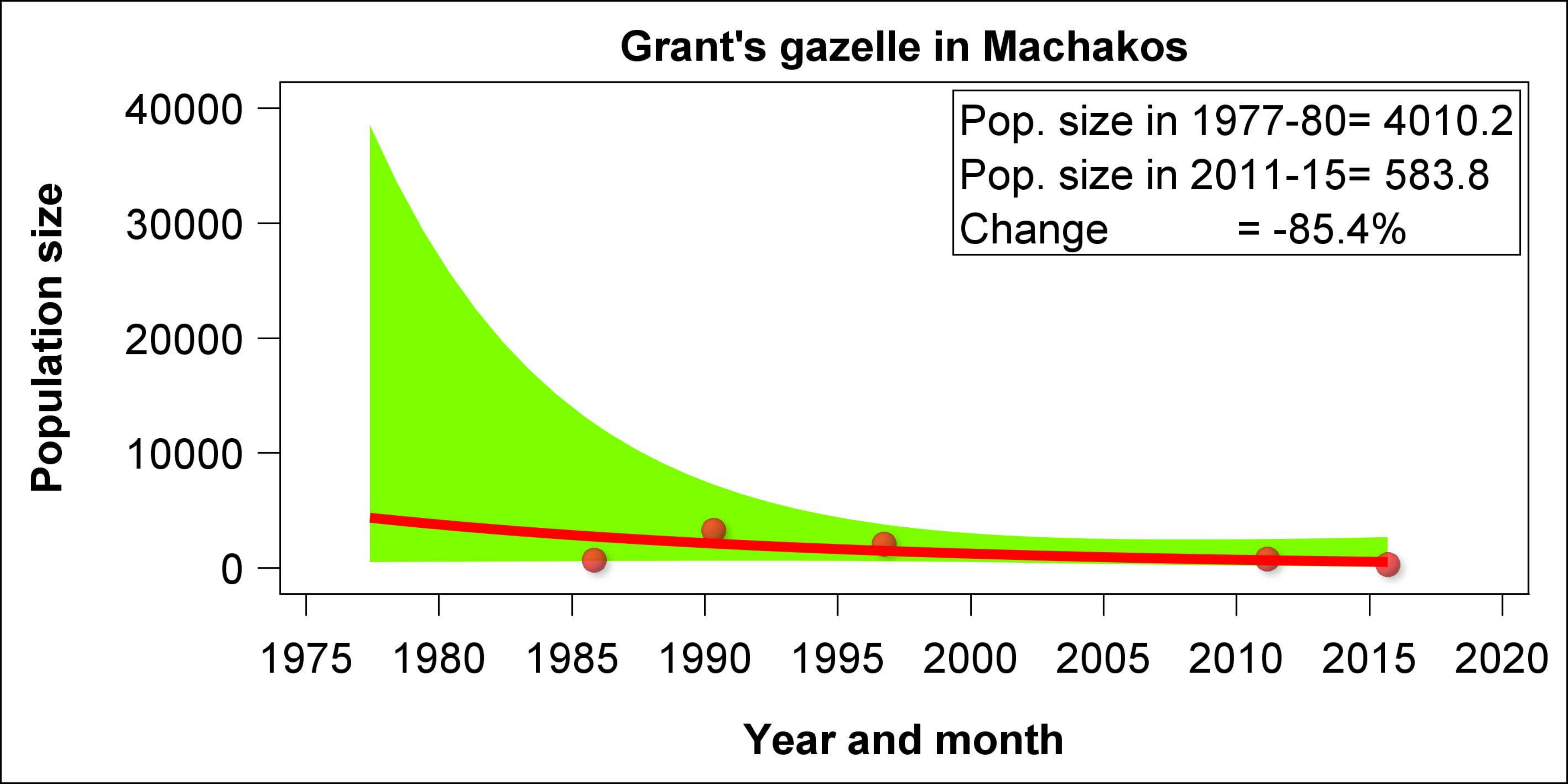
Grant's gazelle in Machakos
Pop. size in 1977-80= 4010.2
Pop. size in 2011-15= 583.8
Change = -85.4%
Population size
40000
30000
20000
10000
0
1975
1980
1985
1990
1995
2000
2005
2010
2015
2020
Year and month

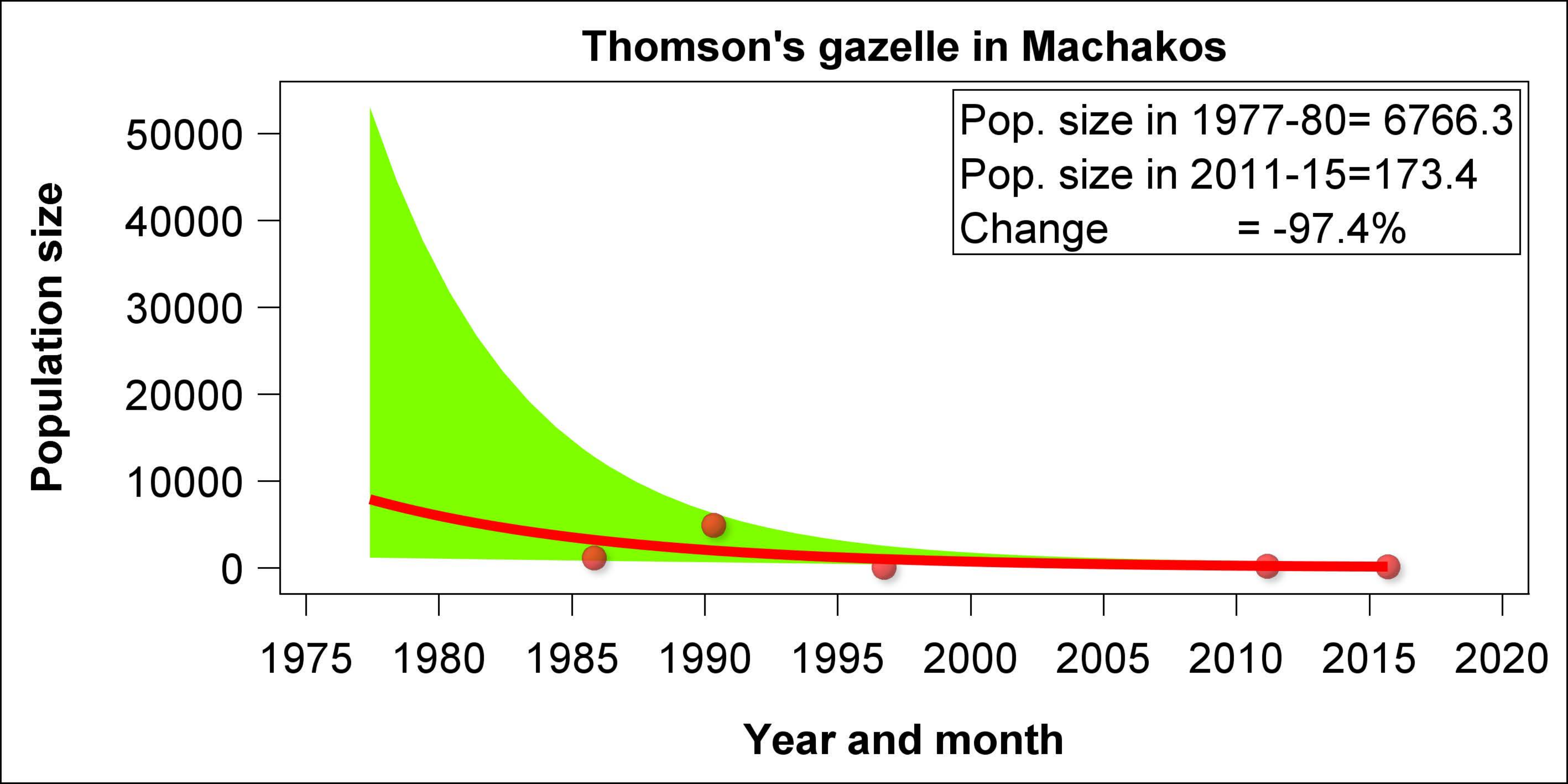

Thomson's gazelle in Machakos
Pop. size in 1977-80= 6766.3
Pop. size in 2011-15=173.4
Change = -97.4%
Population size
50000
40000
30000
20000
10000
0
1975
1980
1985
1990
1995
2000
2005
2010
2015
2020
Year and month

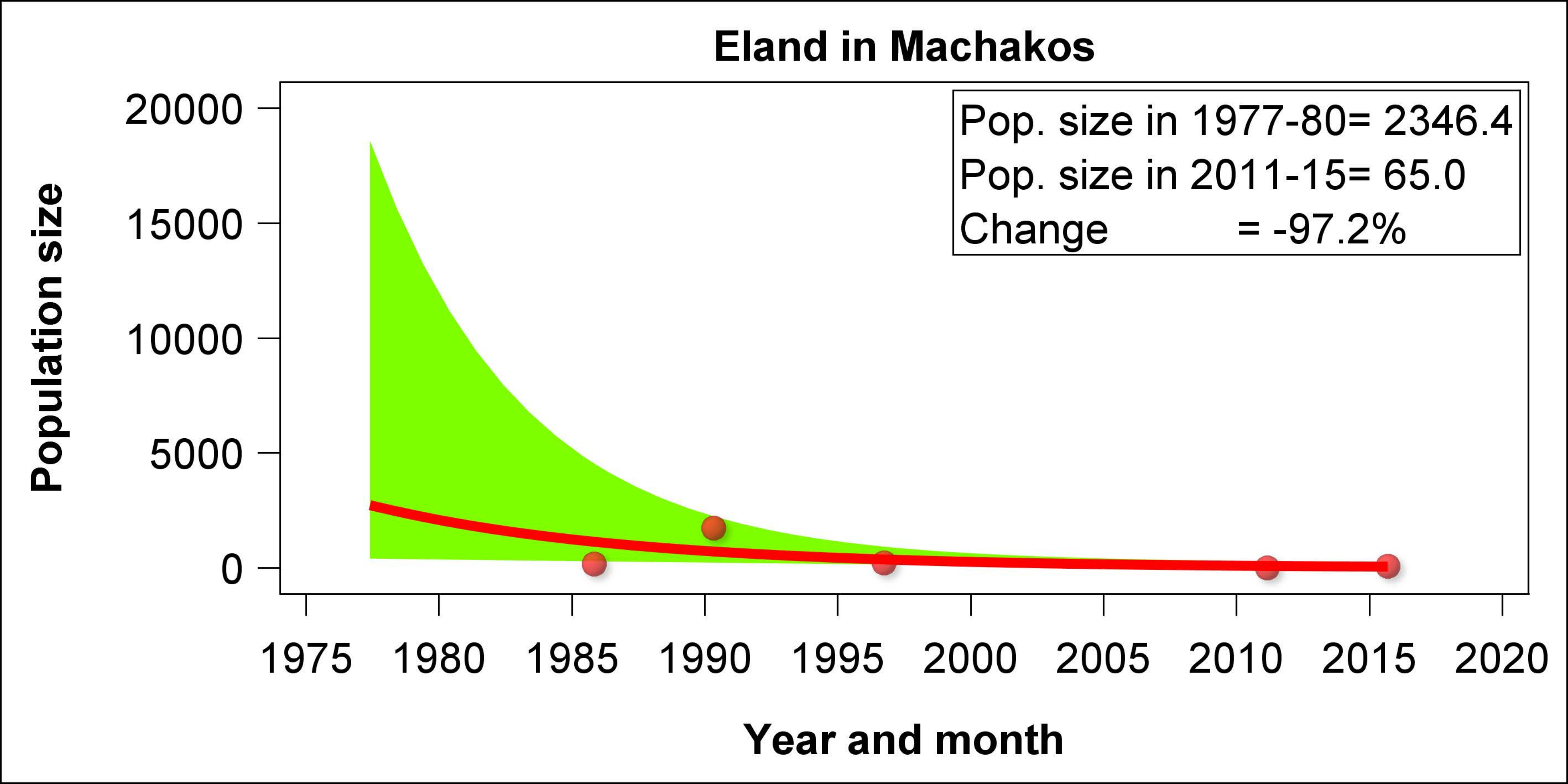

Eland in Machakos
Pop. size in 1977-80= 2346.4
Pop. size in 2011-15= 65.0
Change = -97.2%
Population size
20000
15000
10000
5000
0
1975
1980
1985
1990
1995
2000
2005
2010
2015
2020
Year and month

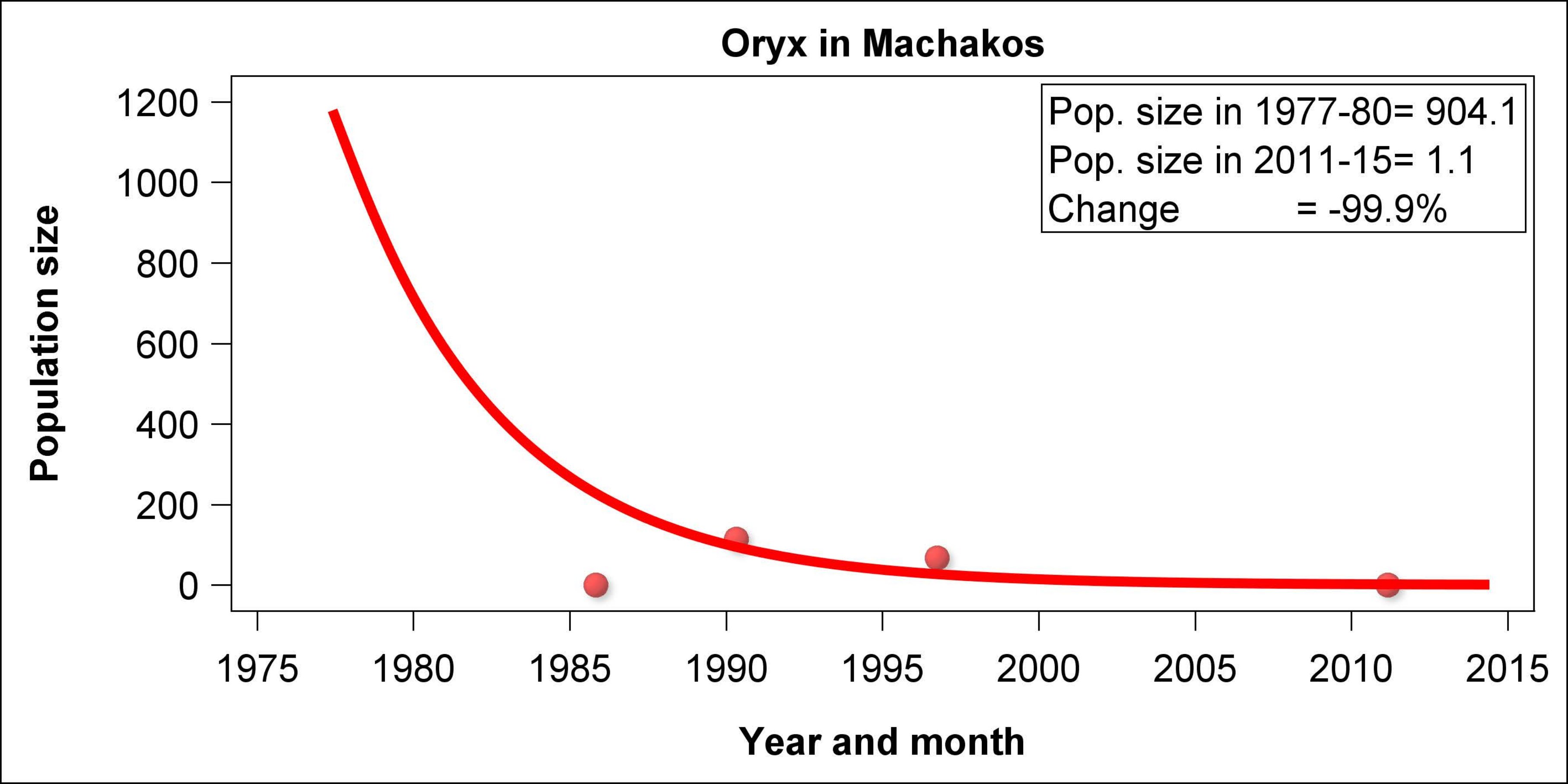

Oryx in Machakos
Pop. size in 1977-80= 904.1
Pop. size in 2011-15= 1.1
Change = -99.9%
Population size
1200
1000
800
600
400
200
0
1975
1980
1985
1990
1995
2000
2005
2010
2015
Year and month

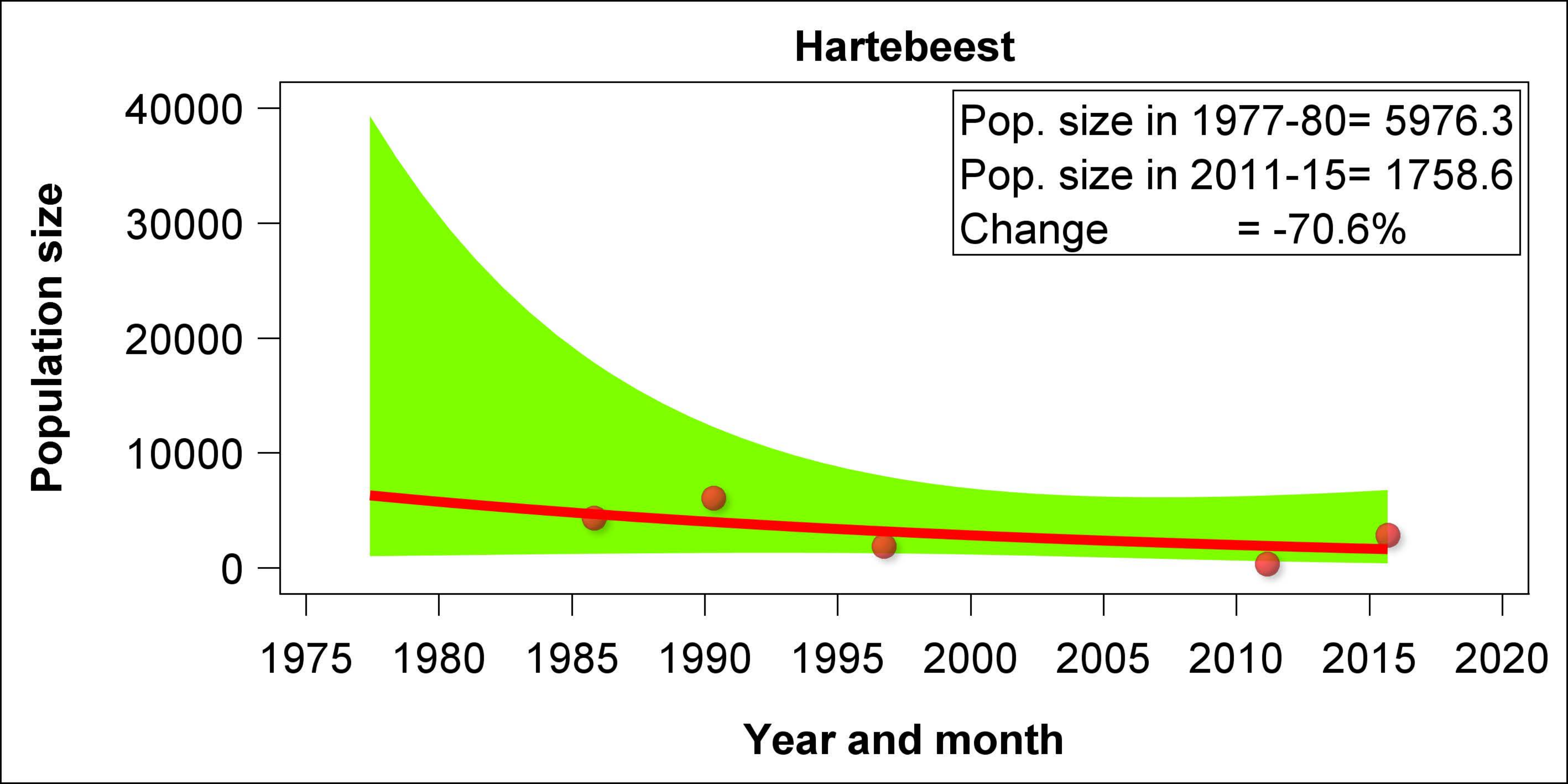
Hartebeest
Pop. size in 1977-80= 5976.3
Pop. size in 2011-15= 1758.6
Change = -70.6%
Population size
40000
30000
20000
10000
0
1975
1980
1985
1990
1995
2000
2005
2010
2015
2020
Year and month

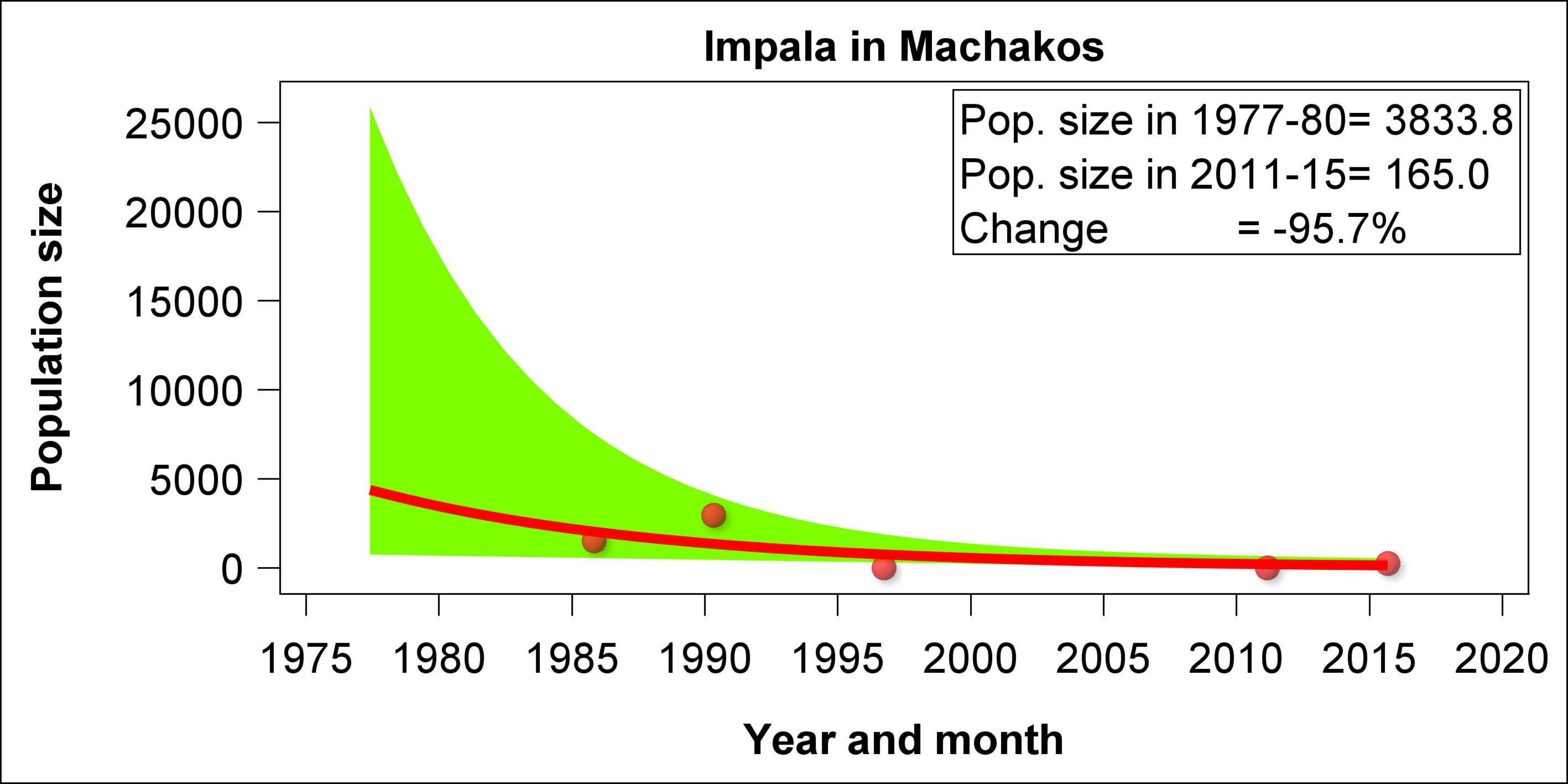
Impala in Machakos
Pop. size in 1977-80= 3833.8
Pop. size in 2011-15= 165.0
Change = -95.7%
25000
20000
15000
10000
5000
0
Population size
1975
1980
1985
1990
1995
2000
2005
2010
2015
2020
Year and month
