# Supplementary material for: Extreme Wildlife Declines and Concurrent Increase in Livestock Numbers in Kenya: What Are the Causes?
Source: PLoS One. 2016 Sep 27;11(9):e0163249. doi: 10.1371/journal.pone.0163249 (PMC5039022; doi:10.1371/journal.pone.0163249)
Supplement: S5 Fig — The solid red line is the fitted trend curve and the shaded chartreuse band is the pointwise 95% confidence band. The estimated average population size in 1977–1980 and 2011–2015 and the percentage change in population size between the two periods are provided in the inset. (PDF) [file pone.0163249.s015.pdf]

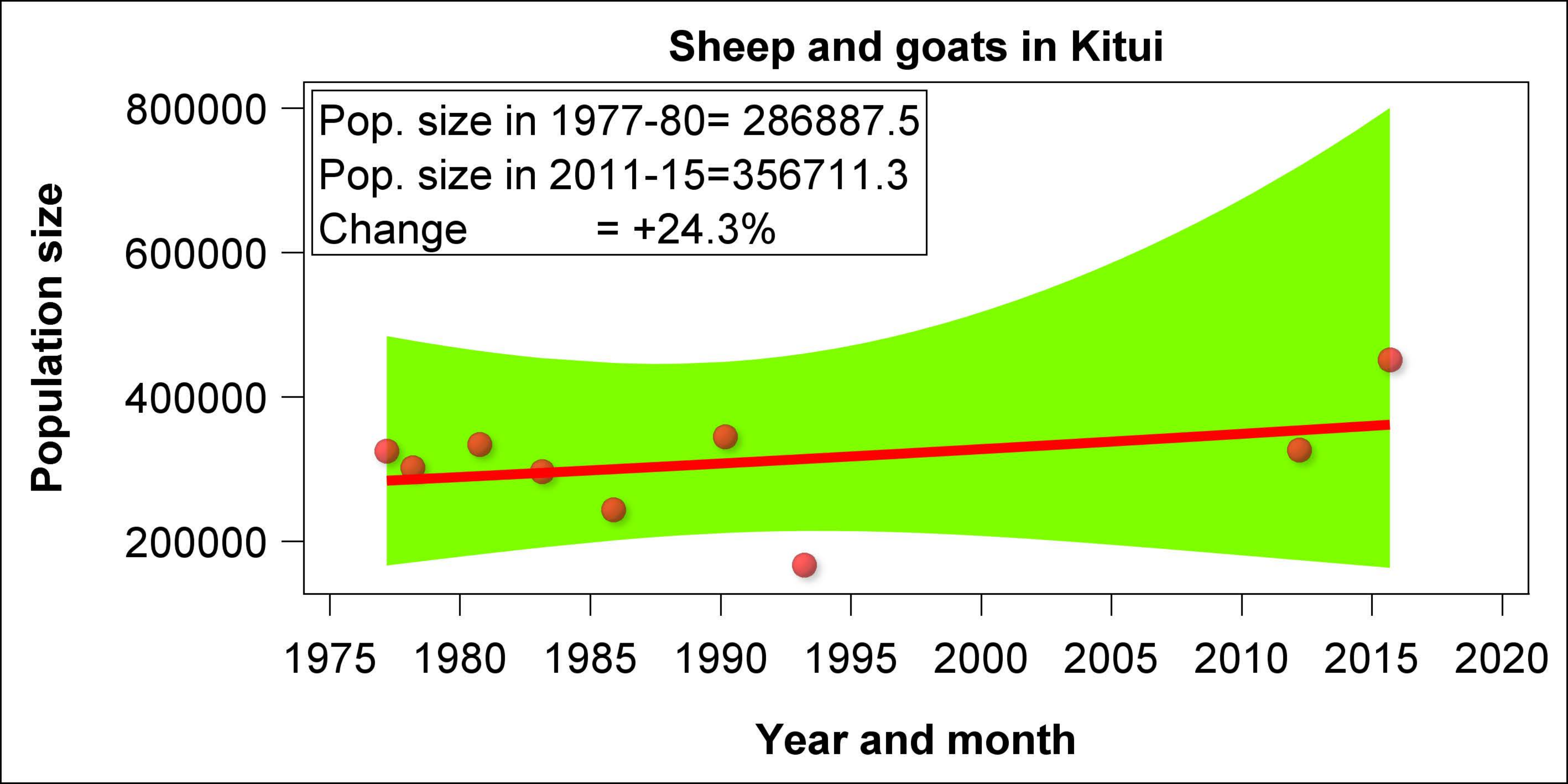

Sheep and goats in Kitui
Pop. size in 1977-80= 286887.5
Pop. size in 2011-15=356711.3
Change = +24.3%
800000
600000
400000
200000
Population size
1975
1980
1985
1990
1995
2000
2005
2010
2015
2020
Year and month

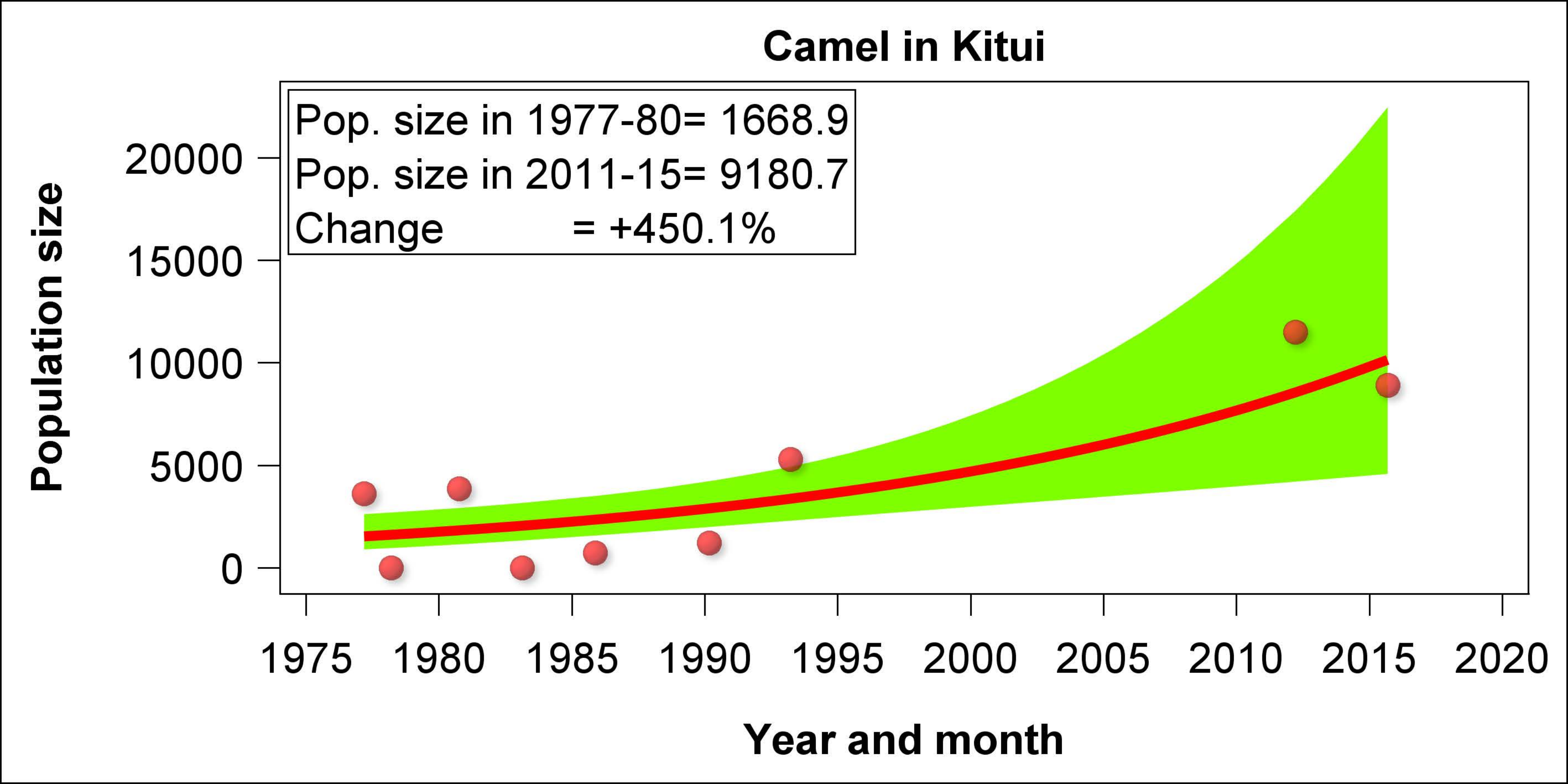
Camel in Kitui
Pop. size in 1977-80= 1668.9
Pop. size in 2011-15= 9180.7
Change = +450.1%
Population size
20000
15000
10000
5000
0
1975
1980
1985
1990
1995
2000
2005
2010
2015
2020
Year and month

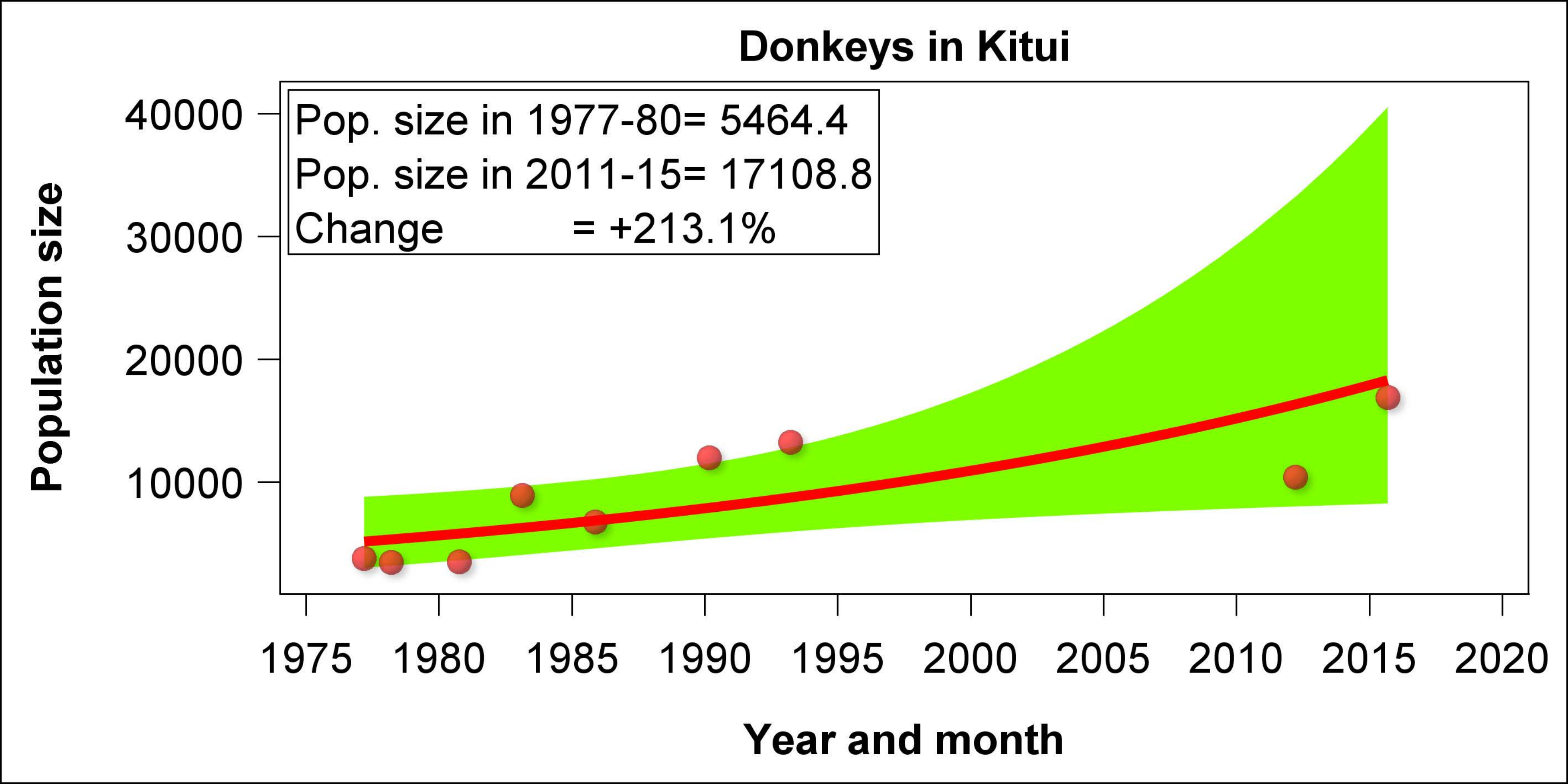

Donkeys in Kitui
Pop. size in 1977-80= 5464.4
Pop. size in 2011-15= 17108.8
Change = +213.1%
40000
30000
20000
10000
Population size
1975
1980
1985
1990
1995
2000
2005
2010
2015
2020
Year and month

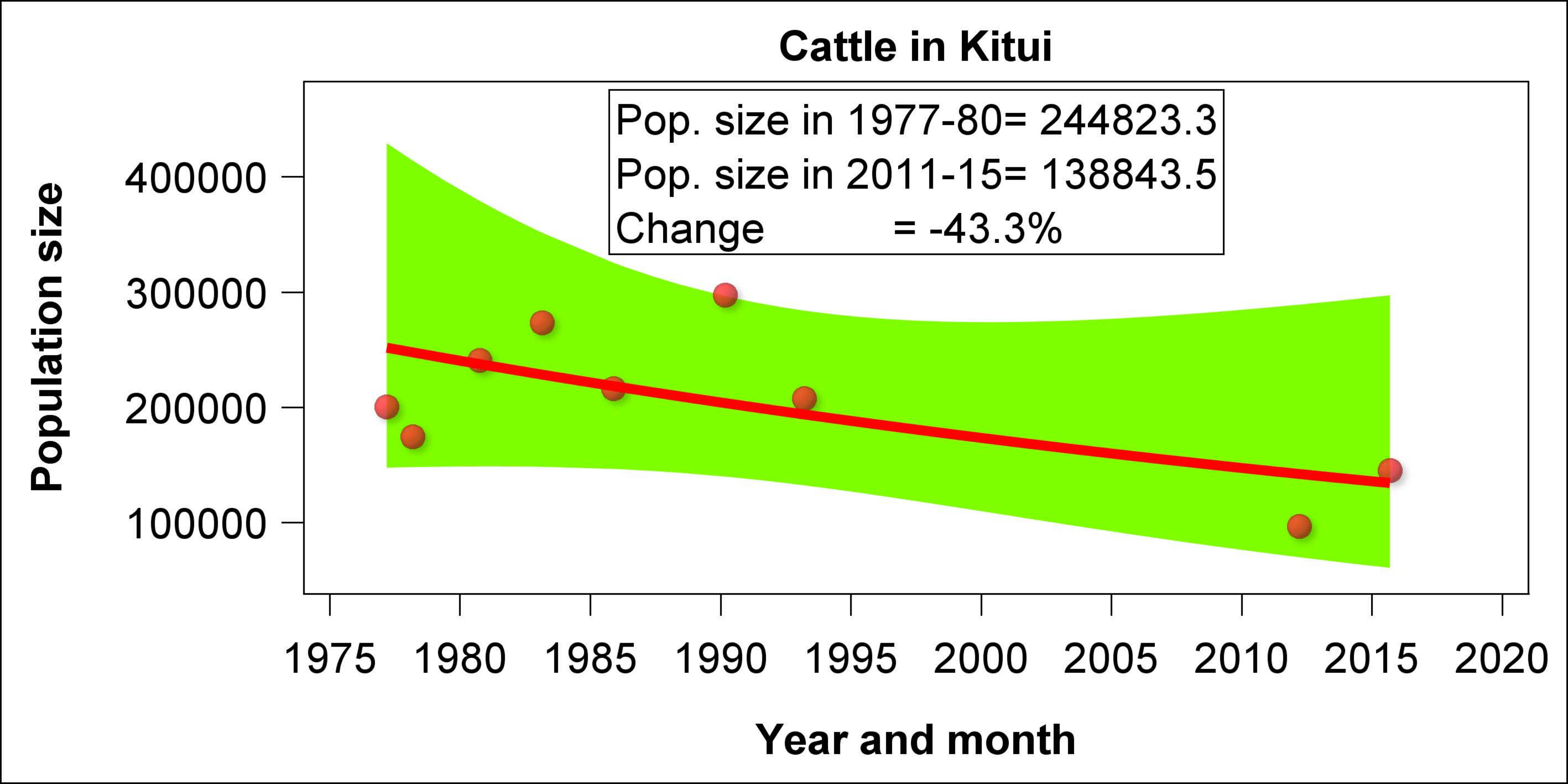

Cattle in Kitui
Pop. size in 1977-80= 244823.3
Pop. size in 2011-15= 138843.5
Change = -43.3%
Population size
400000
300000
200000
100000
1975
1980
1985
1990
1995
2000
2005
2010
2015
2020
Year and month

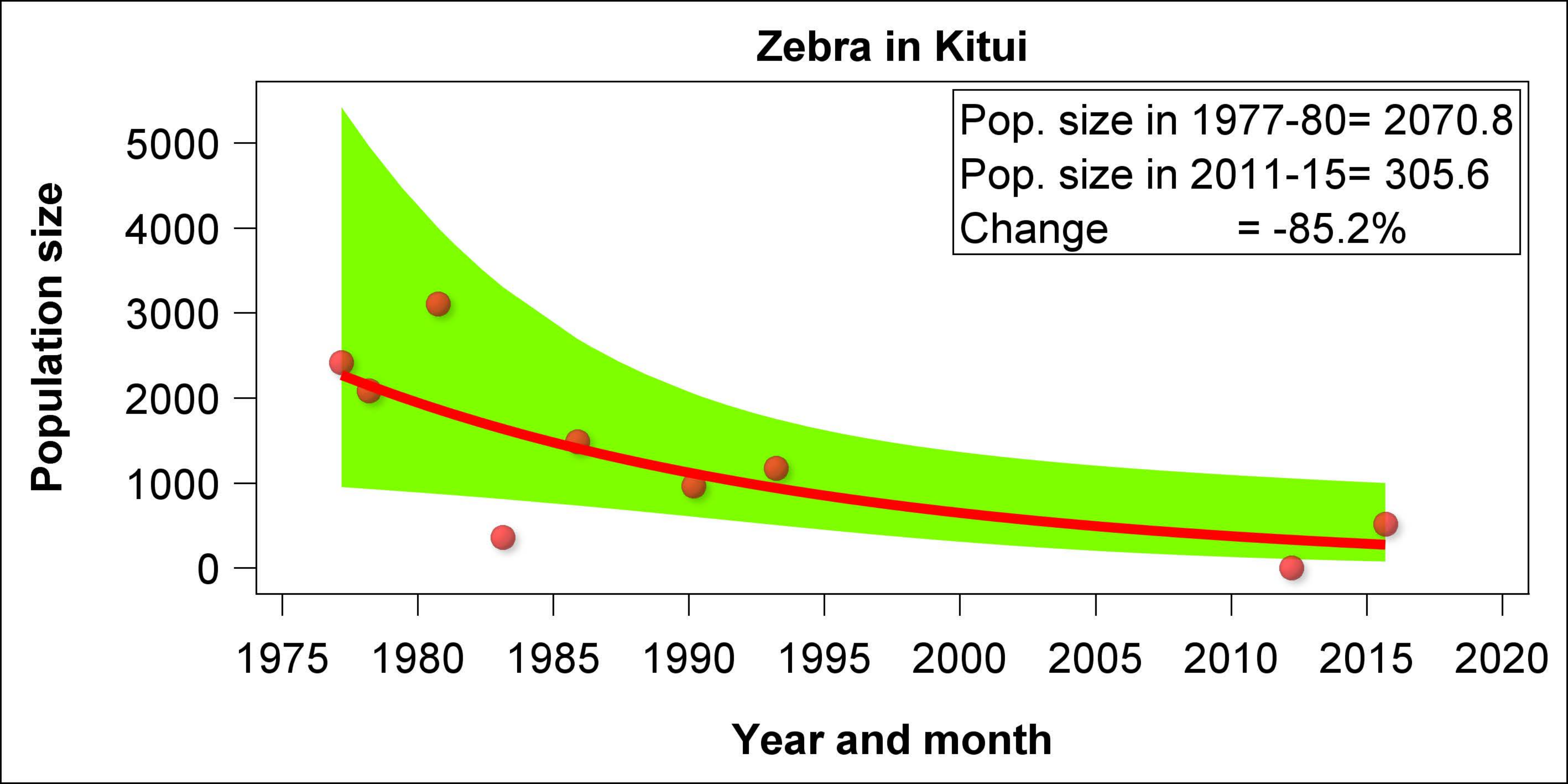
Zebra in Kitui
Pop. size in 1977-80= 2070.8
Pop. size in 2011-15= 305.6
Change = -85.2%
Population size
5000
4000
3000
2000
1000
0
1975
1980
1985
1990
1995
2000
2005
2010
2015
2020
Year and month

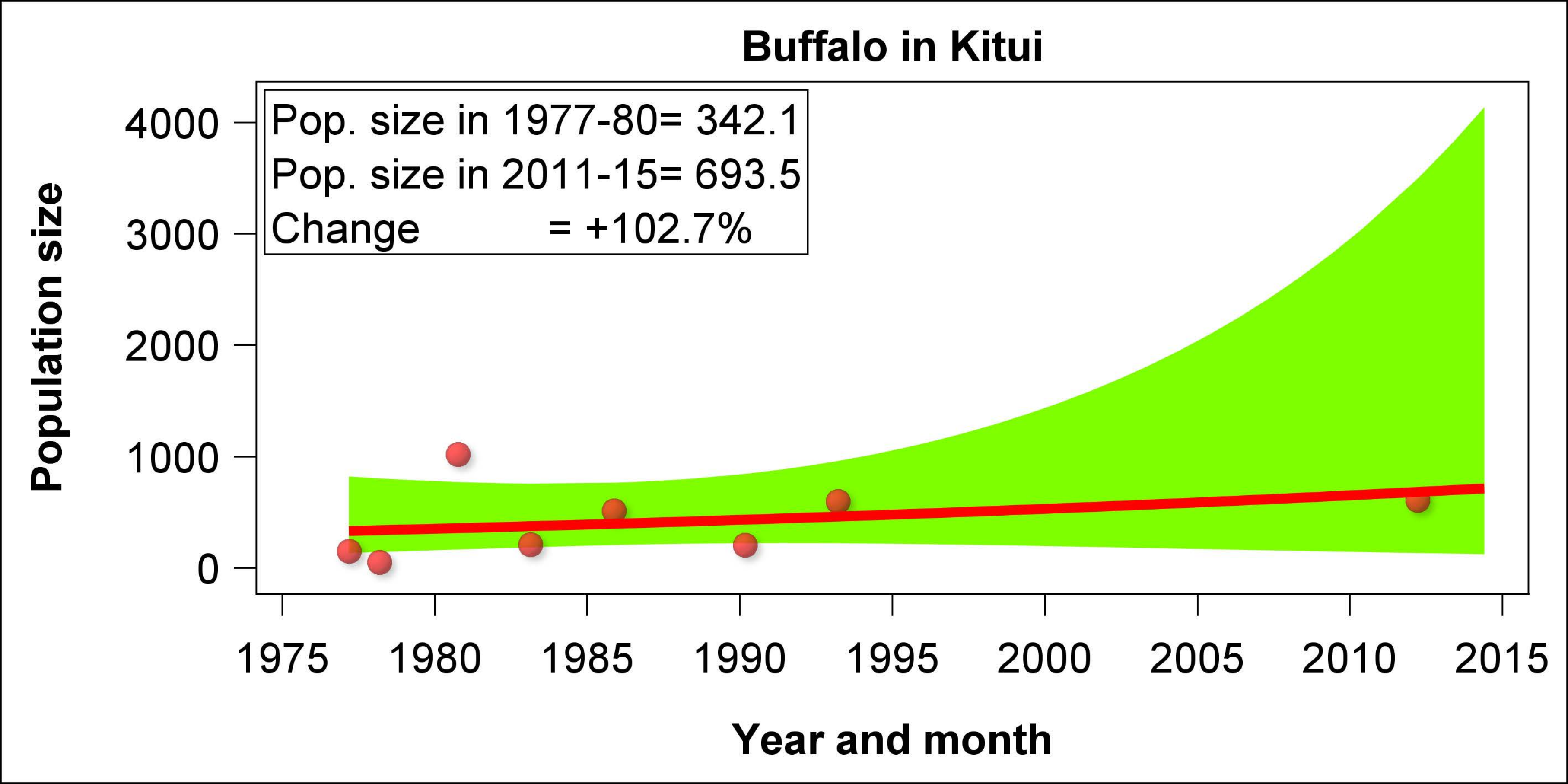

Buffalo in Kitui
Pop. size in 1977-80= 342.1
Pop. size in 2011-15= 693.5
Change = +102.7%
Population size
4000
3000
2000
1000
0
1975
1980
1985
1990
1995
2000
2005
2010
2015
Year and month

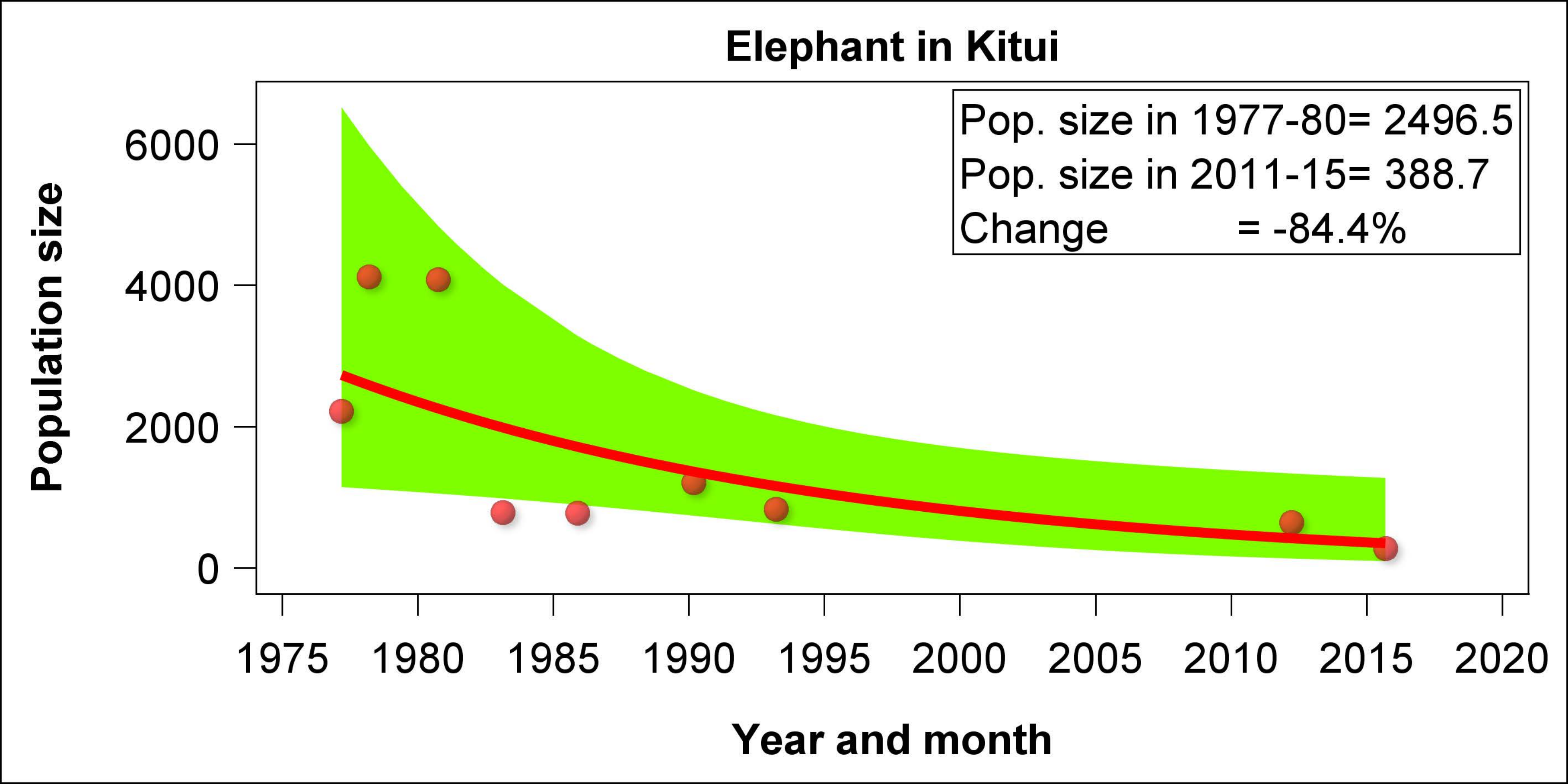

Elephant in Kitui
Pop. size in 1977-80= 2496.5
Pop. size in 2011-15= 388.7
Change = -84.4%
Population size
6000
4000
2000
0
1975
1980
1985
1990
1995
2000
2005
2010
2015
2020
Year and month

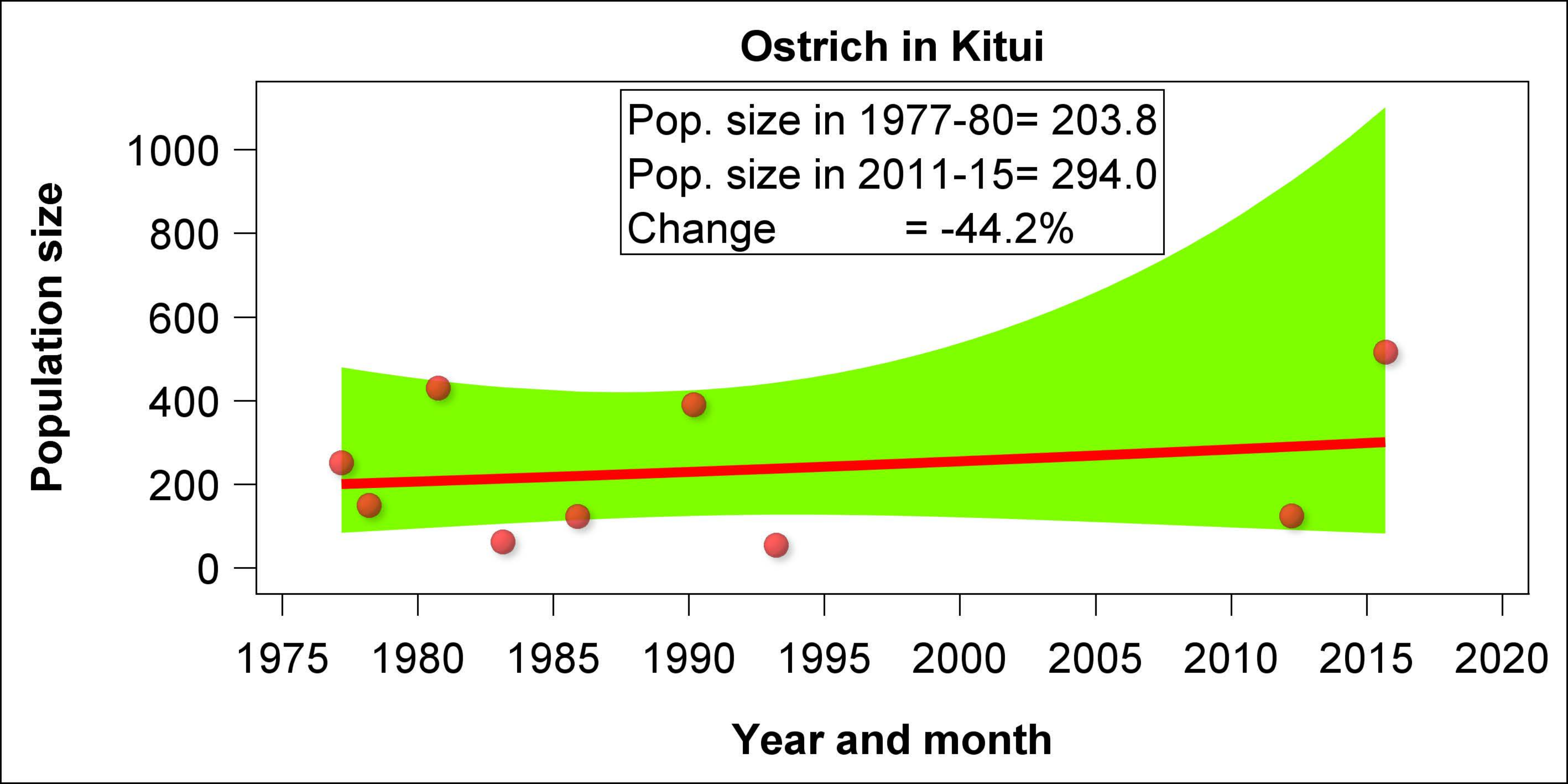
Ostrich in Kitui
Pop. size in 1977-80= 203.8
Pop. size in 2011-15= 294.0
Change = -44.2%
Population size
1000
800
600
400
200
0
1975
1980
1985
1990
1995
2000
2005
2010
2015
2020
Year and month

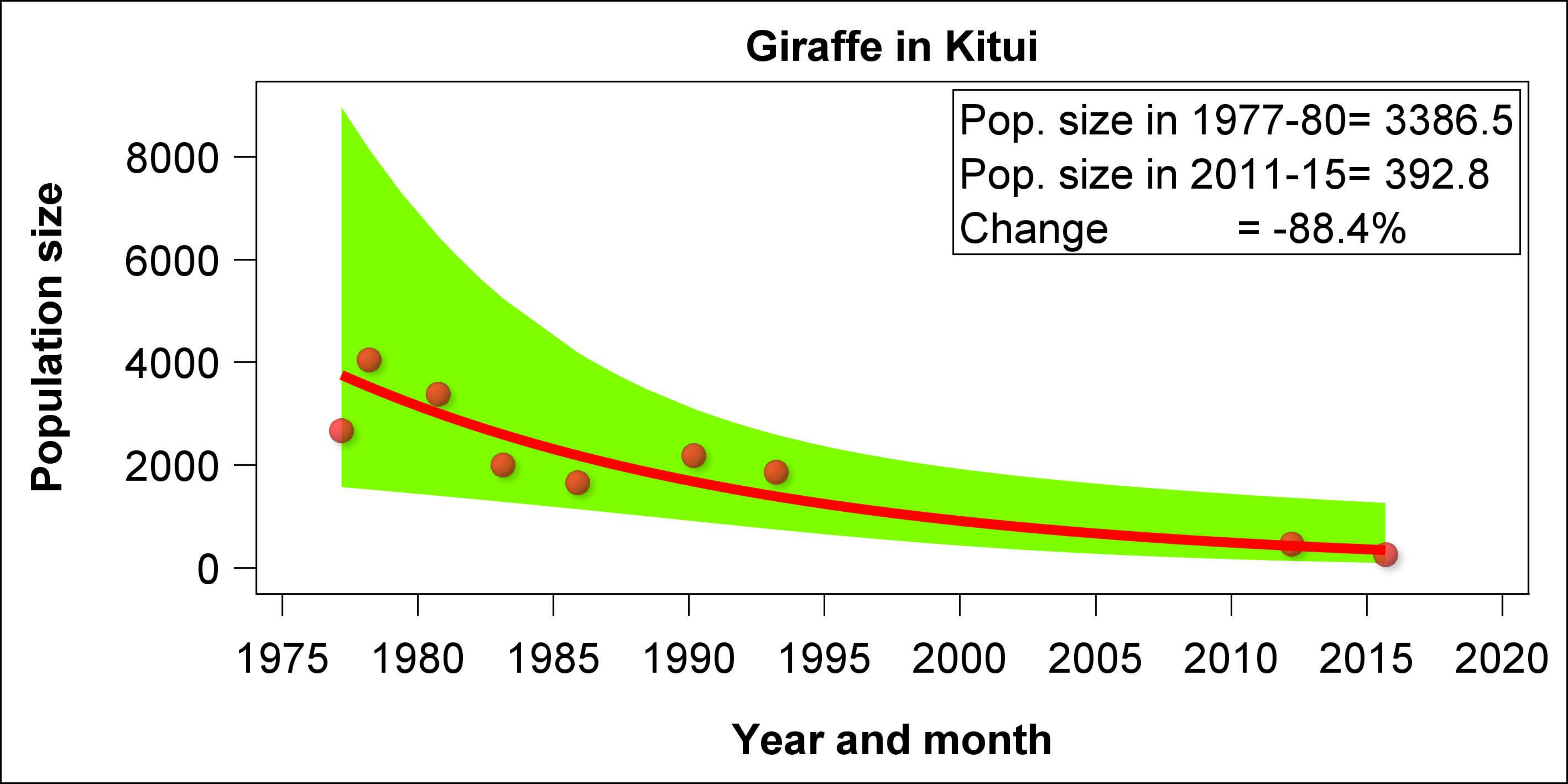

Giraffe in Kitui
Pop. size in 1977-80= 3386.5
Pop. size in 2011-15= 392.8
Change = -88.4%
Population size
8000
6000
4000
2000
0
1975
1980
1985
1990
1995
2000
2005
2010
2015
2020
Year and month

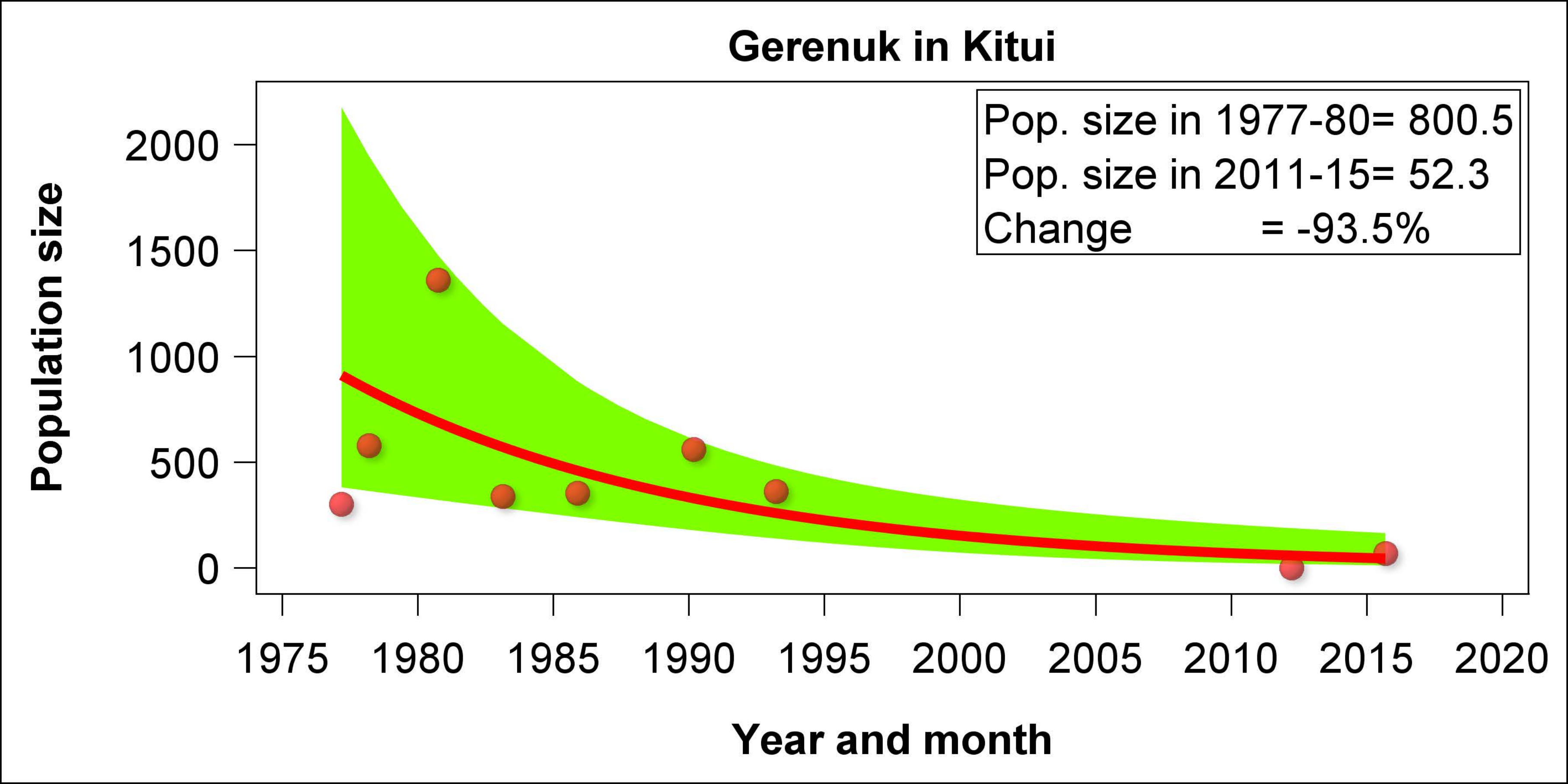
Gerenuk in Kitui
Pop. size in 1977-80= 800.5
Pop. size in 2011-15= 52.3
Change = -93.5%
Population size
0
500
1000
1500
2000
1975
1980
1985
1990
1995
2000
2005
2010
2015
2020
Year and month

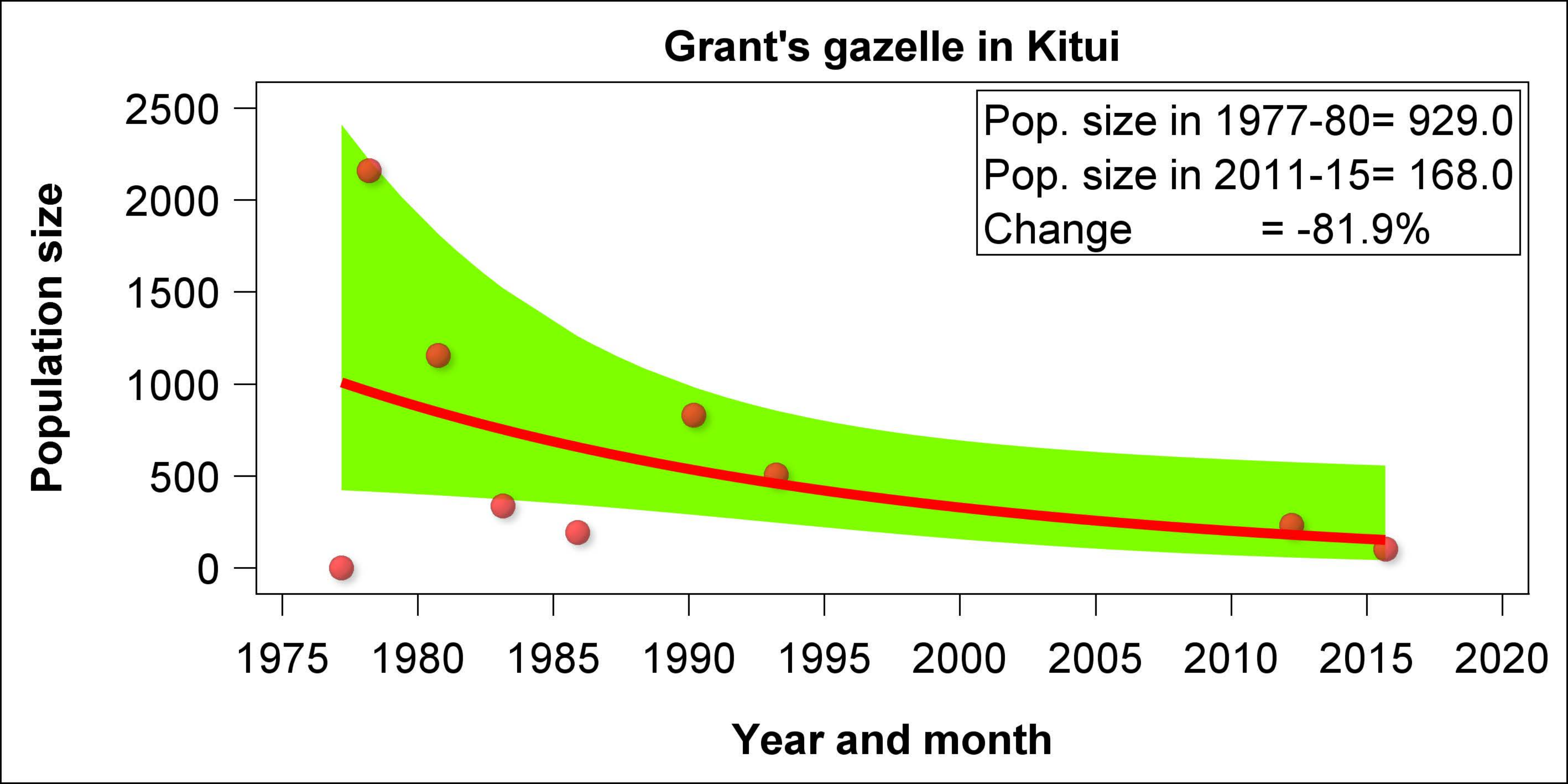

Grant's gazelle in Kitui
Pop. size in 1977-80= 929.0
Pop. size in 2011-15= 168.0
Change = -81.9%
Population size
0
500
1000
1500
2000
2500
1975
1980
1985
1990
1995
2000
2005
2010
2015
2020
Year and month

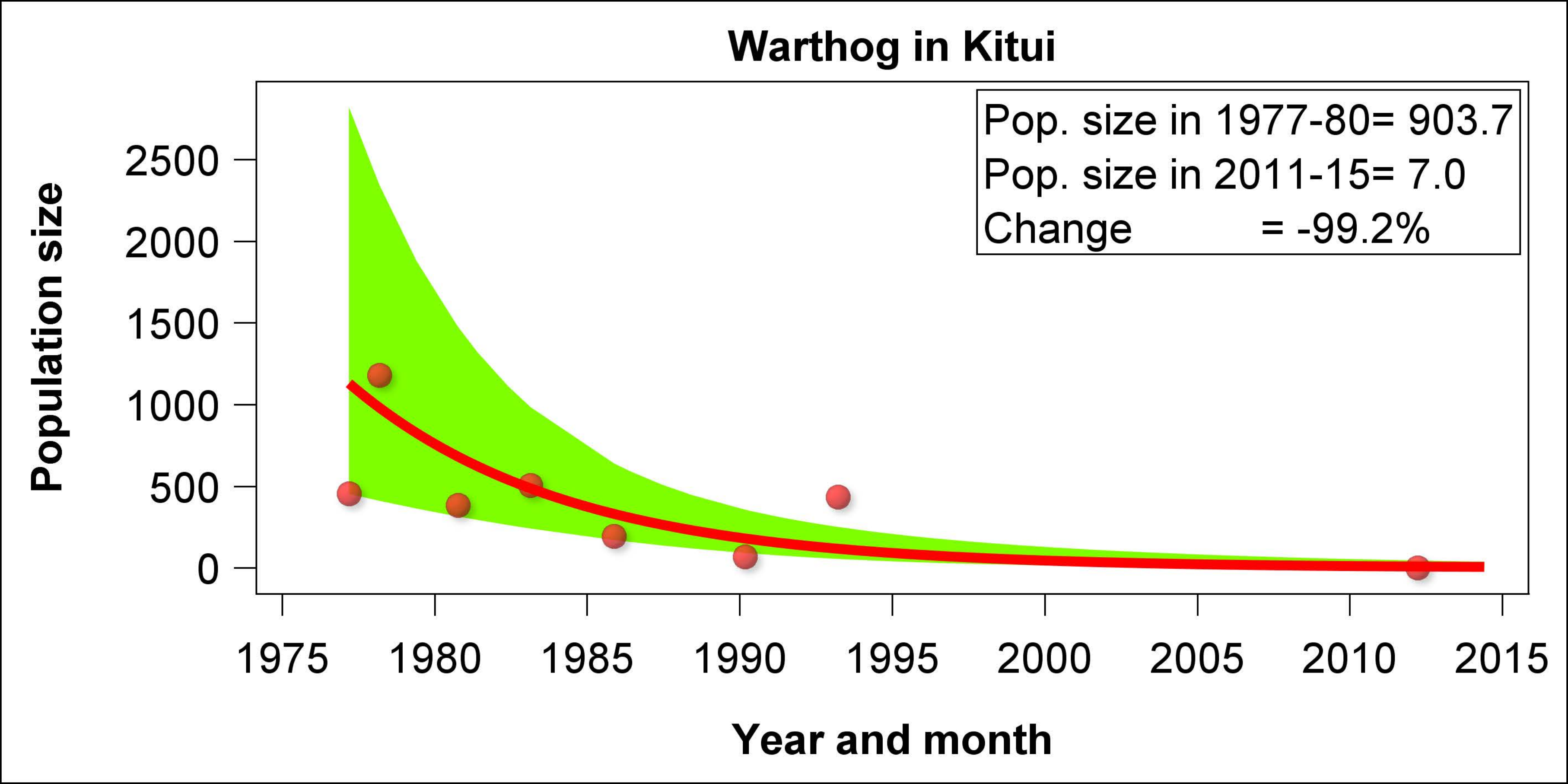
Warthog in Kitui
Pop. size in 1977-80= 903.7
Pop. size in 2011-15= 7.0
Change = -99.2%
Population size
2500
2000
1500
1000
500
0
1975
1980
1985
1990
1995
2000
2005
2010
2015
Year and month

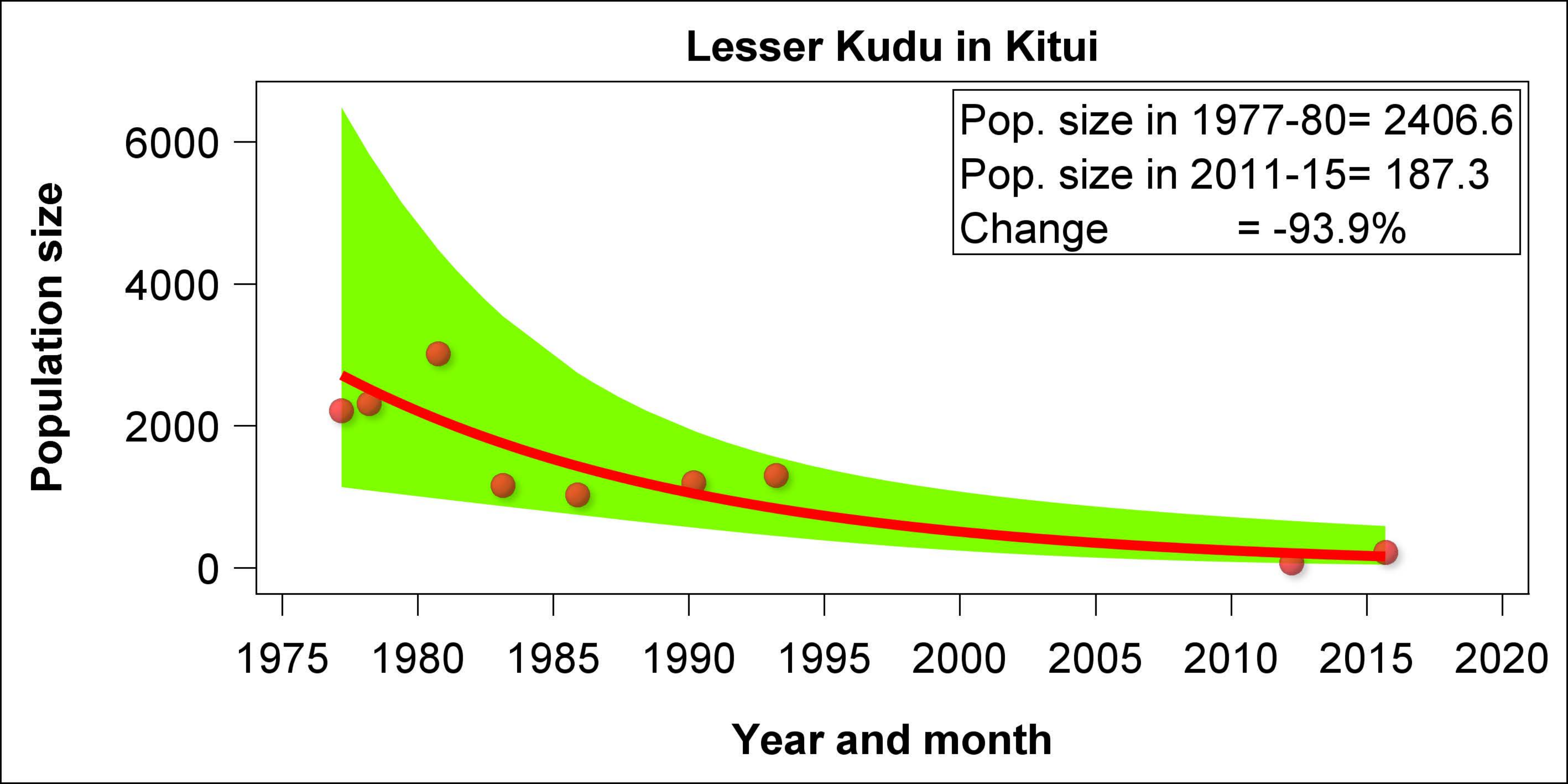
Lesser Kudu in Kitui
Pop. size in 1977-80= 2406.6
Pop. size in 2011-15= 187.3
Change = -93.9%
Population size
6000
4000
2000
0
1975
1980
1985
1990
1995
2000
2005
2010
2015
2020
Year and month

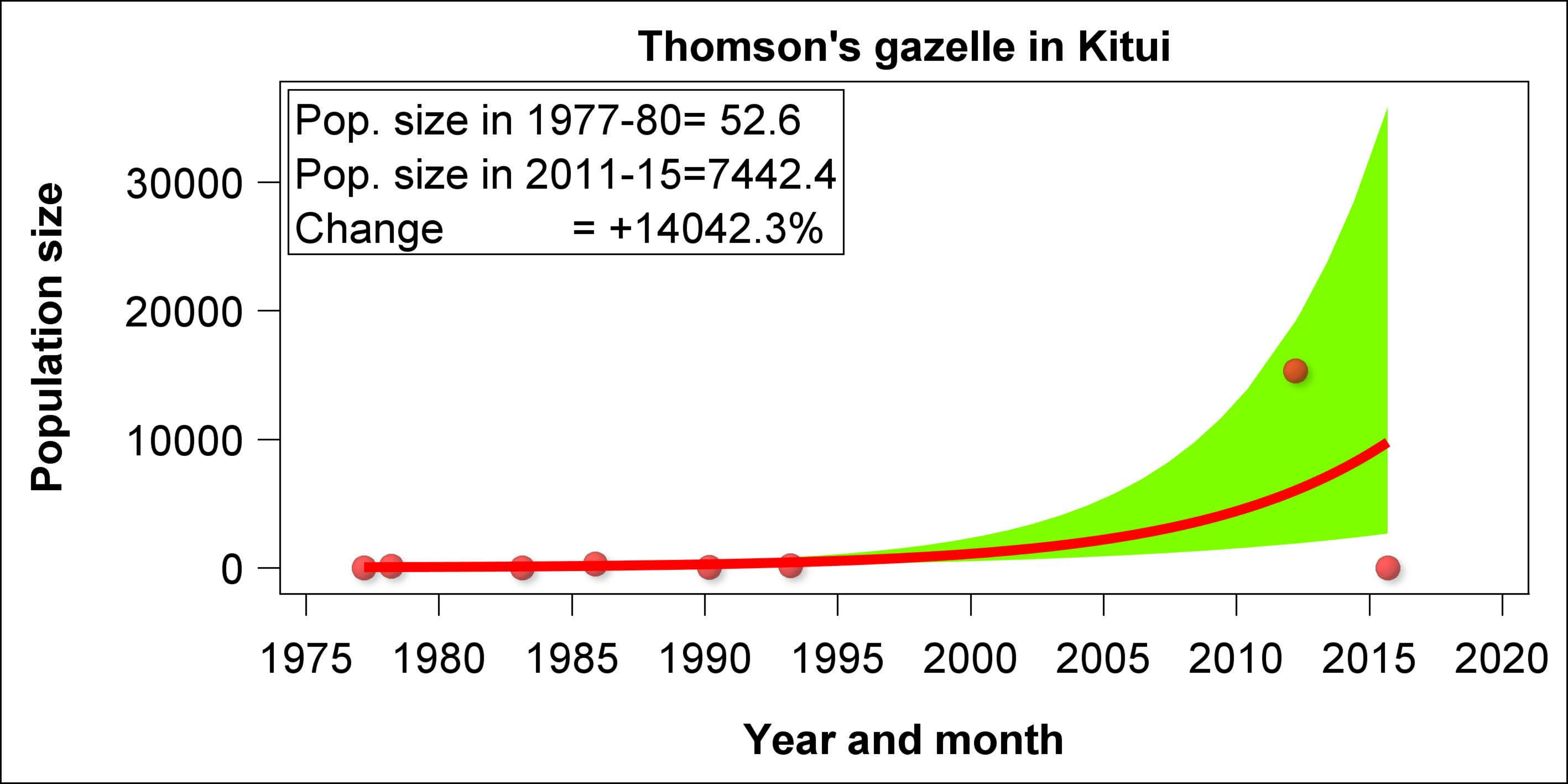
Thomson's gazelle in Kitui
Pop. size in 1977-80= 52.6
Pop. size in 2011-15=7442.4
Change = +14042.3%
Population size
30000
20000
10000
0
1975
1980
1985
1990
1995
2000
2005
2010
2015
2020
Year and month

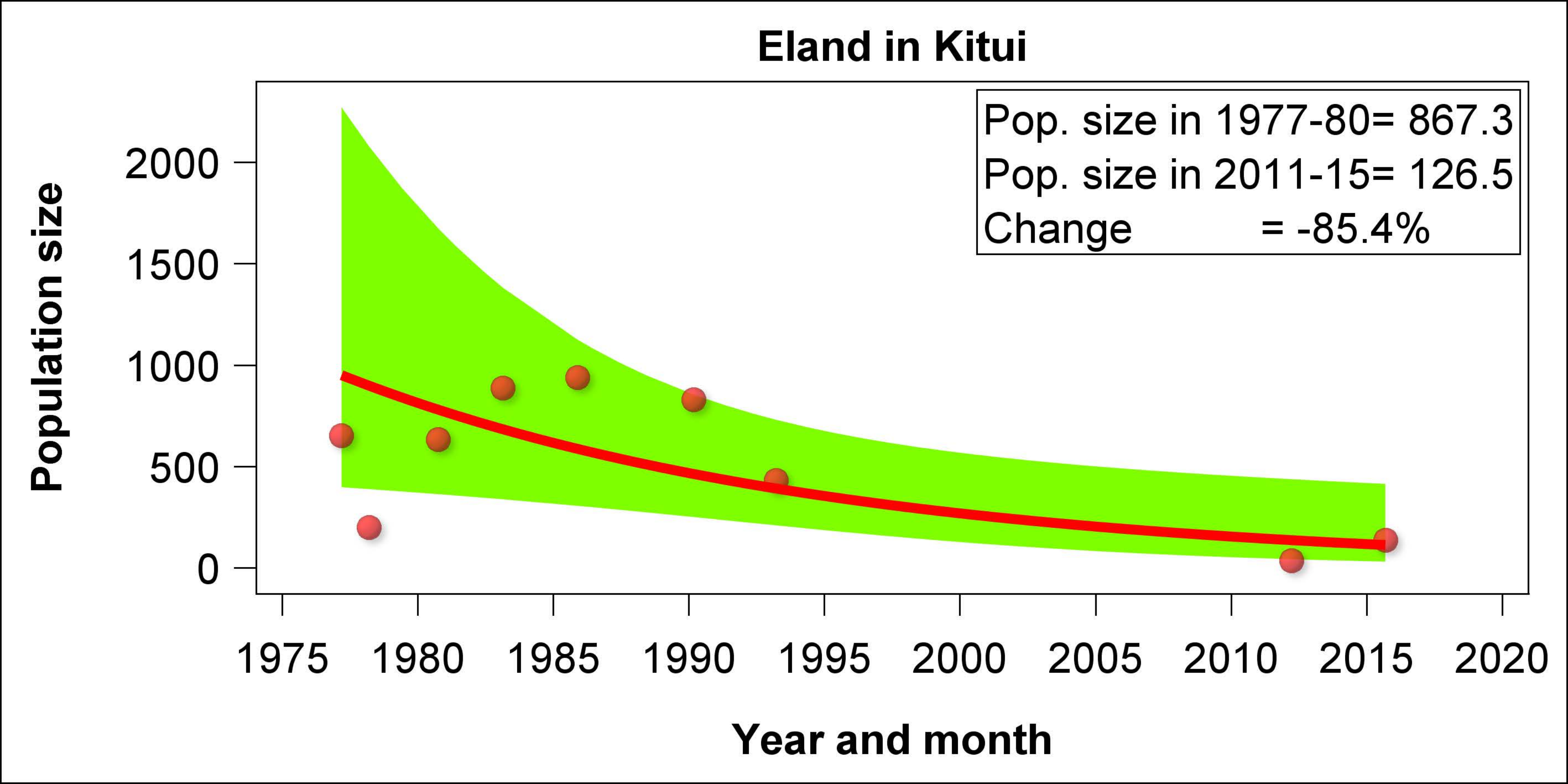

Eland in Kitui
Pop. size in 1977-80= 867.3
Pop. size in 2011-15= 126.5
Change = -85.4%
Population size
2000
1500
1000
500
0
1975
1980
1985
1990
1995
2000
2005
2010
2015
2020
Year and month

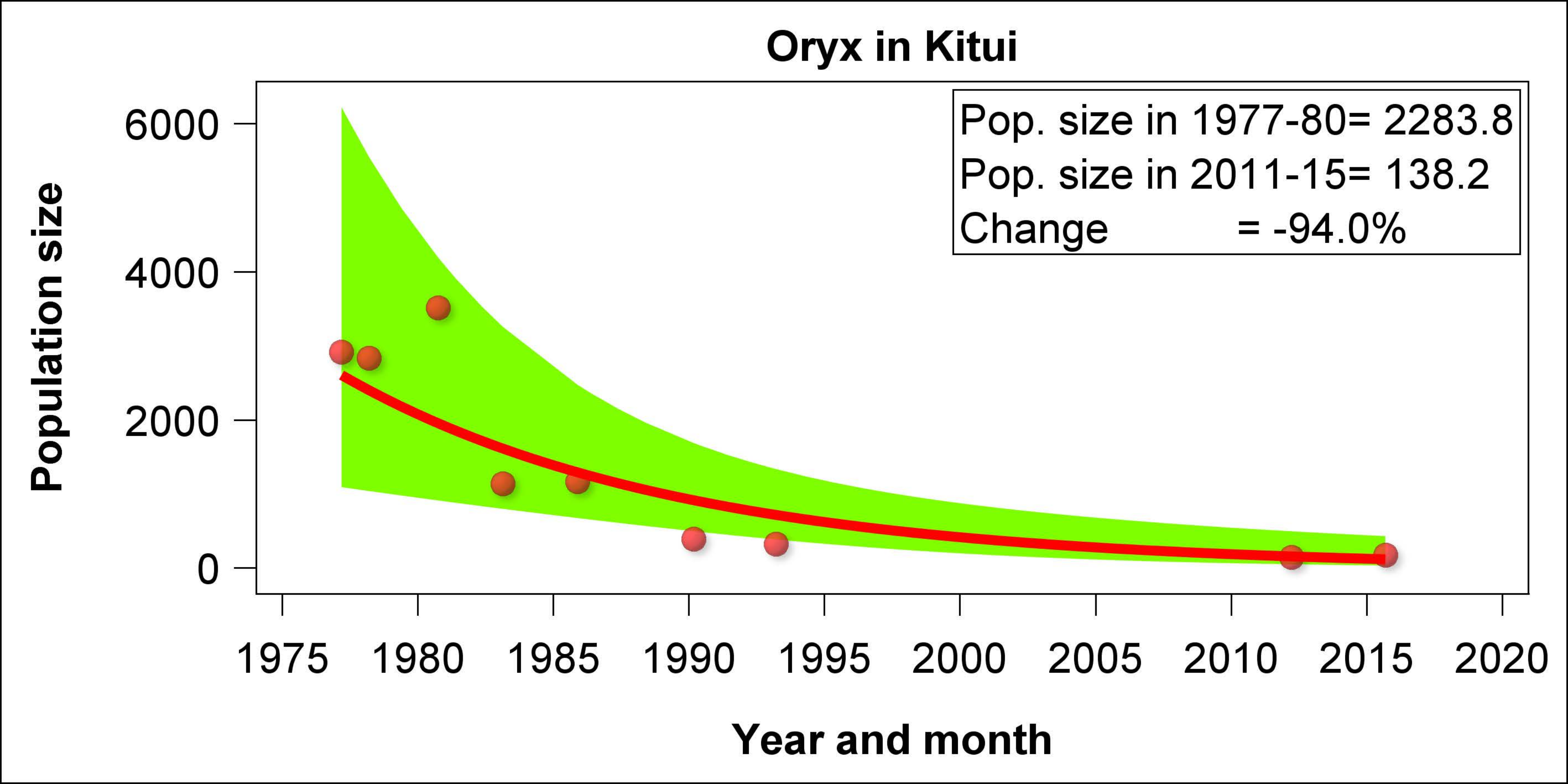

Oryx in Kitui
Pop. size in 1977-80= 2283.8
Pop. size in 2011-15= 138.2
Change = -94.0%
Population size
6000
4000
2000
0
1975
1980
1985
1990
1995
2000
2005
2010
2015
2020
Year and month

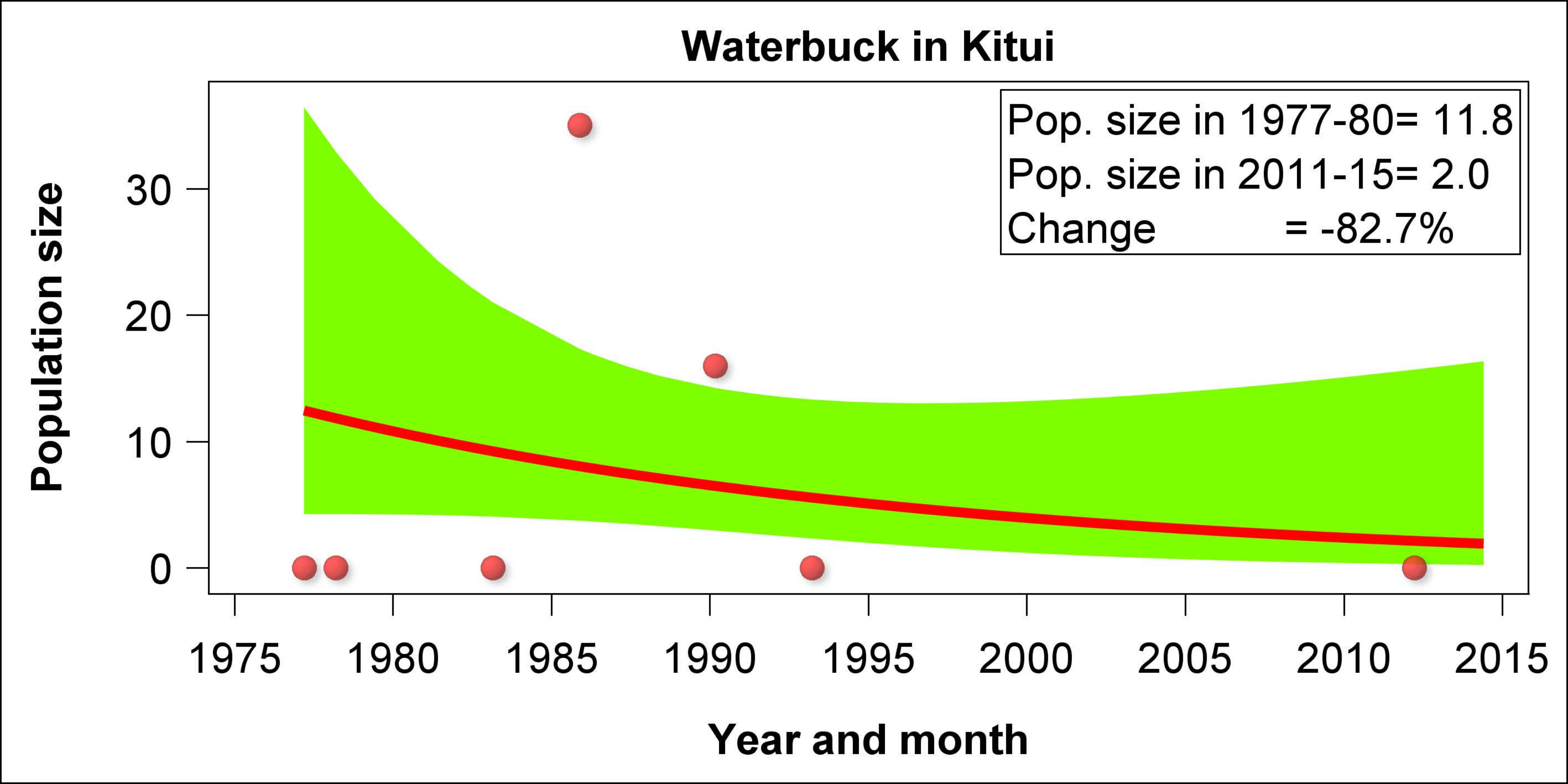

Waterbuck in Kitui
Pop. size in 1977-80= 11.8
Pop. size in 2011-15= 2.0
Change = -82.7%
Population size
0
10
20
30
1975
1980
1985
1990
1995
2000
2005
2010
2015
Year and month

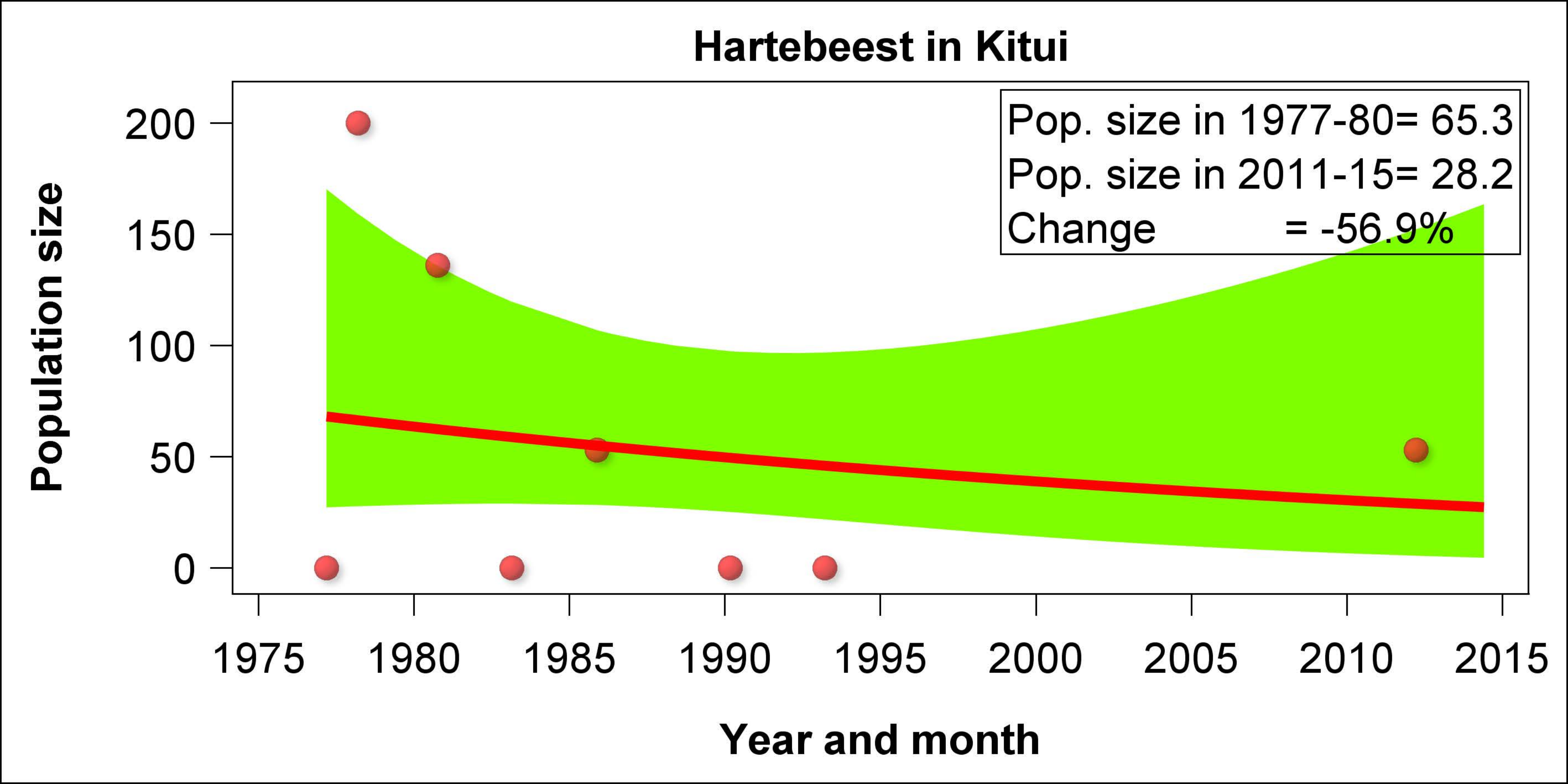
Hartebeest in Kitui
Pop. size in 1977-80= 65.3
Pop. size in 2011-15= 28.2
Change = -56.9%
Population size
200
150
100
50
0
1975
1980
1985
1990
1995
2000
2005
2010
2015
Year and month

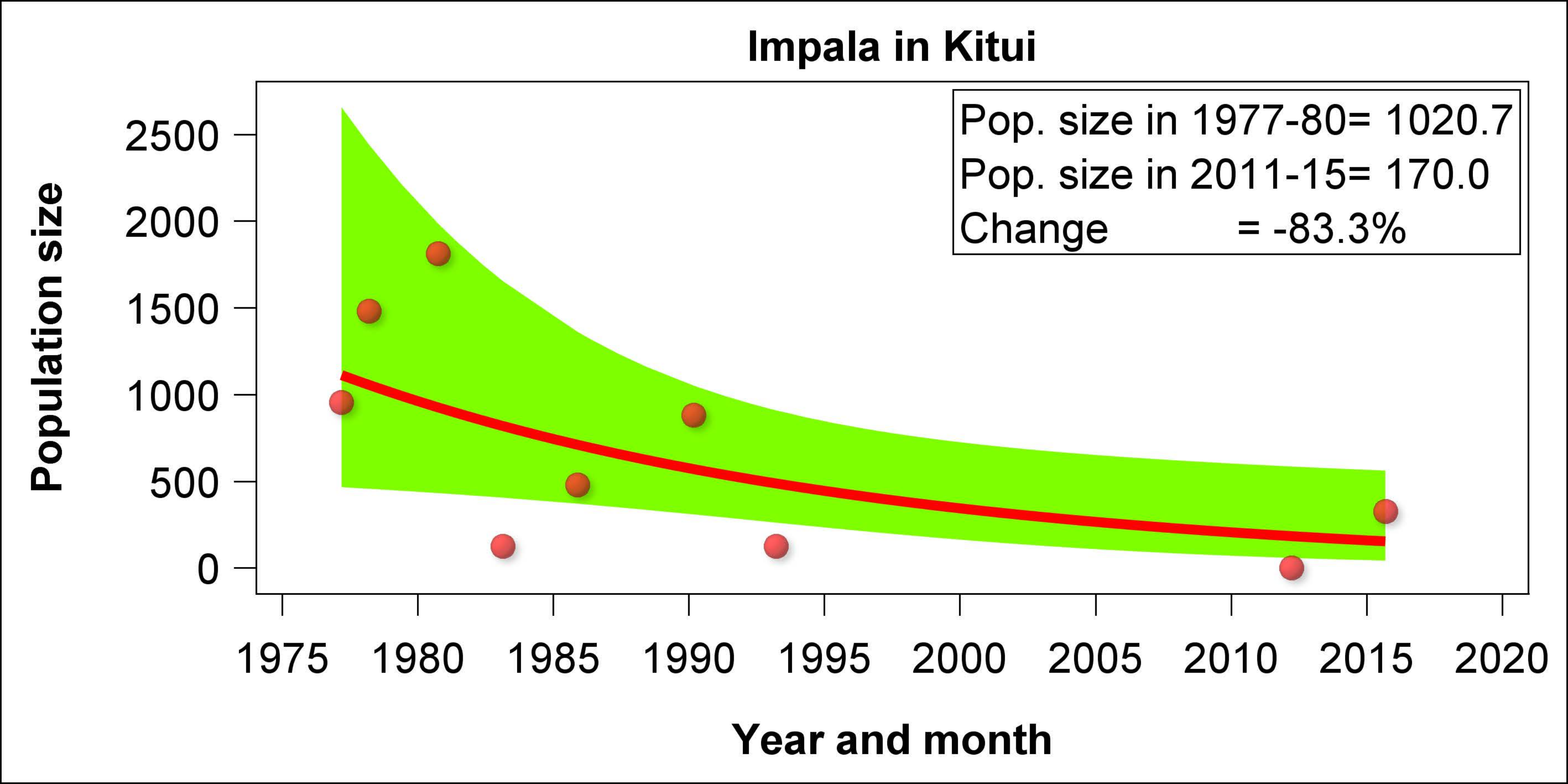
Impala in Kitui
Pop. size in 1977-80= 1020.7
Pop. size in 2011-15= 170.0
Change = -83.3%
Population size
0
500
1000
1500
2000
2500
1975
1980
1985
1990
1995
2000
2005
2010
2015
2020
Year and month

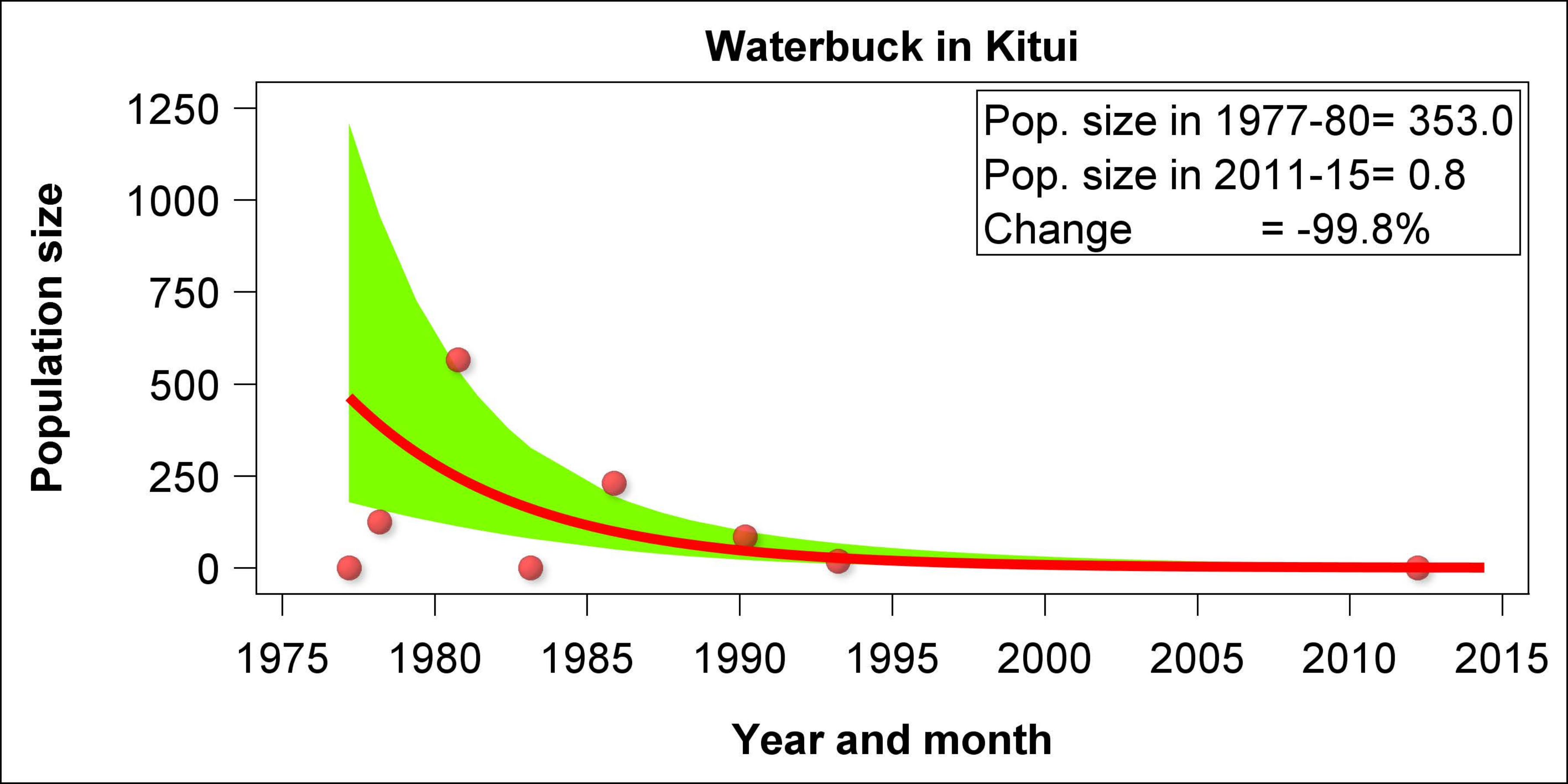
Waterbuck in Kitui
Pop. size in 1977-80= 353.0
Pop. size in 2011-15= 0.8
Change = -99.8%
Population size
1250
1000
750
500
250
0
1975
1980
1985
1990
1995
2000
2005
2010
2015
Year and month
