# Supplementary material for: Extreme Wildlife Declines and Concurrent Increase in Livestock Numbers in Kenya: What Are the Causes?
Source: PLoS One. 2016 Sep 27;11(9):e0163249. doi: 10.1371/journal.pone.0163249 (PMC5039022; doi:10.1371/journal.pone.0163249)
Supplement: S6 Fig — The solid red line is the fitted trend curve and the shaded chartreuse band is the pointwise 95% confidence band. The estimated average population size in 1977–1980 and 2011–2014 and the percentage change in population size between the two periods are provided in the inset. (PDF) [file pone.0163249.s016.pdf]

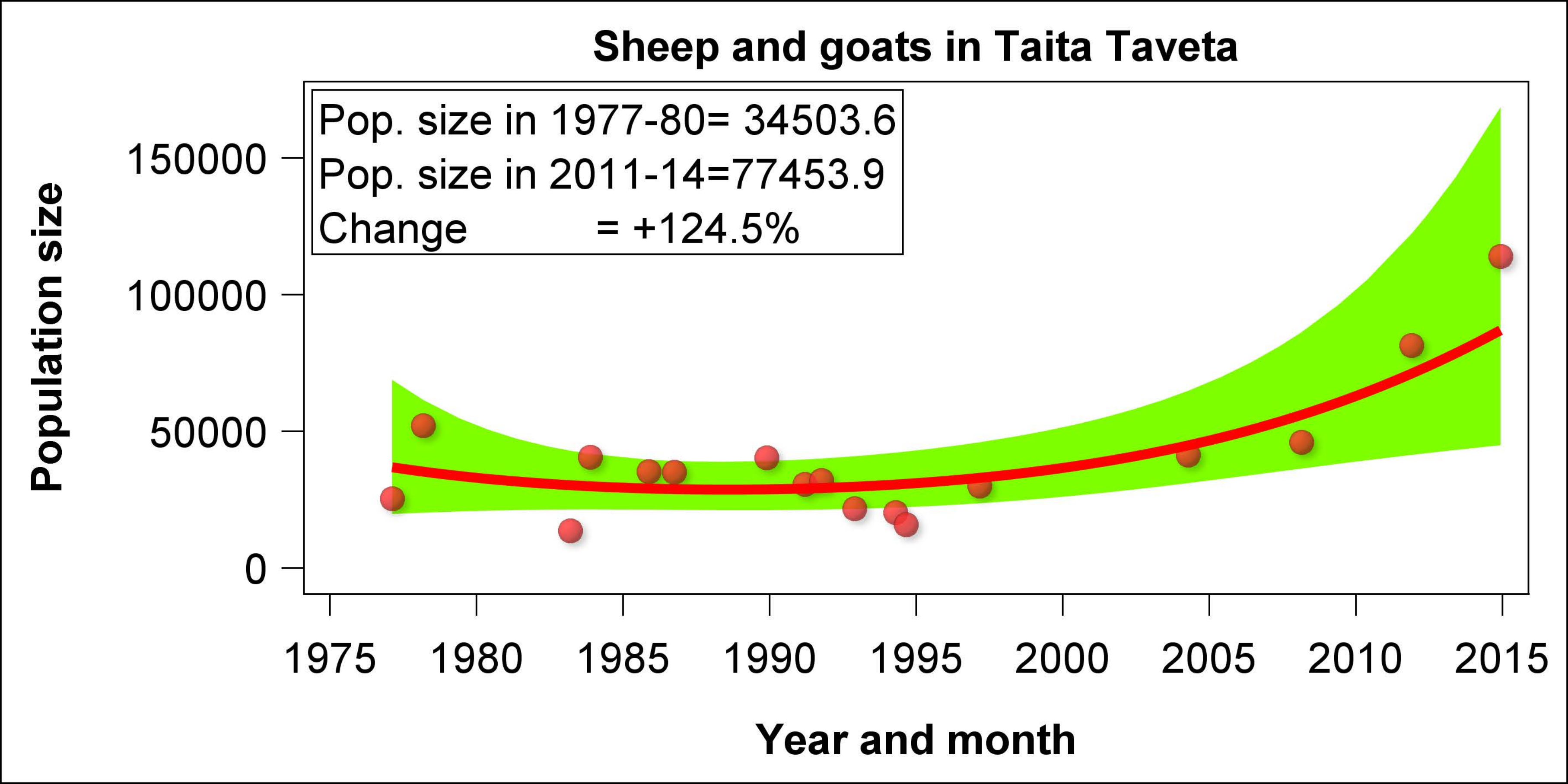

Sheep and goats in Taita Taveta
Pop. size in 1977-80= 34503.6
Pop. size in 2011-14=77453.9
Change = +124.5%
150000
100000
50000
0
Population size
1975
1980
1985
1990
1995
2000
2005
2010
2015
Year and month

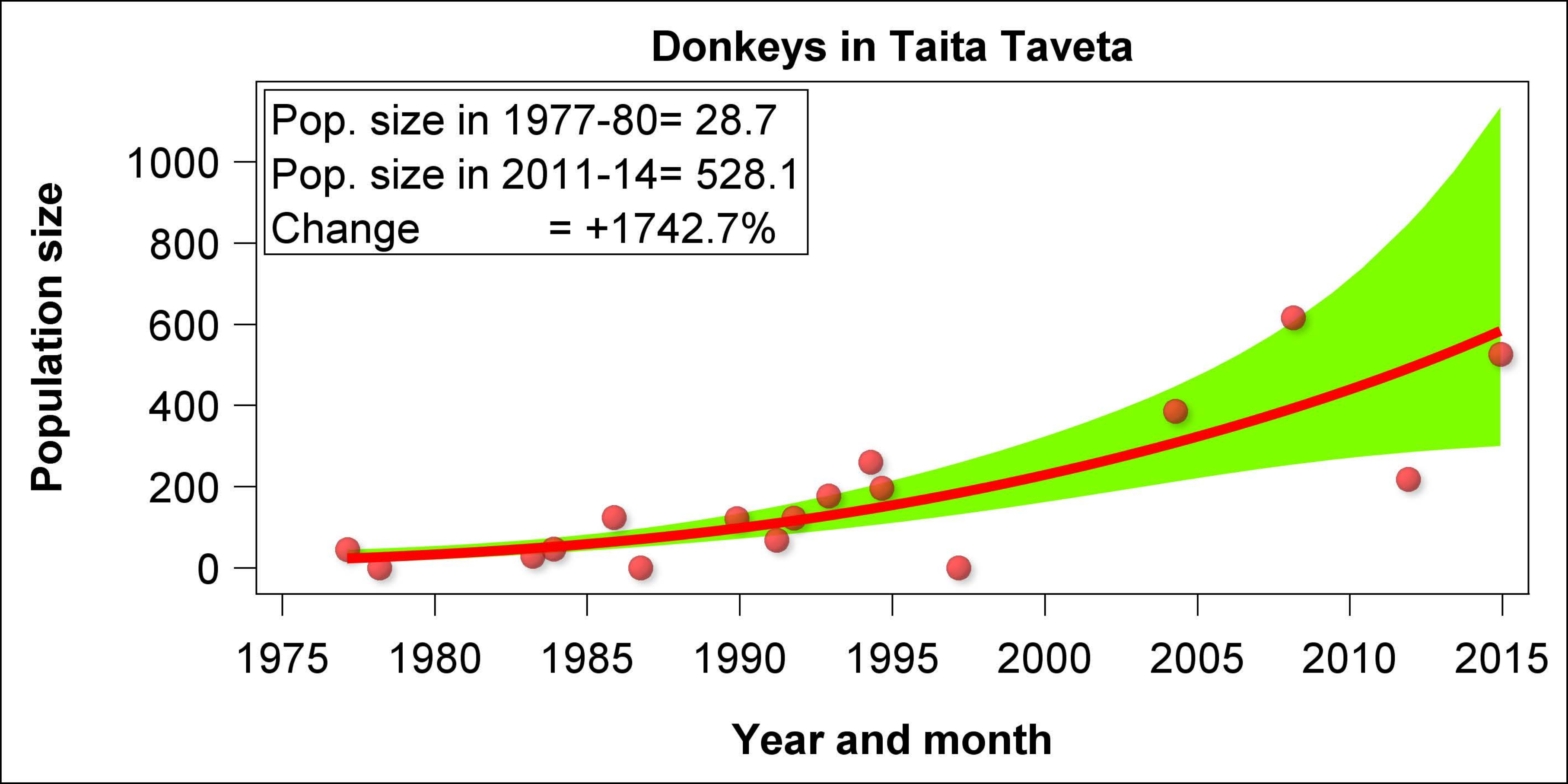
Donkeys in Taita Taveta
Pop. size in 1977-80= 28.7
Pop. size in 2011-14= 528.1
Change = +1742.7%
Population size
0
200
400
600
800
1000
1975
1980
1985
1990
1995
2000
2005
2010
2015
Year and month

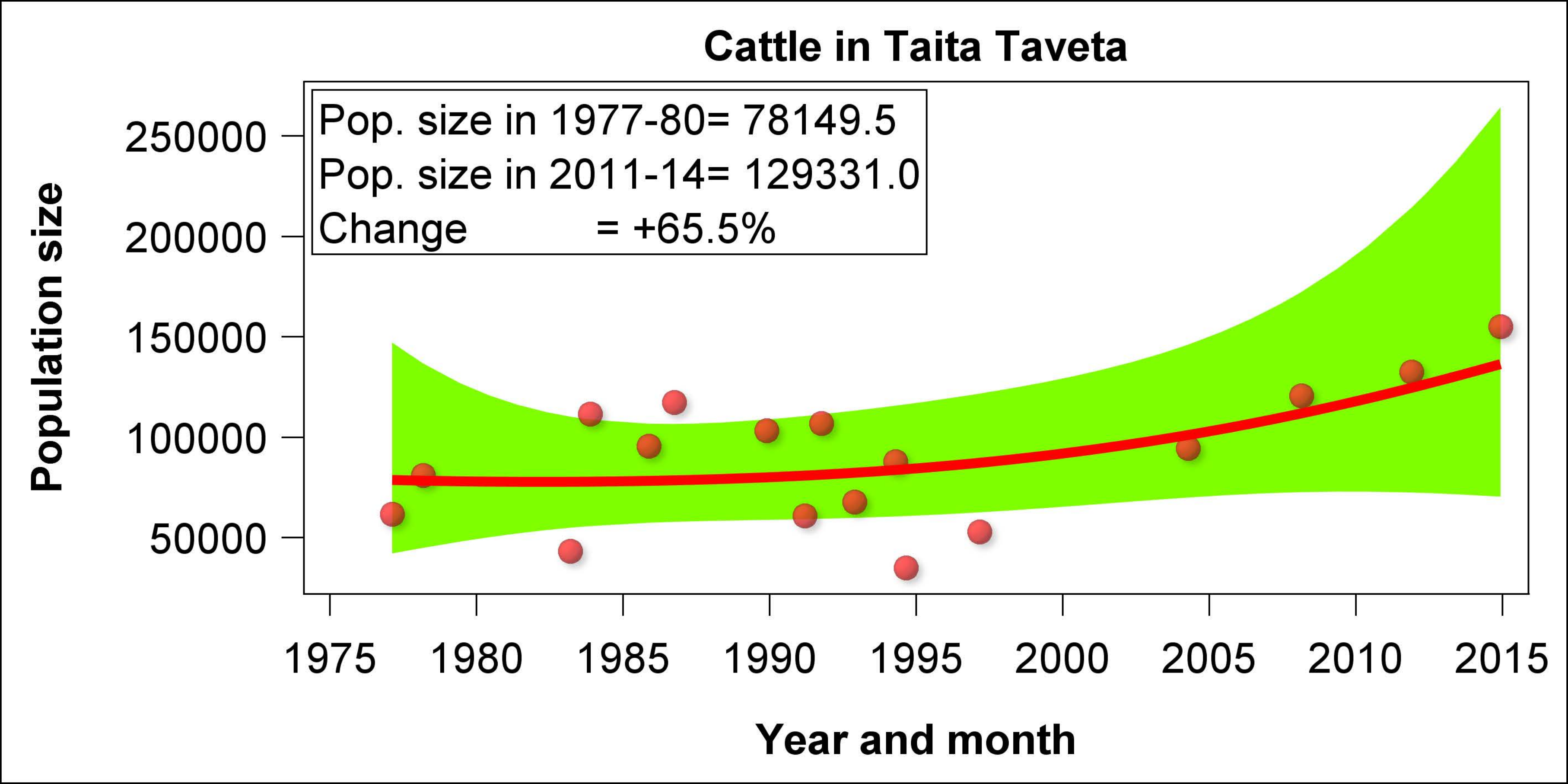
Cattle in Taita Taveta
Pop. size in 1977-80= 78149.5
Pop. size in 2011-14= 129331.0
Change = +65.5%
Population size
250000
200000
150000
100000
50000
1975
1980
1985
1990
1995
2000
2005
2010
2015
Year and month

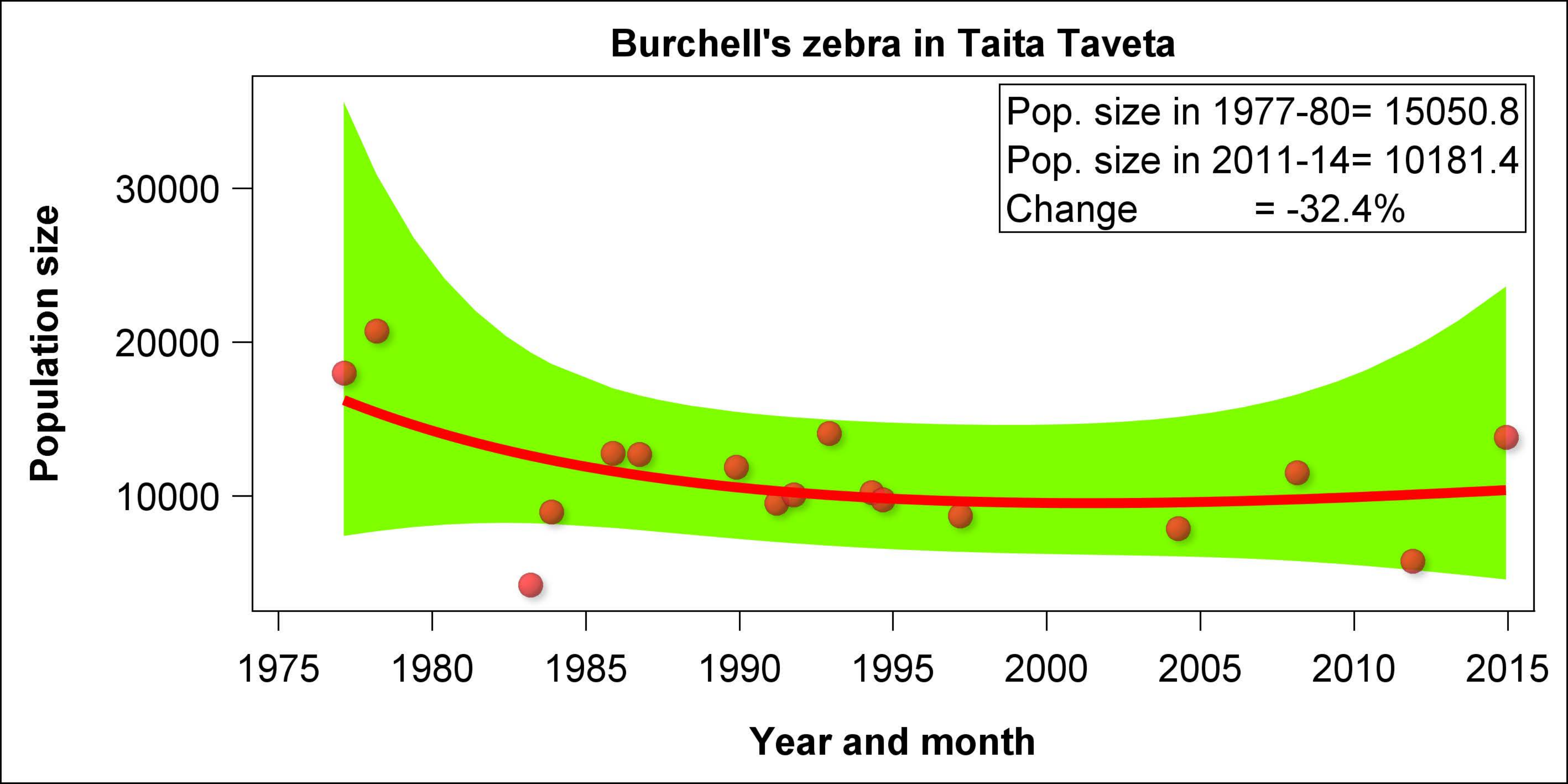
Burchell's zebra in Taita Taveta
Pop. size in 1977-80= 15050.8
Pop. size in 2011-14= 10181.4
Change = -32.4%
Population size
30000
20000
10000
1975
1980
1985
1990
1995
2000
2005
2010
2015
Year and month

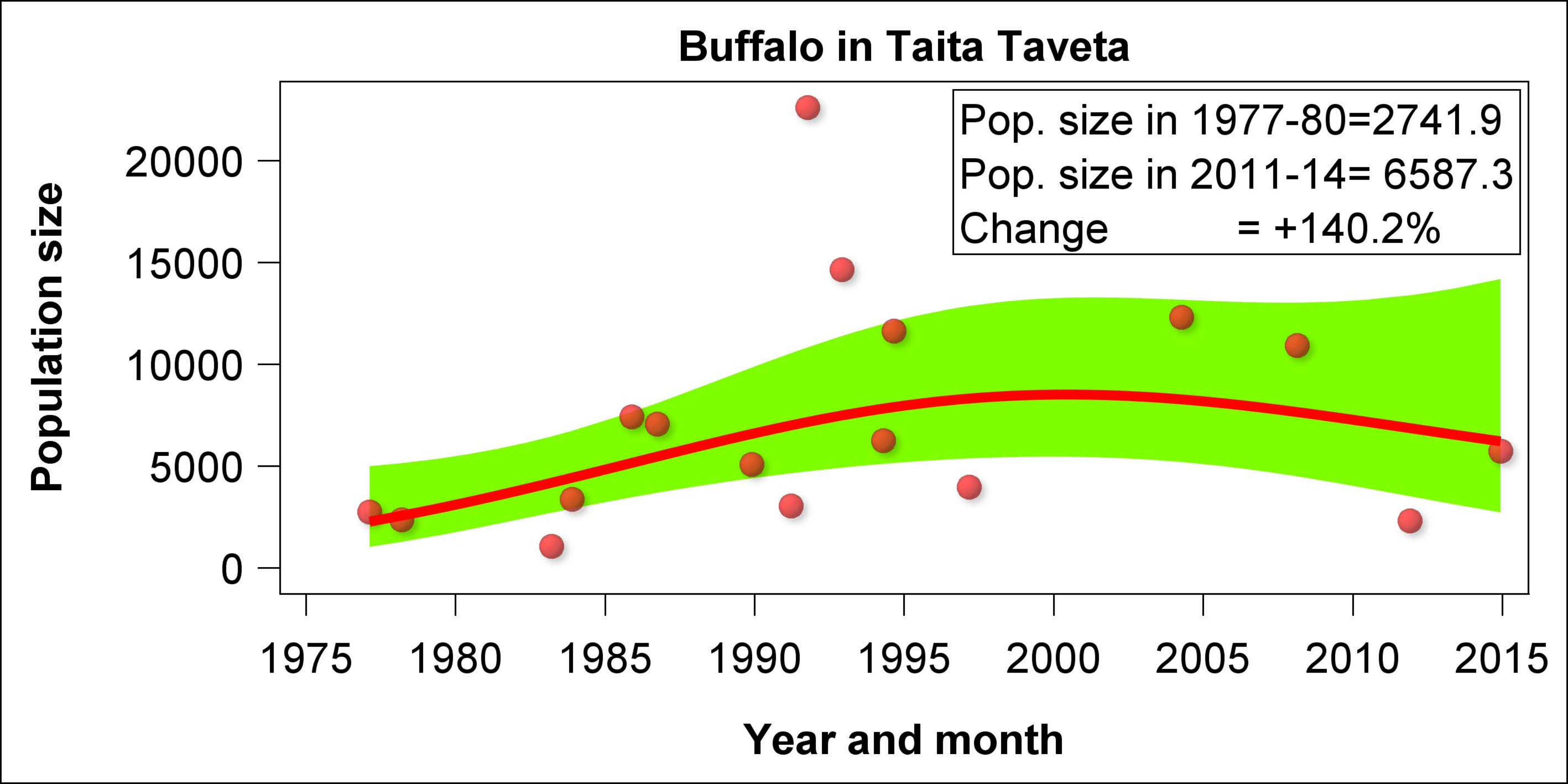
Buffalo in Taita Taveta
Pop. size in 1977-80=2741.9
Pop. size in 2011-14= 6587.3
Change = +140.2%
Population size
20000
15000
10000
5000
0
1975
1980
1985
1990
1995
2000
2005
2010
2015
Year and month

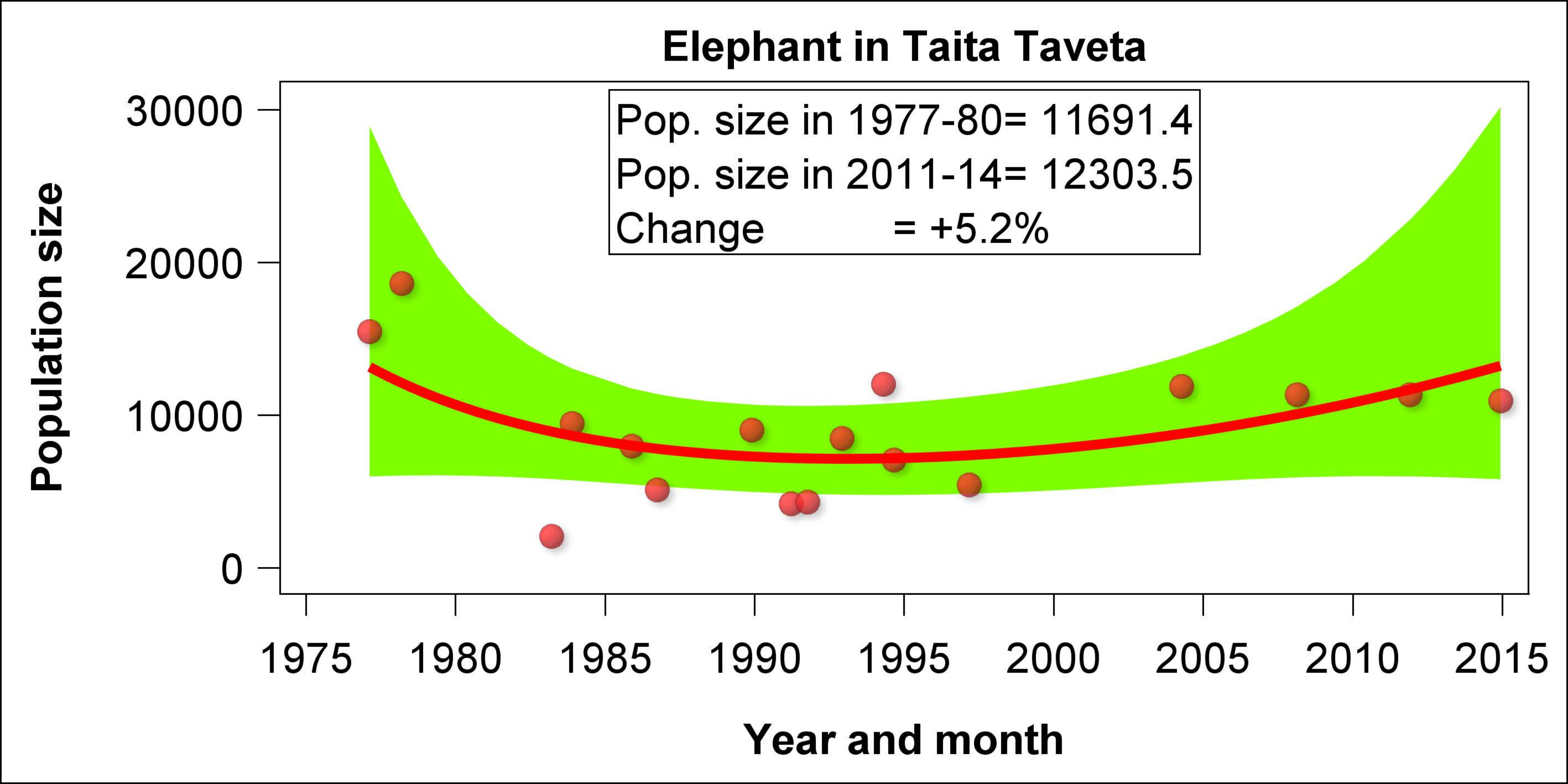

Elephant in Taita Taveta
Pop. size in 1977-80= 11691.4
Pop. size in 2011-14= 12303.5
Change = +5.2%
Population size
30000
20000
10000
0
1975
1980
1985
1990
1995
2000
2005
2010
2015
Year and month

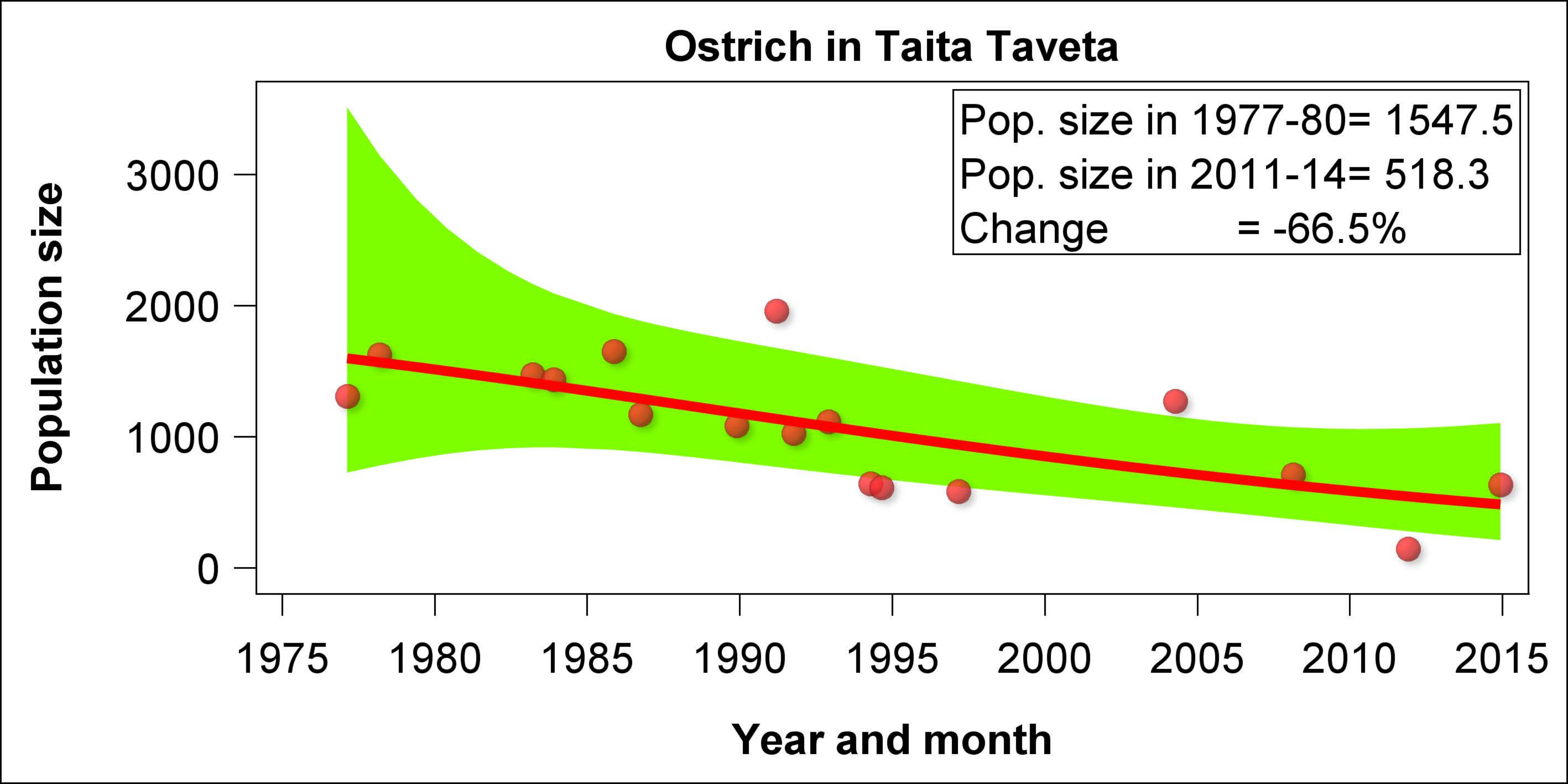
Ostrich in Taita Taveta
Pop. size in 1977-80= 1547.5
Pop. size in 2011-14= 518.3
Change = -66.5%
Population size
3000
2000
1000
0
1975
1980
1985
1990
1995
2000
2005
2010
2015
Year and month

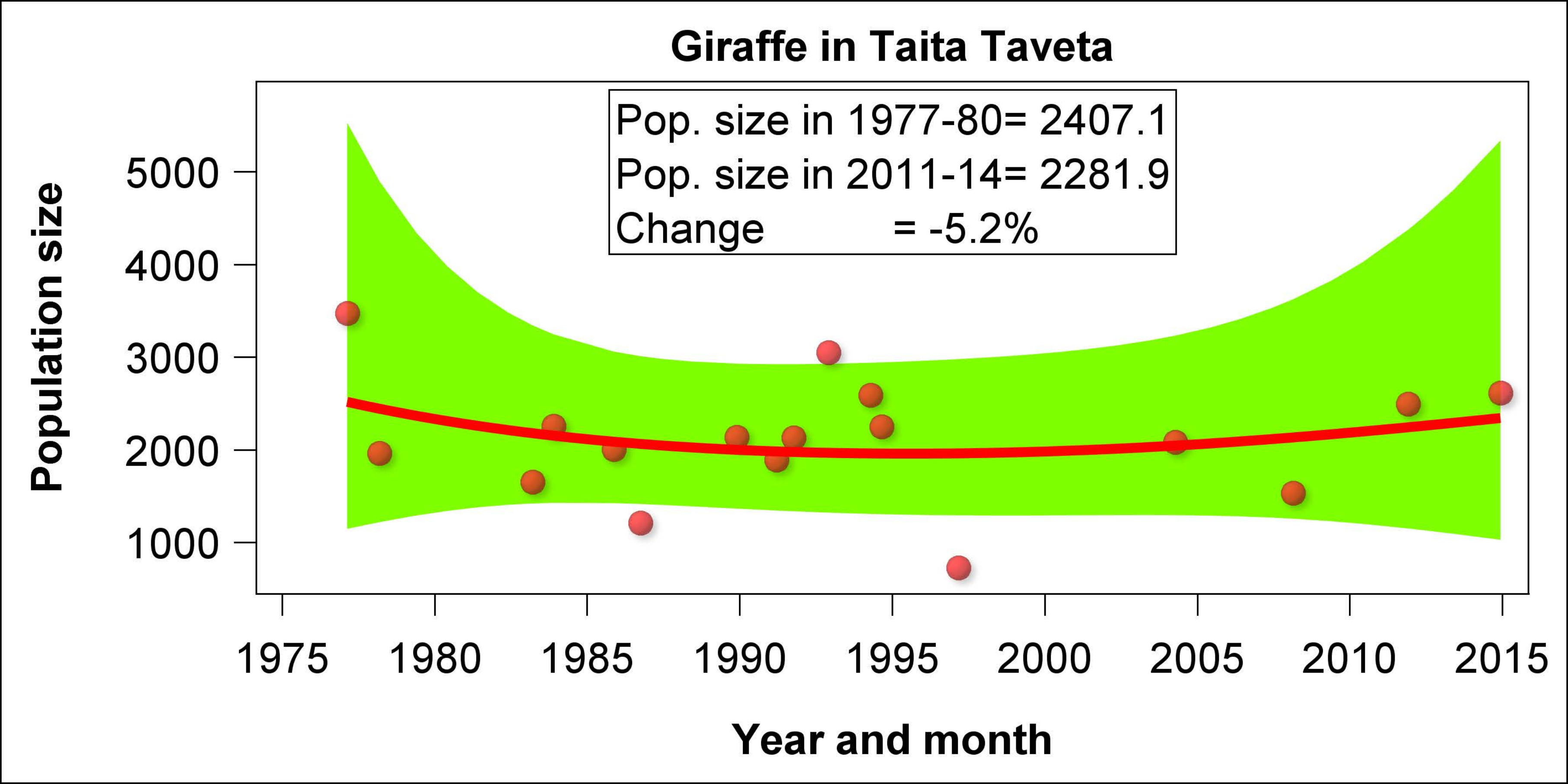

Giraffe in Taita Taveta
Pop. size in 1977-80= 2407.1
Pop. size in 2011-14= 2281.9
Change = -5.2%
Population size
5000
4000
3000
2000
1000
1975
1980
1985
1990
1995
2000
2005
2010
2015
Year and month

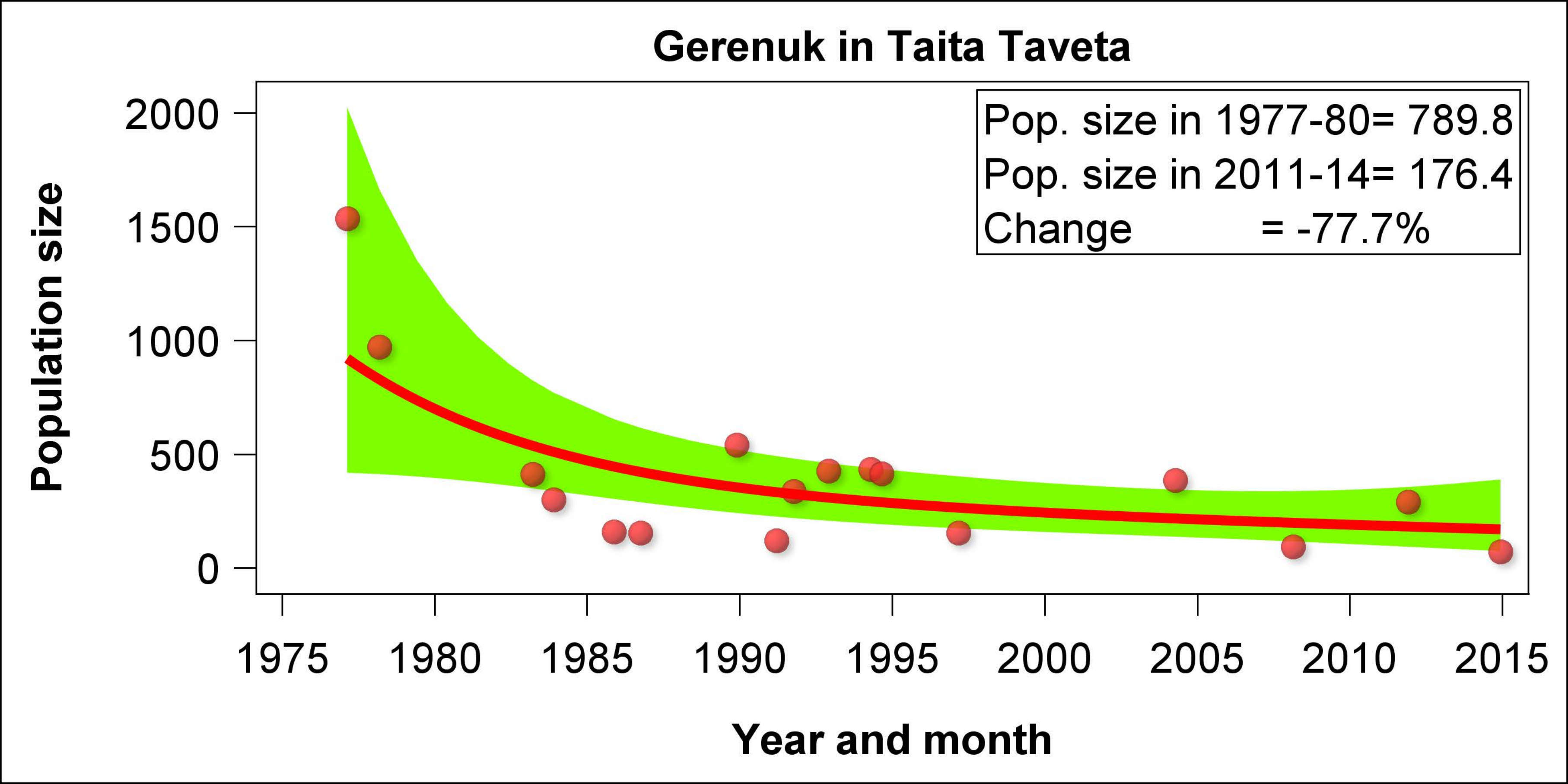

Gerenuk in Taita Taveta
Pop. size in 1977-80= 789.8
Pop. size in 2011-14= 176.4
Change = -77.7%
Population size
2000
1500
1000
500
0
1975
1980
1985
1990
1995
2000
2005
2010
2015
Year and month

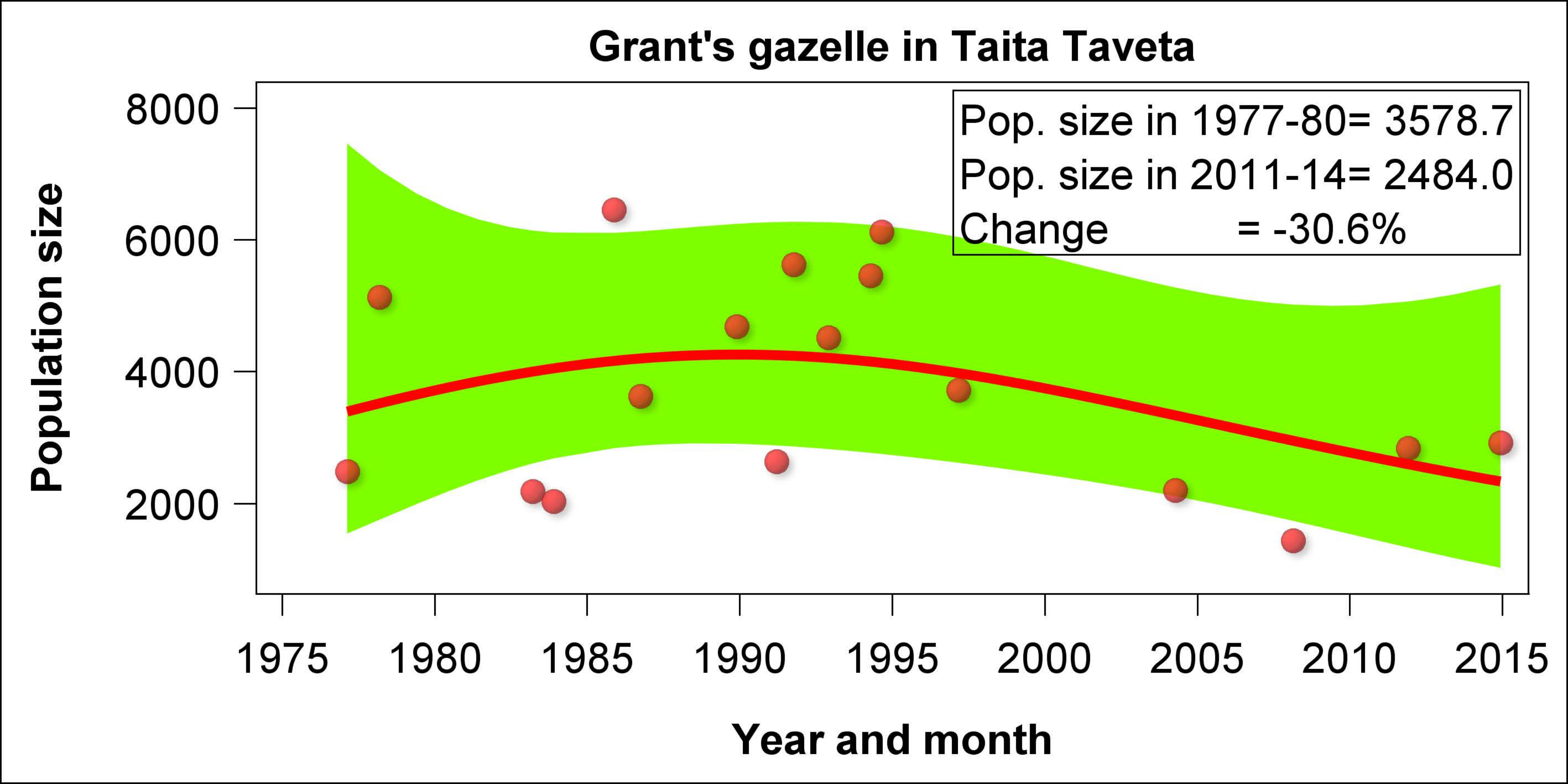

Grant's gazelle in Taita Taveta
Pop. size in 1977-80= 3578.7
Pop. size in 2011-14= 2484.0
Change = -30.6%
8000
6000
4000
2000
Population size
1975
1980
1985
1990
1995
2000
2005
2010
2015
Year and month

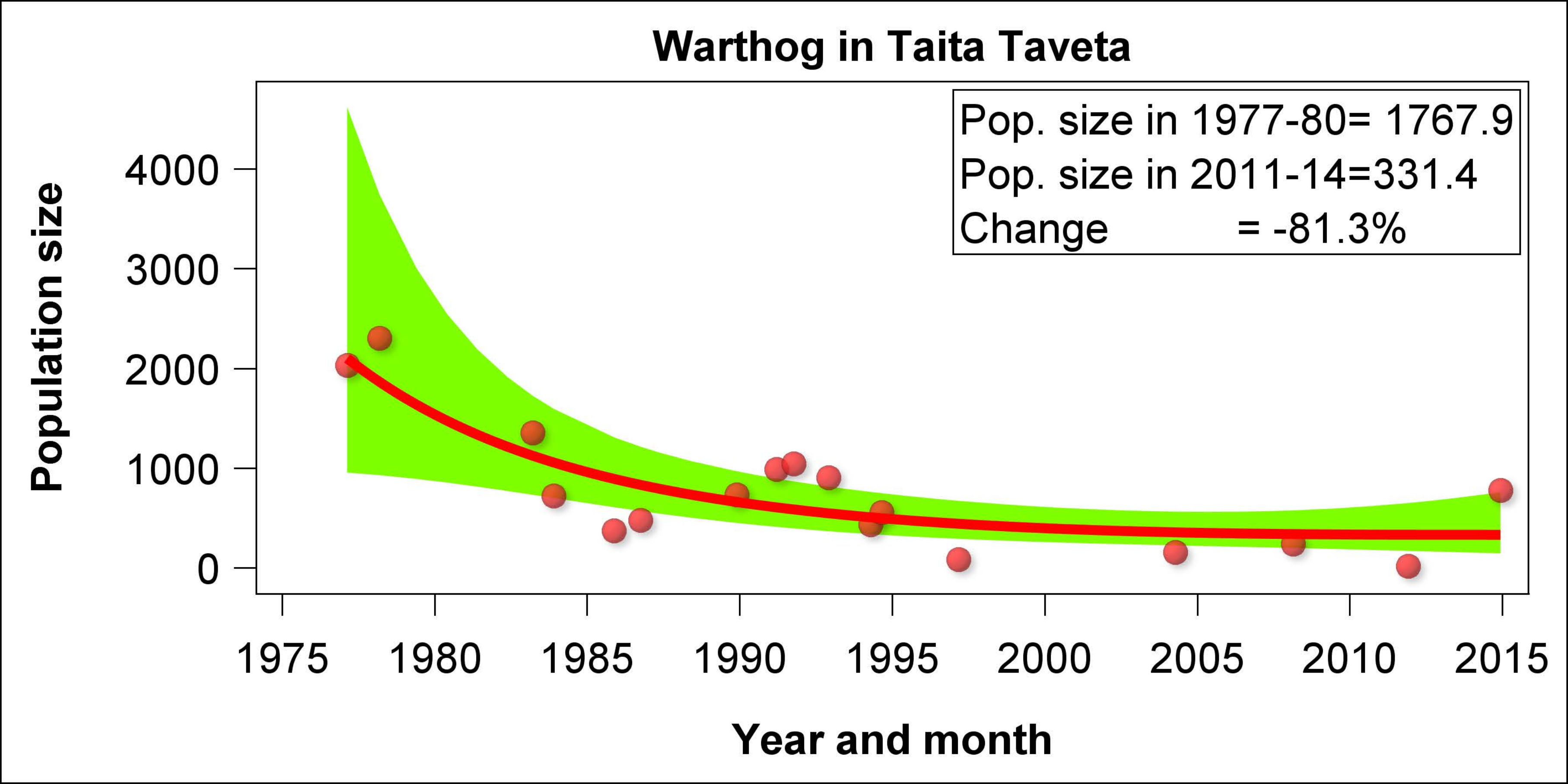
Warthog in Taita Taveta
Pop. size in 1977-80= 1767.9
Pop. size in 2011-14=331.4
Change = -81.3%
Population size
4000
3000
2000
1000
0
1975
1980
1985
1990
1995
2000
2005
2010
2015
Year and month

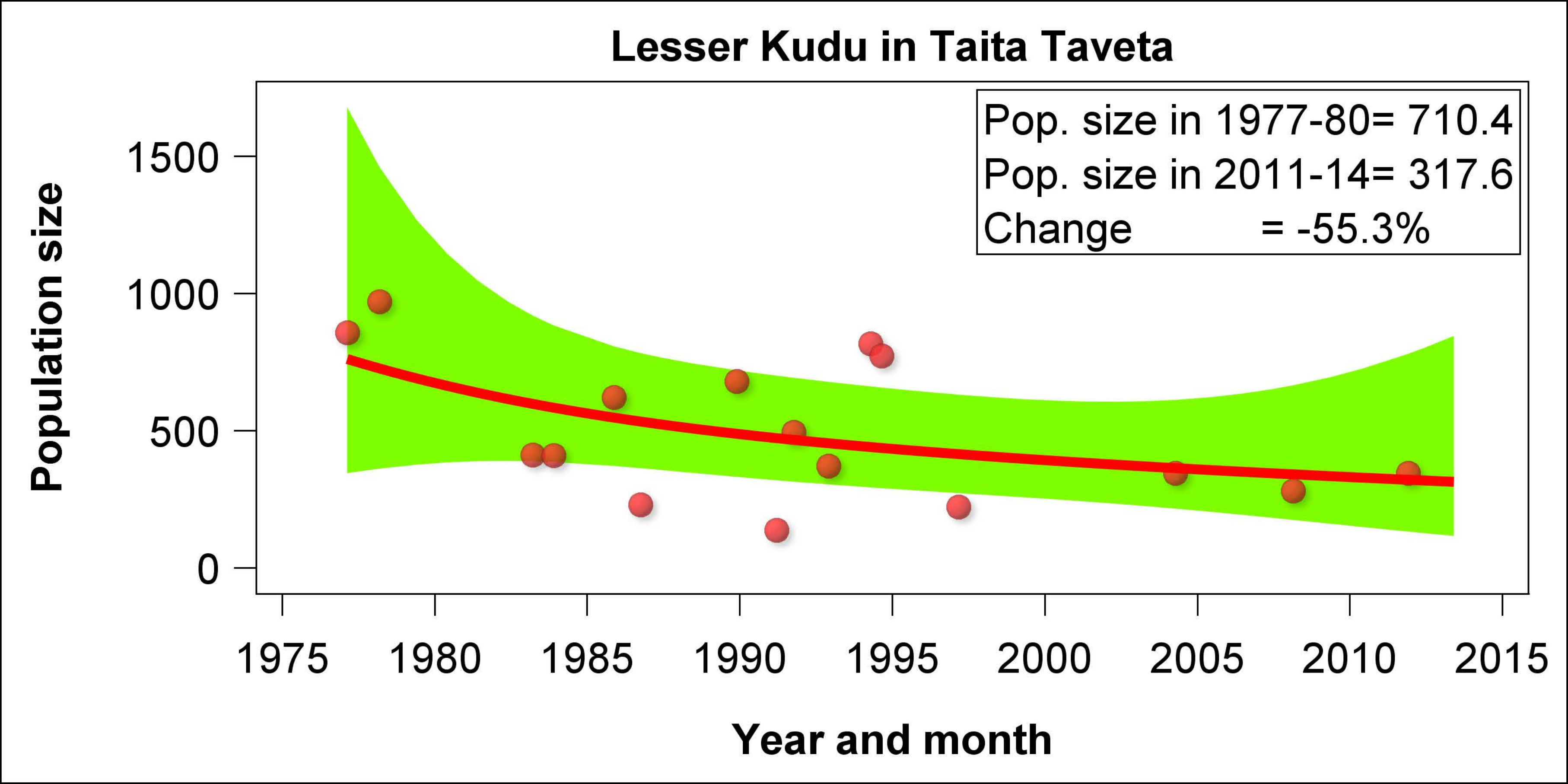

Lesser Kudu in Taita Taveta
Pop. size in 1977-80= 710.4
Pop. size in 2011-14= 317.6
Change = -55.3%
Population size
0
500
1000
1500
1975
1980
1985
1990
1995
2000
2005
2010
2015
Year and month

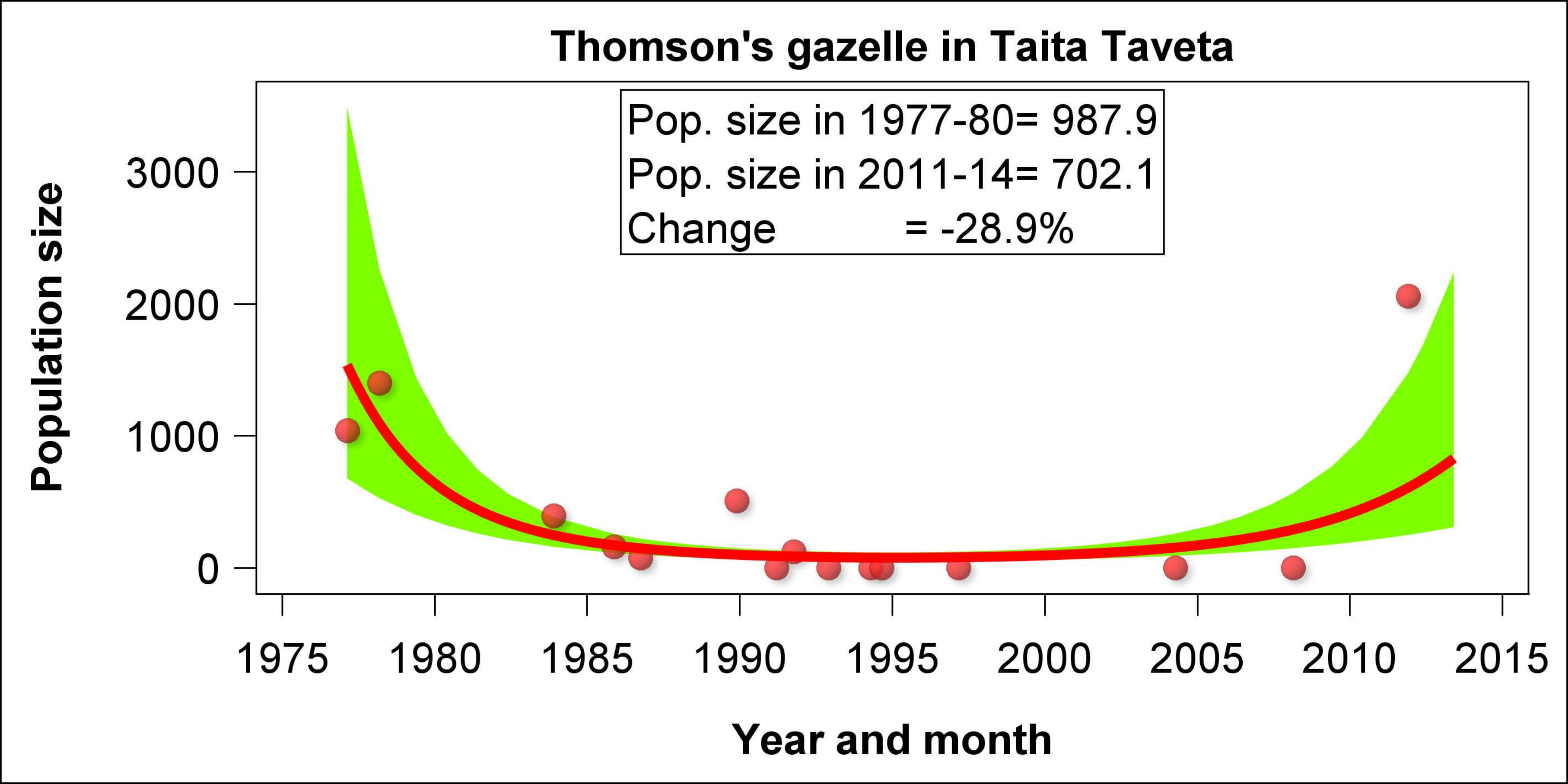

Thomson's gazelle in Taita Taveta
Pop. size in 1977-80= 987.9
Pop. size in 2011-14= 702.1
Change = -28.9%
Population size
0
1000
2000
3000
1975
1980
1985
1990
1995
2000
2005
2010
2015
Year and month

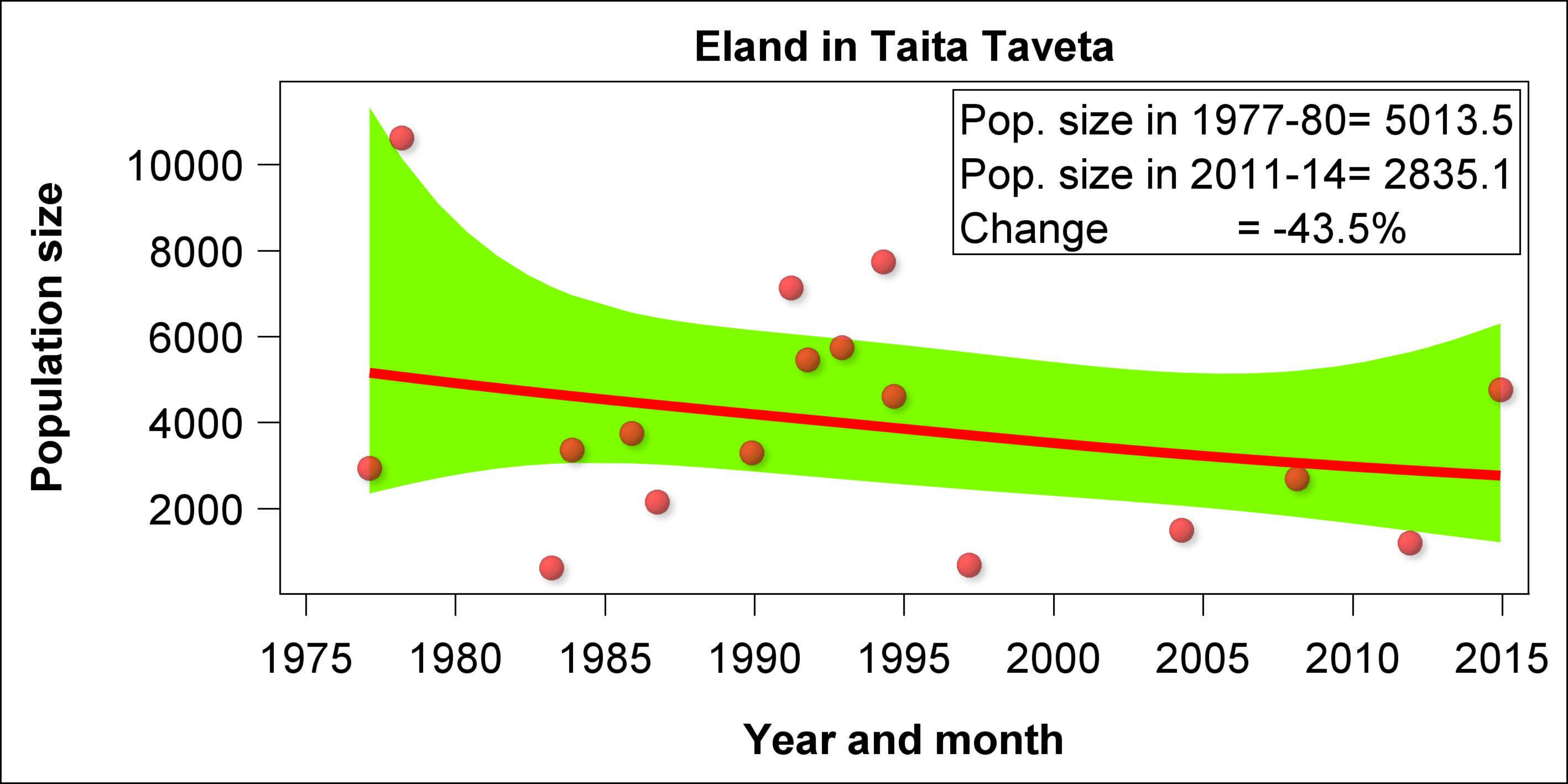

Eland in Taita Taveta
Pop. size in 1977-80= 5013.5
Pop. size in 2011-14= 2835.1
Change = -43.5%
Population size
10000
8000
6000
4000
2000
1975
1980
1985
1990
1995
2000
2005
2010
2015
Year and month

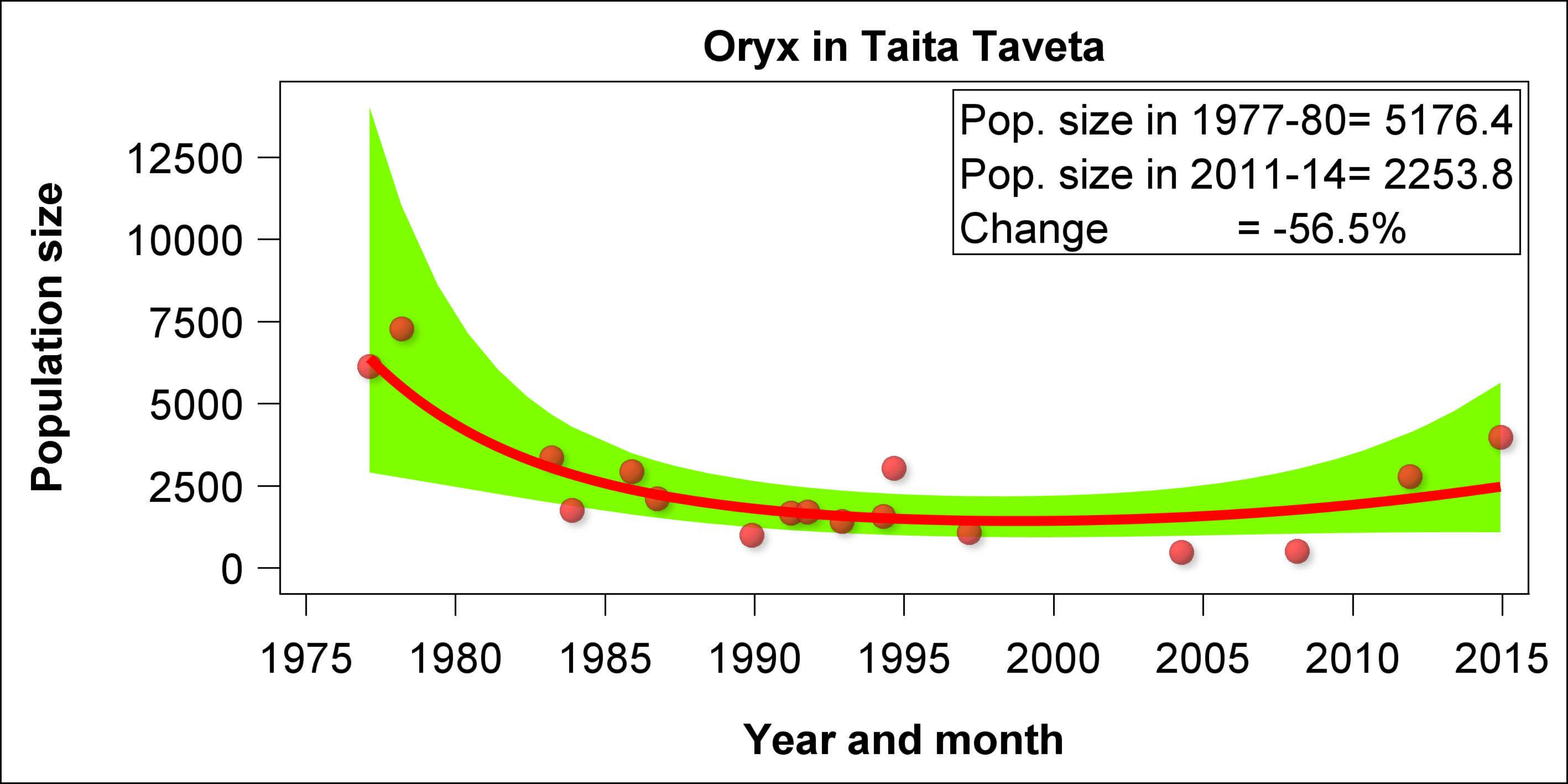

Oryx in Taita Taveta
Pop. size in 1977-80= 5176.4
Pop. size in 2011-14= 2253.8
Change = -56.5%
Population size
12500
10000
7500
5000
2500
0
1975
1980
1985
1990
1995
2000
2005
2010
2015
Year and month

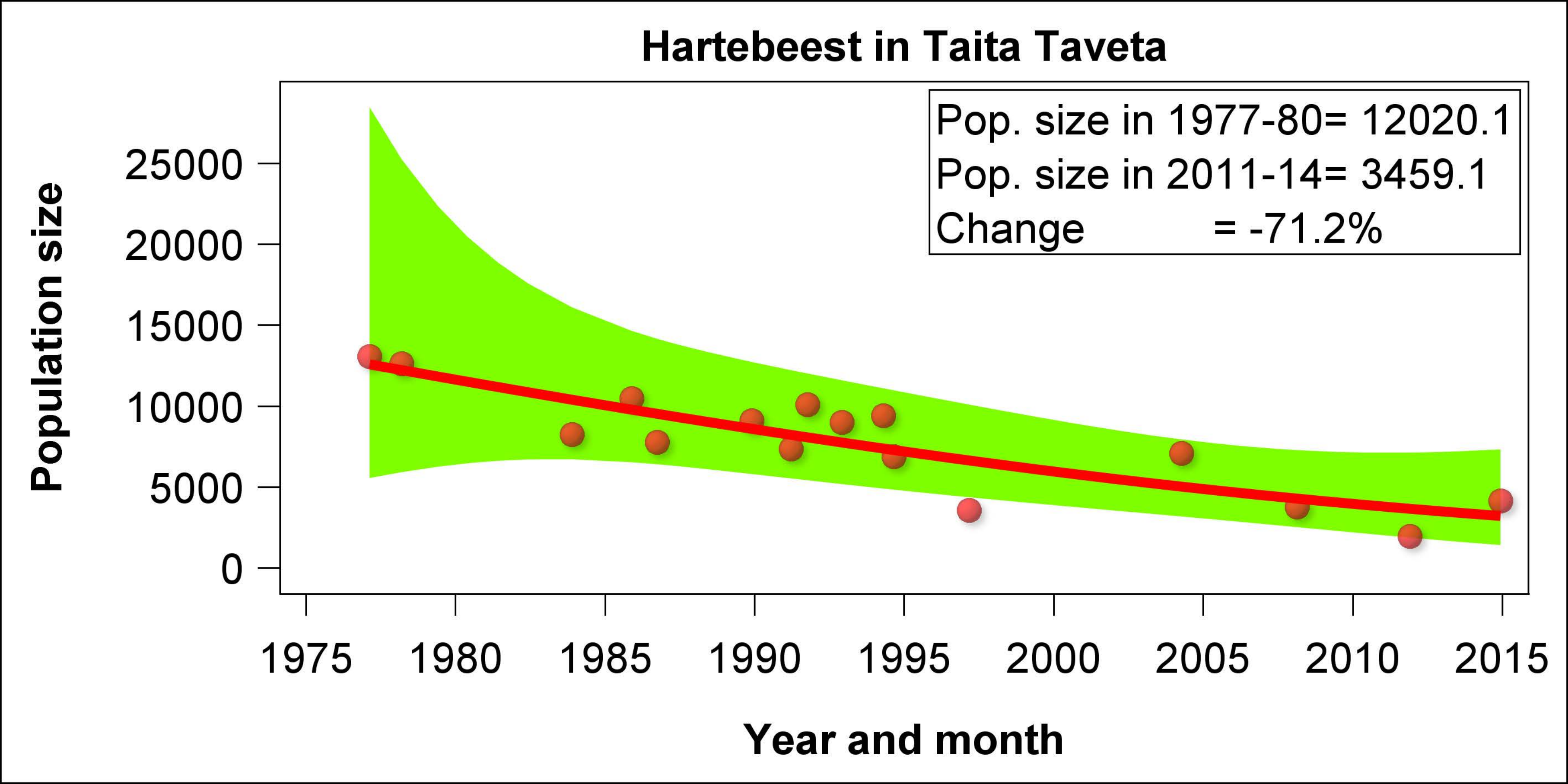

Hartebeest in Taita Taveta
Pop. size in 1977-80= 12020.1
Pop. size in 2011-14= 3459.1
Change = -71.2%
Population size
25000
20000
15000
10000
5000
0
1975
1980
1985
1990
1995
2000
2005
2010
2015
Year and month

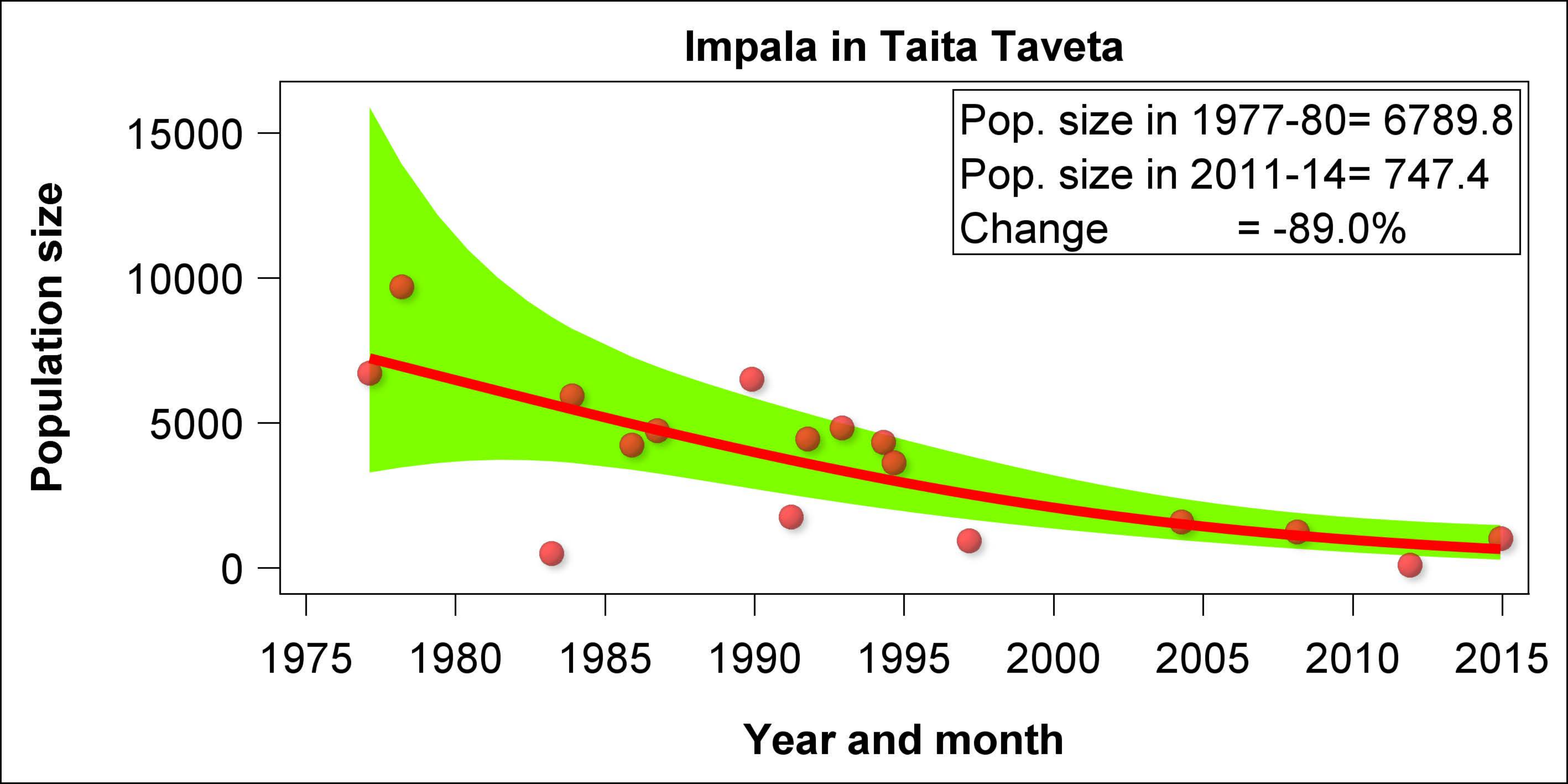

Impala in Taita Taveta
Pop. size in 1977-80= 6789.8
Pop. size in 2011-14= 747.4
Change = -89.0%
Population size
15000
10000
5000
0
1975
1980
1985
1990
1995
2000
2005
2010
2015
Year and month

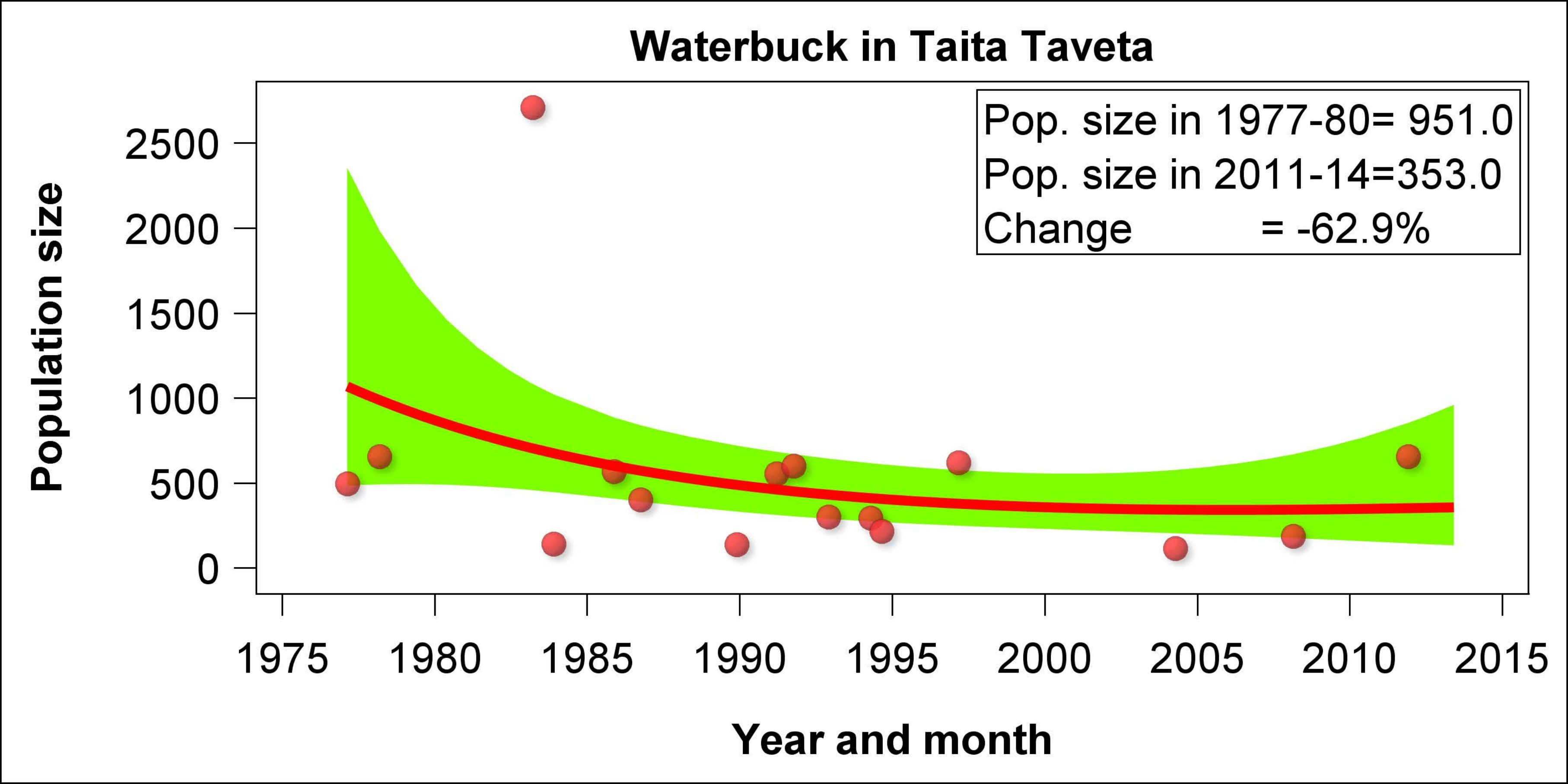
Waterbuck in Taita Taveta
Pop. size in 1977-80= 951.0
Pop. size in 2011-14=353.0
Change = -62.9%
Population size
2500
2000
1500
1000
500
0
1975
1980
1985
1990
1995
2000
2005
2010
2015
Year and month
