# Supplementary material for: Extreme Wildlife Declines and Concurrent Increase in Livestock Numbers in Kenya: What Are the Causes?
Source: PLoS One. 2016 Sep 27;11(9):e0163249. doi: 10.1371/journal.pone.0163249 (PMC5039022; doi:10.1371/journal.pone.0163249)
Supplement: S7 Fig — The solid red line is the fitted trend curve and the shaded chartreuse band is the pointwise 95% confidence band. The estimated average population size in 1977–1980 and 2011–2013 and the percentage change in population size between the two periods are provided in the inset. (PDF) [file pone.0163249.s017.pdf]

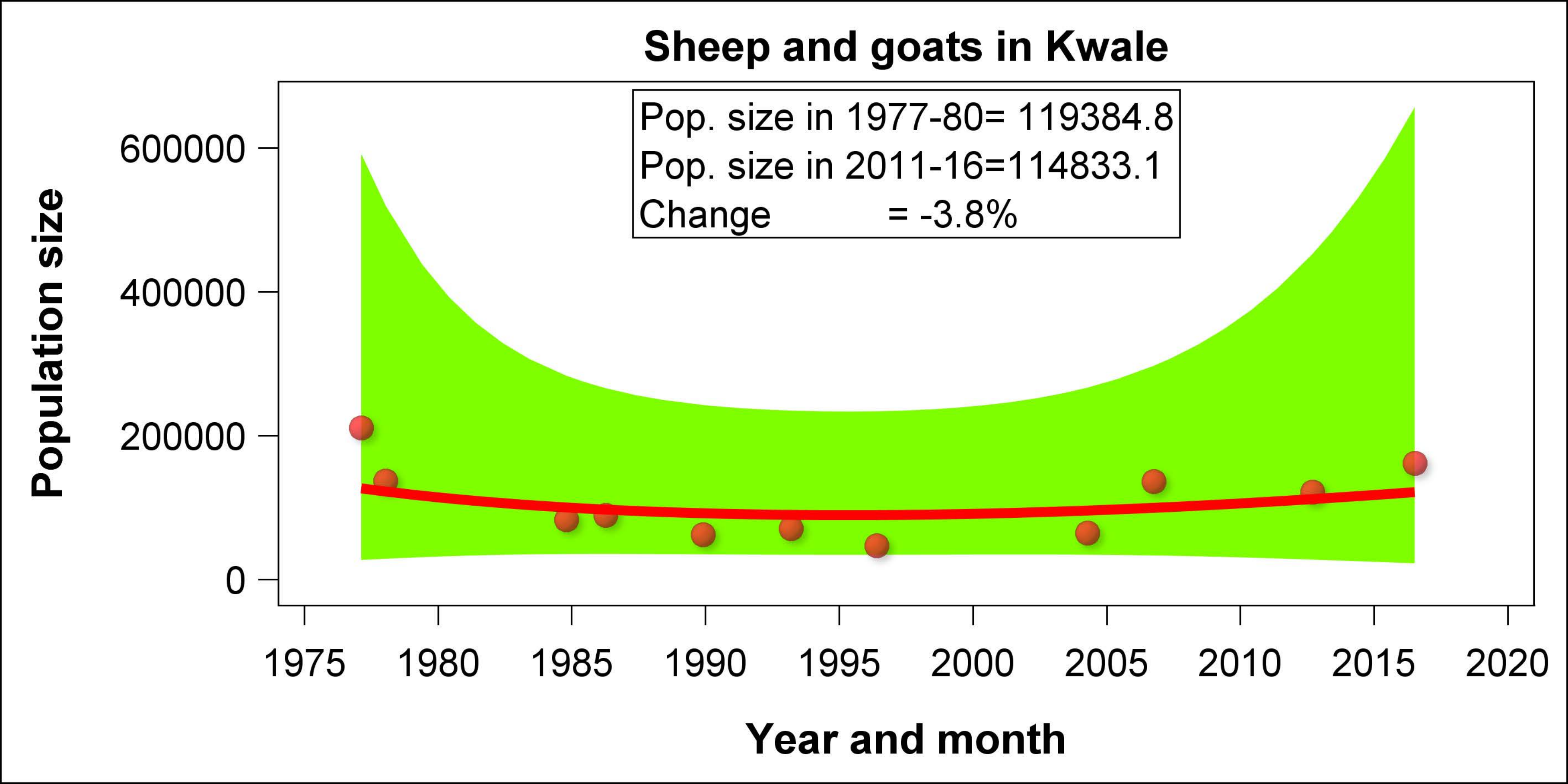
Sheep and goats in Kwale
Pop. size in 1977-80= 119384.8
Pop. size in 2011-16=114833.1
Change = -3.8%
600000
400000
200000
0
Population size
1975
1980
1985
1990
1995
2000
2005
2010
2015
2020
Year and month

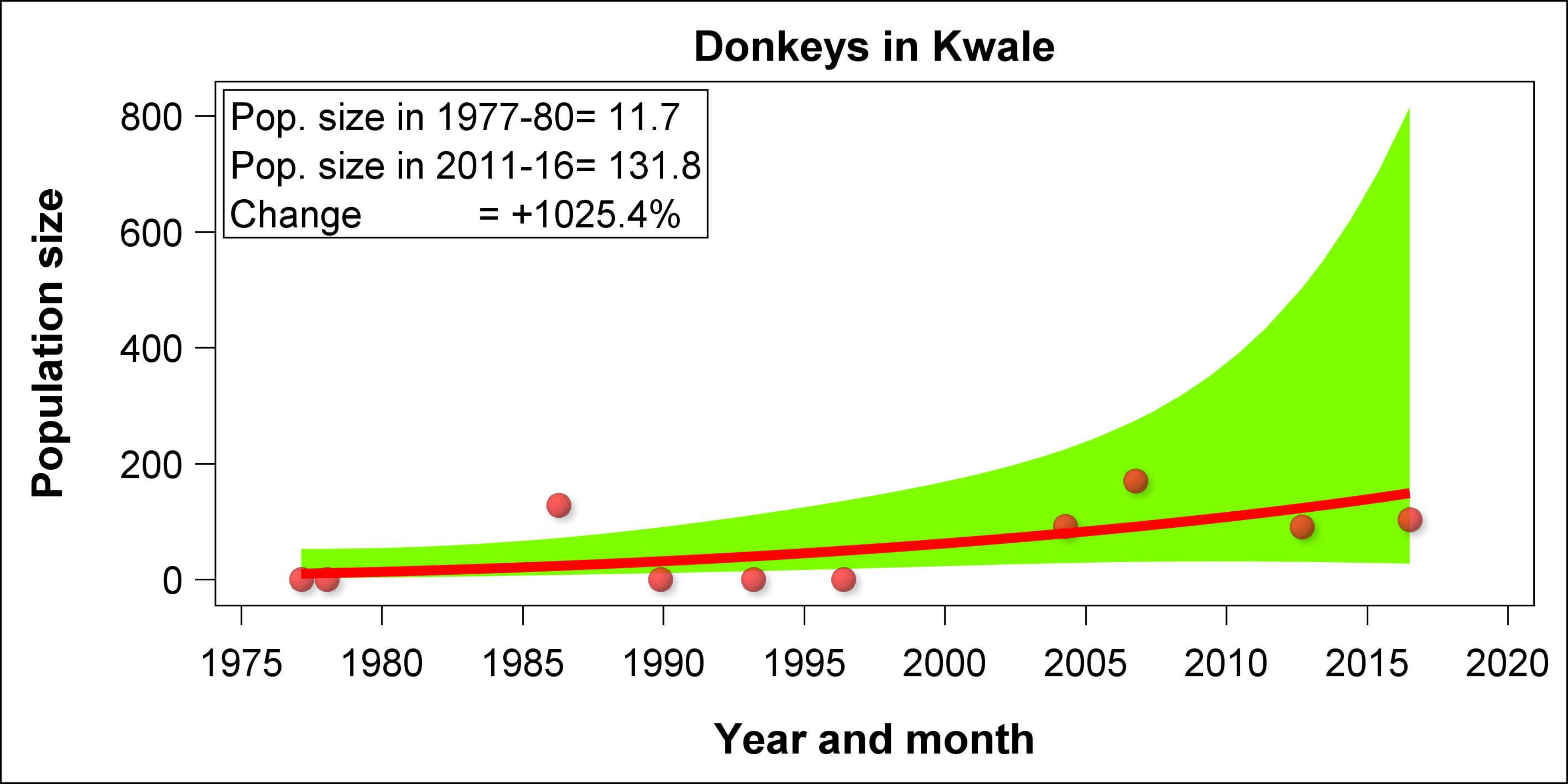

Donkeys in Kwale
Pop. size in 1977-80= 11.7
Pop. size in 2011-16= 131.8
Change = +1025.4%
800
600
400
200
0
Population size
1975
1980
1985
1990
1995
2000
2005
2010
2015
2020
Year and month

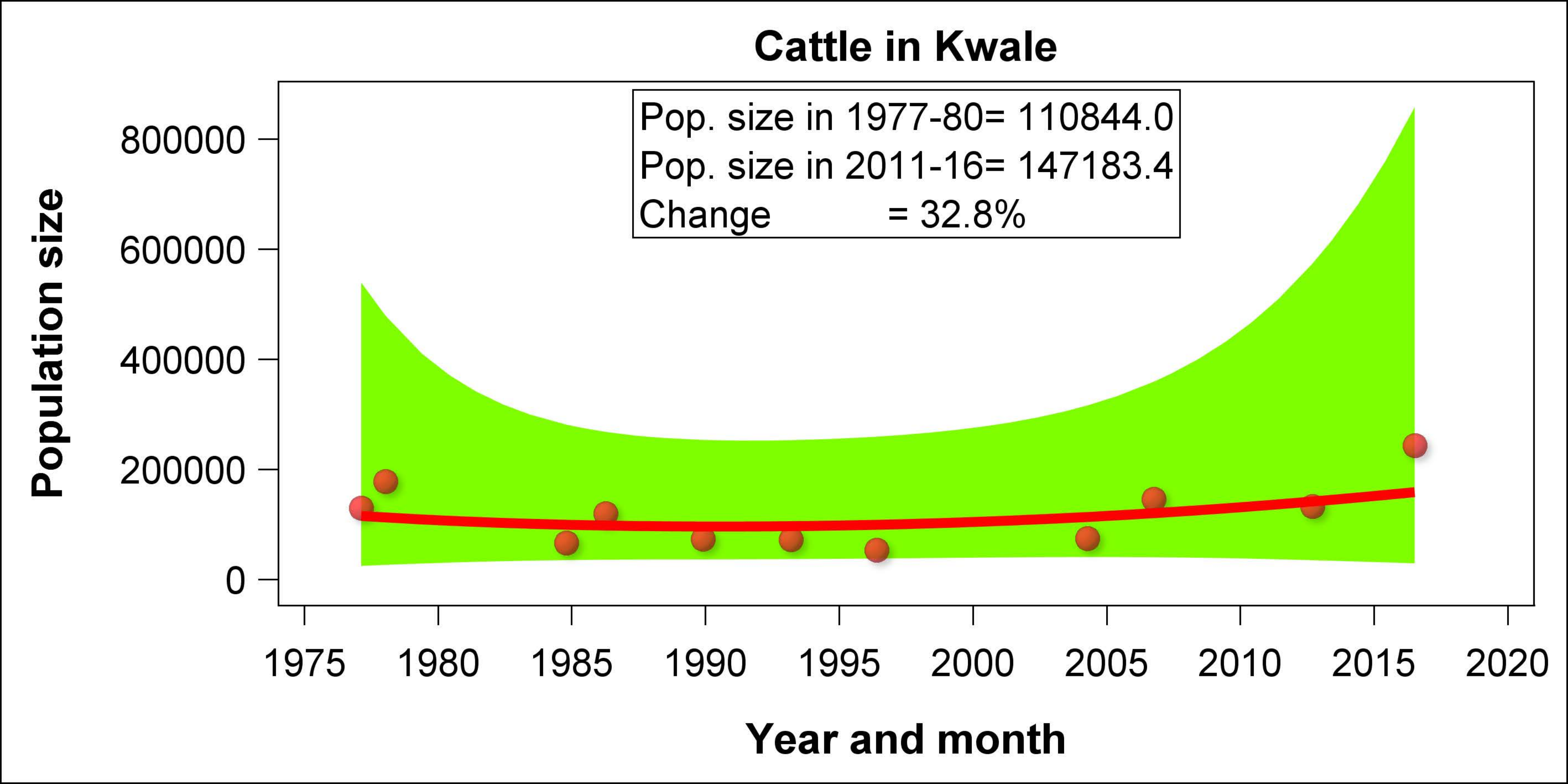

Cattle in Kwale
Pop. size in 1977-80= 110844.0
Pop. size in 2011-16= 147183.4
Change = 32.8%
Population size
800000
600000
400000
200000
0
1975
1980
1985
1990
1995
2000
2005
2010
2015
2020
Year and month

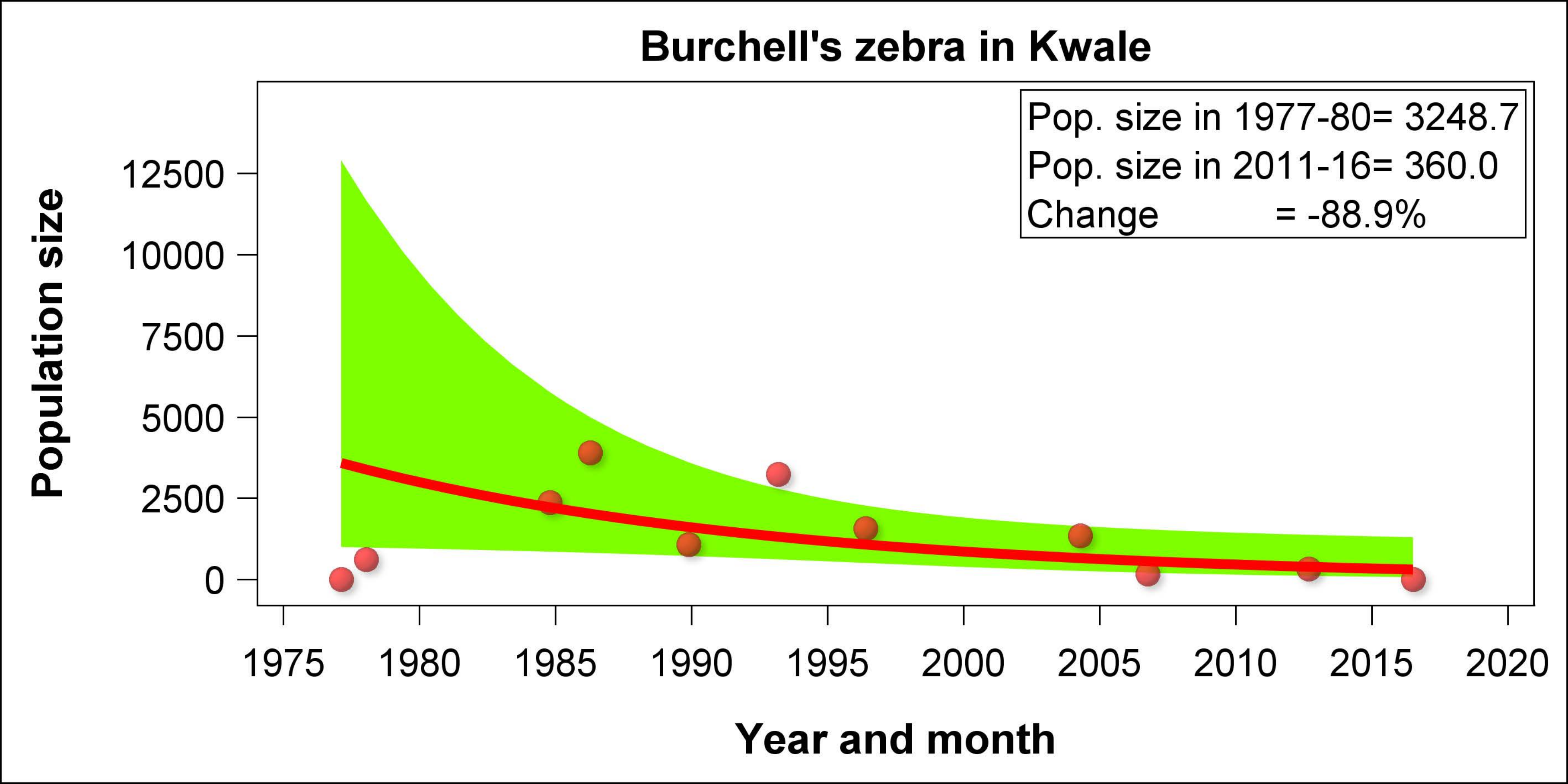
Burchell's zebra in Kwale
Pop. size in 1977-80= 3248.7
Pop. size in 2011-16= 360.0
Change = -88.9%
Population size
12500
10000
7500
5000
2500
0
1975
1980
1985
1990
1995
2000
2005
2010
2015
2020
Year and month

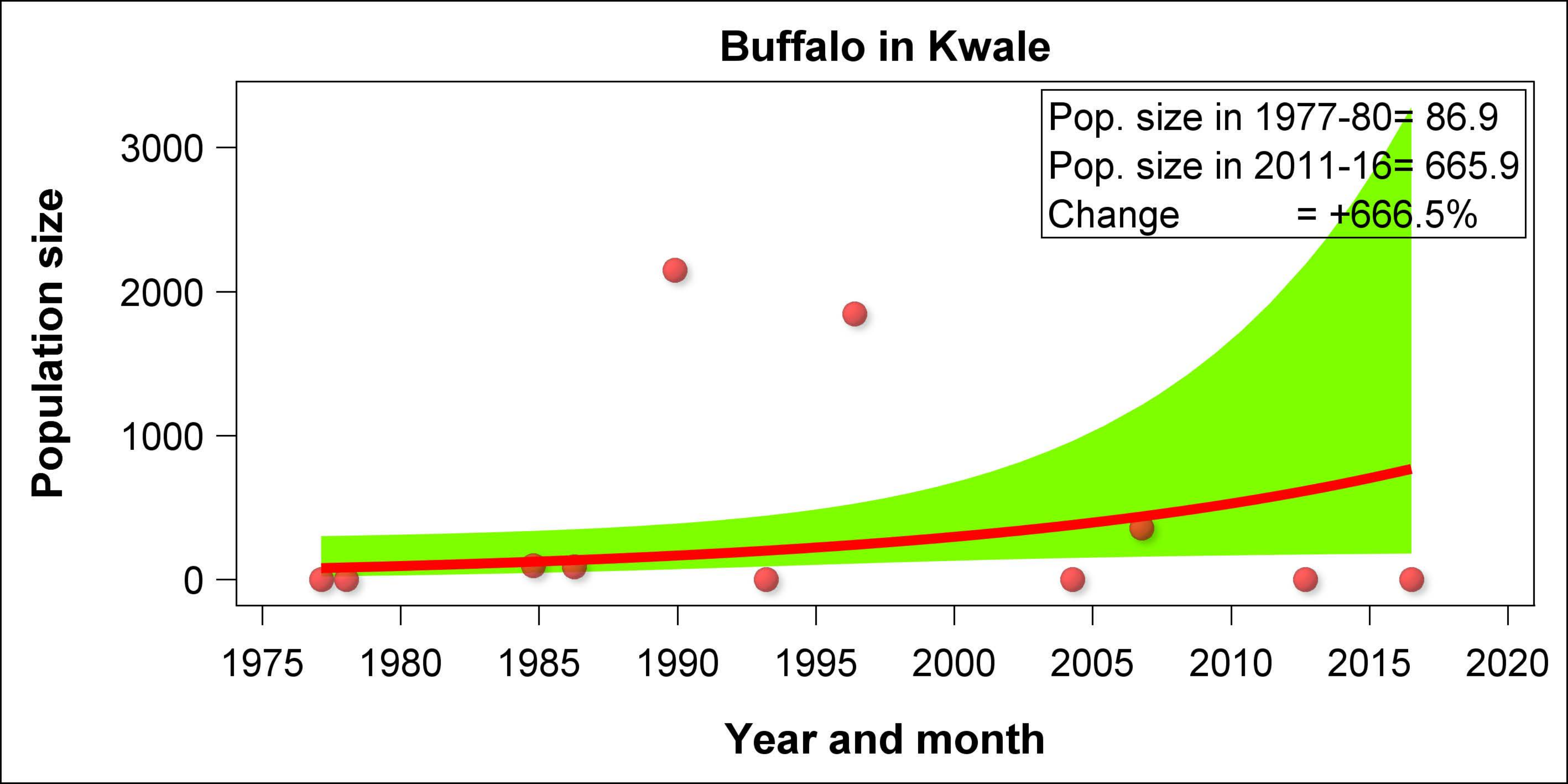

Buffalo in Kwale
Pop. size in 1977-80= 86.9
Pop. size in 2011-16= 665.9
Change = +666.5%
Population size
3000
2000
1000
0
1975
1980
1985
1990
1995
2000
2005
2010
2015
2020
Year and month

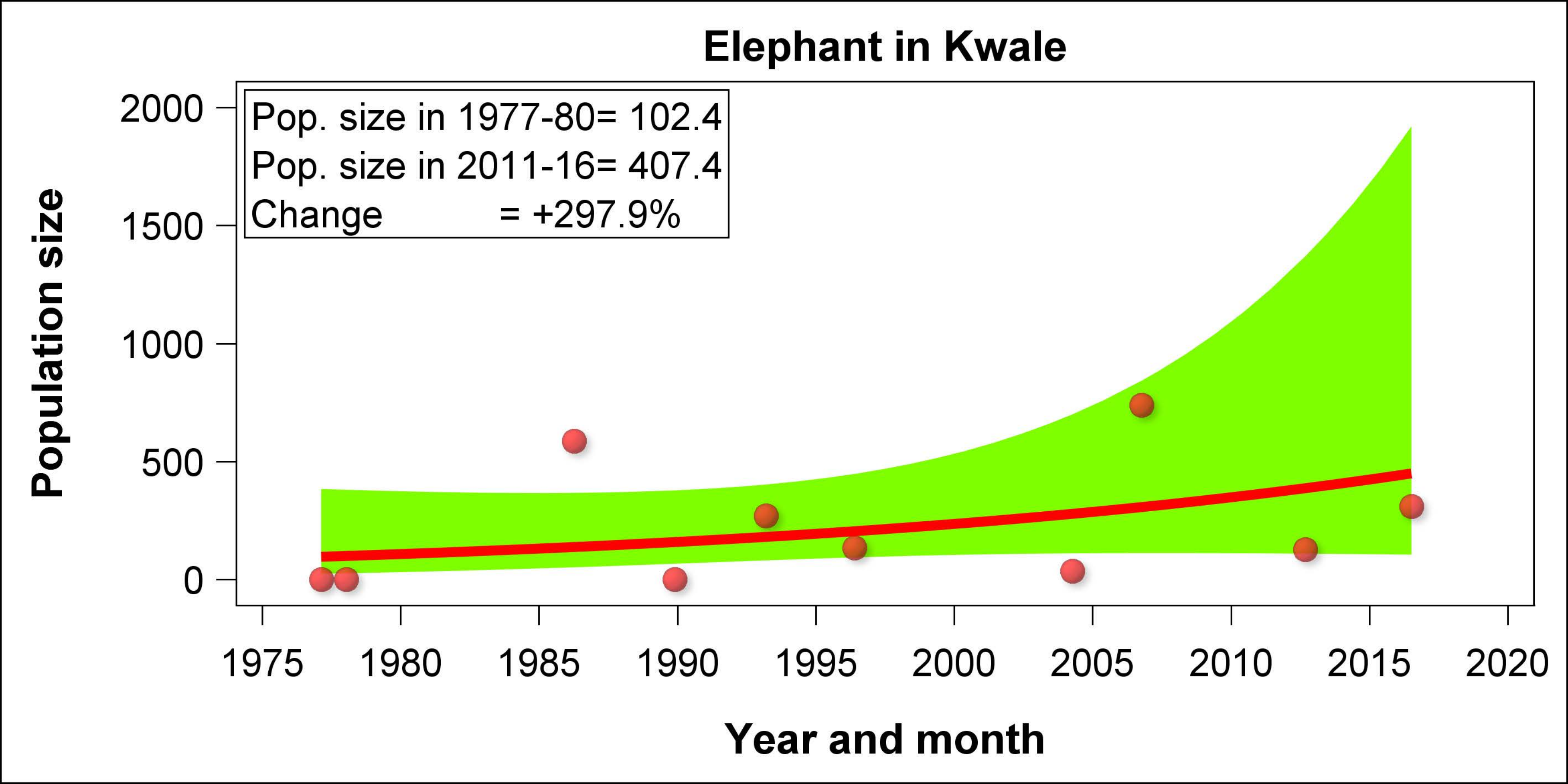

Elephant in Kwale
Pop. size in 1977-80= 102.4
Pop. size in 2011-16= 407.4
Change = +297.9%
2000
1500
1000
500
0
Population size
1975
1980
1985
1990
1995
2000
2005
2010
2015
2020
Year and month

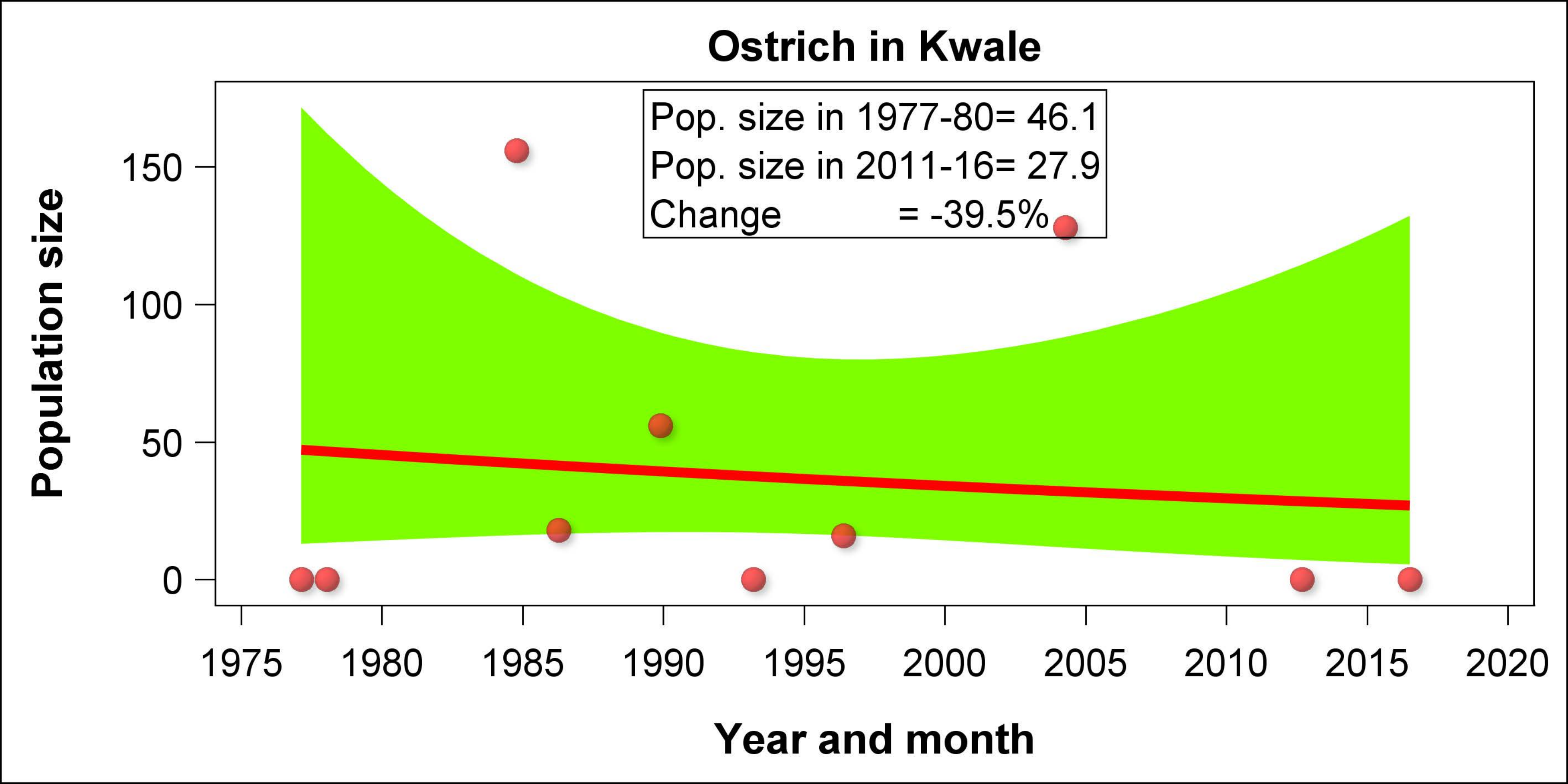
Ostrich in Kwale
Pop. size in 1977-80= 46.1
Pop. size in 2011-16= 27.9
Change = -39.5%
Population size
0
50
100
150
1975
1980
1985
1990
1995
2000
2005
2010
2015
2020
Year and month

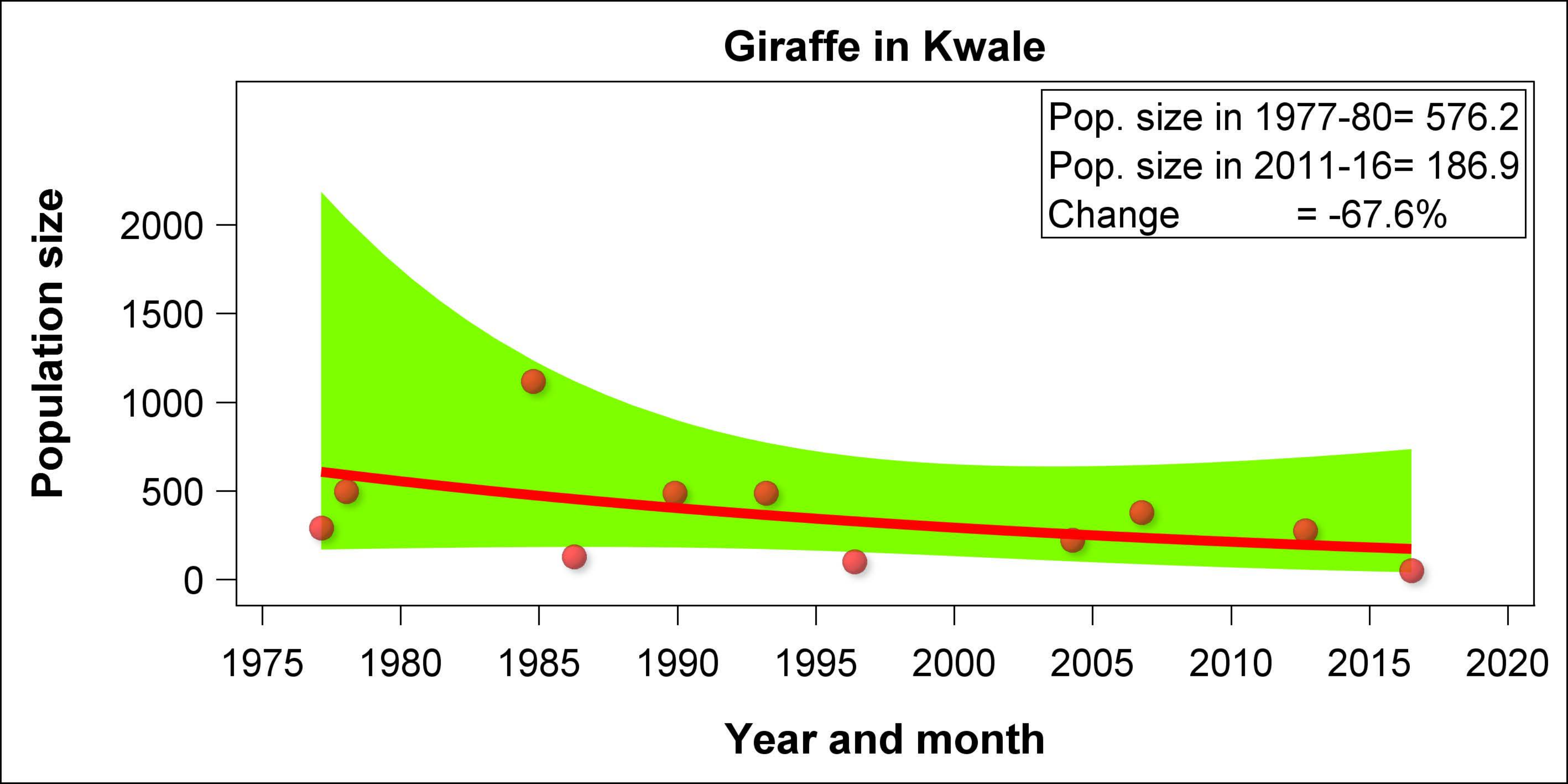

Giraffe in Kwale
Pop. size in 1977-80= 576.2
Pop. size in 2011-16= 186.9
Change = -67.6%
Population size
2000
1500
1000
500
0
1975
1980
1985
1990
1995
2000
2005
2010
2015
2020
Year and month

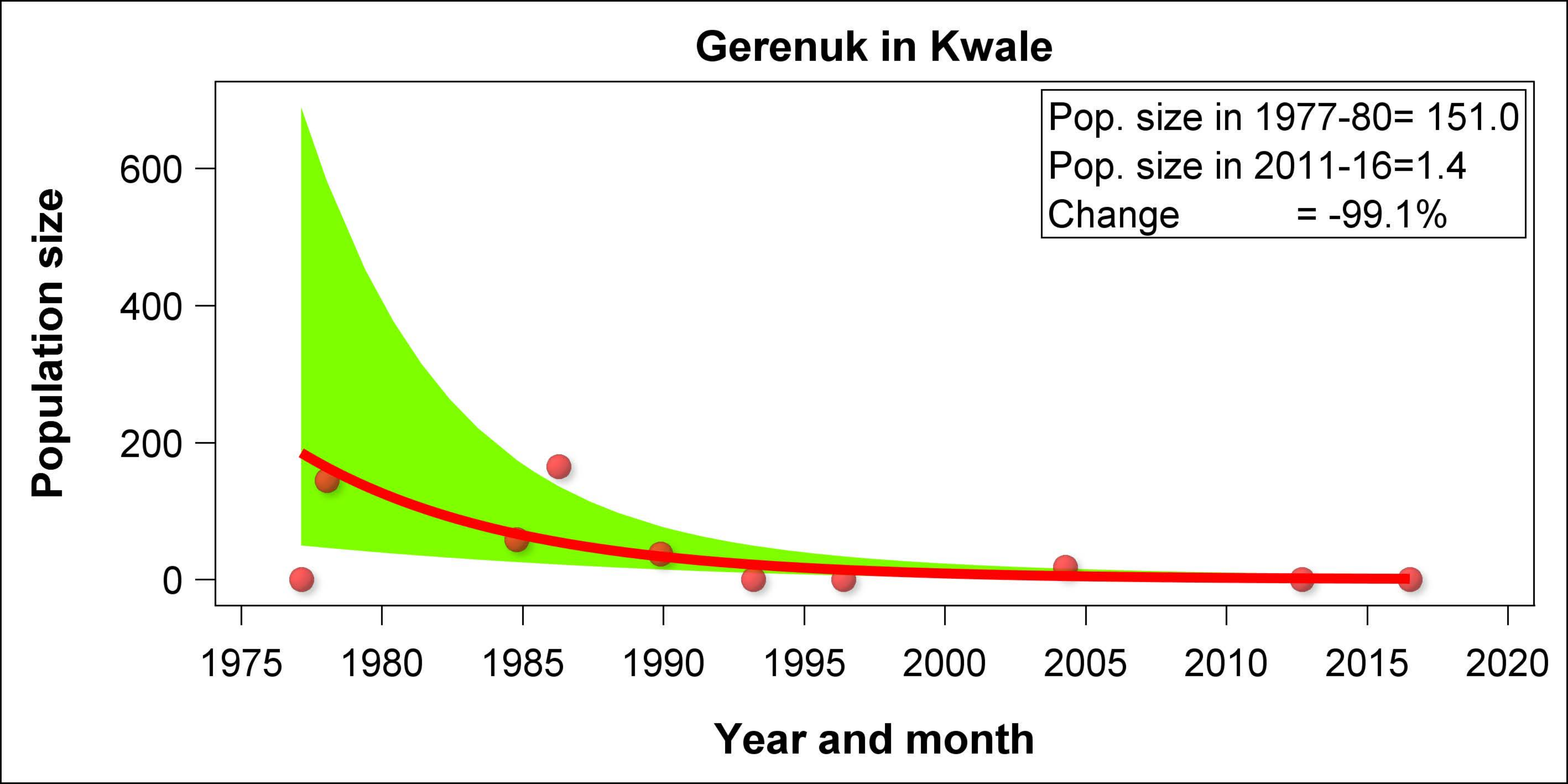
Gerenuk in Kwale
Pop. size in 1977-80= 151.0
Pop. size in 2011-16=1.4
Change = -99.1%
Population size
600
400
200
0
1975
1980
1985
1990
1995
2000
2005
2010
2015
2020
Year and month

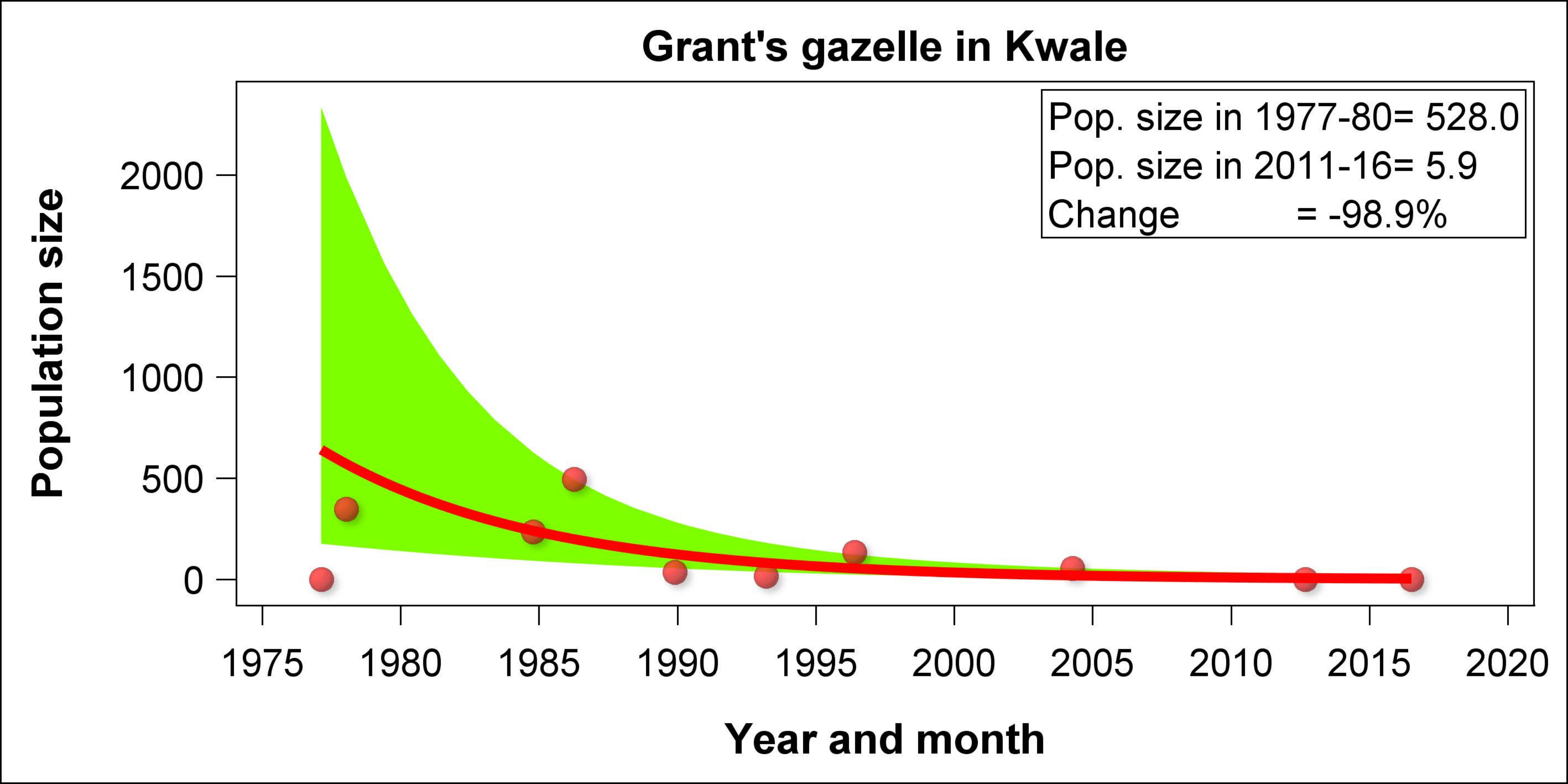
Grant's gazelle in Kwale
Pop. size in 1977-80= 528.0
Pop. size in 2011-16= 5.9
Change = -98.9%
Population size
2000
1500
1000
500
0
1975
1980
1985
1990
1995
2000
2005
2010
2015
2020
Year and month

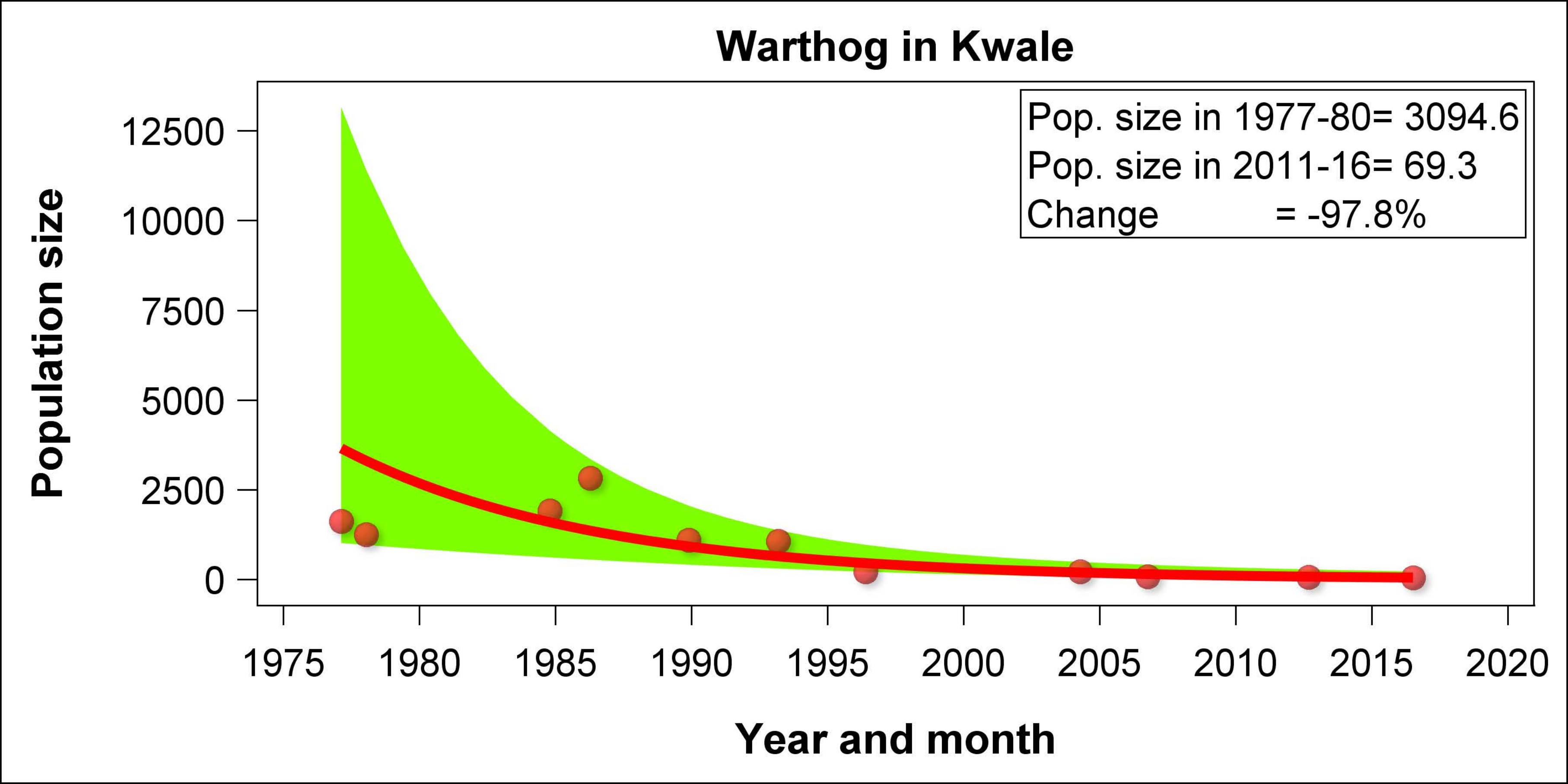
Warthog in Kwale
Pop. size in 1977-80= 3094.6
Pop. size in 2011-16= 69.3
Change = -97.8%
Population size
12500
10000
7500
5000
2500
0
1975
1980
1985
1990
1995
2000
2005
2010
2015
2020
Year and month

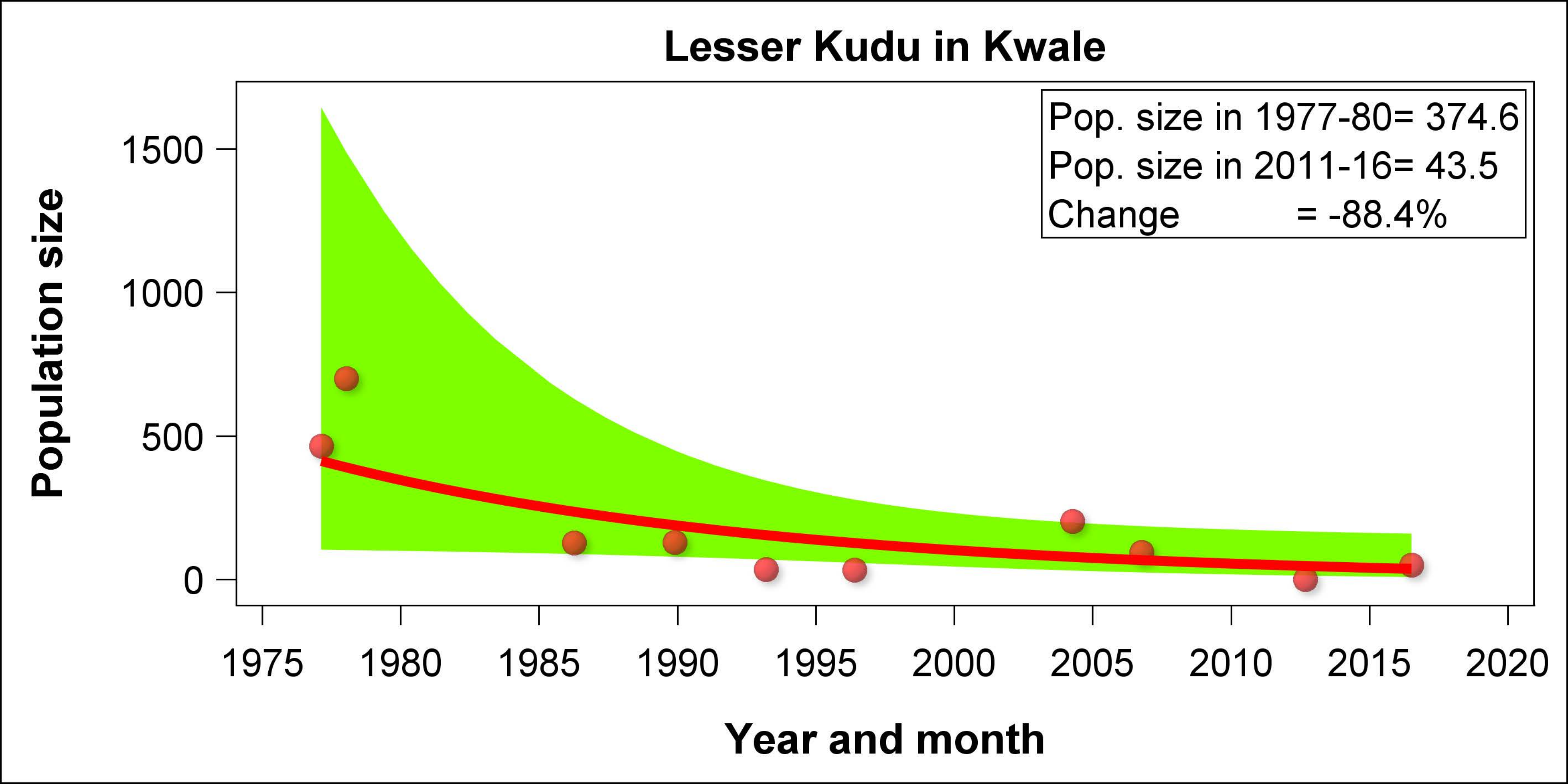
Lesser Kudu in Kwale
Pop. size in 1977-80= 374.6
Pop. size in 2011-16= 43.5
Change = -88.4%
Population size
1500
1000
500
0
1975
1980
1985
1990
1995
2000
2005
2010
2015
2020
Year and month

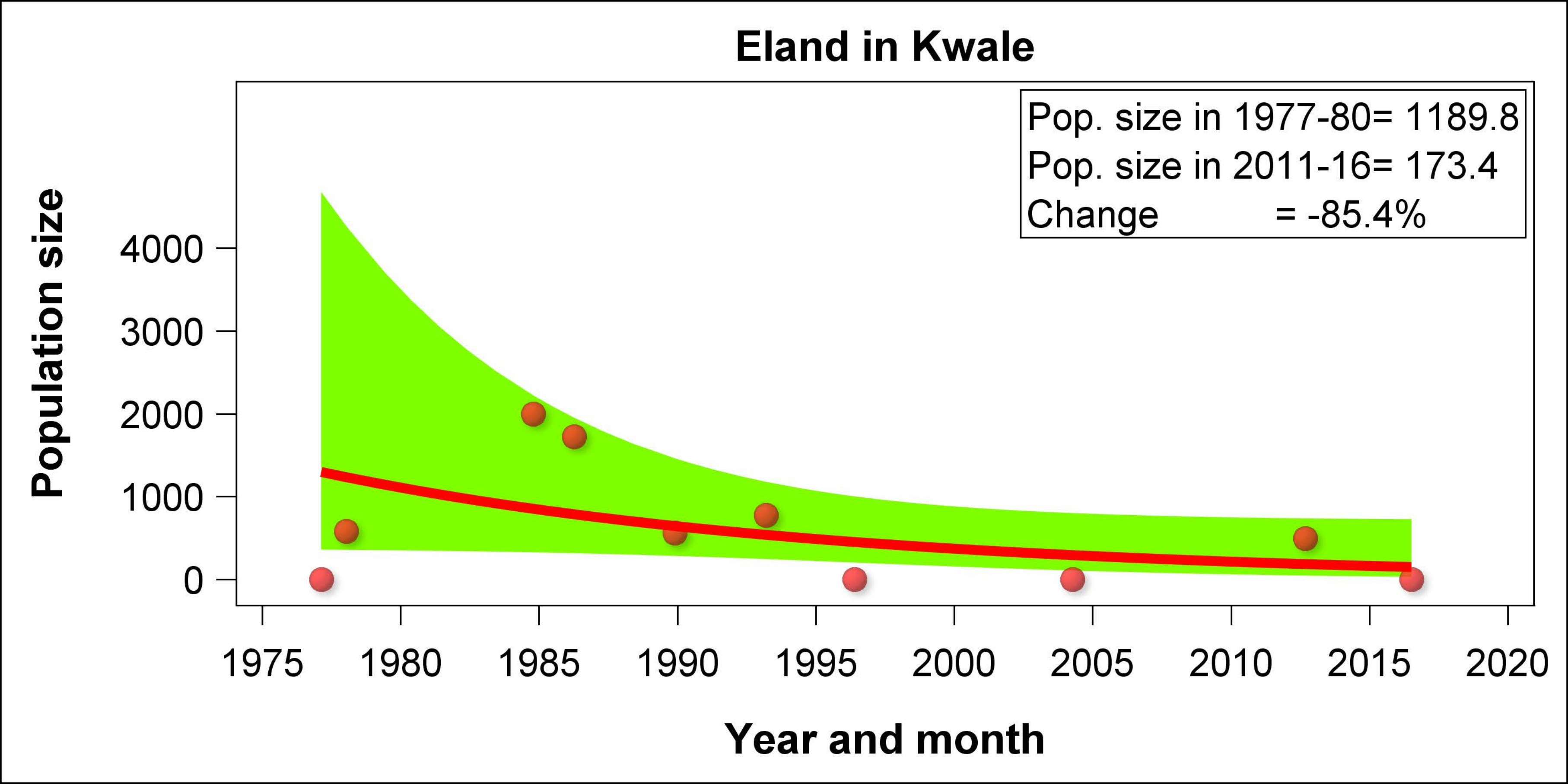

Eland in Kwale
Pop. size in 1977-80= 1189.8
Pop. size in 2011-16= 173.4
Change = -85.4%
Population size
4000
3000
2000
1000
0
1975
1980
1985
1990
1995
2000
2005
2010
2015
2020
Year and month

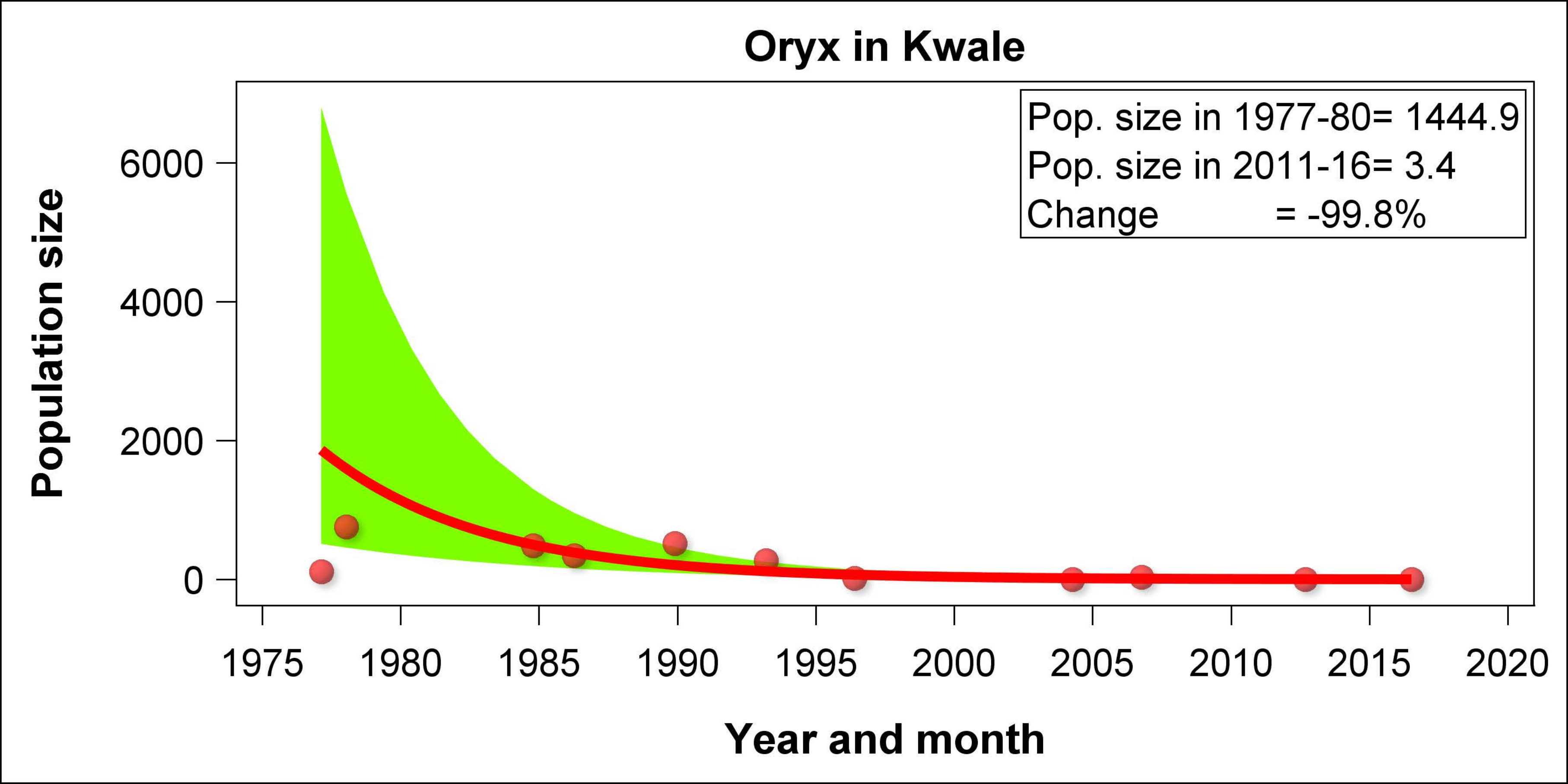
Oryx in Kwale
Pop. size in 1977-80= 1444.9
Pop. size in 2011-16= 3.4
Change = -99.8%
Population size
6000
4000
2000
0
1975
1980
1985
1990
1995
2000
2005
2010
2015
2020
Year and month

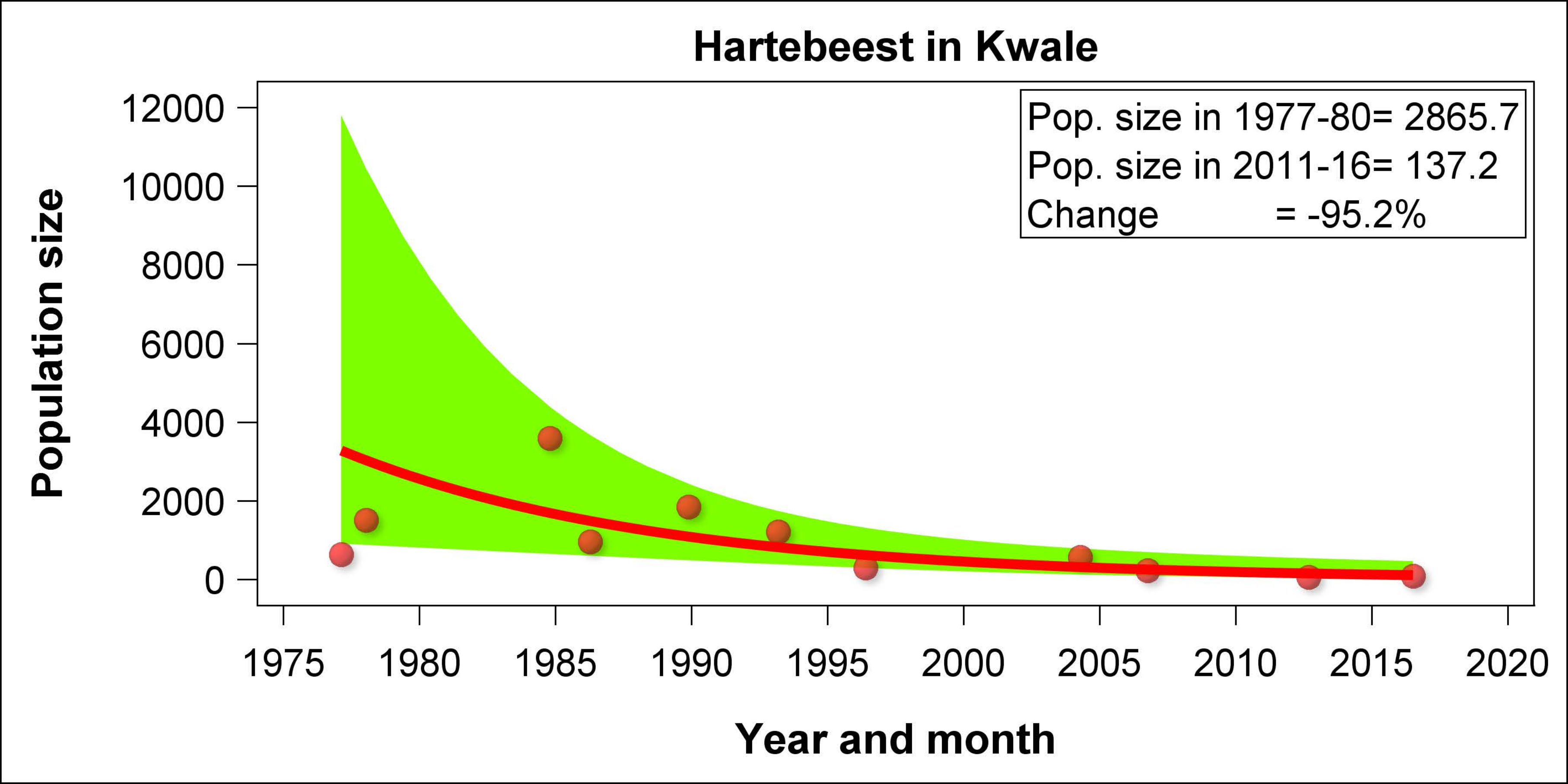
Hartebeest in Kwale
Pop. size in 1977-80= 2865.7
Pop. size in 2011-16= 137.2
Change = -95.2%
Population size
12000
10000
8000
6000
4000
2000
0
1975
1980
1985
1990
1995
2000
2005
2010
2015
2020
Year and month

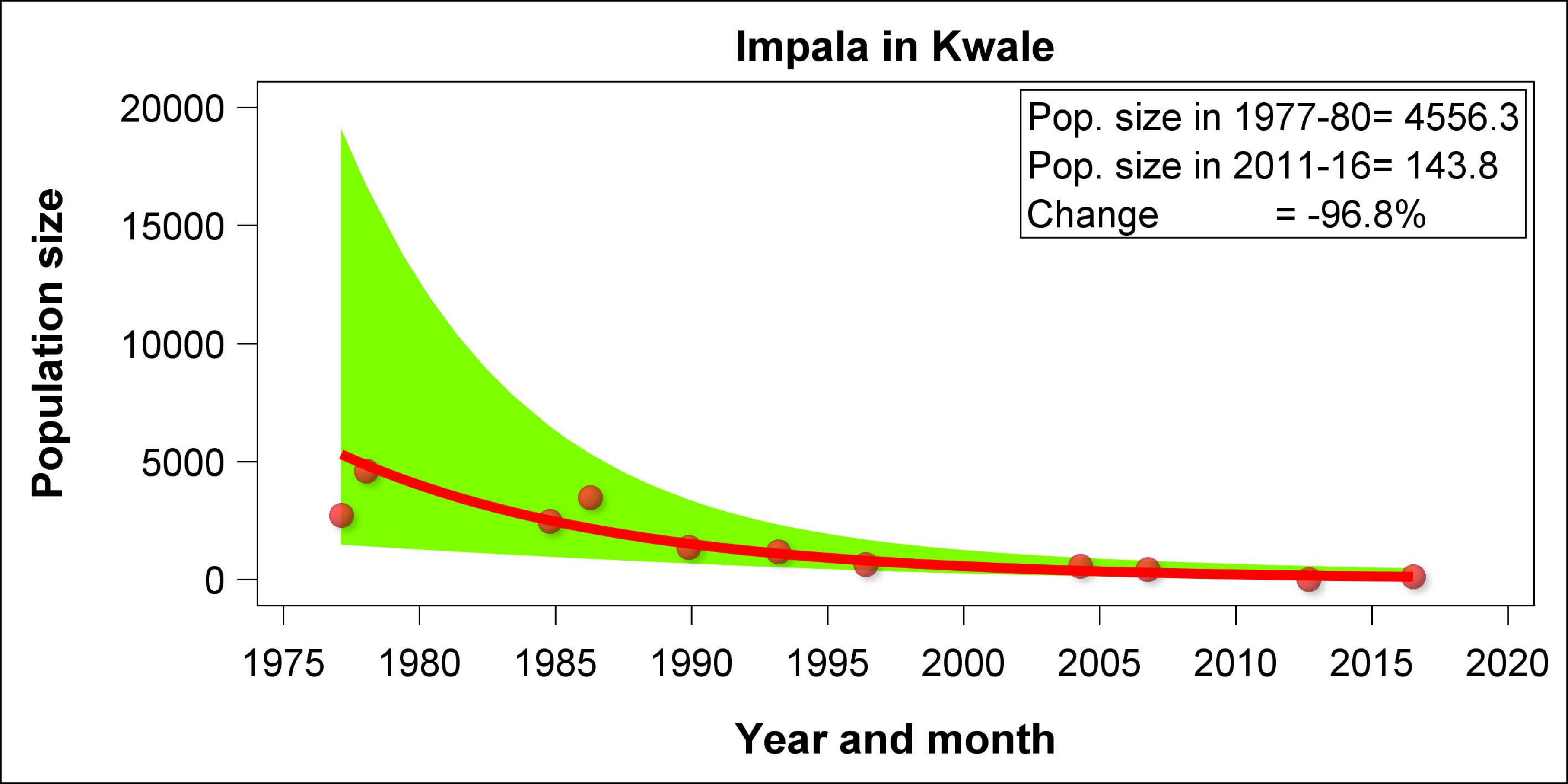

Impala in Kwale
Pop. size in 1977-80= 4556.3
Pop. size in 2011-16= 143.8
Change = -96.8%
Population size
20000
15000
10000
5000
0
1975
1980
1985
1990
1995
2000
2005
2010
2015
2020
Year and month
