# Supplementary material for: Extreme Wildlife Declines and Concurrent Increase in Livestock Numbers in Kenya: What Are the Causes?
Source: PLoS One. 2016 Sep 27;11(9):e0163249. doi: 10.1371/journal.pone.0163249 (PMC5039022; doi:10.1371/journal.pone.0163249)
Supplement: S8 Fig — The solid red line is the fitted trend curve and the shaded chartreuse band is the pointwise 95% confidence band. The estimated average population size in 1977–1980 and 2011–2013 and the percentage change in population size between the two periods are provided in the inset. (PDF) [file pone.0163249.s018.pdf]

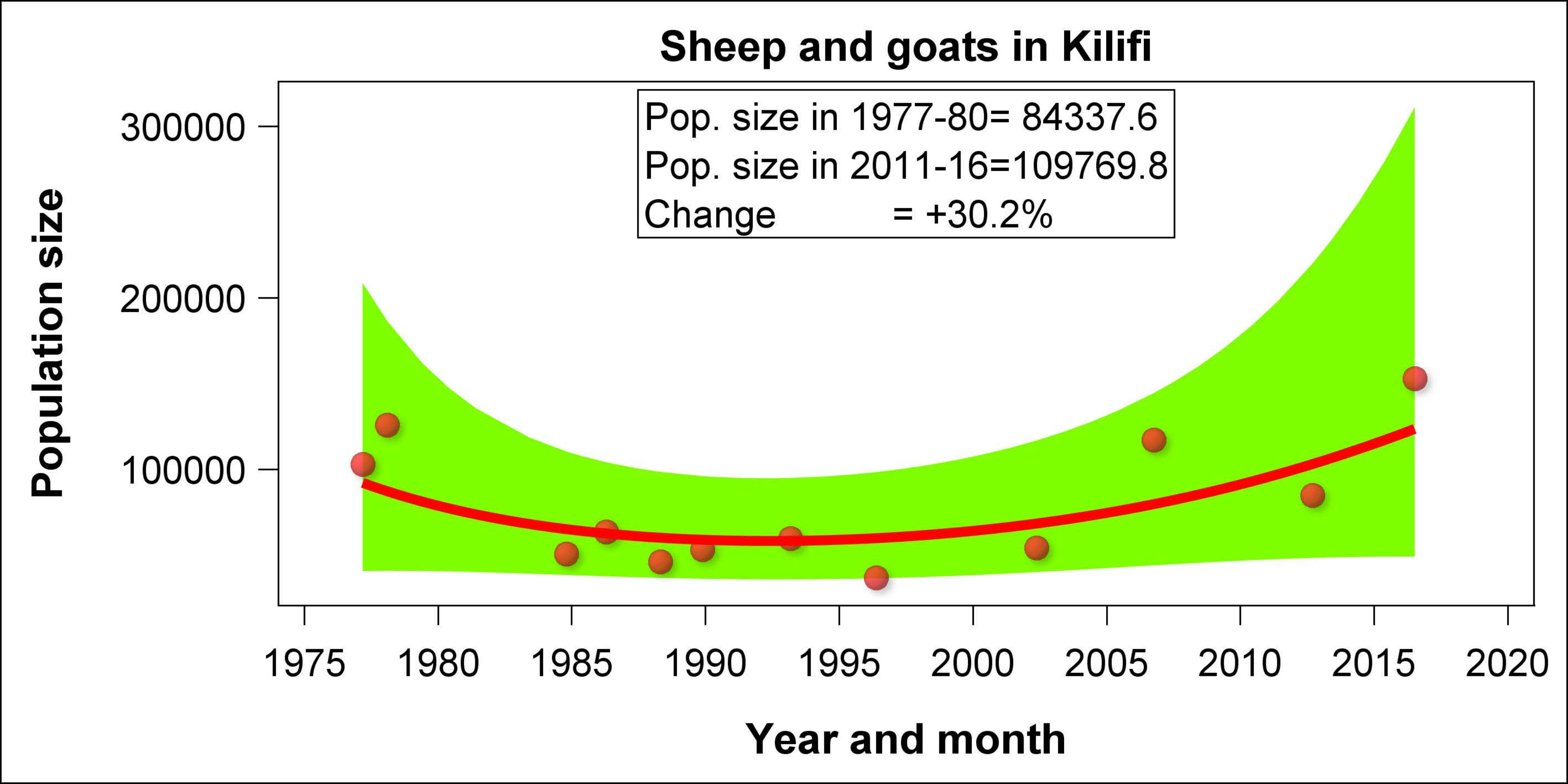

Sheep and goats in Kilifi
Pop. size in 1977-80= 84337.6
Pop. size in 2011-16=109769.8
Change = +30.2%
Population size
300000
200000
100000
1975
1980
1985
1990
1995
2000
2005
2010
2015
2020
Year and month

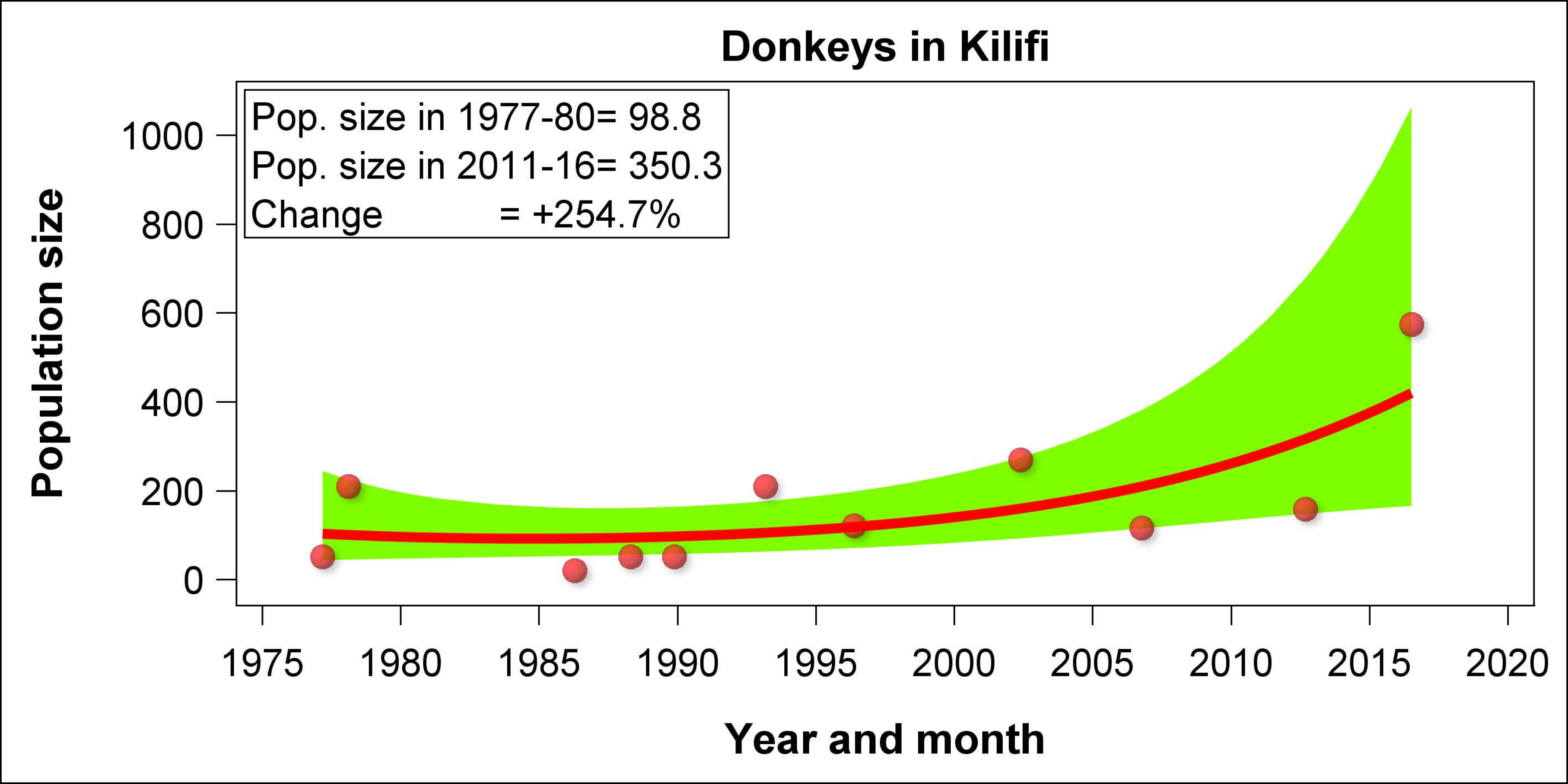
Donkeys in Kilifi
Pop. size in 1977-80= 98.8
Pop. size in 2011-16= 350.3
Change = +254.7%
Population size
0
200
400
600
800
1000
1975
1980
1985
1990
1995
2000
2005
2010
2015
2020
Year and month

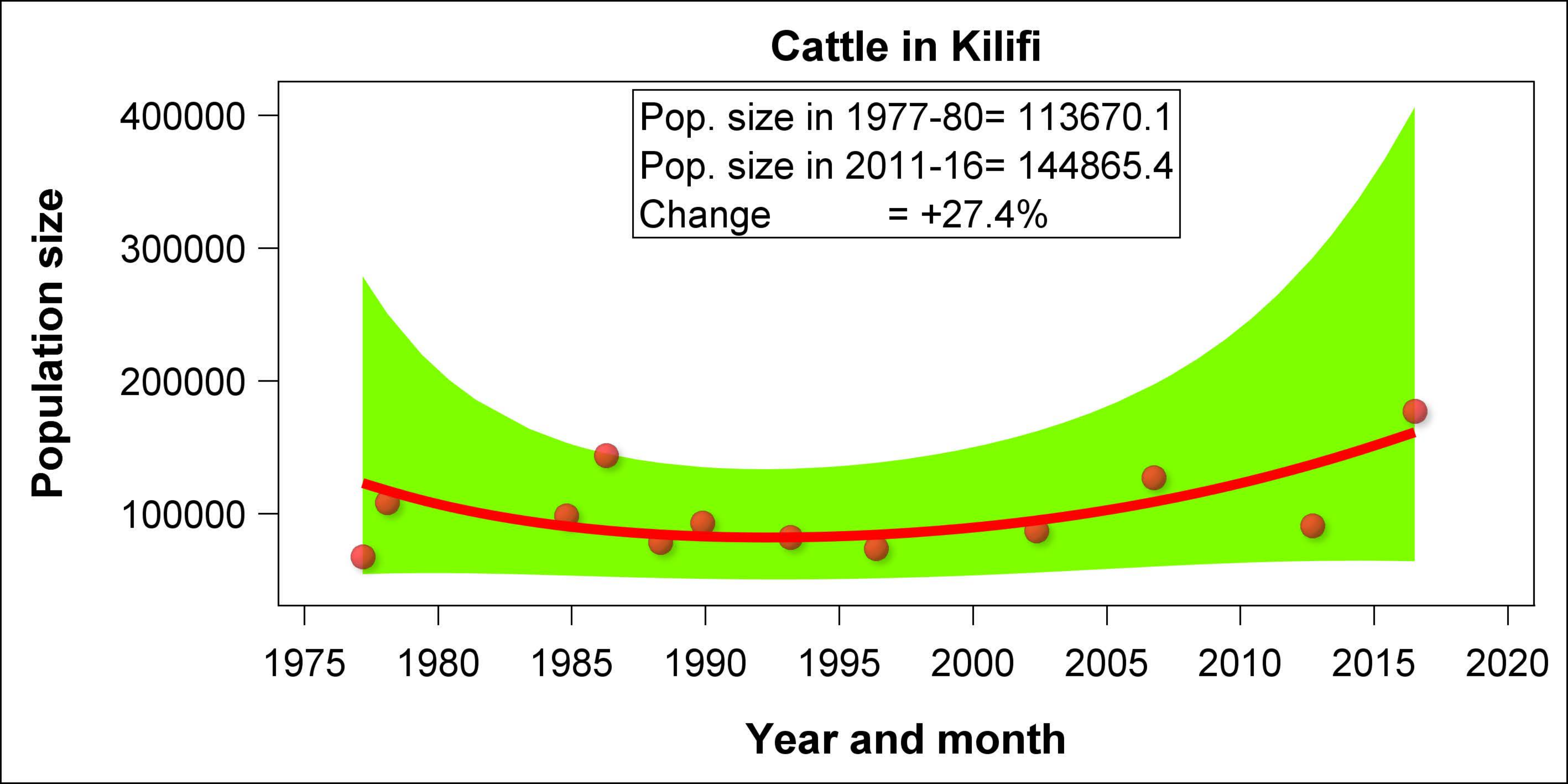

Cattle in Kilifi
Pop. size in 1977-80= 113670.1
Pop. size in 2011-16= 144865.4
Change = +27.4%
400000
300000
200000
100000
Population size
1975
1980
1985
1990
1995
2000
2005
2010
2015
2020
Year and month

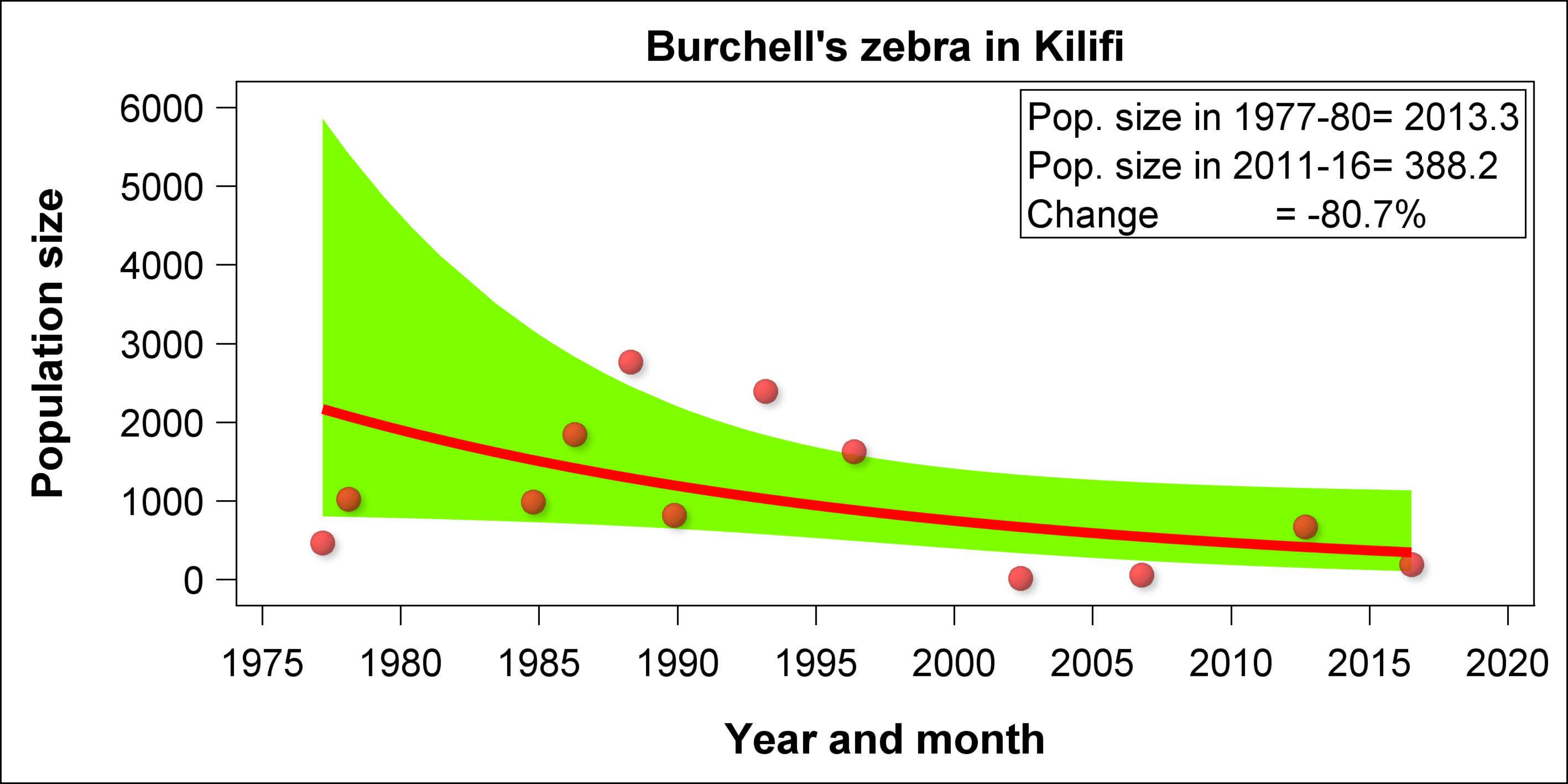
Burchell's zebra in Kilifi
Pop. size in 1977-80= 2013.3
Pop. size in 2011-16= 388.2
Change = -80.7%
Population size
6000
5000
4000
3000
2000
1000
0
1975
1980
1985
1990
1995
2000
2005
2010
2015
2020
Year and month

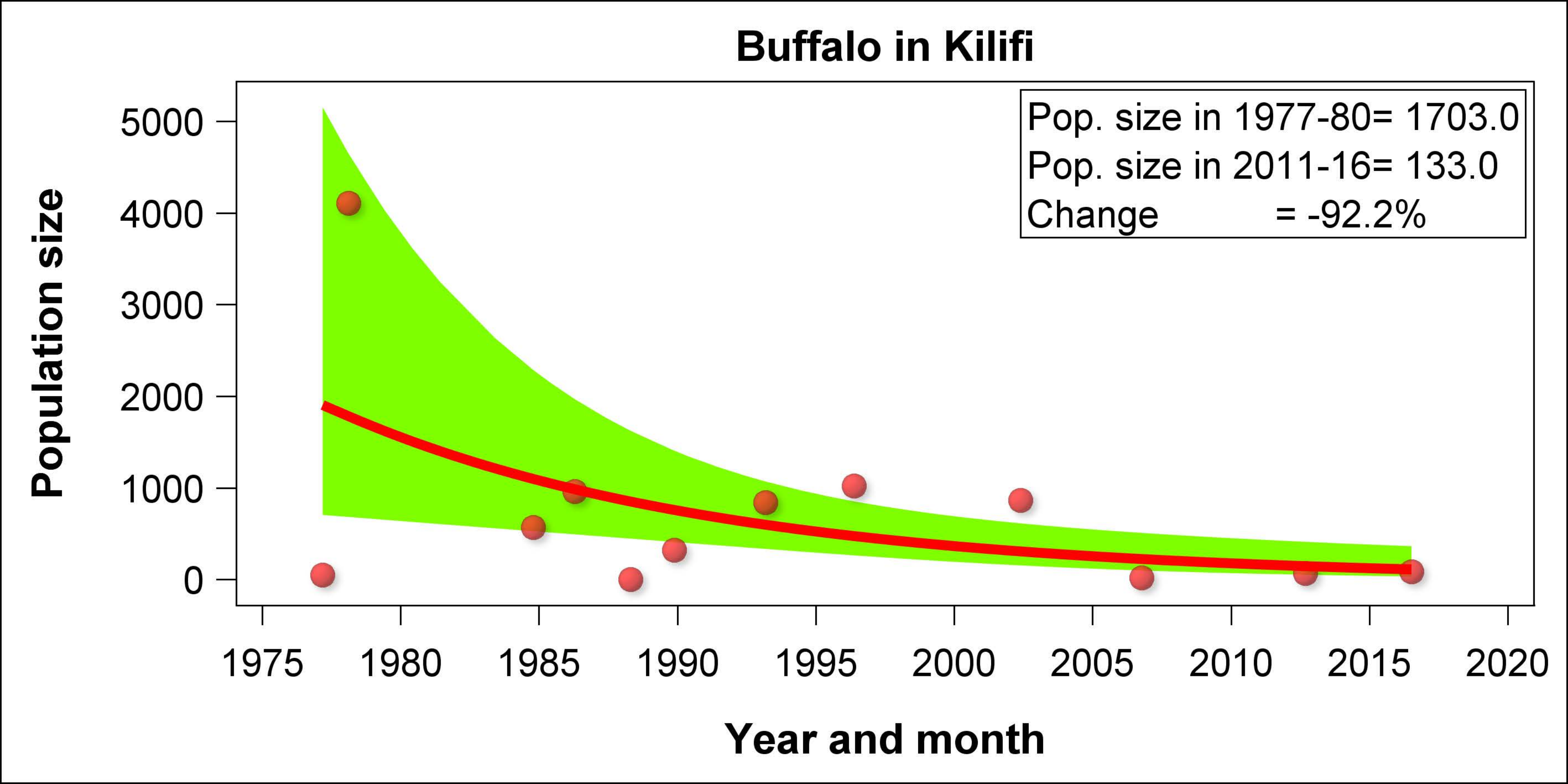

Buffalo in Kilifi
Pop. size in 1977-80= 1703.0
Pop. size in 2011-16= 133.0
Change = -92.2%
Population size
5000
4000
3000
2000
1000
0
1975
1980
1985
1990
1995
2000
2005
2010
2015
2020
Year and month

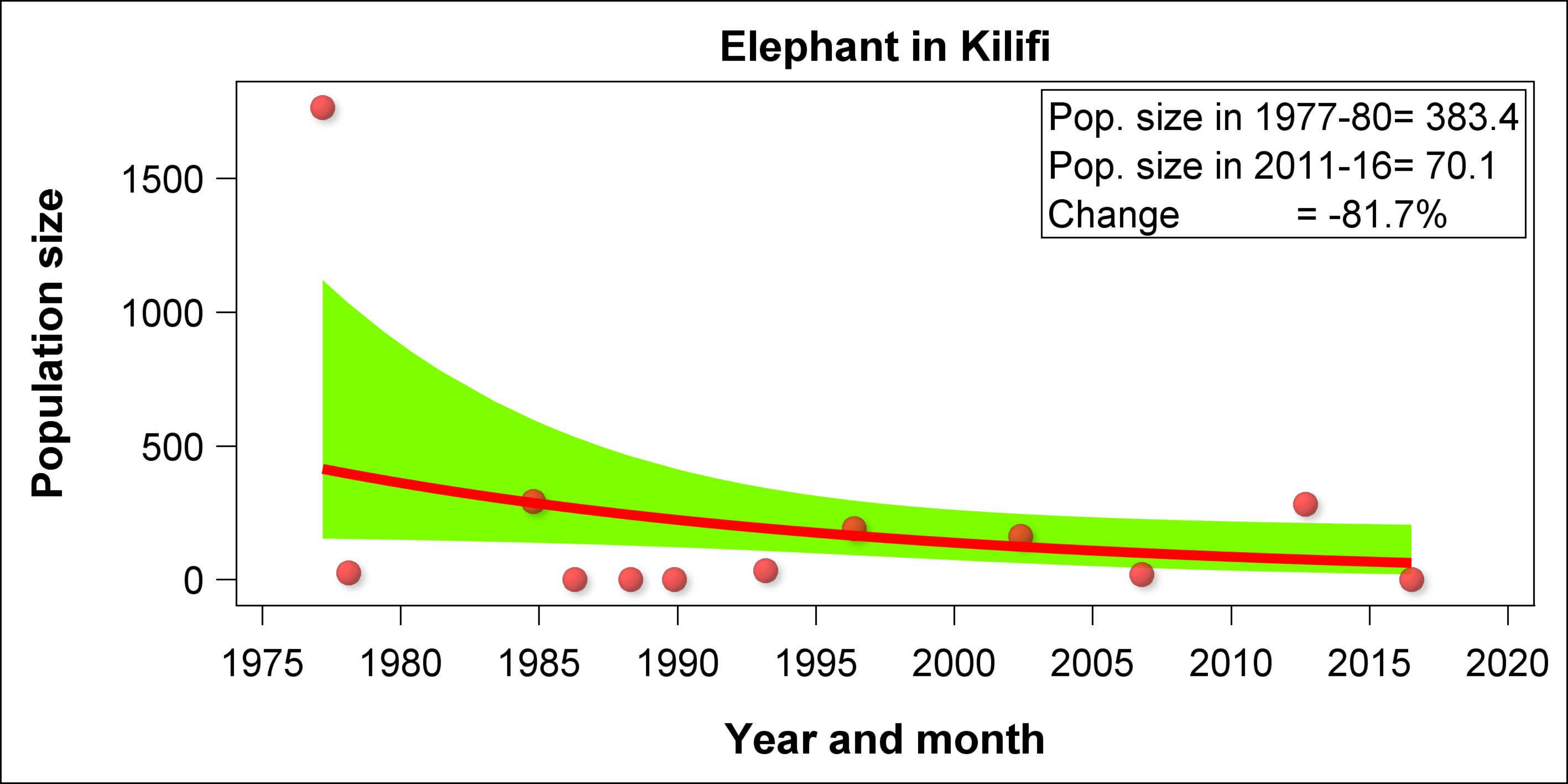

Elephant in Kilifi
Pop. size in 1977-80= 383.4
Pop. size in 2011-16= 70.1
Change = -81.7%
Population size
1500
1000
500
0
1975
1980
1985
1990
1995
2000
2005
2010
2015
2020
Year and month

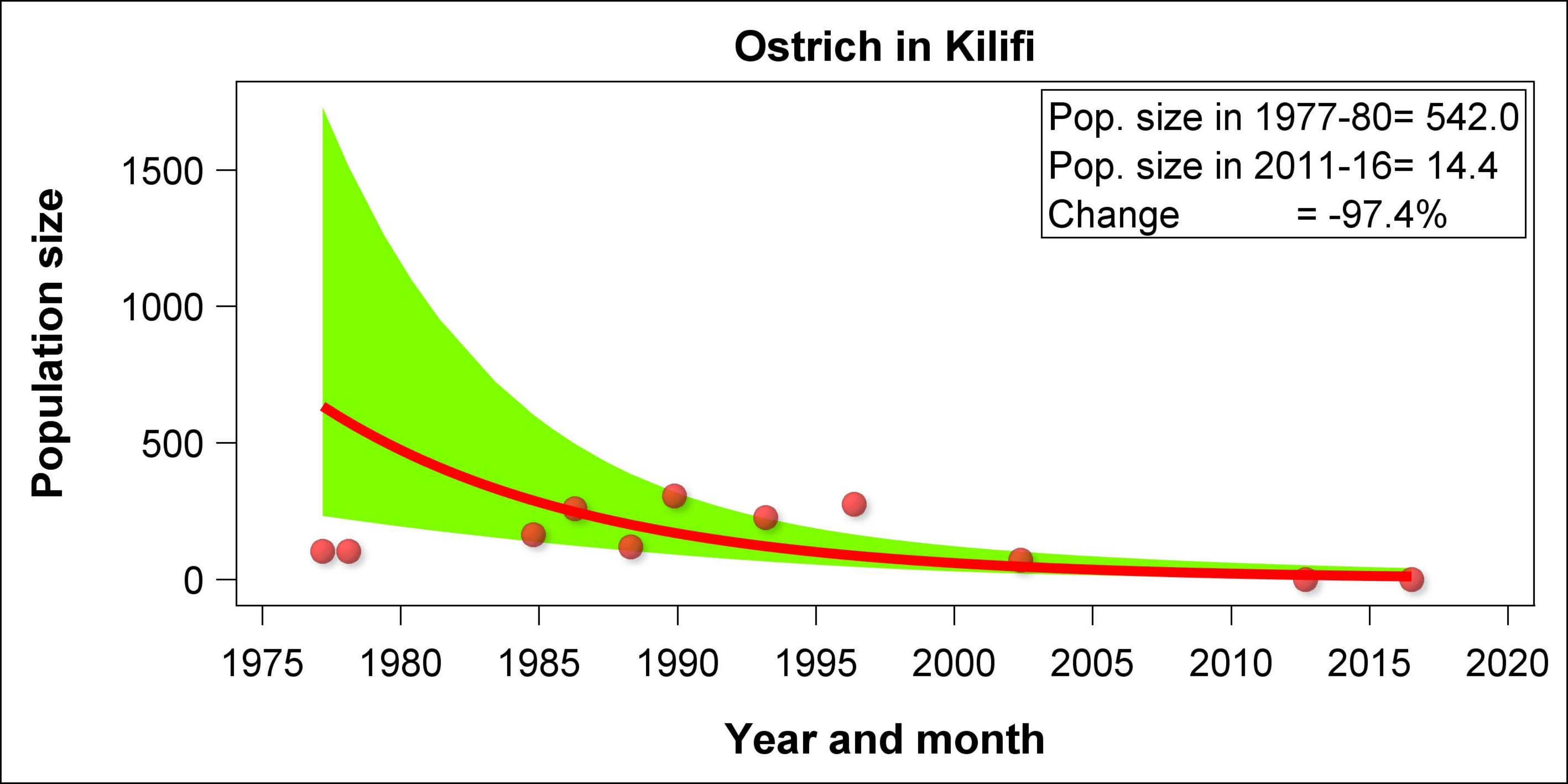
Ostrich in Kilifi
Pop. size in 1977-80= 542.0
Pop. size in 2011-16= 14.4
Change = -97.4%
Population size
1500
1000
500
0
1975
1980
1985
1990
1995
2000
2005
2010
2015
2020
Year and month

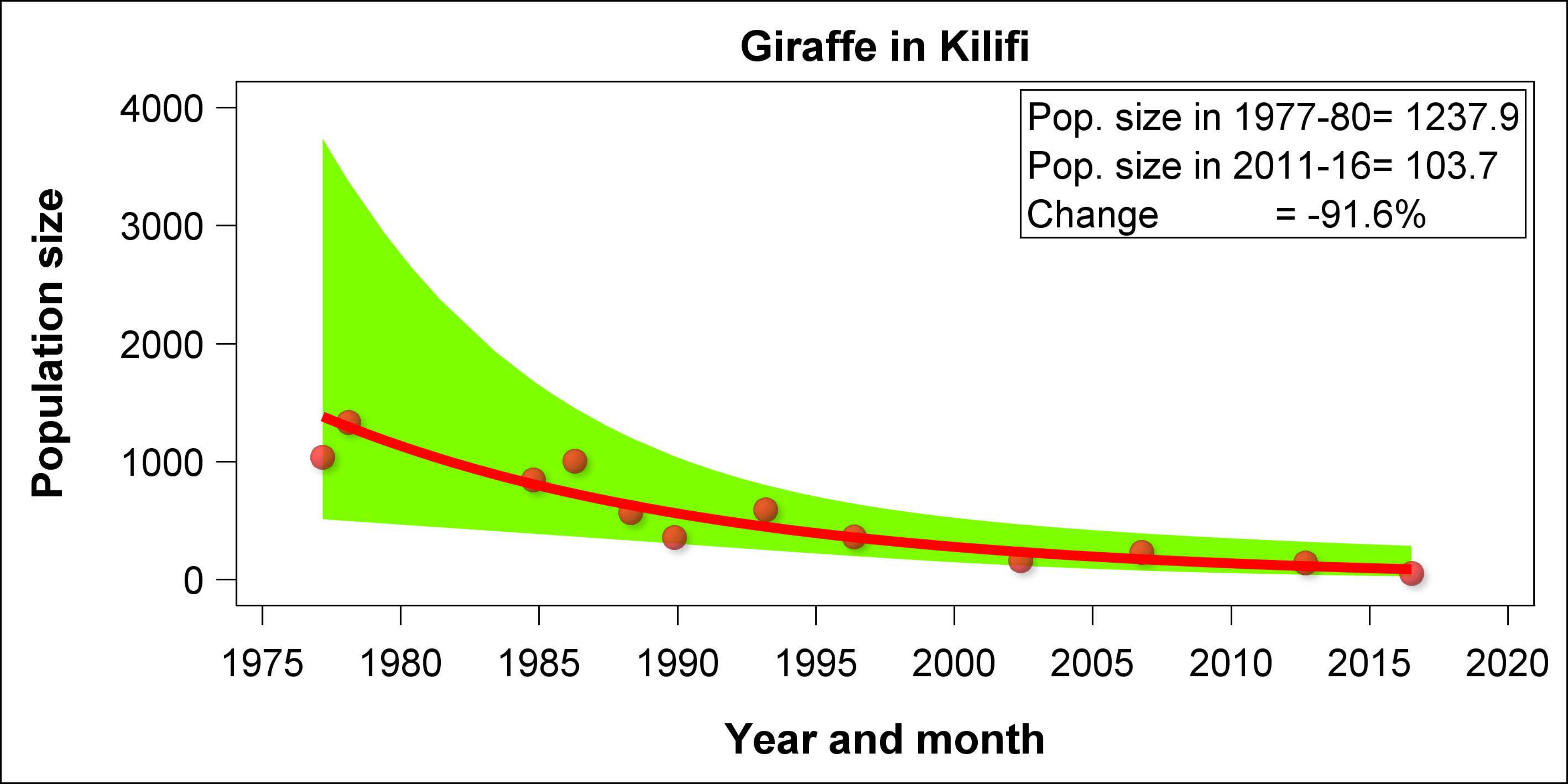
Giraffe in Kilifi
Pop. size in 1977-80= 1237.9
Pop. size in 2011-16= 103.7
Change = -91.6%
Population size
4000
3000
2000
1000
0
1975
1980
1985
1990
1995
2000
2005
2010
2015
2020
Year and month

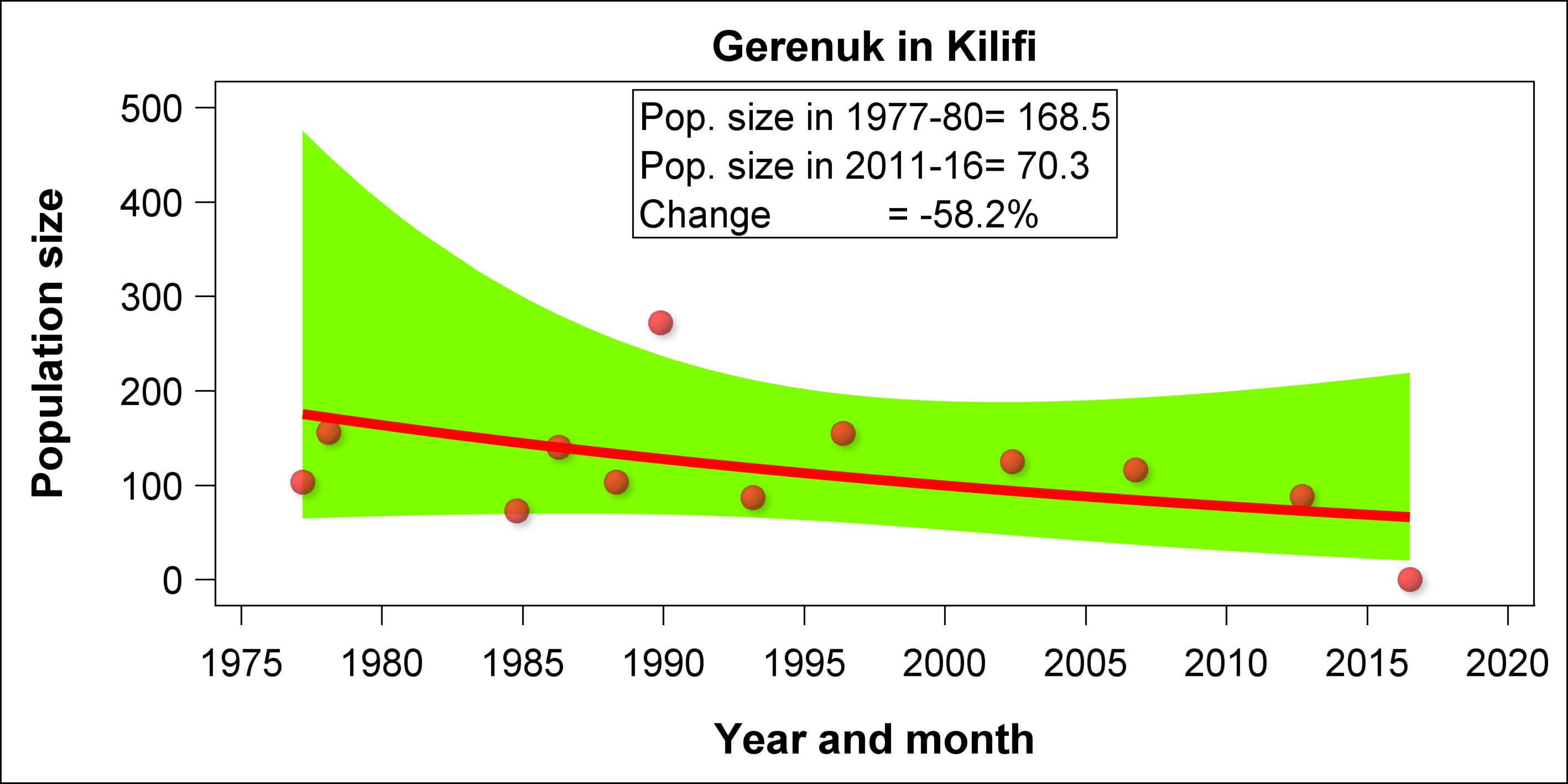
Gerenuk in Kilifi
Pop. size in 1977-80= 168.5
Pop. size in 2011-16= 70.3
Change = -58.2%
Population size
0
100
200
300
400
500
1975
1980
1985
1990
1995
2000
2005
2010
2015
2020
Year and month

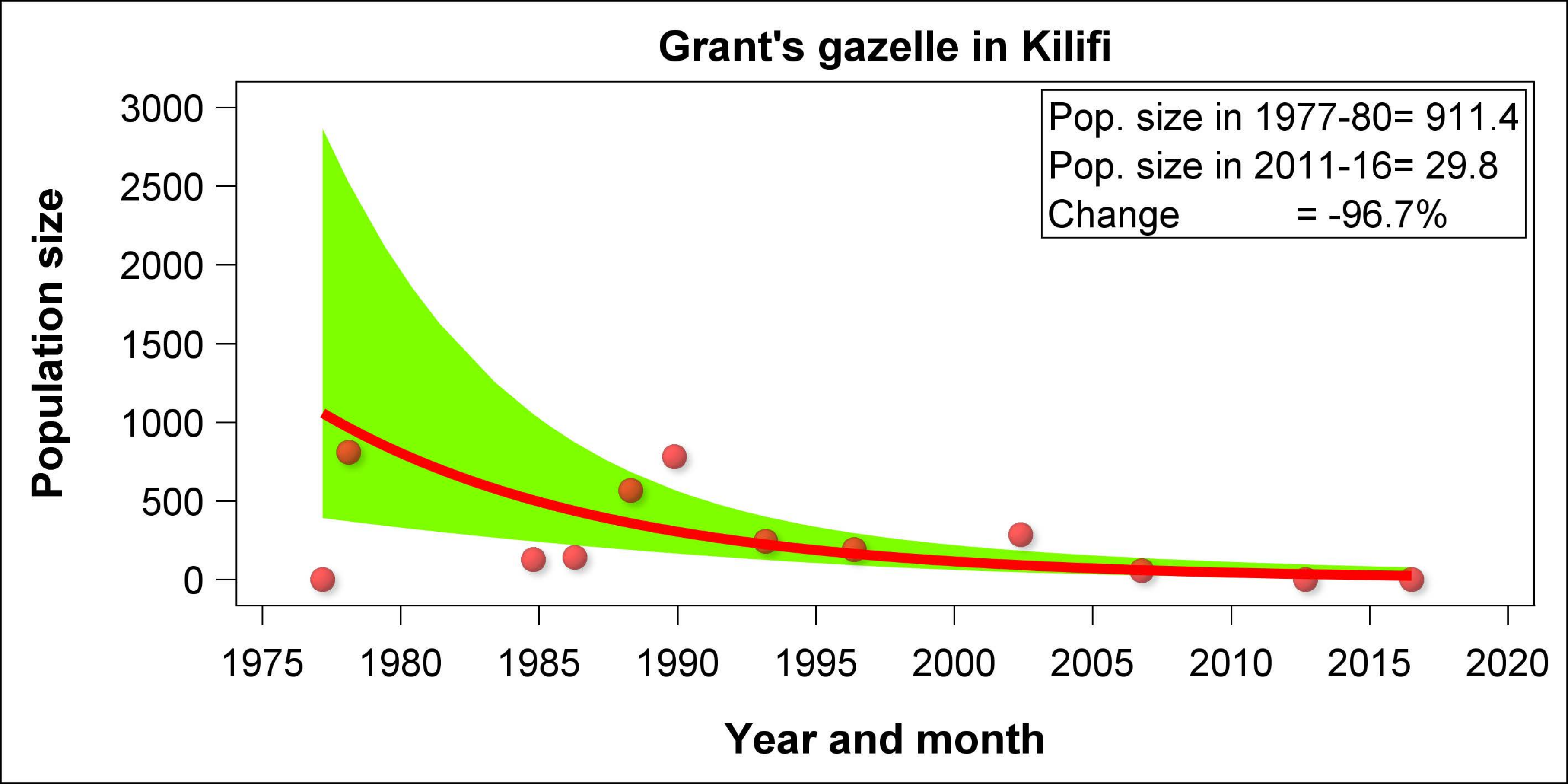
Grant's gazelle in Kilifi
Pop. size in 1977-80= 911.4
Pop. size in 2011-16= 29.8
Change = -96.7%
Population size
3000
2500
2000
1500
1000
500
0
1975
1980
1985
1990
1995
2000
2005
2010
2015
2020
Year and month

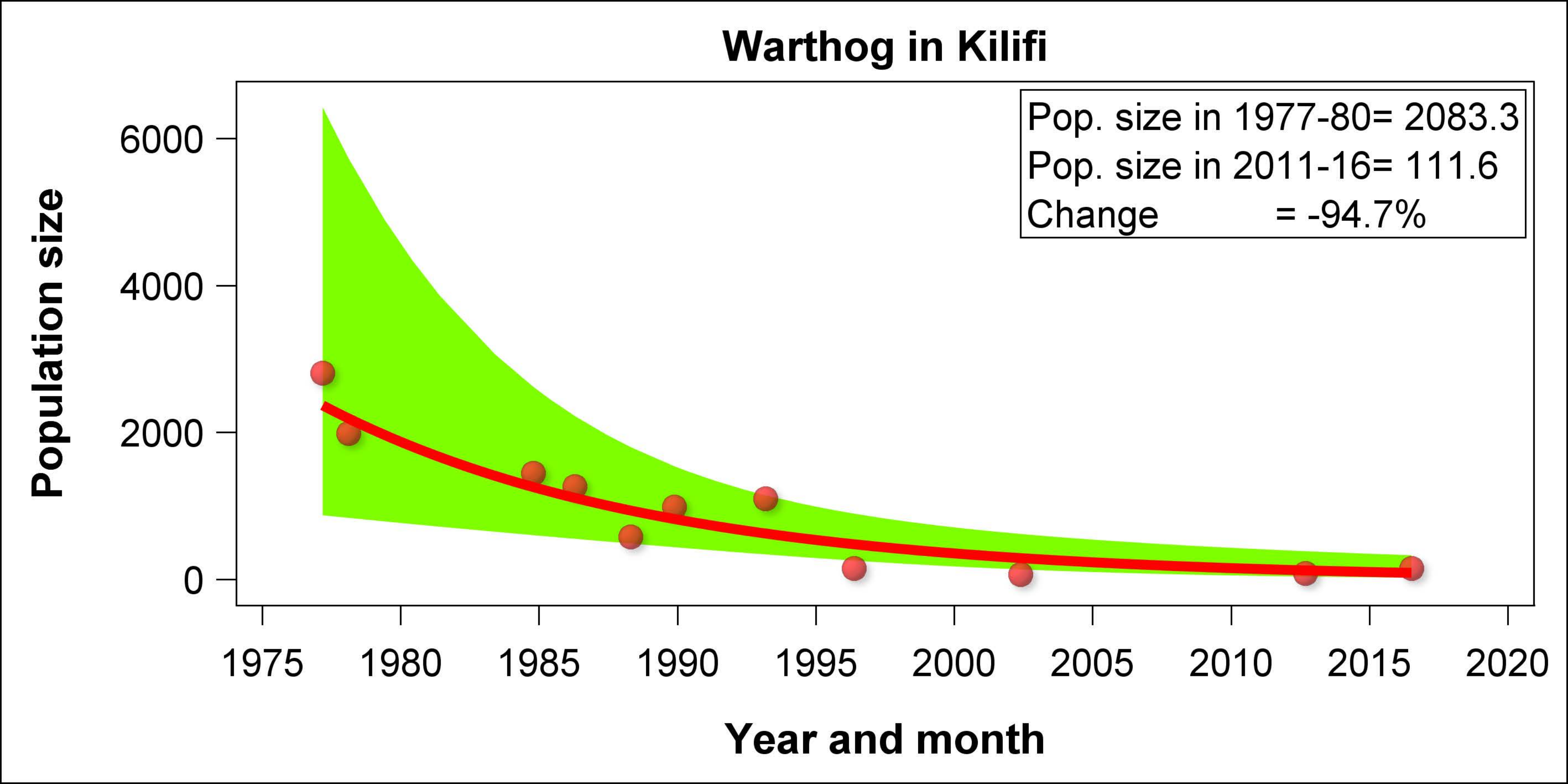

Warthog in Kilifi
Pop. size in 1977-80= 2083.3
Pop. size in 2011-16= 111.6
Change = -94.7%
Population size
6000
4000
2000
0
1975
1980
1985
1990
1995
2000
2005
2010
2015
2020
Year and month

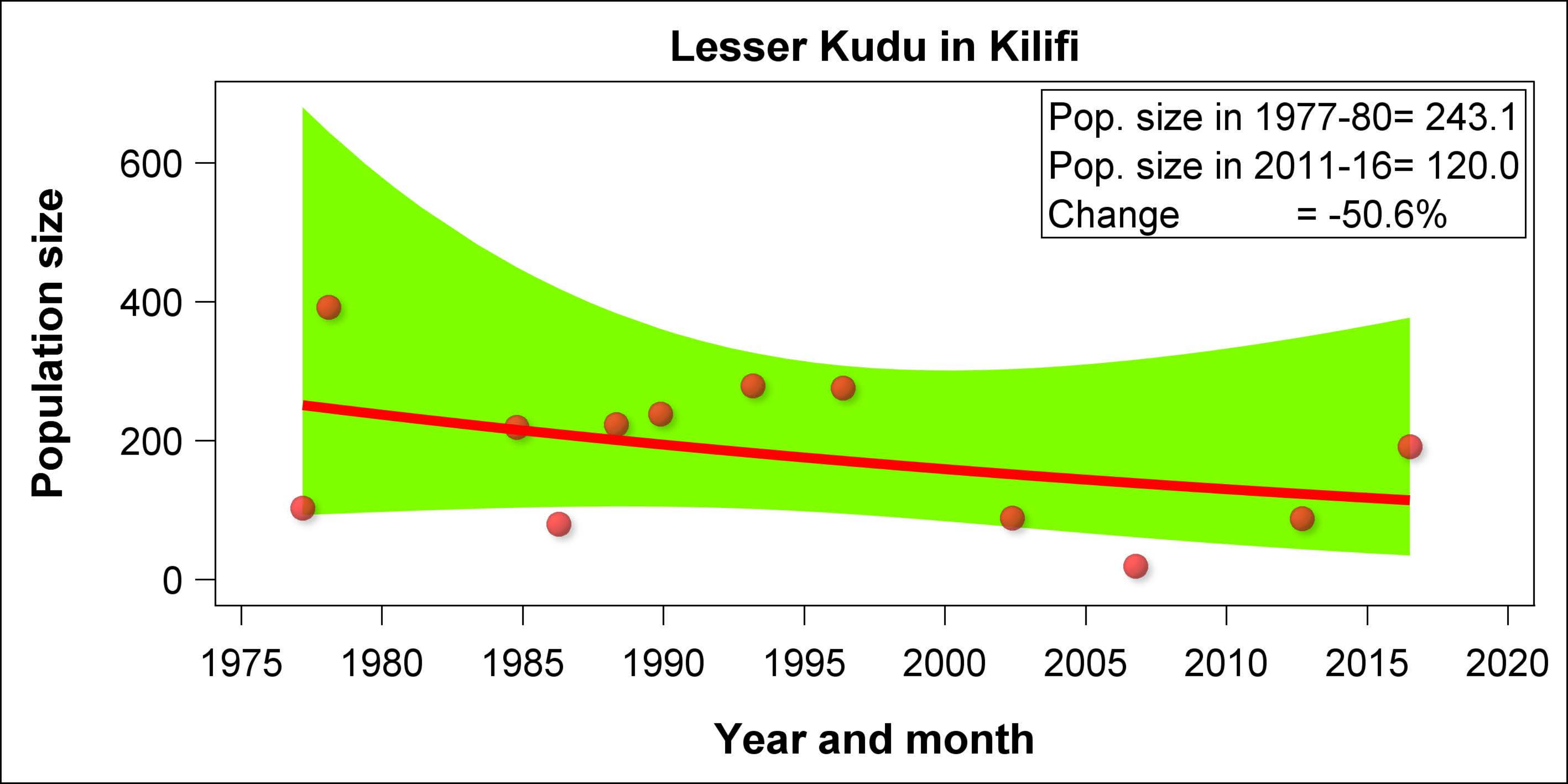

Lesser Kudu in Kilifi
Pop. size in 1977-80= 243.1
Pop. size in 2011-16= 120.0
Change = -50.6%
Population size
0
200
400
600
1975
1980
1985
1990
1995
2000
2005
2010
2015
2020
Year and month

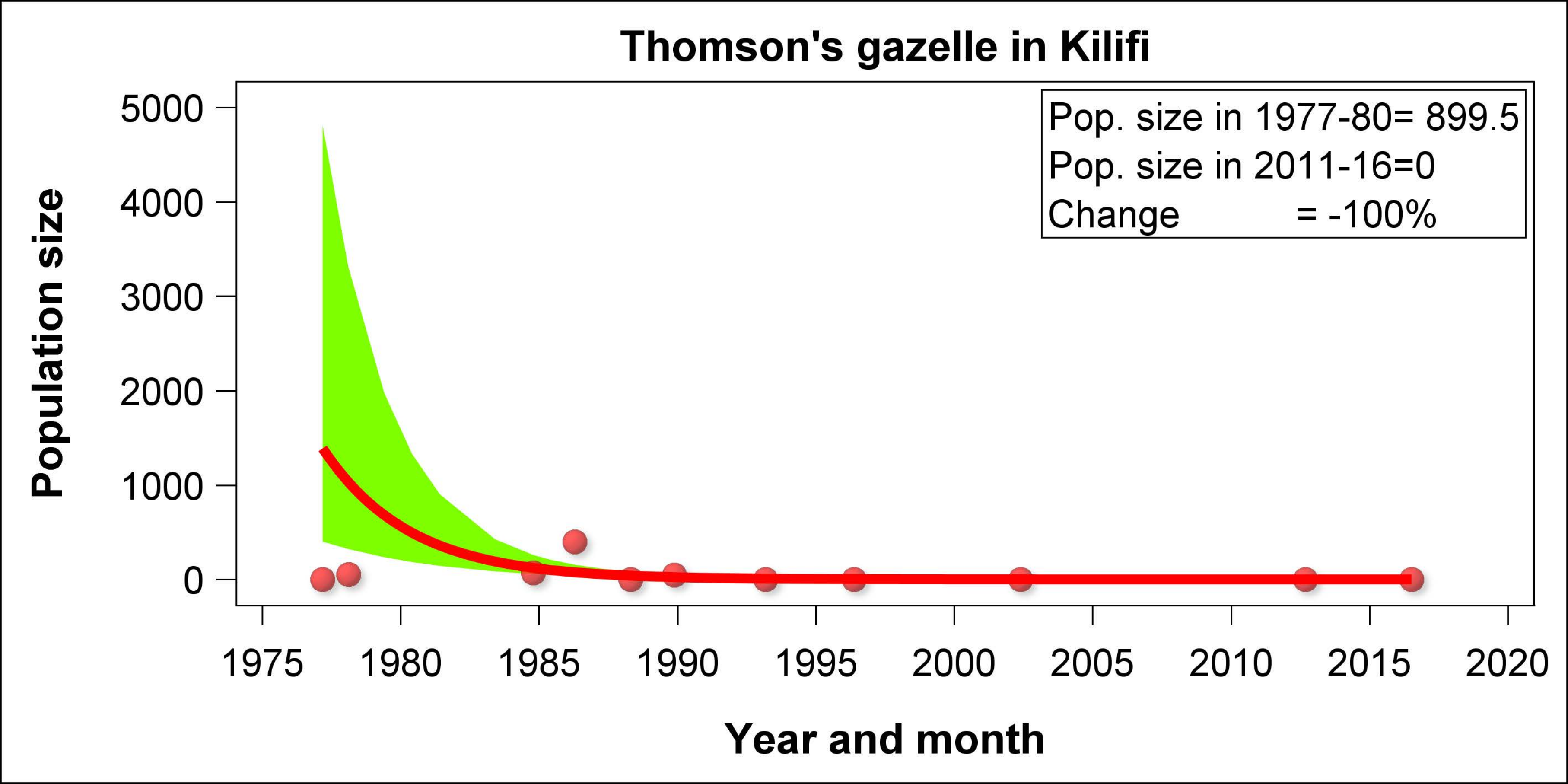

Thomson's gazelle in Kilifi
Pop. size in 1977-80= 899.5
Pop. size in 2011-16=0
Change = -100%
Population size
5000
4000
3000
2000
1000
0
1975
1980
1985
1990
1995
2000
2005
2010
2015
2020
Year and month

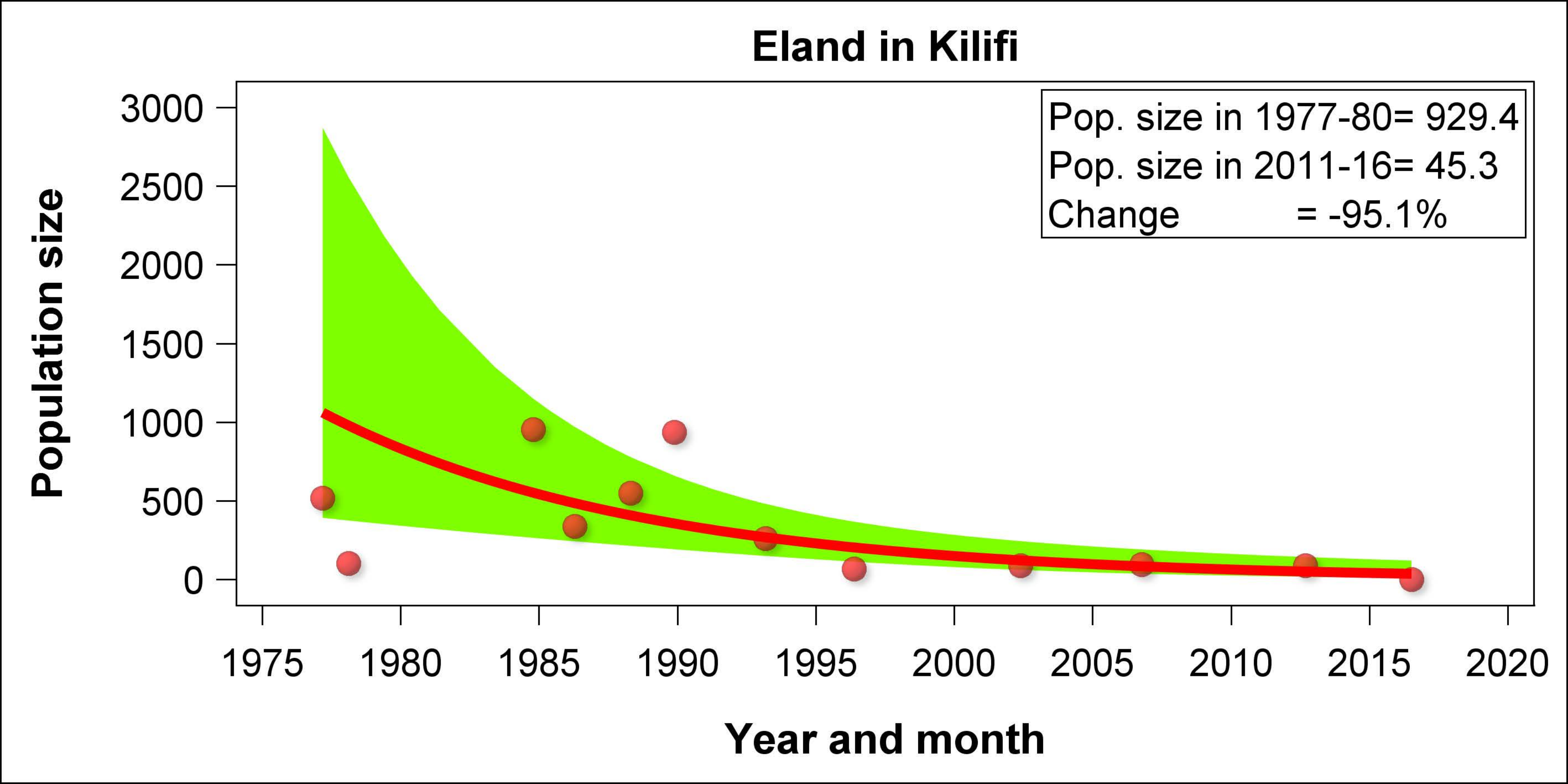

Eland in Kilifi
Pop. size in 1977-80= 929.4
Pop. size in 2011-16= 45.3
Change = -95.1%
Population size
3000
2500
2000
1500
1000
500
0
1975
1980
1985
1990
1995
2000
2005
2010
2015
2020
Year and month

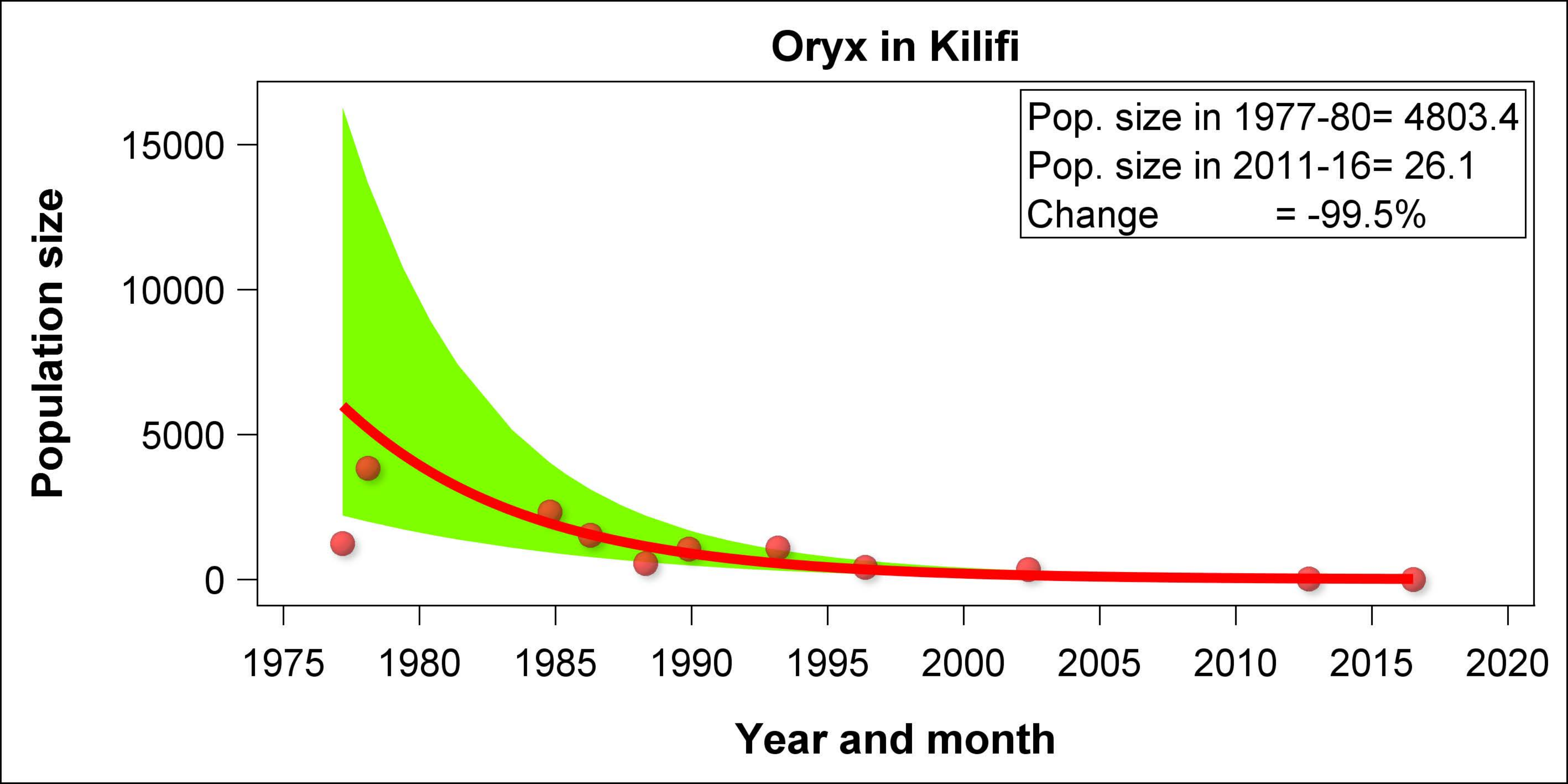
Oryx in Kilifi
Pop. size in 1977-80= 4803.4
Pop. size in 2011-16= 26.1
Change = -99.5%
Population size
15000
10000
5000
0
1975
1980
1985
1990
1995
2000
2005
2010
2015
2020
Year and month

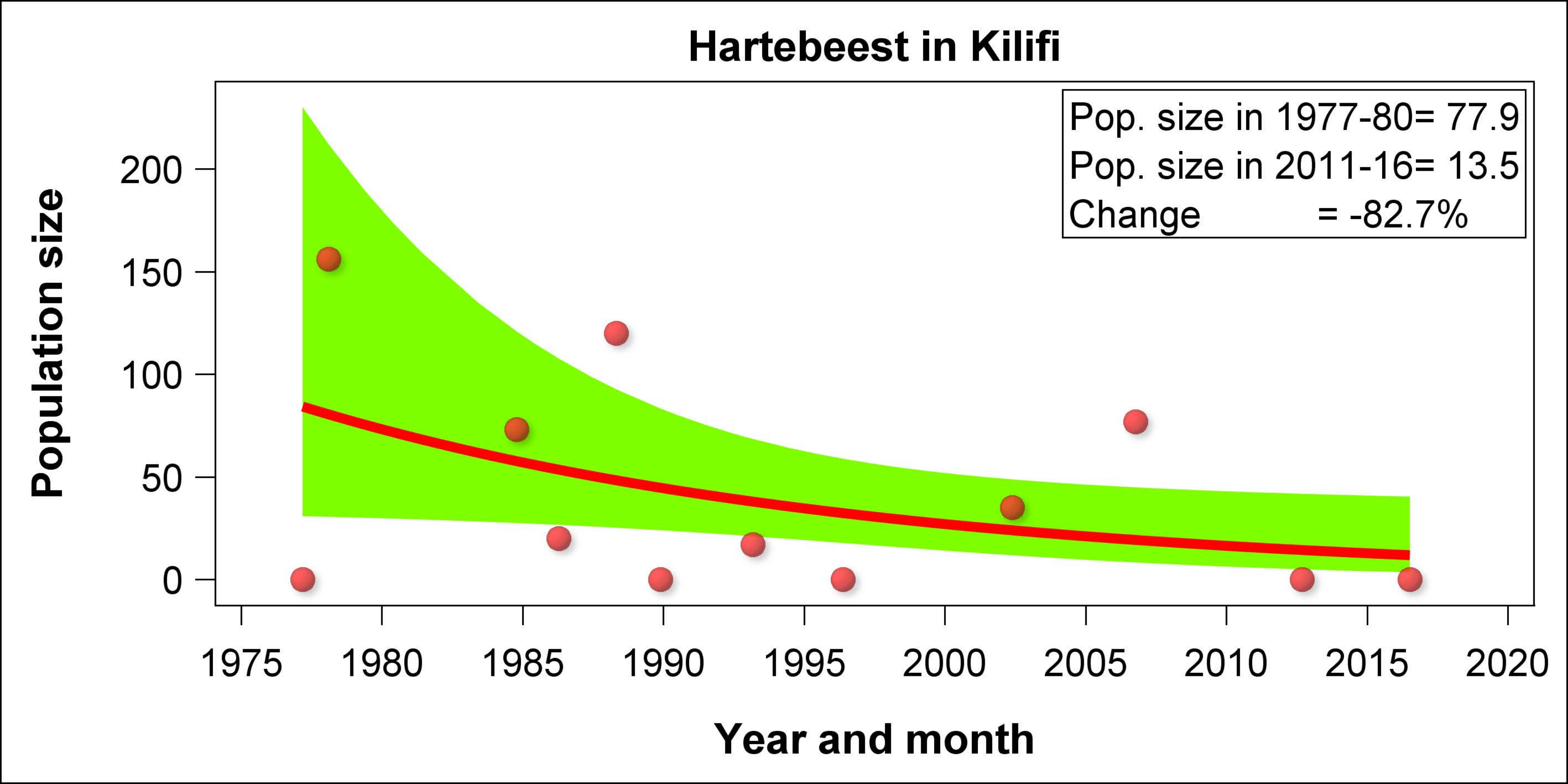
Hartebeest in Kilifi
Pop. size in 1977-80= 77.9
Pop. size in 2011-16= 13.5
Change = -82.7%
Population size
0
50
100
150
200
1975
1980
1985
1990
1995
2000
2005
2010
2015
2020
Year and month

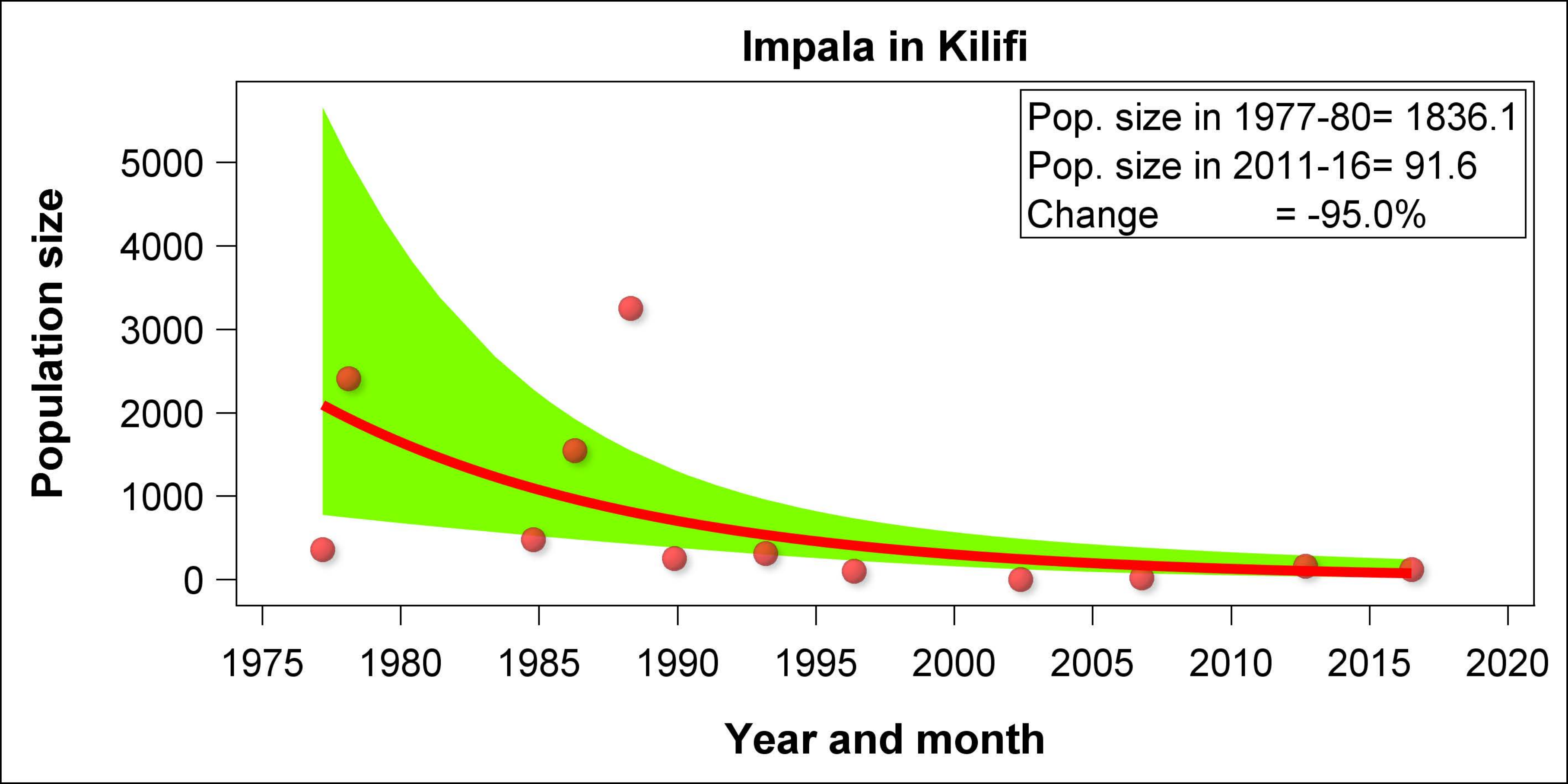
Impala in Kilifi
Pop. size in 1977-80= 1836.1
Pop. size in 2011-16= 91.6
Change = -95.0%
Population size
5000
4000
3000
2000
1000
0
1975
1980
1985
1990
1995
2000
2005
2010
2015
2020
Year and month

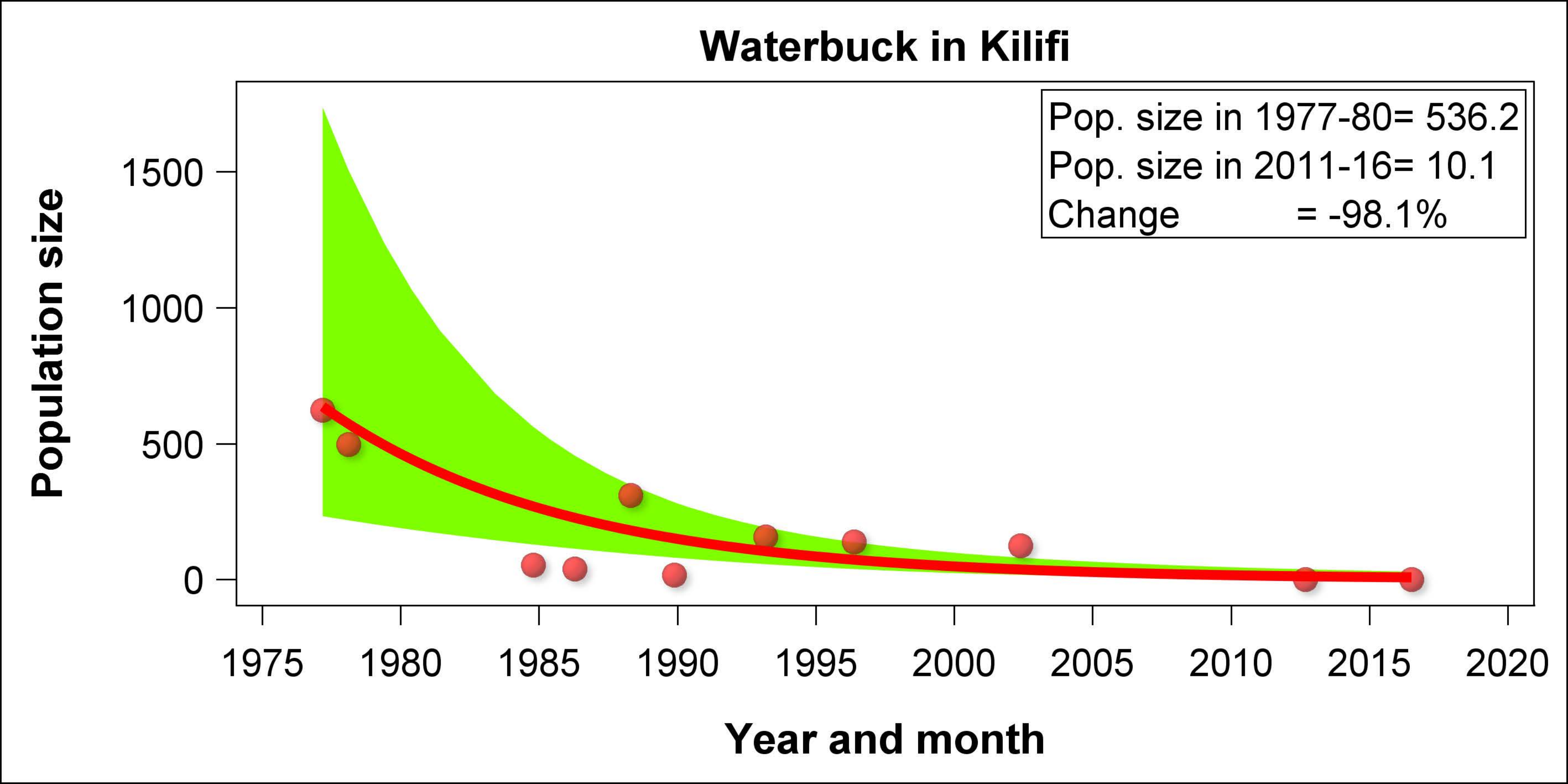
Waterbuck in Kilifi
Pop. size in 1977-80= 536.2
Pop. size in 2011-16= 10.1
Change = -98.1%
Population size
1500
1000
500
0
1975
1980
1985
1990
1995
2000
2005
2010
2015
2020
Year and month
