# Supplementary material for: Extreme Wildlife Declines and Concurrent Increase in Livestock Numbers in Kenya: What Are the Causes?
Source: PLoS One. 2016 Sep 27;11(9):e0163249. doi: 10.1371/journal.pone.0163249 (PMC5039022; doi:10.1371/journal.pone.0163249)
Supplement: S9 Fig — The solid red line is the fitted trend curve and the shaded chartreuse band is the pointwise 95% confidence band. The estimated average population size in 1977–1980 and 2011–2014 and the percentage change in population size between the two periods are provided in the inset. (PDF) [file pone.0163249.s019.pdf]

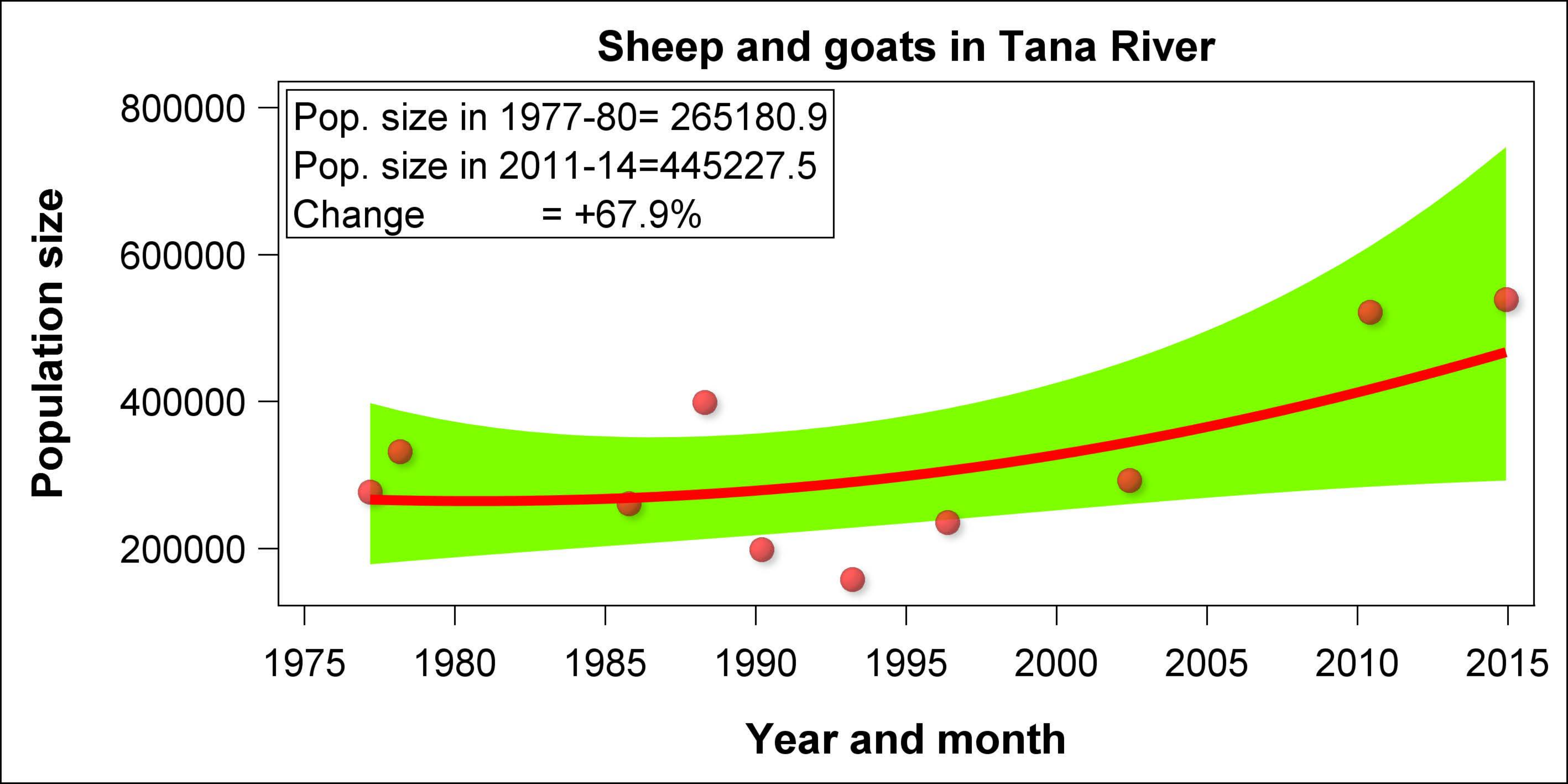
Sheep and goats in Tana River
Pop. size in 1977-80= 265180.9
Pop. size in 2011-14=445227.5
Change = +67.9%
Population size
800000
600000
400000
200000
1975
1980
1985
1990
1995
2000
2005
2010
2015
Year and month

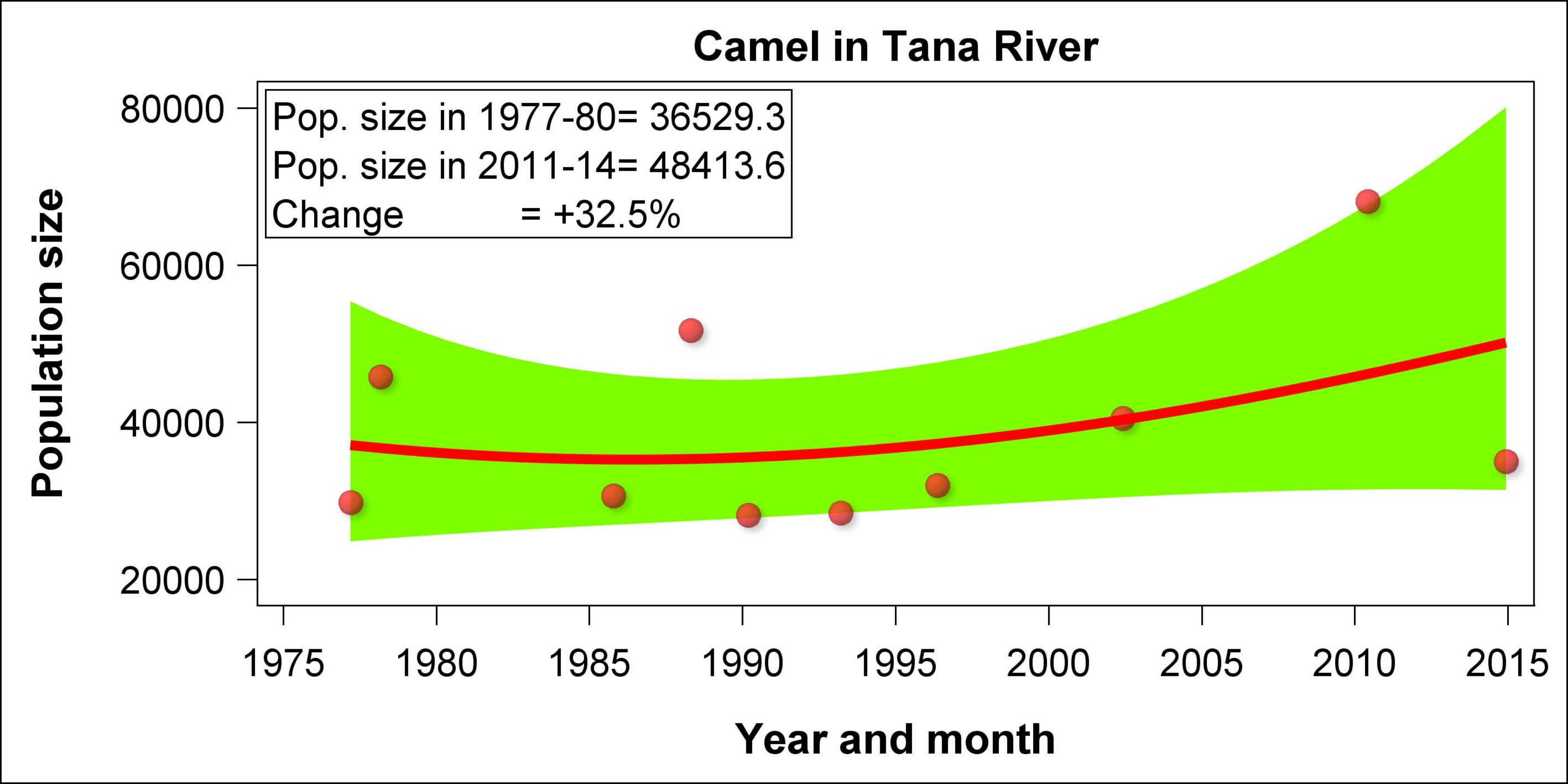
Camel in Tana River
Pop. size in 1977-80= 36529.3
Pop. size in 2011-14= 48413.6
Change = +32.5%
Population size
80000
60000
40000
20000
1975
1980
1985
1990
1995
2000
2005
2010
2015
Year and month

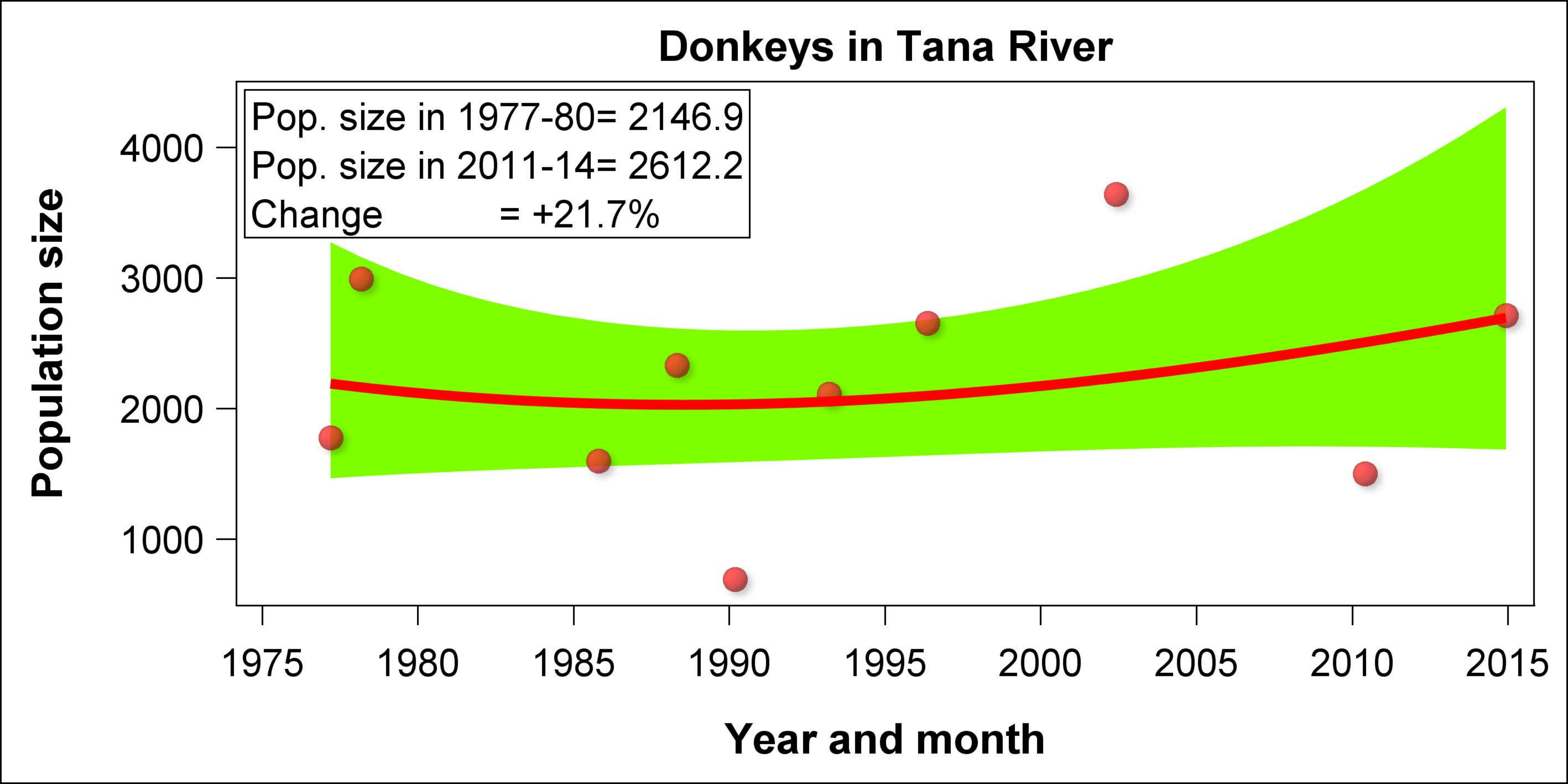

Donkeys in Tana River
Pop. size in 1977-80= 2146.9
Pop. size in 2011-14= 2612.2
Change = +21.7%
Population size
4000
3000
2000
1000
1975
1980
1985
1990
1995
2000
2005
2010
2015
Year and month

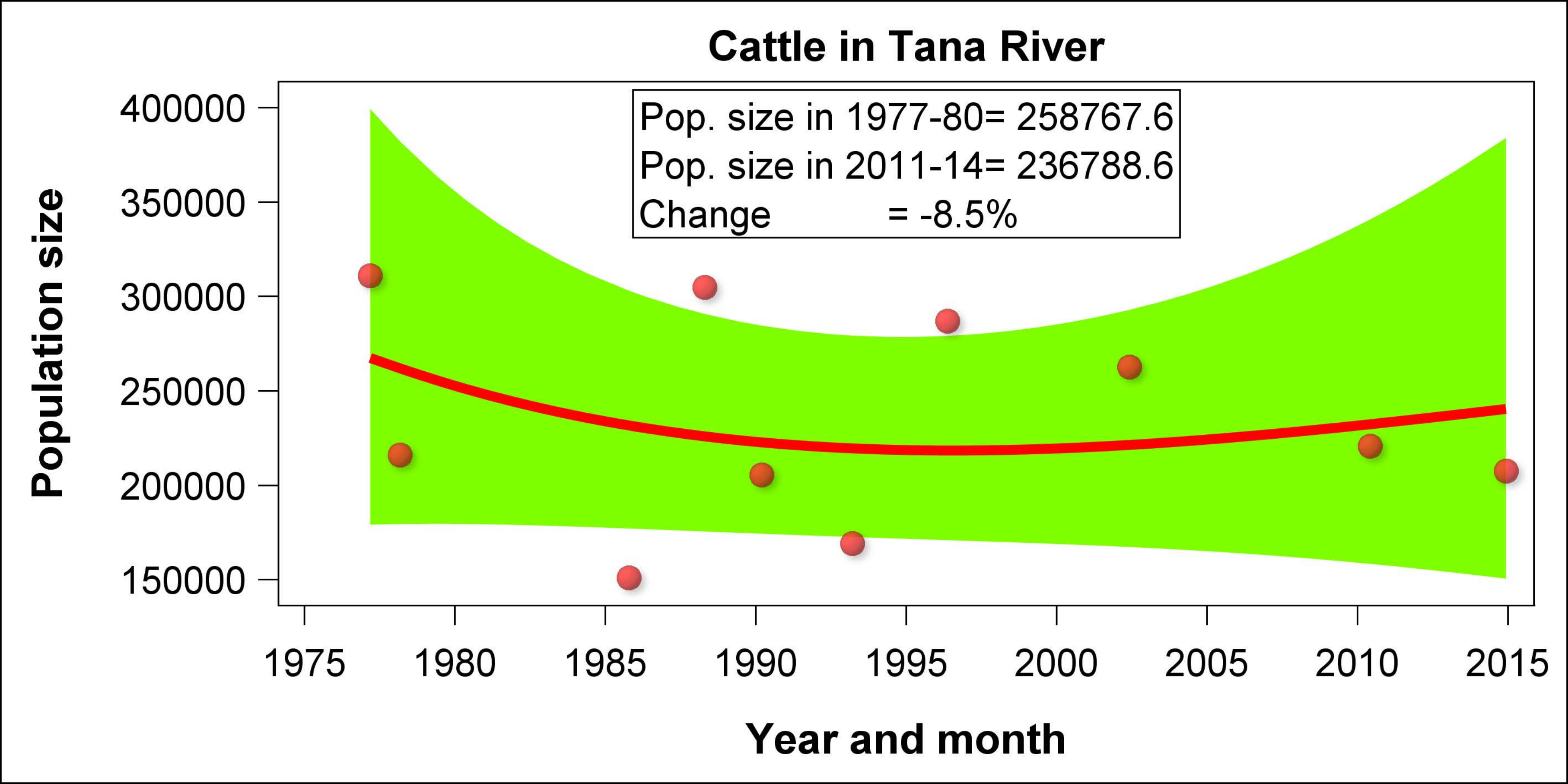
Cattle in Tana River
Pop. size in 1977-80= 258767.6
Pop. size in 2011-14= 236788.6
Change = -8.5%
Population size
400000
350000
300000
250000
200000
150000
1975
1980
1985
1990
1995
2000
2005
2010
2015
Year and month

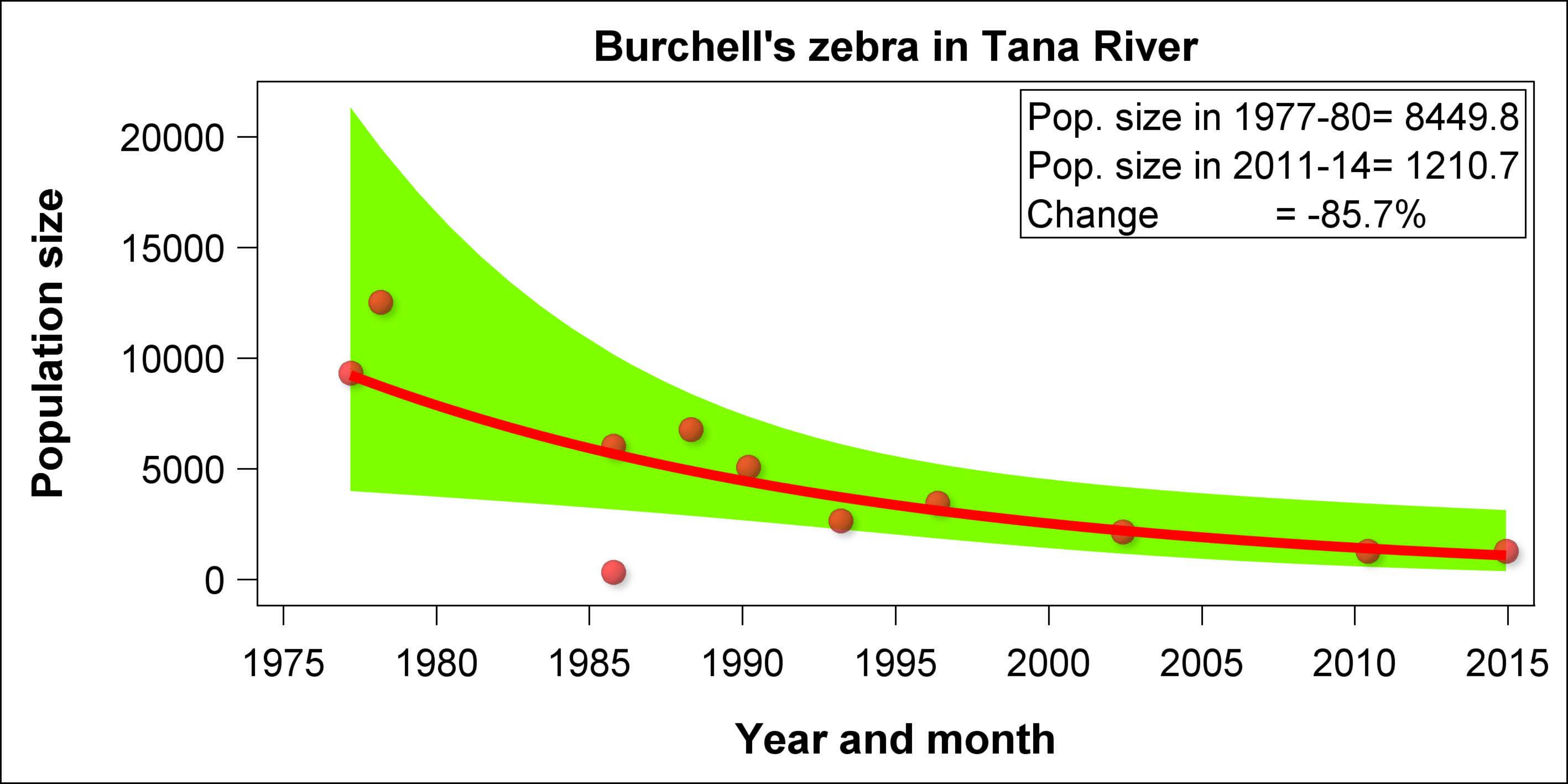

Burchell's zebra in Tana River
Pop. size in 1977-80= 8449.8
Pop. size in 2011-14= 1210.7
Change = -85.7%
Population size
20000
15000
10000
5000
0
1975
1980
1985
1990
1995
2000
2005
2010
2015
Year and month

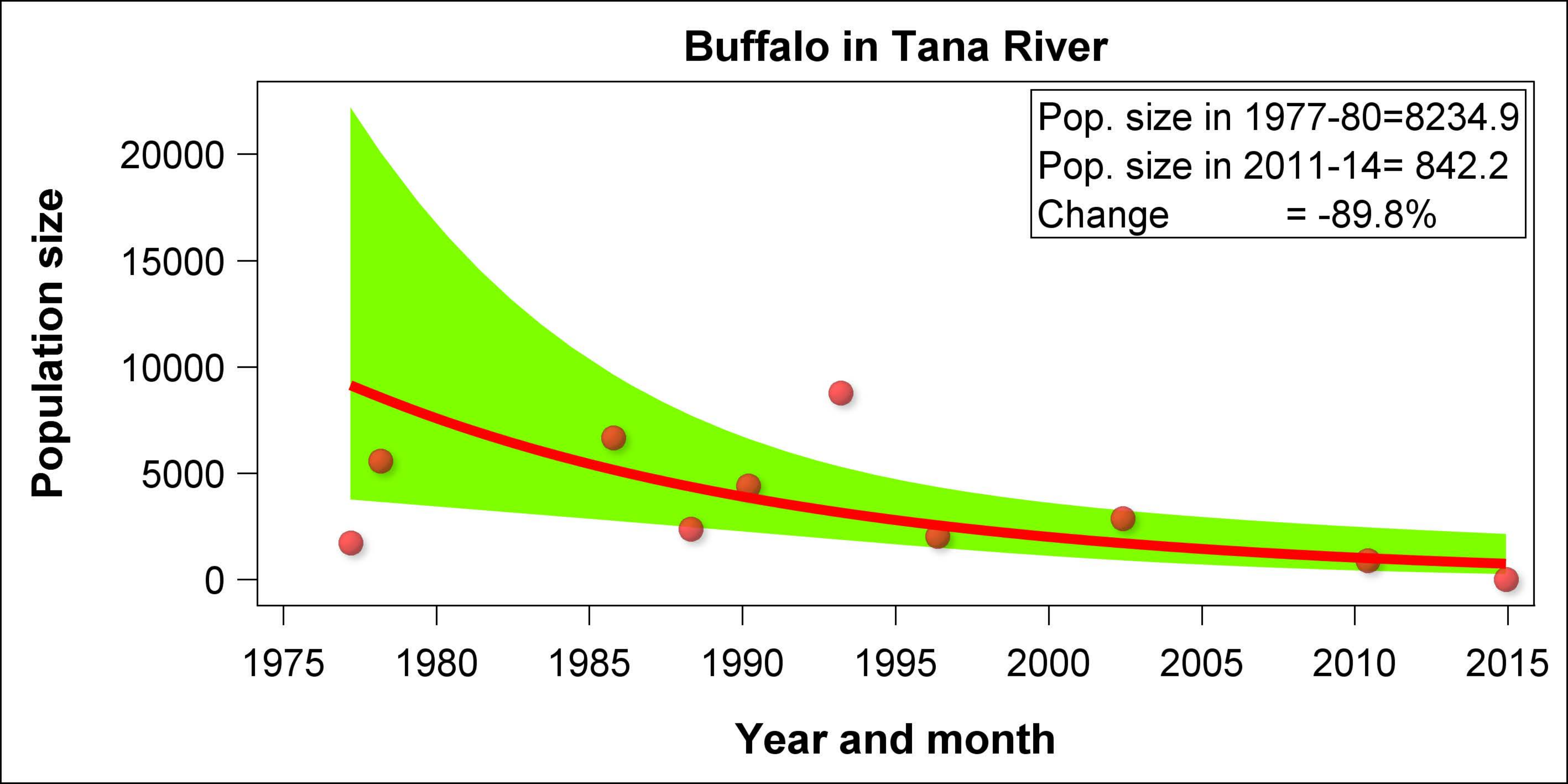

Buffalo in Tana River
Pop. size in 1977-80=8234.9
Pop. size in 2011-14= 842.2
Change = -89.8%
Population size
20000
15000
10000
5000
0
1975
1980
1985
1990
1995
2000
2005
2010
2015
Year and month

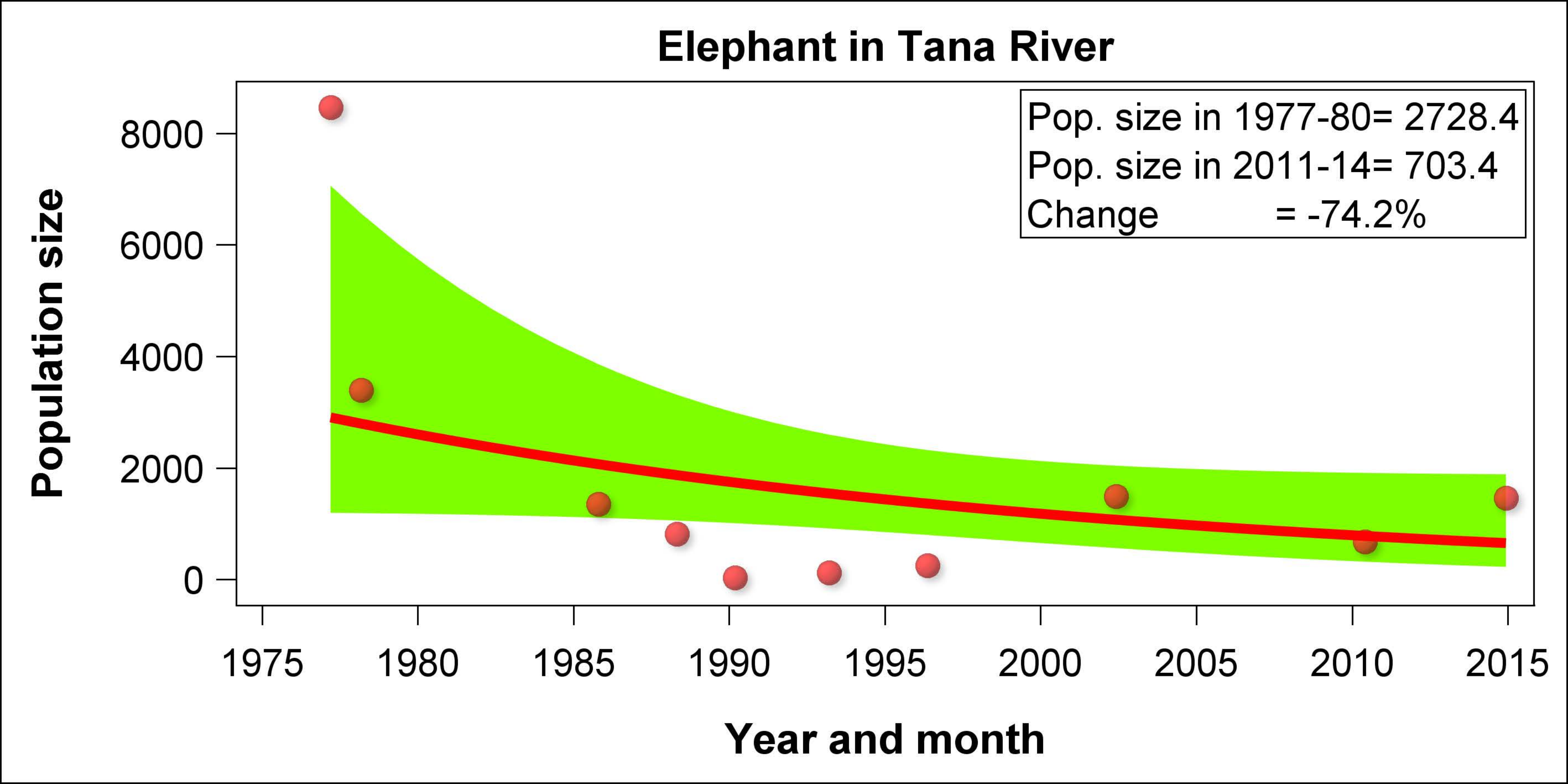
Elephant in Tana River
Pop. size in 1977-80= 2728.4
Pop. size in 2011-14= 703.4
Change = -74.2%
Population size
8000
6000
4000
2000
0
1975
1980
1985
1990
1995
2000
2005
2010
2015
Year and month

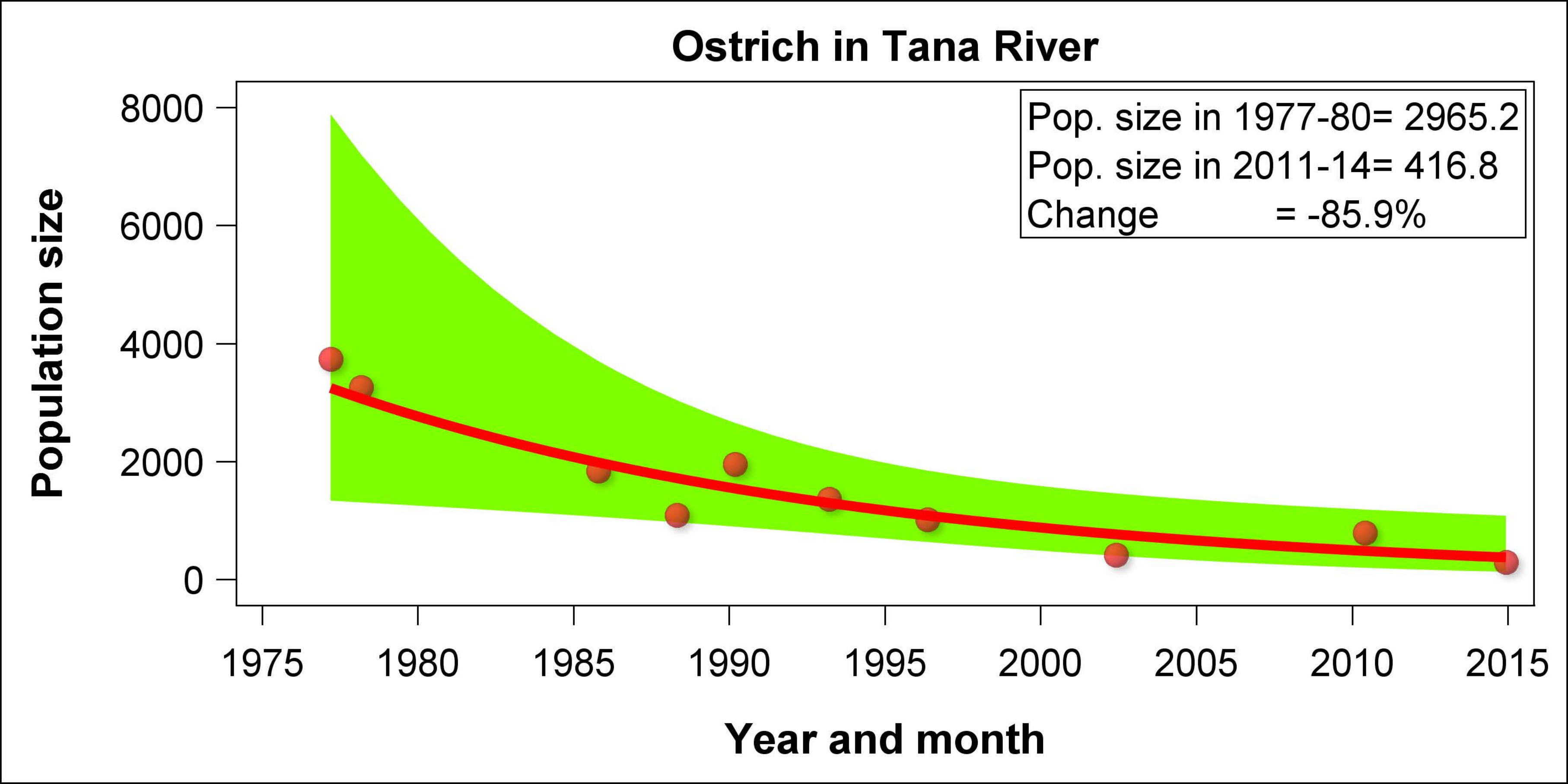

Ostrich in Tana River
Pop. size in 1977-80= 2965.2
Pop. size in 2011-14= 416.8
Change = -85.9%
Population size
8000
6000
4000
2000
0
1975
1980
1985
1990
1995
2000
2005
2010
2015
Year and month

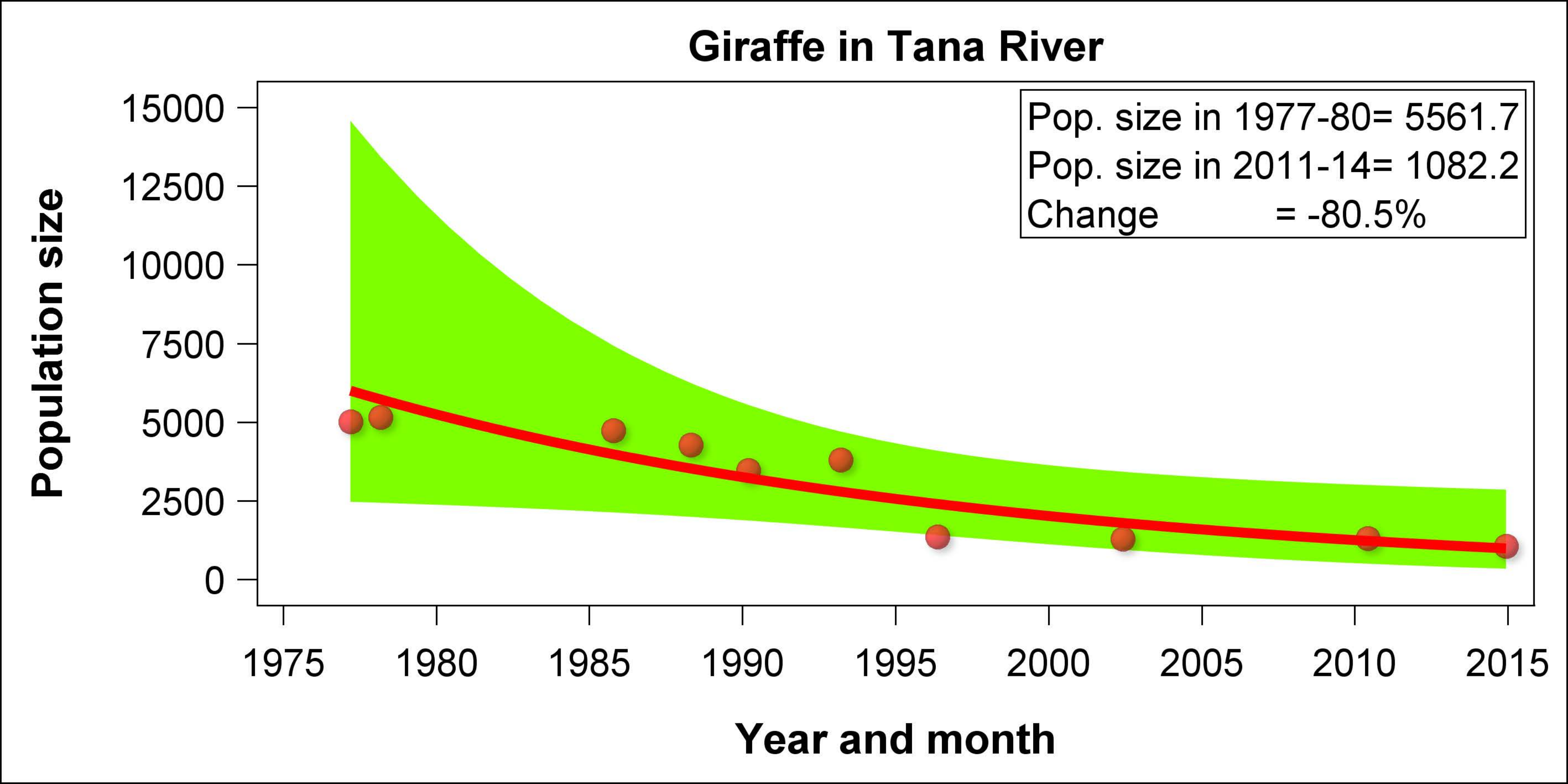
Giraffe in Tana River
Pop. size in 1977-80= 5561.7
Pop. size in 2011-14= 1082.2
Change = -80.5%
Population size
15000
12500
10000
7500
5000
2500
0
1975
1980
1985
1990
1995
2000
2005
2010
2015
Year and month

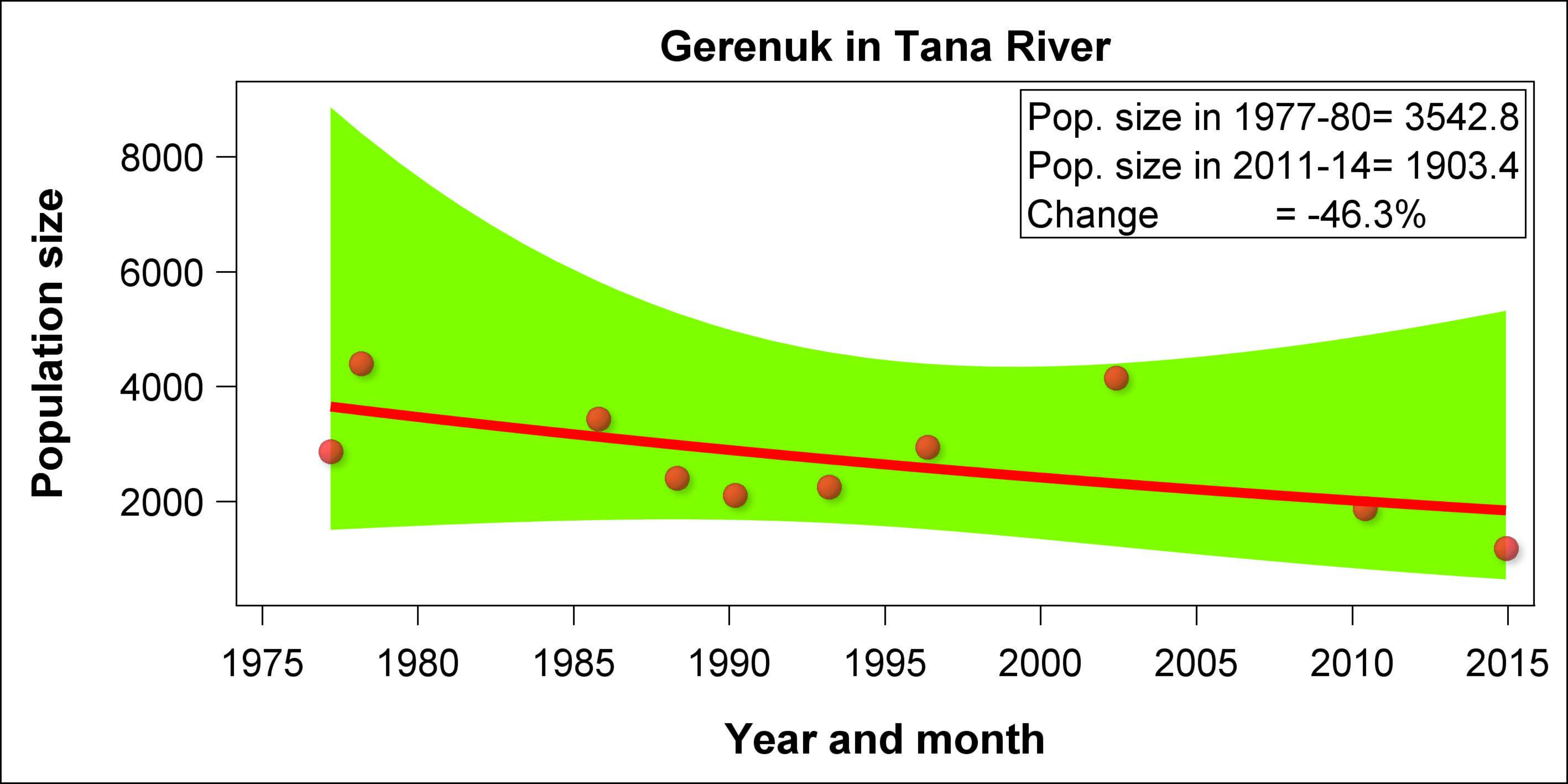
Gerenuk in Tana River
Pop. size in 1977-80= 3542.8
Pop. size in 2011-14= 1903.4
Change = -46.3%
Population size
8000
6000
4000
2000
1975
1980
1985
1990
1995
2000
2005
2010
2015
Year and month

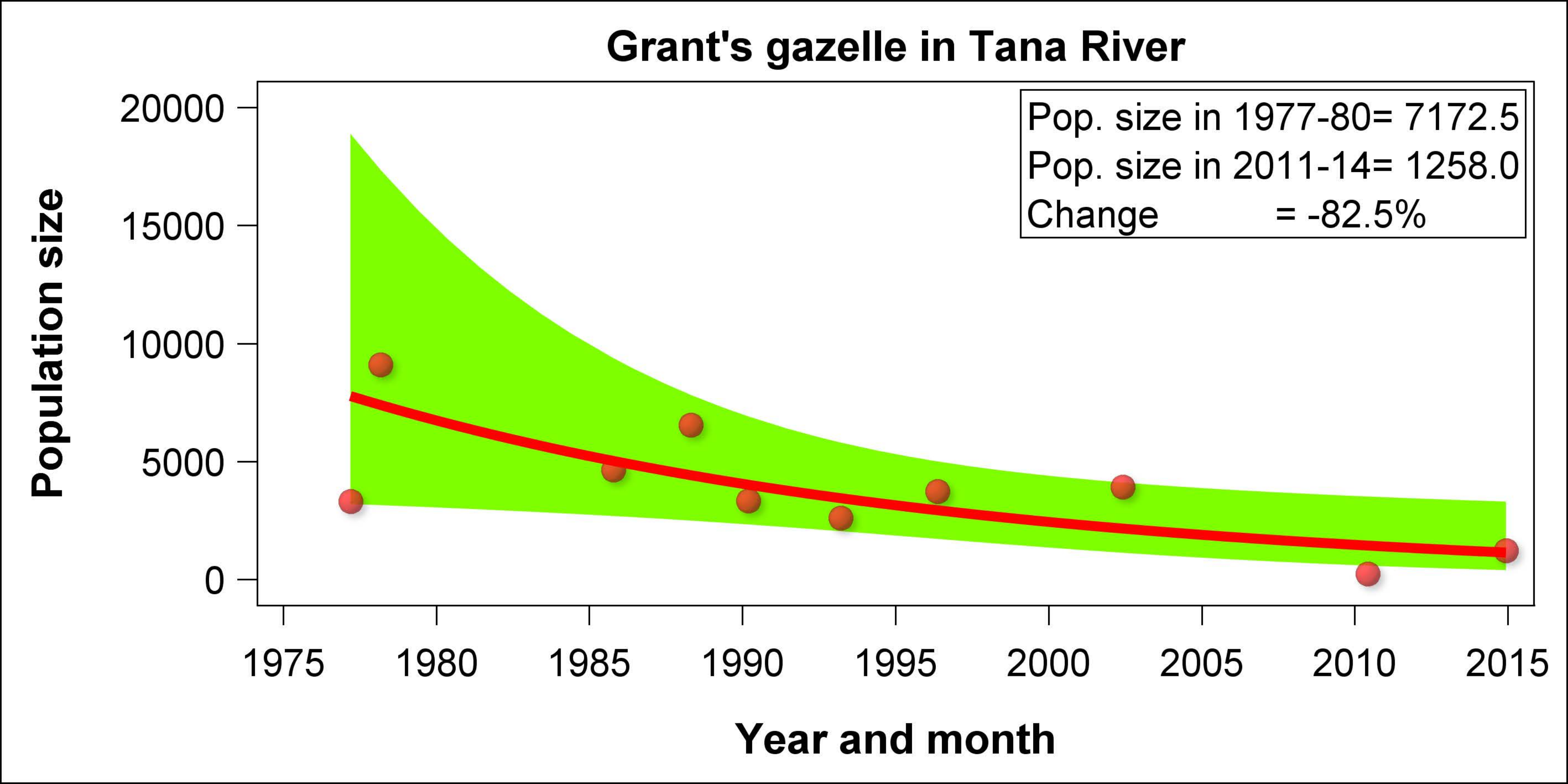

Grant's gazelle in Tana River
Pop. size in 1977-80= 7172.5
Pop. size in 2011-14= 1258.0
Change = -82.5%
Population size
20000
15000
10000
5000
0
1975
1980
1985
1990
1995
2000
2005
2010
2015
Year and month

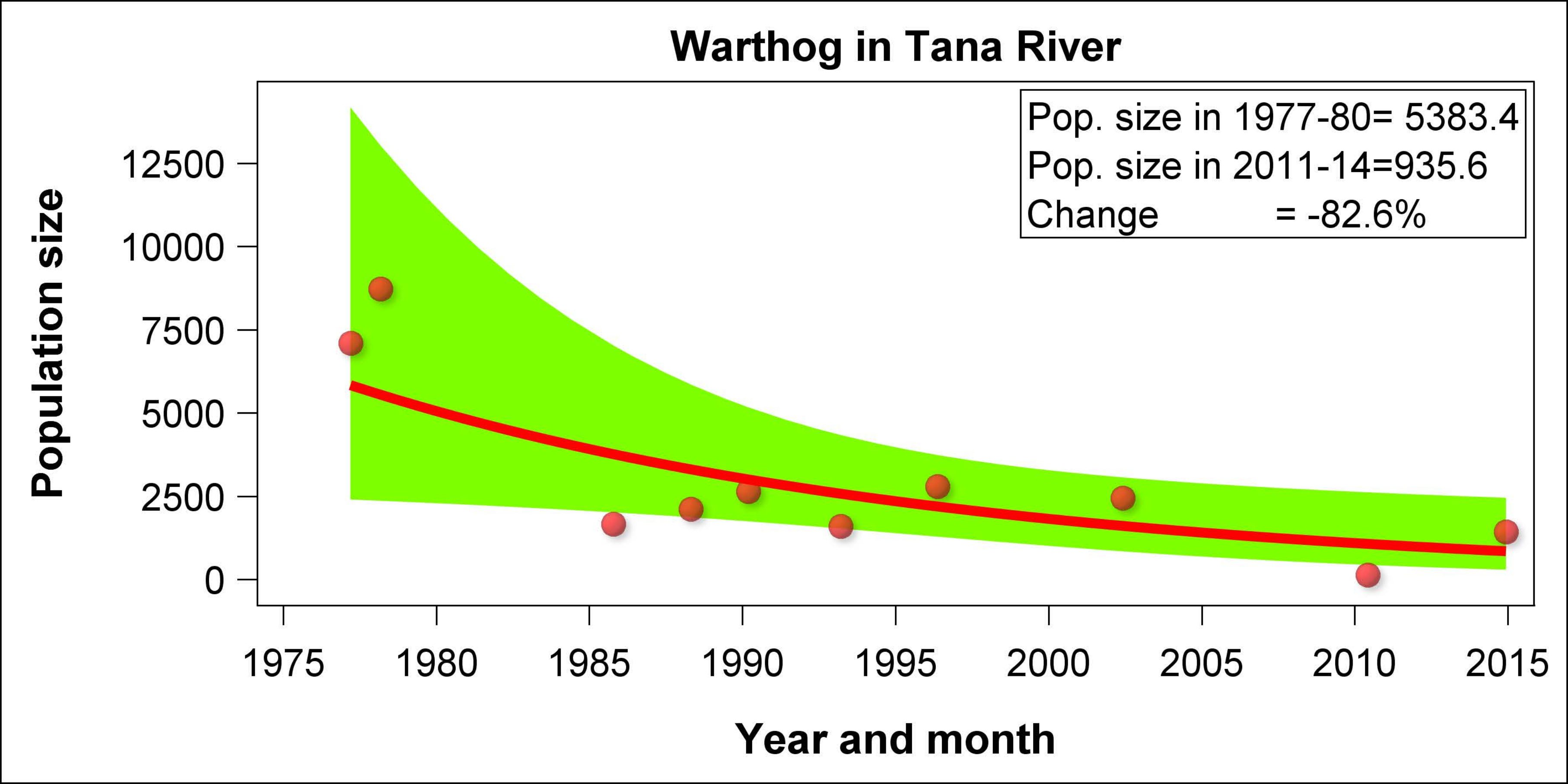
Warthog in Tana River
Pop. size in 1977-80= 5383.4
Pop. size in 2011-14=935.6
Change = -82.6%
Population size
12500
10000
7500
5000
2500
0
1975
1980
1985
1990
1995
2000
2005
2010
2015
Year and month

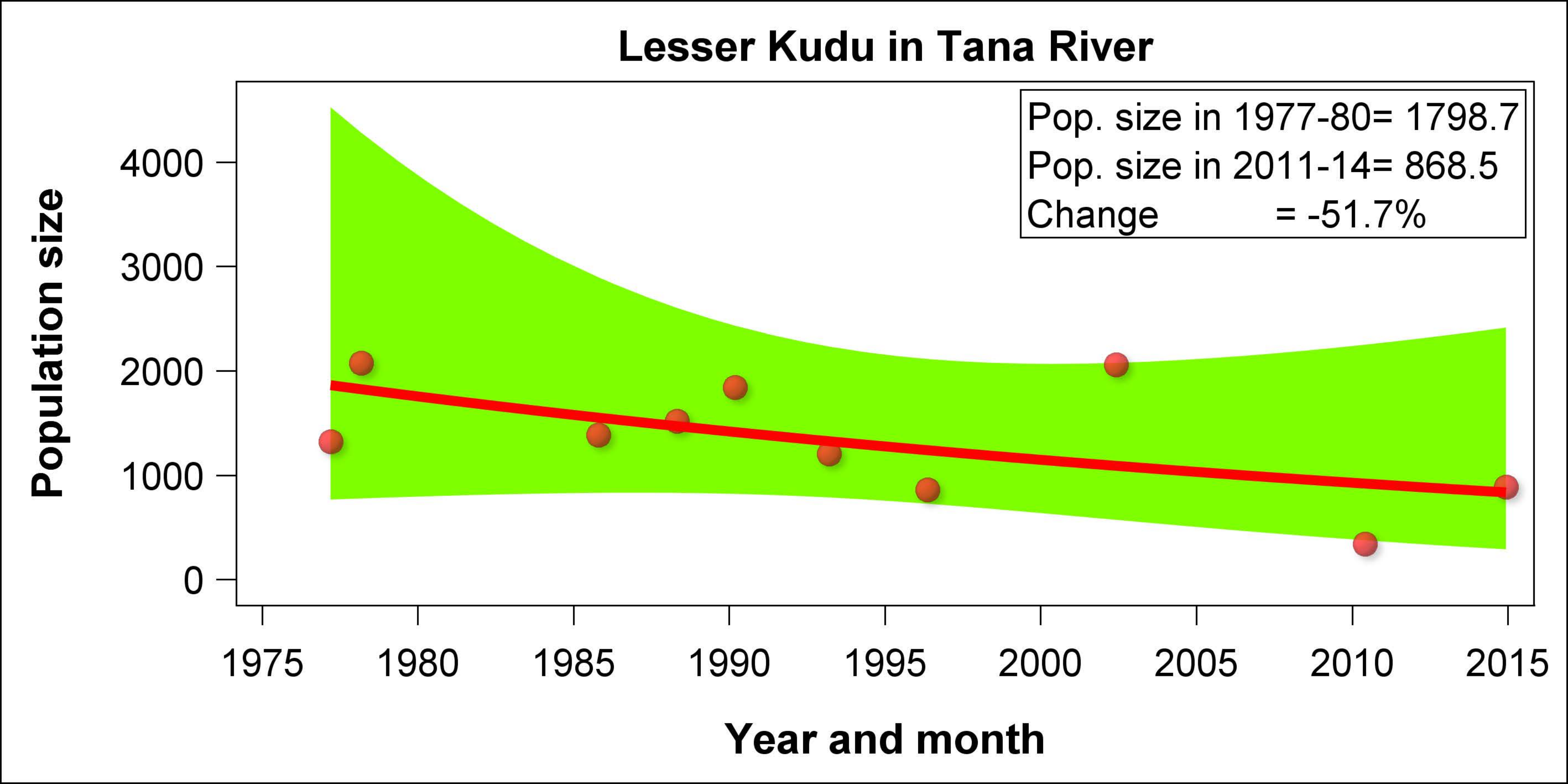

Lesser Kudu in Tana River
Pop. size in 1977-80= 1798.7
Pop. size in 2011-14= 868.5
Change = -51.7%
Population size
4000
3000
2000
1000
0
1975
1980
1985
1990
1995
2000
2005
2010
2015
Year and month

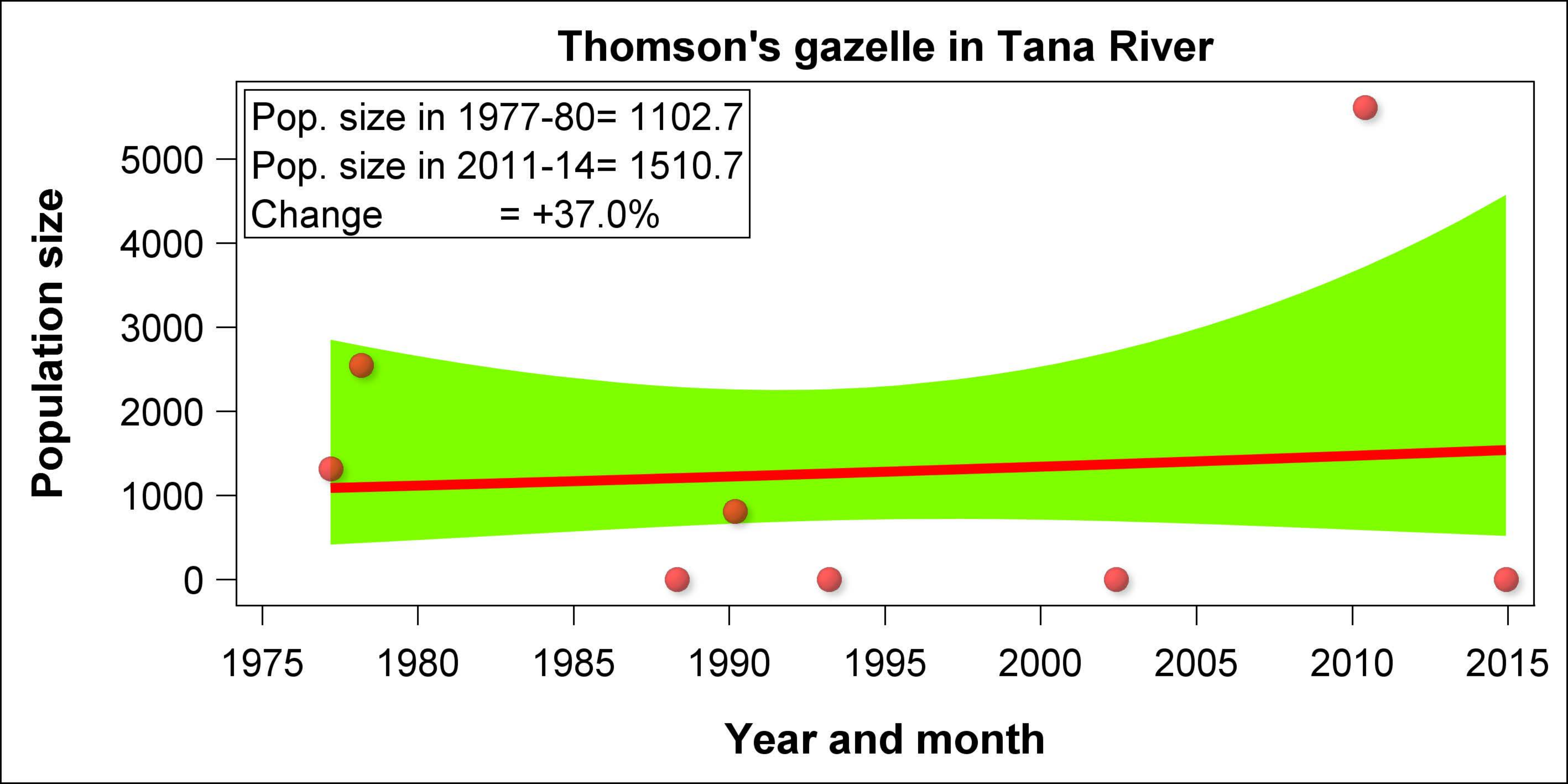
Thomson's gazelle in Tana River
Pop. size in 1977-80= 1102.7
Pop. size in 2011-14= 1510.7
Change = +37.0%
Population size
0
1000
2000
3000
4000
5000
1975
1980
1985
1990
1995
2000
2005
2010
2015
Year and month

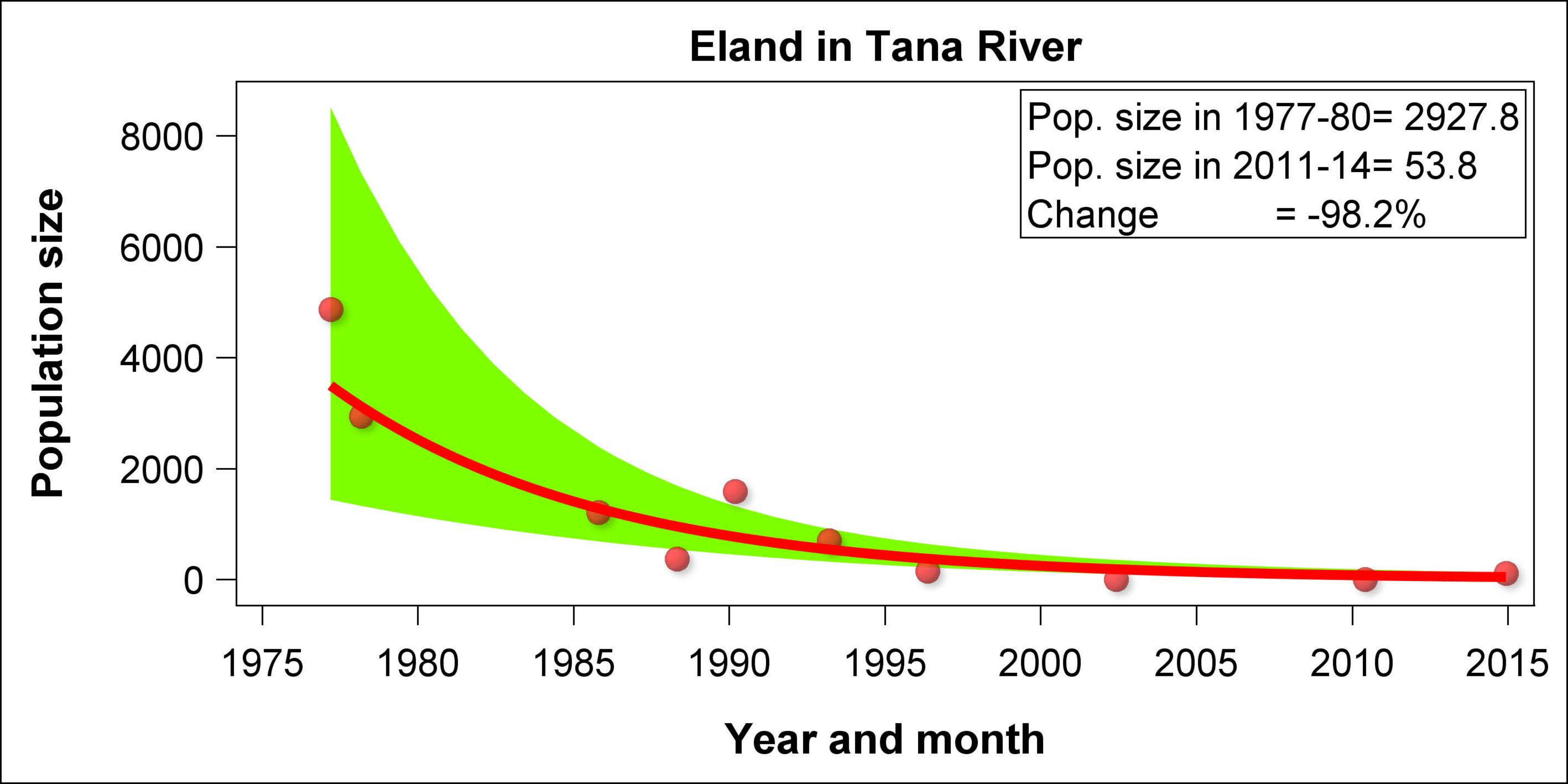

Eland in Tana River
Pop. size in 1977-80= 2927.8
Pop. size in 2011-14= 53.8
Change = -98.2%
Population size
8000
6000
4000
2000
0
1975
1980
1985
1990
1995
2000
2005
2010
2015
Year and month

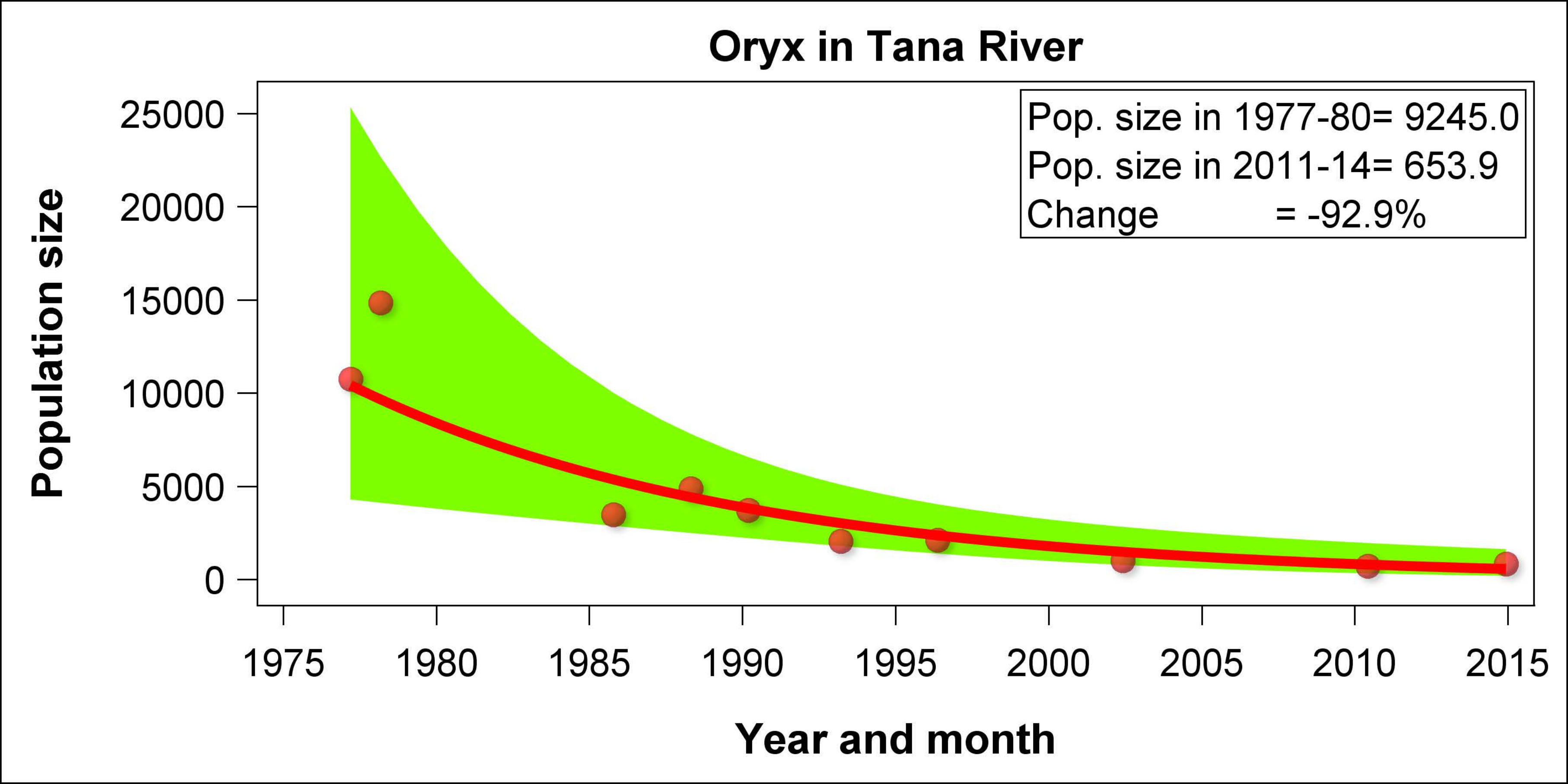
Oryx in Tana River
Pop. size in 1977-80= 9245.0
Pop. size in 2011-14= 653.9
Change = -92.9%
Population size
25000
20000
15000
10000
5000
0
1975
1980
1985
1990
1995
2000
2005
2010
2015
Year and month

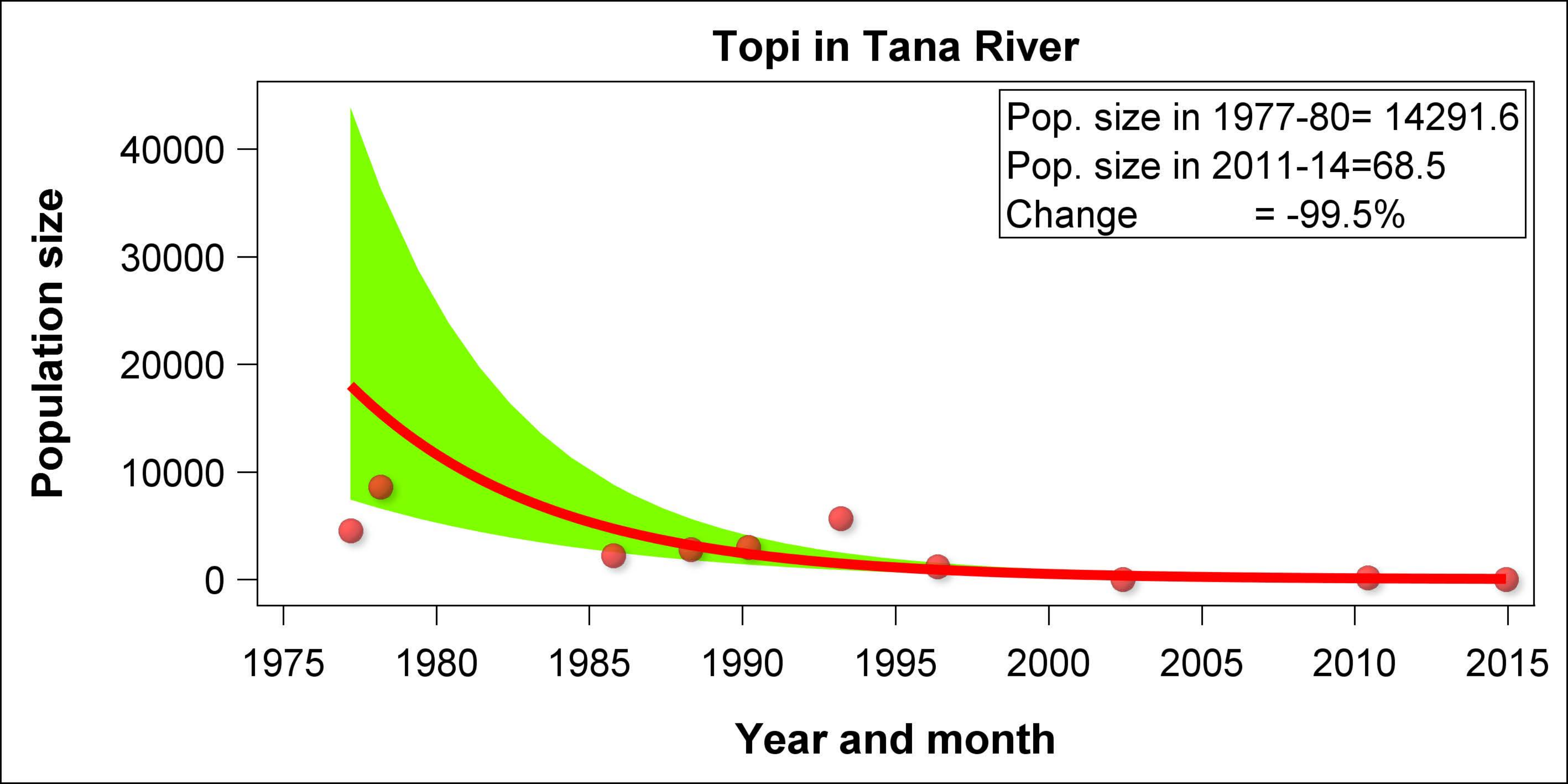

Topi in Tana River
Pop. size in 1977-80= 14291.6
Pop. size in 2011-14=68.5
Change = -99.5%
Population size
40000
30000
20000
10000
0
1975
1980
1985
1990
1995
2000
2005
2010
2015
Year and month

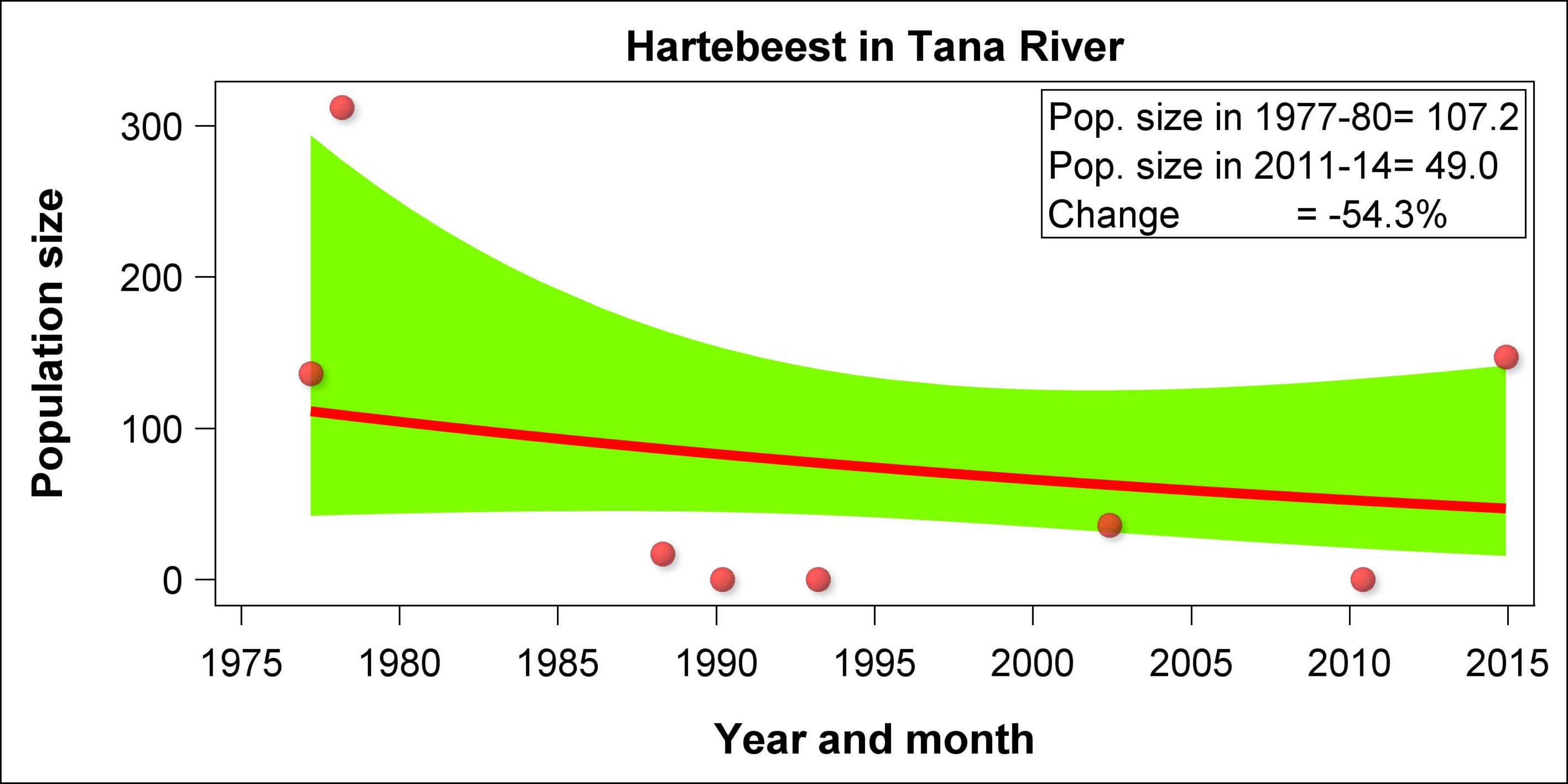

Hartebeest in Tana River
Pop. size in 1977-80= 107.2
Pop. size in 2011-14= 49.0
Change = -54.3%
Population size
300
200
100
0
1975
1980
1985
1990
1995
2000
2005
2010
2015
Year and month

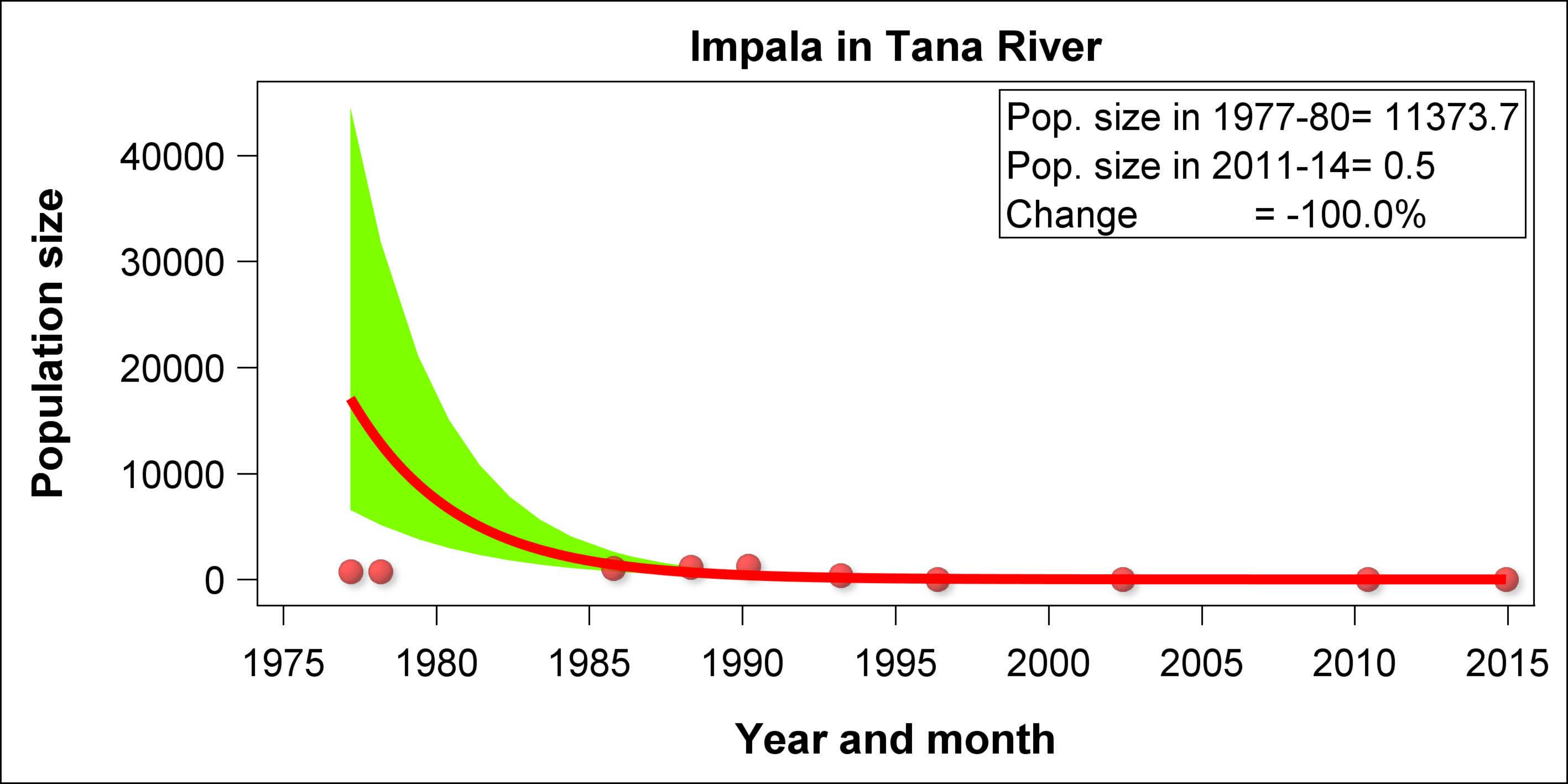

Impala in Tana River
Pop. size in 1977-80= 11373.7
Pop. size in 2011-14= 0.5
Change = -100.0%
Population size
40000
30000
20000
10000
0
1975
1980
1985
1990
1995
2000
2005
2010
2015
Year and month

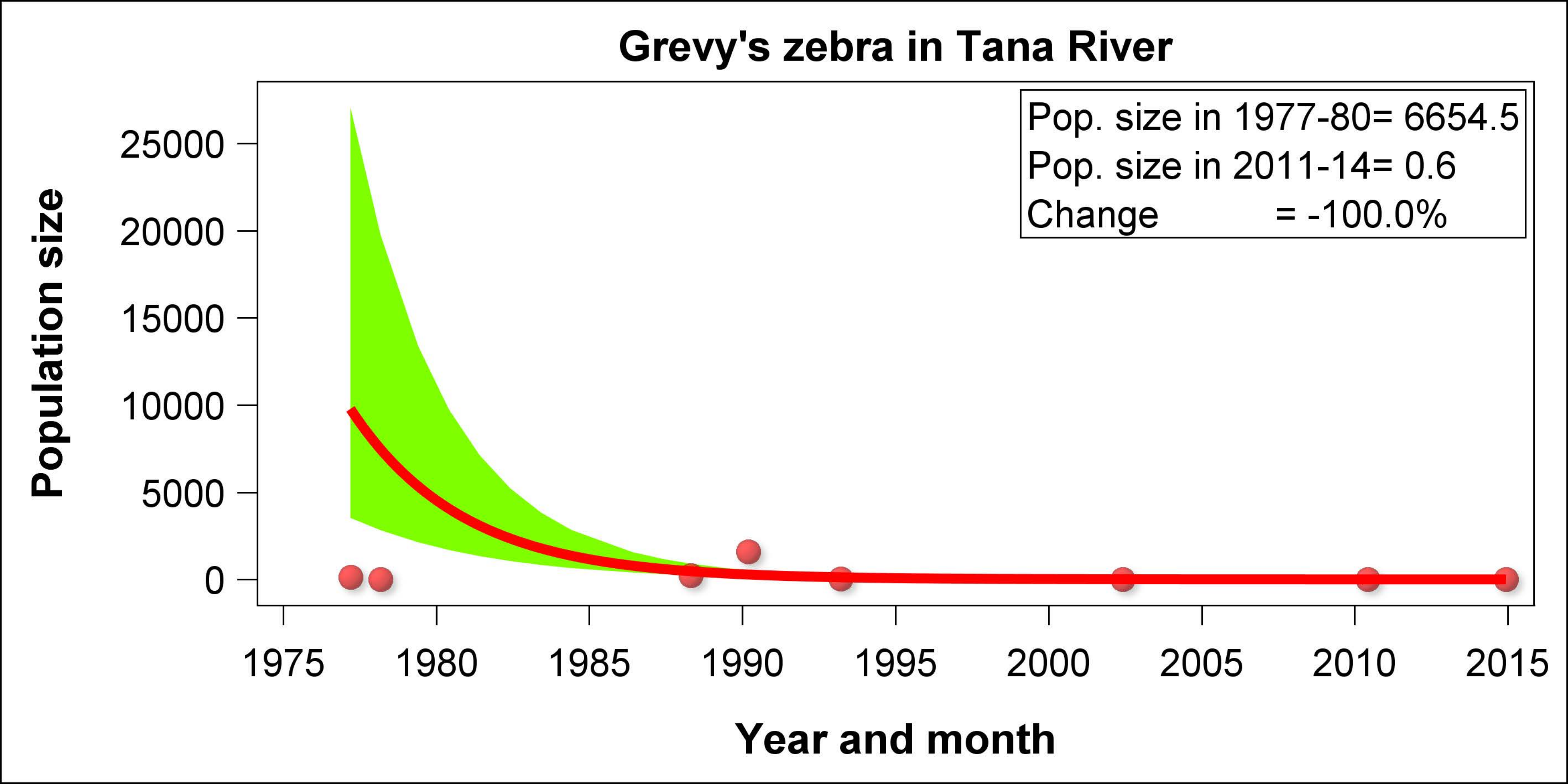
Grevy's zebra in Tana River
Pop. size in 1977-80= 6654.5
Pop. size in 2011-14= 0.6
Change = -100.0%
Population size
25000
20000
15000
10000
5000
0
1975
1980
1985
1990
1995
2000
2005
2010
2015
Year and month

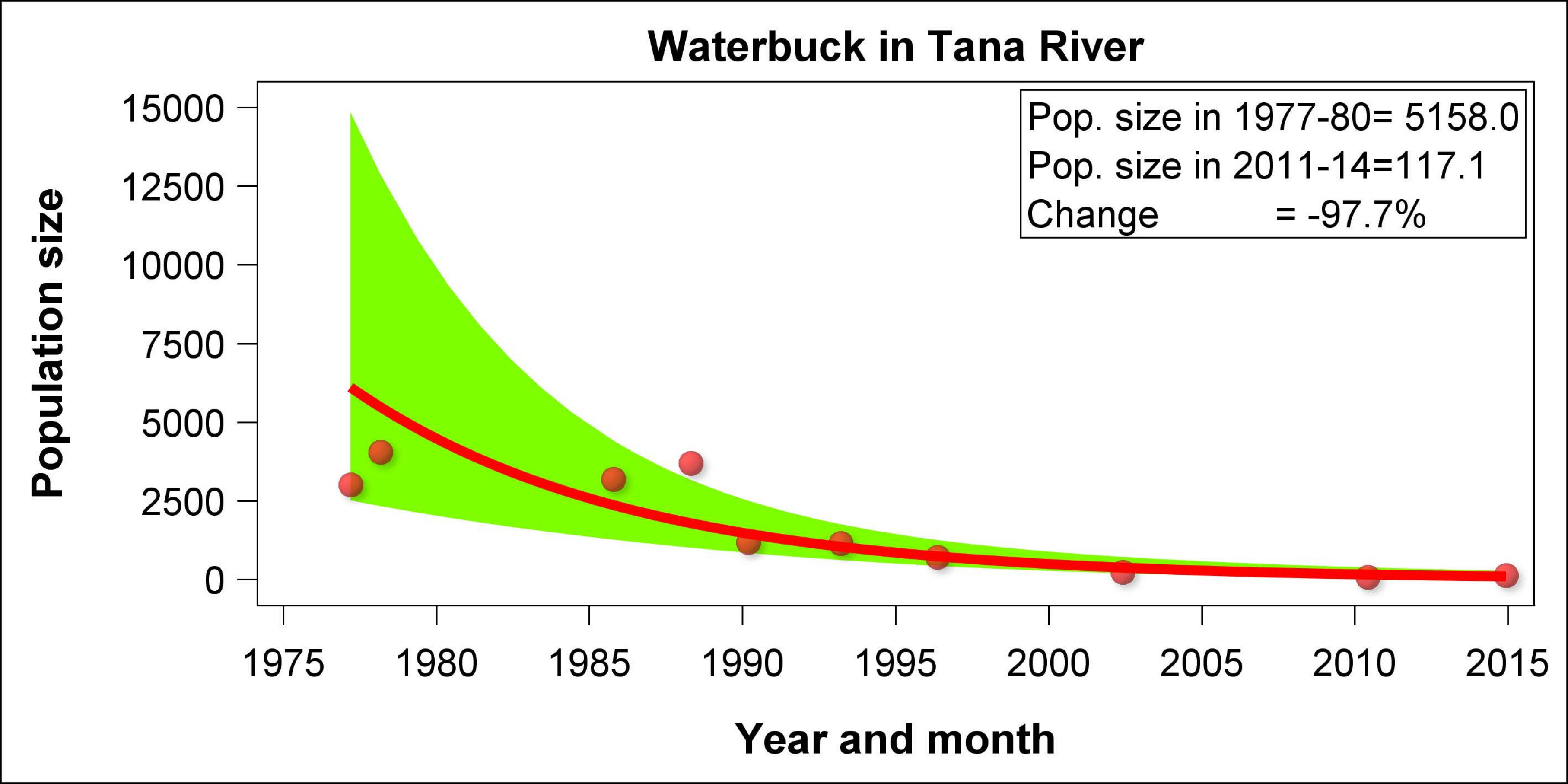

Waterbuck in Tana River
Pop. size in 1977-80= 5158.0
Pop. size in 2011-14=117.1
Change = -97.7%
Population size
15000
12500
10000
7500
5000
2500
0
1975
1980
1985
1990
1995
2000
2005
2010
2015
Year and month
