# Supplementary material for: Extreme Wildlife Declines and Concurrent Increase in Livestock Numbers in Kenya: What Are the Causes?
Source: PLoS One. 2016 Sep 27;11(9):e0163249. doi: 10.1371/journal.pone.0163249 (PMC5039022; doi:10.1371/journal.pone.0163249)
Supplement: S10 Fig — The solid red line is the fitted trend curve and the shaded chartreuse band is the pointwise 95% confidence band. The estimated average population size in 1977–1980 and 2011–2013 and the percentage change in population size between the two periods are provided in the inset. (PDF) [file pone.0163249.s020.pdf]

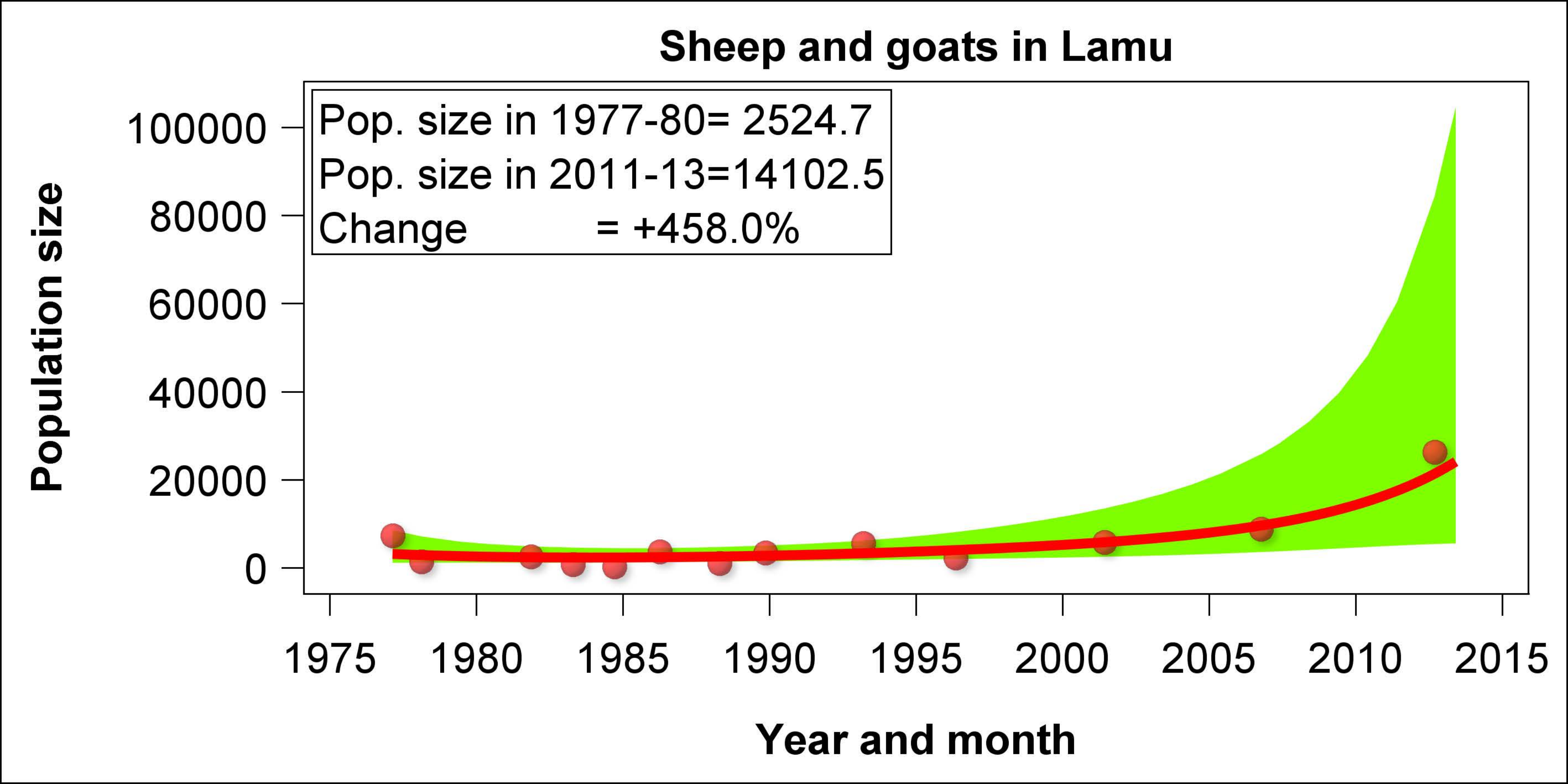

Sheep and goats in Lamu
Pop. size in 1977-80= 2524.7
Pop. size in 2011-13=14102.5
Change = +458.0%
Population size
100000
80000
60000
40000
20000
0
1975
1980
1985
1990
1995
2000
2005
2010
2015
Year and month

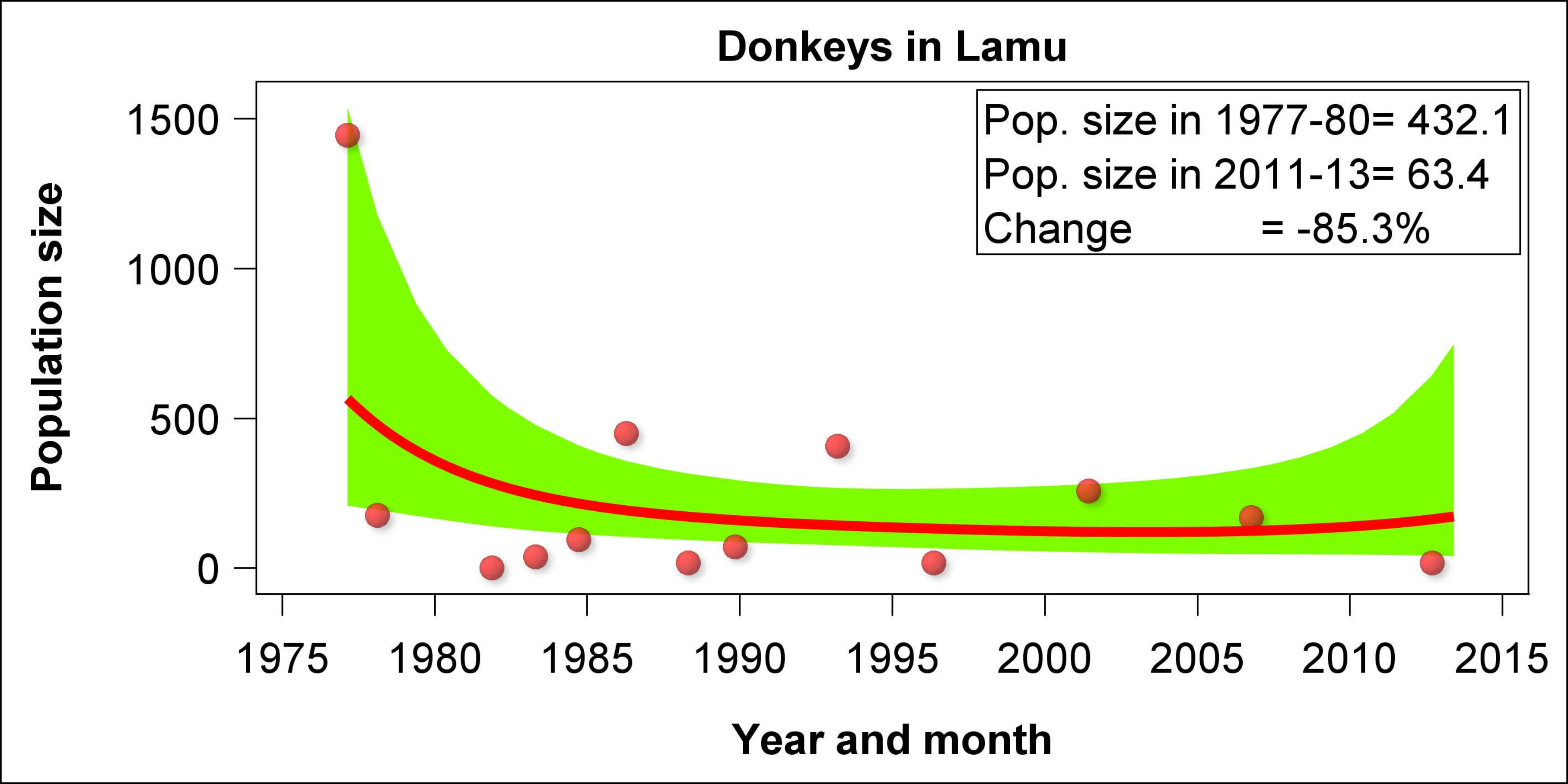
Donkeys in Lamu
Pop. size in 1977-80= 432.1
Pop. size in 2011-13= 63.4
Change = -85.3%
Population size
1500
1000
500
0
1975
1980
1985
1990
1995
2000
2005
2010
2015
Year and month

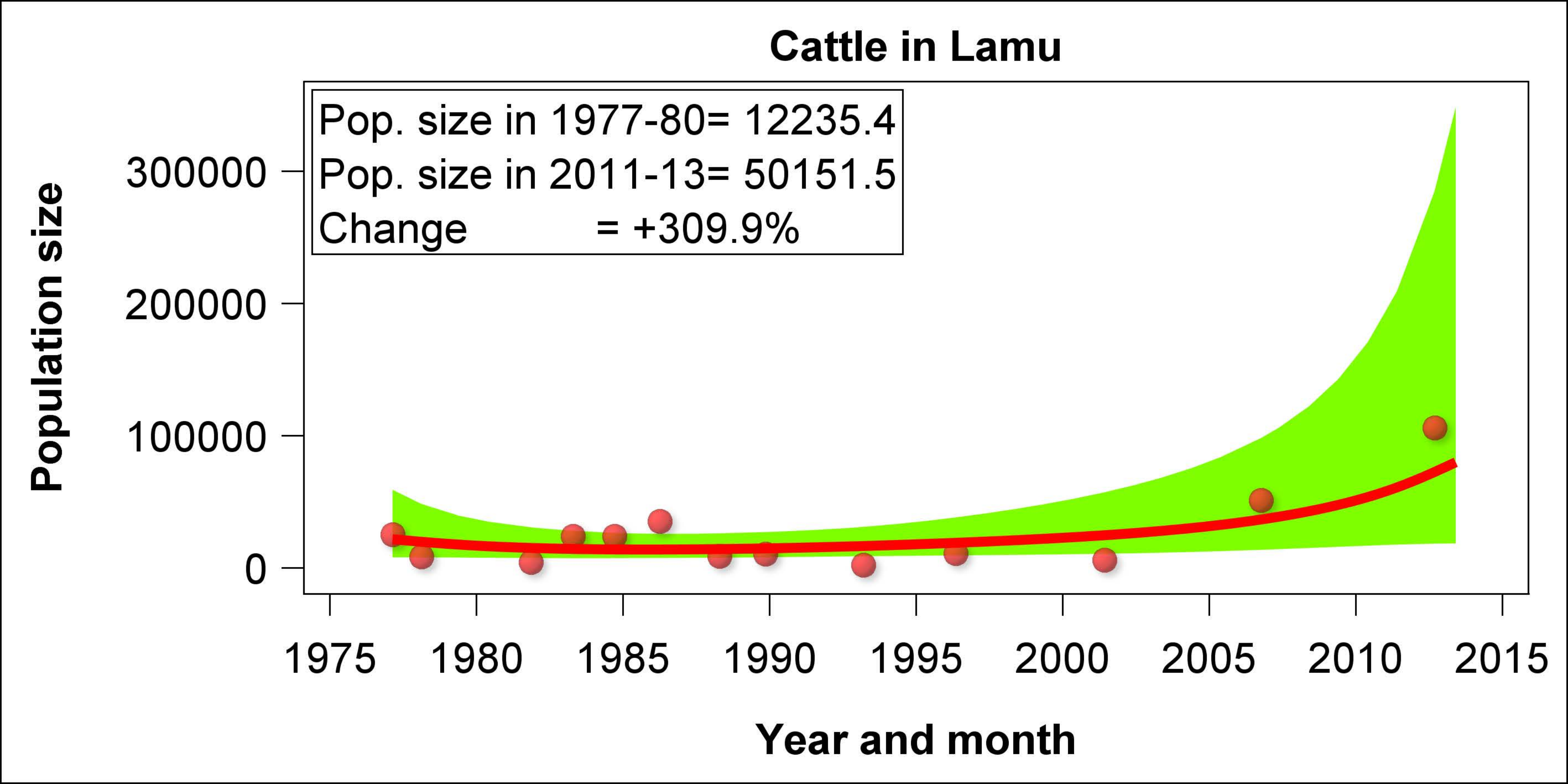

Cattle in Lamu
Pop. size in 1977-80= 12235.4
Pop. size in 2011-13= 50151.5
Change = +309.9%
Population size
300000
200000
100000
0
1975
1980
1985
1990
1995
2000
2005
2010
2015
Year and month

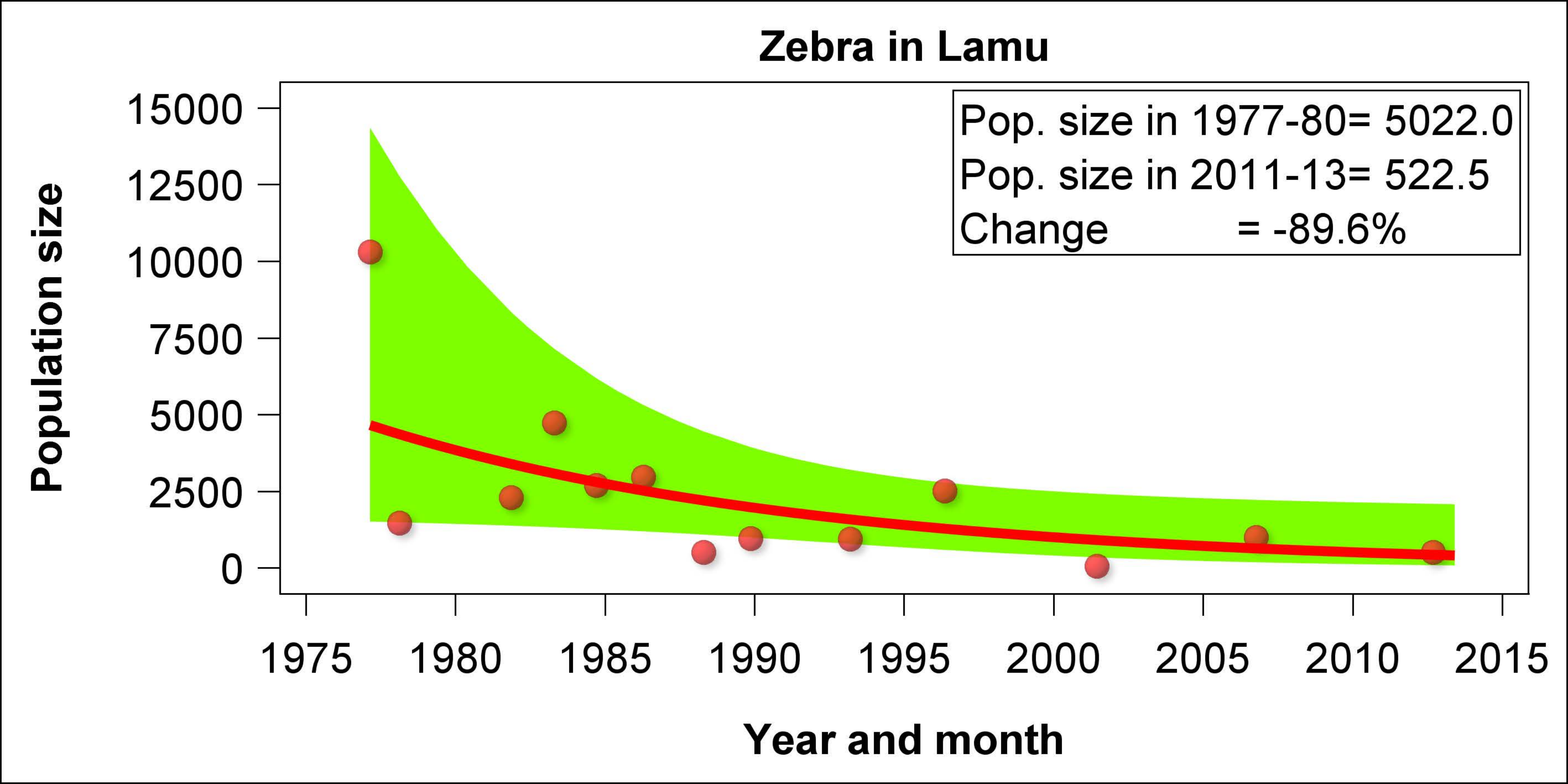
Zebra in Lamu
Pop. size in 1977-80= 5022.0
Pop. size in 2011-13= 522.5
Change = -89.6%
Population size
15000
12500
10000
7500
5000
2500
0
1975
1980
1985
1990
1995
2000
2005
2010
2015
Year and month

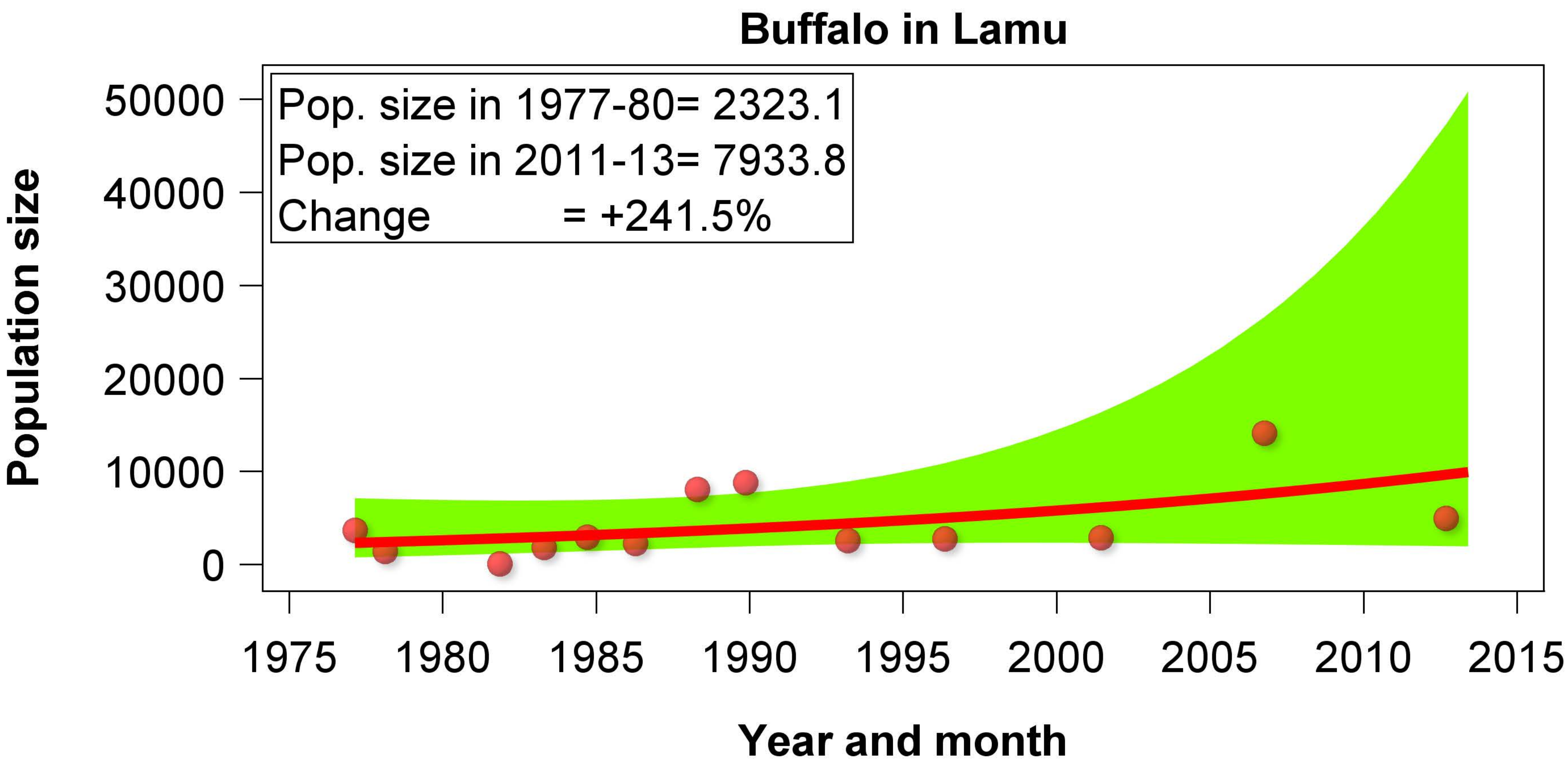

Buffalo in Lamu
Pop. size in 1977-80= 2323.1
Pop. size in 2011-13= 7933.8
Change = +241.5%
Population size
0
10000
20000
30000
40000
50000
1975
1980
1985
1990
1995
2000
2005
2010
2015
Year and month

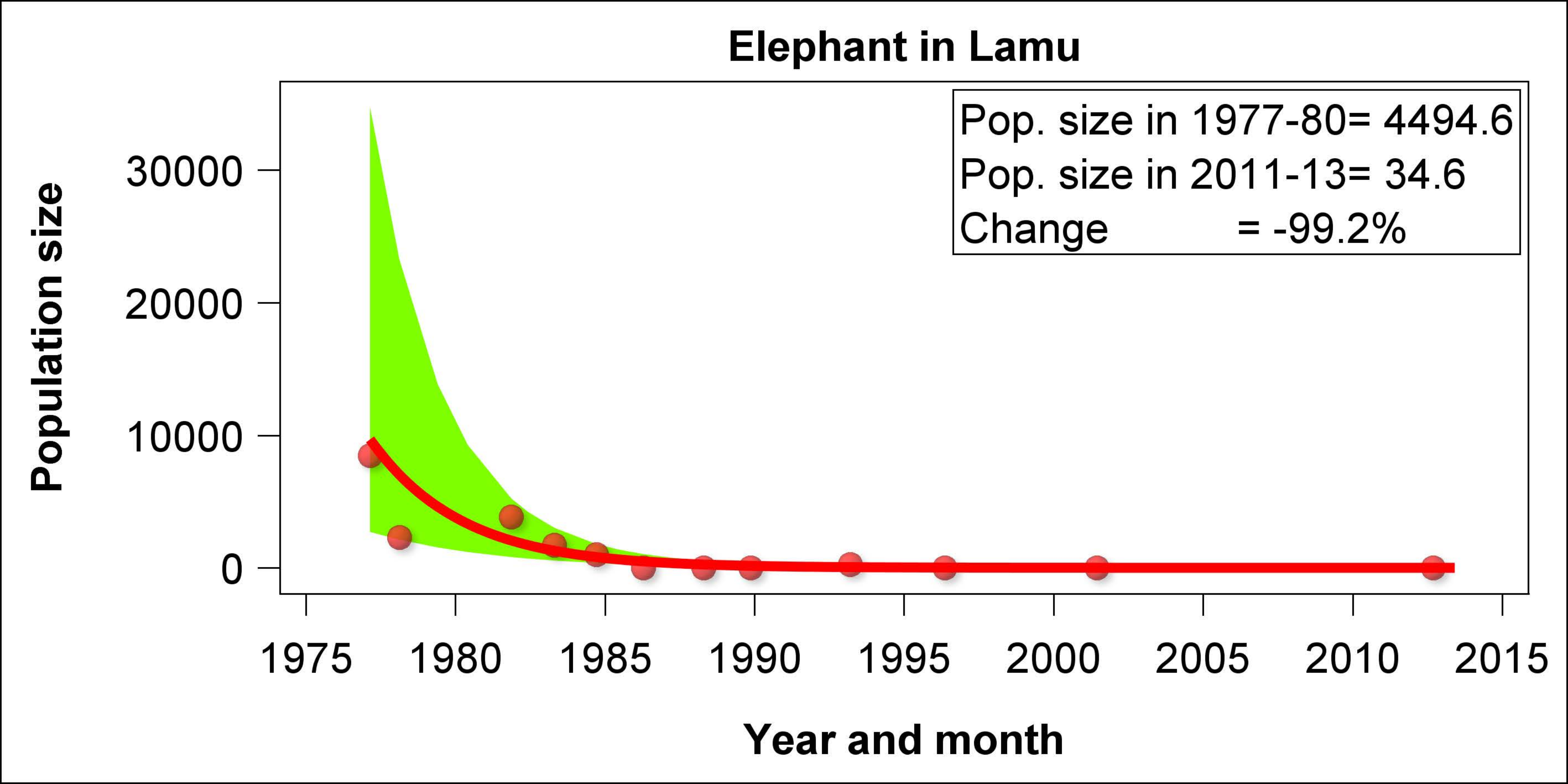

Elephant in Lamu
Pop. size in 1977-80= 4494.6
Pop. size in 2011-13= 34.6
Change = -99.2%
Population size
30000
20000
10000
0
1975
1980
1985
1990
1995
2000
2005
2010
2015
Year and month

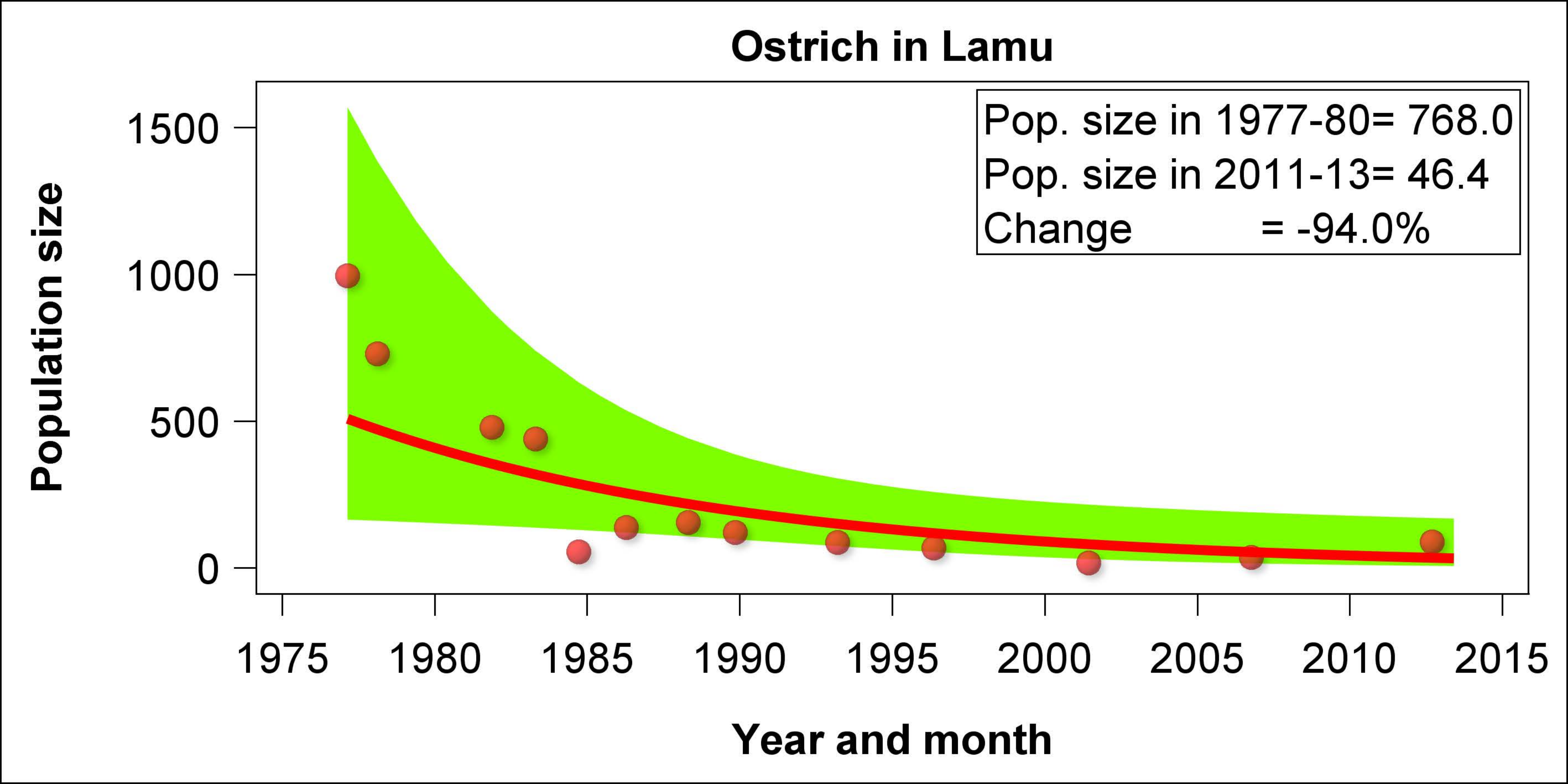
Ostrich in Lamu
Pop. size in 1977-80= 768.0
Pop. size in 2011-13= 46.4
Change = -94.0%
Population size
0
500
1000
1500
1975
1980
1985
1990
1995
2000
2005
2010
2015
Year and month

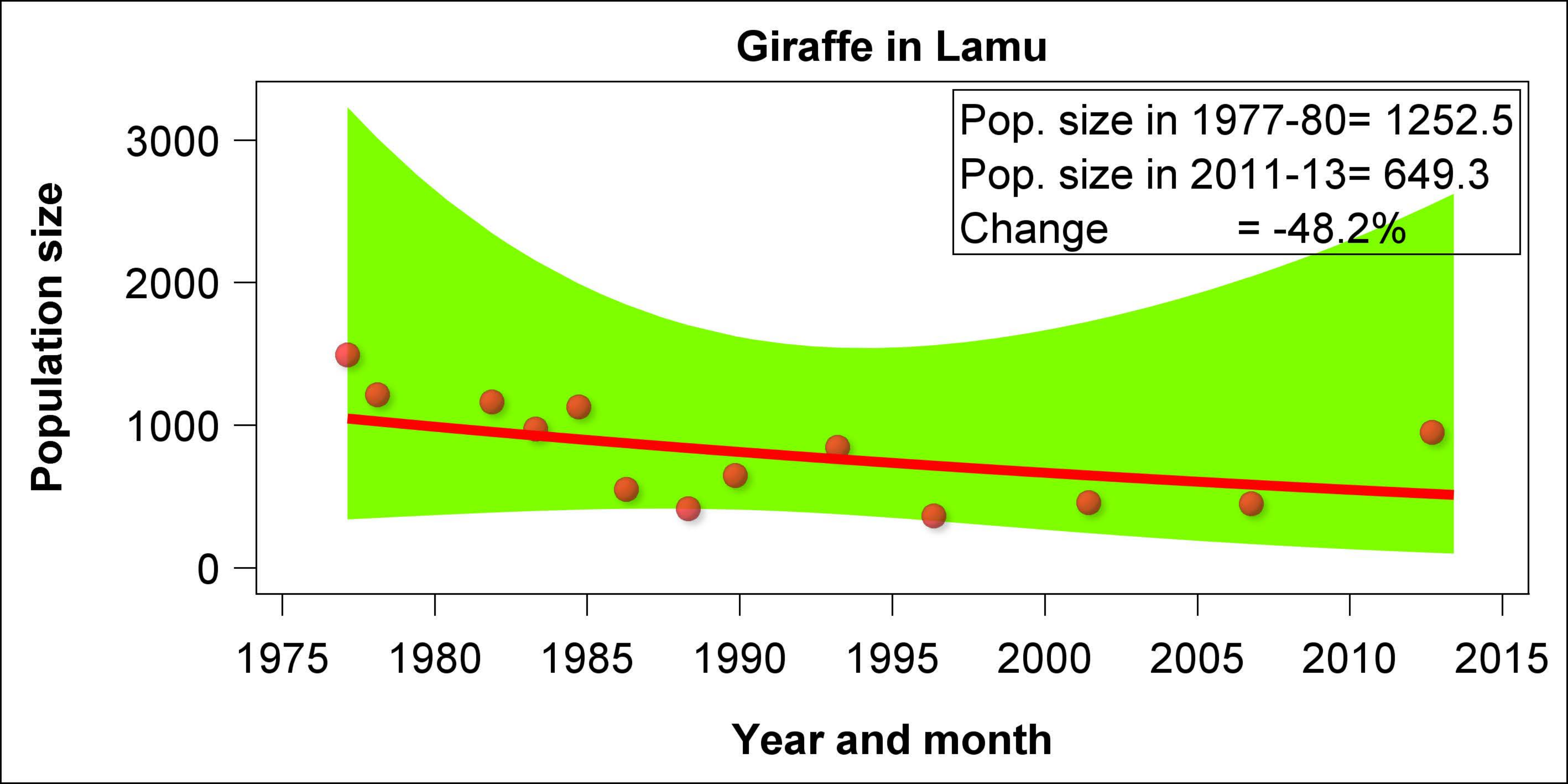
Giraffe in Lamu
Pop. size in 1977-80= 1252.5
Pop. size in 2011-13= 649.3
Change = -48.2%
Population size
3000
2000
1000
0
1975
1980
1985
1990
1995
2000
2005
2010
2015
Year and month

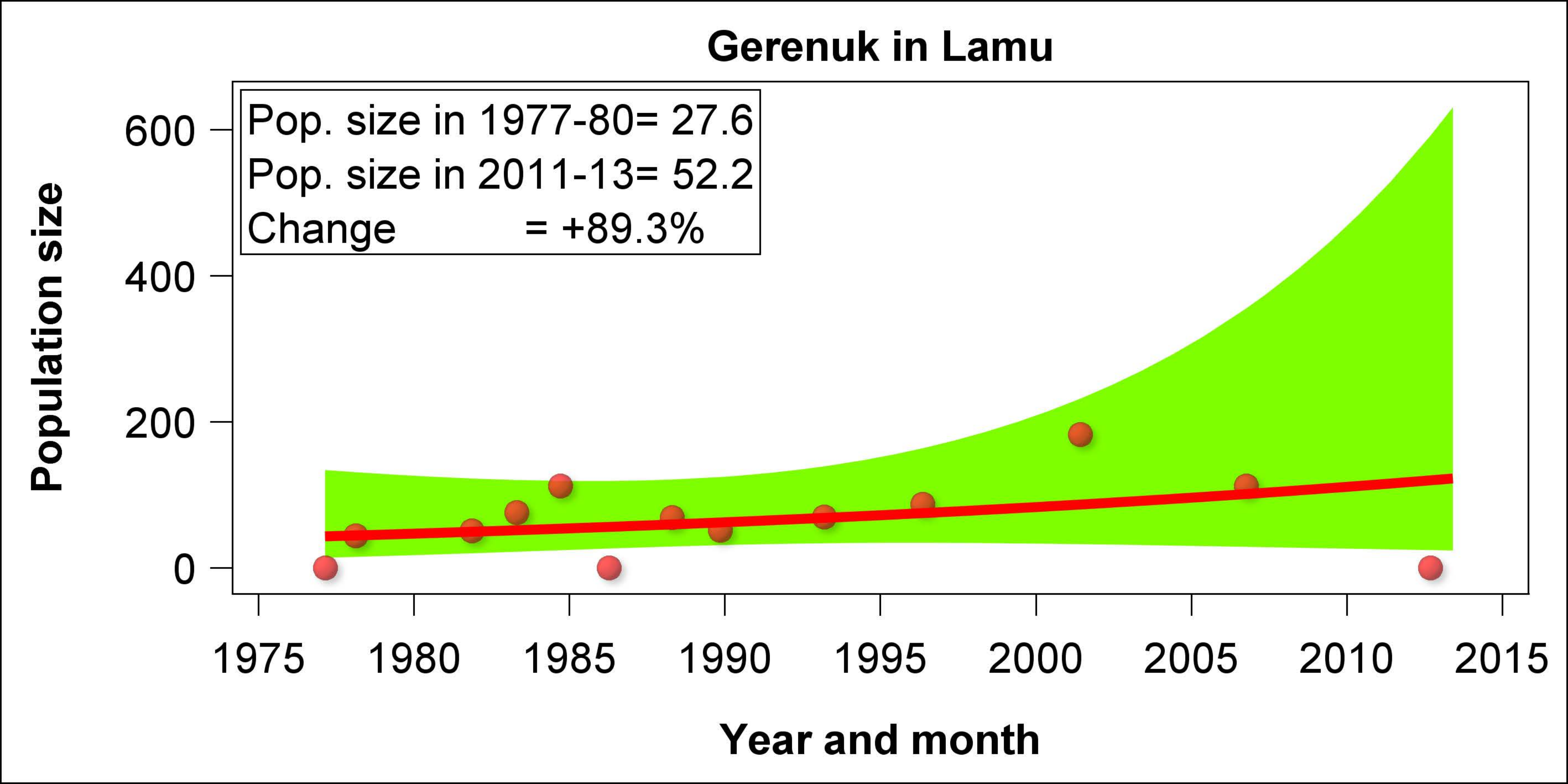

Gerenuk in Lamu
Pop. size in 1977-80= 27.6
Pop. size in 2011-13= 52.2
Change = +89.3%
Population size
0
200
400
600
1975
1980
1985
1990
1995
2000
2005
2010
2015
Year and month

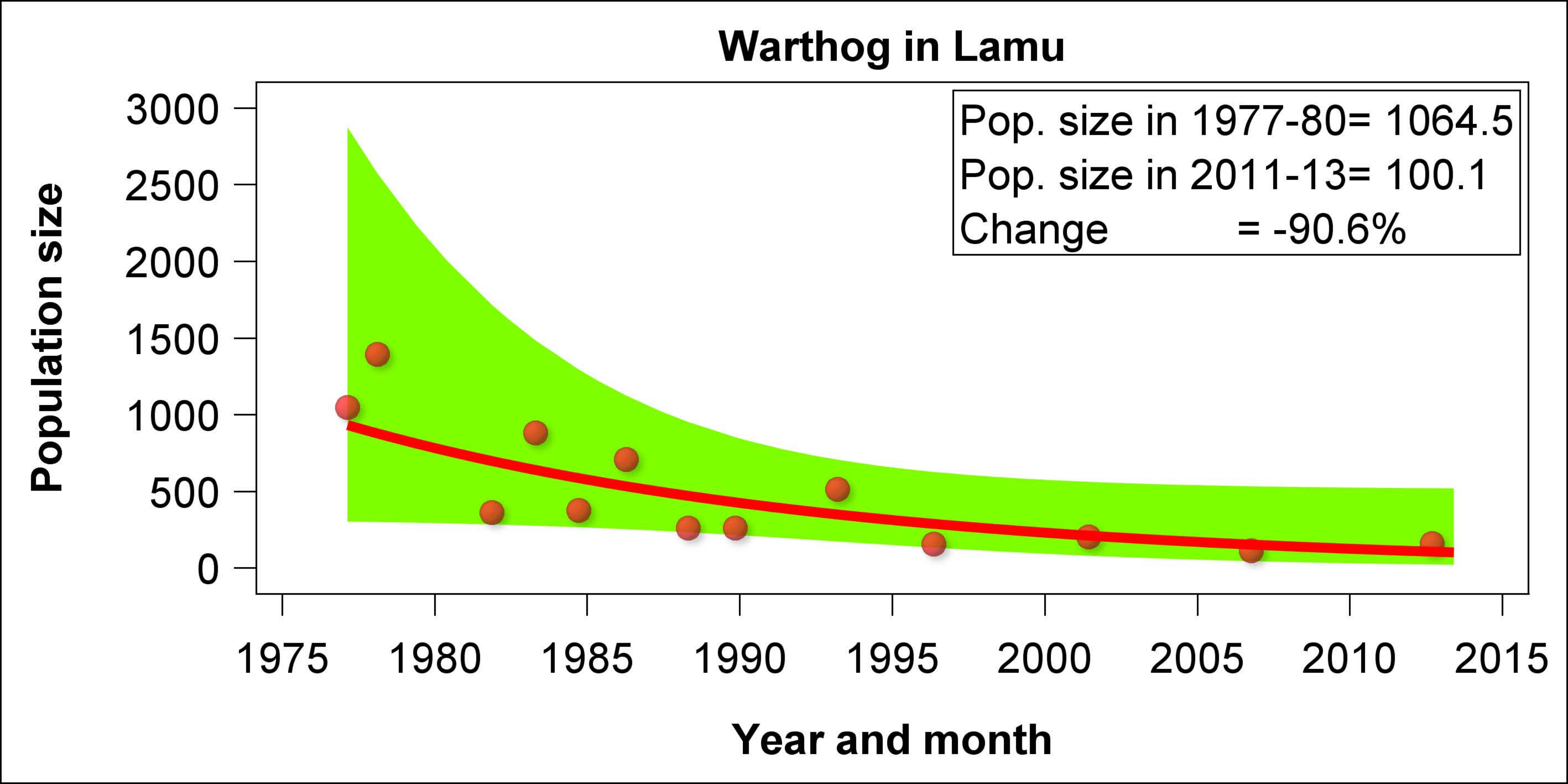

Warthog in Lamu
Pop. size in 1977-80= 1064.5
Pop. size in 2011-13= 100.1
Change = -90.6%
Population size
0
500
1000
1500
2000
2500
3000
1975
1980
1985
1990
1995
2000
2005
2010
2015
Year and month

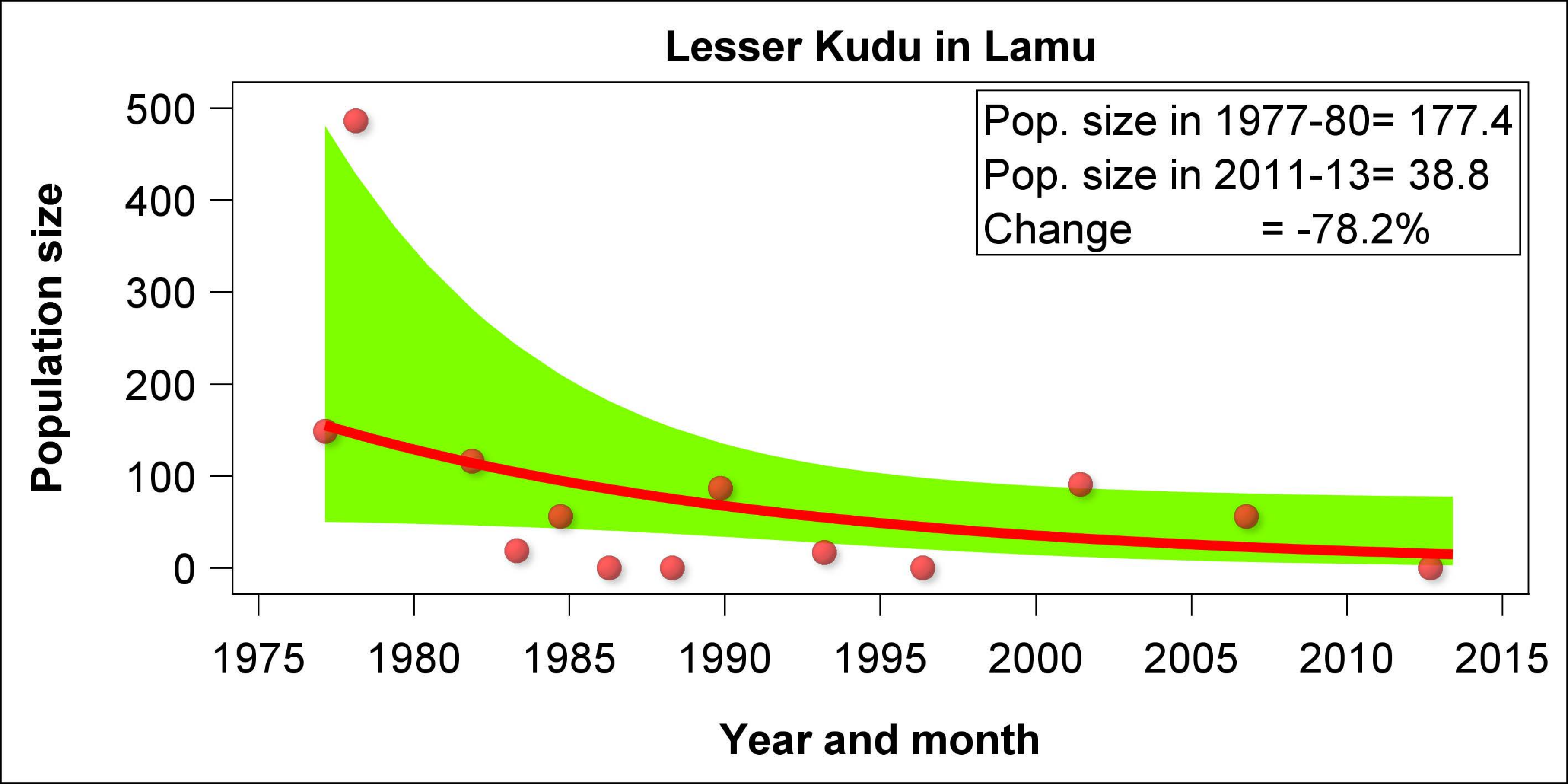

Lesser Kudu in Lamu
Pop. size in 1977-80= 177.4
Pop. size in 2011-13= 38.8
Change = -78.2%
Population size
0
100
200
300
400
500
1975
1980
1985
1990
1995
2000
2005
2010
2015
Year and month

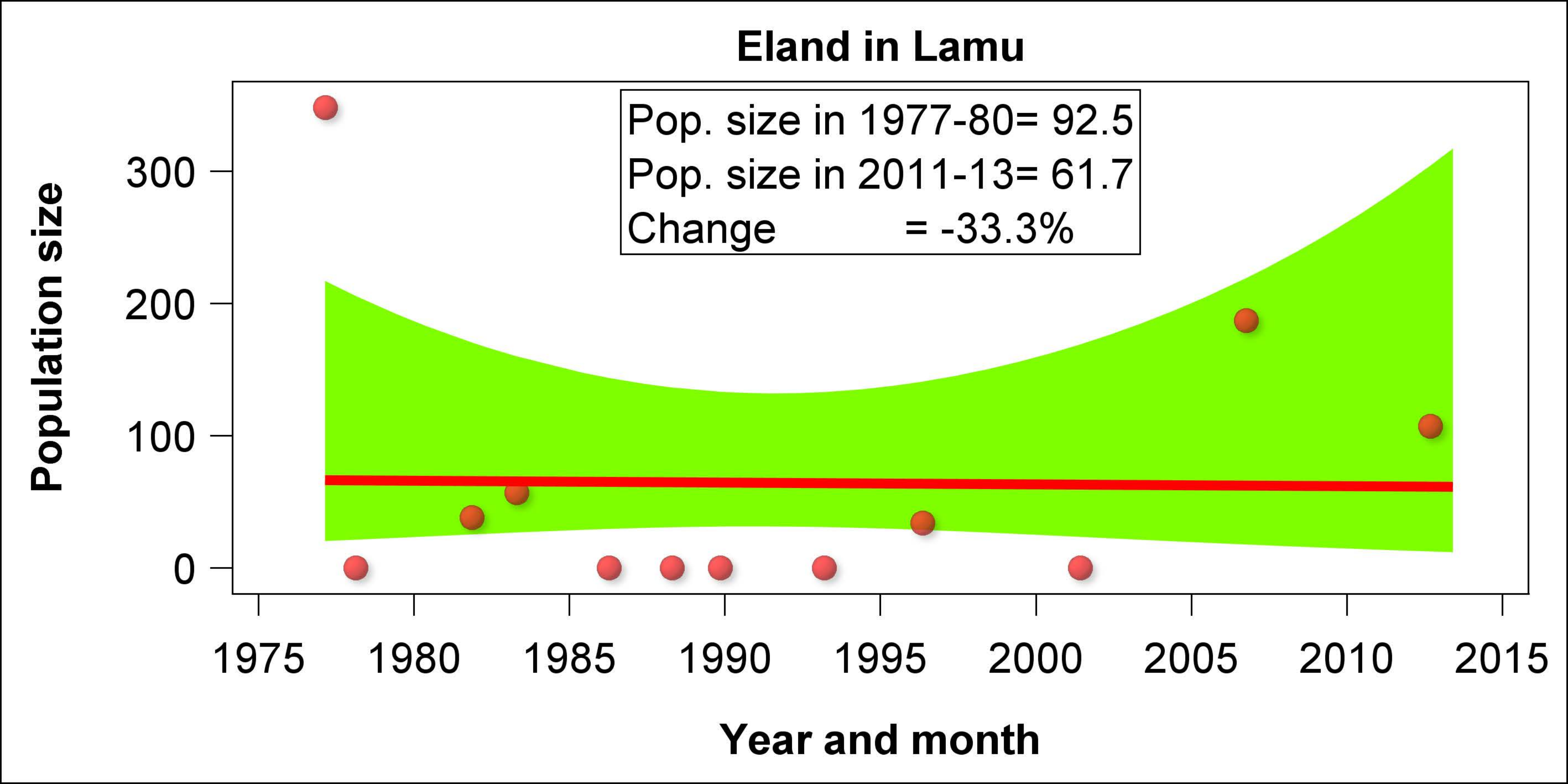
Eland in Lamu
Pop. size in 1977-80= 92.5
Pop. size in 2011-13= 61.7
Change = -33.3%
Population size
300
200
100
0
1975
1980
1985
1990
1995
2000
2005
2010
2015
Year and month

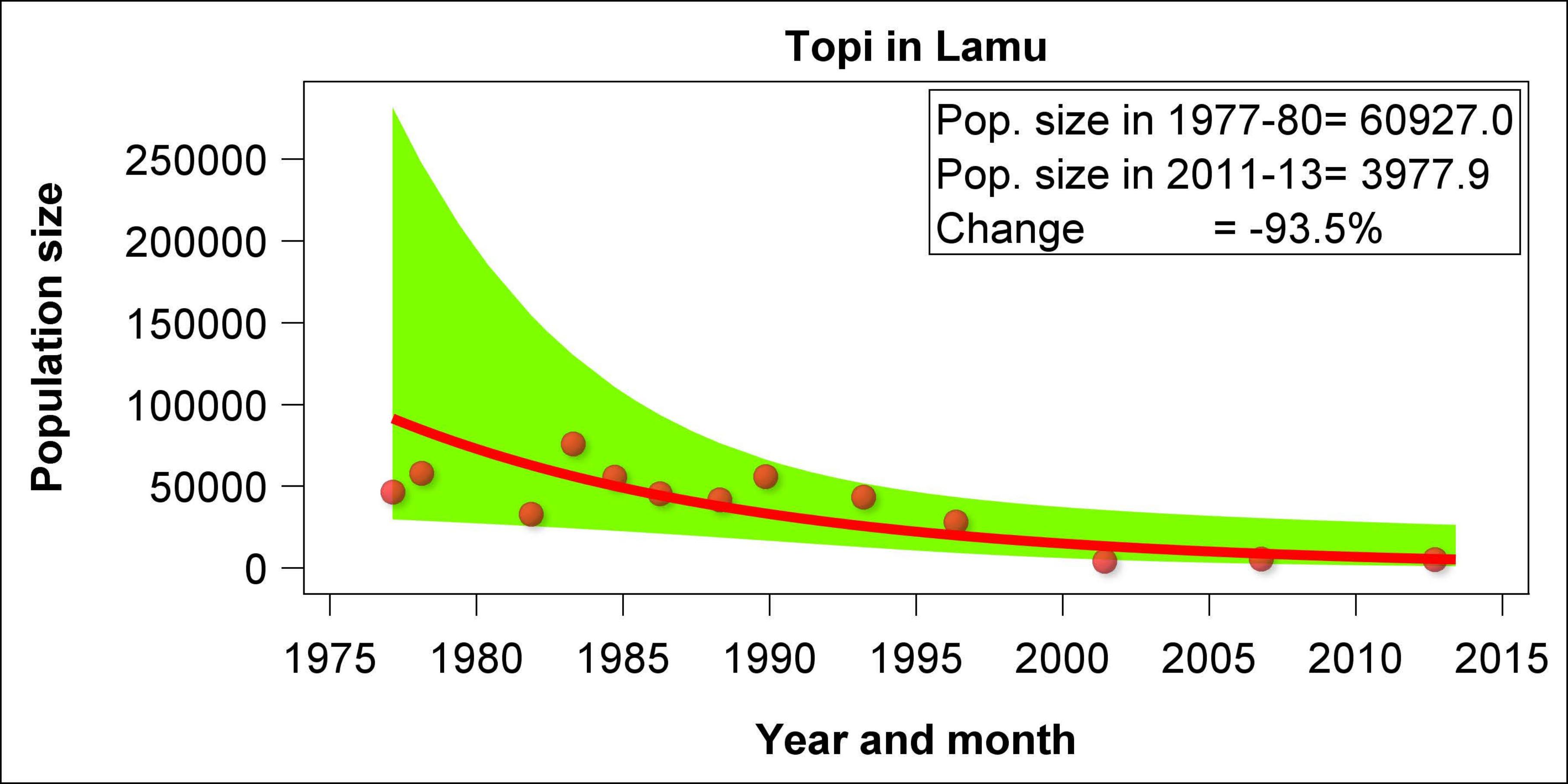
Topi in Lamu
Pop. size in 1977-80= 60927.0
Pop. size in 2011-13= 3977.9
Change = -93.5%
Population size
250000
200000
150000
100000
50000
0
1975
1980
1985
1990
1995
2000
2005
2010
2015
Year and month

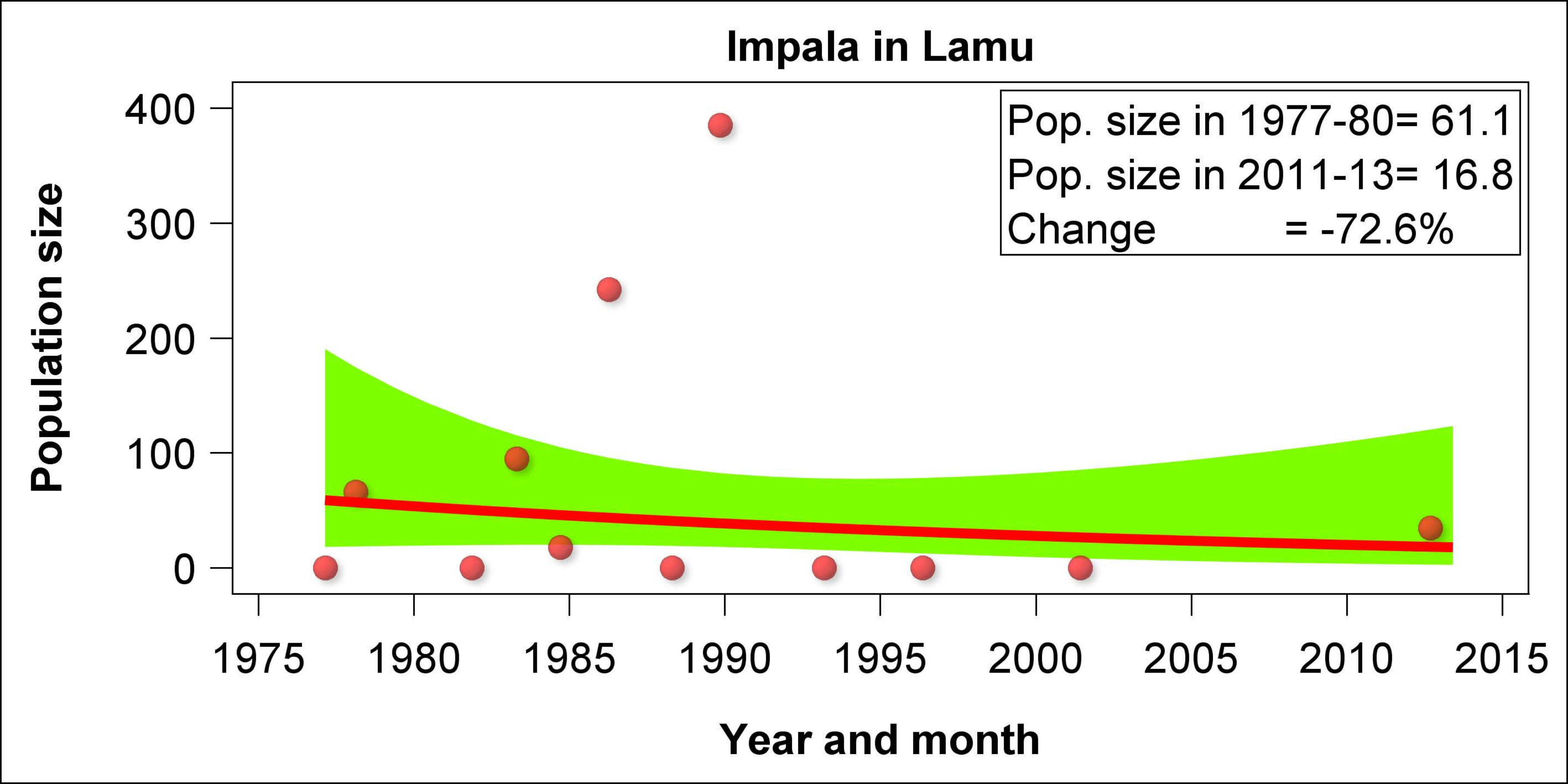
Impala in Lamu
Pop. size in 1977-80= 61.1
Pop. size in 2011-13= 16.8
Change = -72.6%
Population size
0
100
200
300
400
1975
1980
1985
1990
1995
2000
2005
2010
2015
Year and month

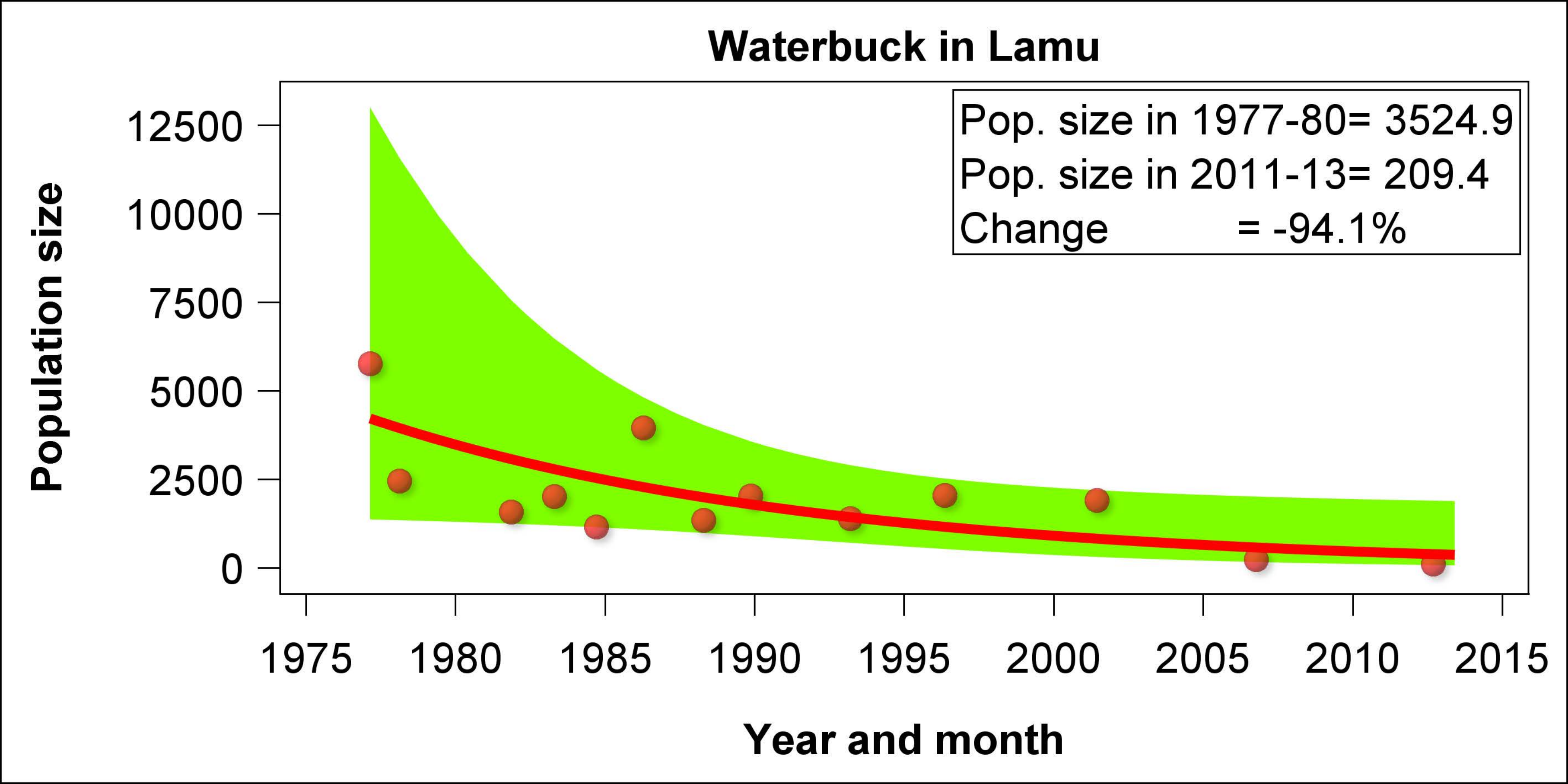
Waterbuck in Lamu
Pop. size in 1977-80= 3524.9
Pop. size in 2011-13= 209.4
Change = -94.1%
Population size
12500
10000
7500
5000
2500
0
1975
1980
1985
1990
1995
2000
2005
2010
2015
Year and month
