# Supplementary material for: Extreme Wildlife Declines and Concurrent Increase in Livestock Numbers in Kenya: What Are the Causes?
Source: PLoS One. 2016 Sep 27;11(9):e0163249. doi: 10.1371/journal.pone.0163249 (PMC5039022; doi:10.1371/journal.pone.0163249)
Supplement: S11 Fig — The solid red line is the fitted trend curve and the shaded chartreuse band is the pointwise 95% confidence band. The estimated average population size in 1977–1980 and 2011–2013 and the percentage change in population size between the two periods are provided in the inset. (PDF) [file pone.0163249.s021.pdf]

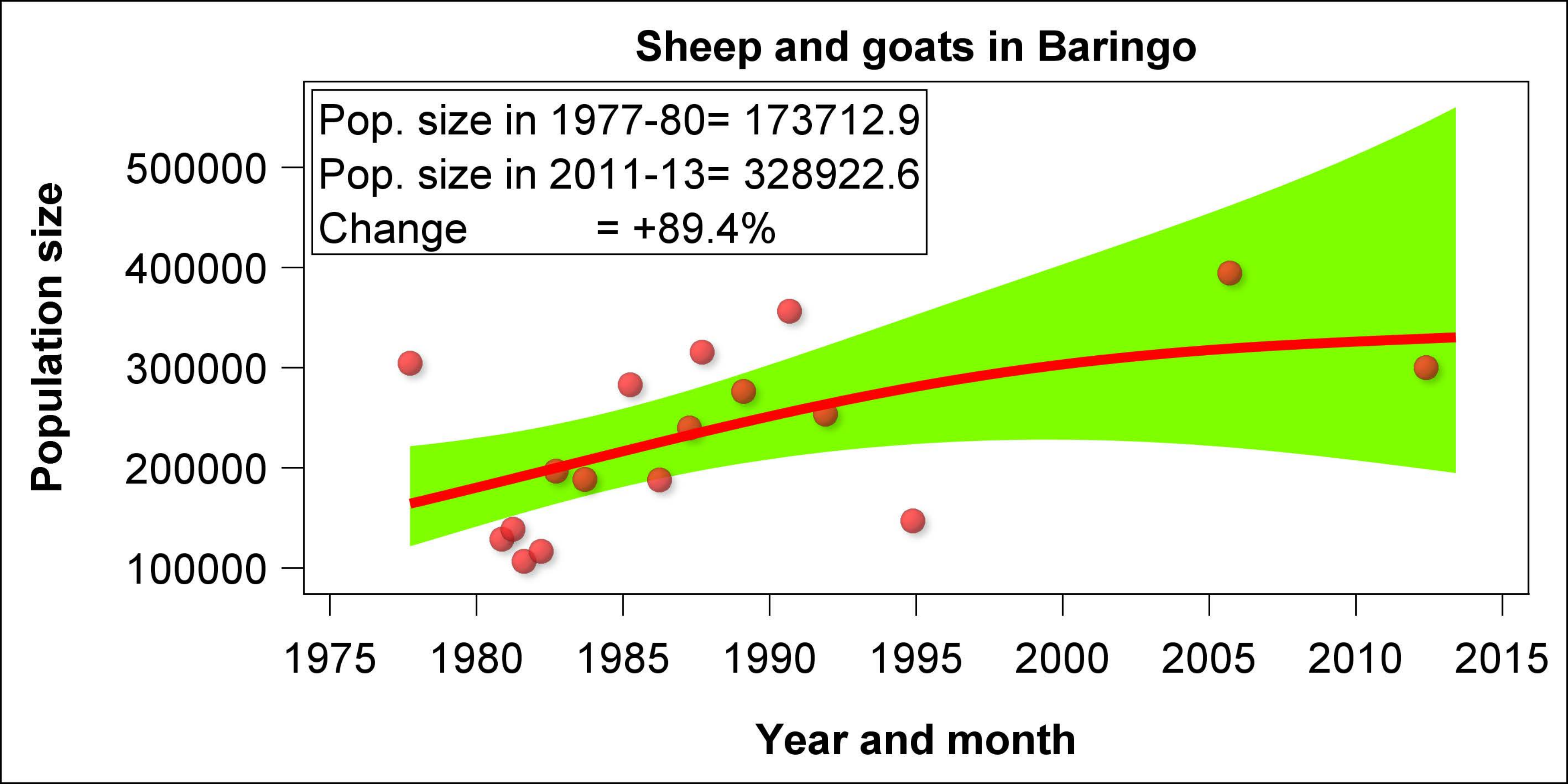

Sheep and goats in Baringo
Pop. size in 1977-80= 173712.9
Pop. size in 2011-13= 328922.6
Change = +89.4%
Population size
500000
400000
300000
200000
100000
1975
1980
1985
1990
1995
2000
2005
2010
2015
Year and month

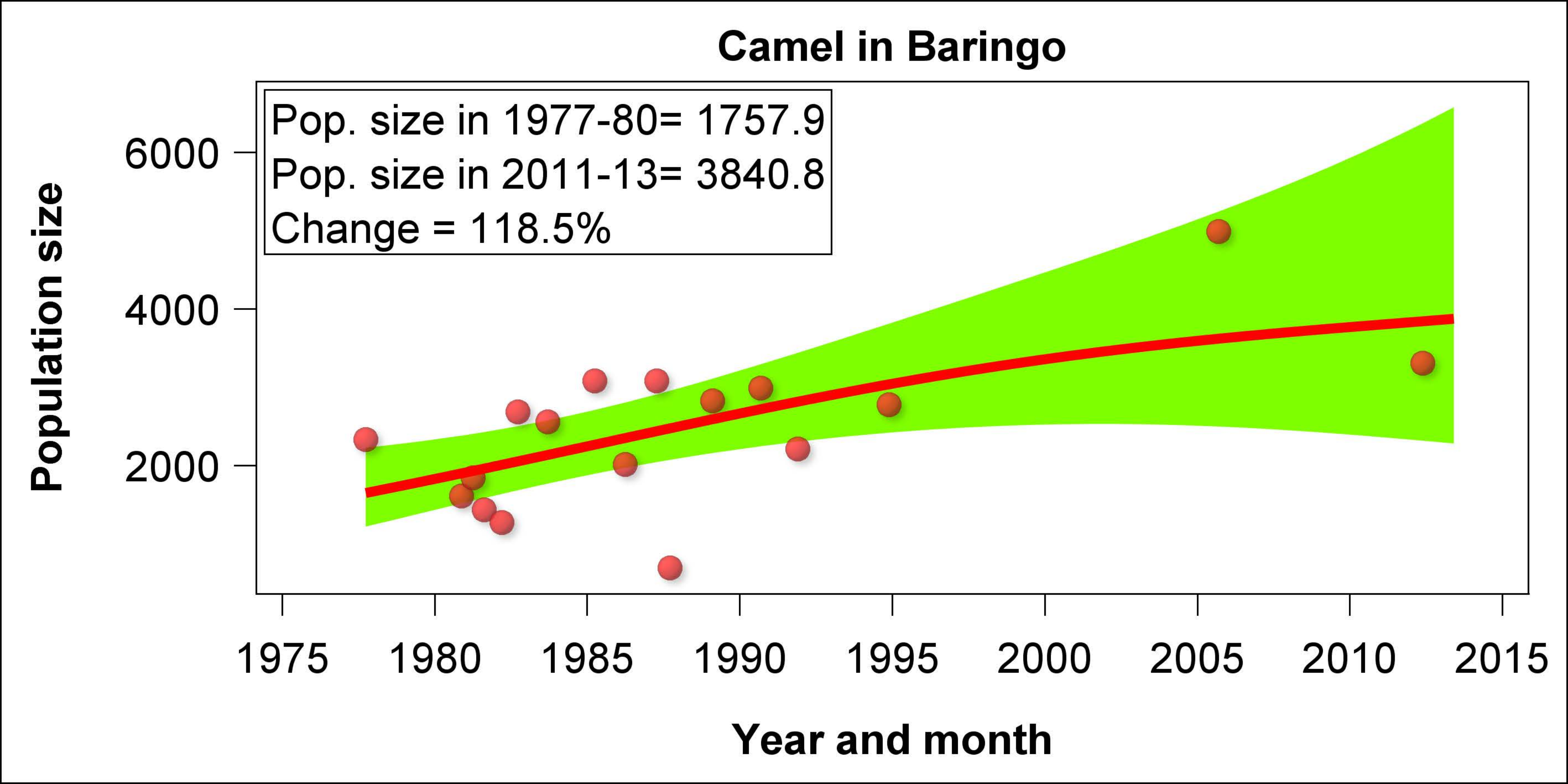

Camel in Baringo
Pop. size in 1977-80= 1757.9
Pop. size in 2011-13= 3840.8
Change = 118.5%
Population size
6000
4000
2000
1975
1980
1985
1990
1995
2000
2005
2010
2015
Year and month

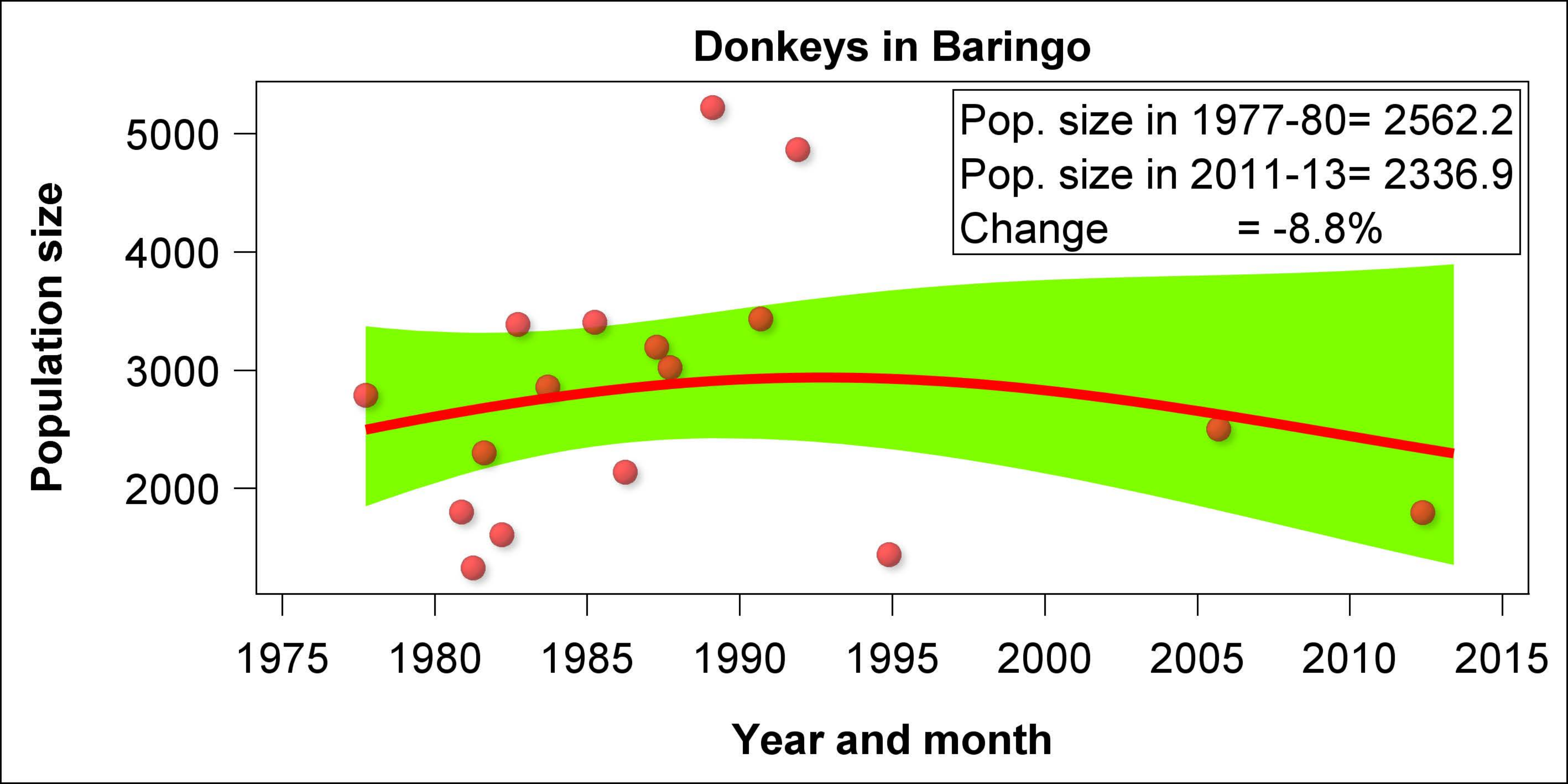

Donkeys in Baringo
Pop. size in 1977-80= 2562.2
Pop. size in 2011-13= 2336.9
Change = -8.8%
Population size
5000
4000
3000
2000
1975
1980
1985
1990
1995
2000
2005
2010
2015
Year and month

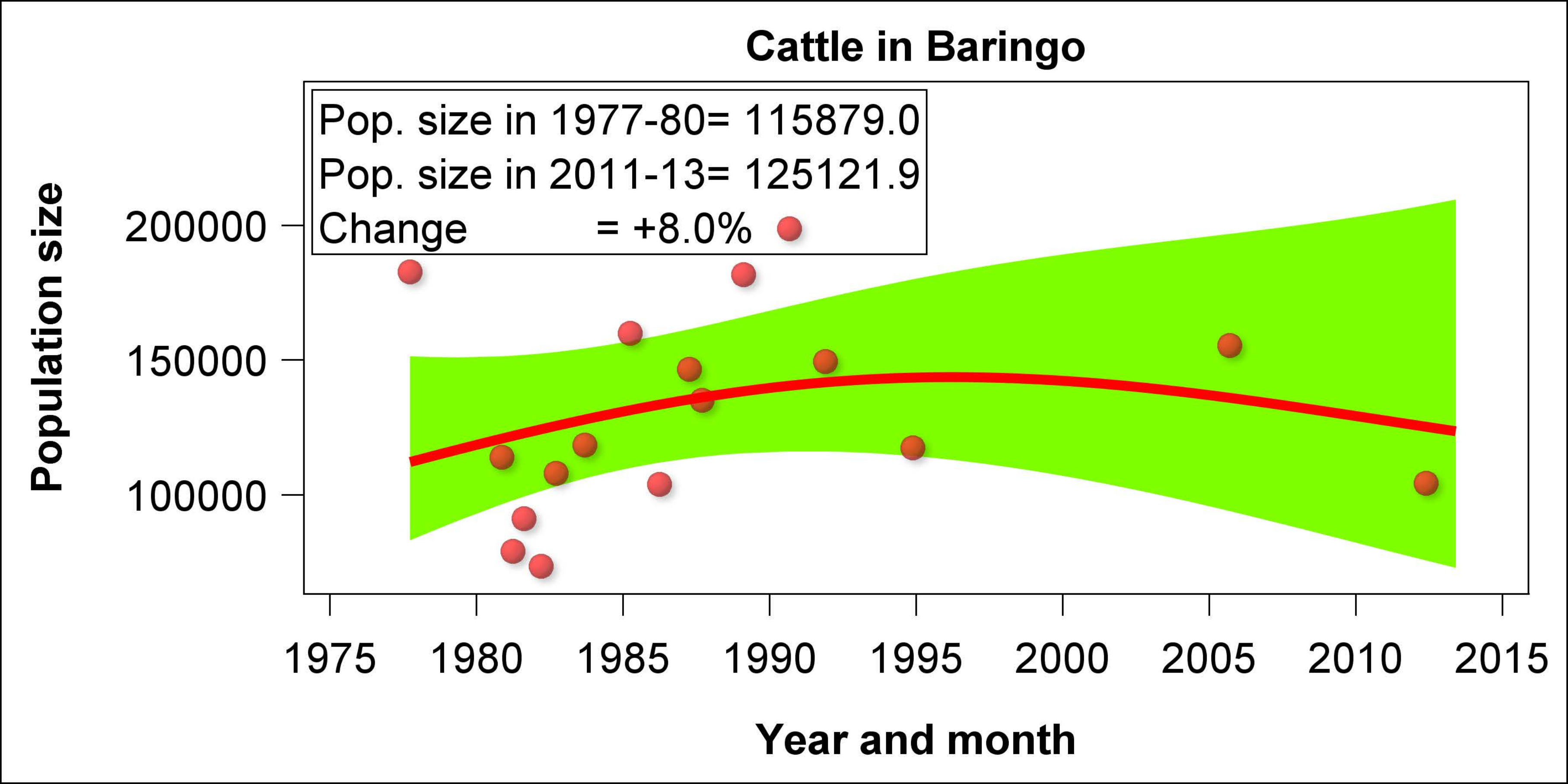
Cattle in Baringo
Pop. size in 1977-80= 115879.0
Pop. size in 2011-13= 125121.9
Change = +8.0%
Population size
200000
150000
100000
1975
1980
1985
1990
1995
2000
2005
2010
2015
Year and month

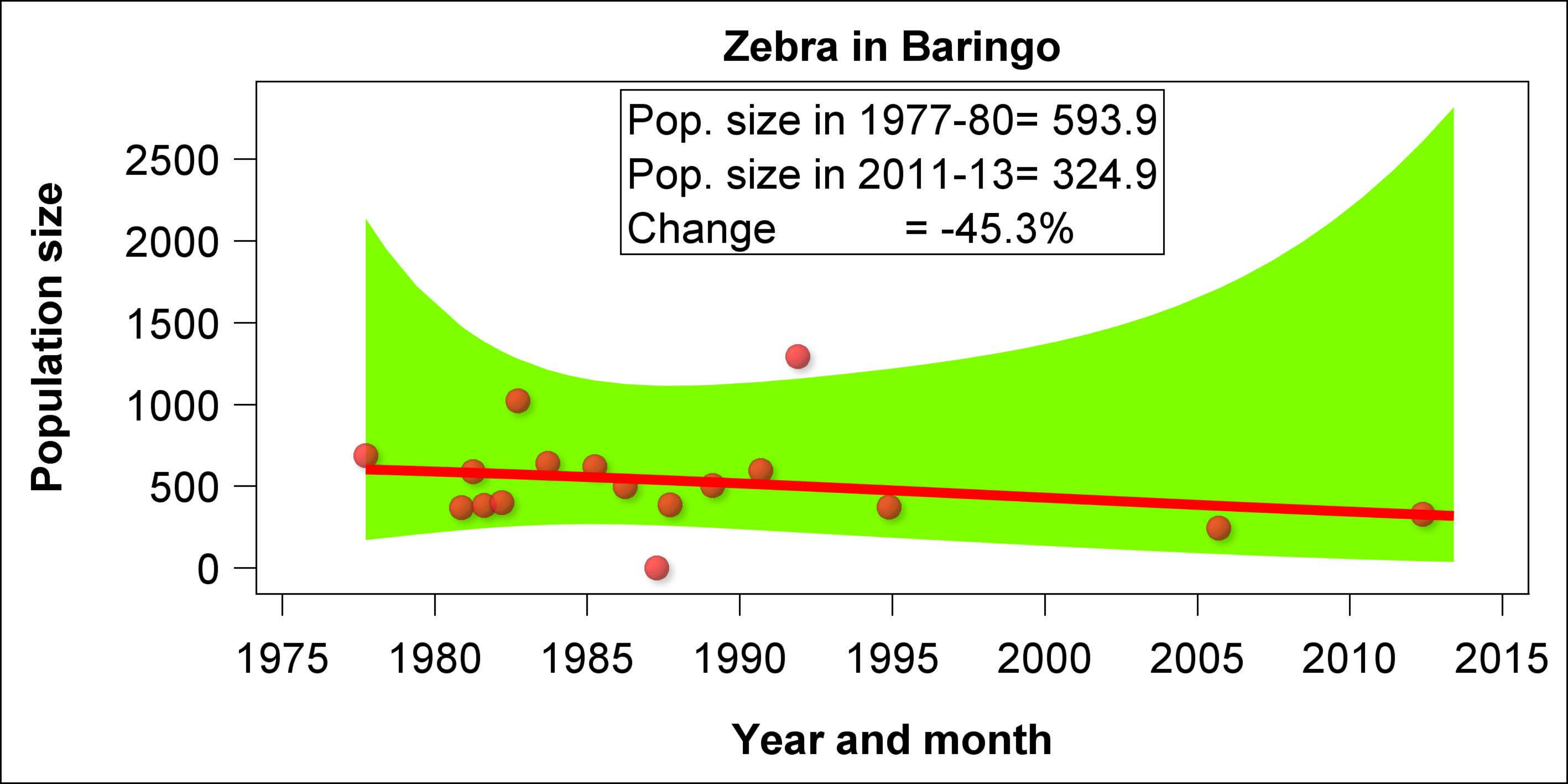
Zebra in Baringo
Pop. size in 1977-80= 593.9
Pop. size in 2011-13= 324.9
Change = -45.3%
Population size
2500
2000
1500
1000
500
0
1975
1980
1985
1990
1995
2000
2005
2010
2015
Year and month

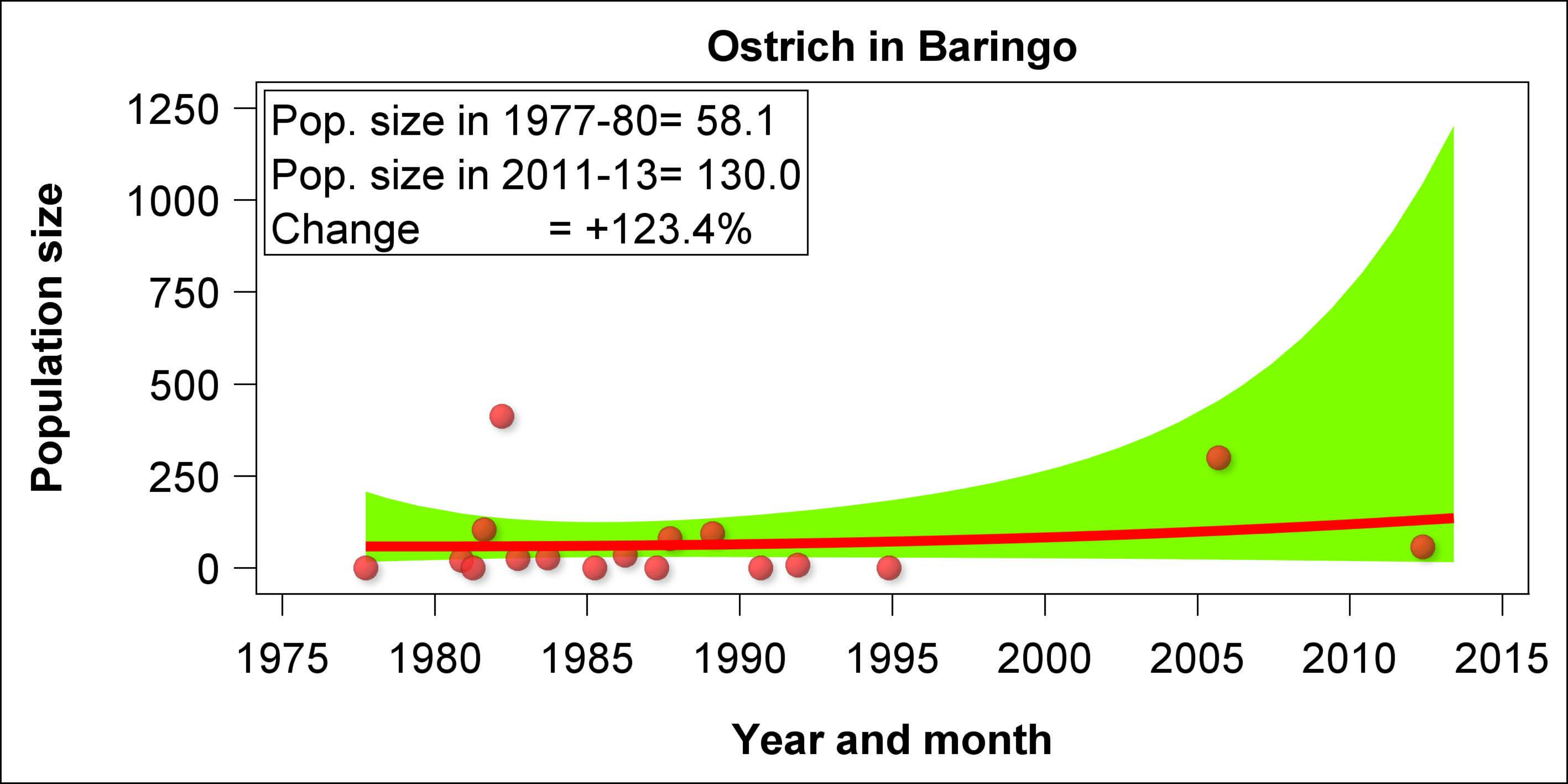
Ostrich in Baringo
Pop. size in 1977-80= 58.1
Pop. size in 2011-13= 130.0
Change = +123.4%
Population size
1250
1000
750
500
250
0
1975
1980
1985
1990
1995
2000
2005
2010
2015
Year and month

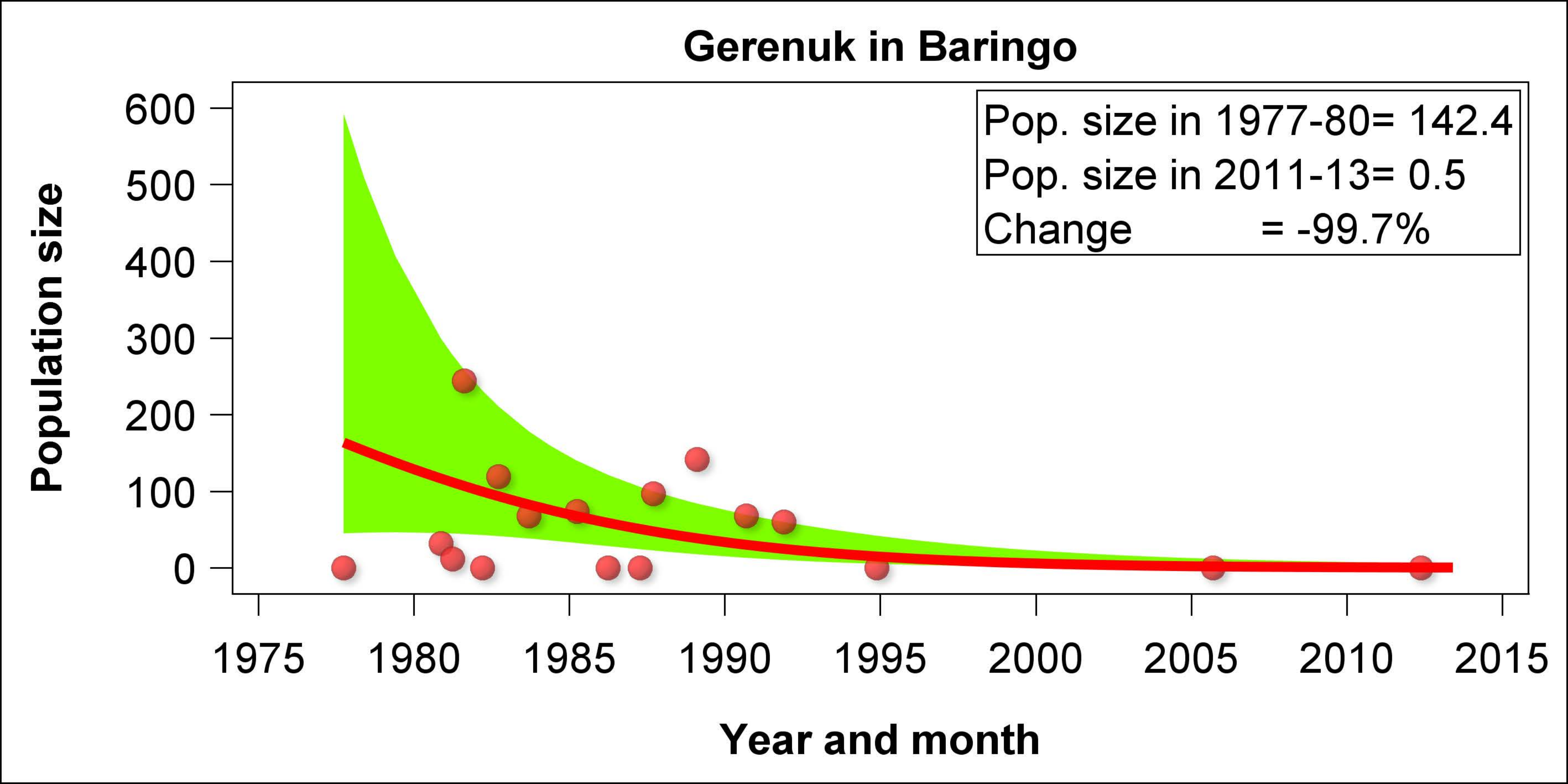

Gerenuk in Baringo
Pop. size in 1977-80= 142.4
Pop. size in 2011-13= 0.5
Change = -99.7%
Population size
600
500
400
300
200
100
0
1975
1980
1985
1990
1995
2000
2005
2010
2015
Year and month

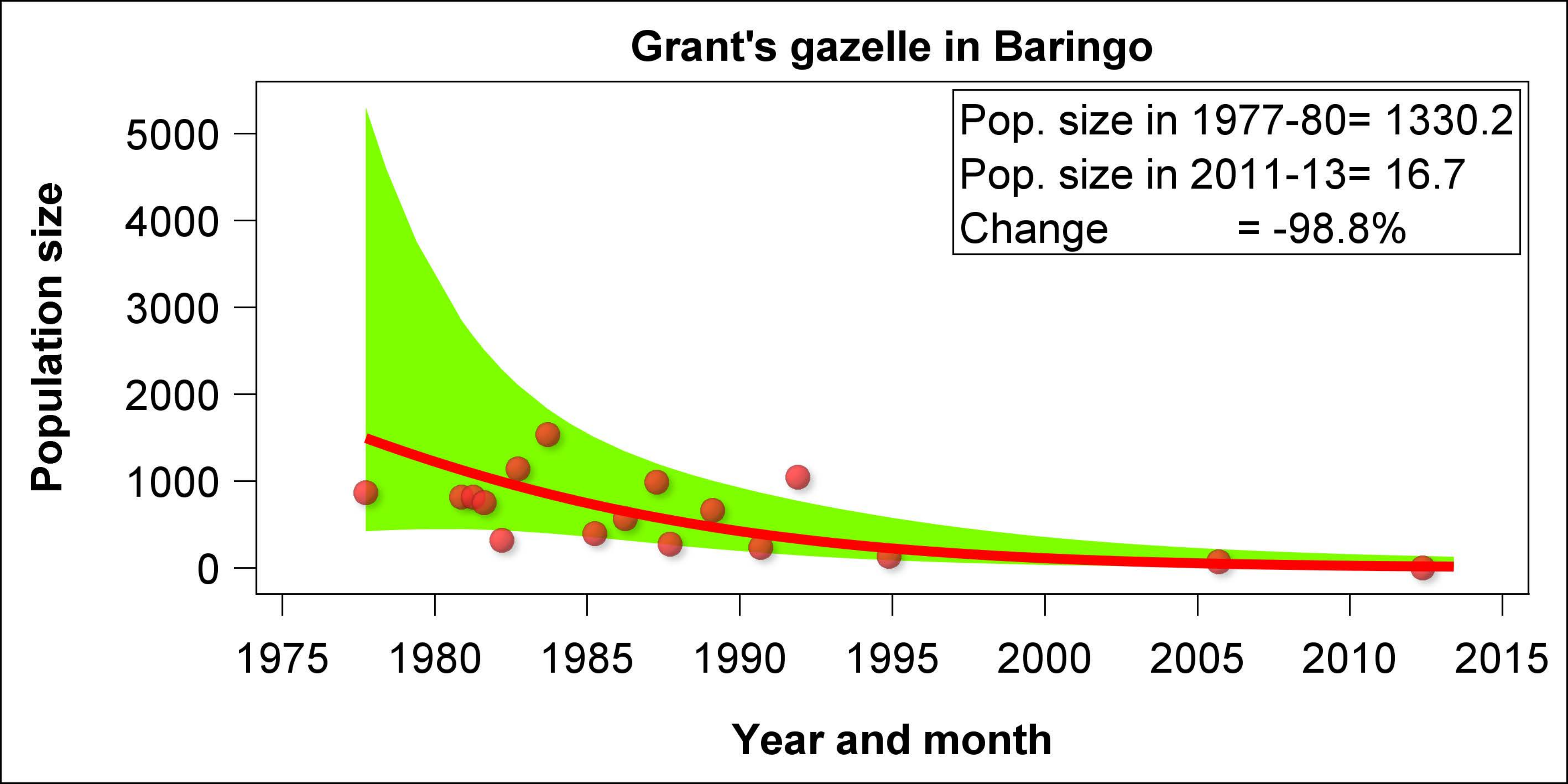
Grant's gazelle in Baringo
Pop. size in 1977-80= 1330.2
Pop. size in 2011-13= 16.7
Change = -98.8%
Population size
5000
4000
3000
2000
1000
0
1975
1980
1985
1990
1995
2000
2005
2010
2015
Year and month

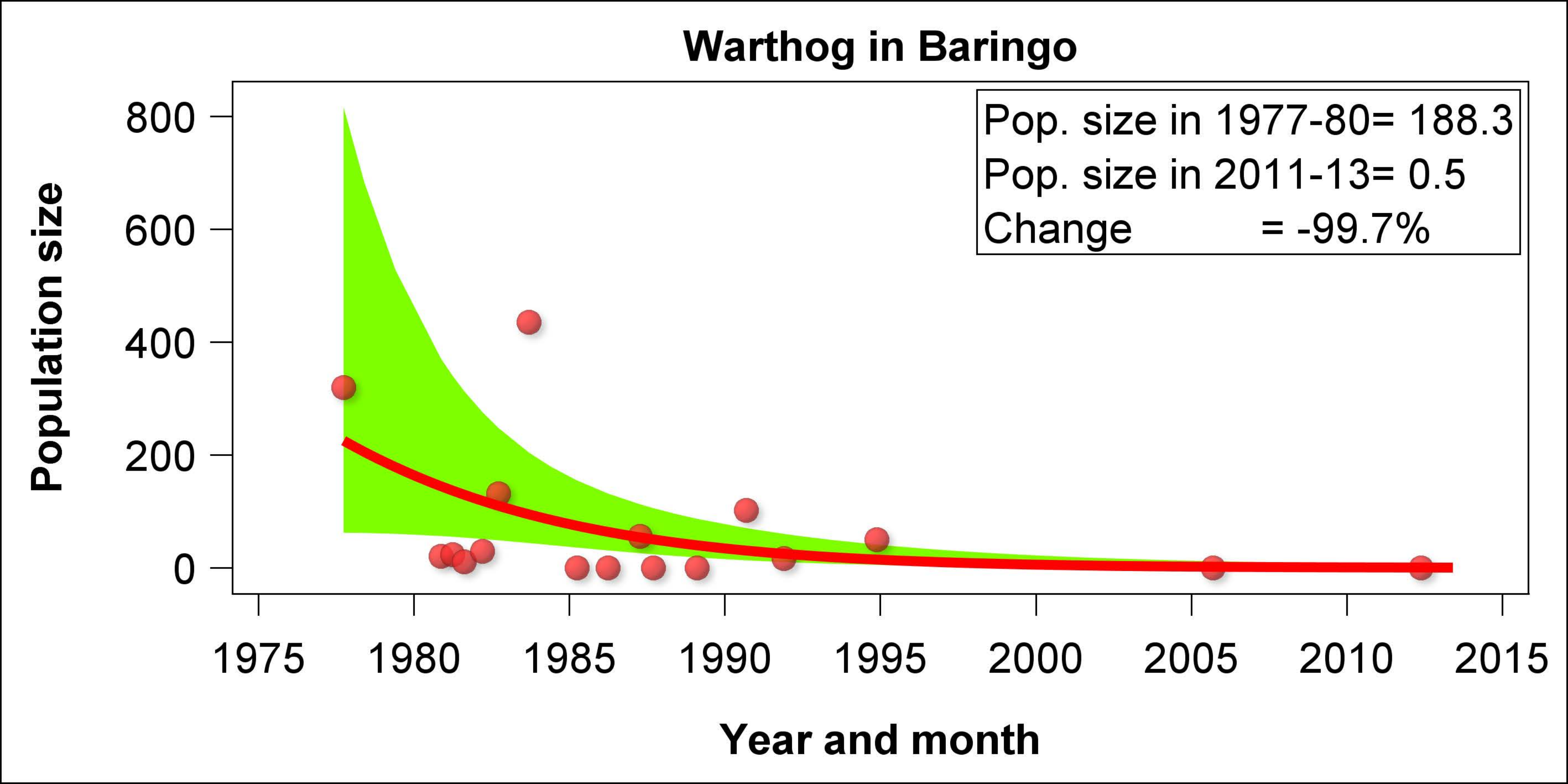
Warthog in Baringo
Pop. size in 1977-80= 188.3
Pop. size in 2011-13= 0.5
Change = -99.7%
Population size
800
600
400
200
0
1975
1980
1985
1990
1995
2000
2005
2010
2015
Year and month

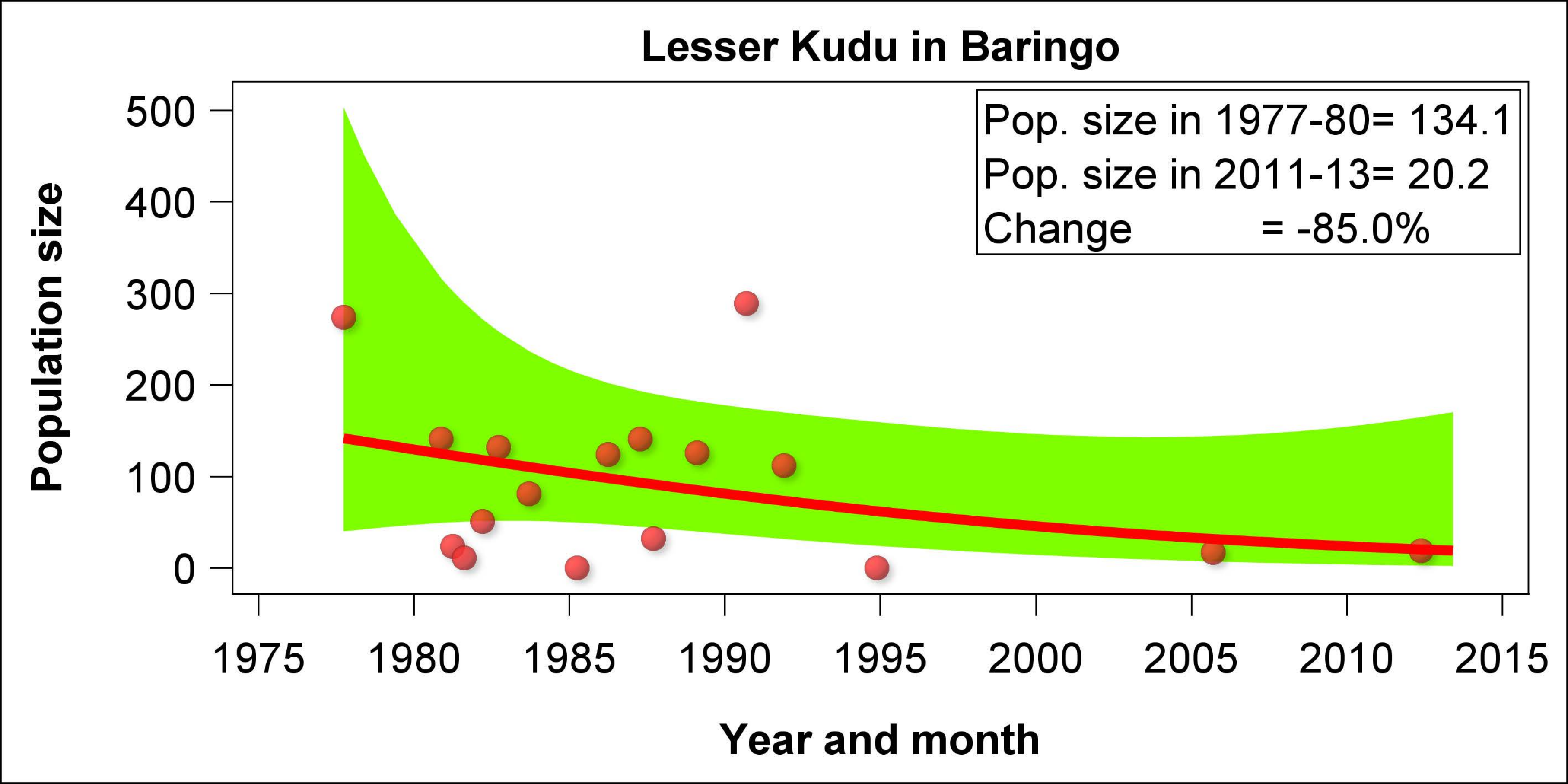
Lesser Kudu in Baringo
Pop. size in 1977-80= 134.1
Pop. size in 2011-13= 20.2
Change = -85.0%
Population size
500
400
300
200
100
0
1975
1980
1985
1990
1995
2000
2005
2010
2015
Year and month

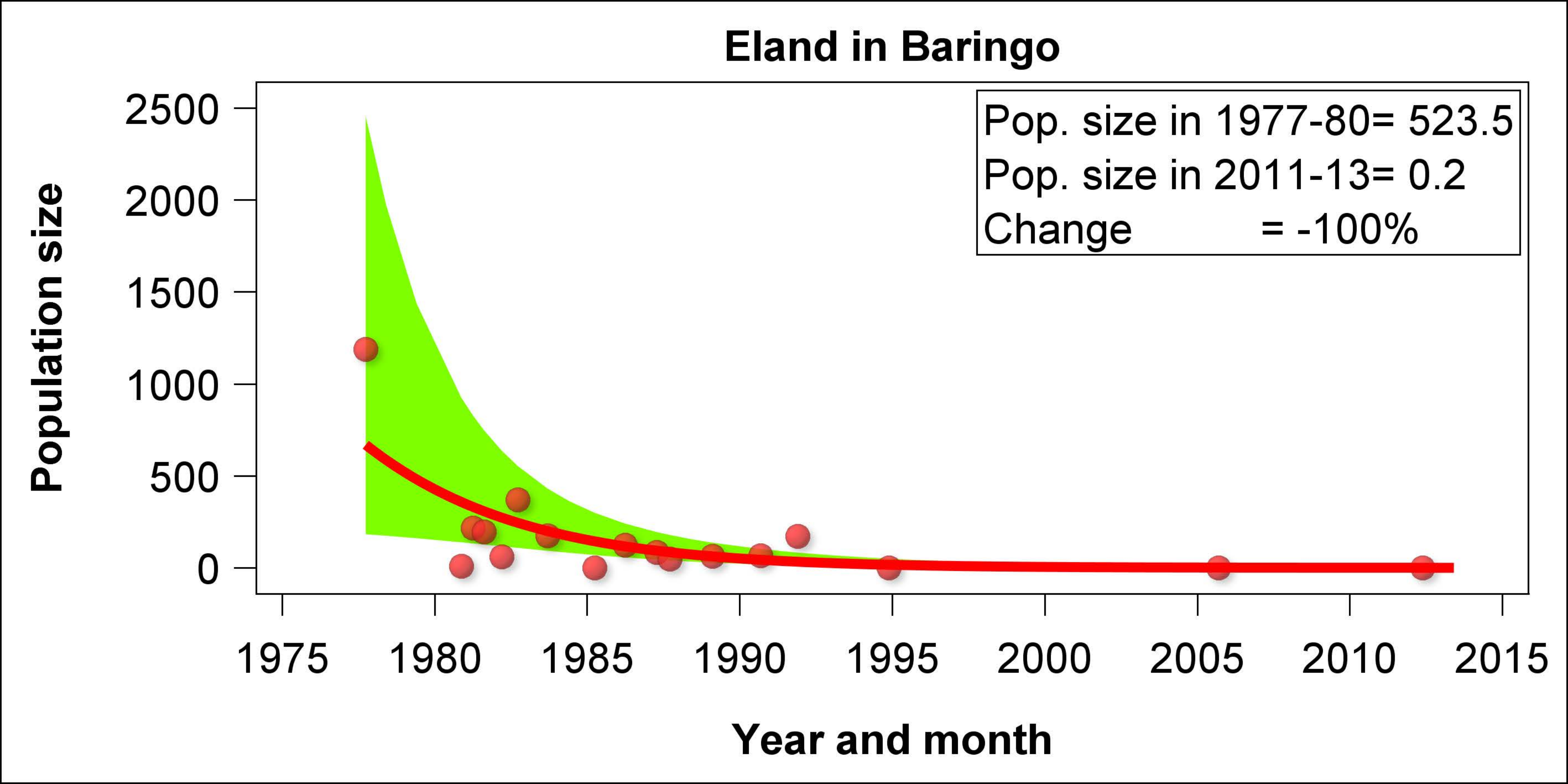

Eland in Baringo
Pop. size in 1977-80= 523.5
Pop. size in 2011-13= 0.2
Change = -100%
Population size
2500
2000
1500
1000
500
0
1975
1980
1985
1990
1995
2000
2005
2010
2015
Year and month

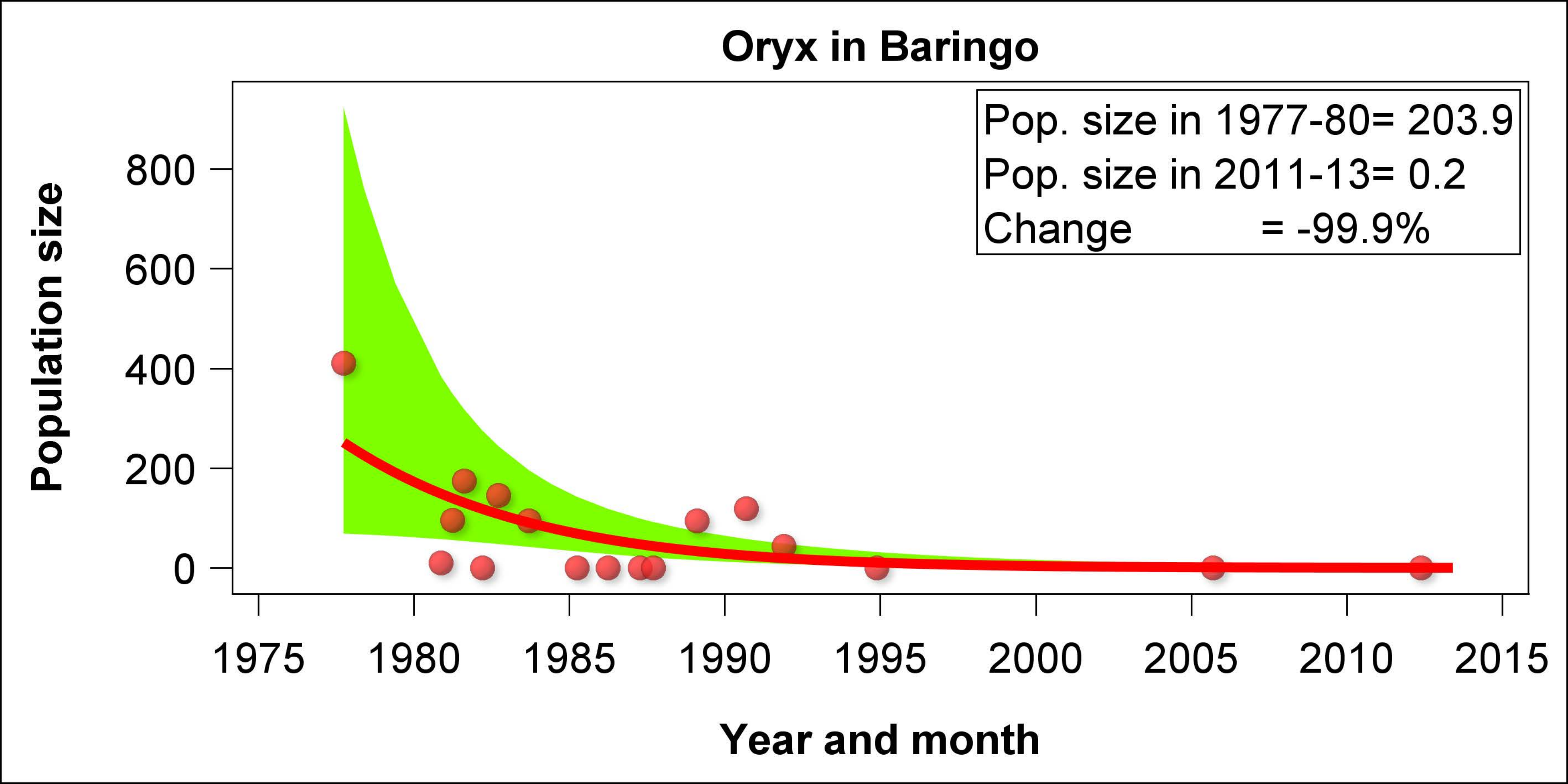
Oryx in Baringo
Pop. size in 1977-80= 203.9
Pop. size in 2011-13= 0.2
Change = -99.9%
800
600
400
200
0
Population size
1975
1980
1985
1990
1995
2000
2005
2010
2015
Year and month

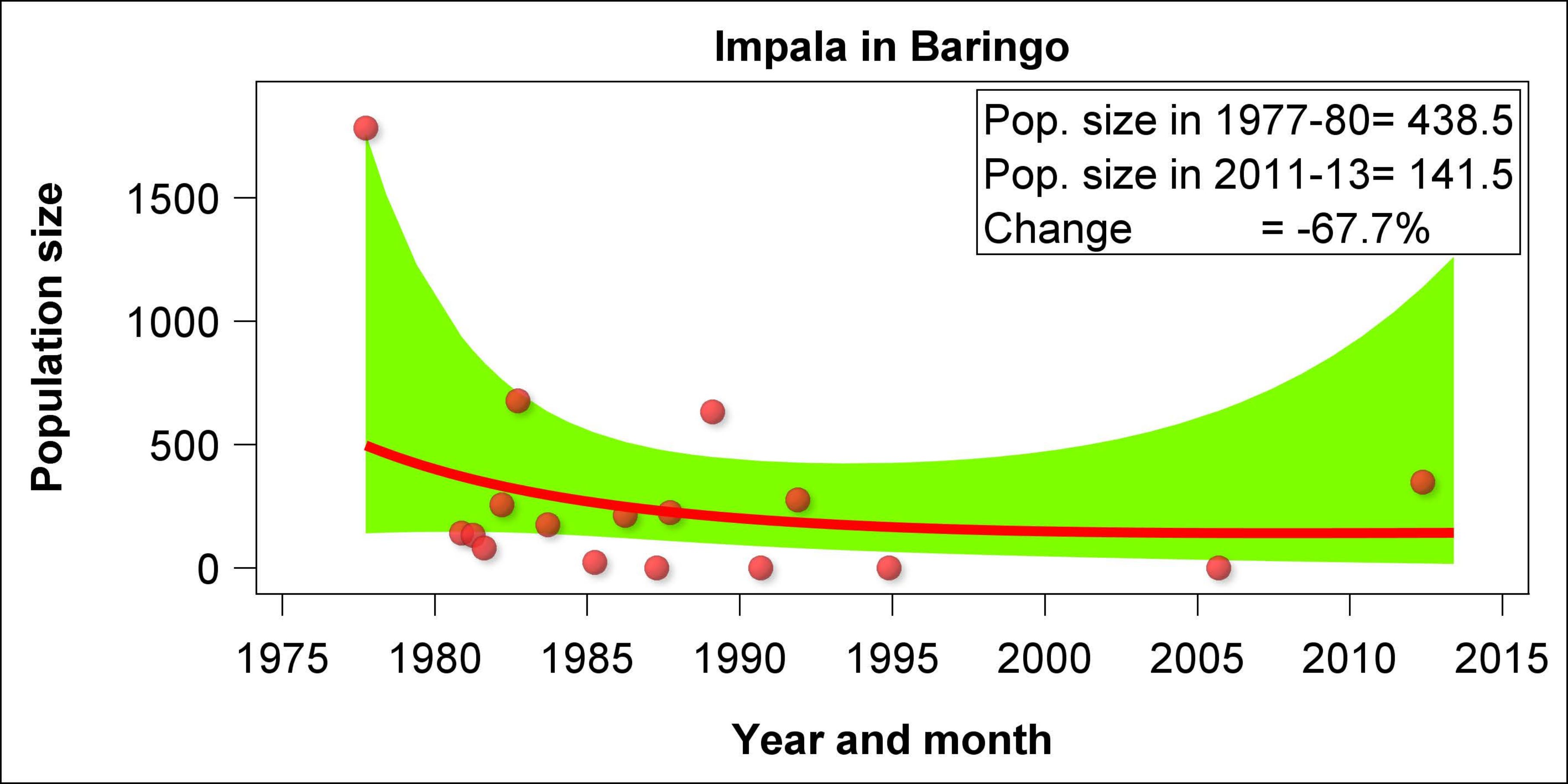

Impala in Baringo
Pop. size in 1977-80= 438.5
Pop. size in 2011-13= 141.5
Change = -67.7%
Population size
0
500
1000
1500
1975
1980
1985
1990
1995
2000
2005
2010
2015
Year and month

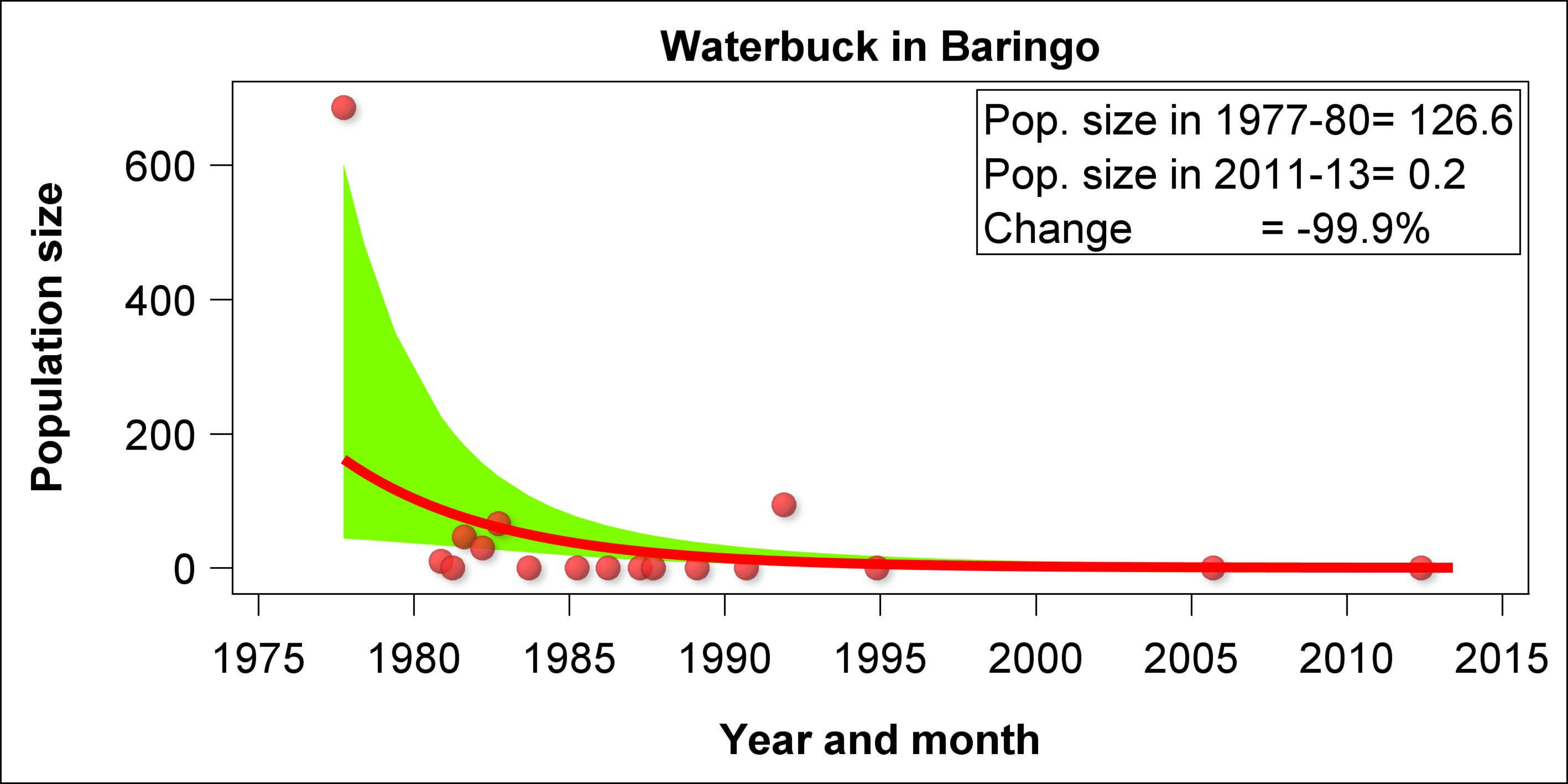
Waterbuck in Baringo
Pop. size in 1977-80= 126.6
Pop. size in 2011-13= 0.2
Change = -99.9%
Population size
600
400
200
0
1975
1980
1985
1990
1995
2000
2005
2010
2015
Year and month
