# Supplementary material for: Extreme Wildlife Declines and Concurrent Increase in Livestock Numbers in Kenya: What Are the Causes?
Source: PLoS One. 2016 Sep 27;11(9):e0163249. doi: 10.1371/journal.pone.0163249 (PMC5039022; doi:10.1371/journal.pone.0163249)
Supplement: S12 Fig — The solid red line is the fitted trend curve and the shaded chartreuse band is the pointwise 95% confidence band. The estimated average population size in 1977–1980 and 2011–2016 and the percentage change in population size between the two periods are provided in the inset. (PDF) [file pone.0163249.s022.pdf]

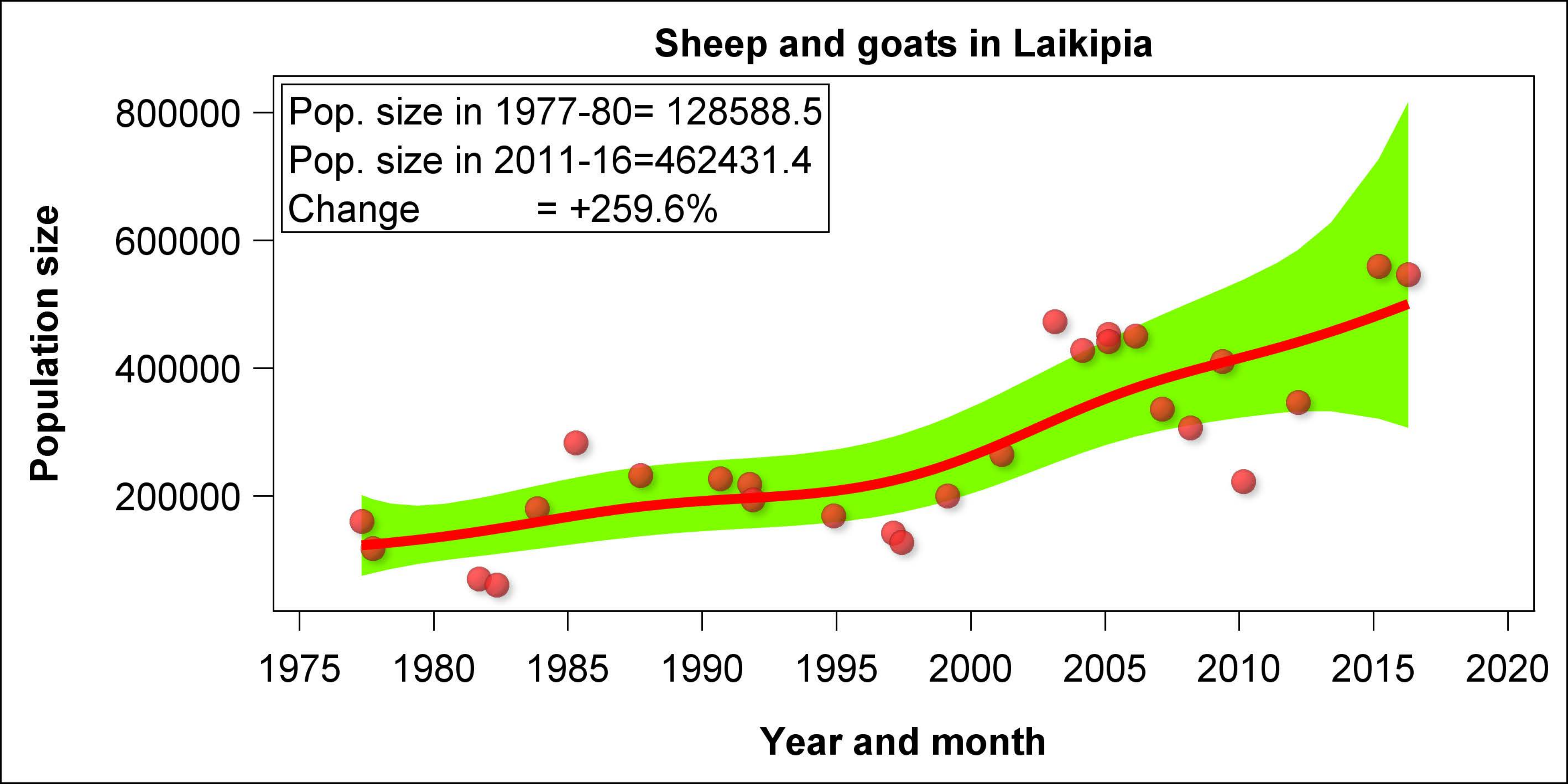

Sheep and goats in Laikipia
Pop. size in 1977-80= 128588.5
Pop. size in 2011-16=462431.4
Change = +259.6%
Population size
800000
600000
400000
200000
1975
1980
1985
1990
1995
2000
2005
2010
2015
2020
Year and month

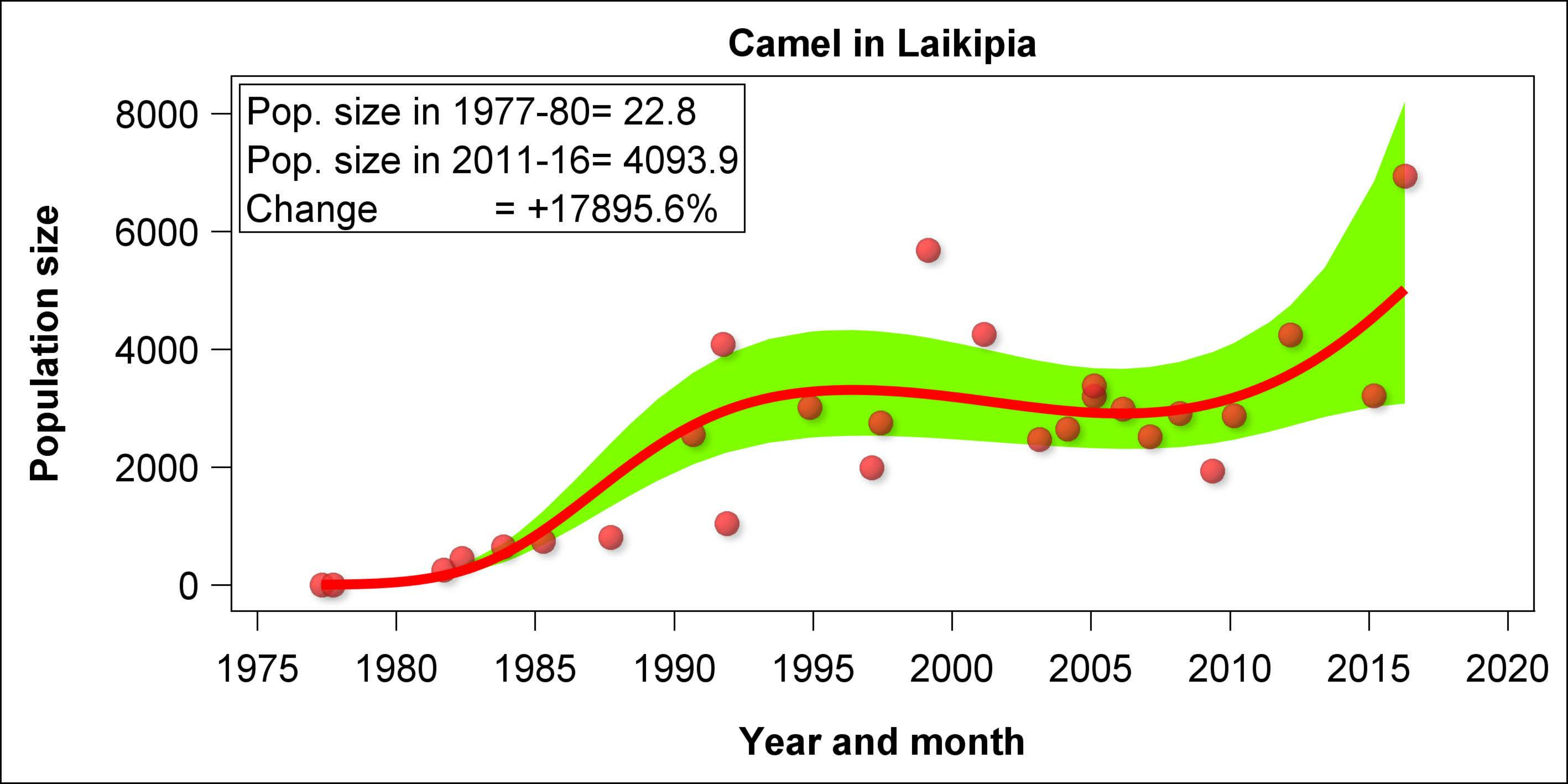

Camel in Laikipia
Pop. size in 1977-80= 22.8
Pop. size in 2011-16= 4093.9
Change = +17895.6%
Population size
8000
6000
4000
2000
0
1975
1980
1985
1990
1995
2000
2005
2010
2015
2020
Year and month

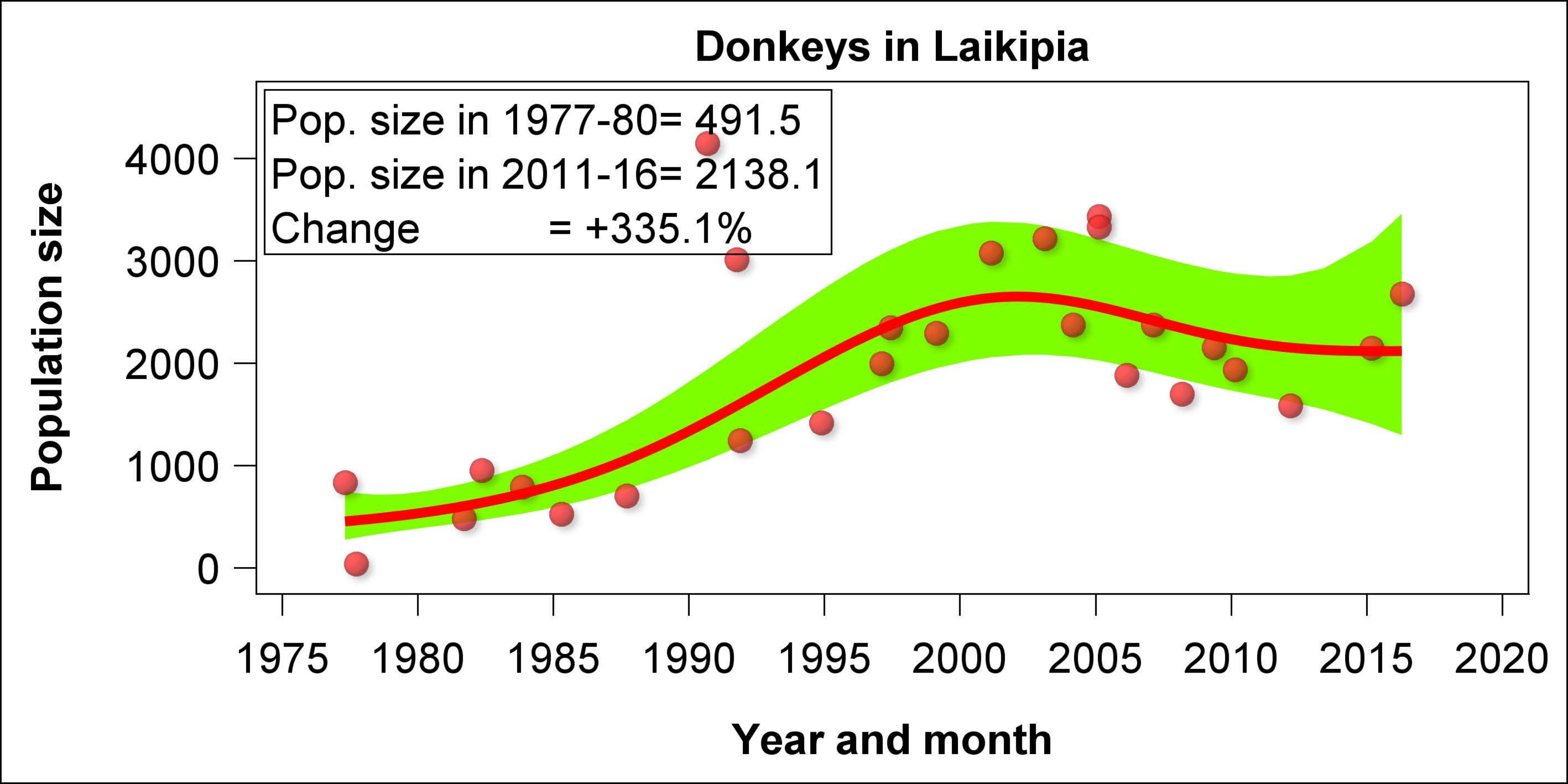
Donkeys in Laikipia
Pop. size in 1977-80= 491.5
Pop. size in 2011-16= 2138.1
Change = +335.1%
Population size
0
1000
2000
3000
4000
1975
1980
1985
1990
1995
2000
2005
2010
2015
2020
Year and month

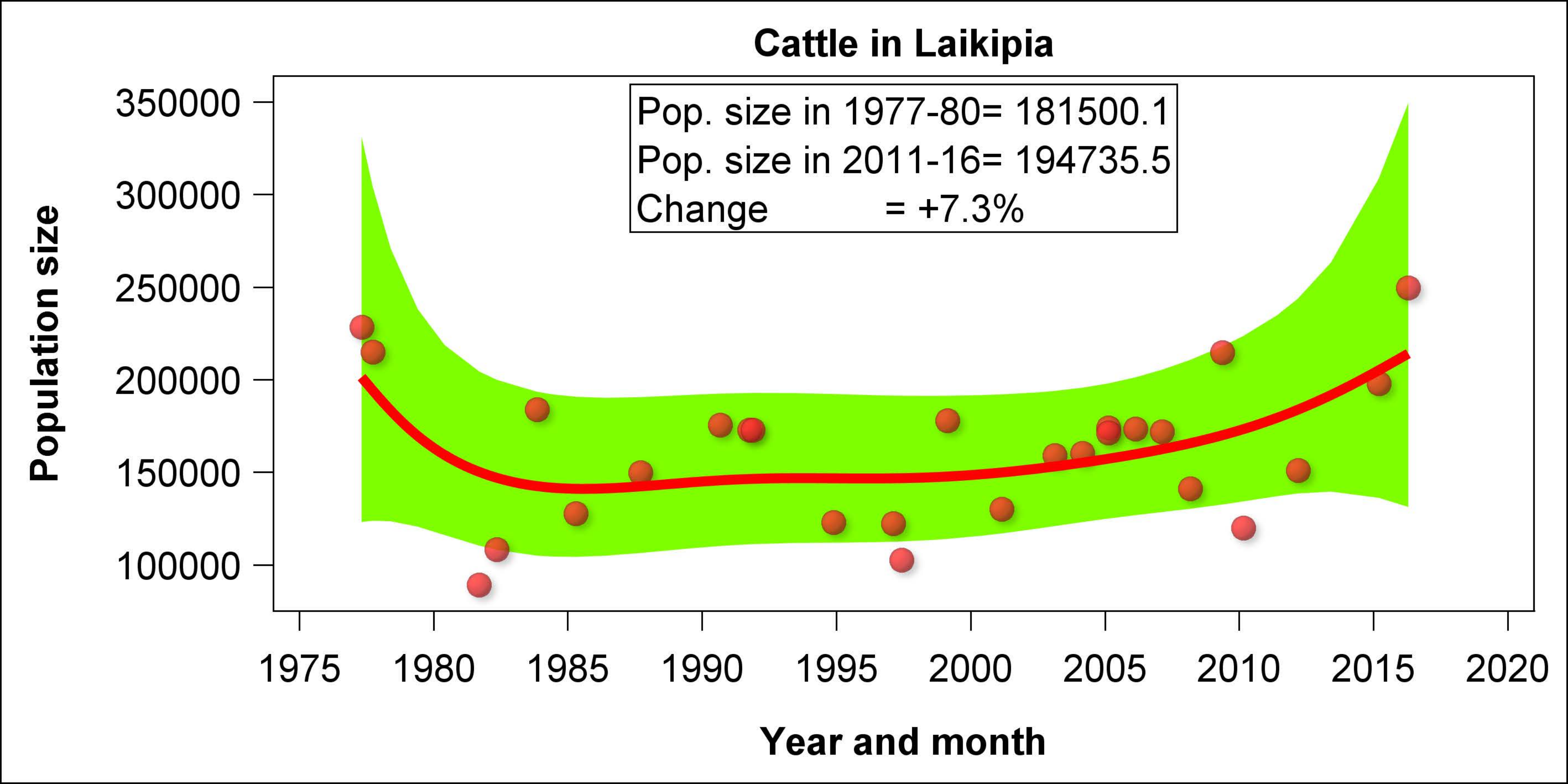

Cattle in Laikipia
Pop. size in 1977-80= 181500.1
Pop. size in 2011-16= 194735.5
Change = +7.3%
Population size
350000
300000
250000
200000
150000
100000
1975
1980
1985
1990
1995
2000
2005
2010
2015
2020
Year and month

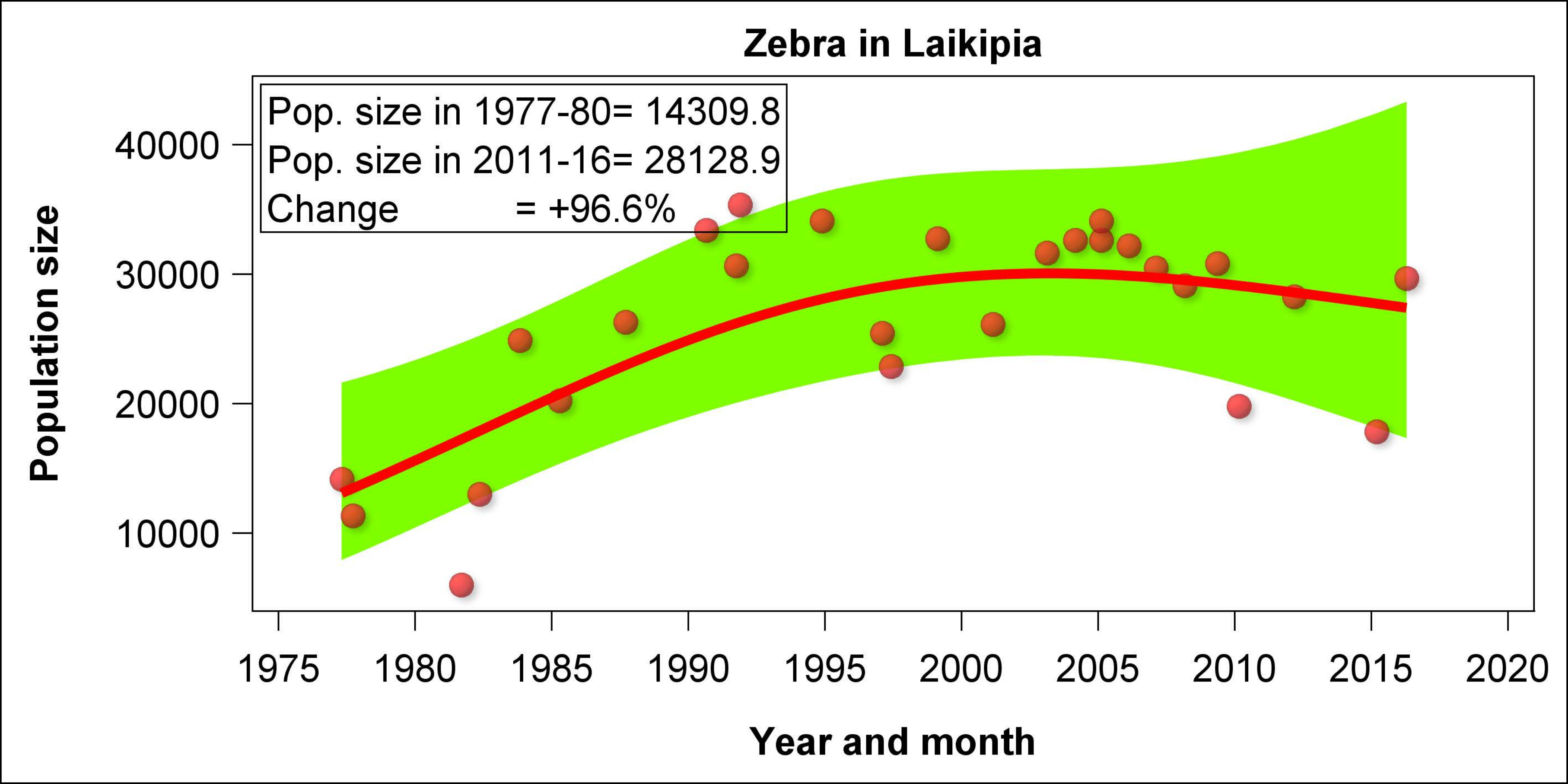

Zebra in Laikipia
Pop. size in 1977-80= 14309.8
Pop. size in 2011-16= 28128.9
Change = +96.6%
Population size
40000
30000
20000
10000
1975
1980
1985
1990
1995
2000
2005
2010
2015
2020
Year and month

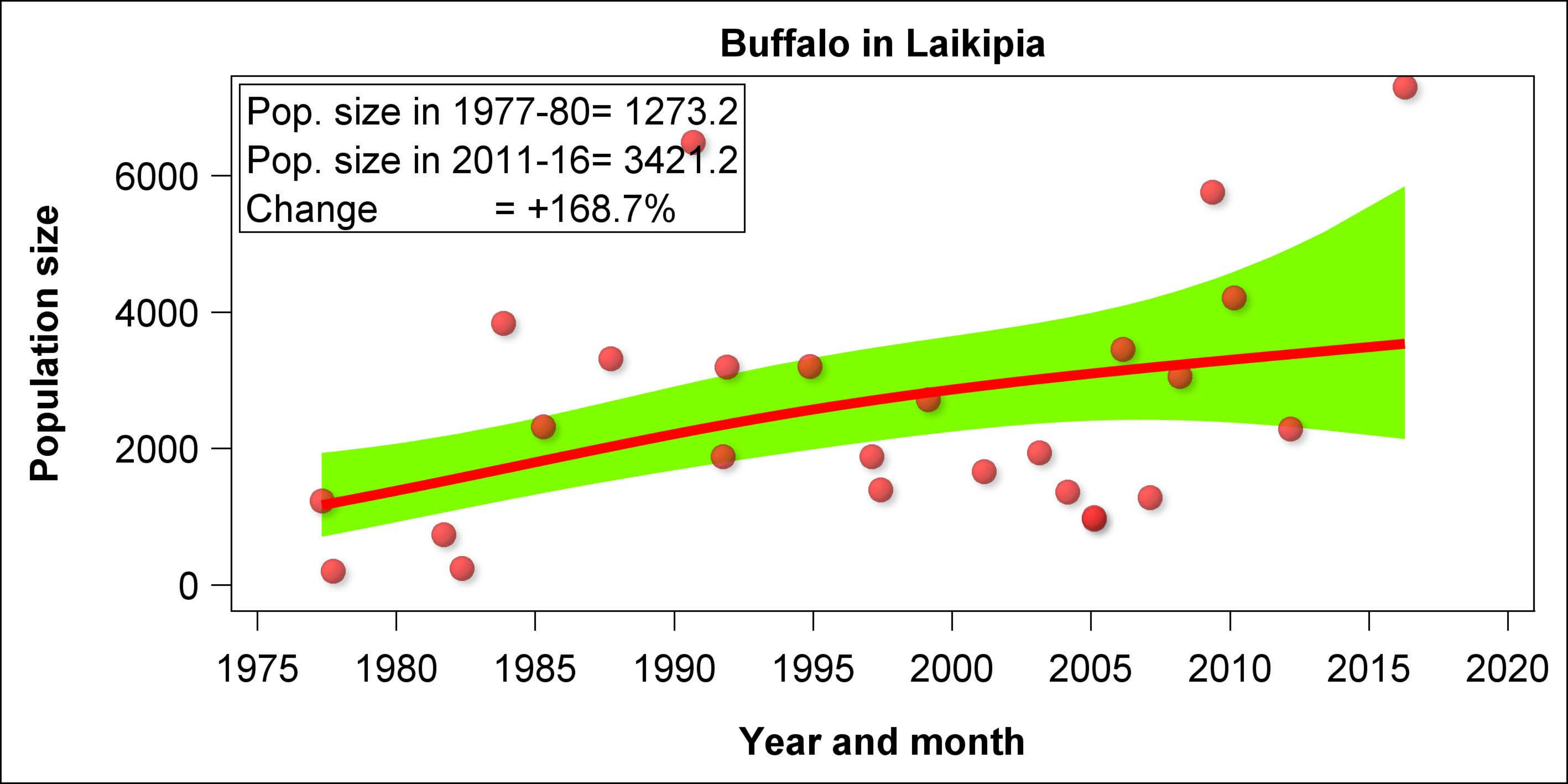
Buffalo in Laikipia
Pop. size in 1977-80= 1273.2
Pop. size in 2011-16= 3421.2
Change = +168.7%
Population size
0
2000
4000
6000
1975
1980
1985
1990
1995
2000
2005
2010
2015
2020
Year and month

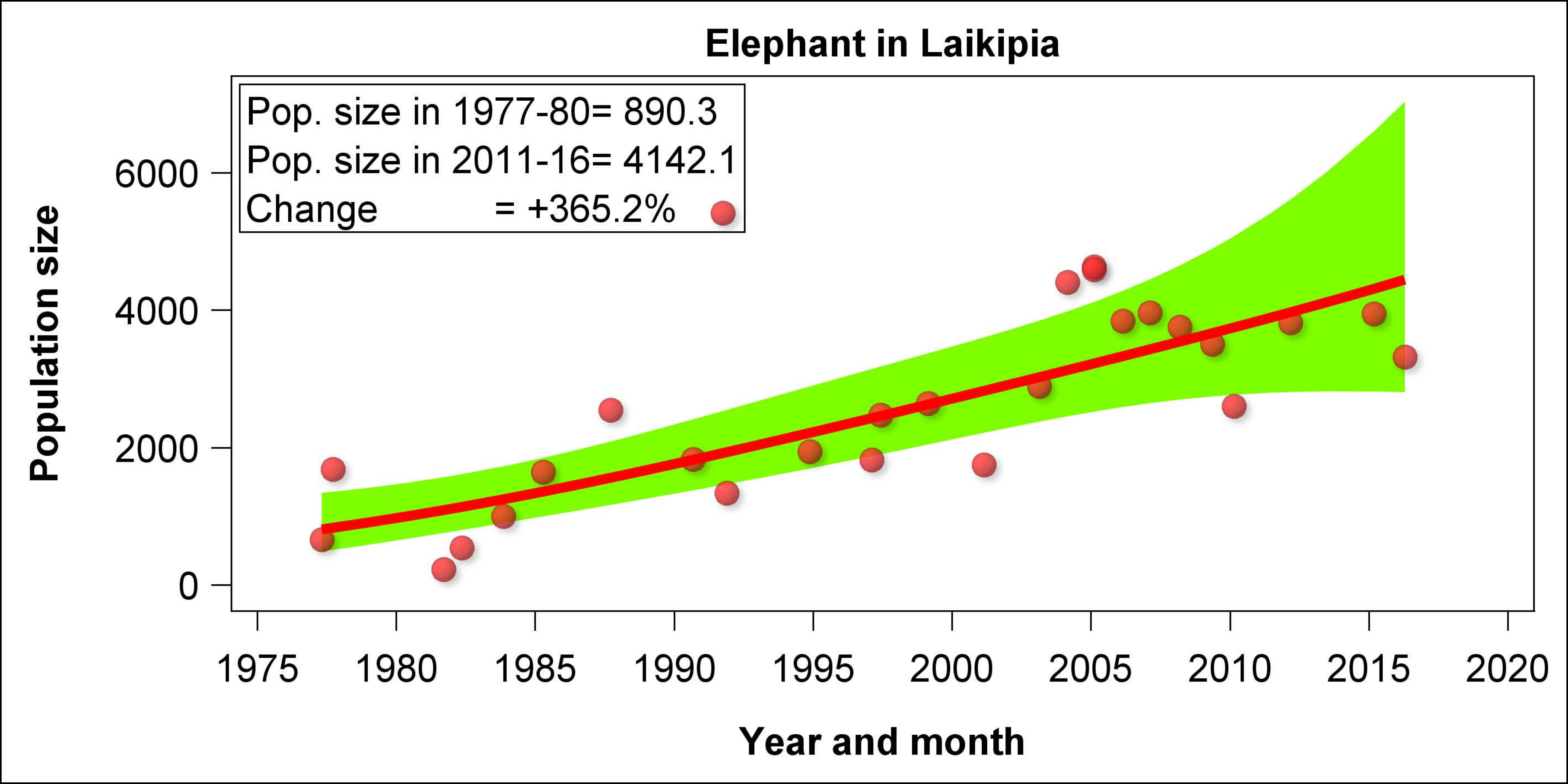
Elephant in Laikipia
Pop. size in 1977-80= 890.3
Pop. size in 2011-16= 4142.1
Change = +365.2%
Population size
6000
4000
2000
0
1975
1980
1985
1990
1995
2000
2005
2010
2015
2020
Year and month

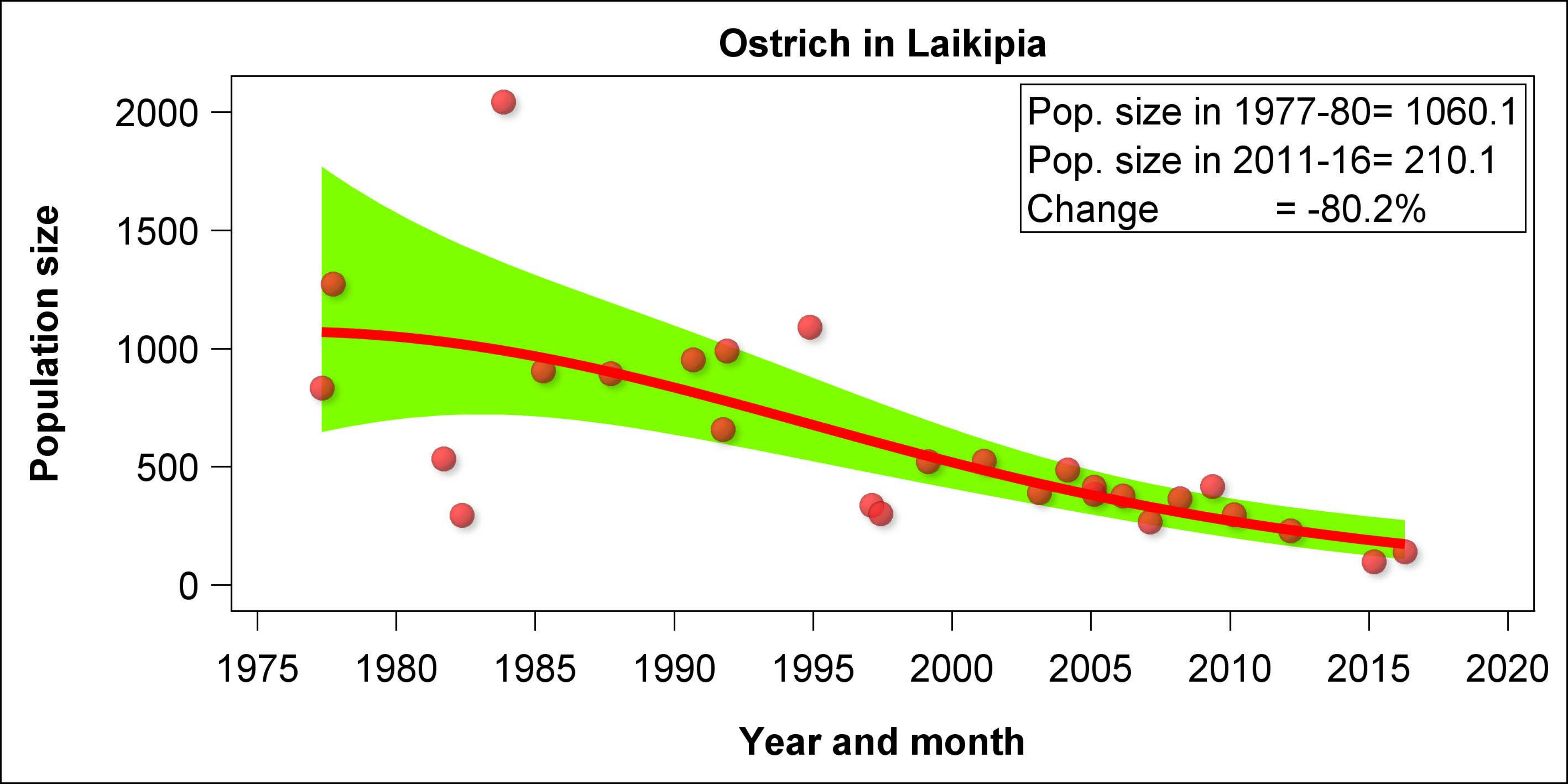

Ostrich in Laikipia
Pop. size in 1977-80= 1060.1
Pop. size in 2011-16= 210.1
Change = -80.2%
Population size
0
500
1000
1500
2000
1975
1980
1985
1990
1995
2000
2005
2010
2015
2020
Year and month

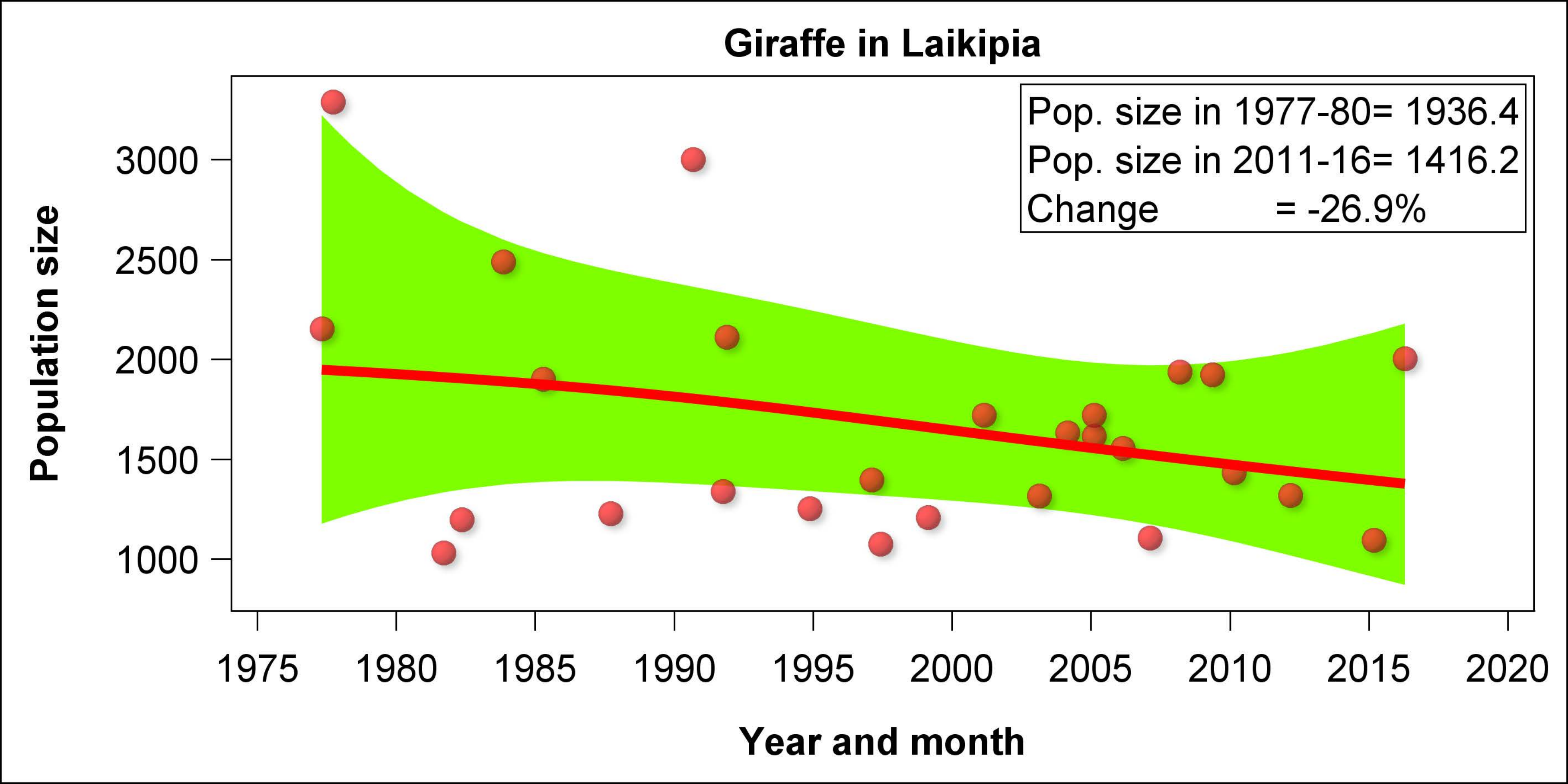

Giraffe in Laikipia
Pop. size in 1977-80= 1936.4
Pop. size in 2011-16= 1416.2
Change = -26.9%
Population size
3000
2500
2000
1500
1000
1975
1980
1985
1990
1995
2000
2005
2010
2015
2020
Year and month

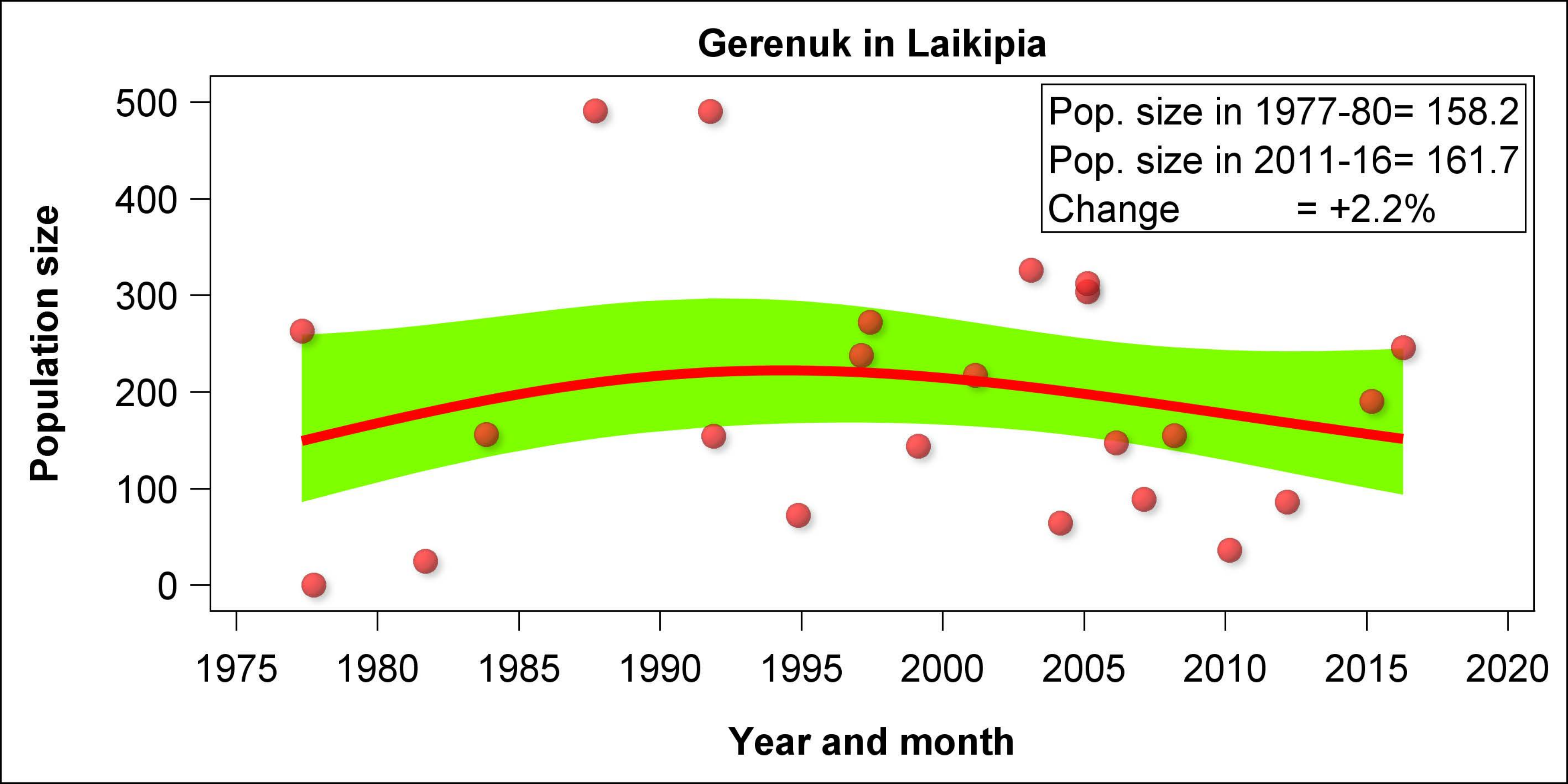

Gerenuk in Laikipia
Pop. size in 1977-80= 158.2
Pop. size in 2011-16= 161.7
Change = +2.2%
Population size
500
400
300
200
100
0
1975
1980
1985
1990
1995
2000
2005
2010
2015
2020
Year and month

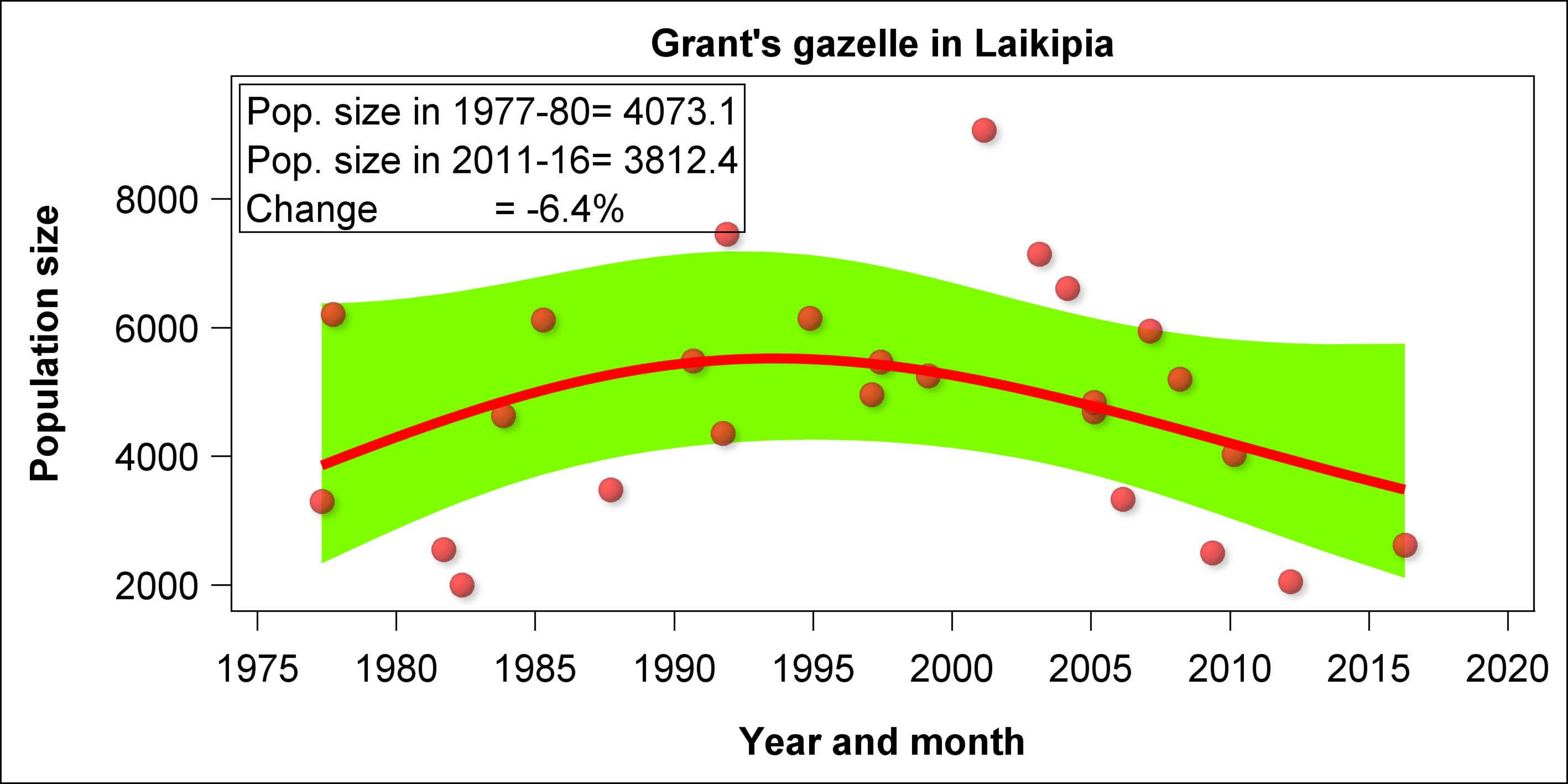
Grant's gazelle in Laikipia
Pop. size in 1977-80= 4073.1
Pop. size in 2011-16= 3812.4
Change = -6.4%
Population size
8000
6000
4000
2000
1975
1980
1985
1990
1995
2000
2005
2010
2015
2020
Year and month

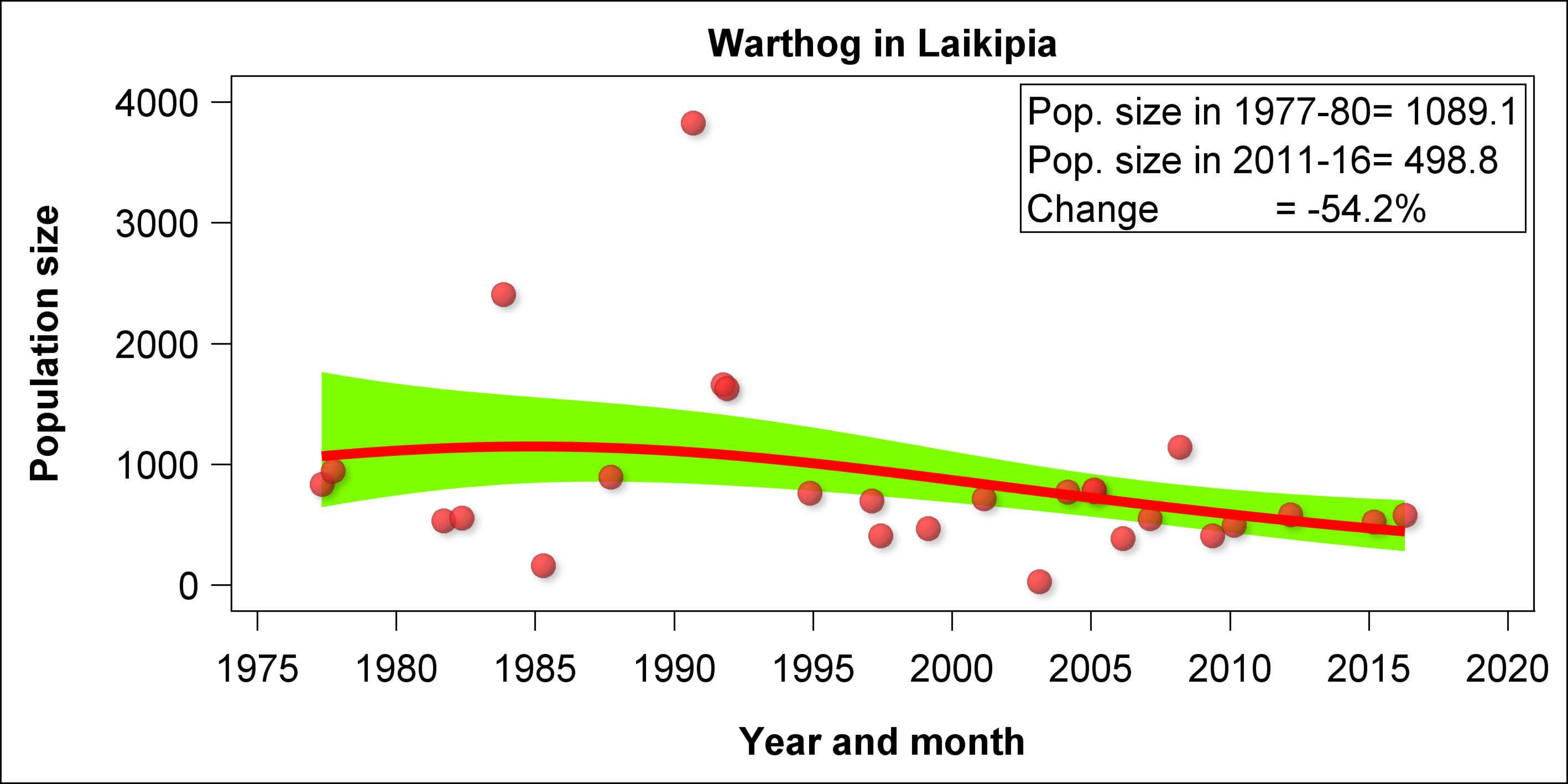

Warthog in Laikipia
Pop. size in 1977-80= 1089.1
Pop. size in 2011-16= 498.8
Change = -54.2%
Population size
4000
3000
2000
1000
0
1975
1980
1985
1990
1995
2000
2005
2010
2015
2020
Year and month

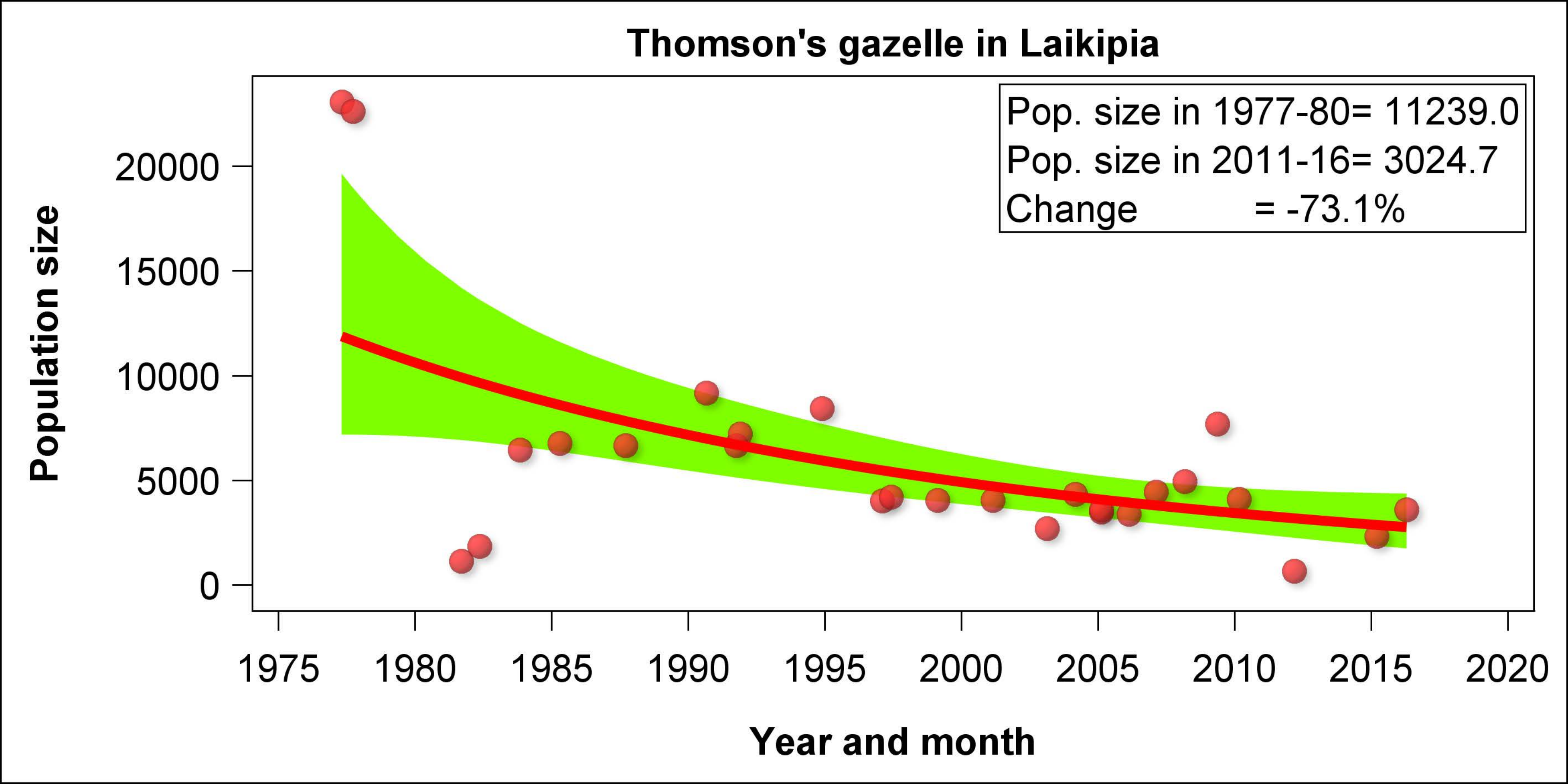

Thomson's gazelle in Laikipia
Pop. size in 1977-80= 11239.0
Pop. size in 2011-16= 3024.7
Change = -73.1%
Population size
20000
15000
10000
5000
0
1975
1980
1985
1990
1995
2000
2005
2010
2015
2020
Year and month

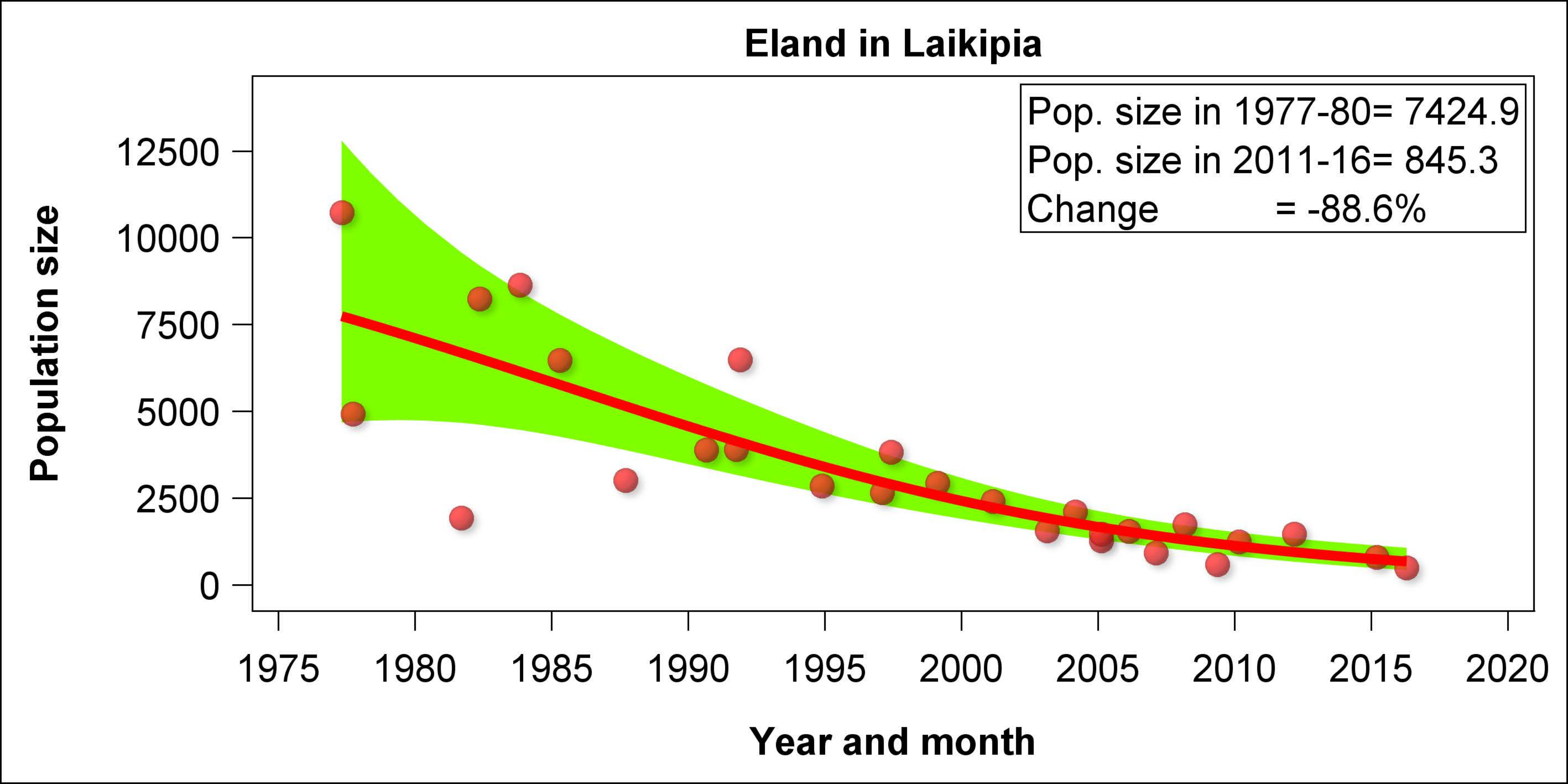
Eland in Laikipia
Pop. size in 1977-80= 7424.9
Pop. size in 2011-16= 845.3
Change = -88.6%
Population size
12500
10000
7500
5000
2500
0
1975
1980
1985
1990
1995
2000
2005
2010
2015
2020
Year and month

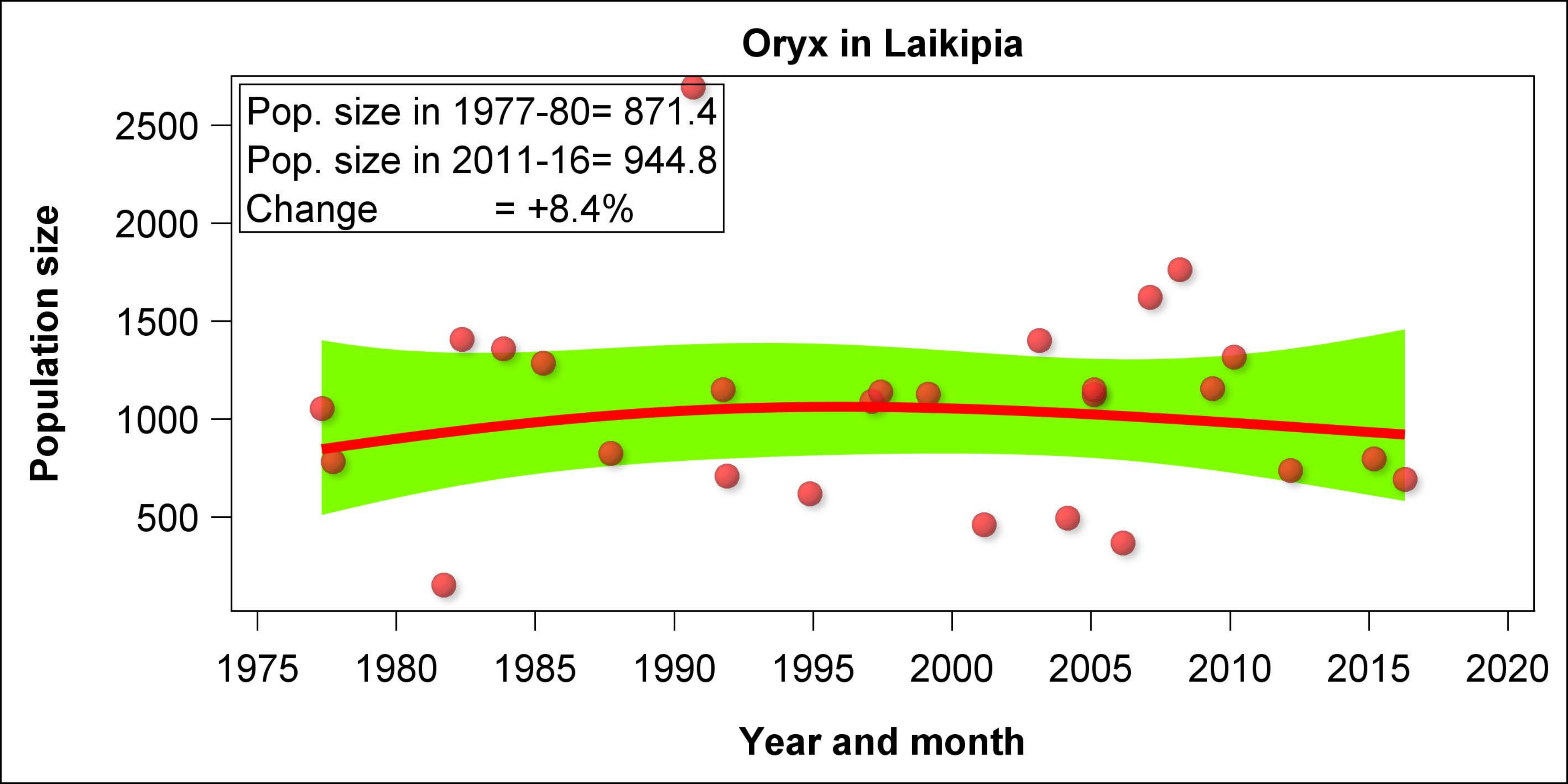

Oryx in Laikipia
Pop. size in 1977-80= 871.4
Pop. size in 2011-16= 944.8
Change = +8.4%
Population size
2500
2000
1500
1000
500
1975
1980
1985
1990
1995
2000
2005
2010
2015
2020
Year and month

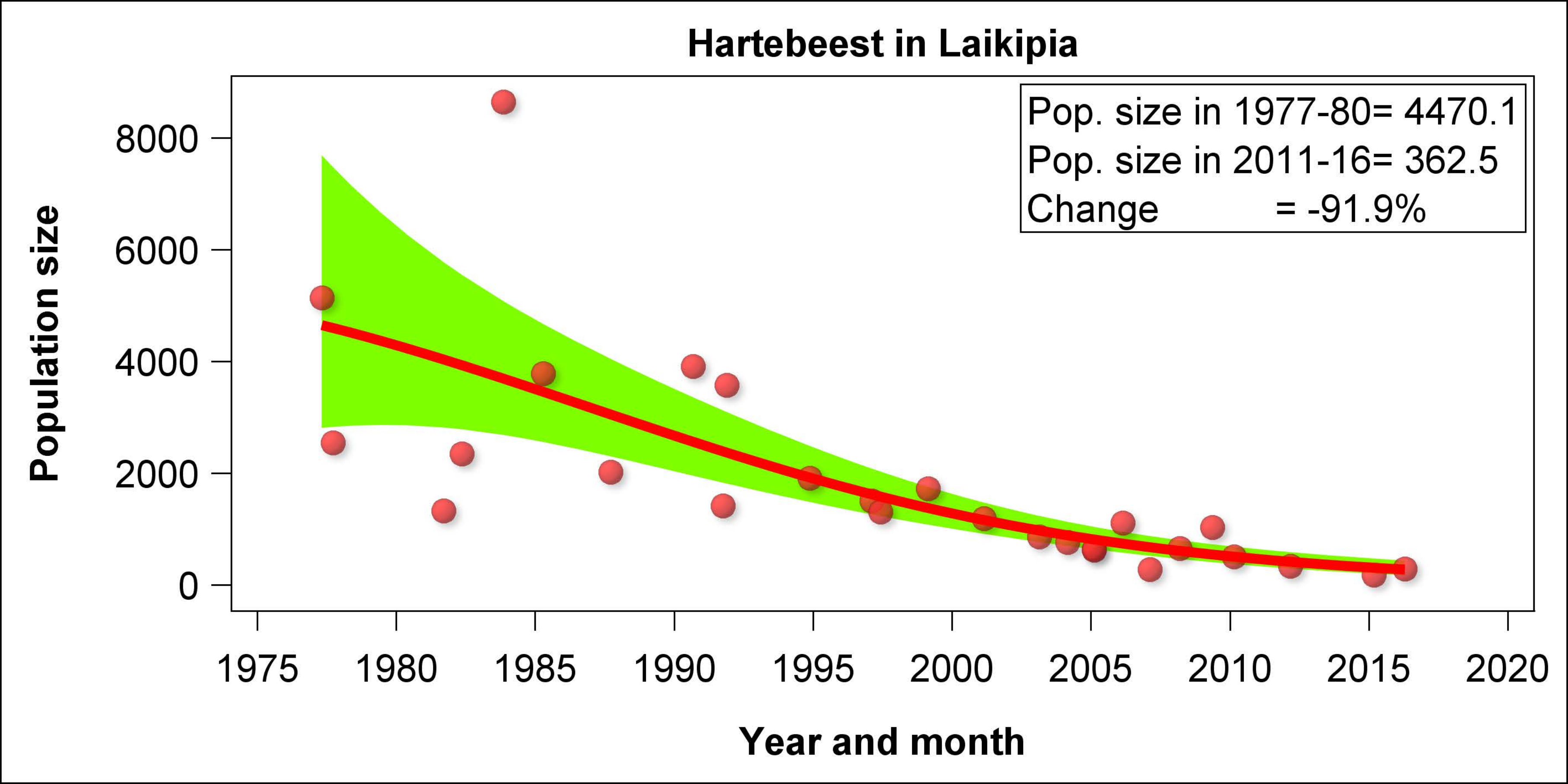
Hartebeest in Laikipia
Pop. size in 1977-80= 4470.1
Pop. size in 2011-16= 362.5
Change = -91.9%
Population size
8000
6000
4000
2000
0
1975
1980
1985
1990
1995
2000
2005
2010
2015
2020
Year and month

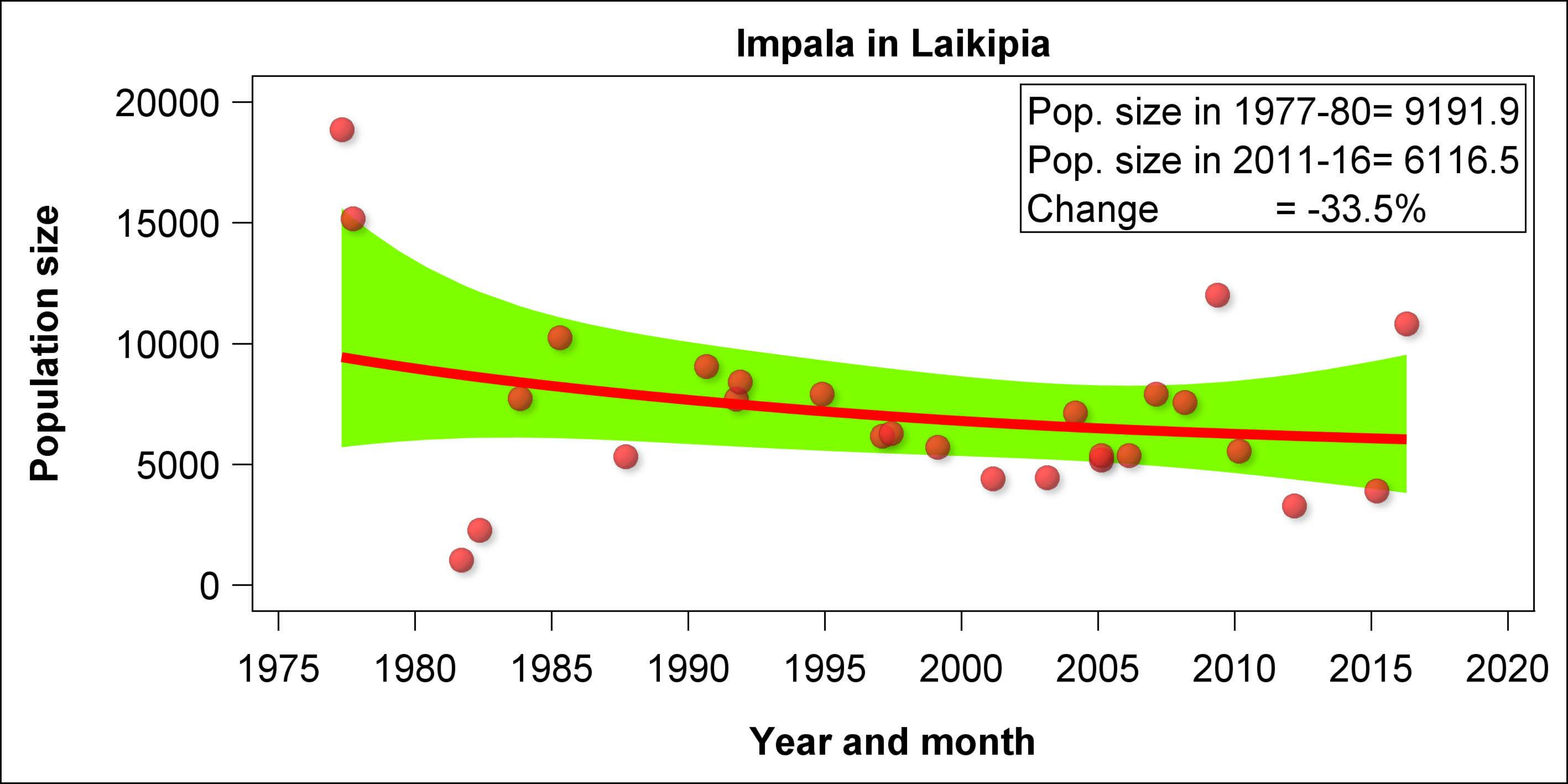

Impala in Laikipia
Pop. size in 1977-80= 9191.9
Pop. size in 2011-16= 6116.5
Change = -33.5%
Population size
20000
15000
10000
5000
0
1975
1980
1985
1990
1995
2000
2005
2010
2015
2020
Year and month

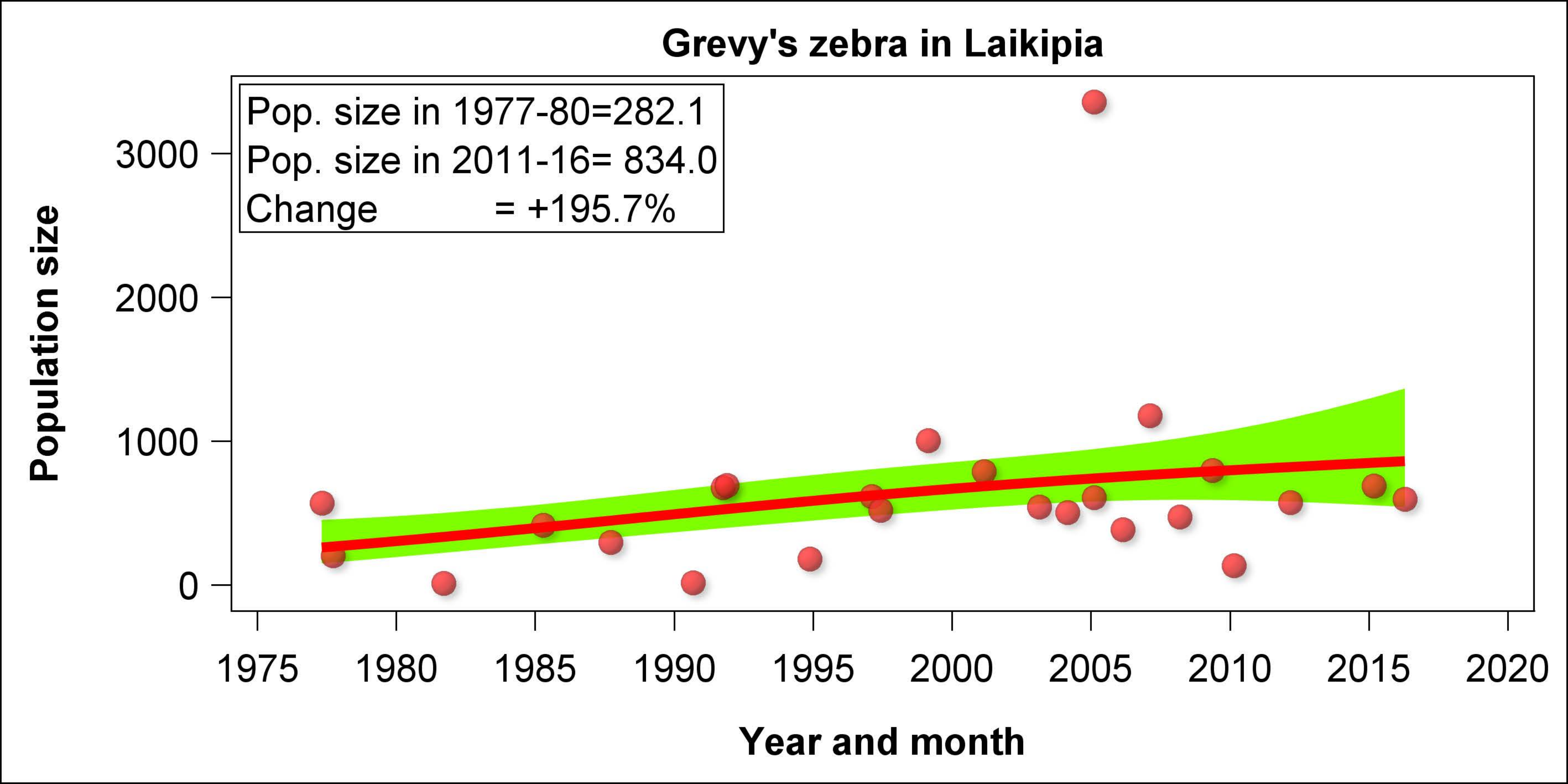
Grevy's zebra in Laikipia
Pop. size in 1977-80=282.1
Pop. size in 2011-16= 834.0
Change = +195.7%
Population size
3000
2000
1000
0
1975
1980
1985
1990
1995
2000
2005
2010
2015
2020
Year and month

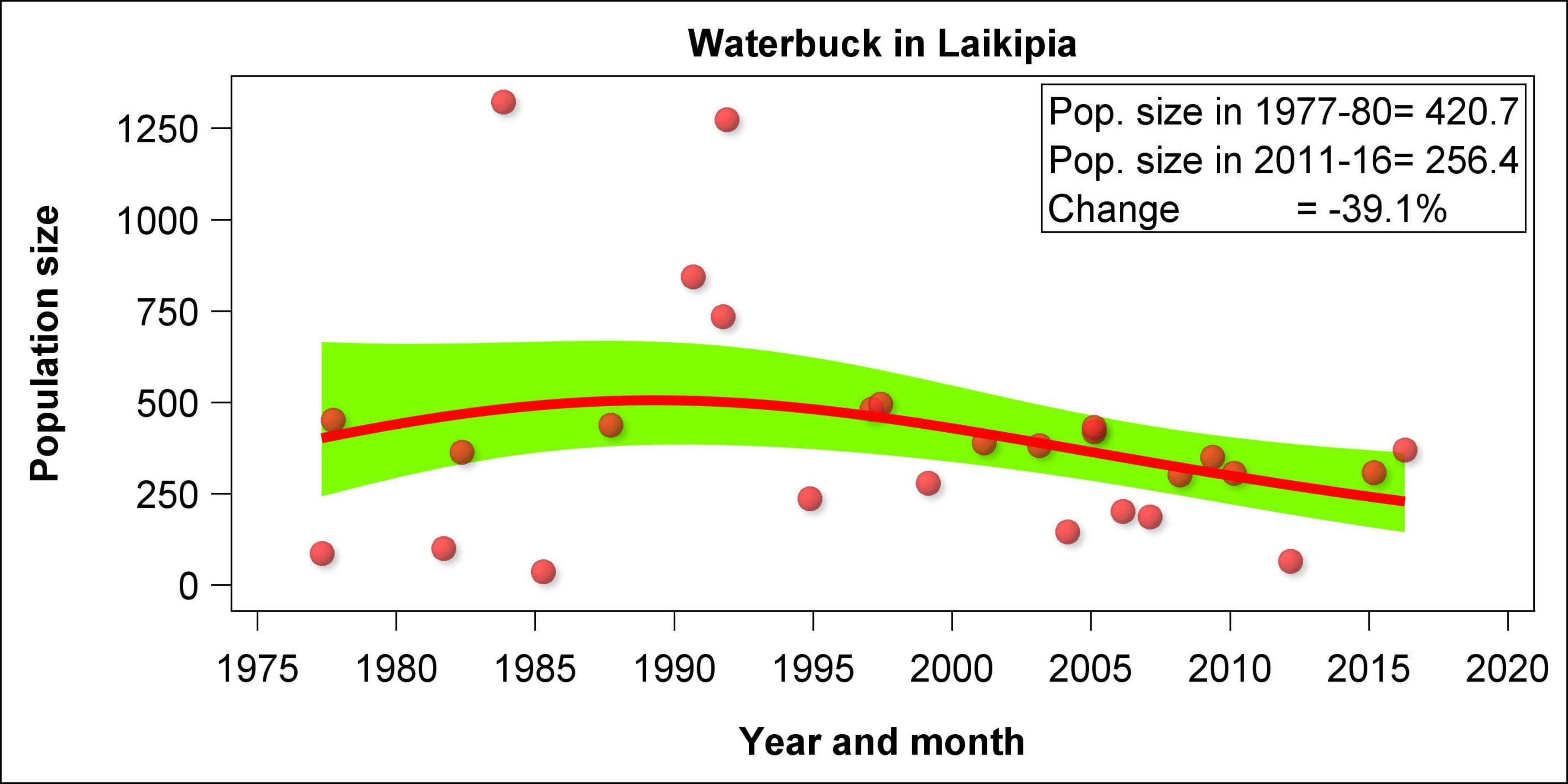

Waterbuck in Laikipia
Pop. size in 1977-80= 420.7
Pop. size in 2011-16= 256.4
Change = -39.1%
Population size
1250
1000
750
500
250
0
1975
1980
1985
1990
1995
2000
2005
2010
2015
2020
Year and month
