# Supplementary material for: Extreme Wildlife Declines and Concurrent Increase in Livestock Numbers in Kenya: What Are the Causes?
Source: PLoS One. 2016 Sep 27;11(9):e0163249. doi: 10.1371/journal.pone.0163249 (PMC5039022; doi:10.1371/journal.pone.0163249)
Supplement: S13 Fig — The solid red line is the fitted trend curve and the shaded chartreuse band is the pointwise 95% confidence band. The estimated average population size in 1977–1980 and 2011–2015 and the percentage change in population size between the two periods are provided in the inset. (PDF) [file pone.0163249.s023.pdf]

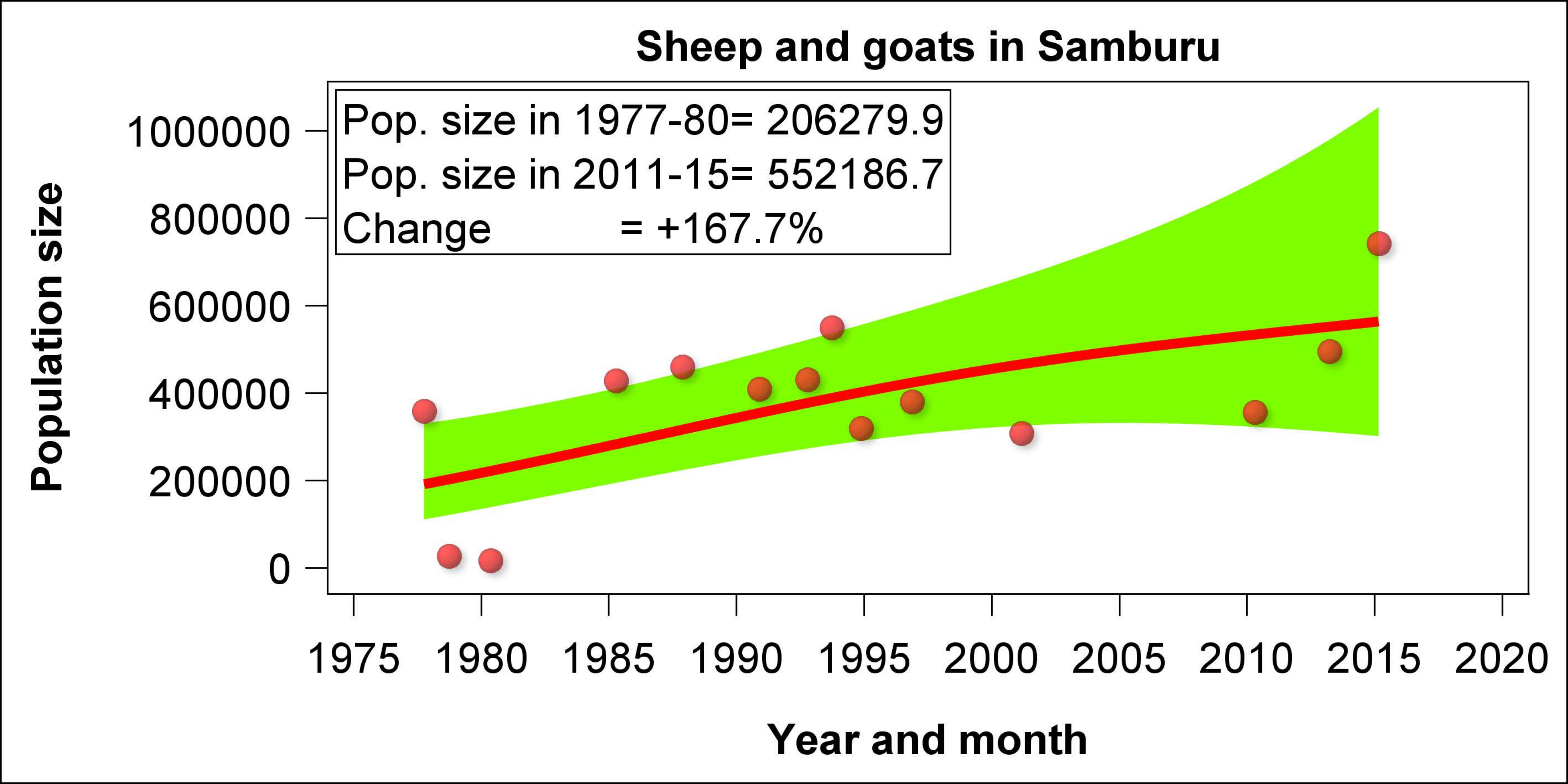

Sheep and goats in Samburu
Pop. size in 1977-80= 206279.9
Pop. size in 2011-15= 552186.7
Change = +167.7%
Population size
1000000
800000
600000
400000
200000
0
1975
1980
1985
1990
1995
2000
2005
2010
2015
2020
Year and month

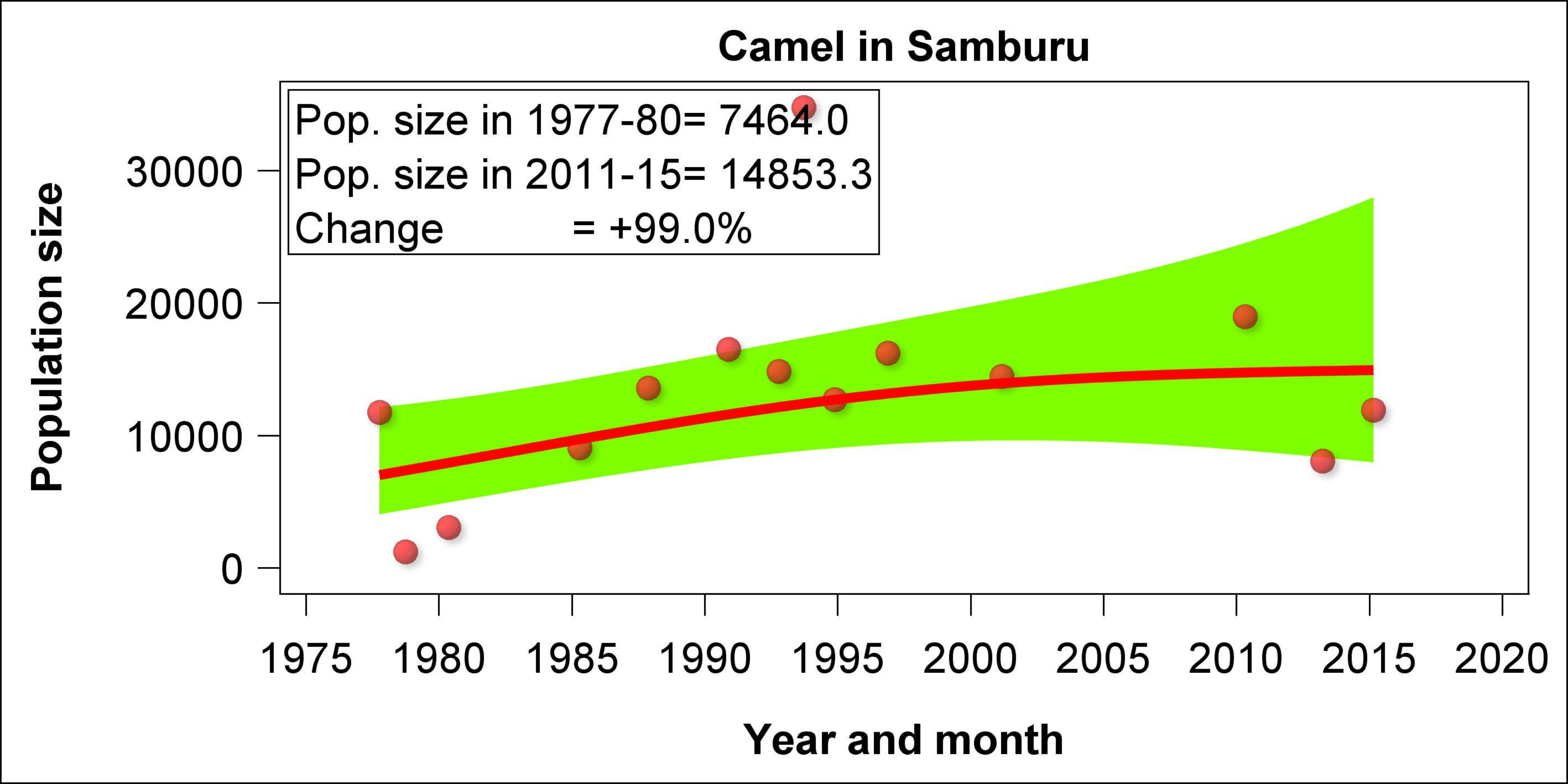
Camel in Samburu
Pop. size in 1977-80= 7464.0
Pop. size in 2011-15= 14853.3
Change = +99.0%
Population size
30000
20000
10000
0
1975
1980
1985
1990
1995
2000
2005
2010
2015
2020
Year and month

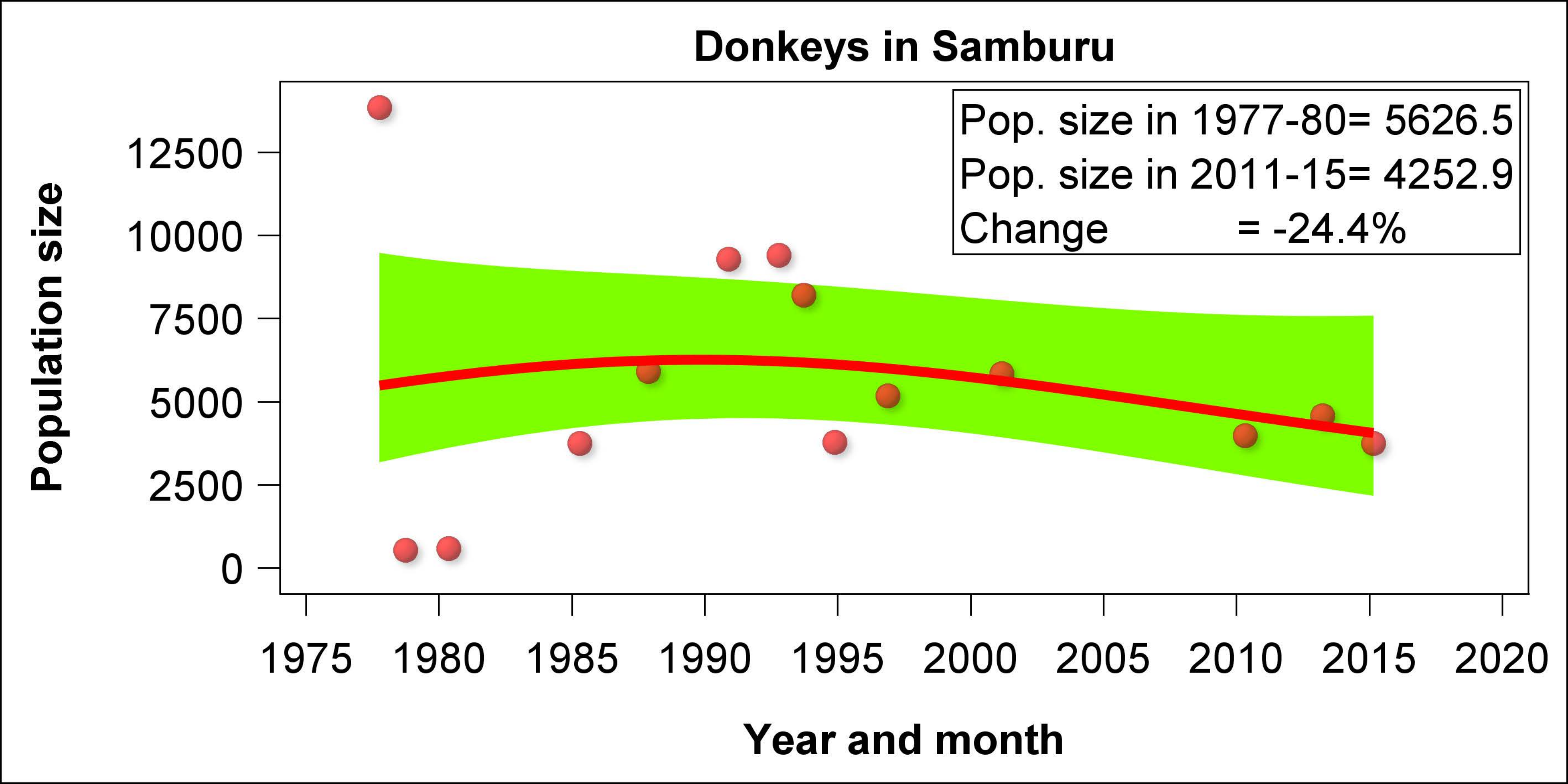

Donkeys in Samburu
Pop. size in 1977-80= 5626.5
Pop. size in 2011-15= 4252.9
Change = -24.4%
Population size
12500
10000
7500
5000
2500
0
1975
1980
1985
1990
1995
2000
2005
2010
2015
2020
Year and month

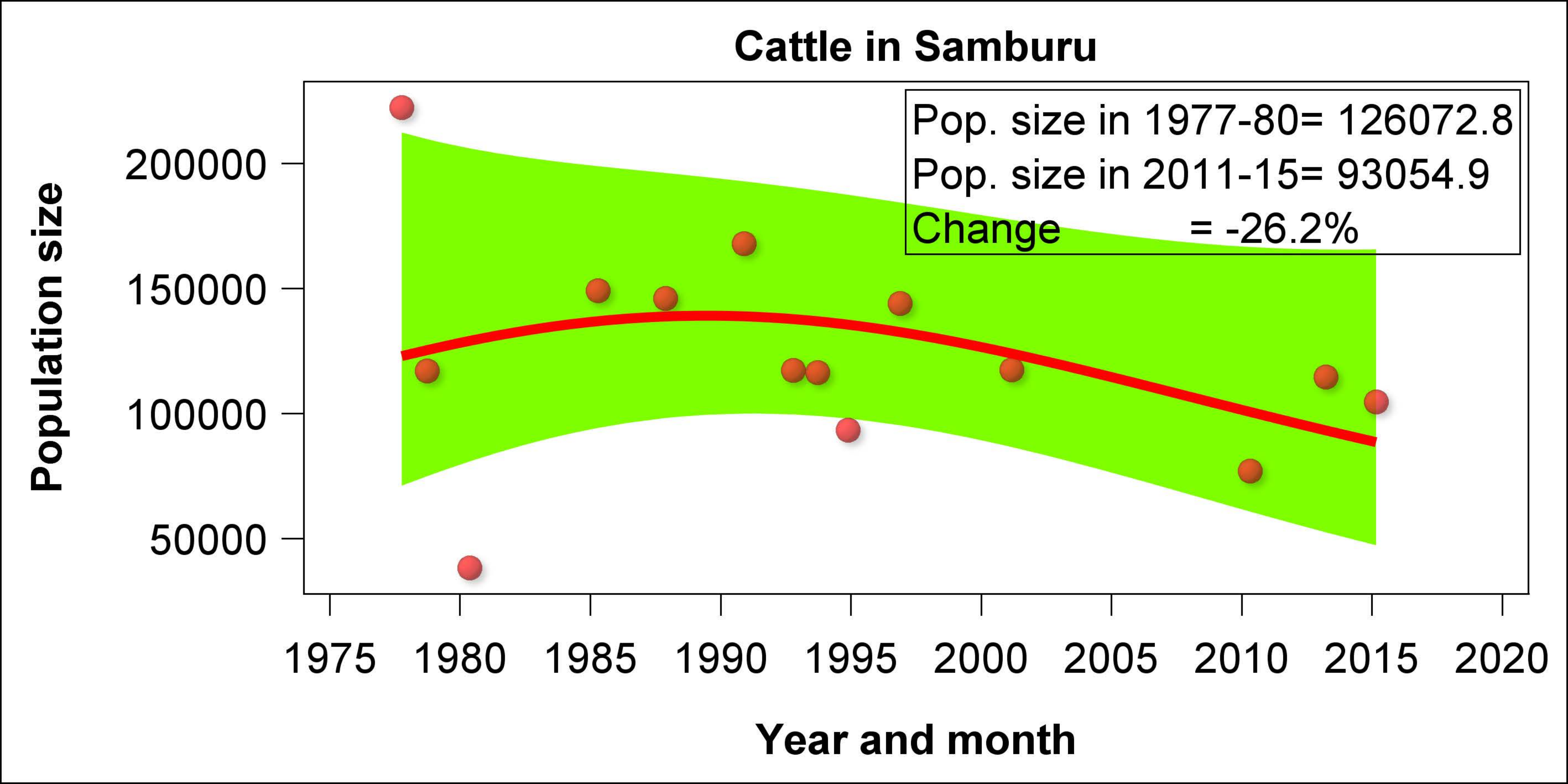

Cattle in Samburu
Pop. size in 1977-80= 126072.8
Pop. size in 2011-15= 93054.9
Change = -26.2%
200000
150000
100000
50000
Population size
1975
1980
1985
1990
1995
2000
2005
2010
2015
2020
Year and month

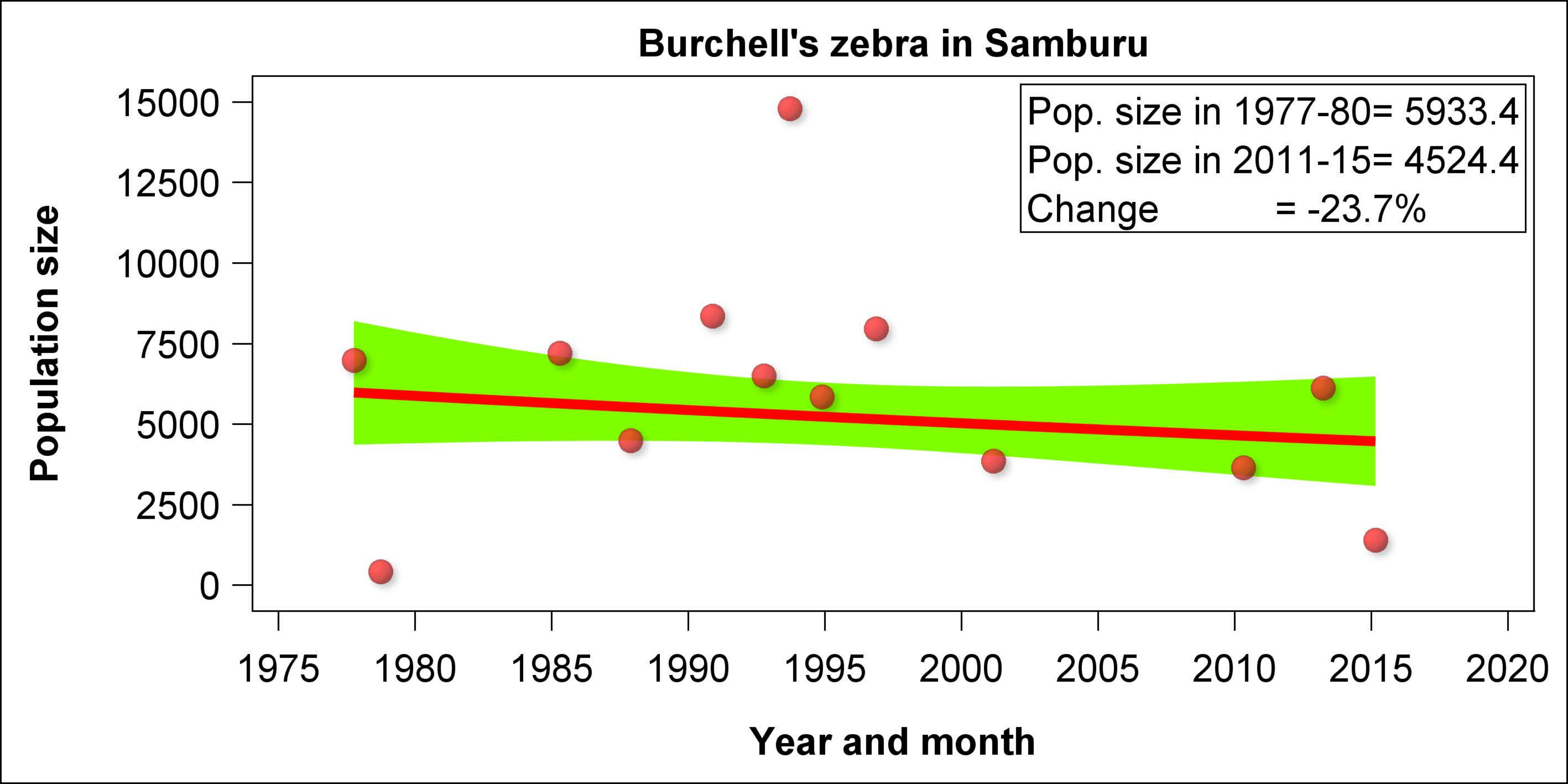
Burchell's zebra in Samburu
Pop. size in 1977-80= 5933.4
Pop. size in 2011-15= 4524.4
Change = -23.7%
Population size
15000
12500
10000
7500
5000
2500
0
1975
1980
1985
1990
1995
2000
2005
2010
2015
2020
Year and month

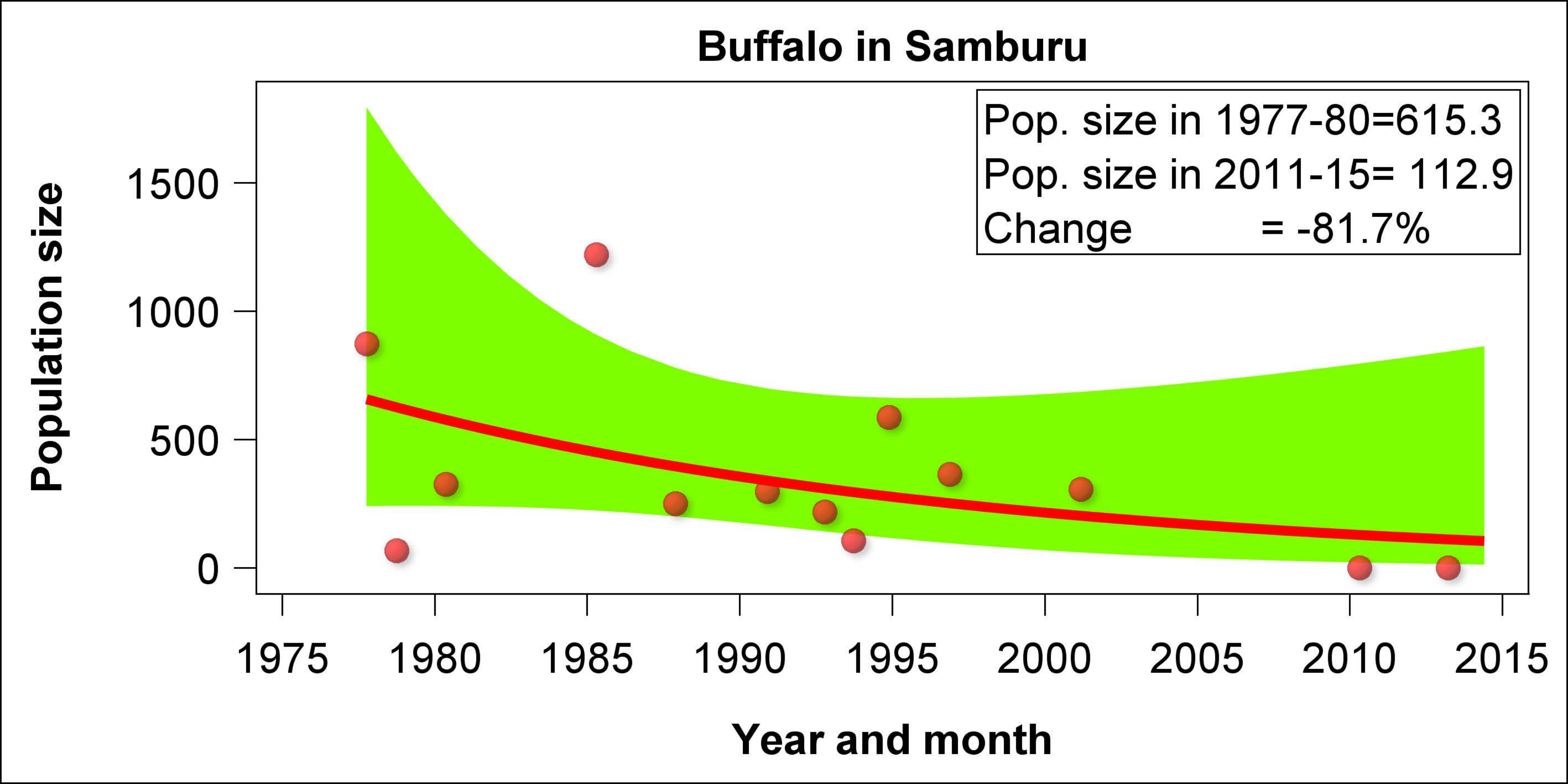

Buffalo in Samburu
Pop. size in 1977-80=615.3
Pop. size in 2011-15= 112.9
Change = -81.7%
Population size
1500
1000
500
0
1975
1980
1985
1990
1995
2000
2005
2010
2015
Year and month

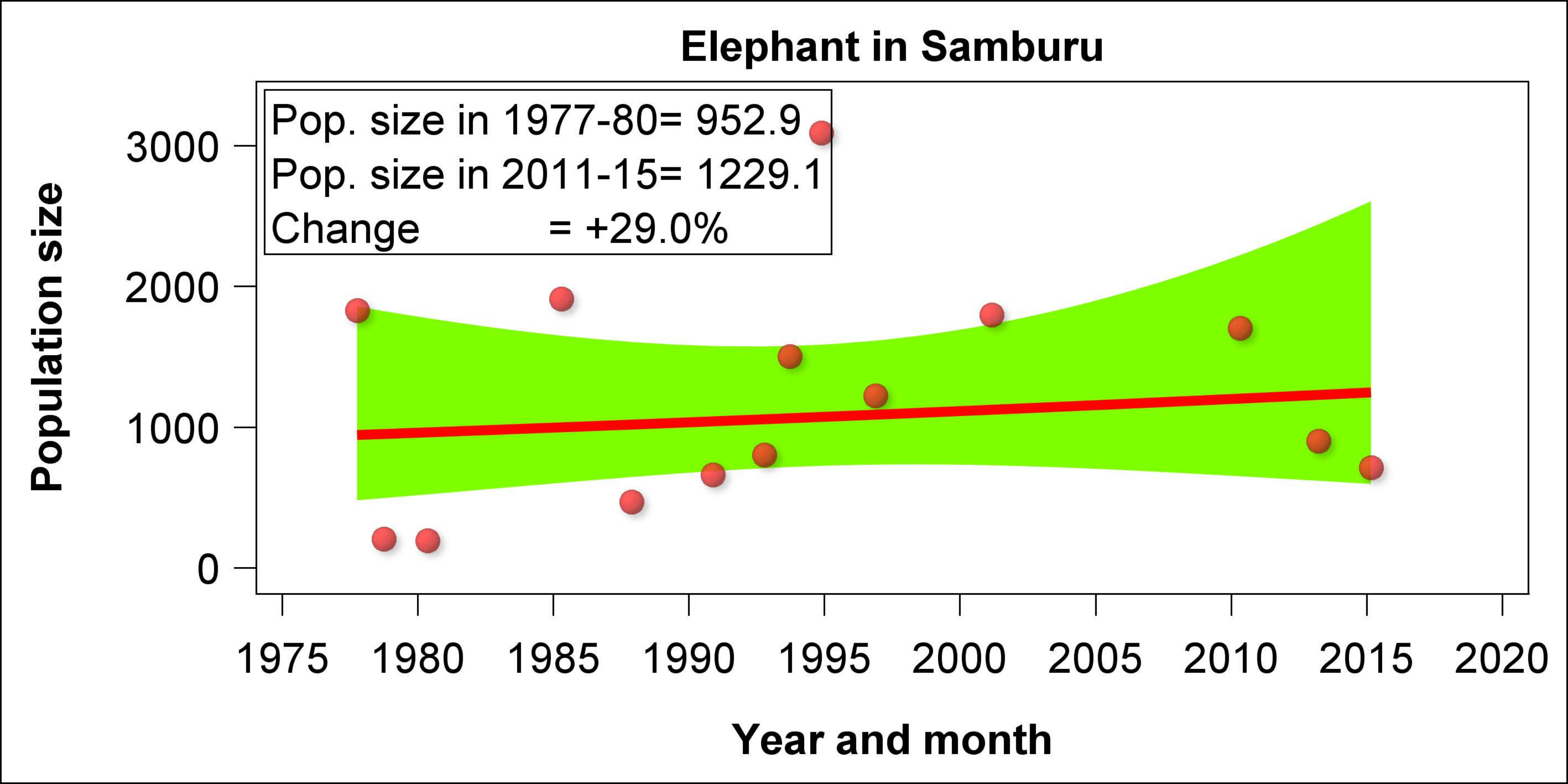
Elephant in Samburu
Pop. size in 1977-80= 952.9
Pop. size in 2011-15= 1229.1
Change = +29.0%
Population size
0
1000
2000
3000
1975
1980
1985
1990
1995
2000
2005
2010
2015
2020
Year and month

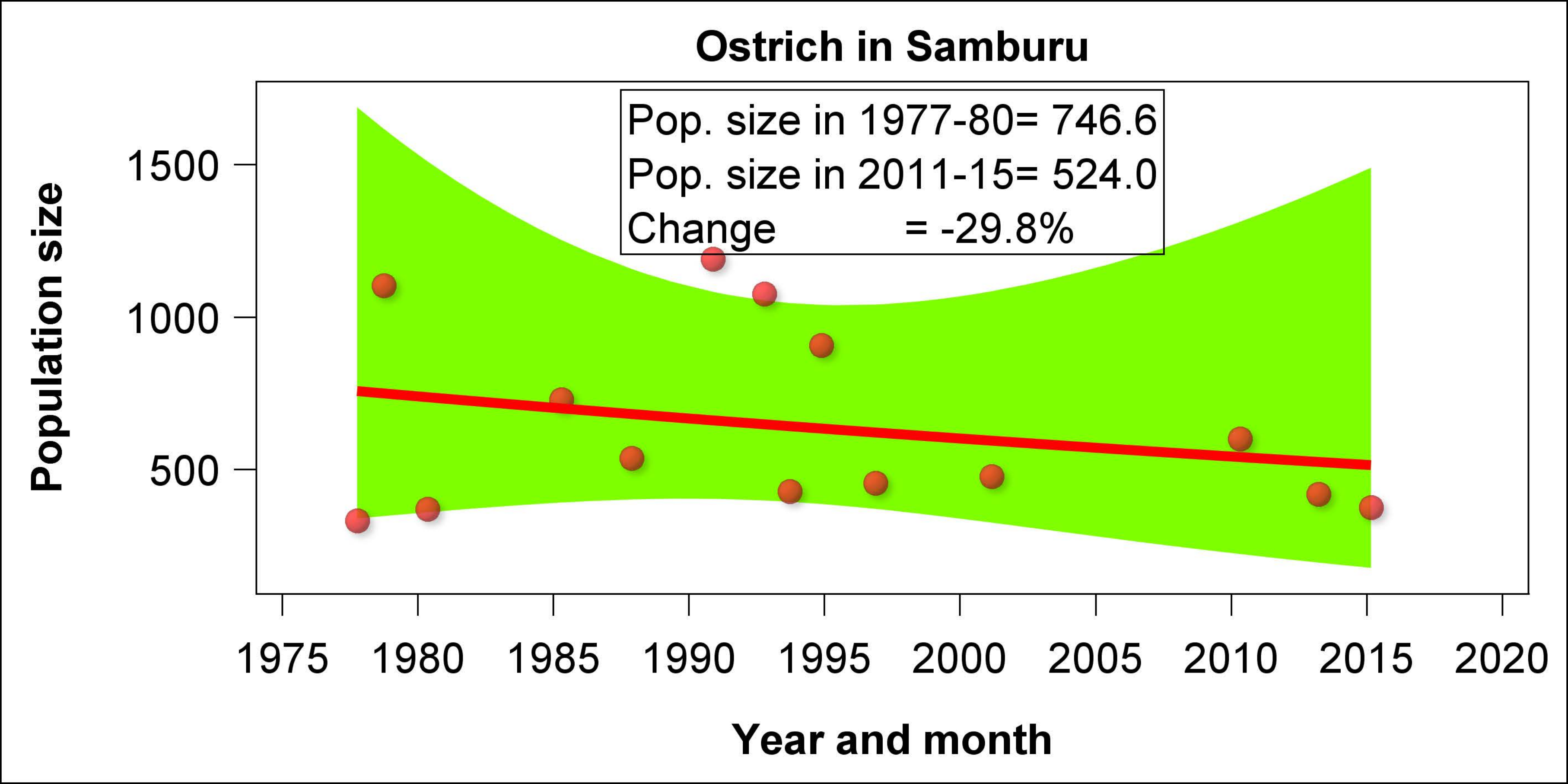
Ostrich in Samburu
Pop. size in 1977-80= 746.6
Pop. size in 2011-15= 524.0
Change = -29.8%
Population size
1500
1000
500
1975
1980
1985
1990
1995
2000
2005
2010
2015
2020
Year and month

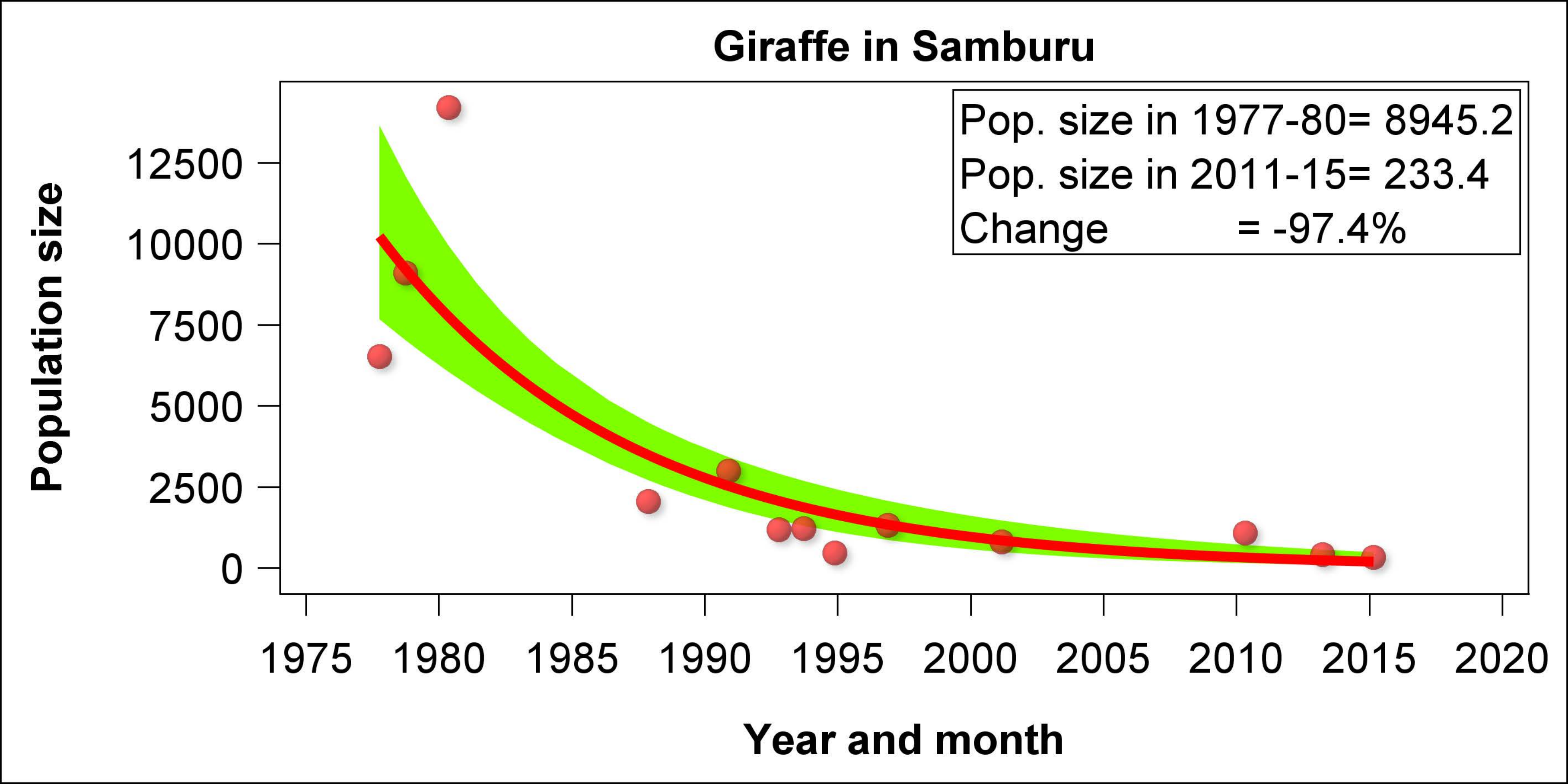

Giraffe in Samburu
Pop. size in 1977-80= 8945.2
Pop. size in 2011-15= 233.4
Change = -97.4%
Population size
12500
10000
7500
5000
2500
0
1975
1980
1985
1990
1995
2000
2005
2010
2015
2020
Year and month

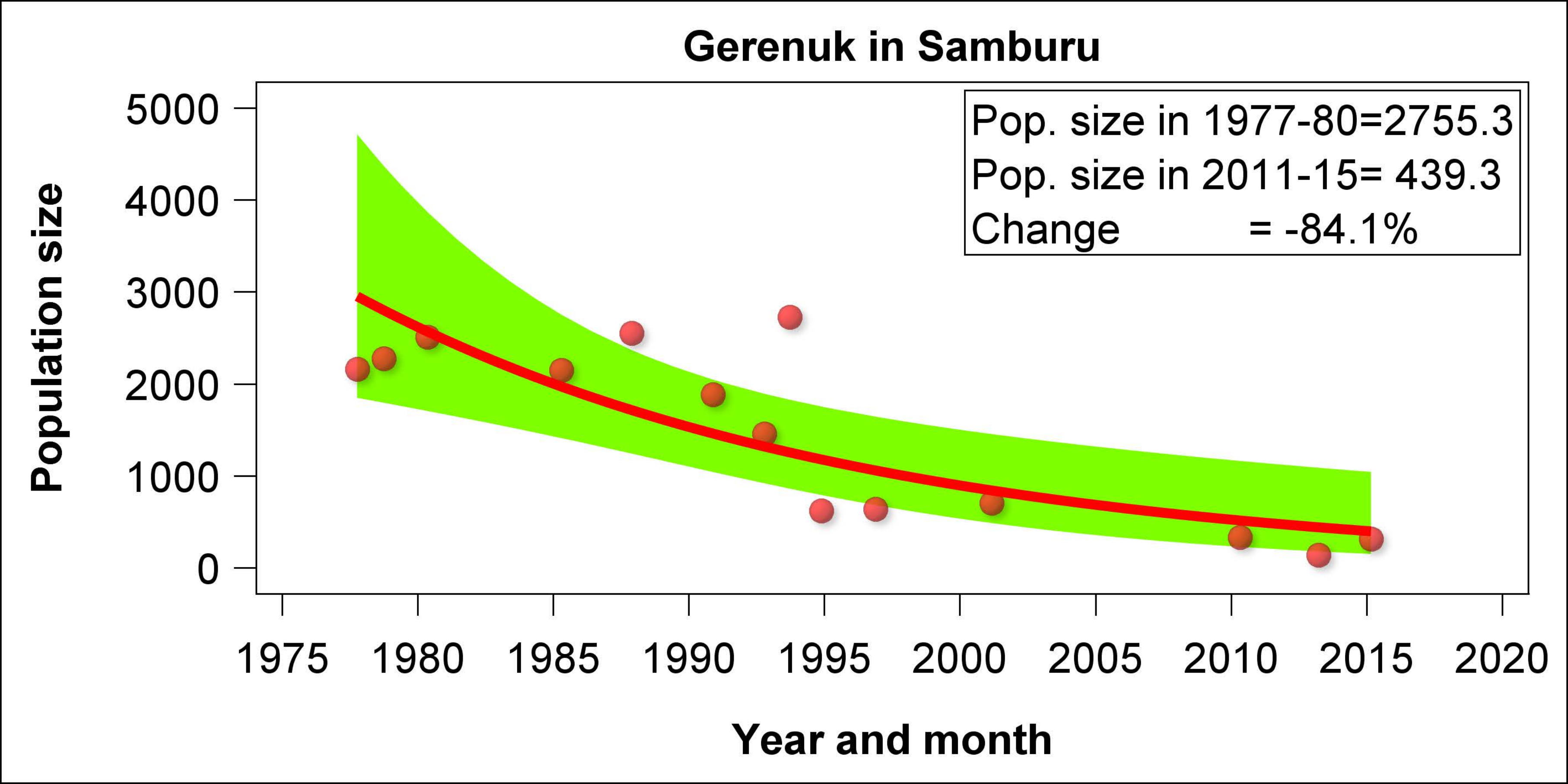
Gerenuk in Samburu
Pop. size in 1977-80=2755.3
Pop. size in 2011-15= 439.3
Change = -84.1%
Population size
5000
4000
3000
2000
1000
0
1975
1980
1985
1990
1995
2000
2005
2010
2015
2020
Year and month

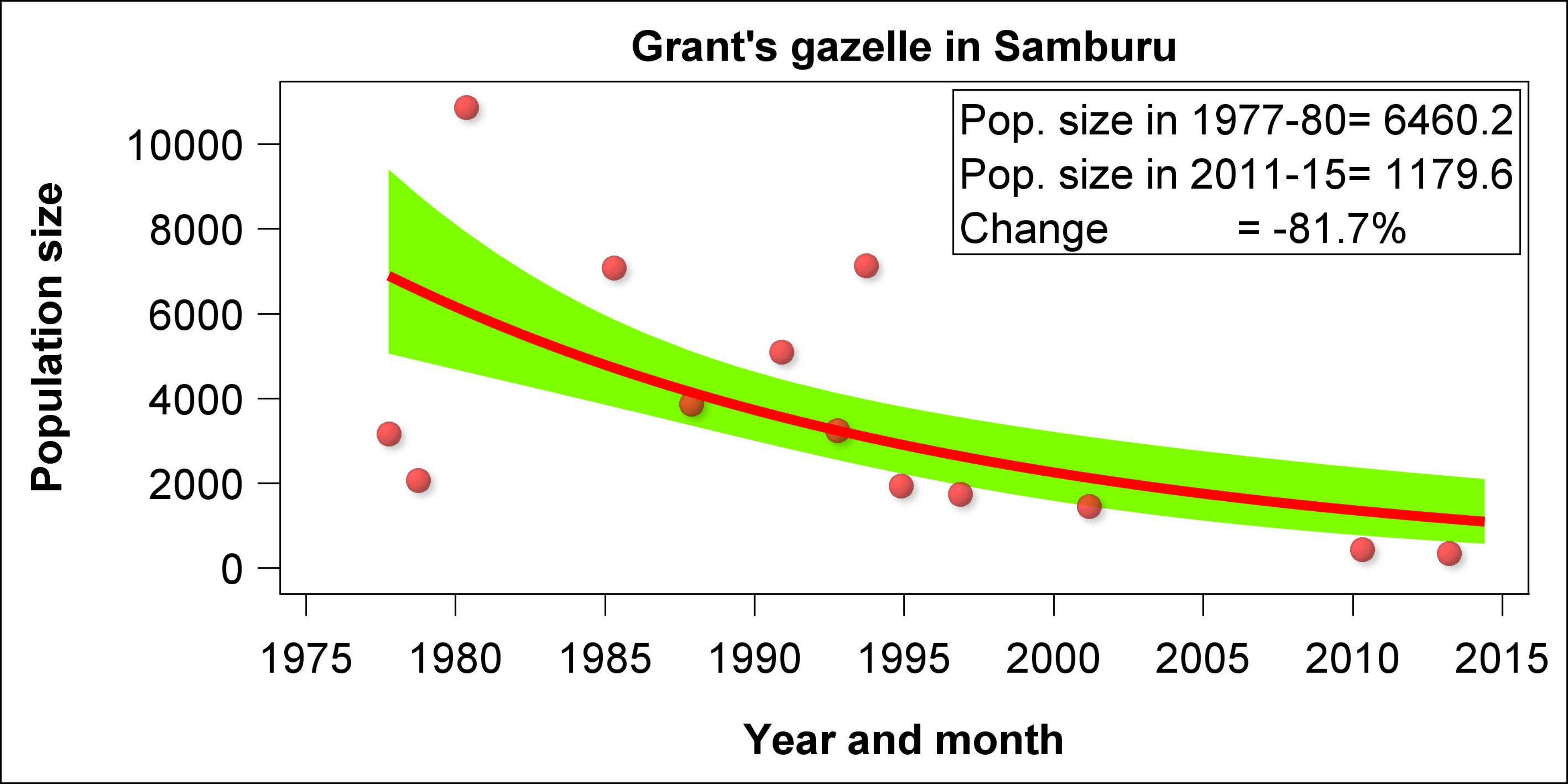

Grant's gazelle in Samburu
Pop. size in 1977-80= 6460.2
Pop. size in 2011-15= 1179.6
Change = -81.7%
Population size
10000
8000
6000
4000
2000
0
1975
1980
1985
1990
1995
2000
2005
2010
2015
Year and month

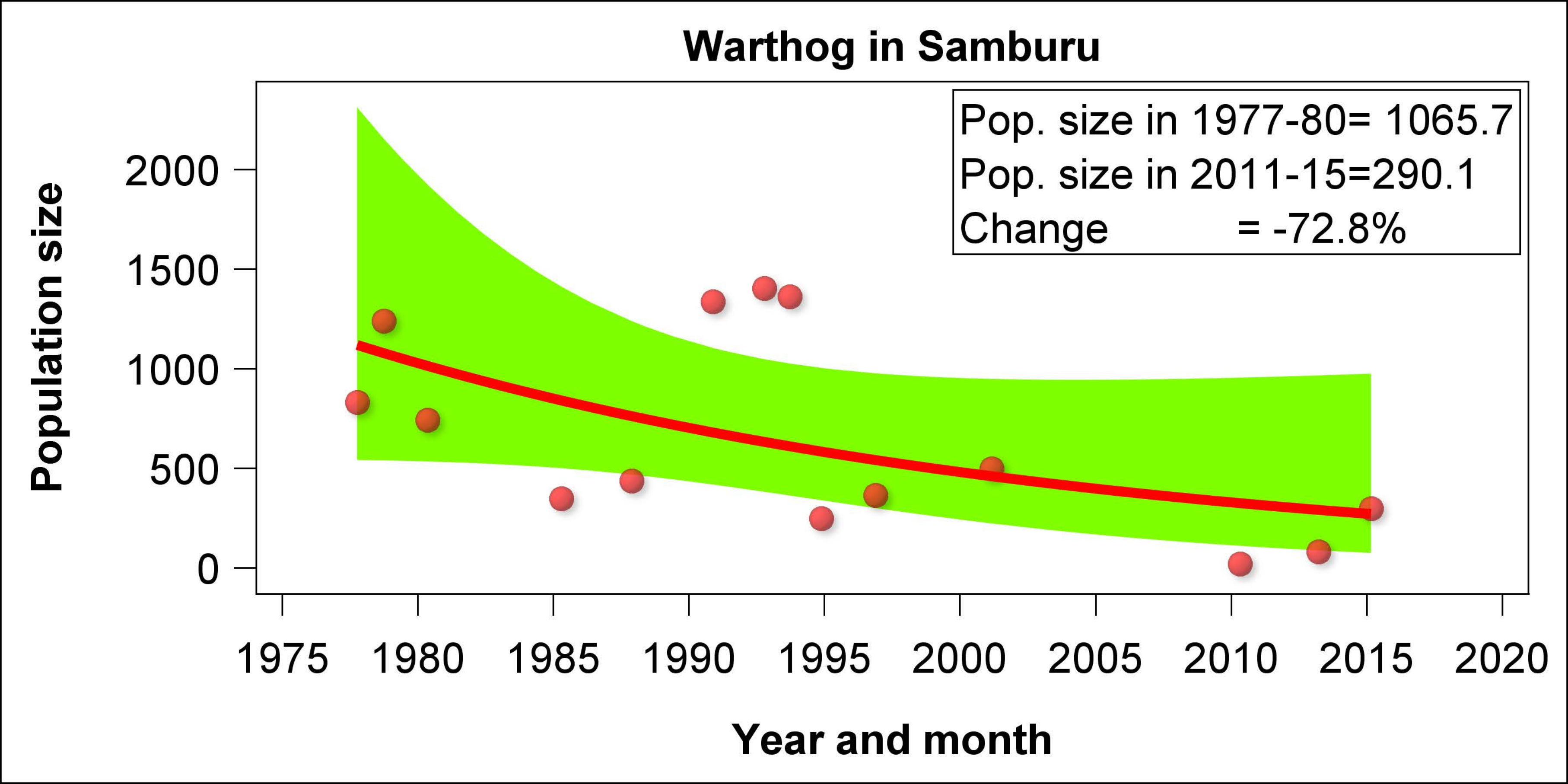
Warthog in Samburu
Pop. size in 1977-80= 1065.7
Pop. size in 2011-15=290.1
Change = -72.8%
Population size
0
500
1000
1500
2000
1975
1980
1985
1990
1995
2000
2005
2010
2015
2020
Year and month

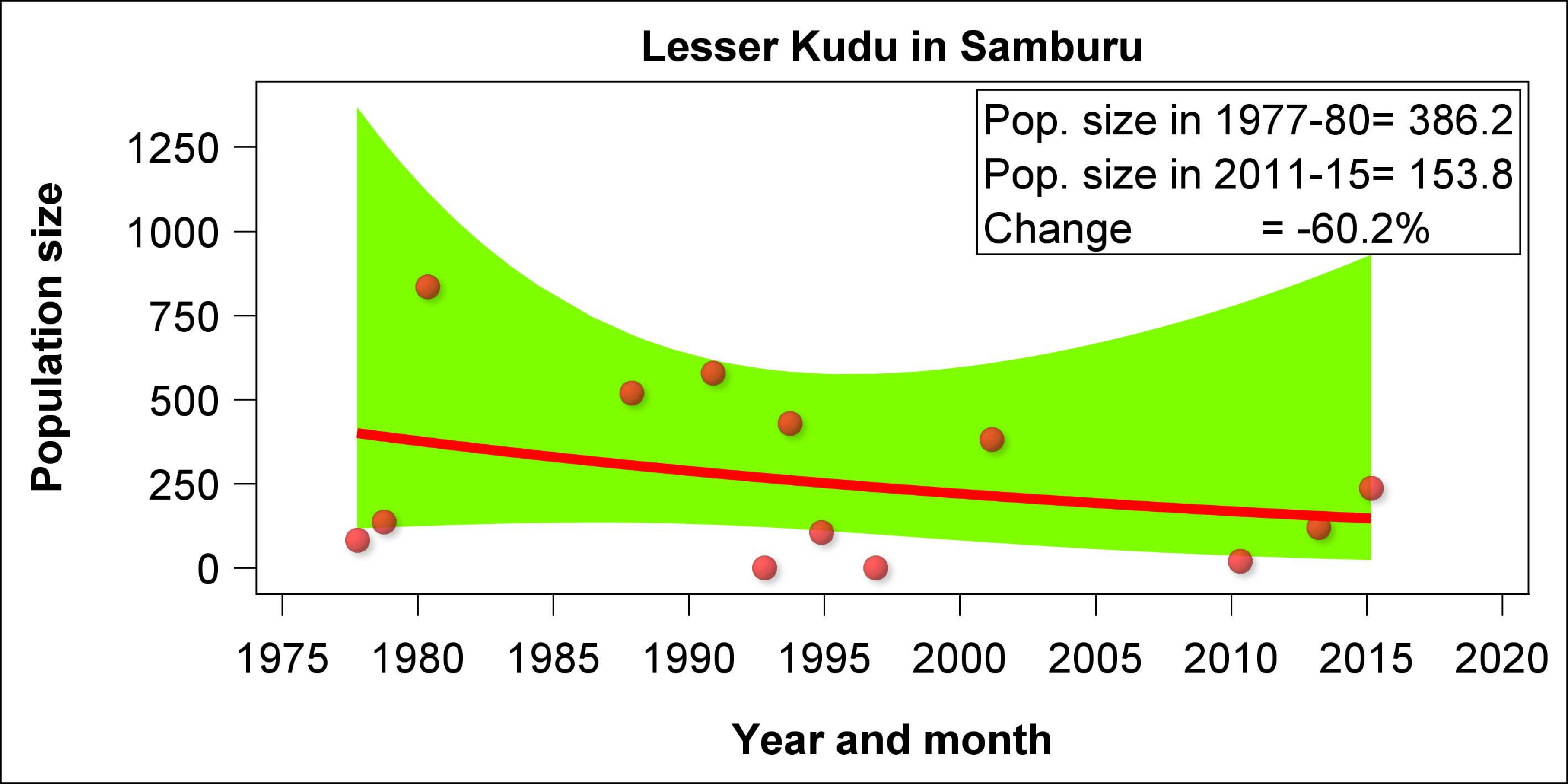
Lesser Kudu in Samburu
Pop. size in 1977-80= 386.2
Pop. size in 2011-15= 153.8
Change = -60.2%
Population size
1250
1000
750
500
250
0
1975
1980
1985
1990
1995
2000
2005
2010
2015
2020
Year and month

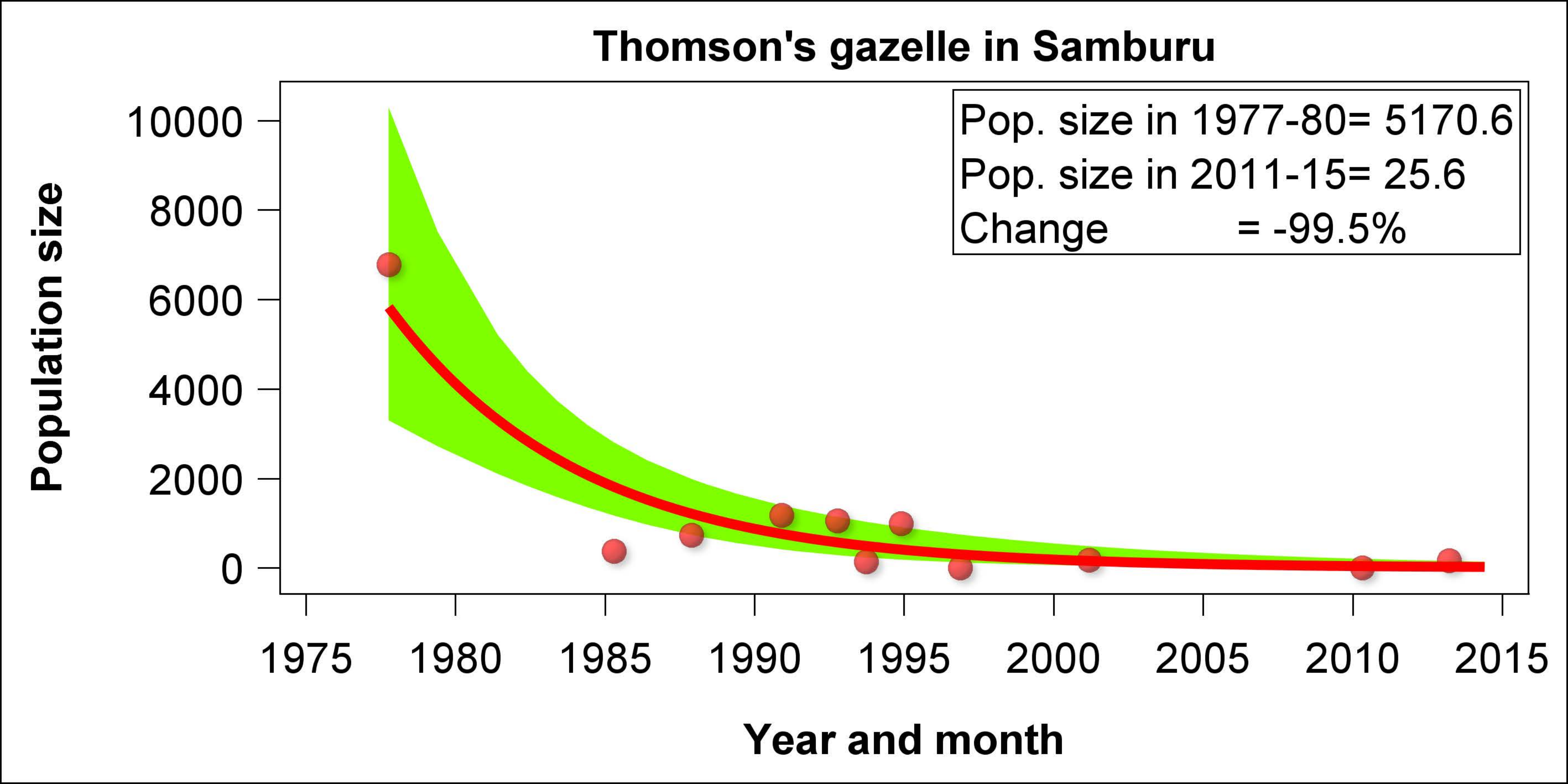

Thomson's gazelle in Samburu
Pop. size in 1977-80= 5170.6
Pop. size in 2011-15= 25.6
Change = -99.5%
Population size
10000
8000
6000
4000
2000
0
1975
1980
1985
1990
1995
2000
2005
2010
2015
Year and month

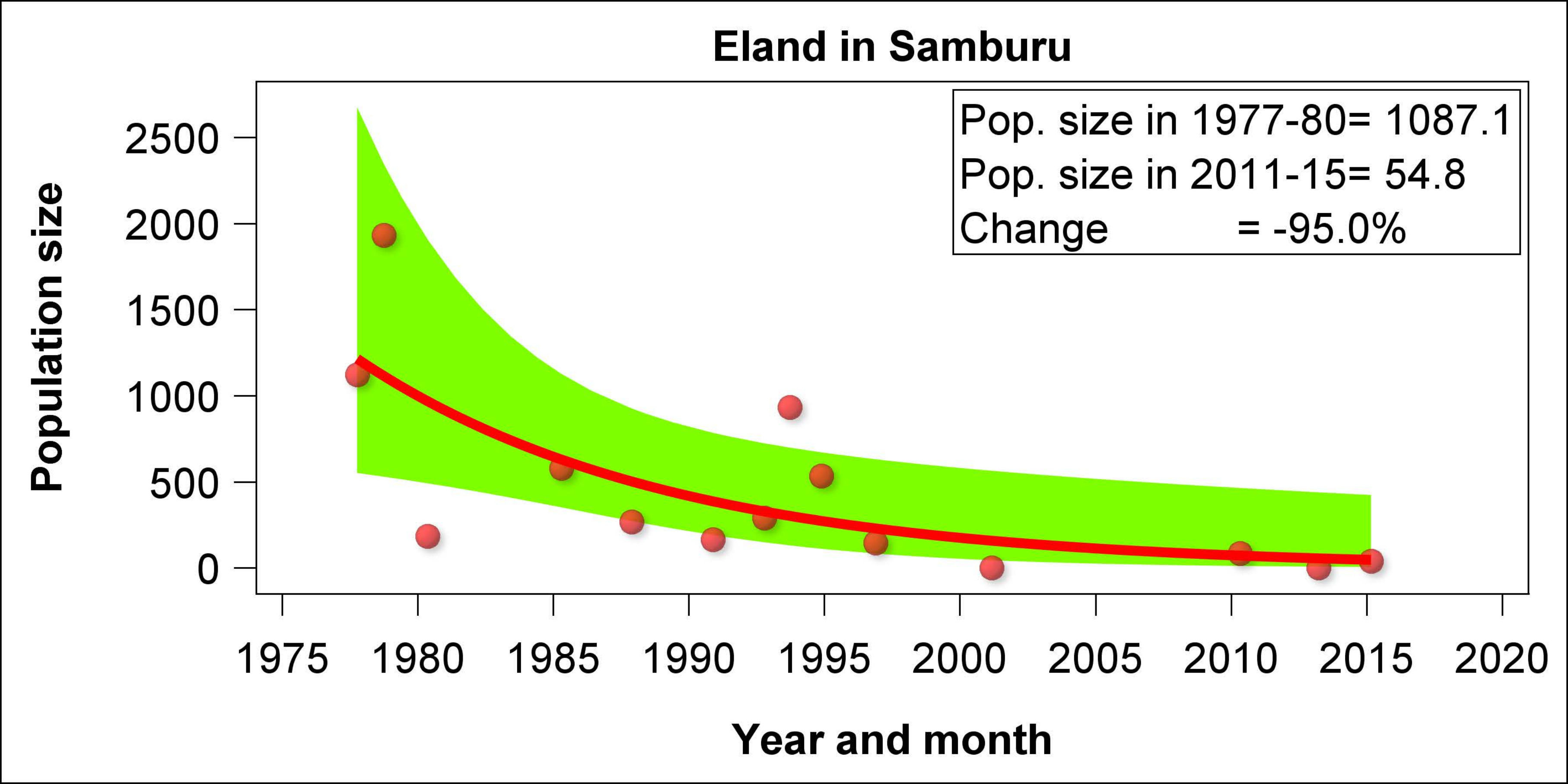

Eland in Samburu
Pop. size in 1977-80= 1087.1
Pop. size in 2011-15= 54.8
Change = -95.0%
Population size
2500
2000
1500
1000
500
0
1975
1980
1985
1990
1995
2000
2005
2010
2015
2020
Year and month

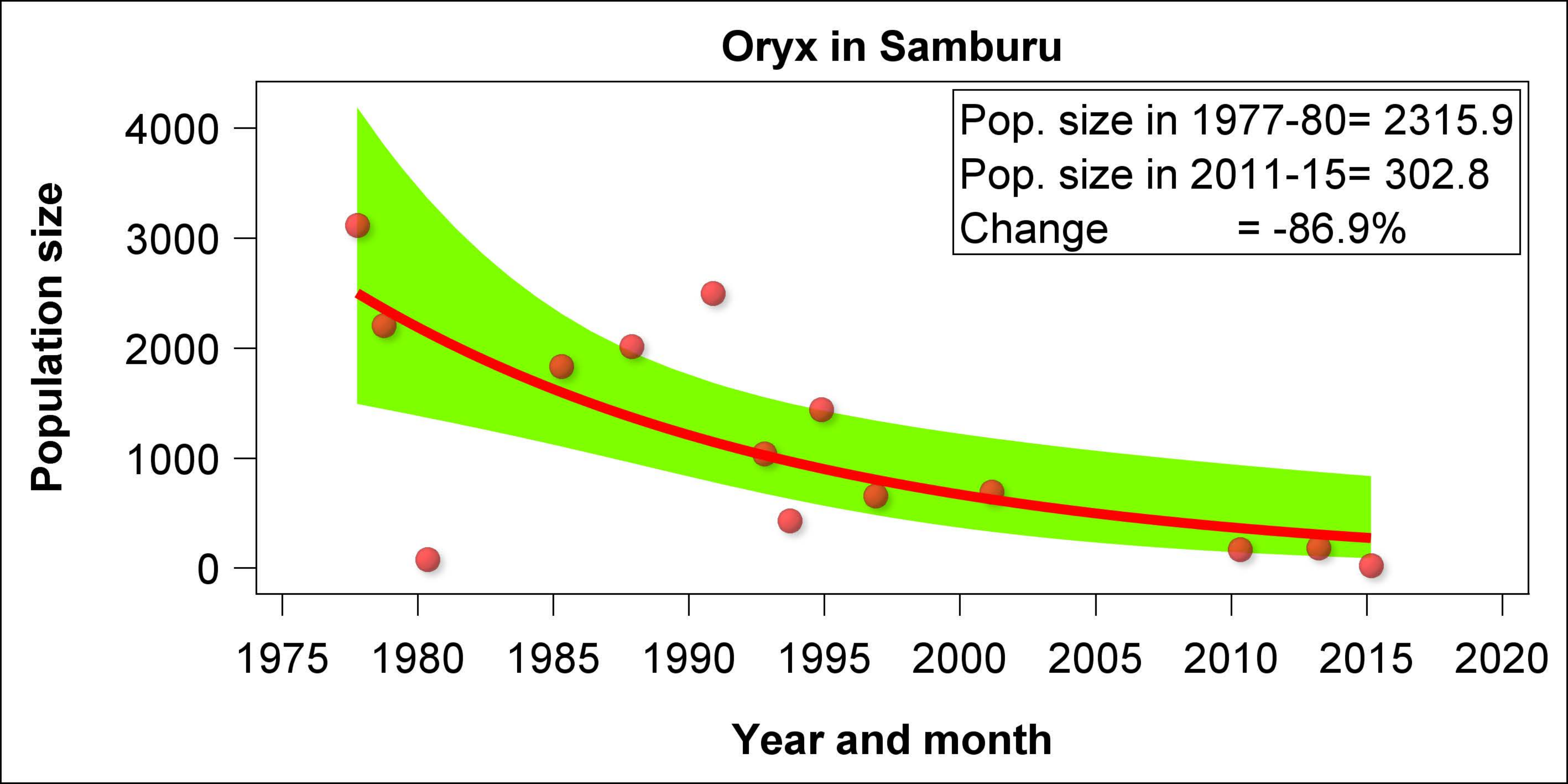

Oryx in Samburu
Pop. size in 1977-80= 2315.9
Pop. size in 2011-15= 302.8
Change = -86.9%
Population size
4000
3000
2000
1000
0
1975
1980
1985
1990
1995
2000
2005
2010
2015
2020
Year and month

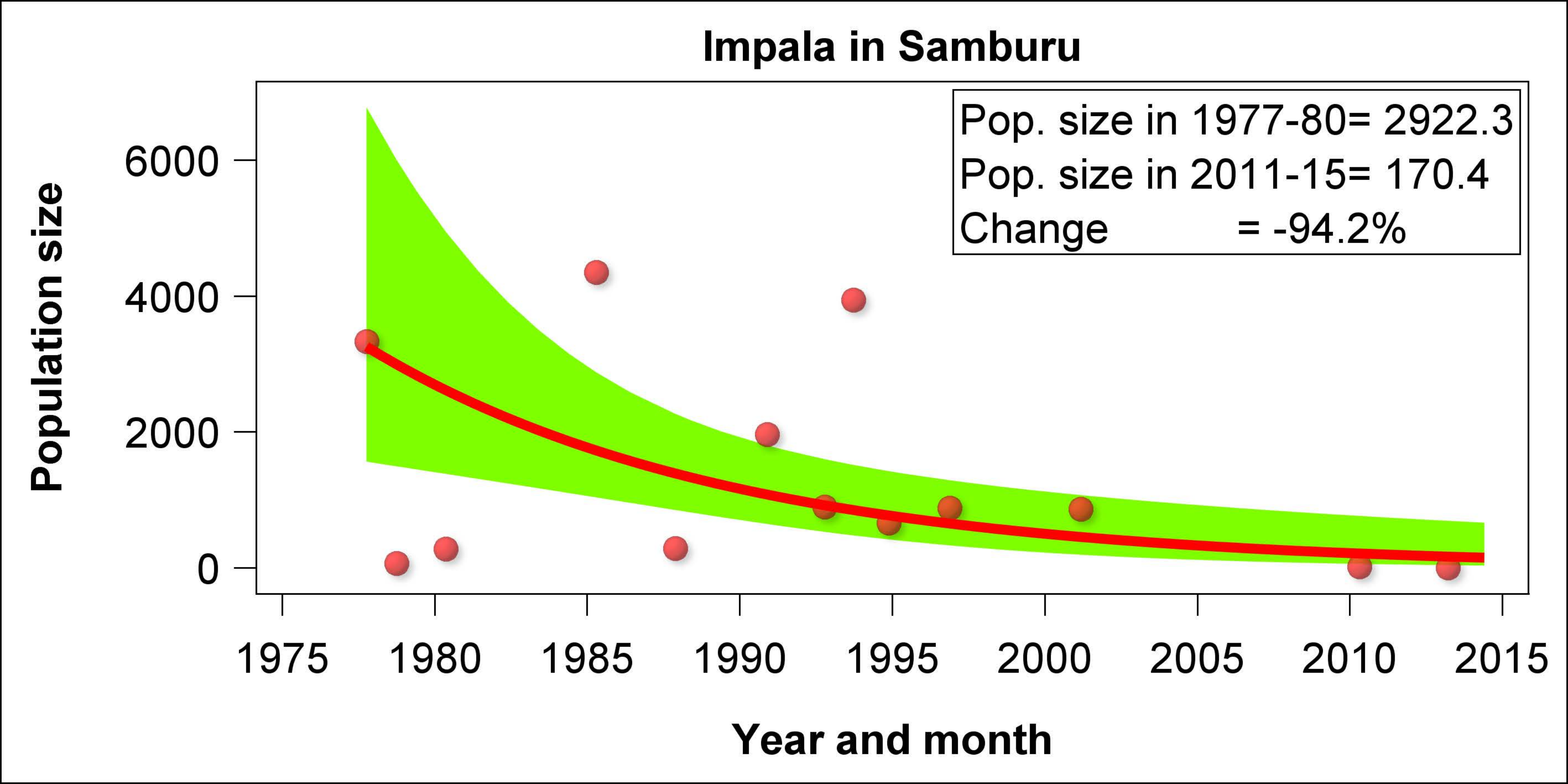

Impala in Samburu
Pop. size in 1977-80= 2922.3
Pop. size in 2011-15= 170.4
Change = -94.2%
6000
4000
2000
0
Population size
1975
1980
1985
1990
1995
2000
2005
2010
2015
Year and month

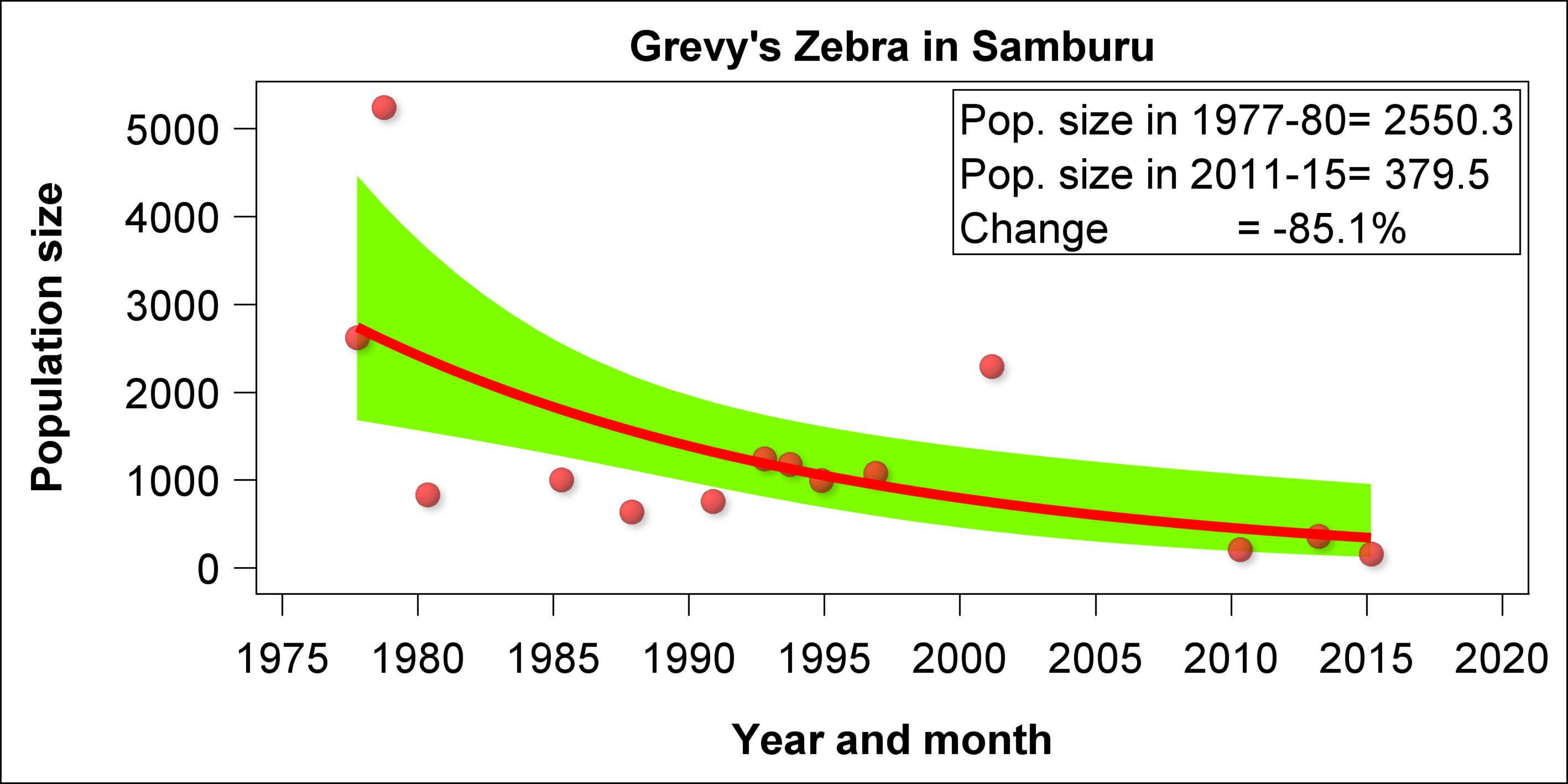
Grevy's Zebra in Samburu
Pop. size in 1977-80= 2550.3
Pop. size in 2011-15= 379.5
Change = -85.1%
Population size
5000
4000
3000
2000
1000
0
1975
1980
1985
1990
1995
2000
2005
2010
2015
2020
Year and month

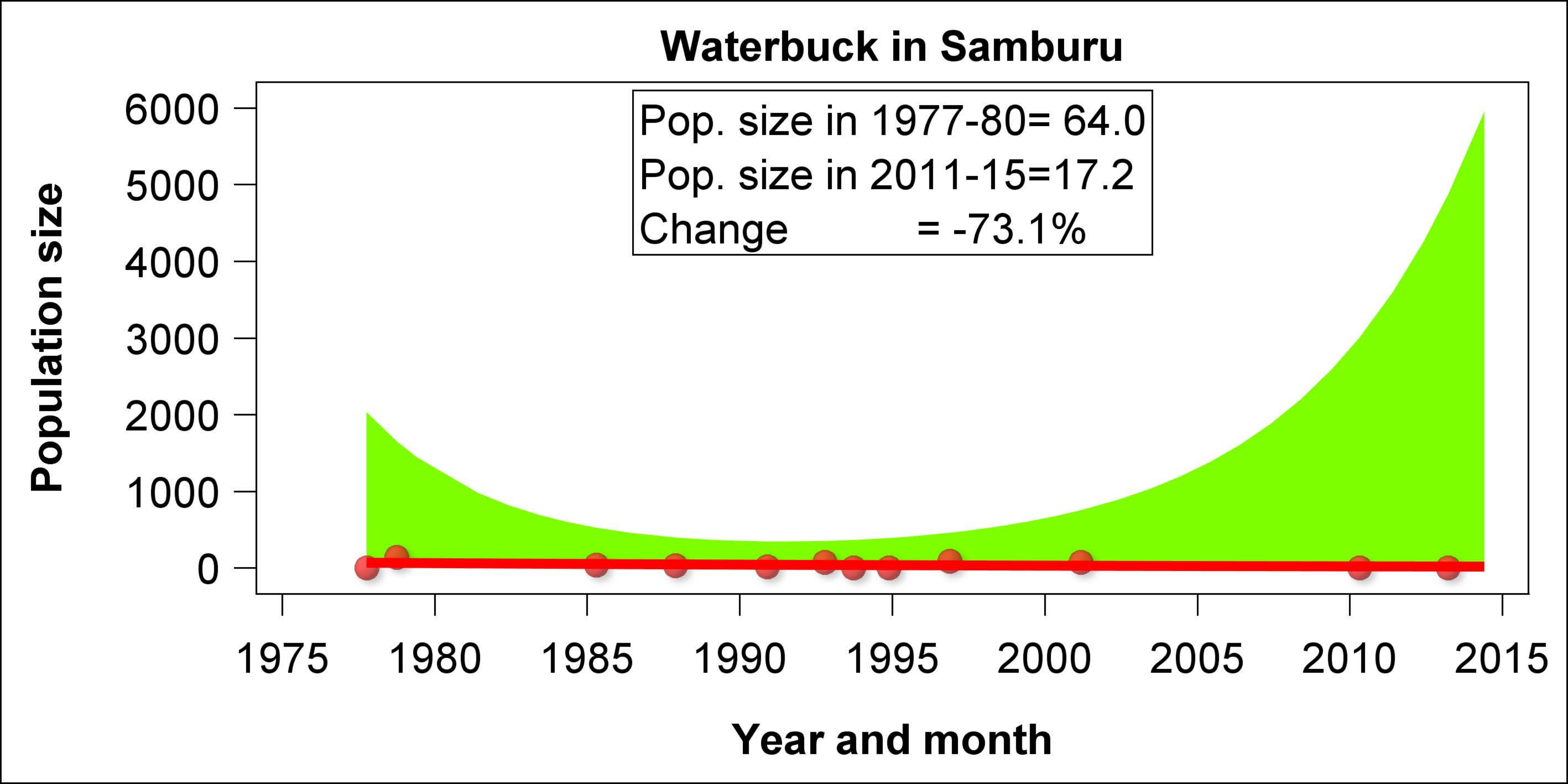
Waterbuck in Samburu
Pop. size in 1977-80= 64.0
Pop. size in 2011-15=17.2
Change = -73.1%
Population size
6000
5000
4000
3000
2000
1000
0
1975
1980
1985
1990
1995
2000
2005
2010
2015
Year and month
