# Supplementary material for: Extreme Wildlife Declines and Concurrent Increase in Livestock Numbers in Kenya: What Are the Causes?
Source: PLoS One. 2016 Sep 27;11(9):e0163249. doi: 10.1371/journal.pone.0163249 (PMC5039022; doi:10.1371/journal.pone.0163249)
Supplement: S14 Fig — The solid red line is the fitted trend curve and the shaded chartreuse band is the pointwise 95% confidence band. The estimated average population size in 1977–1980 and 2011–2015 and the percentage change in population size between the two periods are provided in the inset. (PDF) [file pone.0163249.s024.pdf]

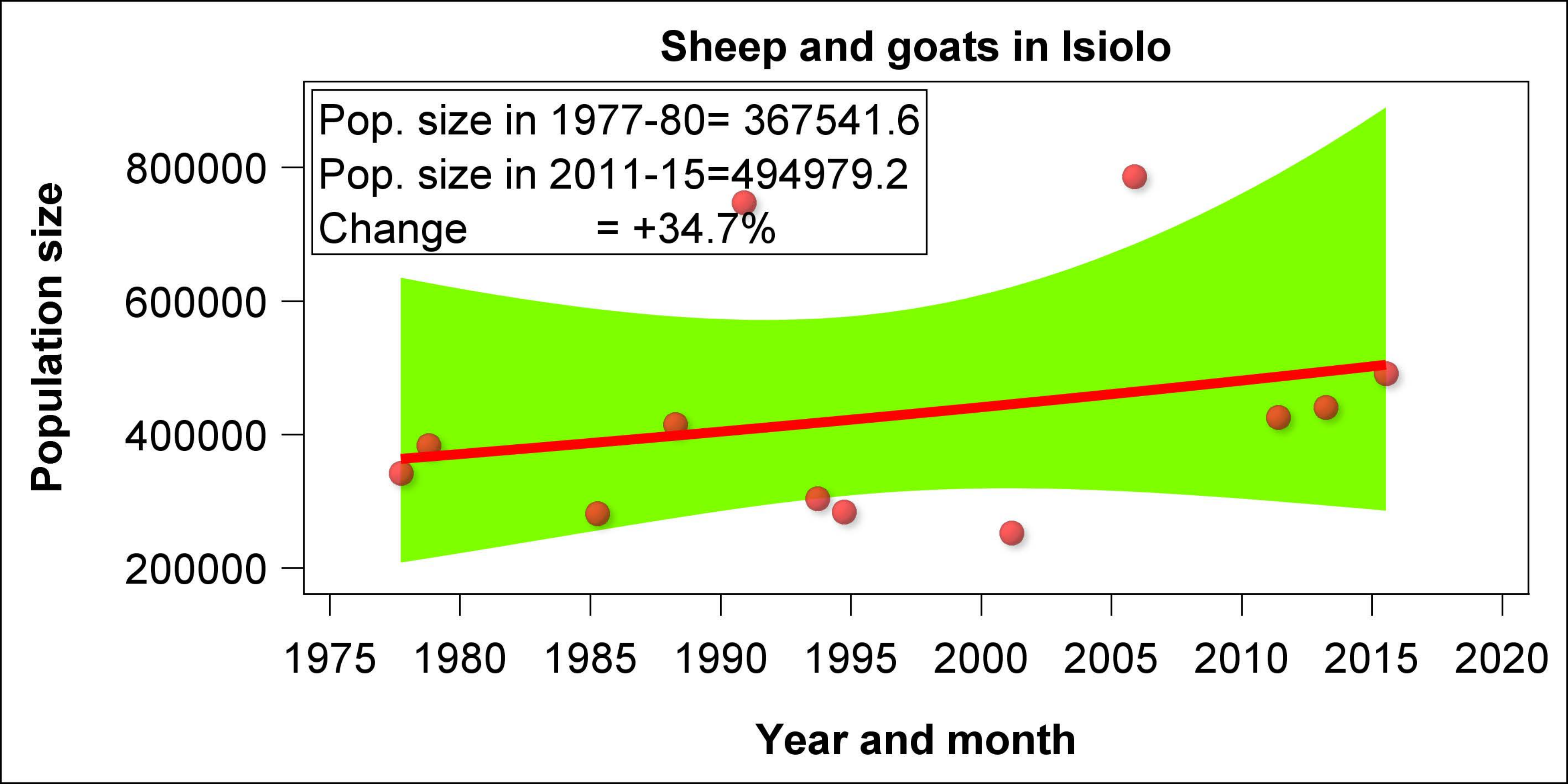

Sheep and goats in Isiolo
Pop. size in 1977-80= 367541.6
Pop. size in 2011-15=494979.2
Change = +34.7%
800000
600000
400000
200000
Population size
1975
1980
1985
1990
1995
2000
2005
2010
2015
2020
Year and month

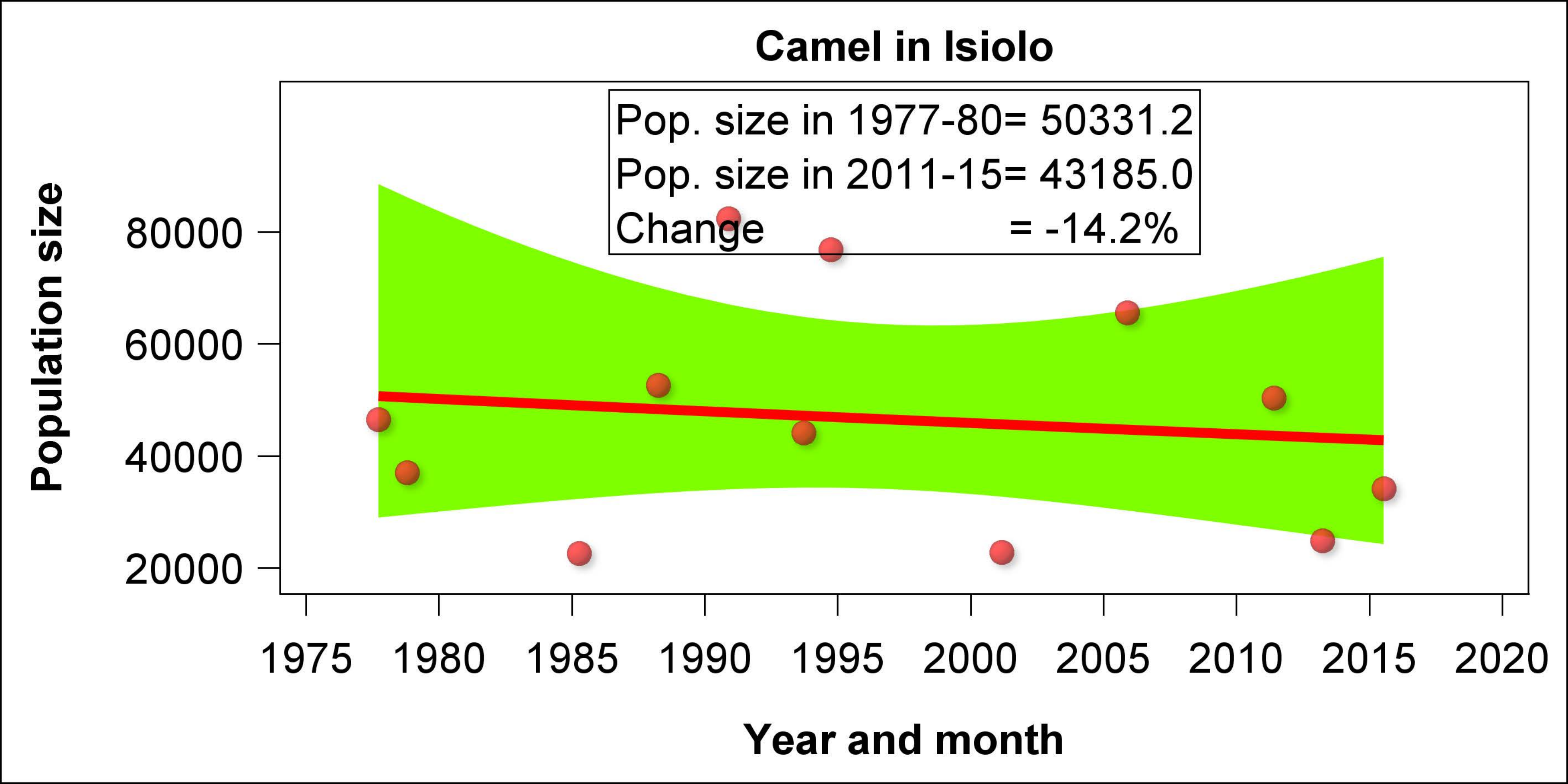
Camel in Isiolo
Pop. size in 1977-80= 50331.2
Pop. size in 2011-15= 43185.0
Change = -14.2%
Population size
80000
60000
40000
20000
1975
1980
1985
1990
1995
2000
2005
2010
2015
2020
Year and month

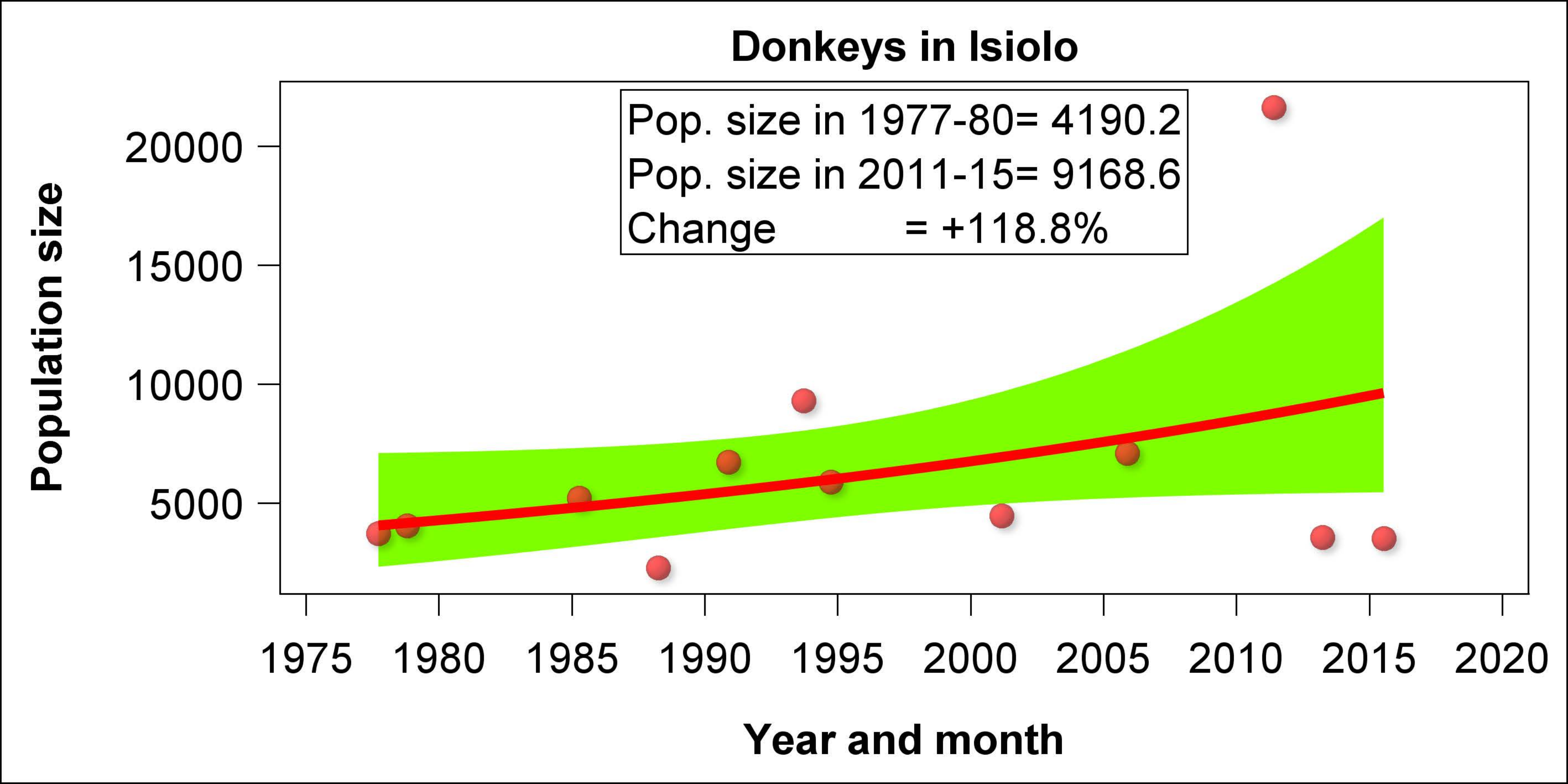
Donkeys in Isiolo
Pop. size in 1977-80= 4190.2
Pop. size in 2011-15= 9168.6
Change = +118.8%
20000
15000
10000
5000
Population size
1975
1980
1985
1990
1995
2000
2005
2010
2015
2020
Year and month

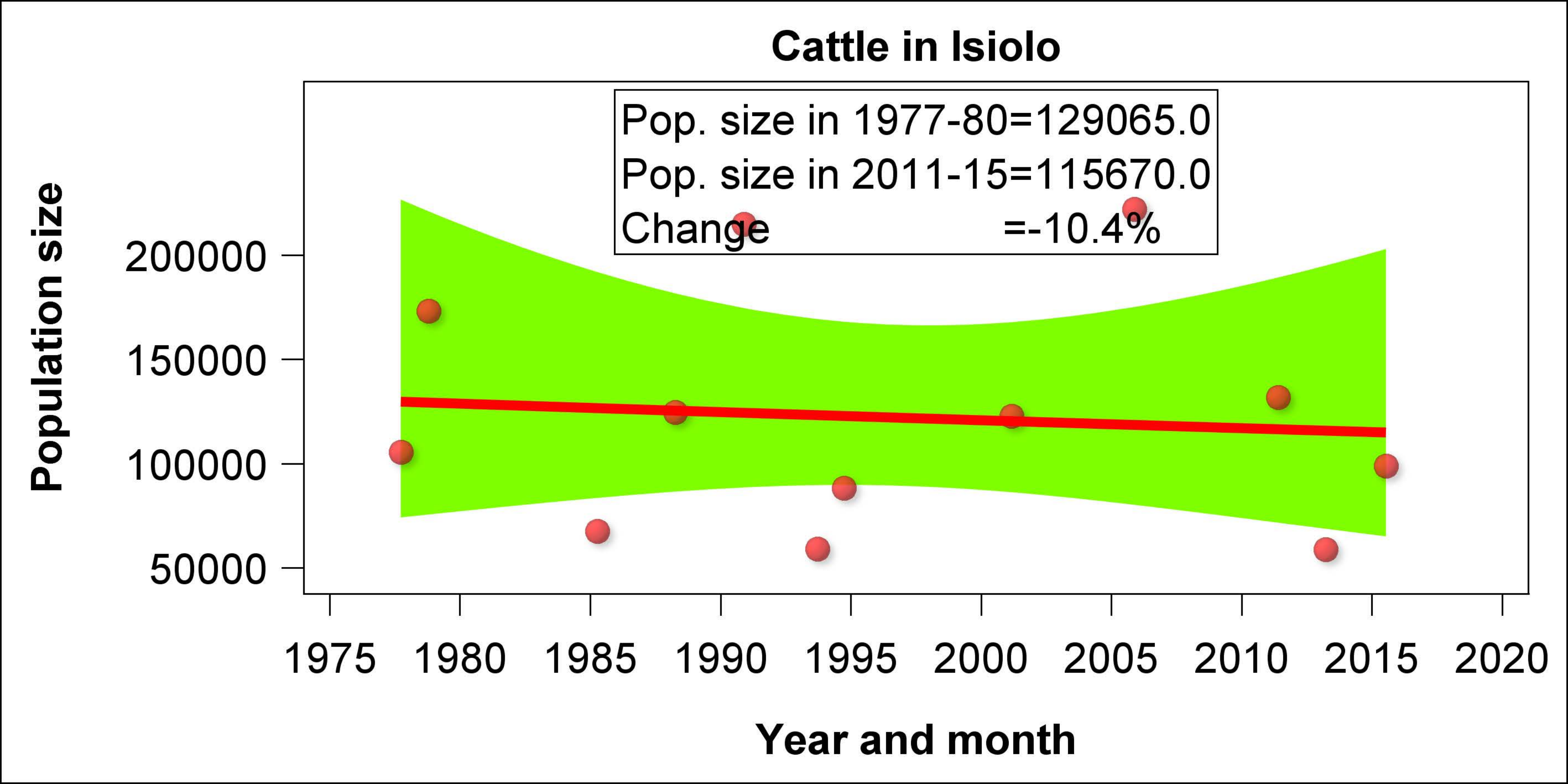
Cattle in Isiolo
Pop. size in 1977-80=129065.0
Pop. size in 2011-15=115670.0
Change =-10.4%
Population size
200000
150000
100000
50000
1975
1980
1985
1990
1995
2000
2005
2010
2015
2020
Year and month

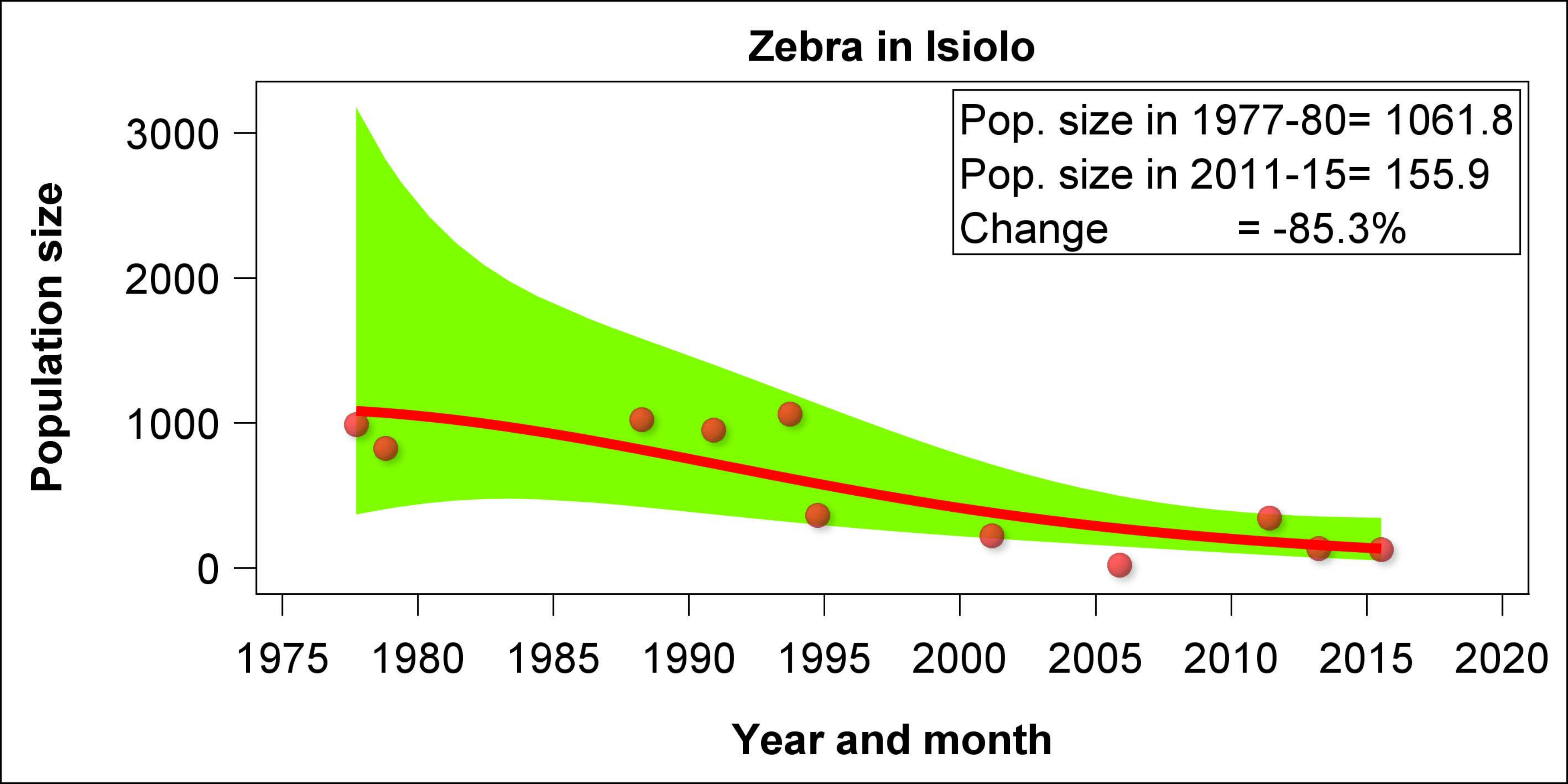

Zebra in Isiolo
Pop. size in 1977-80= 1061.8
Pop. size in 2011-15= 155.9
Change = -85.3%
Population size
3000
2000
1000
0
1975
1980
1985
1990
1995
2000
2005
2010
2015
2020
Year and month

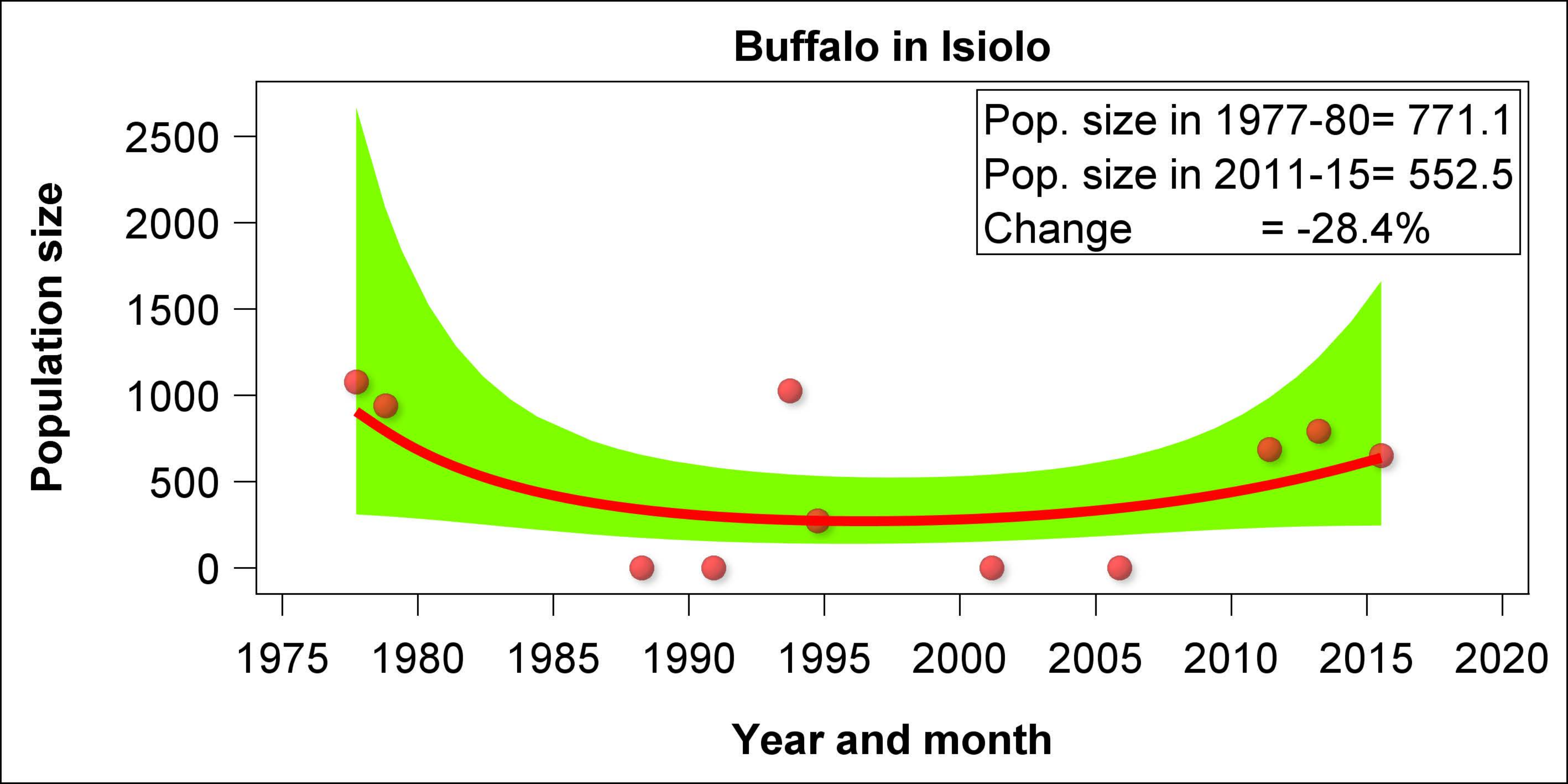
Buffalo in Isiolo
Pop. size in 1977-80= 771.1
Pop. size in 2011-15= 552.5
Change = -28.4%
Population size
2500
2000
1500
1000
500
0
1975
1980
1985
1990
1995
2000
2005
2010
2015
2020
Year and month

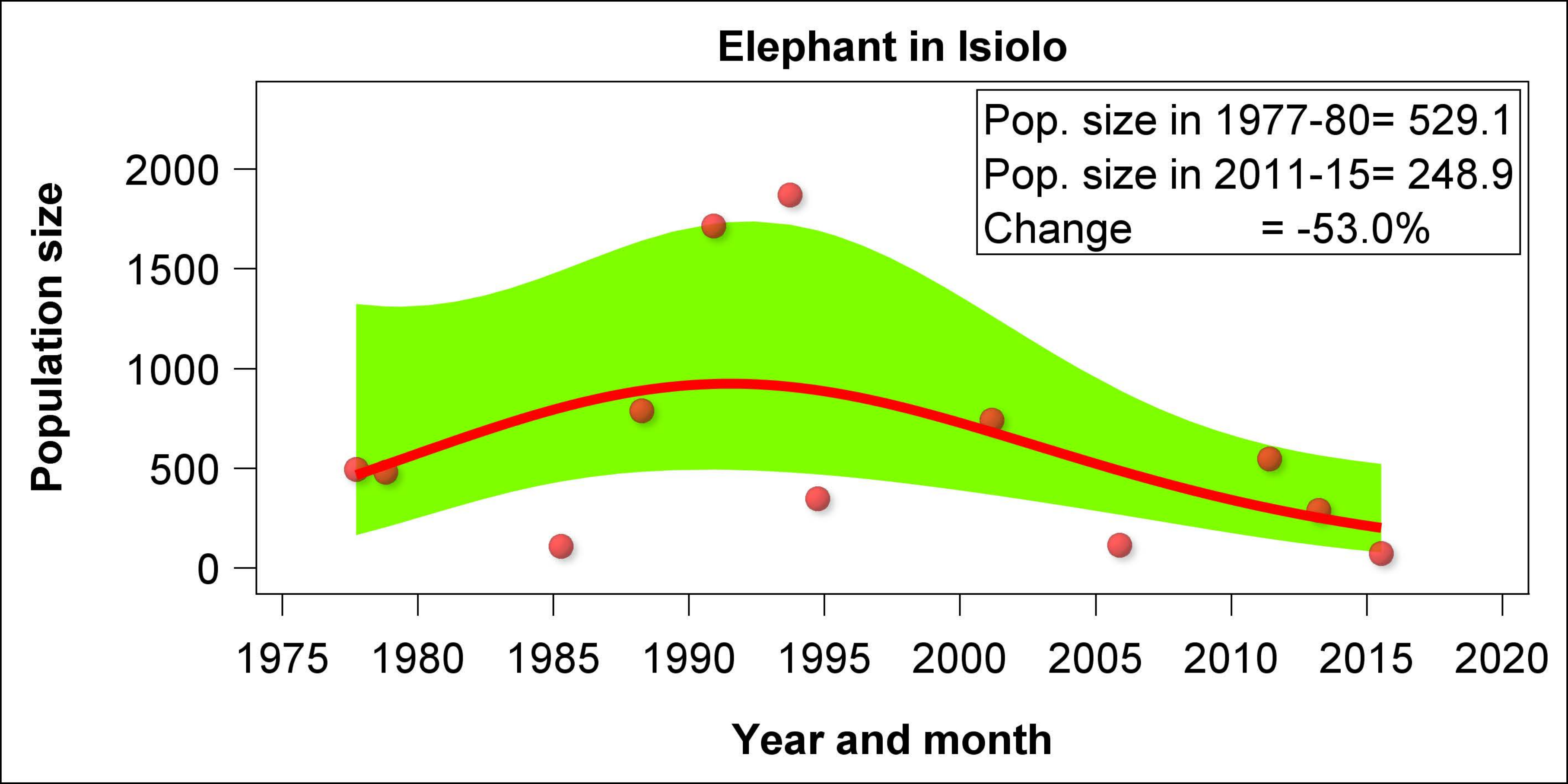

Elephant in Isiolo
Pop. size in 1977-80= 529.1
Pop. size in 2011-15= 248.9
Change = -53.0%
Population size
2000
1500
1000
500
0
1975
1980
1985
1990
1995
2000
2005
2010
2015
2020
Year and month

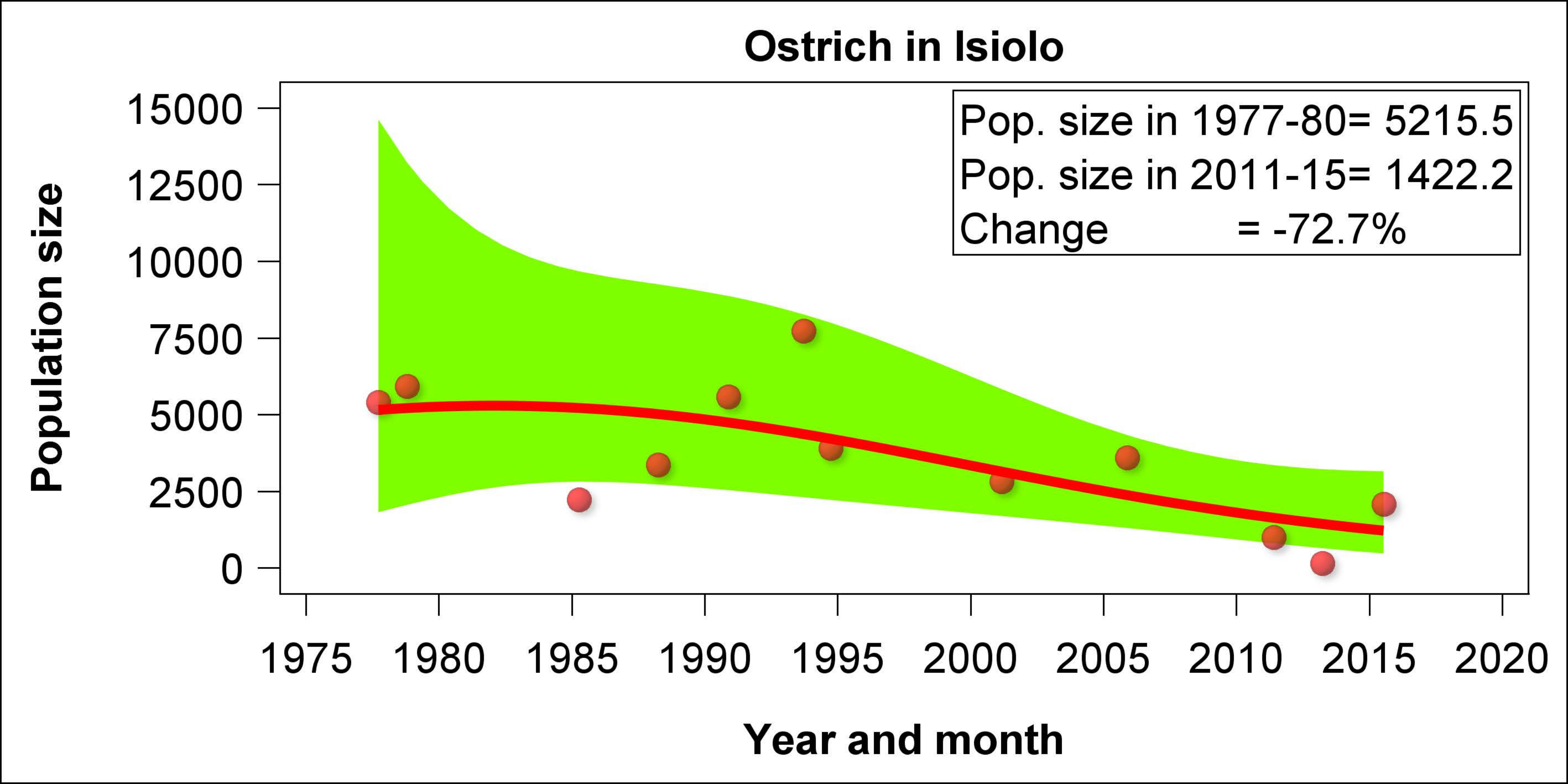
Ostrich in Isiolo
Pop. size in 1977-80= 5215.5
Pop. size in 2011-15= 1422.2
Change = -72.7%
Population size
15000
12500
10000
7500
5000
2500
0
1975
1980
1985
1990
1995
2000
2005
2010
2015
2020
Year and month

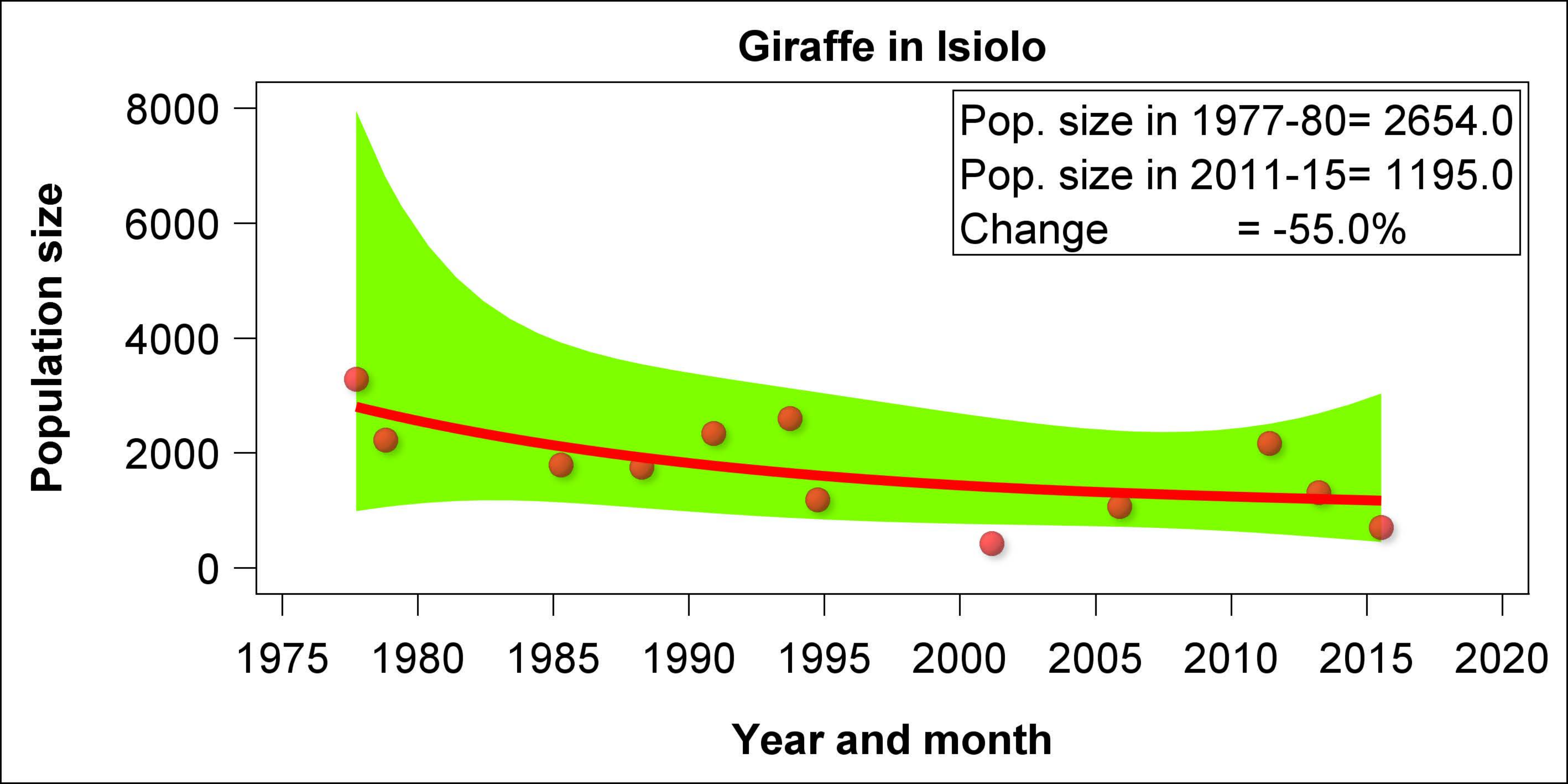
Giraffe in Isiolo
Pop. size in 1977-80= 2654.0
Pop. size in 2011-15= 1195.0
Change = -55.0%
Population size
8000
6000
4000
2000
0
1975
1980
1985
1990
1995
2000
2005
2010
2015
2020
Year and month

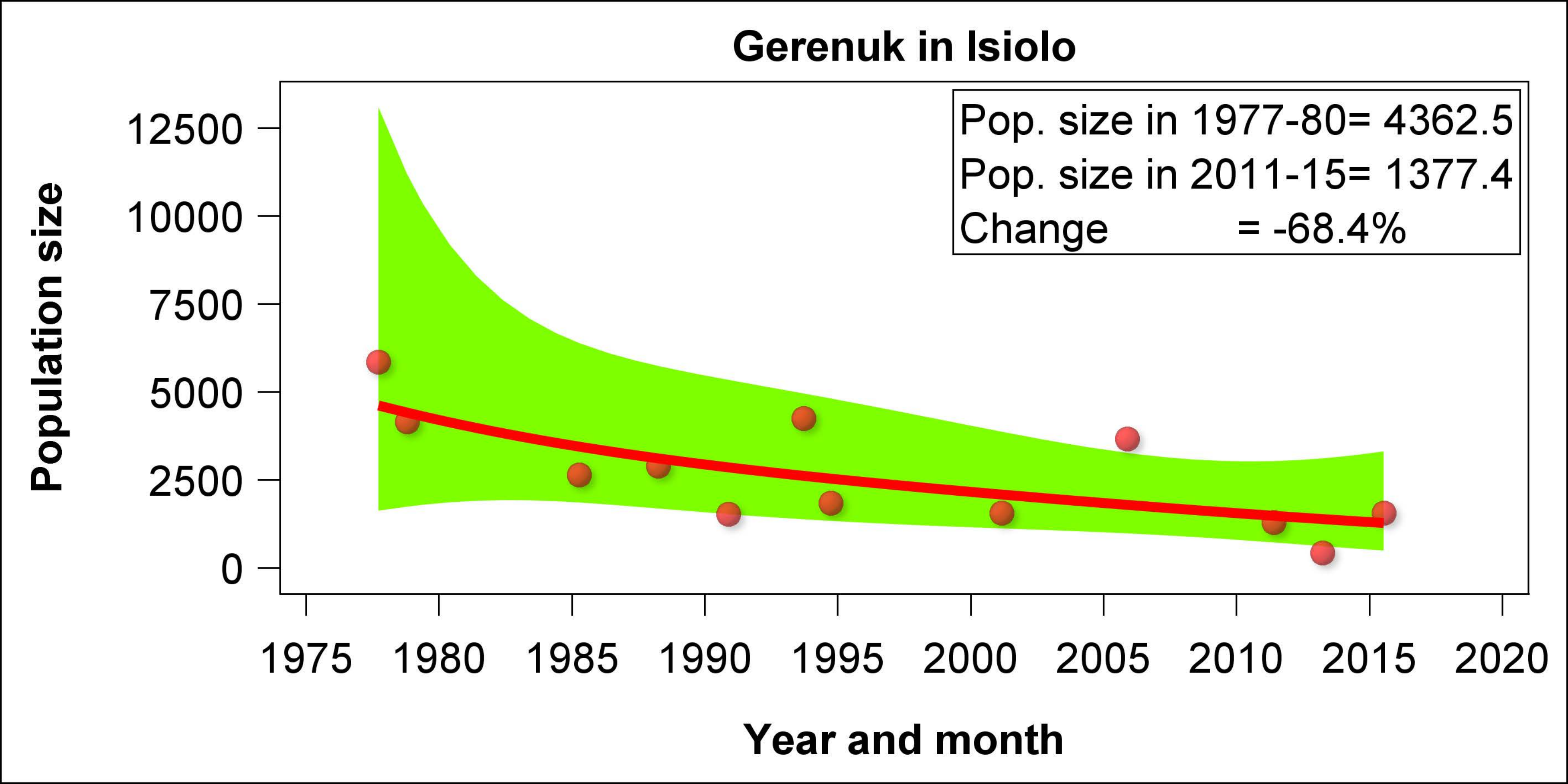

Gerenuk in Isiolo
Pop. size in 1977-80= 4362.5
Pop. size in 2011-15= 1377.4
Change = -68.4%
Population size
12500
10000
7500
5000
2500
0
1975
1980
1985
1990
1995
2000
2005
2010
2015
2020
Year and month

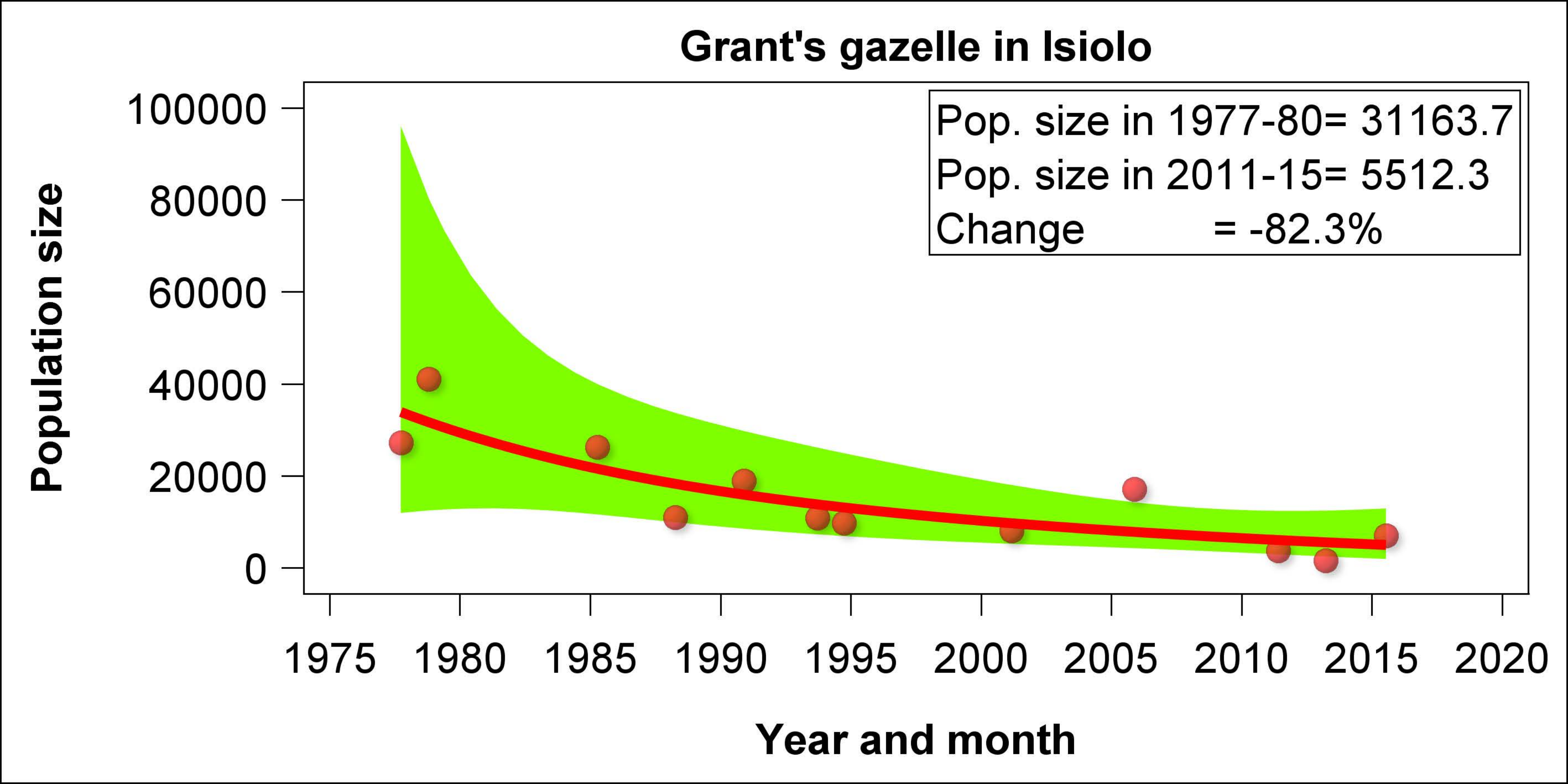
Grant's gazelle in Isiolo
Pop. size in 1977-80= 31163.7
Pop. size in 2011-15= 5512.3
Change = -82.3%
Population size
100000
80000
60000
40000
20000
0
1975
1980
1985
1990
1995
2000
2005
2010
2015
2020
Year and month

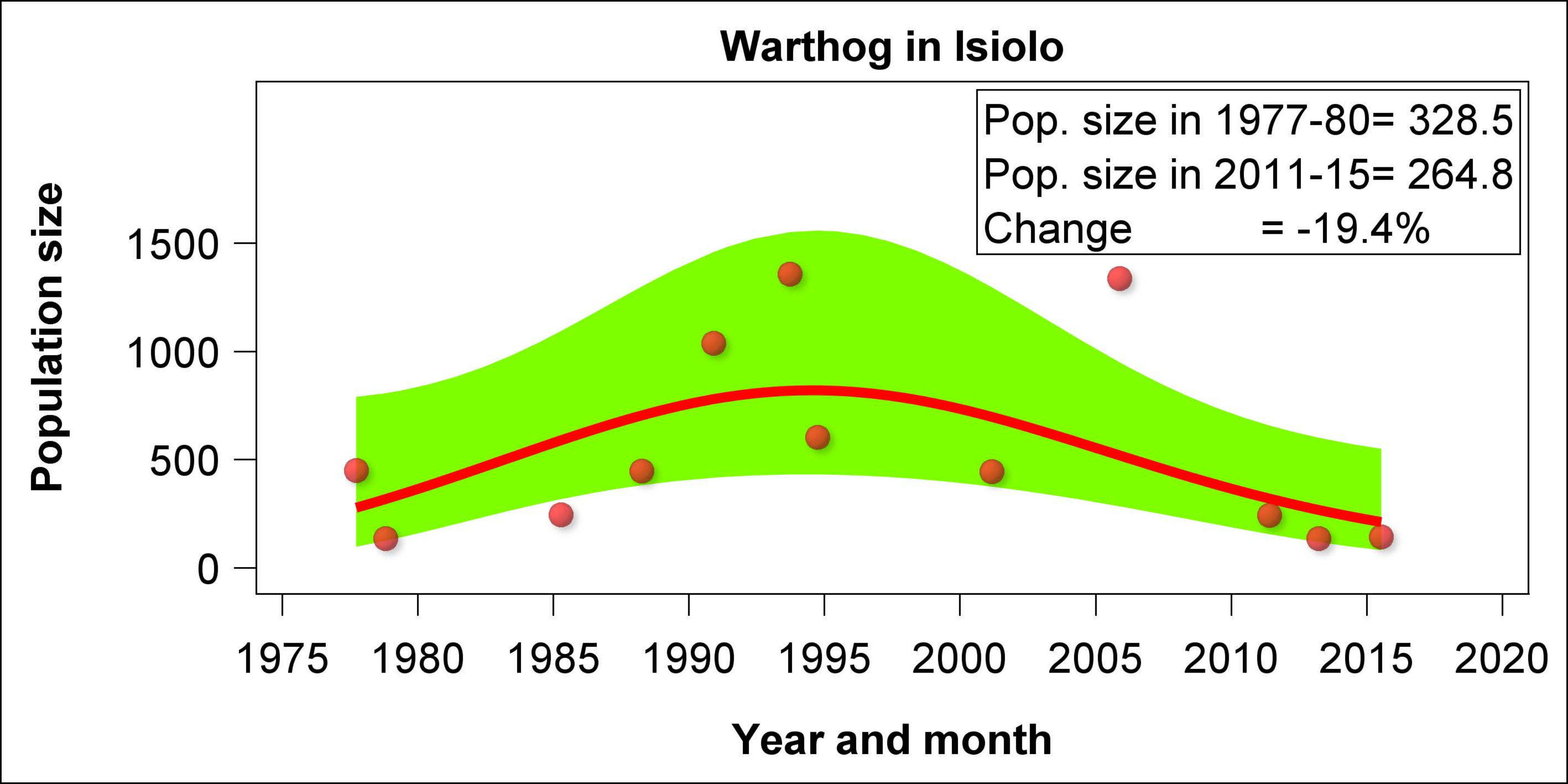
Warthog in Isiolo
Pop. size in 1977-80= 328.5
Pop. size in 2011-15= 264.8
Change = -19.4%
Population size
0
500
1000
1500
1975
1980
1985
1990
1995
2000
2005
2010
2015
2020
Year and month

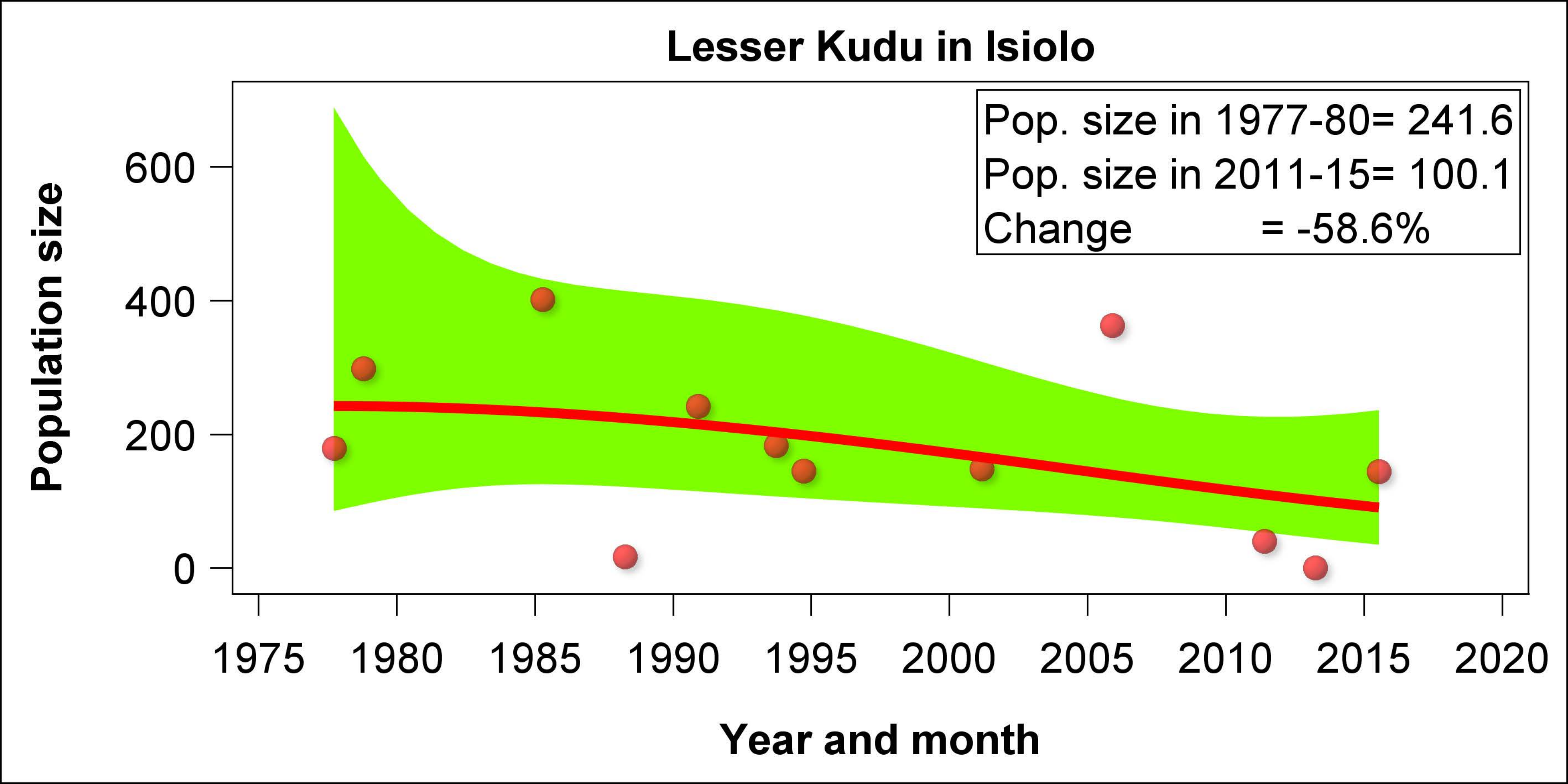

Lesser Kudu in Isiolo
Pop. size in 1977-80= 241.6
Pop. size in 2011-15= 100.1
Change = -58.6%
Population size
600
400
200
0
1975
1980
1985
1990
1995
2000
2005
2010
2015
2020
Year and month

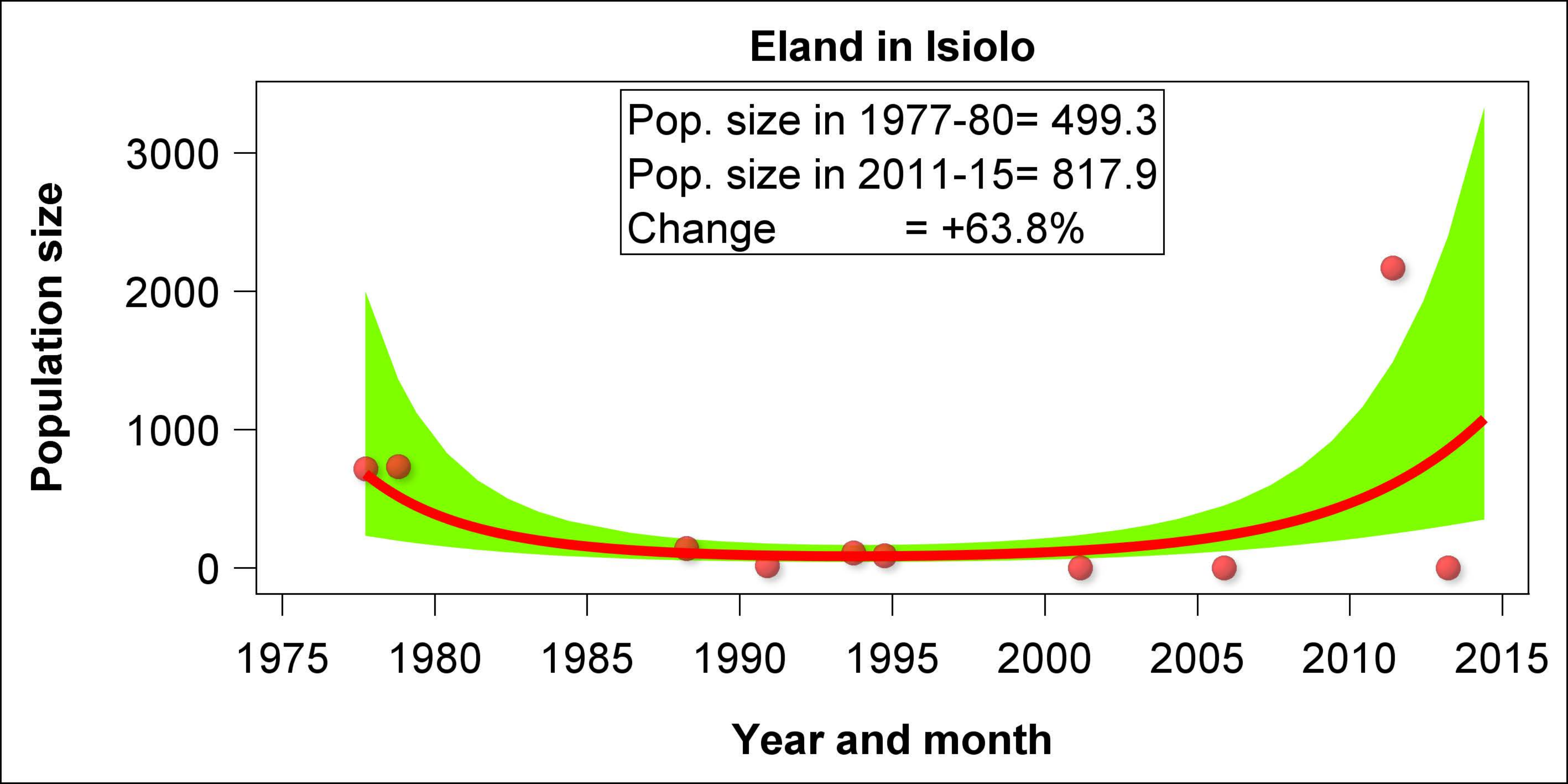
Eland in Isiolo
Pop. size in 1977-80= 499.3
Pop. size in 2011-15= 817.9
Change = +63.8%
Population size
3000
2000
1000
0
1975
1980
1985
1990
1995
2000
2005
2010
2015
Year and month

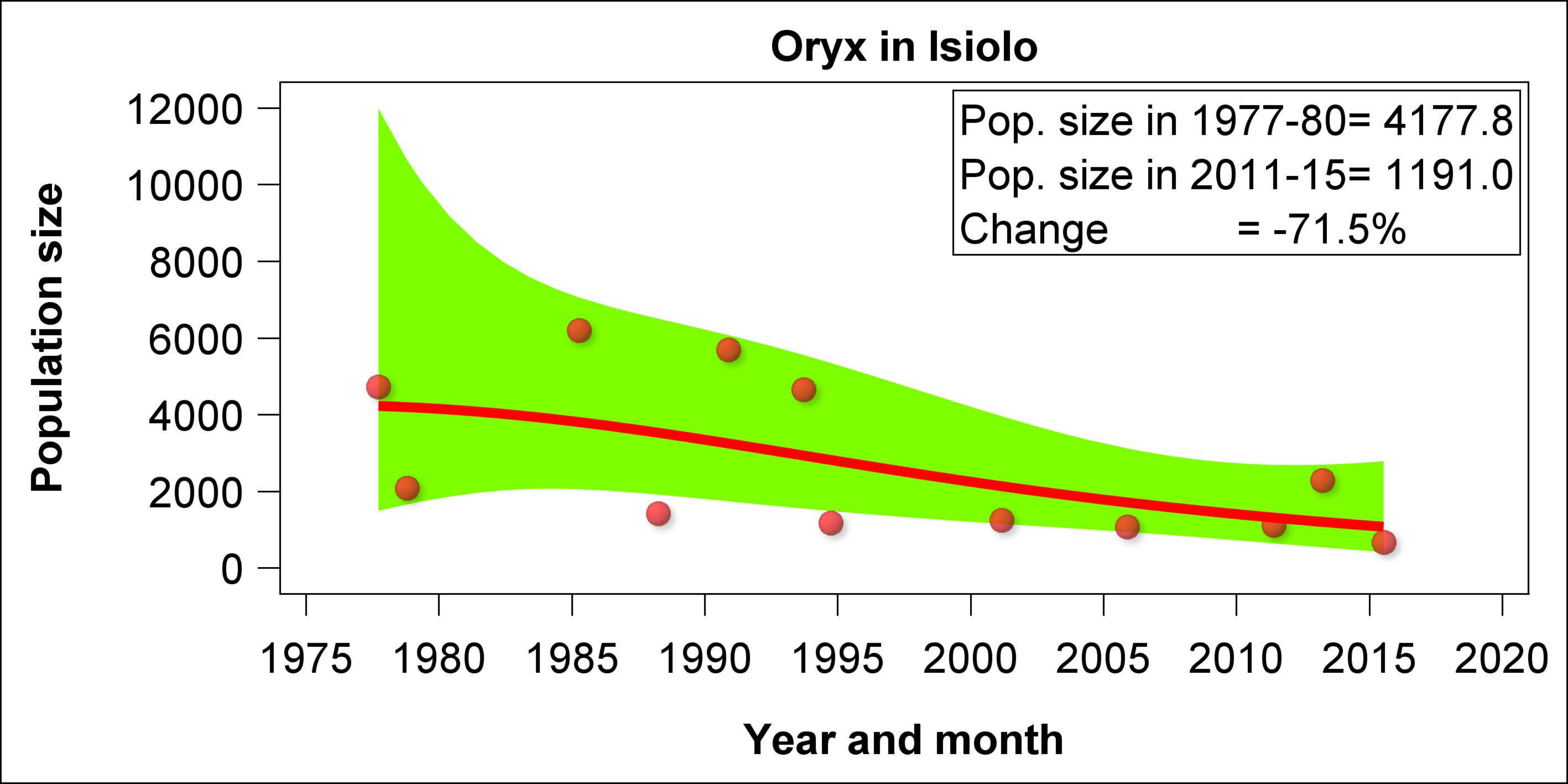

Oryx in Isiolo
Pop. size in 1977-80= 4177.8
Pop. size in 2011-15= 1191.0
Change = -71.5%
Population size
12000
10000
8000
6000
4000
2000
0
1975
1980
1985
1990
1995
2000
2005
2010
2015
2020
Year and month

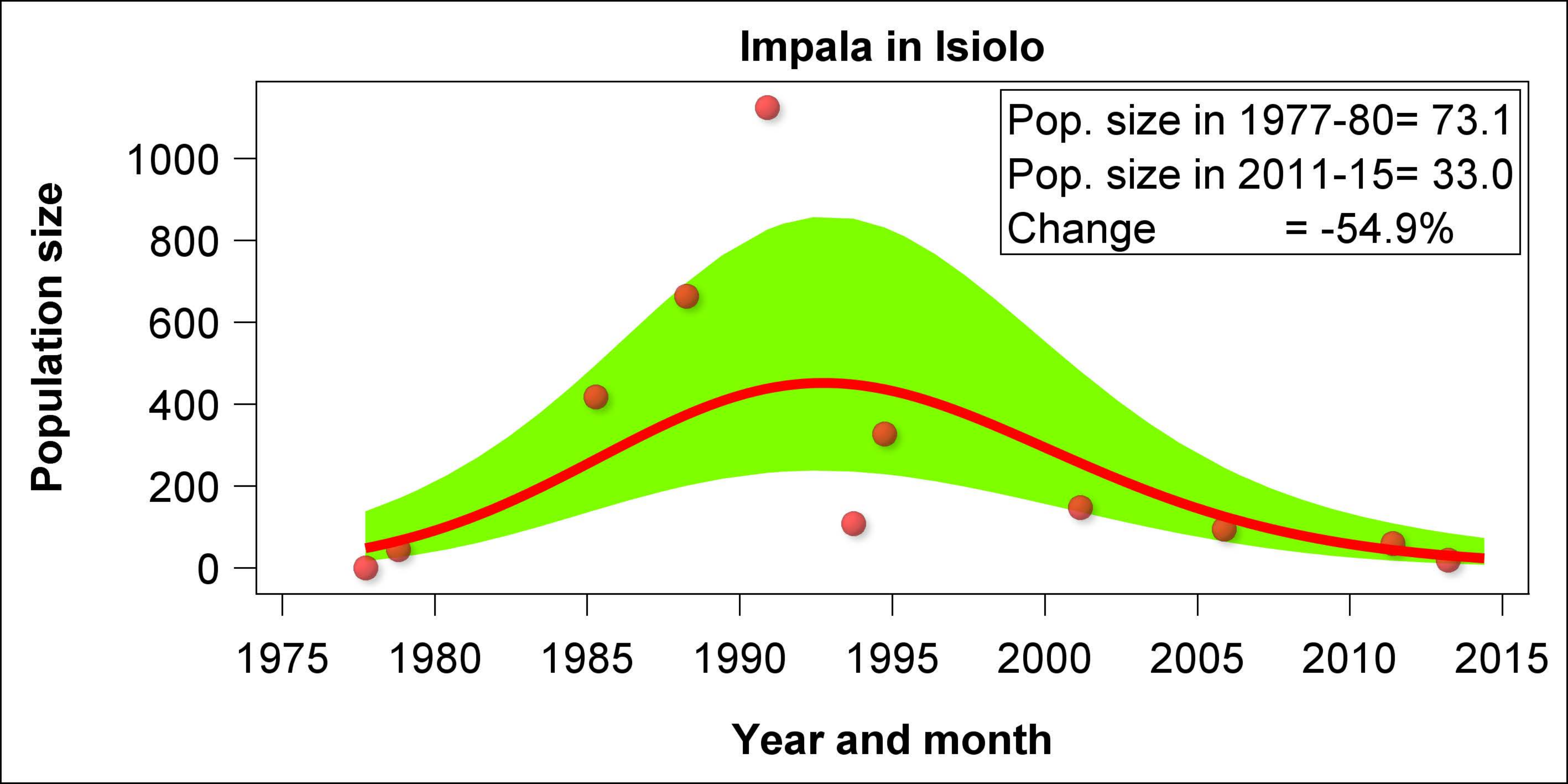
Impala in Isiolo
Pop. size in 1977-80= 73.1
Pop. size in 2011-15= 33.0
Change = -54.9%
Population size
0
200
400
600
800
1000
1975
1980
1985
1990
1995
2000
2005
2010
2015
Year and month

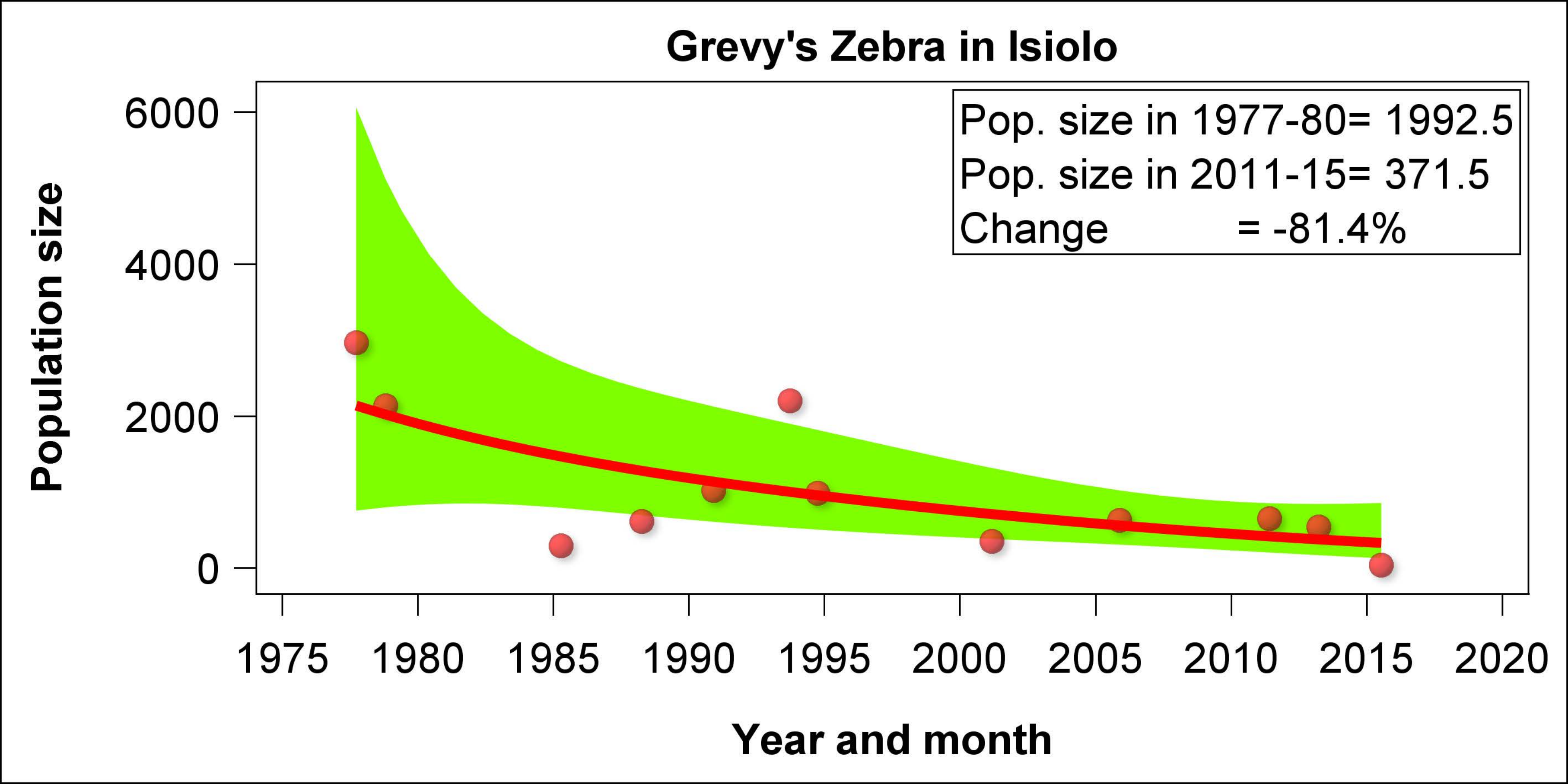

Grevy's Zebra in Isiolo
Pop. size in 1977-80= 1992.5
Pop. size in 2011-15= 371.5
Change = -81.4%
Population size
6000
4000
2000
0
1975
1980
1985
1990
1995
2000
2005
2010
2015
2020
Year and month

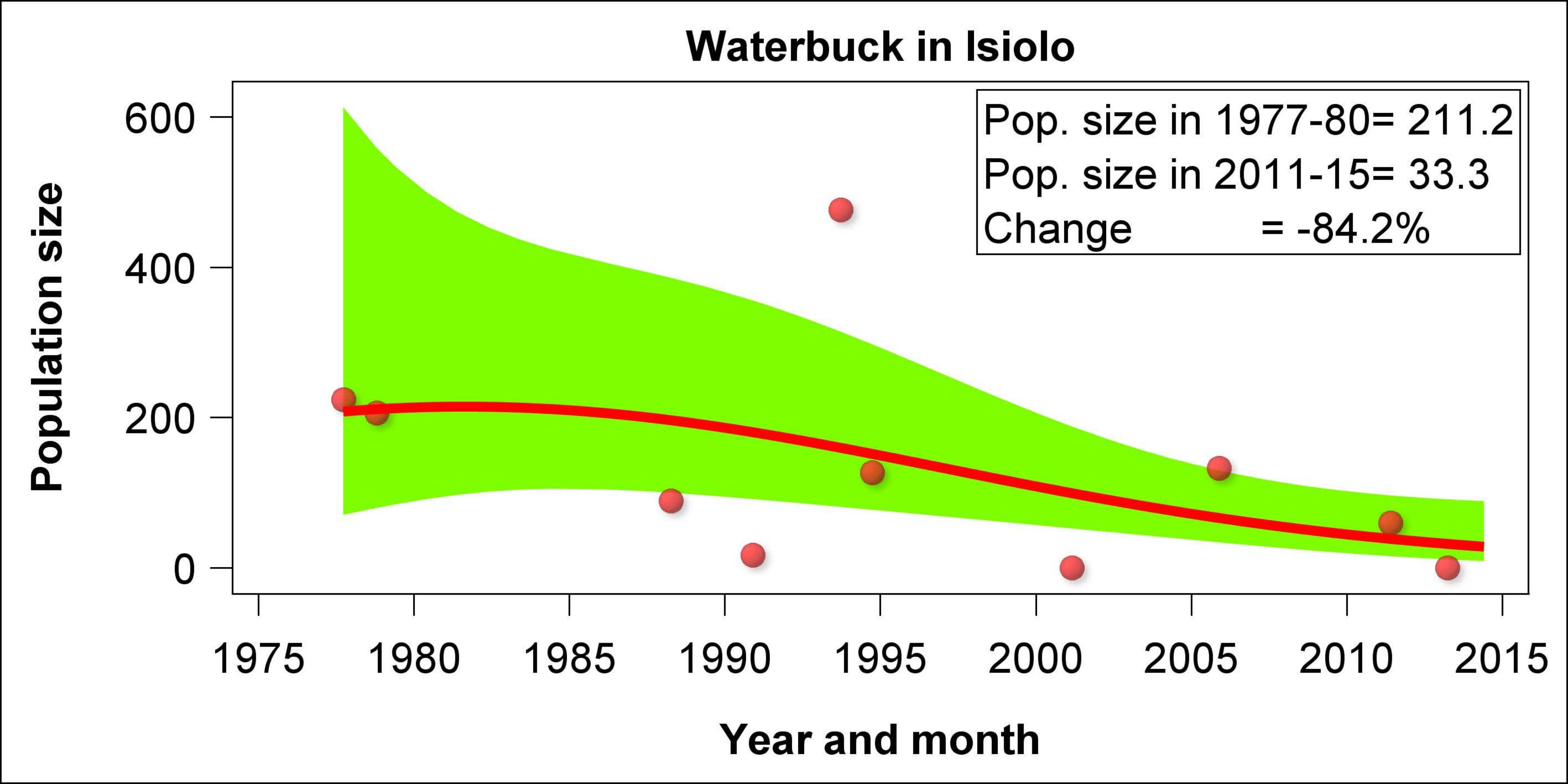
Waterbuck in Isiolo
Pop. size in 1977-80= 211.2
Pop. size in 2011-15= 33.3
Change = -84.2%
Population size
600
400
200
0
1975
1980
1985
1990
1995
2000
2005
2010
2015
Year and month
