# Supplementary material for: Extreme Wildlife Declines and Concurrent Increase in Livestock Numbers in Kenya: What Are the Causes?
Source: PLoS One. 2016 Sep 27;11(9):e0163249. doi: 10.1371/journal.pone.0163249 (PMC5039022; doi:10.1371/journal.pone.0163249)
Supplement: S15 Fig — The solid red line is the fitted trend curve and the shaded chartreuse band is the pointwise 95% confidence band. The estimated average population size in 1977–1980 and 2011–2013 and the percentage change in population size between the two periods are provided in the inset. (PDF) [file pone.0163249.s025.pdf]

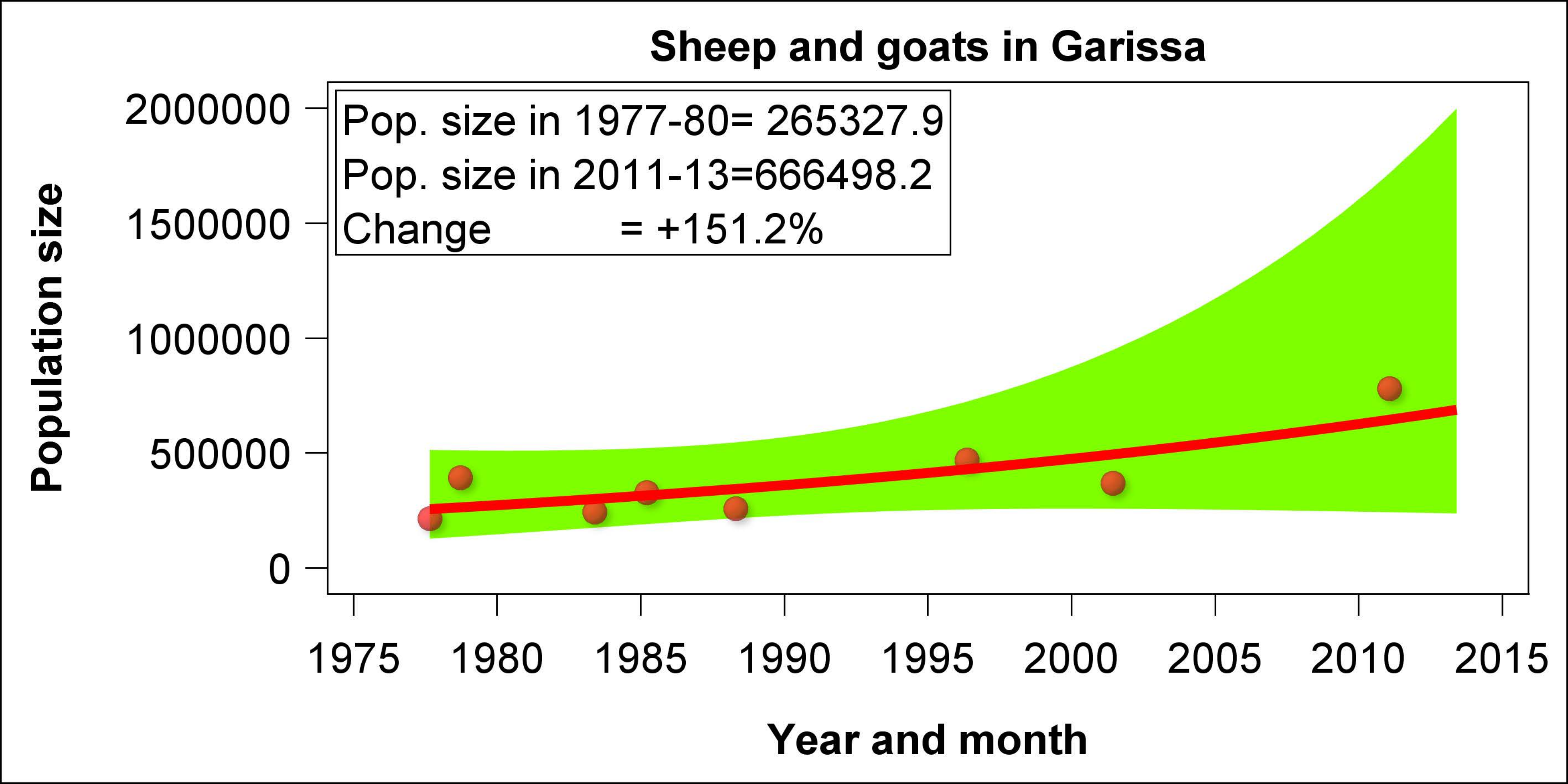

Sheep and goats in Garissa
Pop. size in 1977-80= 265327.9
Pop. size in 2011-13=666498.2
Change = +151.2%
Population size
2000000
1500000
1000000
500000
0
1975
1980
1985
1990
1995
2000
2005
2010
2015
Year and month

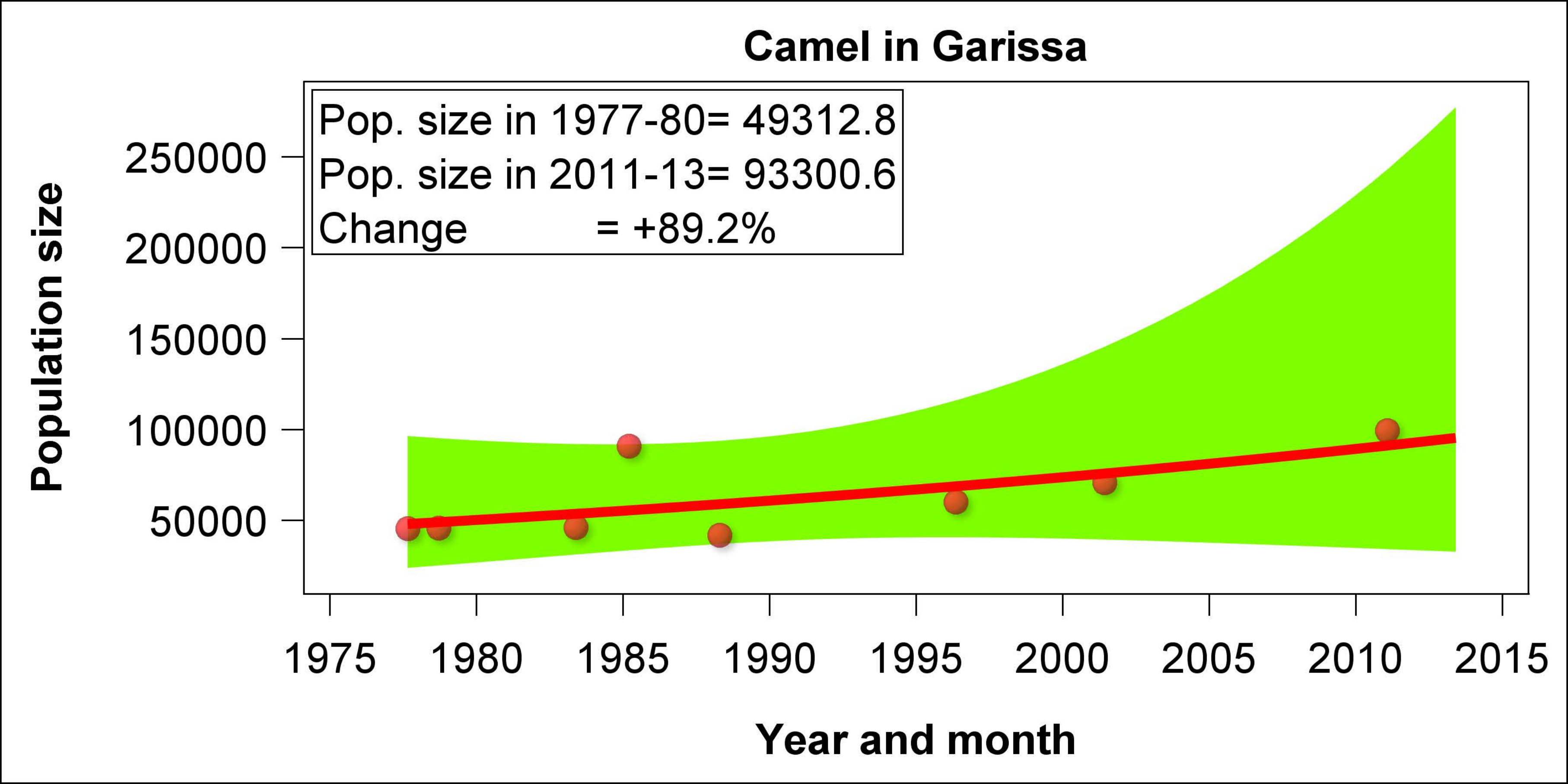
Camel in Garissa
Pop. size in 1977-80= 49312.8
Pop. size in 2011-13= 93300.6
Change = +89.2%
Population size
250000
200000
150000
100000
50000
1975
1980
1985
1990
1995
2000
2005
2010
2015
Year and month

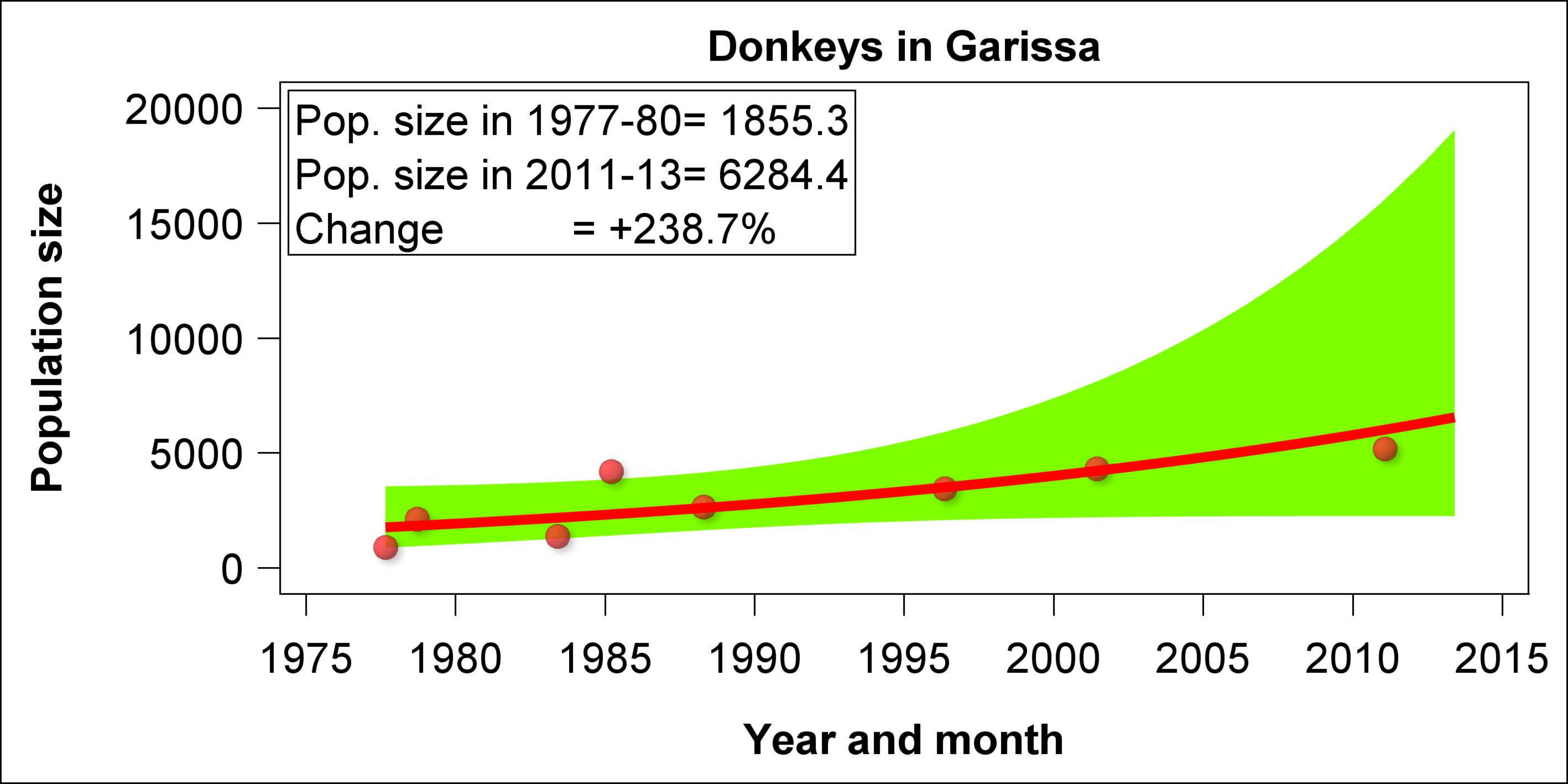
Donkeys in Garissa
Pop. size in 1977-80= 1855.3
Pop. size in 2011-13= 6284.4
Change = +238.7%
20000
15000
10000
5000
0
Population size
1975
1980
1985
1990
1995
2000
2005
2010
2015
Year and month

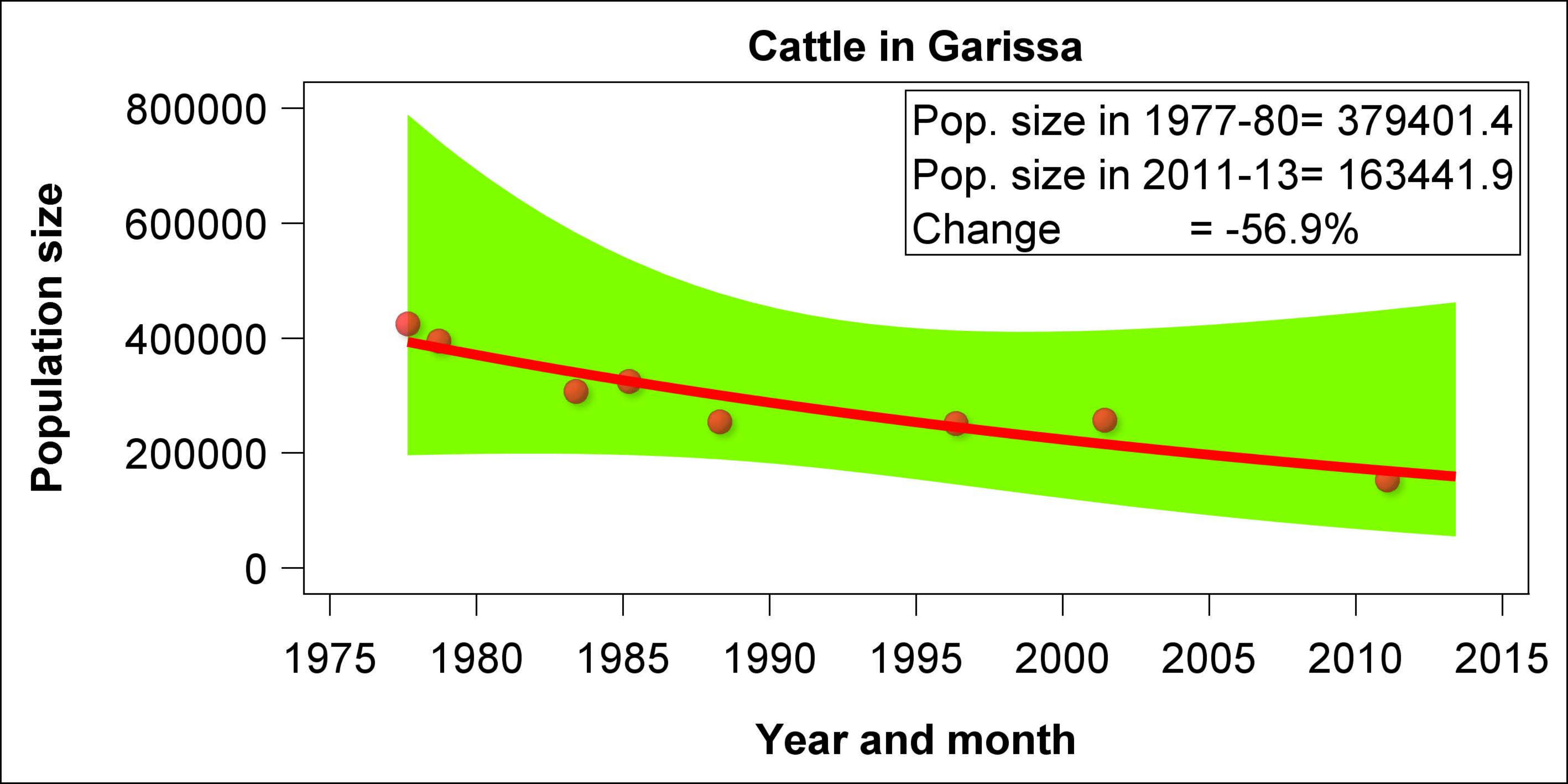

Cattle in Garissa
Pop. size in 1977-80= 379401.4
Pop. size in 2011-13= 163441.9
Change = -56.9%
Population size
800000
600000
400000
200000
0
1975
1980
1985
1990
1995
2000
2005
2010
2015
Year and month

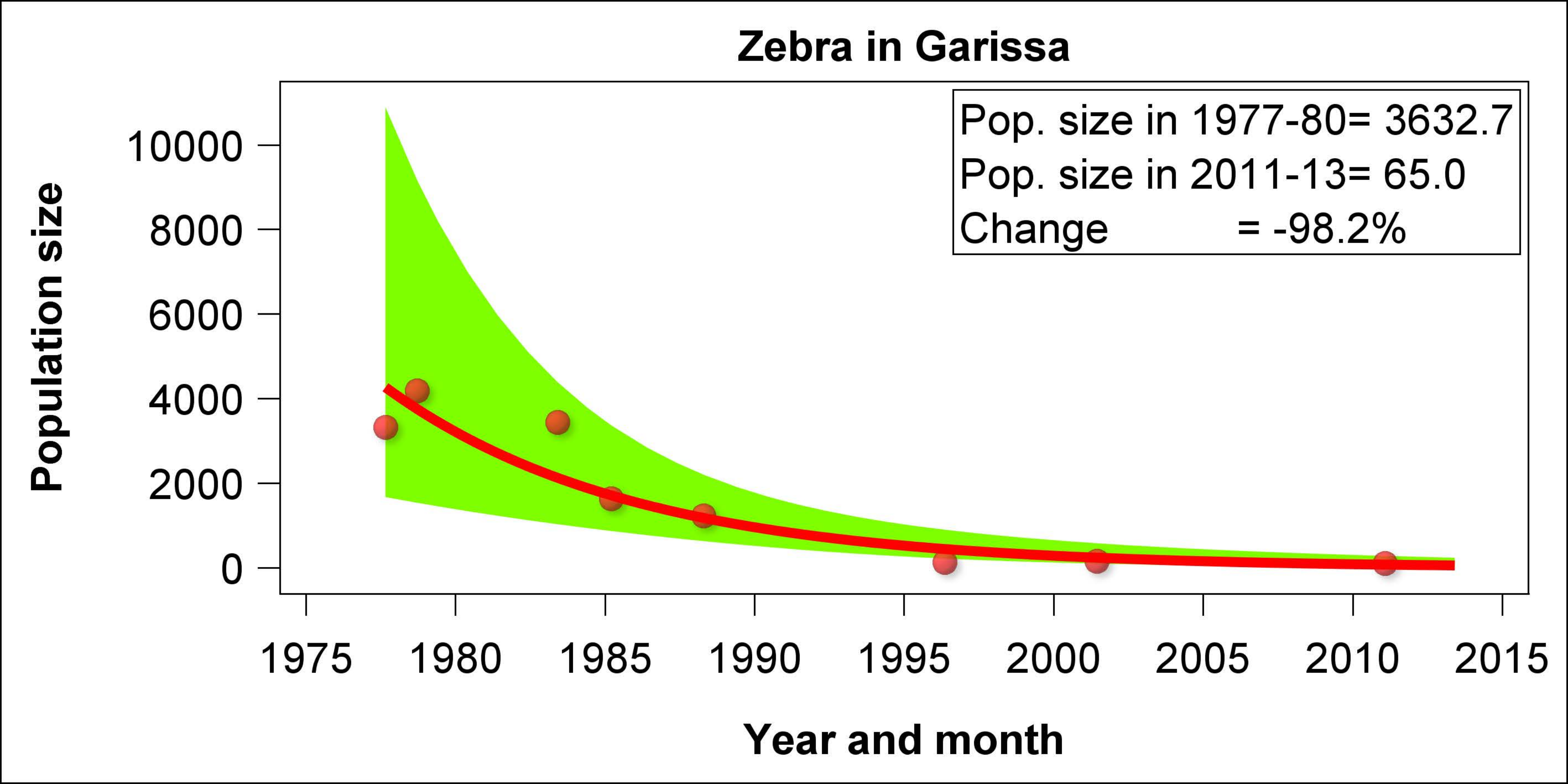

Zebra in Garissa
Pop. size in 1977-80= 3632.7
Pop. size in 2011-13= 65.0
Change = -98.2%
Population size
10000
8000
6000
4000
2000
0
1975
1980
1985
1990
1995
2000
2005
2010
2015
Year and month

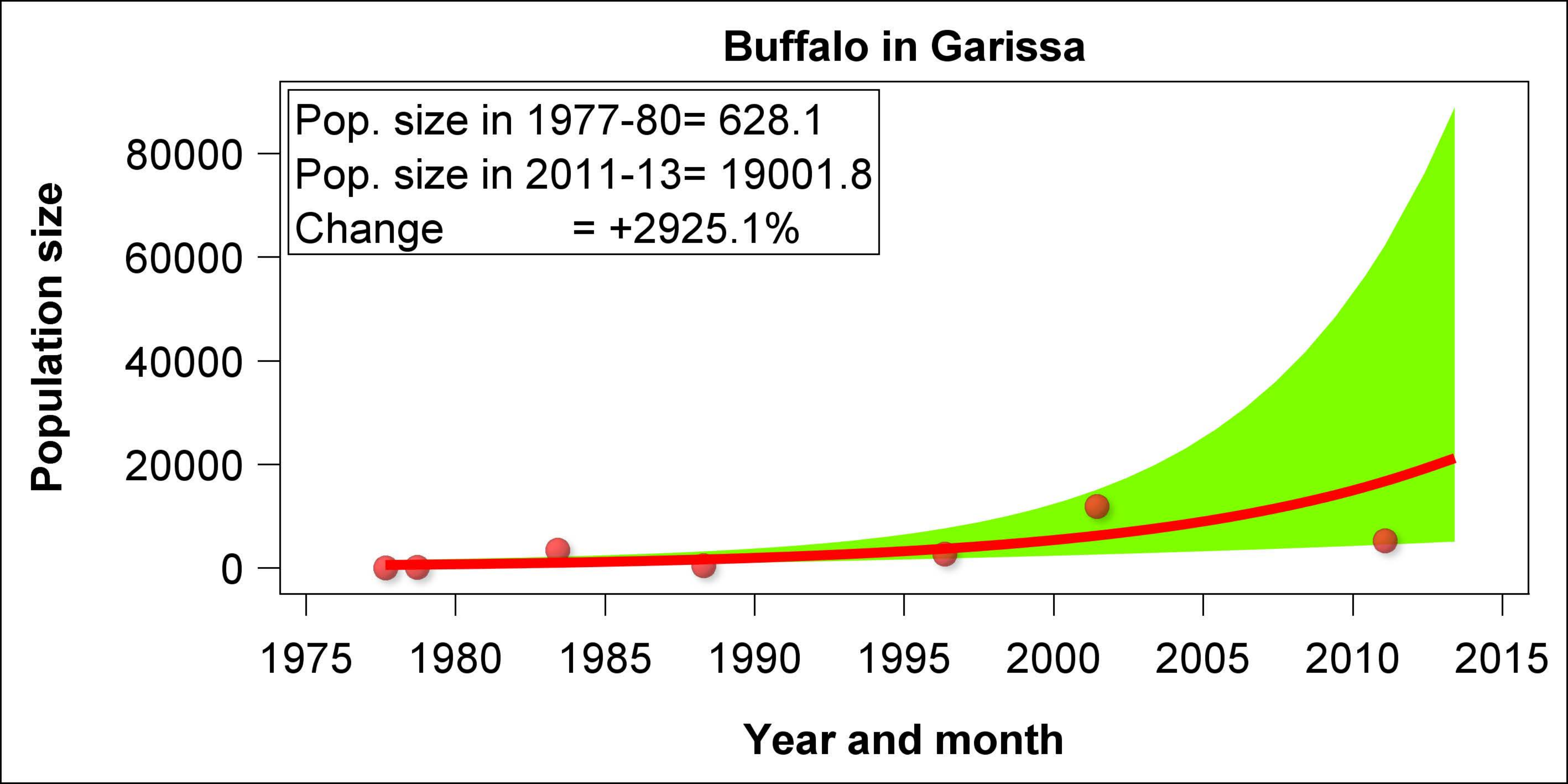

Buffalo in Garissa
Pop. size in 1977-80= 628.1
Pop. size in 2011-13= 19001.8
Change = +2925.1%
80000
60000
40000
20000
0
Population size
1975
1980
1985
1990
1995
2000
2005
2010
2015
Year and month

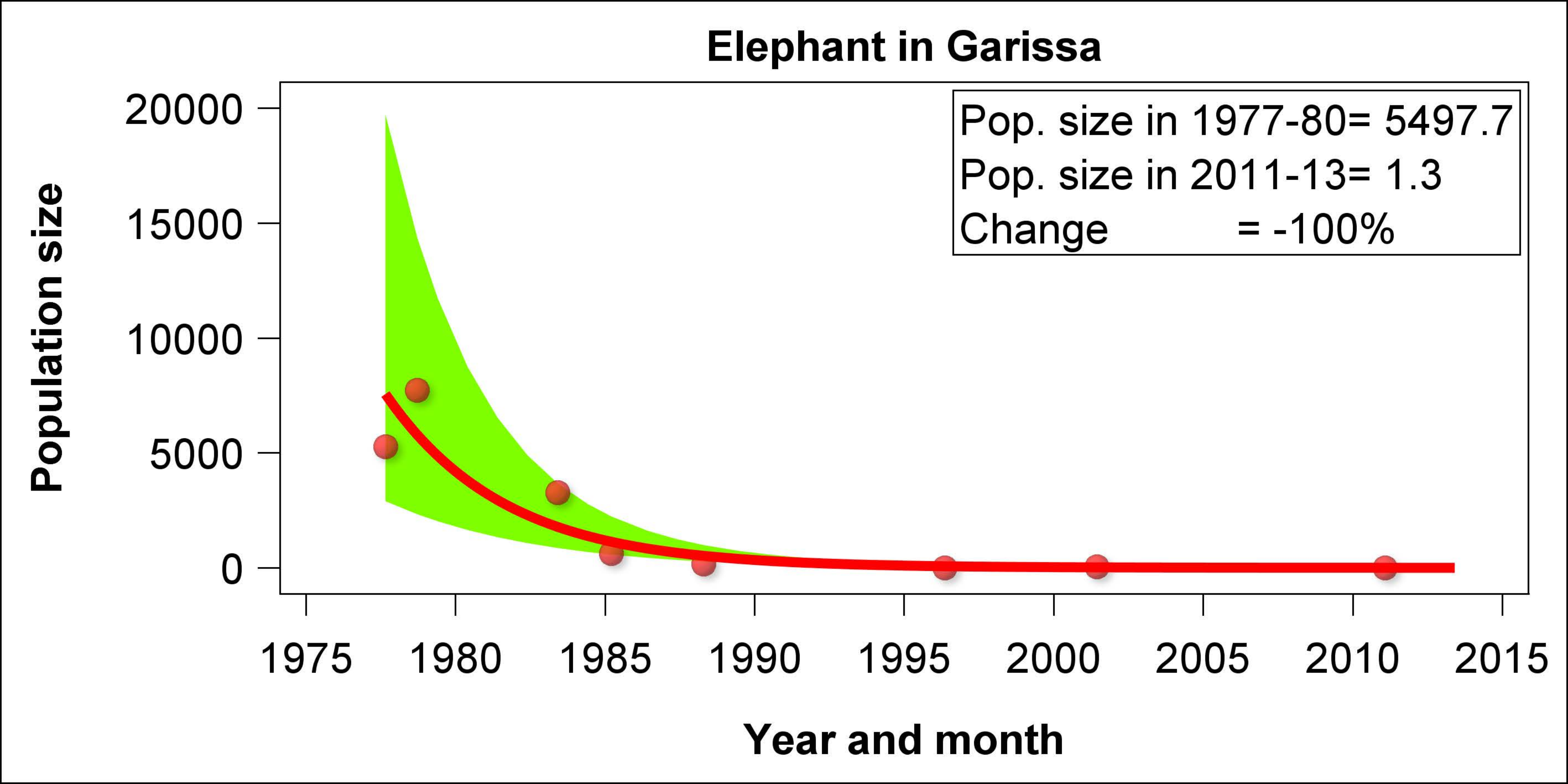
Elephant in Garissa
Pop. size in 1977-80= 5497.7
Pop. size in 2011-13= 1.3
Change = -100%
Population size
20000
15000
10000
5000
0
1975
1980
1985
1990
1995
2000
2005
2010
2015
Year and month

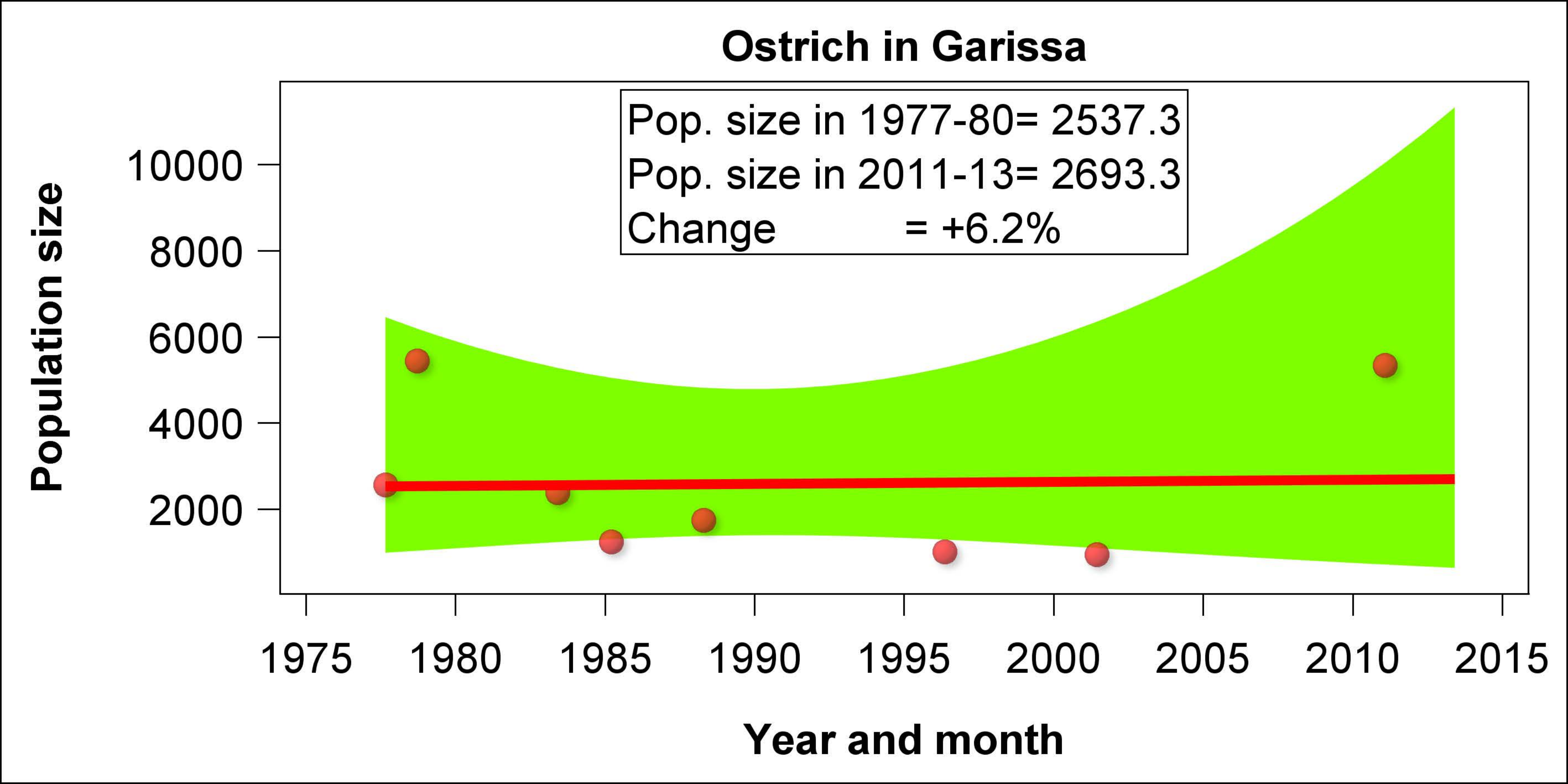
Ostrich in Garissa
Pop. size in 1977-80= 2537.3
Pop. size in 2011-13= 2693.3
Change = +6.2%
Population size
10000
8000
6000
4000
2000
1975
1980
1985
1990
1995
2000
2005
2010
2015
Year and month

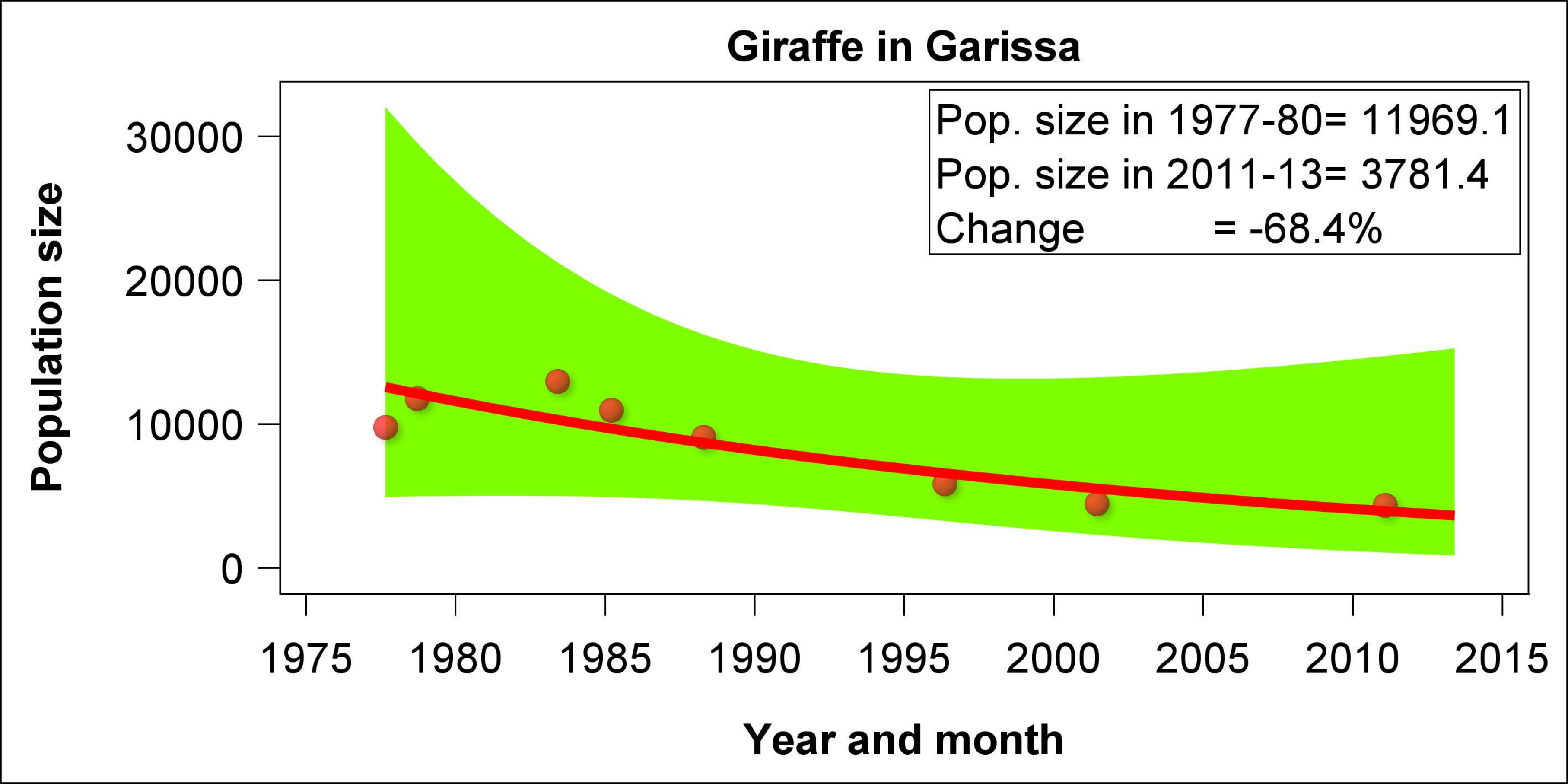
Giraffe in Garissa
Pop. size in 1977-80= 11969.1
Pop. size in 2011-13= 3781.4
Change = -68.4%
Population size
30000
20000
10000
0
1975
1980
1985
1990
1995
2000
2005
2010
2015
Year and month

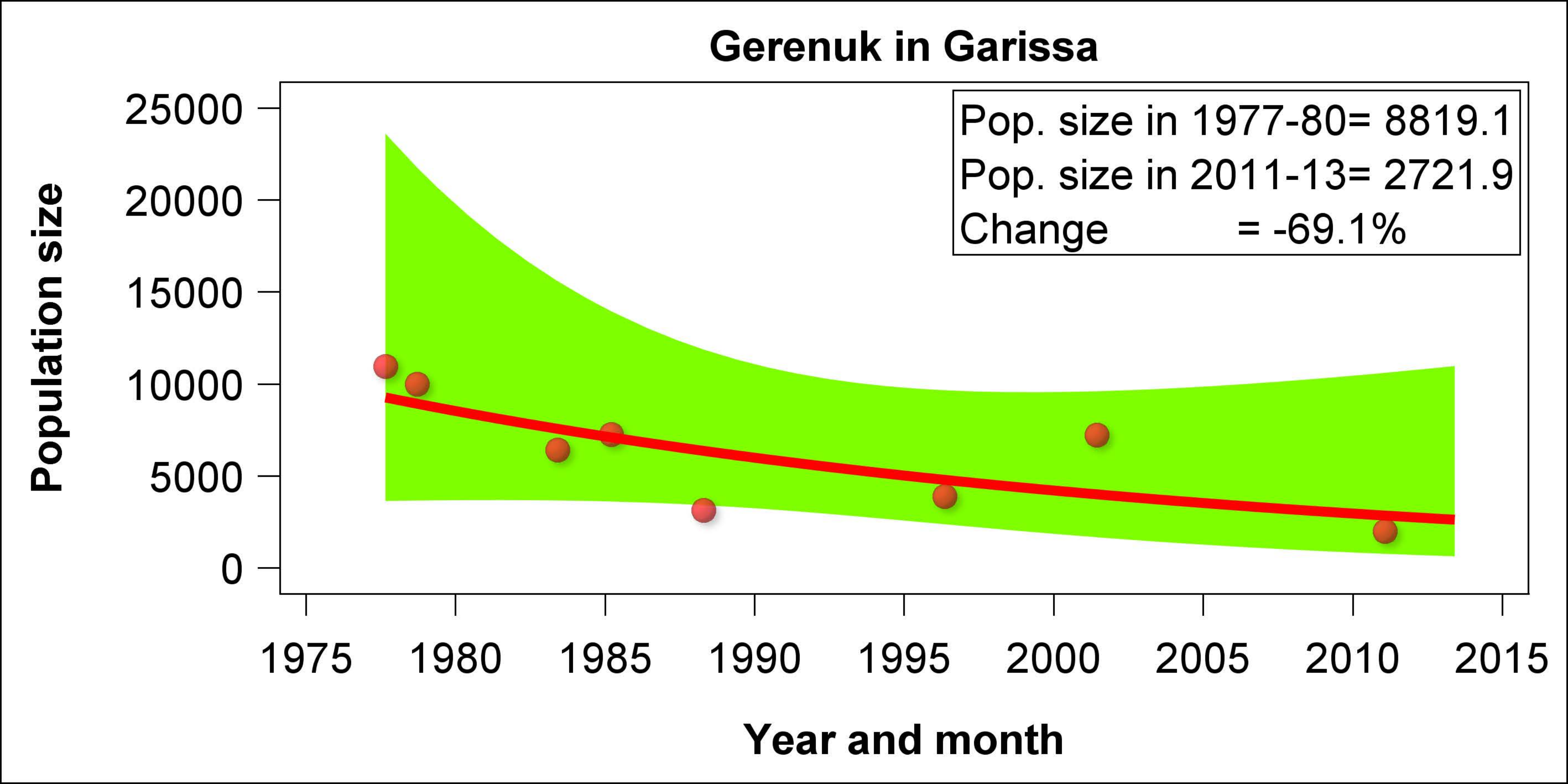

Gerenuk in Garissa
Pop. size in 1977-80= 8819.1
Pop. size in 2011-13= 2721.9
Change = -69.1%
Population size
25000
20000
15000
10000
5000
0
1975
1980
1985
1990
1995
2000
2005
2010
2015
Year and month

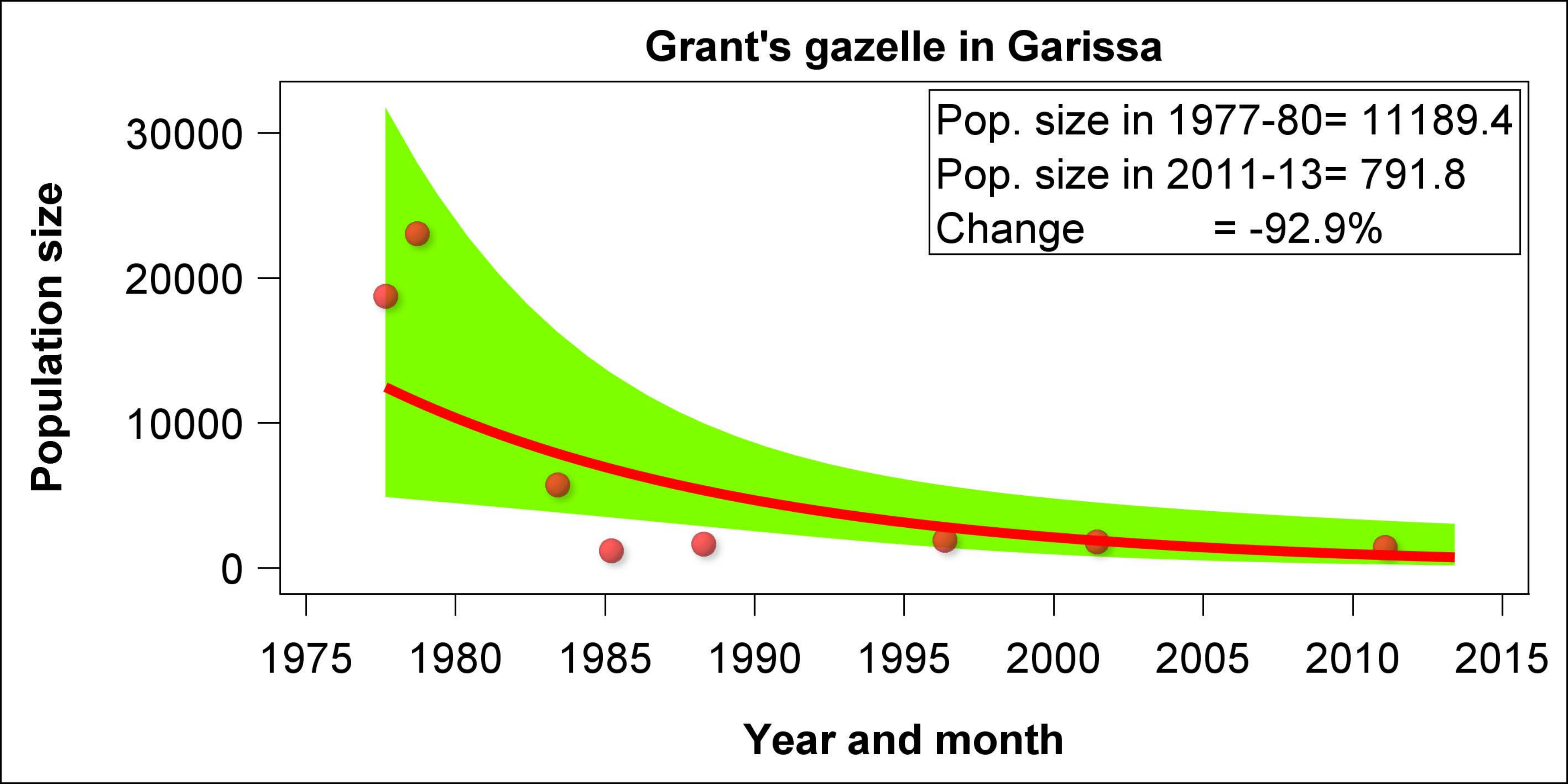
Grant's gazelle in Garissa
Pop. size in 1977-80= 11189.4
Pop. size in 2011-13= 791.8
Change = -92.9%
Population size
30000
20000
10000
0
1975
1980
1985
1990
1995
2000
2005
2010
2015
Year and month

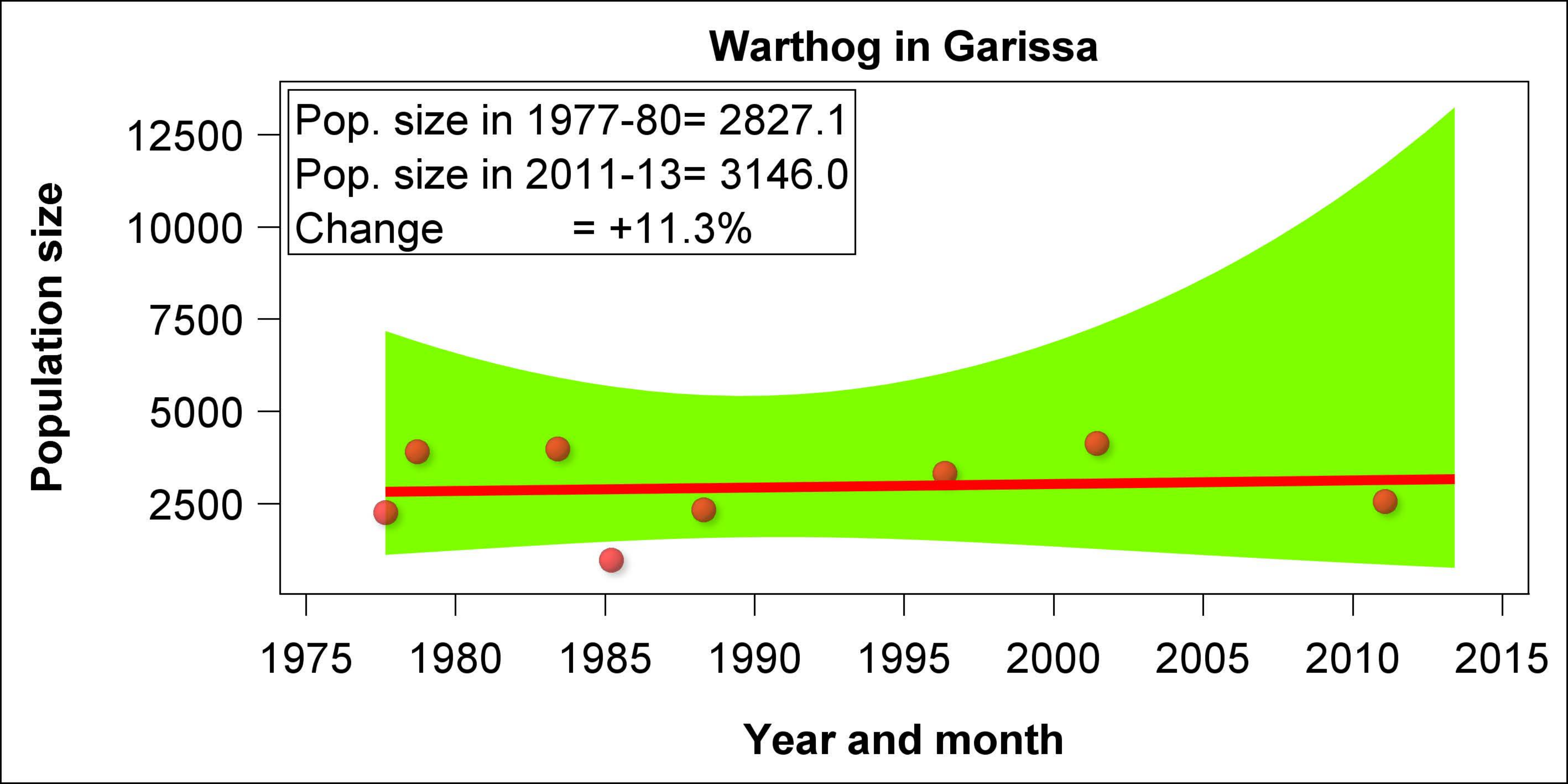
Warthog in Garissa
Pop. size in 1977-80= 2827.1
Pop. size in 2011-13= 3146.0
Change = +11.3%
12500
10000
7500
5000
2500
Population size
1975
1980
1985
1990
1995
2000
2005
2010
2015
Year and month

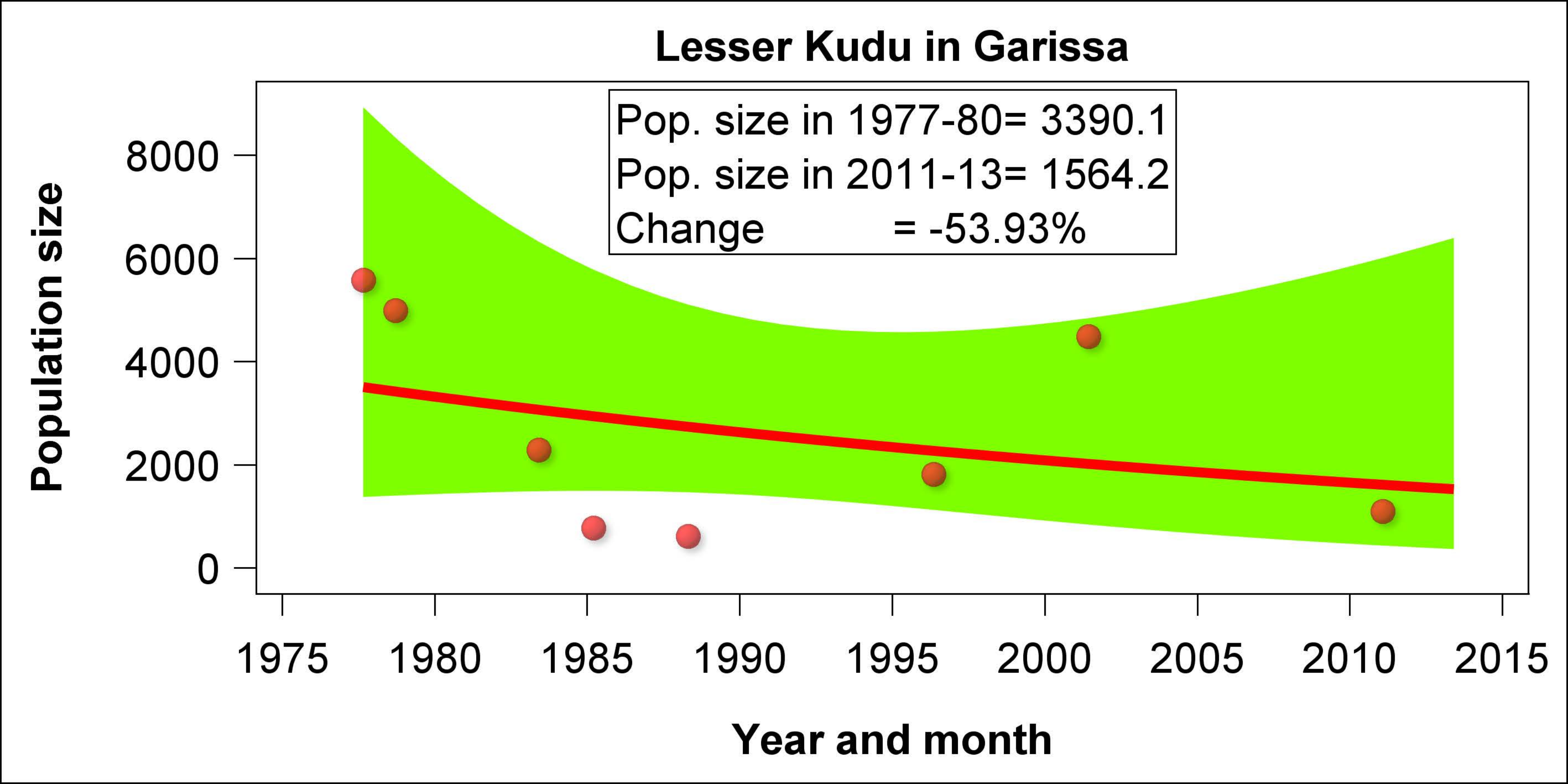
Lesser Kudu in Garissa
Pop. size in 1977-80= 3390.1
Pop. size in 2011-13= 1564.2
Change = -53.93%
Population size
8000
6000
4000
2000
0
1975
1980
1985
1990
1995
2000
2005
2010
2015
Year and month

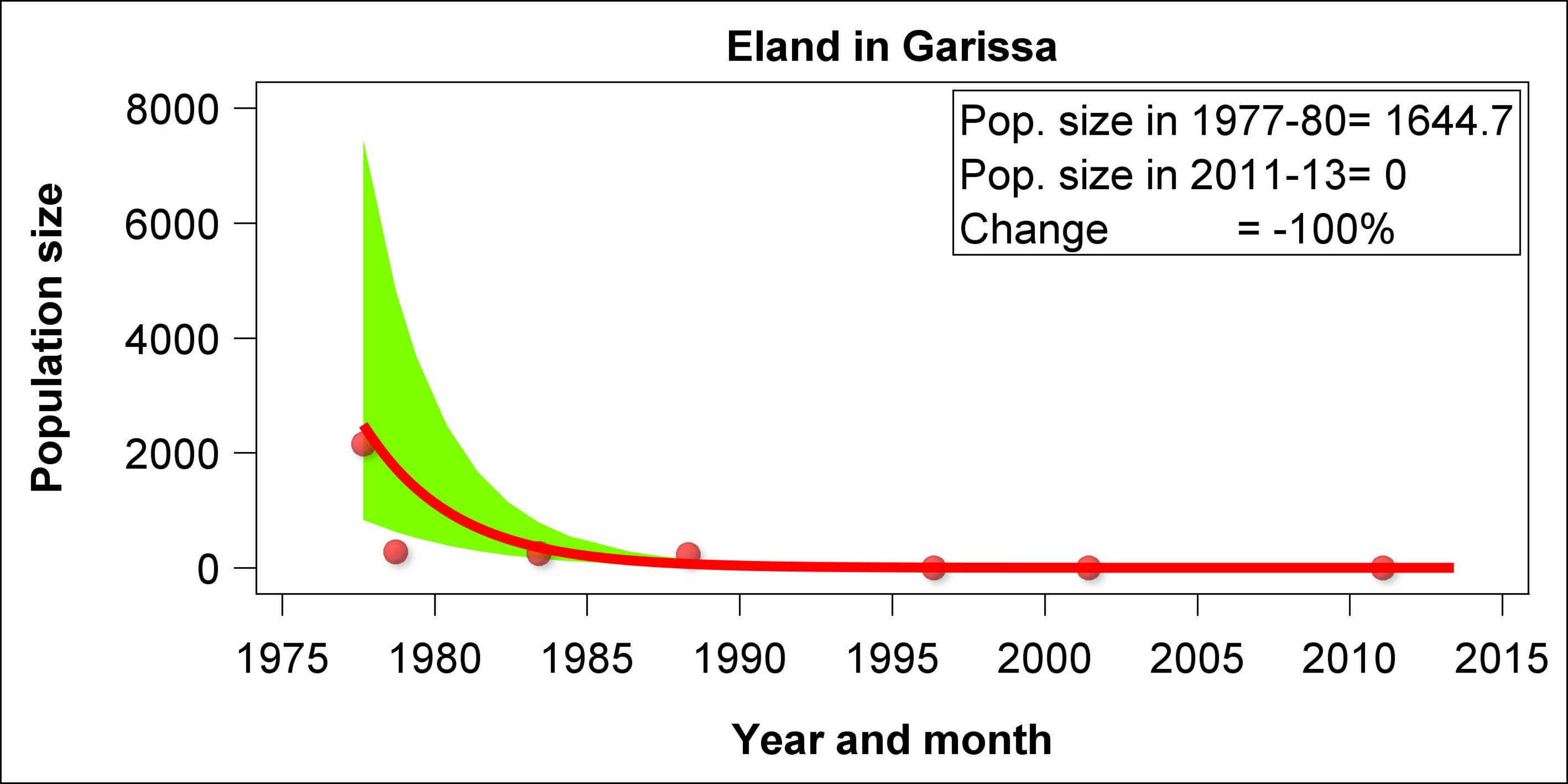
Eland in Garissa
Pop. size in 1977-80= 1644.7
Pop. size in 2011-13= 0
Change = -100%
Population size
8000
6000
4000
2000
0
1975
1980
1985
1990
1995
2000
2005
2010
2015
Year and month

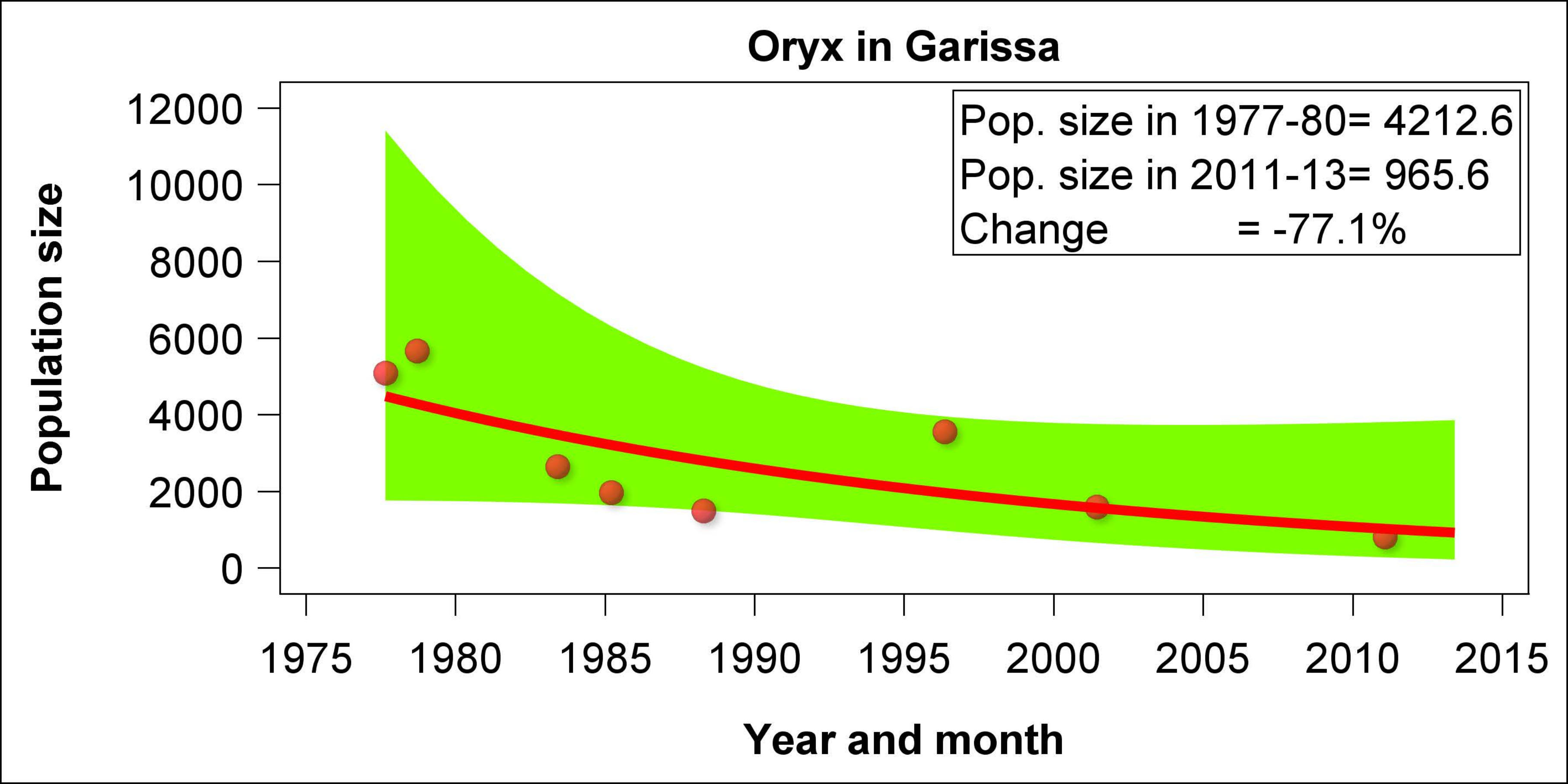

Oryx in Garissa
Pop. size in 1977-80= 4212.6
Pop. size in 2011-13= 965.6
Change = -77.1%
Population size
12000
10000
8000
6000
4000
2000
0
1975
1980
1985
1990
1995
2000
2005
2010
2015
Year and month

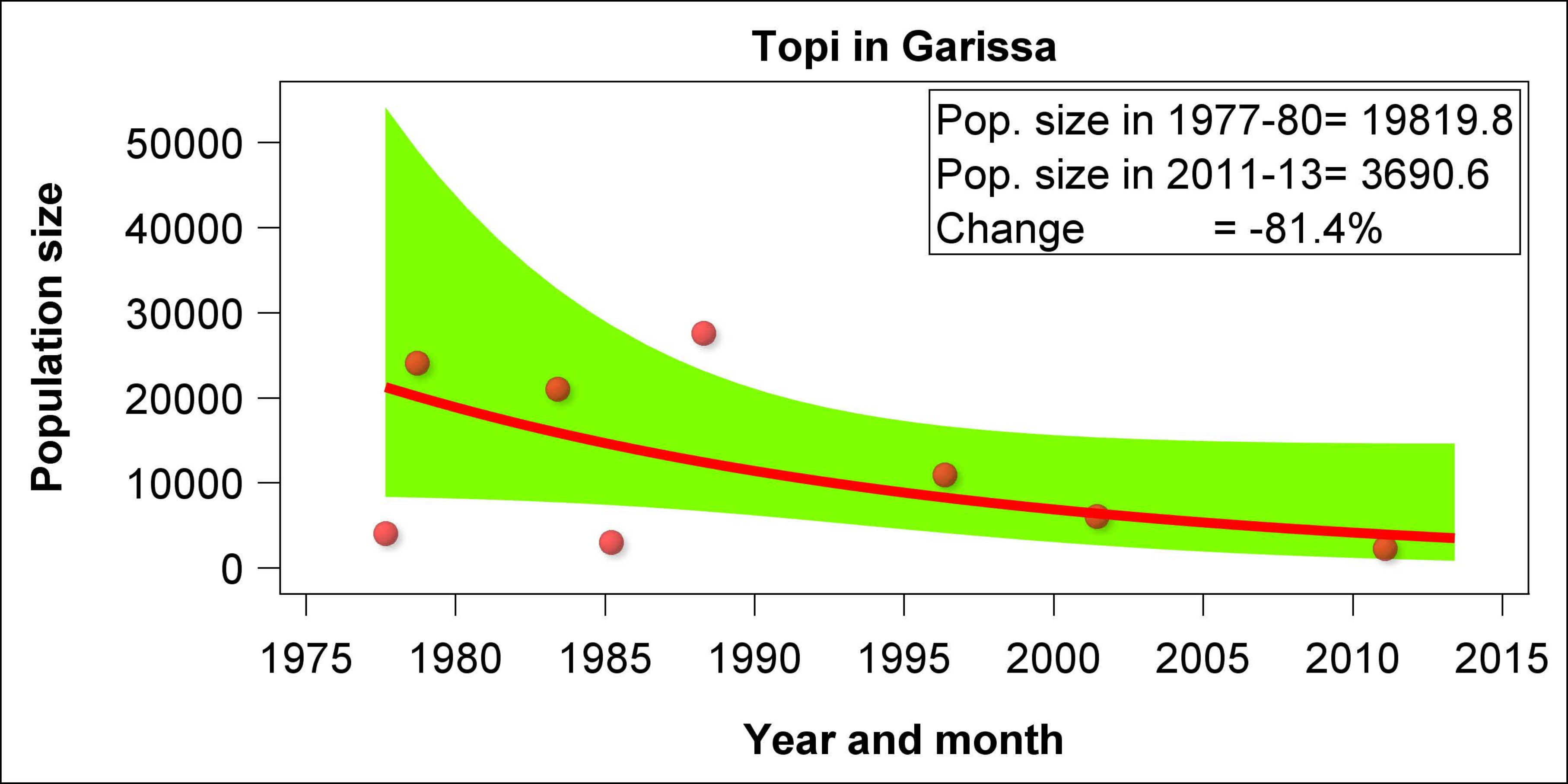

Topi in Garissa
Pop. size in 1977-80= 19819.8
Pop. size in 2011-13= 3690.6
Change = -81.4%
Population size
50000
40000
30000
20000
10000
0
1975
1980
1985
1990
1995
2000
2005
2010
2015
Year and month

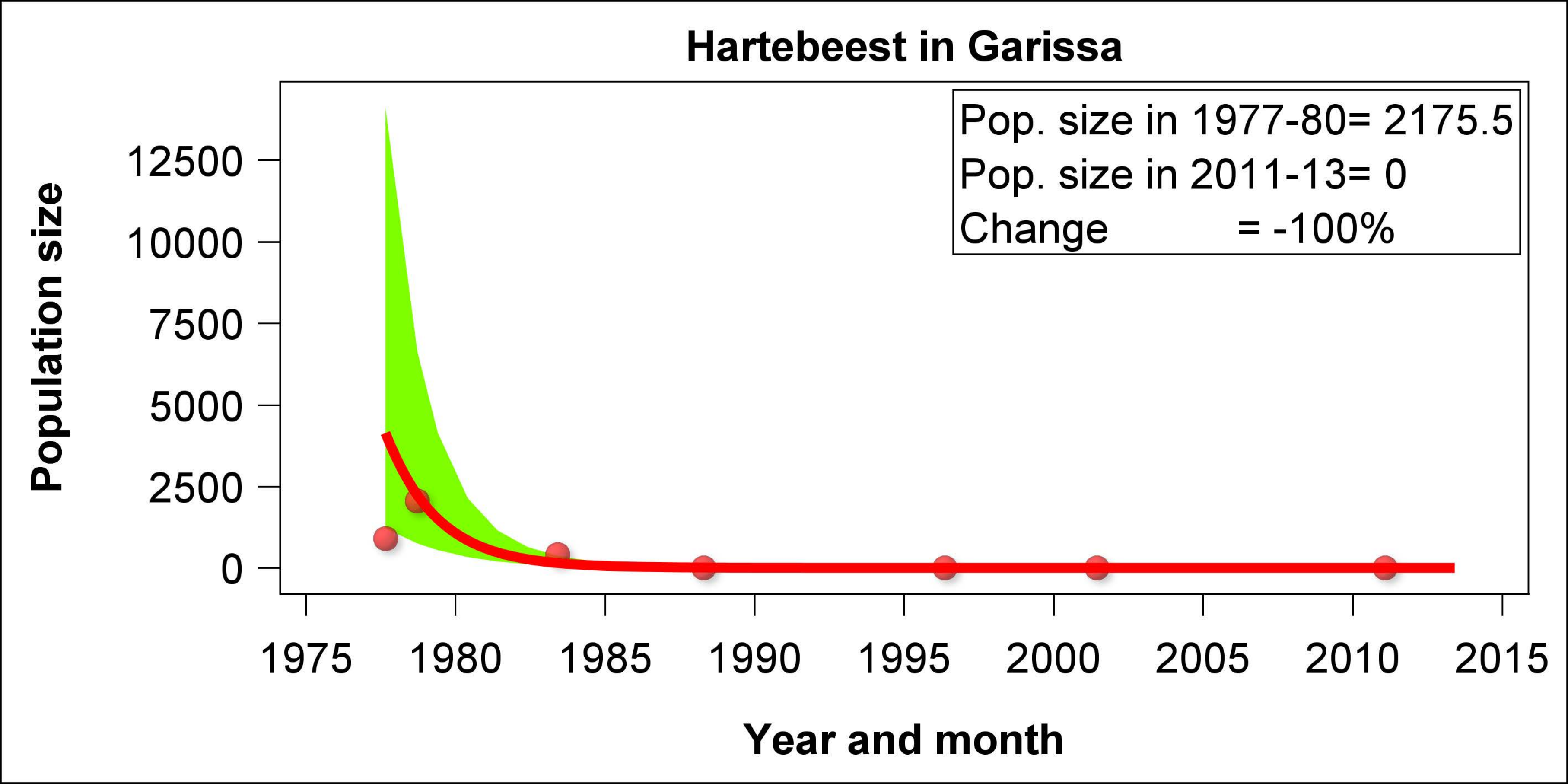
Hartebeest in Garissa
Pop. size in 1977-80= 2175.5
Pop. size in 2011-13= 0
Change = -100%
Population size
12500
10000
7500
5000
2500
0
1975
1980
1985
1990
1995
2000
2005
2010
2015
Year and month

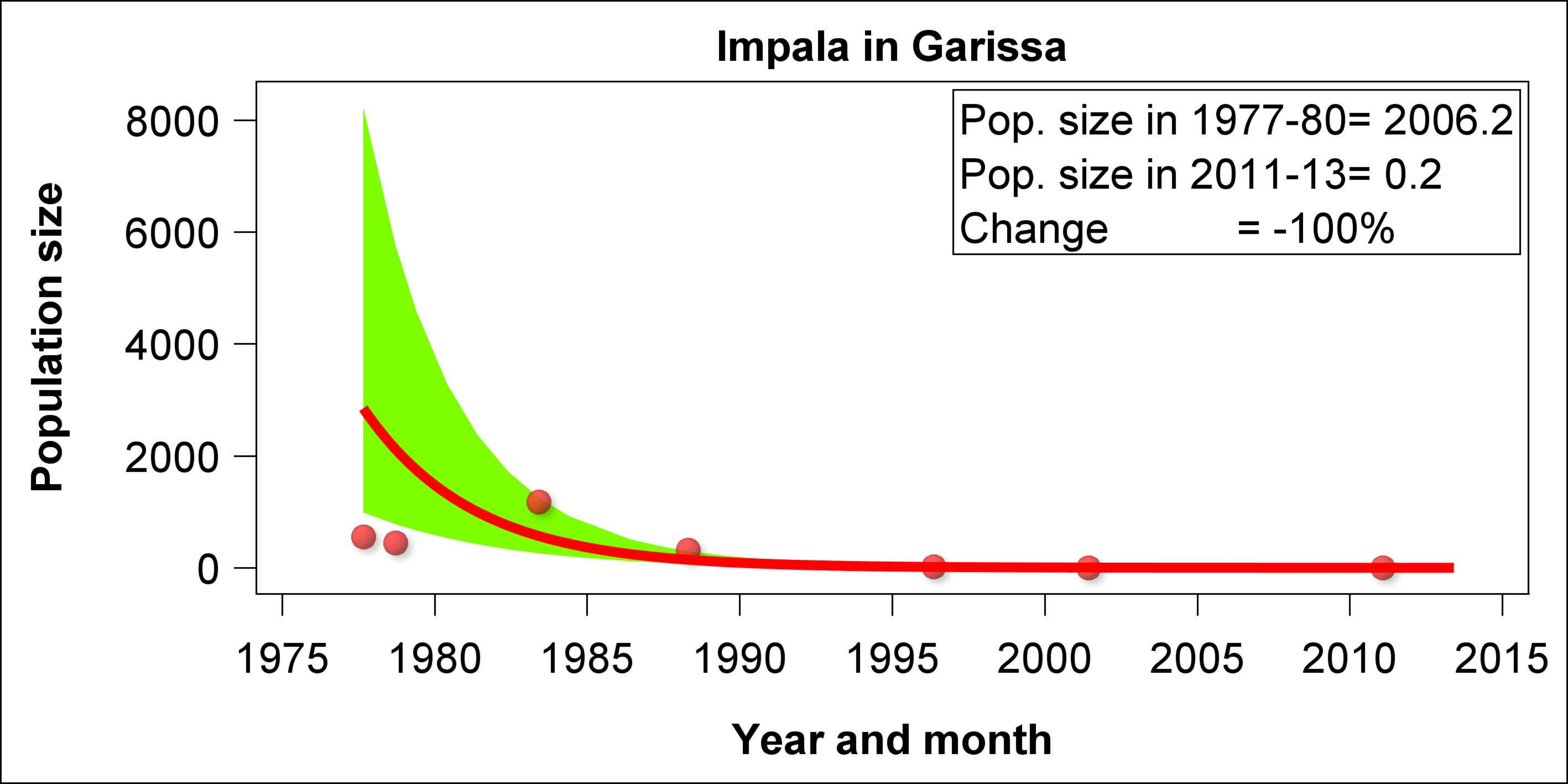

Impala in Garissa
Pop. size in 1977-80= 2006.2
Pop. size in 2011-13= 0.2
Change = -100%
Population size
8000
6000
4000
2000
0
1975
1980
1985
1990
1995
2000
2005
2010
2015
Year and month

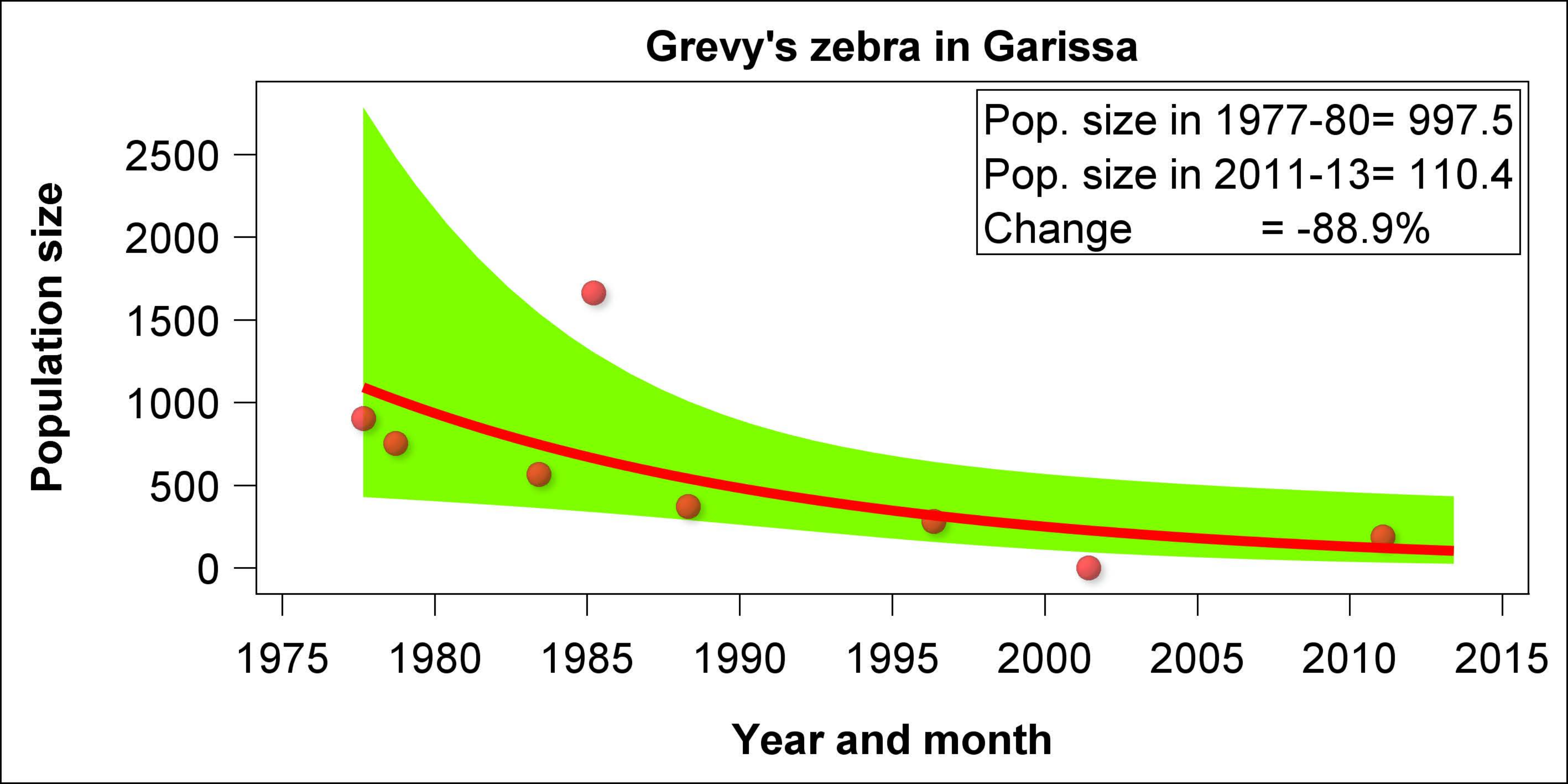

Grevy's zebra in Garissa
Pop. size in 1977-80= 997.5
Pop. size in 2011-13= 110.4
Change = -88.9%
Population size
0
500
1000
1500
2000
2500
1975
1980
1985
1990
1995
2000
2005
2010
2015
Year and month

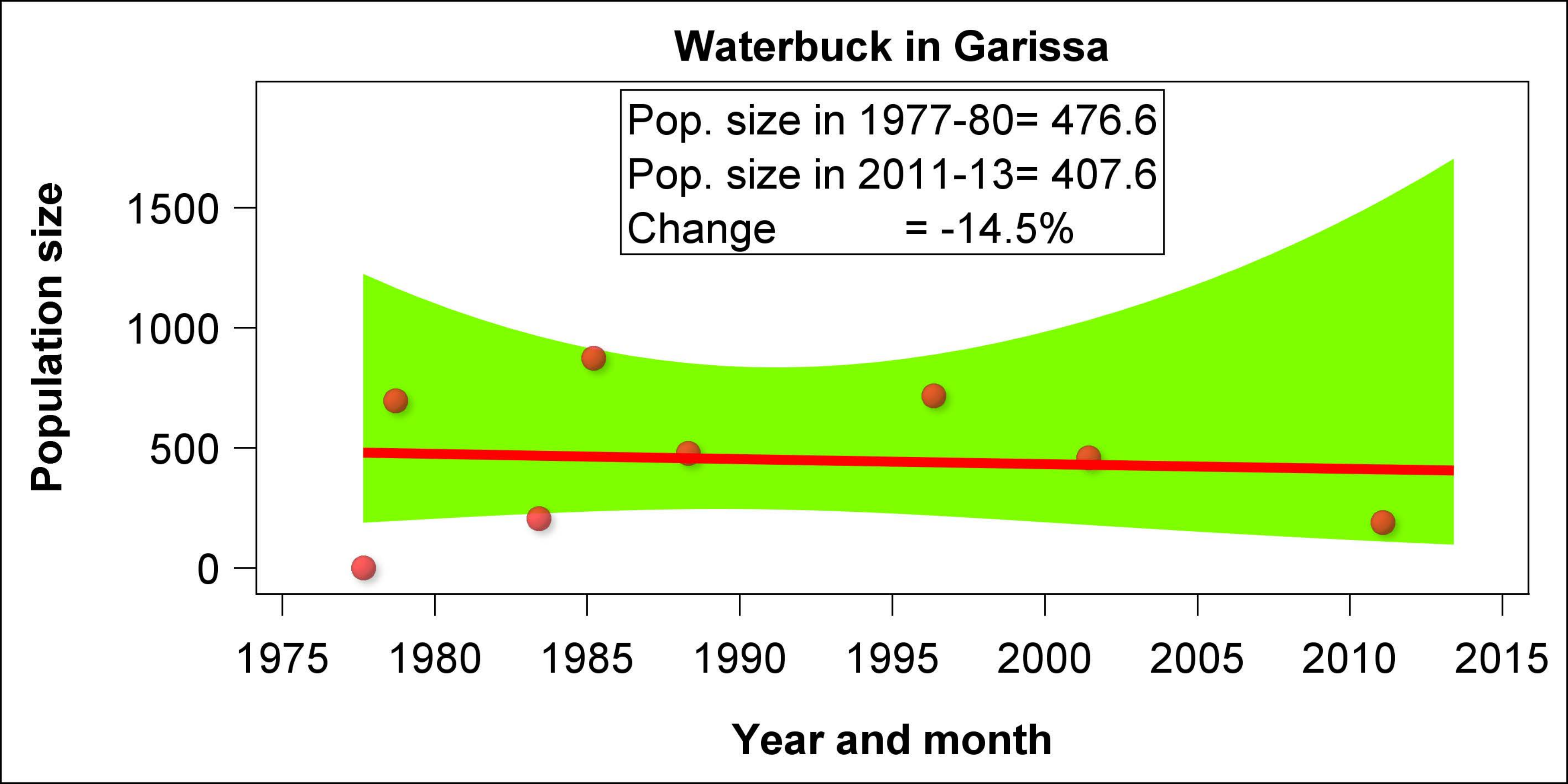

Waterbuck in Garissa
Pop. size in 1977-80= 476.6
Pop. size in 2011-13= 407.6
Change = -14.5%
Population size
1500
1000
500
0
1975
1980
1985
1990
1995
2000
2005
2010
2015
Year and month
