# Supplementary material for: Extreme Wildlife Declines and Concurrent Increase in Livestock Numbers in Kenya: What Are the Causes?
Source: PLoS One. 2016 Sep 27;11(9):e0163249. doi: 10.1371/journal.pone.0163249 (PMC5039022; doi:10.1371/journal.pone.0163249)
Supplement: S16 Fig — The solid red line is the fitted trend curve and the shaded chartreuse band is the pointwise 95% confidence band. The estimated average population size in 1977–1980 and 2011–2013 and the percentage change in population size between the two periods are provided in the inset. (PDF) [file pone.0163249.s026.pdf]

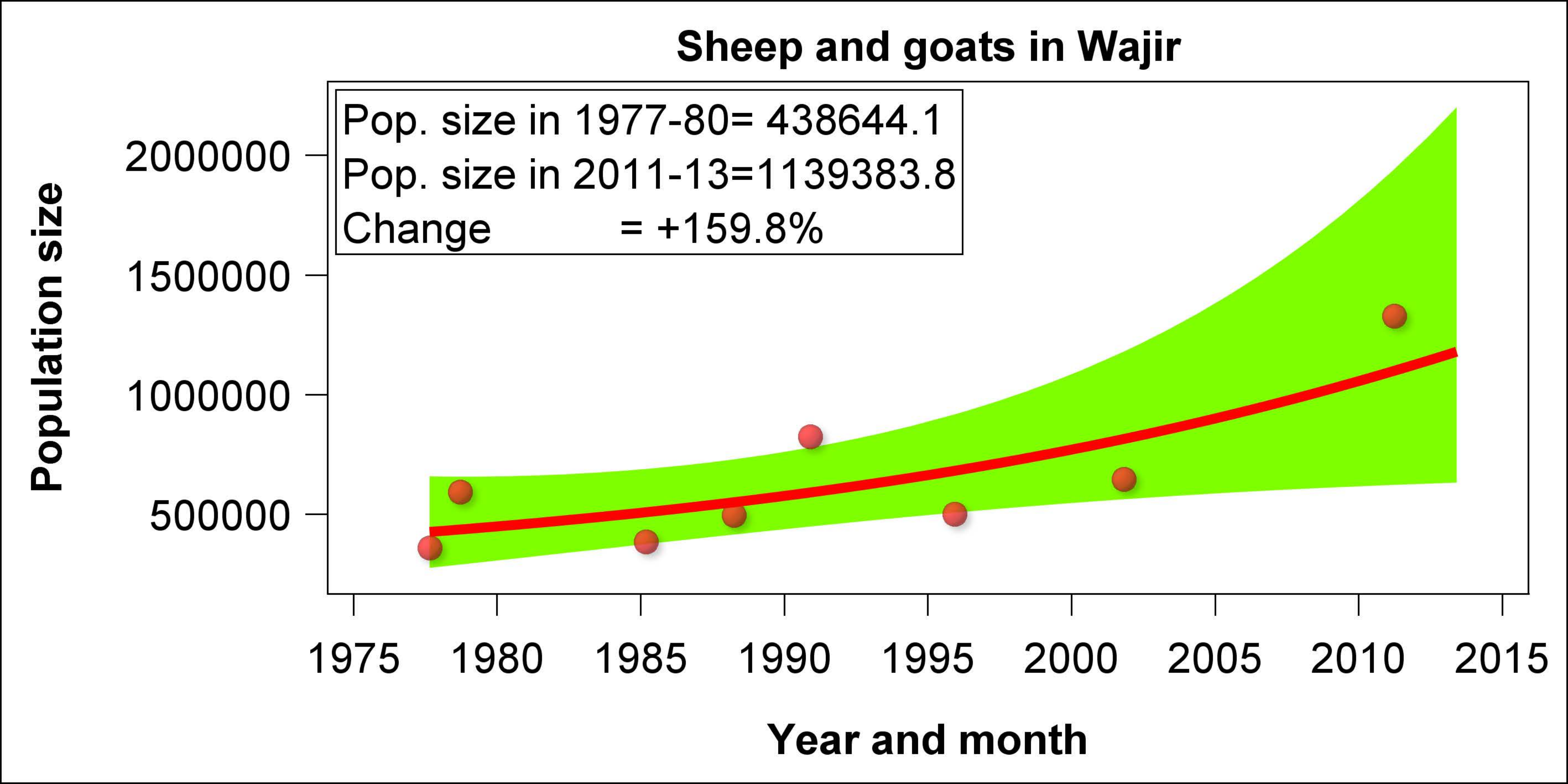
Sheep and goats in Wajir
Pop. size in 1977-80= 438644.1
Pop. size in 2011-13=1139383.8
Change = +159.8%
Population size
2000000
1500000
1000000
500000
1975
1980
1985
1990
1995
2000
2005
2010
2015
Year and month

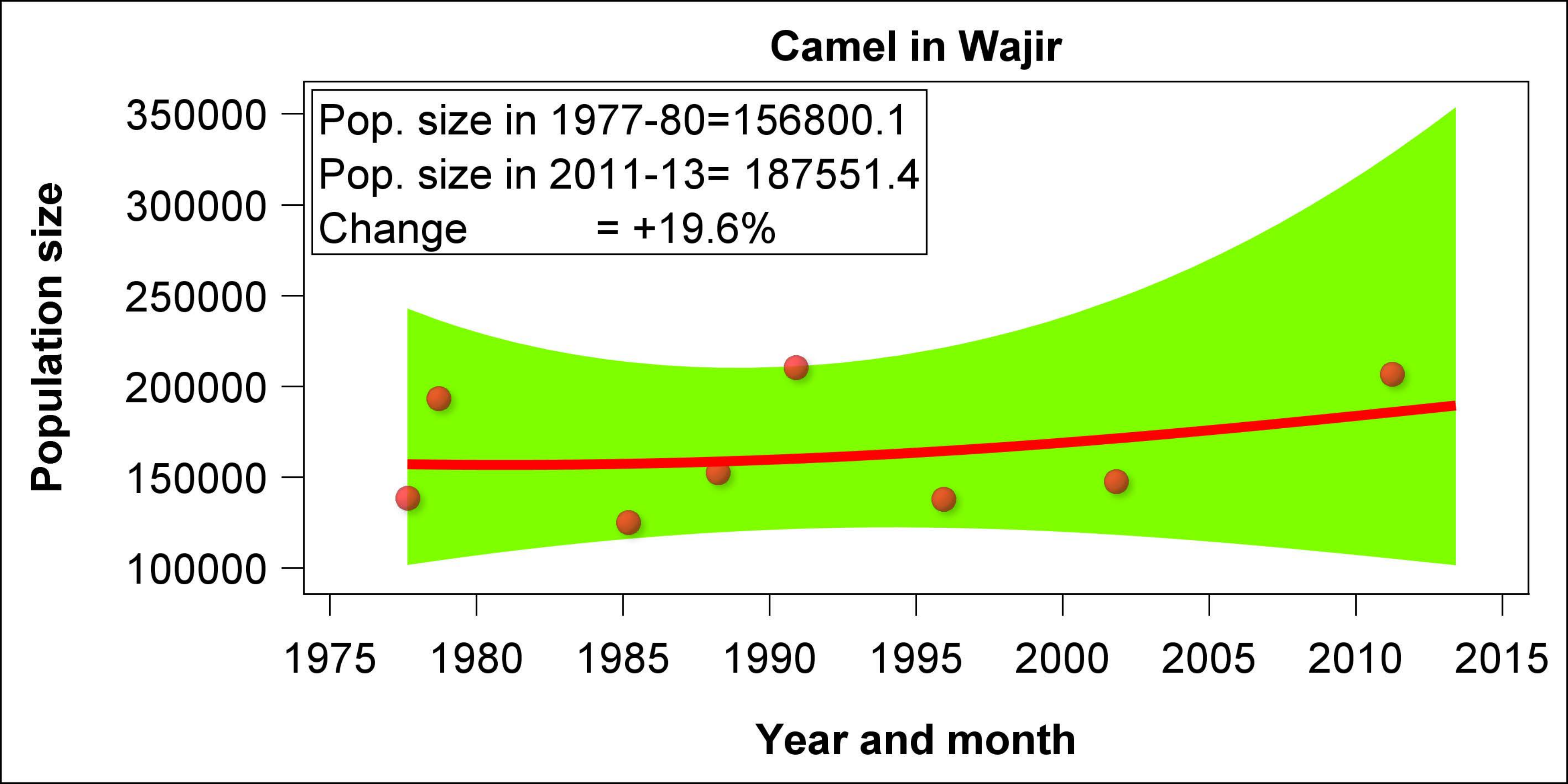
Camel in Wajir
Pop. size in 1977-80=156800.1
Pop. size in 2011-13= 187551.4
Change = +19.6%
Population size
350000
300000
250000
200000
150000
100000
1975
1980
1985
1990
1995
2000
2005
2010
2015
Year and month

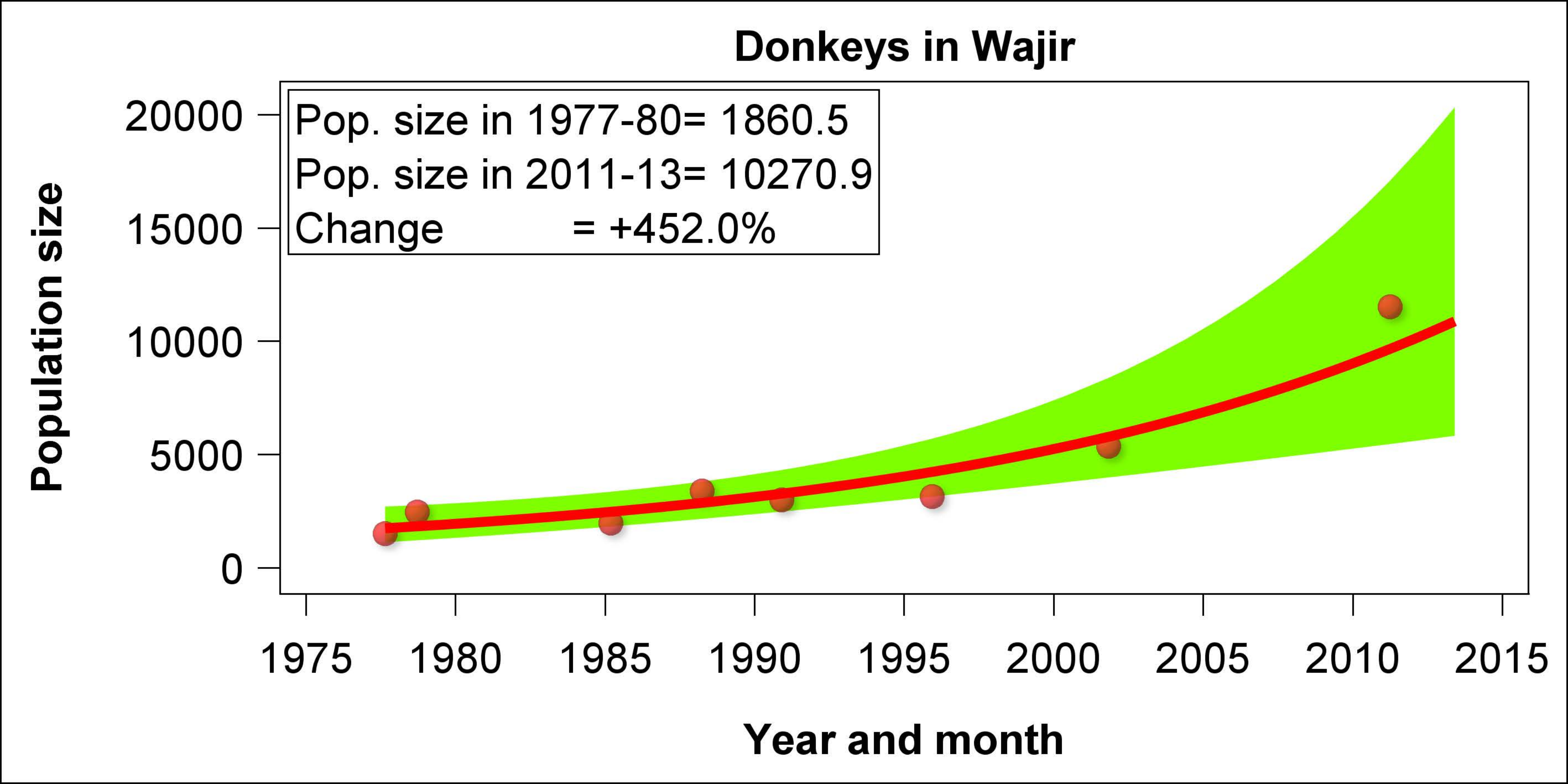

Donkeys in Wajir
Pop. size in 1977-80= 1860.5
Pop. size in 2011-13= 10270.9
Change = +452.0%
Population size
20000
15000
10000
5000
0
1975
1980
1985
1990
1995
2000
2005
2010
2015
Year and month

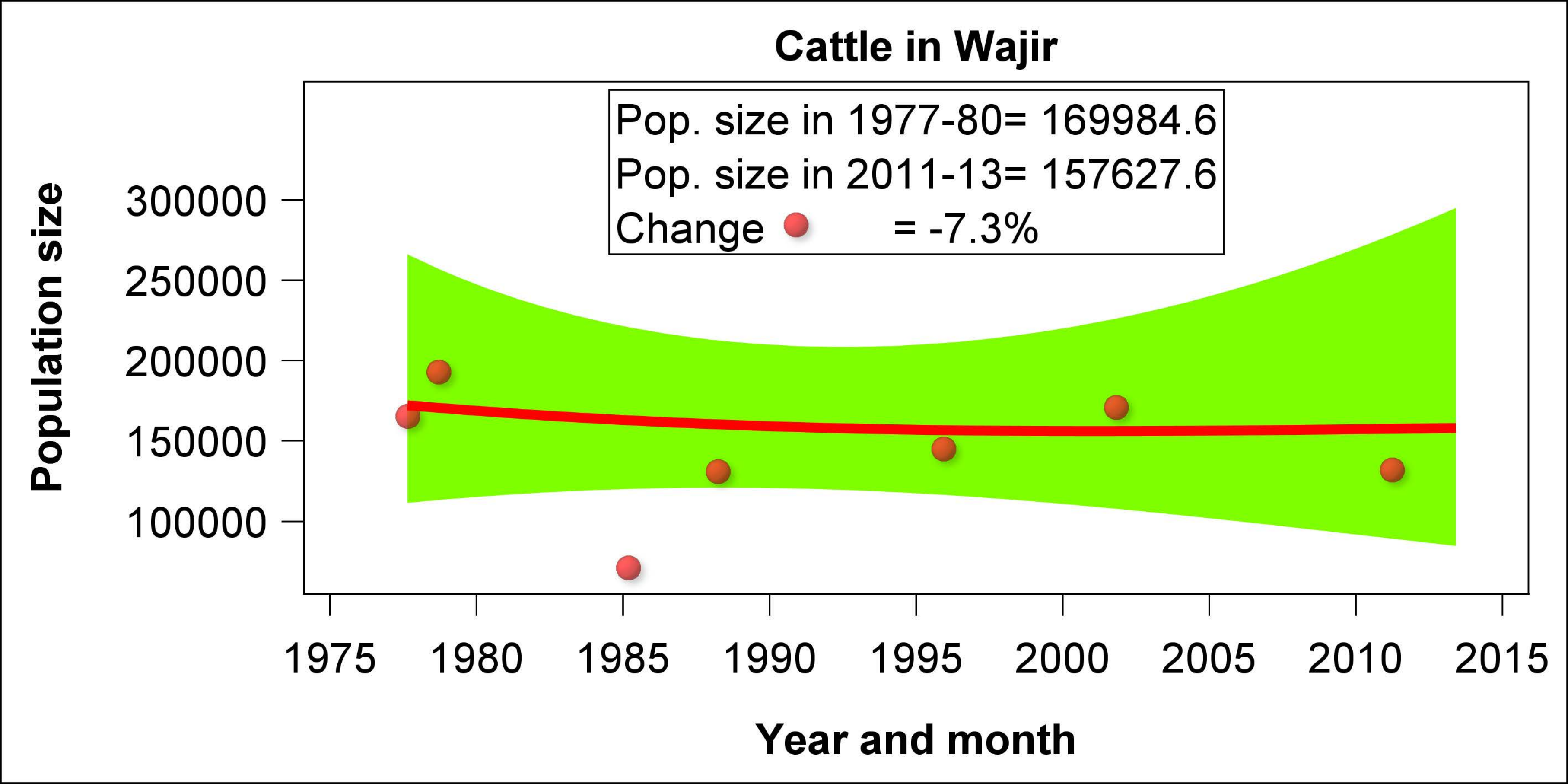

Cattle in Wajir
Pop. size in 1977-80= 169984.6
Pop. size in 2011-13= 157627.6
Change = -7.3%
300000
250000
200000
150000
100000
Population size
1975
1980
1985
1990
1995
2000
2005
2010
2015
Year and month

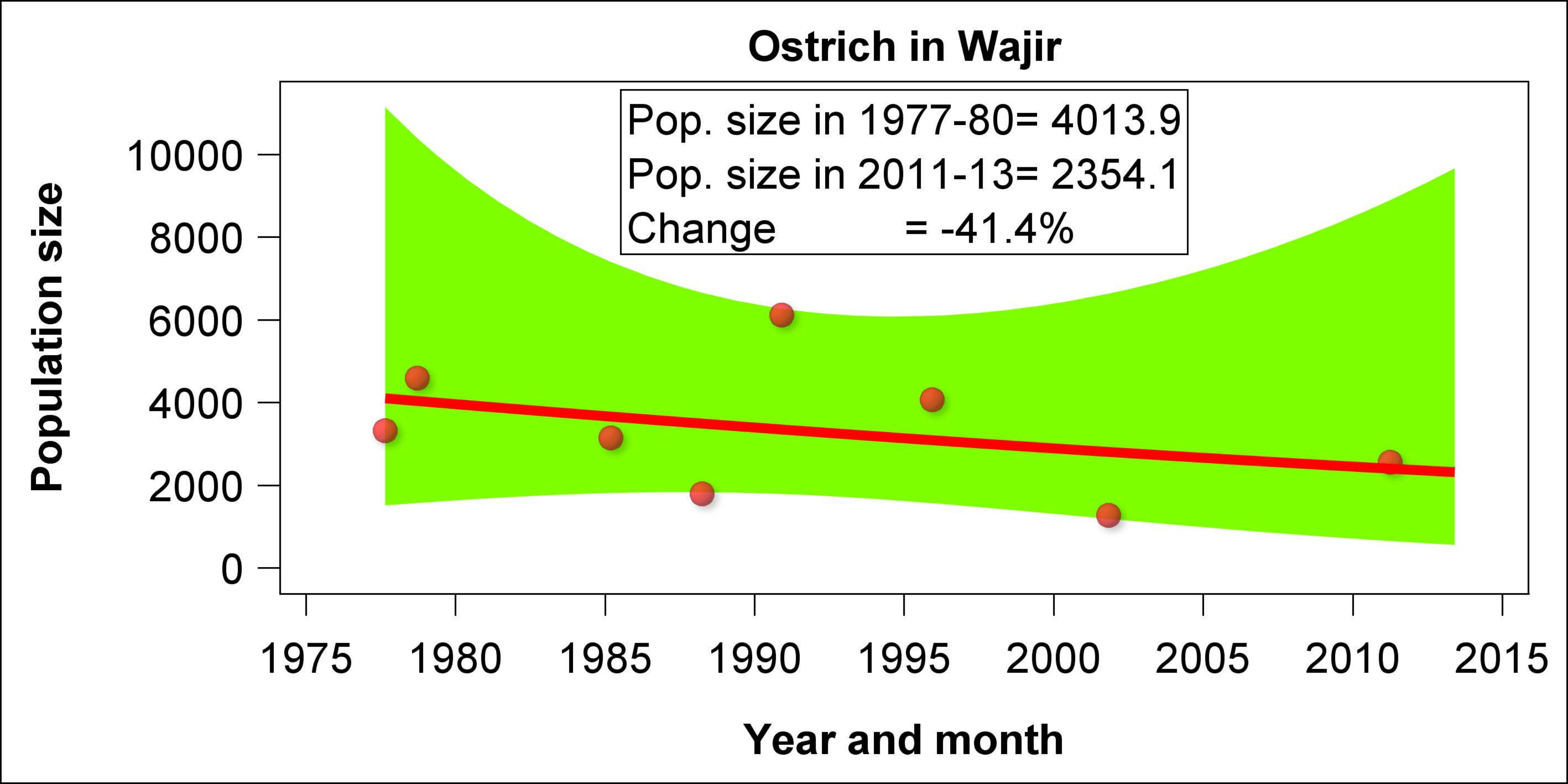

Ostrich in Wajir
Pop. size in 1977-80= 4013.9
Pop. size in 2011-13= 2354.1
Change = -41.4%
Population size
10000
8000
6000
4000
2000
0
1975
1980
1985
1990
1995
2000
2005
2010
2015
Year and month

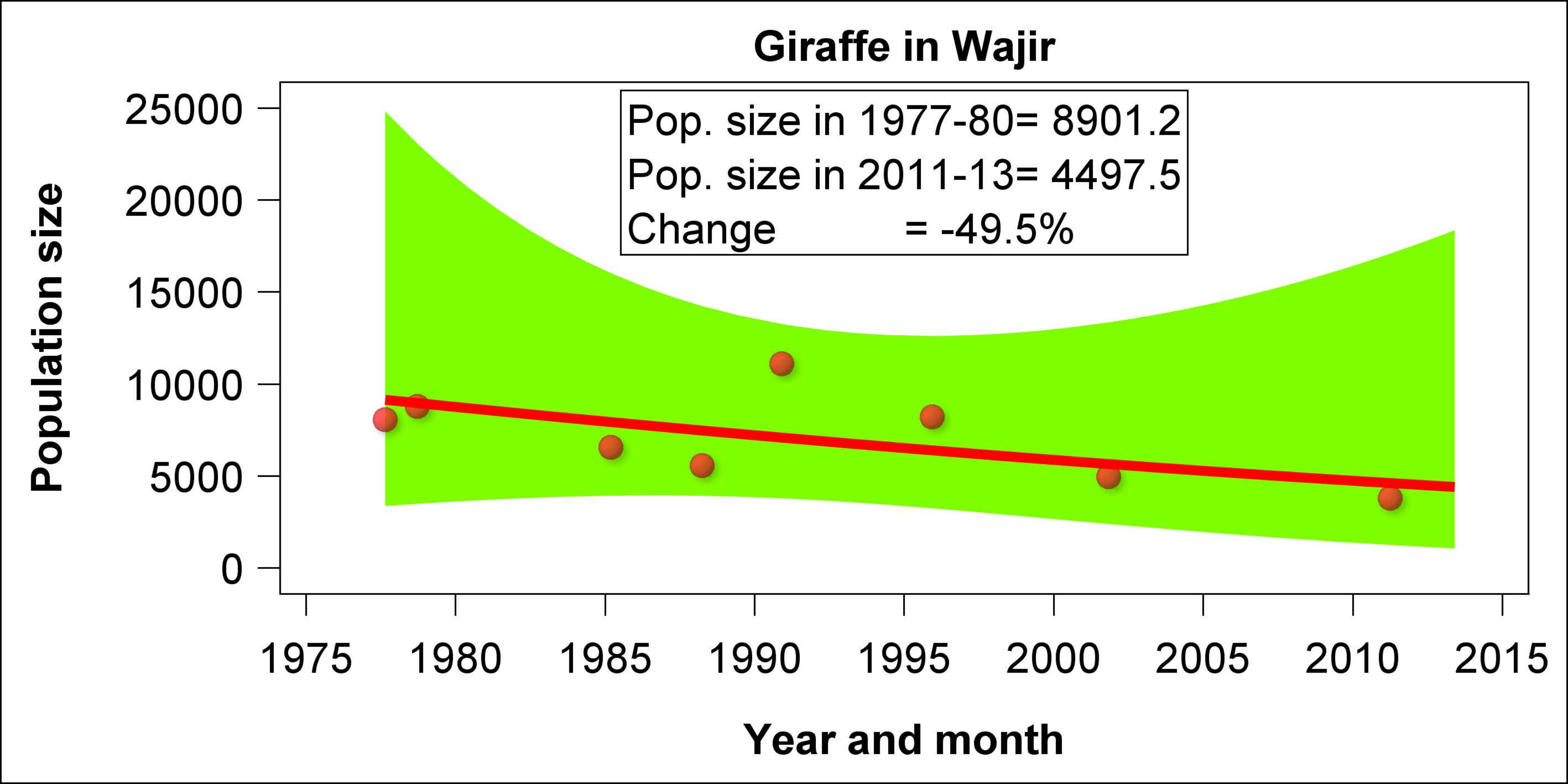
Giraffe in Wajir
Pop. size in 1977-80= 8901.2
Pop. size in 2011-13= 4497.5
Change = -49.5%
Population size
25000
20000
15000
10000
5000
0
1975
1980
1985
1990
1995
2000
2005
2010
2015
Year and month

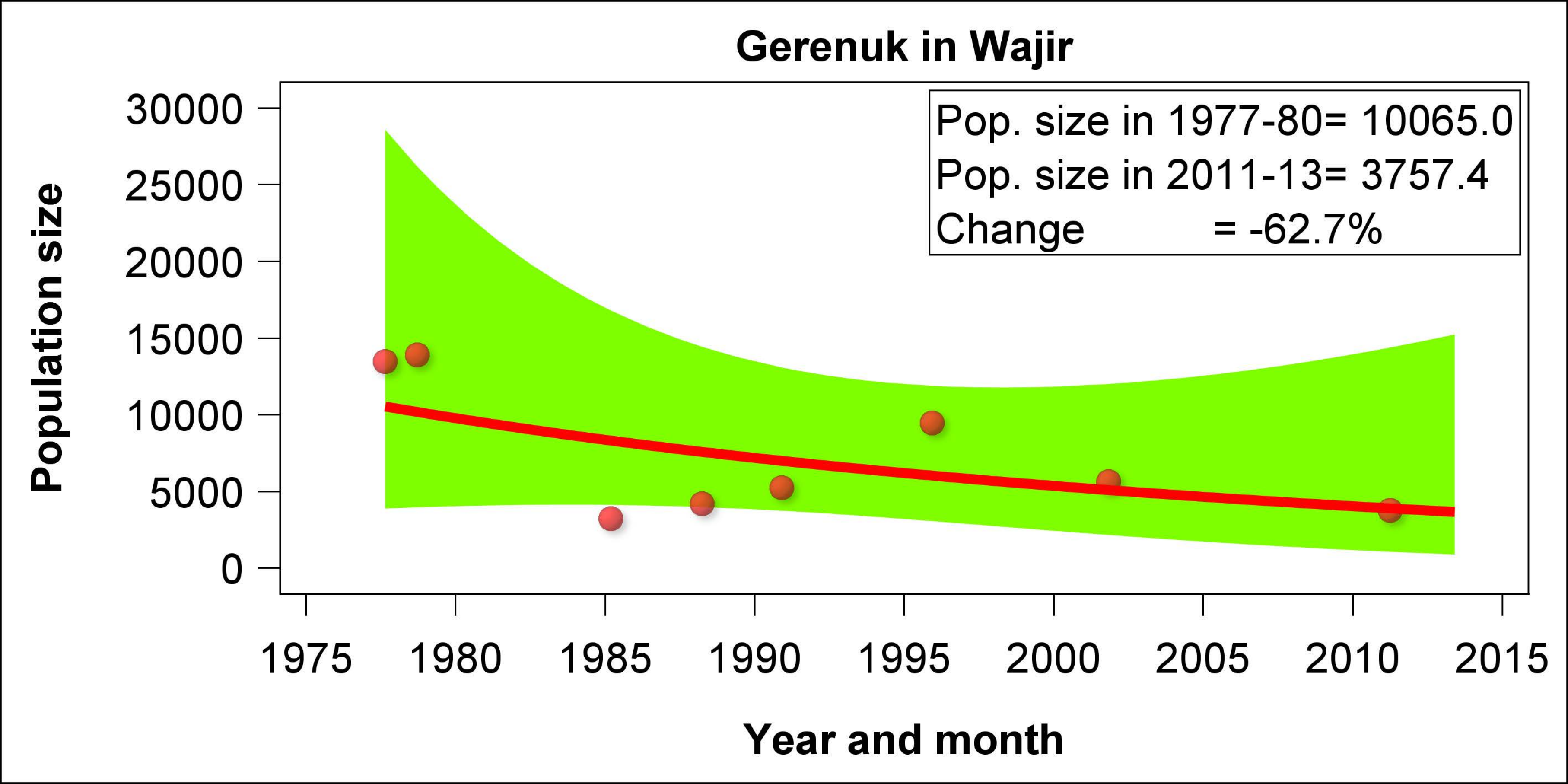
Gerenuk in Wajir
Pop. size in 1977-80= 10065.0
Pop. size in 2011-13= 3757.4
Change = -62.7%
Population size
30000
25000
20000
15000
10000
5000
0
1975
1980
1985
1990
1995
2000
2005
2010
2015
Year and month

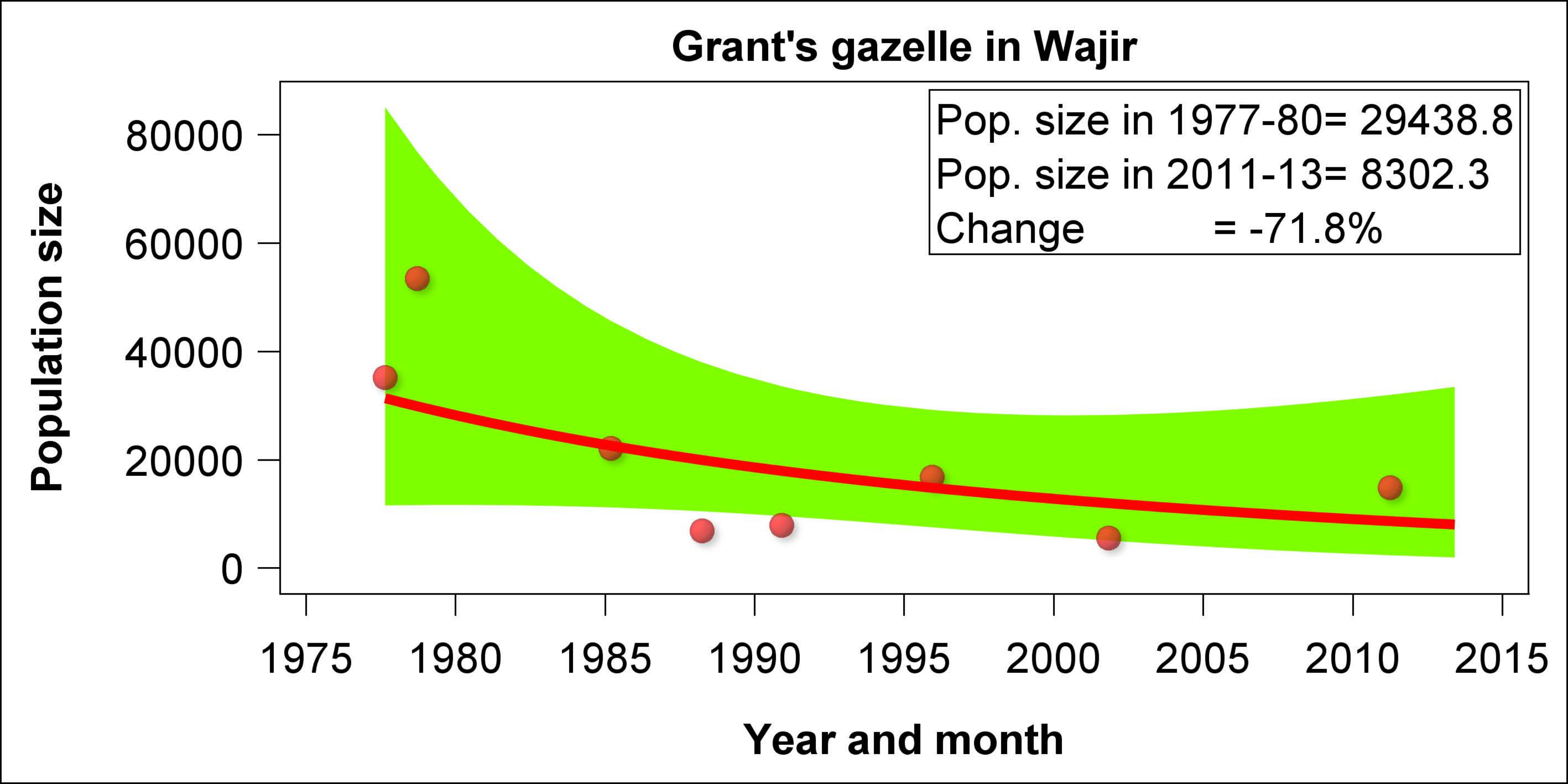

Grant's gazelle in Wajir
Pop. size in 1977-80= 29438.8
Pop. size in 2011-13= 8302.3
Change = -71.8%
Population size
80000
60000
40000
20000
0
1975
1980
1985
1990
1995
2000
2005
2010
2015
Year and month

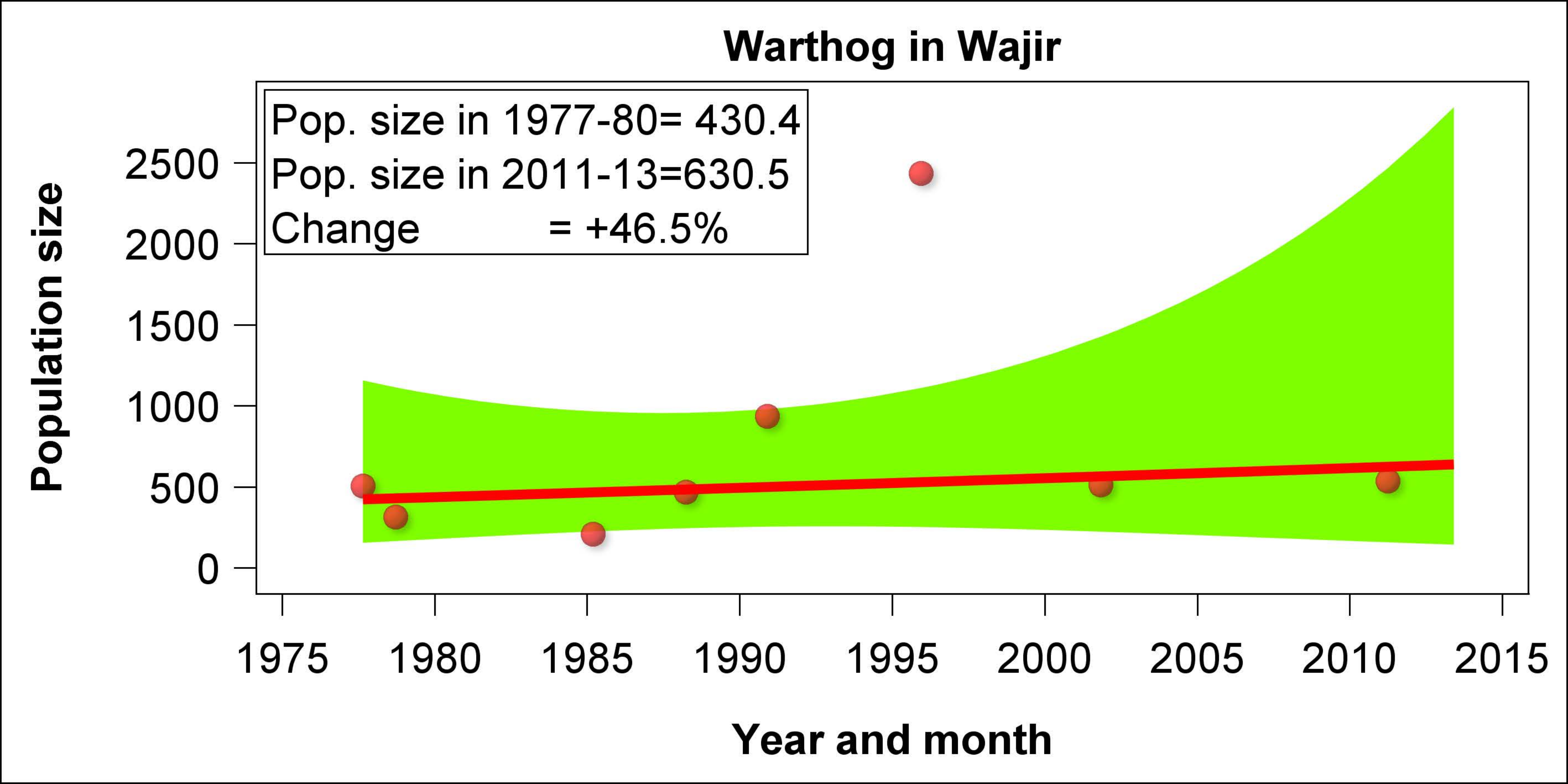

Warthog in Wajir
Pop. size in 1977-80= 430.4
Pop. size in 2011-13=630.5
Change = +46.5%
Population size
0
500
1000
1500
2000
2500
1975
1980
1985
1990
1995
2000
2005
2010
2015
Year and month

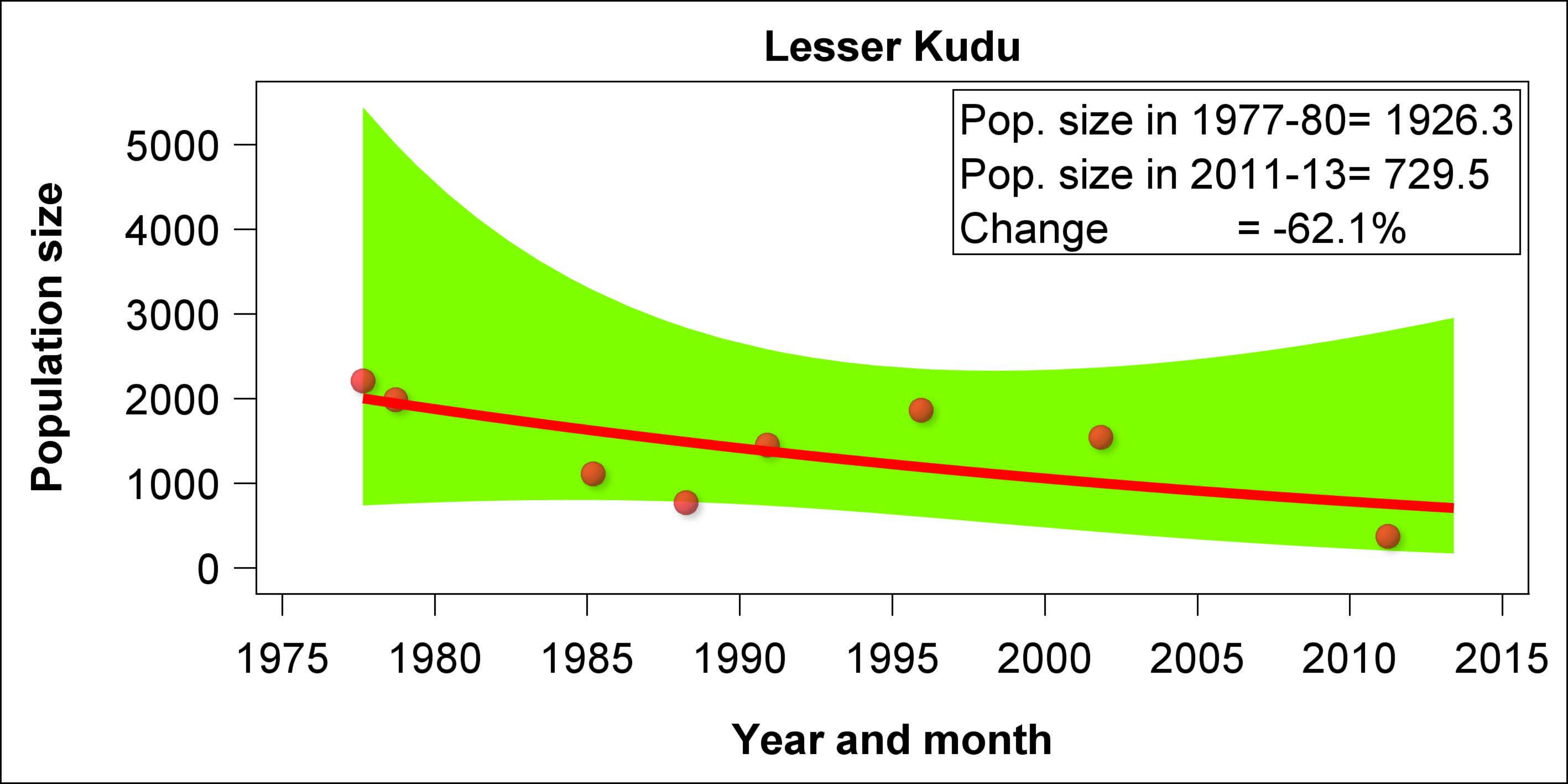

Lesser Kudu
Pop. size in 1977-80= 1926.3
Pop. size in 2011-13= 729.5
Change = -62.1%
Population size
5000
4000
3000
2000
1000
0
1975
1980
1985
1990
1995
2000
2005
2010
2015
Year and month

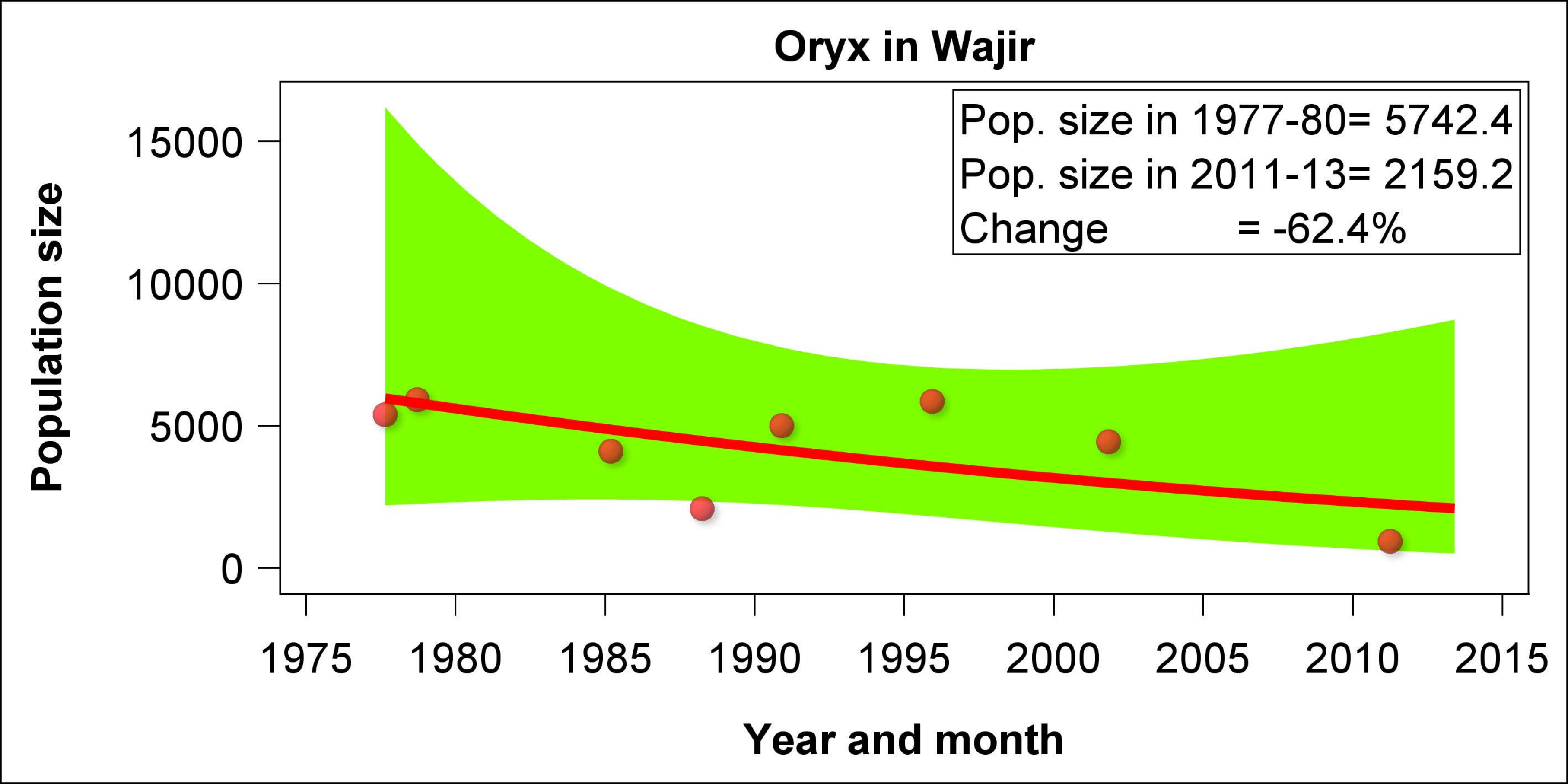
Oryx in Wajir
Pop. size in 1977-80= 5742.4
Pop. size in 2011-13= 2159.2
Change = -62.4%
Population size
15000
10000
5000
0
1975
1980
1985
1990
1995
2000
2005
2010
2015
Year and month

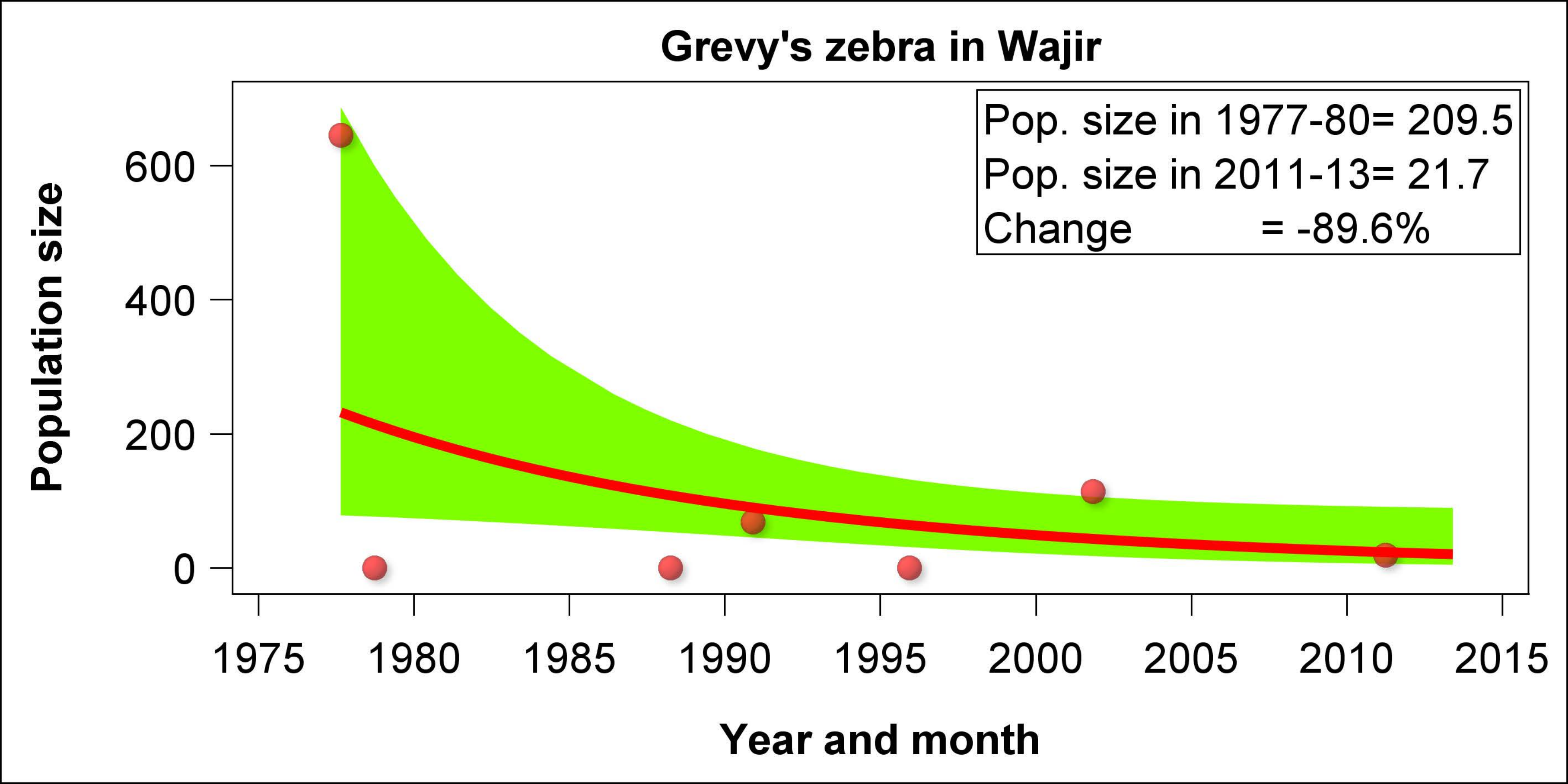

Grevy's zebra in Wajir
Pop. size in 1977-80= 209.5
Pop. size in 2011-13= 21.7
Change = -89.6%
Population size
600
400
200
0
1975
1980
1985
1990
1995
2000
2005
2010
2015
Year and month
