# Supplementary material for: Extreme Wildlife Declines and Concurrent Increase in Livestock Numbers in Kenya: What Are the Causes?
Source: PLoS One. 2016 Sep 27;11(9):e0163249. doi: 10.1371/journal.pone.0163249 (PMC5039022; doi:10.1371/journal.pone.0163249)
Supplement: S17 Fig — The solid red line is the fitted trend curve and the shaded chartreuse band is the pointwise 95% confidence band. The estimated average population size in 1977–1980 and 2011–2013 and the percentage change in population size between the two periods are provided in the inset. (PDF) [file pone.0163249.s027.pdf]

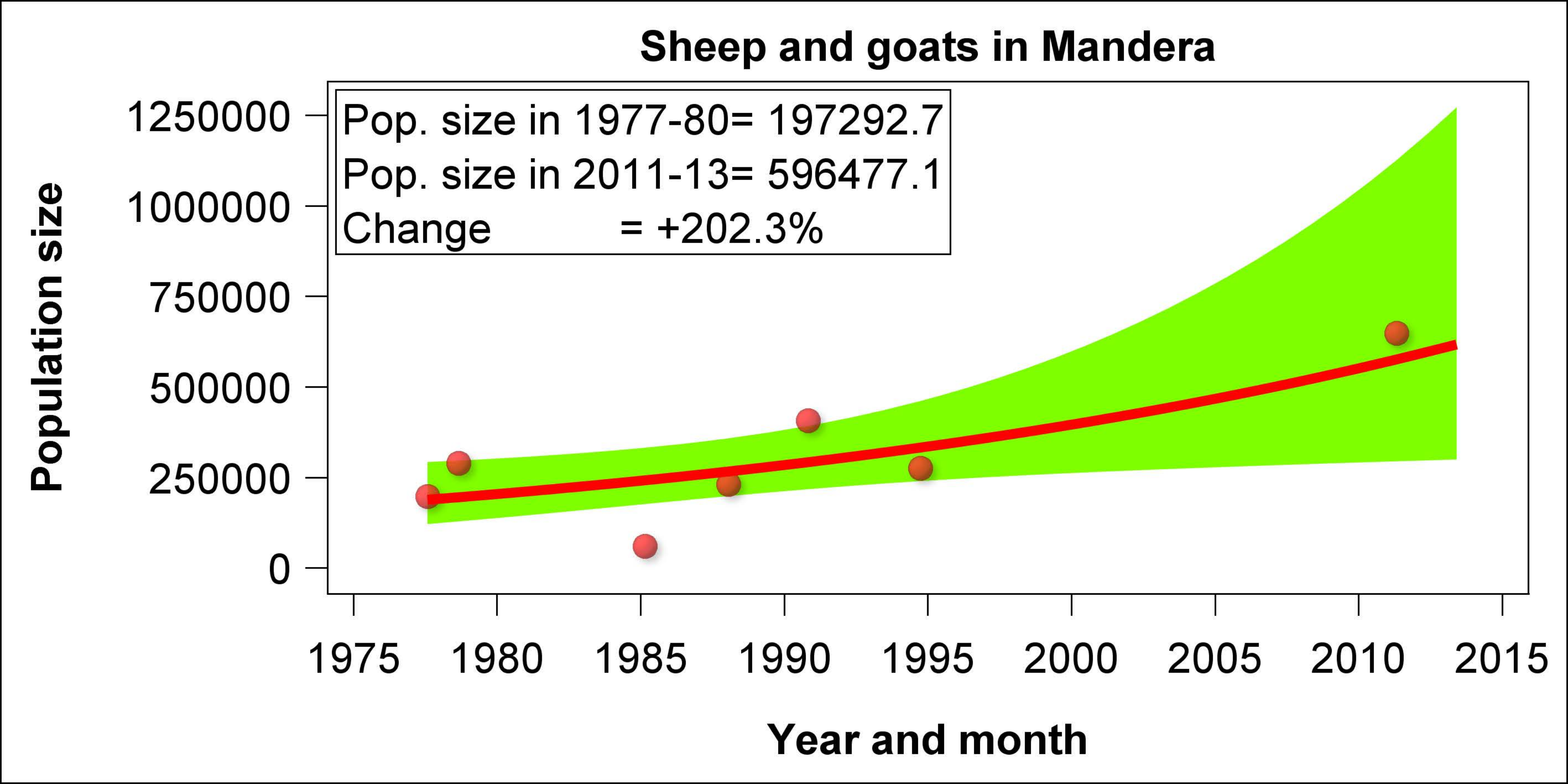
Sheep and goats in Mandera
Pop. size in 1977-80= 197292.7
Pop. size in 2011-13= 596477.1
Change = +202.3%
Population size
1250000
1000000
750000
500000
250000
0
1975
1980
1985
1990
1995
2000
2005
2010
2015
Year and month

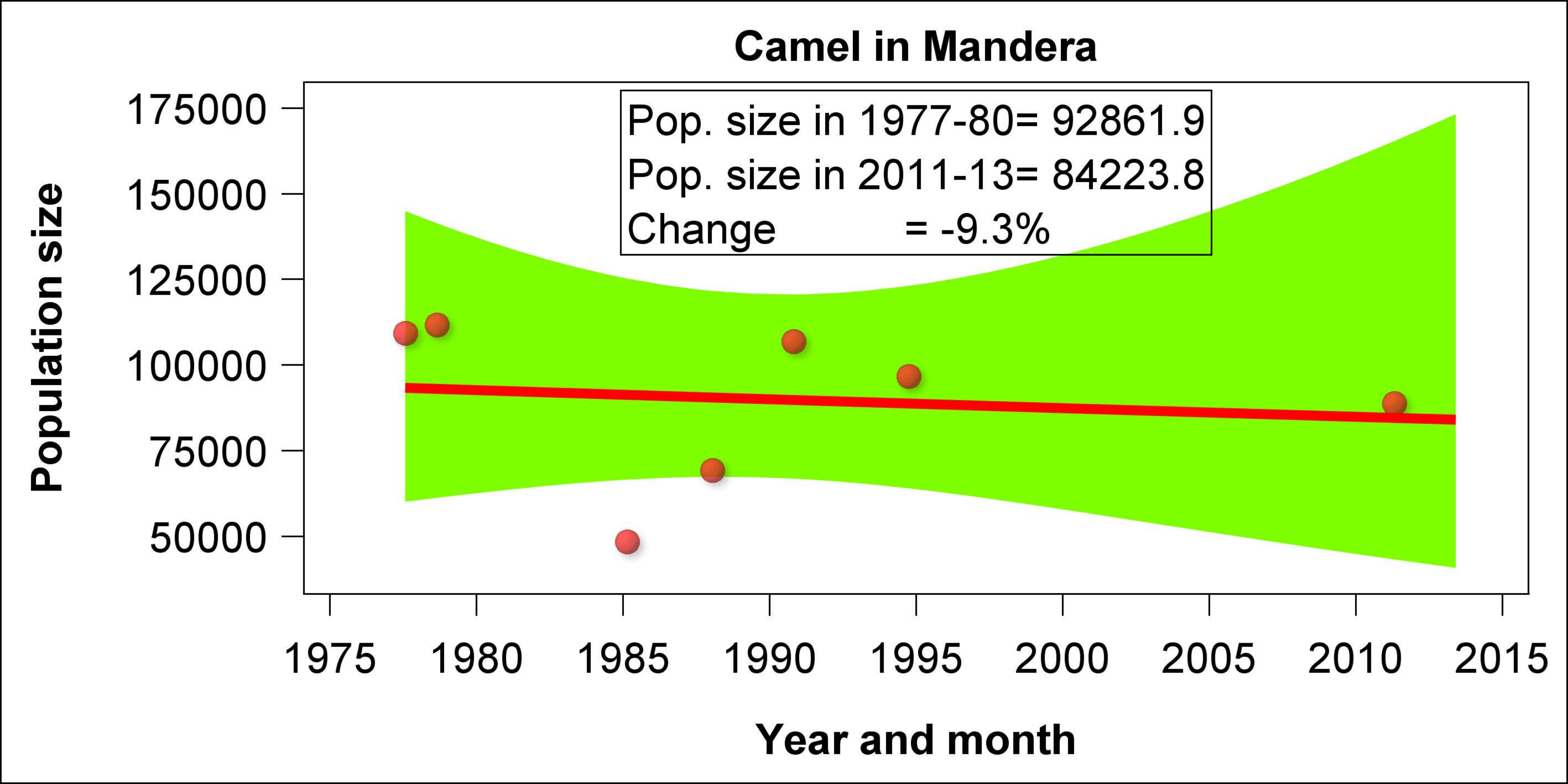
Camel in Mandera
Pop. size in 1977-80= 92861.9
Pop. size in 2011-13= 84223.8
Change = -9.3%
Population size
175000
150000
125000
100000
75000
50000
1975
1980
1985
1990
1995
2000
2005
2010
2015
Year and month

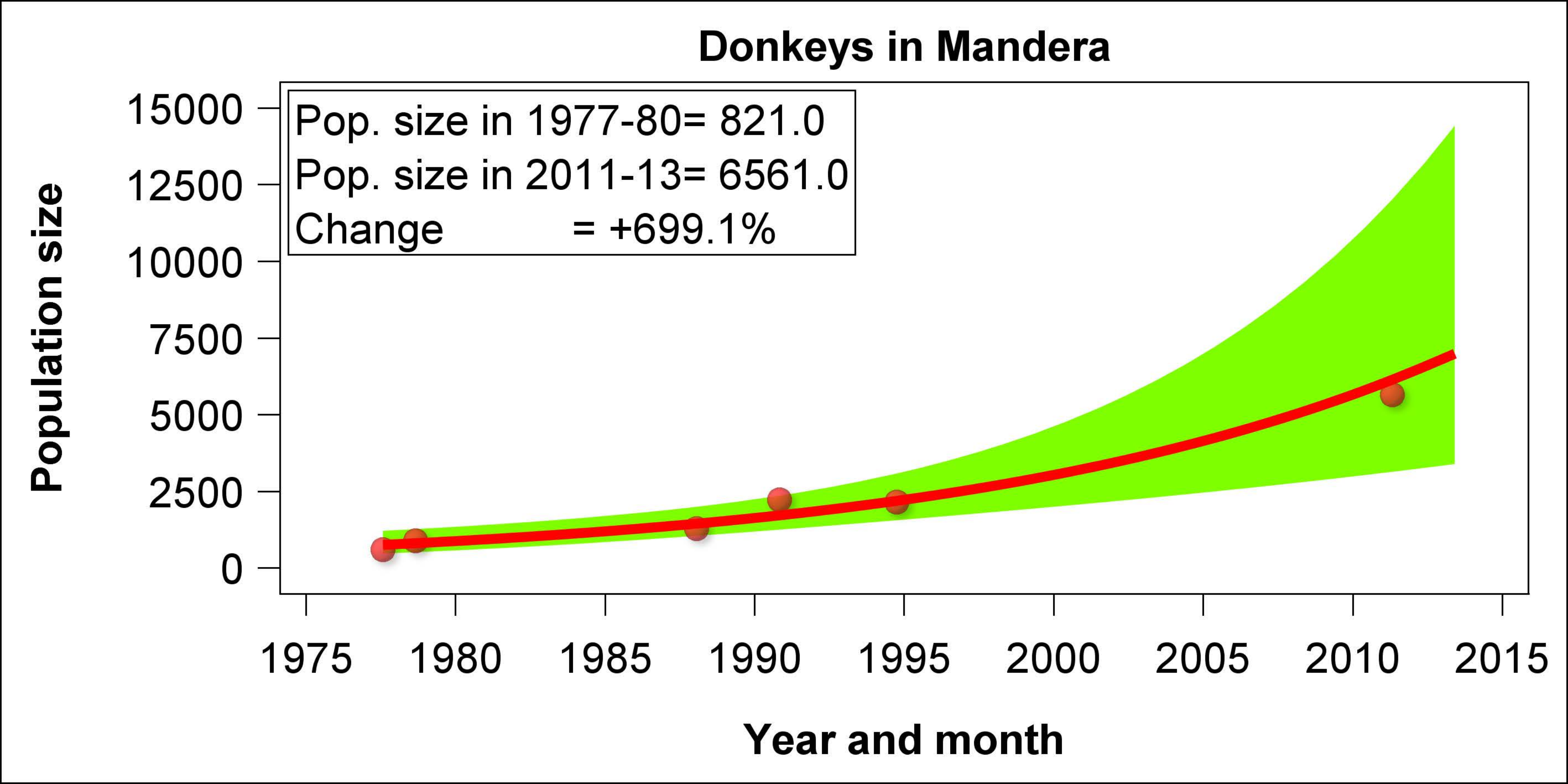
Donkeys in Mandera
Pop. size in 1977-80= 821.0
Pop. size in 2011-13= 6561.0
Change = +699.1%
Population size
15000
12500
10000
7500
5000
2500
0
1975
1980
1985
1990
1995
2000
2005
2010
2015
Year and month

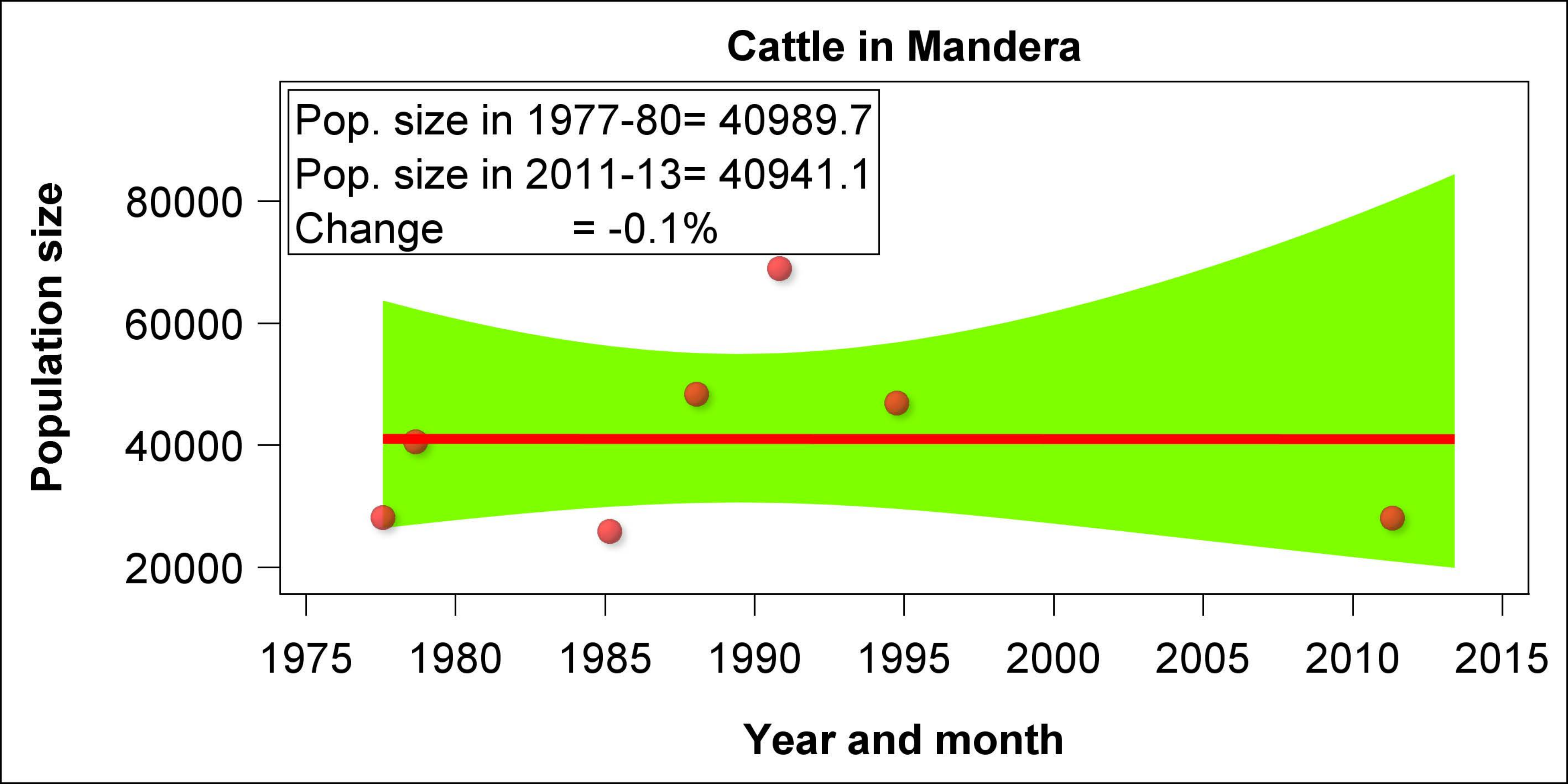

Cattle in Mandera
Pop. size in 1977-80= 40989.7
Pop. size in 2011-13= 40941.1
Change = -0.1%
Population size
80000
60000
40000
20000
1975
1980
1985
1990
1995
2000
2005
2010
2015
Year and month

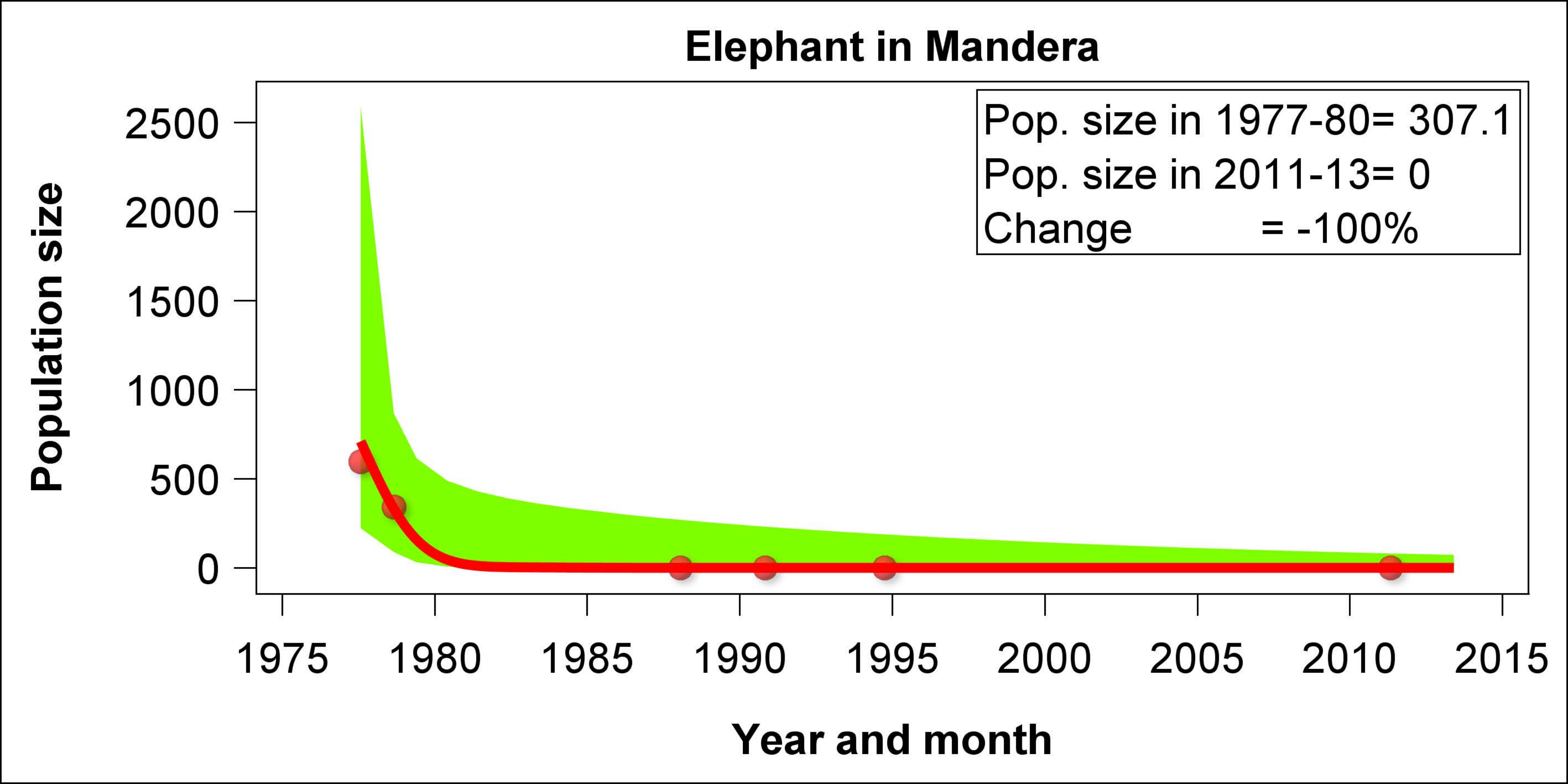
Elephant in Mandera
Pop. size in 1977-80= 307.1
Pop. size in 2011-13= 0
Change = -100%
Population size
2500
2000
1500
1000
500
0
1975
1980
1985
1990
1995
2000
2005
2010
2015
Year and month

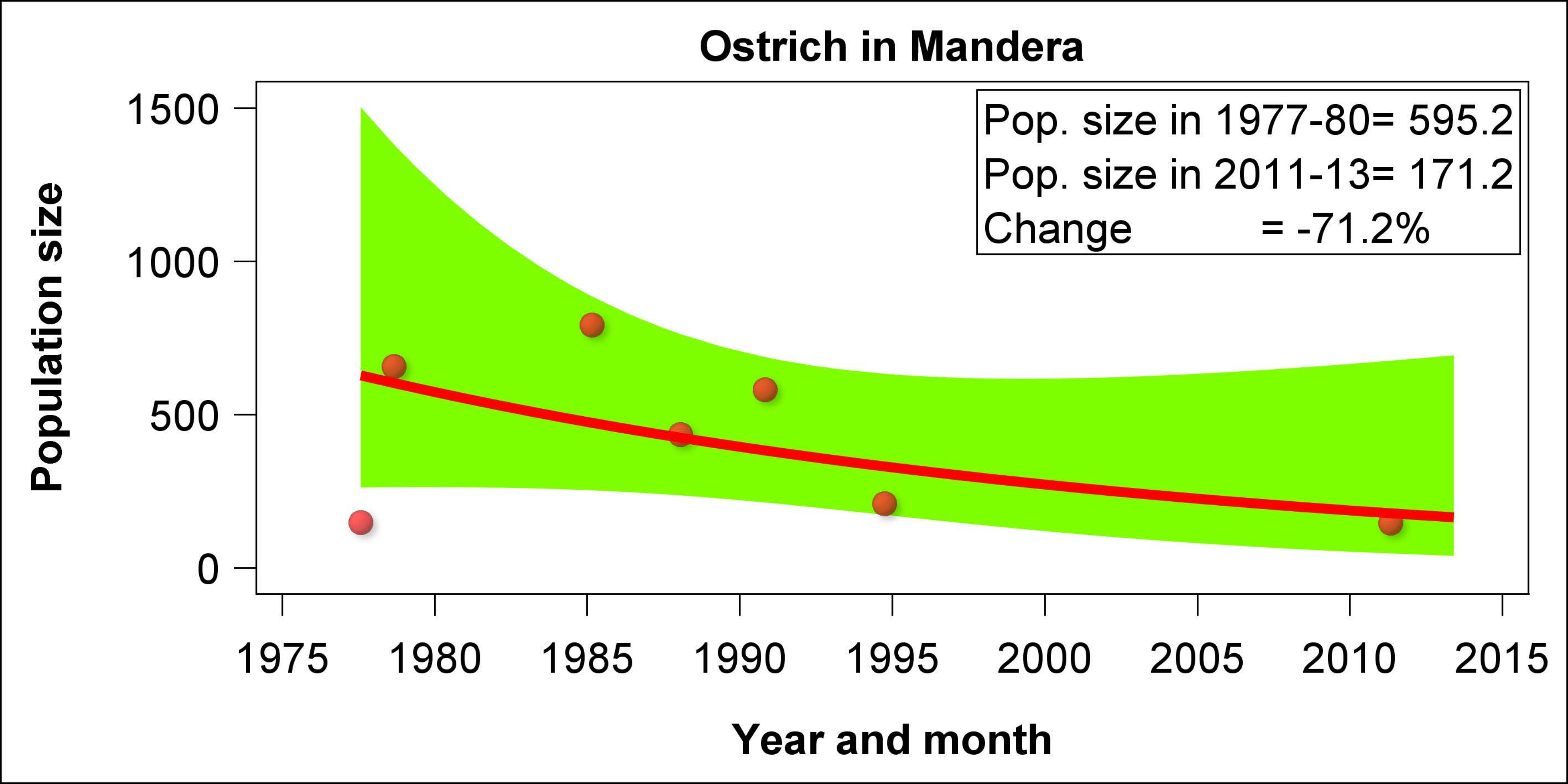
Ostrich in Mandera
Pop. size in 1977-80= 595.2
Pop. size in 2011-13= 171.2
Change = -71.2%
Population size
1500
1000
500
0
1975
1980
1985
1990
1995
2000
2005
2010
2015
Year and month

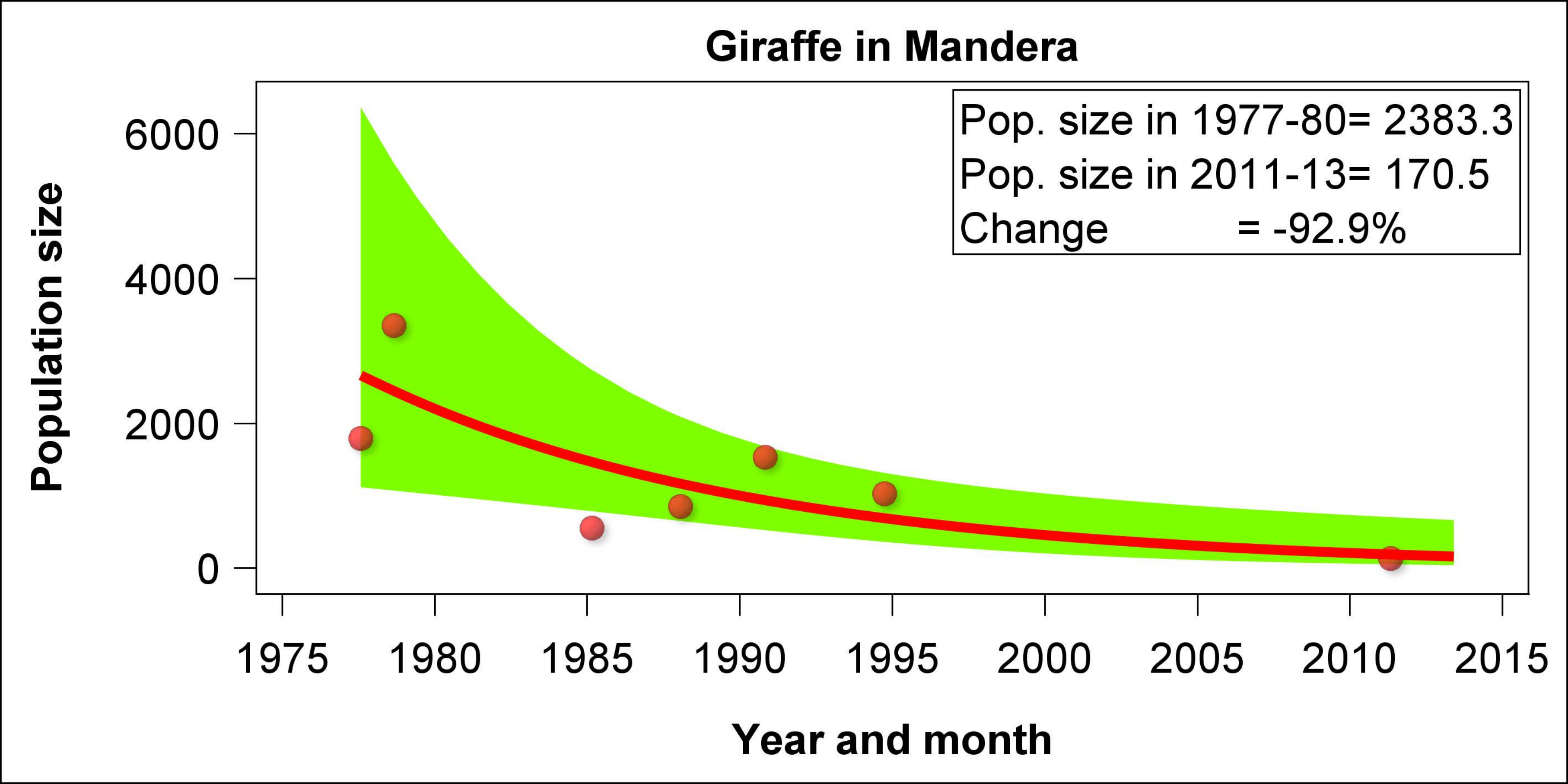

Giraffe in Mandera
Pop. size in 1977-80= 2383.3
Pop. size in 2011-13= 170.5
Change = -92.9%
Population size
6000
4000
2000
0
1975
1980
1985
1990
1995
2000
2005
2010
2015
Year and month

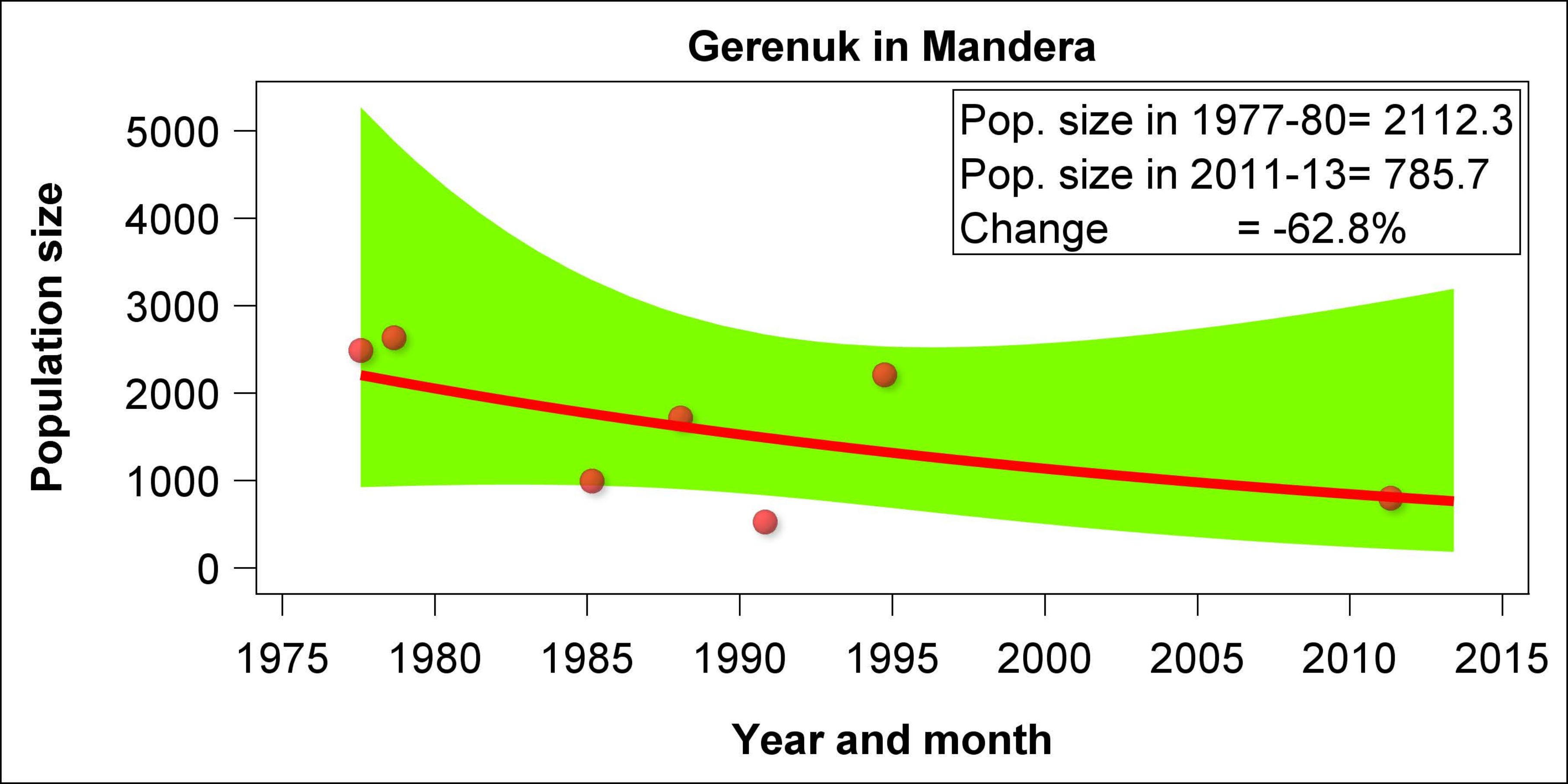

Gerenuk in Mandera
Pop. size in 1977-80= 2112.3
Pop. size in 2011-13= 785.7
Change = -62.8%
Population size
0
1000
2000
3000
4000
5000
1975
1980
1985
1990
1995
2000
2005
2010
2015
Year and month

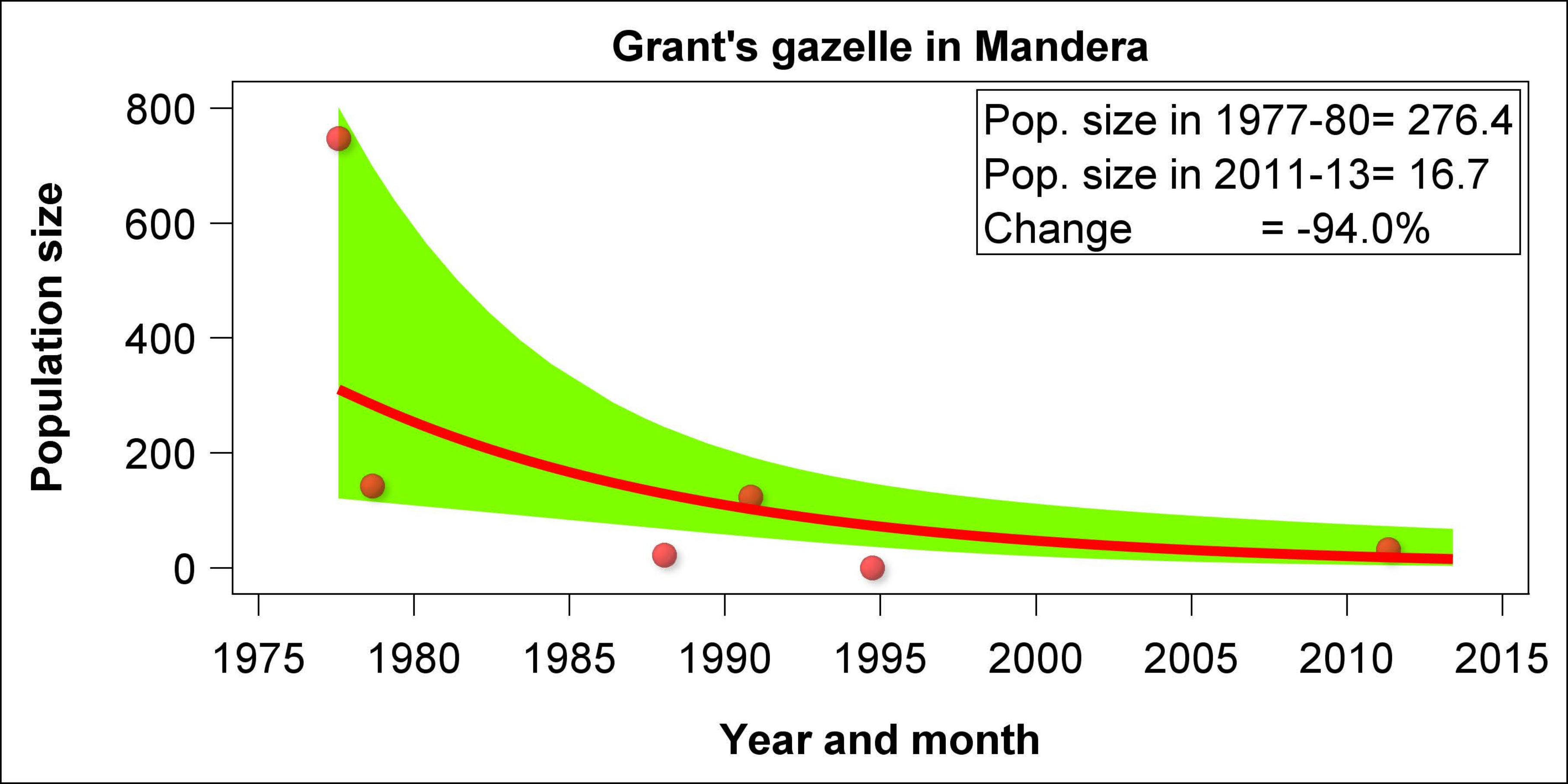

Grant's gazelle in Mandera
Pop. size in 1977-80= 276.4
Pop. size in 2011-13= 16.7
Change = -94.0%
800
600
400
200
0
Population size
1975
1980
1985
1990
1995
2000
2005
2010
2015
Year and month

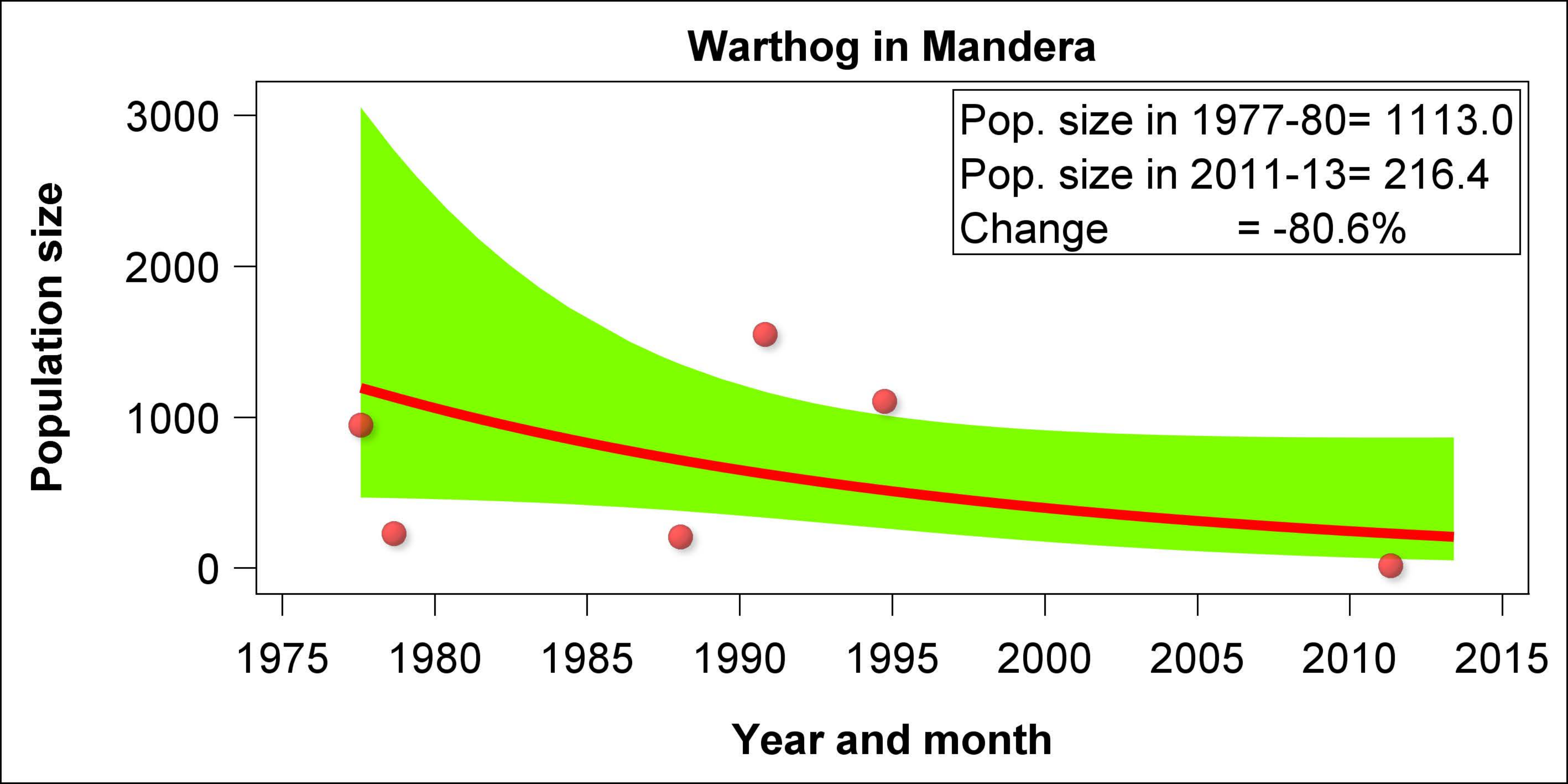

Warthog in Mandera
Pop. size in 1977-80= 1113.0
Pop. size in 2011-13= 216.4
Change = -80.6%
Population size
3000
2000
1000
0
1975
1980
1985
1990
1995
2000
2005
2010
2015
Year and month

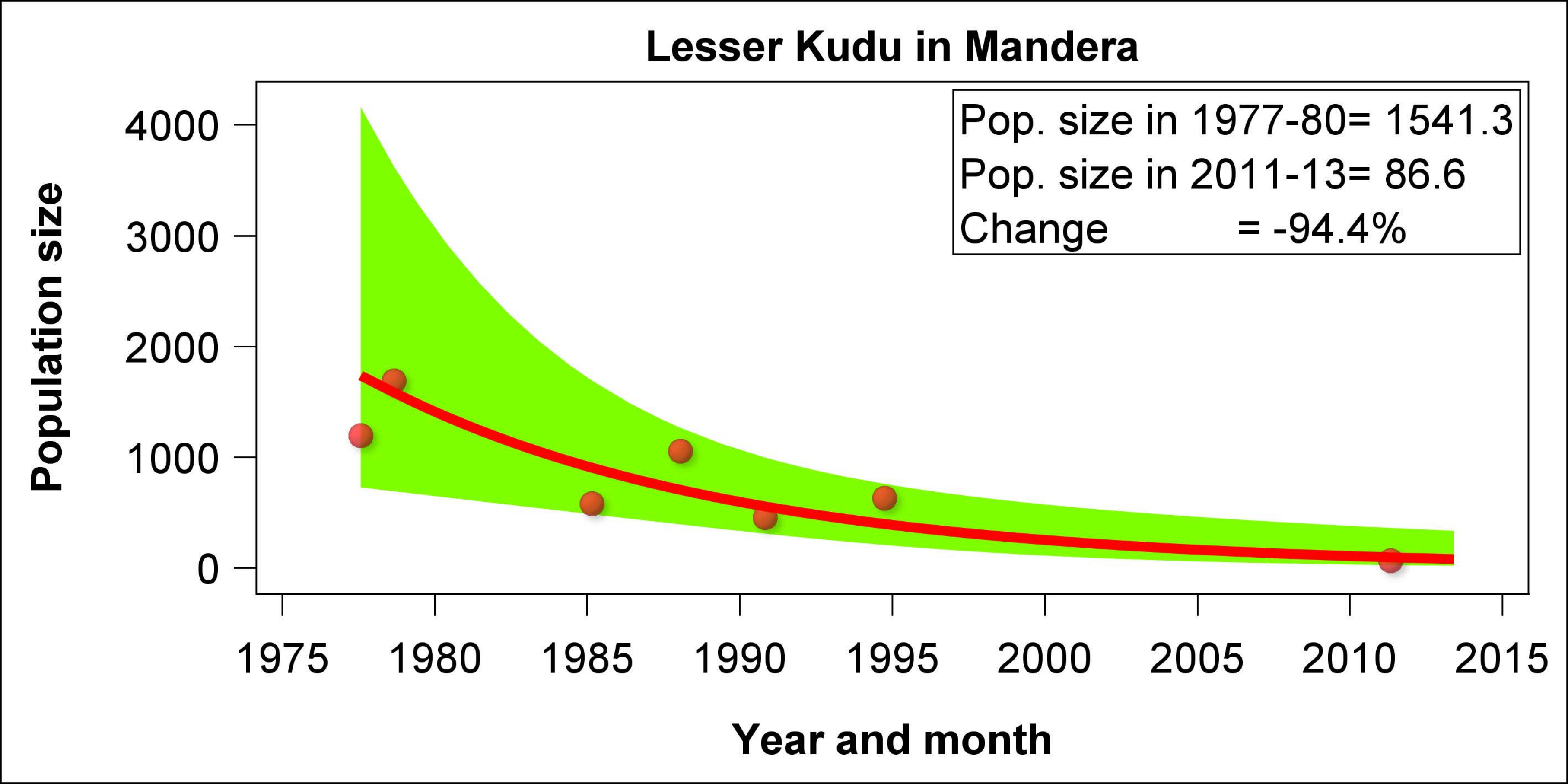

Lesser Kudu in Mandera
Pop. size in 1977-80= 1541.3
Pop. size in 2011-13= 86.6
Change = -94.4%
Population size
4000
3000
2000
1000
0
1975
1980
1985
1990
1995
2000
2005
2010
2015
Year and month

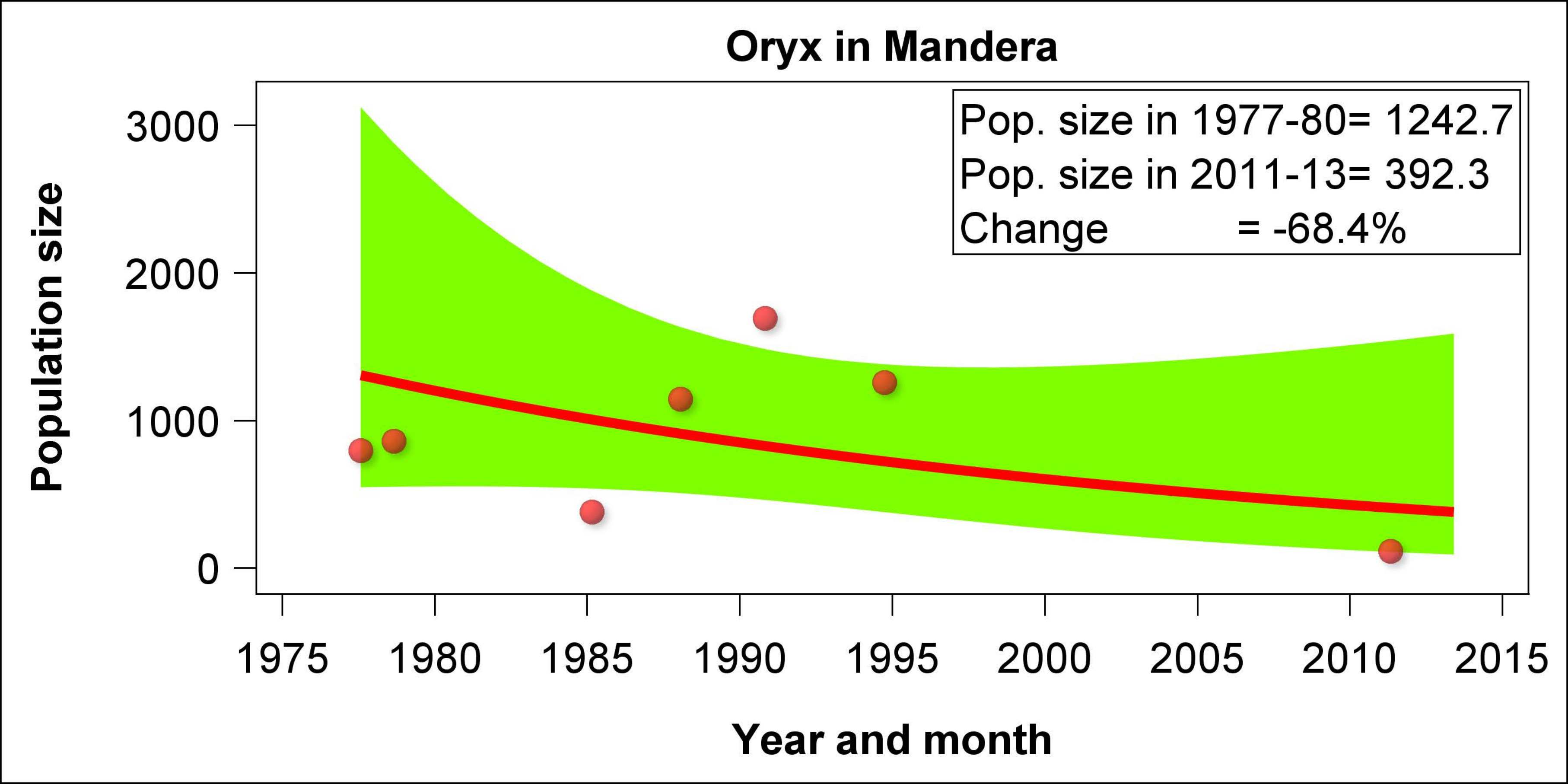

Oryx in Mandera
Pop. size in 1977-80= 1242.7
Pop. size in 2011-13= 392.3
Change = -68.4%
Population size
3000
2000
1000
0
1975
1980
1985
1990
1995
2000
2005
2010
2015
Year and month
