# Supplementary material for: Extreme Wildlife Declines and Concurrent Increase in Livestock Numbers in Kenya: What Are the Causes?
Source: PLoS One. 2016 Sep 27;11(9):e0163249. doi: 10.1371/journal.pone.0163249 (PMC5039022; doi:10.1371/journal.pone.0163249)
Supplement: S18 Fig — The solid red line is the fitted trend curve and the shaded chartreuse band is the pointwise 95% confidence band. The estimated average population size in 1977–1980 and 2011–2014 and the percentage change in population size between the two periods are provided in the inset. (PDF) [file pone.0163249.s028.pdf]

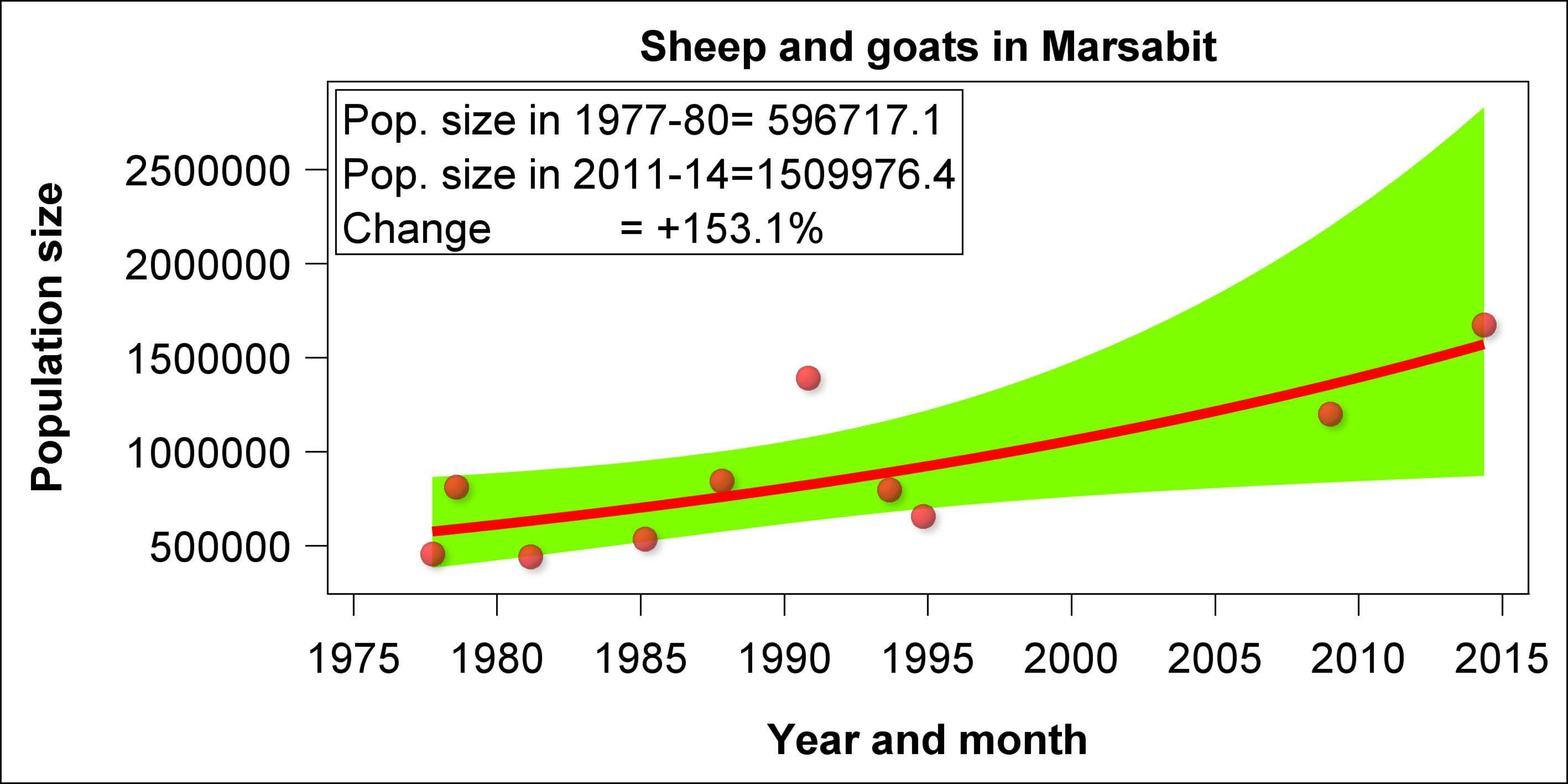
Sheep and goats in Marsabit
Pop. size in 1977-80= 596717.1
Pop. size in 2011-14=1509976.4
Change = +153.1%
Population size
2500000
2000000
1500000
1000000
500000
1975
1980
1985
1990
1995
2000
2005
2010
2015
Year and month

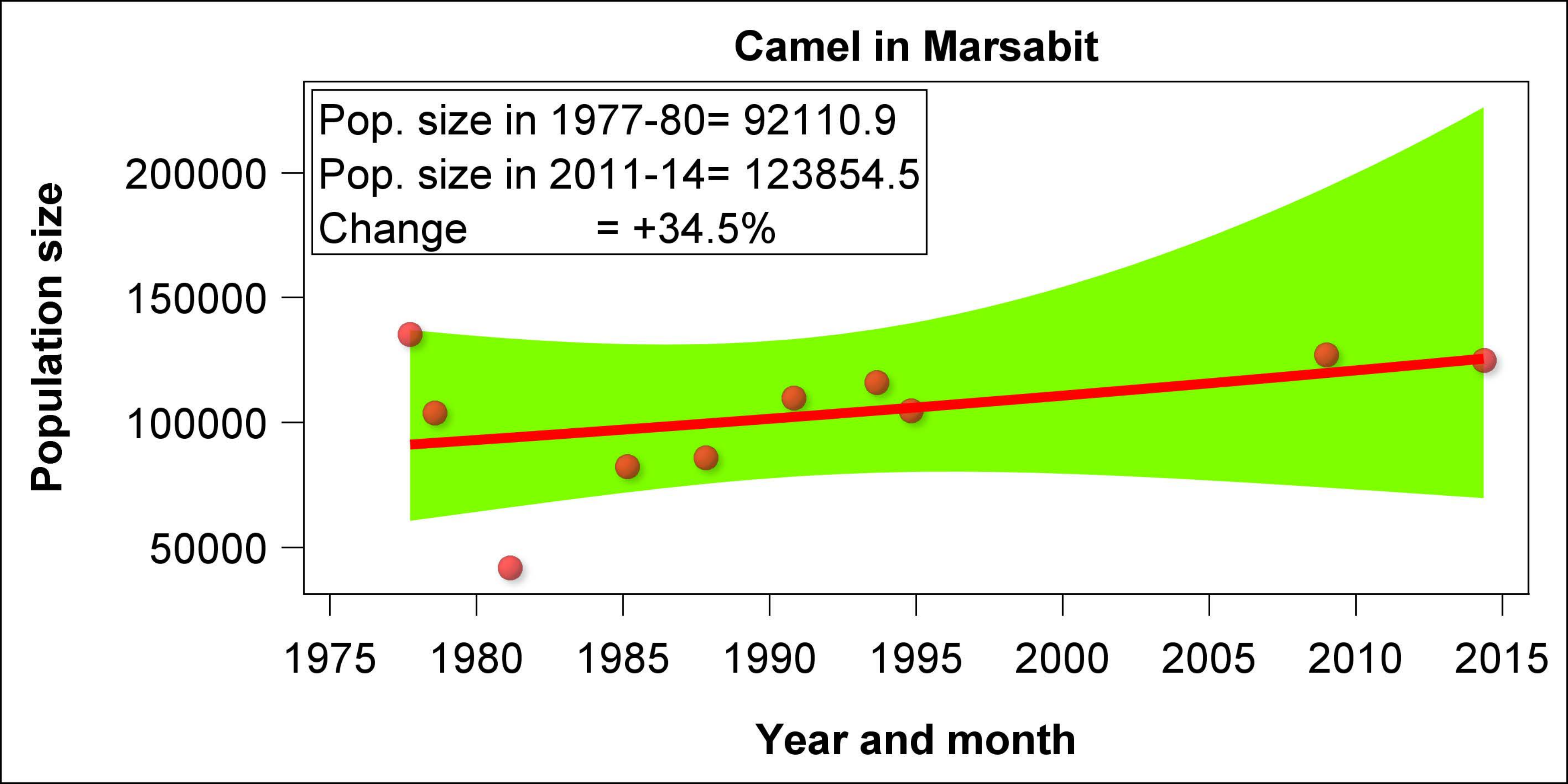

Camel in Marsabit
Pop. size in 1977-80= 92110.9
Pop. size in 2011-14= 123854.5
Change = +34.5%
Population size
200000
150000
100000
50000
1975
1980
1985
1990
1995
2000
2005
2010
2015
Year and month

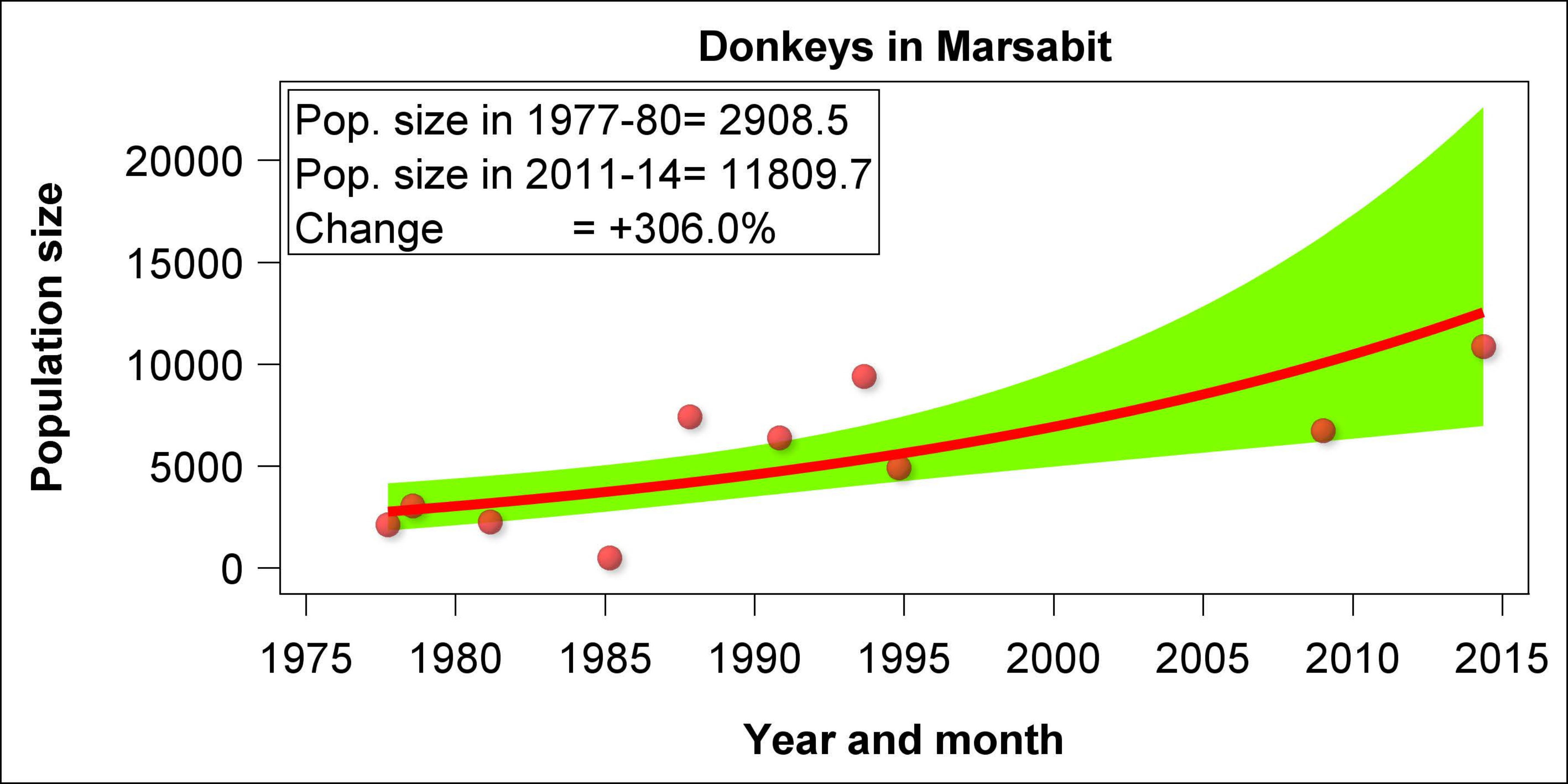

Donkeys in Marsabit
Pop. size in 1977-80= 2908.5
Pop. size in 2011-14= 11809.7
Change = +306.0%
Population size
20000
15000
10000
5000
0
1975
1980
1985
1990
1995
2000
2005
2010
2015
Year and month

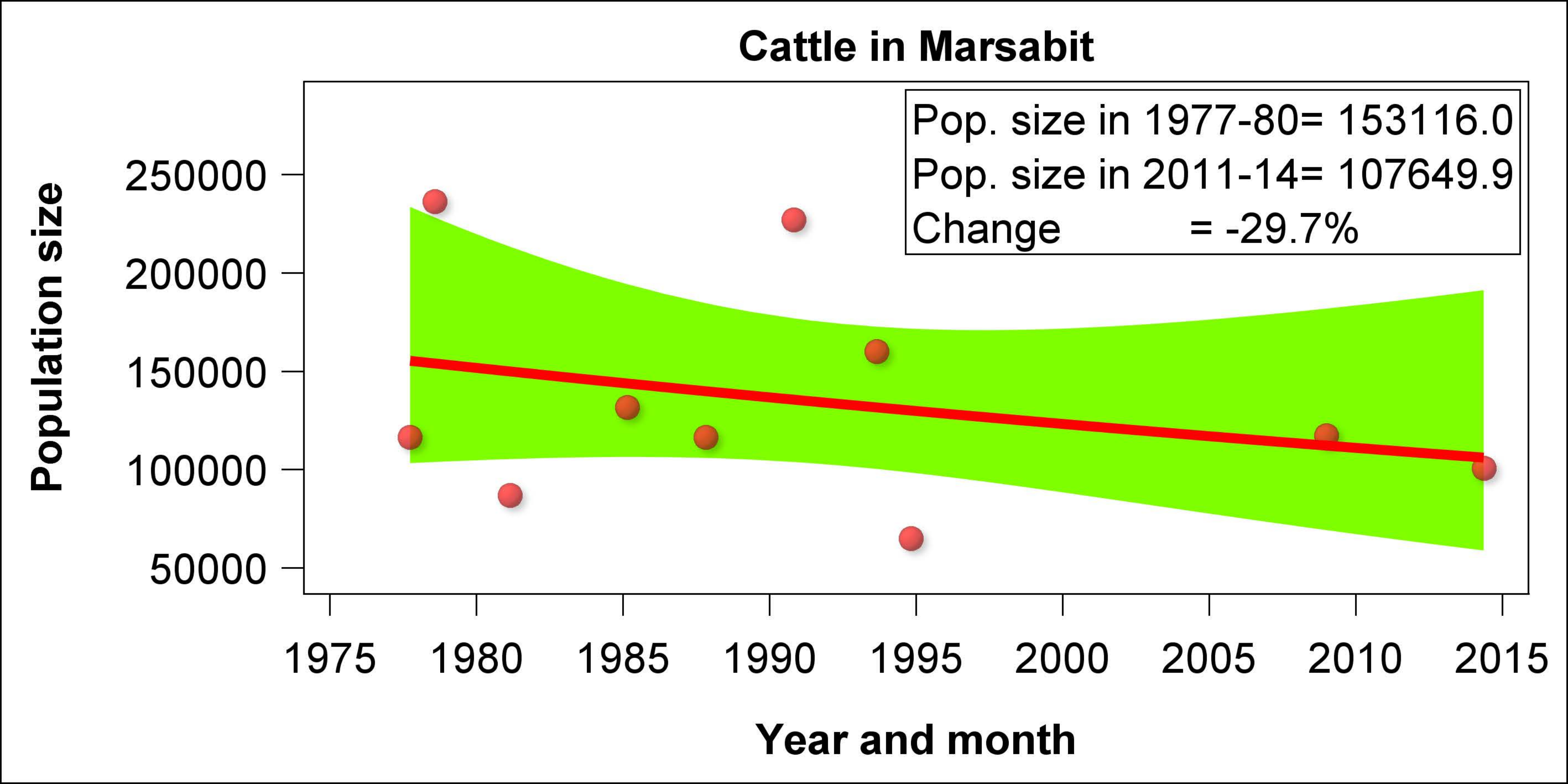

Cattle in Marsabit
Pop. size in 1977-80= 153116.0
Pop. size in 2011-14= 107649.9
Change = -29.7%
Population size
250000
200000
150000
100000
50000
1975
1980
1985
1990
1995
2000
2005
2010
2015
Year and month

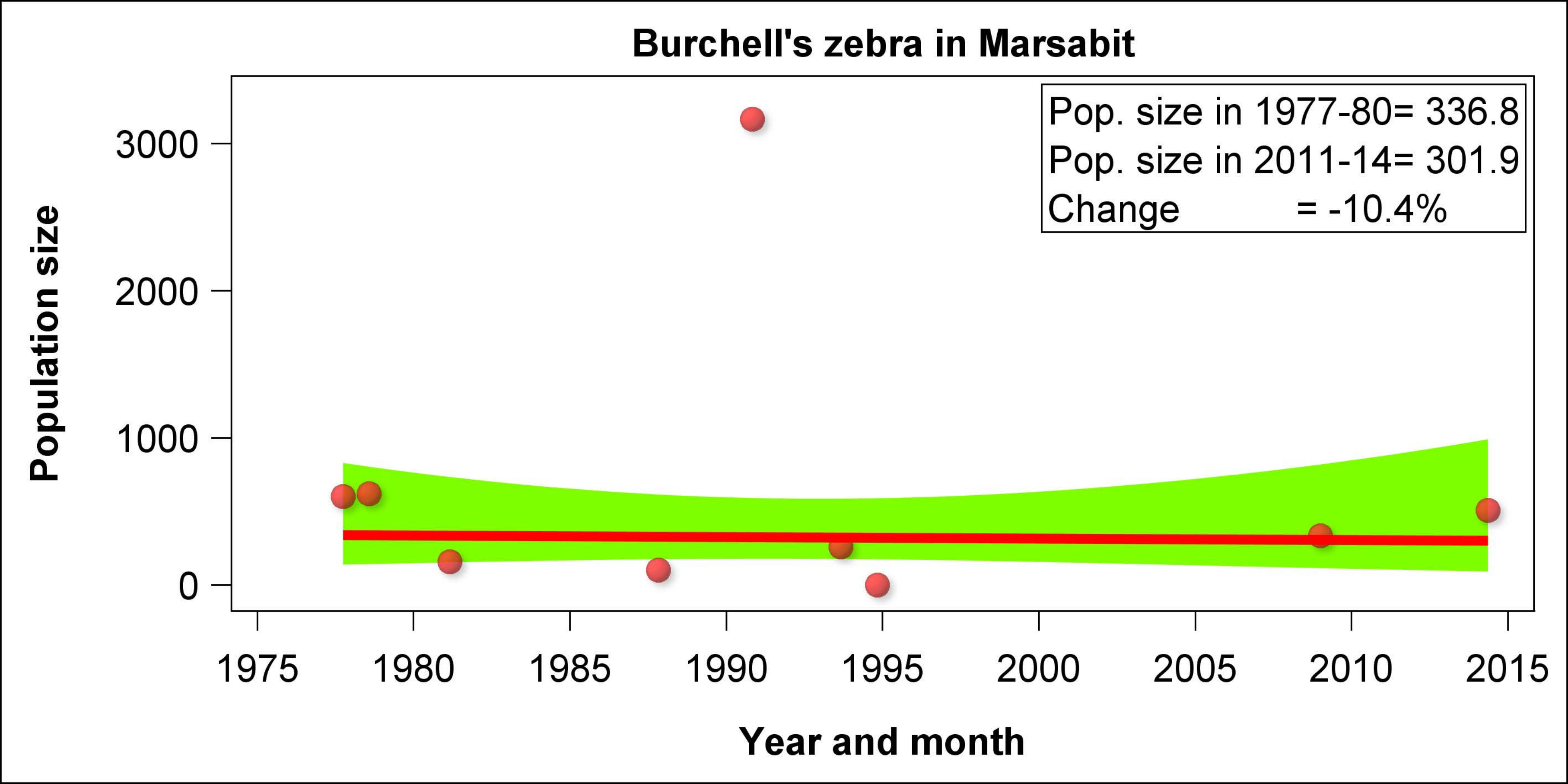
Burchell's zebra in Marsabit
Pop. size in 1977-80= 336.8
Pop. size in 2011-14= 301.9
Change = -10.4%
Population size
3000
2000
1000
0
1975
1980
1985
1990
1995
2000
2005
2010
2015
Year and month

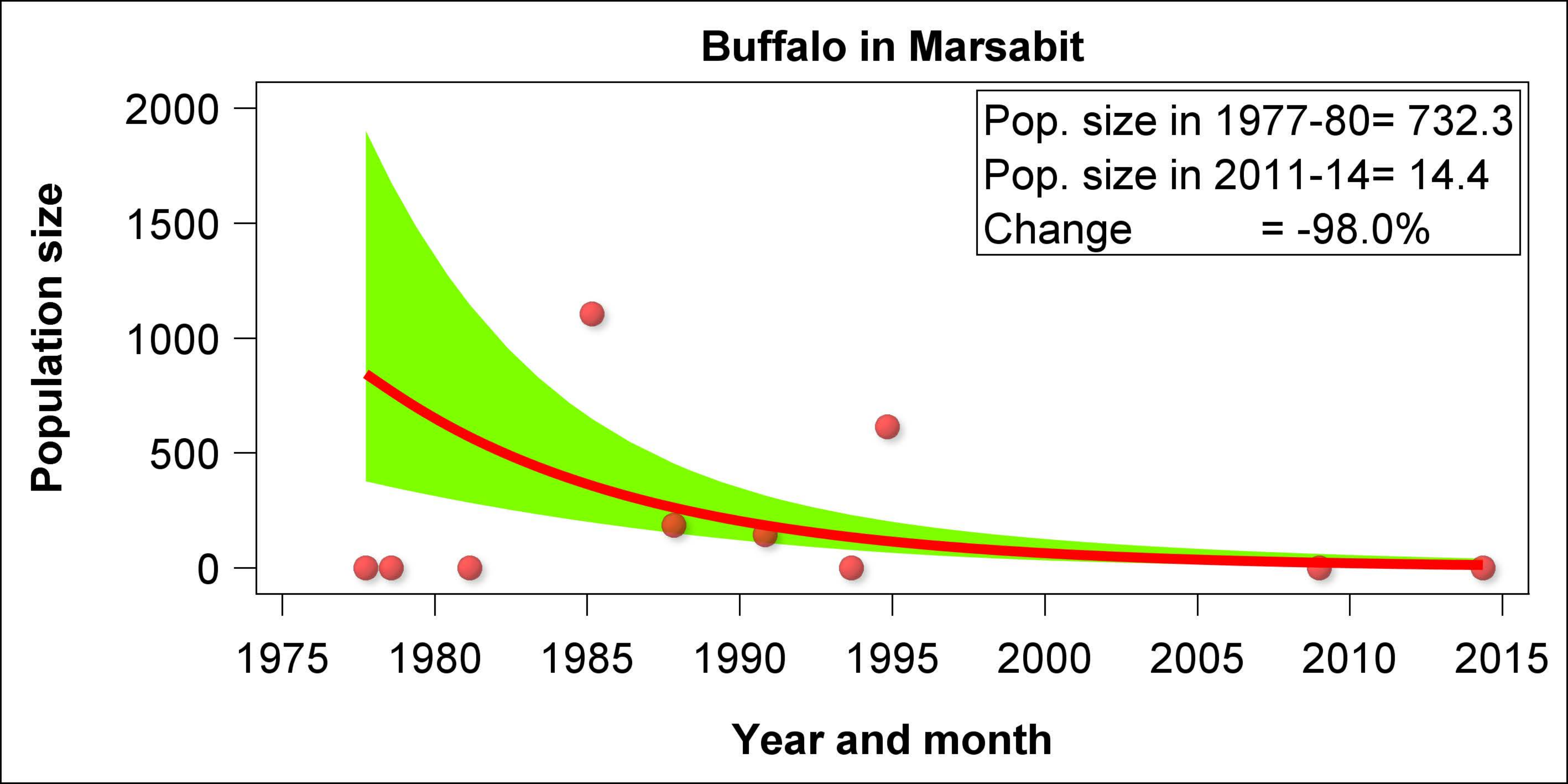

Buffalo in Marsabit
Pop. size in 1977-80= 732.3
Pop. size in 2011-14= 14.4
Change = -98.0%
Population size
2000
1500
1000
500
0
1975
1980
1985
1990
1995
2000
2005
2010
2015
Year and month

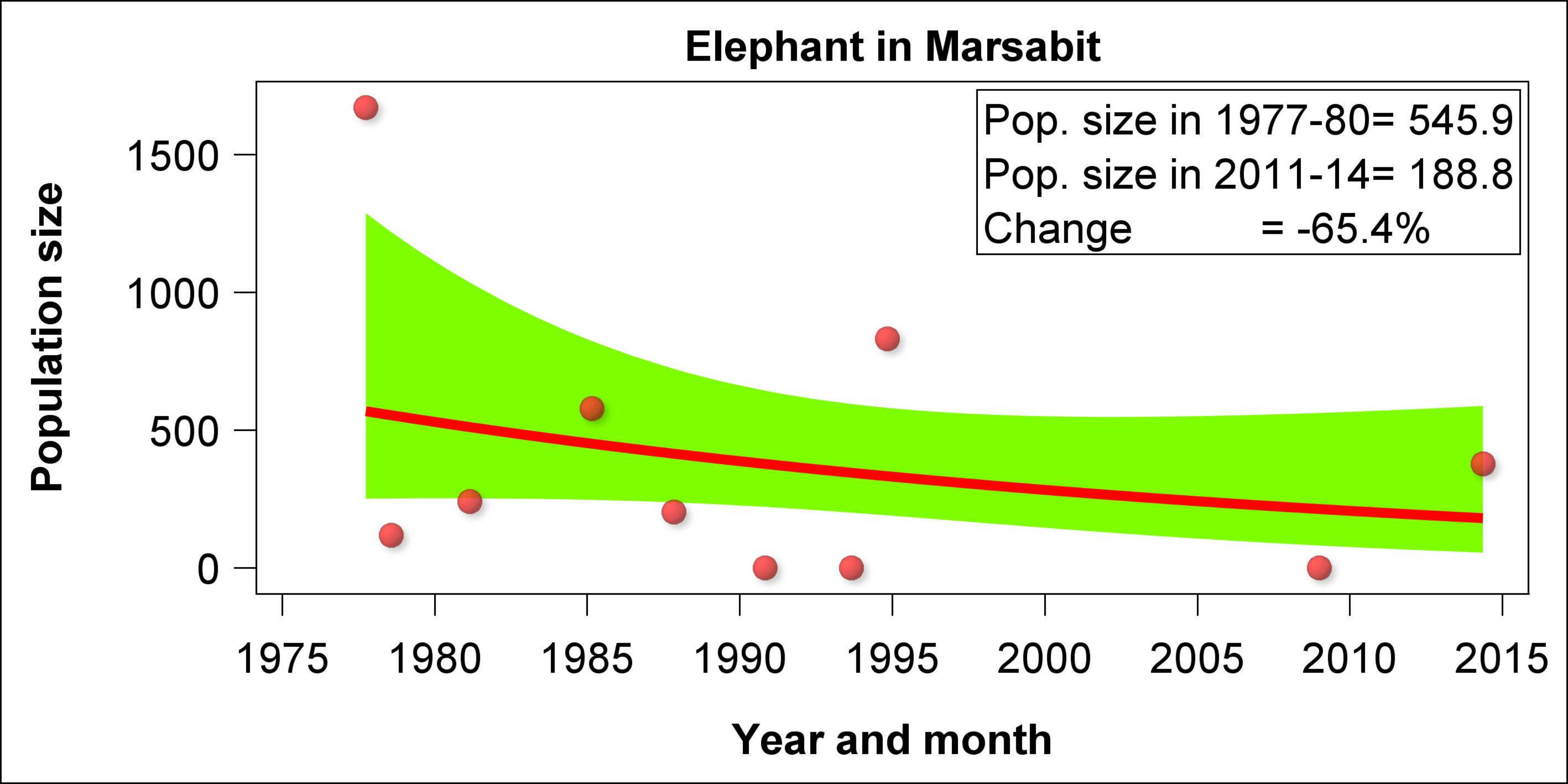

Elephant in Marsabit
Pop. size in 1977-80= 545.9
Pop. size in 2011-14= 188.8
Change = -65.4%
Population size
1500
1000
500
0
1975
1980
1985
1990
1995
2000
2005
2010
2015
Year and month

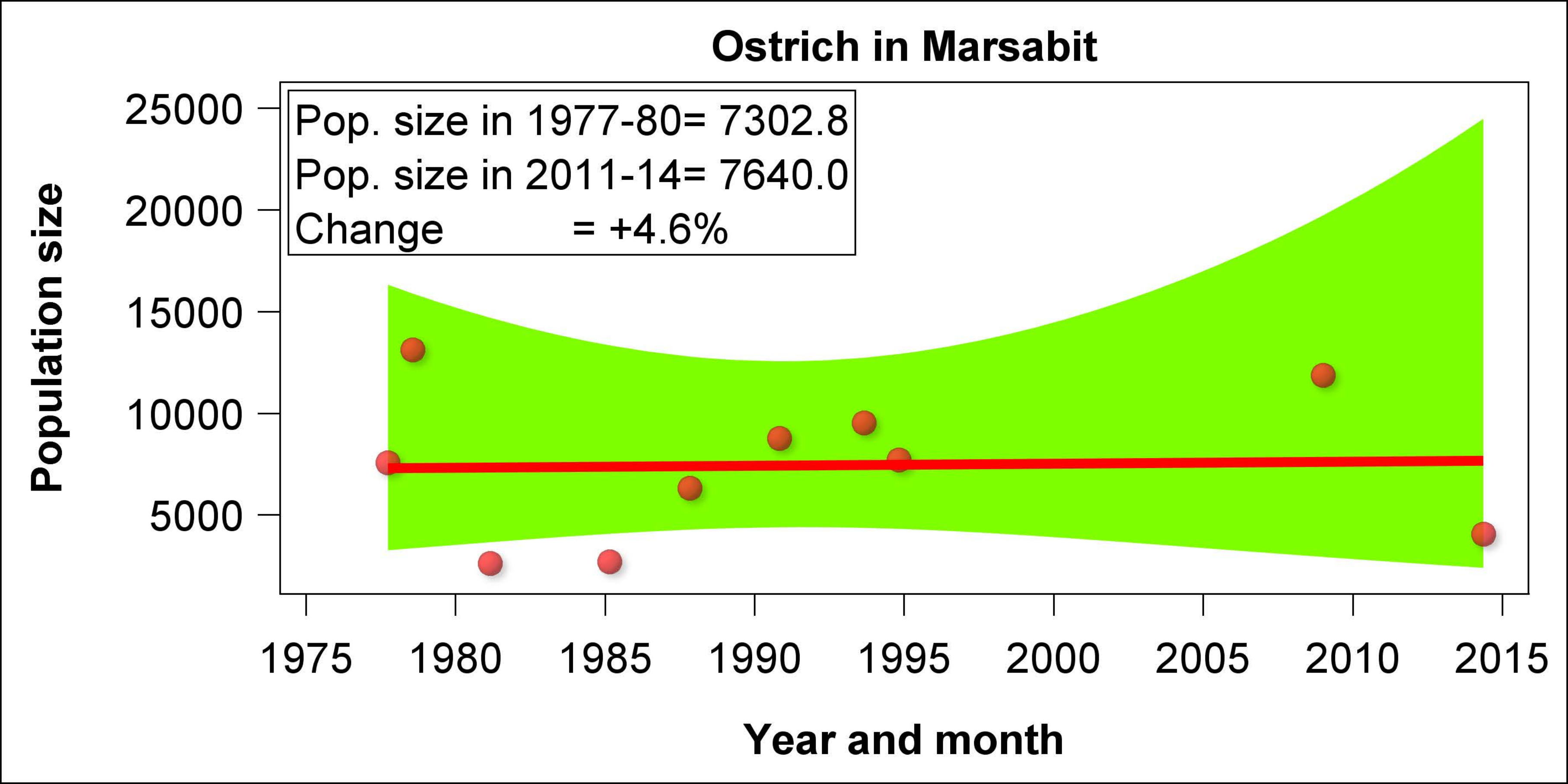
Ostrich in Marsabit
Pop. size in 1977-80= 7302.8
Pop. size in 2011-14= 7640.0
Change = +4.6%
Population size
25000
20000
15000
10000
5000
1975
1980
1985
1990
1995
2000
2005
2010
2015
Year and month

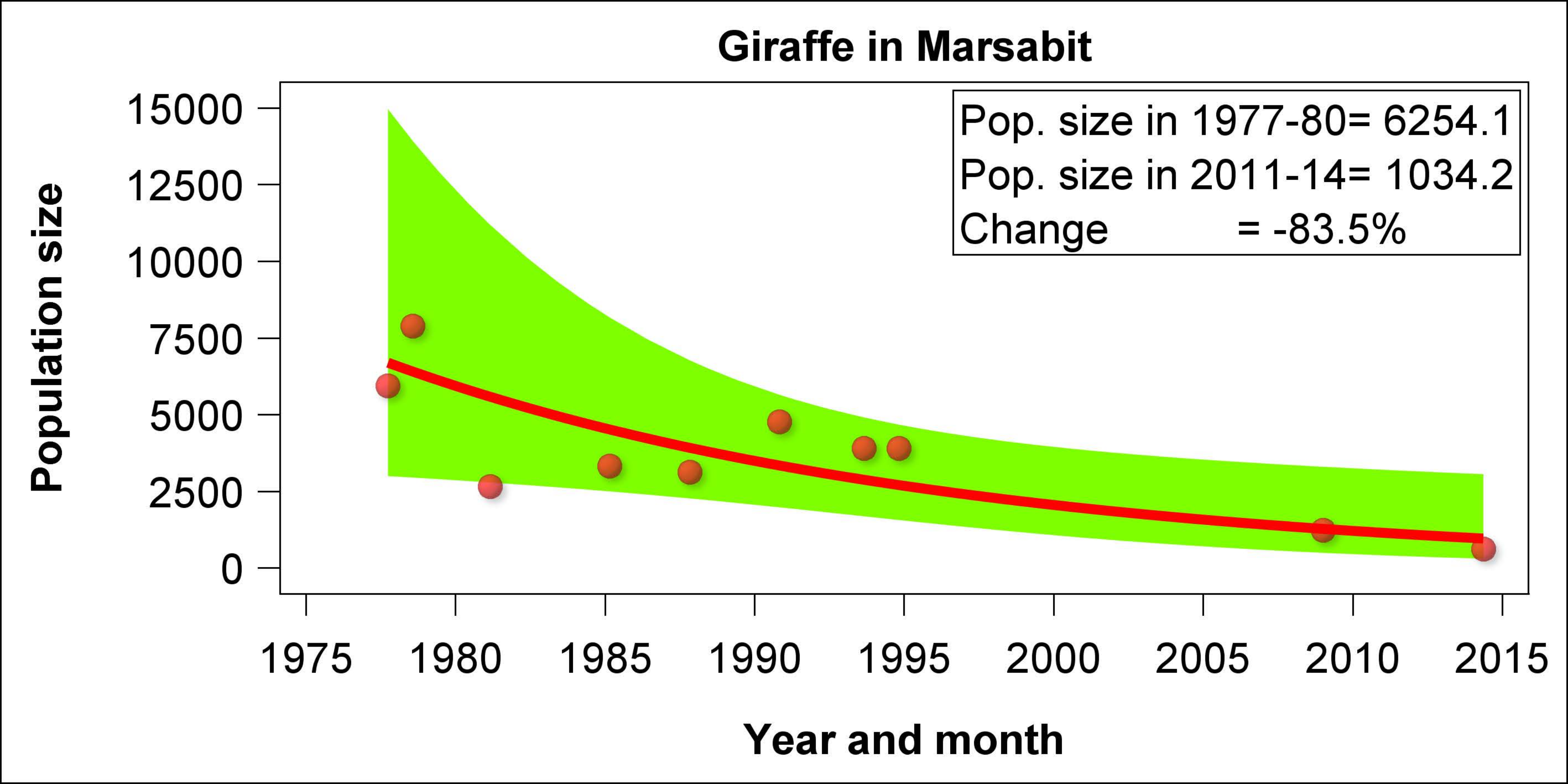

Giraffe in Marsabit
Pop. size in 1977-80= 6254.1
Pop. size in 2011-14= 1034.2
Change = -83.5%
Population size
15000
12500
10000
7500
5000
2500
0
1975
1980
1985
1990
1995
2000
2005
2010
2015
Year and month

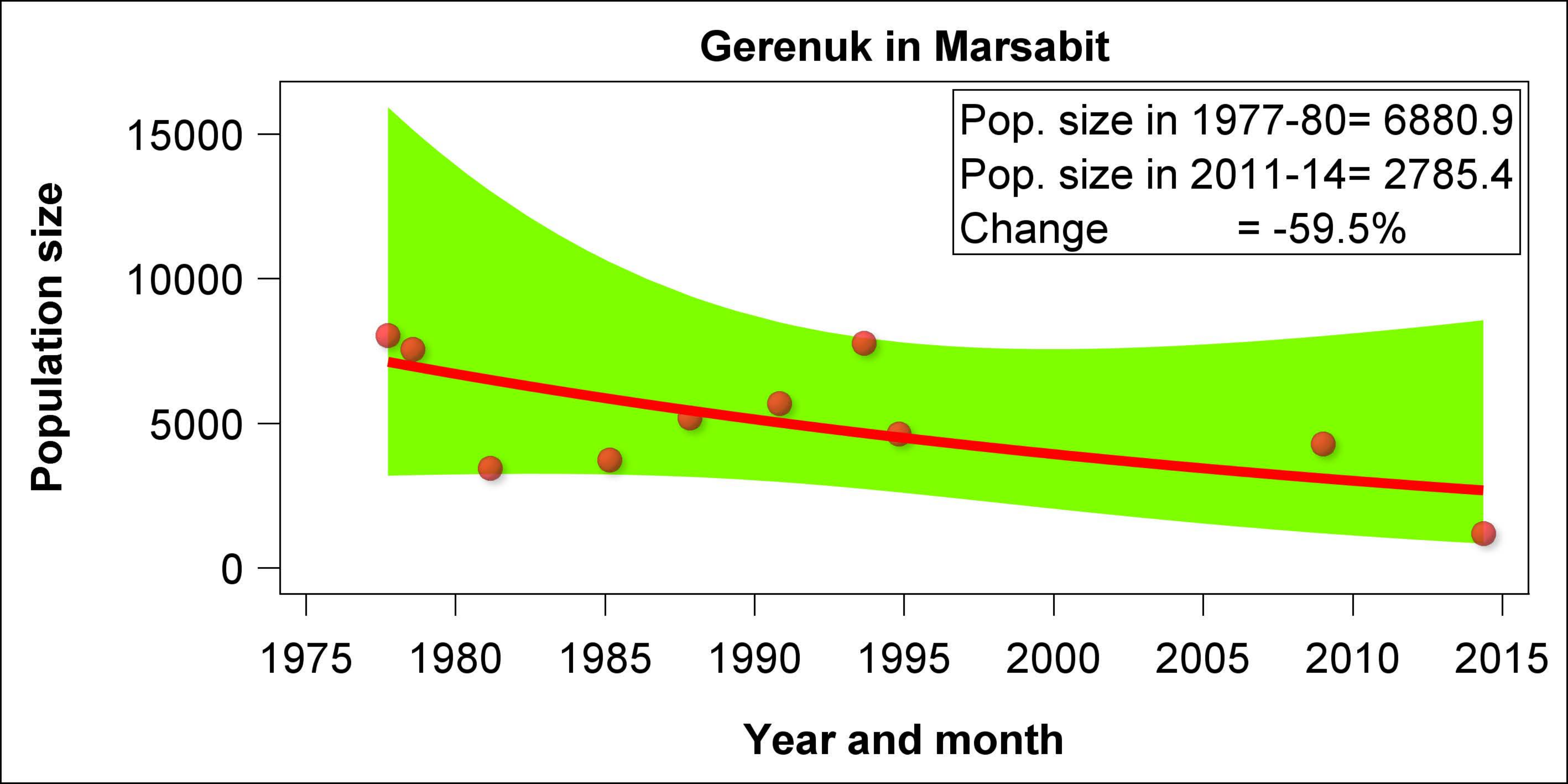
Gerenuk in Marsabit
Pop. size in 1977-80= 6880.9
Pop. size in 2011-14= 2785.4
Change = -59.5%
Population size
15000
10000
5000
0
1975
1980
1985
1990
1995
2000
2005
2010
2015
Year and month

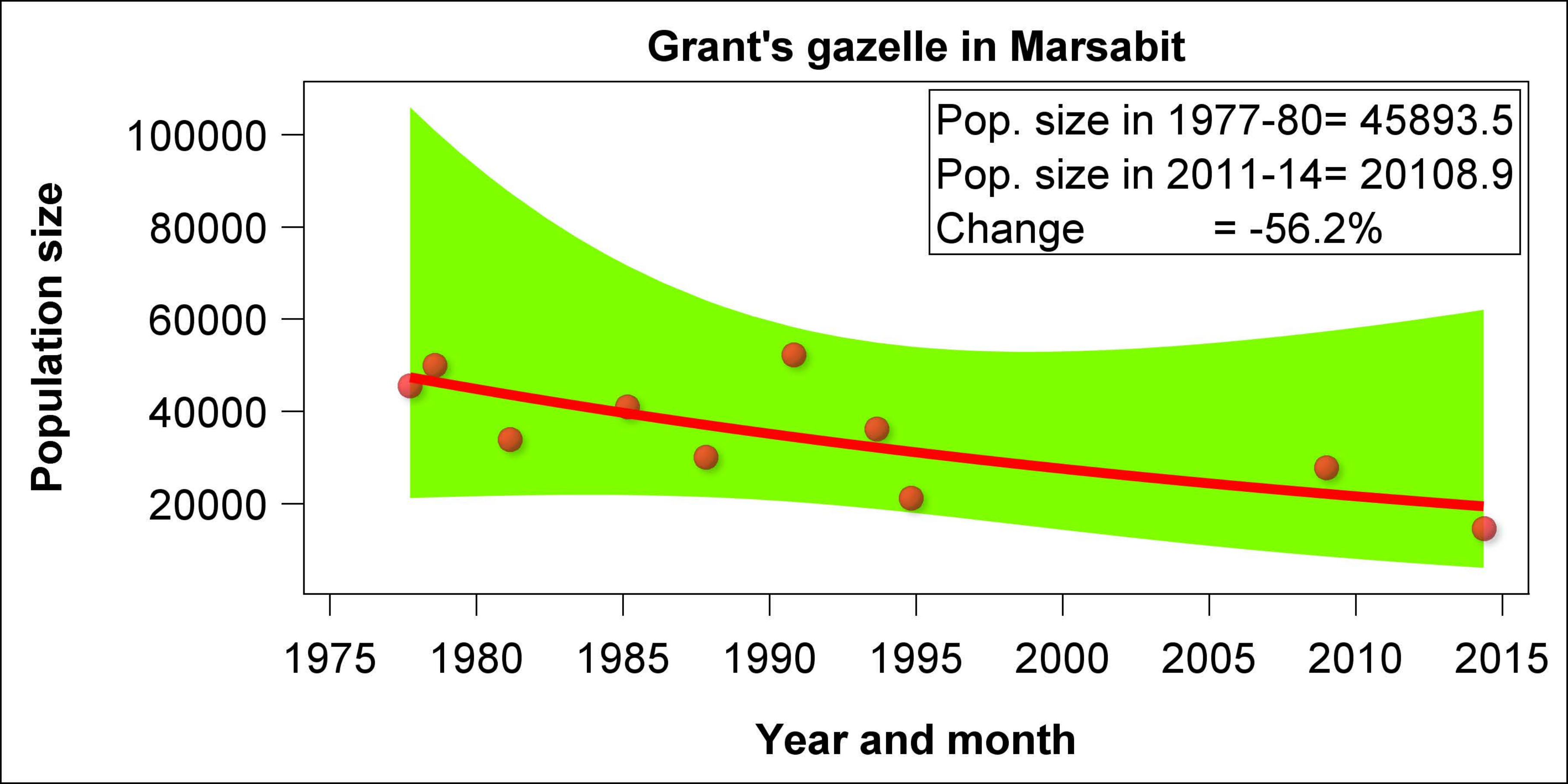
Grant's gazelle in Marsabit
Pop. size in 1977-80= 45893.5
Pop. size in 2011-14= 20108.9
Change = -56.2%
100000
80000
60000
40000
20000
Population size
1975
1980
1985
1990
1995
2000
2005
2010
2015
Year and month

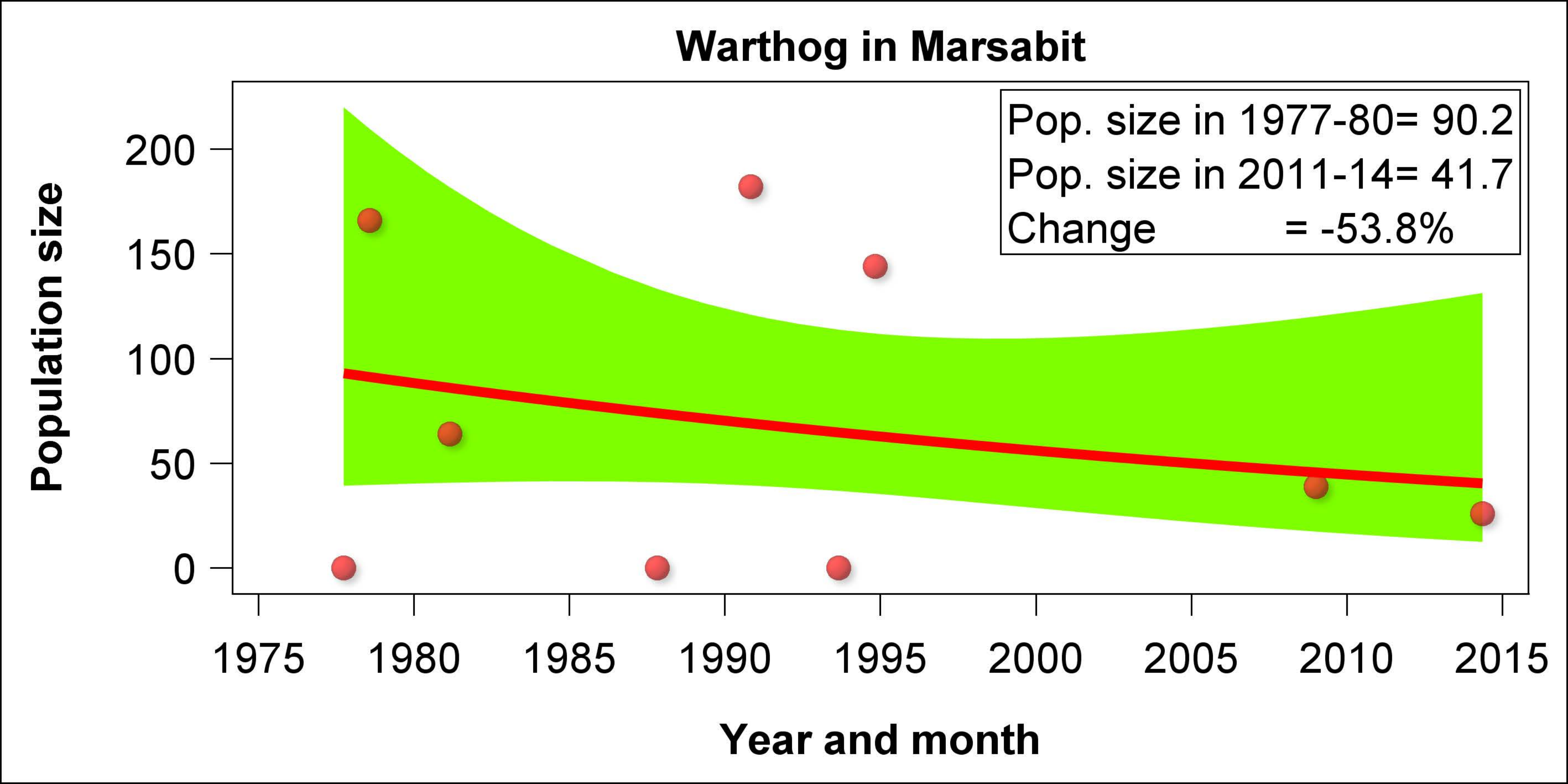
Warthog in Marsabit
Pop. size in 1977-80= 90.2
Pop. size in 2011-14= 41.7
Change = -53.8%
Population size
200
150
100
50
0
1975
1980
1985
1990
1995
2000
2005
2010
2015
Year and month

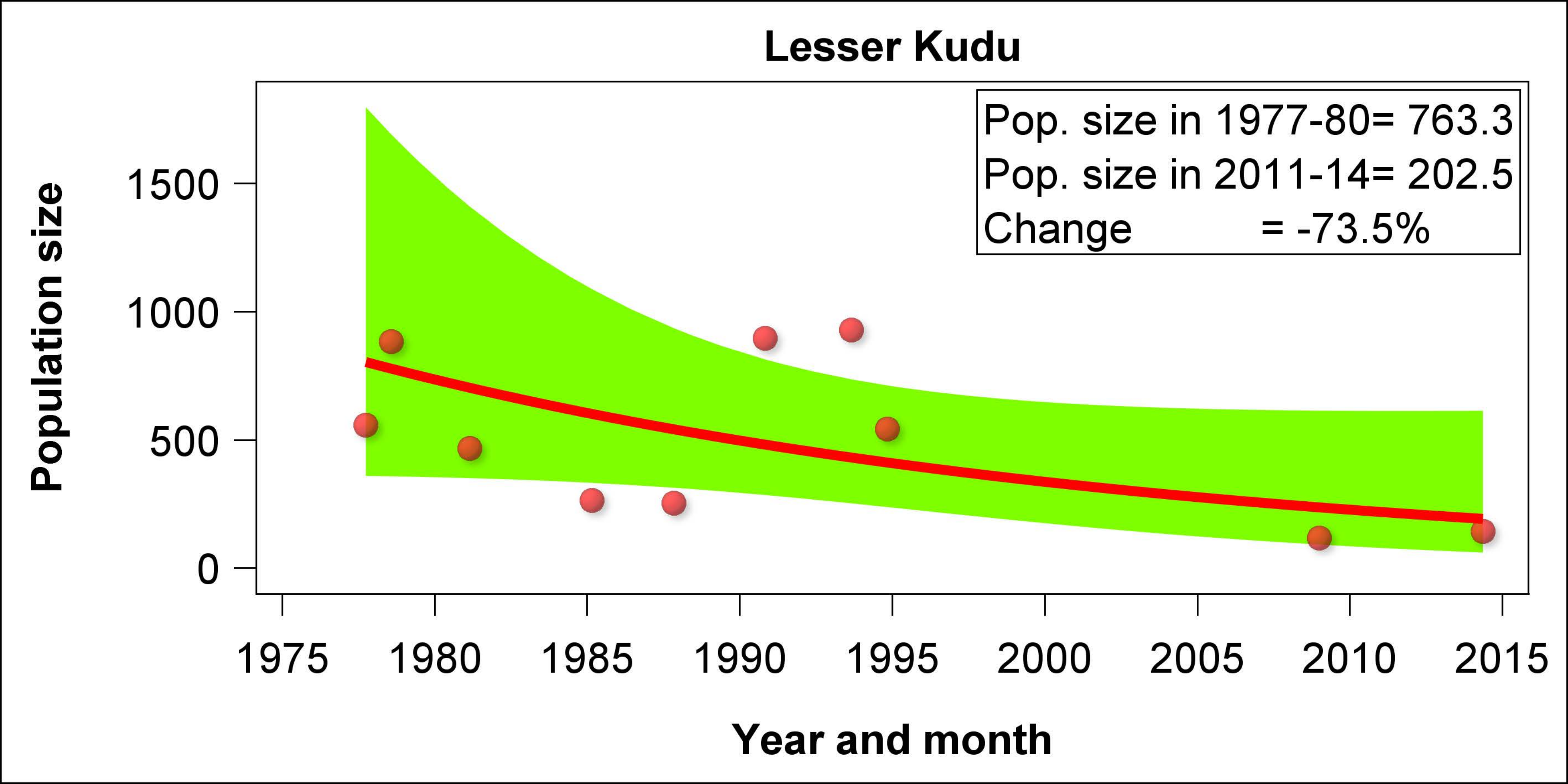

Lesser Kudu
Pop. size in 1977-80= 763.3
Pop. size in 2011-14= 202.5
Change = -73.5%
Population size
1500
1000
500
0
1975
1980
1985
1990
1995
2000
2005
2010
2015
Year and month

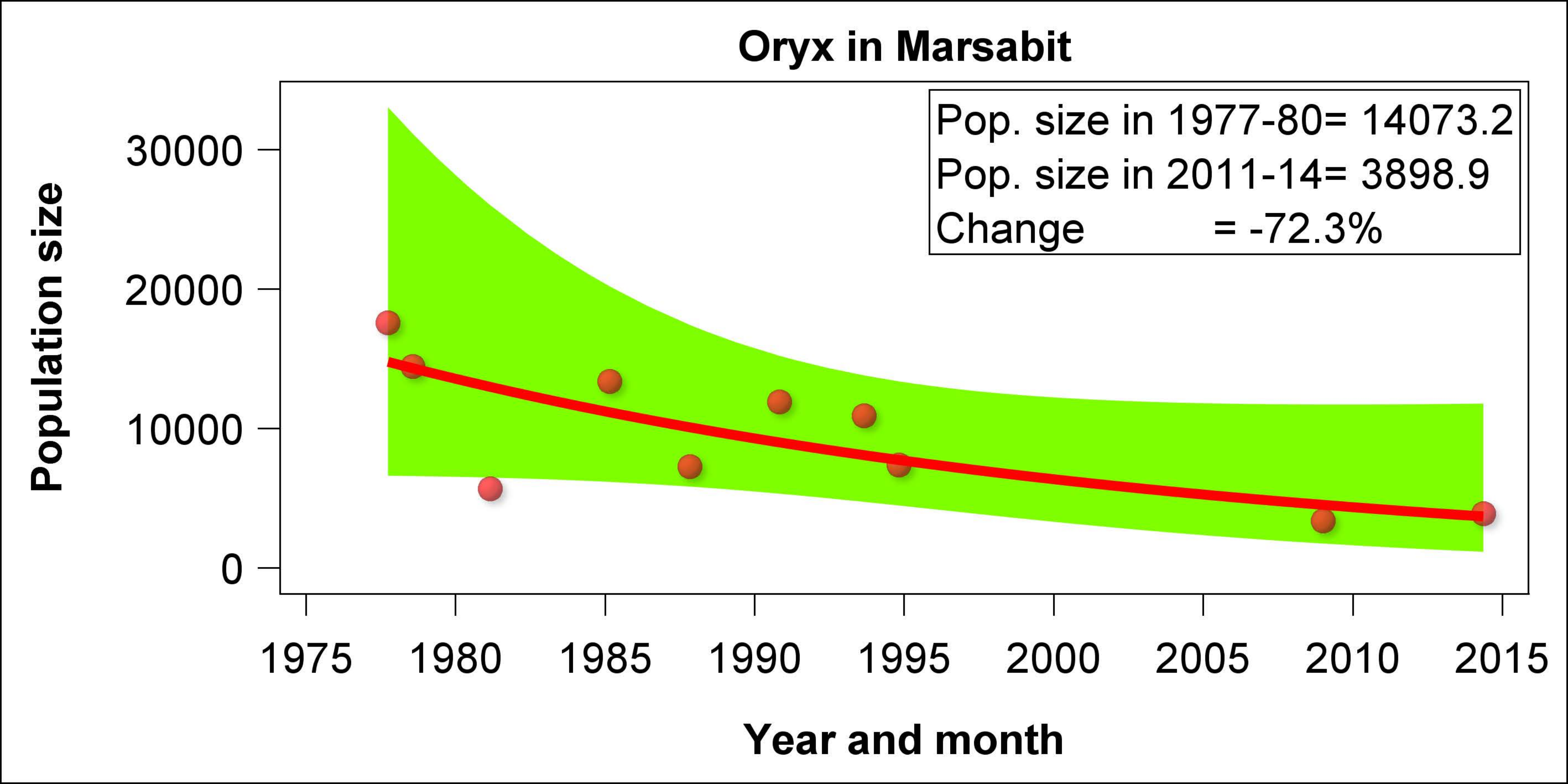
Oryx in Marsabit
Pop. size in 1977-80= 14073.2
Pop. size in 2011-14= 3898.9
Change = -72.3%
Population size
30000
20000
10000
0
1975
1980
1985
1990
1995
2000
2005
2010
2015
Year and month

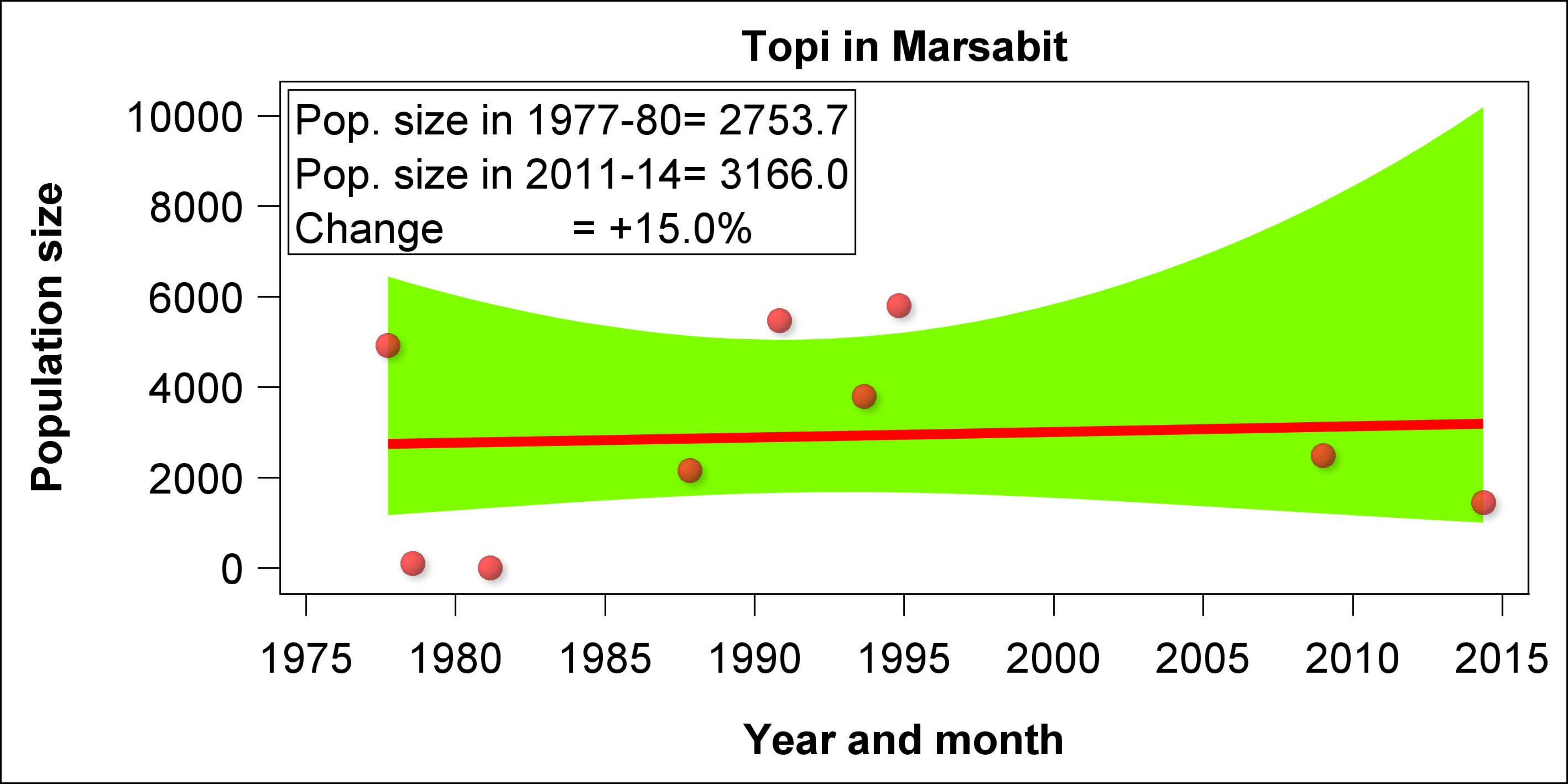
Topi in Marsabit
Pop. size in 1977-80= 2753.7
Pop. size in 2011-14= 3166.0
Change = +15.0%
Population size
10000
8000
6000
4000
2000
0
1975
1980
1985
1990
1995
2000
2005
2010
2015
Year and month

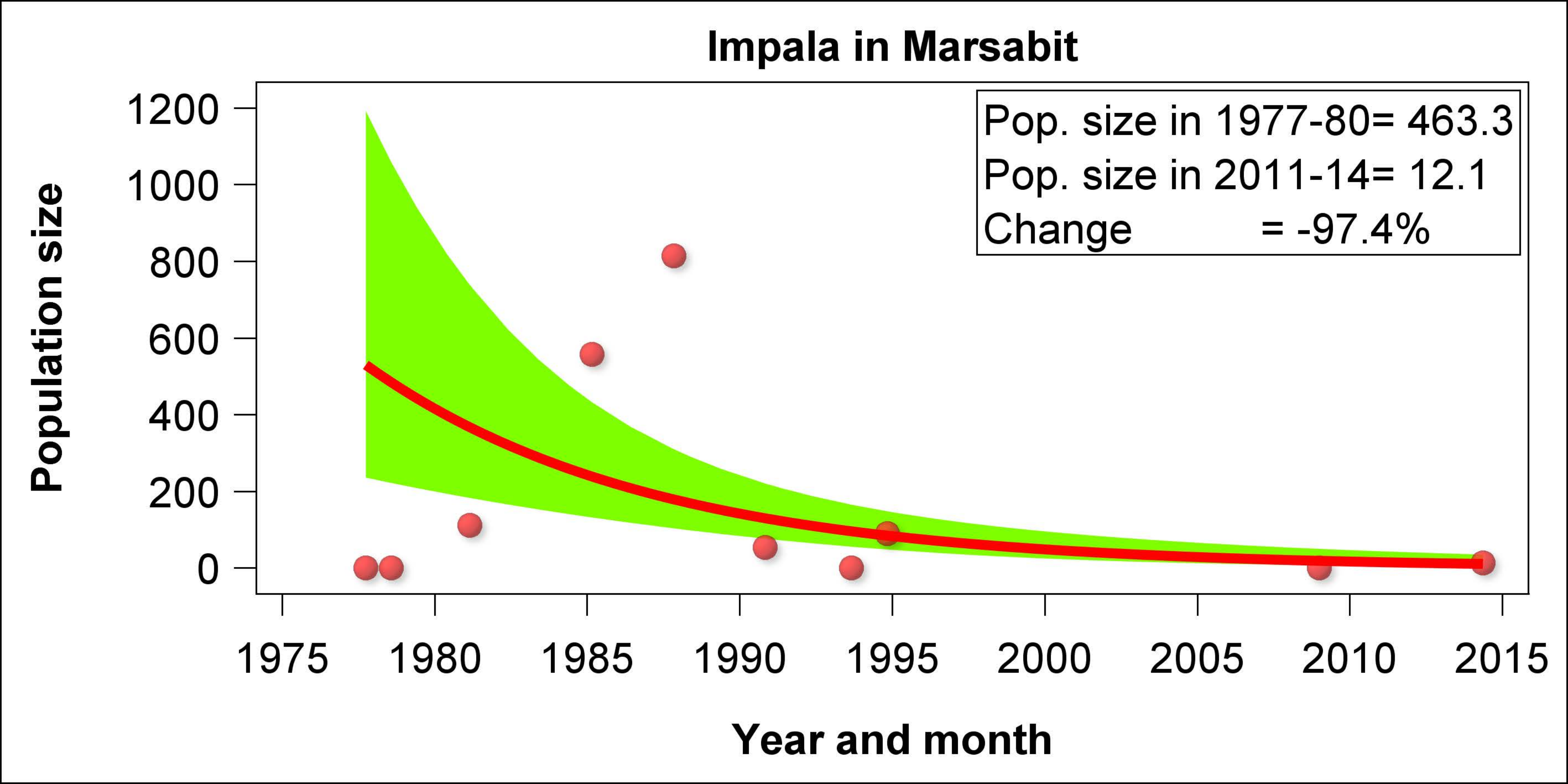
Impala in Marsabit
Pop. size in 1977-80= 463.3
Pop. size in 2011-14= 12.1
Change = -97.4%
Population size
1200
1000
800
600
400
200
0
1975
1980
1985
1990
1995
2000
2005
2010
2015
Year and month

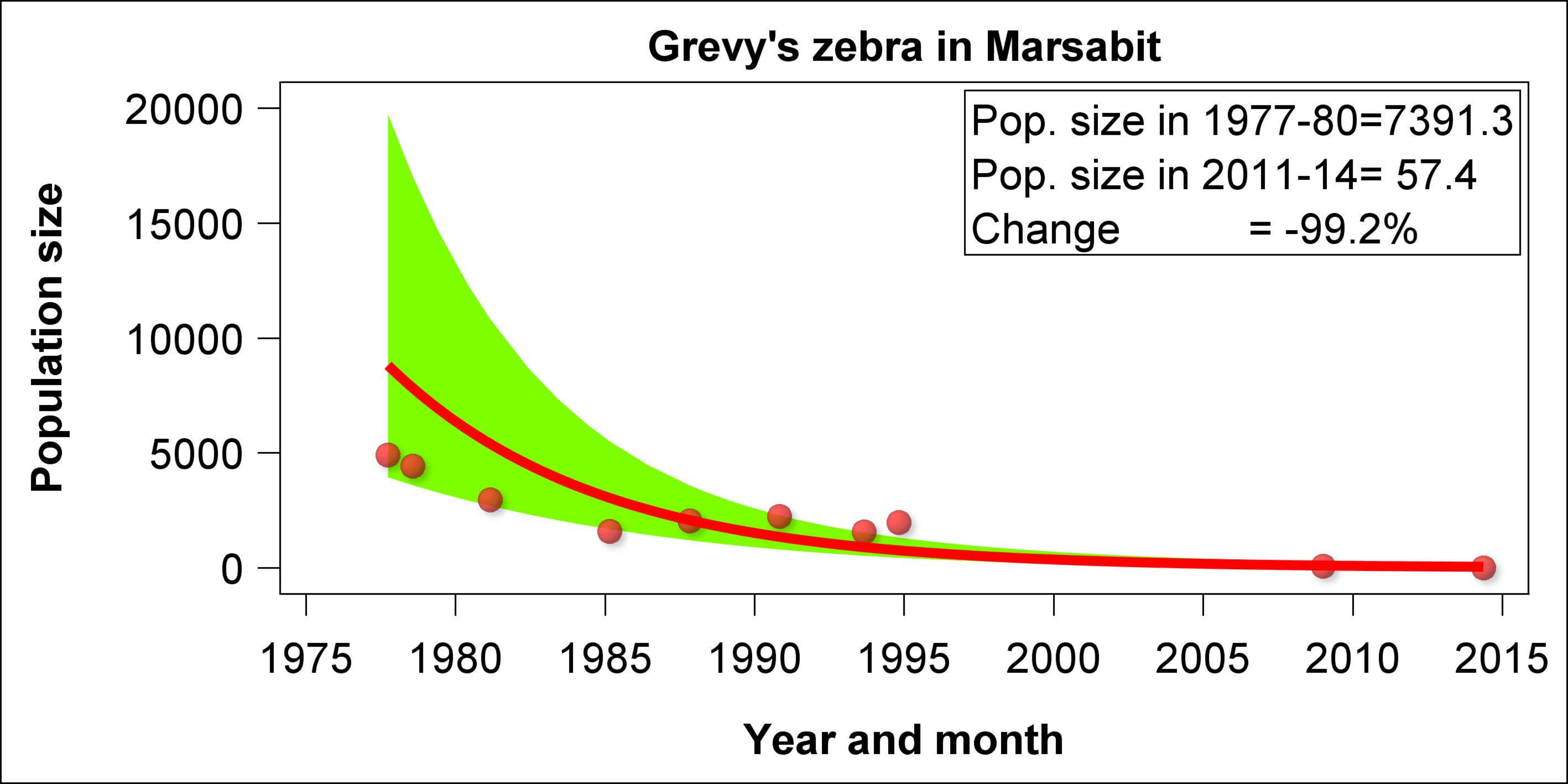
Grevy's zebra in Marsabit
Pop. size in 1977-80=7391.3
Pop. size in 2011-14= 57.4
Change = -99.2%
Population size
20000
15000
10000
5000
0
1975
1980
1985
1990
1995
2000
2005
2010
2015
Year and month
