# Supplementary material for: Extreme Wildlife Declines and Concurrent Increase in Livestock Numbers in Kenya: What Are the Causes?
Source: PLoS One. 2016 Sep 27;11(9):e0163249. doi: 10.1371/journal.pone.0163249 (PMC5039022; doi:10.1371/journal.pone.0163249)
Supplement: S19 Fig — The solid red line is the fitted trend curve and the shaded chartreuse band is the pointwise 95% confidence band. The estimated average population size in 1977–1980 and 2011–2013 and the percentage change in population size between the two periods are provided in the inset. (PDF) [file pone.0163249.s029.pdf]

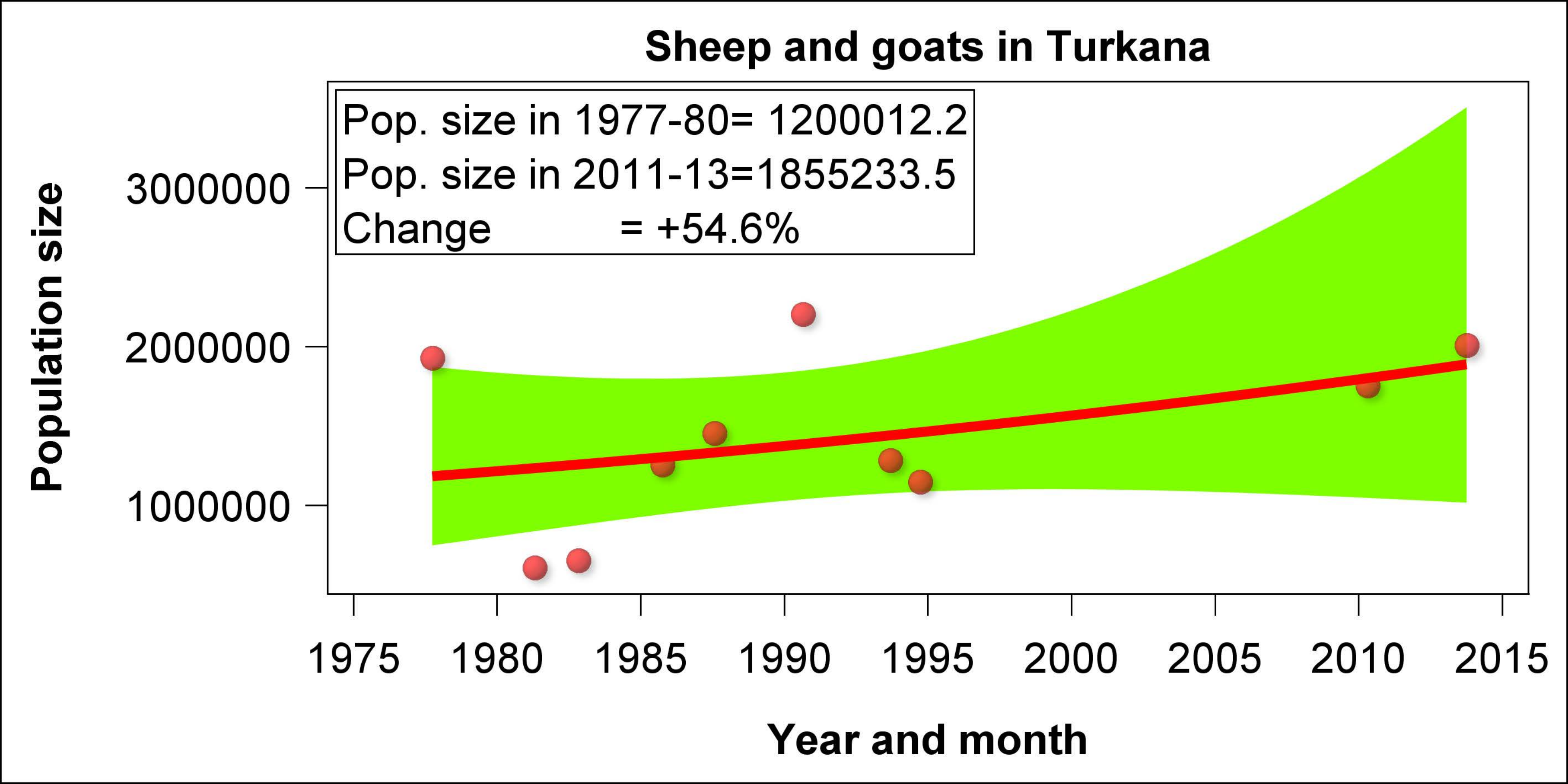
Sheep and goats in Turkana
Pop. size in 1977-80= 1200012.2
Pop. size in 2011-13=1855233.5
Change = +54.6%
3000000
2000000
1000000
Population size
1975
1980
1985
1990
1995
2000
2005
2010
2015
Year and month

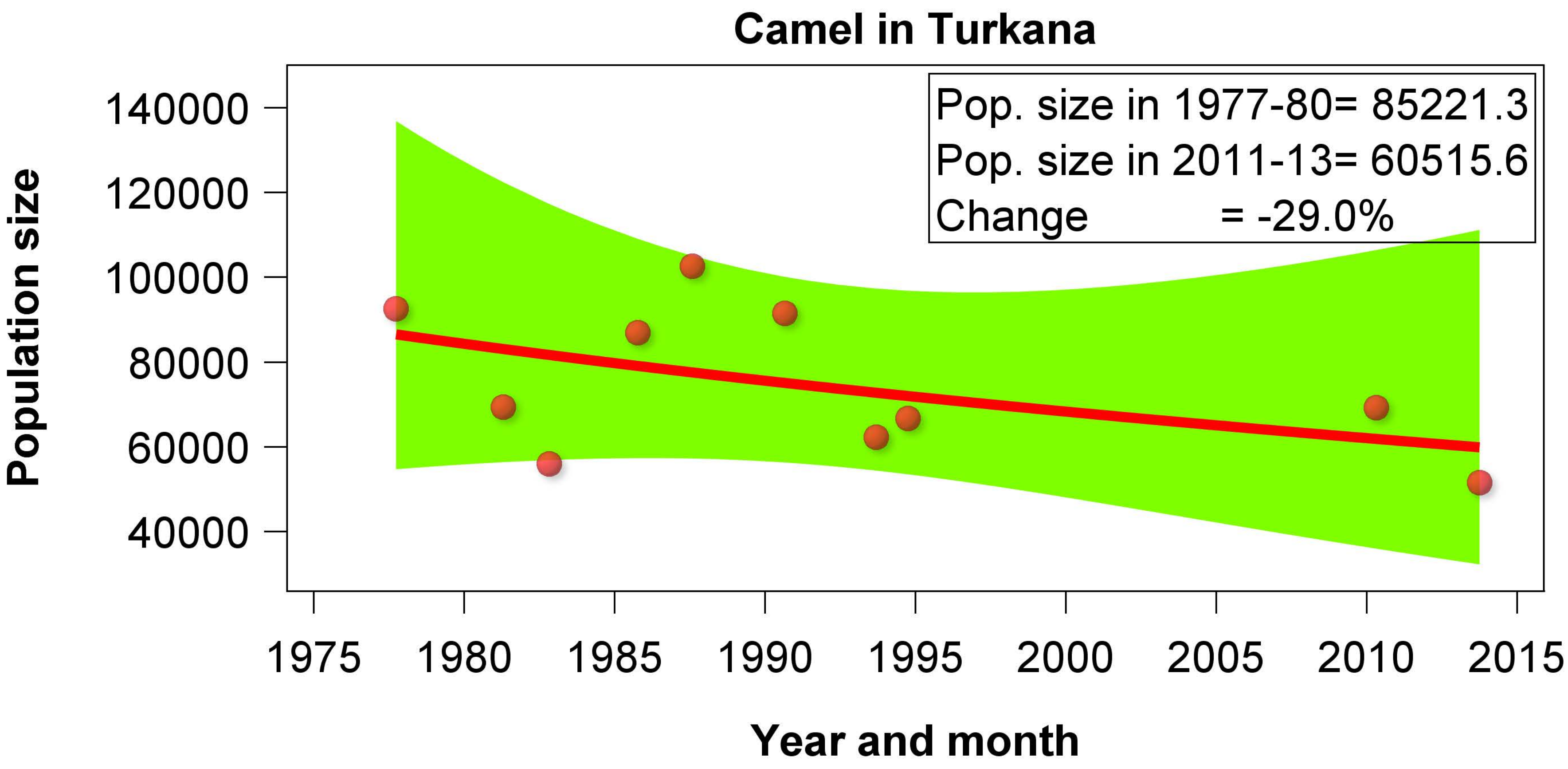
Camel in Turkana
Pop. size in 1977-80= 85221.3
Pop. size in 2011-13= 60515.6
Change = -29.0%
140000
120000
100000
80000
60000
40000
Population size
1975
1980
1985
1990
1995
2000
2005
2010
2015
Year and month

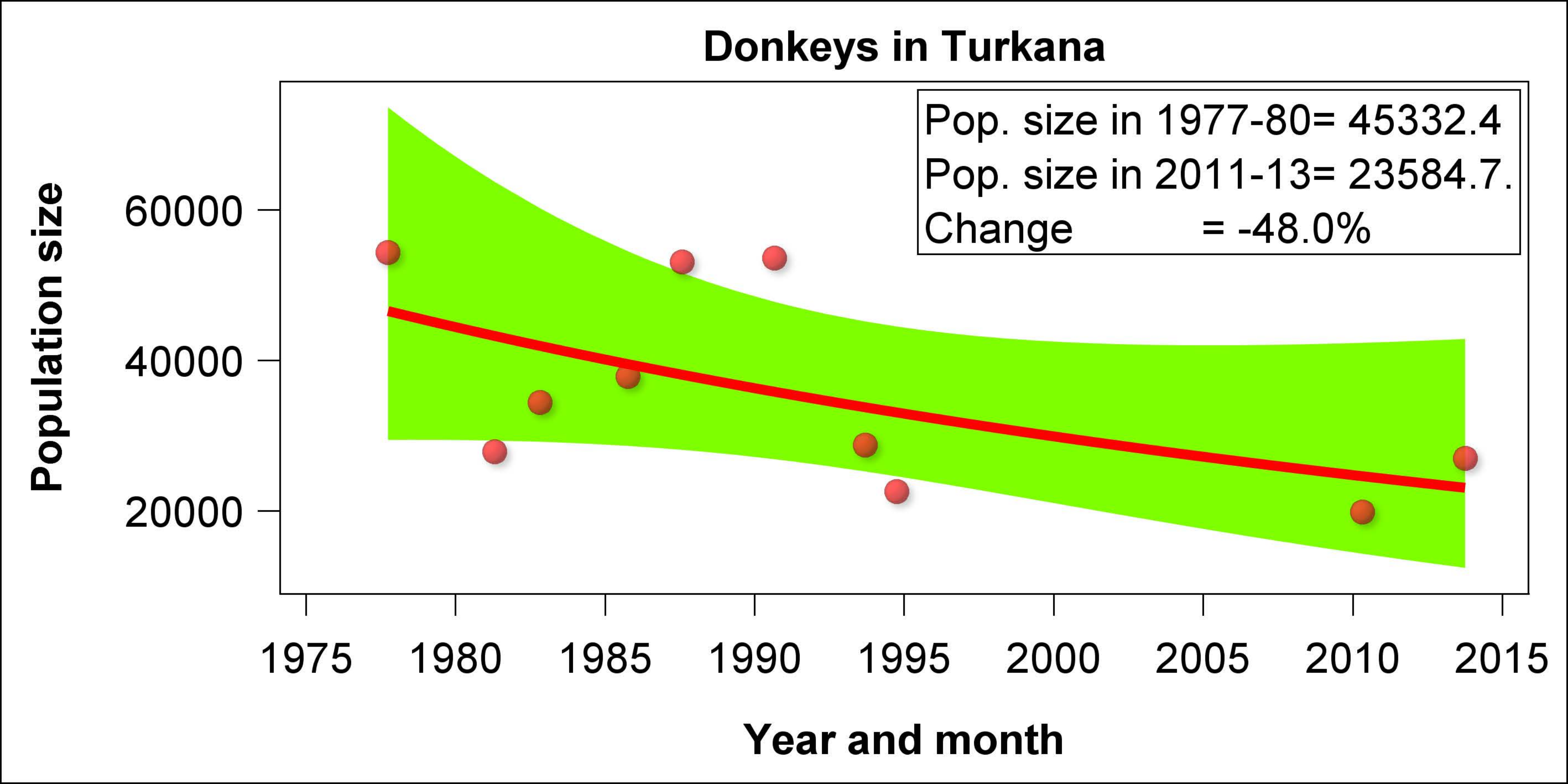
Donkeys in Turkana
Pop. size in 1977-80= 45332.4
Pop. size in 2011-13= 23584.7.
Change = -48.0%
Population size
60000
40000
20000
1975
1980
1985
1990
1995
2000
2005
2010
2015
Year and month

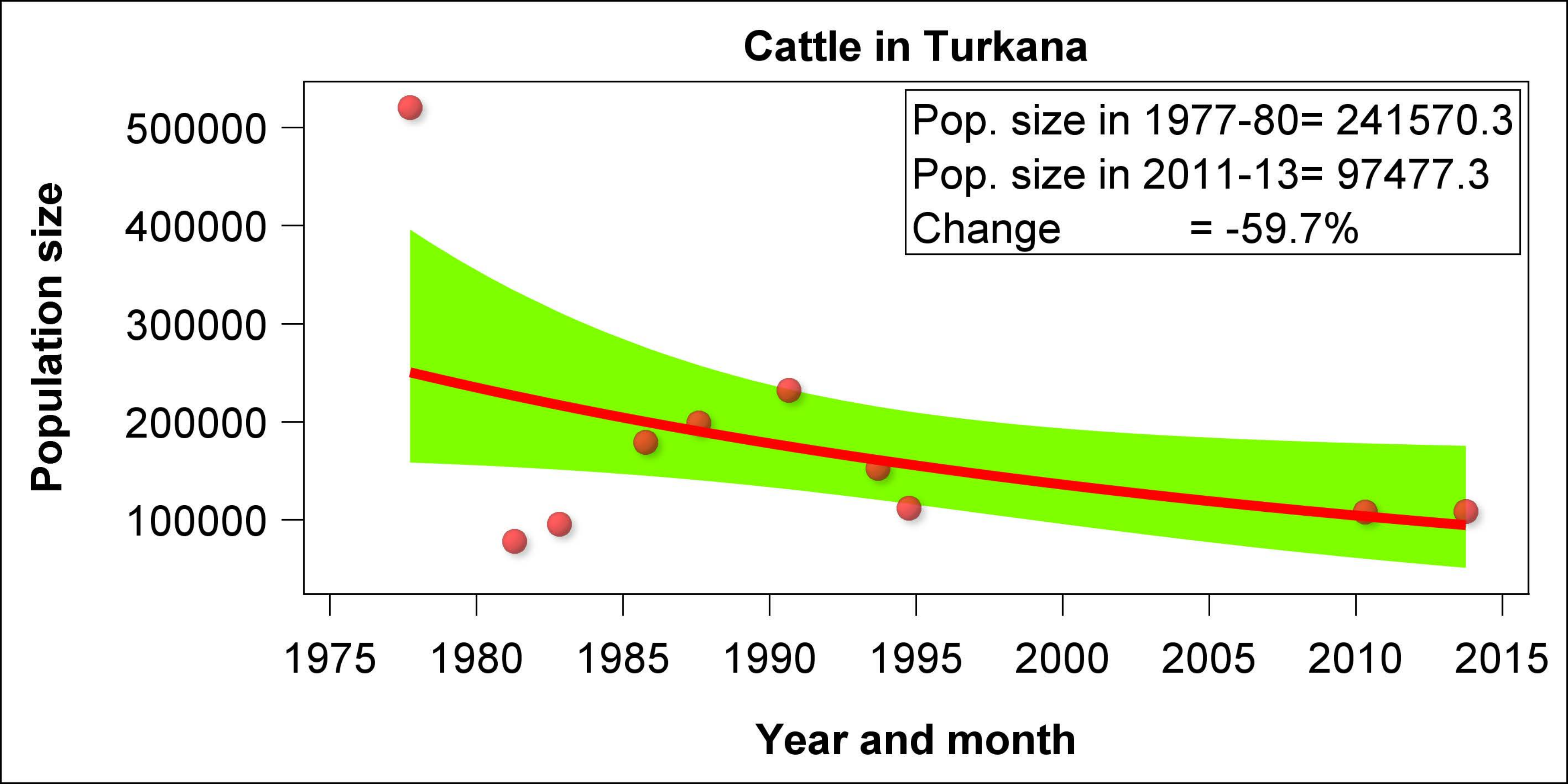

Cattle in Turkana
Pop. size in 1977-80= 241570.3
Pop. size in 2011-13= 97477.3
Change = -59.7%
500000
400000
300000
200000
100000
Population size
1975
1980
1985
1990
1995
2000
2005
2010
2015
Year and month

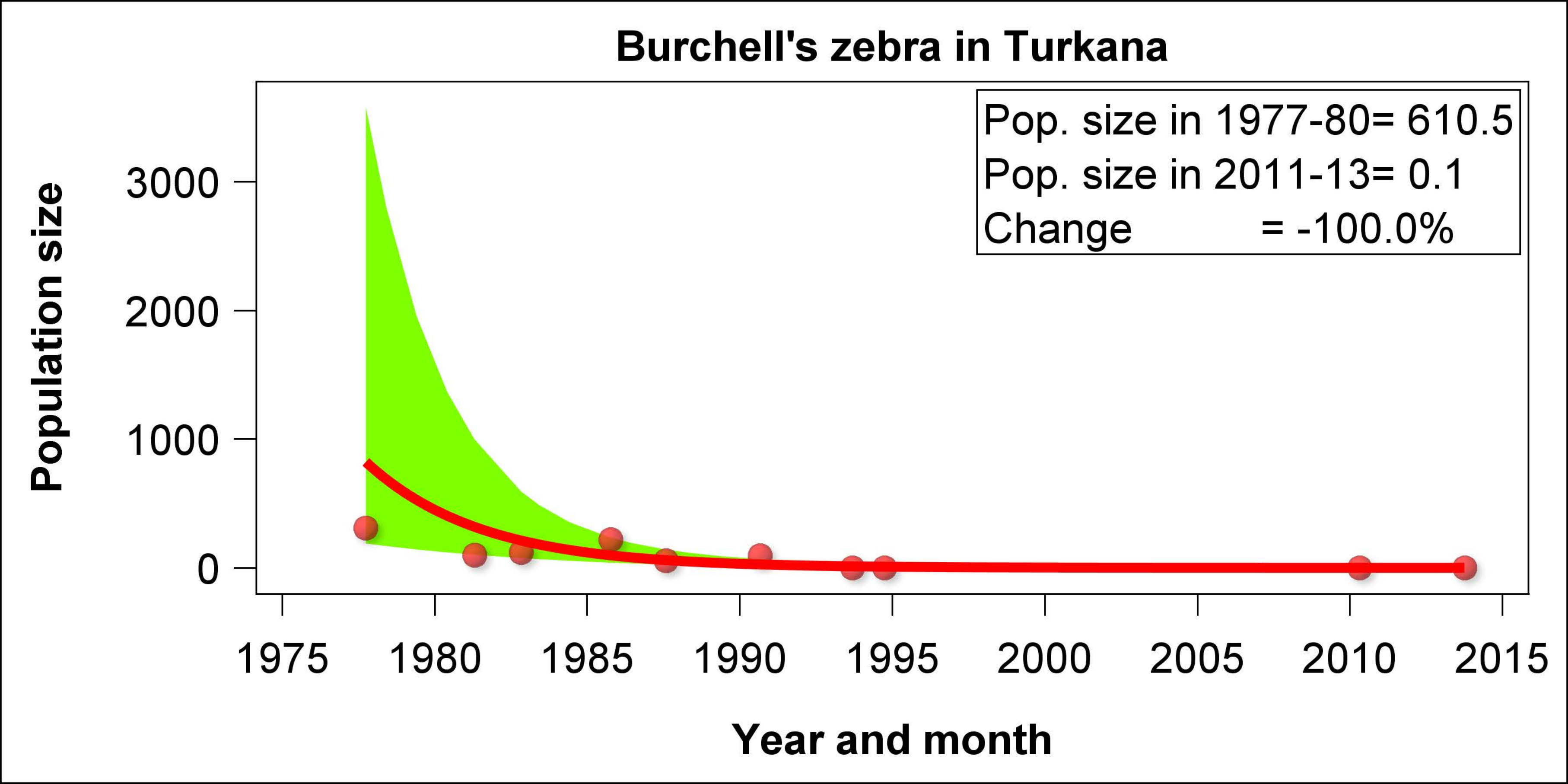
Burchell's zebra in Turkana
Pop. size in 1977-80= 610.5
Pop. size in 2011-13= 0.1
Change = -100.0%
Population size
3000
2000
1000
0
1975
1980
1985
1990
1995
2000
2005
2010
2015
Year and month

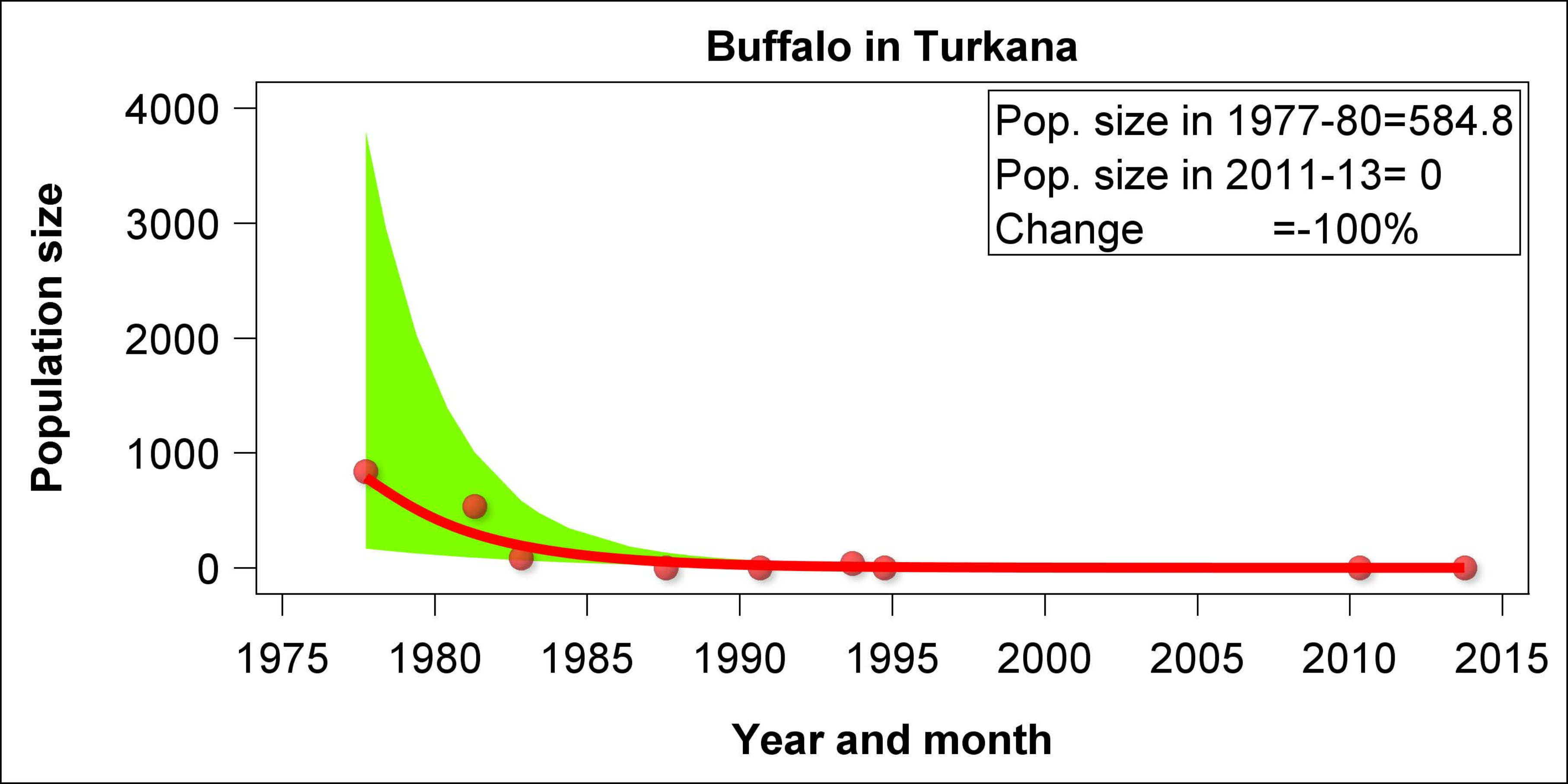
Buffalo in Turkana
Pop. size in 1977-80=584.8
Pop. size in 2011-13= 0
Change =-100%
Population size
4000
3000
2000
1000
0
1975
1980
1985
1990
1995
2000
2005
2010
2015
Year and month

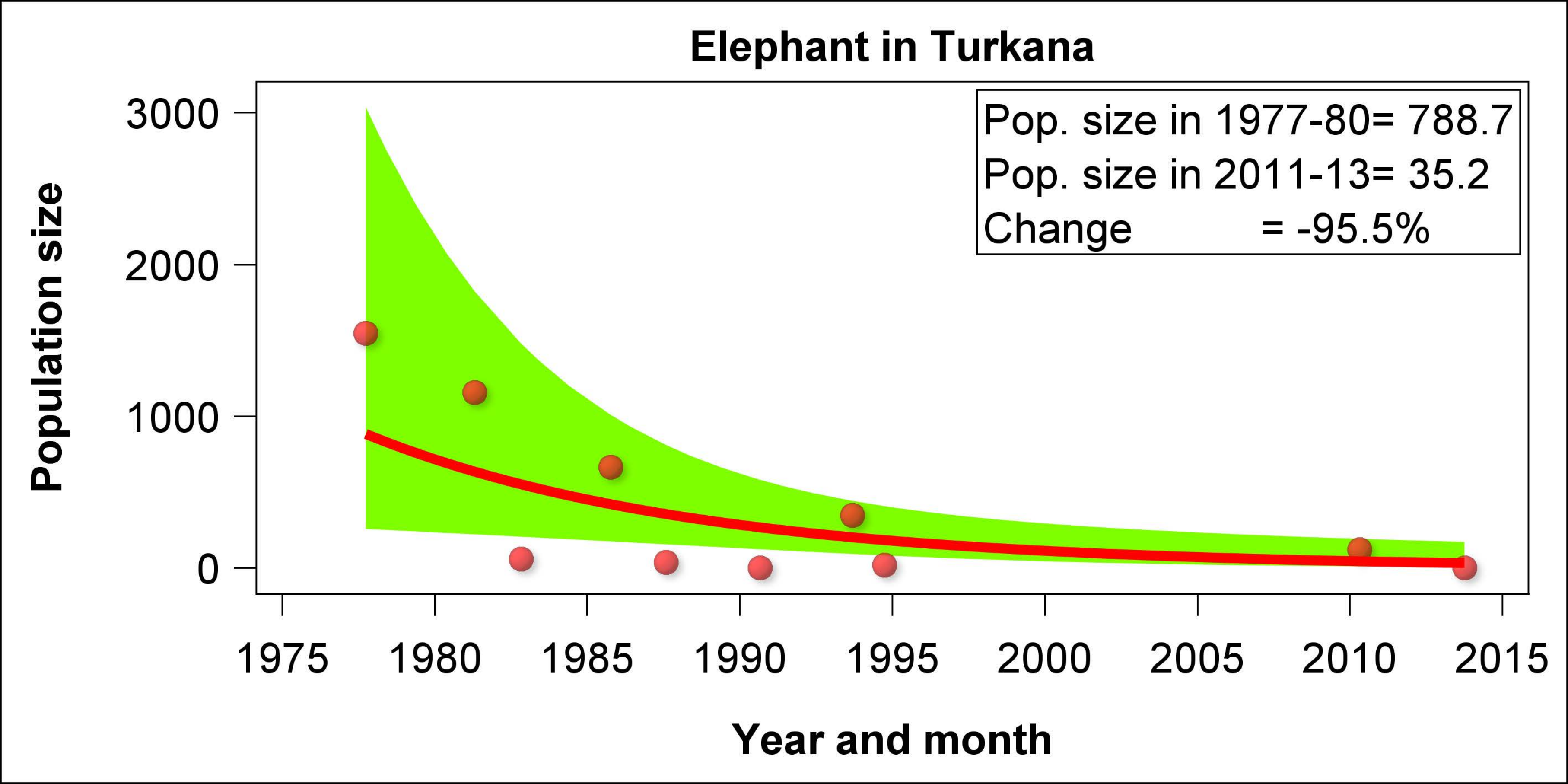
Elephant in Turkana
Pop. size in 1977-80= 788.7
Pop. size in 2011-13= 35.2
Change = -95.5%
Population size
3000
2000
1000
0
1975
1980
1985
1990
1995
2000
2005
2010
2015
Year and month

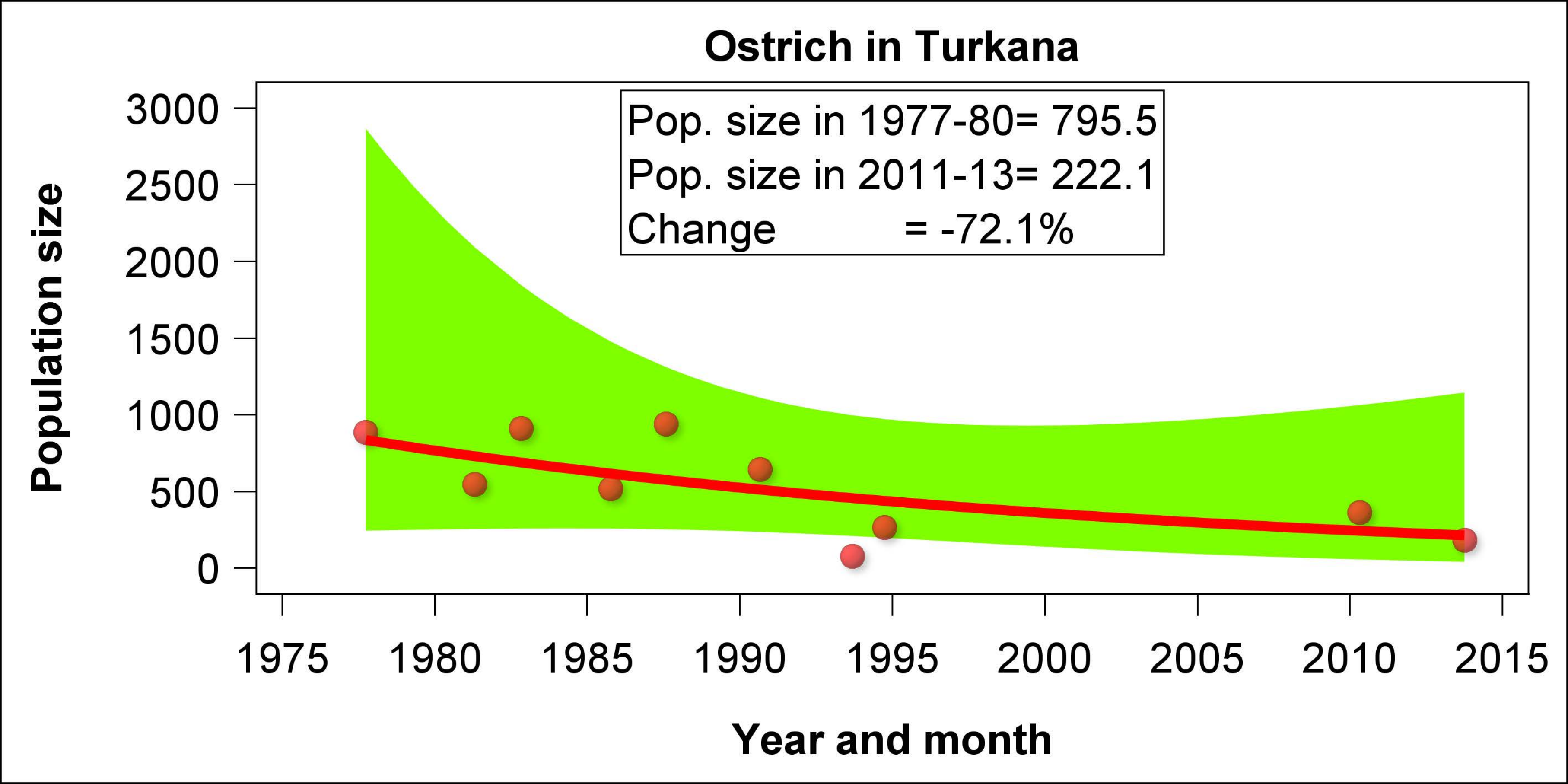
Ostrich in Turkana
Pop. size in 1977-80= 795.5
Pop. size in 2011-13= 222.1
Change = -72.1%
Population size
3000
2500
2000
1500
1000
500
0
1975
1980
1985
1990
1995
2000
2005
2010
2015
Year and month

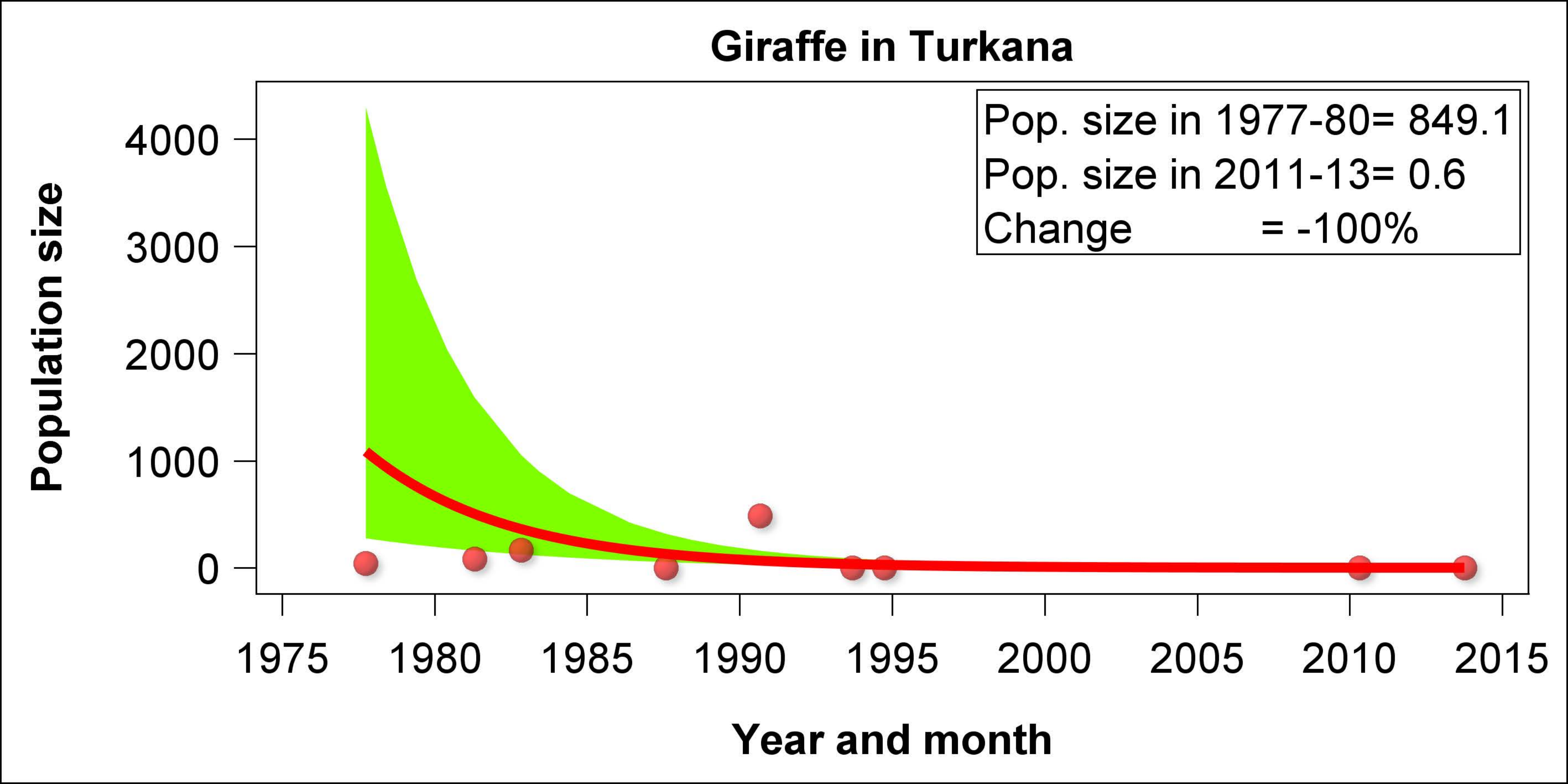

Giraffe in Turkana
Pop. size in 1977-80= 849.1
Pop. size in 2011-13= 0.6
Change = -100%
Population size
4000
3000
2000
1000
0
1975
1980
1985
1990
1995
2000
2005
2010
2015
Year and month

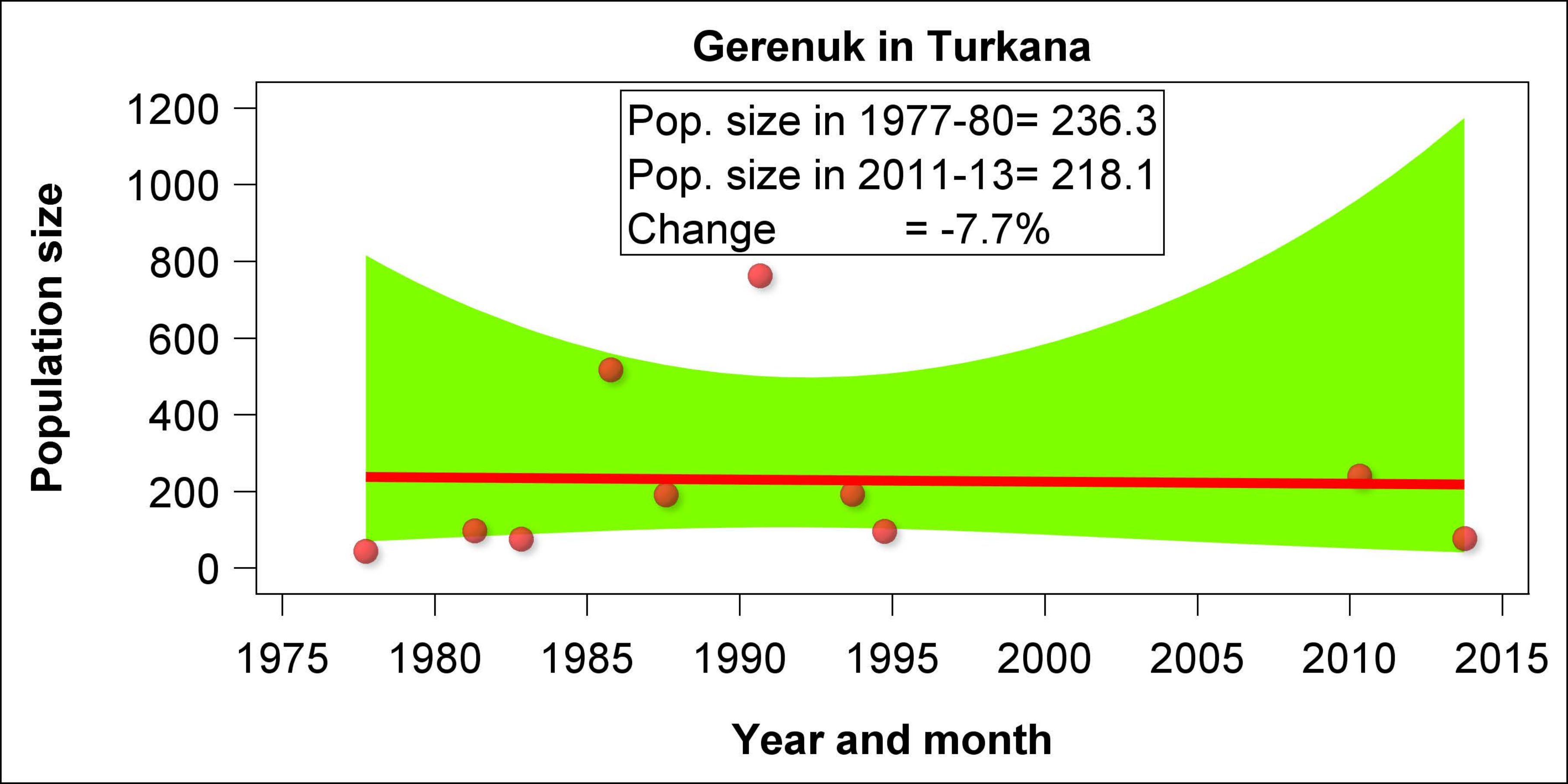
Gerenuk in Turkana
Pop. size in 1977-80= 236.3
Pop. size in 2011-13= 218.1
Change = -7.7%
Population size
0
200
400
600
800
1000
1200
1975
1980
1985
1990
1995
2000
2005
2010
2015
Year and month

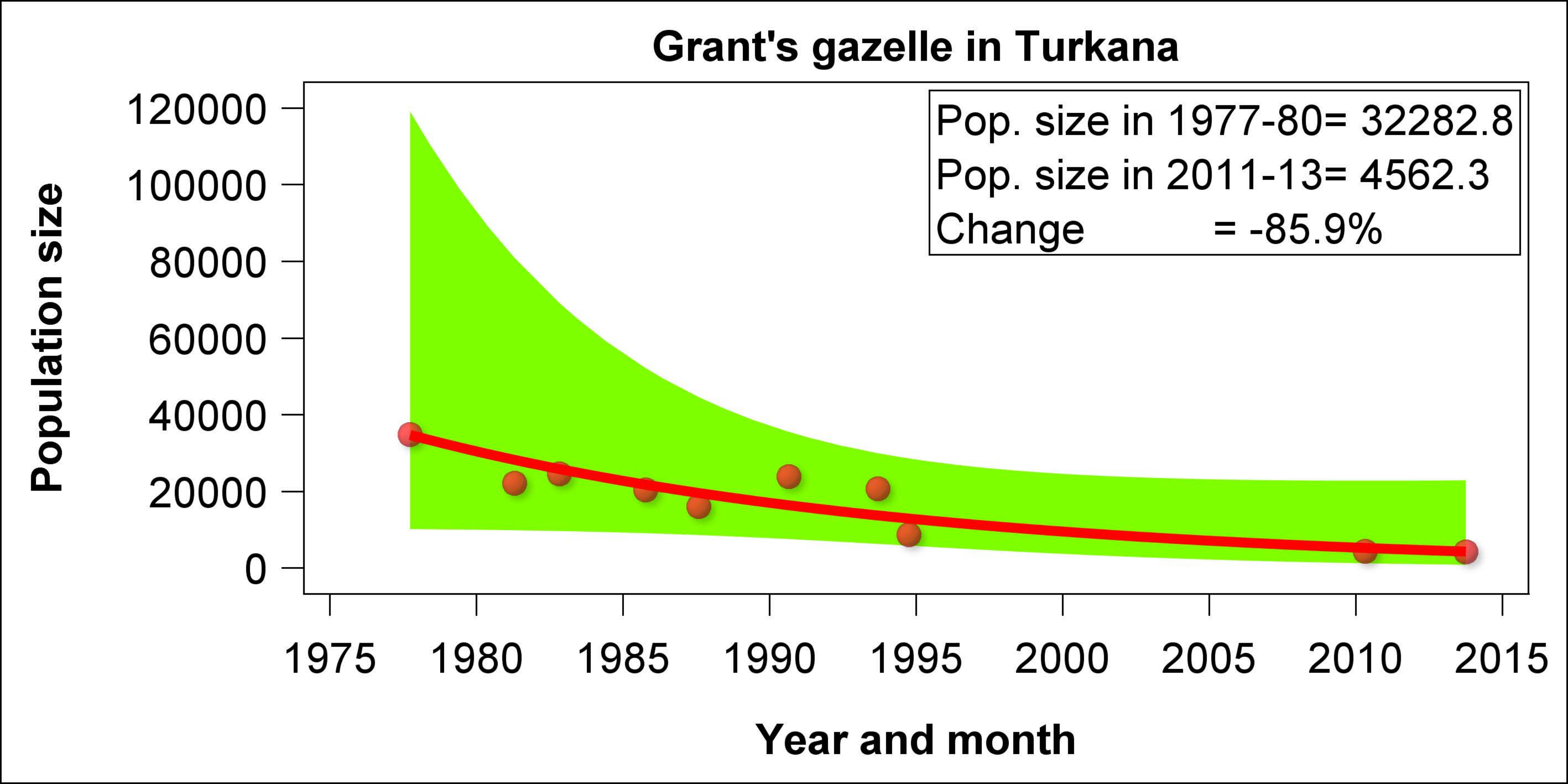

Grant's gazelle in Turkana
Pop. size in 1977-80= 32282.8
Pop. size in 2011-13= 4562.3
Change = -85.9%
Population size
120000
100000
80000
60000
40000
20000
0
1975
1980
1985
1990
1995
2000
2005
2010
2015
Year and month

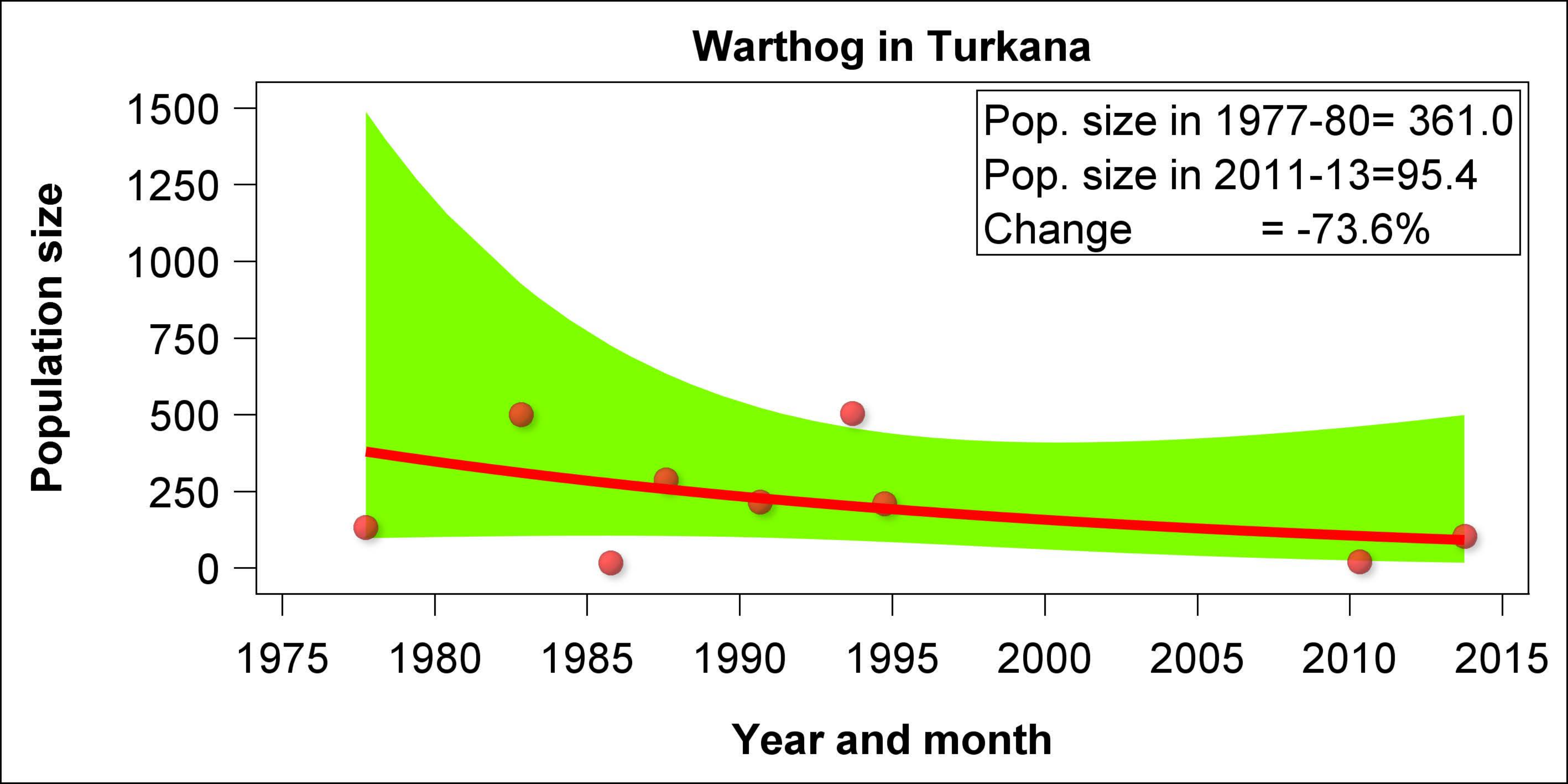
Warthog in Turkana
Pop. size in 1977-80= 361.0
Pop. size in 2011-13=95.4
Change = -73.6%
Population size
1500
1250
1000
750
500
250
0
1975
1980
1985
1990
1995
2000
2005
2010
2015
Year and month

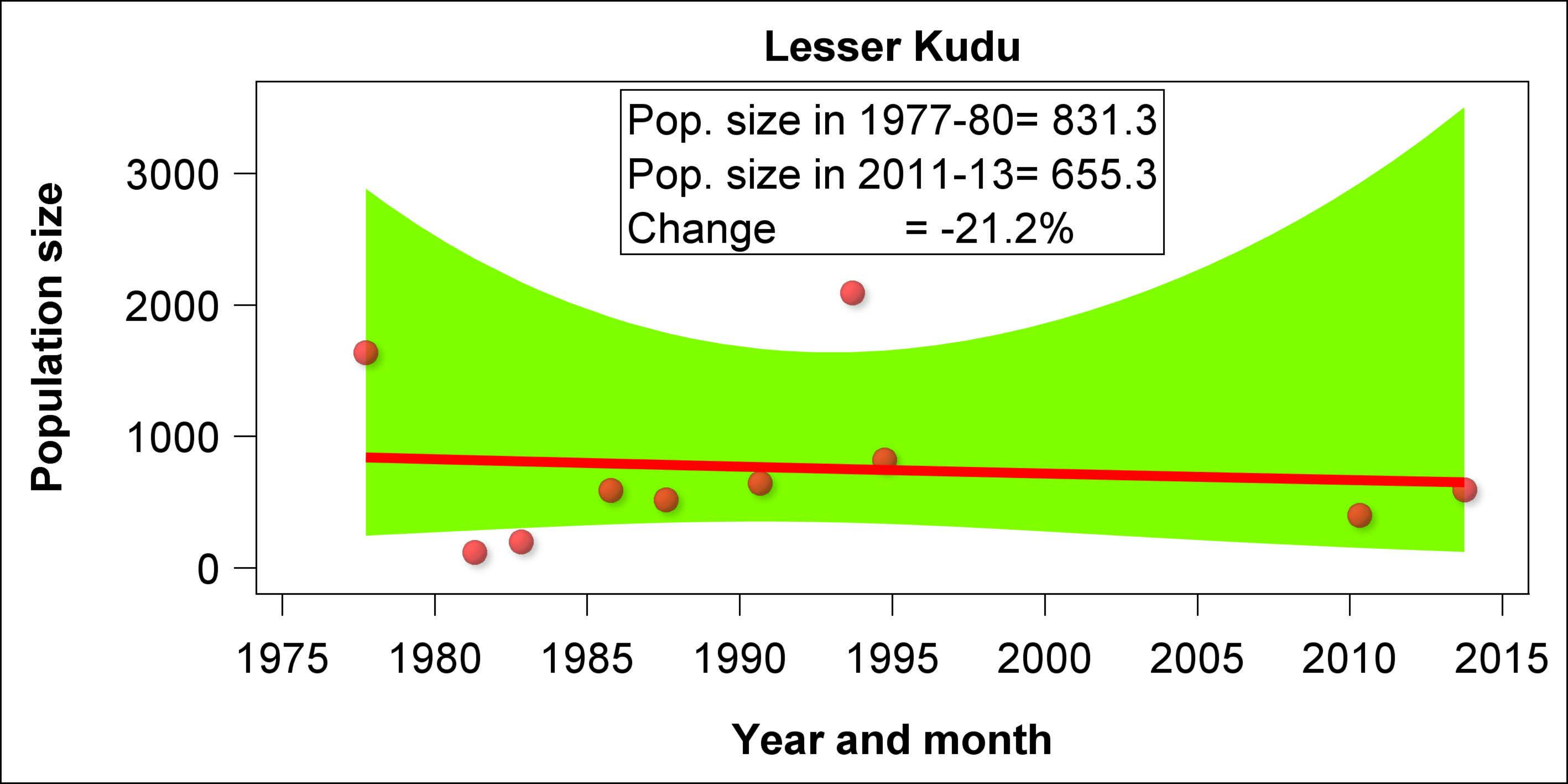

Lesser Kudu
Pop. size in 1977-80= 831.3
Pop. size in 2011-13= 655.3
Change = -21.2%
Population size
0
1000
2000
3000
1975
1980
1985
1990
1995
2000
2005
2010
2015
Year and month

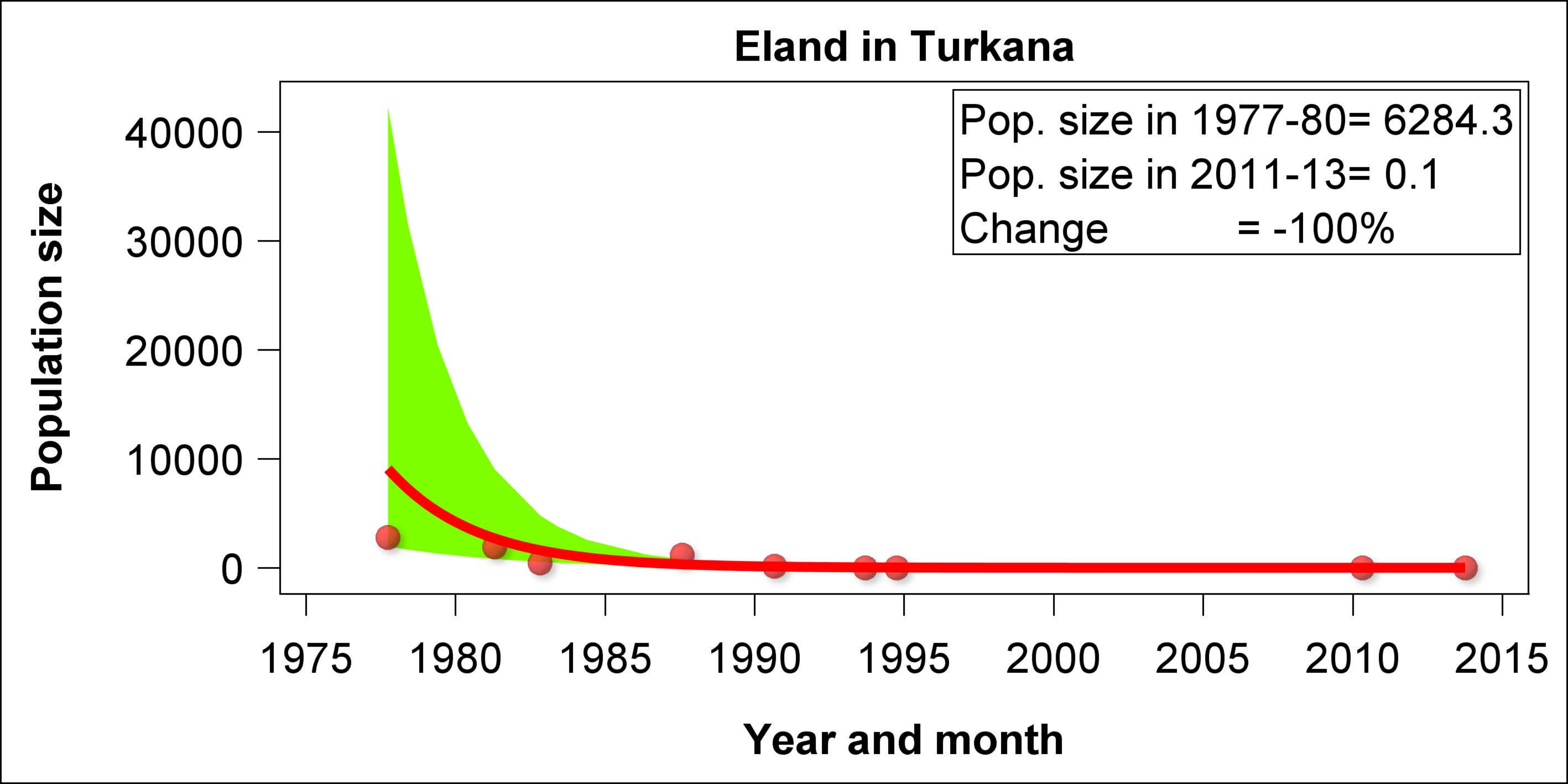
Eland in Turkana
Pop. size in 1977-80= 6284.3
Pop. size in 2011-13= 0.1
Change = -100%
Population size
40000
30000
20000
10000
0
1975
1980
1985
1990
1995
2000
2005
2010
2015
Year and month

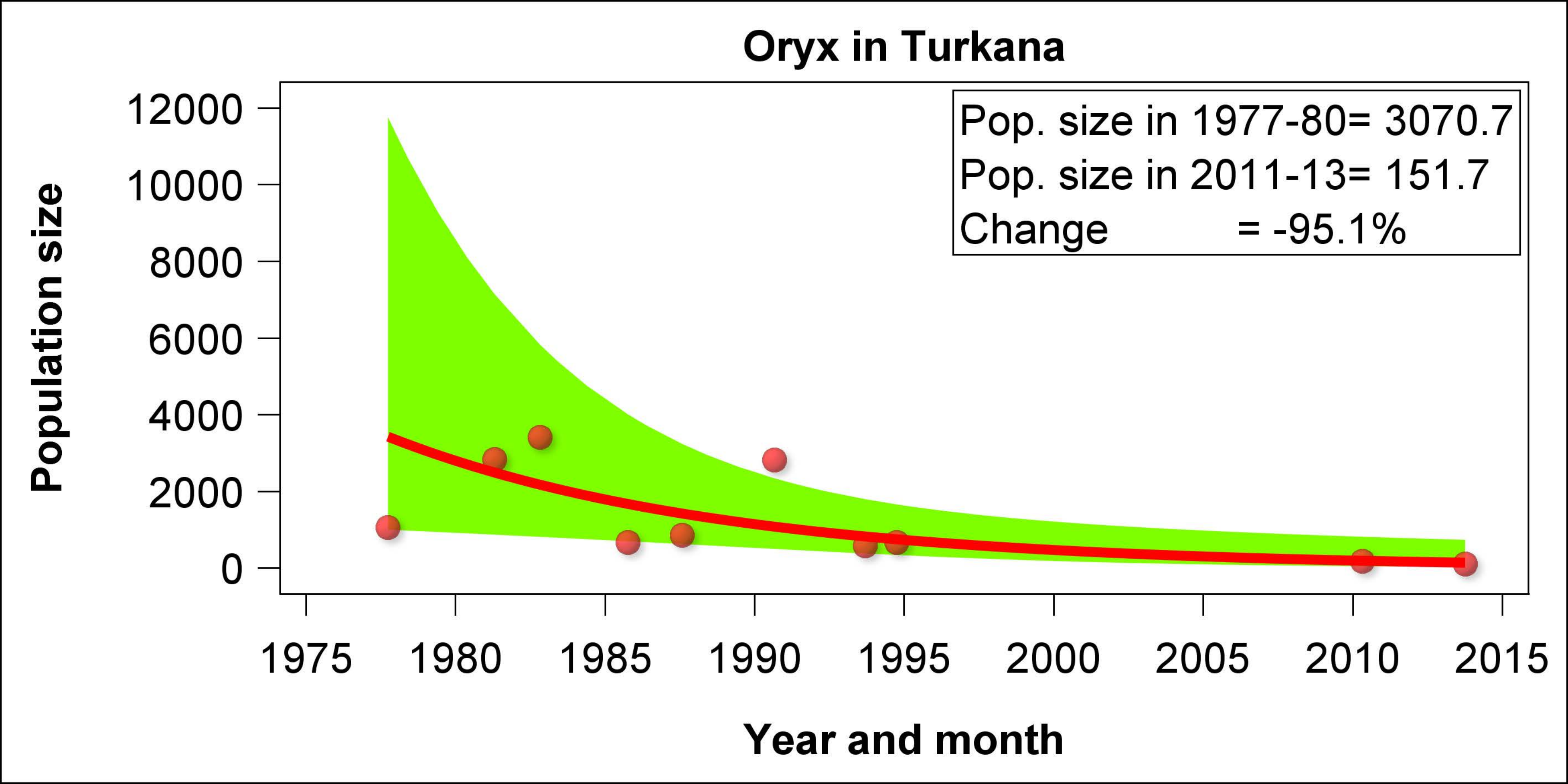

Oryx in Turkana
Pop. size in 1977-80= 3070.7
Pop. size in 2011-13= 151.7
Change = -95.1%
Population size
0
2000
4000
6000
8000
10000
12000
1975
1980
1985
1990
1995
2000
2005
2010
2015
Year and month

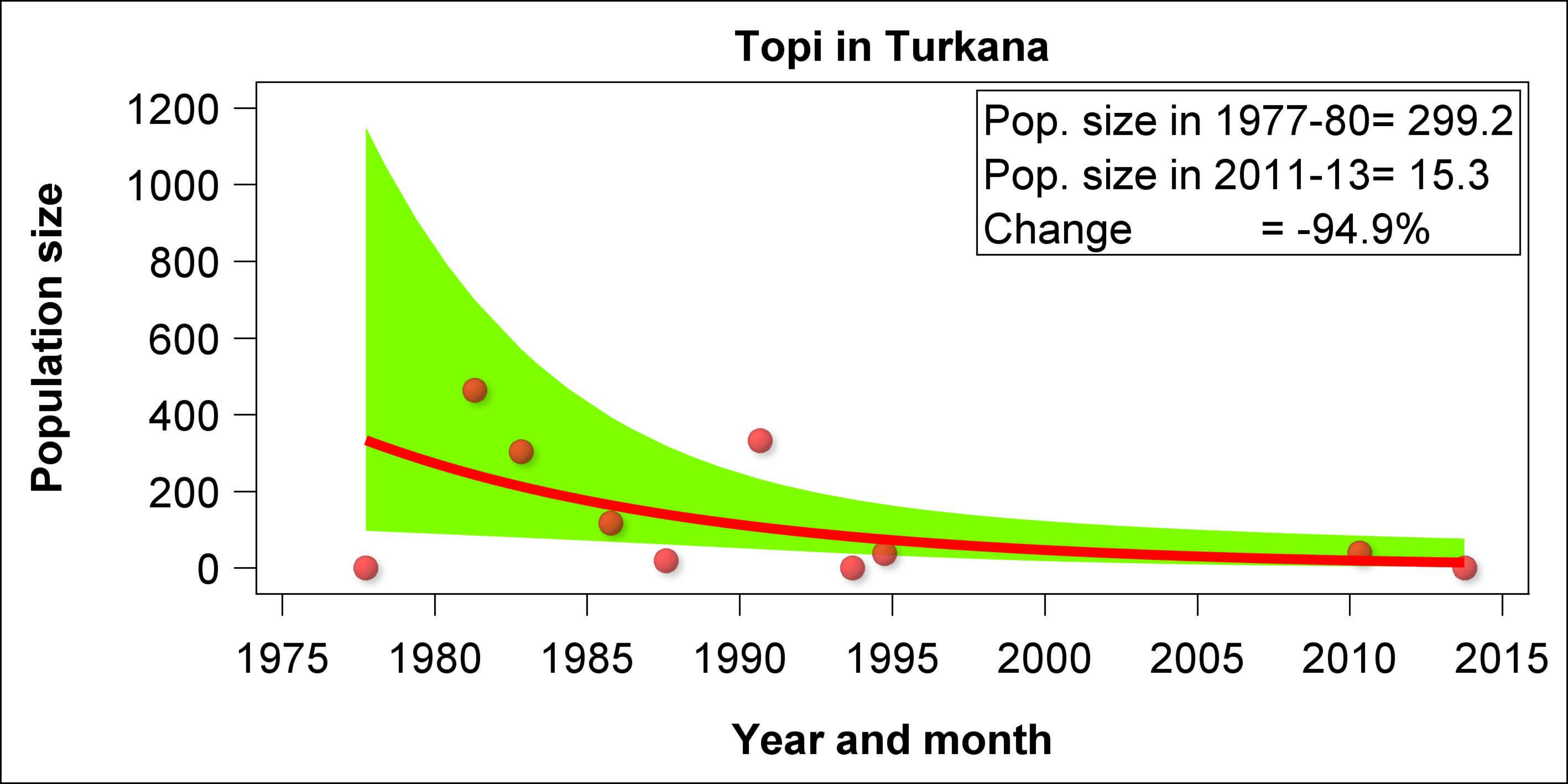
Topi in Turkana
Pop. size in 1977-80= 299.2
Pop. size in 2011-13= 15.3
Change = -94.9%
Population size
1200
1000
800
600
400
200
0
1975
1980
1985
1990
1995
2000
2005
2010
2015
Year and month

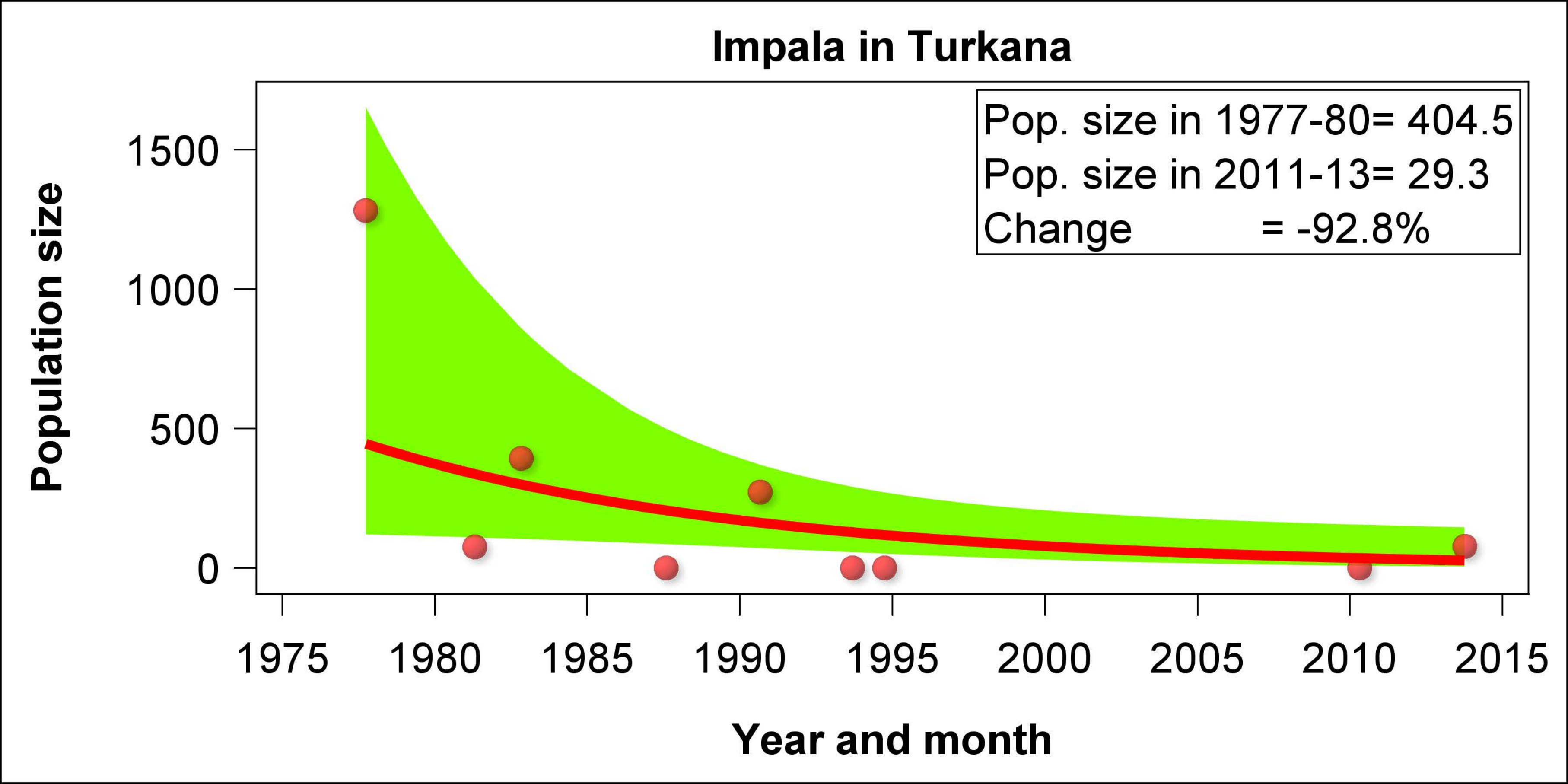
Impala in Turkana
Pop. size in 1977-80= 404.5
Pop. size in 2011-13= 29.3
Change = -92.8%
Population size
1500
1000
500
0
1975
1980
1985
1990
1995
2000
2005
2010
2015
Year and month
