# Supplementary material for: Extreme Wildlife Declines and Concurrent Increase in Livestock Numbers in Kenya: What Are the Causes?
Source: PLoS One. 2016 Sep 27;11(9):e0163249. doi: 10.1371/journal.pone.0163249 (PMC5039022; doi:10.1371/journal.pone.0163249)
Supplement: S20 Fig — The solid red line is the fitted trend curve and the shaded chartreuse band is the pointwise 95% confidence band. The estimated average population size in 1977–1980 and 2011–2013 and the percentage change in population size between the two periods are provided in the inset. (PDF) [file pone.0163249.s030.pdf]

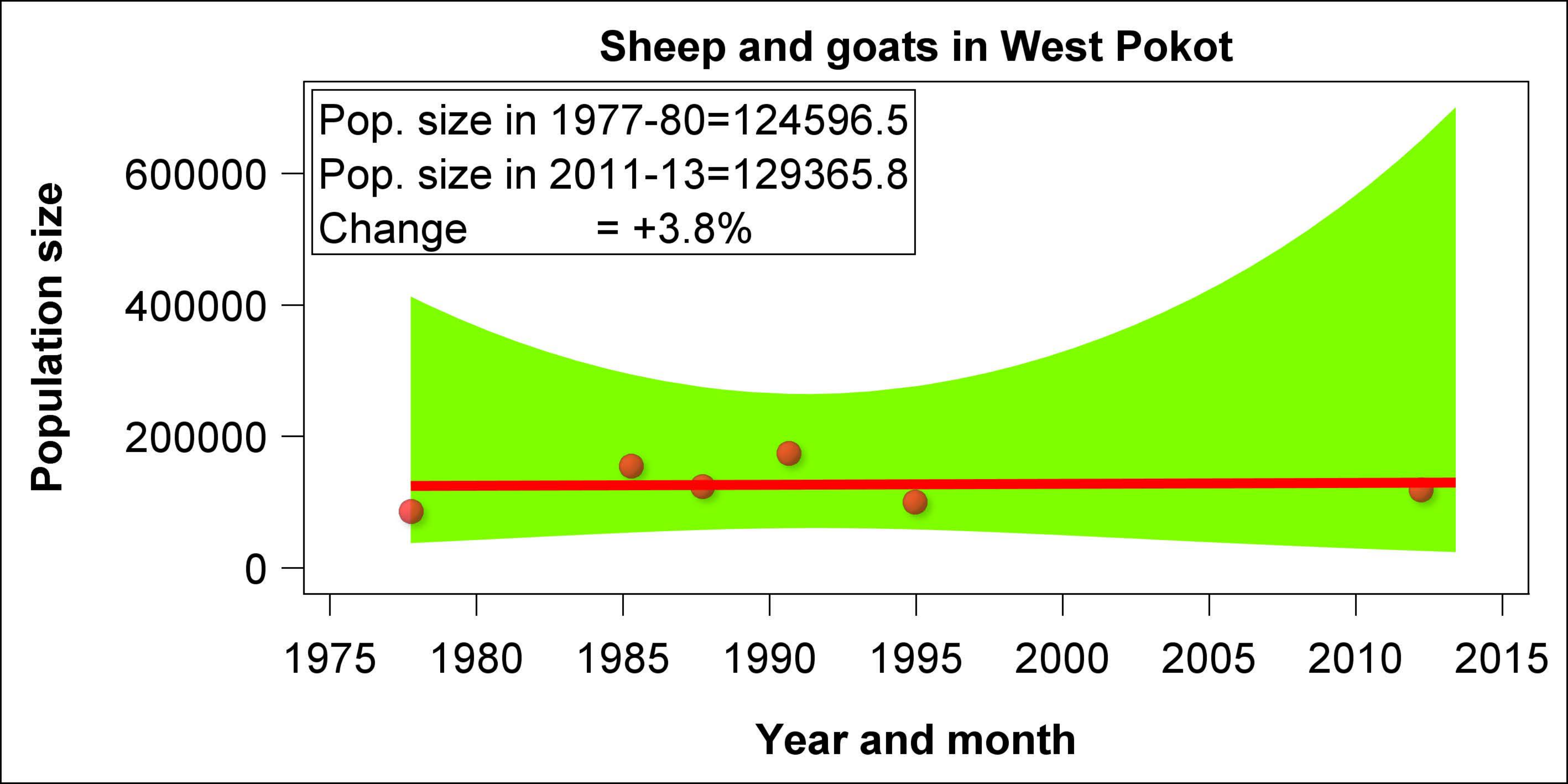
Sheep and goats in West Pokot
Pop. size in 1977-80=124596.5
Pop. size in 2011-13=129365.8
Change = +3.8%
Population size
600000
400000
200000
0
1975
1980
1985
1990
1995
2000
2005
2010
2015
Year and month

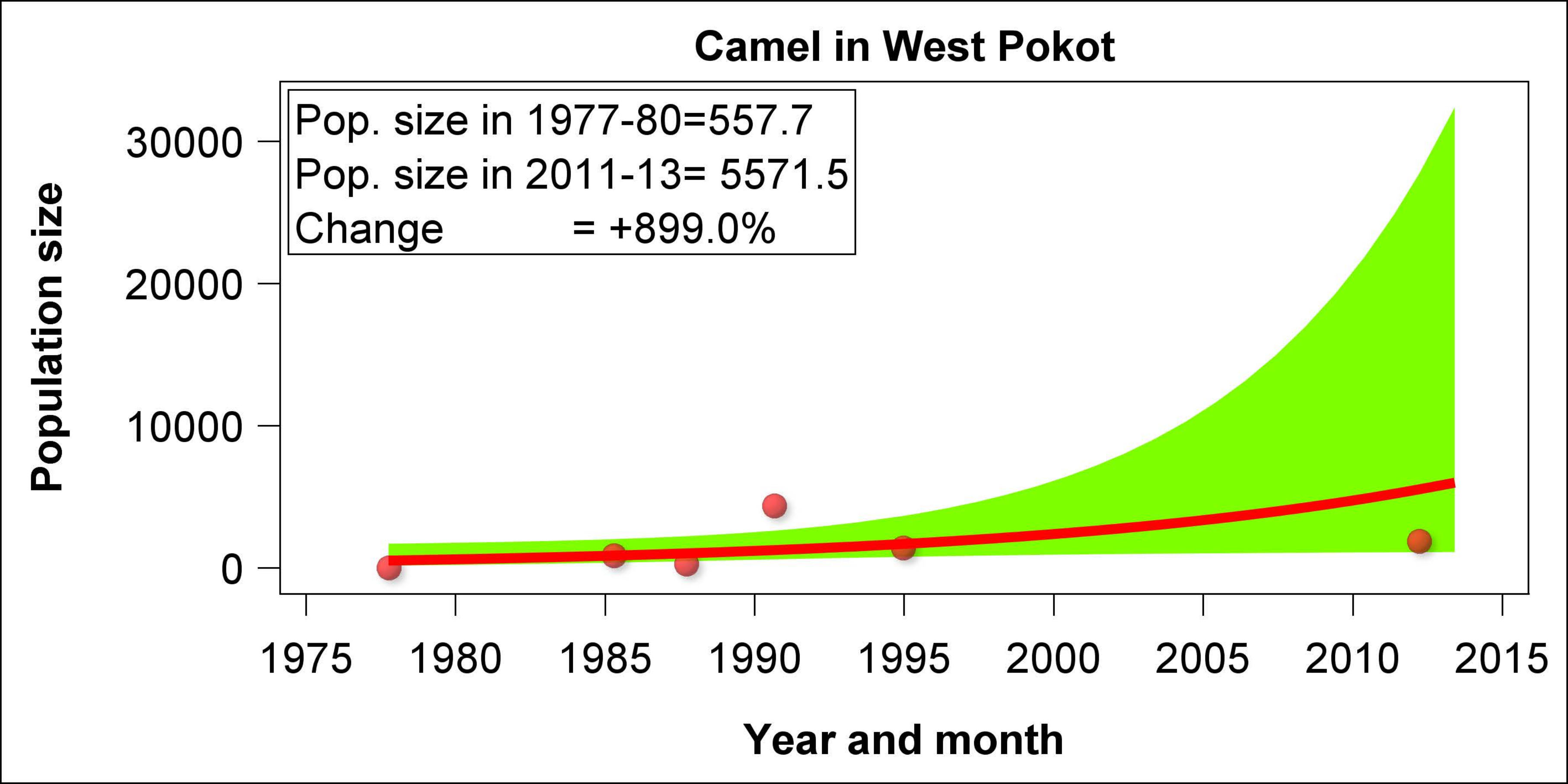
Camel in West Pokot
Pop. size in 1977-80=557.7
Pop. size in 2011-13= 5571.5
Change = +899.0%
Population size
30000
20000
10000
0
1975
1980
1985
1990
1995
2000
2005
2010
2015
Year and month

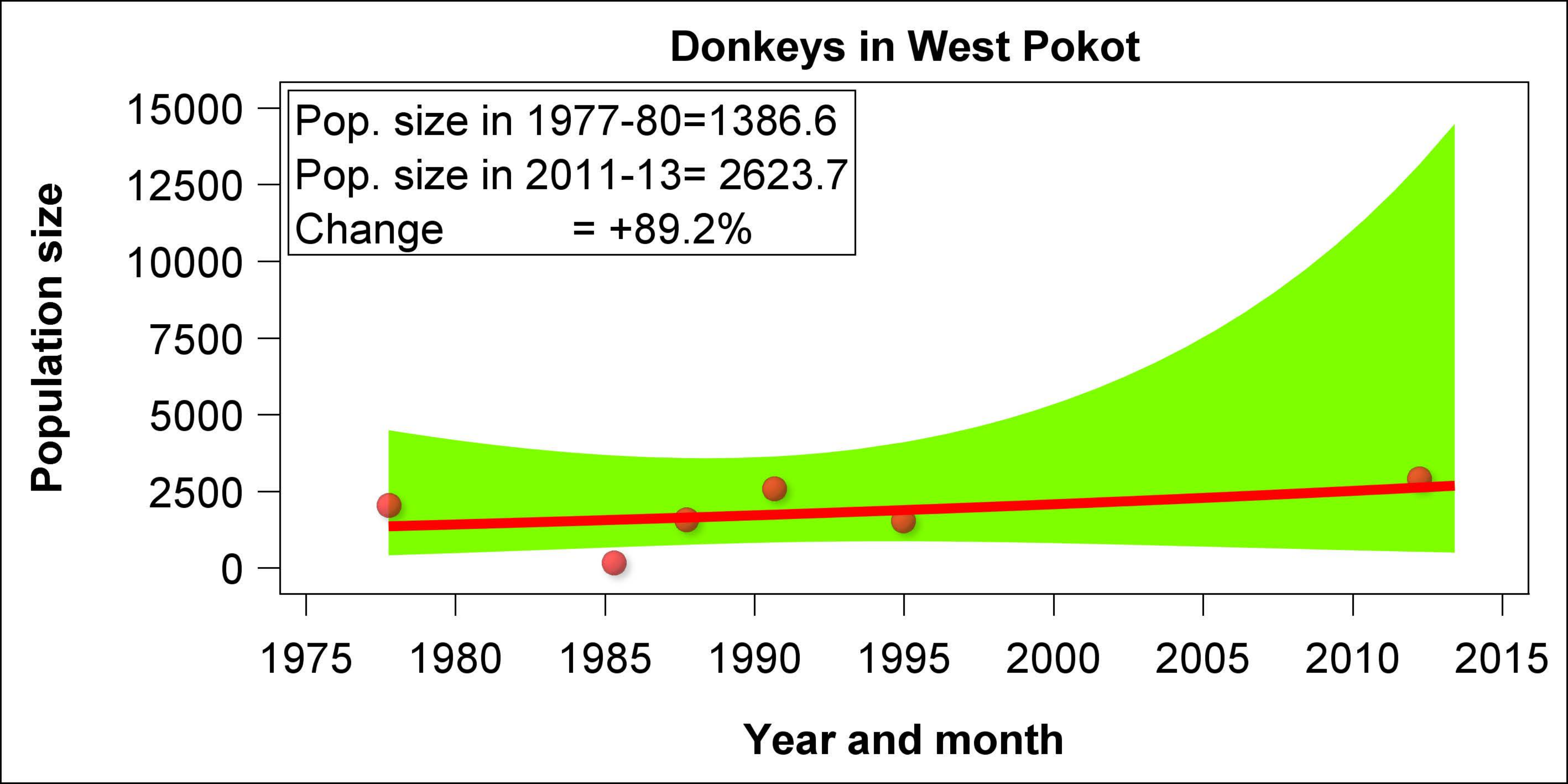

Donkeys in West Pokot
Pop. size in 1977-80=1386.6
Pop. size in 2011-13= 2623.7
Change = +89.2%
Population size
15000
12500
10000
7500
5000
2500
0
1975
1980
1985
1990
1995
2000
2005
2010
2015
Year and month

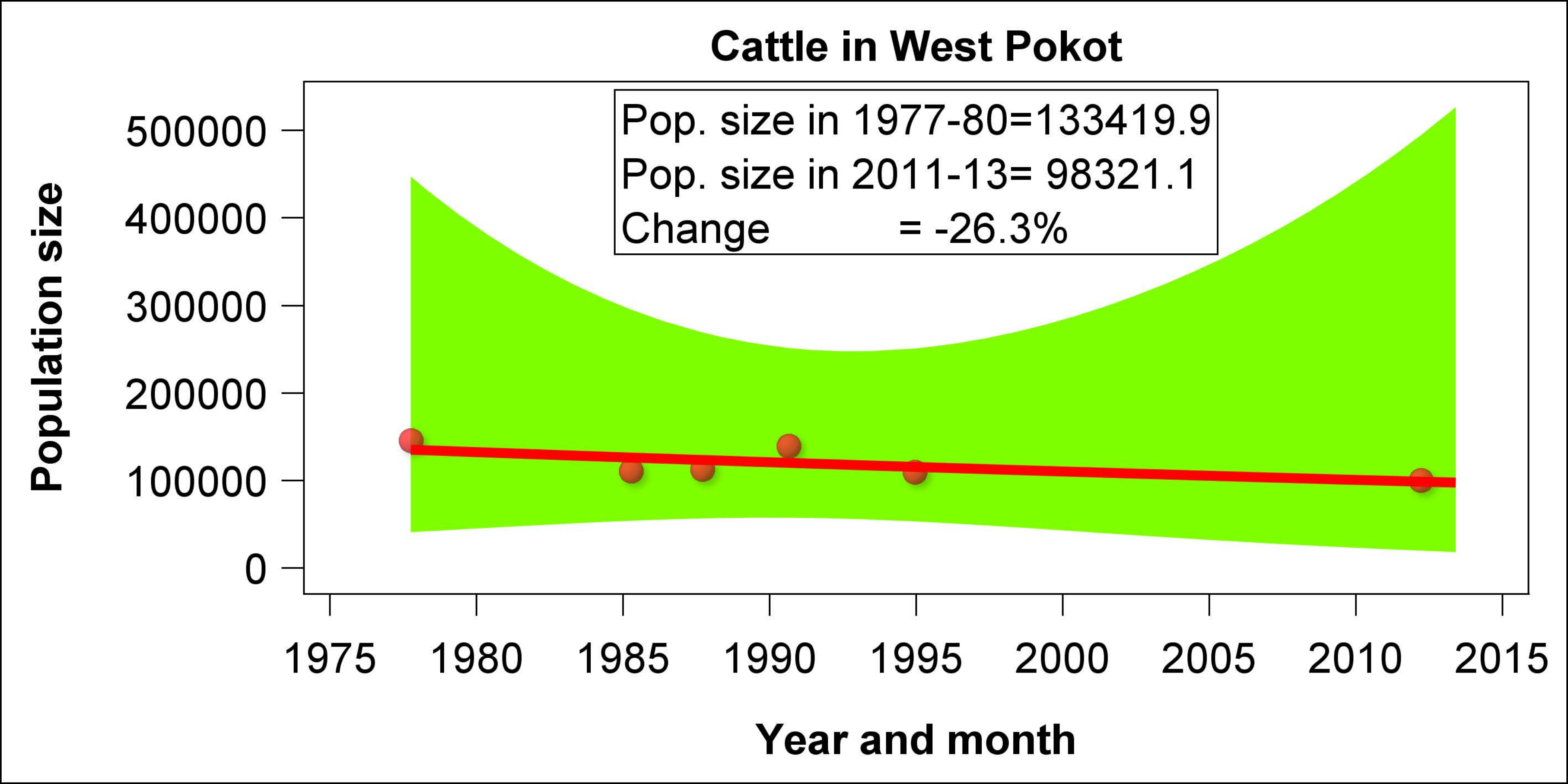
Cattle in West Pokot
Pop. size in 1977-80=133419.9
Pop. size in 2011-13= 98321.1
Change = -26.3%
Population size
500000
400000
300000
200000
100000
0
1975
1980
1985
1990
1995
2000
2005
2010
2015
Year and month

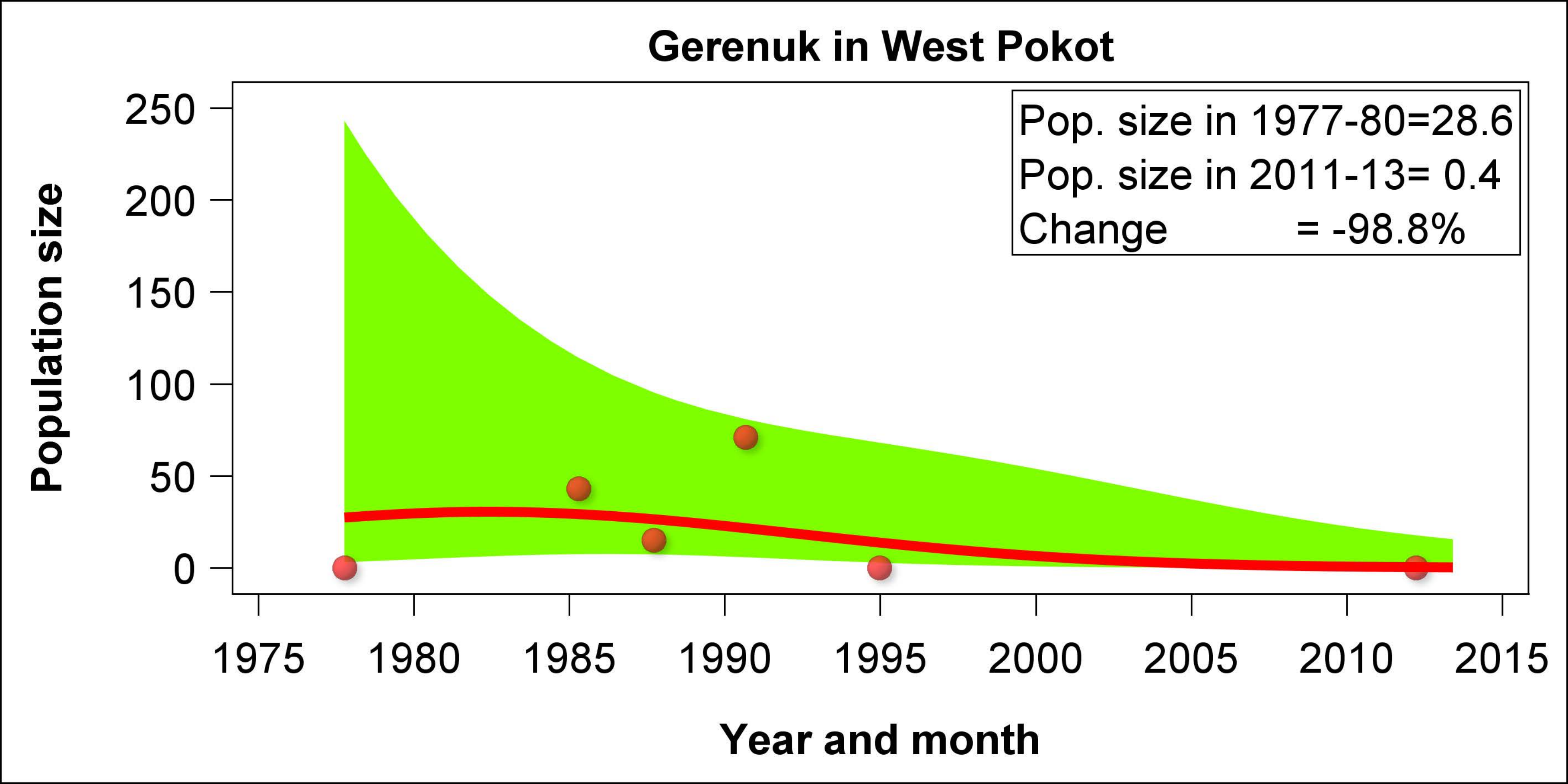
Gerenuk in West Pokot
Pop. size in 1977-80=28.6
Pop. size in 2011-13= 0.4
Change = -98.8%
Population size
0
50
100
150
200
250
1975
1980
1985
1990
1995
2000
2005
2010
2015
Year and month

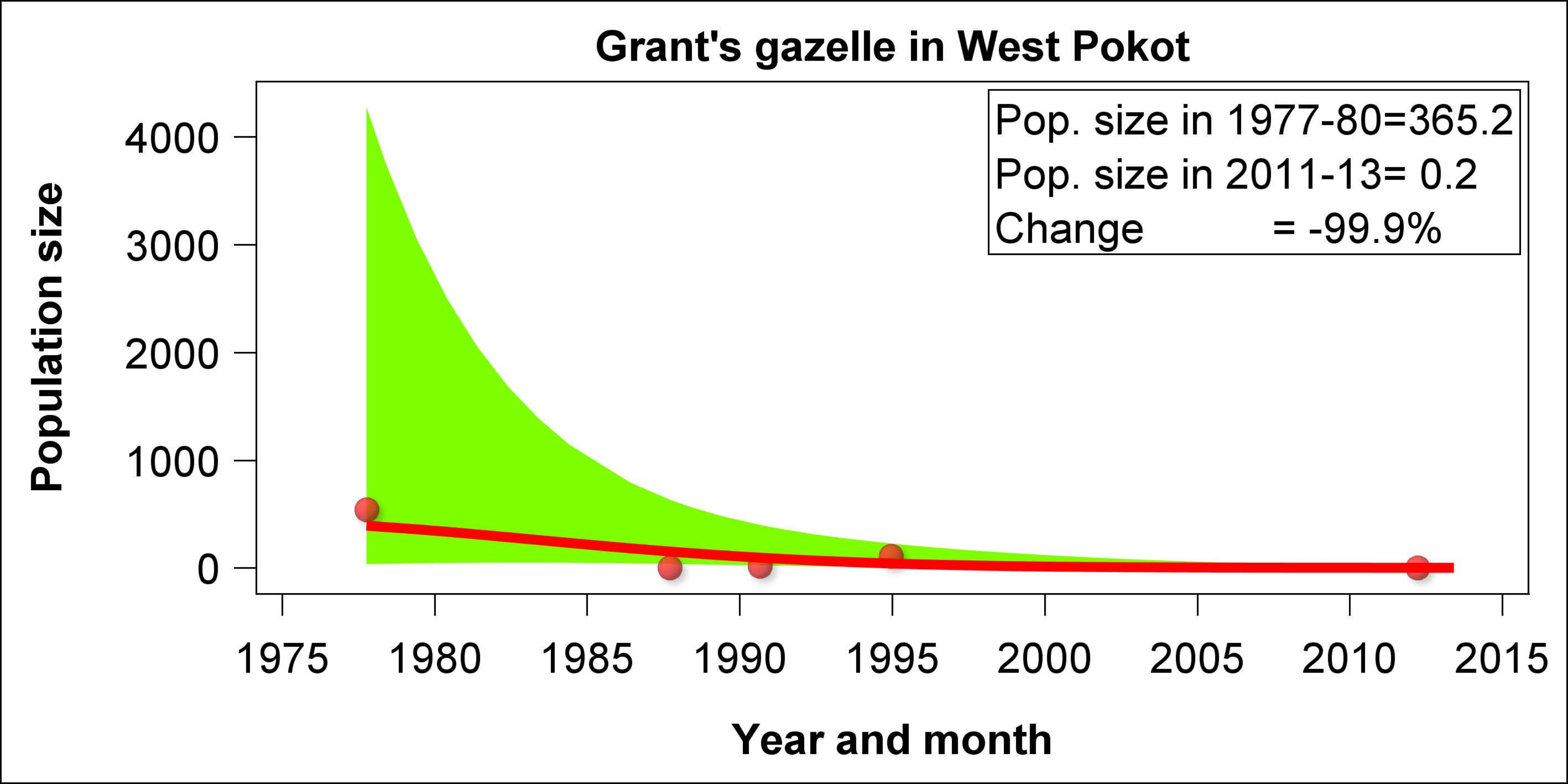
Grant's gazelle in West Pokot
Pop. size in 1977-80=365.2
Pop. size in 2011-13= 0.2
Change = -99.9%
Population size
4000
3000
2000
1000
0
1975
1980
1985
1990
1995
2000
2005
2010
2015
Year and month

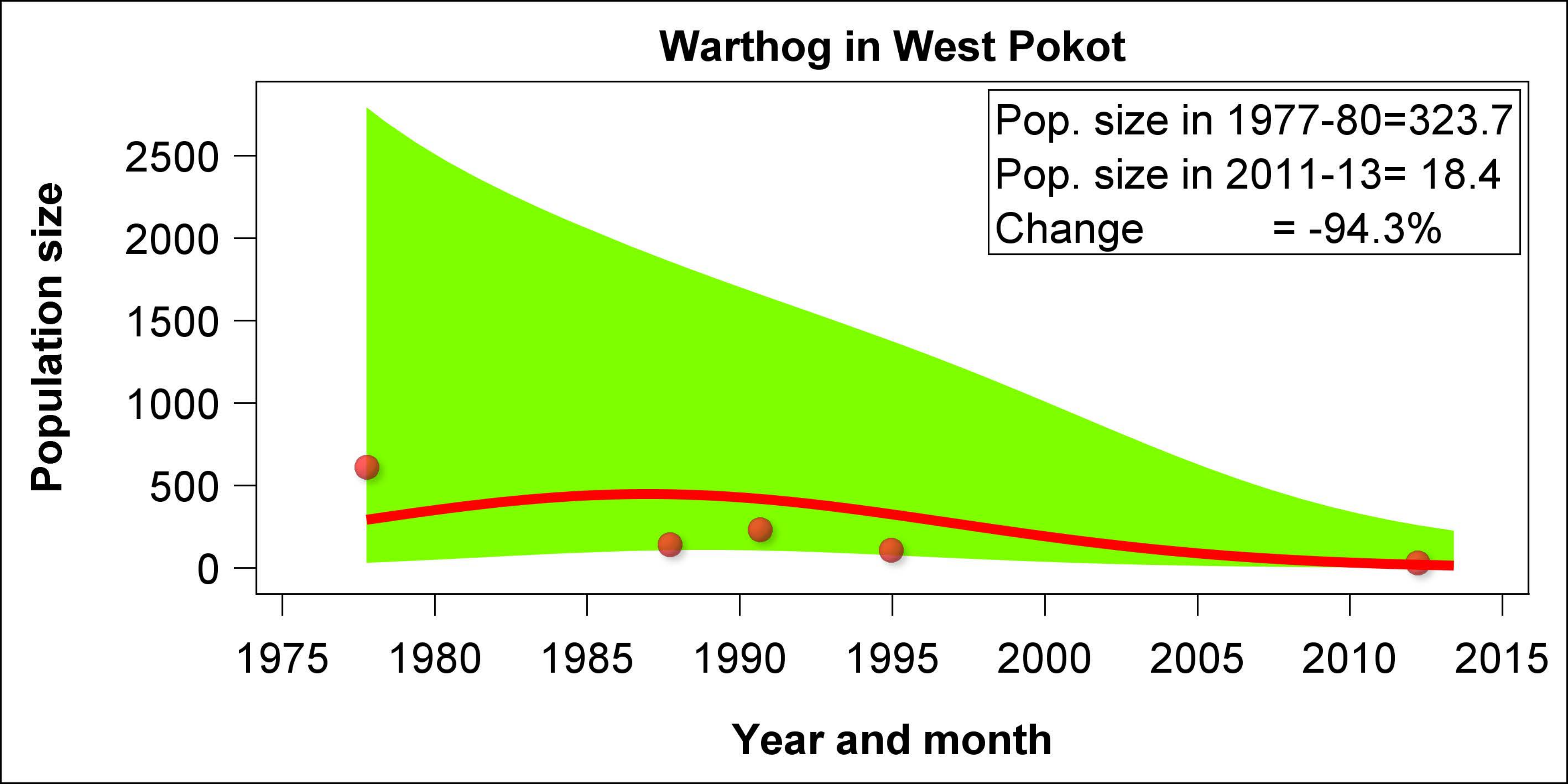
Warthog in West Pokot
Pop. size in 1977-80=323.7
Pop. size in 2011-13= 18.4
Change = -94.3%
Population size
0
500
1000
1500
2000
2500
1975
1980
1985
1990
1995
2000
2005
2010
2015
Year and month

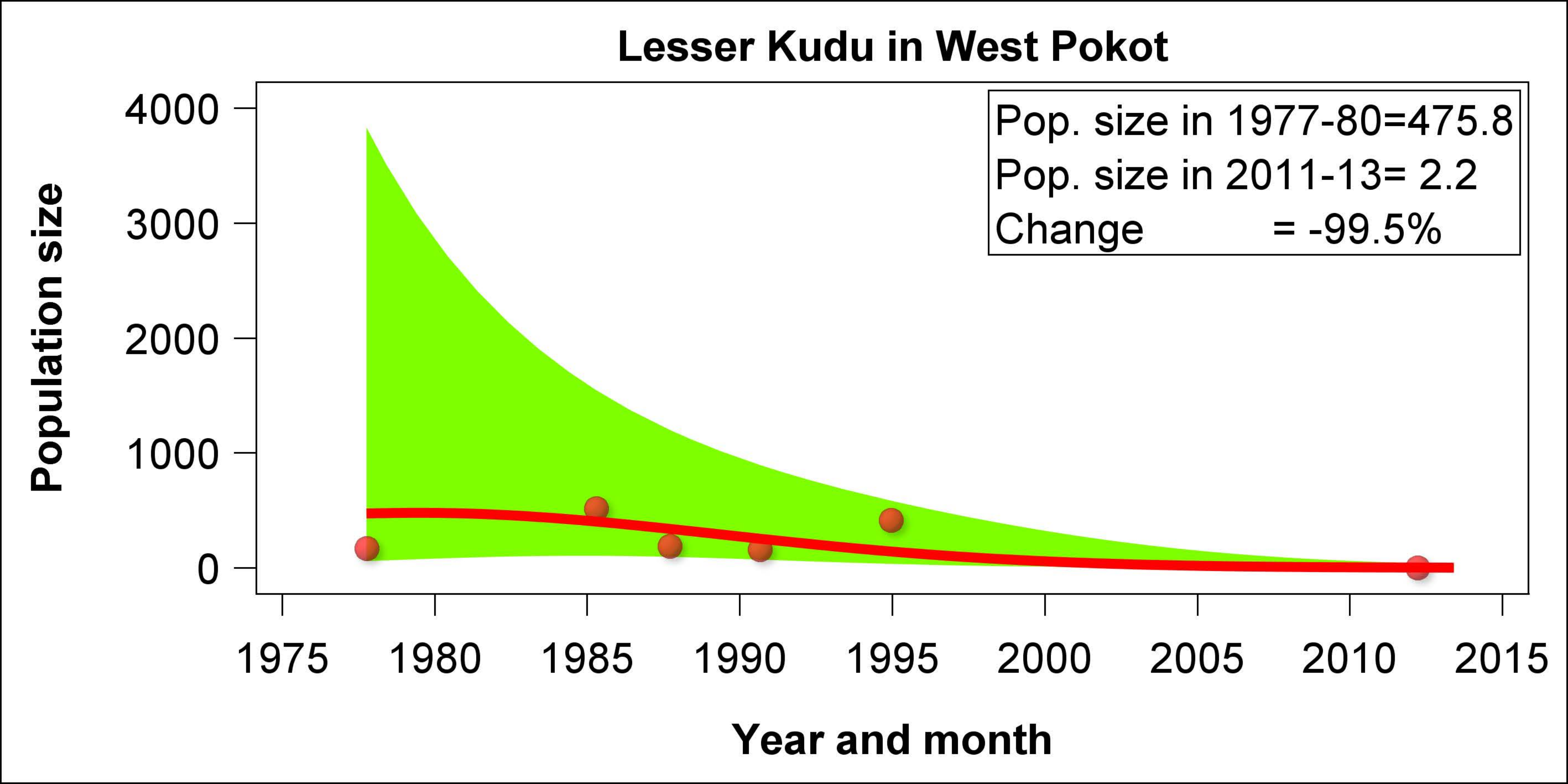
Lesser Kudu in West Pokot
Pop. size in 1977-80=475.8
Pop. size in 2011-13= 2.2
Change = -99.5%
Population size
4000
3000
2000
1000
0
1975
1980
1985
1990
1995
2000
2005
2010
2015
Year and month

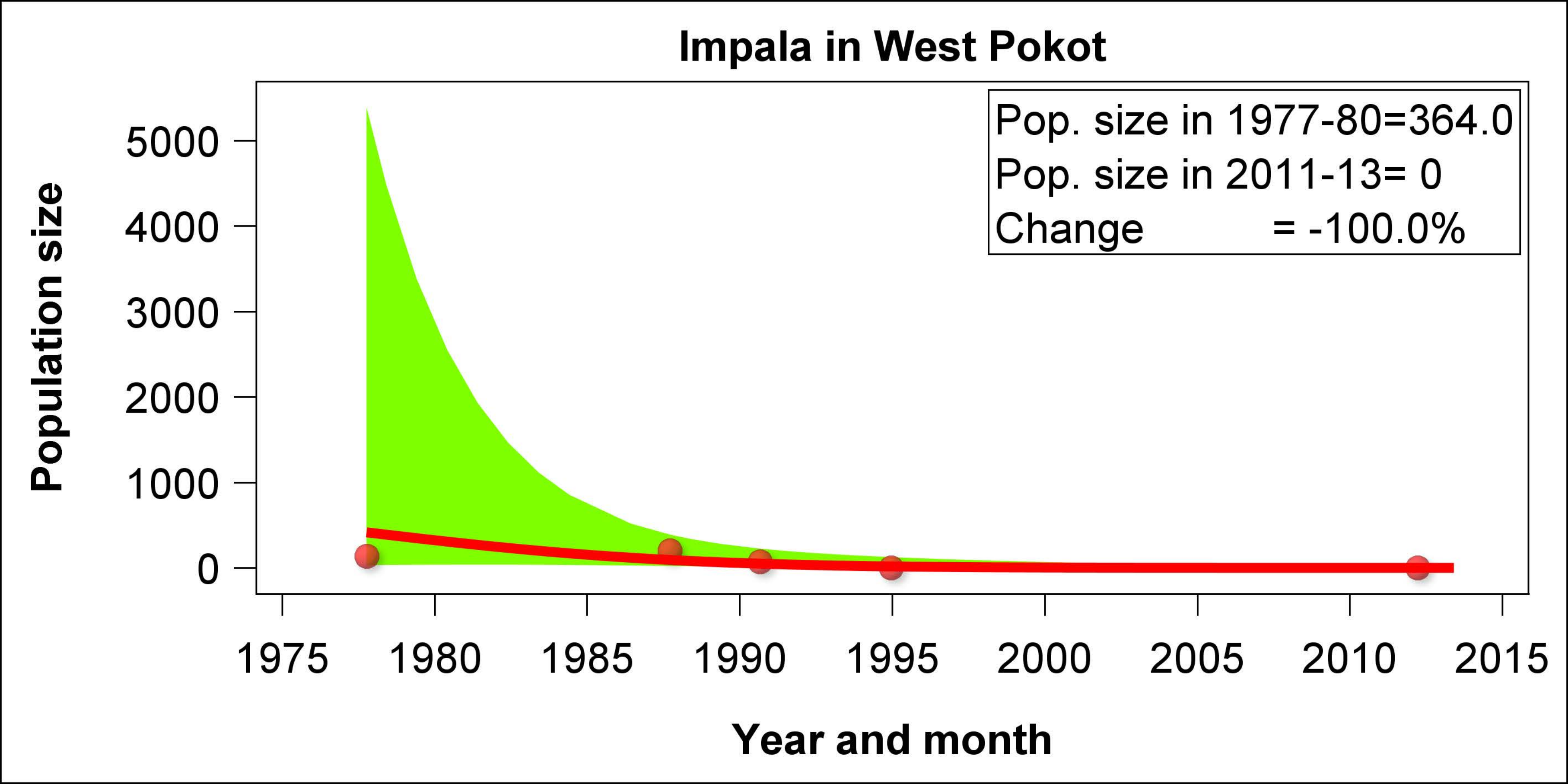
Impala in West Pokot
Pop. size in 1977-80=364.0
Pop. size in 2011-13= 0
Change = -100.0%
Population size
5000
4000
3000
2000
1000
0
1975
1980
1985
1990
1995
2000
2005
2010
2015
Year and month
