# Supplementary material for: Extreme Wildlife Declines and Concurrent Increase in Livestock Numbers in Kenya: What Are the Causes?
Source: PLoS One. 2016 Sep 27;11(9):e0163249. doi: 10.1371/journal.pone.0163249 (PMC5039022; doi:10.1371/journal.pone.0163249)
Supplement: S22 Fig — Numeric data labels refer to population sizes from decadal censuses were conducted in 1962, 1969, 1979, 1989, 1999 and 2009. Population sizes for the remaining years were obtained using interpolation based on a formula developed by Kenya National Bureau of statistics. (PDF) [file pone.0163249.s032.pdf]

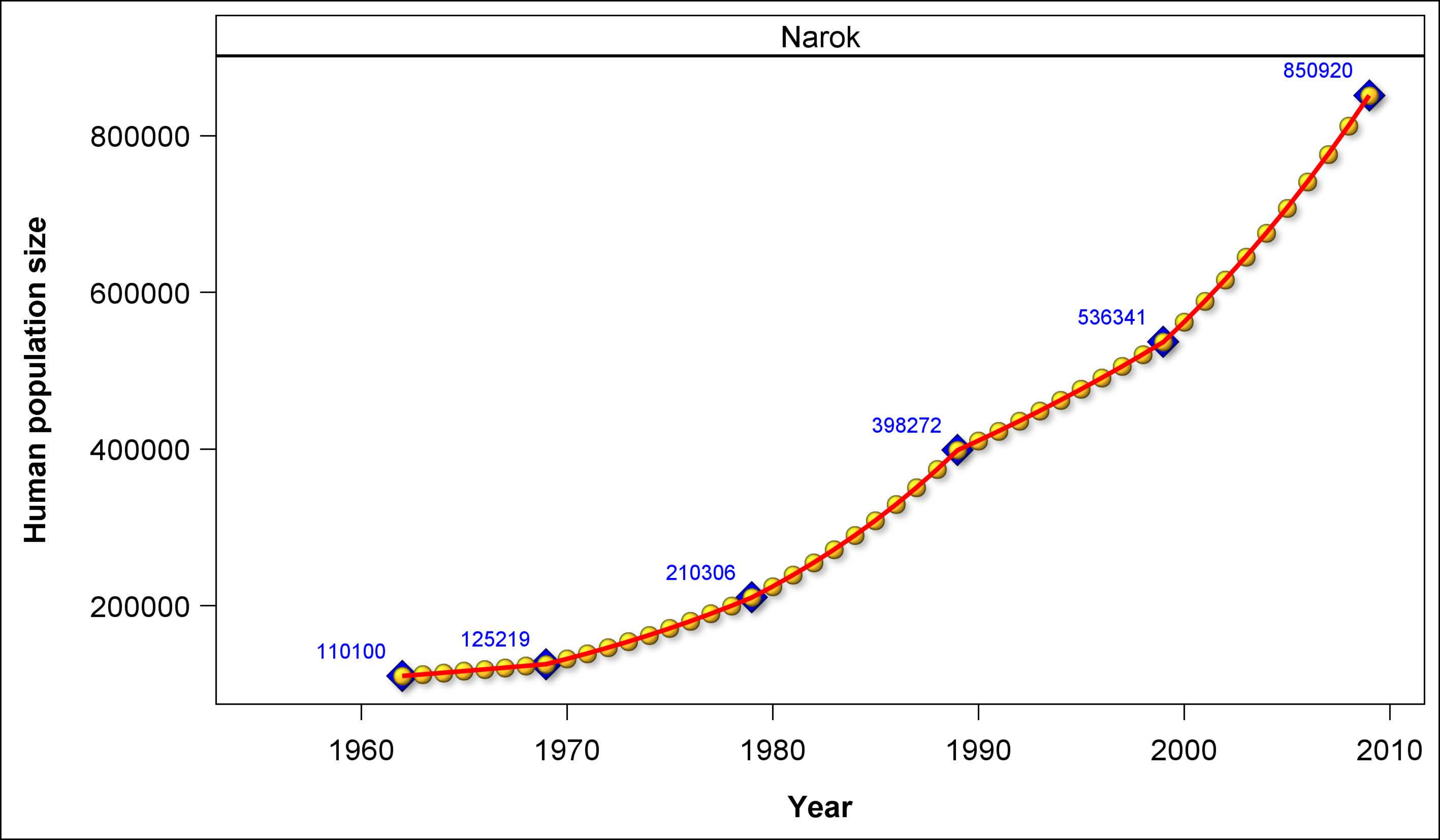

Narok
850920
536341
398272
210306
125219
110100
800000
600000
400000
200000
Human population size
1960
1970
1980
1990
2000
2010
Year

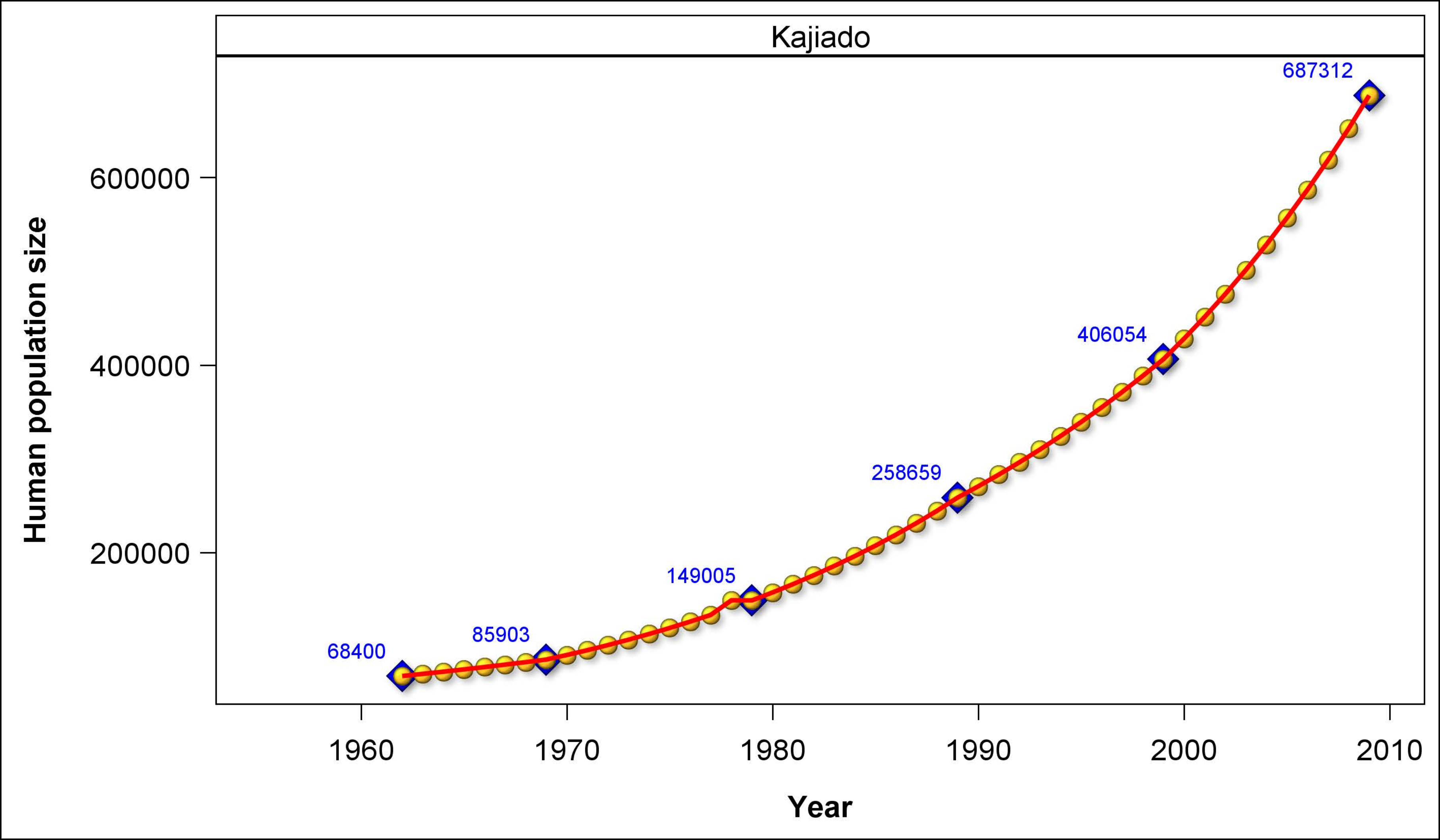
Kajiado
687312
406054
258659
149005
85903
68400
600000
400000
200000
Human population size
1960
1970
1980
1990
2000
2010
Year

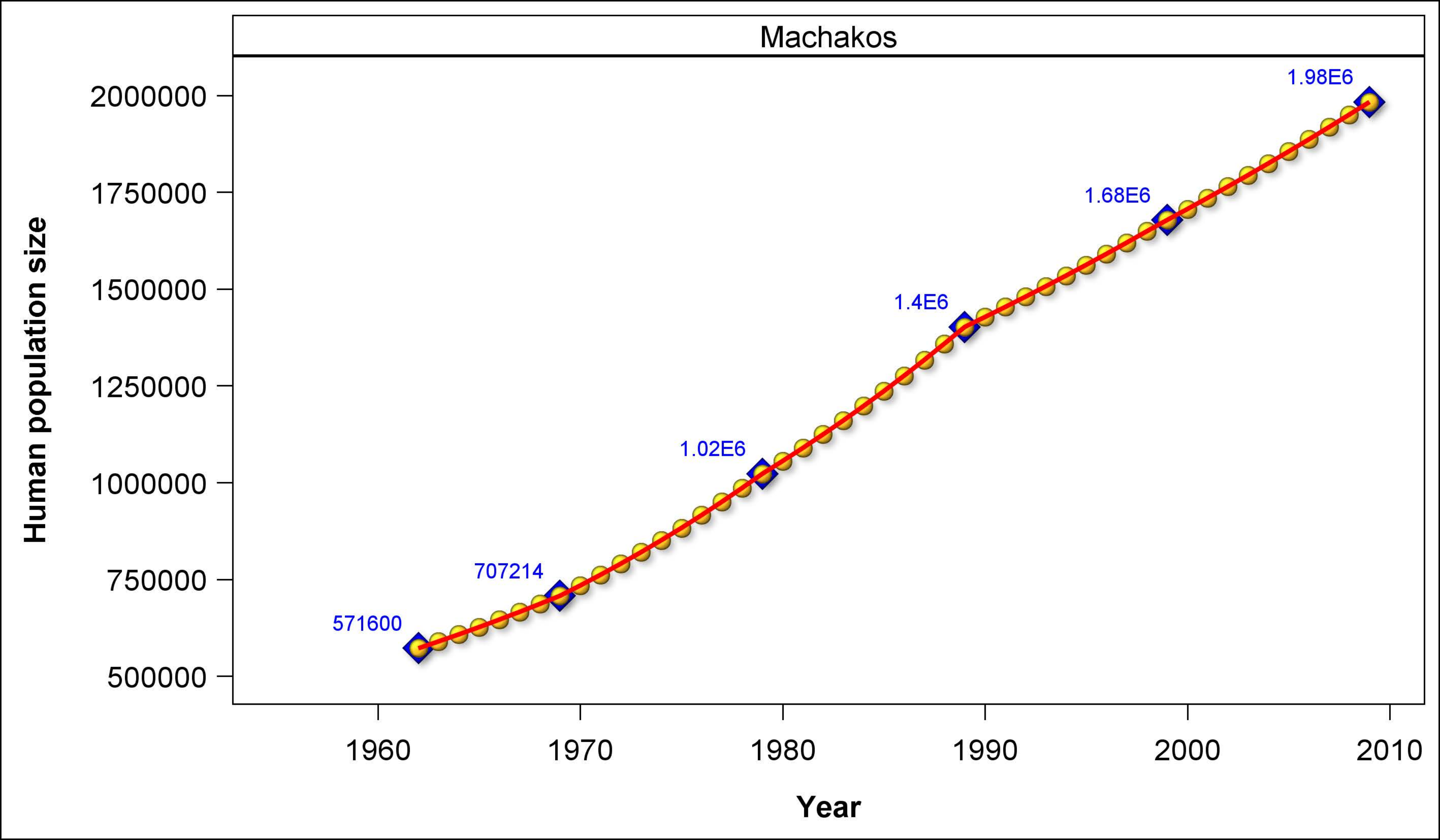

Machakos
Human population size
2000000
1750000
1500000
1250000
1000000
750000
500000
571600
707214
1.02E6
1.4E6
1.68E6
1.98E6
1960
1970
1980
1990
2000
2010
Year

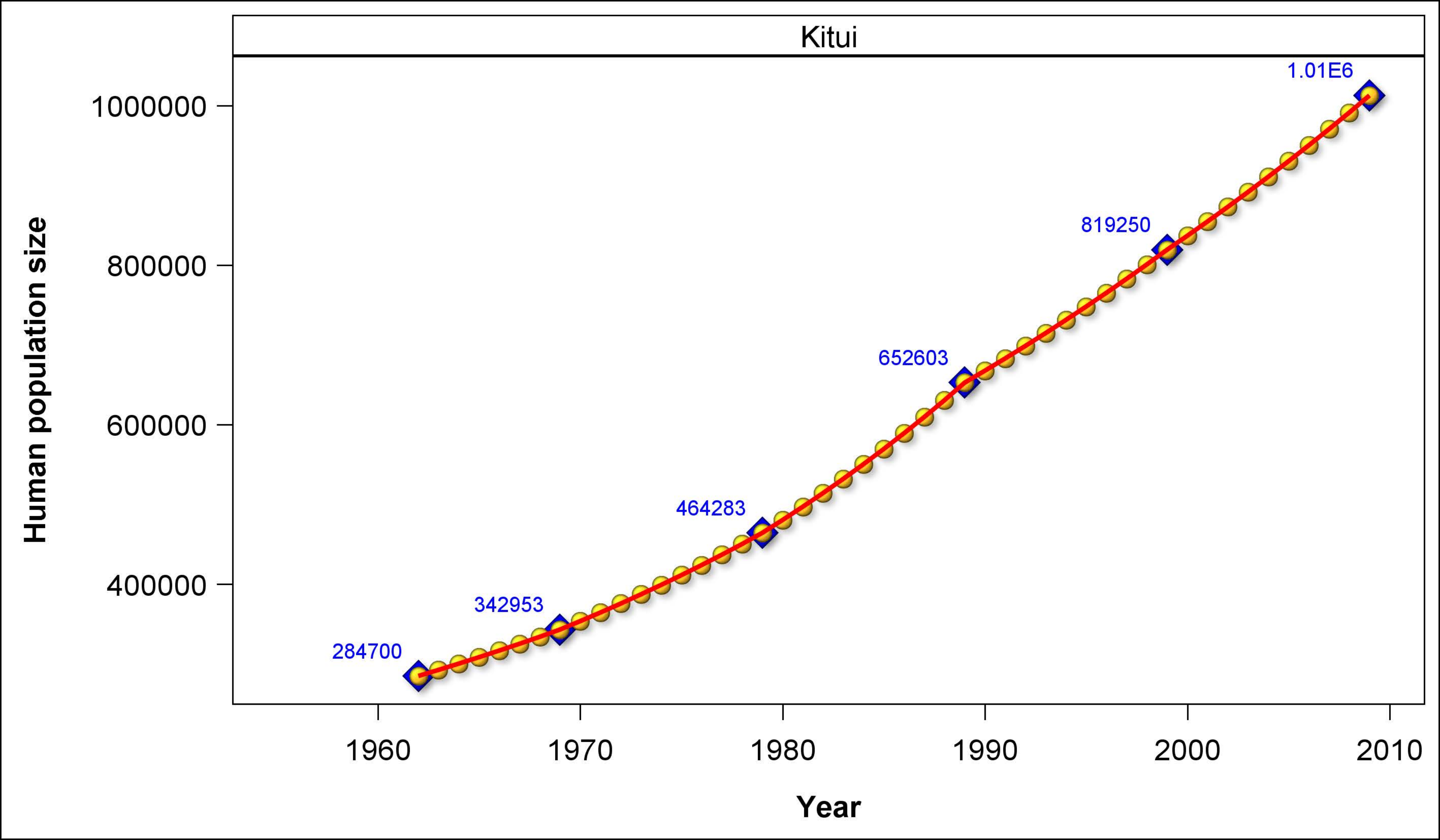
Kitui
Human population size
1000000
800000
600000
400000
1960
1970
1980
1990
2000
2010
Year
284700
342953
464283
652603
819250
1.01E6

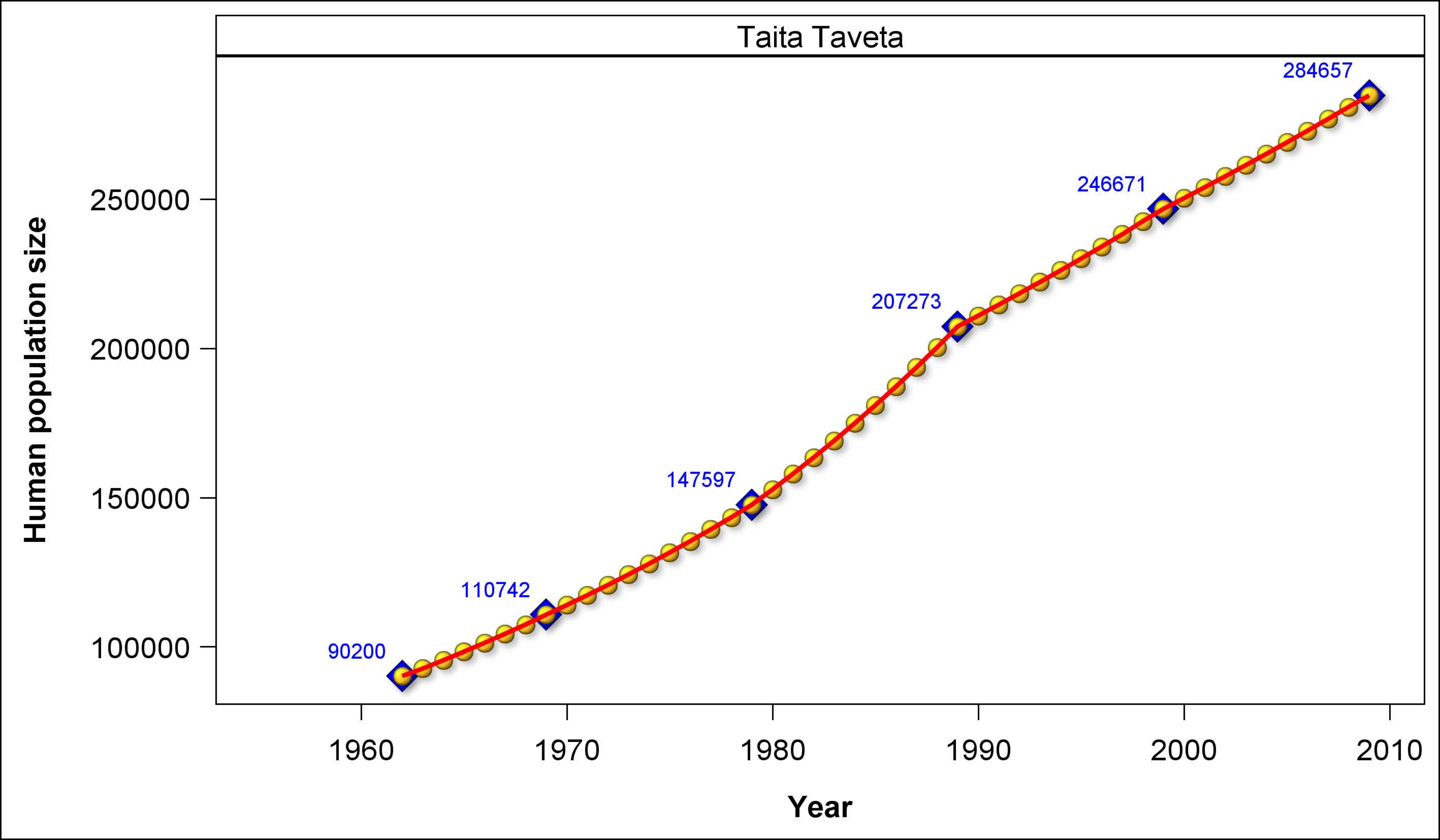

Taita Taveta
Human population size
Year
250000
200000
150000
100000
1960
1970
1980
1990
2000
2010
90200
110742
147597
207273
246671
284657

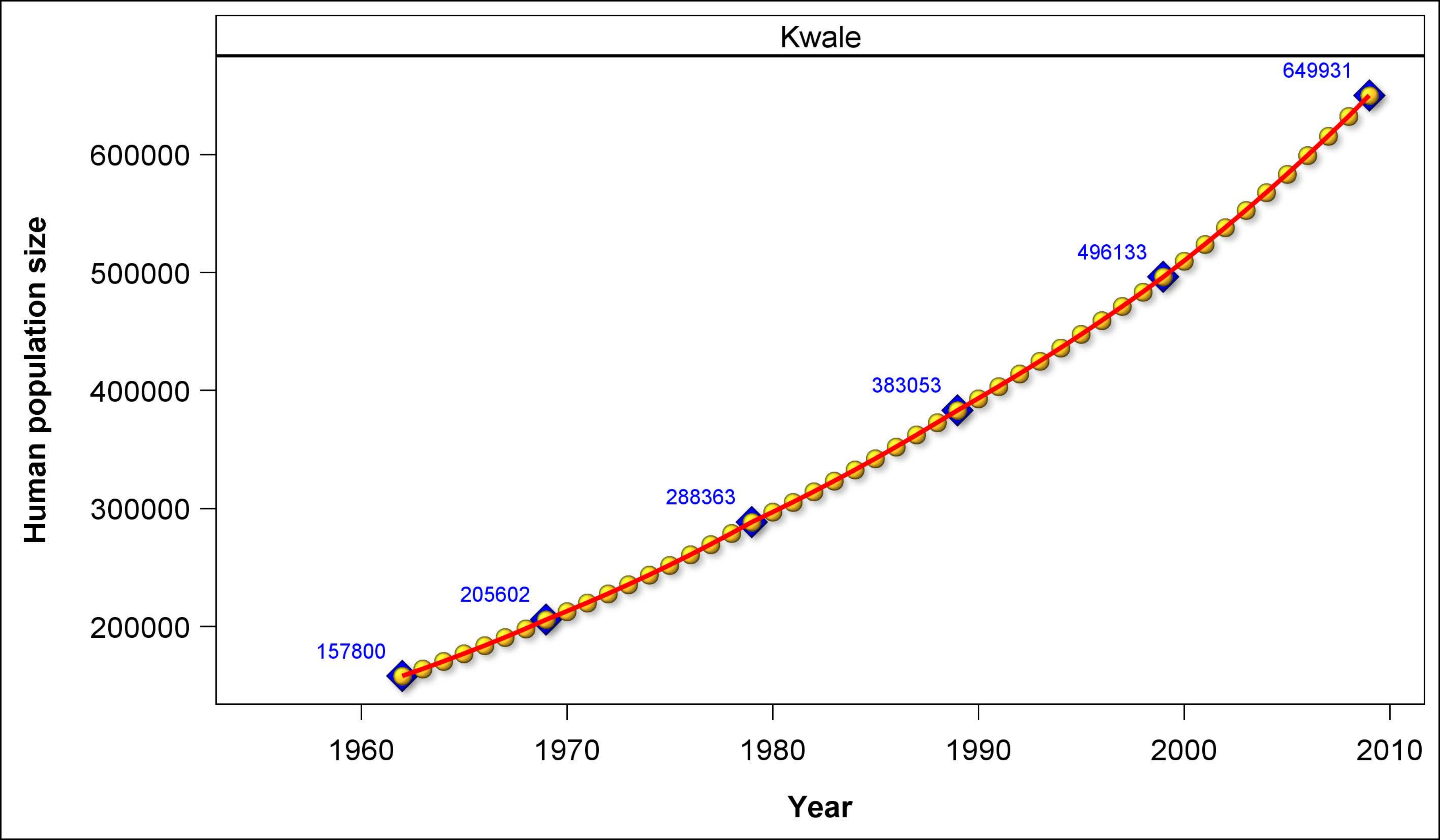
Kwale
Human population size
Year
157800
205602
288363
383053
496133
649931
200000
300000
400000
500000
600000
1960
1970
1980
1990
2000
2010

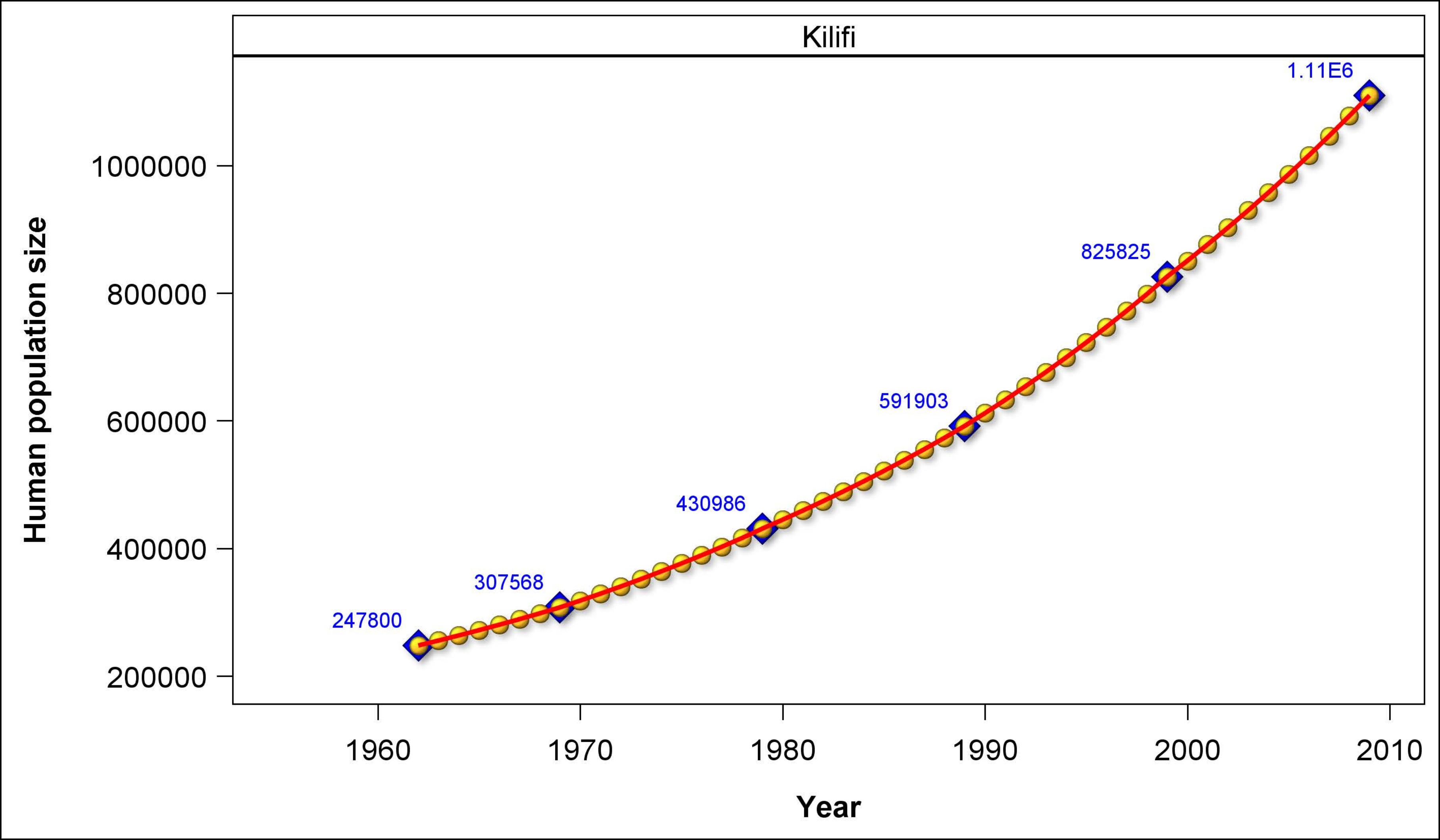
Kilifi
1.11E6
825825
591903
430986
307568
247800
1000000
800000
600000
400000
200000
Human population size
1960
1970
1980
1990
2000
2010
Year

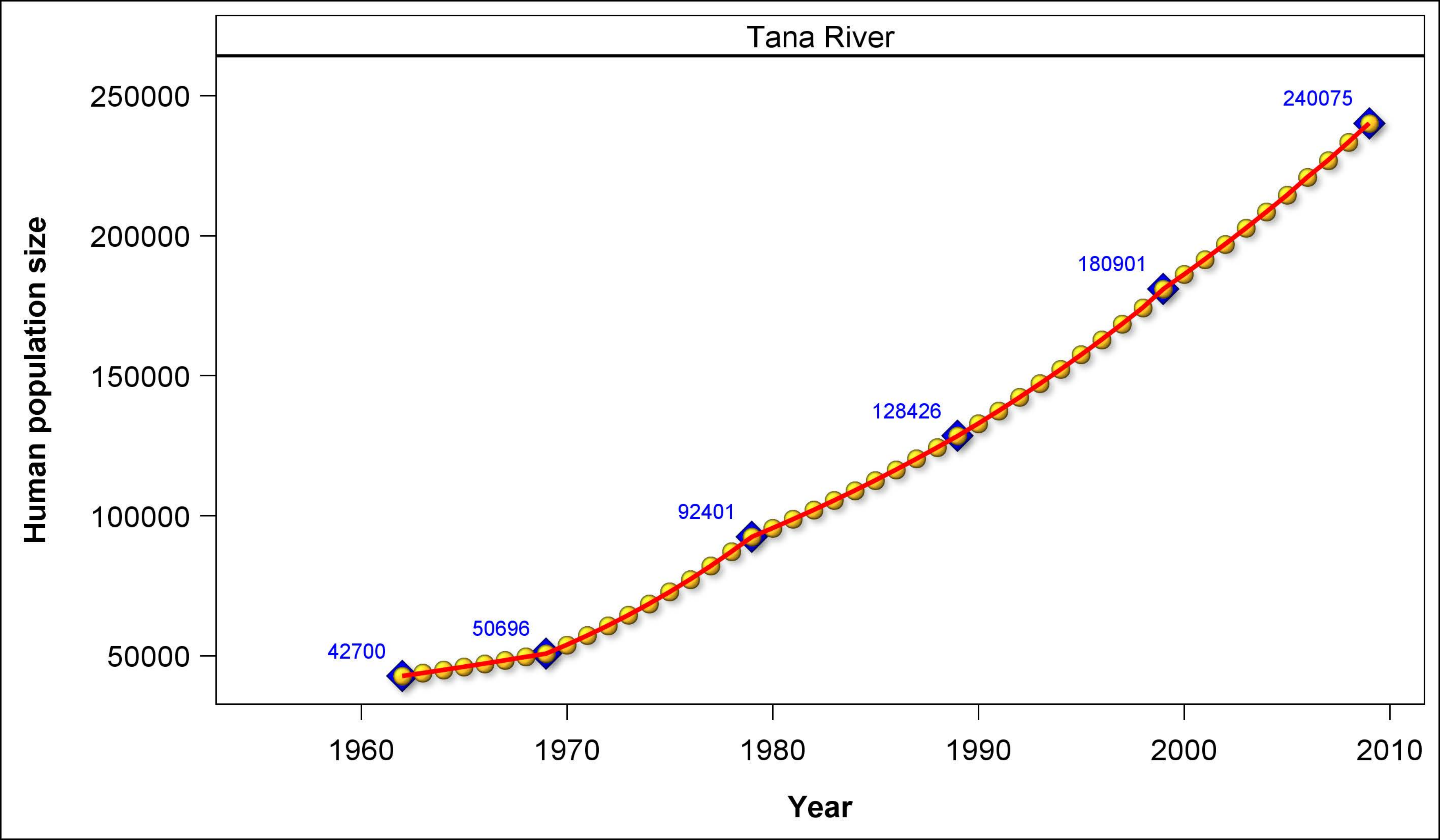
Tana River
250000
200000
150000
100000
50000
Human population size
42700
50696
92401
128426
180901
240075
1960
1970
1980
1990
2000
2010
Year

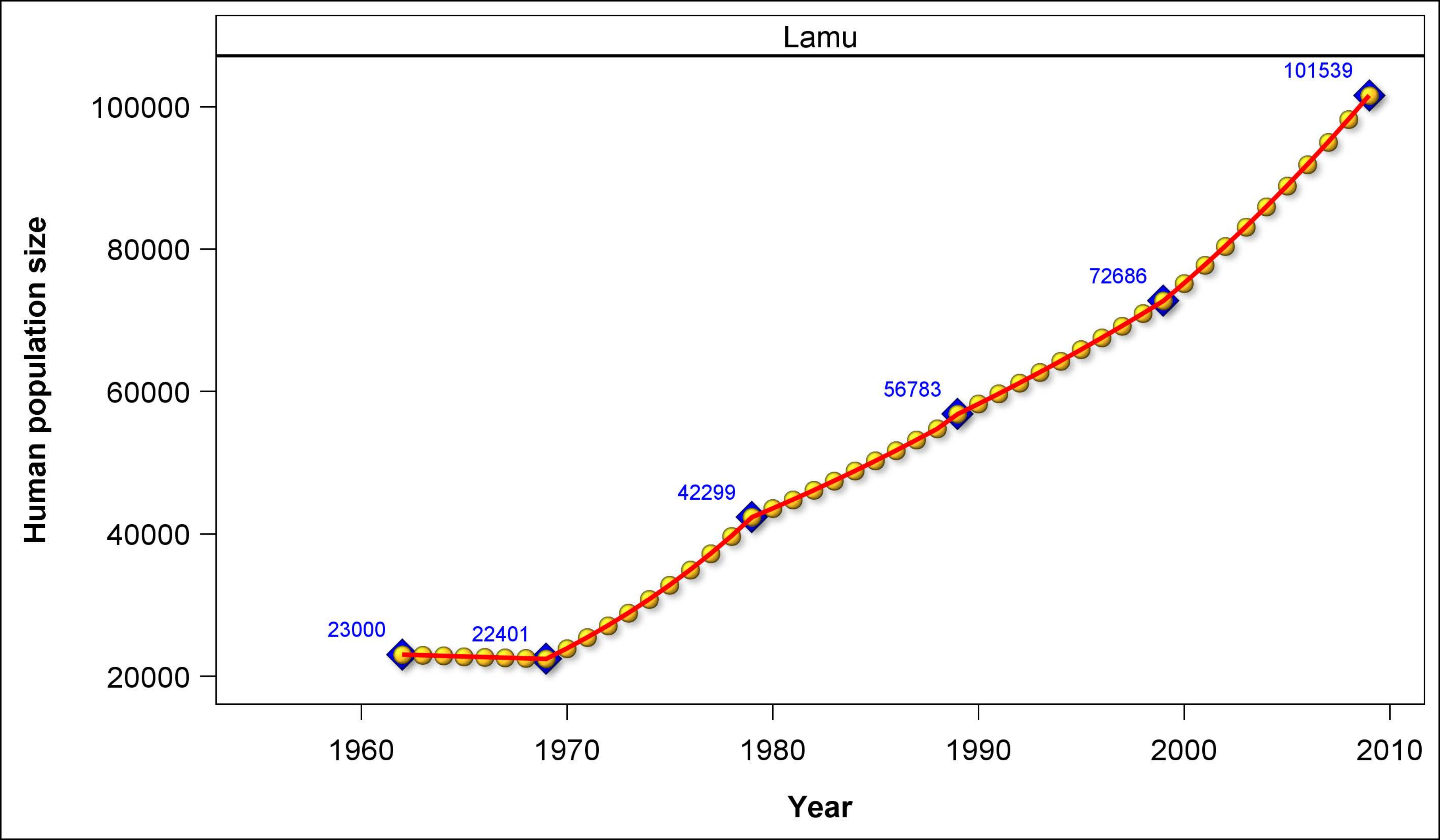
Lamu
Human population size
100000
80000
60000
40000
20000
1960
1970
1980
1990
2000
2010
Year
23000
22401
42299
56783
72686
101539

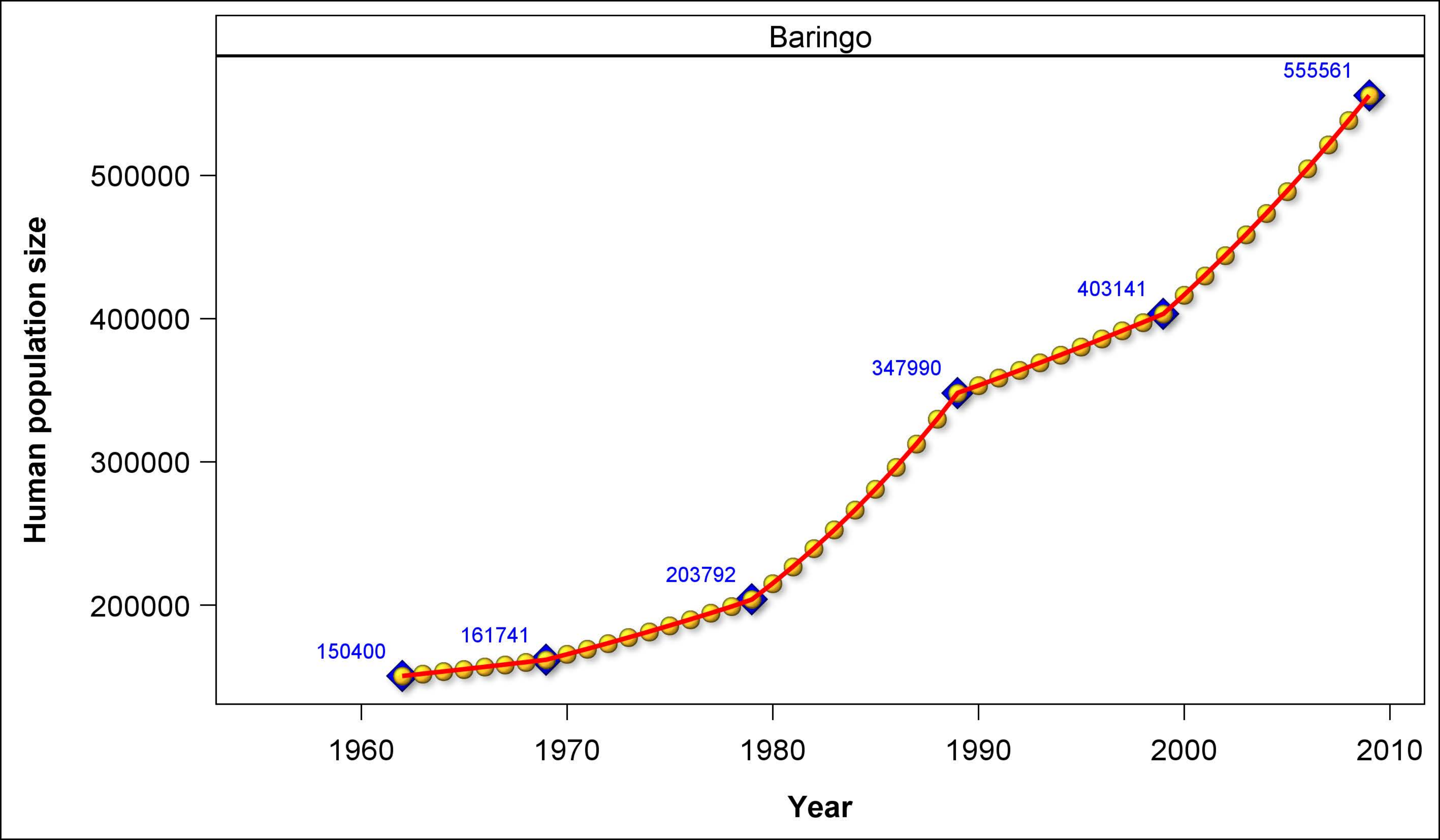

Baringo
555561
403141
347990
203792
161741
150400
500000
400000
300000
200000
1960
1970
1980
1990
2000
2010
Human population size
Year

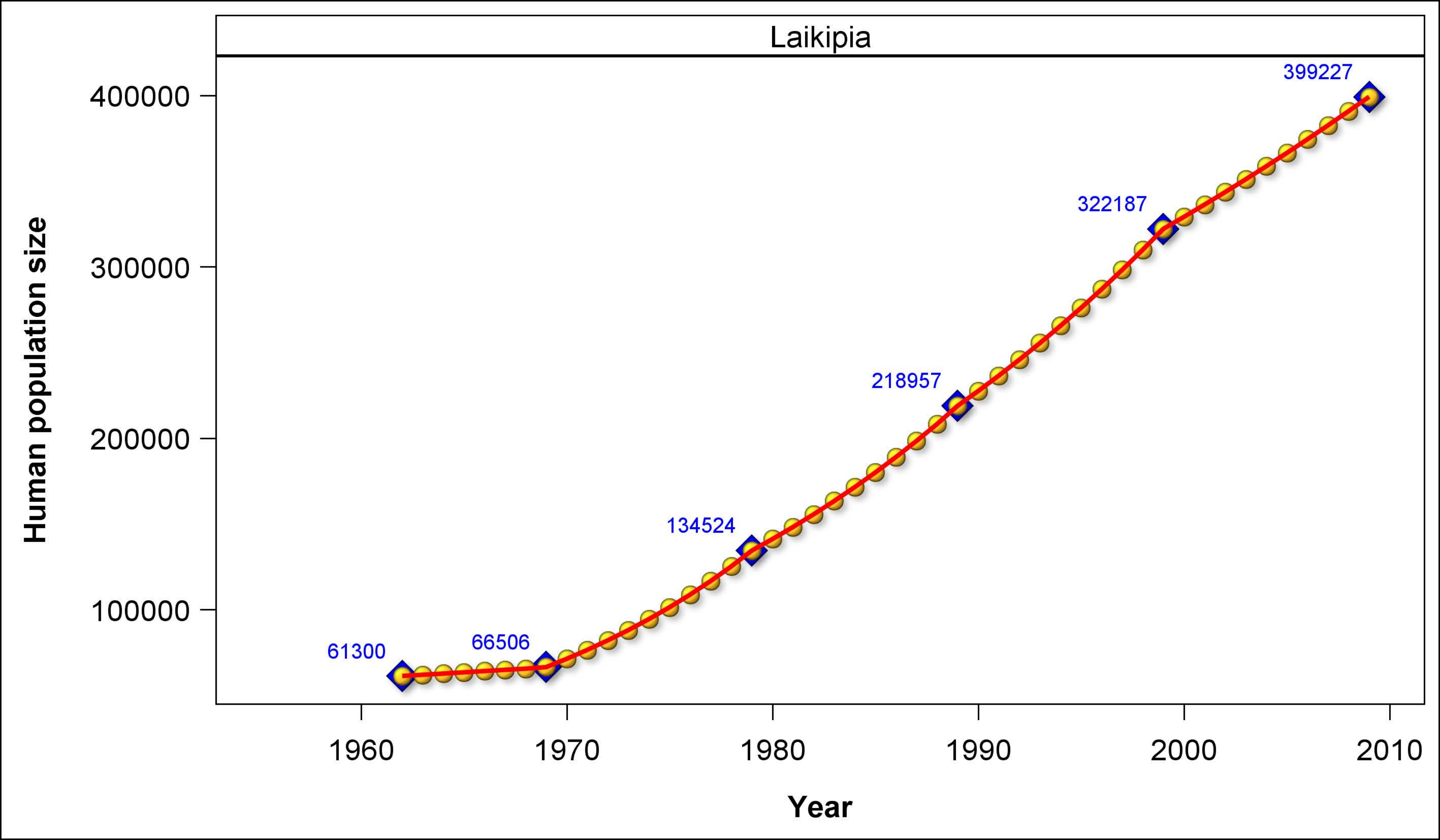
Laikipia
399227
322187
218957
134524
66506
61300
400000
300000
200000
100000
1960
1970
1980
1990
2000
2010
Human population size
Year

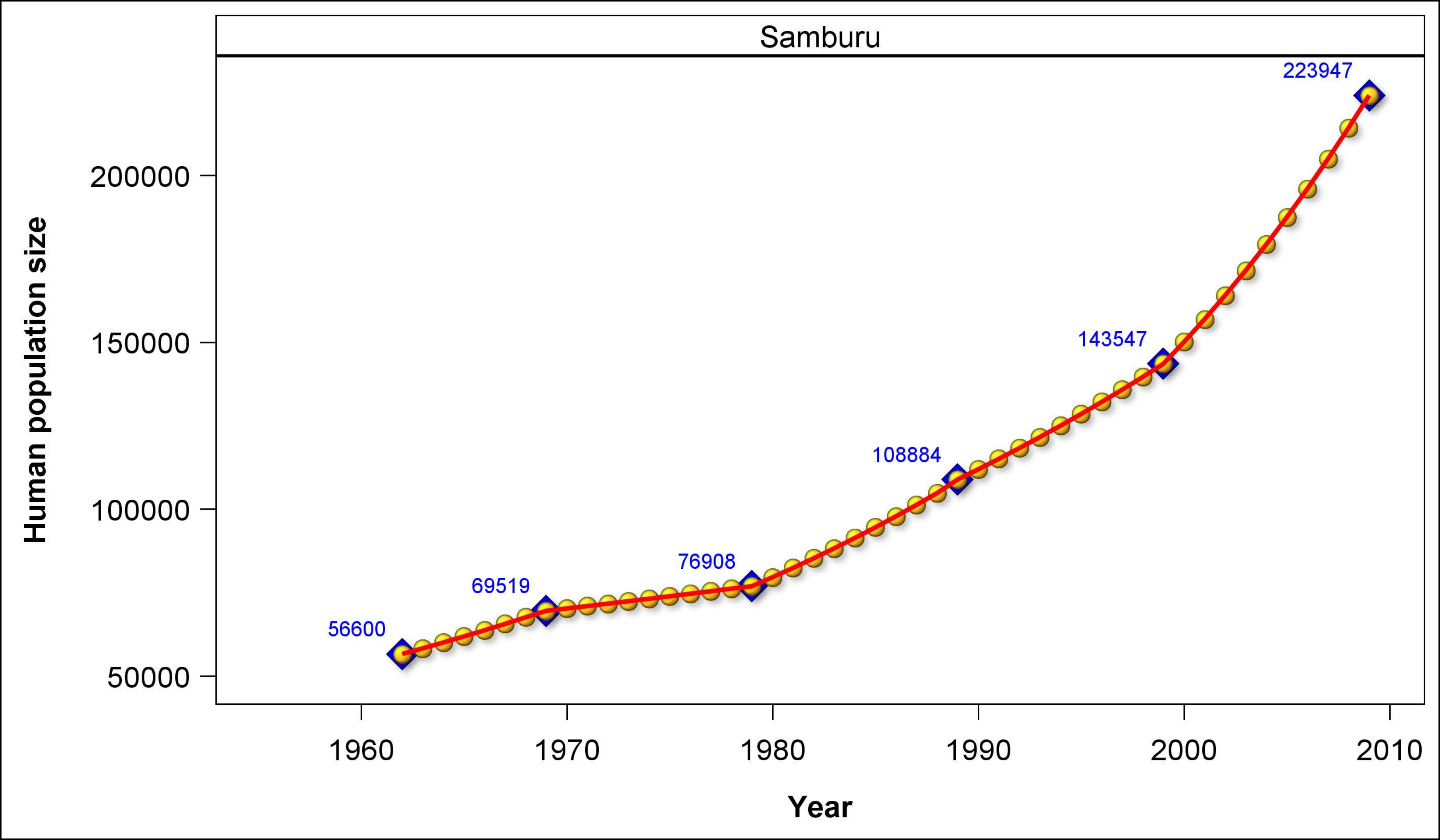

Samburu
223947
143547
108884
76908
69519
56600
200000
150000
100000
50000
Human population size
1960
1970
1980
1990
2000
2010
Year

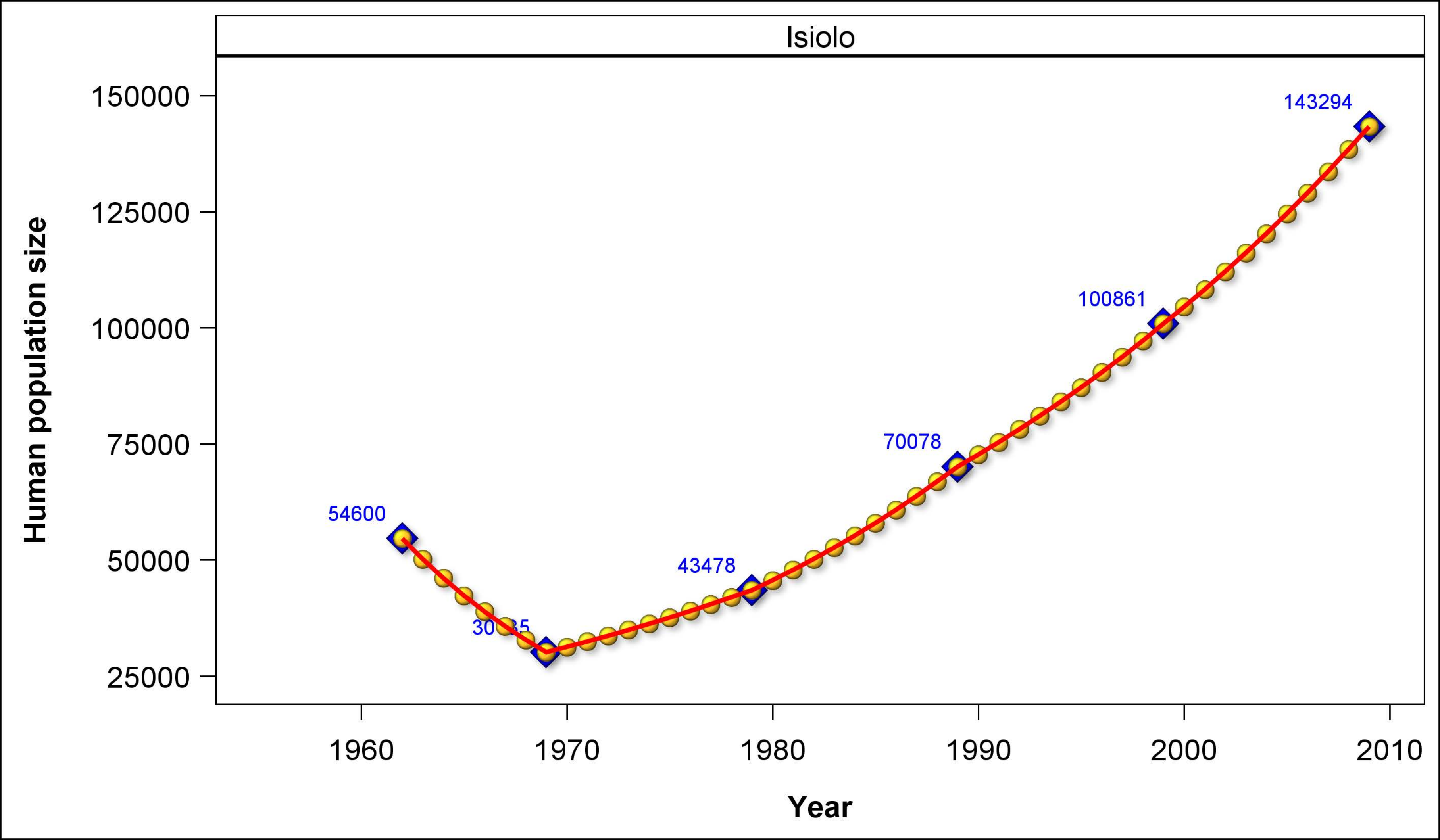
Isiolo
Human population size
150000
125000
100000
75000
50000
25000
Year
1960
1970
1980
1990
2000
2010
54600
43478
70078
100861
143294

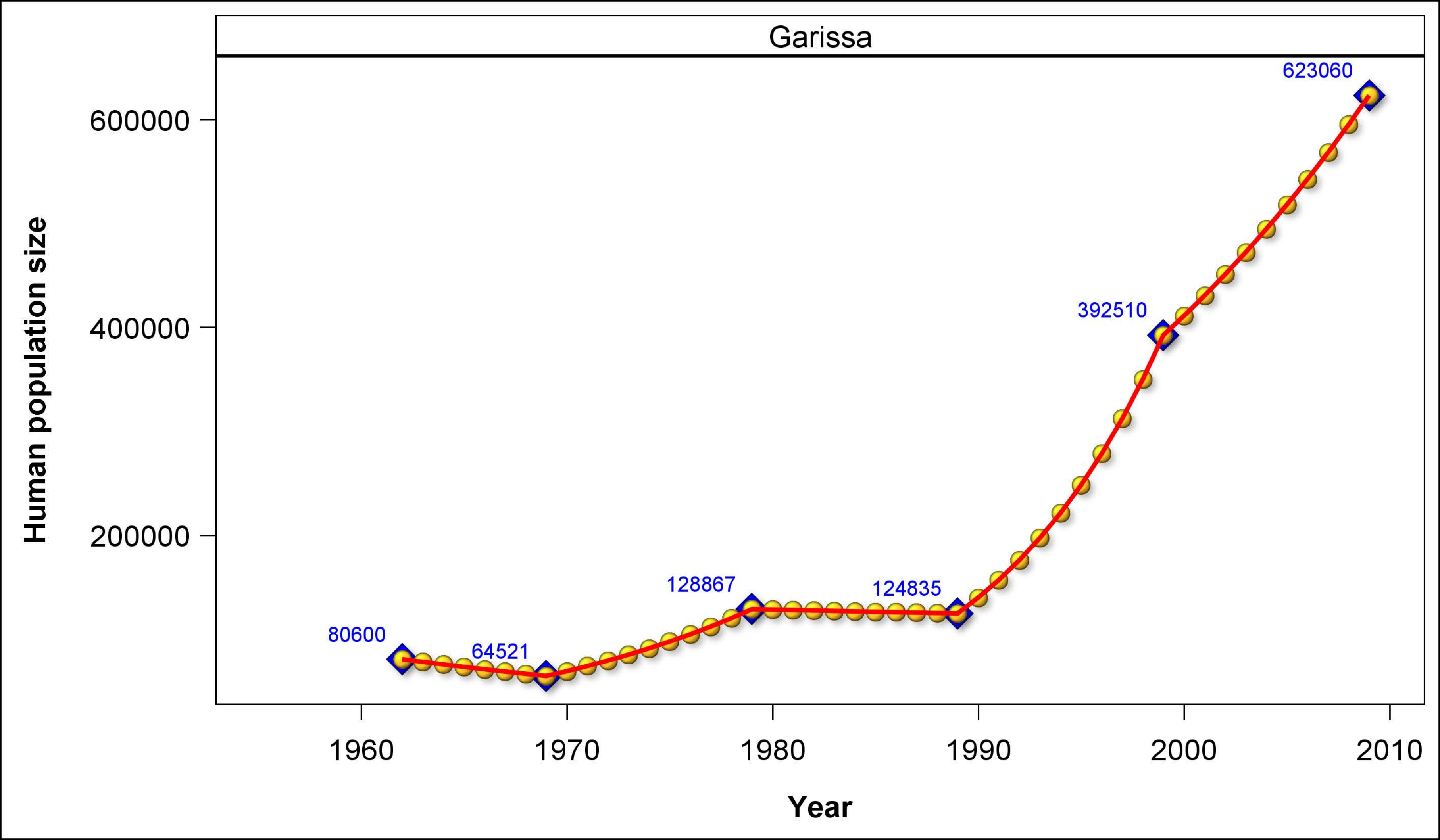

Garissa
623060
600000
392510
400000
200000
128867
124835
80600
64521
1960
1970
1980
1990
2000
2010
Year
Human population size

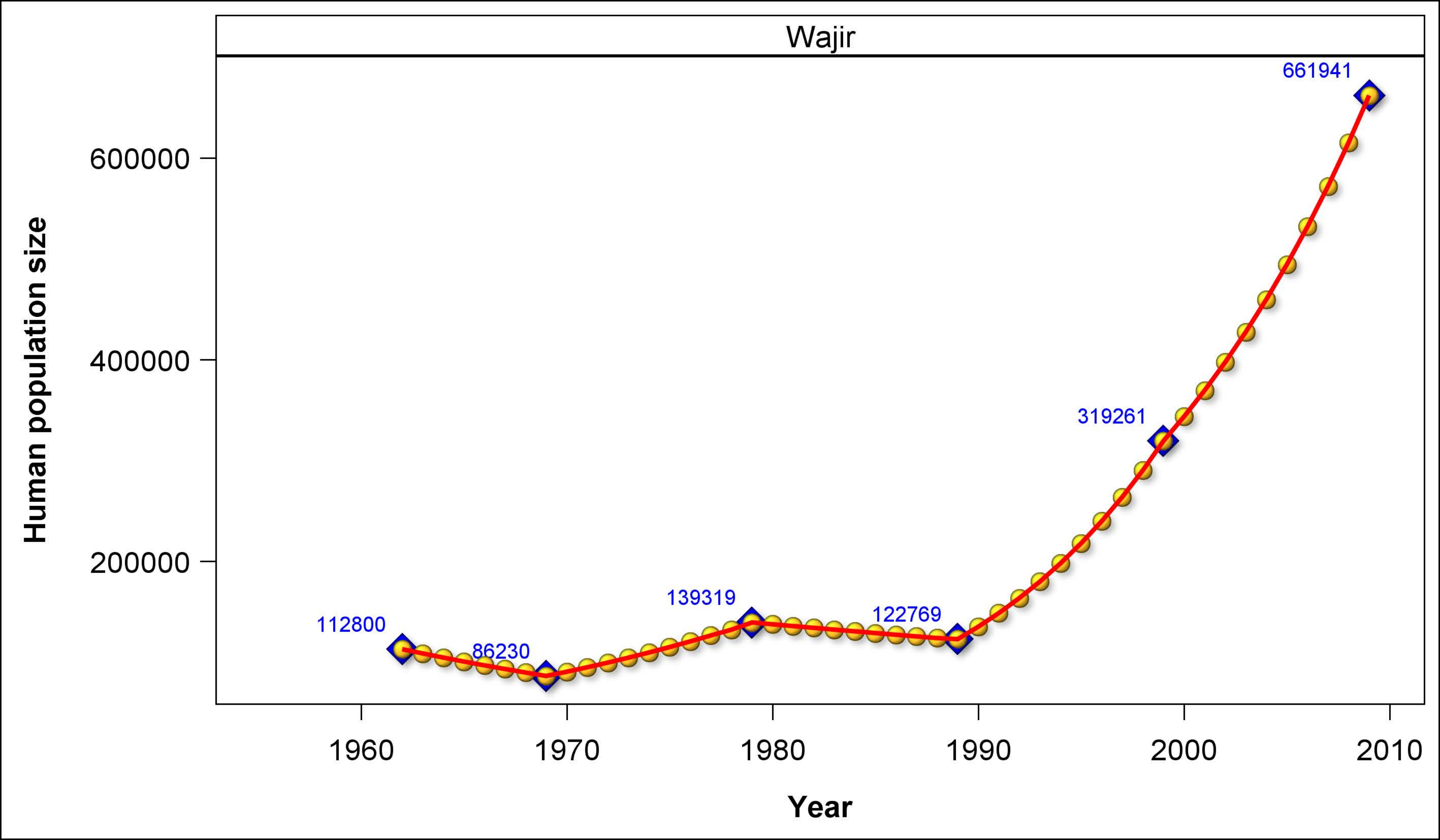

Wajir
Human population size
600000
400000
200000
661941
319261
139319
122769
112800
86230
1960
1970
1980
1990
2000
2010
Year

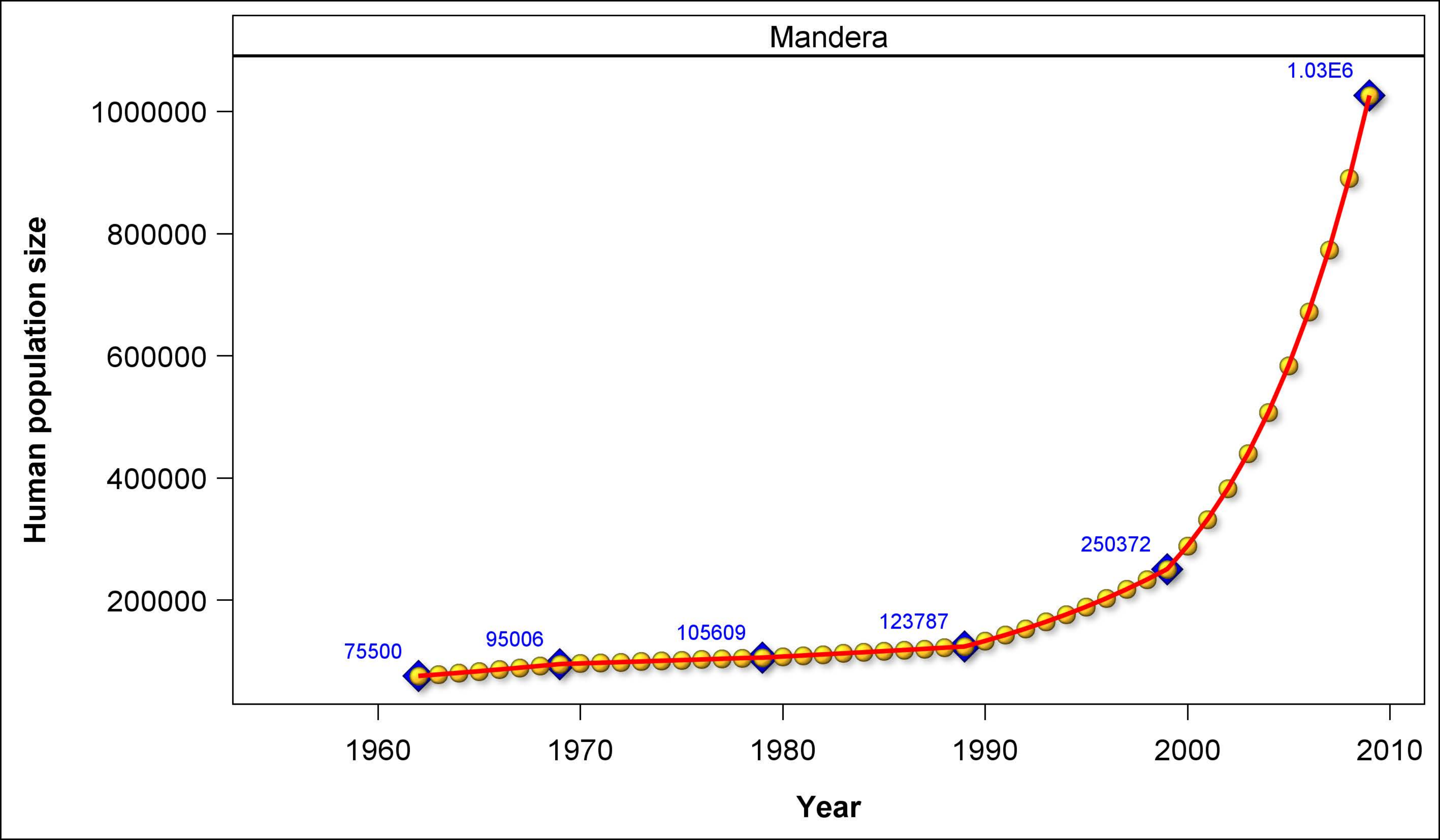
Mandera
Human population size
1000000
800000
600000
400000
200000
1.03E6
250372
123787
105609
95006
75500
1960
1970
1980
1990
2000
2010
Year

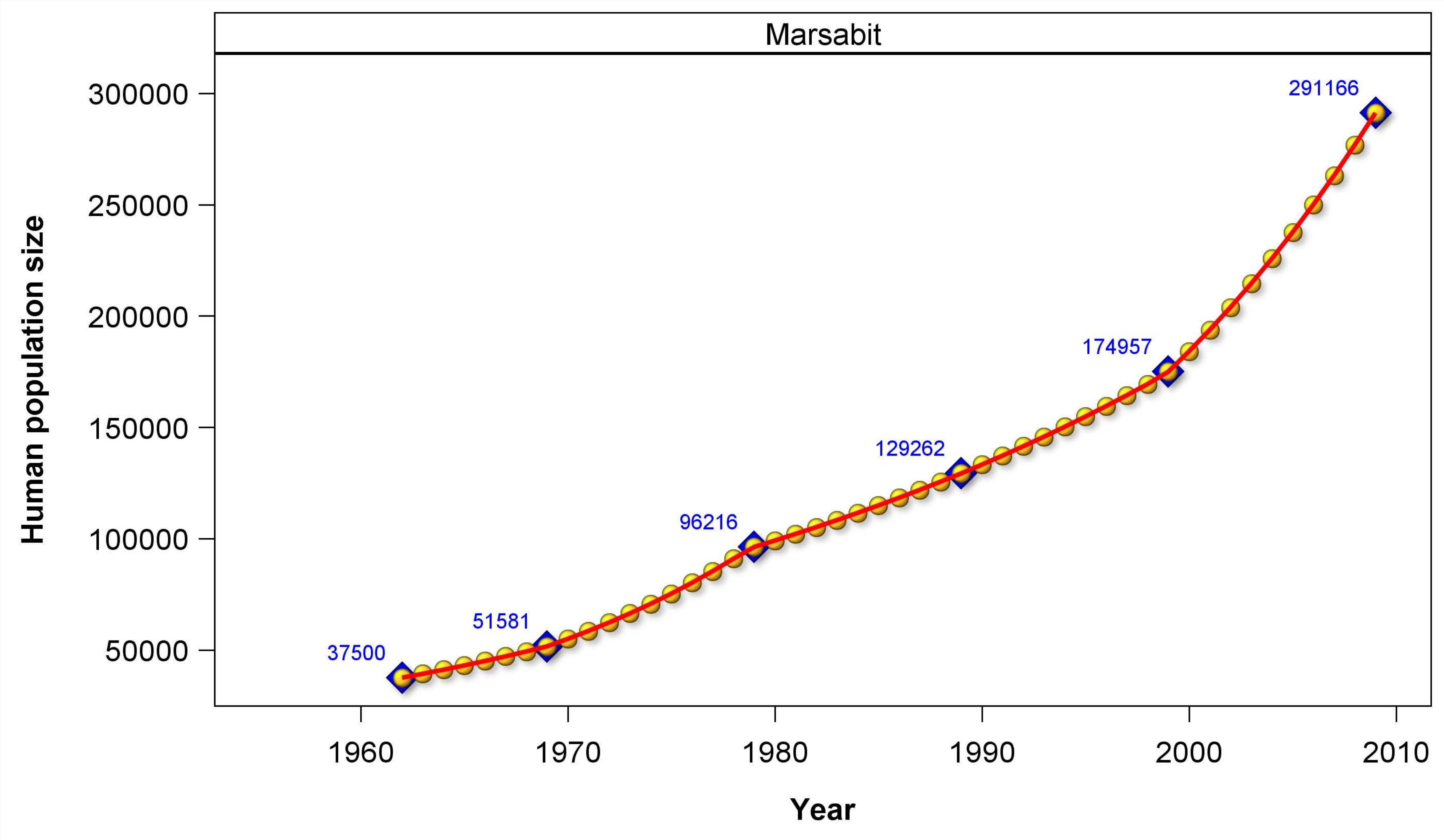
Marsabit
Human population size
300000
250000
200000
150000
100000
50000
1960
1970
1980
1990
2000
2010
Year
37500
51581
96216
129262
174957
291166

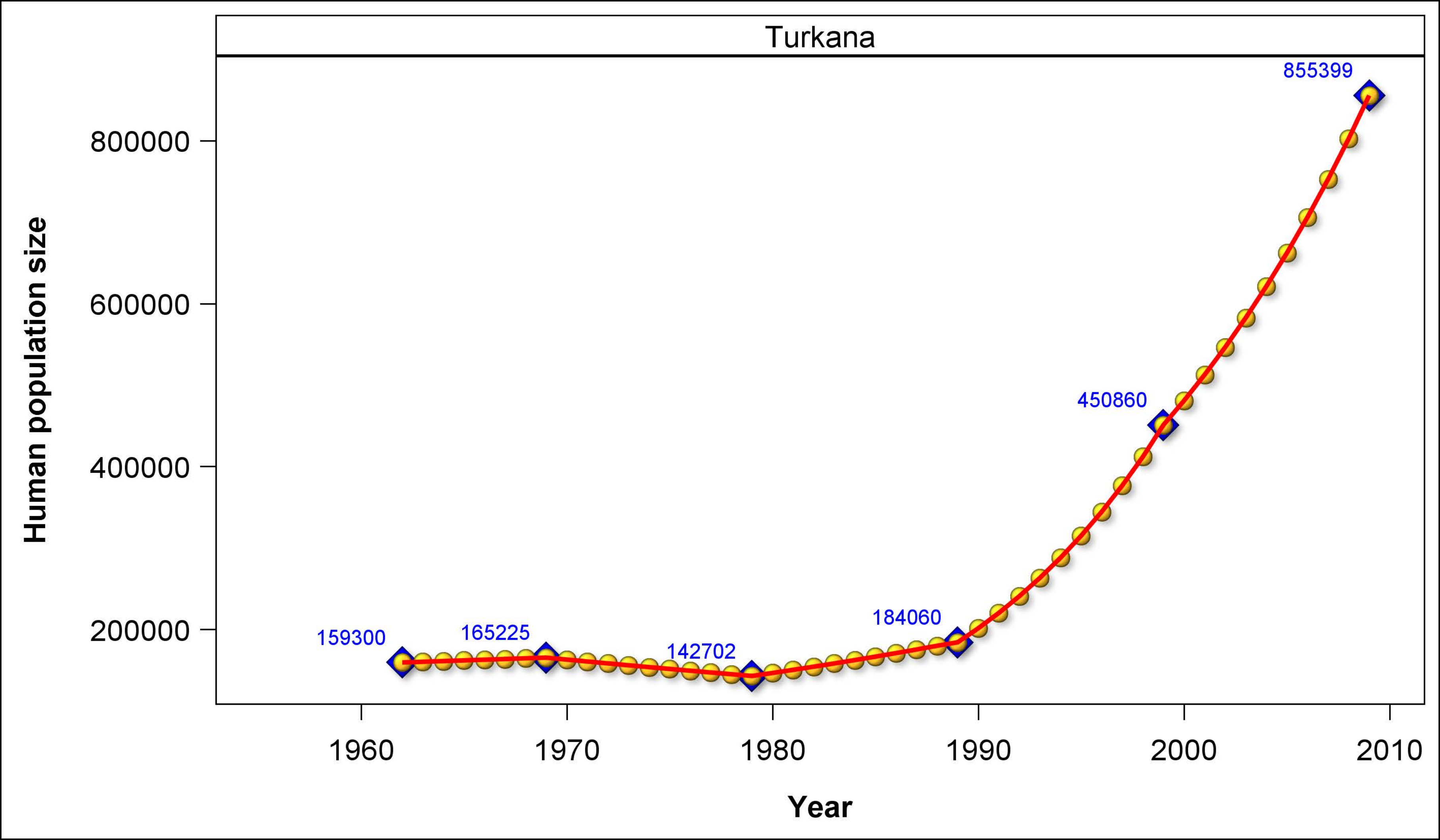

Turkana
Human population size
800000
600000
400000
200000
1960
1970
1980
1990
2000
2010
Year
855399
450860
184060
159300
165225
142702

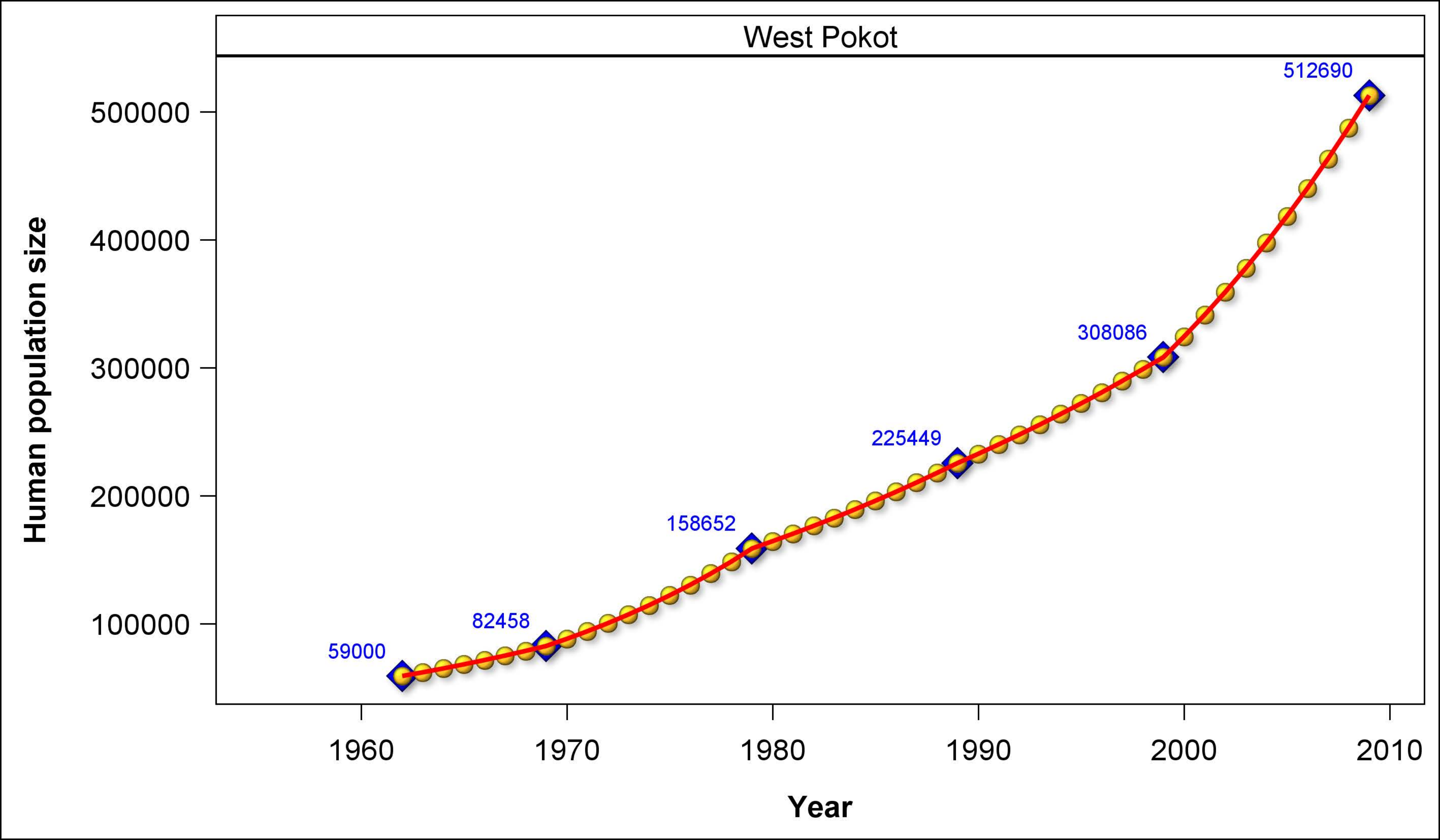
West Pokot
Human population size
Year
500000
400000
300000
200000
100000
1960
1970
1980
1990
2000
2010
59000
82458
158652
225449
308086
512690

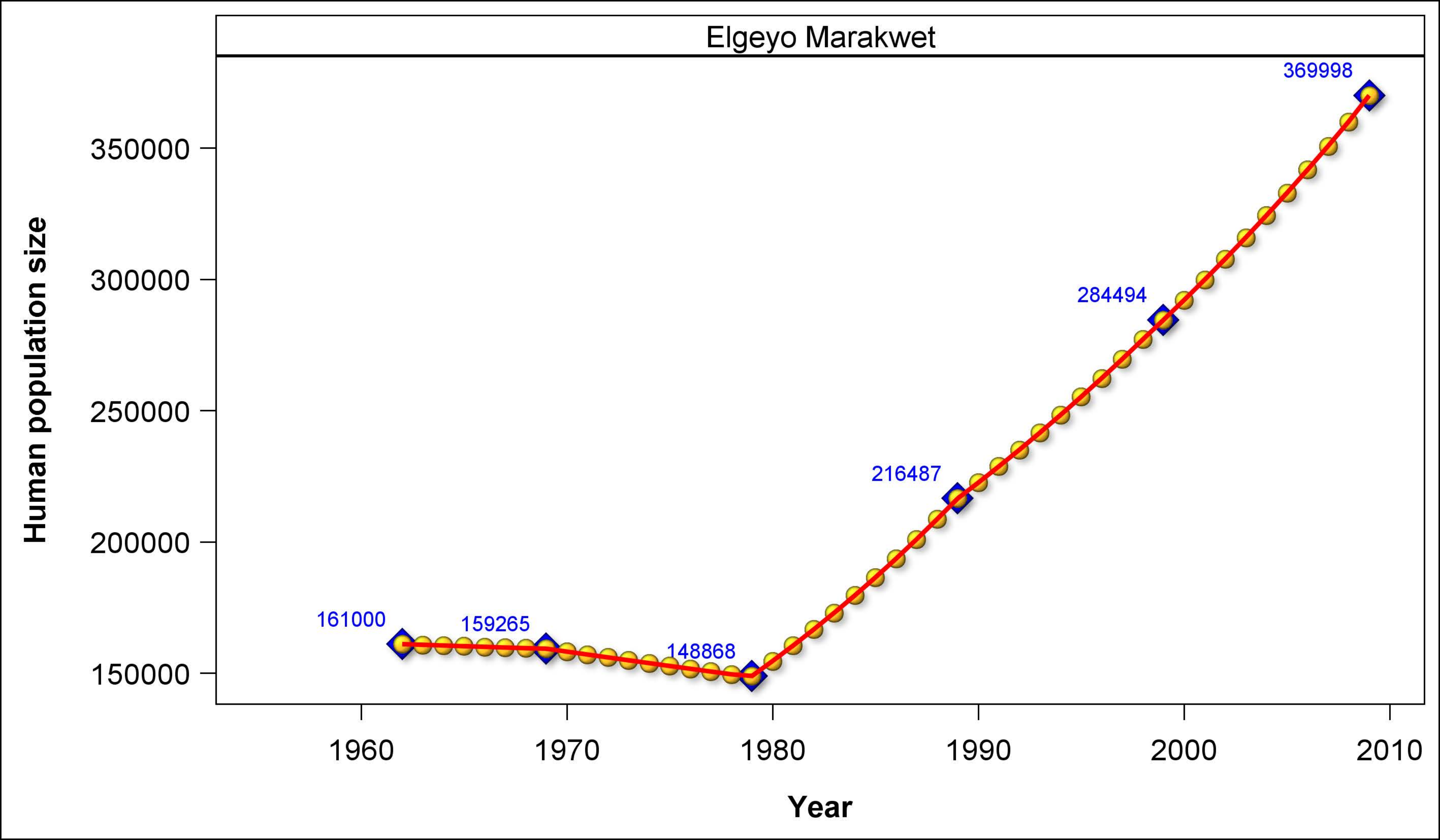

Elgeyo Marakwet
Human population size
Year
350000
300000
250000
200000
150000
1960
1970
1980
1990
2000
2010
161000
159265
148868
216487
284494
369998
