# Supplementary material for: Extreme Wildlife Declines and Concurrent Increase in Livestock Numbers in Kenya: What Are the Causes?
Source: PLoS One. 2016 Sep 27;11(9):e0163249. doi: 10.1371/journal.pone.0163249 (PMC5039022; doi:10.1371/journal.pone.0163249)
Supplement: S23 Fig — The filled goldenrod circles denote the observations, the solid magenta curve the fitted trend curve whereas the cadet blue band the pointwise 95% confidence band. (PDF) [file pone.0163249.s033.pdf]

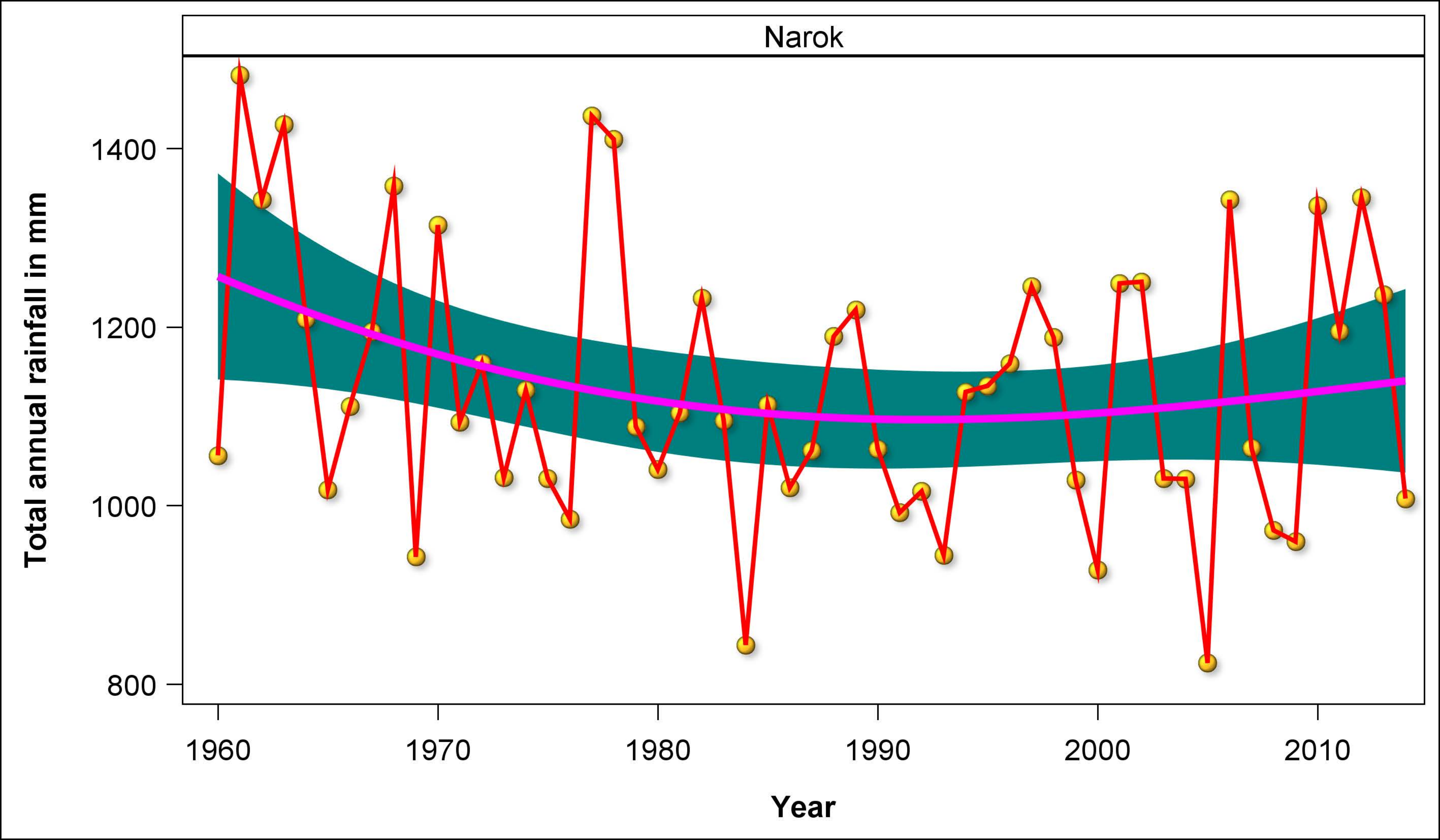
Narok
Total annual rainfall in mm
1400
1200
1000
800
1960
1970
1980
1990
2000
2010
Year

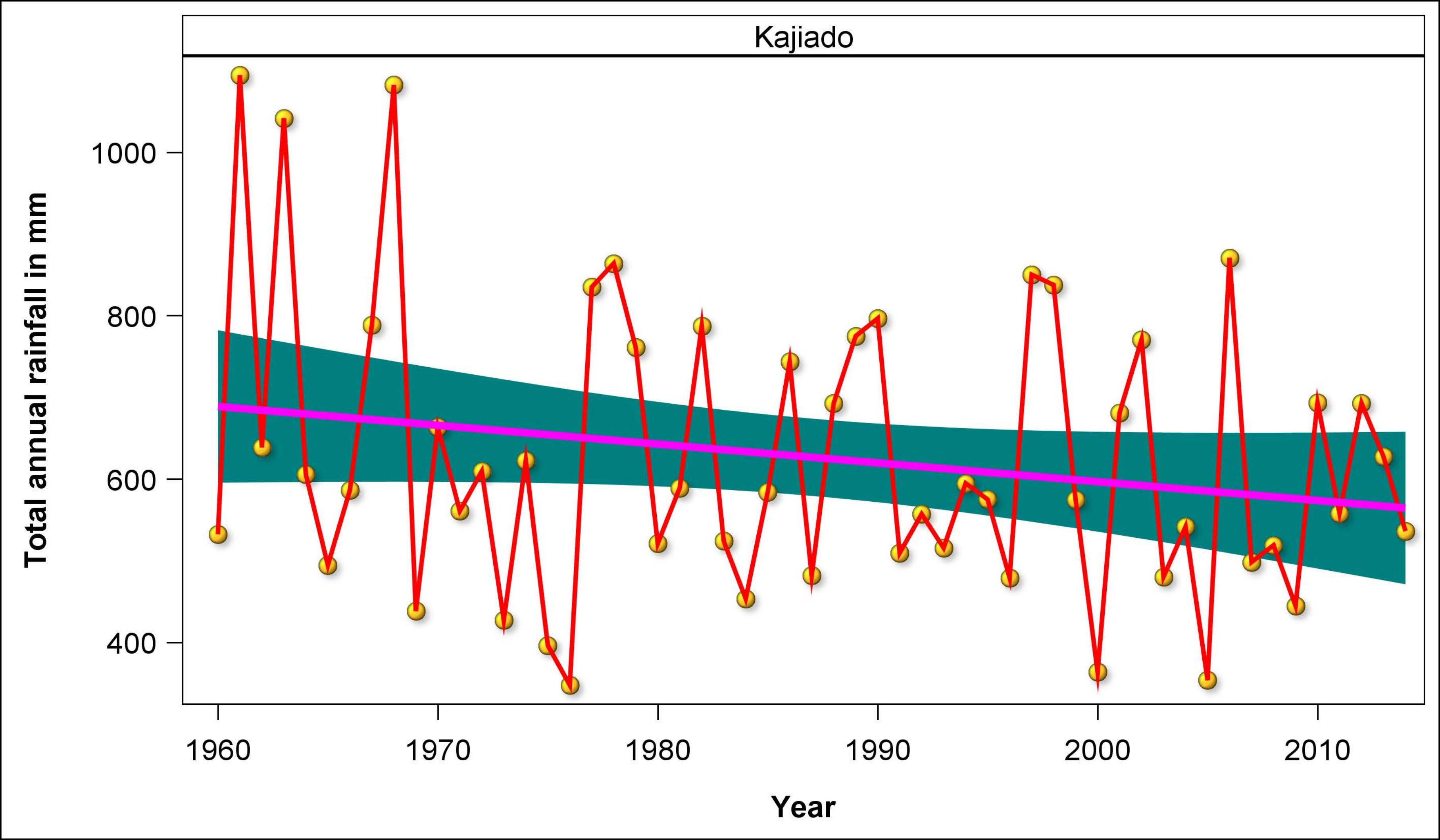

Kajiado
Total annual rainfall in mm
1000
800
600
400
1960
1970
1980
1990
2000
2010
Year

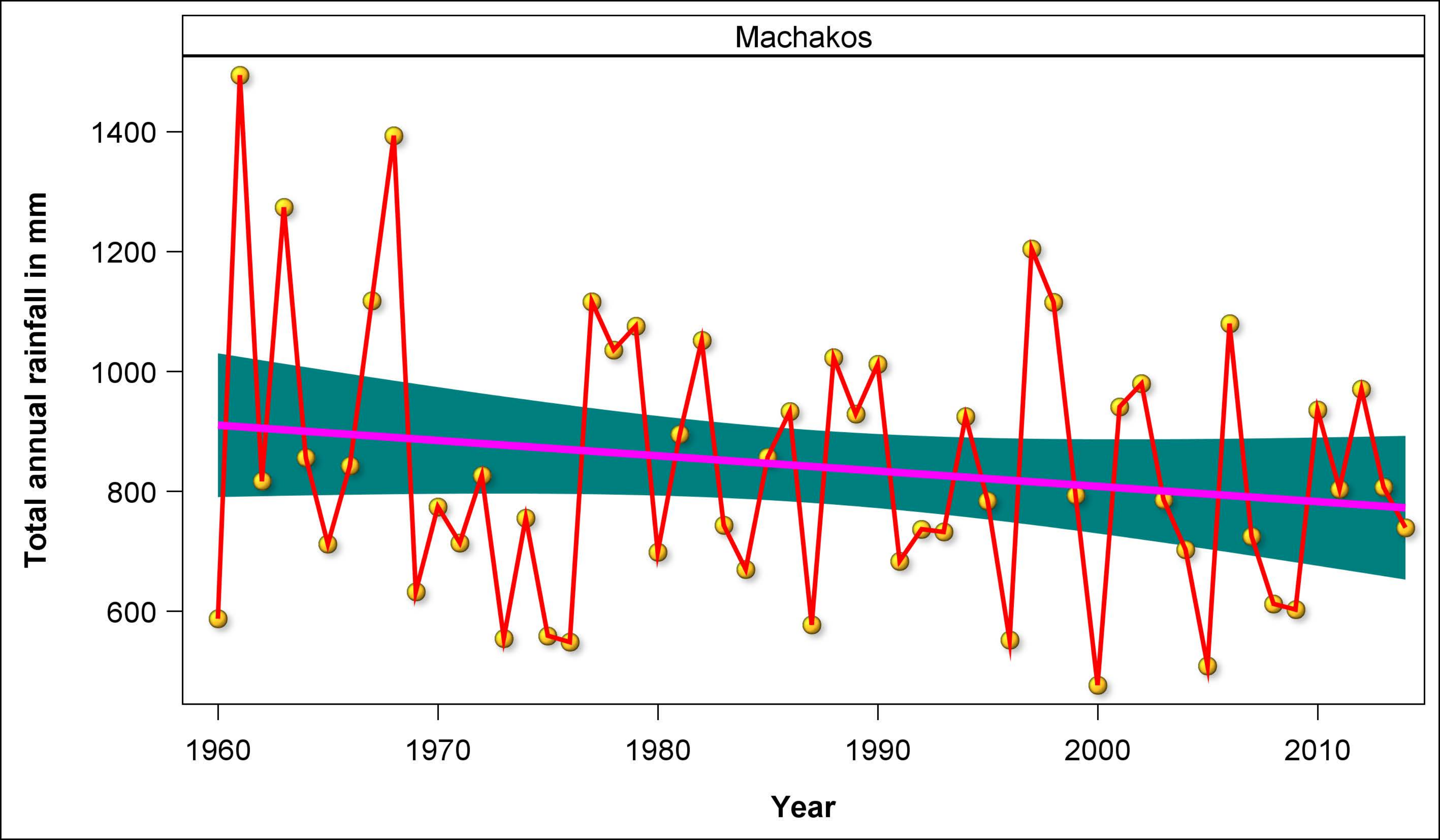
Machakos
Total annual rainfall in mm
1400
1200
1000
800
600
1960
1970
1980
1990
2000
2010
Year

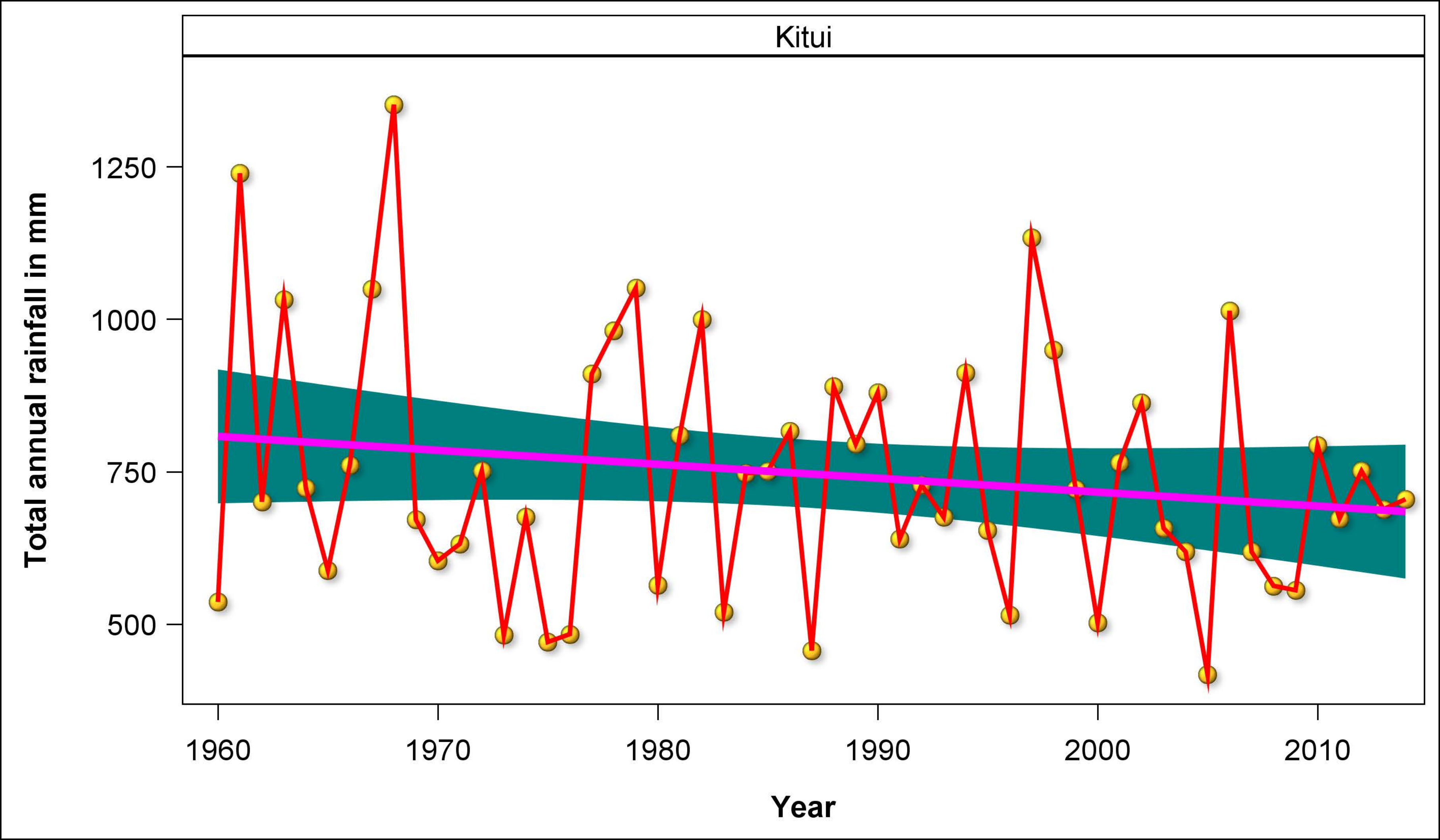
Kitui
Total annual rainfall in mm
1250
1000
750
500
1960
1970
1980
1990
2000
2010
Year

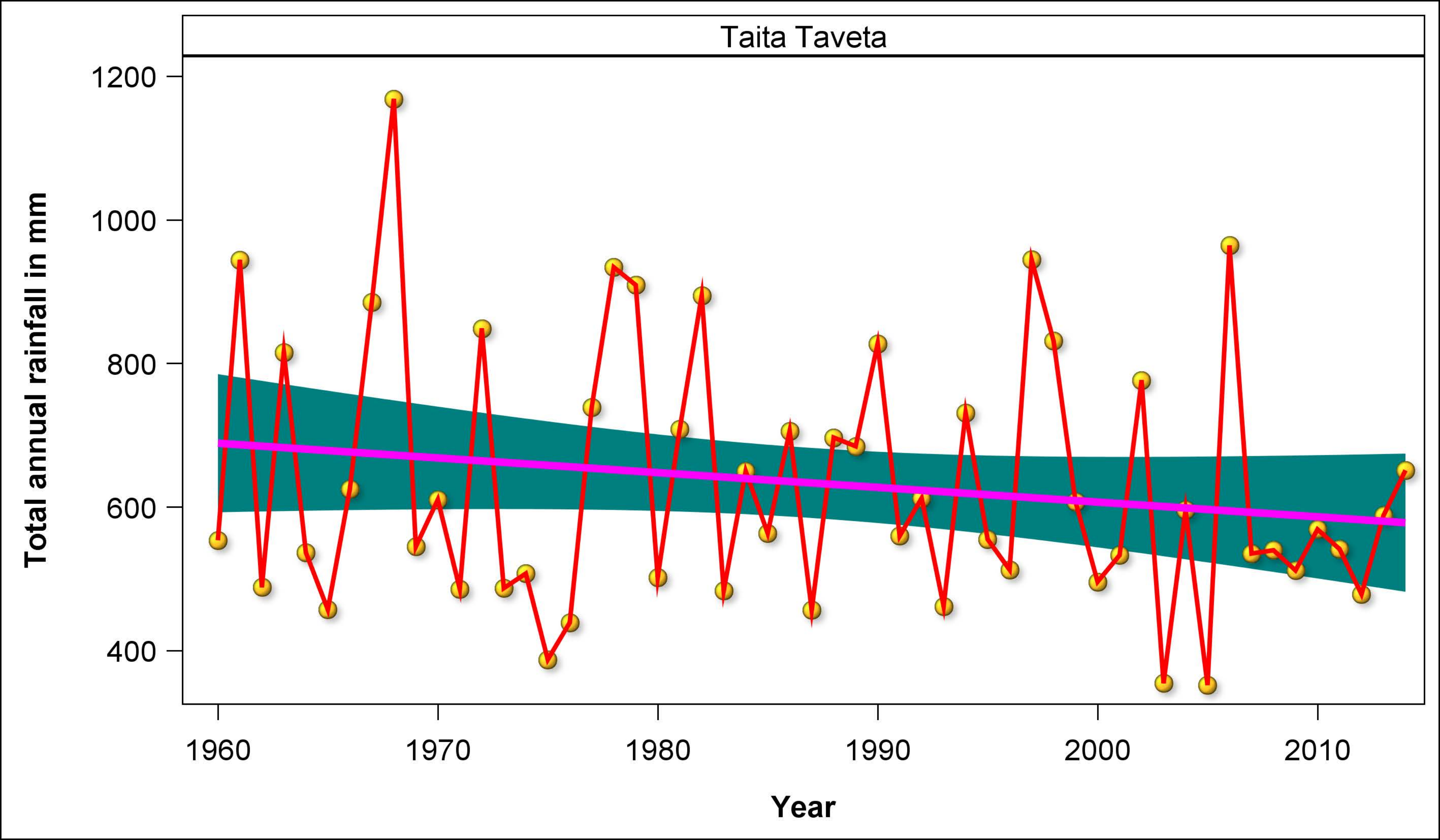

Taita Taveta
Total annual rainfall in mm
1200
1000
800
600
400
1960
1970
1980
1990
2000
2010
Year

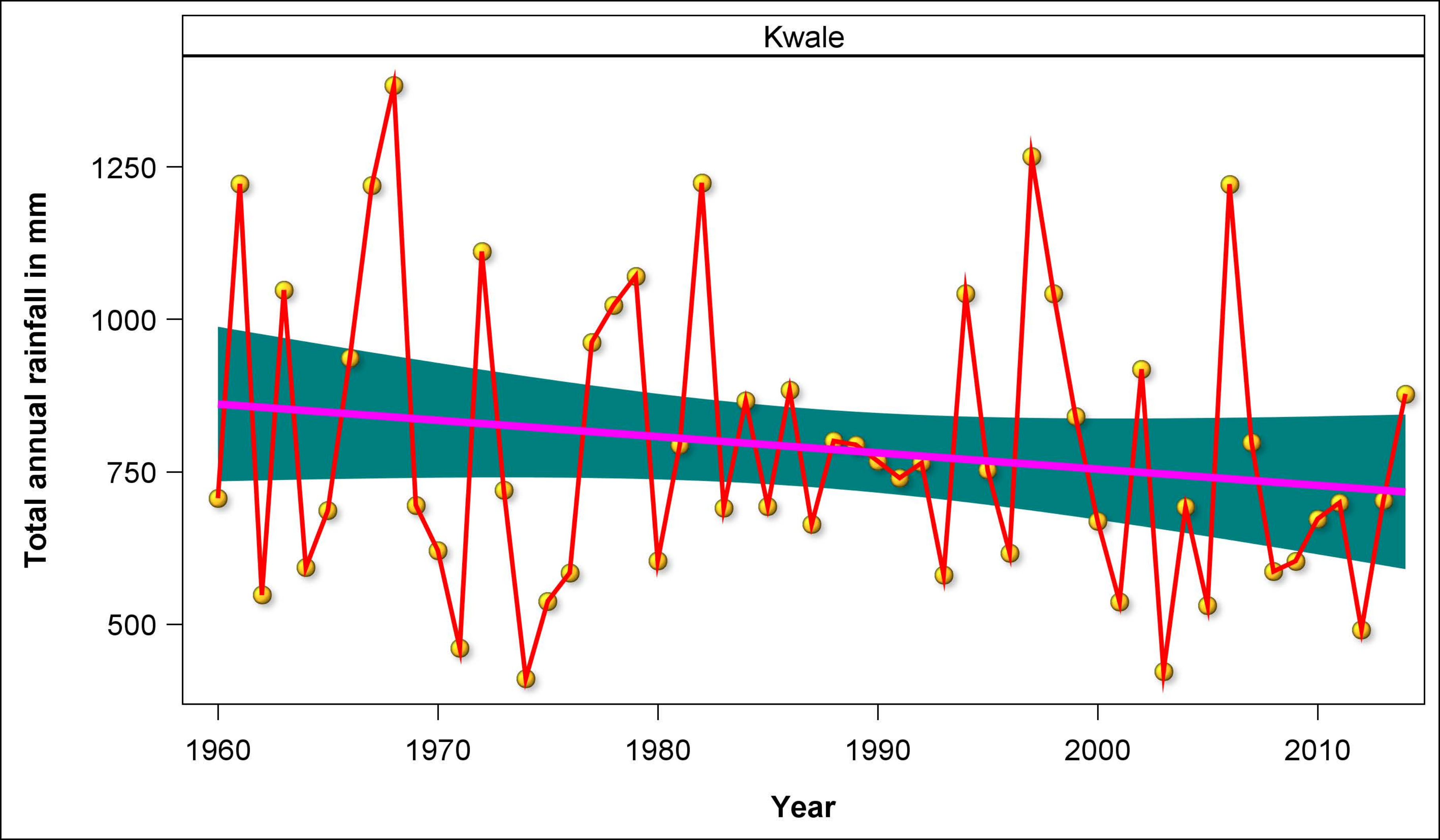
Kwale
Total annual rainfall in mm
1250
1000
750
500
1960
1970
1980
1990
2000
2010
Year

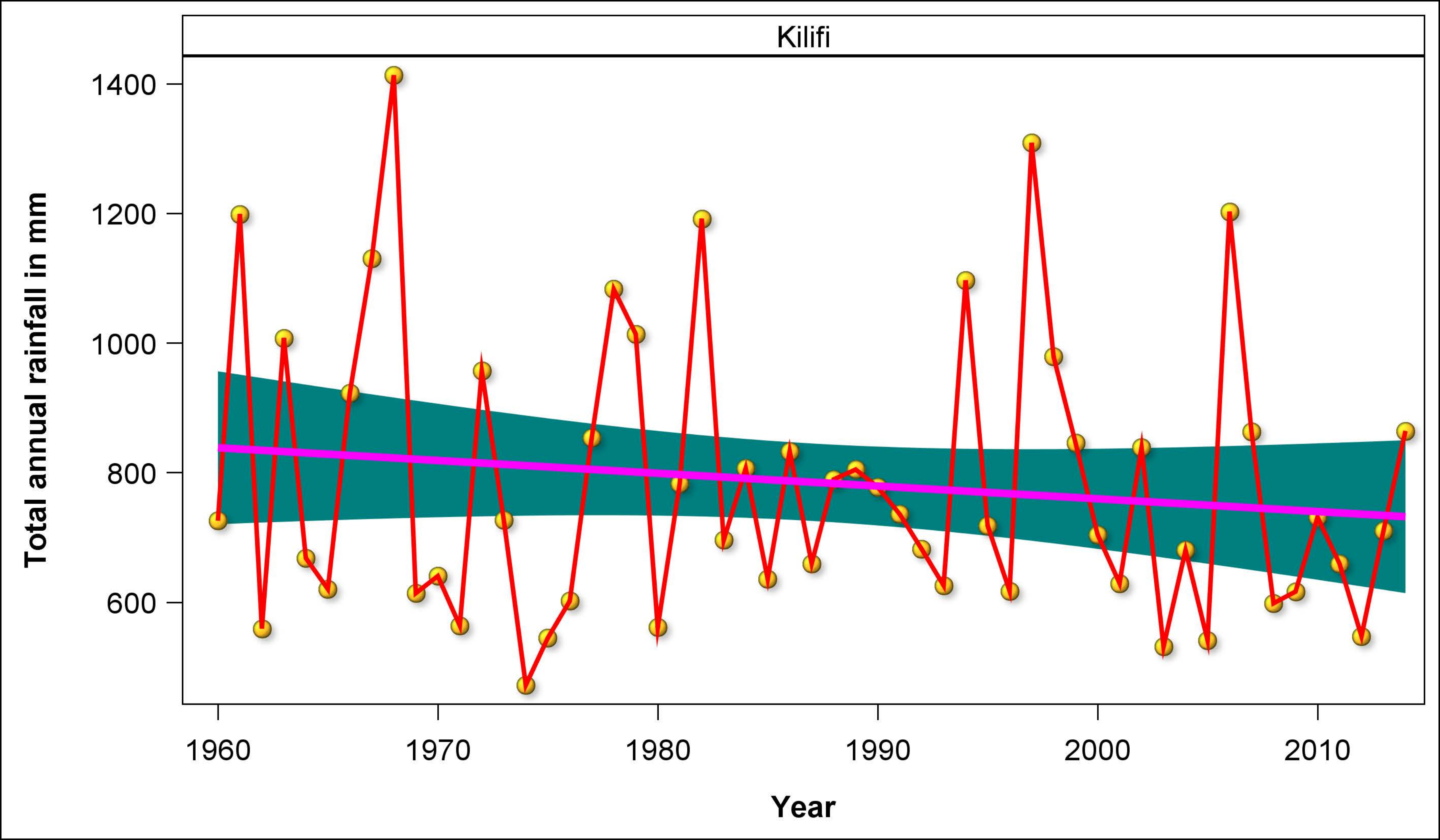
Kilifi
Total annual rainfall in mm
1400
1200
1000
800
600
1960
1970
1980
1990
2000
2010
Year

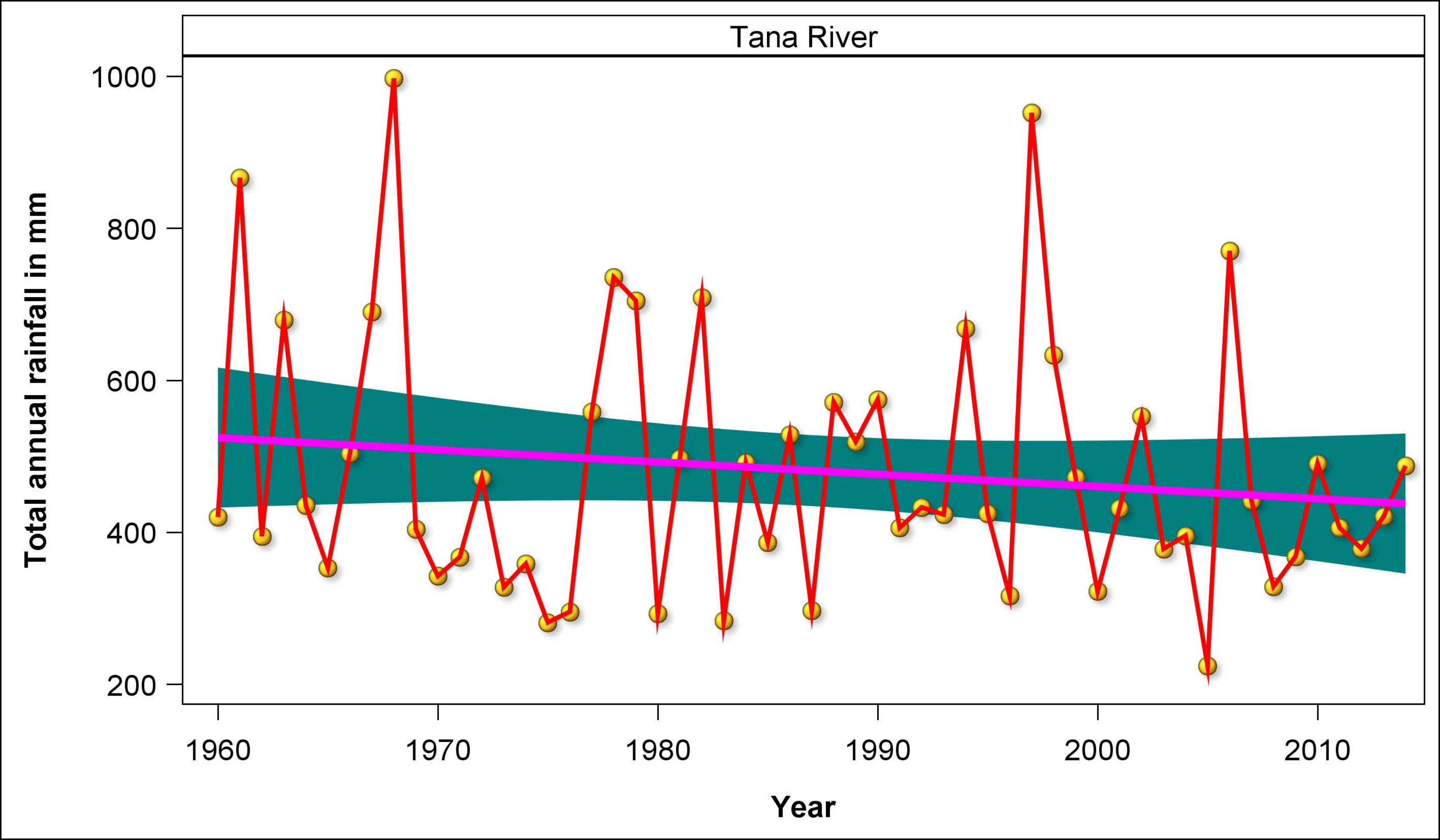
Tana River
Total annual rainfall in mm
1000
800
600
400
200
1960
1970
1980
1990
2000
2010
Year

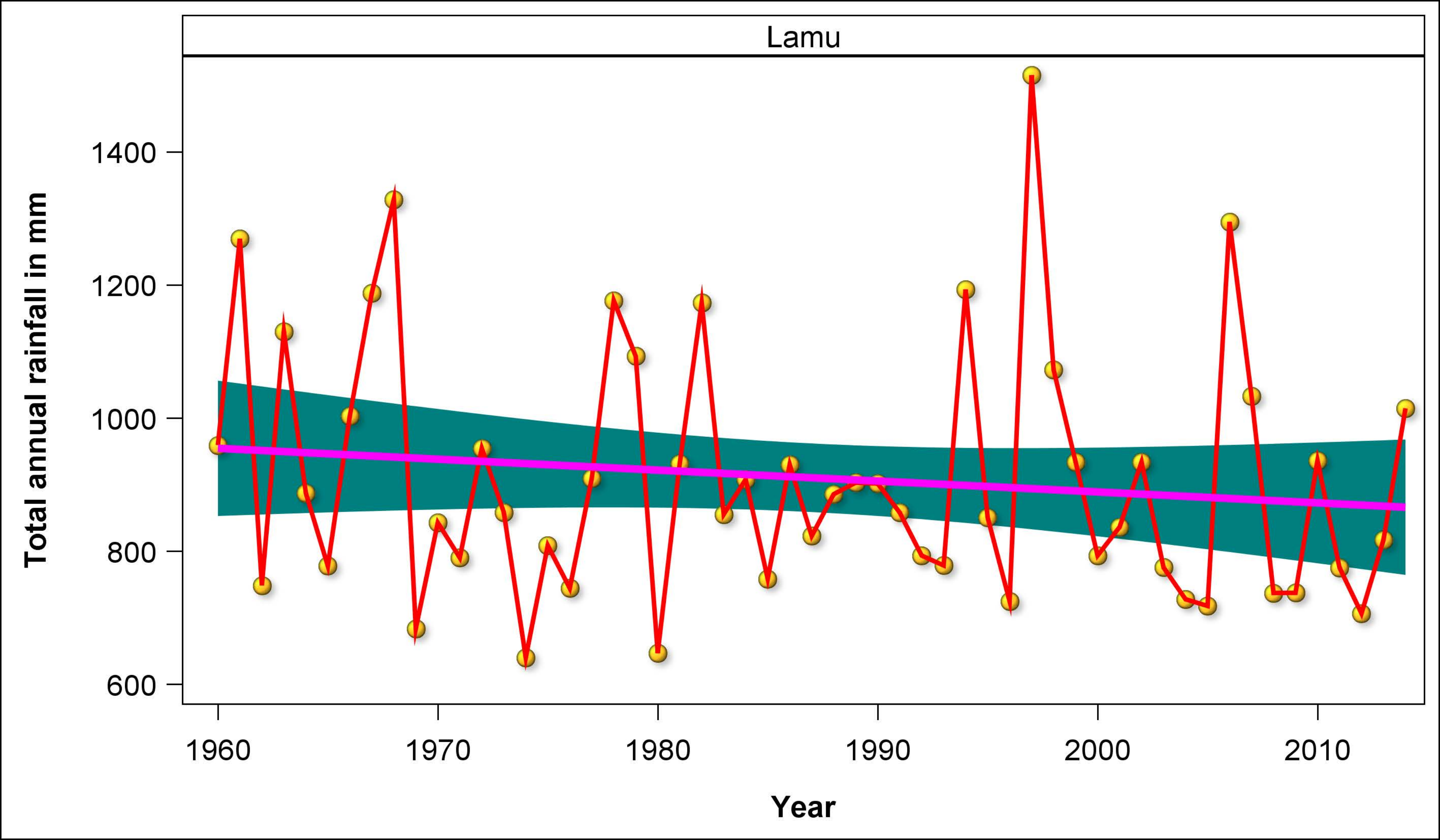
Lamu
Total annual rainfall in mm
1400
1200
1000
800
600
1960
1970
1980
1990
2000
2010
Year

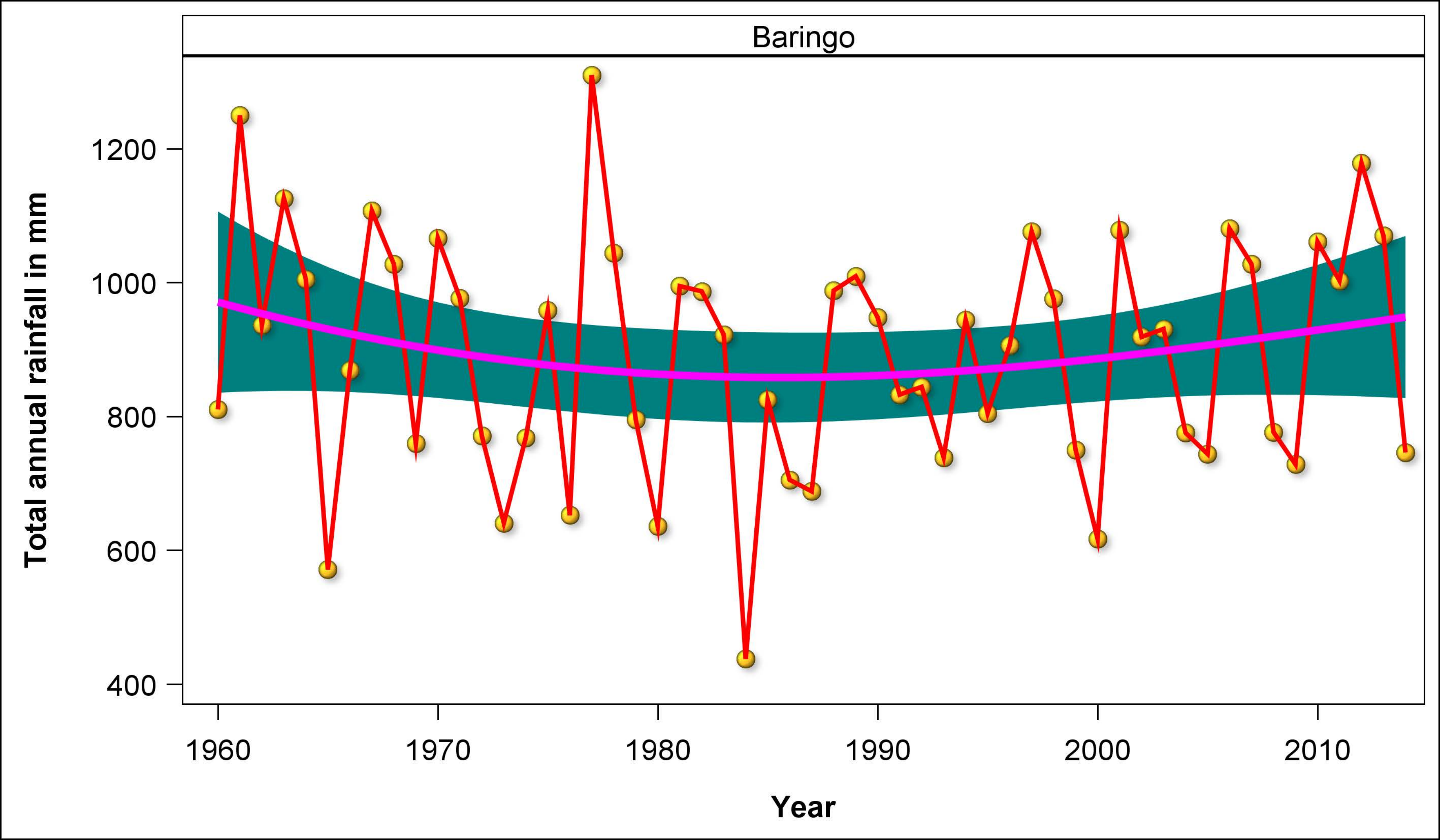
Baringo
Total annual rainfall in mm
1200
1000
800
600
400
1960
1970
1980
1990
2000
2010
Year

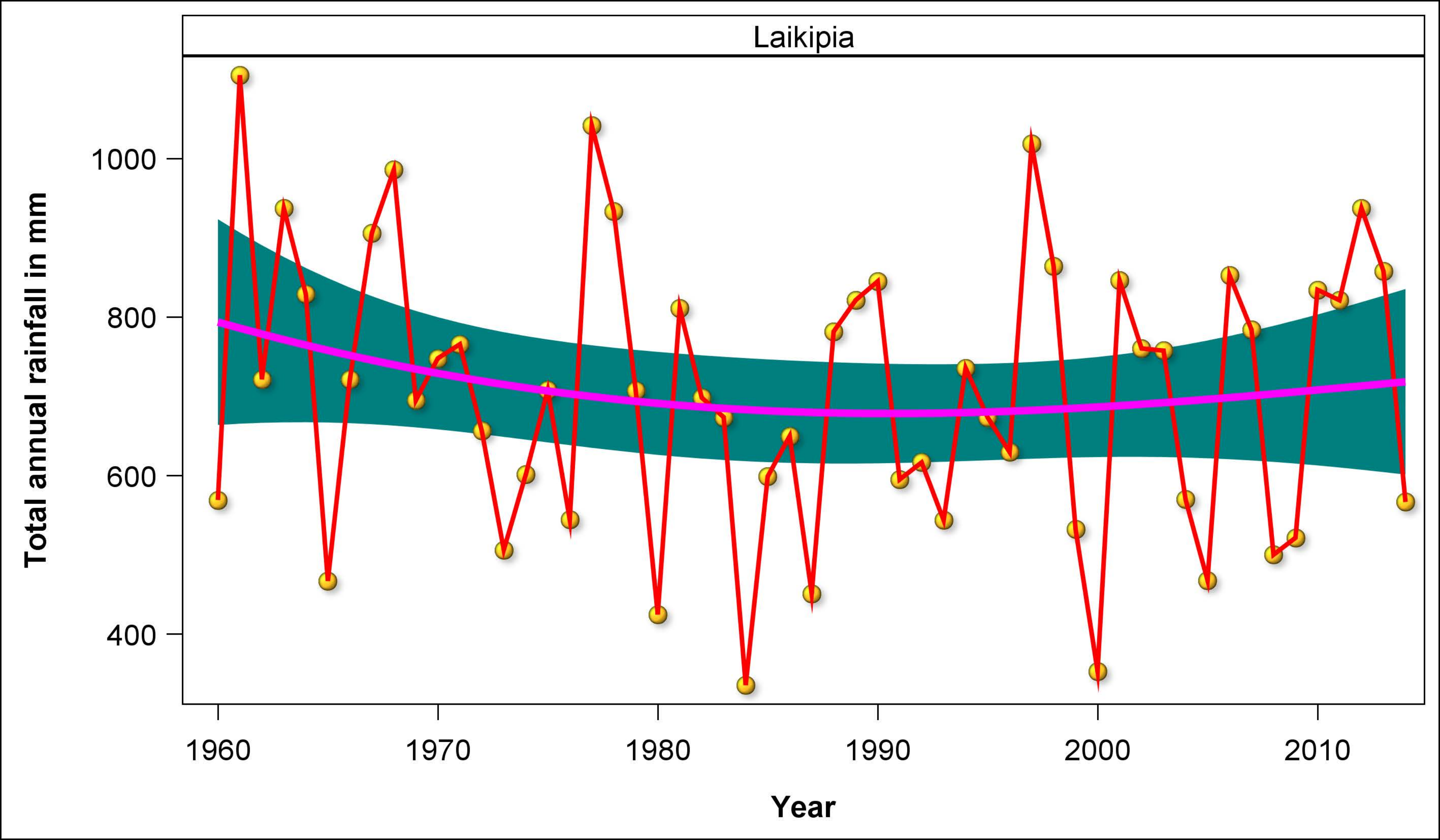
Laikipia
1000
800
600
400
Total annual rainfall in mm
1960
1970
1980
1990
2000
2010
Year

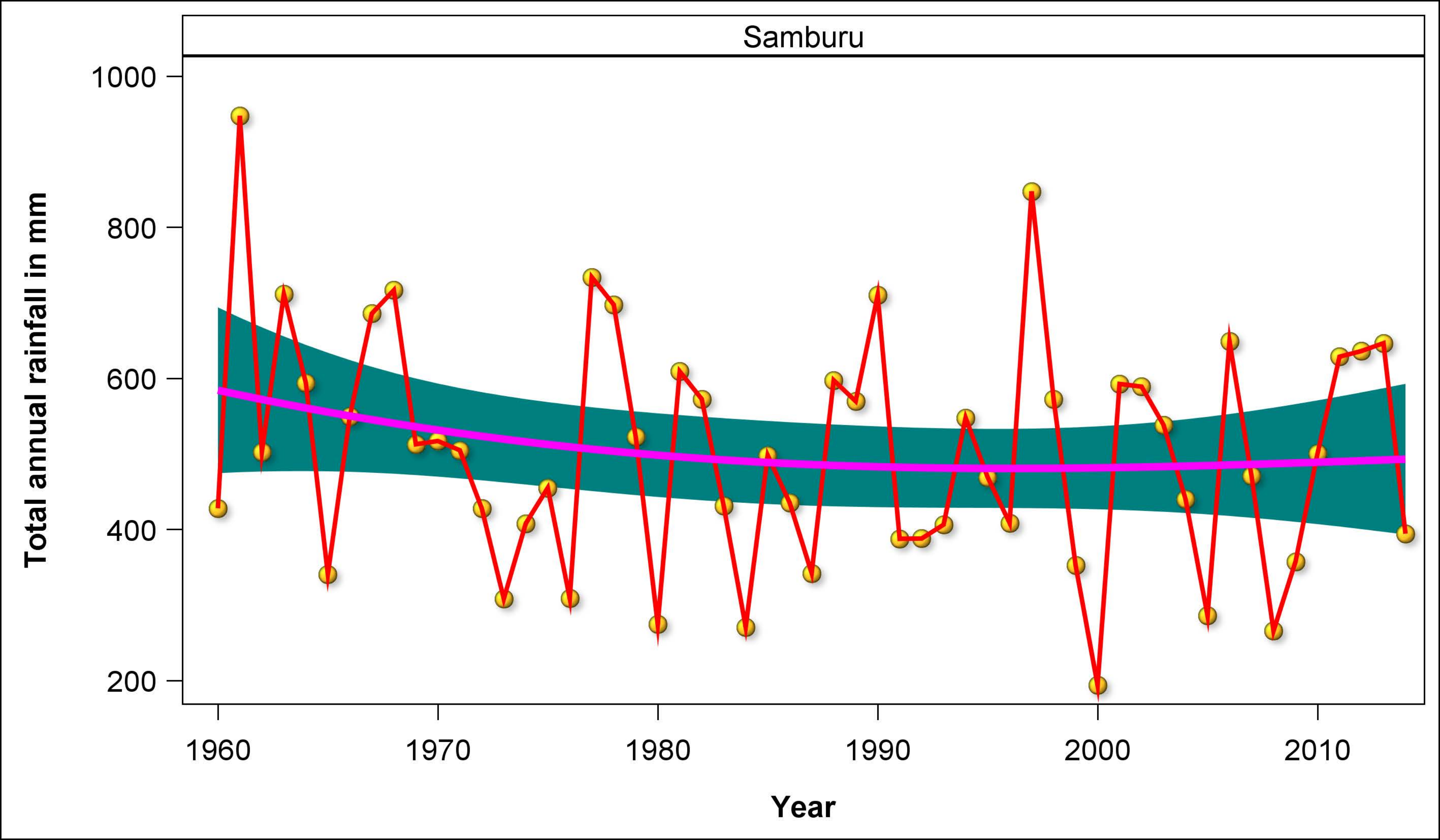
Samburu
Total annual rainfall in mm
1000
800
600
400
200
1960
1970
1980
1990
2000
2010
Year

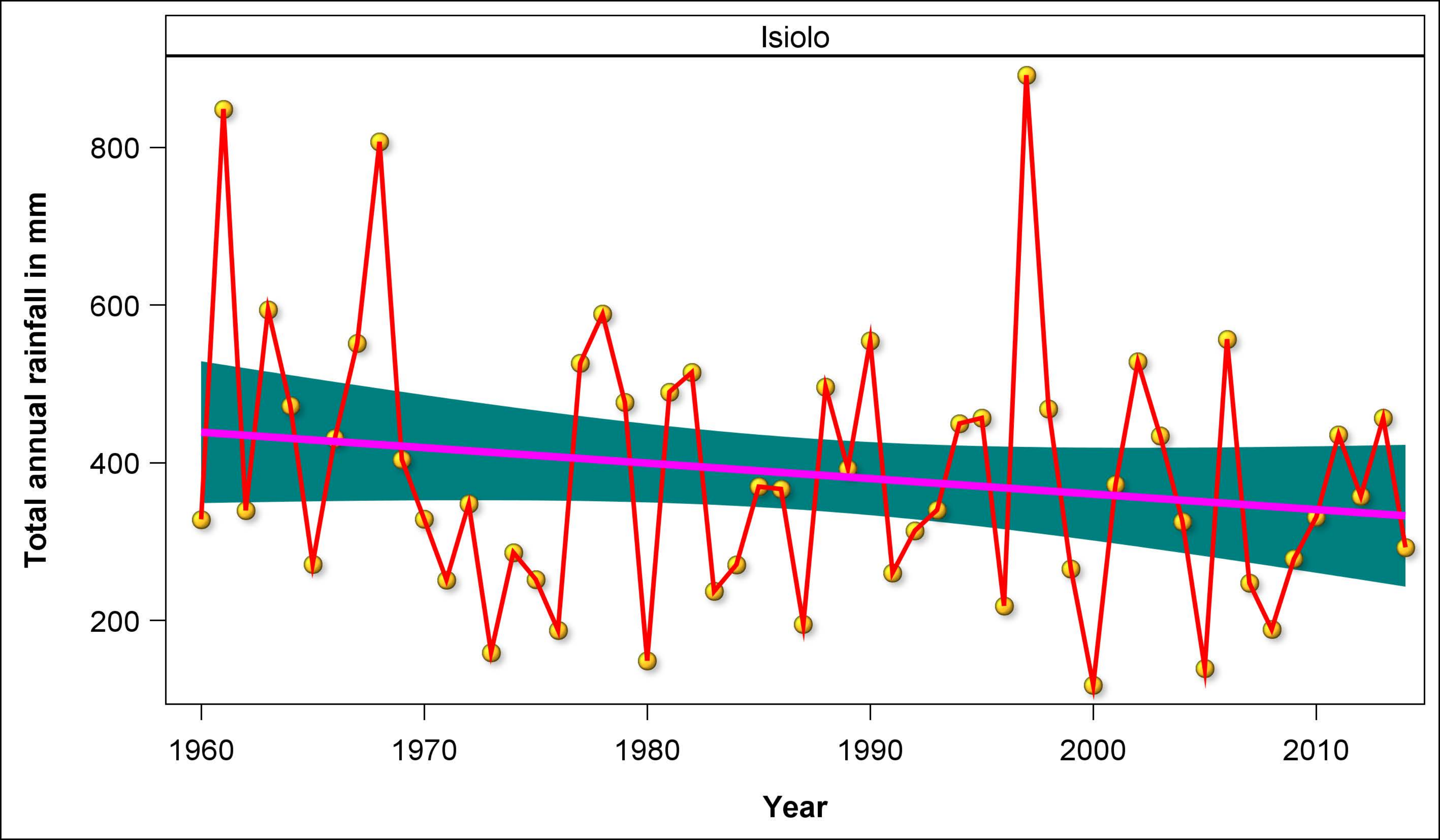
Isiolo
Total annual rainfall in mm
800
600
400
200
1960
1970
1980
1990
2000
2010
Year

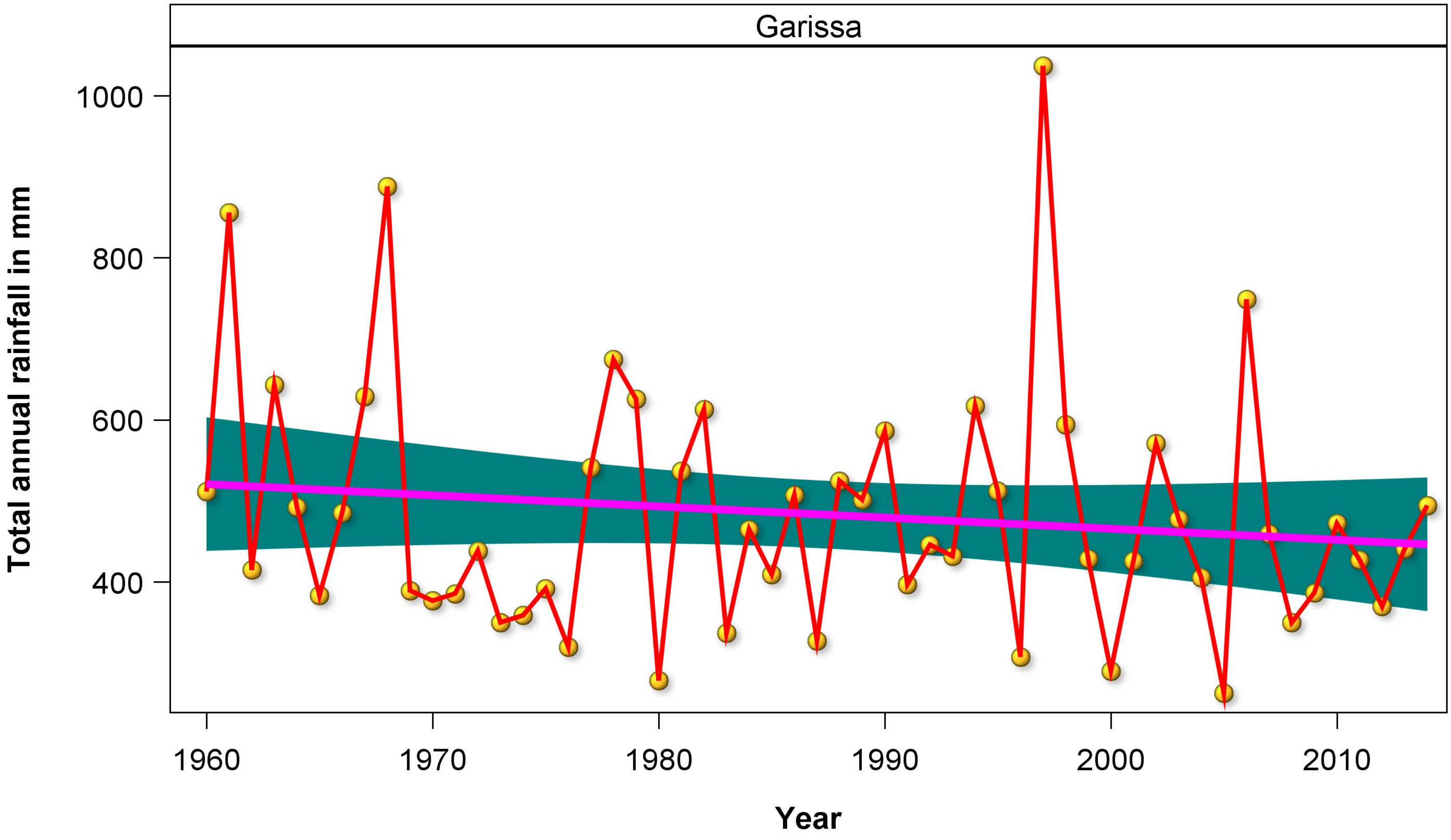
Garissa
Total annual rainfall in mm
1000
800
600
400
1960
1970
1980
1990
2000
2010
Year

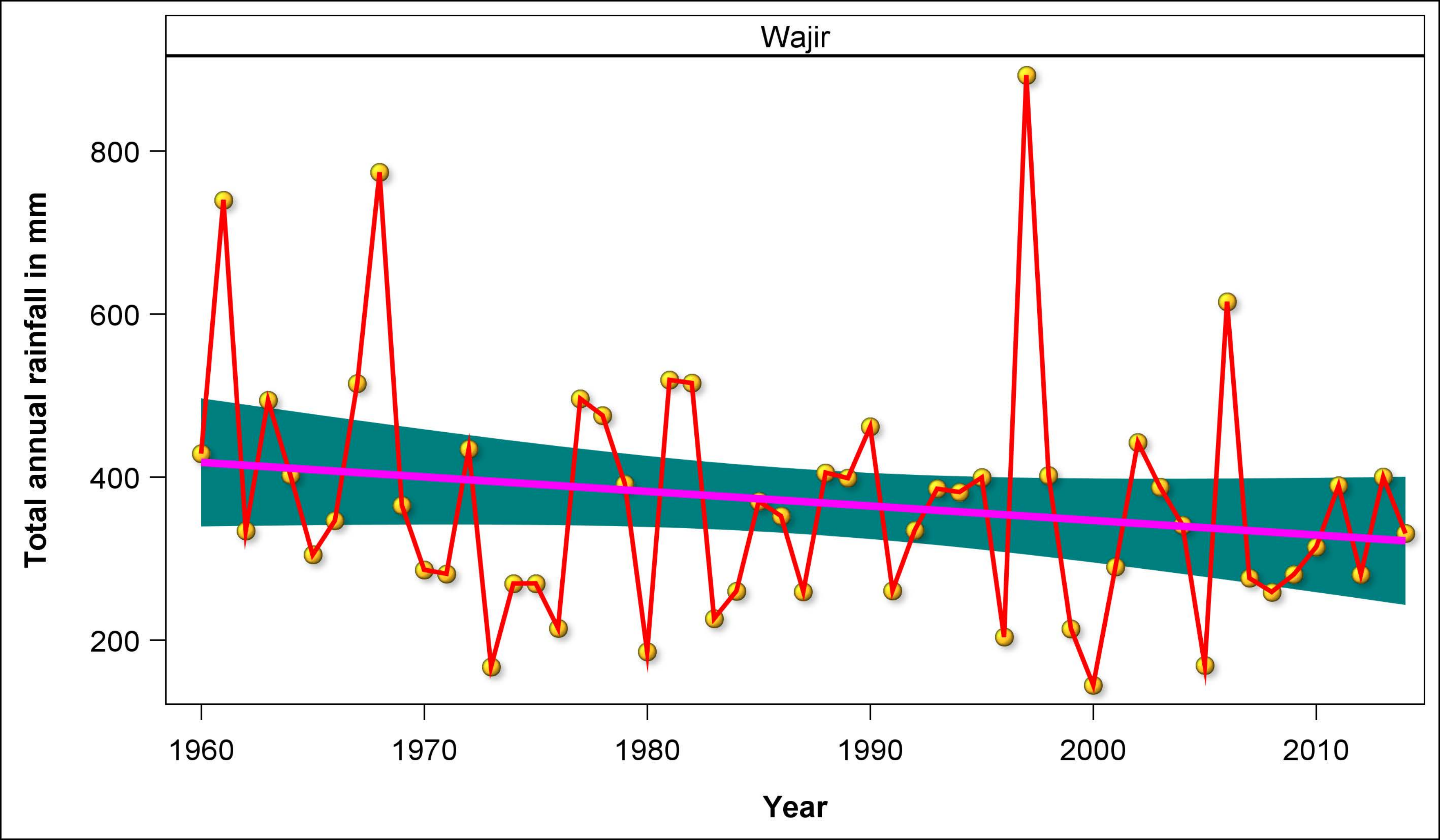

Wajir
Total annual rainfall in mm
800
600
400
200
1960
1970
1980
1990
2000
2010
Year

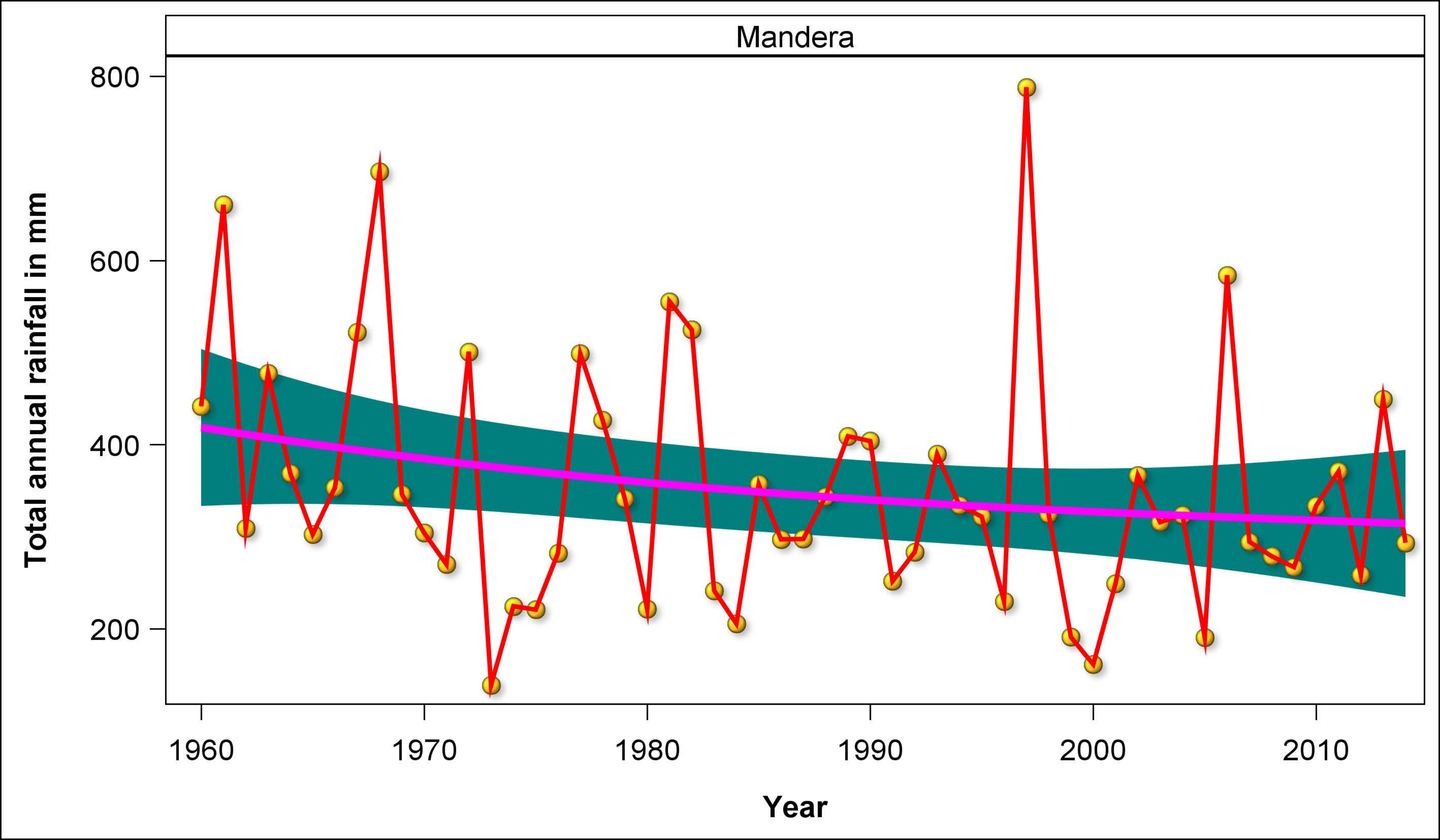
Mandera
Total annual rainfall in mm
800
600
400
200
1960
1970
1980
1990
2000
2010
Year

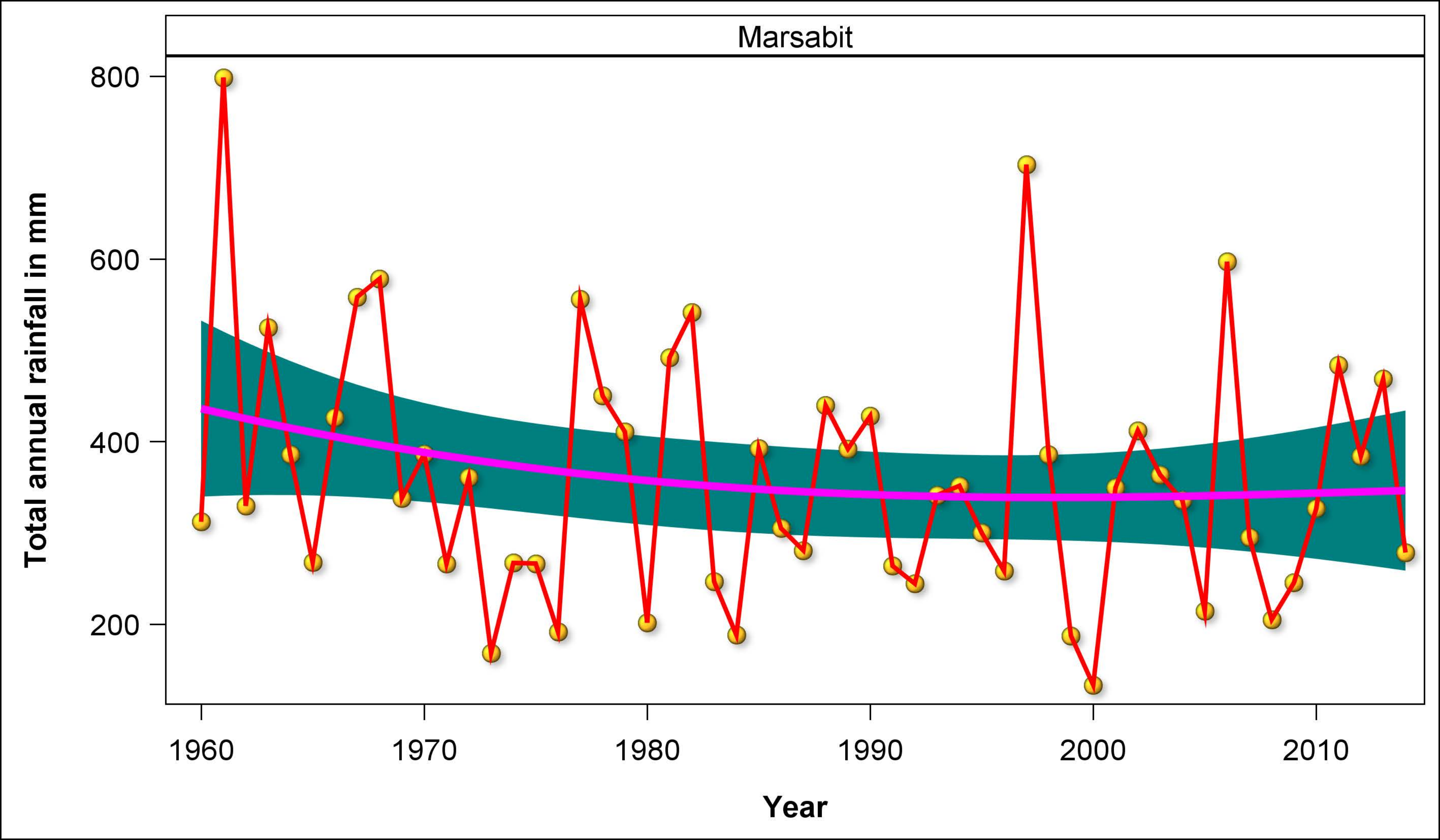

Marsabit
800
600
400
200
Total annual rainfall in mm
1960
1970
1980
1990
2000
2010
Year

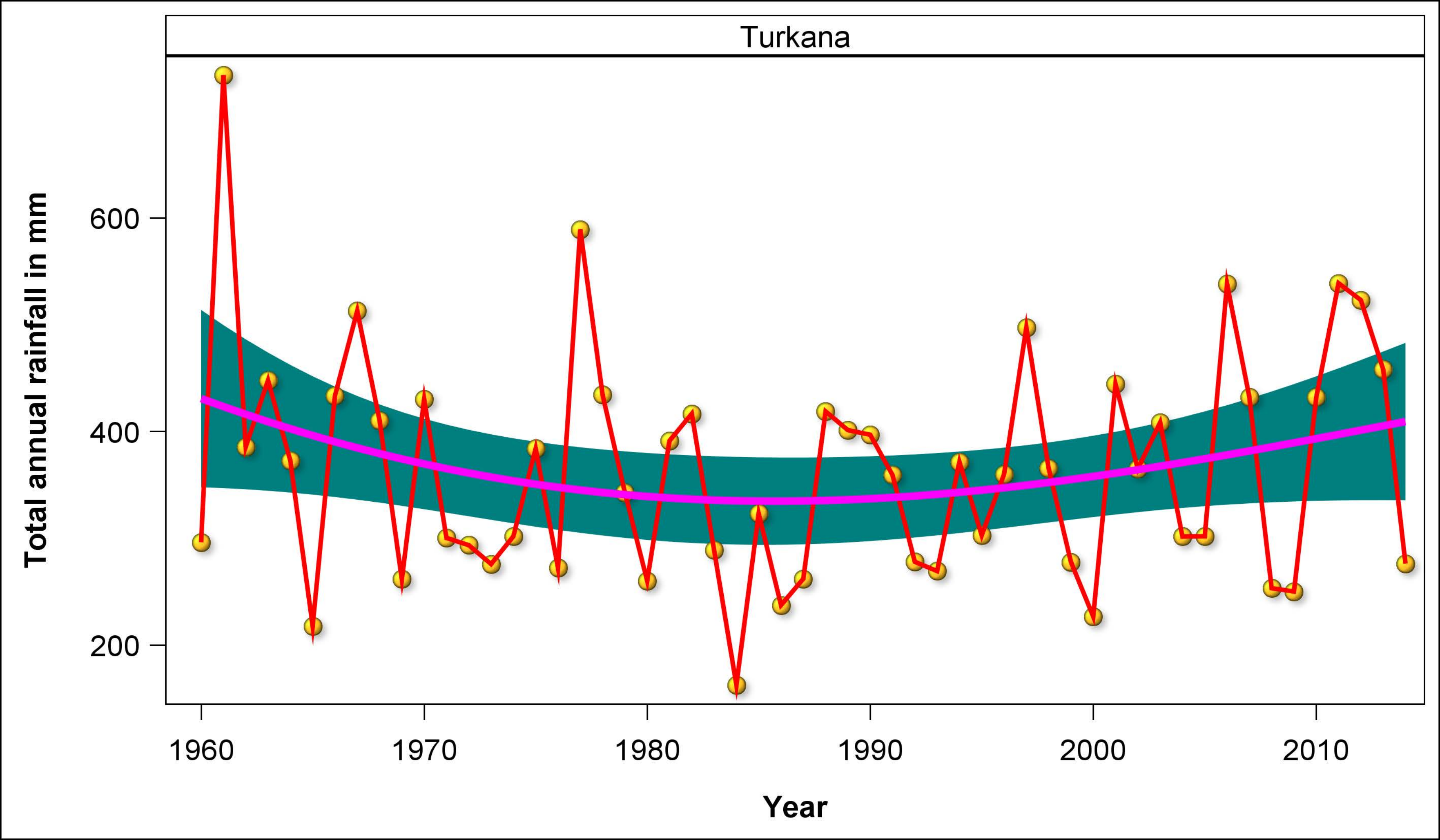
Turkana
Total annual rainfall in mm
600
400
200
1960
1970
1980
1990
2000
2010
Year

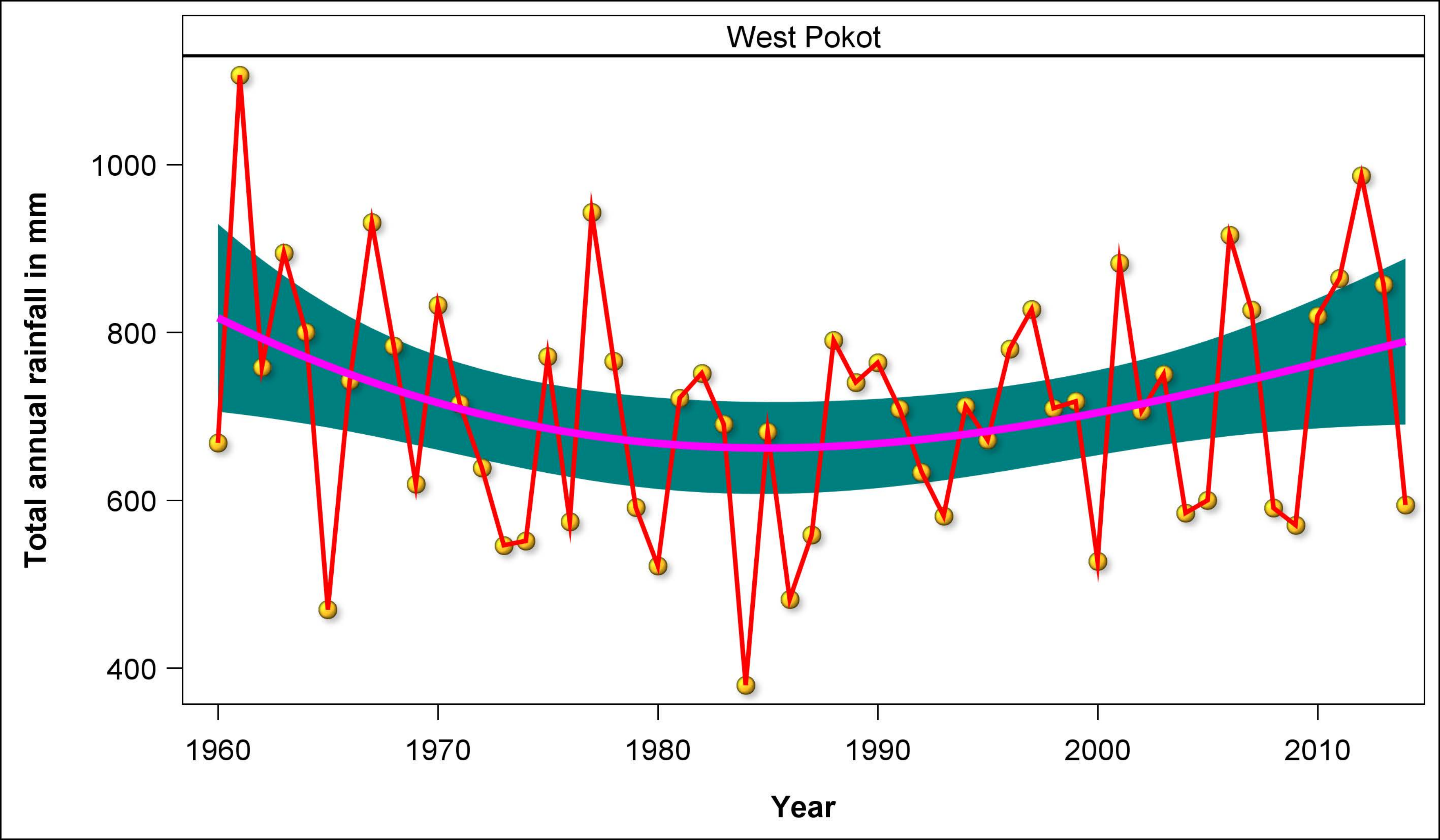
West Pokot
Total annual rainfall in mm
1000
800
600
400
1960
1970
1980
1990
2000
2010
Year

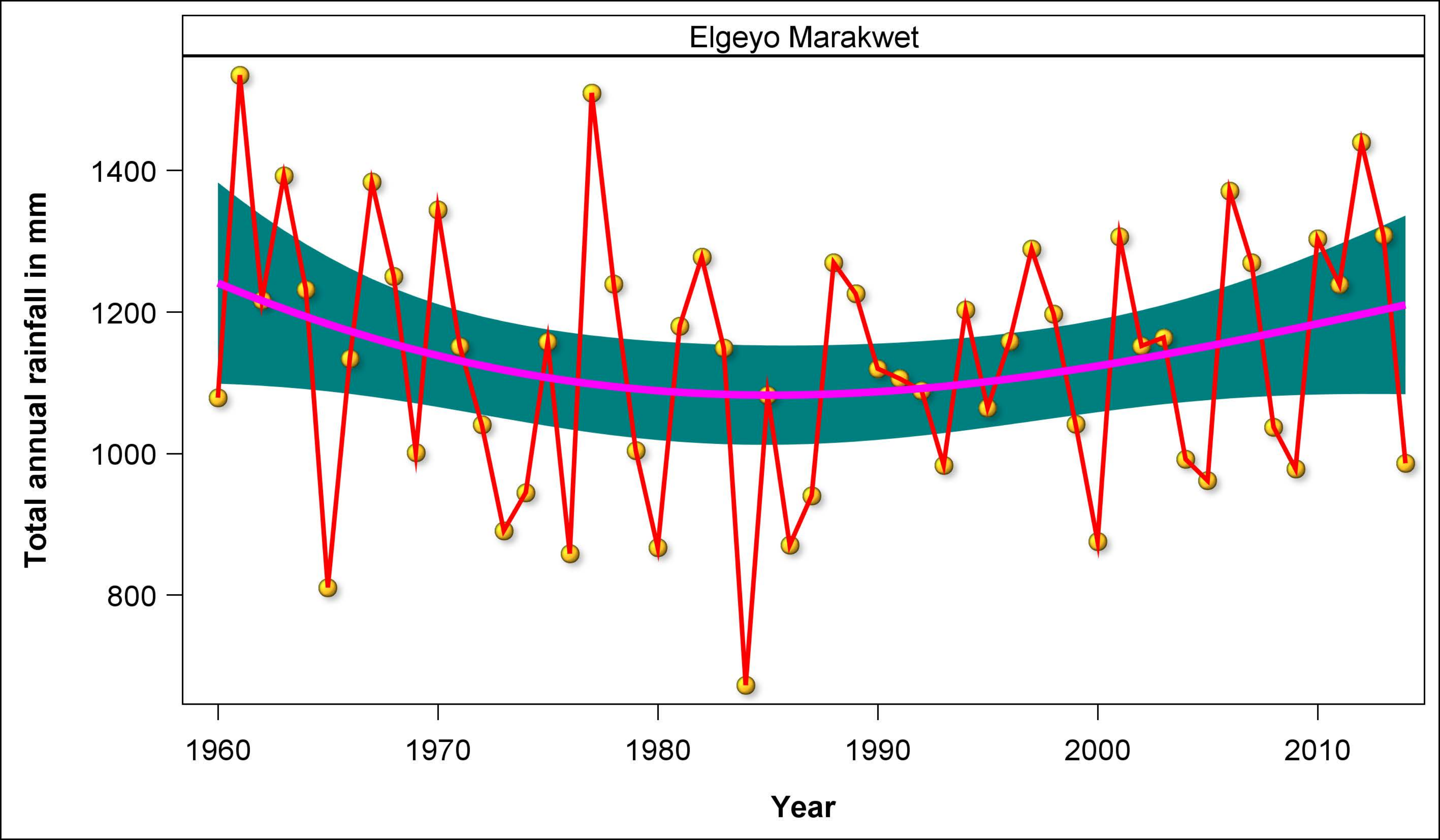
Elgeyo Marakwet
Total annual rainfall in mm
1400
1200
1000
800
1960
1970
1980
1990
2000
2010
Year
