# Supplementary material for: Extreme Wildlife Declines and Concurrent Increase in Livestock Numbers in Kenya: What Are the Causes?
Source: PLoS One. 2016 Sep 27;11(9):e0163249. doi: 10.1371/journal.pone.0163249 (PMC5039022; doi:10.1371/journal.pone.0163249)
Supplement: S24 Fig — The filled goldenrod circles denote the observations, the solid magenta curve the fitted trend curve whereas the forest green band the pointwise 95% confidence band. (PDF) [file pone.0163249.s034.pdf]

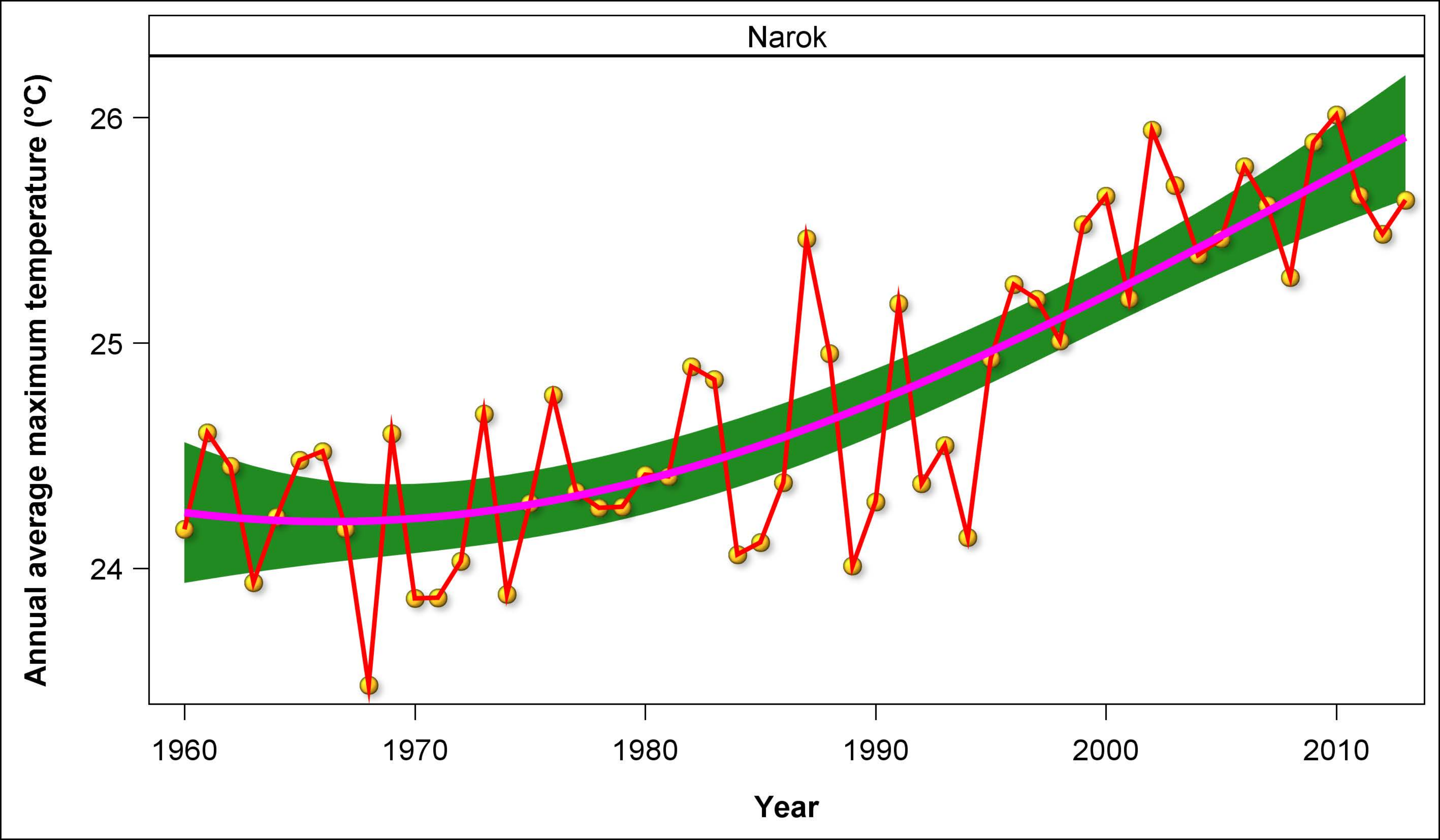

Narok
Annual average maximum temperature (°C)
26
25
24
1960
1970
1980
1990
2000
2010
Year

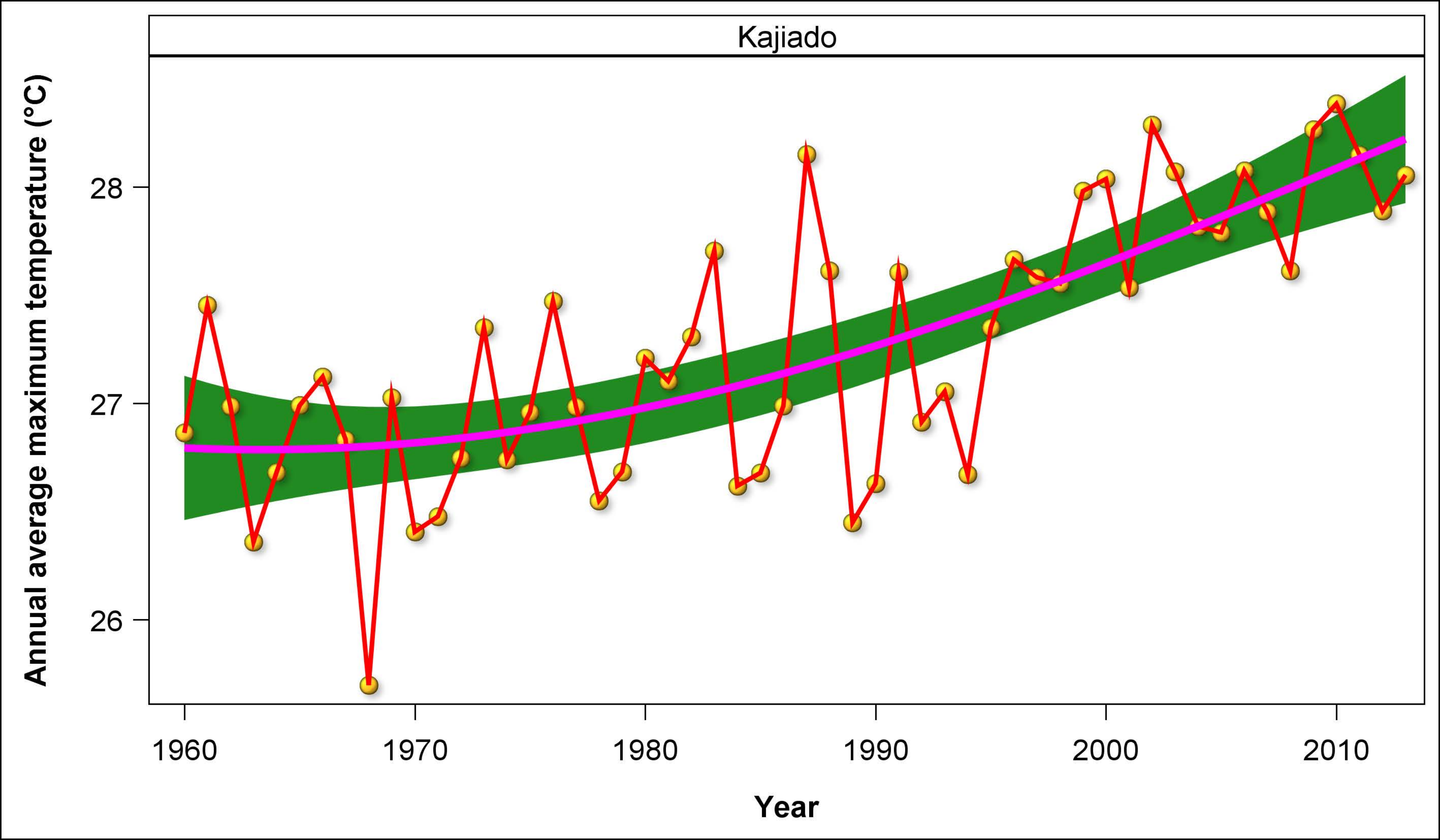

Kajiado
Annual average maximum temperature (°C)
28
27
26
1960
1970
1980
1990
2000
2010
Year

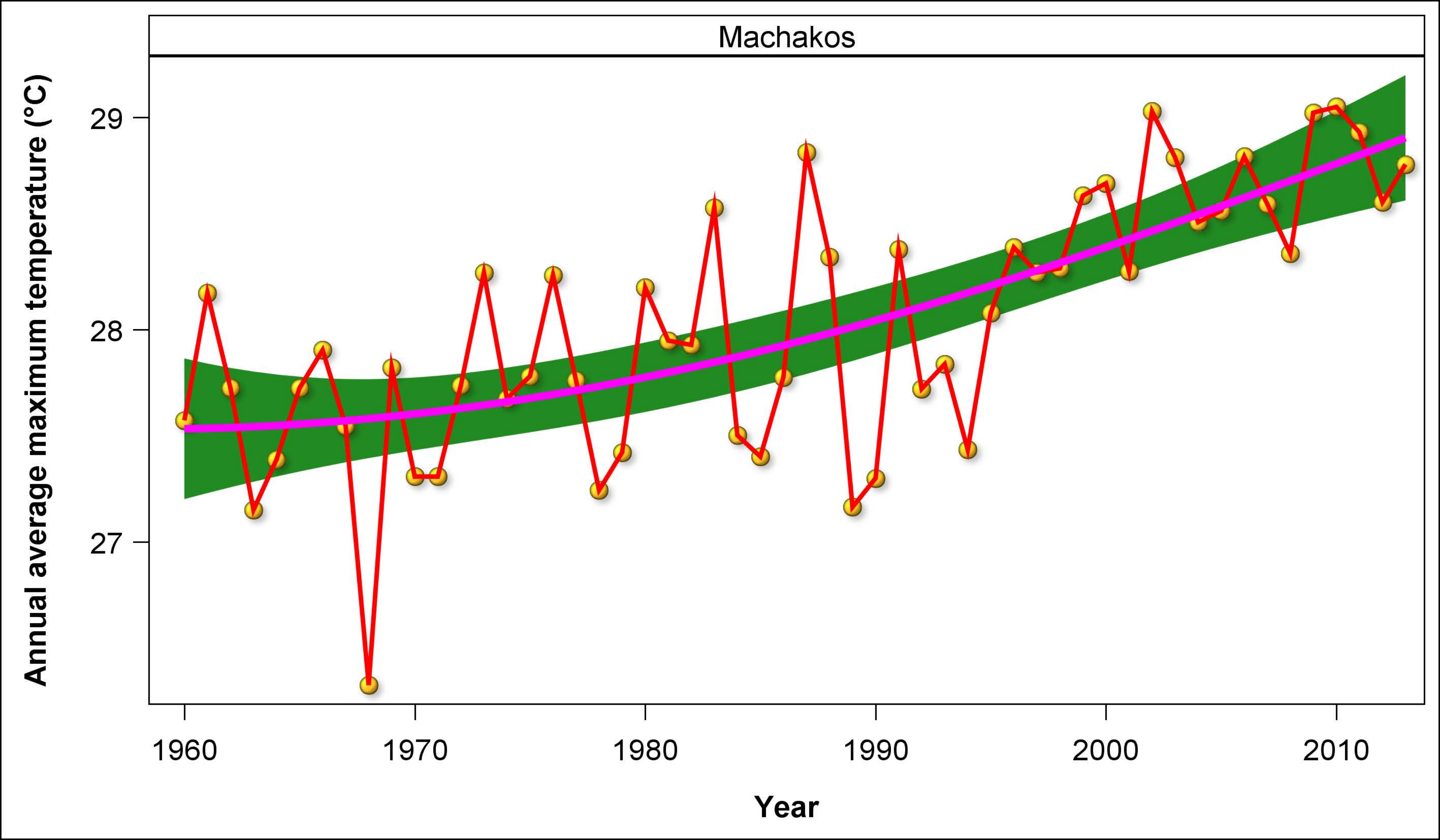
Machakos
Annual average maximum temperature (°C)
29
28
27
1960
1970
1980
1990
2000
2010
Year

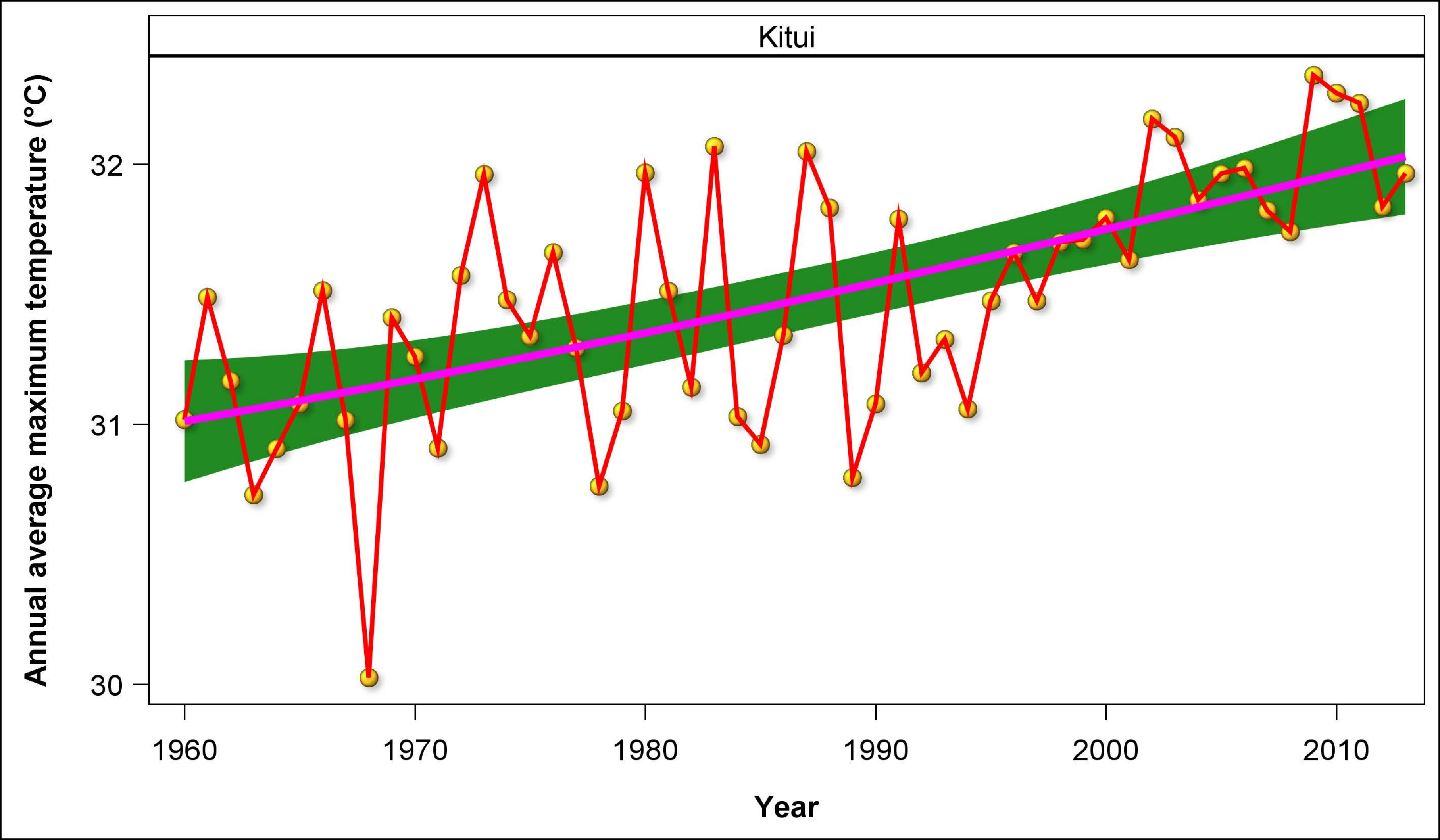

Kitui
Annual average maximum temperature (°C)
32
31
30
1960
1970
1980
1990
2000
2010
Year

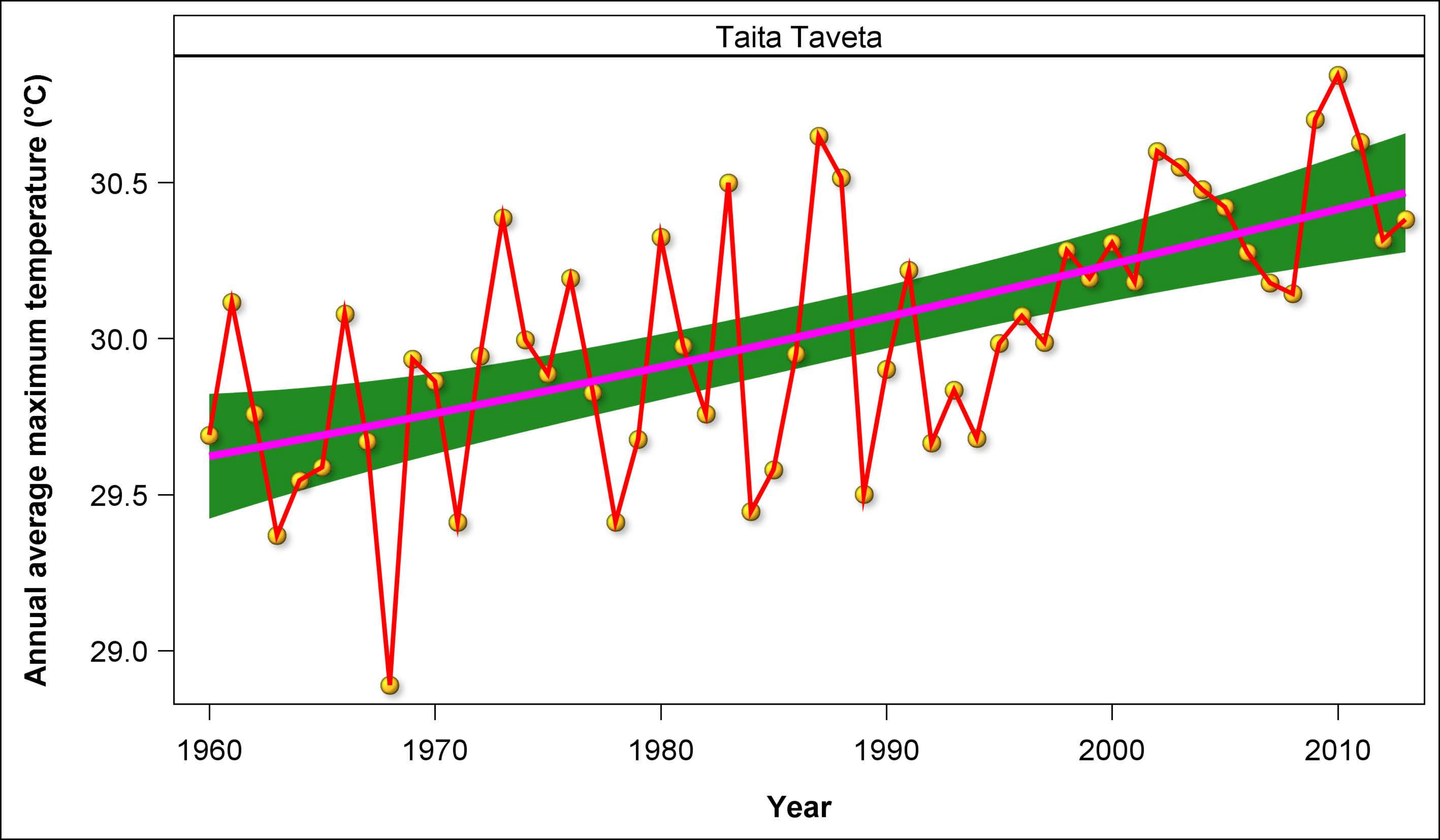

Taita Taveta
Annual average maximum temperature (°C)
30.5
30.0
29.5
29.0
1960
1970
1980
1990
2000
2010
Year

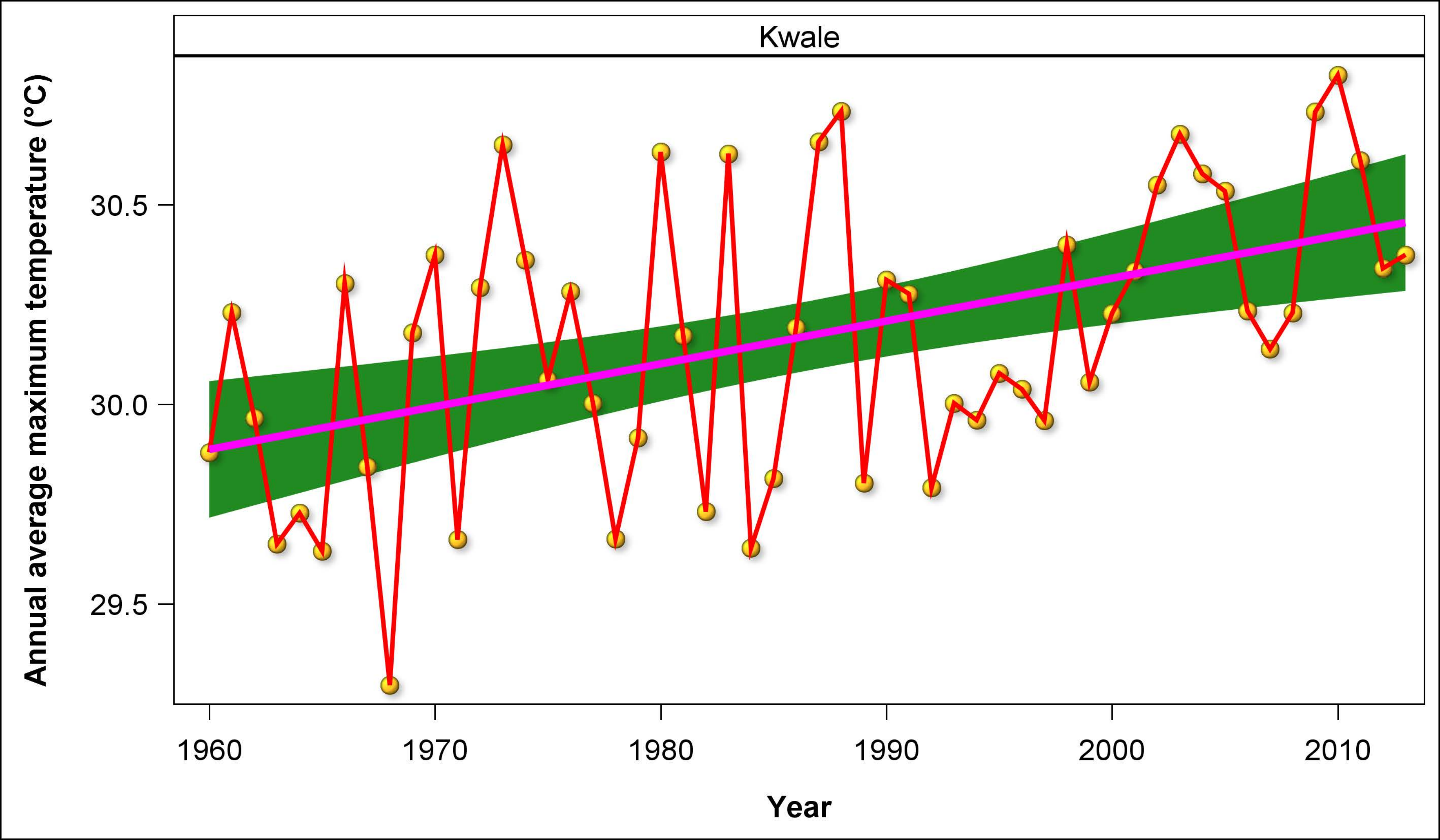

Kwale
Annual average maximum temperature (°C)
30.5
30.0
29.5
1960
1970
1980
1990
2000
2010
Year

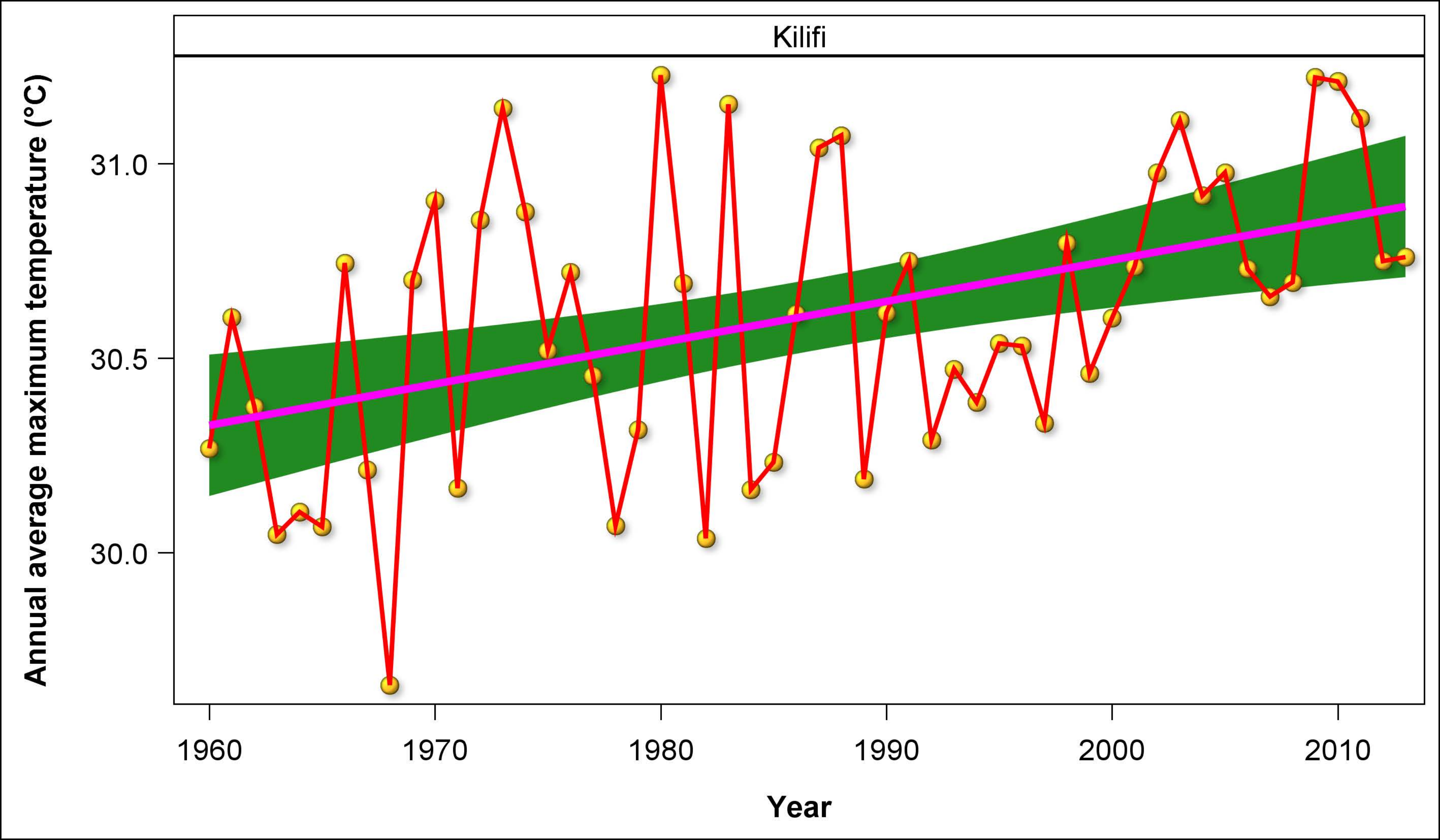
Kilifi
Annual average maximum temperature (°C)
31.0
30.5
30.0
1960
1970
1980
1990
2000
2010
Year

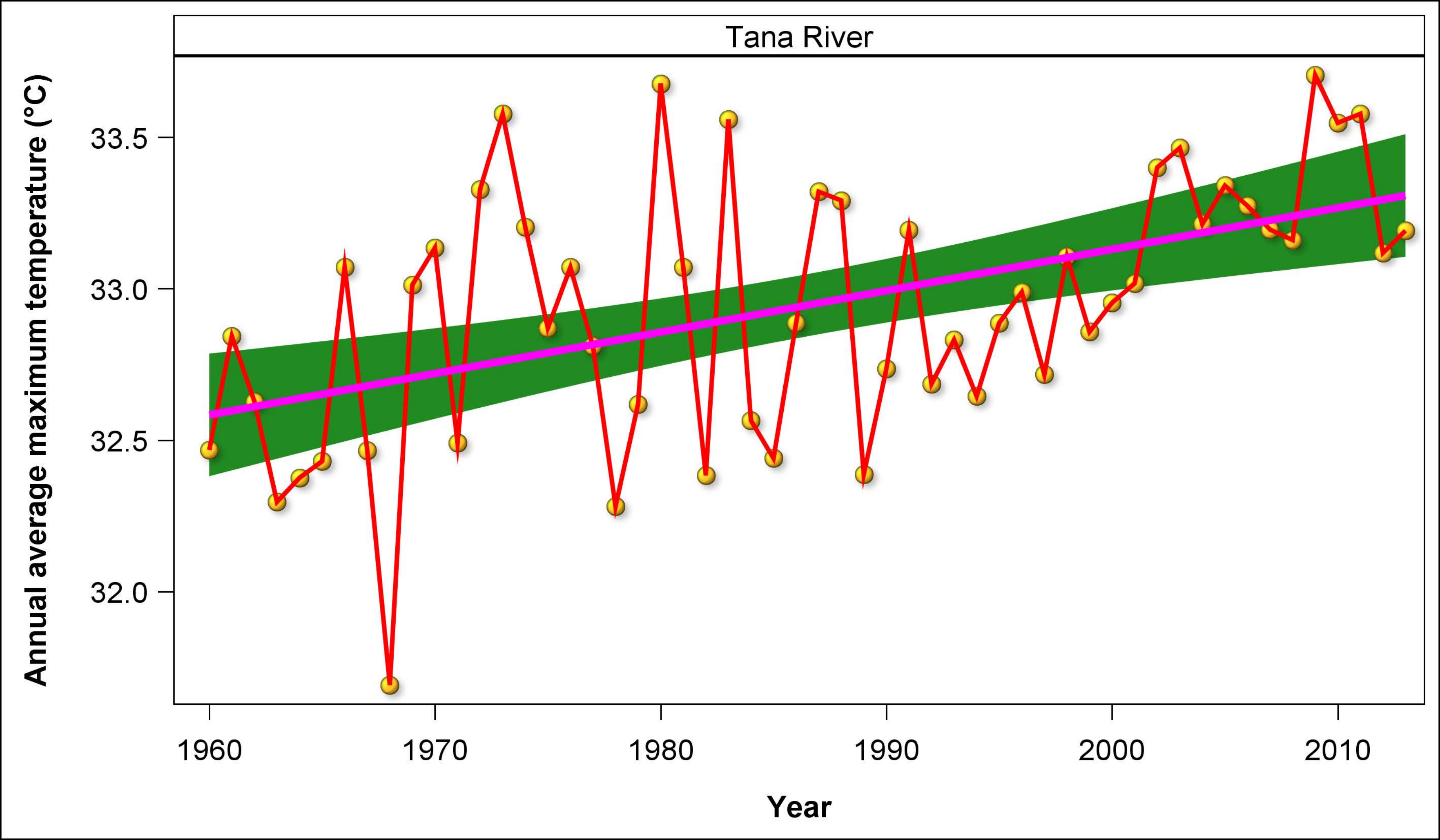

Tana River
Annual average maximum temperature (°C)
33.5
33.0
32.5
32.0
1960
1970
1980
1990
2000
2010
Year

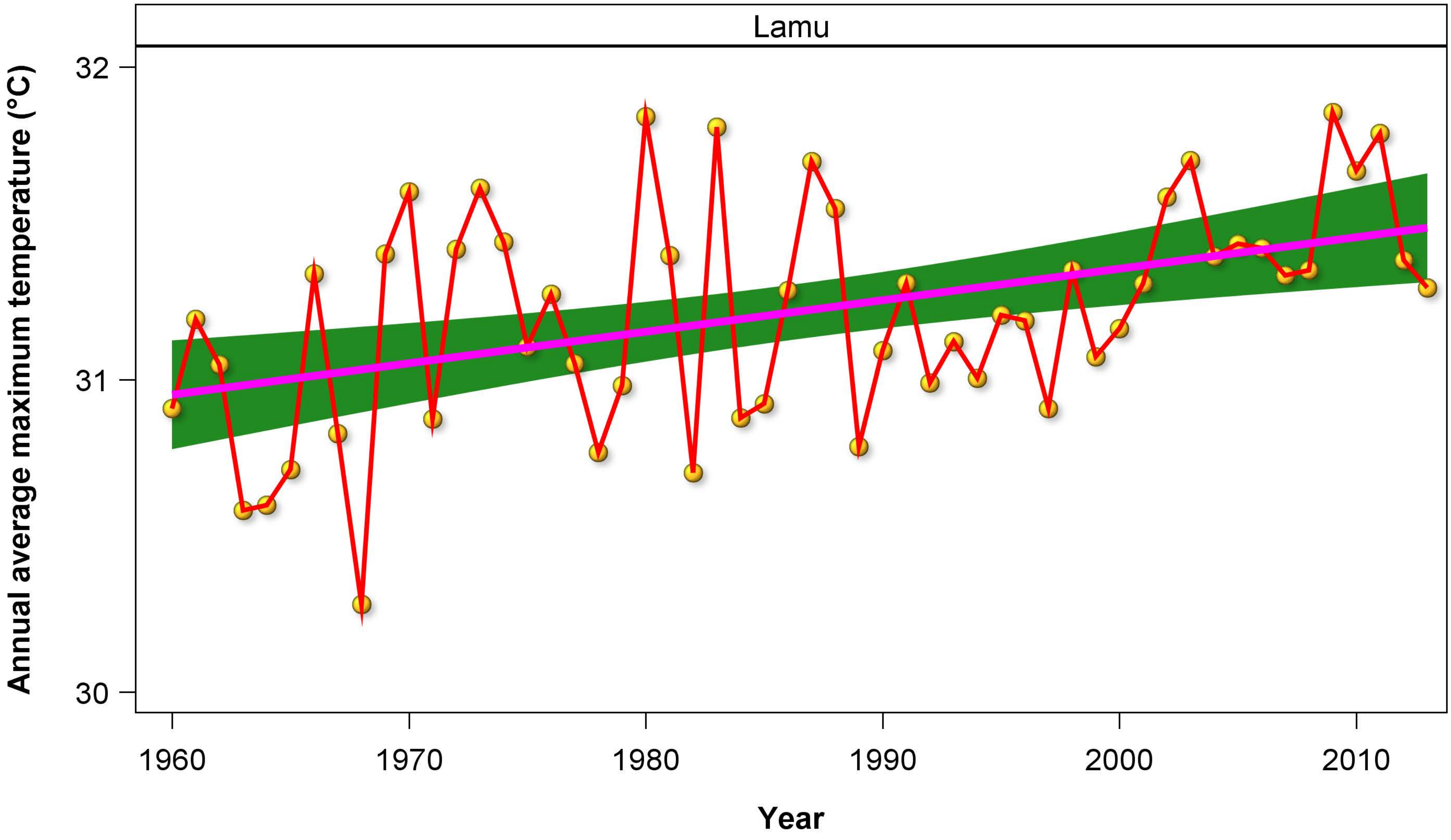

Lamu
Annual average maximum temperature (°C)
32
31
30
1960
1970
1980
1990
2000
2010
Year

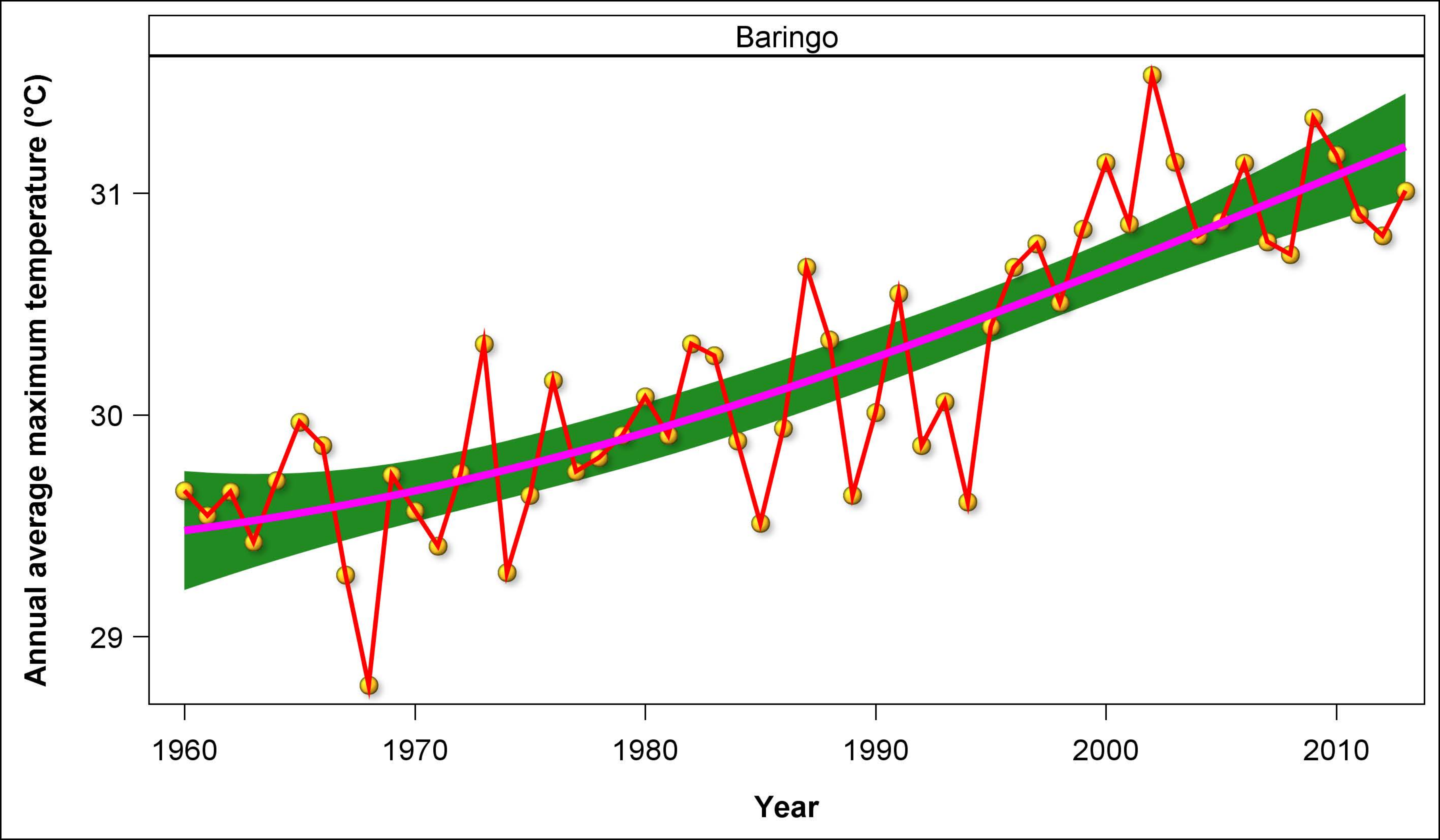
Baringo
Annual average maximum temperature (°C)
31
30
29
1960
1970
1980
1990
2000
2010
Year

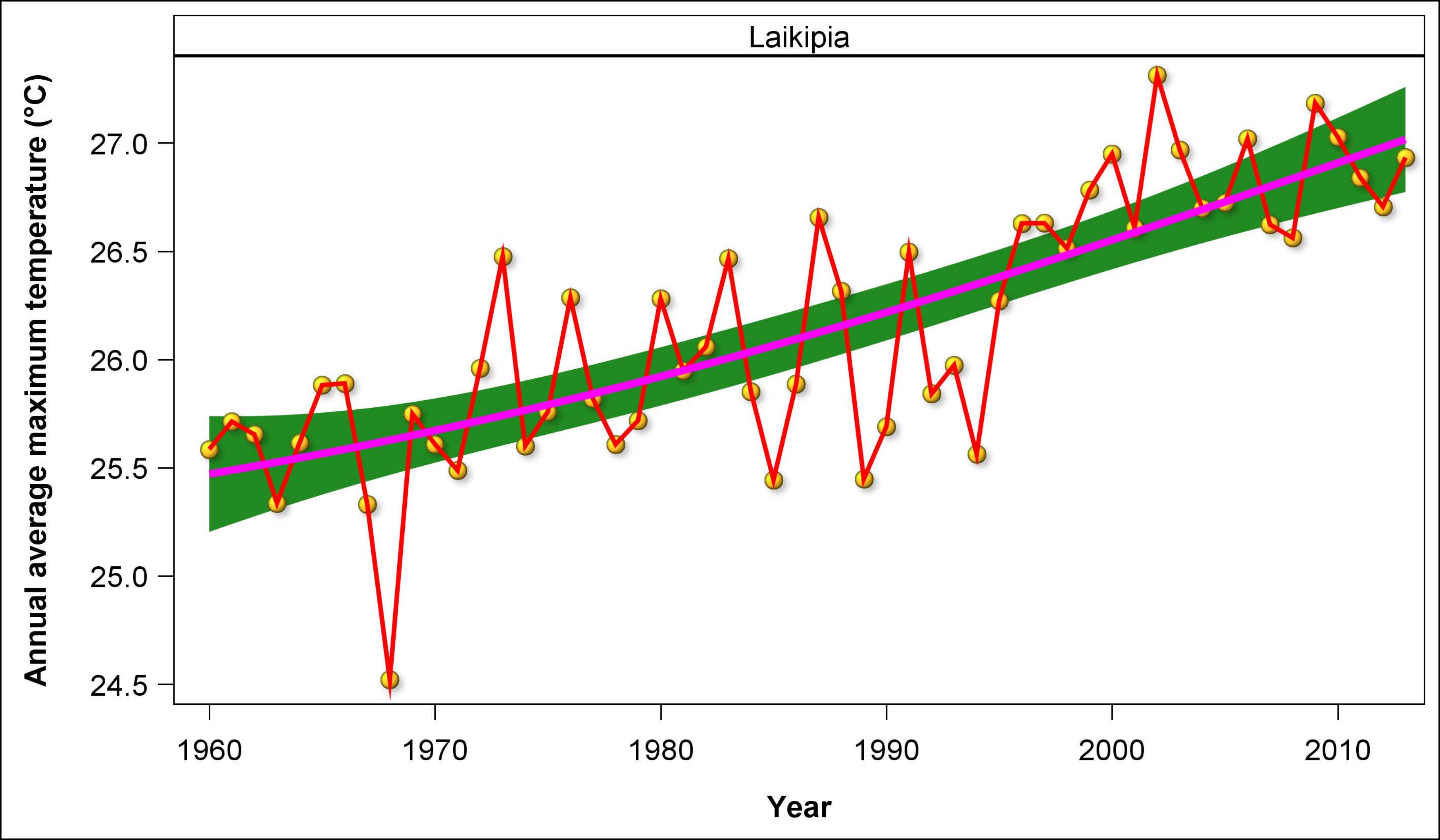
Laikipia
Annual average maximum temperature (°C)
27.0
26.5
26.0
25.5
25.0
24.5
1960
1970
1980
1990
2000
2010
Year

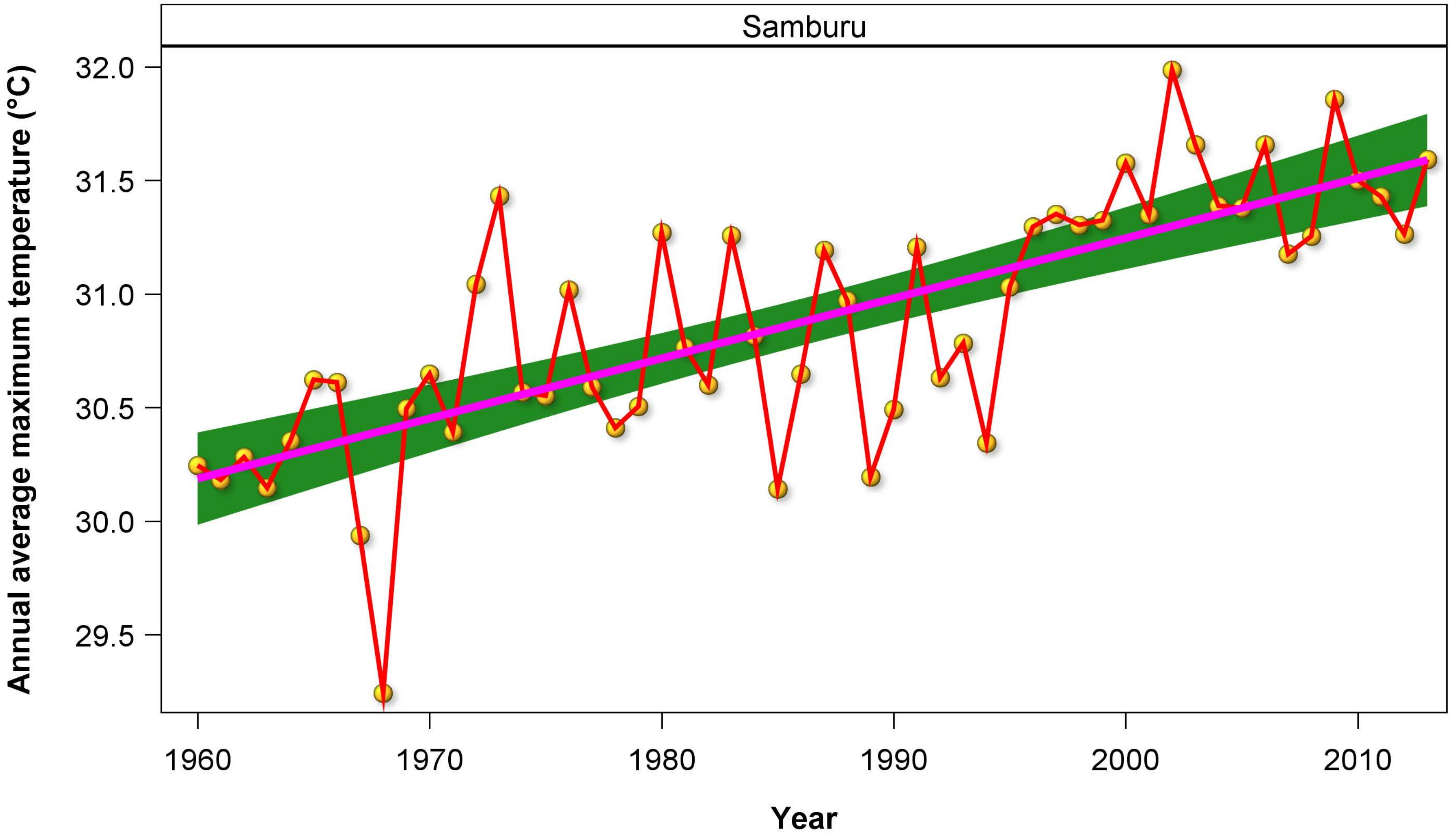

Samburu
Annual average maximum temperature (°C)
32.0
31.5
31.0
30.5
30.0
29.5
1960
1970
1980
1990
2000
2010
Year

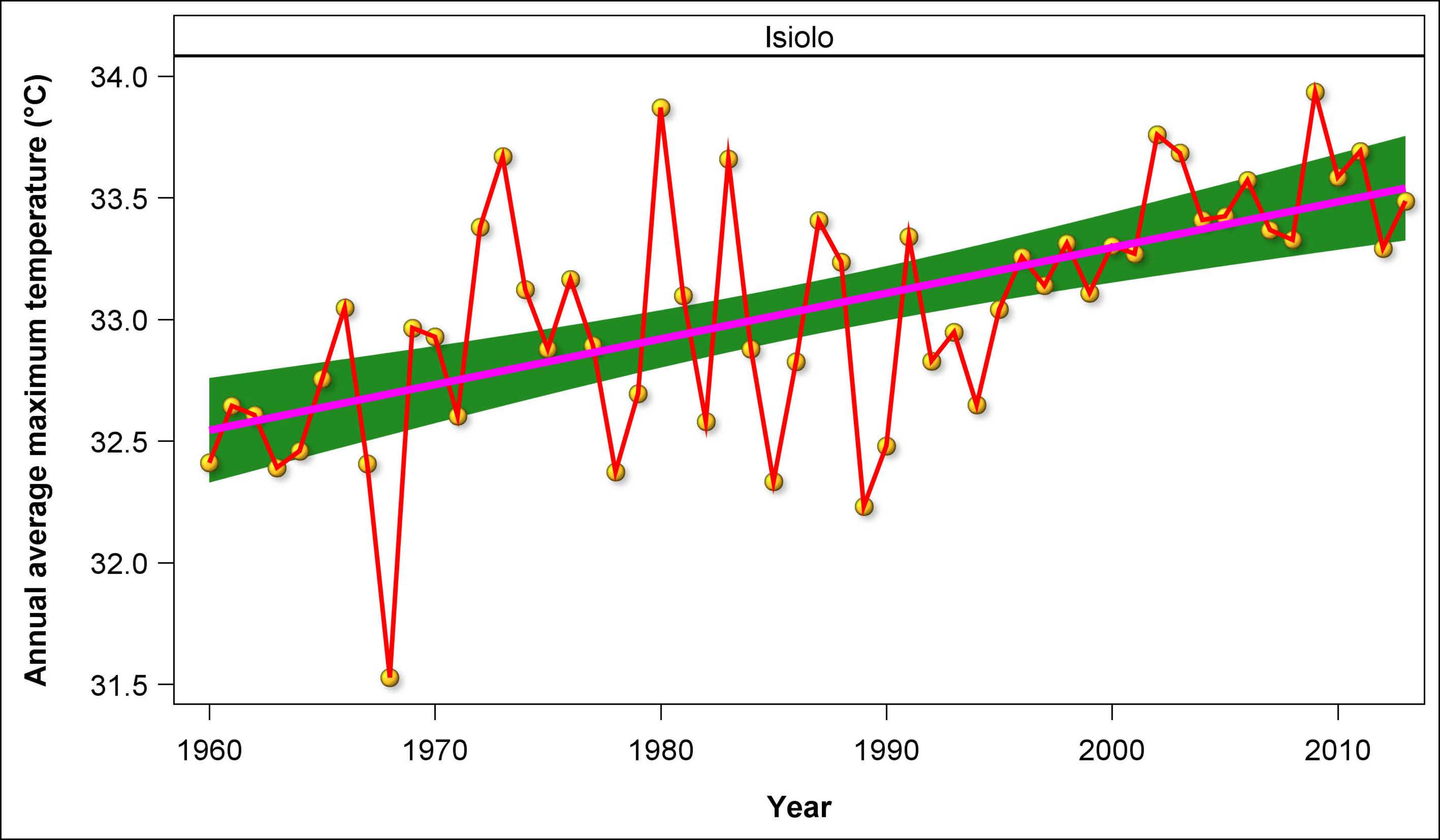

Isiolo
34.0
33.5
33.0
32.5
32.0
31.5
Annual average maximum temperature (°C)
1960
1970
1980
1990
2000
2010
Year

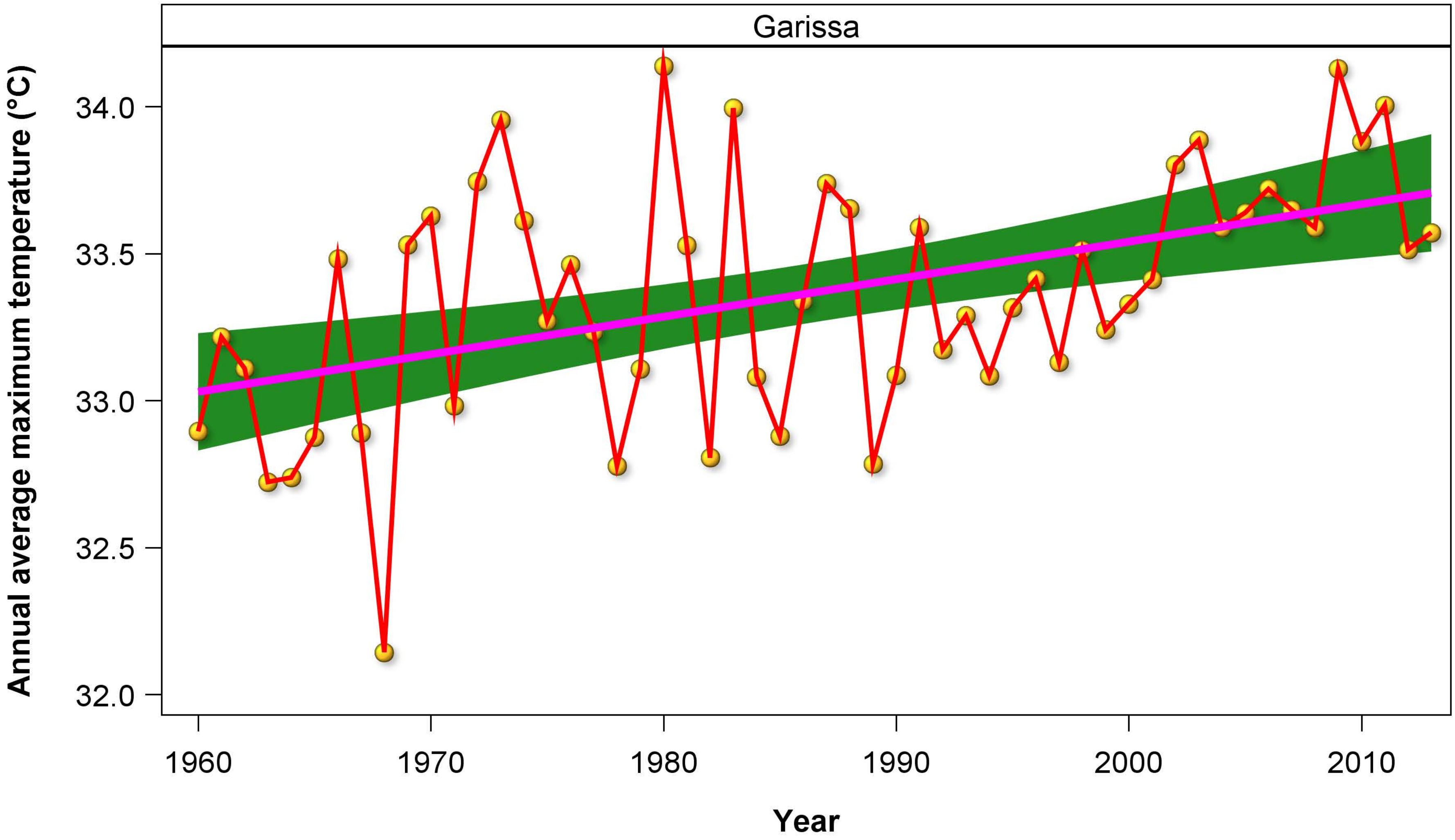
Garissa
Annual average maximum temperature (°C)
34.0
33.5
33.0
32.5
32.0
1960
1970
1980
1990
2000
2010
Year

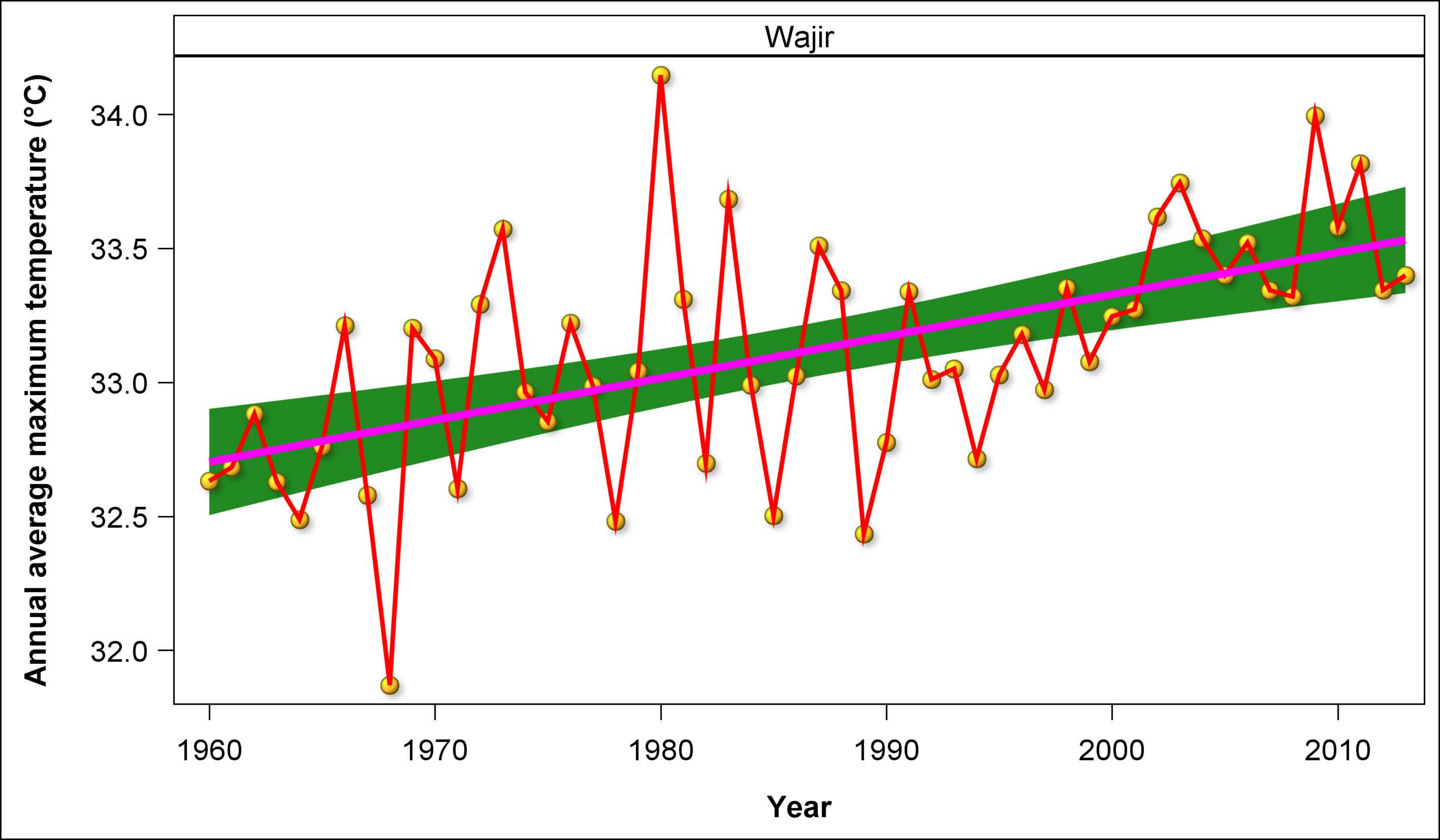
Wajir
Annual average maximum temperature (°C)
34.0
33.5
33.0
32.5
32.0
1960
1970
1980
1990
2000
2010
Year

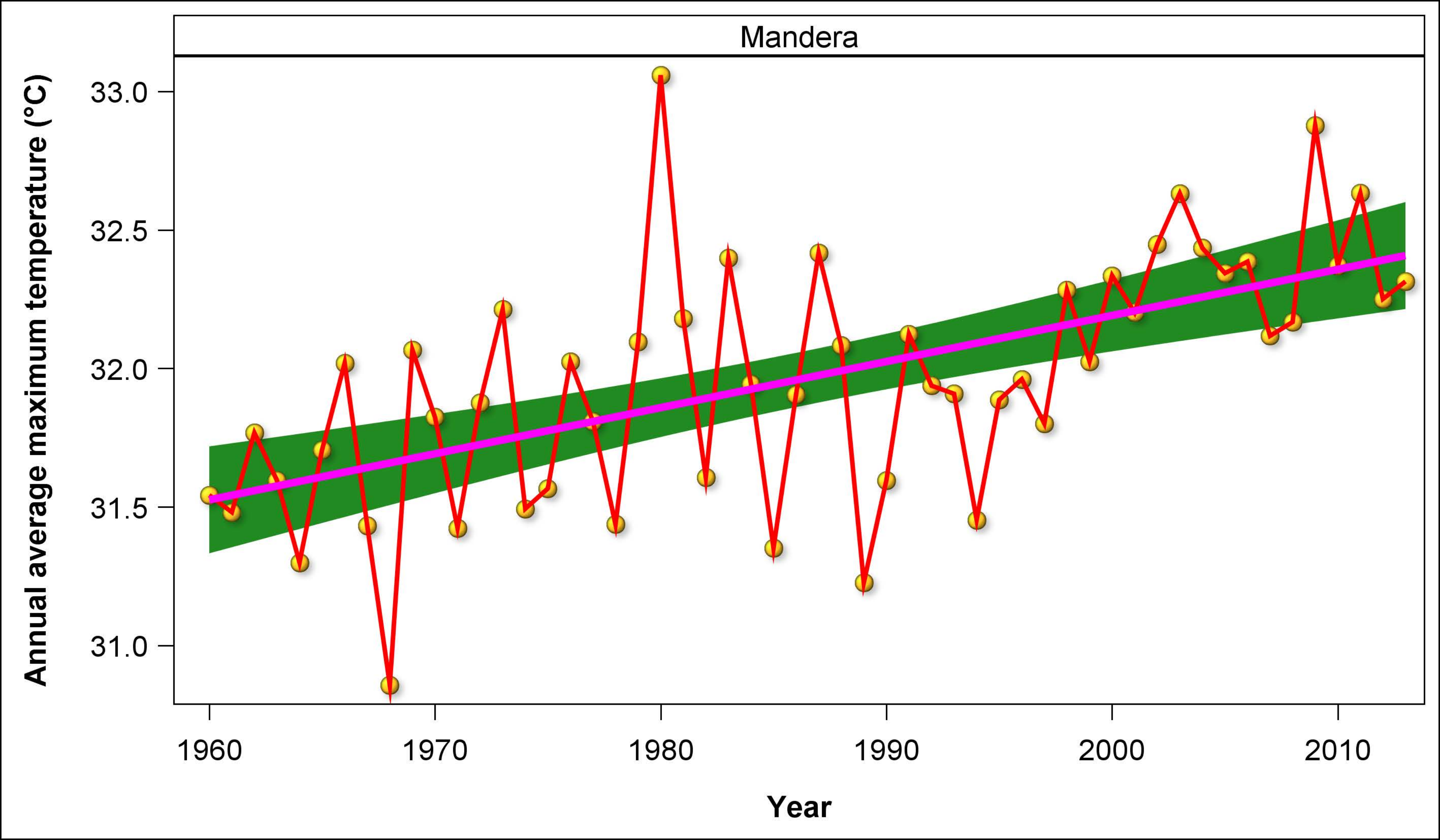
Mandera
Annual average maximum temperature (°C)
33.0
32.5
32.0
31.5
31.0
1960
1970
1980
1990
2000
2010
Year

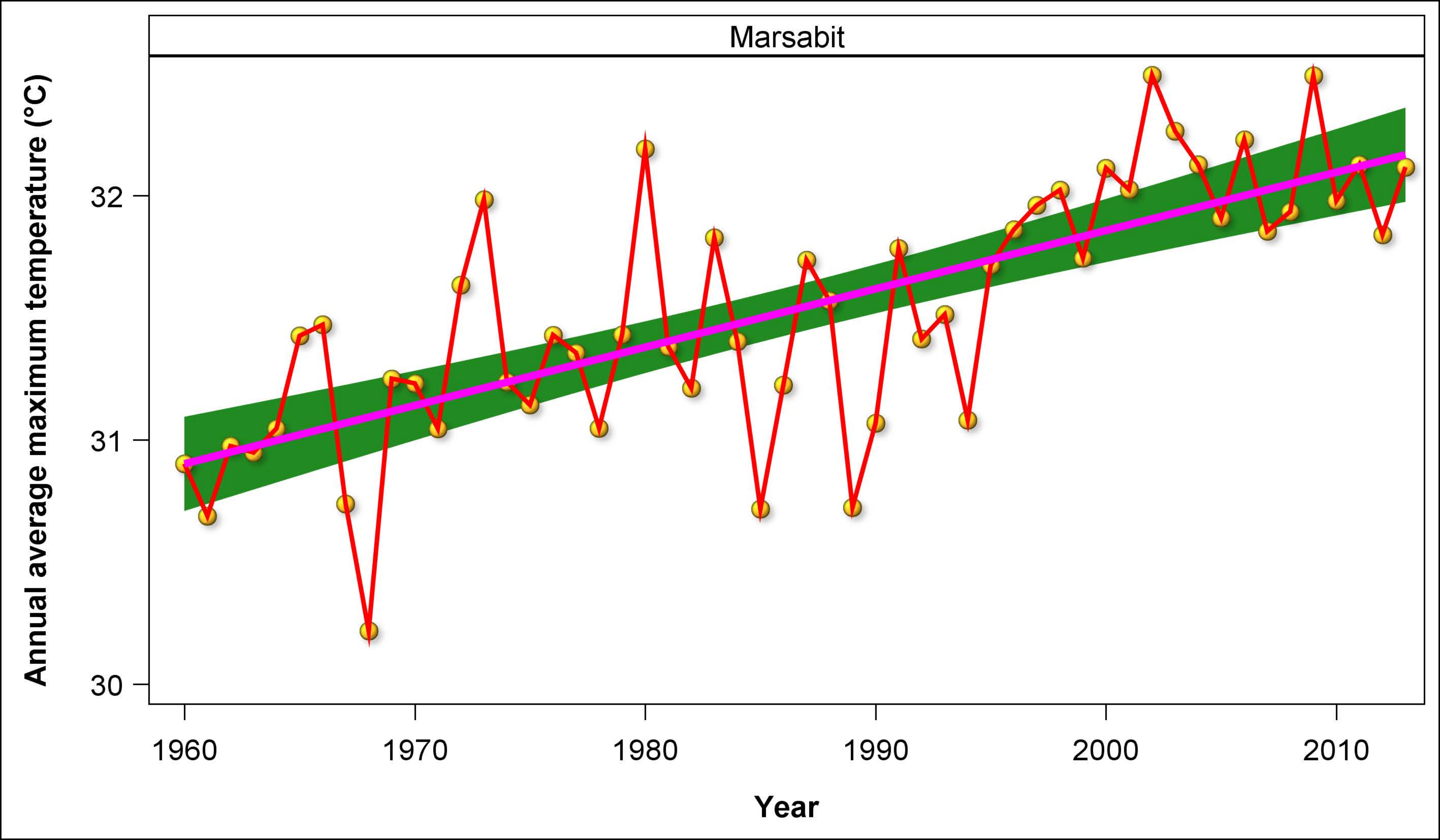

Marsabit
Annual average maximum temperature (°C)
32
31
30
1960
1970
1980
1990
2000
2010
Year

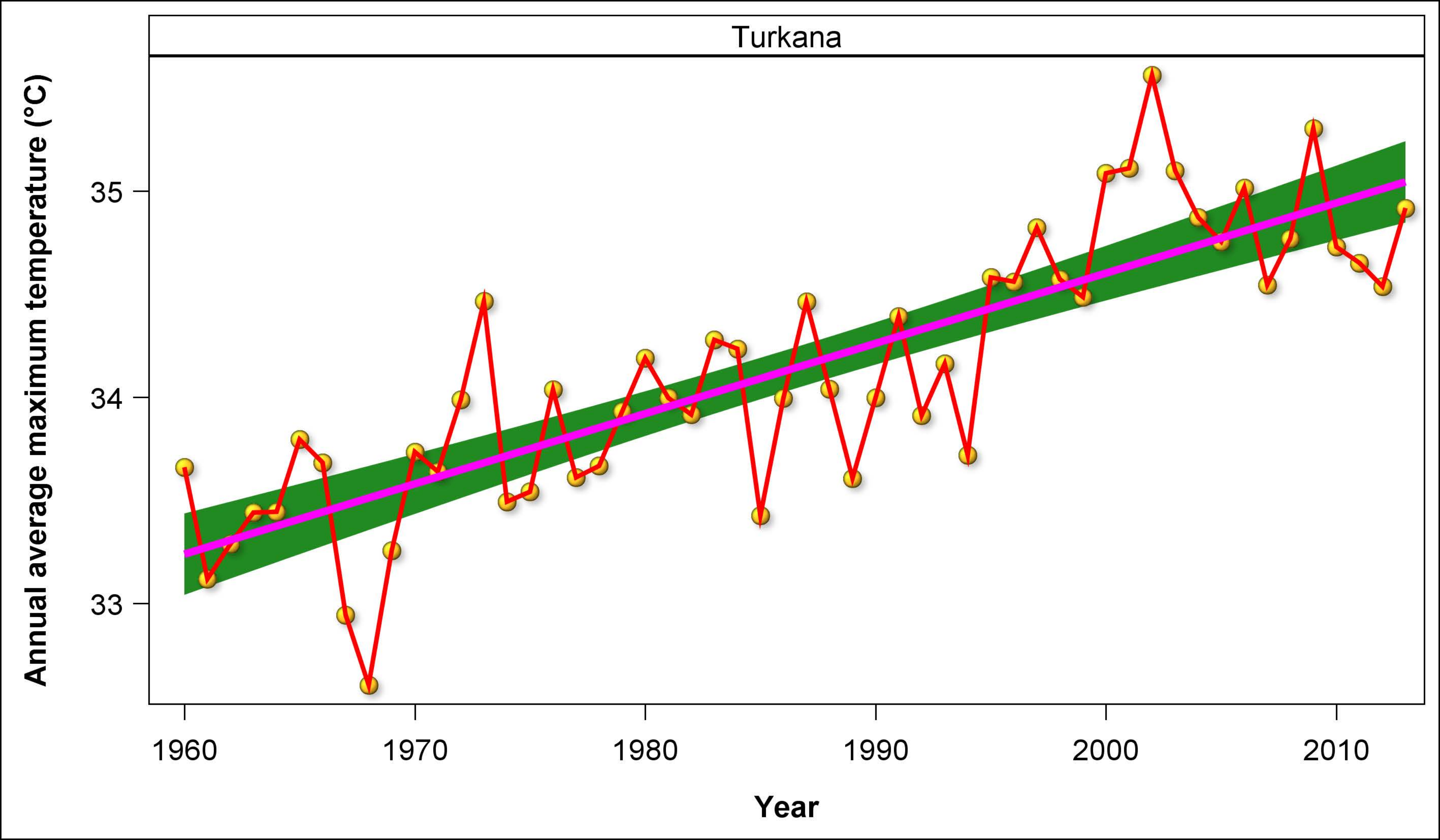
Turkana
Annual average maximum temperature (°C)
35
34
33
1960
1970
1980
1990
2000
2010
Year

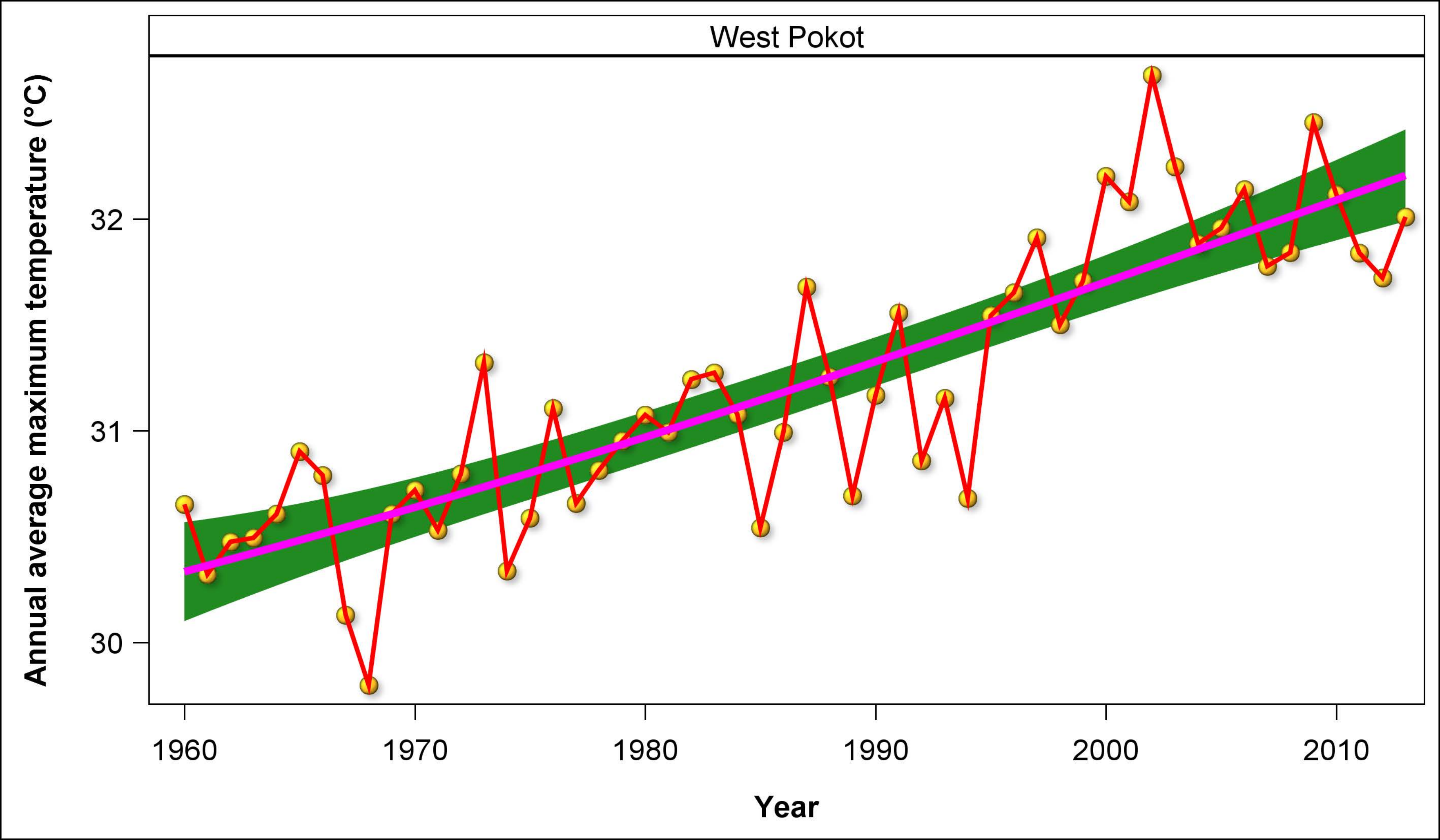
West Pokot
Annual average maximum temperature (°C)
32
31
30
1960
1970
1980
1990
2000
2010
Year

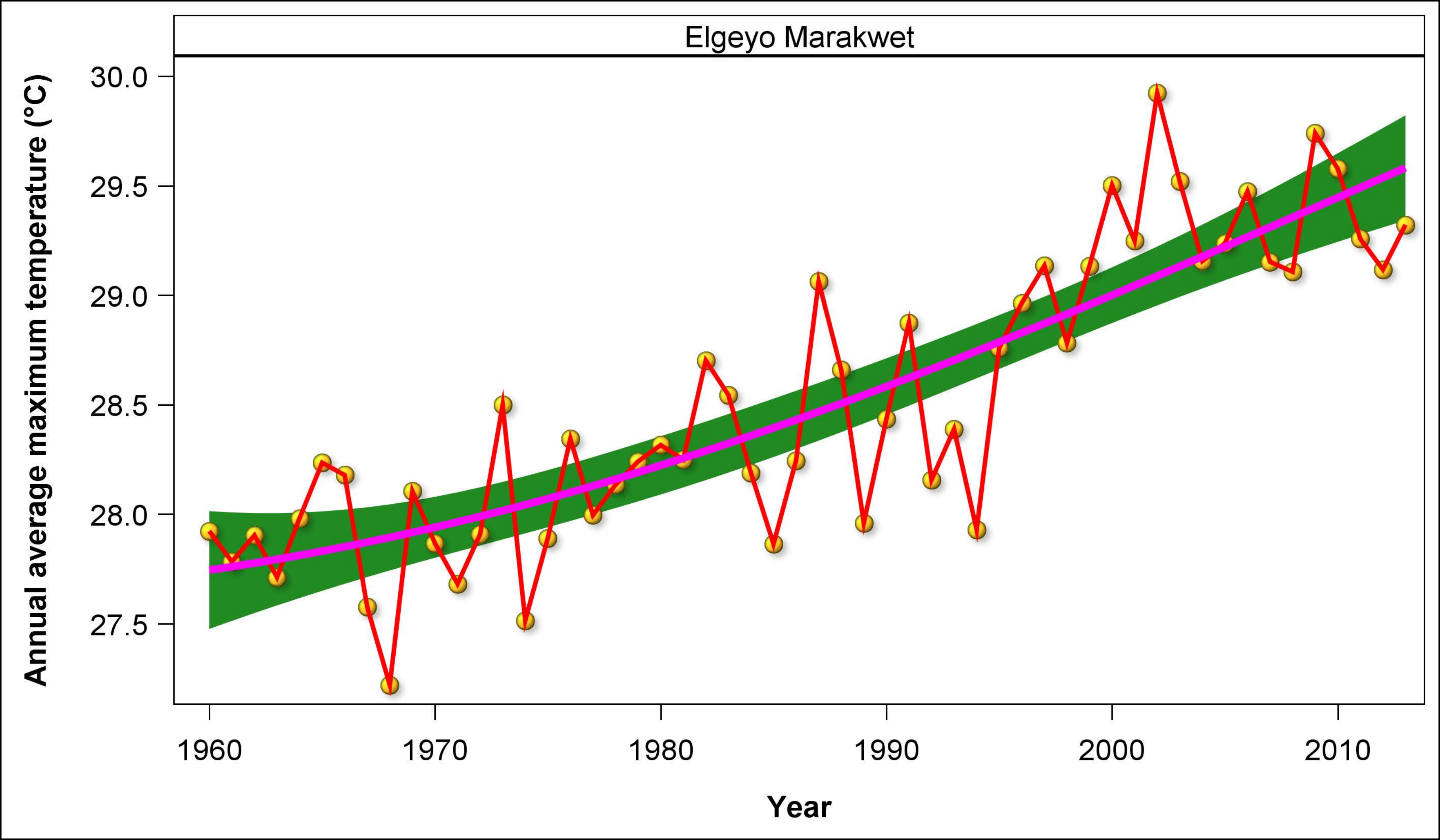
Elgeyo Marakwet
Annual average maximum temperature (°C)
30.0
29.5
29.0
28.5
28.0
27.5
1960
1970
1980
1990
2000
2010
Year
