# Supplementary material for: Extreme Wildlife Declines and Concurrent Increase in Livestock Numbers in Kenya: What Are the Causes?
Source: PLoS One. 2016 Sep 27;11(9):e0163249. doi: 10.1371/journal.pone.0163249 (PMC5039022; doi:10.1371/journal.pone.0163249)
Supplement: S25 Fig — The filled goldenrod circles denote the observations, the solid blue curve the fitted trend curve whereas the chartreuse band the pointwise 95% confidence band. (PDF) [file pone.0163249.s035.pdf]

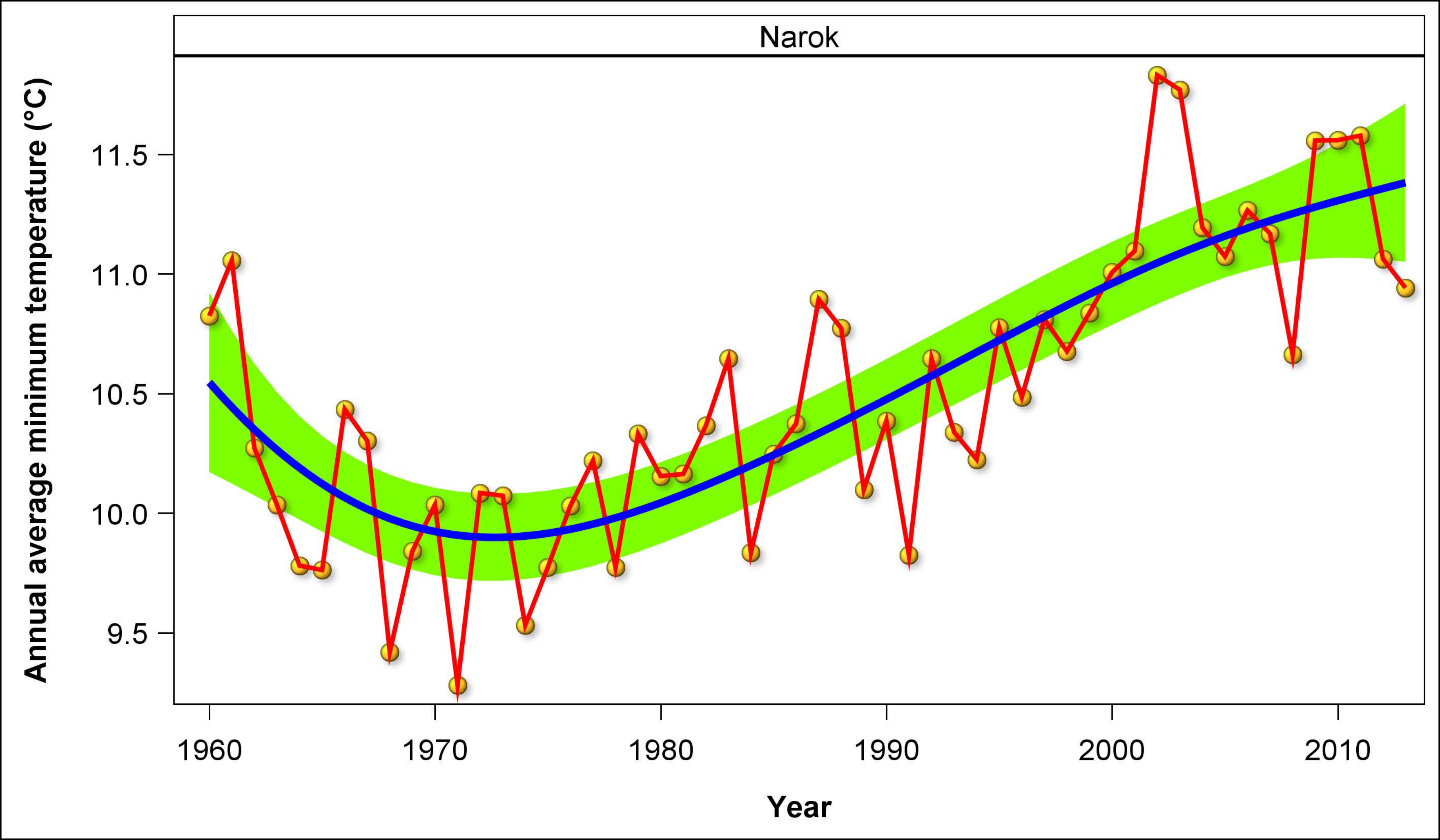

Narok
Annual average minimum temperature (°C)
11.5
11.0
10.5
10.0
9.5
1960
1970
1980
1990
2000
2010
Year

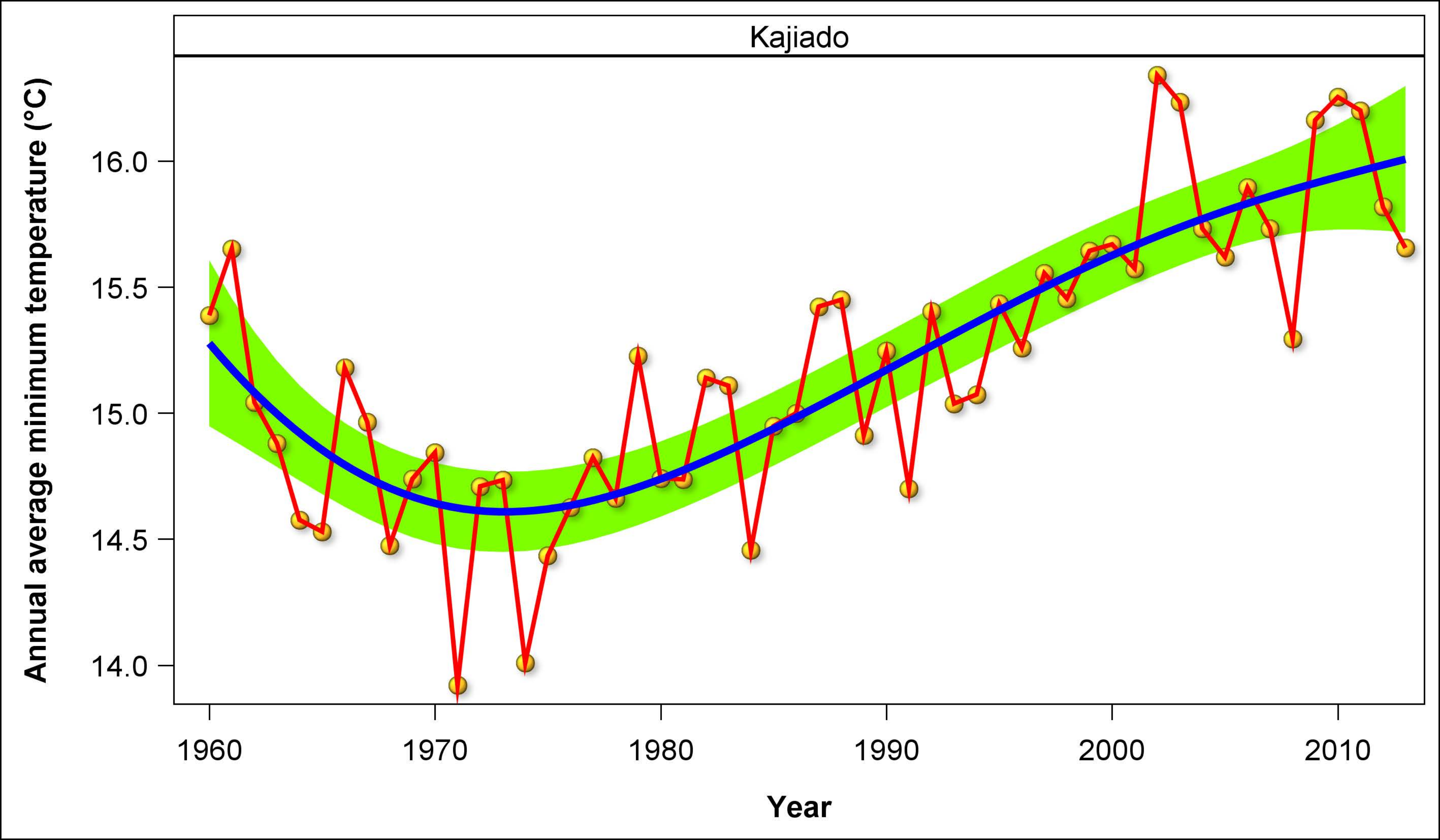
Kajiado
Annual average minimum temperature (°C)
16.0
15.5
15.0
14.5
14.0
1960
1970
1980
1990
2000
2010
Year

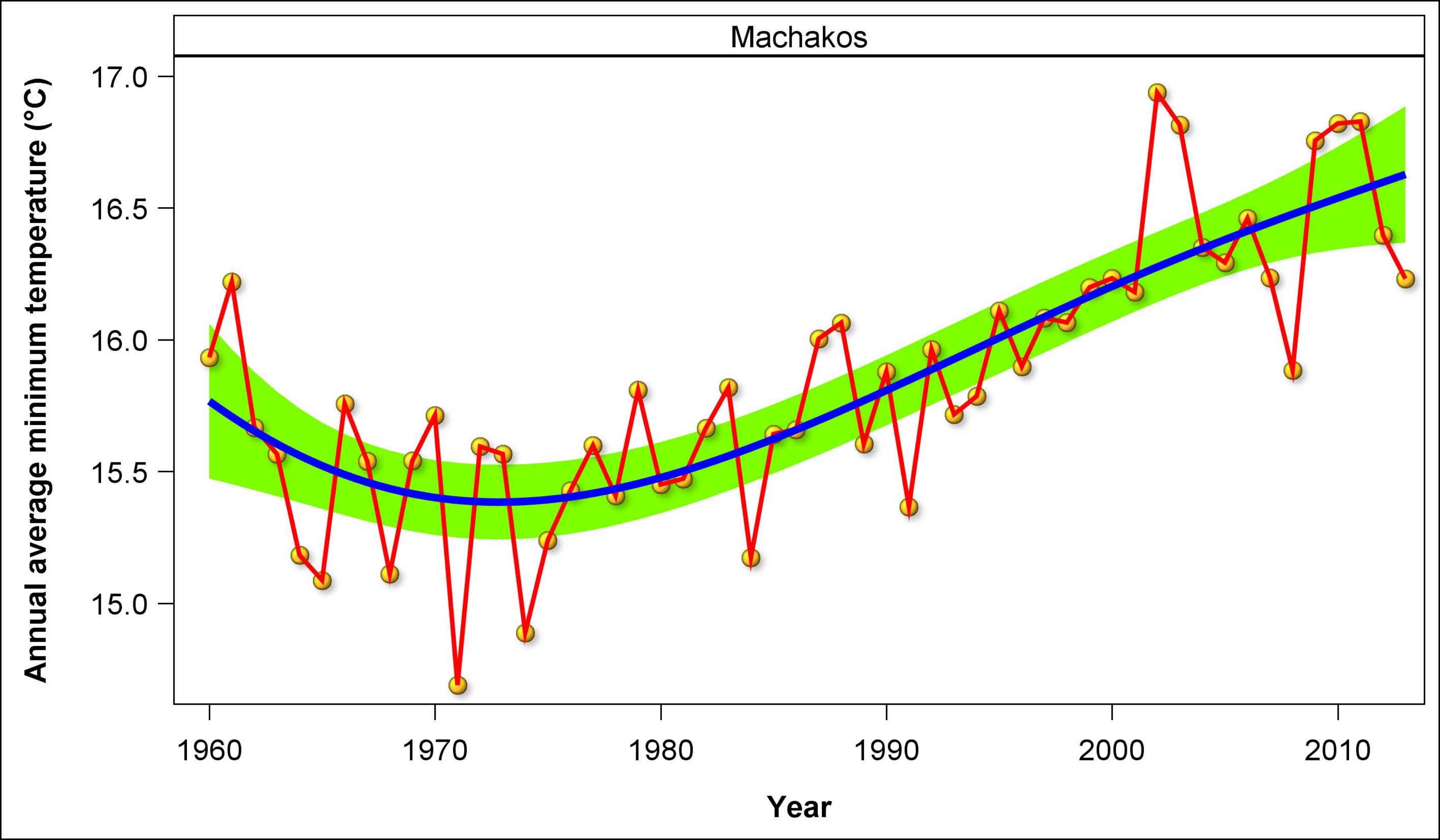
Machakos
Annual average minimum temperature (°C)
17.0
16.5
16.0
15.5
15.0
1960
1970
1980
1990
2000
2010
Year

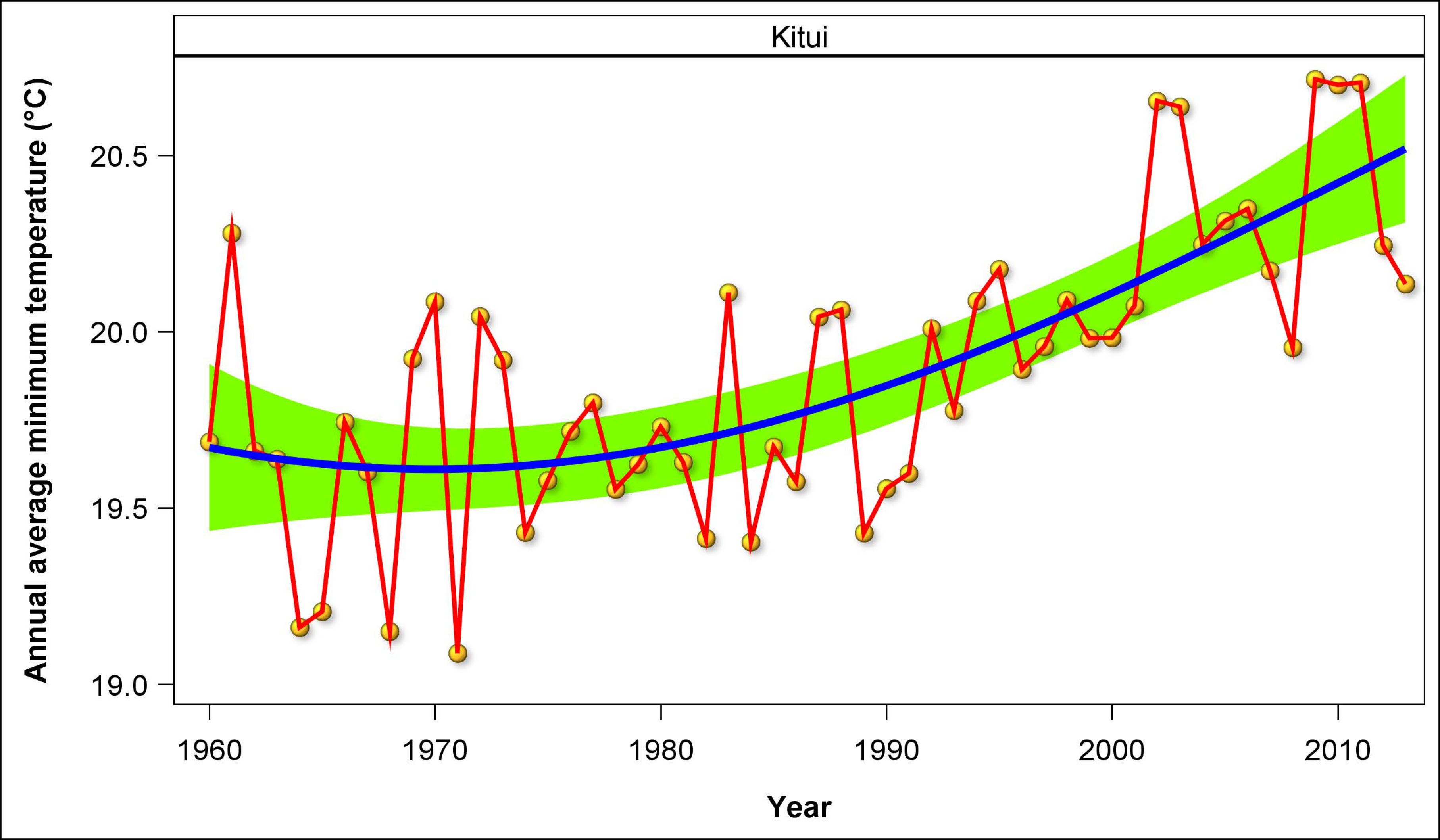

Kitui
Annual average minimum temperature (°C)
20.5
20.0
19.5
19.0
1960
1970
1980
1990
2000
2010
Year

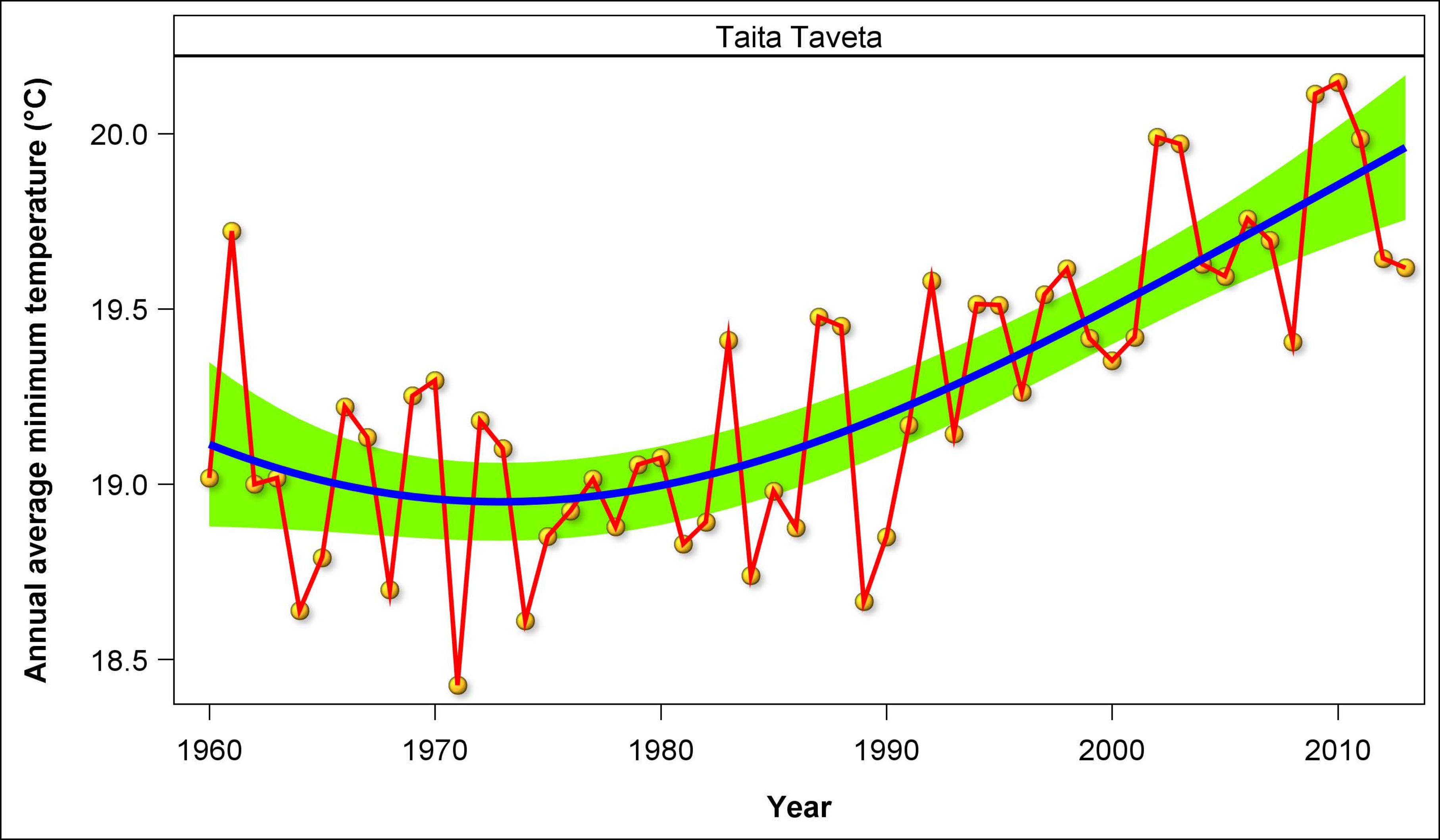
Taita Taveta
Annual average minimum temperature (°C)
20.0
19.5
19.0
18.5
1960
1970
1980
1990
2000
2010
Year

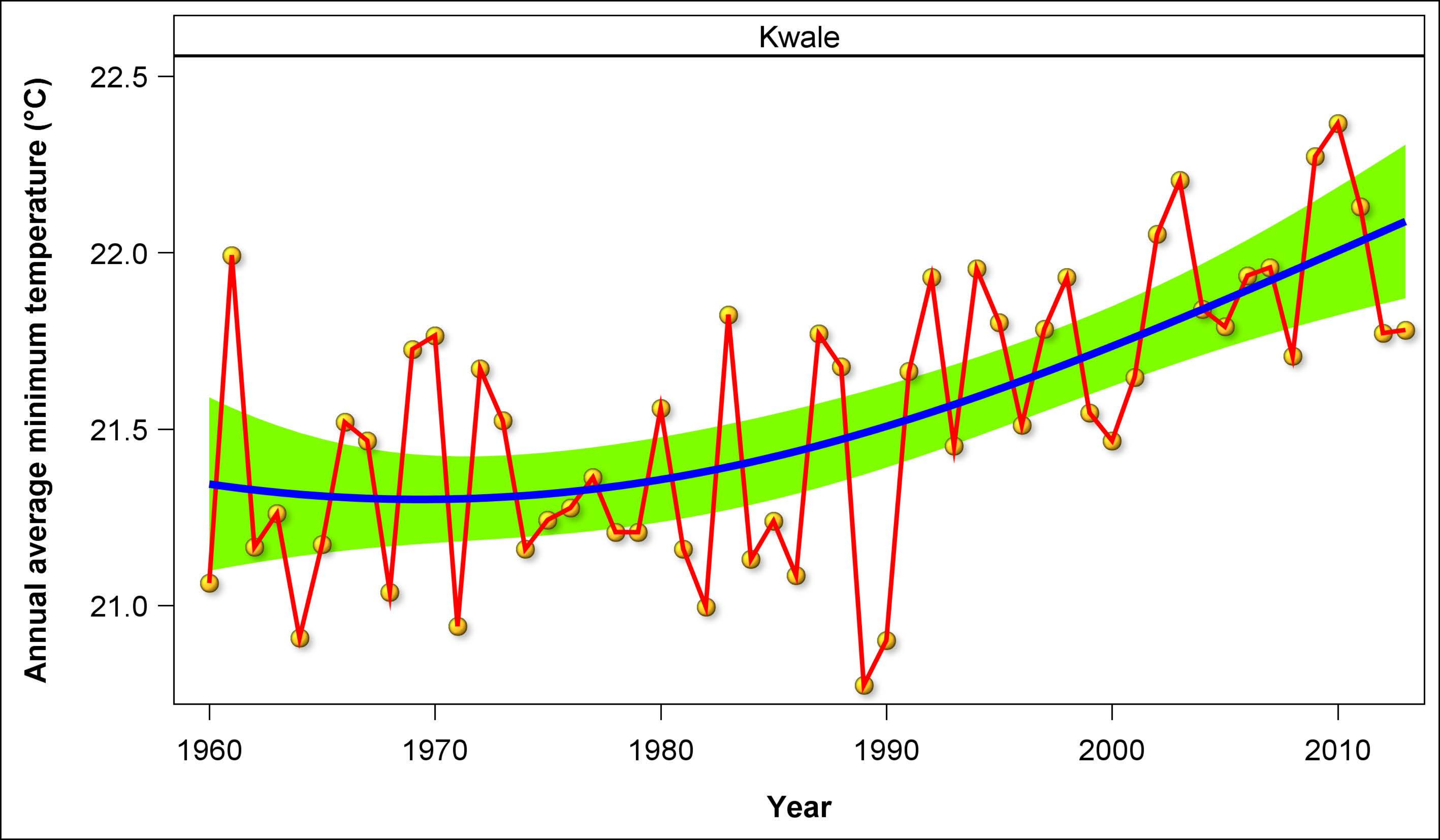
Kwale
Annual average minimum temperature (°C)
22.5
22.0
21.5
21.0
1960
1970
1980
1990
2000
2010
Year

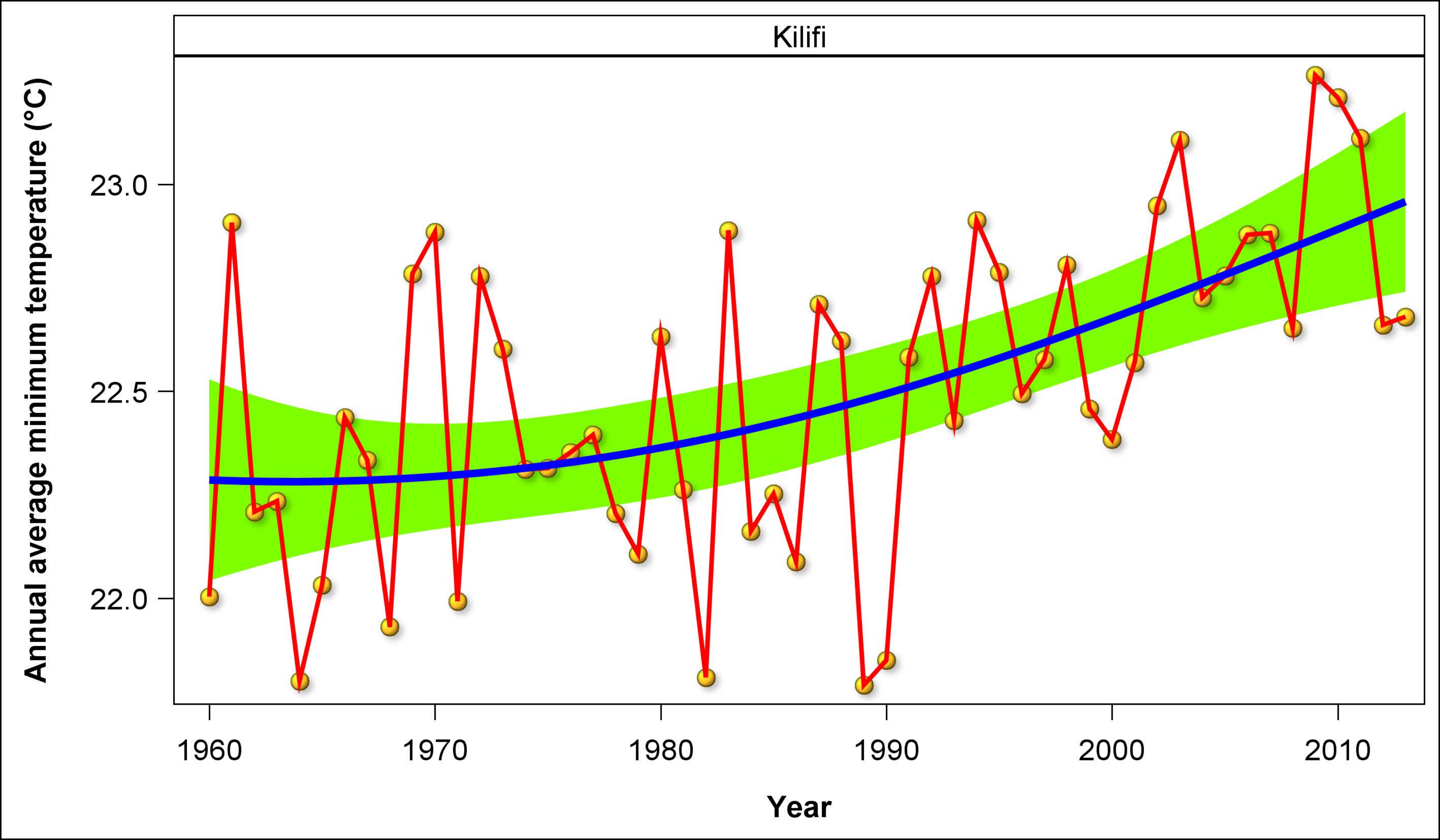
Kilifi
Annual average minimum temperature (°C)
23.0
22.5
22.0
1960
1970
1980
1990
2000
2010
Year

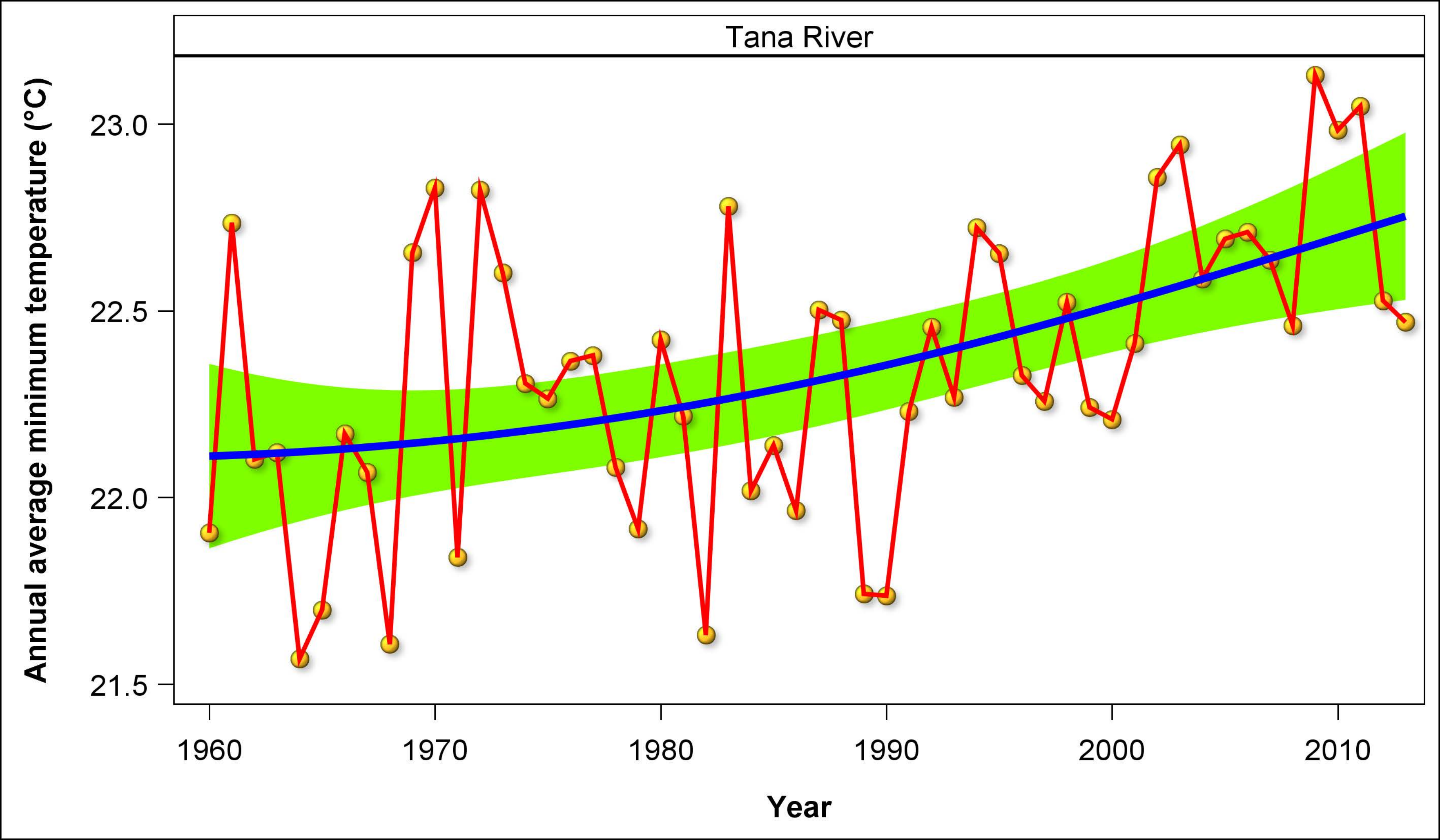
Tana River
Annual average minimum temperature (°C)
23.0
22.5
22.0
21.5
1960
1970
1980
1990
2000
2010
Year

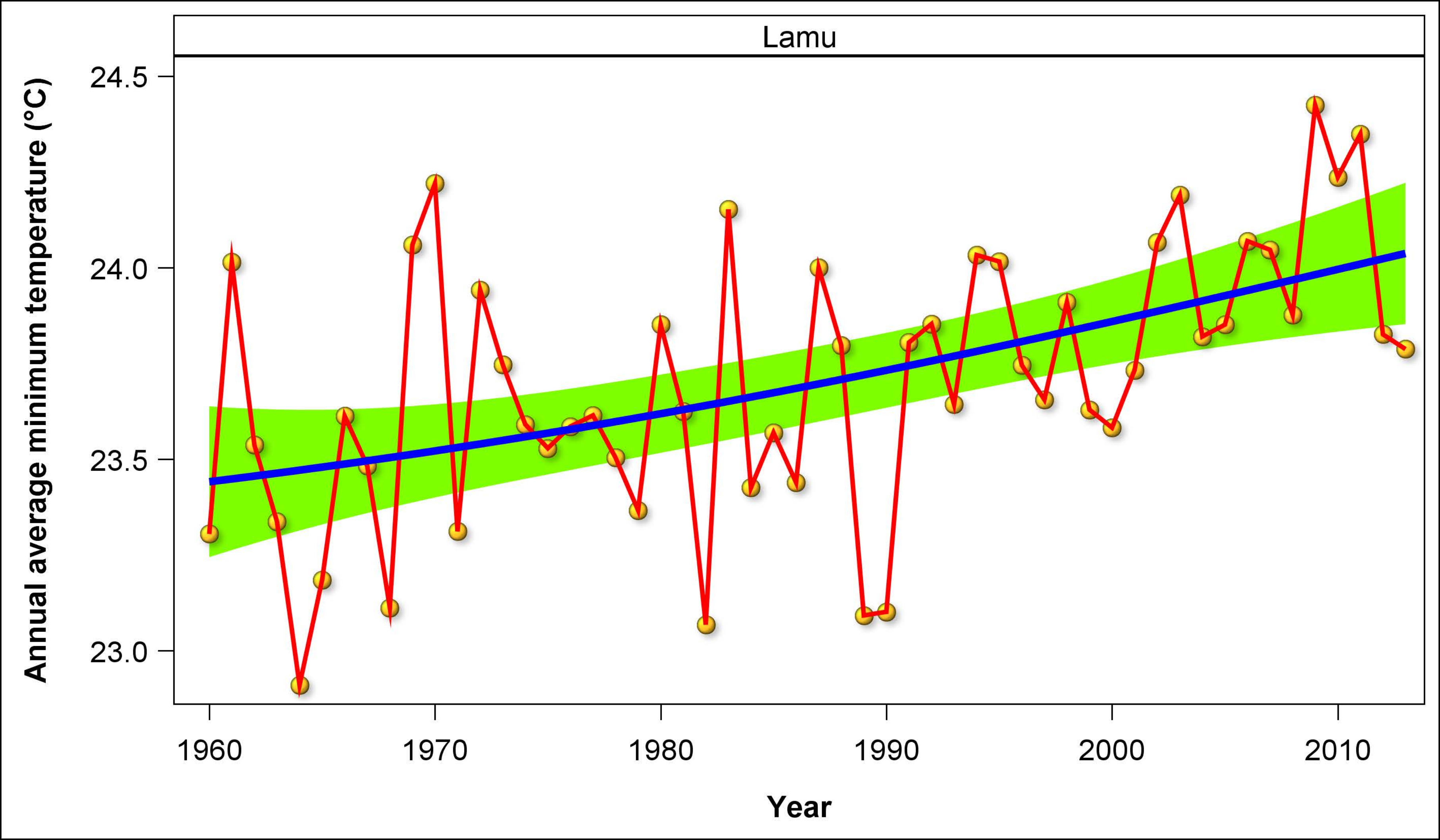
Lamu
Annual average minimum temperature (°C)
24.5
24.0
23.5
23.0
1960
1970
1980
1990
2000
2010
Year

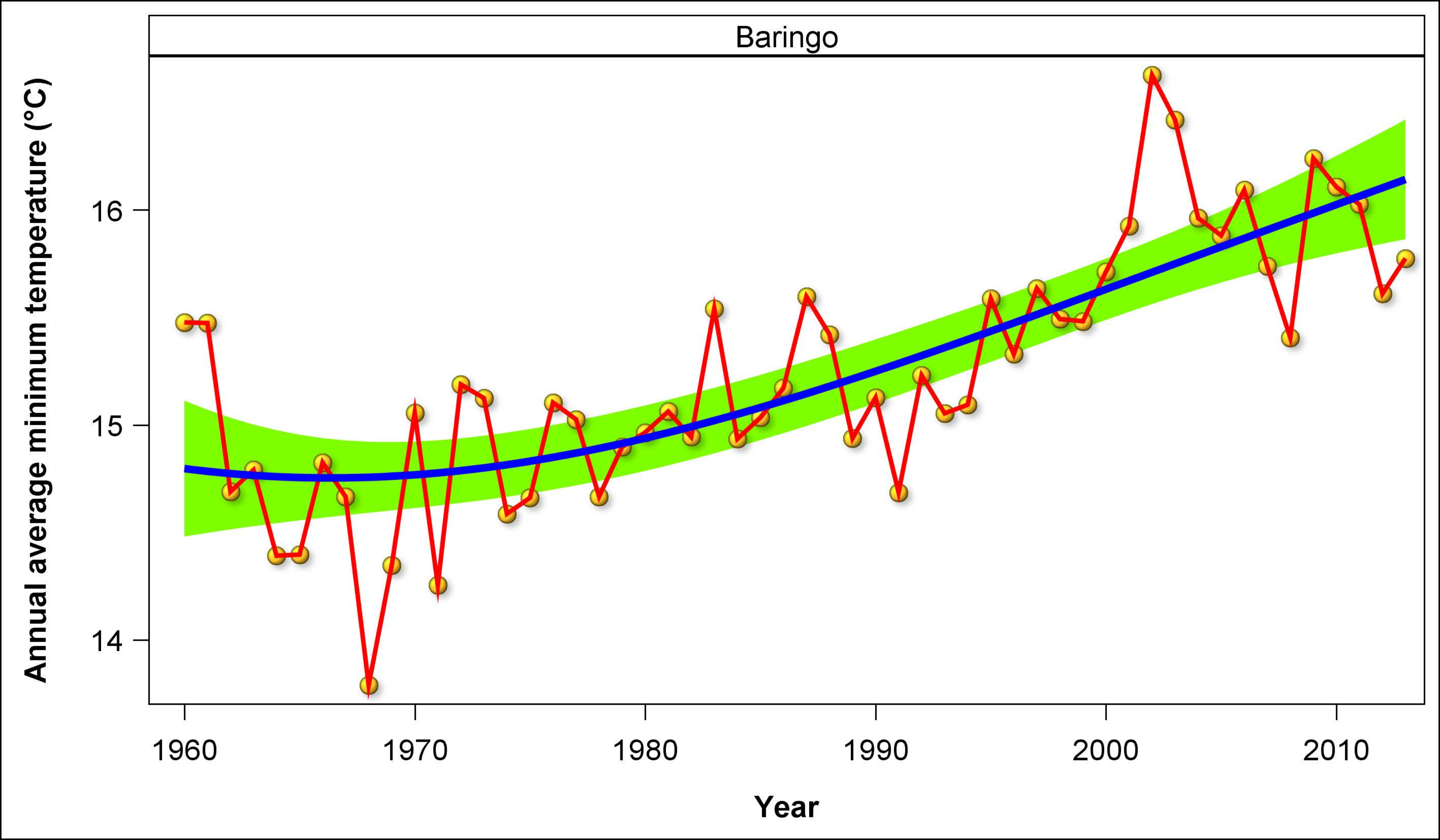
Baringo
Annual average minimum temperature (°C)
14
15
16
1960
1970
1980
1990
2000
2010
Year

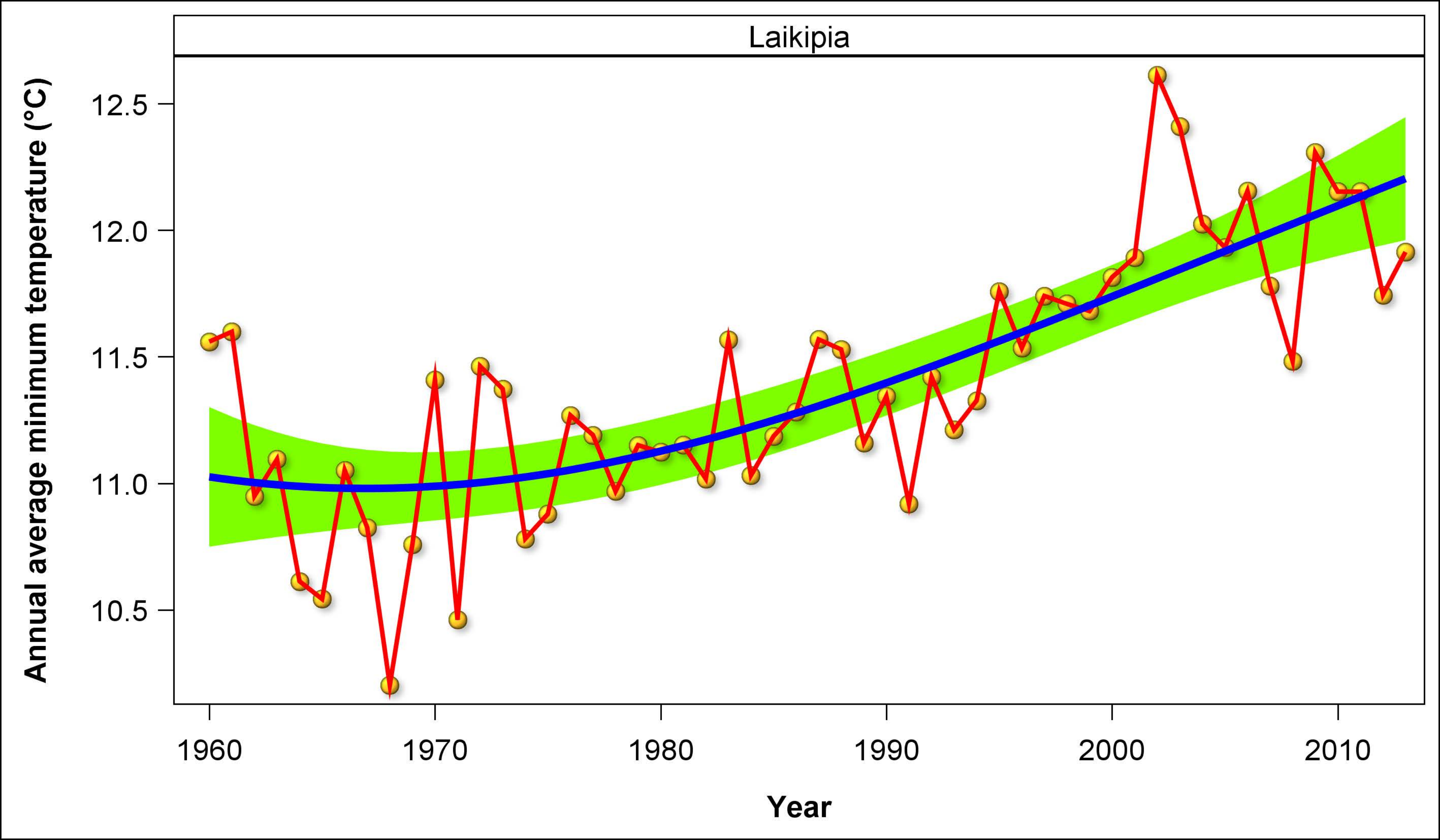
Laikipia
Annual average minimum temperature (°C)
12.5
12.0
11.5
11.0
10.5
1960
1970
1980
1990
2000
2010
Year

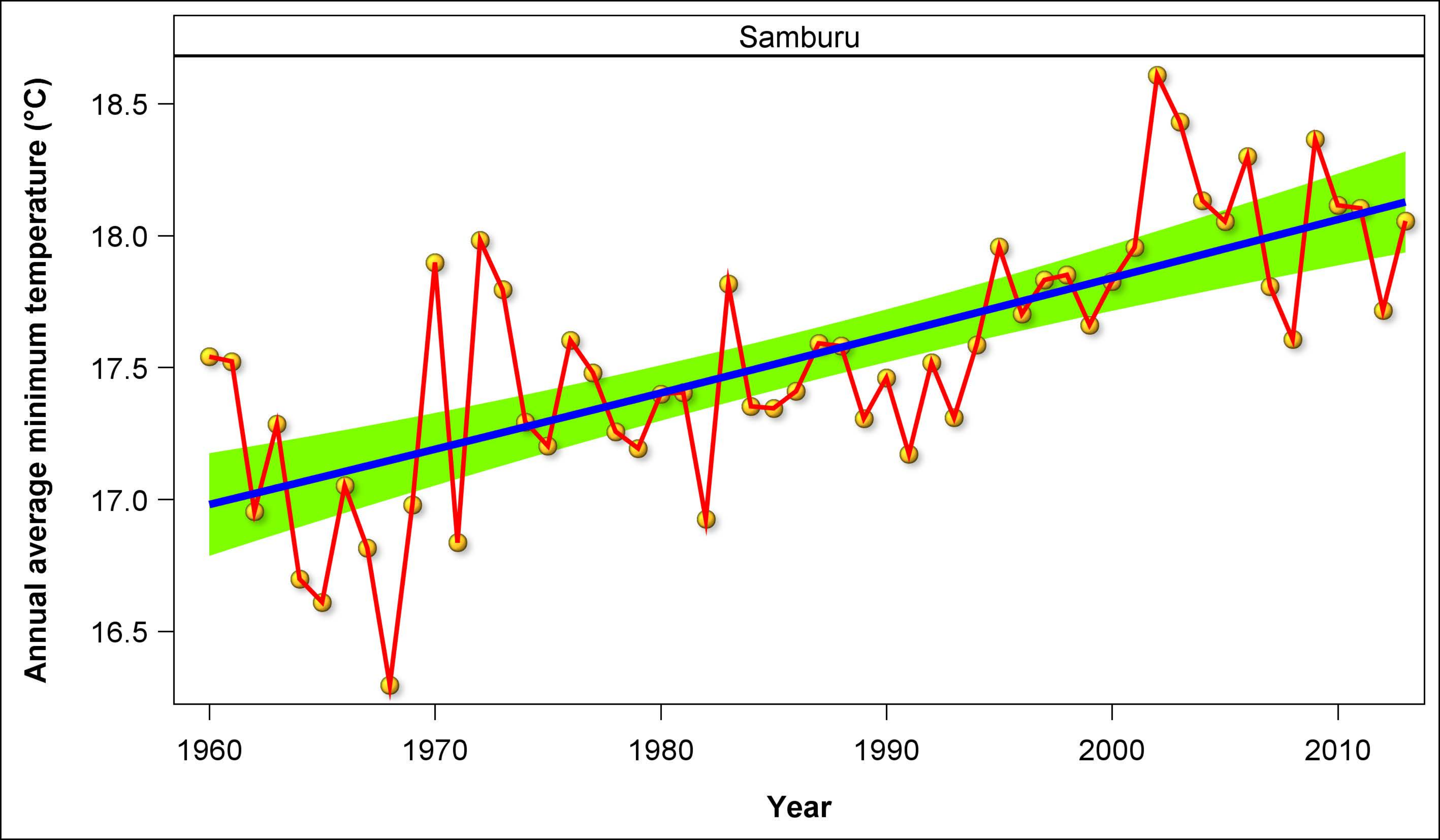
Samburu
Annual average minimum temperature (°C)
18.5
18.0
17.5
17.0
16.5
1960
1970
1980
1990
2000
2010
Year

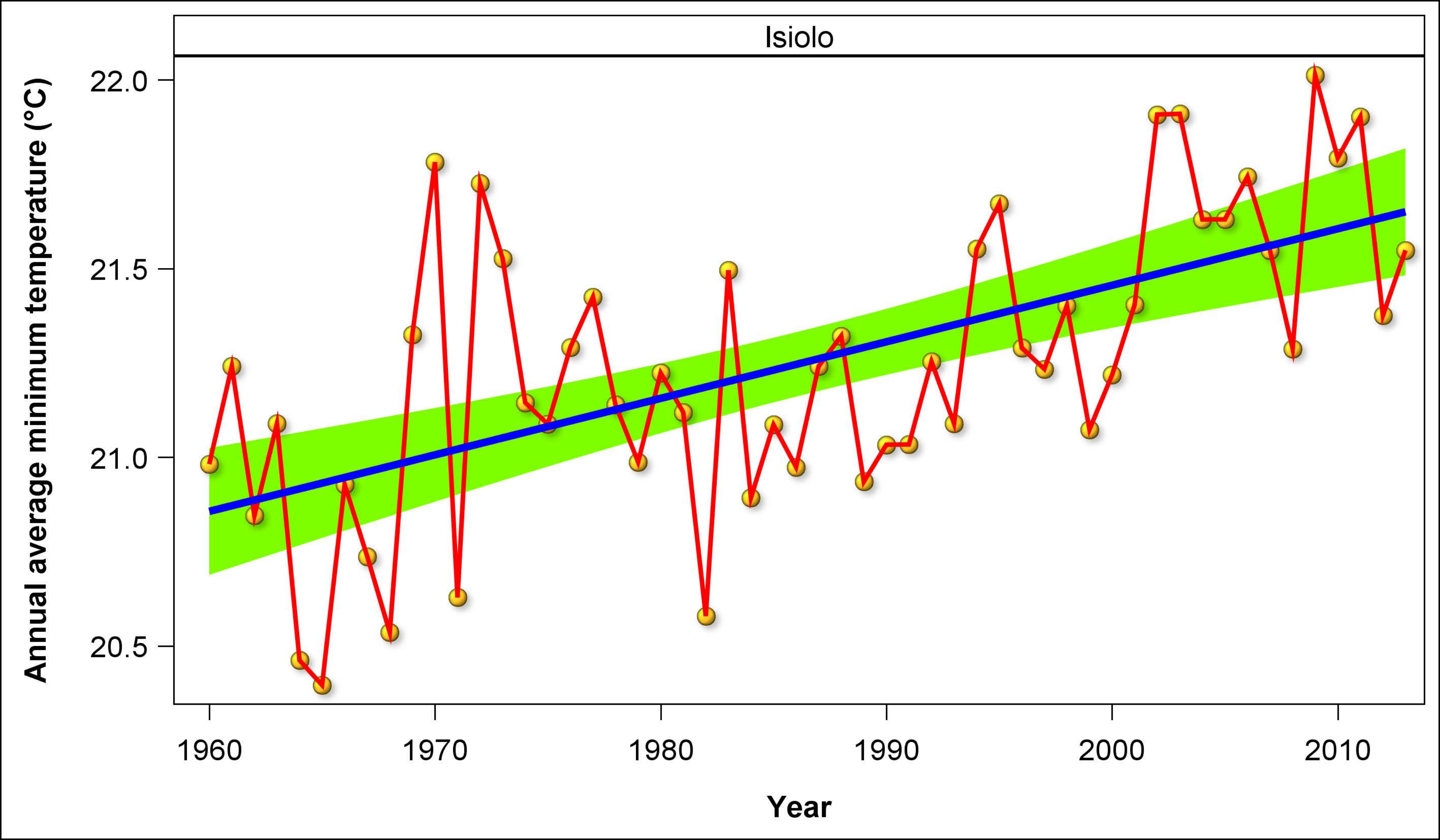
Isiolo
Annual average minimum temperature (°C)
22.0
21.5
21.0
20.5
1960
1970
1980
1990
2000
2010
Year

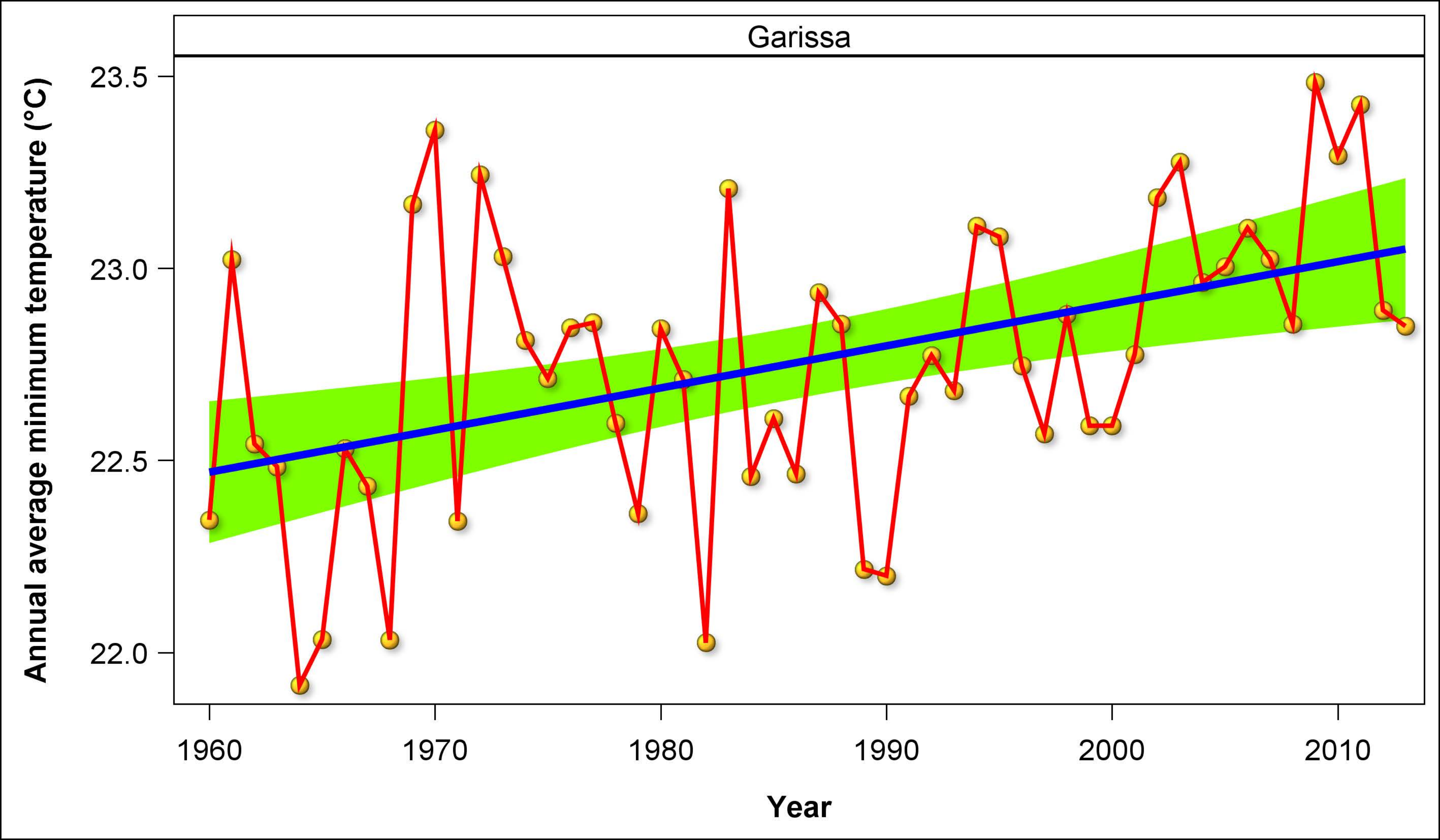
Garissa
Annual average minimum temperature (°C)
23.5
23.0
22.5
22.0
1960
1970
1980
1990
2000
2010
Year

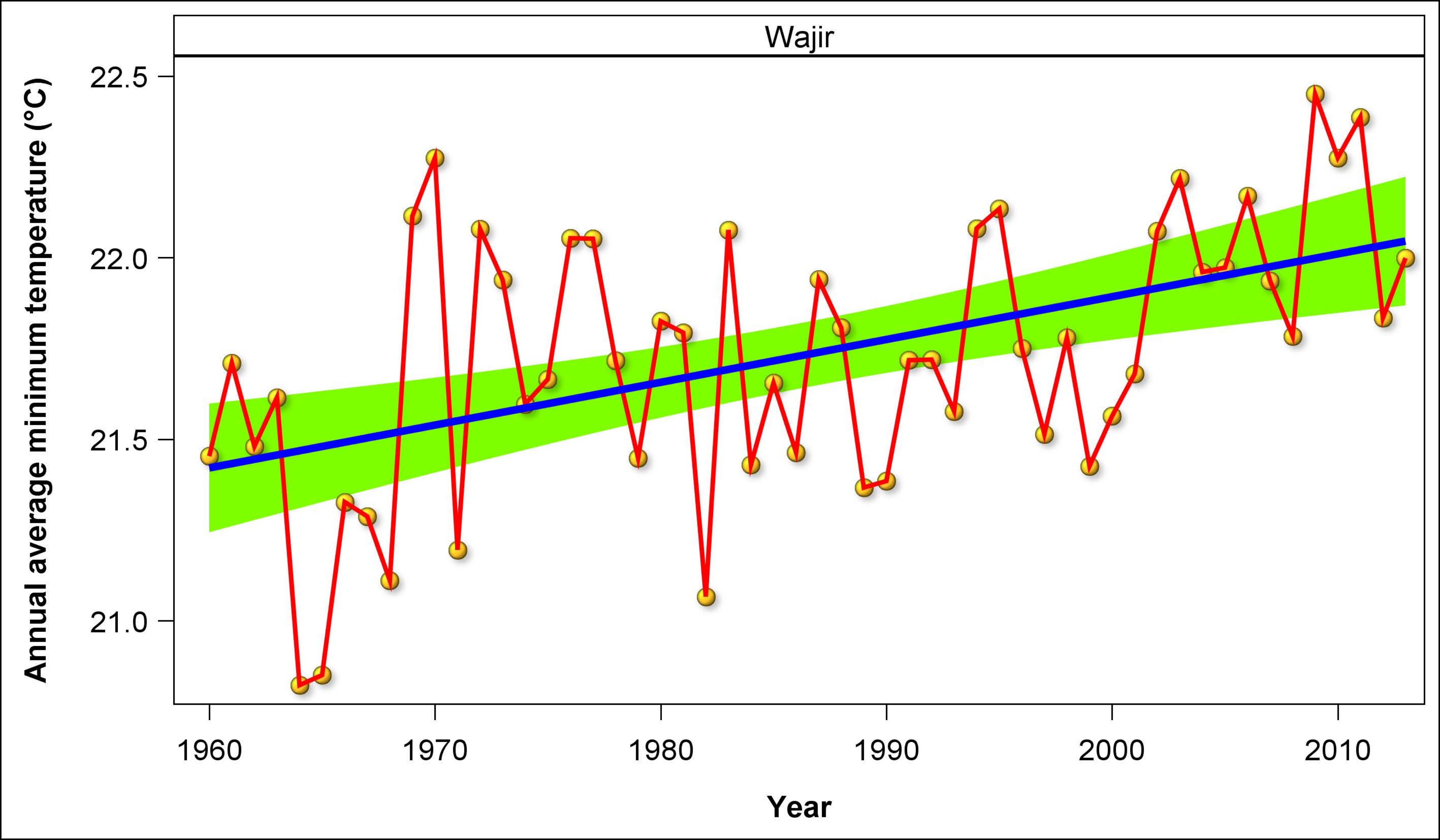

Wajir
Annual average minimum temperature (°C)
22.5
22.0
21.5
21.0
1960
1970
1980
1990
2000
2010
Year

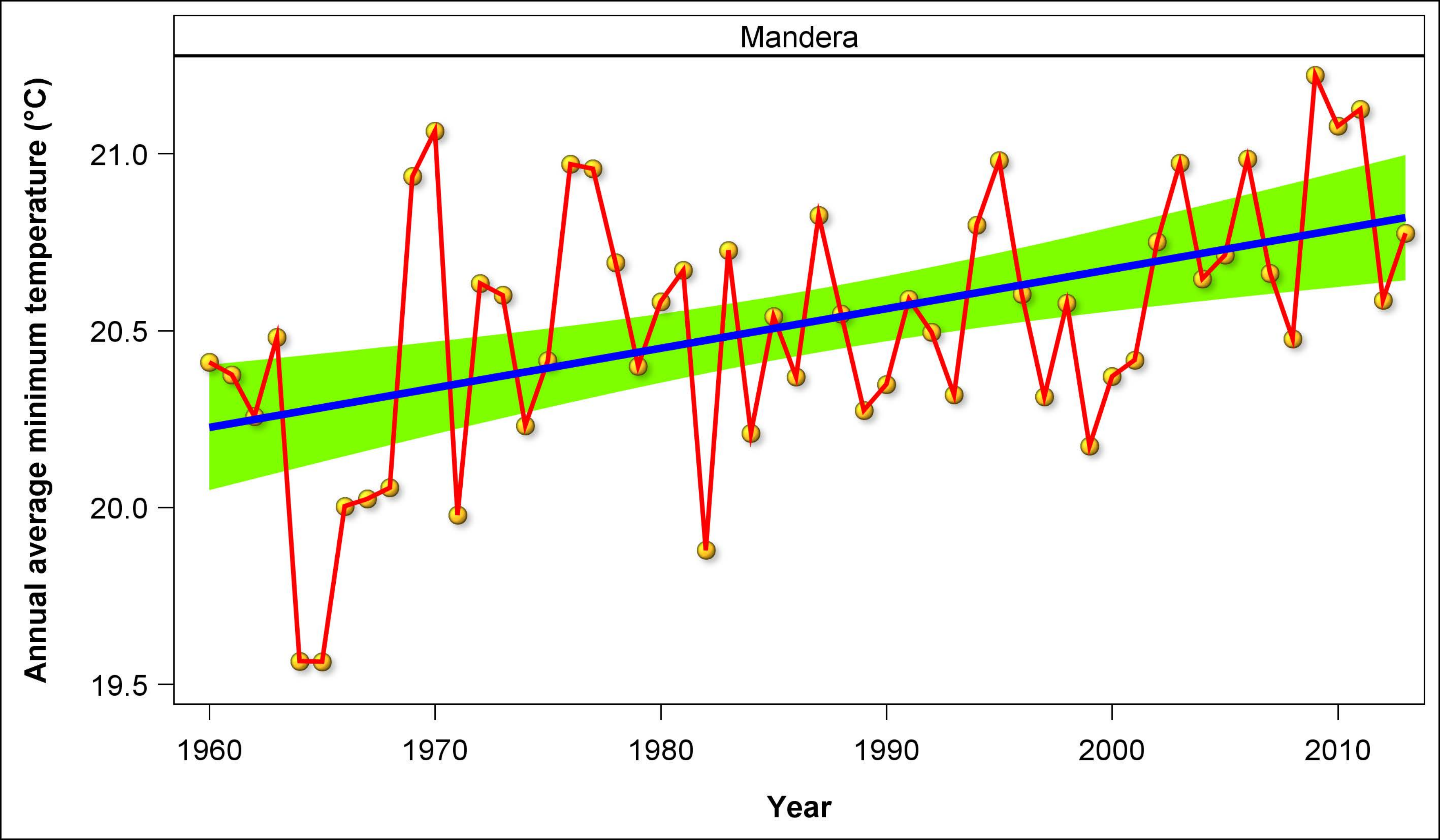
Mandera
Annual average minimum temperature (°C)
21.0
20.5
20.0
19.5
1960
1970
1980
1990
2000
2010
Year

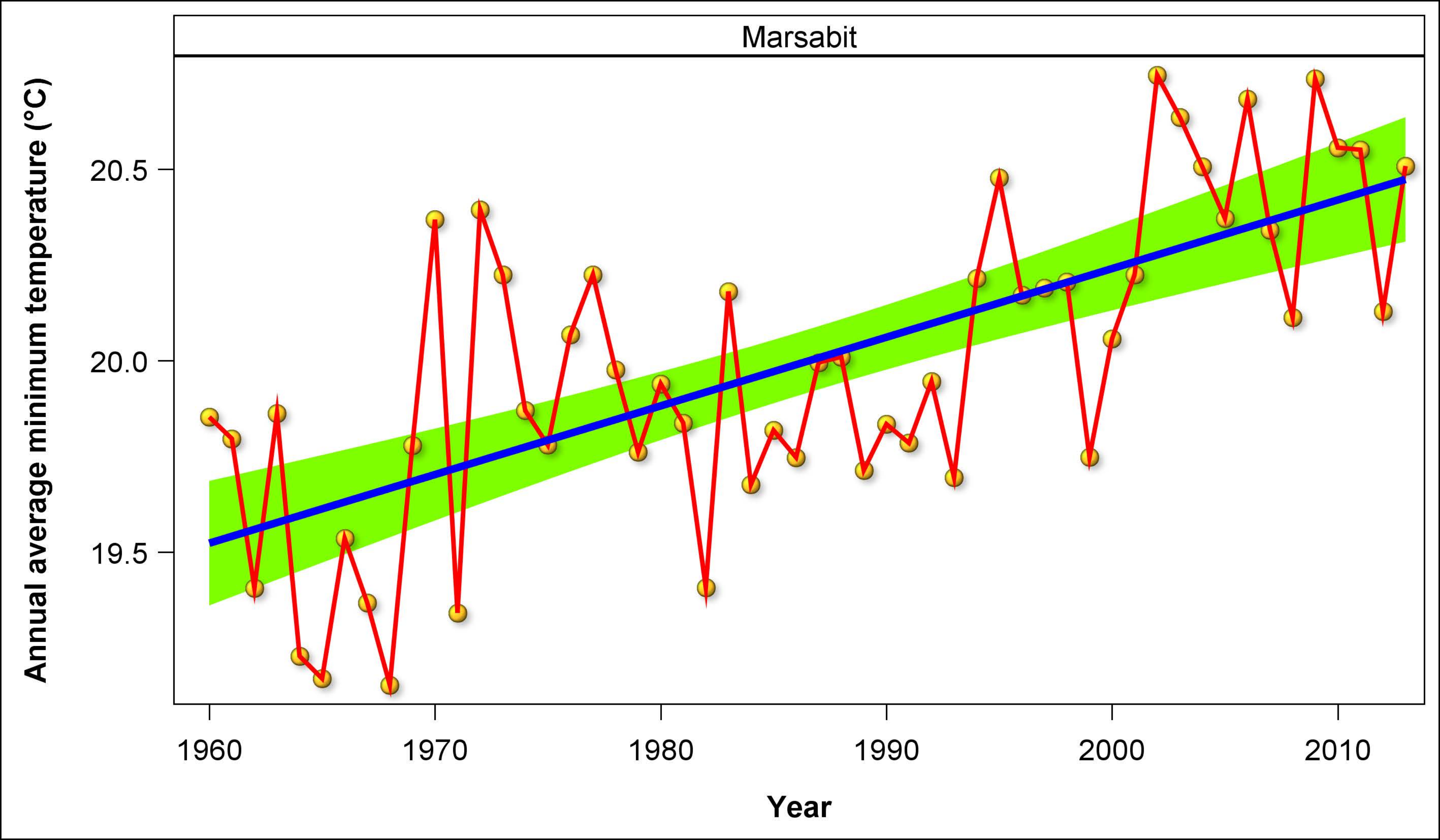
Marsabit
Annual average minimum temperature (°C)
20.5
20.0
19.5
1960
1970
1980
1990
2000
2010
Year

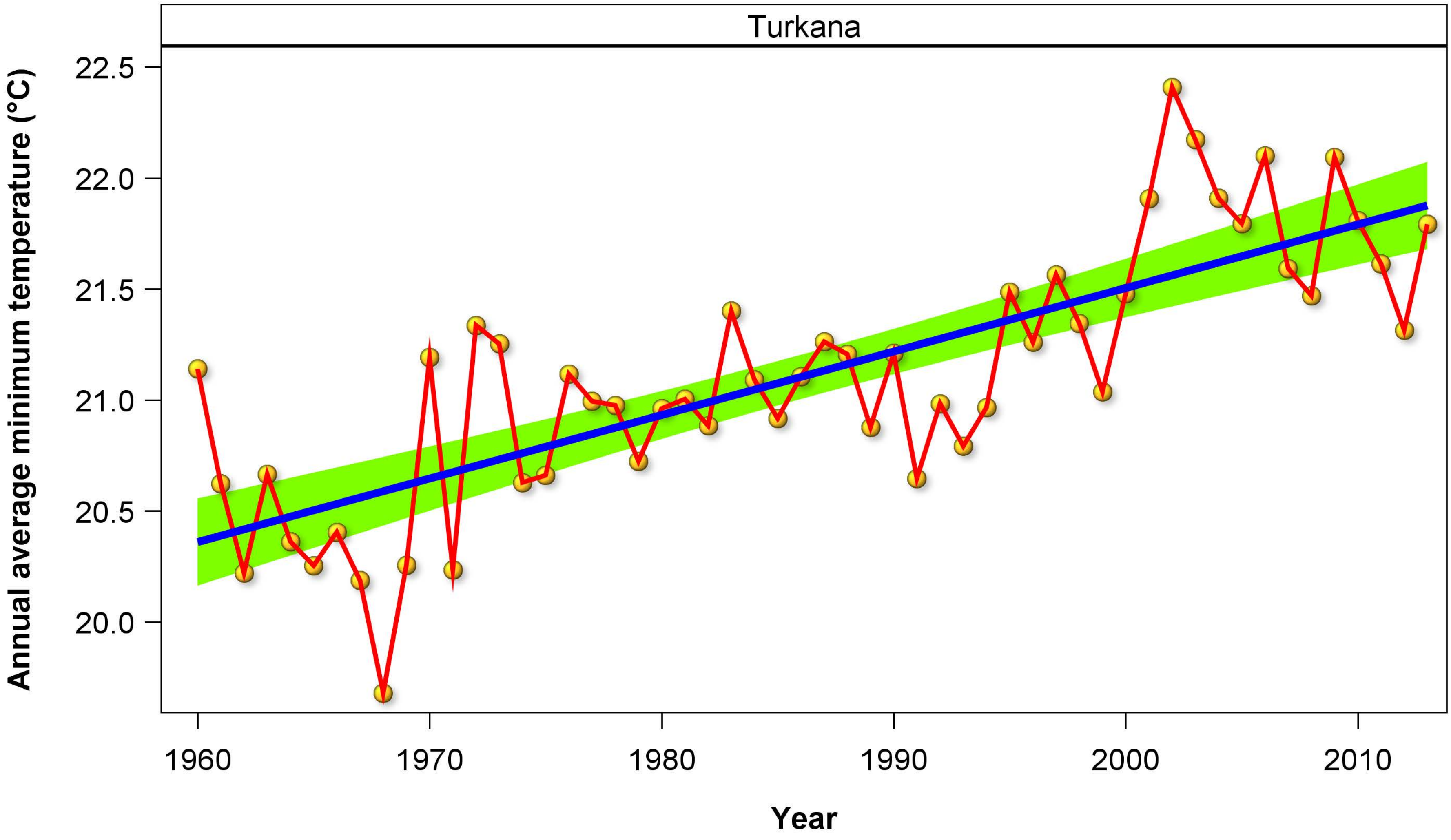
Turkana
Annual average minimum temperature (°C)
22.5
22.0
21.5
21.0
20.5
20.0
1960
1970
1980
1990
2000
2010
Year

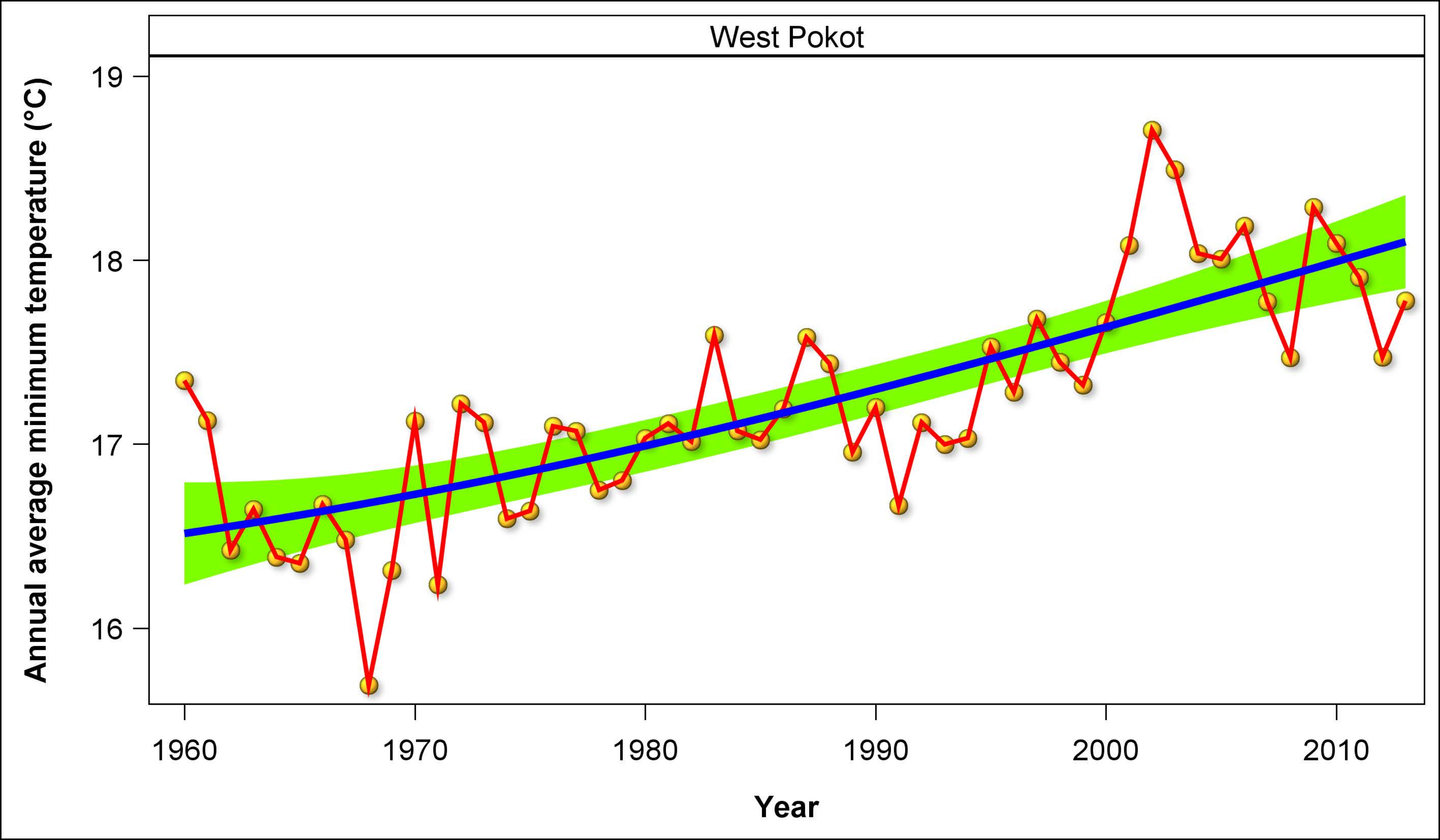

West Pokot
Annual average minimum temperature (°C)
19
18
17
16
1960
1970
1980
1990
2000
2010
Year

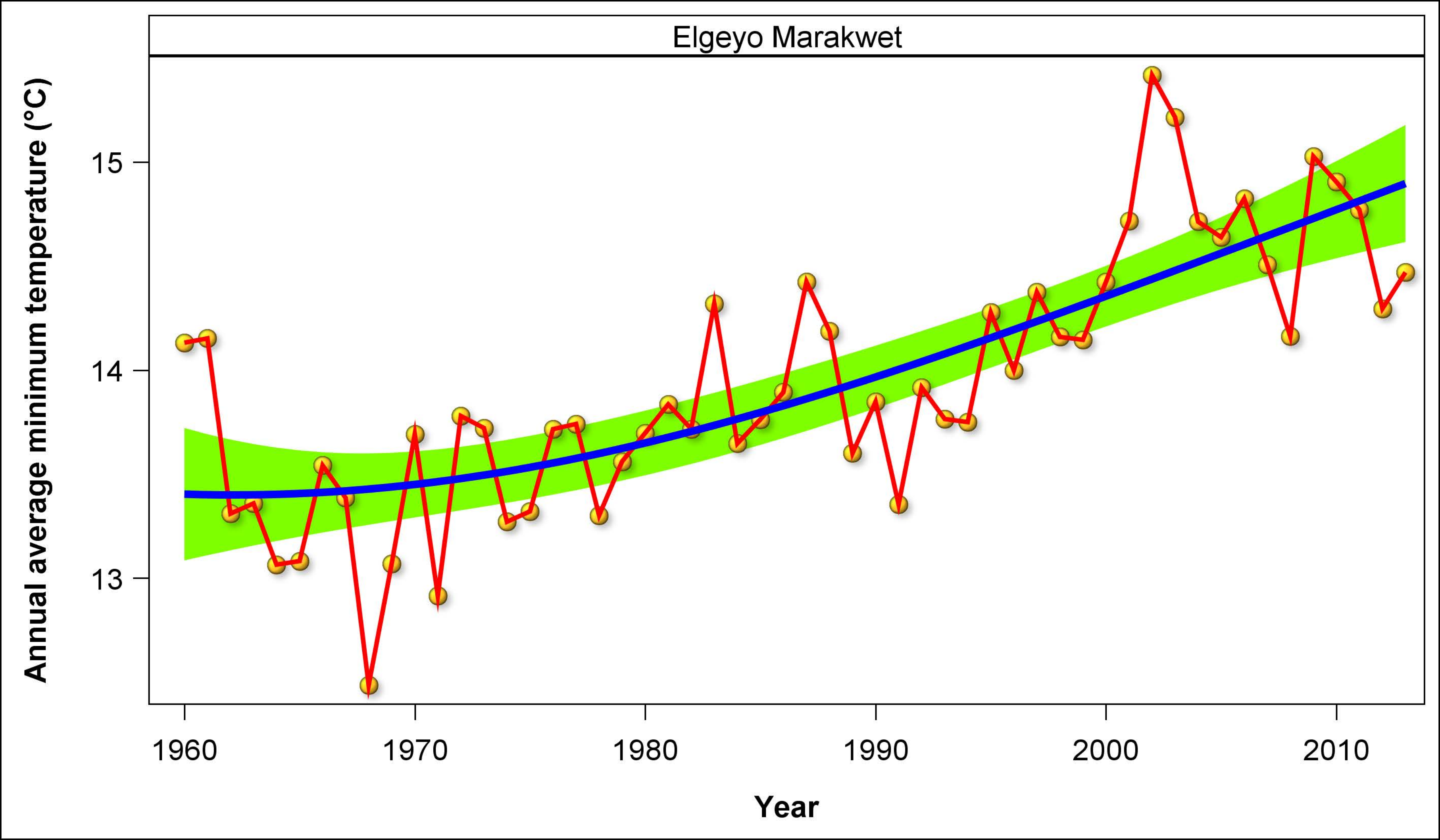

Elgeyo Marakwet
Annual average minimum temperature (°C)
15
14
13
1960
1970
1980
1990
2000
2010
Year
