# Supplementary material for: Extreme Wildlife Declines and Concurrent Increase in Livestock Numbers in Kenya: What Are the Causes?
Source: PLoS One. 2016 Sep 27;11(9):e0163249. doi: 10.1371/journal.pone.0163249 (PMC5039022; doi:10.1371/journal.pone.0163249)
Supplement: S26 Fig — The filled circles are the observations, the solid lines are the quadratic regression lines while the shaded bands are the 95% pointwise confidence bands. (PDF) [file pone.0163249.s036.pdf]

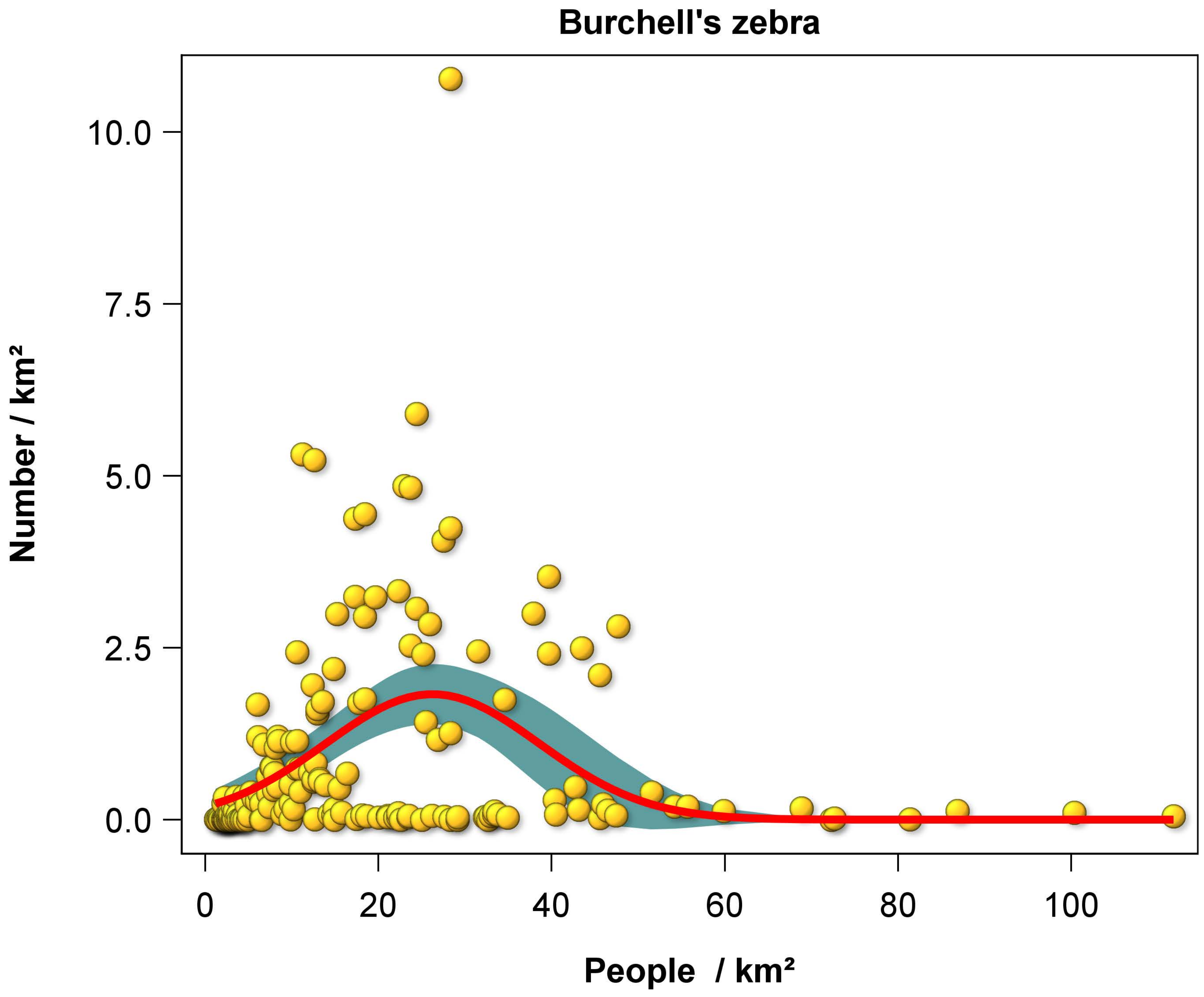
Burchell's zebra
Number / km²
10.0
7.5
5.0
2.5
0.0
0
20
40
60
80
100
People / km²

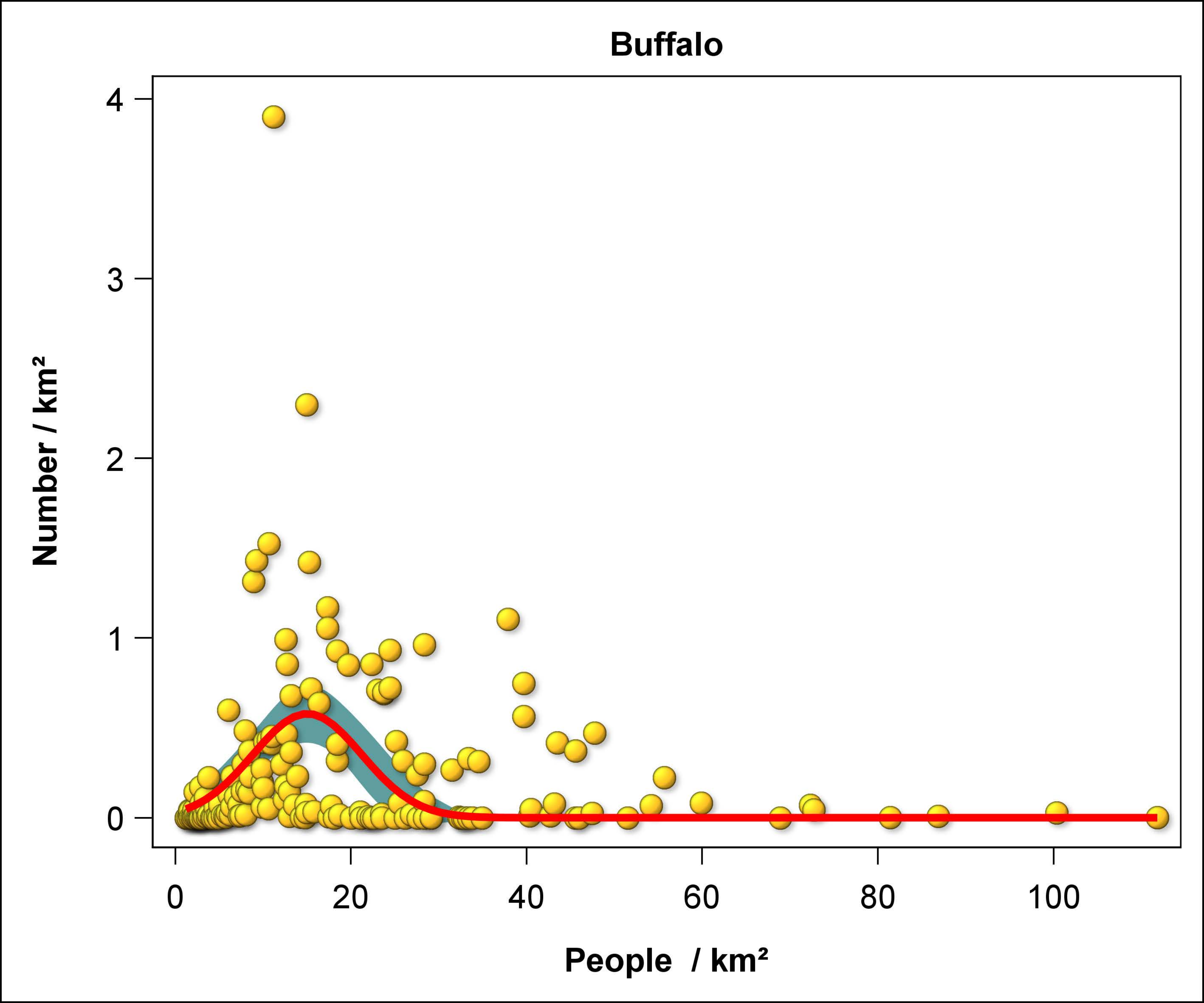

Buffalo
Number / km²
4
3
2
1
0
0
20
40
60
80
100
People / km²

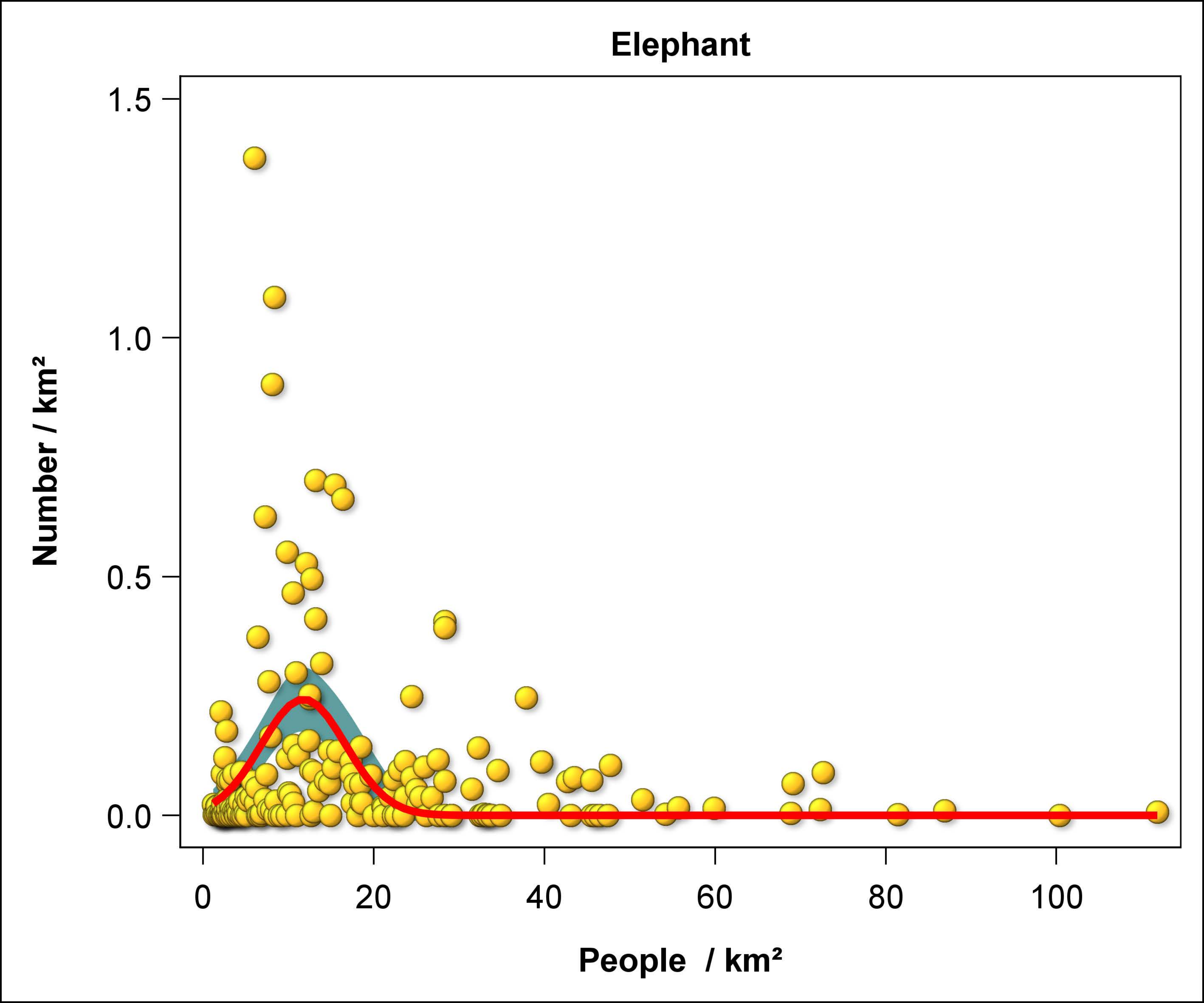

Elephant
1.5
1.0
0.5
0.0
Number / km²
0
20
40
60
80
100
People / km²

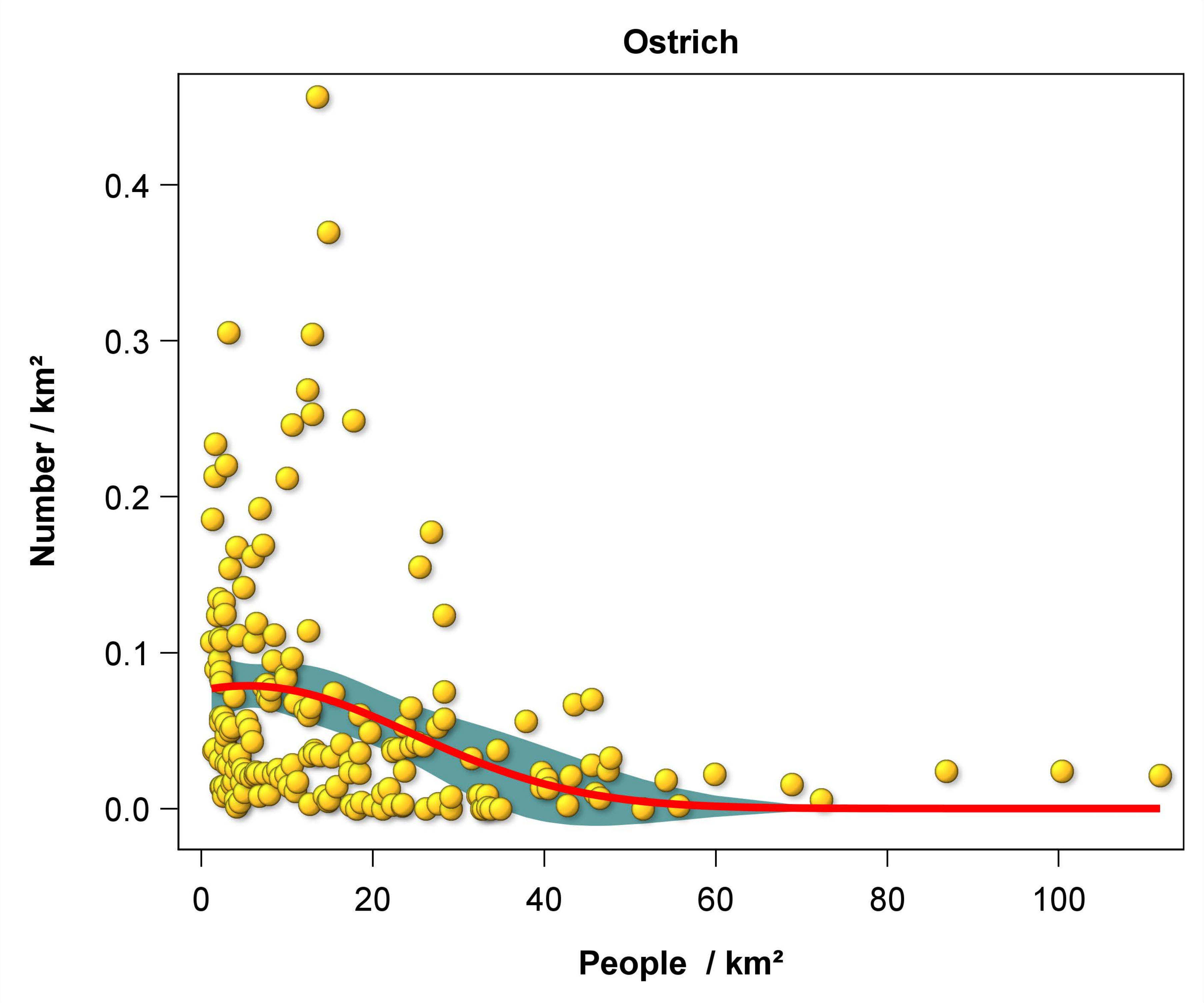

Ostrich
Number / km²
People / km²
0.0
0.1
0.2
0.3
0.4
0
20
40
60
80
100

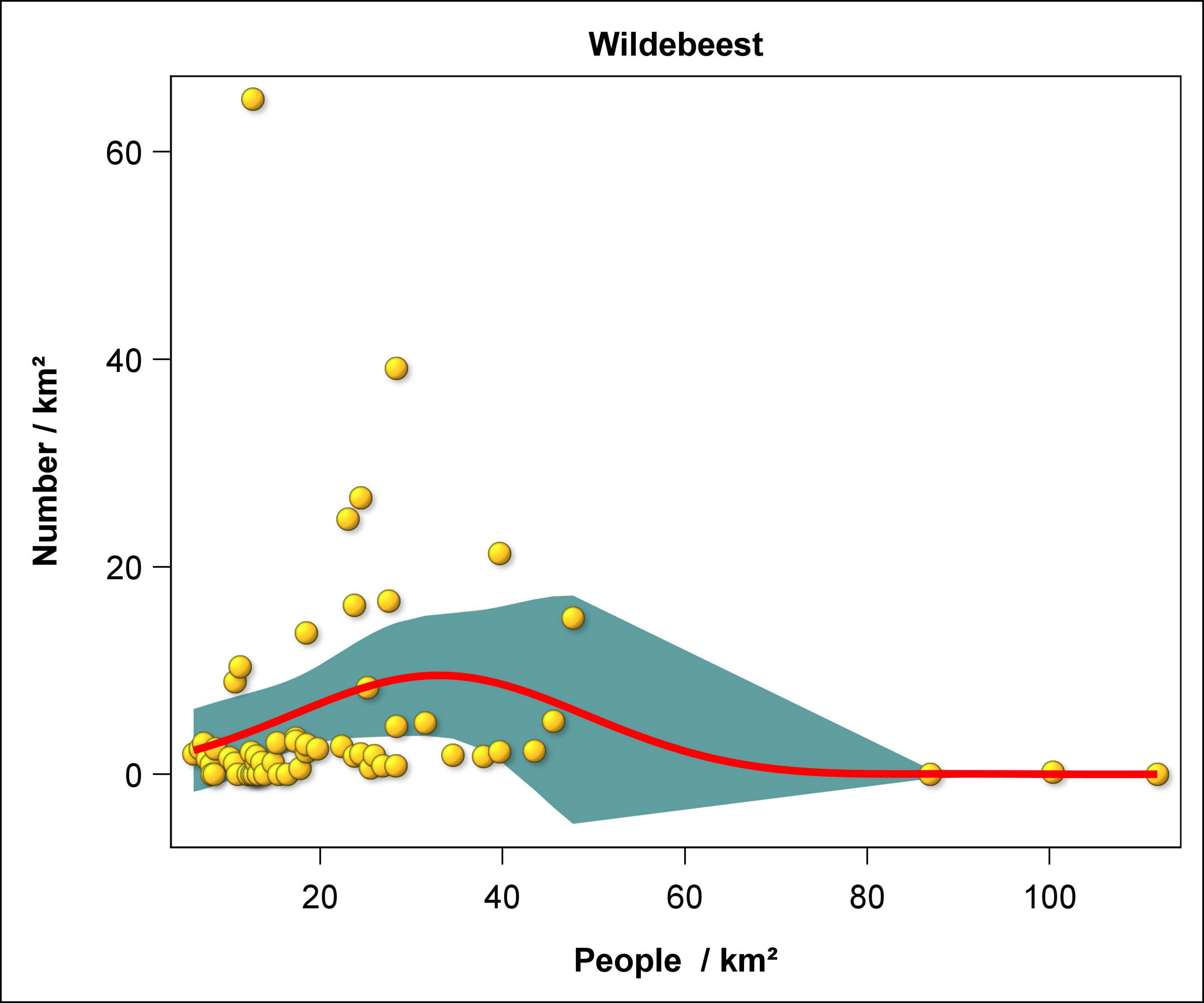

Wildebeest
60
40
20
0
Number / km²
20
40
60
80
100
People / km²

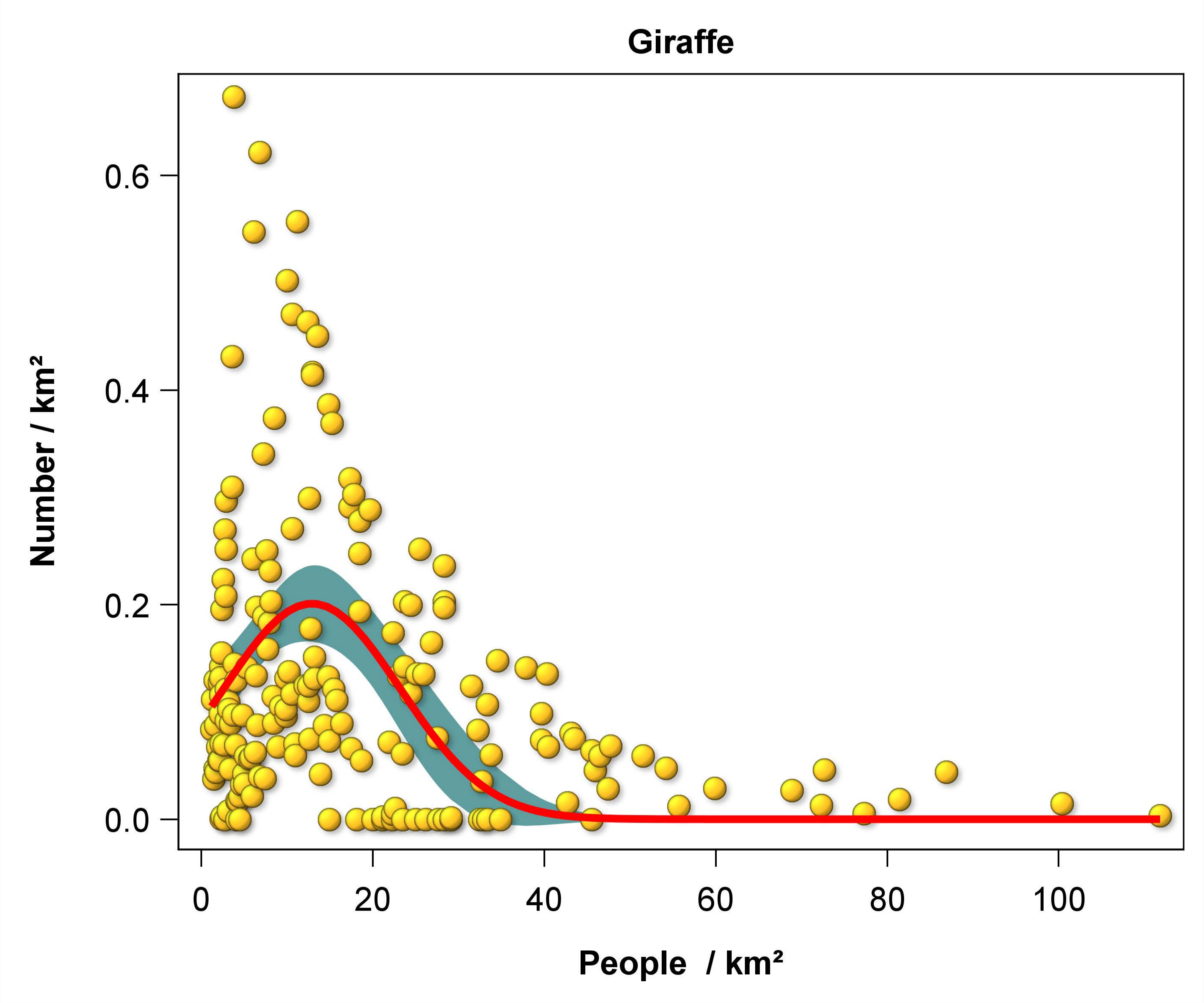

Giraffe
Number / km²
People / km²
0.0
0.2
0.4
0.6
0
20
40
60
80
100

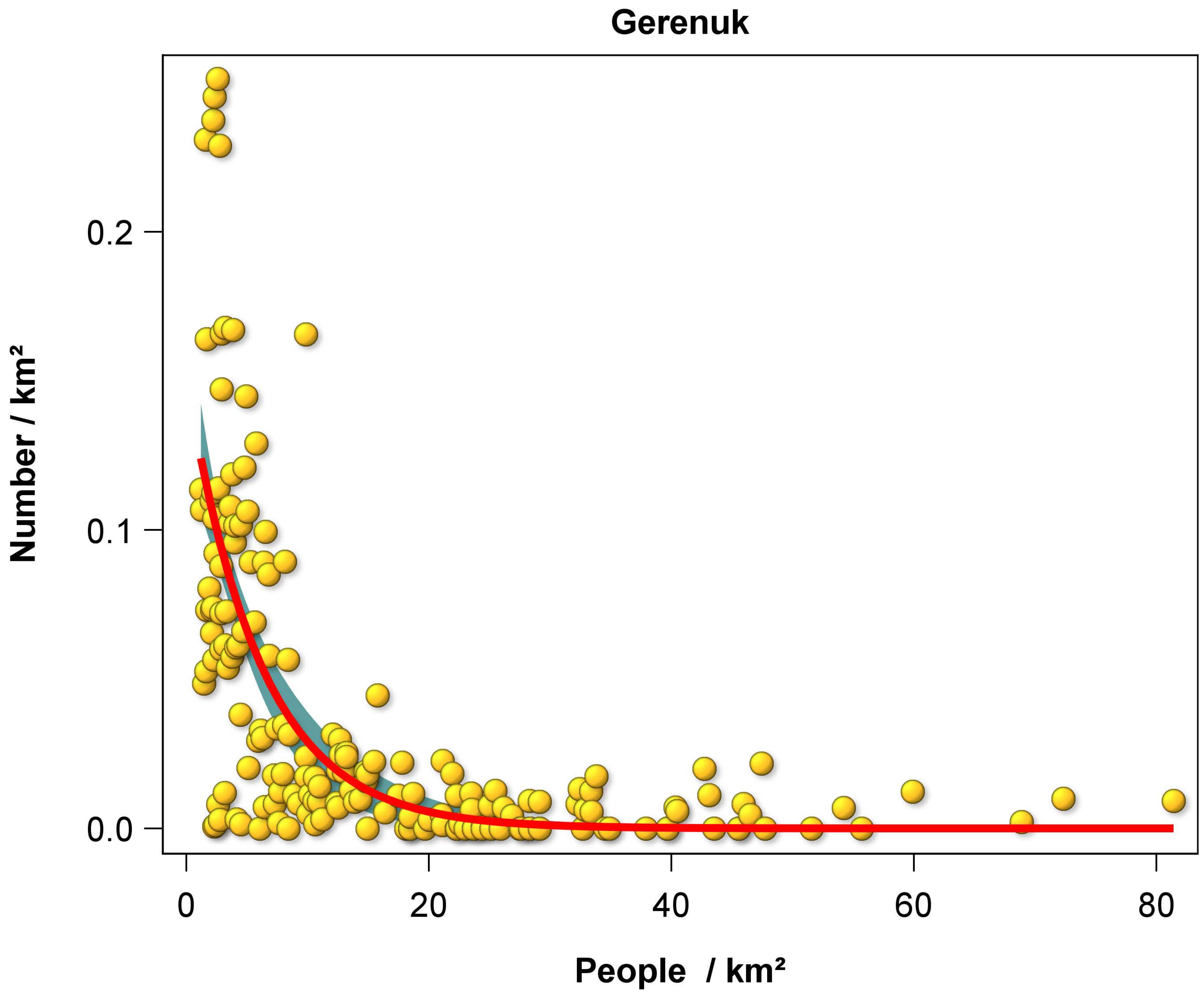

Gerenuk
Number / km²
People / km²
0.0
0.1
0.2
0
20
40
60
80

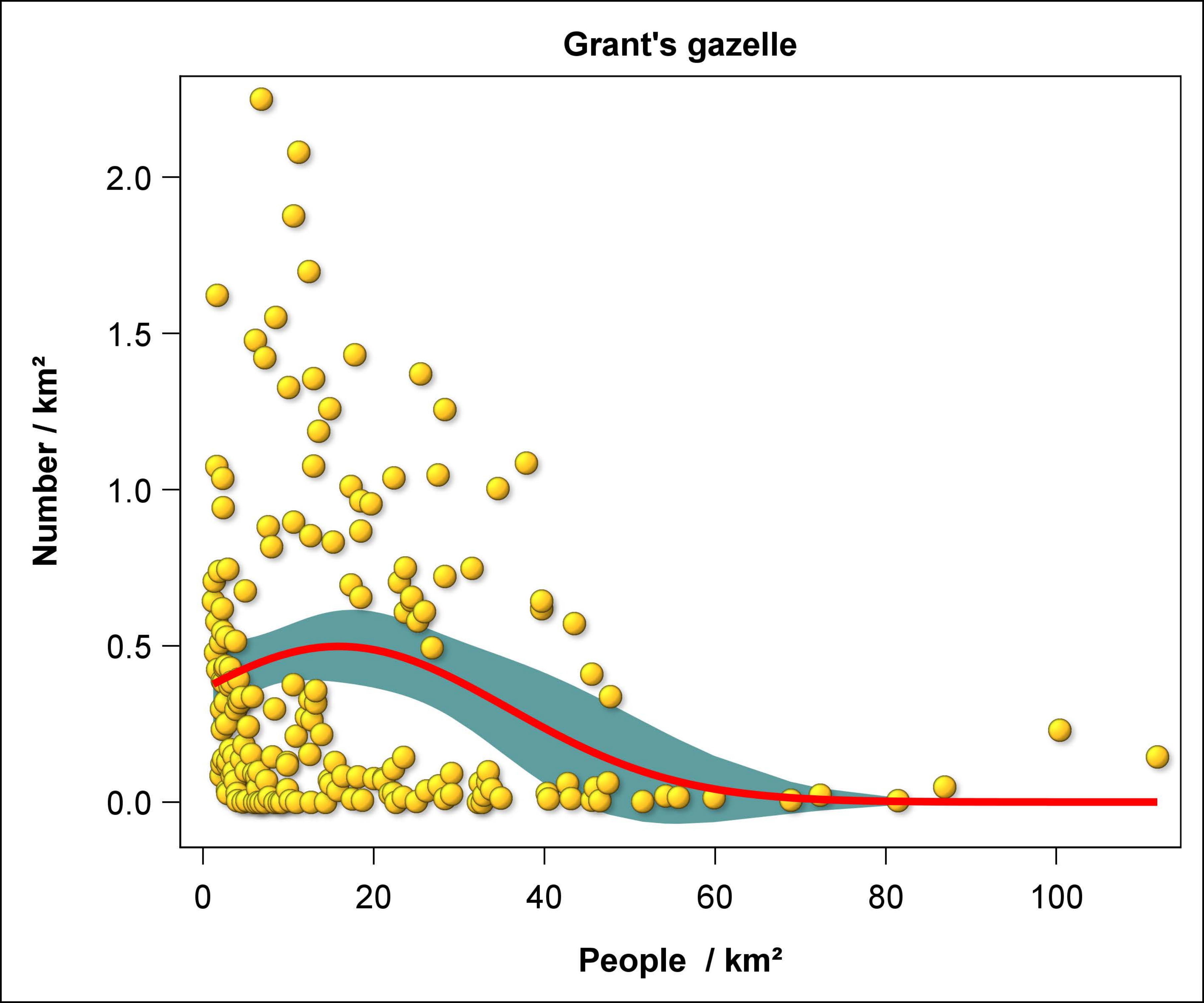
Grant's gazelle
Number / km²
People / km²
0.0
0.5
1.0
1.5
2.0
0
20
40
60
80
100

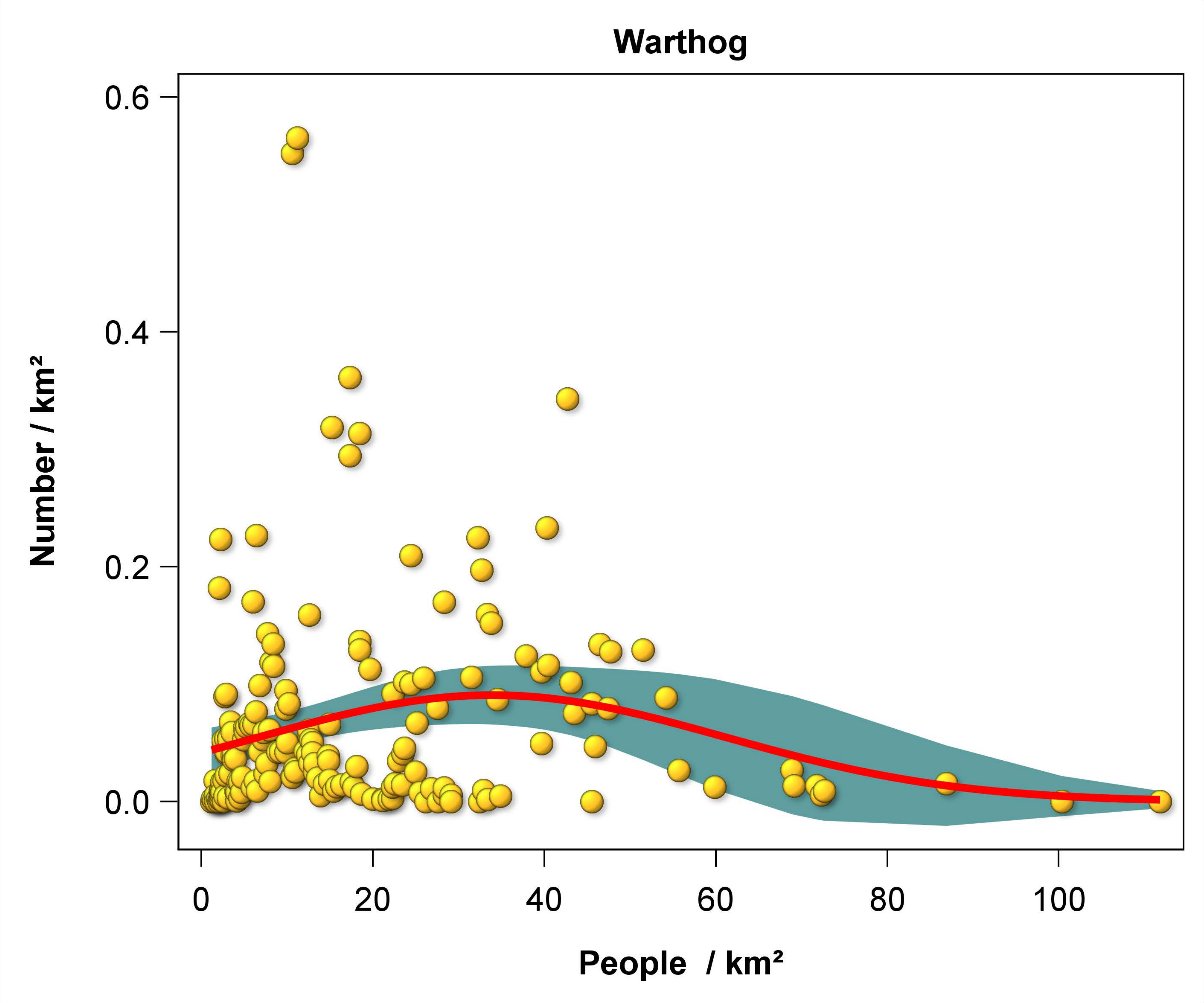
Warthog
0.6
0.4
0.2
0.0
Number / km²
0
20
40
60
80
100
People / km²

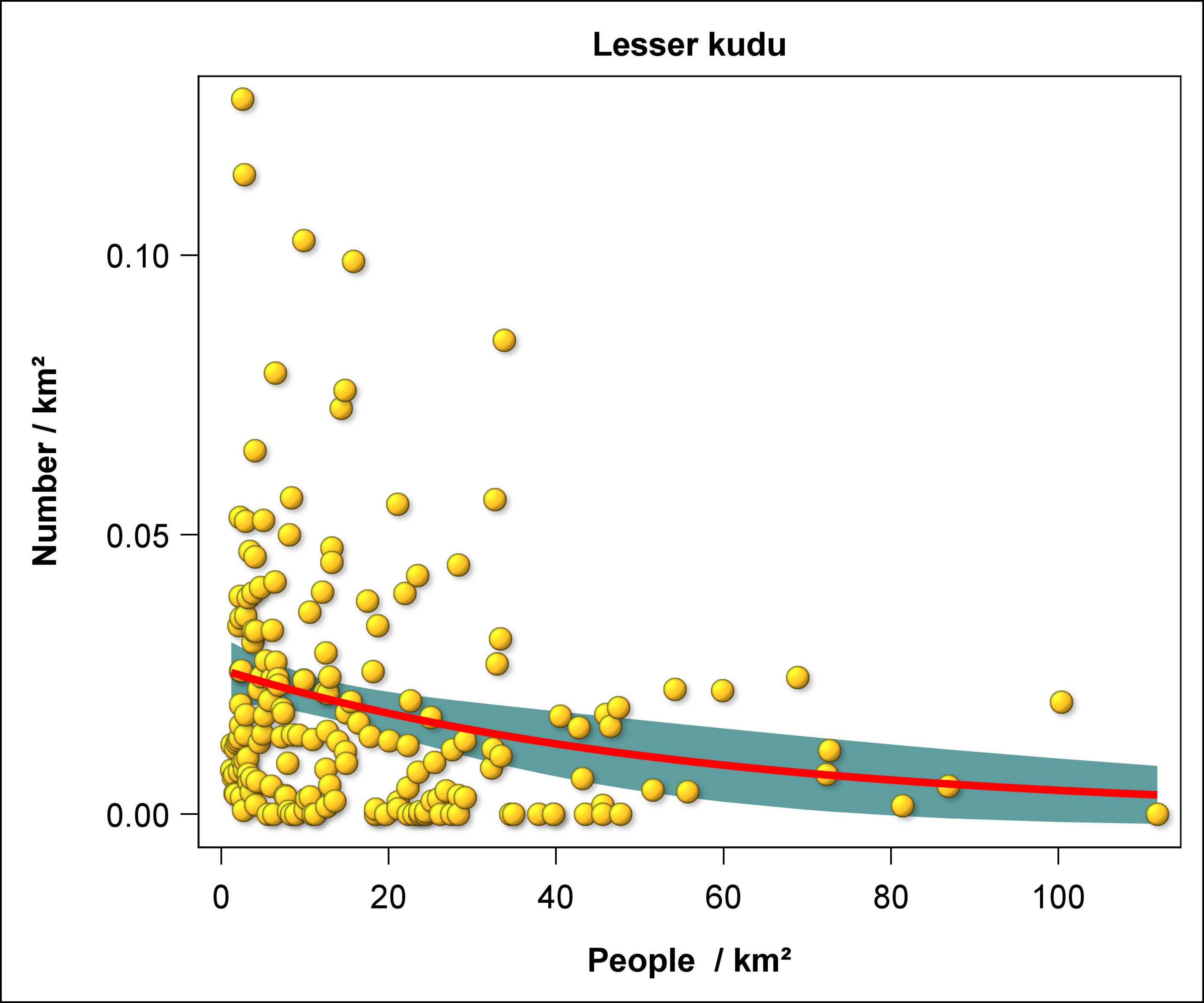

Lesser kudu
Number / km²
0.10
0.05
0.00
0
20
40
60
80
100
People / km²

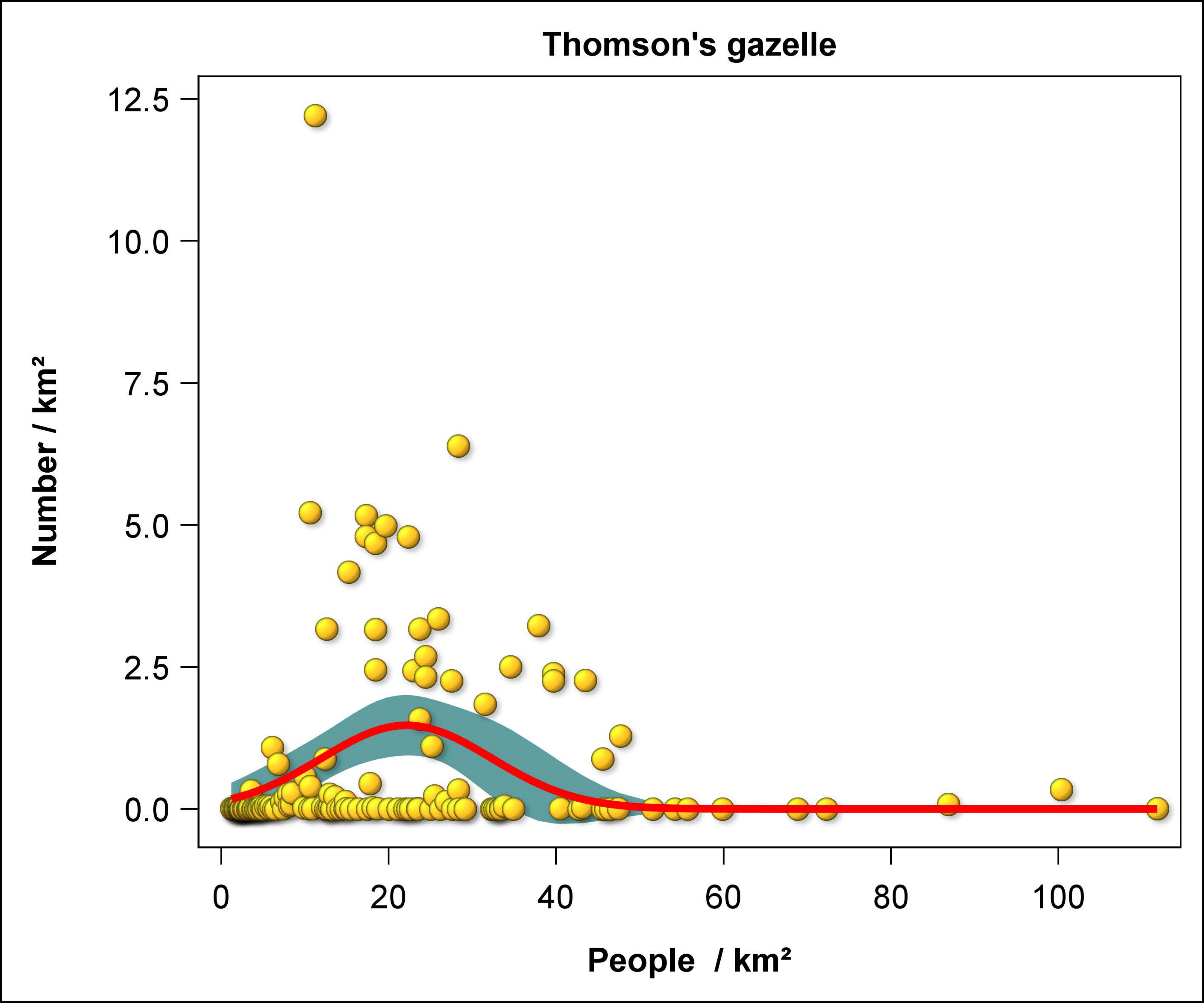
Thomson's gazelle
12.5
10.0
7.5
5.0
2.5
0.0
Number / km²
0
20
40
60
80
100
People / km²

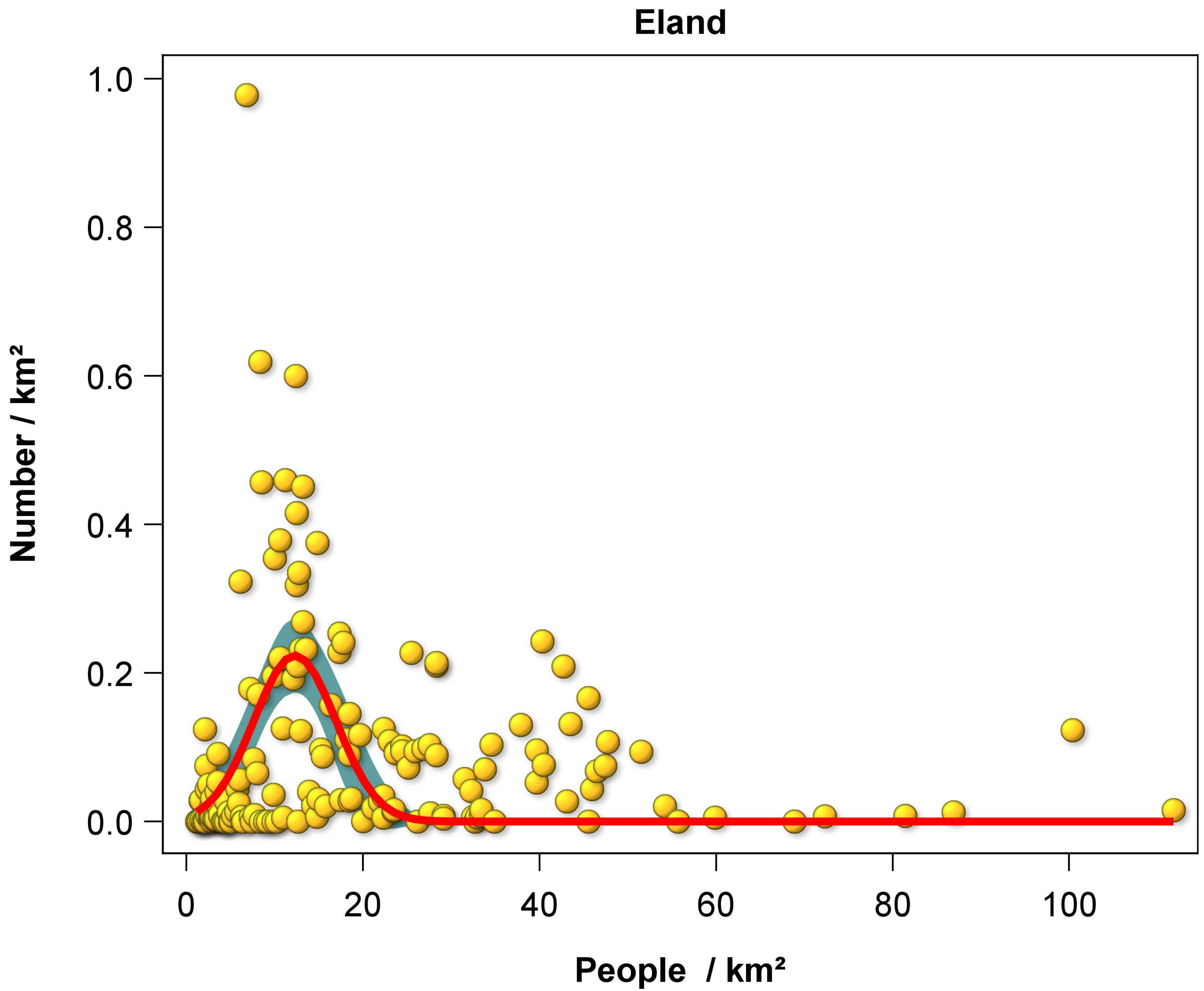
Eland
1.0
0.8
0.6
0.4
0.2
0.0
Number / km²
0
20
40
60
80
100
People / km²

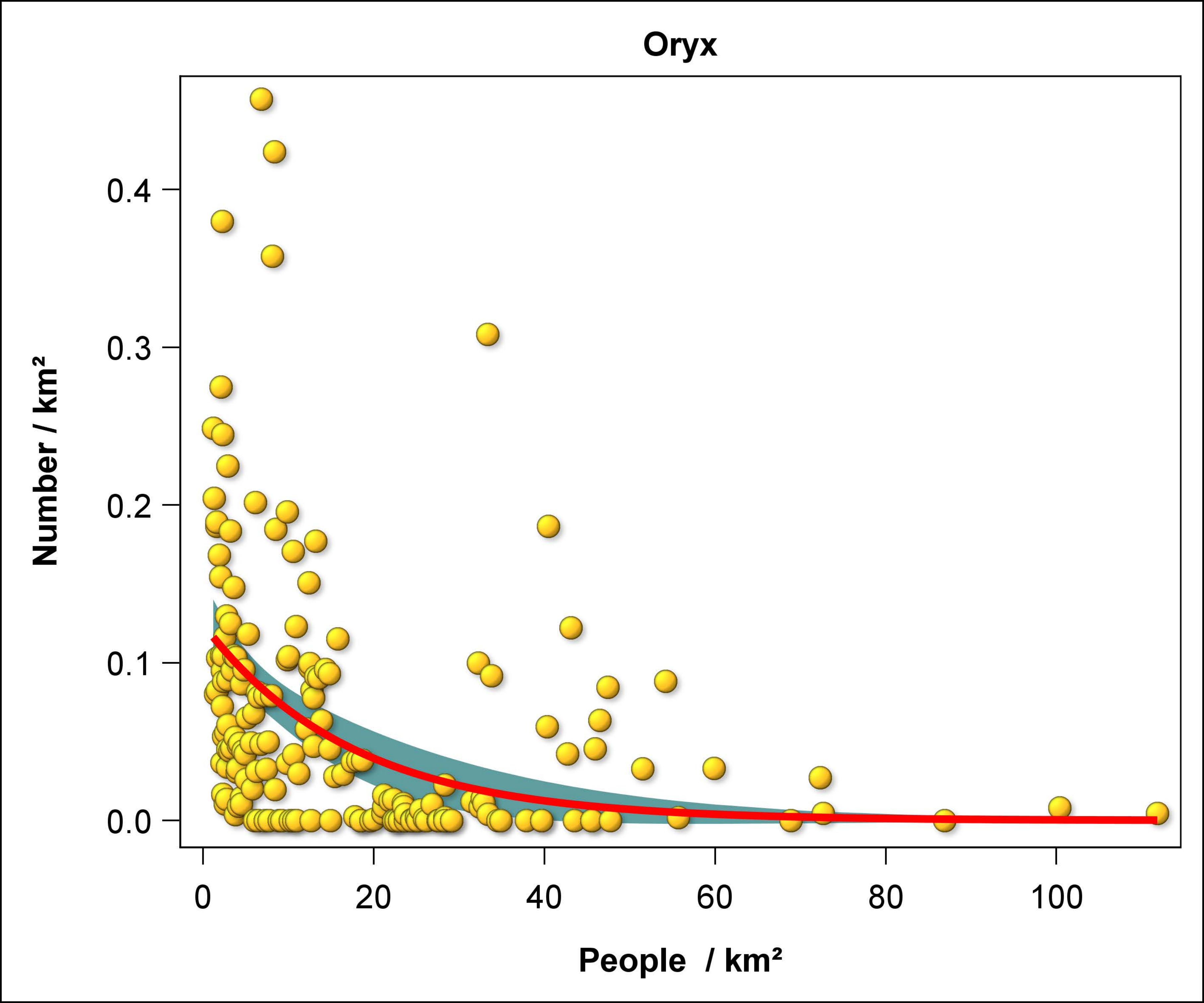

Oryx
Number / km²
0.0
0.1
0.2
0.3
0.4
0
20
40
60
80
100
People / km²

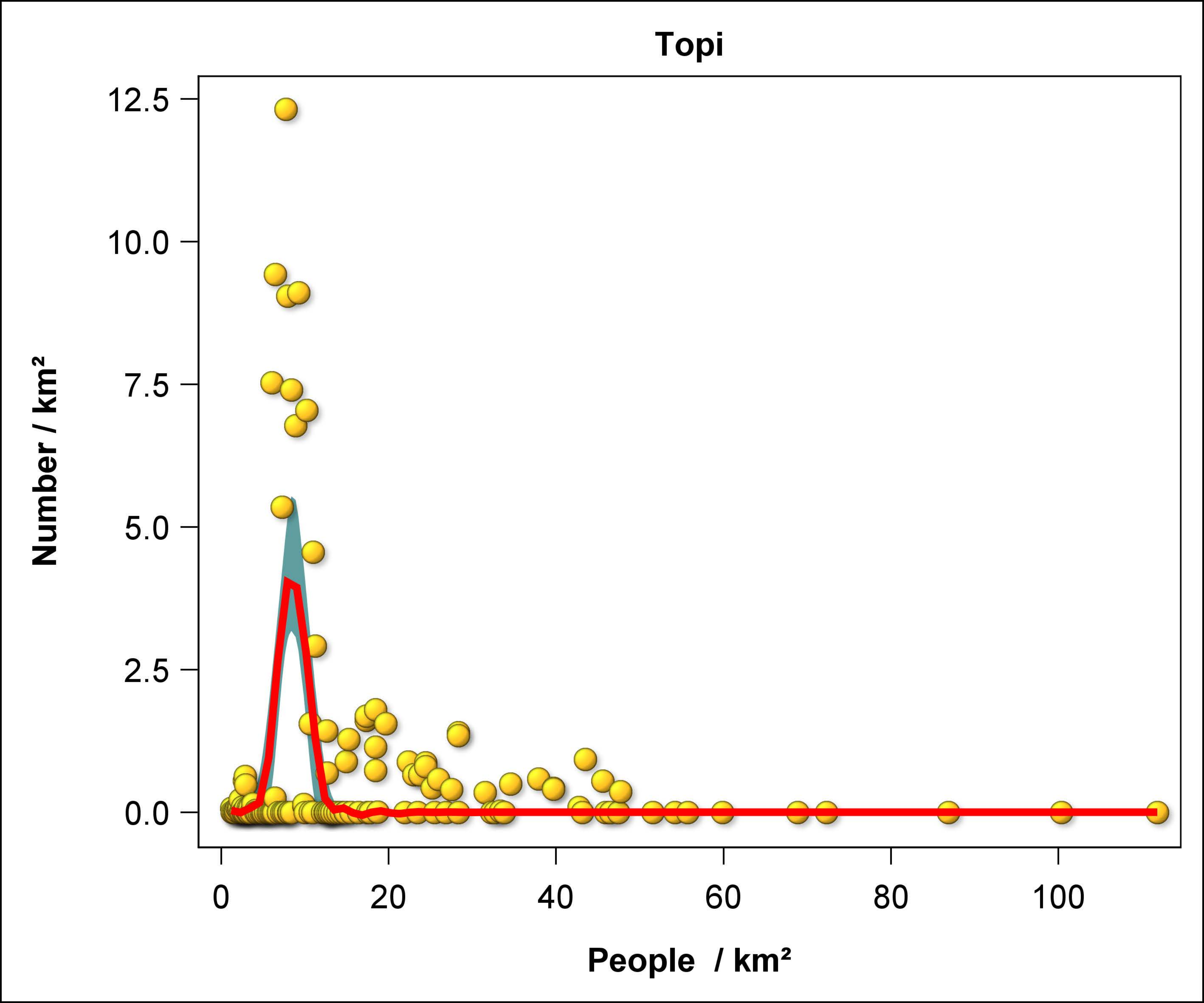

Topi
12.5
10.0
7.5
5.0
2.5
0.0
Number / km²
0
20
40
60
80
100
People / km²

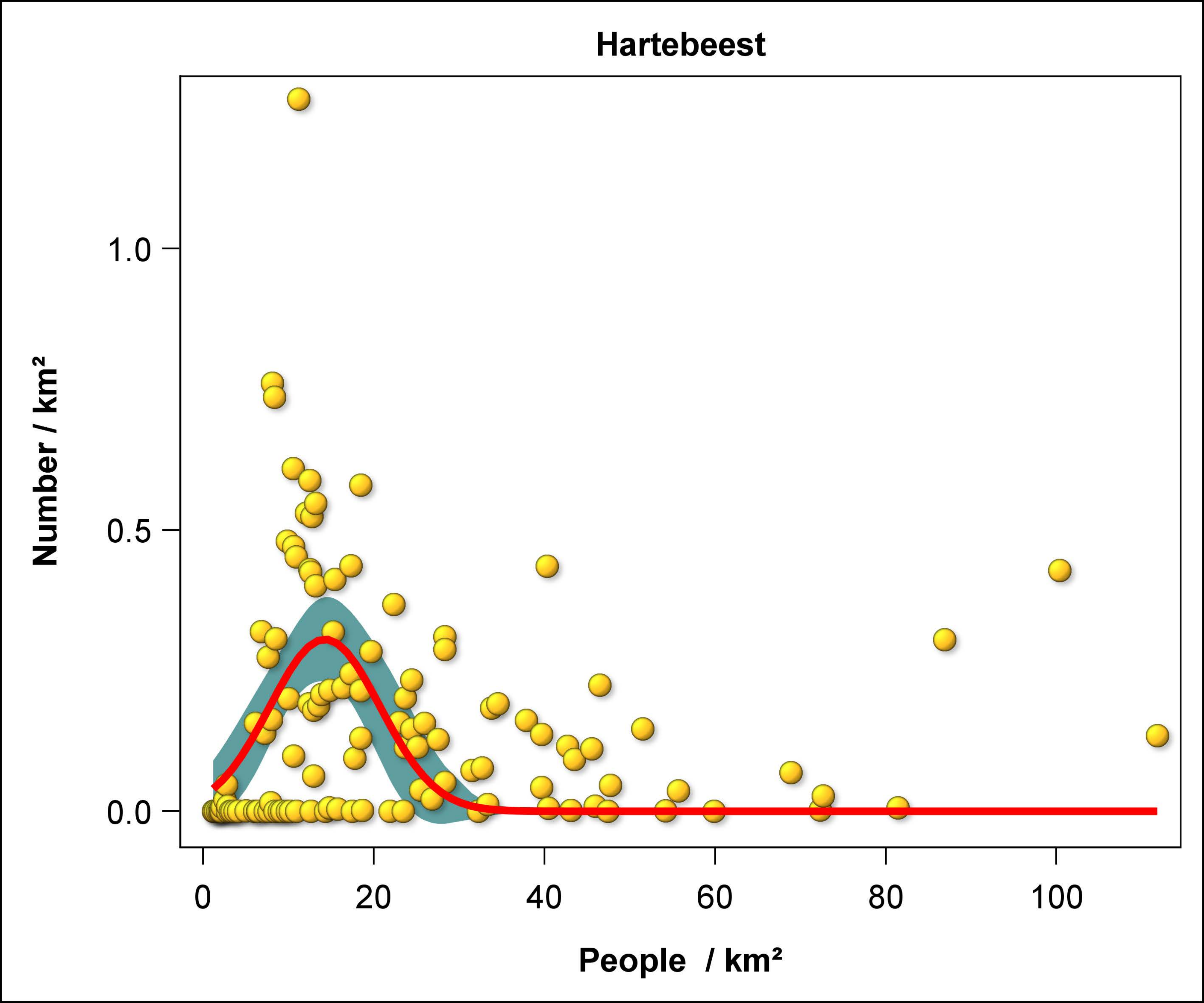
Hartebeest
1.0
0.5
0.0
Number / km²
0
20
40
60
80
100
People / km²

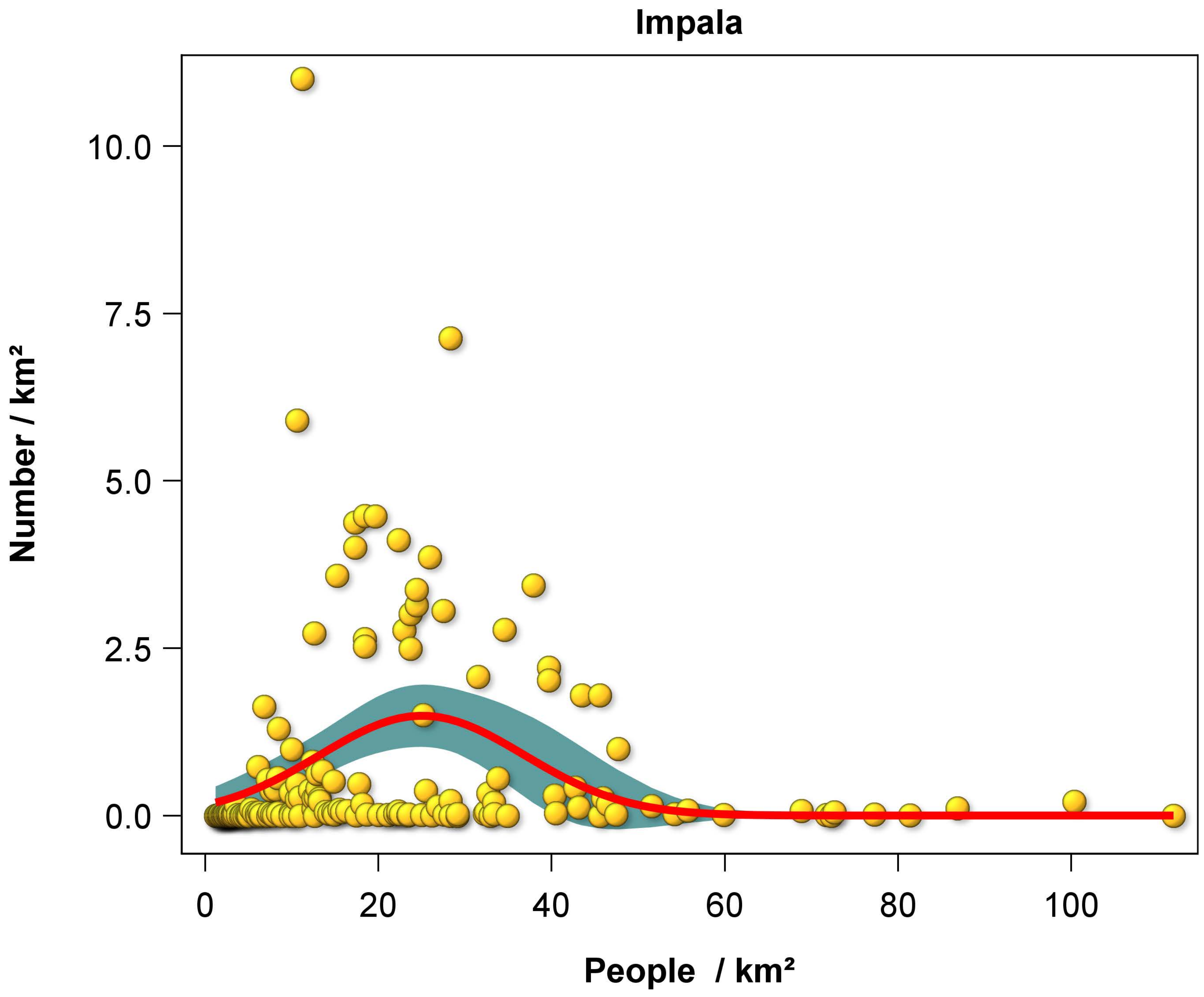

Impala
10.0
7.5
5.0
2.5
0.0
Number / km²
0
20
40
60
80
100
People / km²

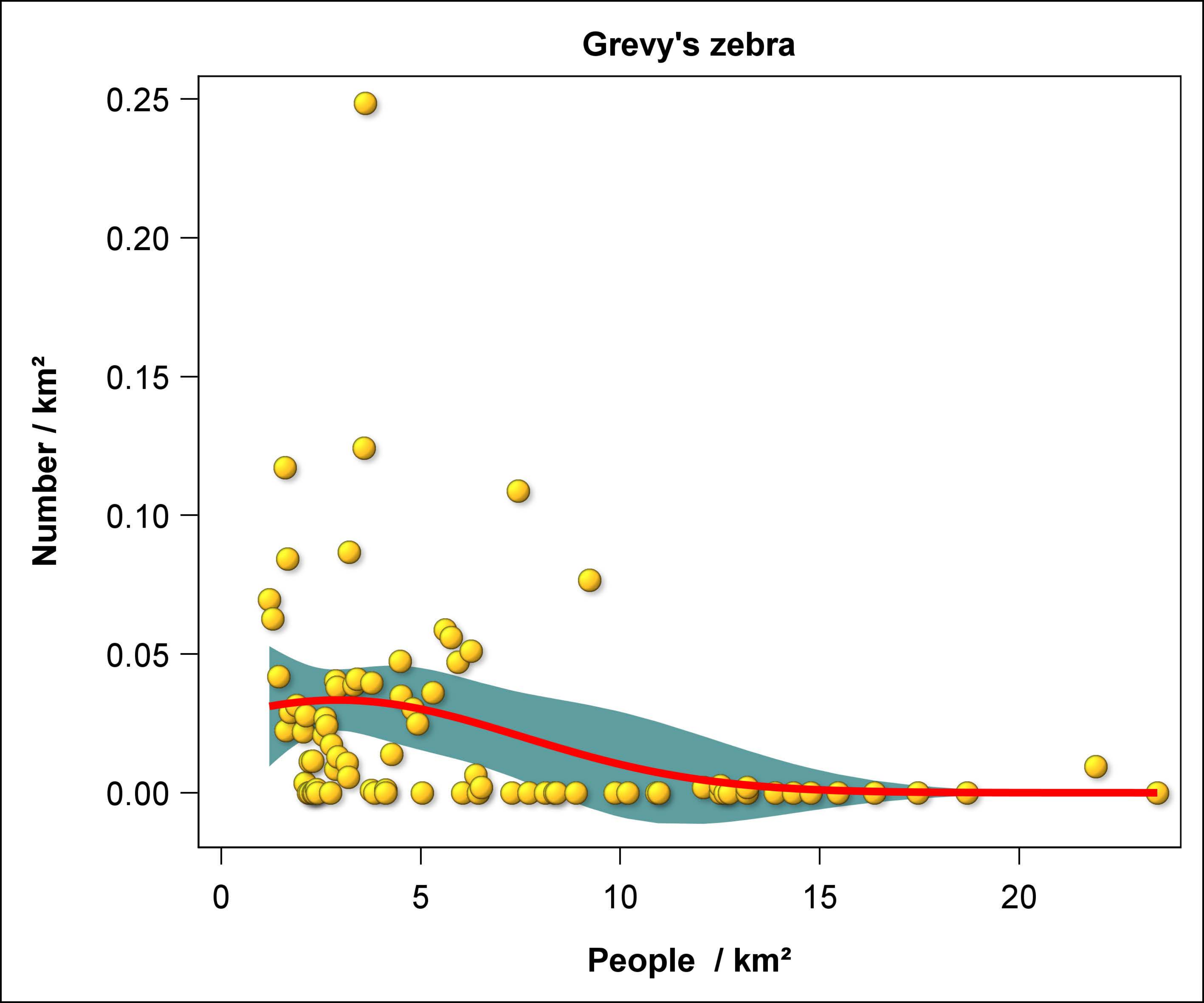

Grevy's zebra
0.25
0.20
0.15
0.10
0.05
0.00
Number / km²
0
5
10
15
20
People / km²

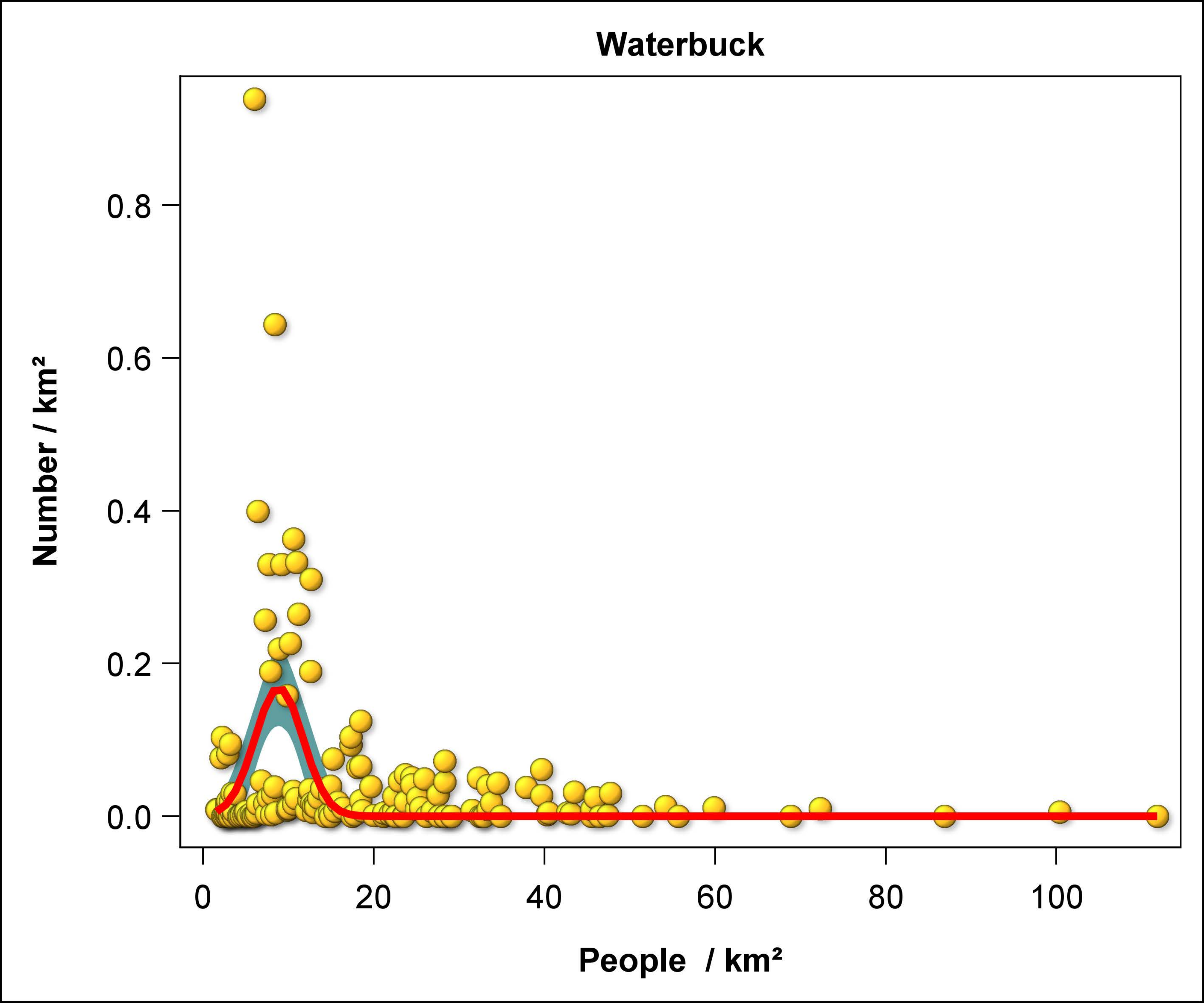

Waterbuck
Number / km²
0.0
0.2
0.4
0.6
0.8
0
20
40
60
80
100
People / km²
