# Supplementary material for: Extreme Wildlife Declines and Concurrent Increase in Livestock Numbers in Kenya: What Are the Causes?
Source: PLoS One. 2016 Sep 27;11(9):e0163249. doi: 10.1371/journal.pone.0163249 (PMC5039022; doi:10.1371/journal.pone.0163249)
Supplement: S27 Fig — The filled circles are the observations, the solid lines are the quadratic regression lines while the shaded bands are the 95% pointwise confidence bands. (PDF) [file pone.0163249.s037.pdf]

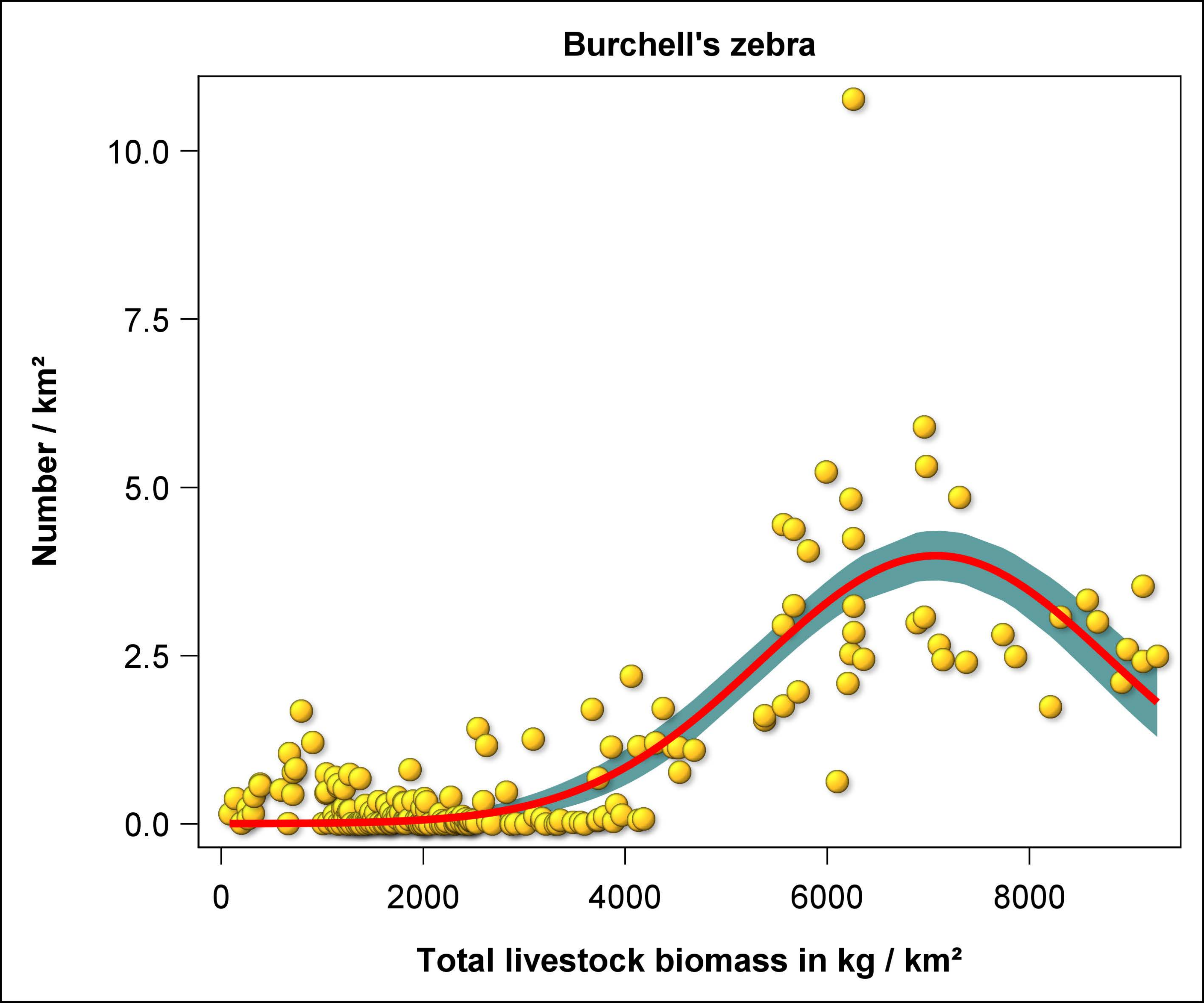

Burchell's zebra
10.0
7.5
5.0
2.5
0.0
Number / km²
0
2000
4000
6000
8000
Total livestock biomass in kg / km²

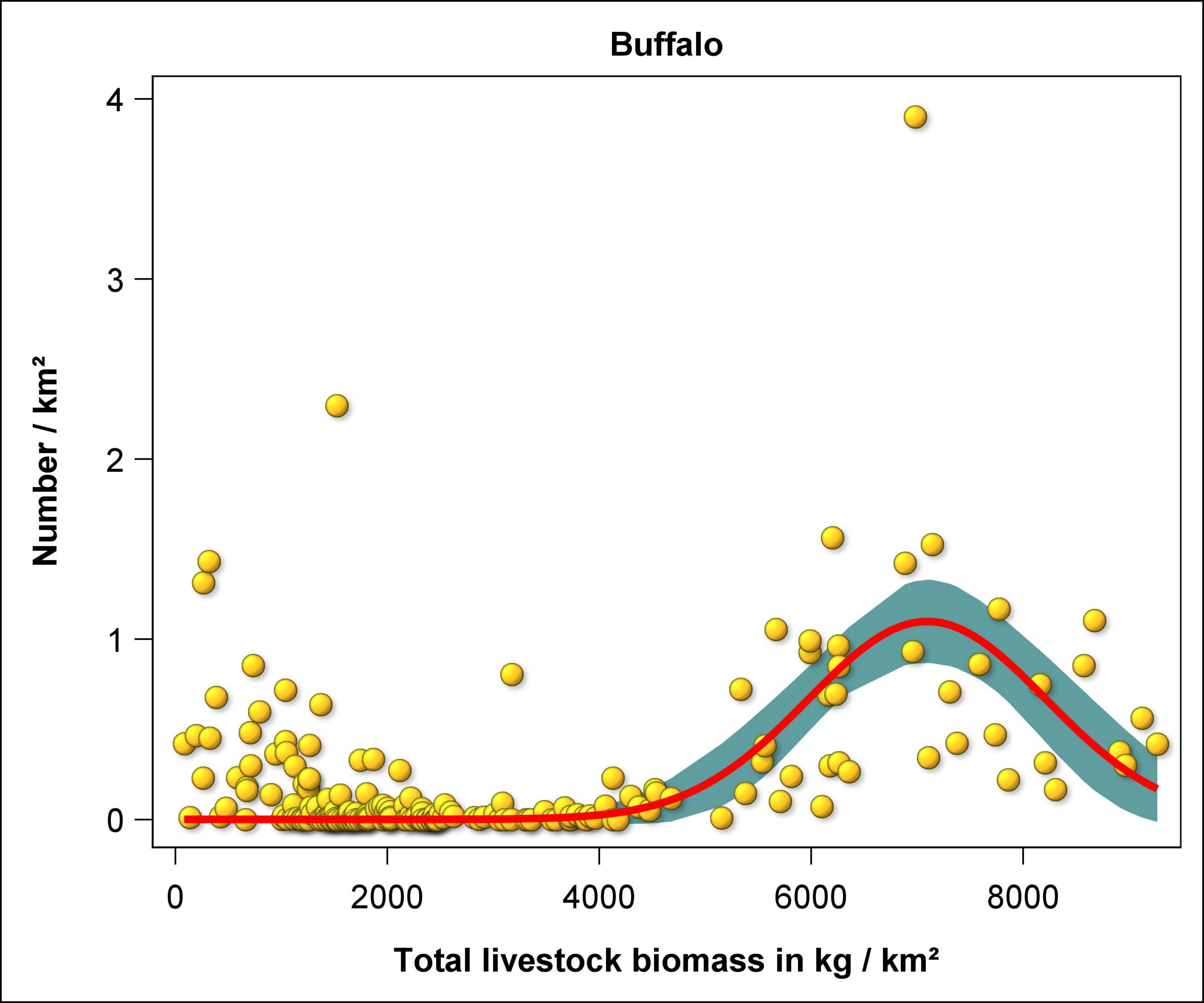
Buffalo
Number / km²
0
1
2
3
4
0
2000
4000
6000
8000
Total livestock biomass in kg / km²

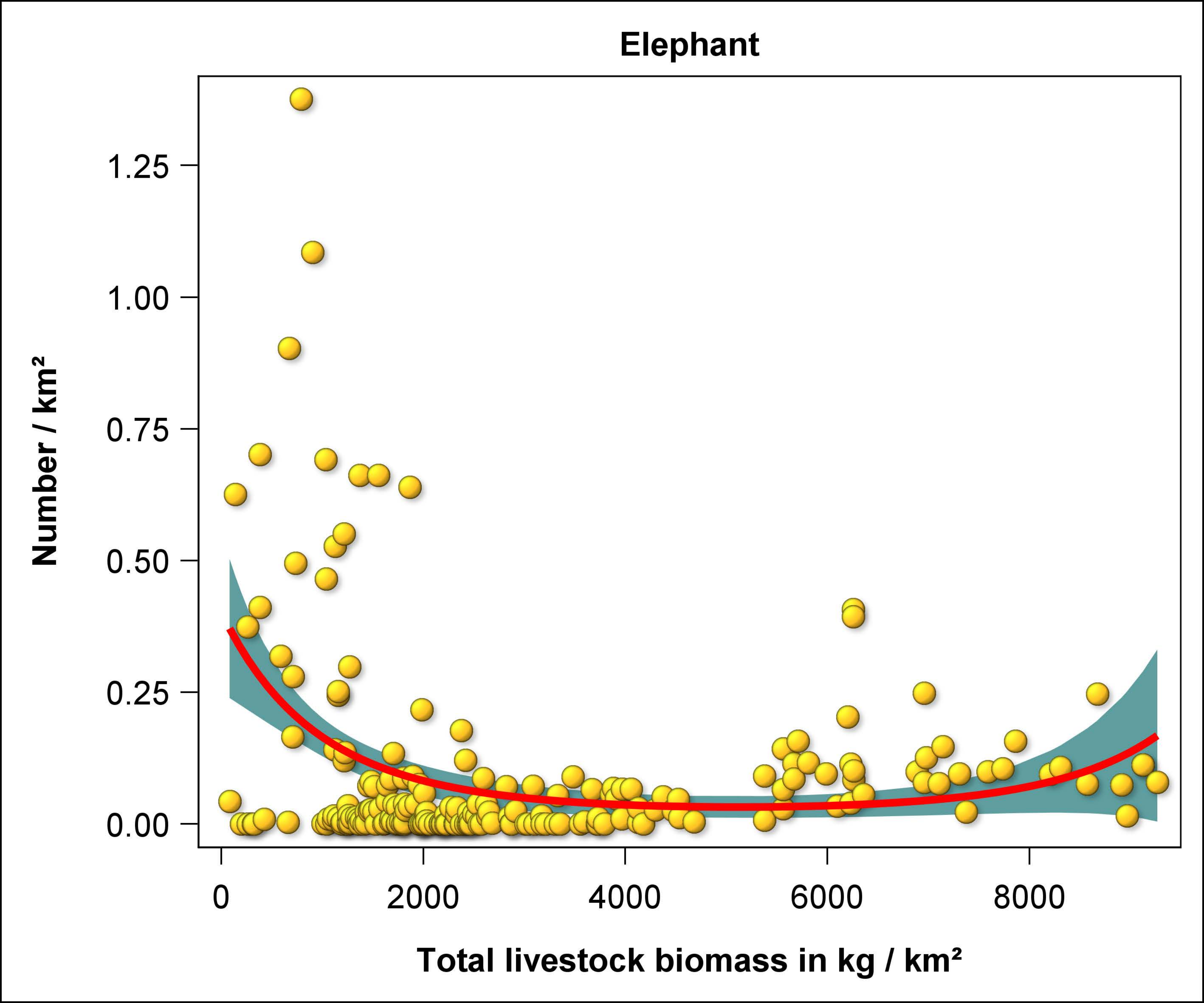

Elephant
Number / km²
1.25
1.00
0.75
0.50
0.25
0.00
0
2000
4000
6000
8000
Total livestock biomass in kg / km²

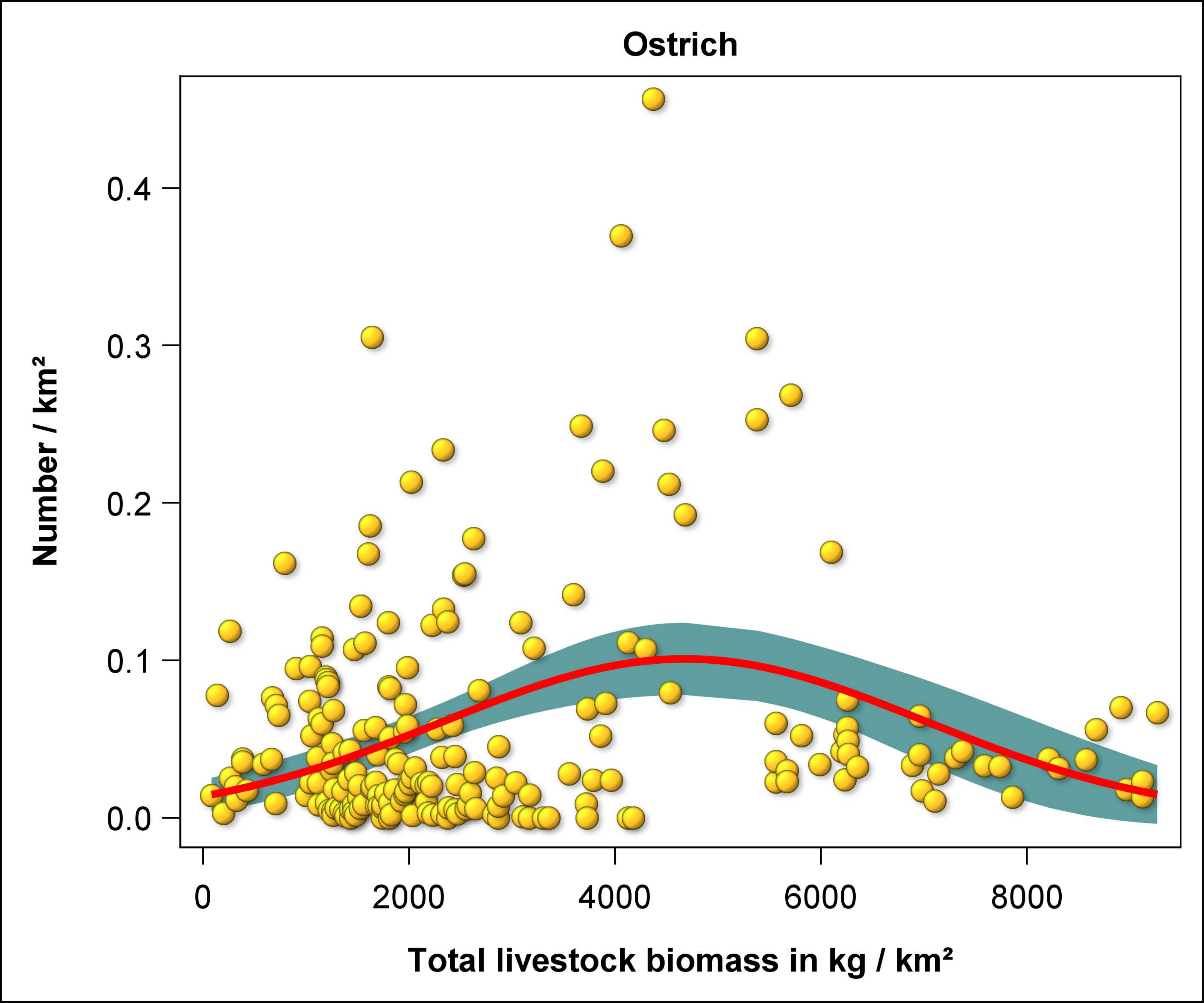
Ostrich
Number / km²
0.0
0.1
0.2
0.3
0.4
0
2000
4000
6000
8000
Total livestock biomass in kg / km²

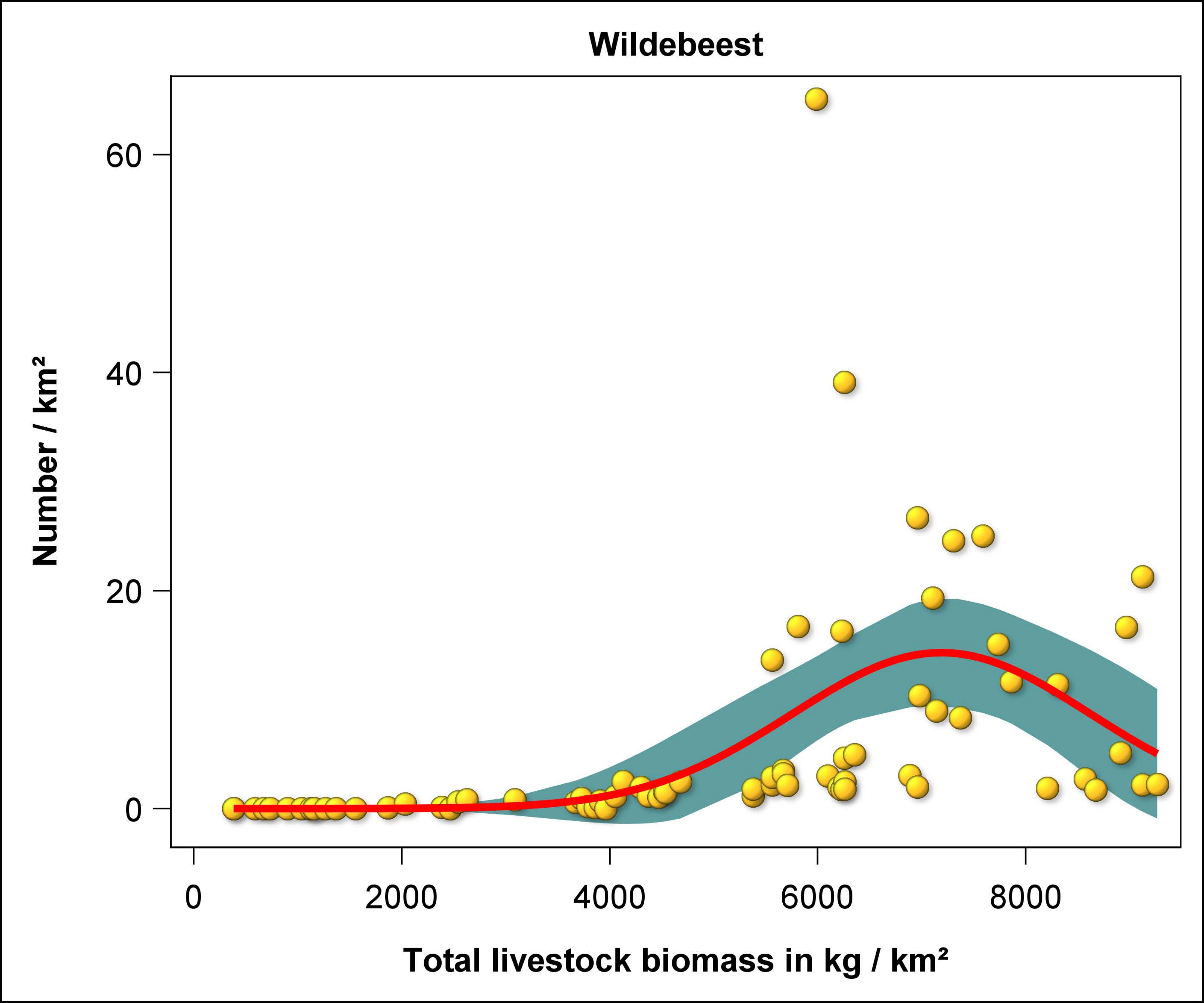

Wildebeest
Number / km²
60
40
20
0
0
2000
4000
6000
8000
Total livestock biomass in kg / km²

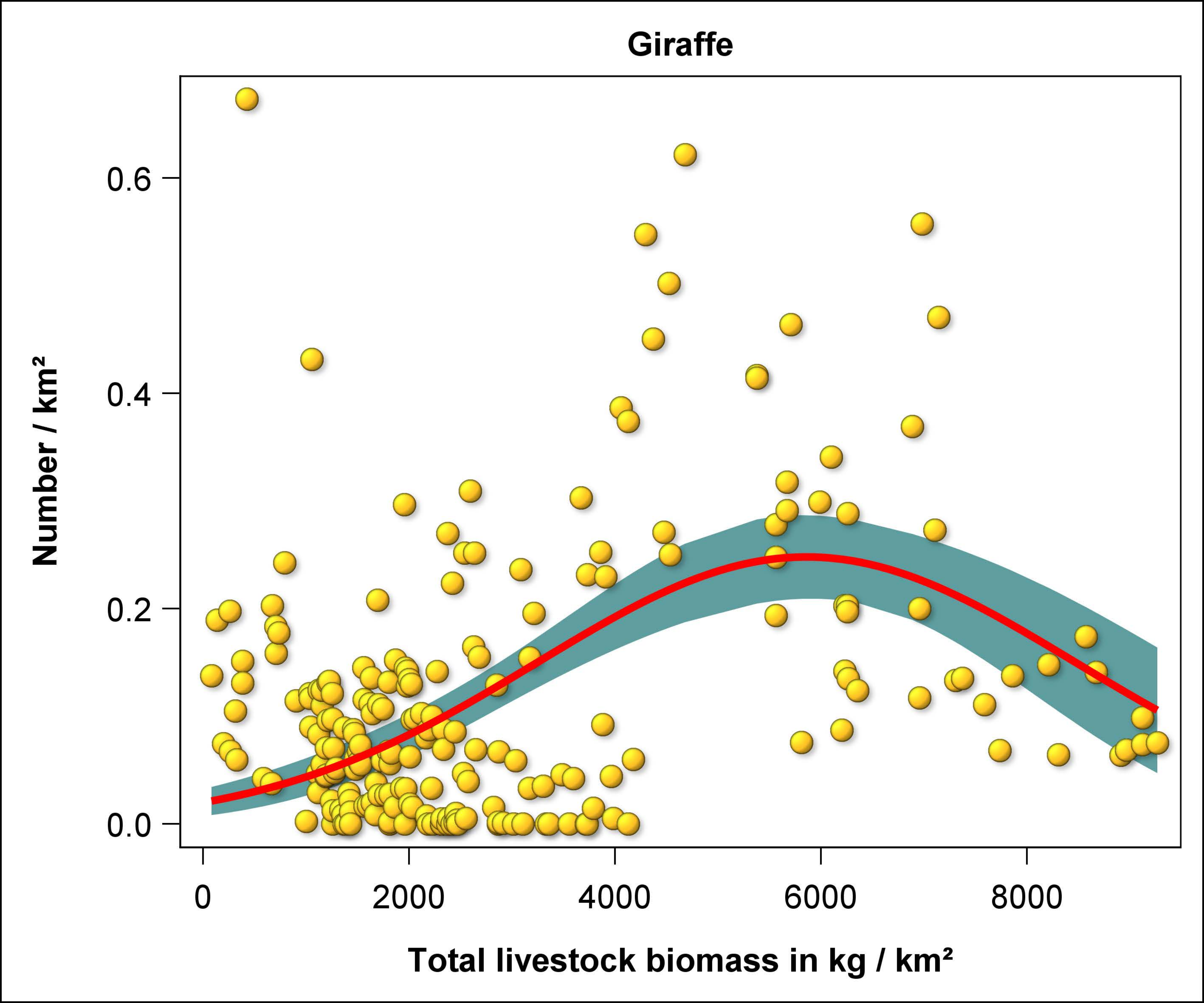
Giraffe
Number / km²
0.0
0.2
0.4
0.6
0
2000
4000
6000
8000
Total livestock biomass in kg / km²

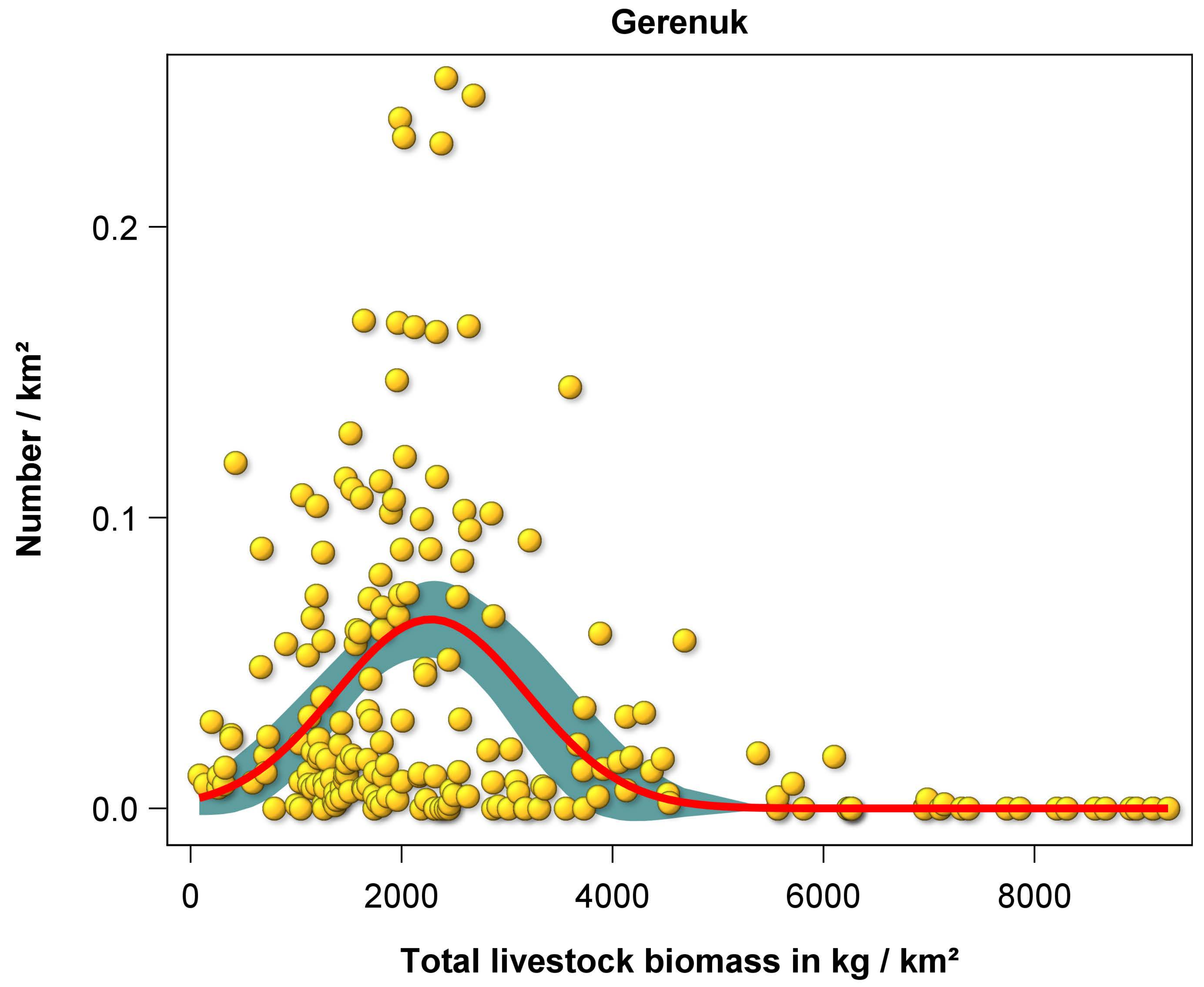
Gerenuk
Number / km²
0.0
0.1
0.2
0
2000
4000
6000
8000
Total livestock biomass in kg / km²

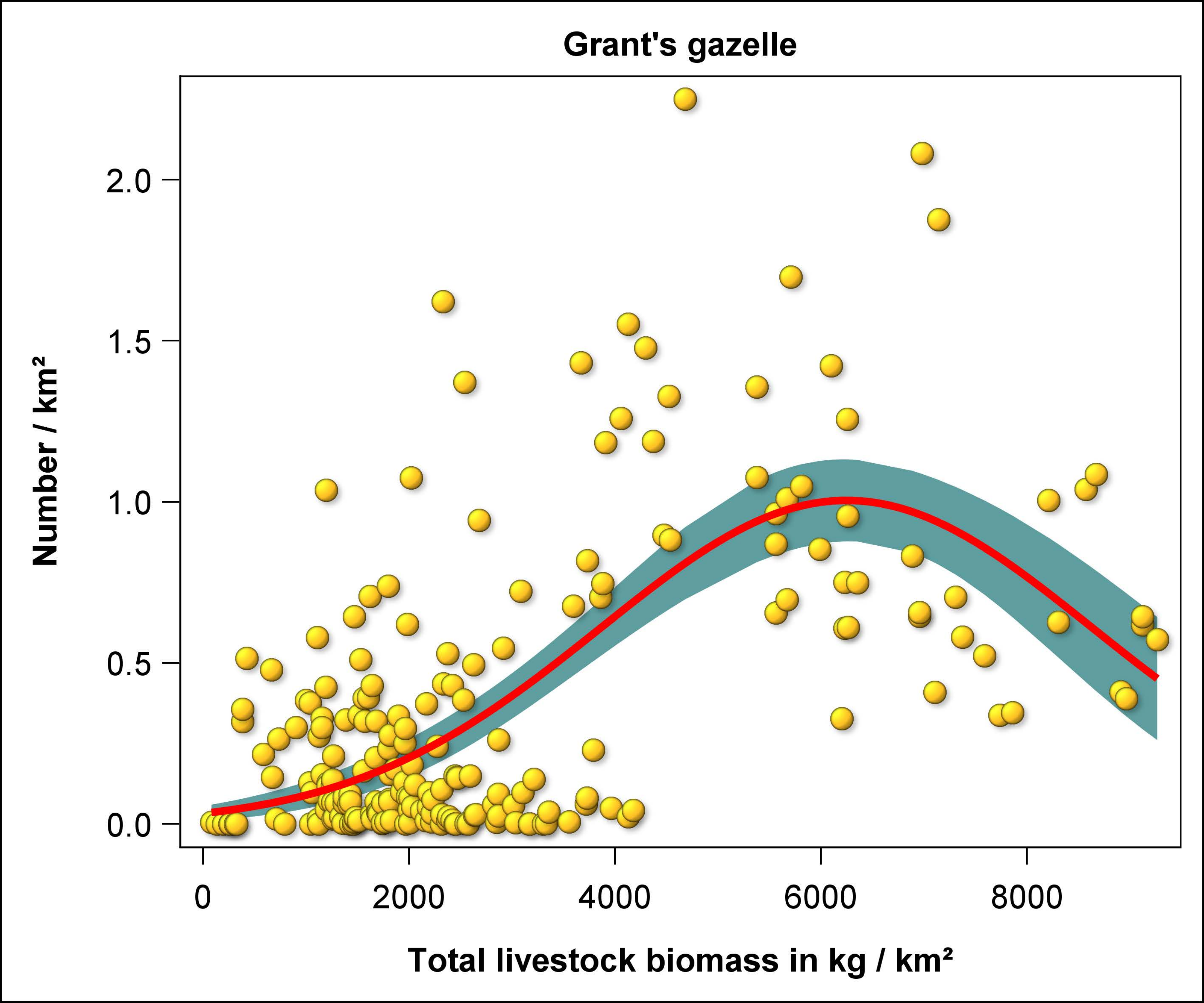

Grant's gazelle
Number / km²
0.0
0.5
1.0
1.5
2.0
0
2000
4000
6000
8000
Total livestock biomass in kg / km²

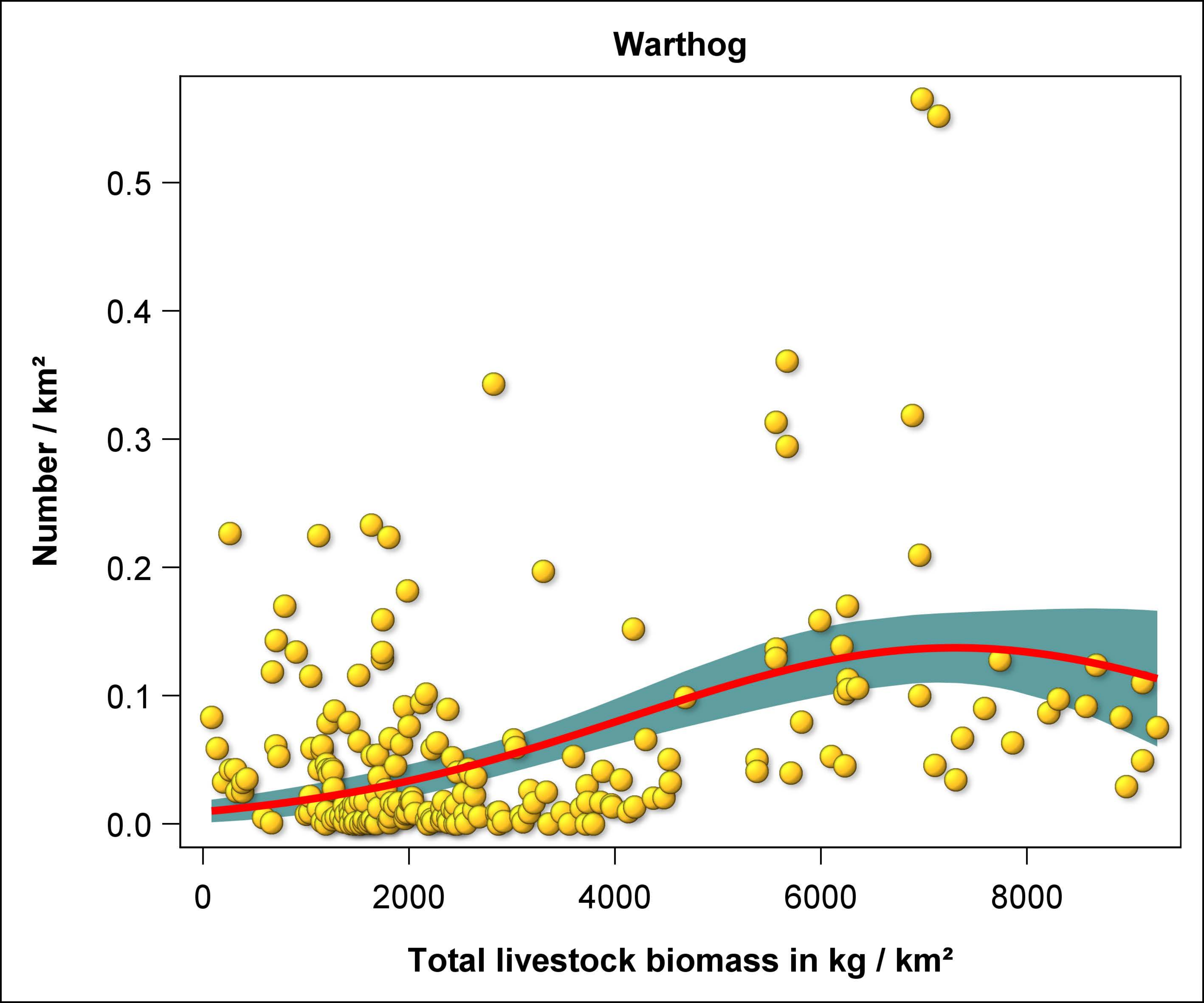
Warthog
Number / km²
0.0
0.1
0.2
0.3
0.4
0.5
0
2000
4000
6000
8000
Total livestock biomass in kg / km²

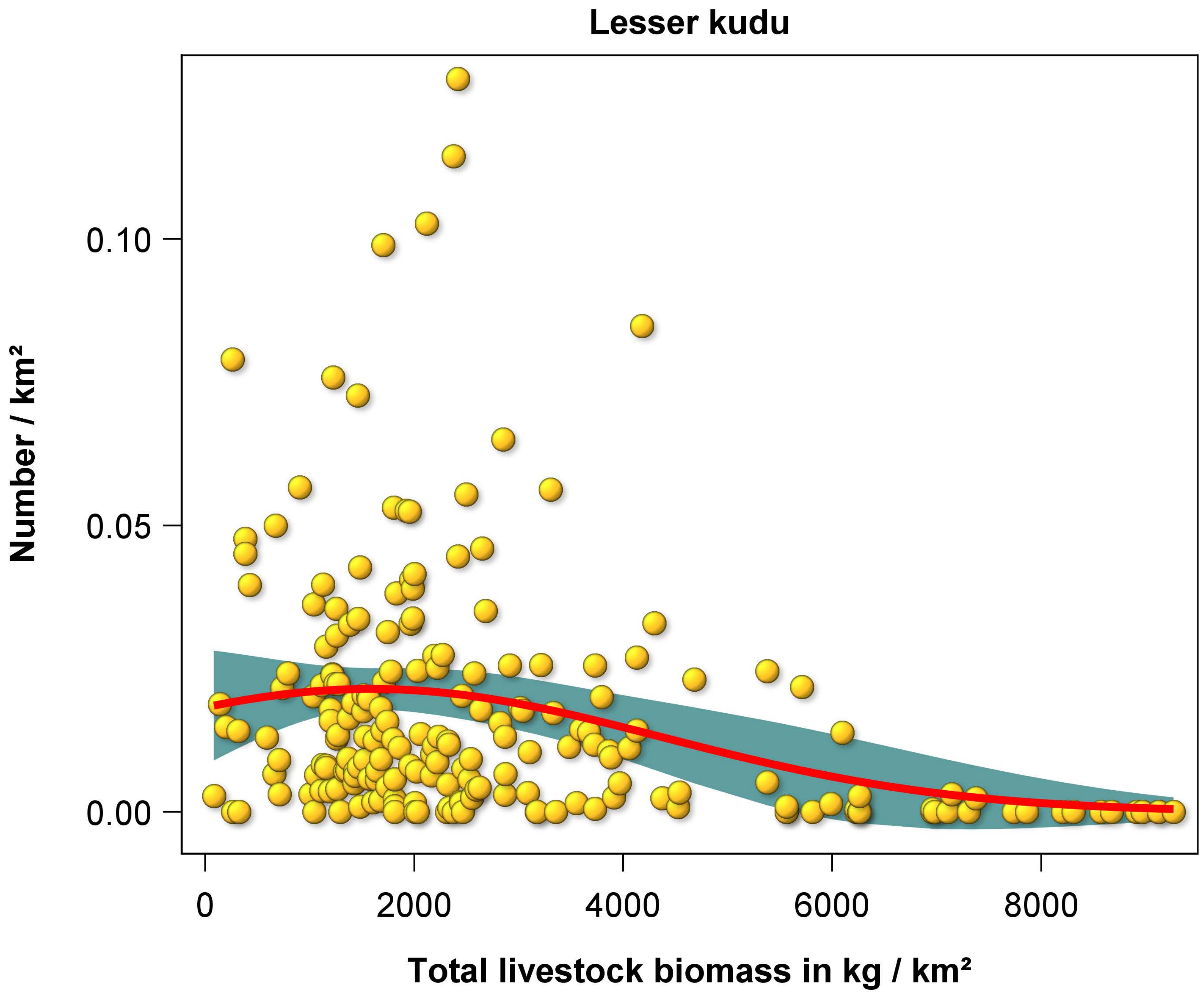
Lesser kudu
Number / km²
0.10
0.05
0.00
0
2000
4000
6000
8000
Total livestock biomass in kg / km²

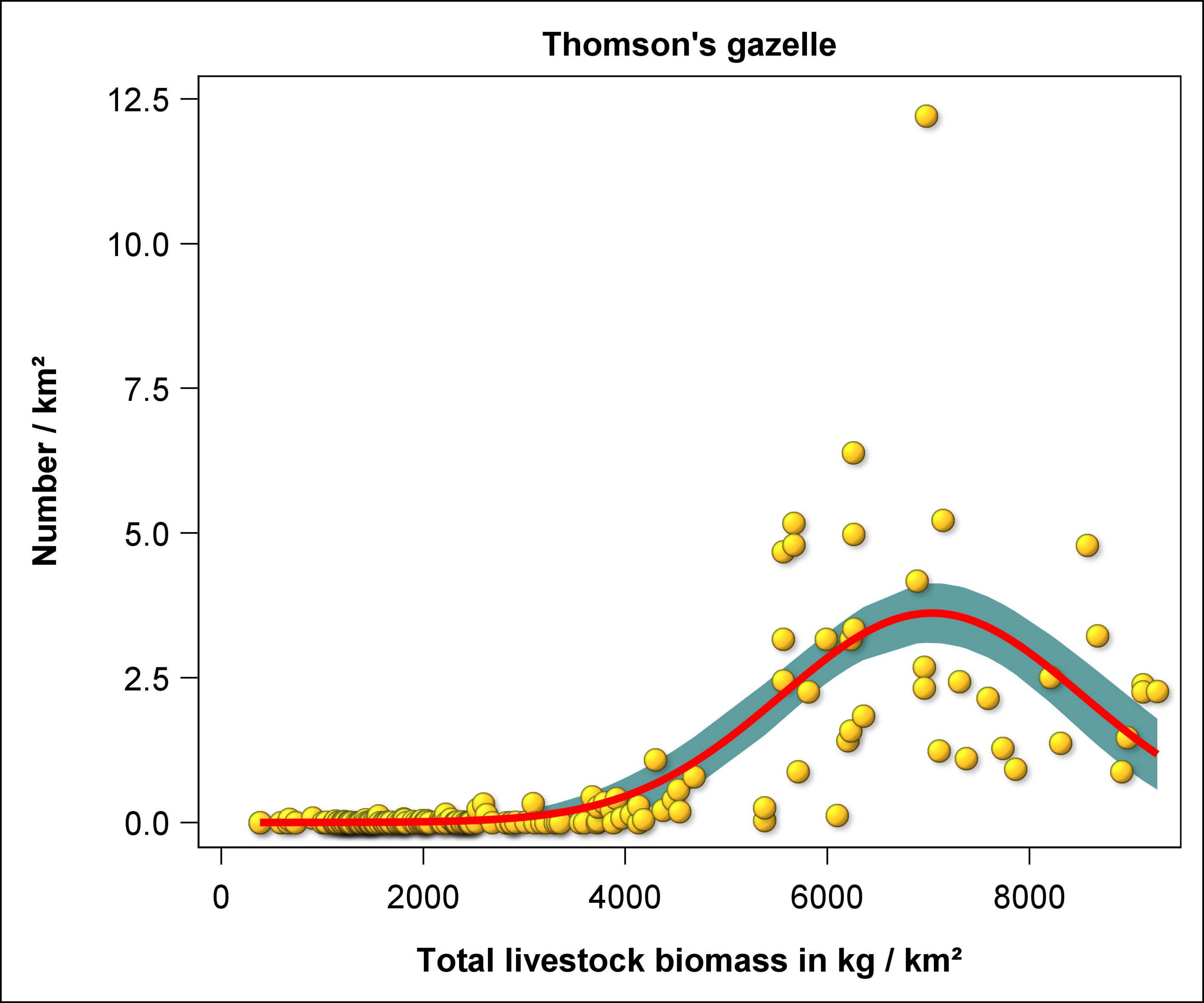

Thomson's gazelle
Number / km²
Total livestock biomass in kg / km²
12.5
10.0
7.5
5.0
2.5
0.0
0
2000
4000
6000
8000

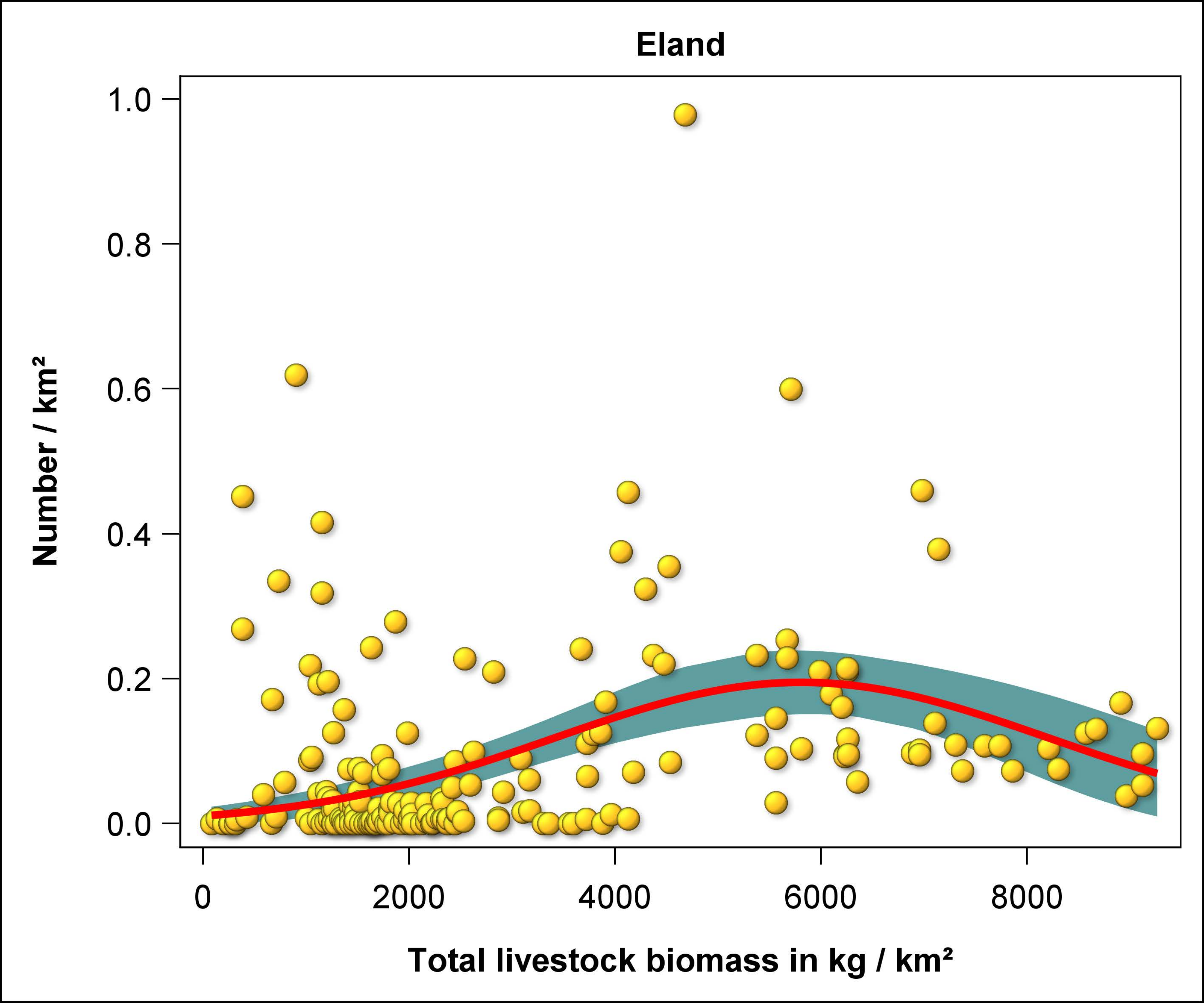
Eland
Number / km²
Total livestock biomass in kg / km²
1.0
0.8
0.6
0.4
0.2
0.0
0
2000
4000
6000
8000

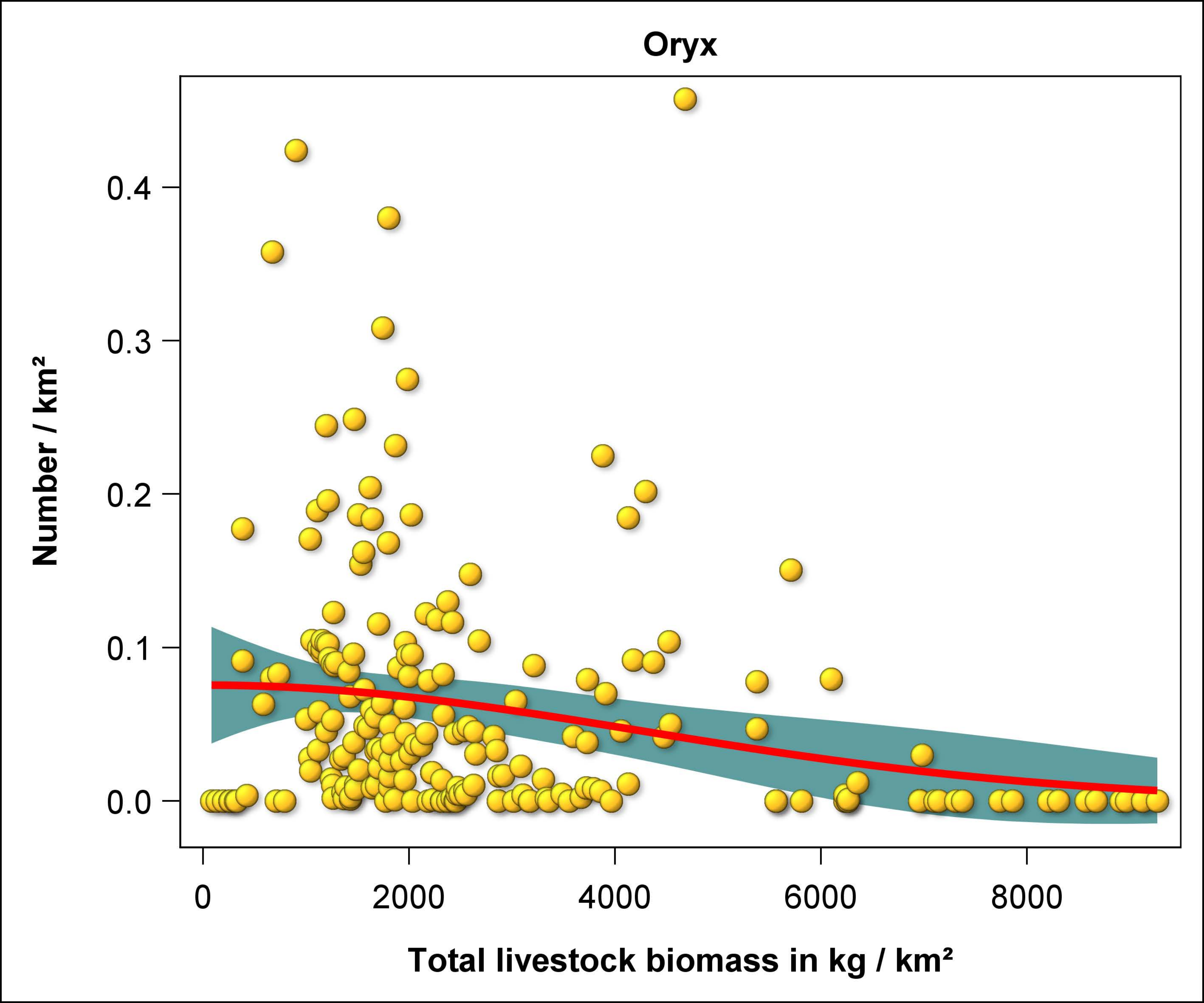

Oryx
Number / km²
0.0
0.1
0.2
0.3
0.4
0
2000
4000
6000
8000
Total livestock biomass in kg / km²

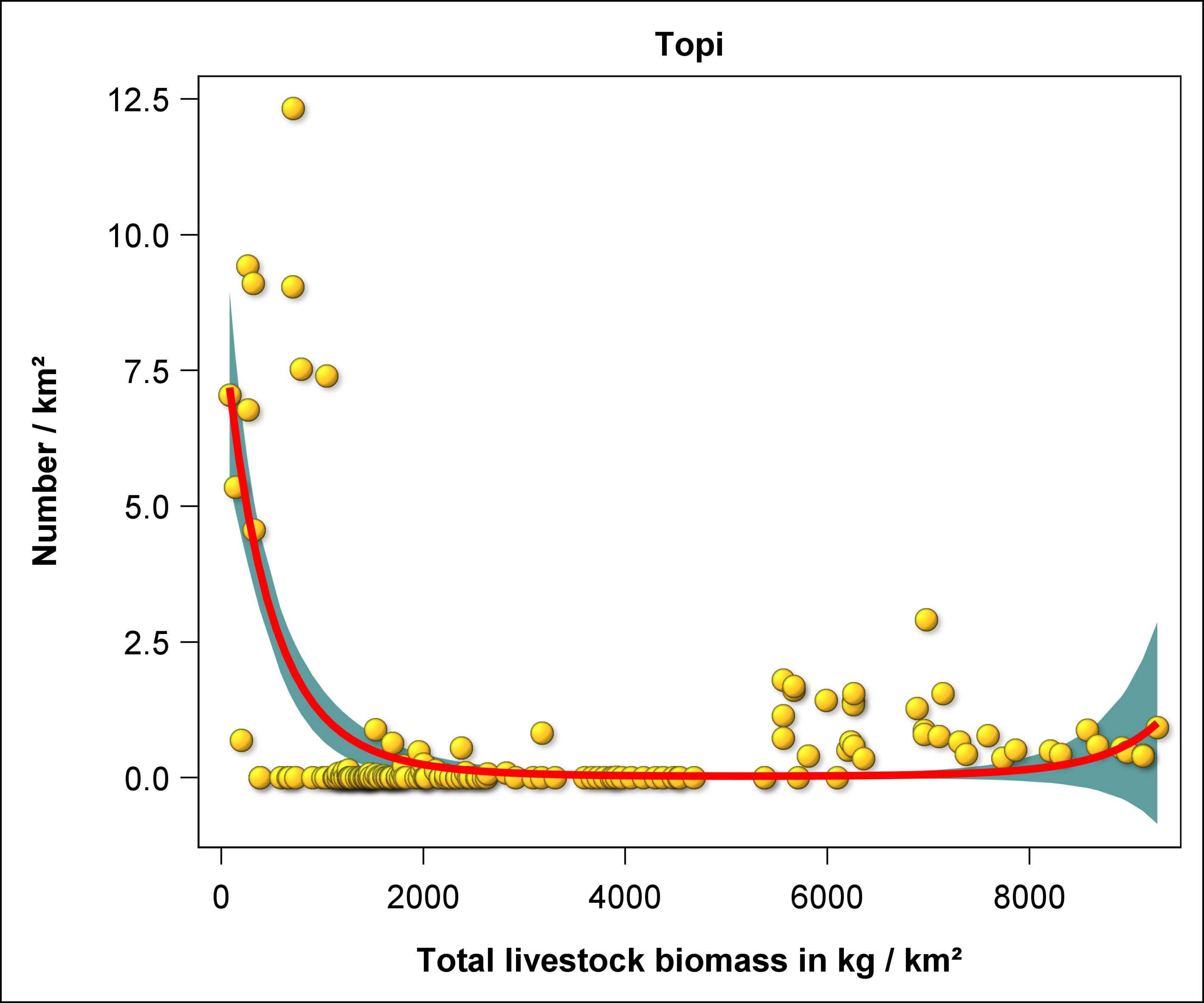

Topi
12.5
10.0
7.5
5.0
2.5
0.0
Number / km²
0
2000
4000
6000
8000
Total livestock biomass in kg / km²

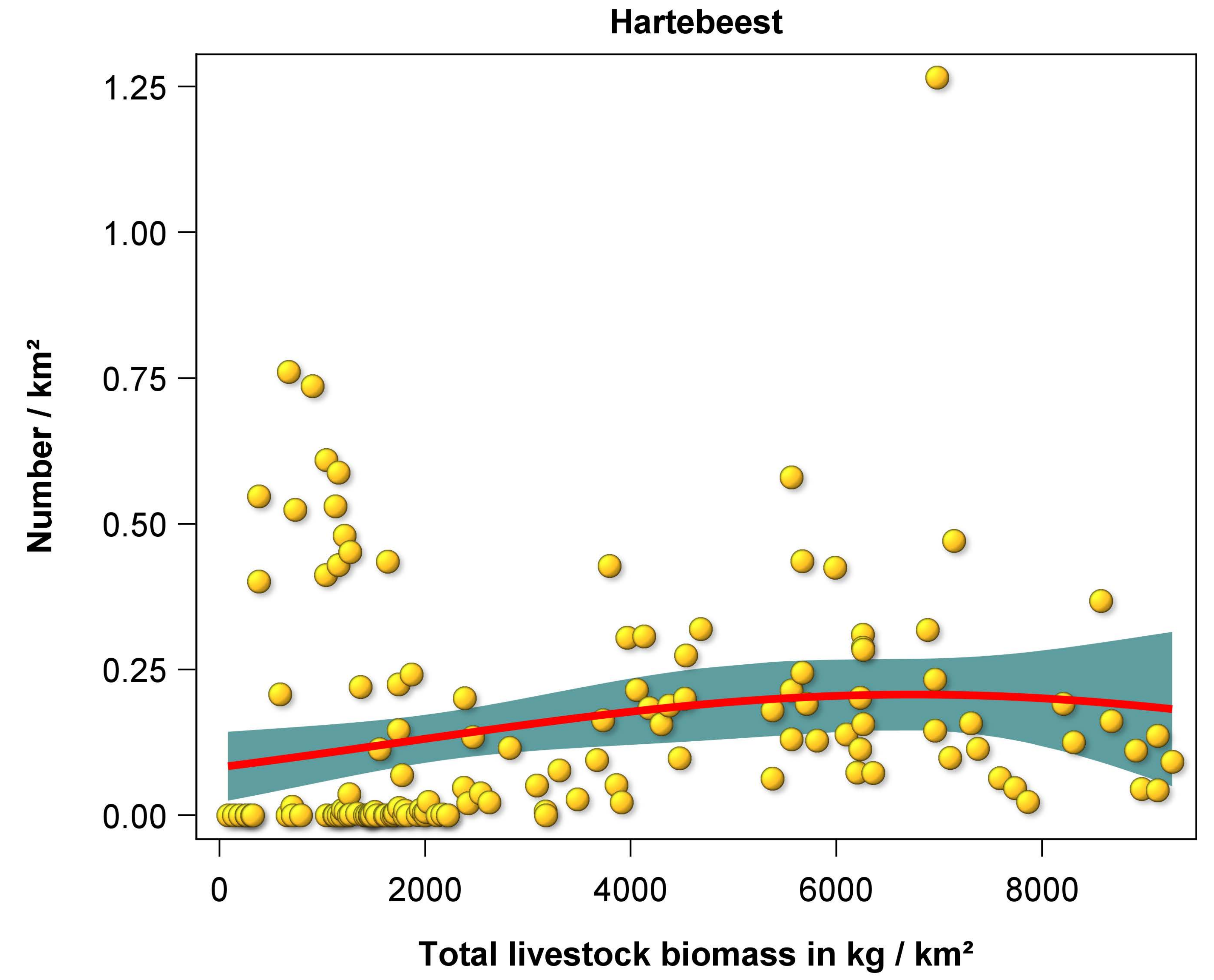

Hartebeest
Number / km²
1.25
1.00
0.75
0.50
0.25
0.00
0
2000
4000
6000
8000
Total livestock biomass in kg / km²

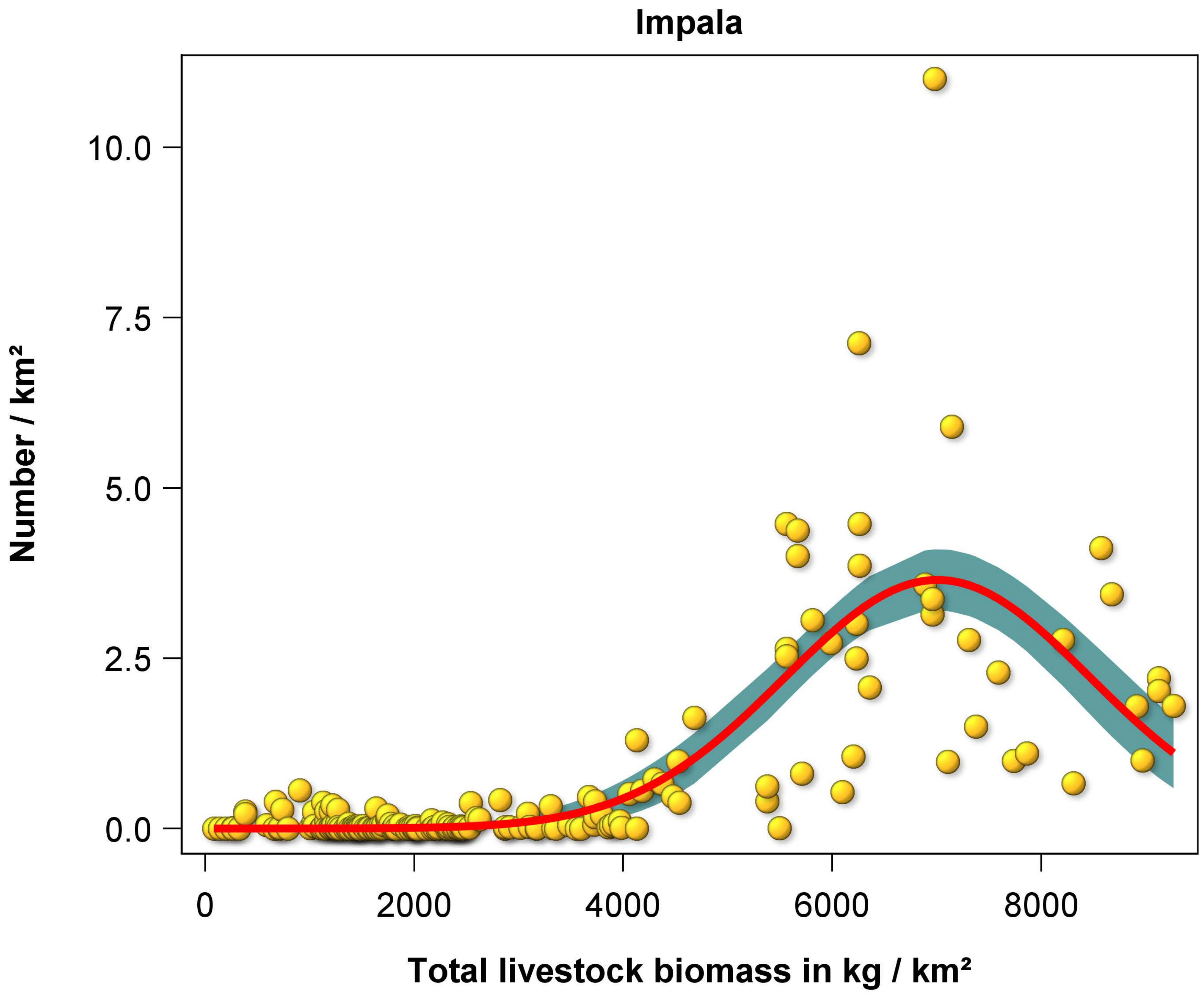
Impala
Number / km²
10.0
7.5
5.0
2.5
0.0
0
2000
4000
6000
8000
Total livestock biomass in kg / km²

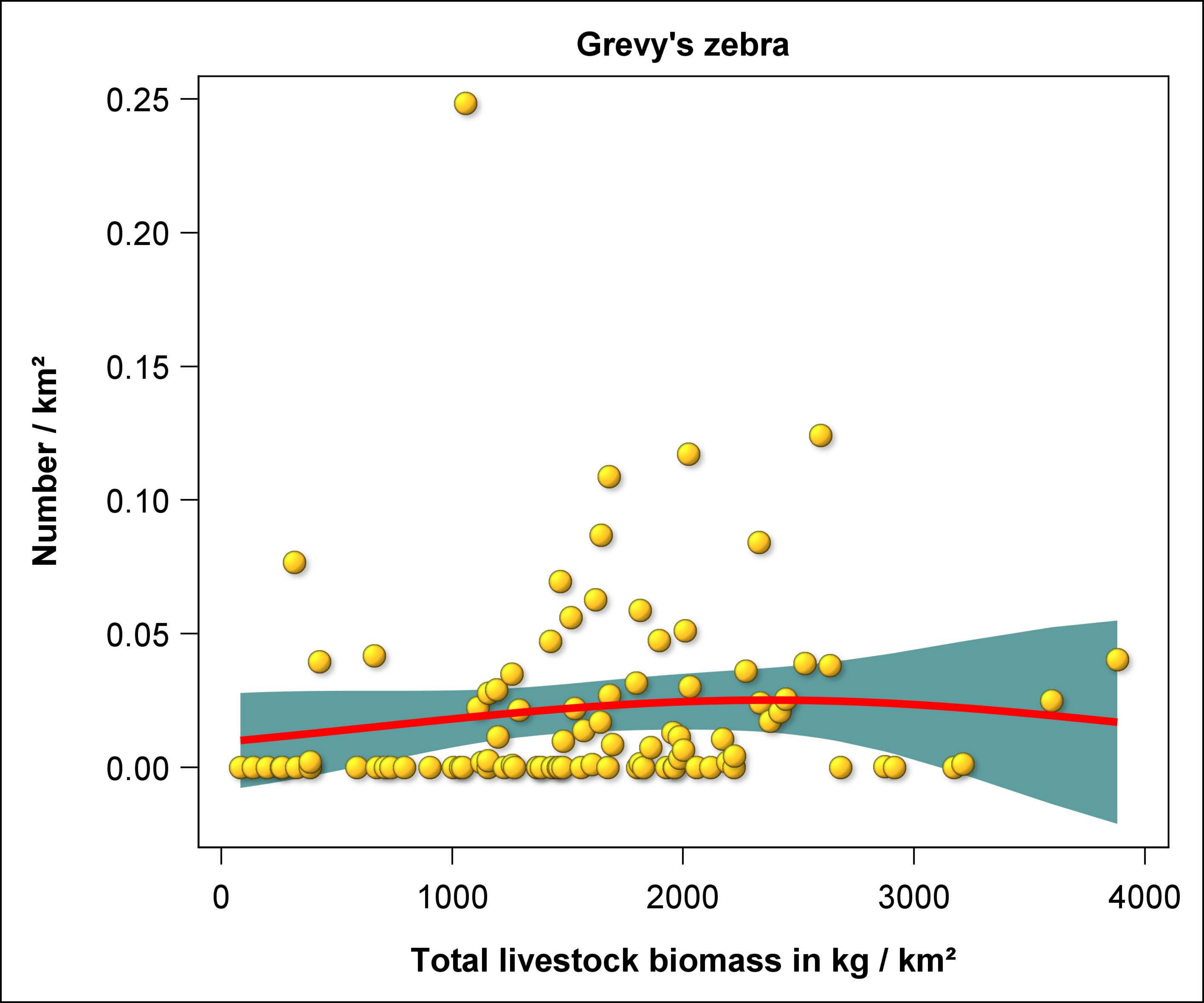
Grevy's zebra
0.25
0.20
0.15
0.10
0.05
0.00
Number / km²
0
1000
2000
3000
4000
Total livestock biomass in kg / km²

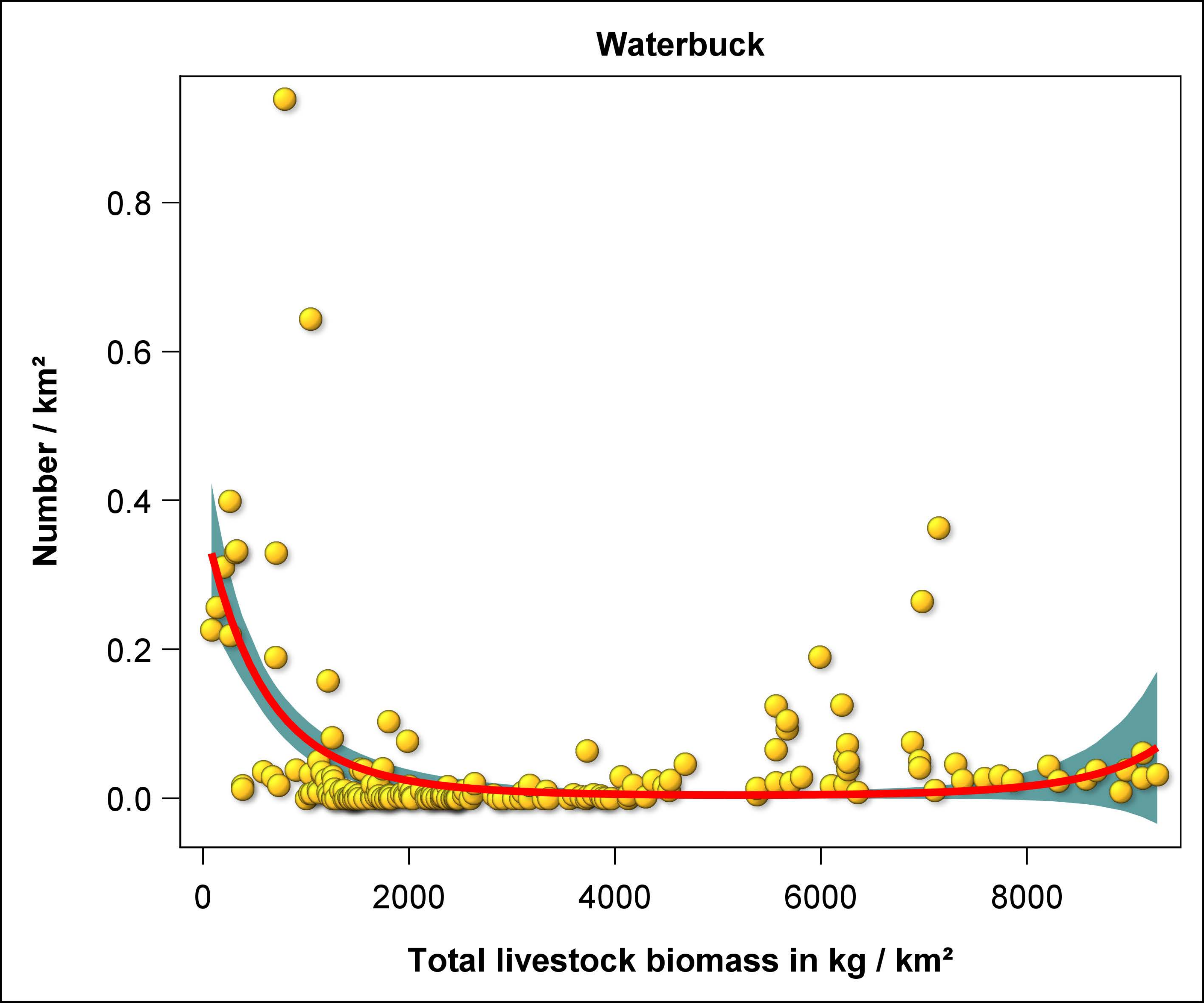

Waterbuck
Number / km²
0.0
0.2
0.4
0.6
0.8
0
2000
4000
6000
8000
Total livestock biomass in kg / km²
