# Supplementary material for: Extreme Wildlife Declines and Concurrent Increase in Livestock Numbers in Kenya: What Are the Causes?
Source: PLoS One. 2016 Sep 27;11(9):e0163249. doi: 10.1371/journal.pone.0163249 (PMC5039022; doi:10.1371/journal.pone.0163249)
Supplement: S28 Fig — The filled circles are the observations, the solid lines are the quadratic regression lines while the shaded bands are the 95% pointwise confidence bands. (PDF) [file pone.0163249.s038.pdf]

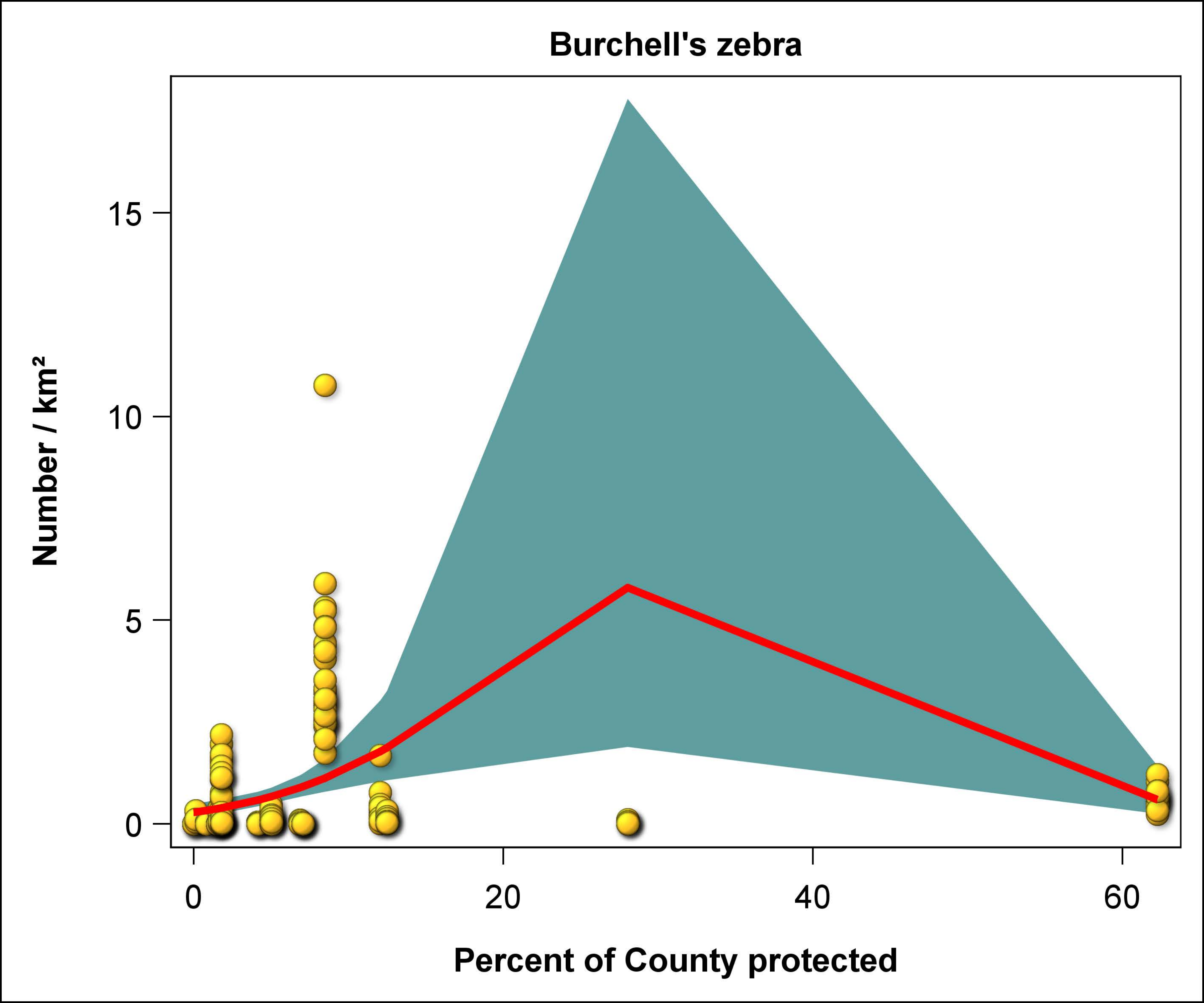
Burchell's zebra
Number / km²
15
10
5
0
0
20
40
60
Percent of County protected

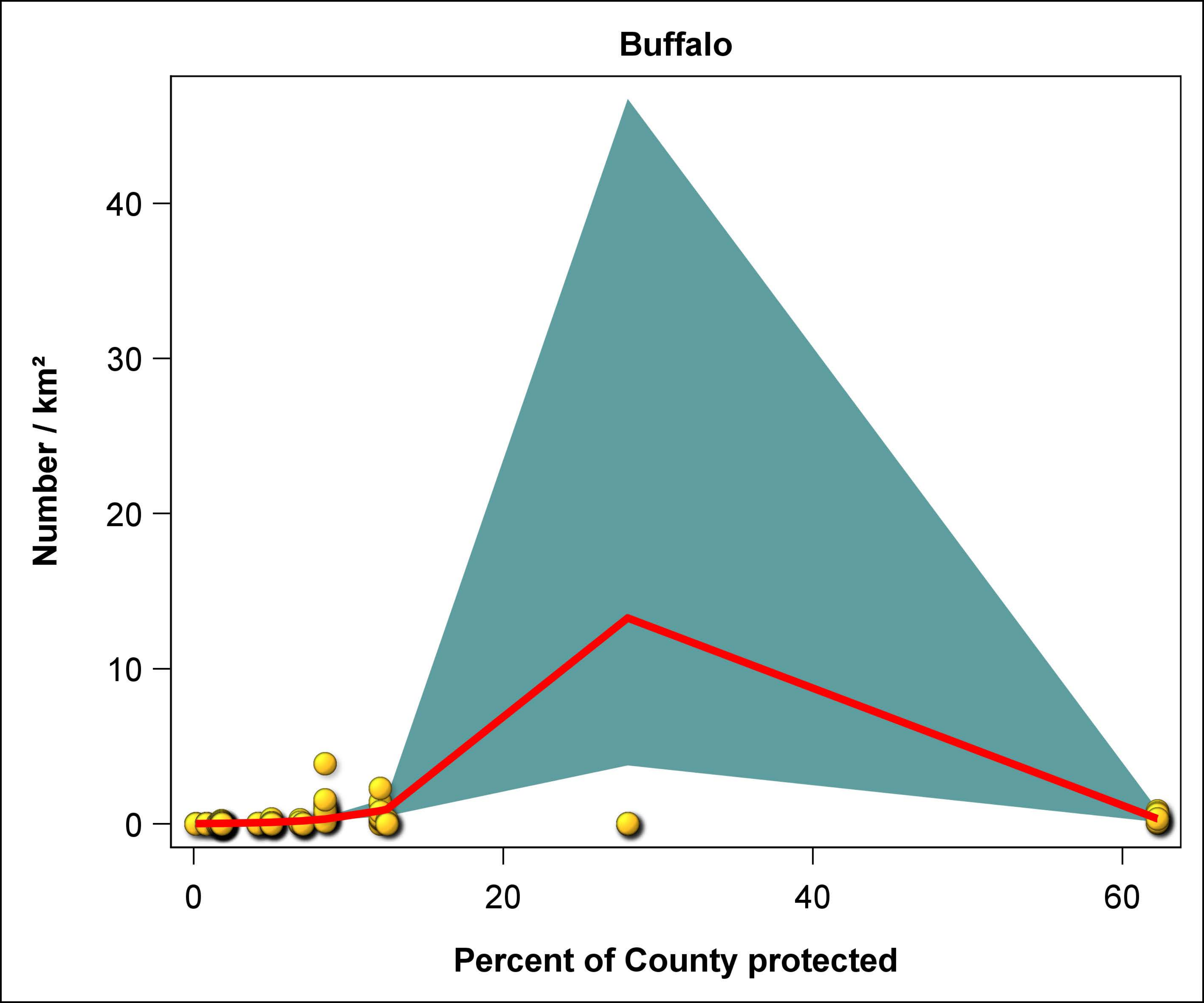

Buffalo
Number / km²
Percent of County protected
0
10
20
30
40
0
20
40
60

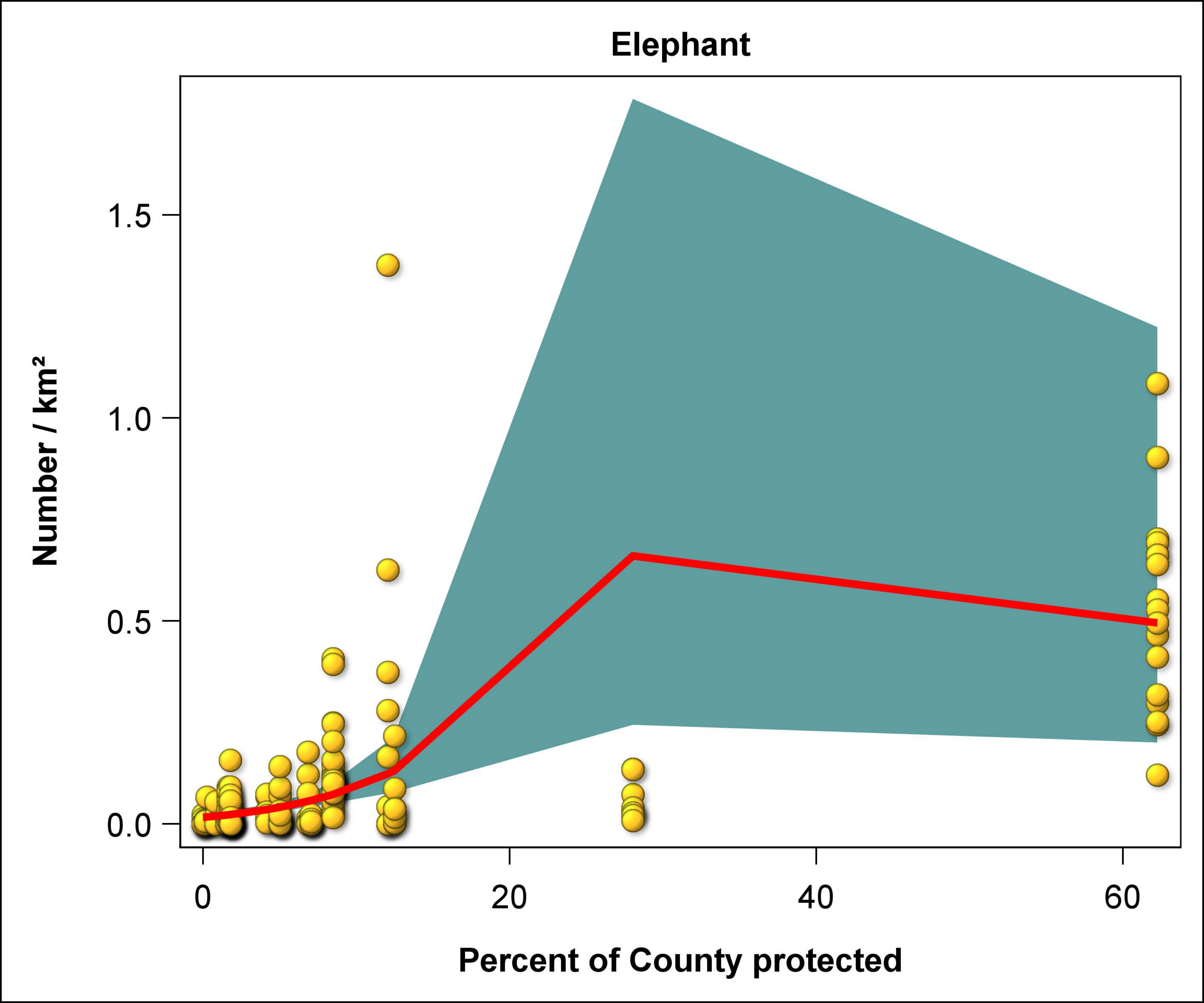

Elephant
Number / km²
0.0
0.5
1.0
1.5
0
20
40
60
Percent of County protected

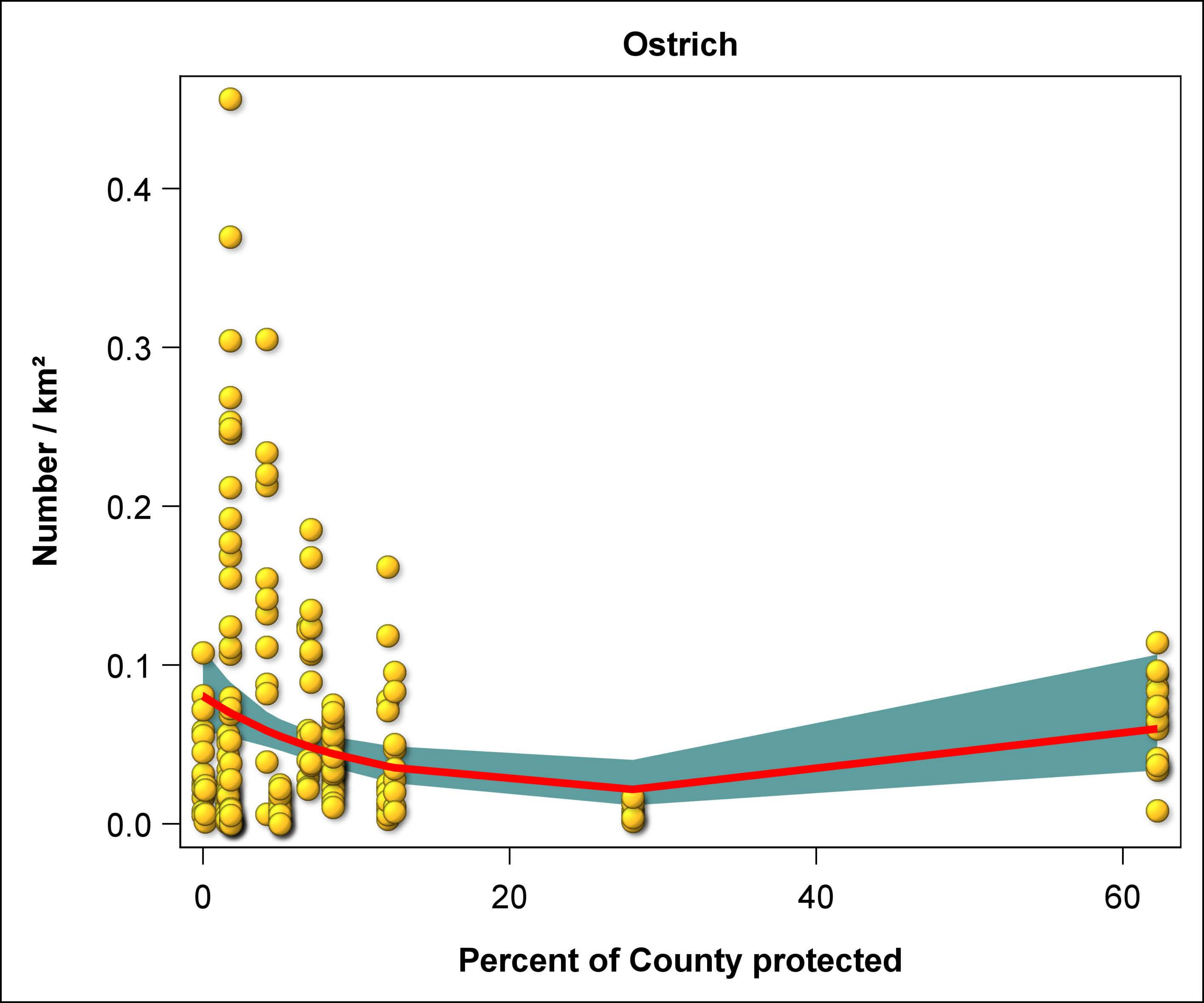

Ostrich
Number / km²
0.0
0.1
0.2
0.3
0.4
0
20
40
60
Percent of County protected

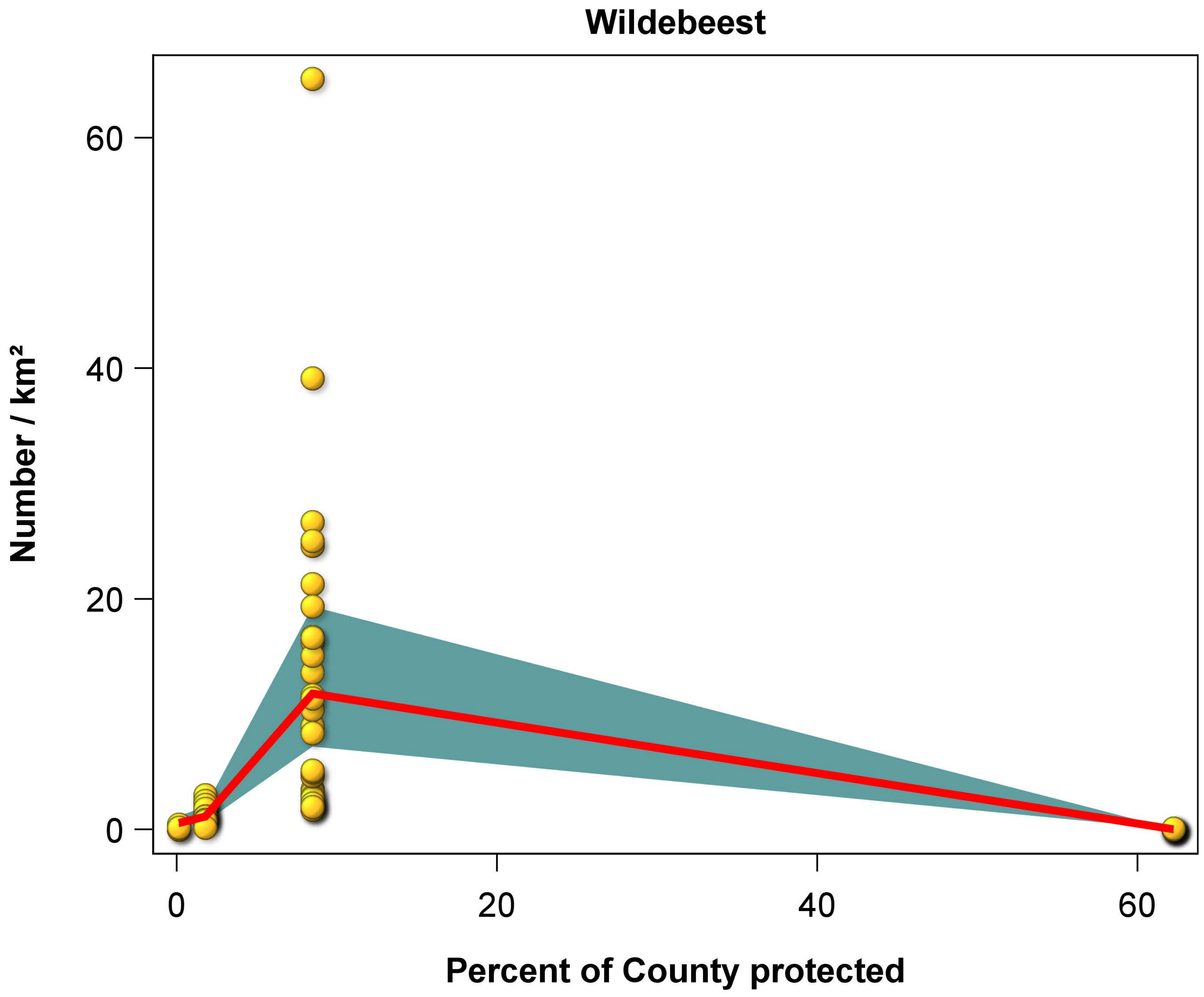
Wildebeest
Number / km²
0
20
40
60
0
20
40
60
Percent of County protected

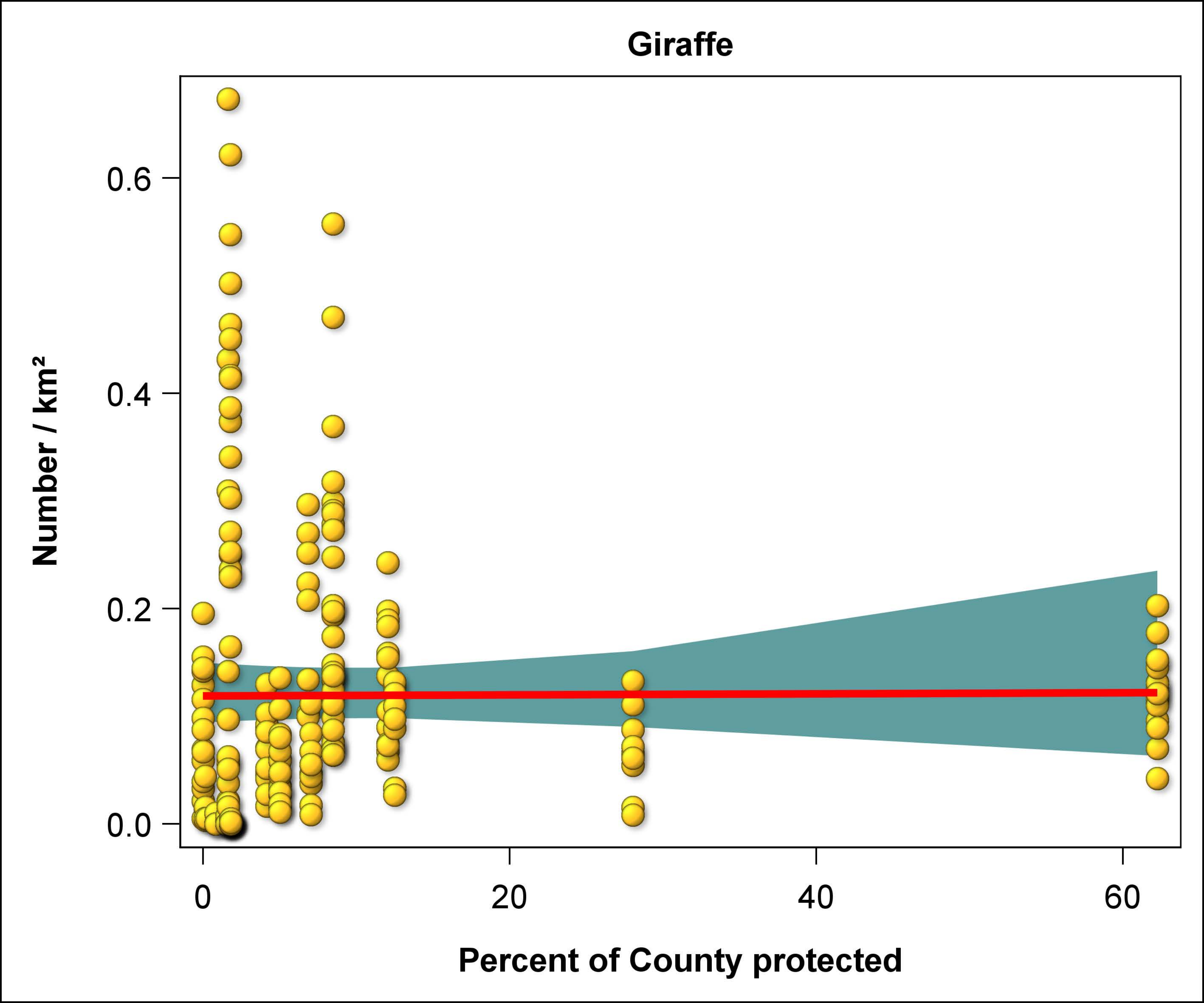

Giraffe
0.0
0.2
0.4
0.6
Number / km²
0
20
40
60
Percent of County protected

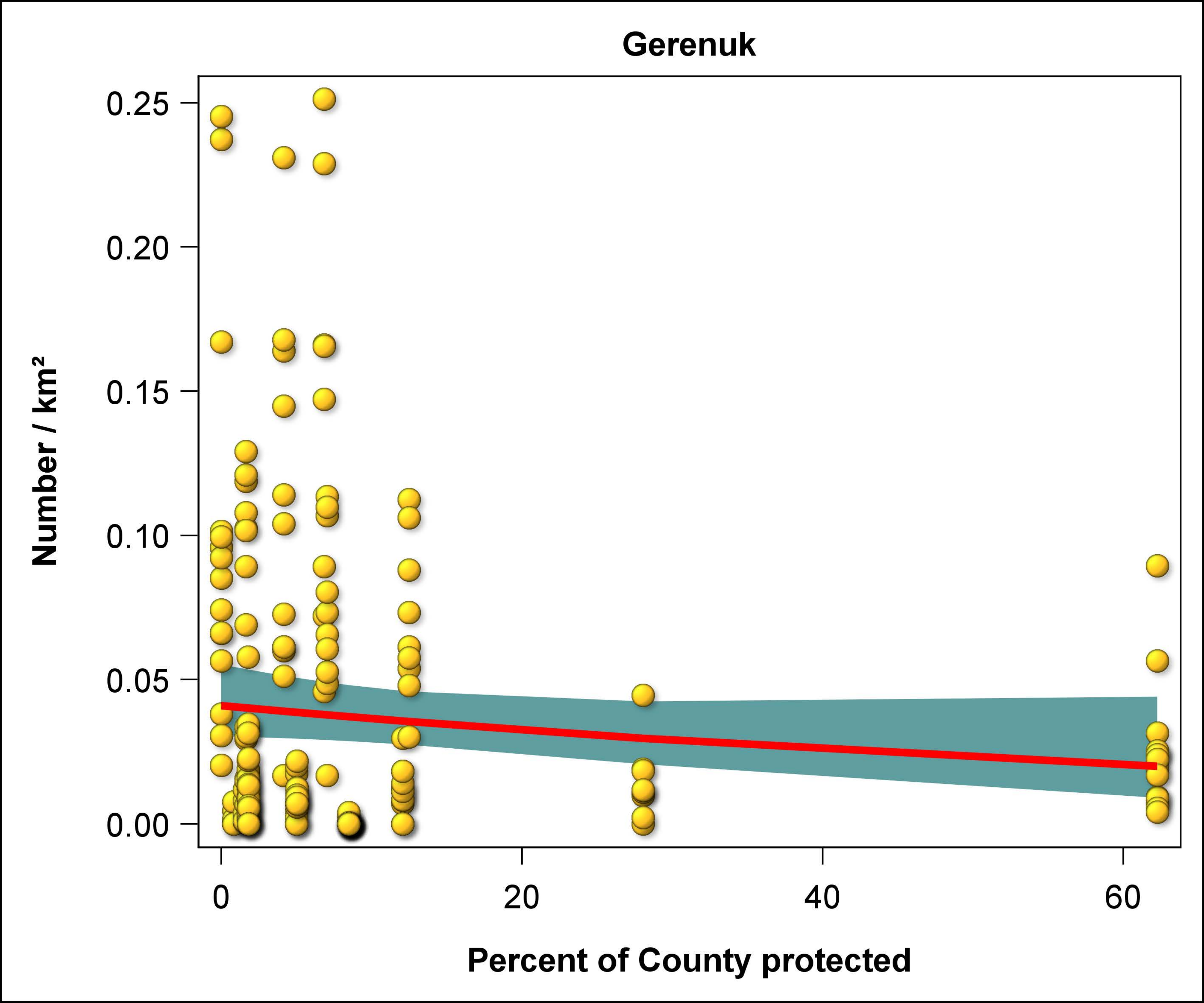

Gerenuk
Number / km²
0.25
0.20
0.15
0.10
0.05
0.00
0
20
40
60
Percent of County protected

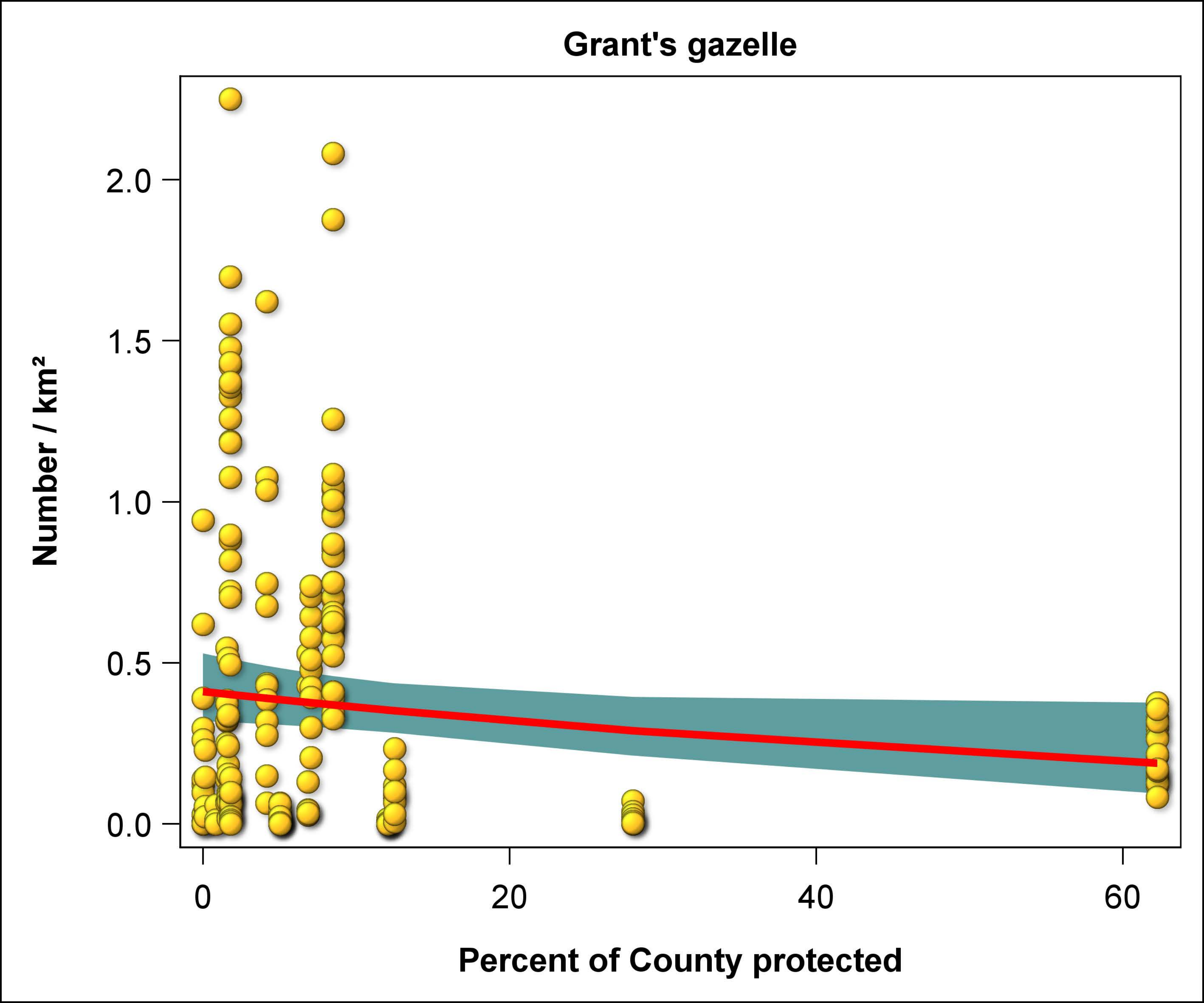

Grant's gazelle
Number / km²
0.0
0.5
1.0
1.5
2.0
0
20
40
60
Percent of County protected

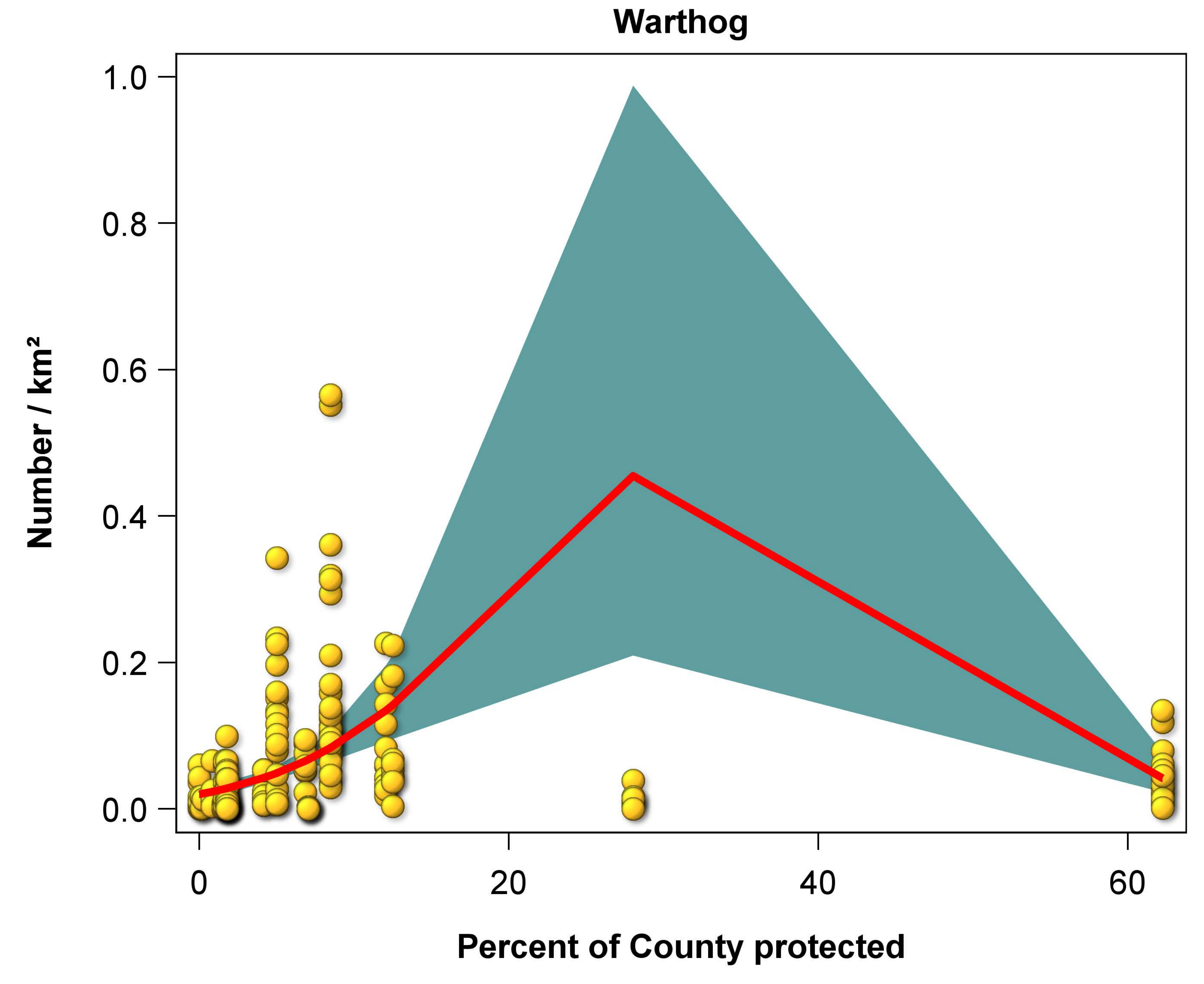
Warthog
Number / km²
1.0
0.8
0.6
0.4
0.2
0.0
0
20
40
60
Percent of County protected

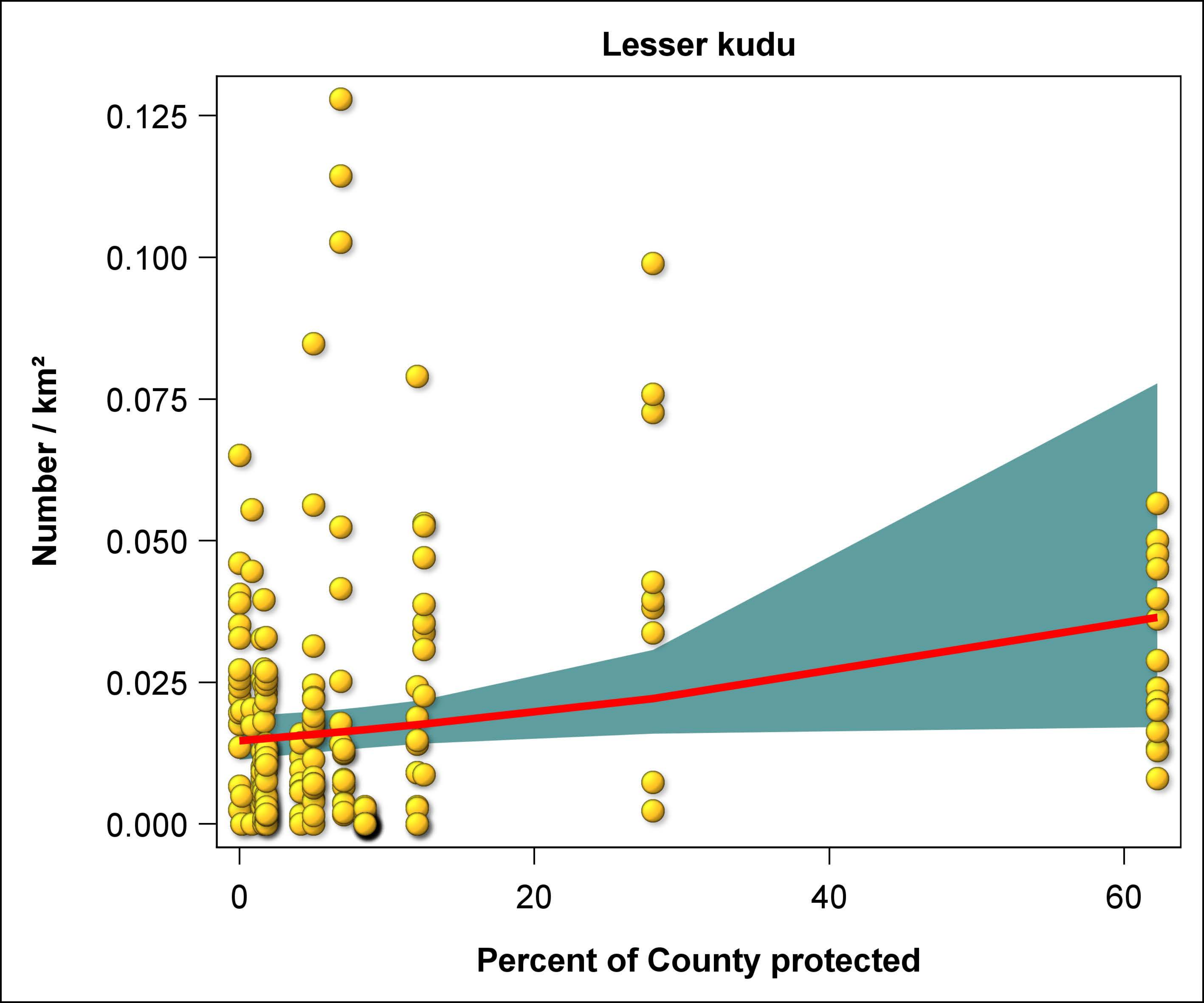

Lesser kudu
0.125
0.100
0.075
0.050
0.025
0.000
Number / km²
0
20
40
60
Percent of County protected

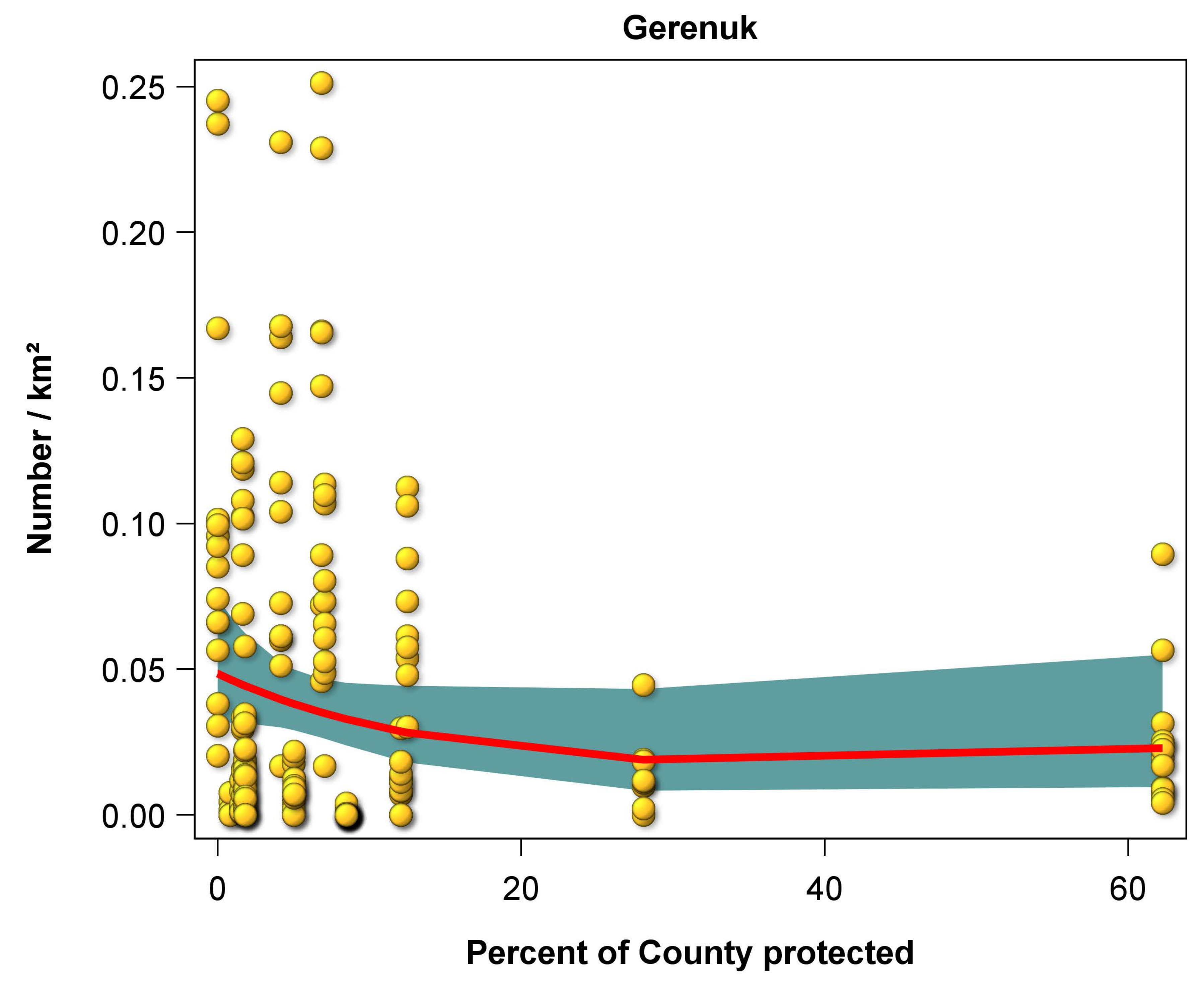

Gerenuk
Number / km²
0.00
0.05
0.10
0.15
0.20
0.25
0
20
40
60
Percent of County protected

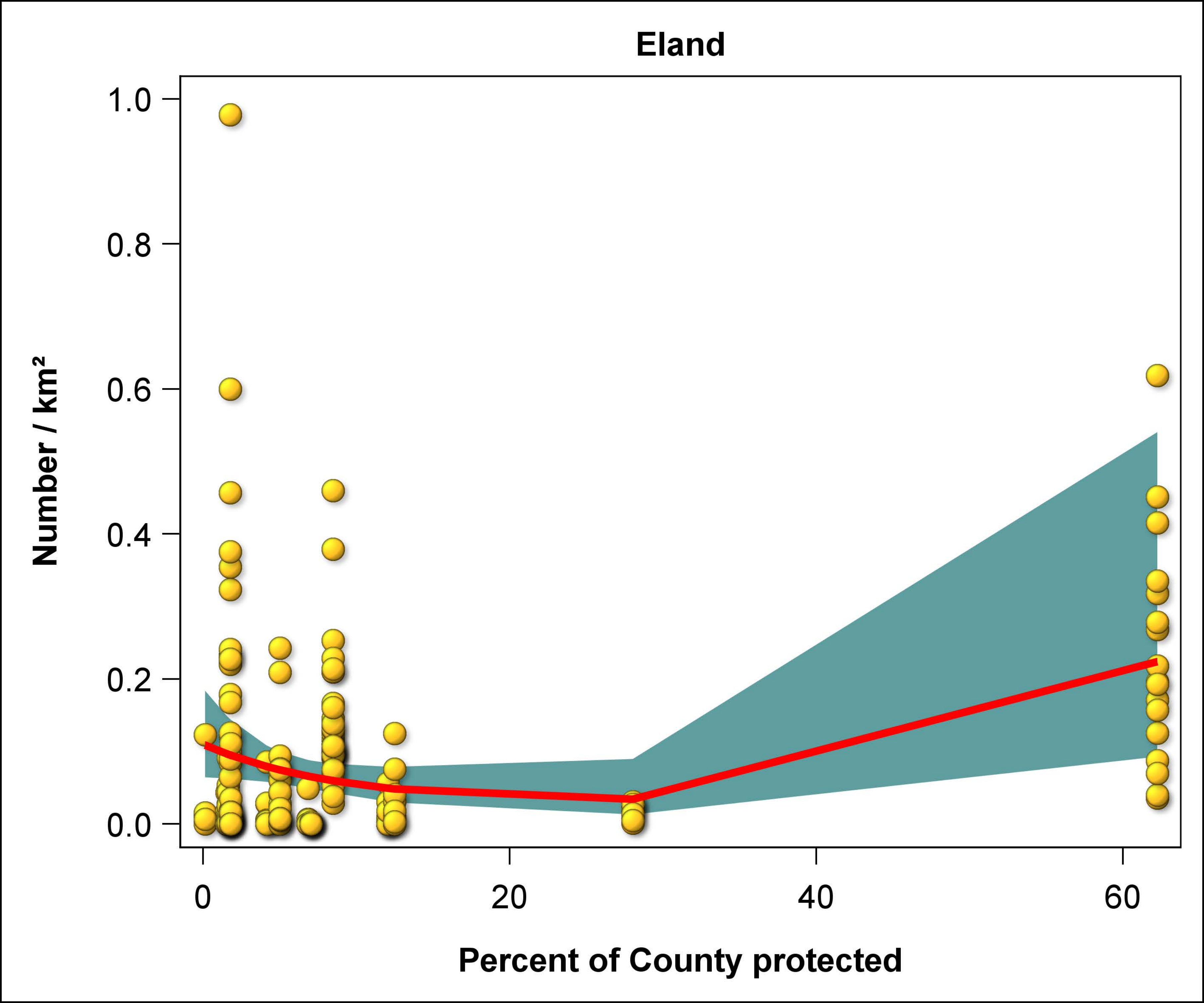
Eland
Number / km²
1.0
0.8
0.6
0.4
0.2
0.0
0
20
40
60
Percent of County protected

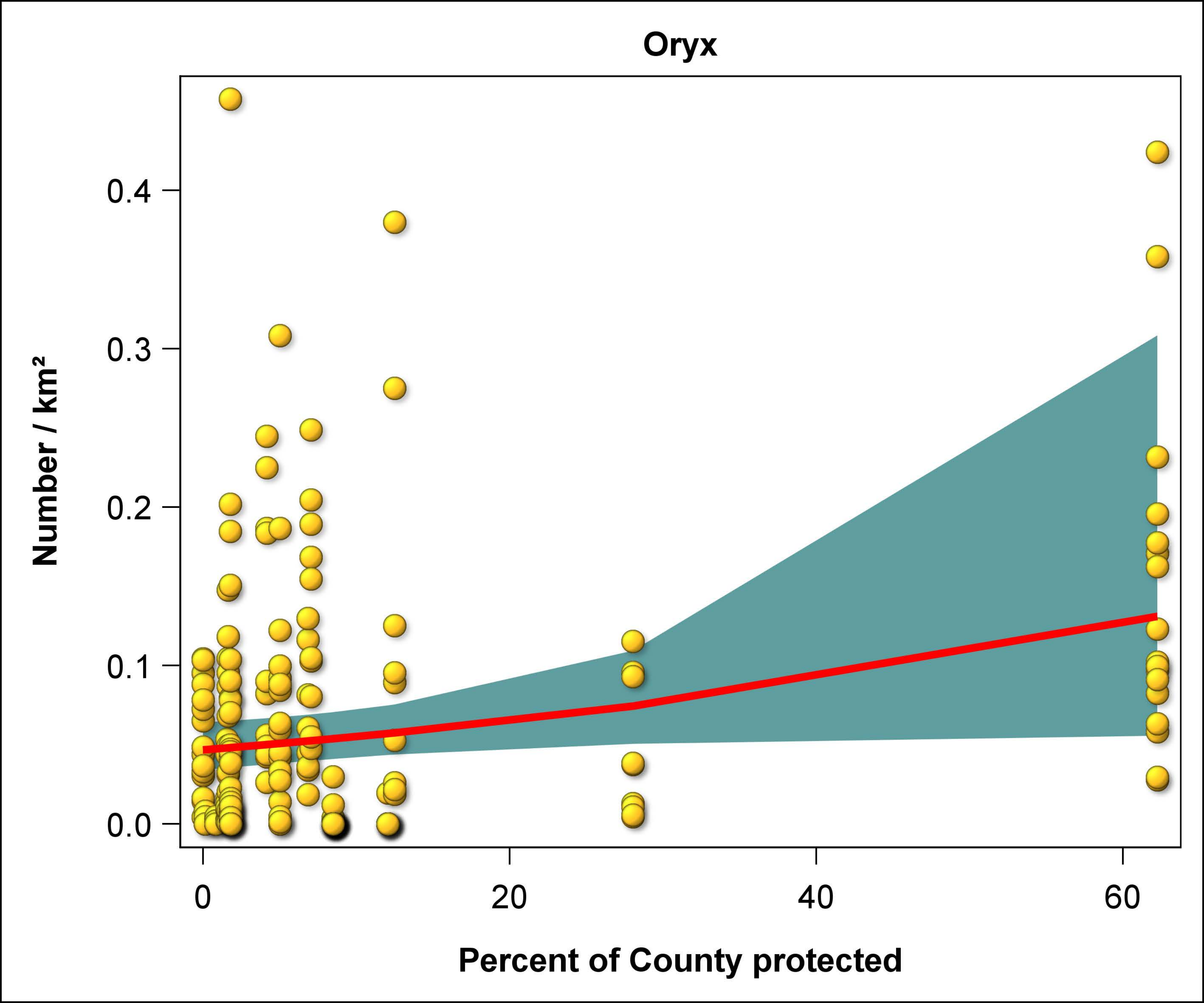

Oryx
Number / km²
0.0
0.1
0.2
0.3
0.4
0
20
40
60
Percent of County protected

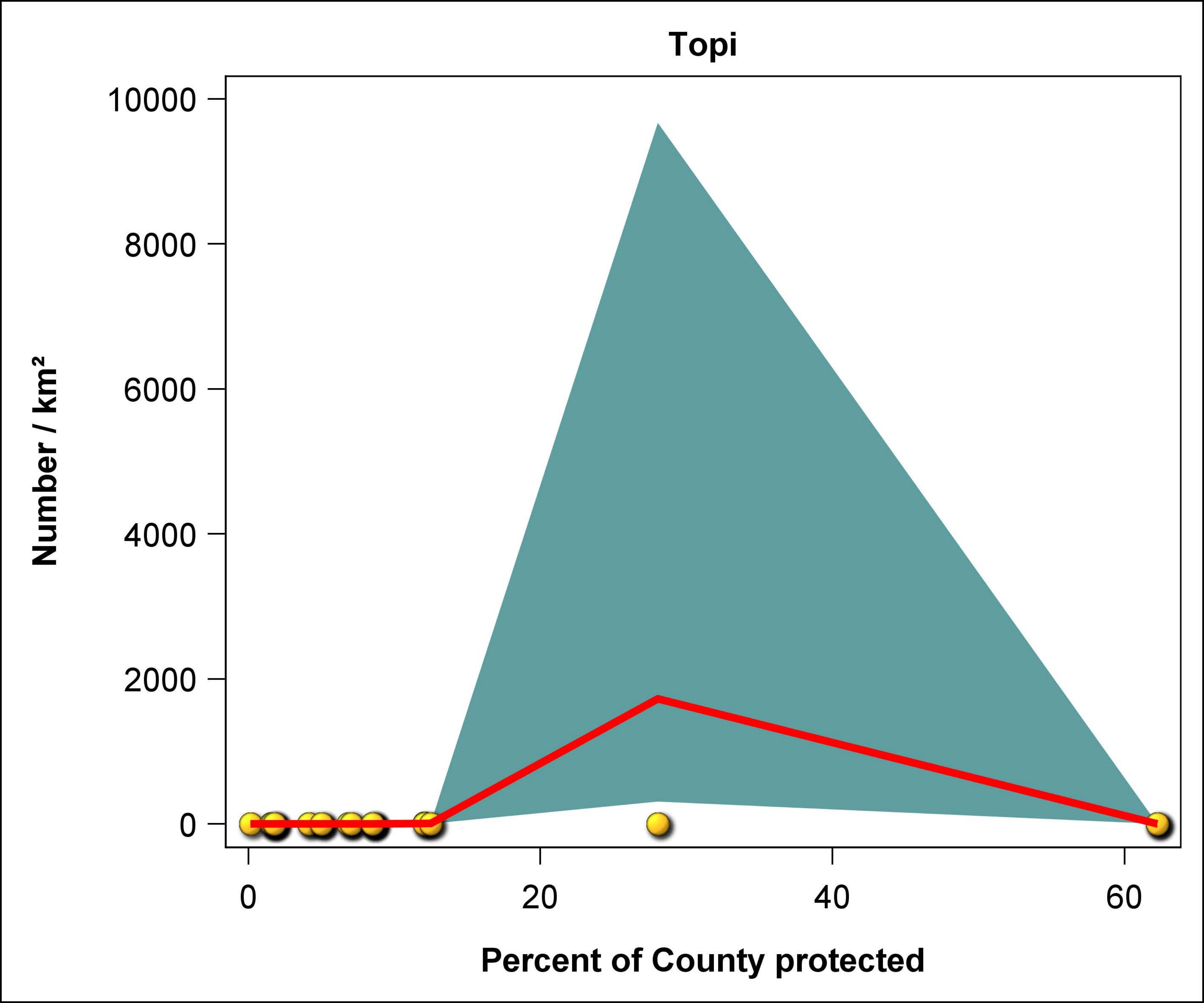

Topi
10000
8000
6000
4000
2000
0
Number / km²
0
20
40
60
Percent of County protected

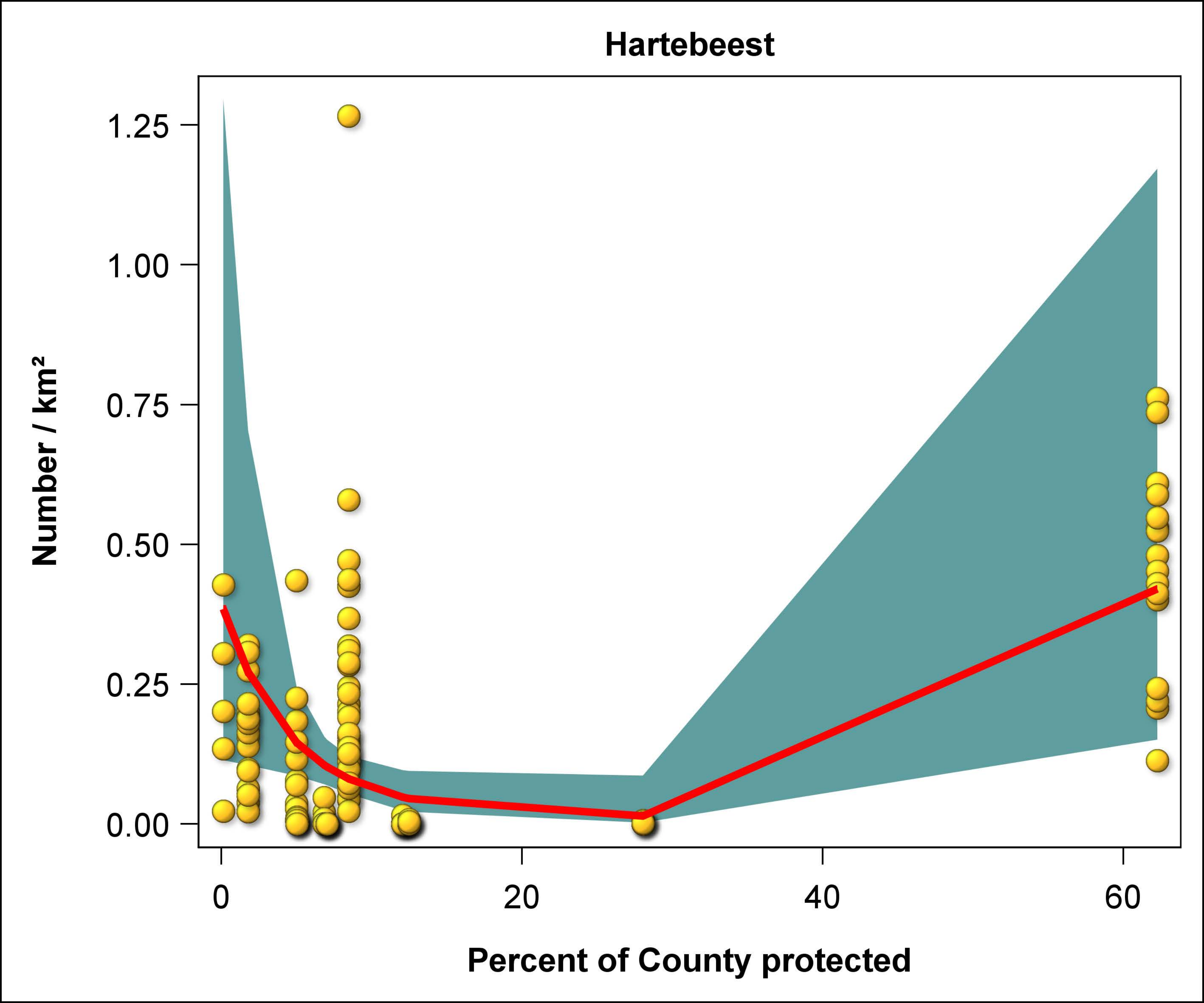

Hartebeest
Number / km²
1.25
1.00
0.75
0.50
0.25
0.00
0
20
40
60
Percent of County protected

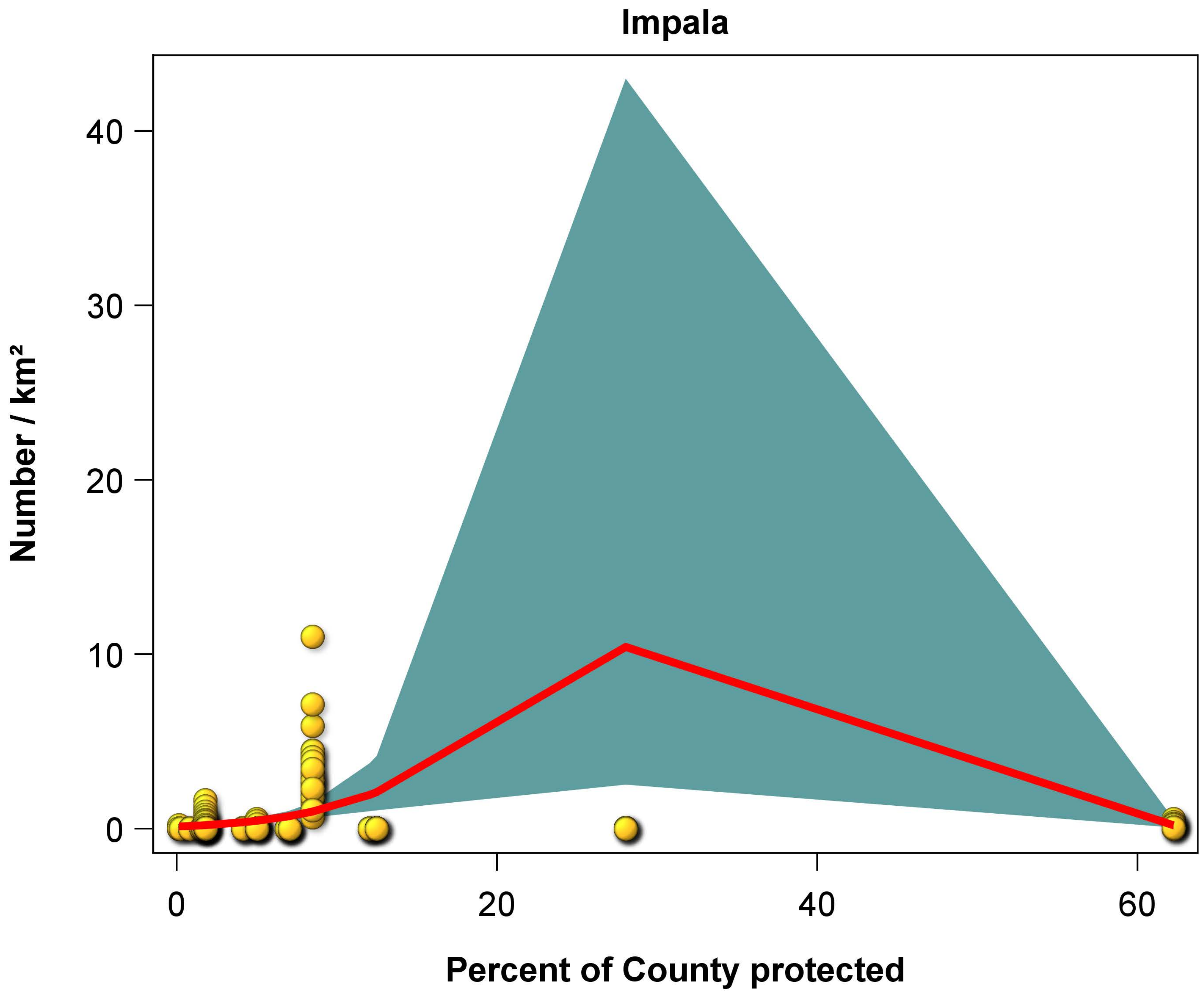
Impala
Number / km²
0
10
20
30
40
0
20
40
60
Percent of County protected

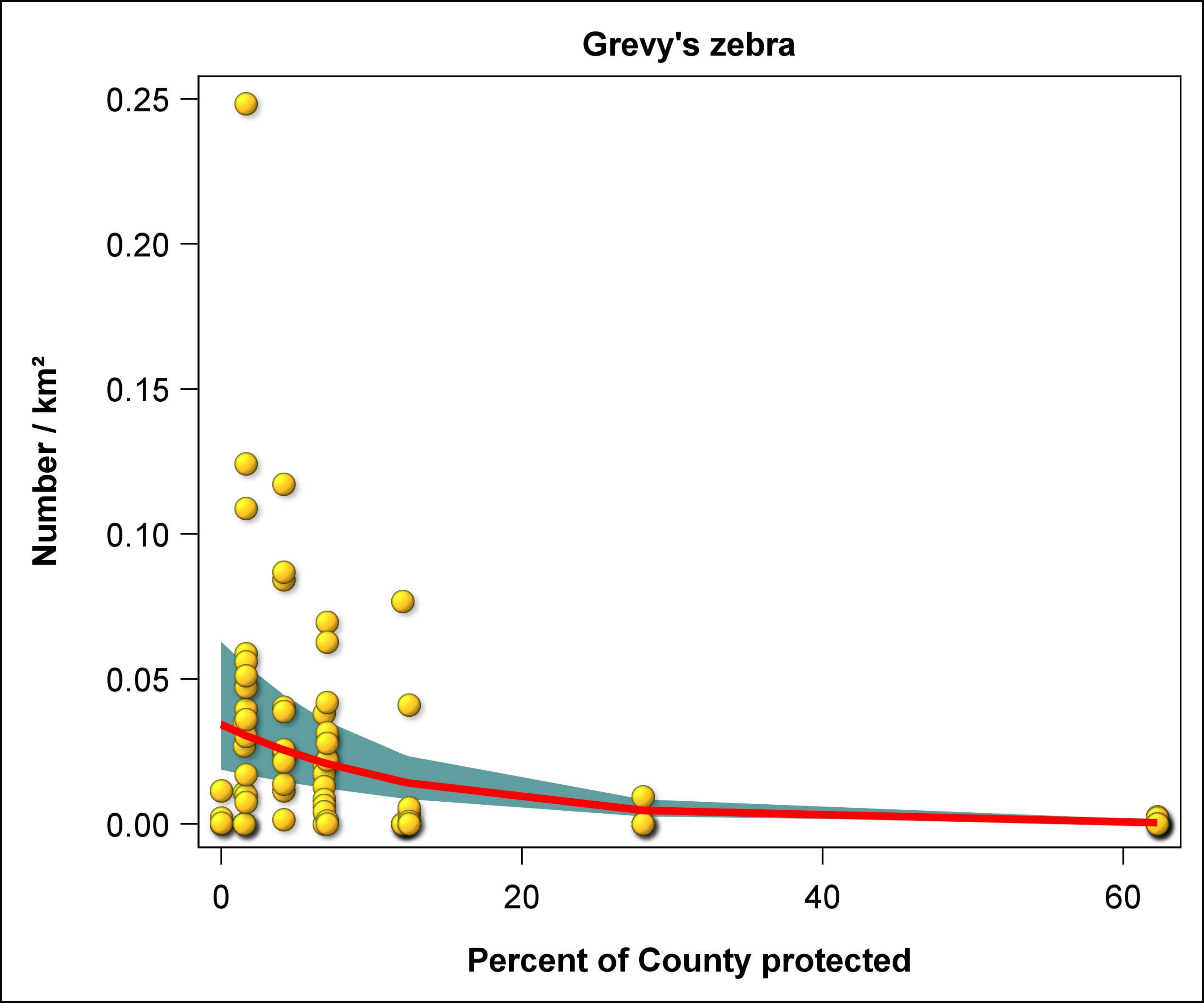

Grevy's zebra
Number / km²
0.25
0.20
0.15
0.10
0.05
0.00
0
20
40
60
Percent of County protected

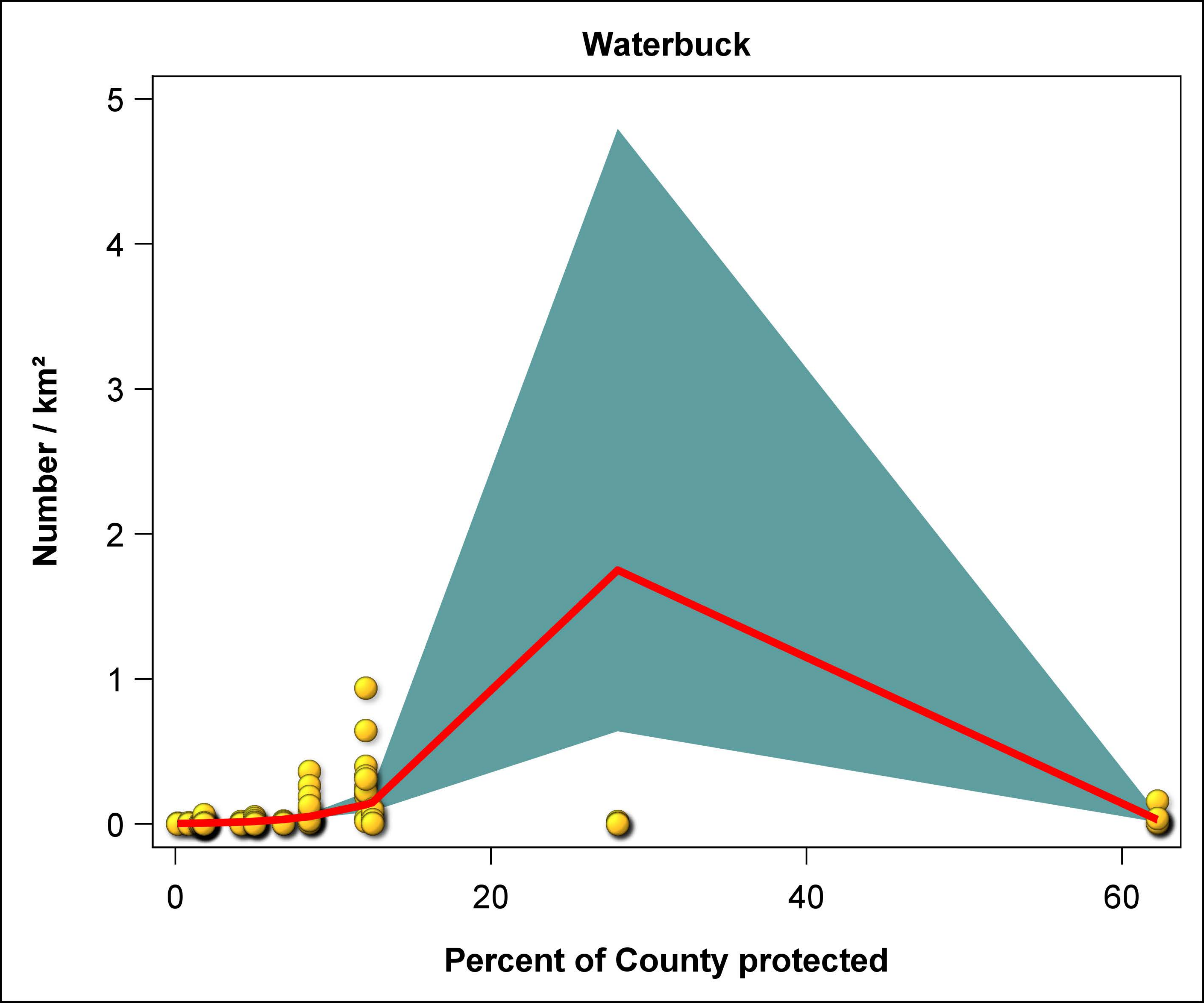

Waterbuck
Number / km²
0
1
2
3
4
5
0
20
40
60
Percent of County protected
