# Supplementary material for: Extreme Wildlife Declines and Concurrent Increase in Livestock Numbers in Kenya: What Are the Causes?
Source: PLoS One. 2016 Sep 27;11(9):e0163249. doi: 10.1371/journal.pone.0163249 (PMC5039022; doi:10.1371/journal.pone.0163249)
Supplement: S29 Fig — The filled circles are the observations, the solid lines are the quadratic regression lines while the shaded bands are the 95% pointwise confidence bands. (PDF) [file pone.0163249.s039.pdf]

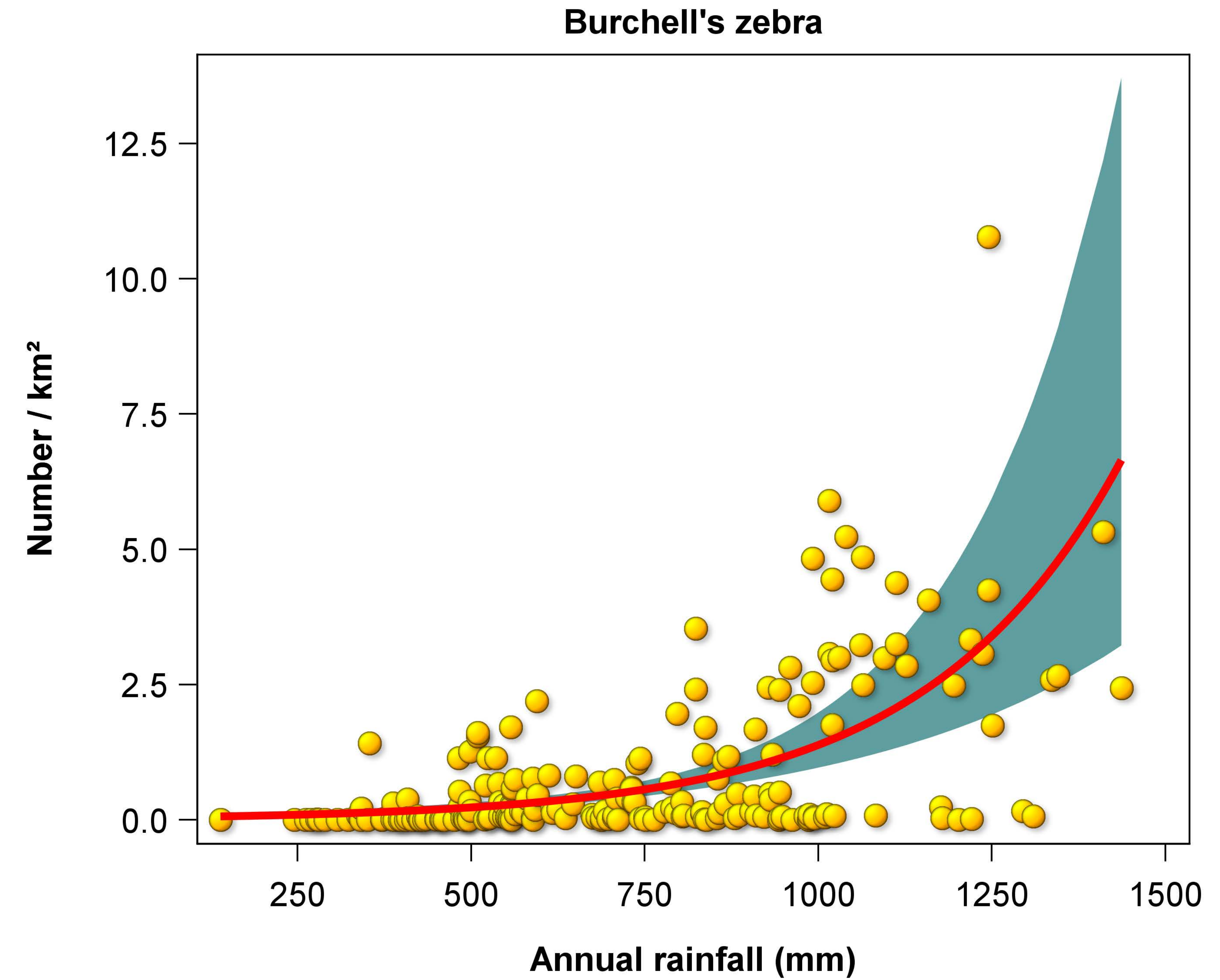

Burchell's zebra
Number / km²
12.5
10.0
7.5
5.0
2.5
0.0
250
500
750
1000
1250
1500
Annual rainfall (mm)

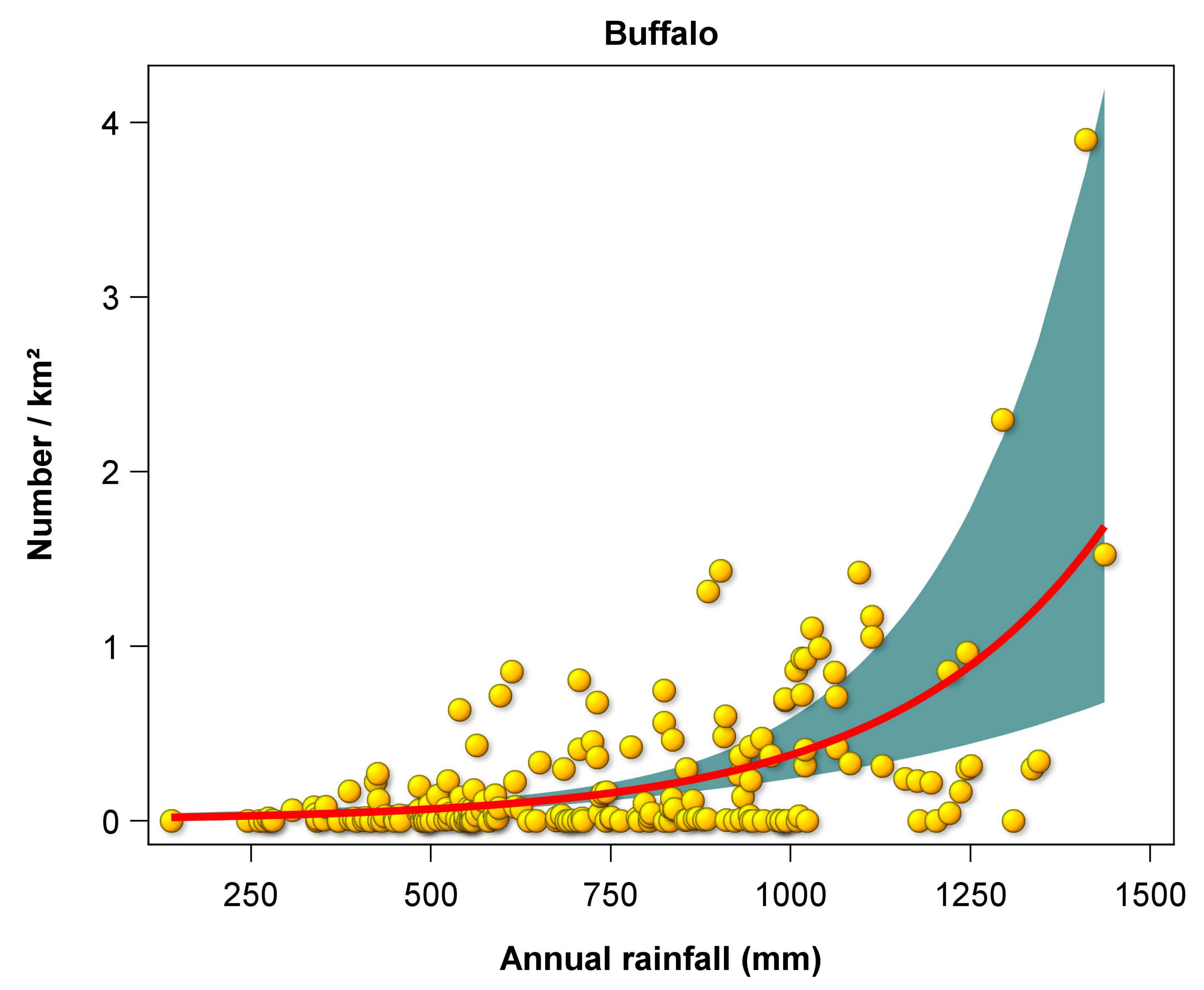

Buffalo
Number / km²
0
1
2
3
4
250
500
750
1000
1250
1500
Annual rainfall (mm)

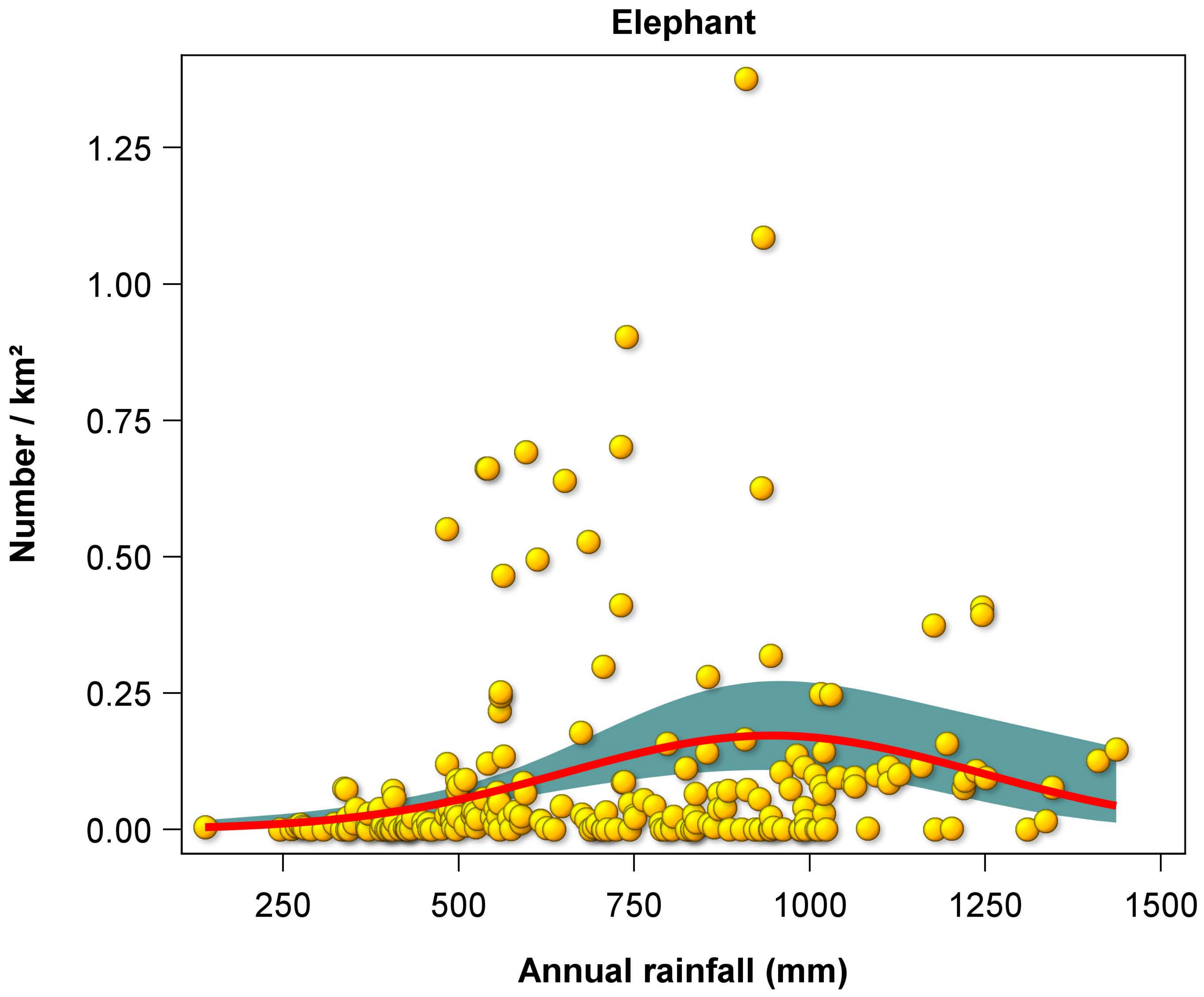

Elephant
Number / km²
Annual rainfall (mm)
0.00
0.25
0.50
0.75
1.00
1.25
250
500
750
1000
1250
1500

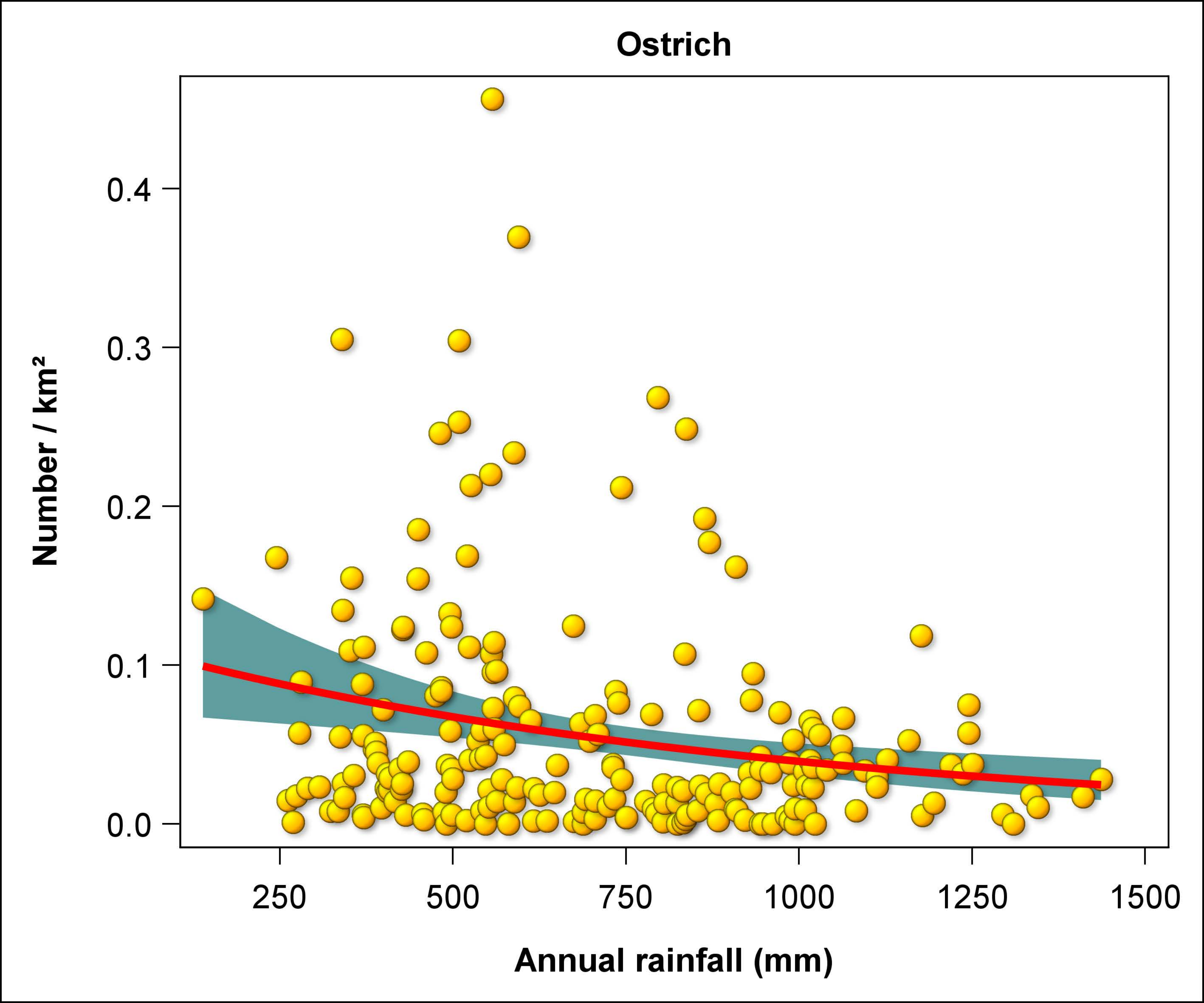
Ostrich
Number / km²
0.0
0.1
0.2
0.3
0.4
250
500
750
1000
1250
1500
Annual rainfall (mm)

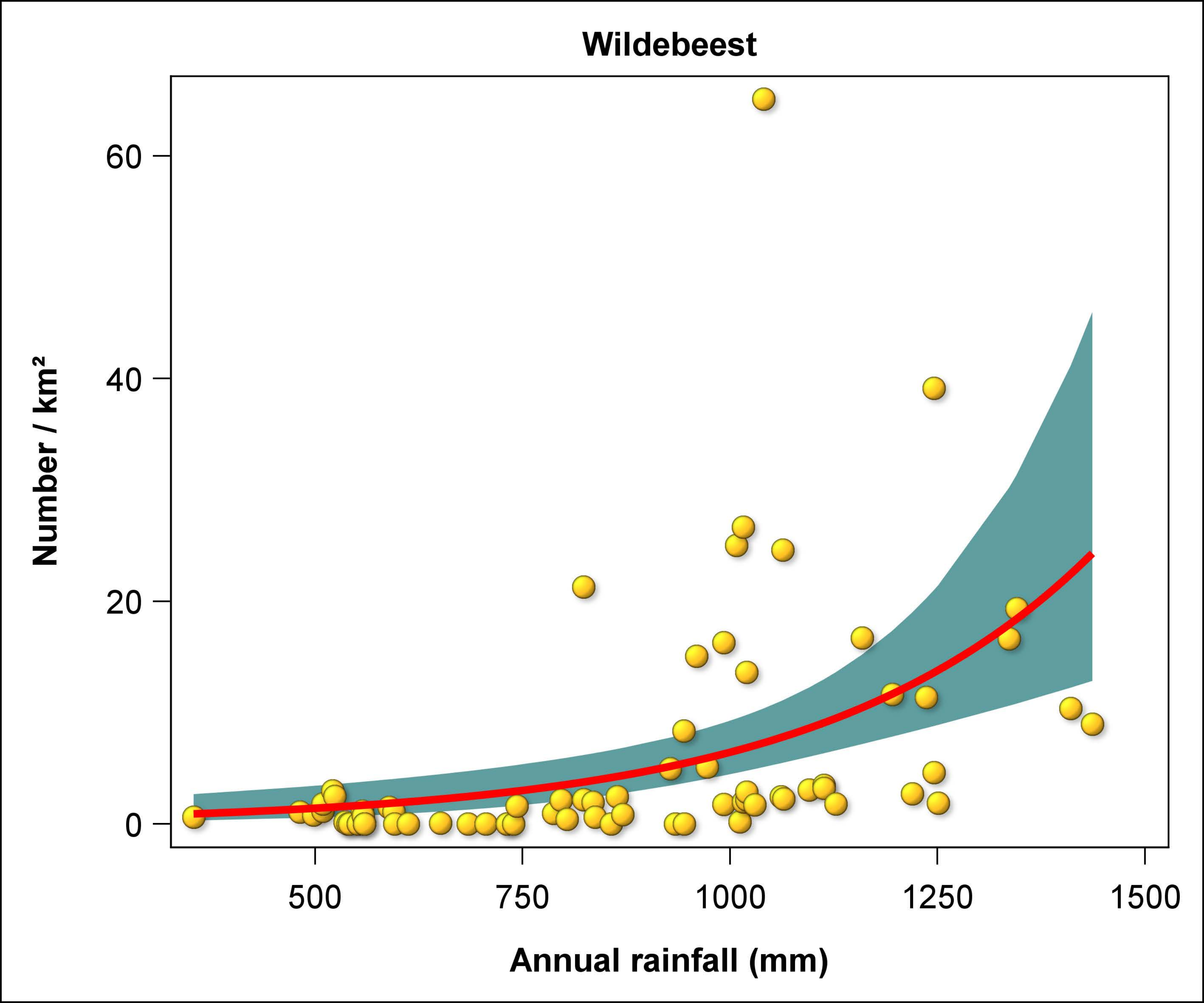
Wildebeest
Number / km²
0
20
40
60
500
750
1000
1250
1500
Annual rainfall (mm)

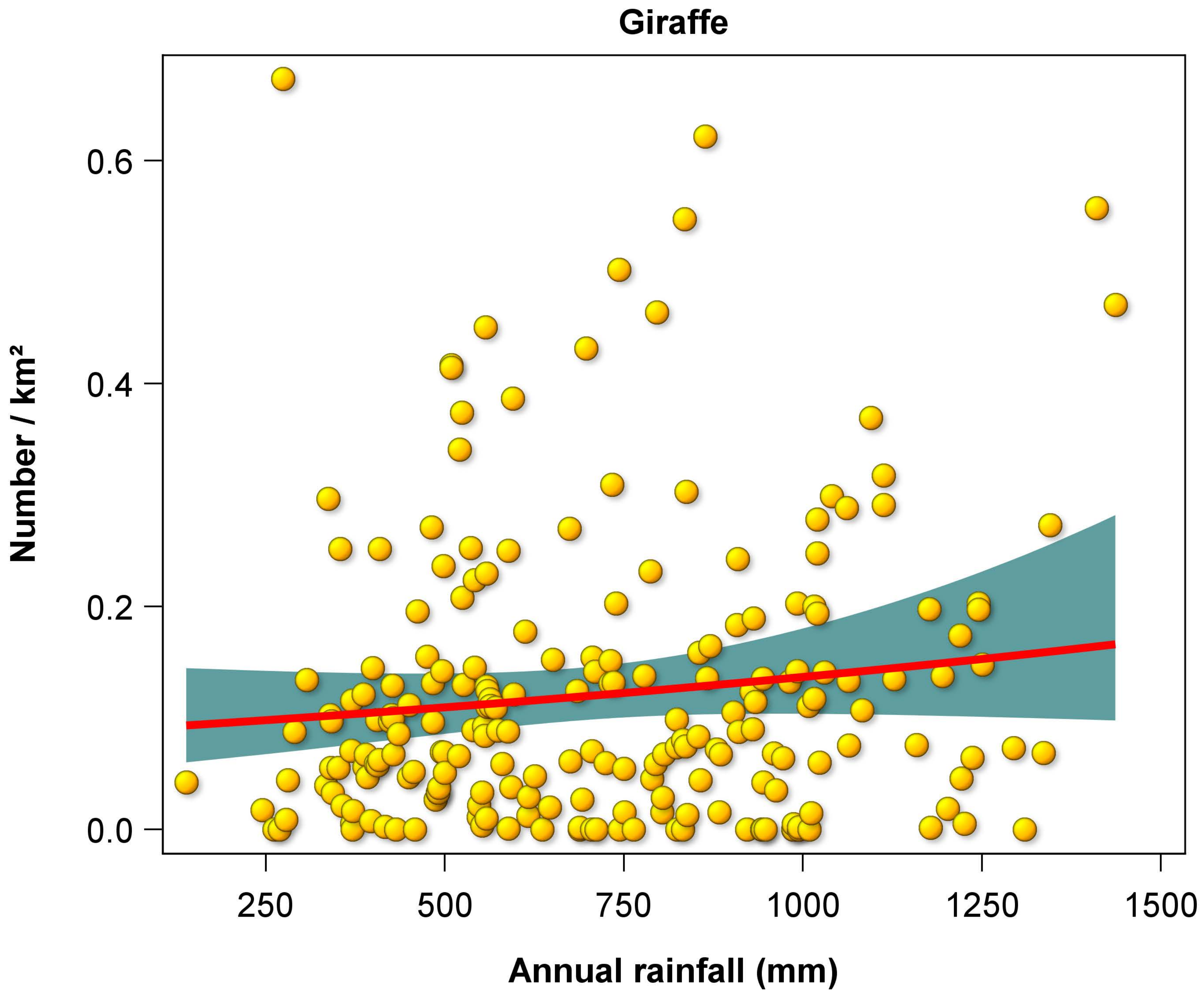
Giraffe
Number / km²
0.0
0.2
0.4
0.6
250
500
750
1000
1250
1500
Annual rainfall (mm)

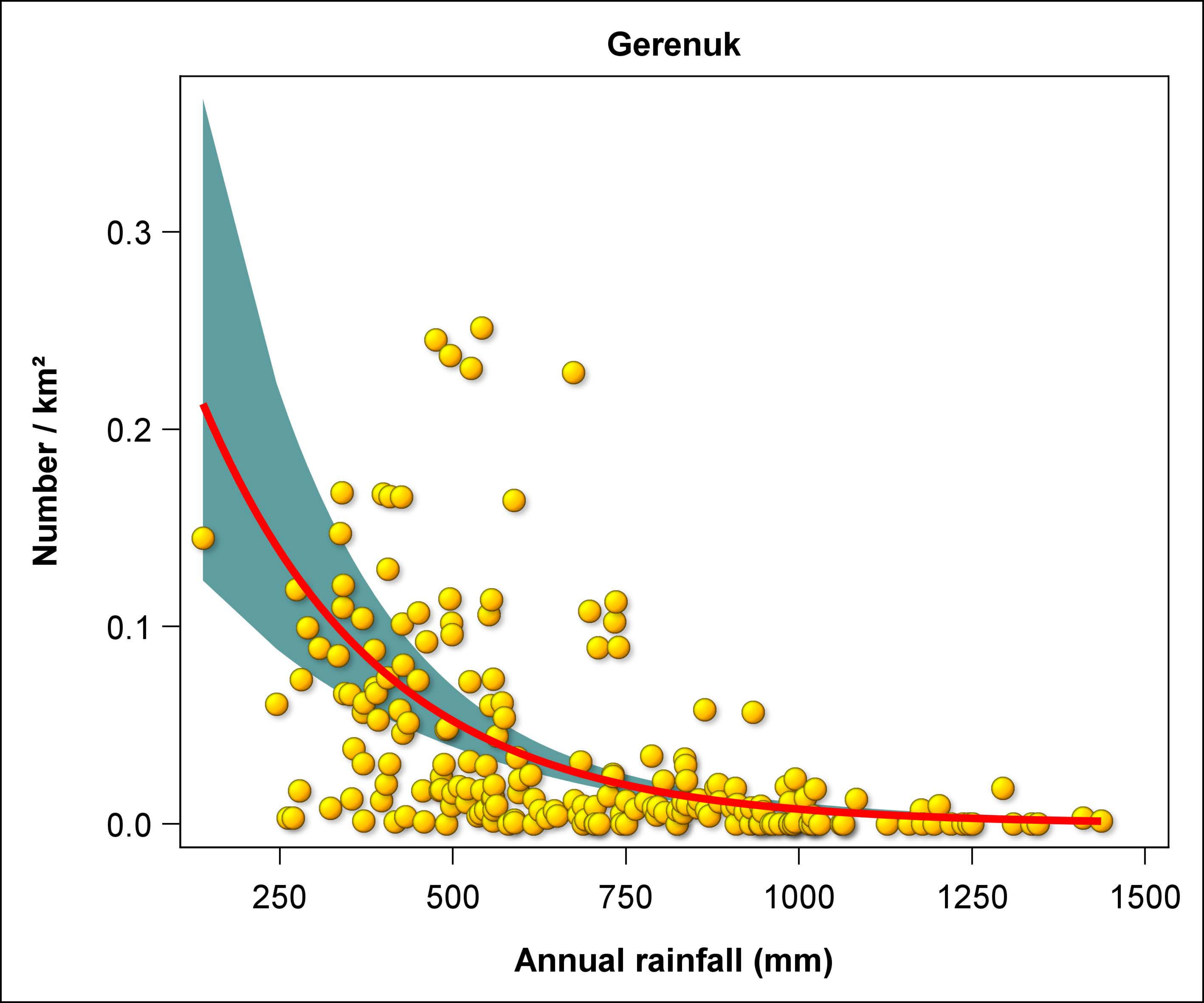

Gerenuk
Number / km²
0.0
0.1
0.2
0.3
250
500
750
1000
1250
1500
Annual rainfall (mm)

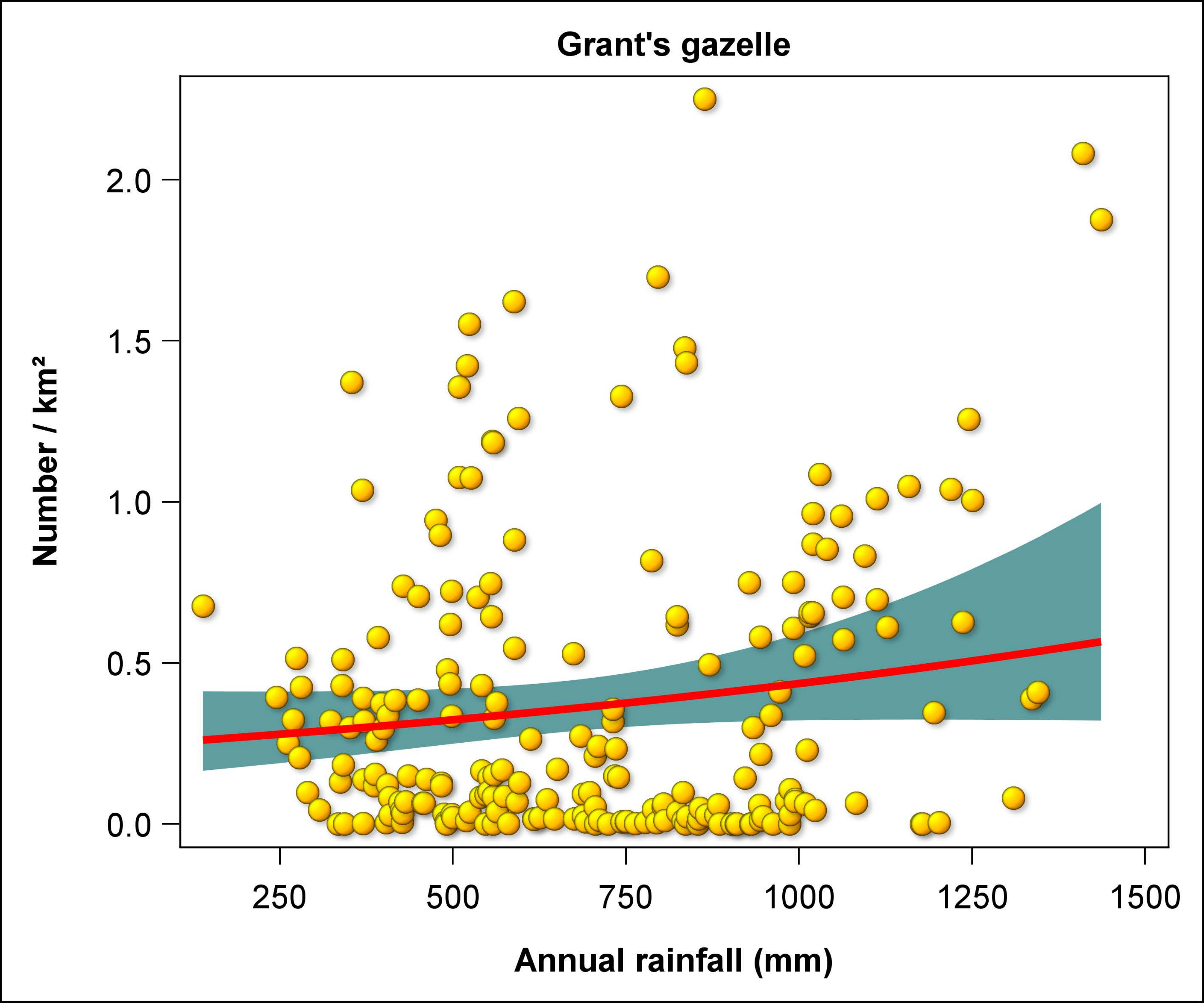

Grant's gazelle
Number / km²
2.0
1.5
1.0
0.5
0.0
250
500
750
1000
1250
1500
Annual rainfall (mm)

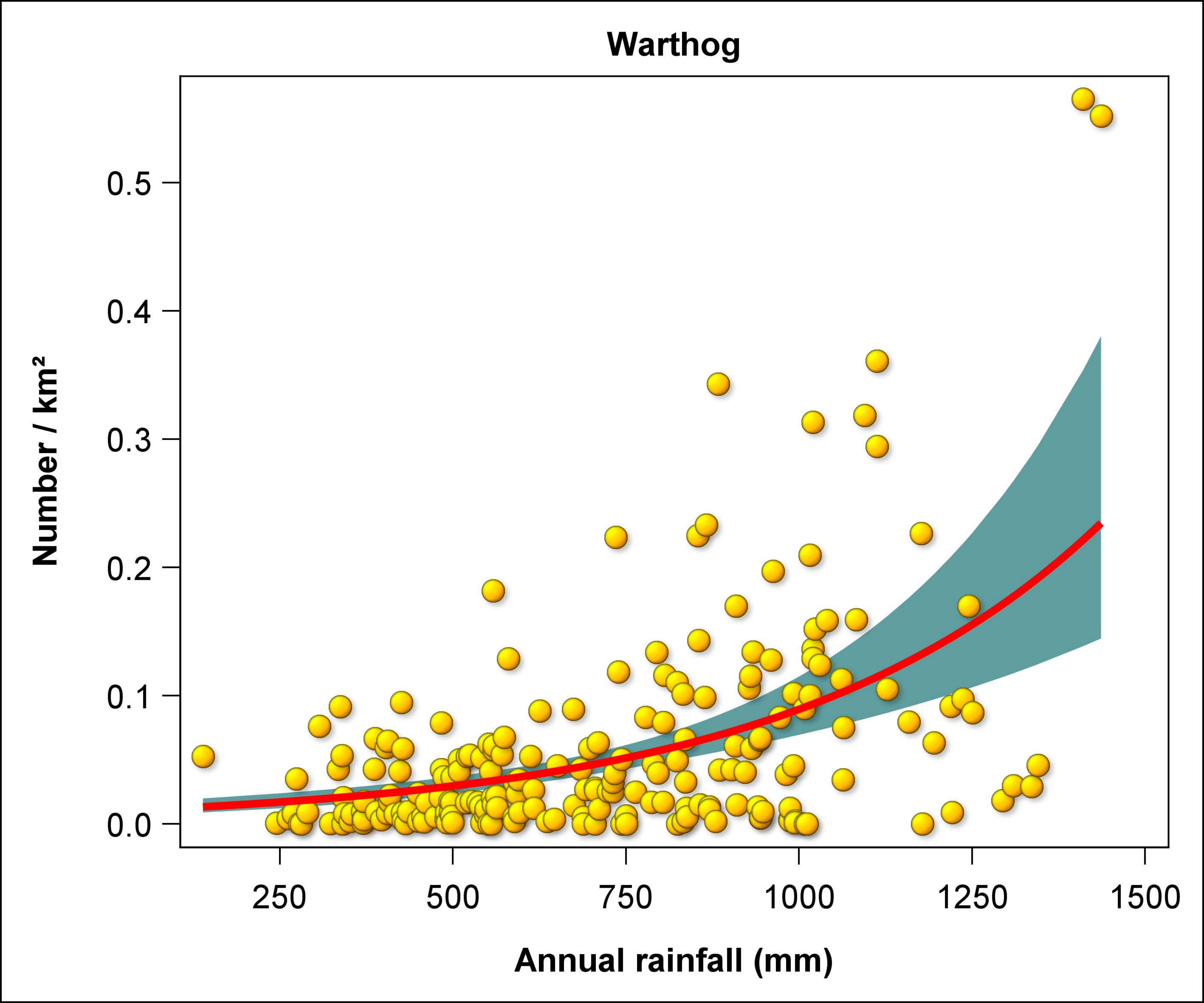

Warthog
Number / km²
0.0
0.1
0.2
0.3
0.4
0.5
250
500
750
1000
1250
1500
Annual rainfall (mm)

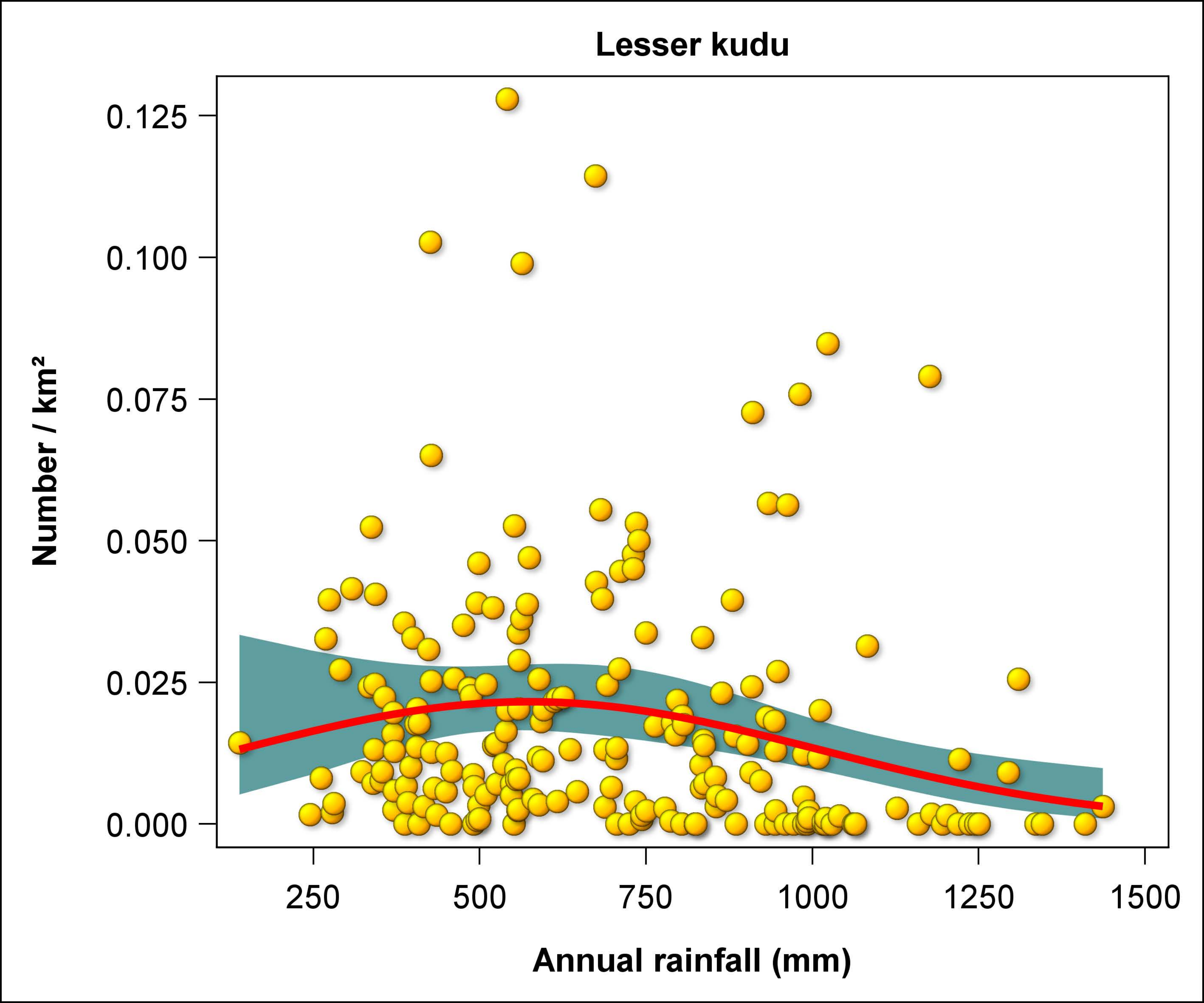

Lesser kudu
Number / km²
0.125
0.100
0.075
0.050
0.025
0.000
250
500
750
1000
1250
1500
Annual rainfall (mm)

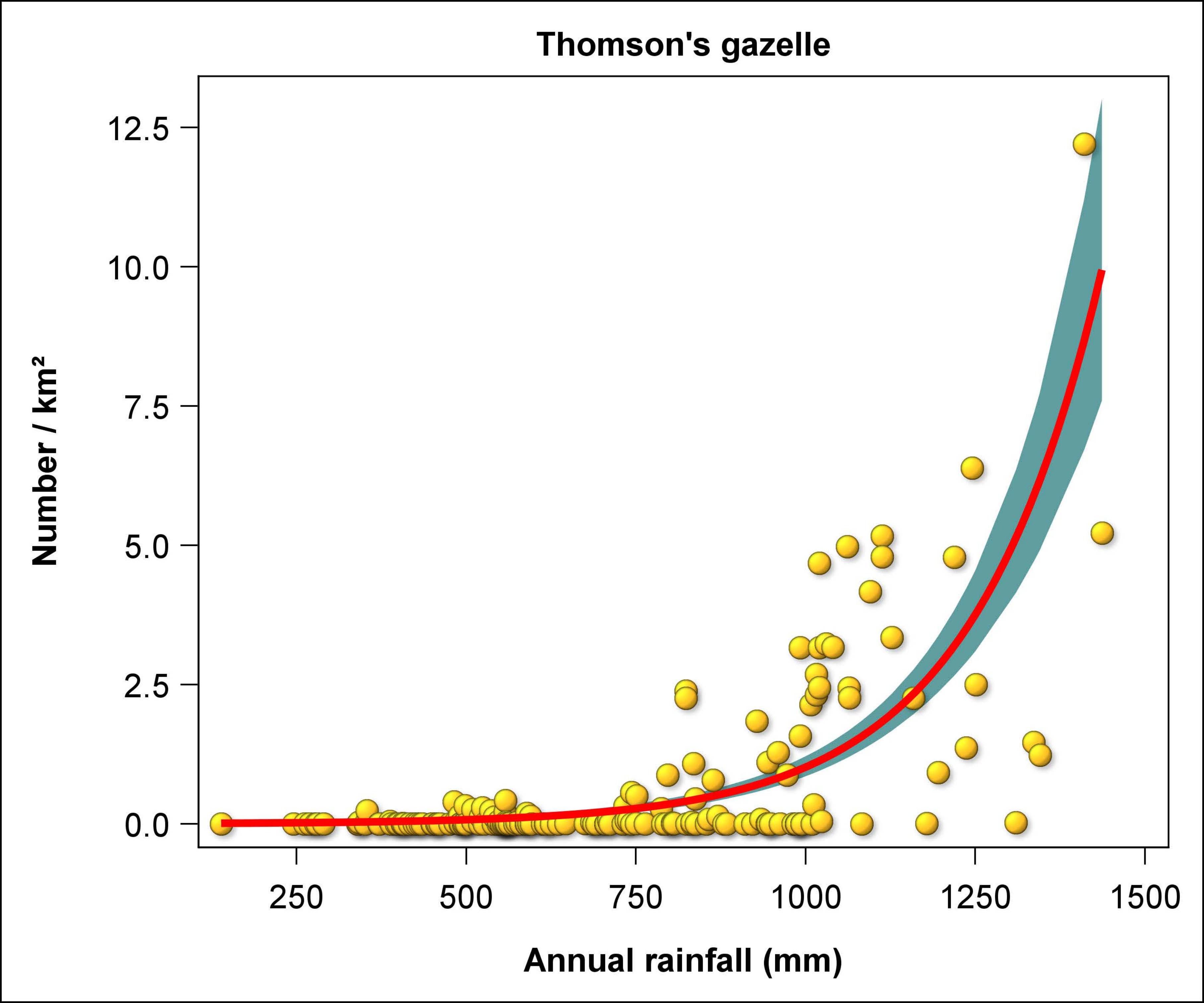

Thomson's gazelle
12.5
10.0
7.5
5.0
2.5
0.0
Number / km²
250
500
750
1000
1250
1500
Annual rainfall (mm)

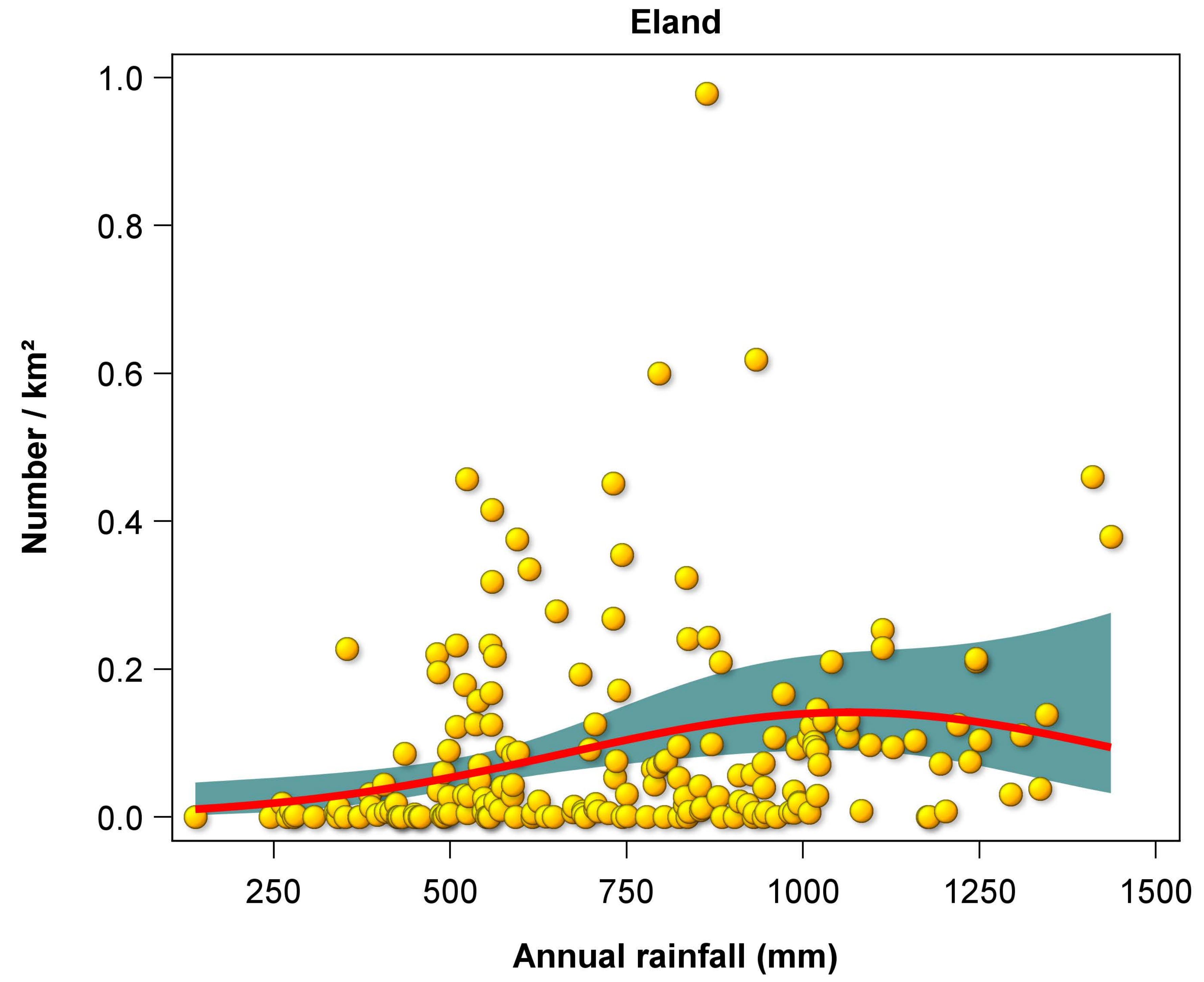
Eland
Number / km²
Annual rainfall (mm)
1.0
0.8
0.6
0.4
0.2
0.0
250
500
750
1000
1250
1500

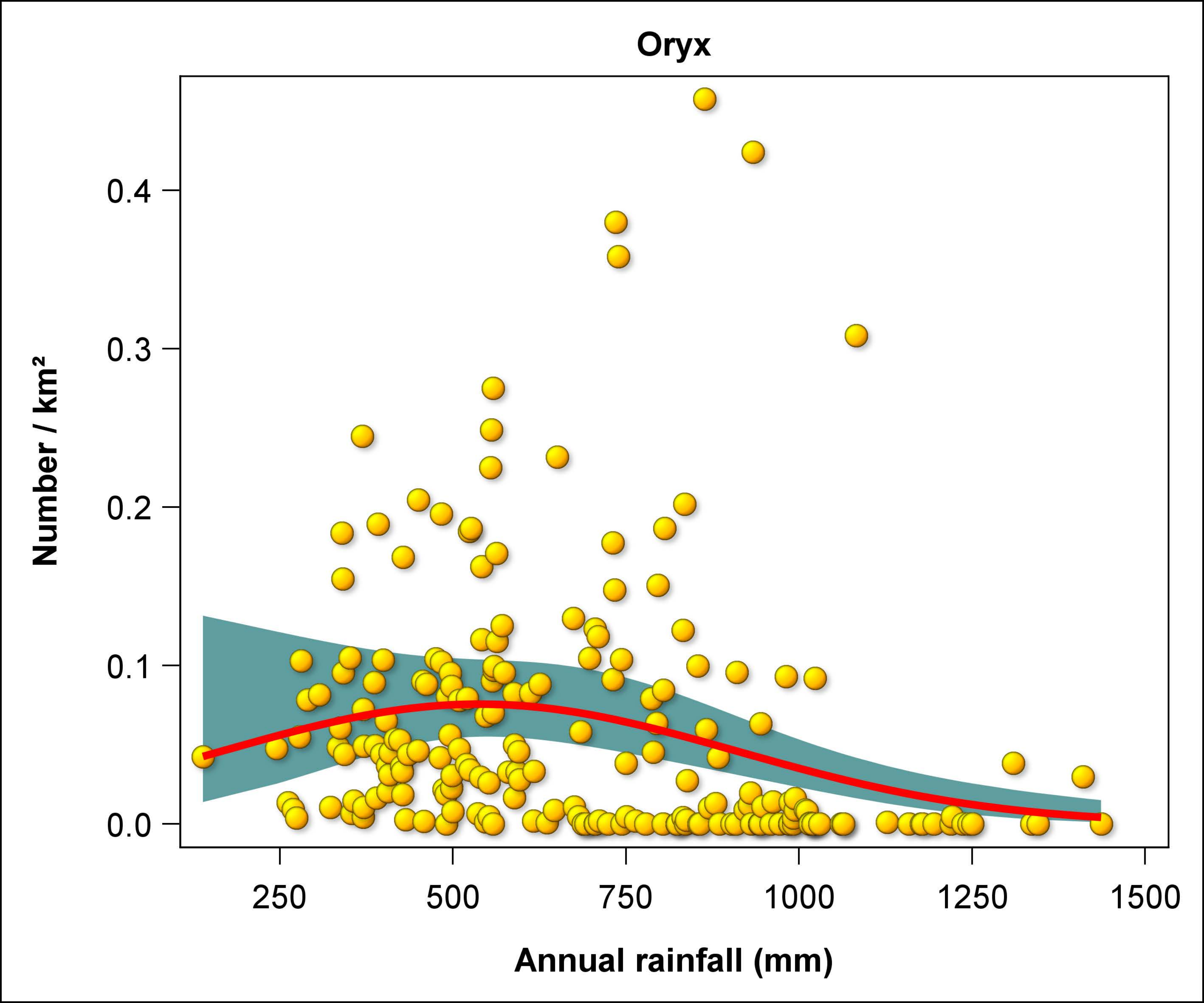

Oryx
Number / km²
0.0
0.1
0.2
0.3
0.4
250
500
750
1000
1250
1500
Annual rainfall (mm)

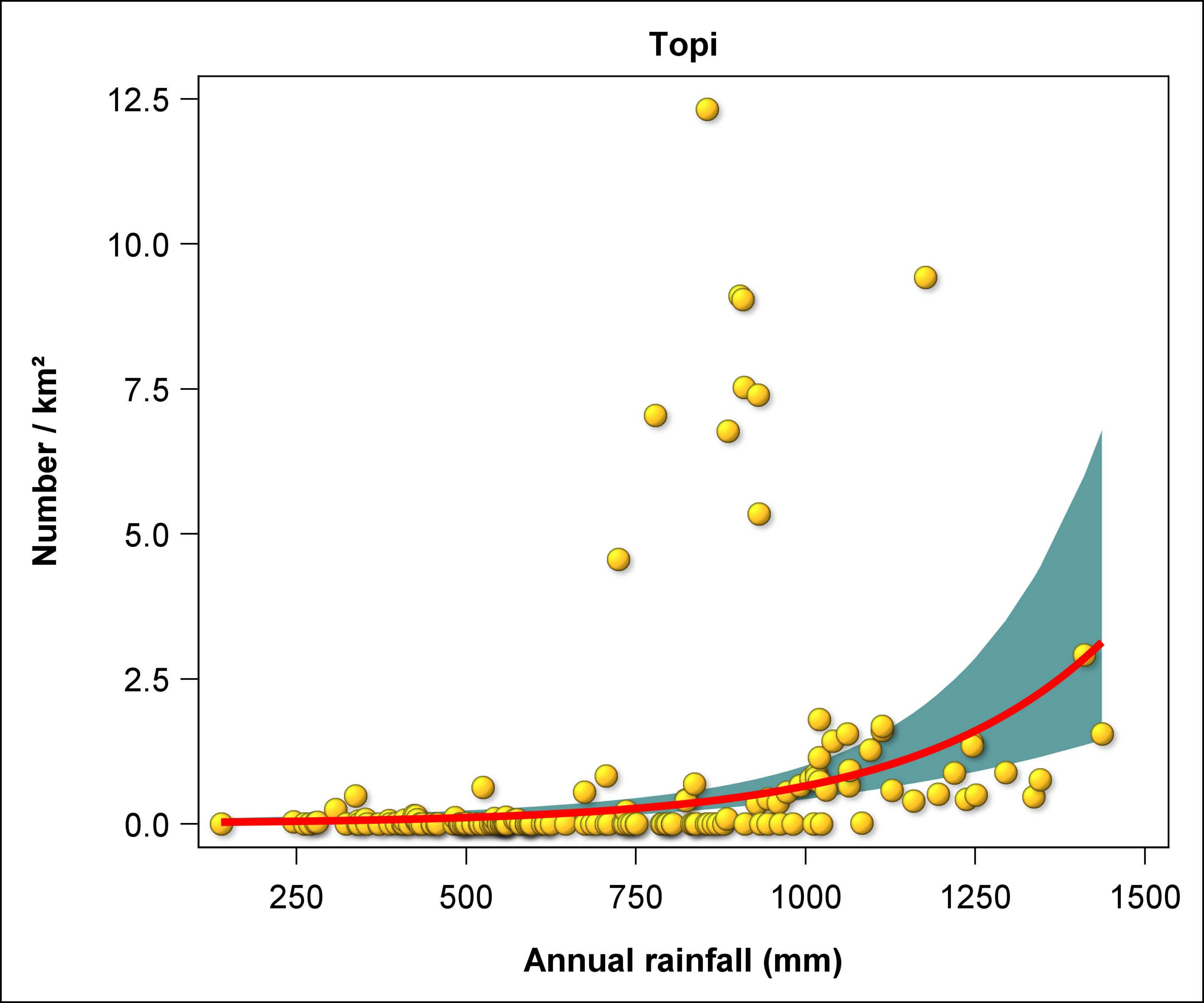

Topi
12.5
10.0
7.5
5.0
2.5
0.0
Number / km²
250
500
750
1000
1250
1500
Annual rainfall (mm)

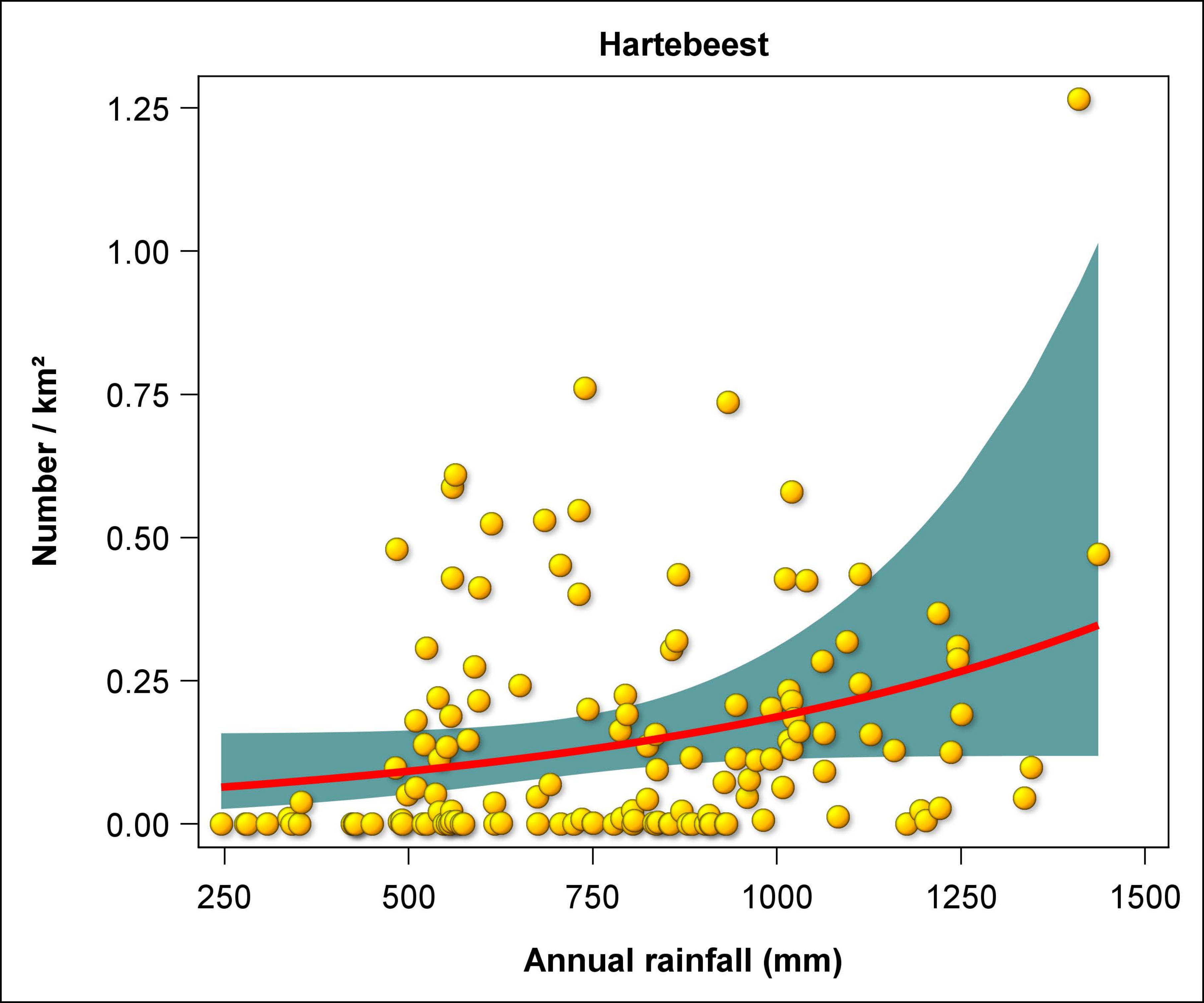

Hartebeest
Number / km²
Annual rainfall (mm)
1.25
1.00
0.75
0.50
0.25
0.00
250
500
750
1000
1250
1500

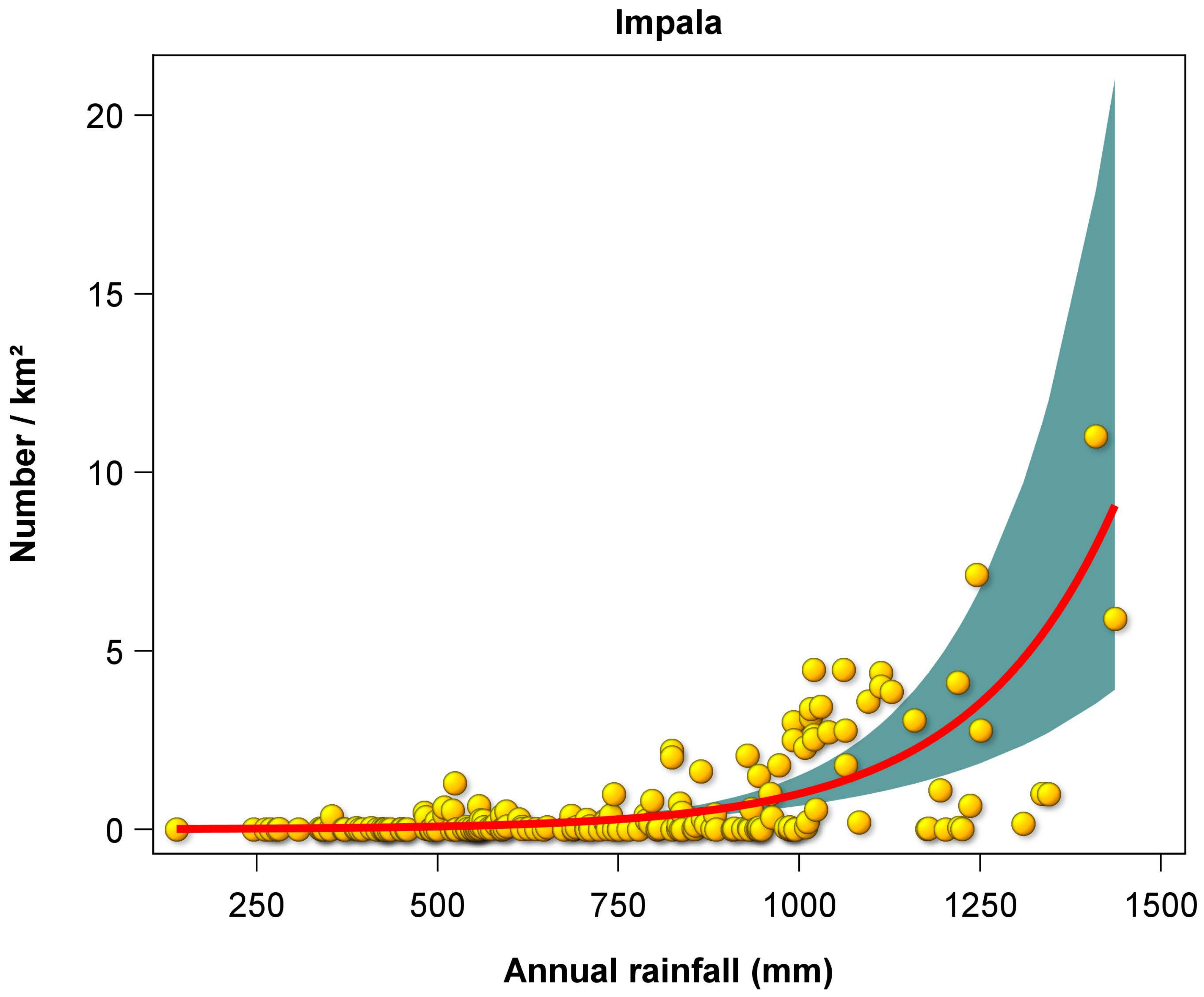
Impala
Number / km²
0
5
10
15
20
250
500
750
1000
1250
1500
Annual rainfall (mm)

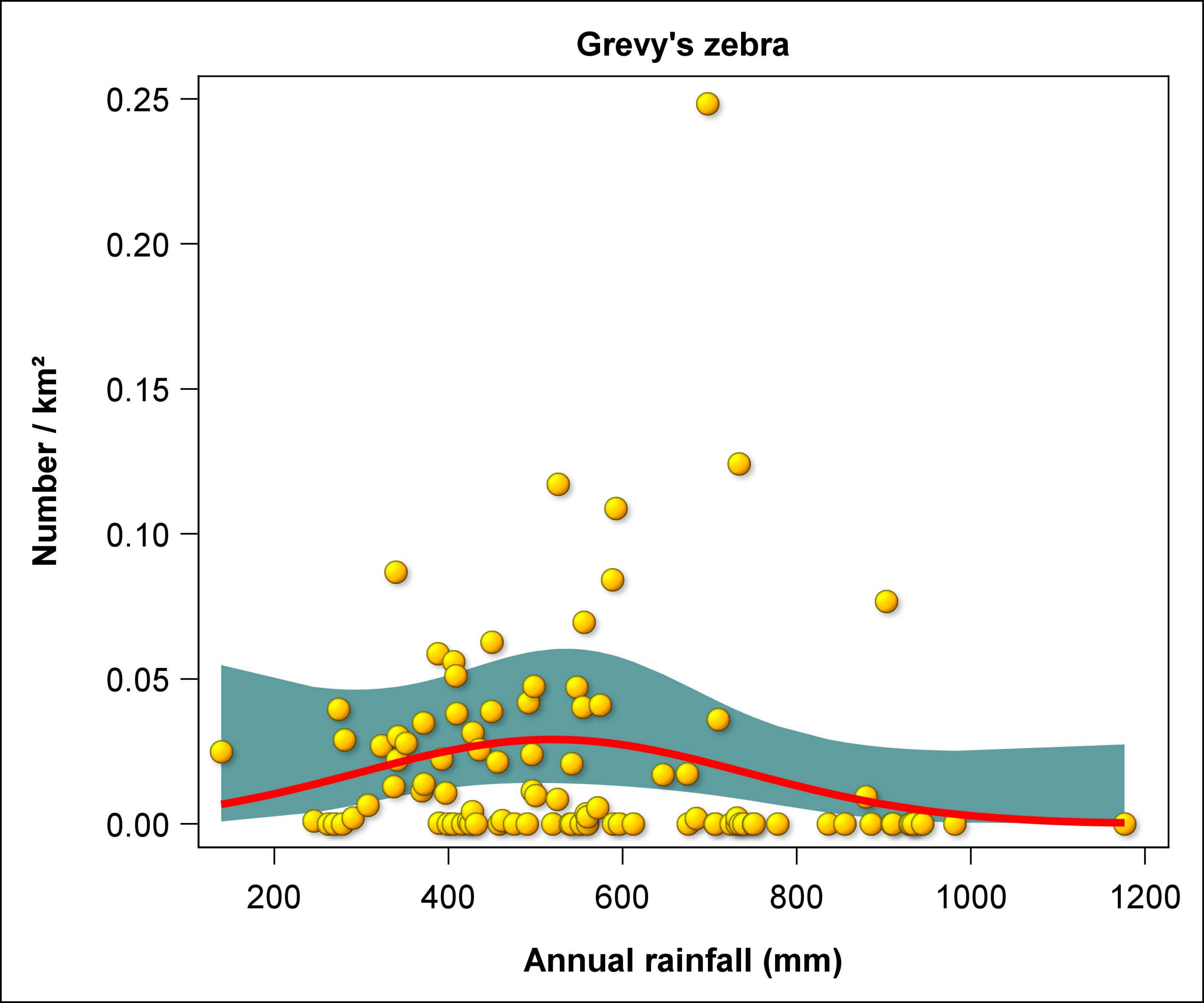

Grevy's zebra
Number / km²
0.25
0.20
0.15
0.10
0.05
0.00
200
400
600
800
1000
1200
Annual rainfall (mm)

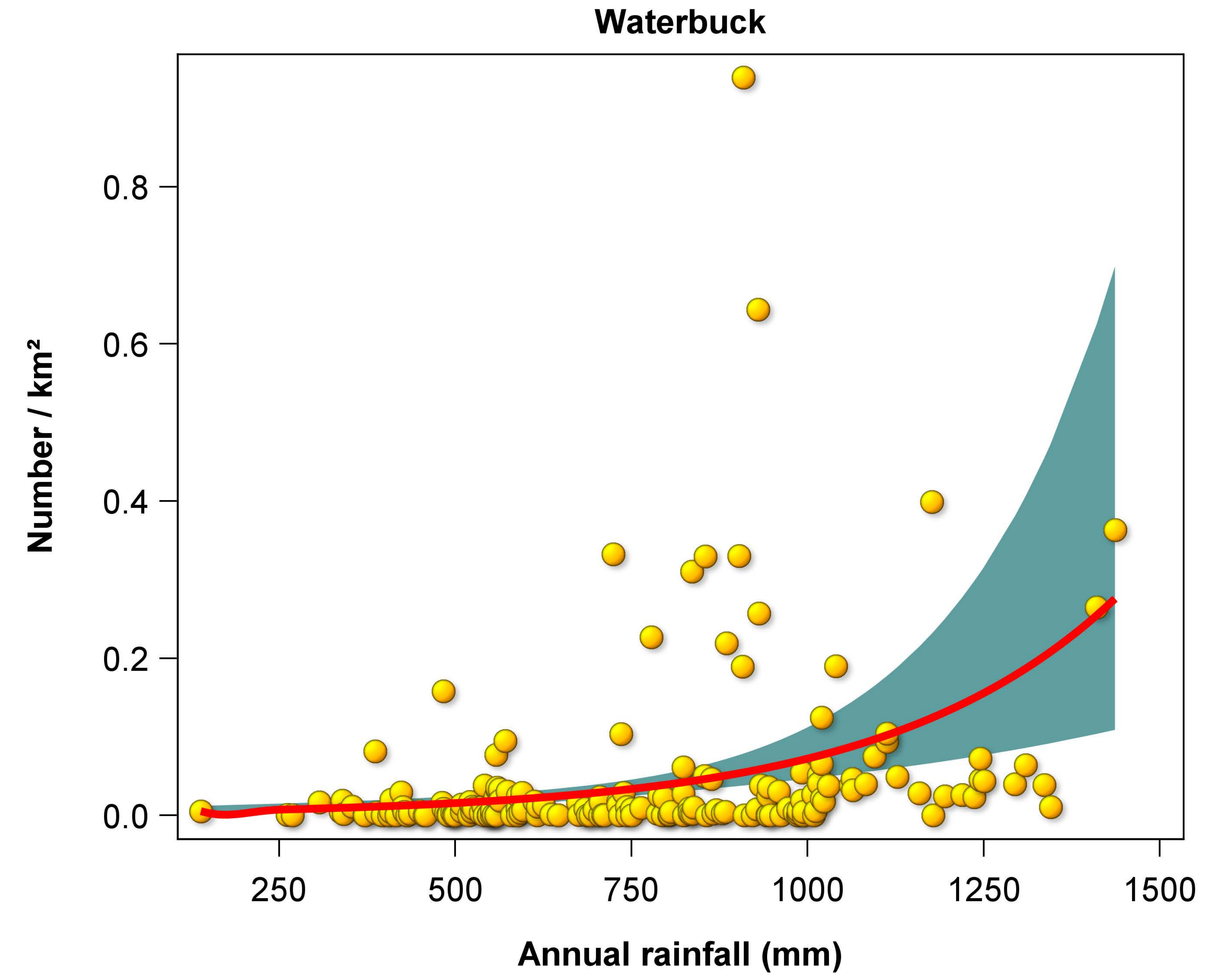

Waterbuck
Number / km²
0.0
0.2
0.4
0.6
0.8
250
500
750
1000
1250
1500
Annual rainfall (mm)
