# Supplementary material for: Extreme Wildlife Declines and Concurrent Increase in Livestock Numbers in Kenya: What Are the Causes?
Source: PLoS One. 2016 Sep 27;11(9):e0163249. doi: 10.1371/journal.pone.0163249 (PMC5039022; doi:10.1371/journal.pone.0163249)
Supplement: S30 Fig — The filled circles are the observations, the solid lines are the quadratic regression lines while the shaded bands are the 95% pointwise confidence bands. (PDF) [file pone.0163249.s040.pdf]

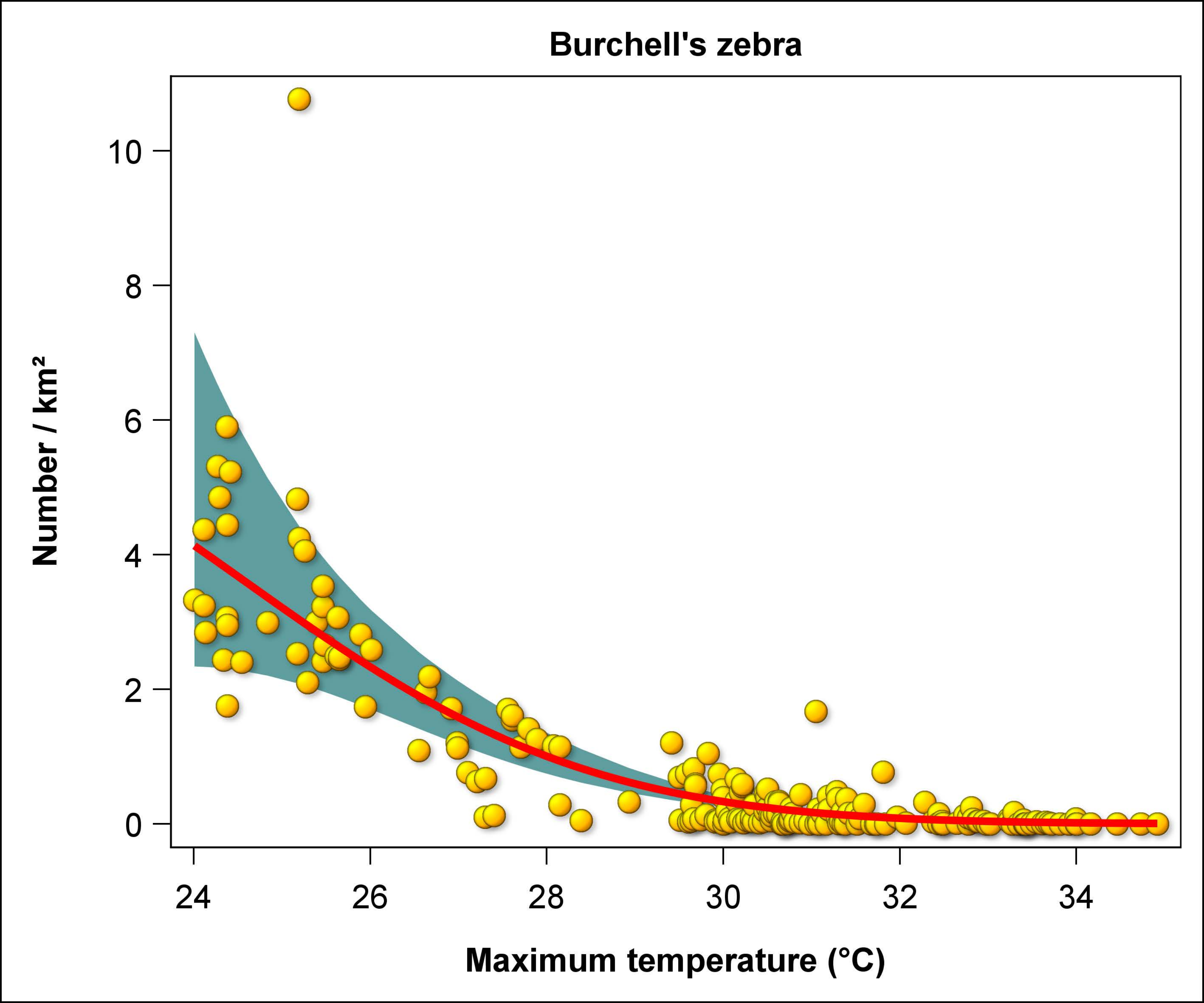

Burchell's zebra
Number / km²
Maximum temperature (°C)
0
2
4
6
8
10
24
26
28
30
32
34

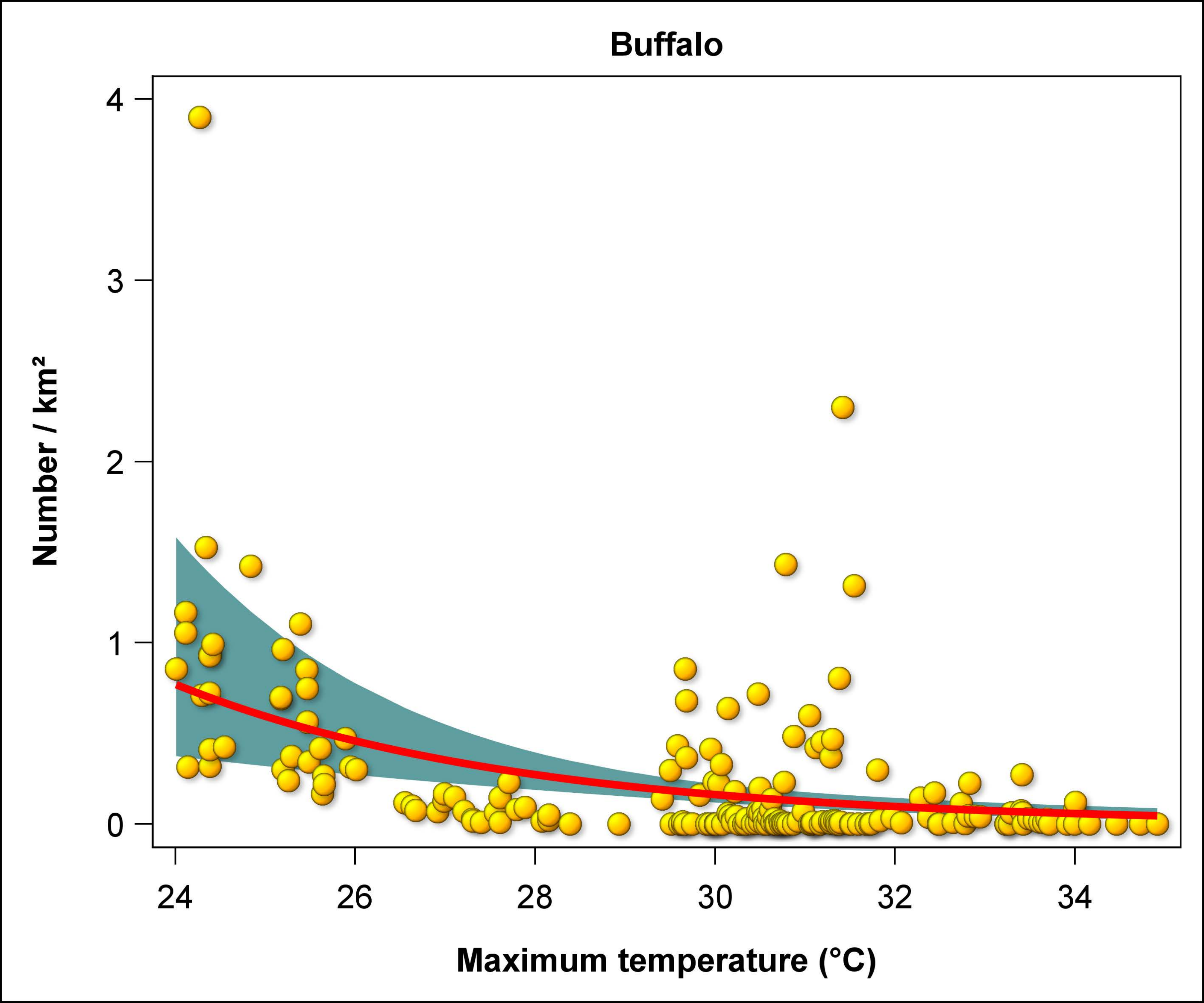

Buffalo
Number / km²
0
1
2
3
4
24
26
28
30
32
34
Maximum temperature (°C)

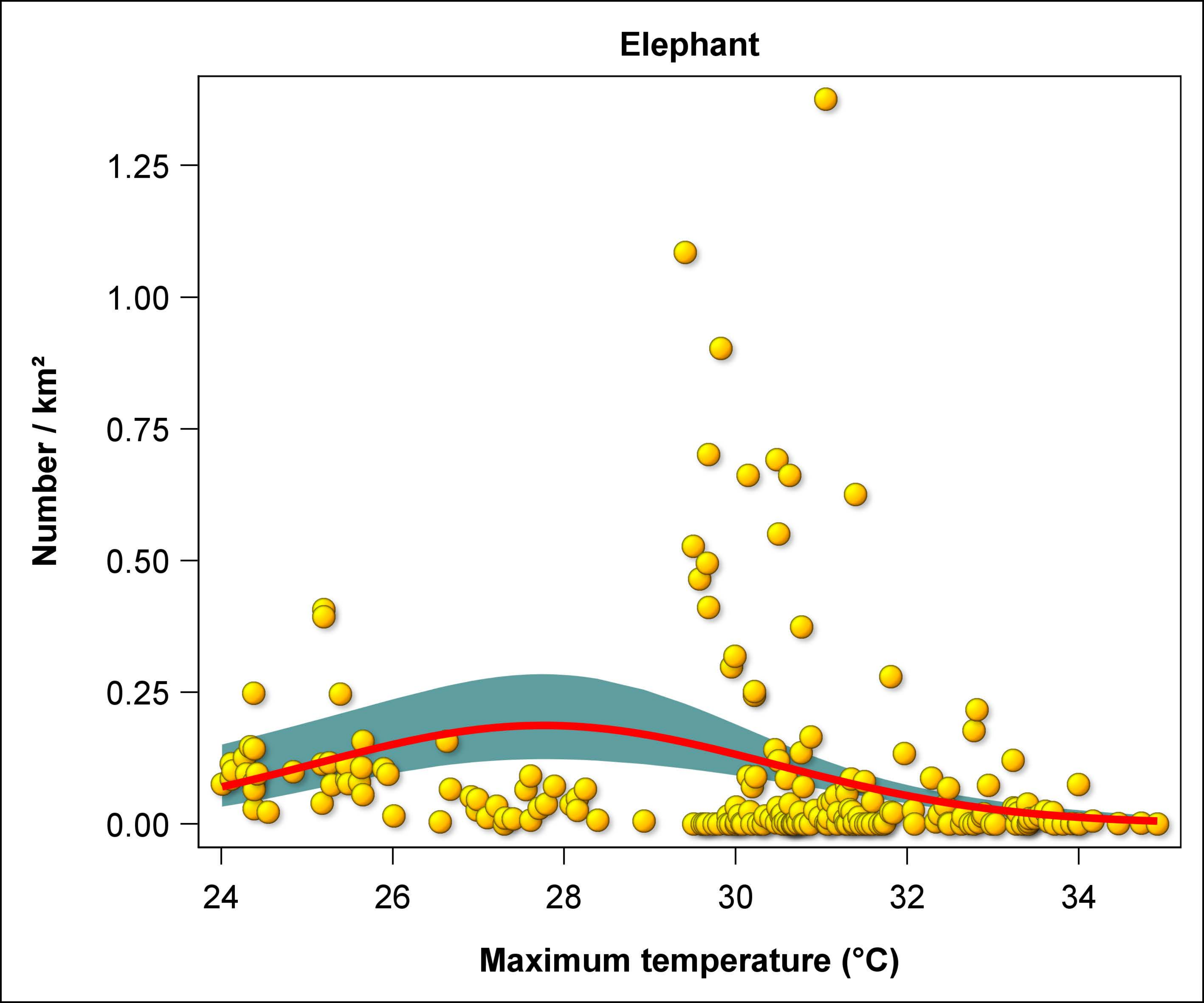
Elephant
Number / km²
1.25
1.00
0.75
0.50
0.25
0.00
24
26
28
30
32
34
Maximum temperature (°C)

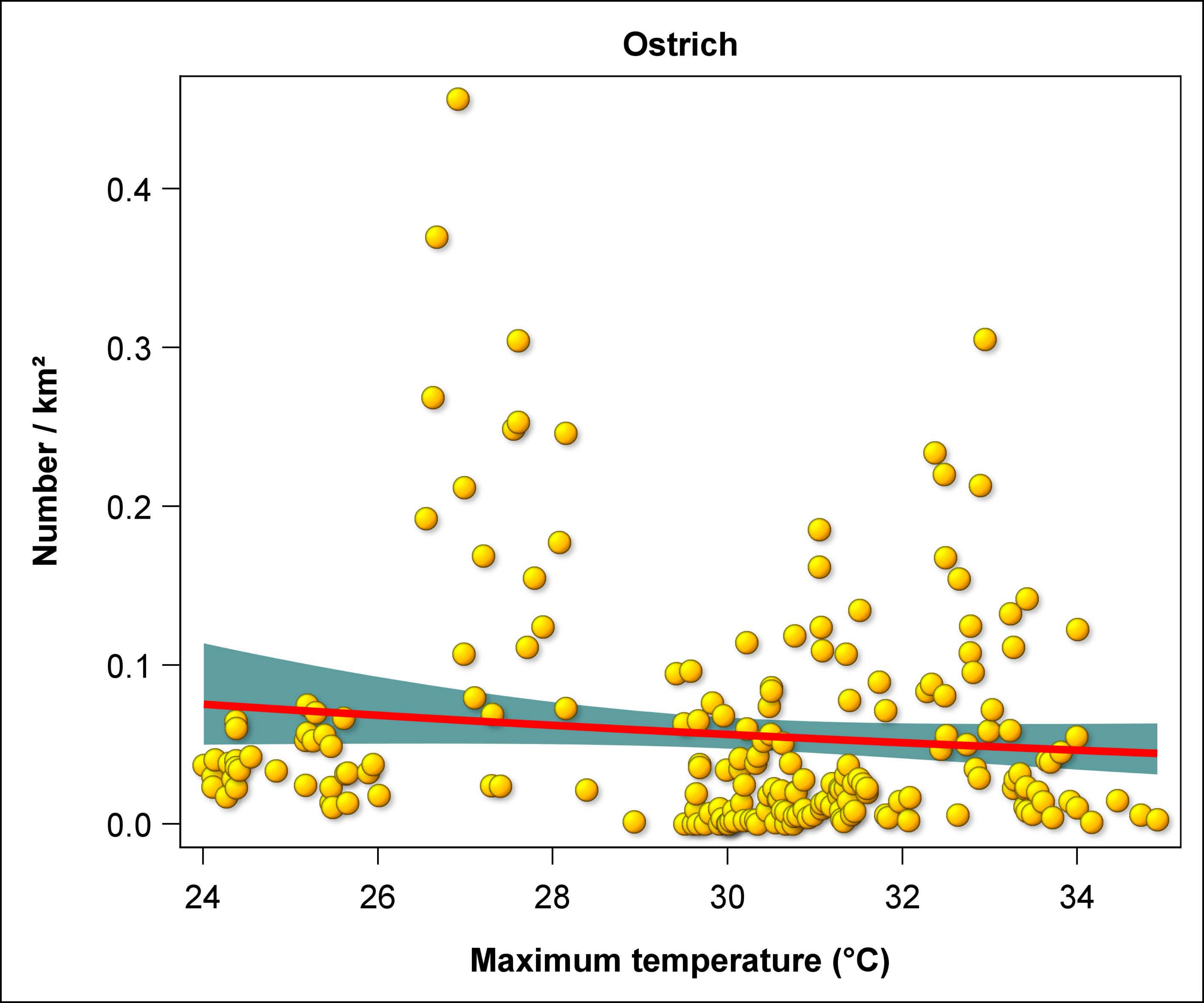

Ostrich
Number / km²
0.0
0.1
0.2
0.3
0.4
24
26
28
30
32
34
Maximum temperature (°C)

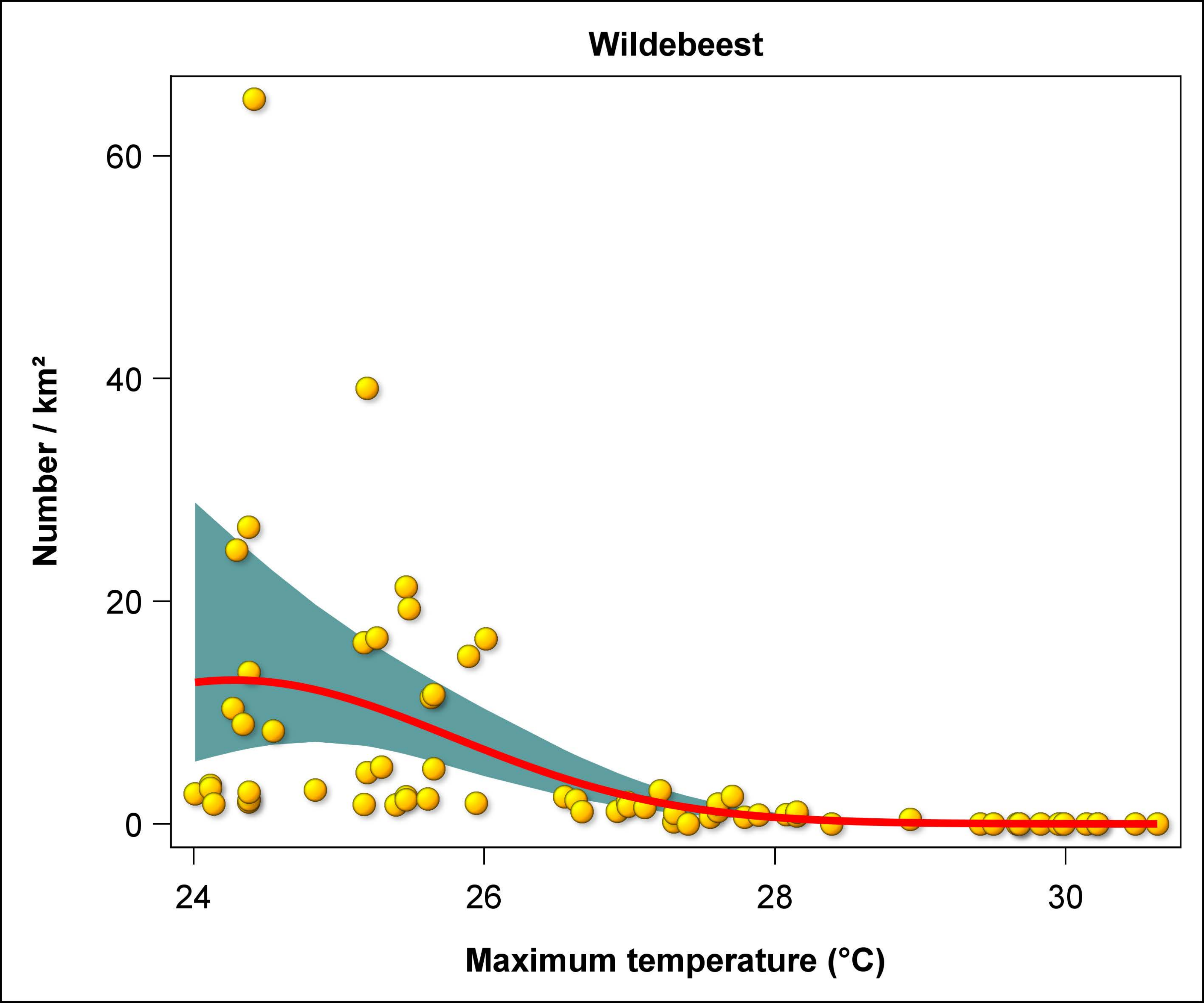

Wildebeest
Number / km²
60
40
20
0
24
26
28
30
Maximum temperature (°C)

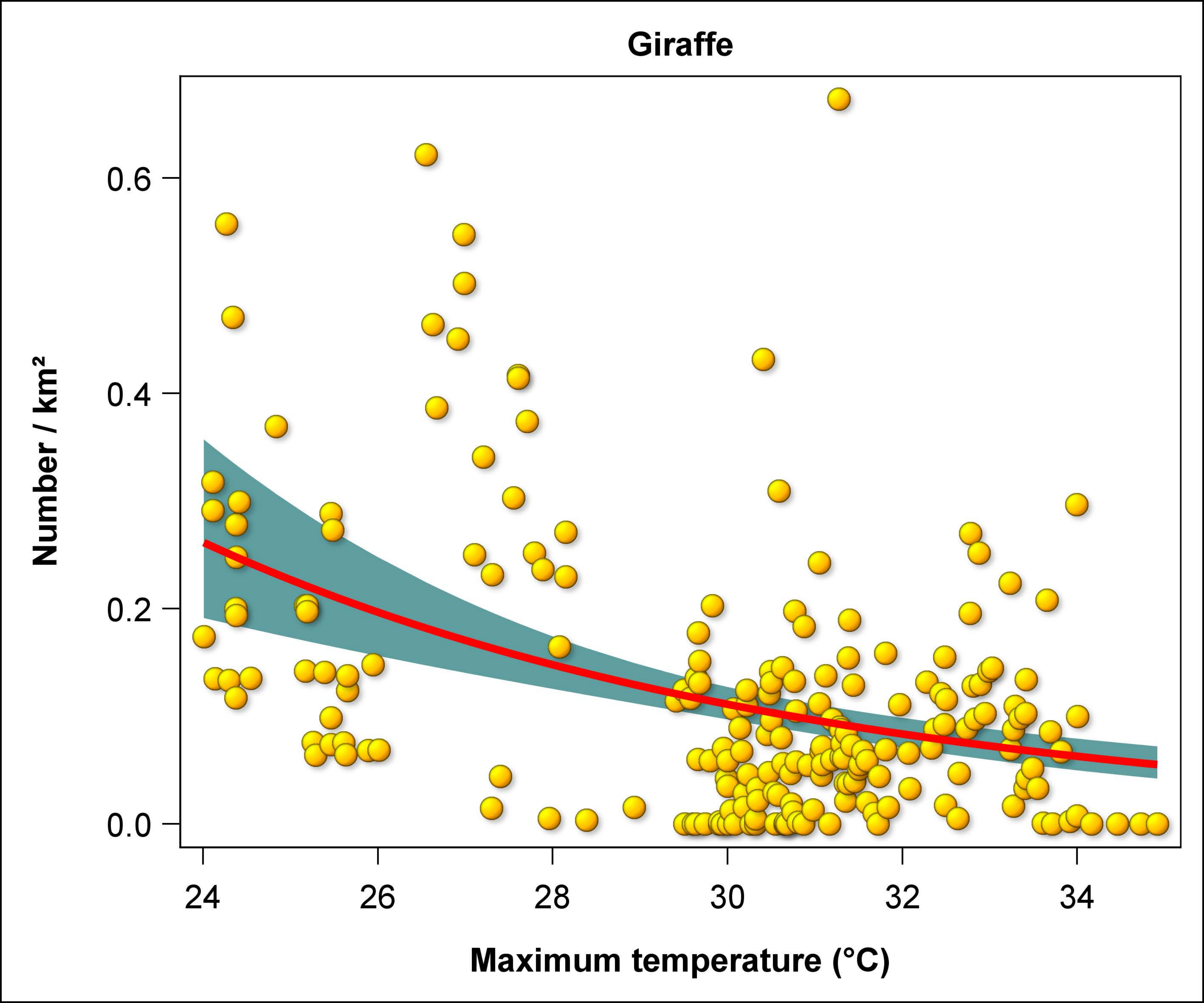

Giraffe
Number / km²
Maximum temperature (°C)
0.0
0.2
0.4
0.6
24
26
28
30
32
34

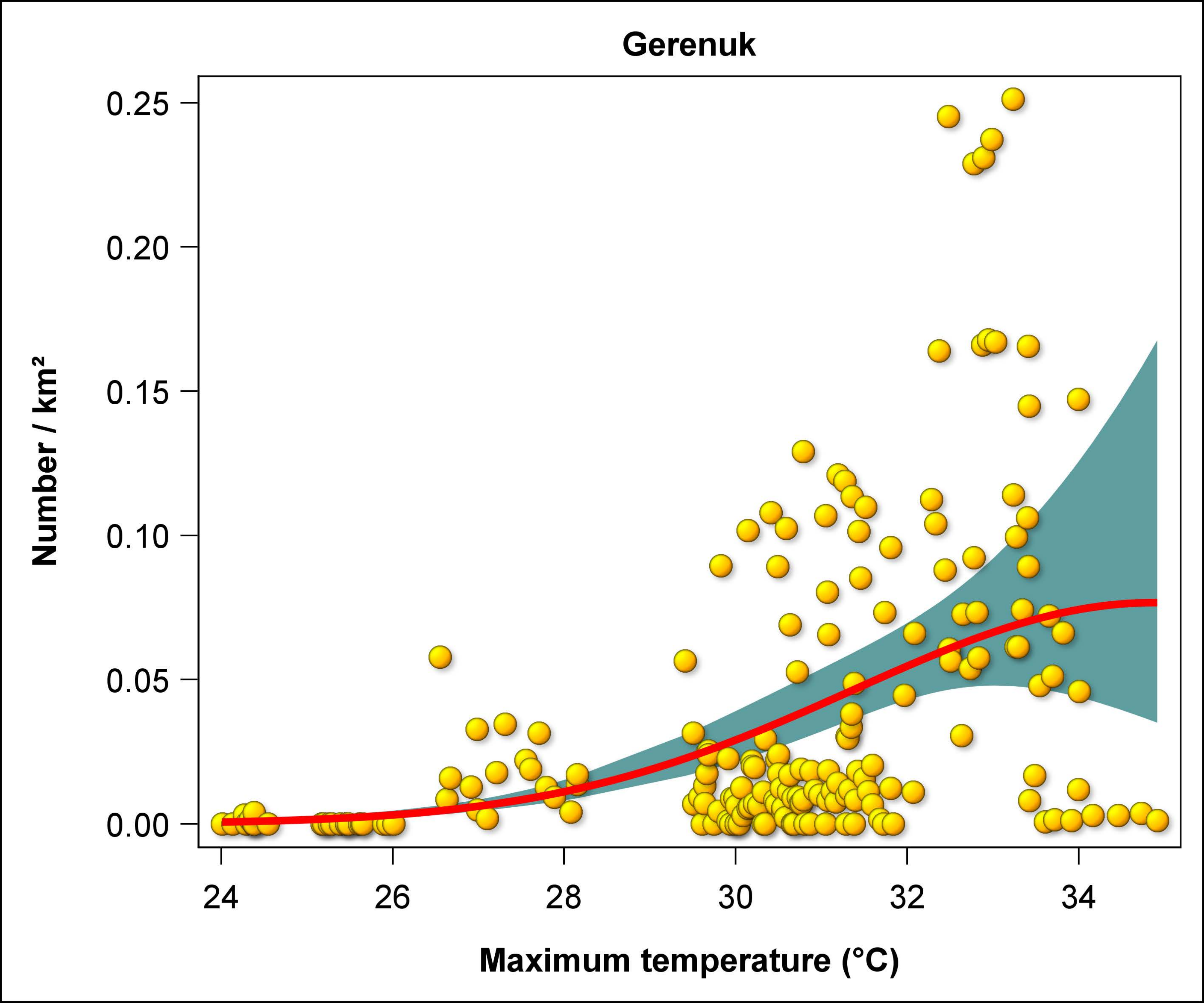

Gerenuk
Number / km²
0.00
0.05
0.10
0.15
0.20
0.25
24
26
28
30
32
34
Maximum temperature (°C)

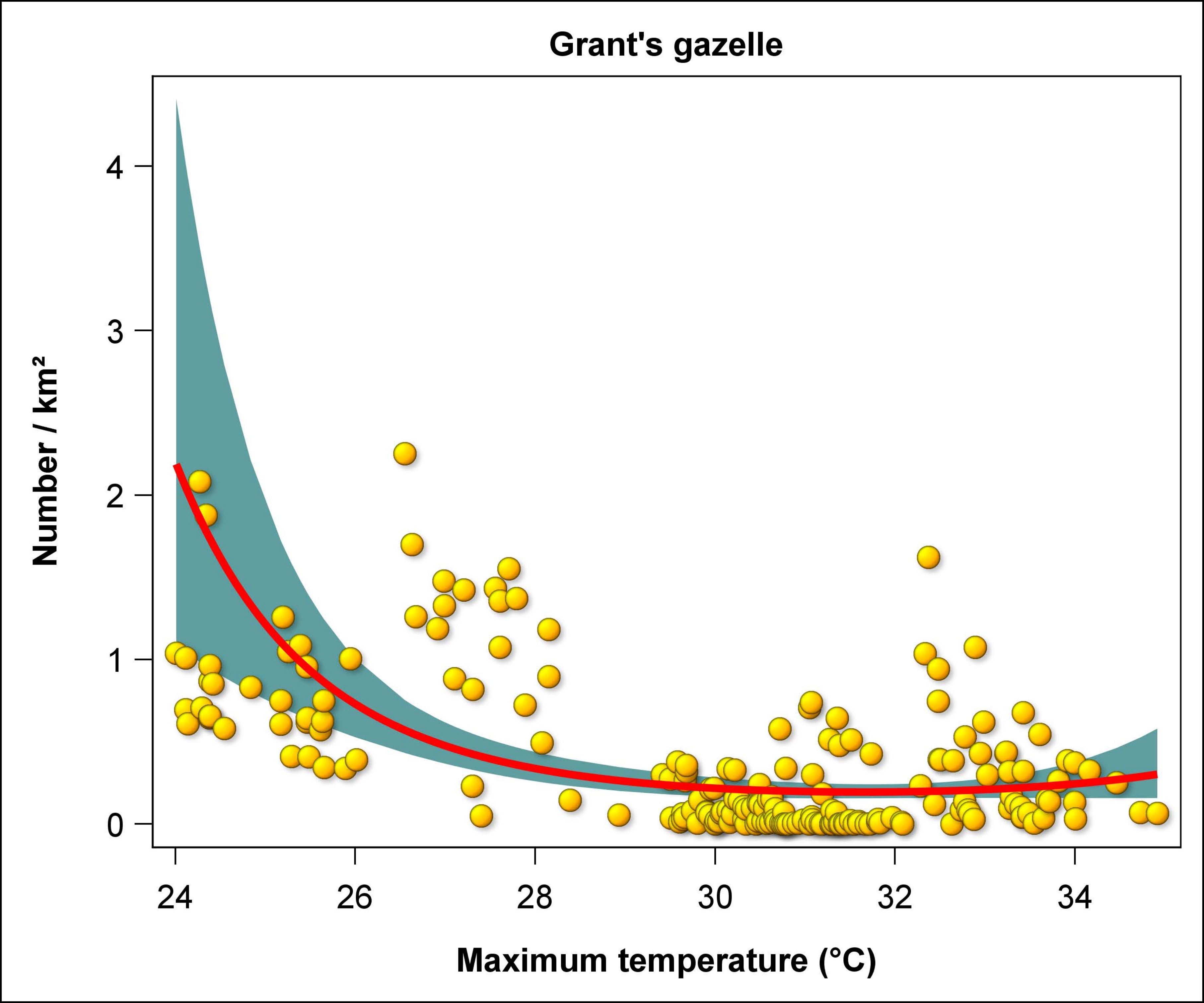

Grant's gazelle
Number / km²
Maximum temperature (°C)
0
1
2
3
4
24
26
28
30
32
34

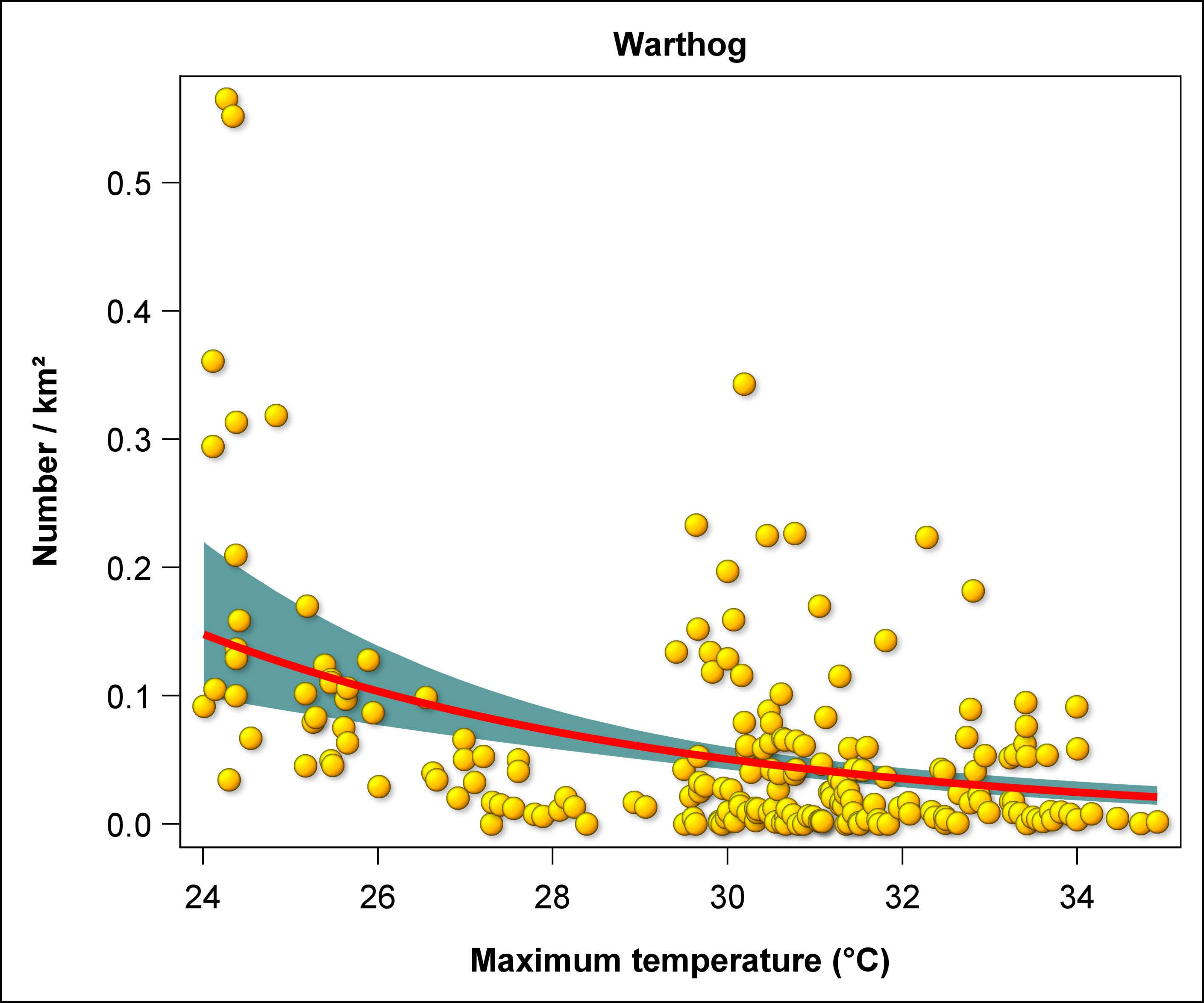

Warthog
Number / km²
0.0
0.1
0.2
0.3
0.4
0.5
24
26
28
30
32
34
Maximum temperature (°C)

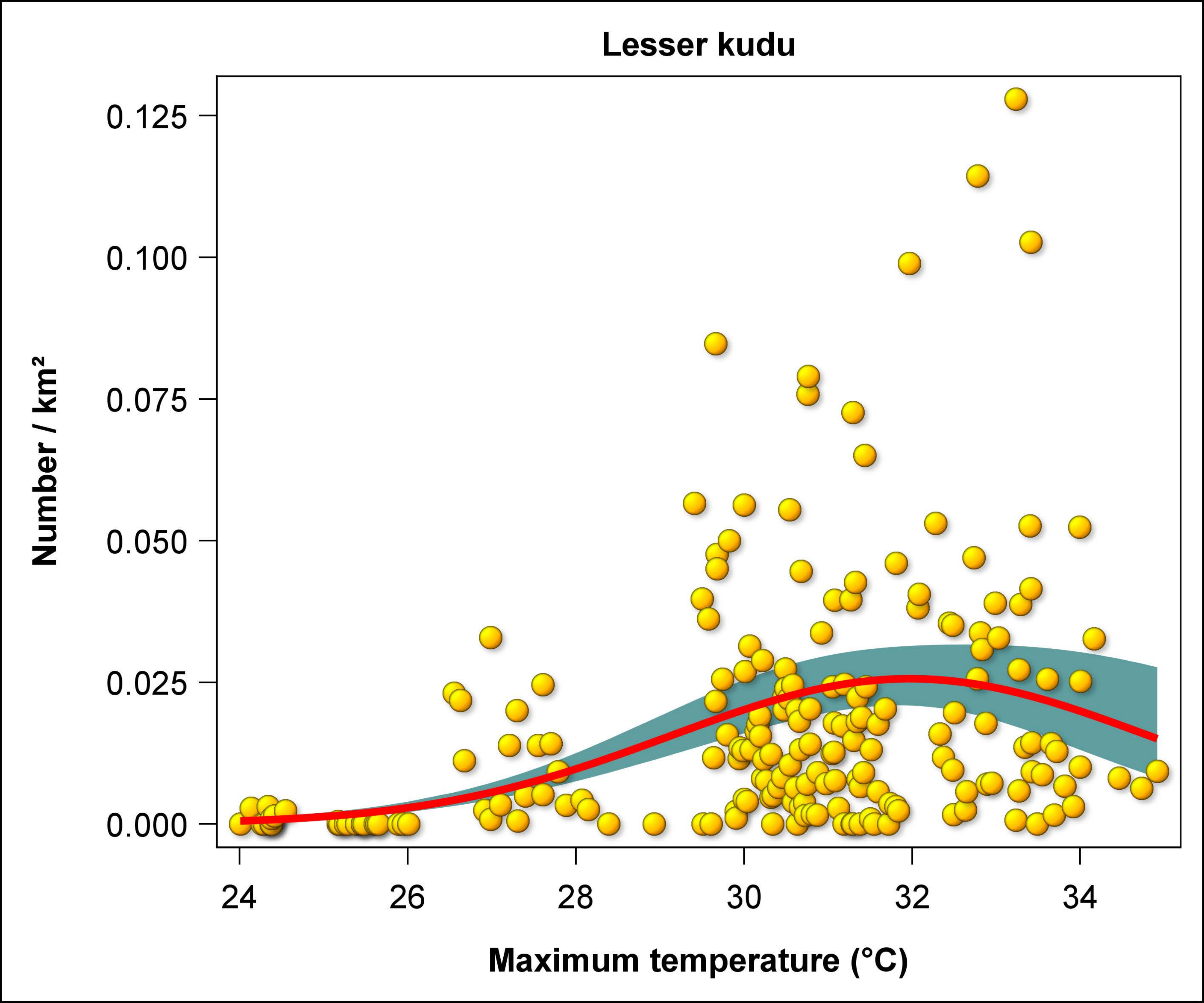
Lesser kudu
Number / km²
0.125
0.100
0.075
0.050
0.025
0.000
24
26
28
30
32
34
Maximum temperature (°C)

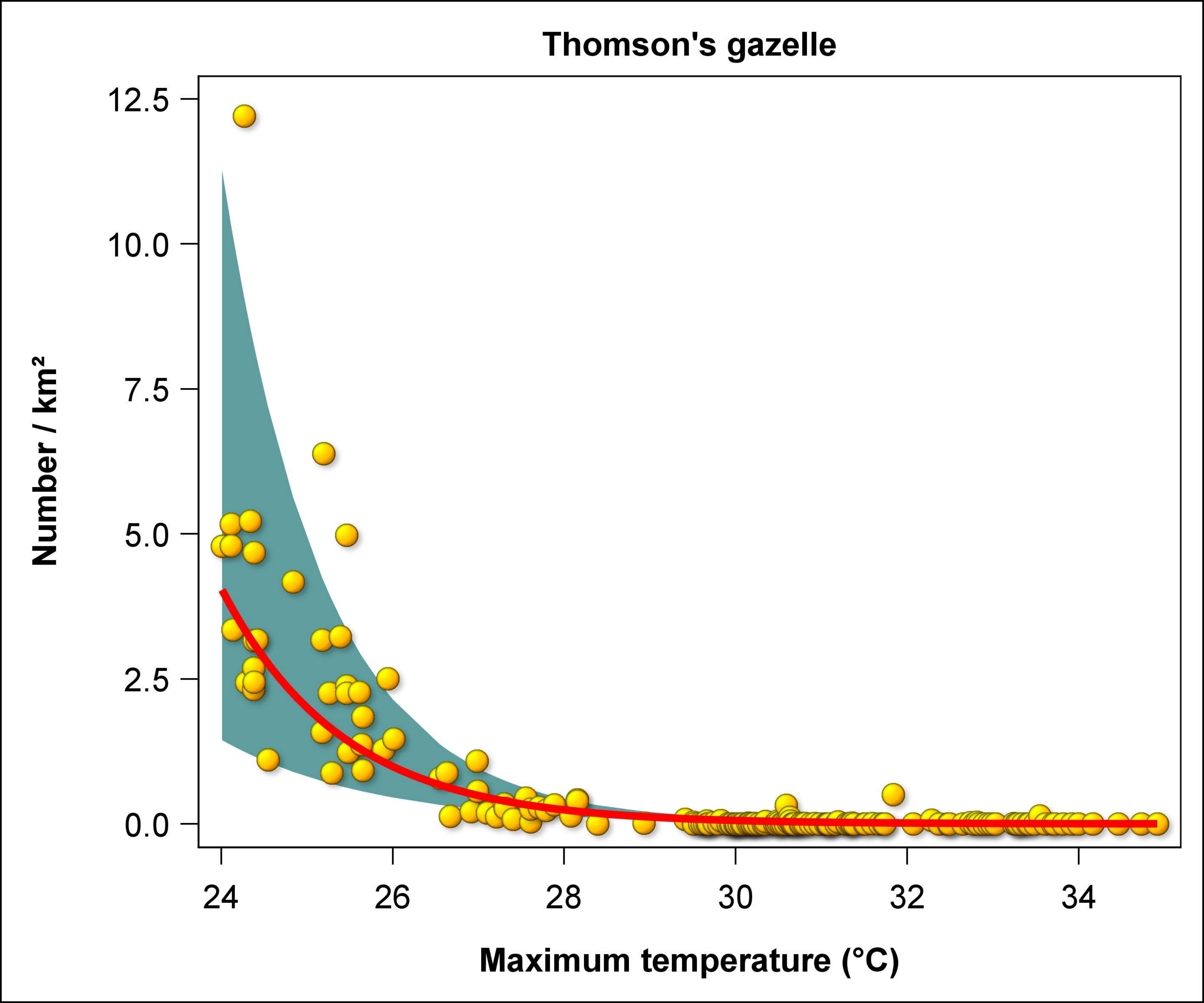

Thomson's gazelle
12.5
10.0
7.5
5.0
2.5
0.0
Number / km²
24
26
28
30
32
34
Maximum temperature (°C)

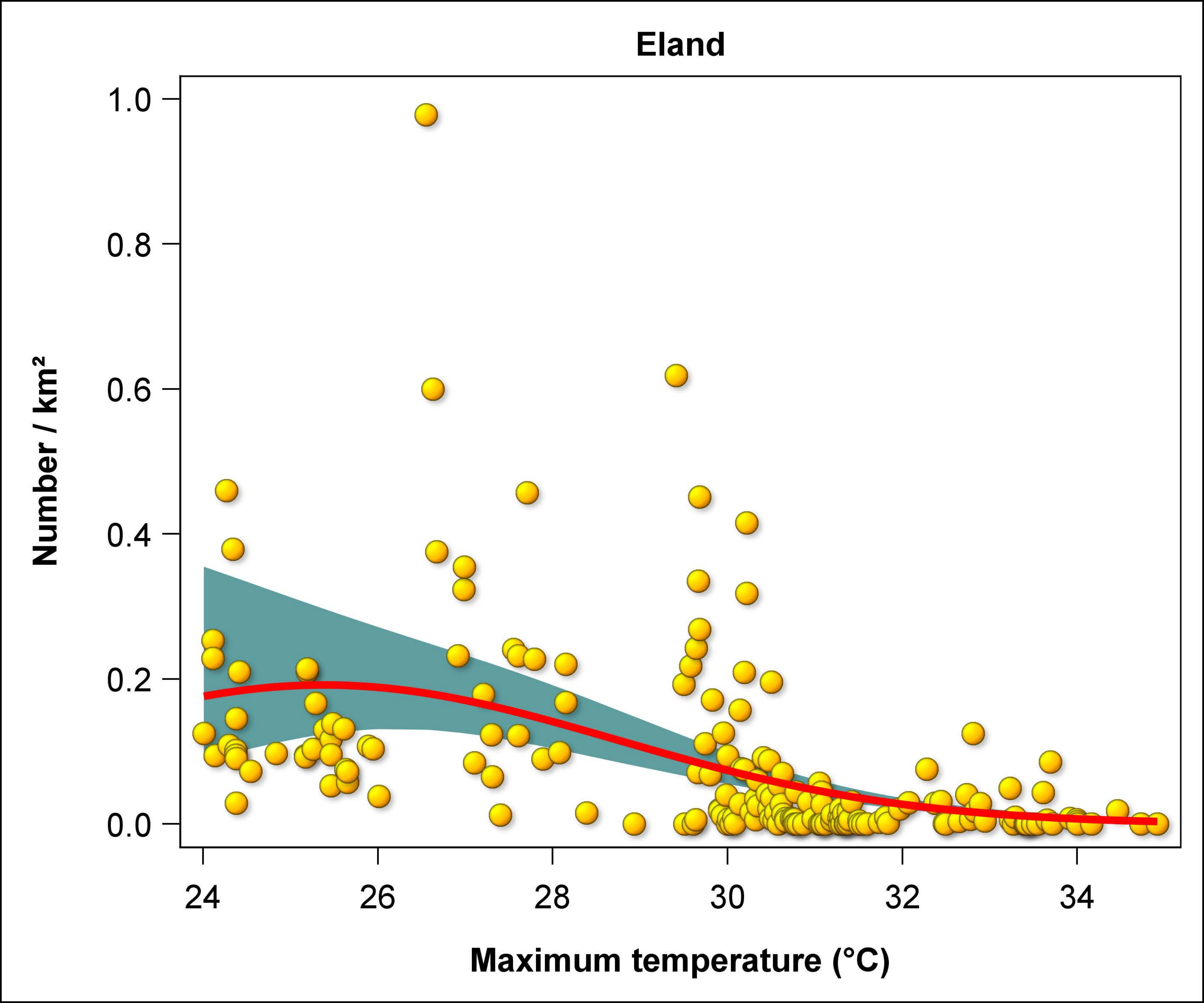

Eland
1.0
0.8
0.6
0.4
0.2
0.0
Number / km²
24
26
28
30
32
34
Maximum temperature (°C)

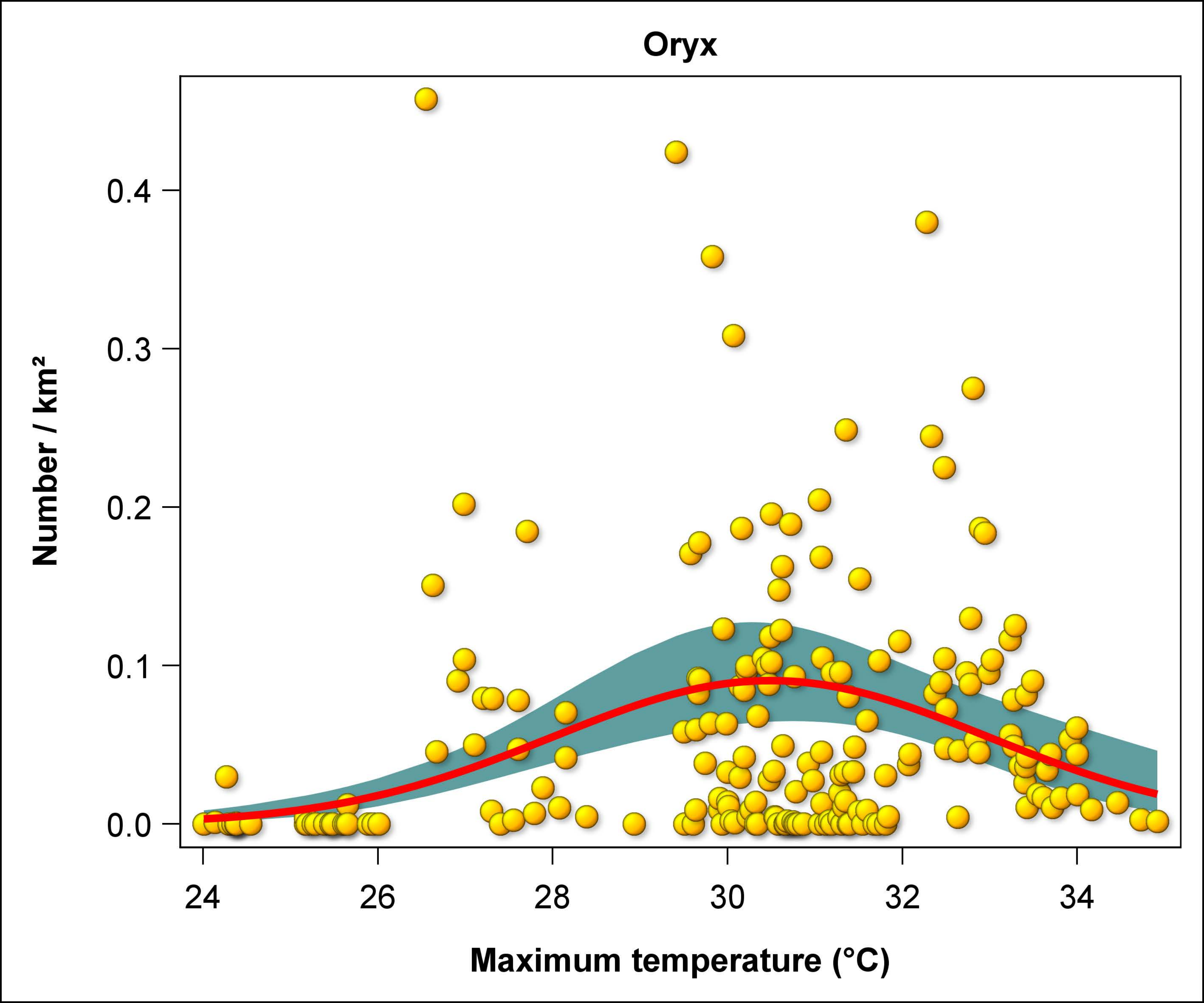

Oryx
Number / km²
0.0
0.1
0.2
0.3
0.4
24
26
28
30
32
34
Maximum temperature (°C)

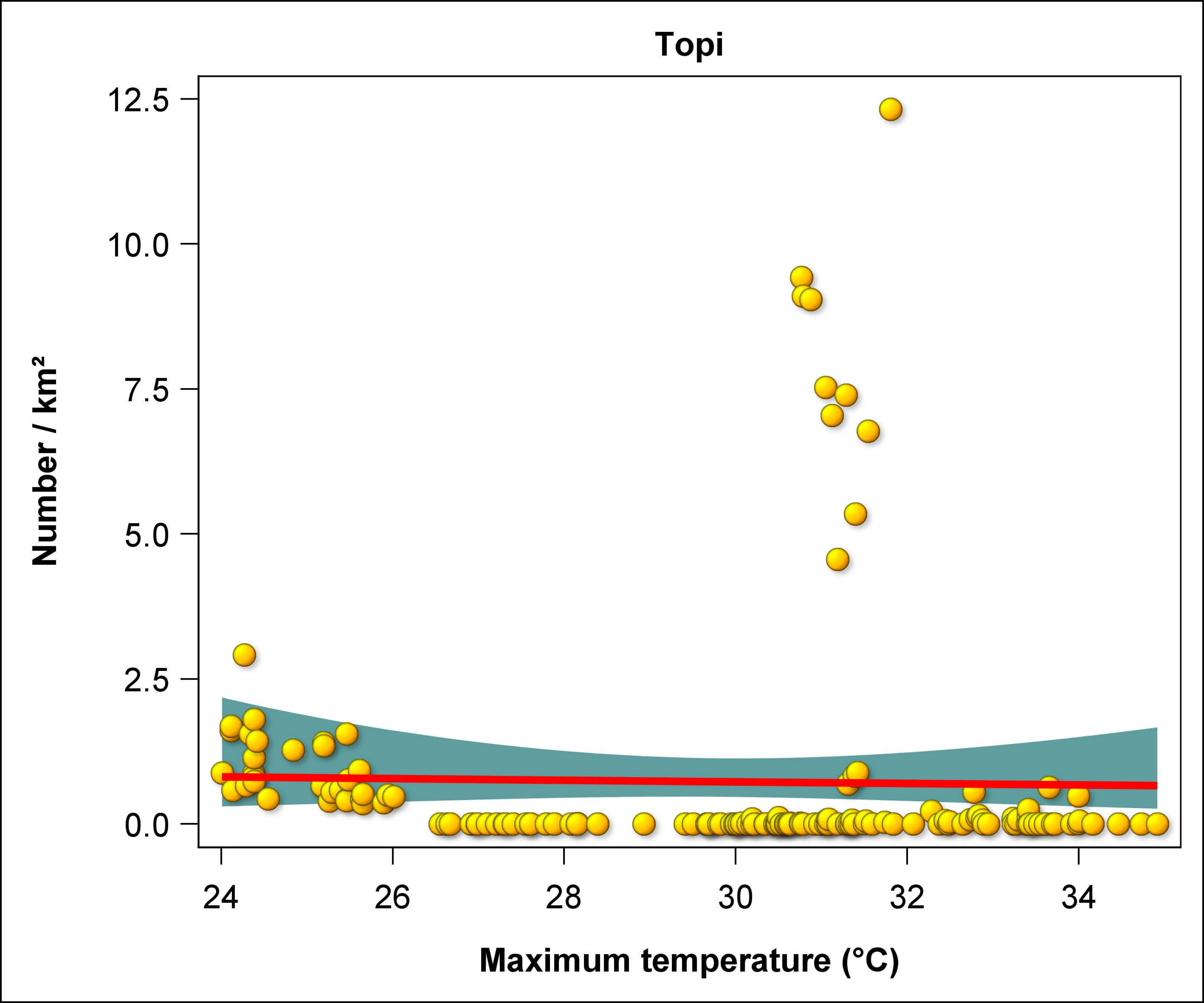

Topi
Number / km²
12.5
10.0
7.5
5.0
2.5
0.0
24
26
28
30
32
34
Maximum temperature (°C)

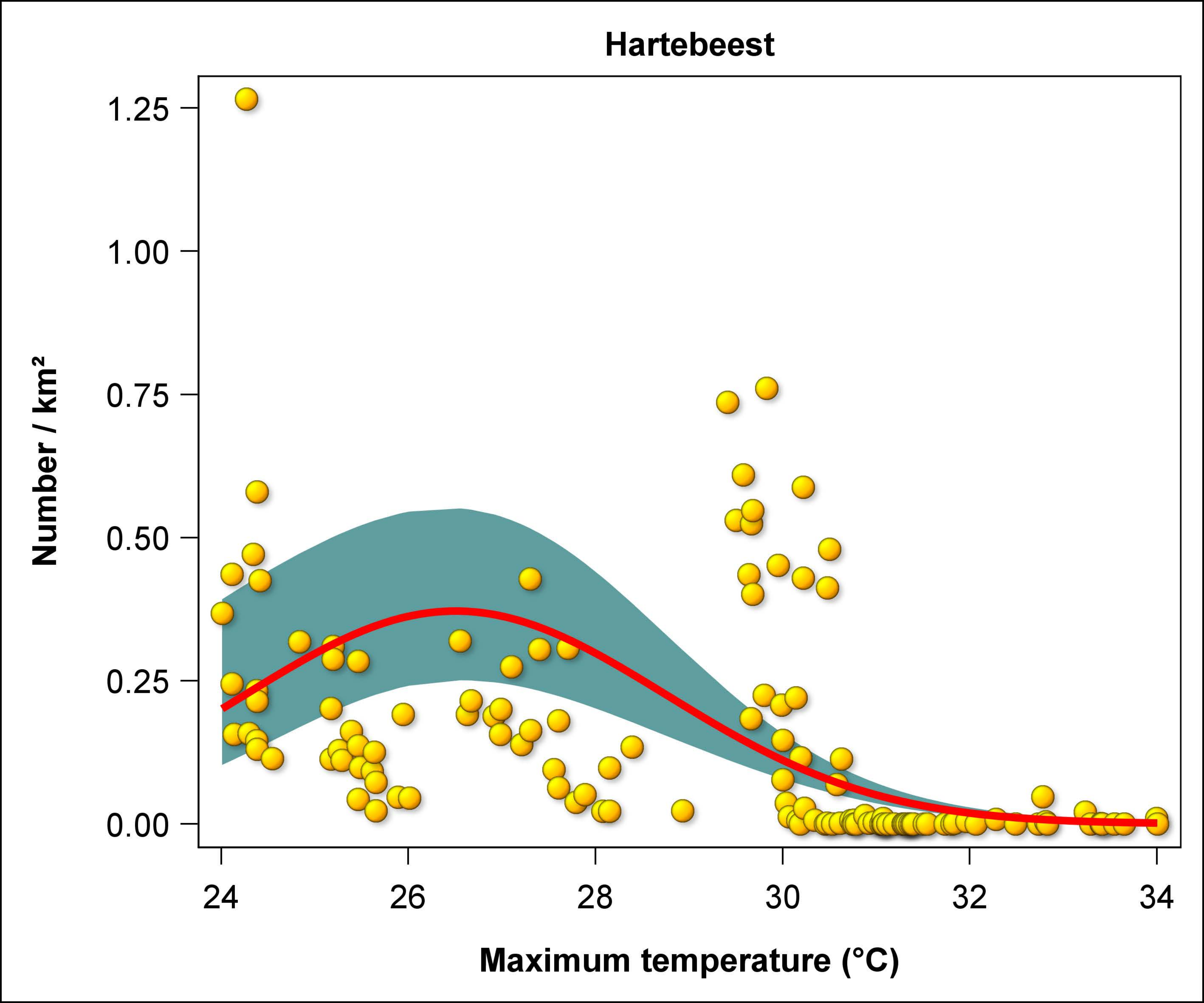
Hartebeest
Number / km²
1.25
1.00
0.75
0.50
0.25
0.00
24
26
28
30
32
34
Maximum temperature (°C)

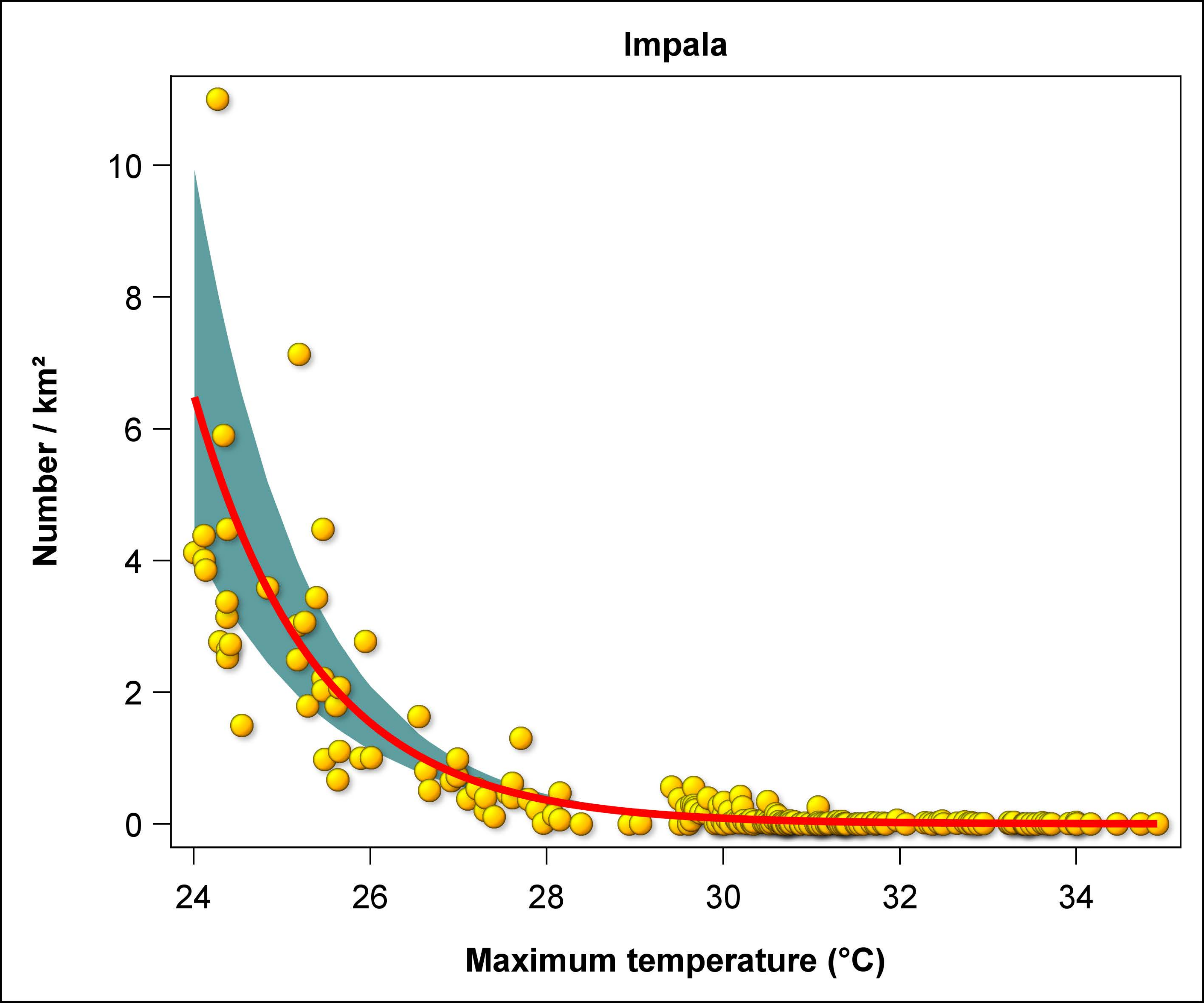

Impala
Number / km²
Maximum temperature (°C)
0
2
4
6
8
10
24
26
28
30
32
34

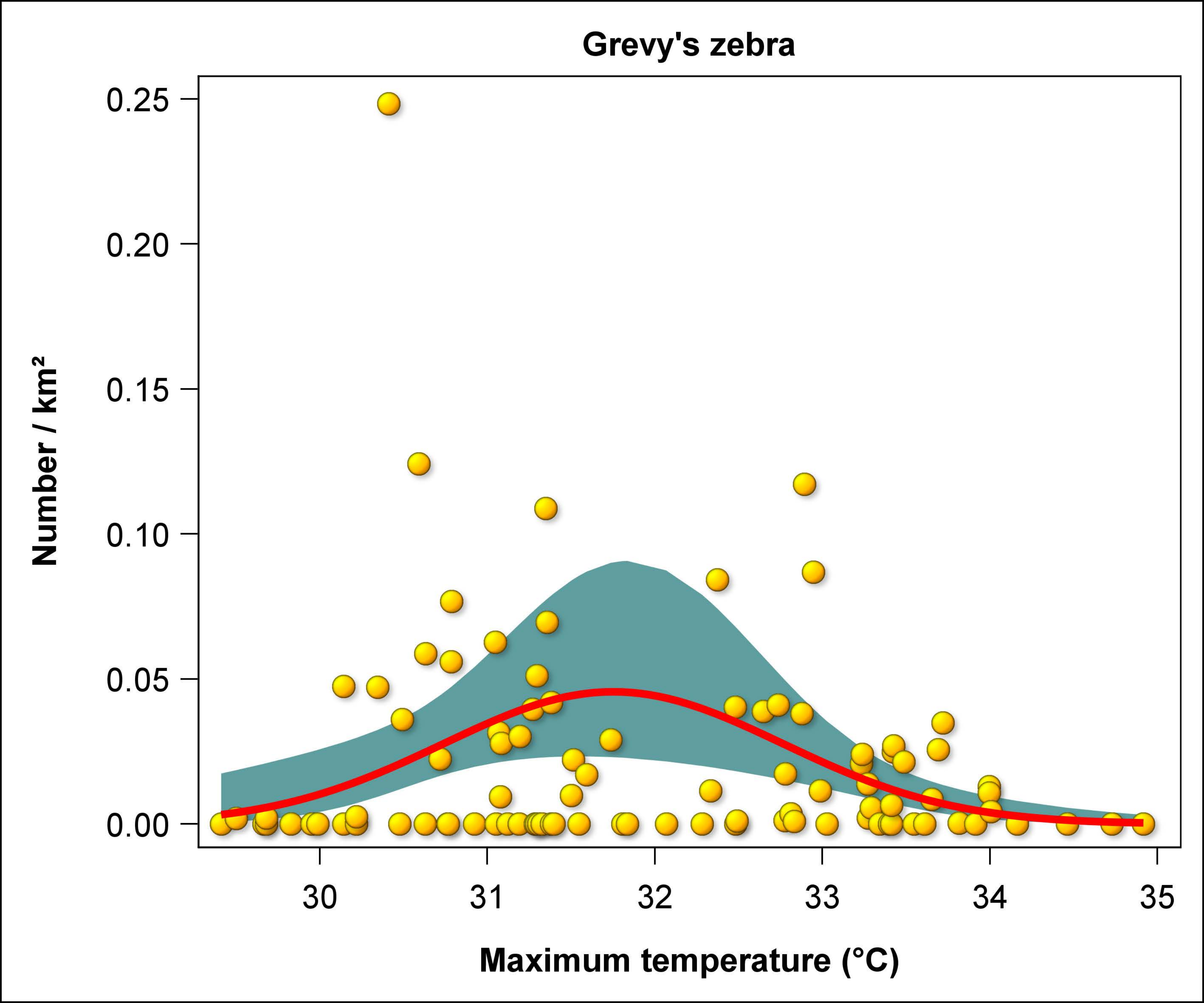

Grevy's zebra
Number / km²
0.25
0.20
0.15
0.10
0.05
0.00
30
31
32
33
34
35
Maximum temperature (°C)

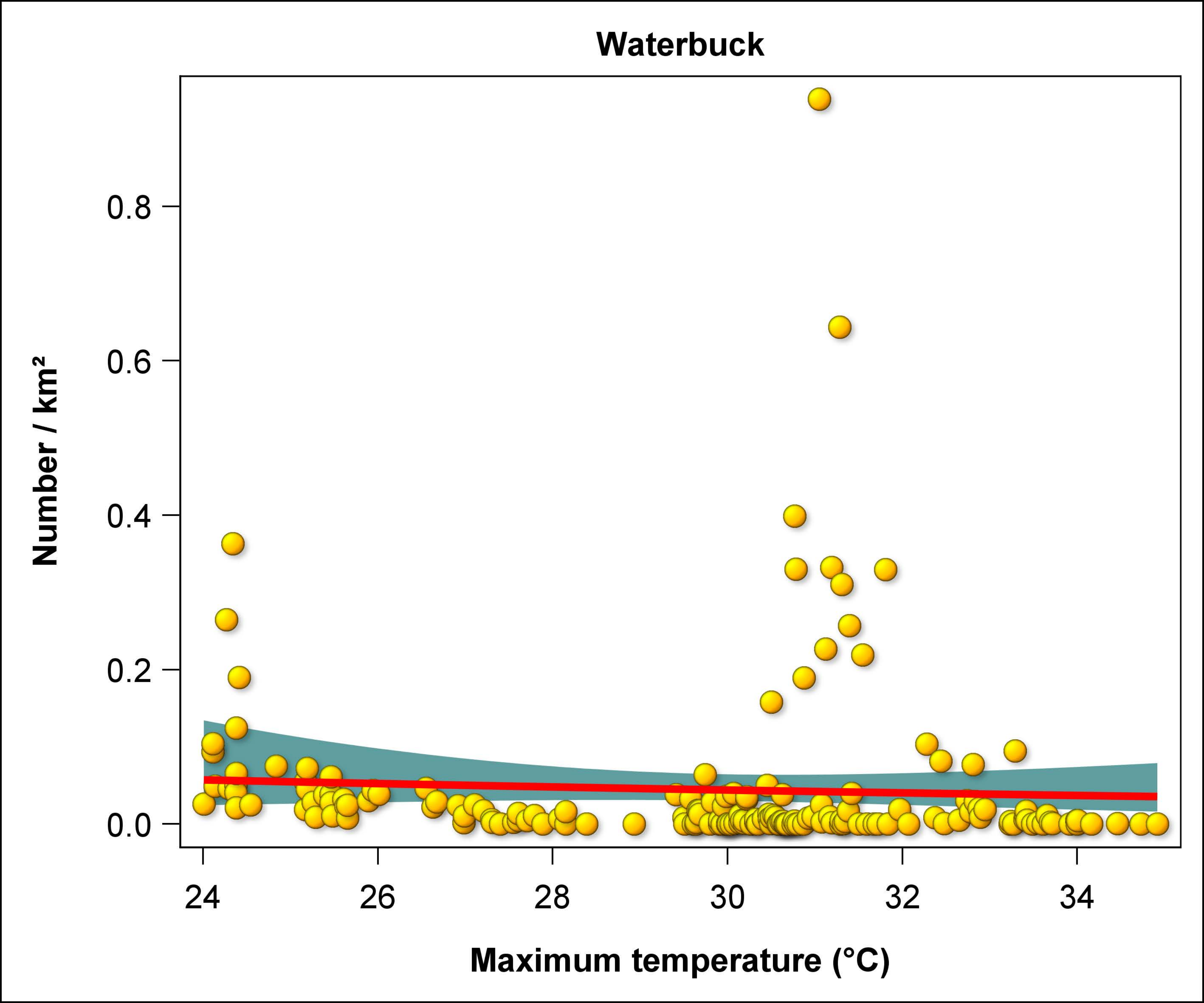

Waterbuck
Number / km²
0.0
0.2
0.4
0.6
0.8
24
26
28
30
32
34
Maximum temperature (°C)
