# Supplementary material for: Extreme Wildlife Declines and Concurrent Increase in Livestock Numbers in Kenya: What Are the Causes?
Source: PLoS One. 2016 Sep 27;11(9):e0163249. doi: 10.1371/journal.pone.0163249 (PMC5039022; doi:10.1371/journal.pone.0163249)
Supplement: S31 Fig — The filled circles are the observations, the solid lines are the quadratic regression lines while the shaded bands are the 95% pointwise confidence bands. (PDF) [file pone.0163249.s041.pdf]

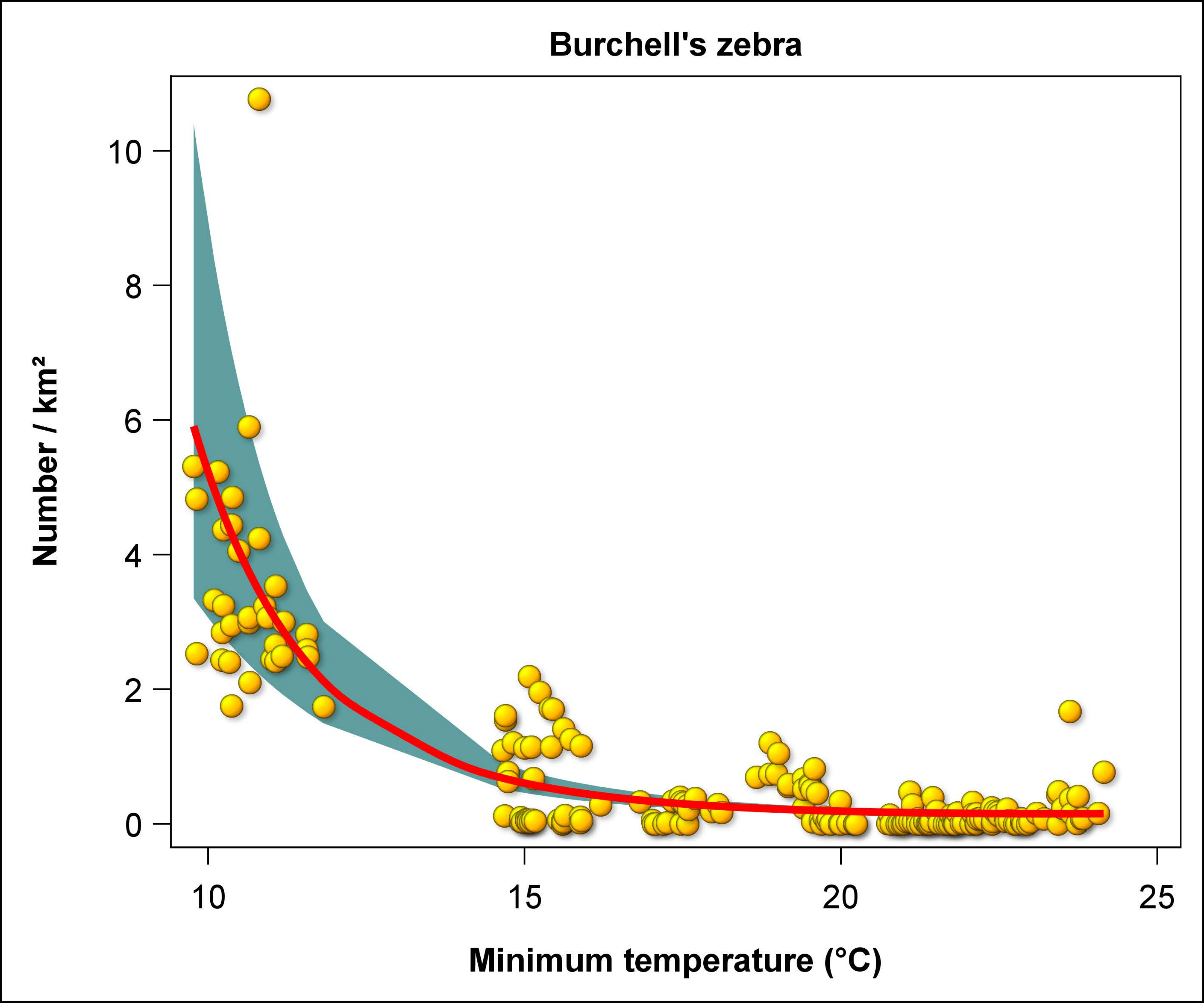
Burchell's zebra
Number / km²
Minimum temperature (°C)
0
2
4
6
8
10
10
15
20
25

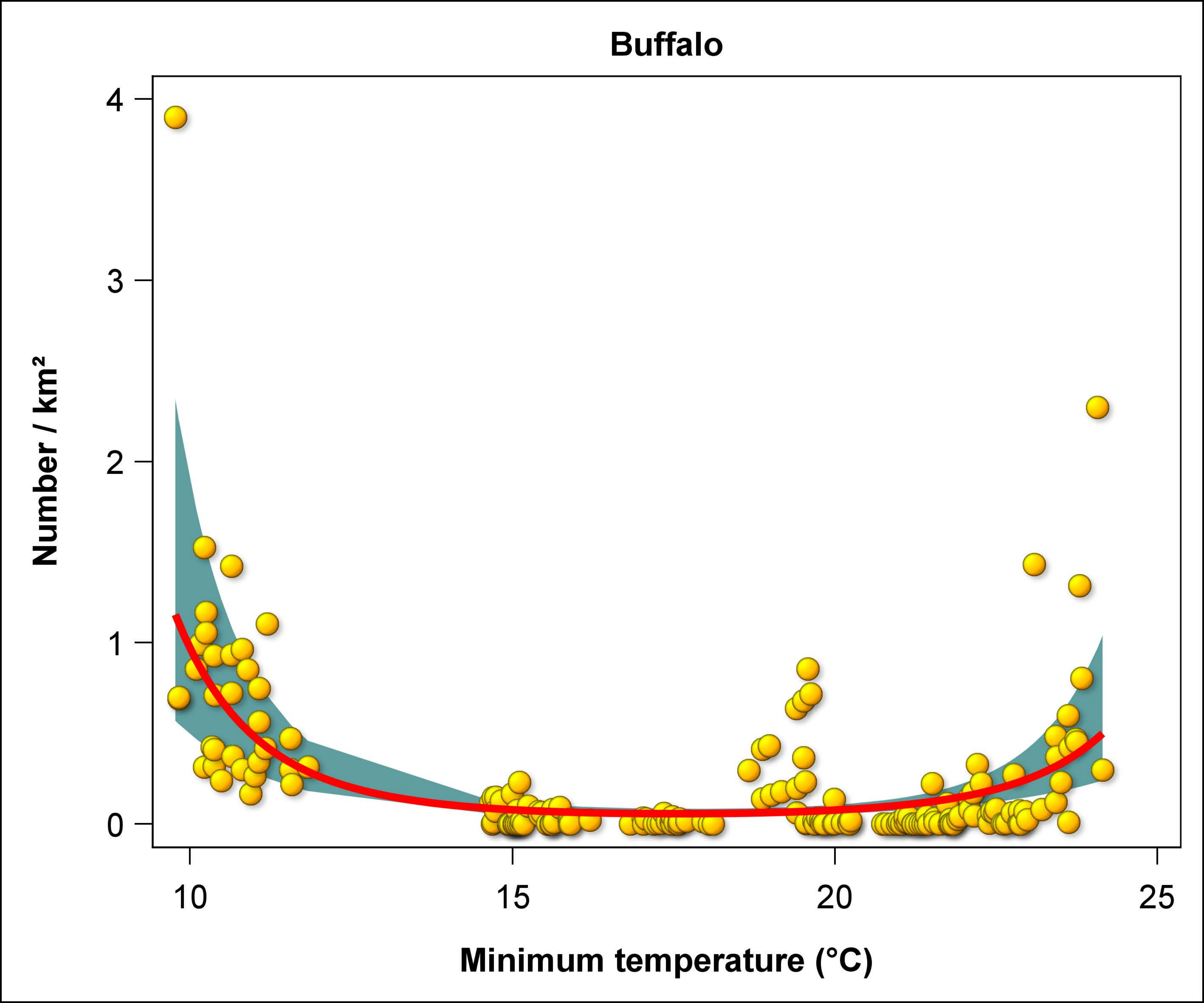
Buffalo
Number / km²
4
3
2
1
0
10
15
20
25
Minimum temperature (°C)

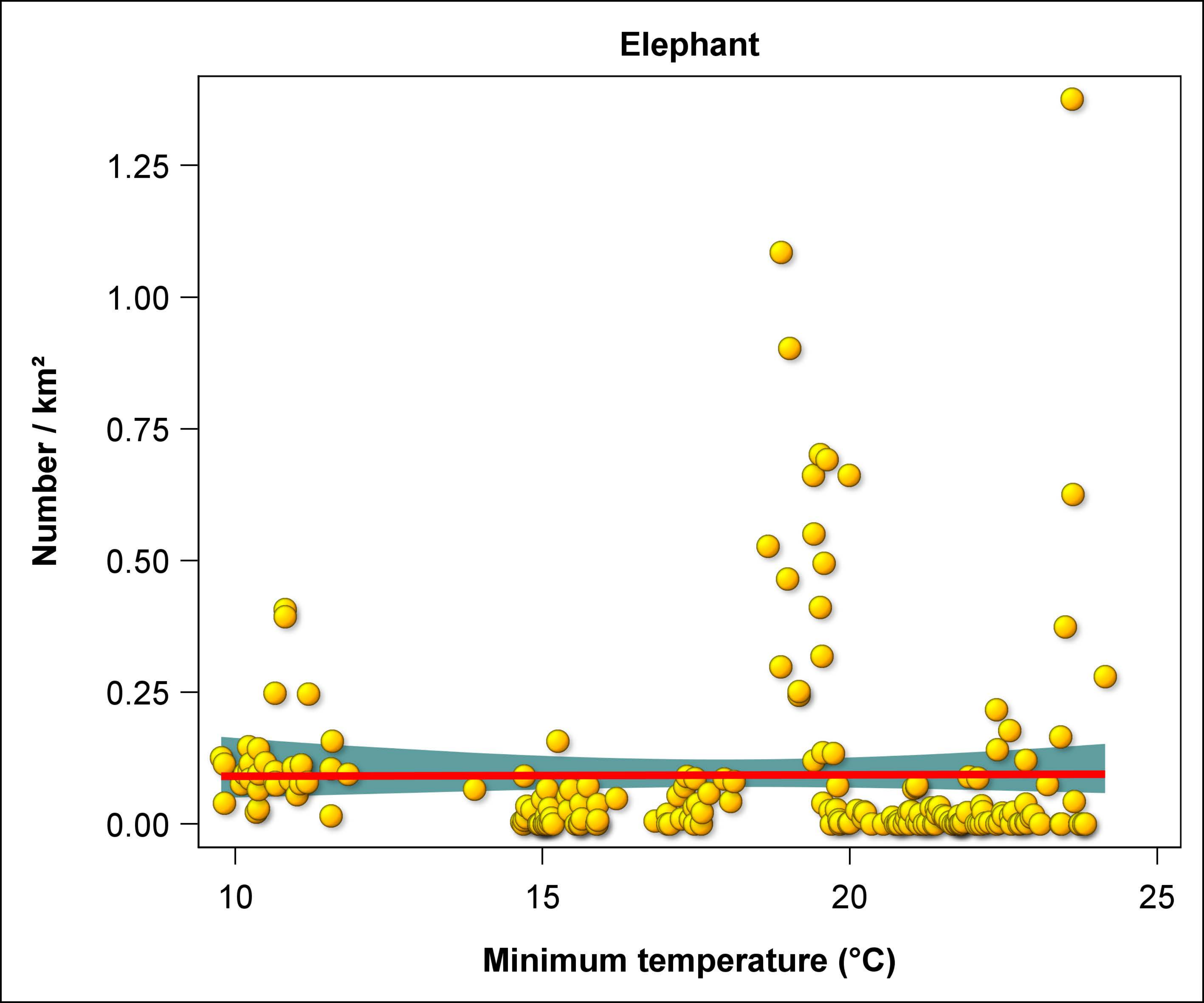

Elephant
Number / km²
1.25
1.00
0.75
0.50
0.25
0.00
10
15
20
25
Minimum temperature (°C)

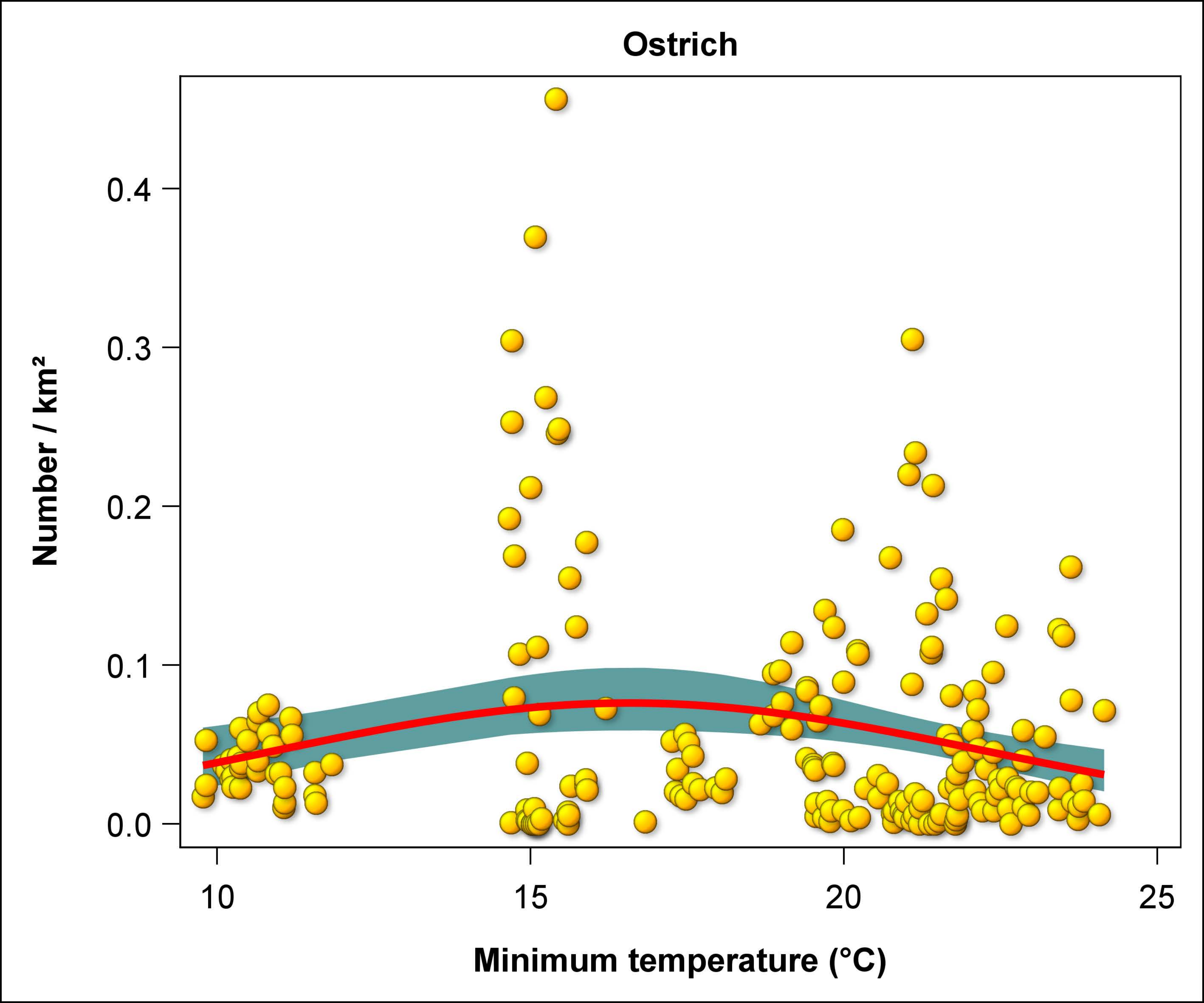

Ostrich
Number / km²
0.0
0.1
0.2
0.3
0.4
10
15
20
25
Minimum temperature (°C)

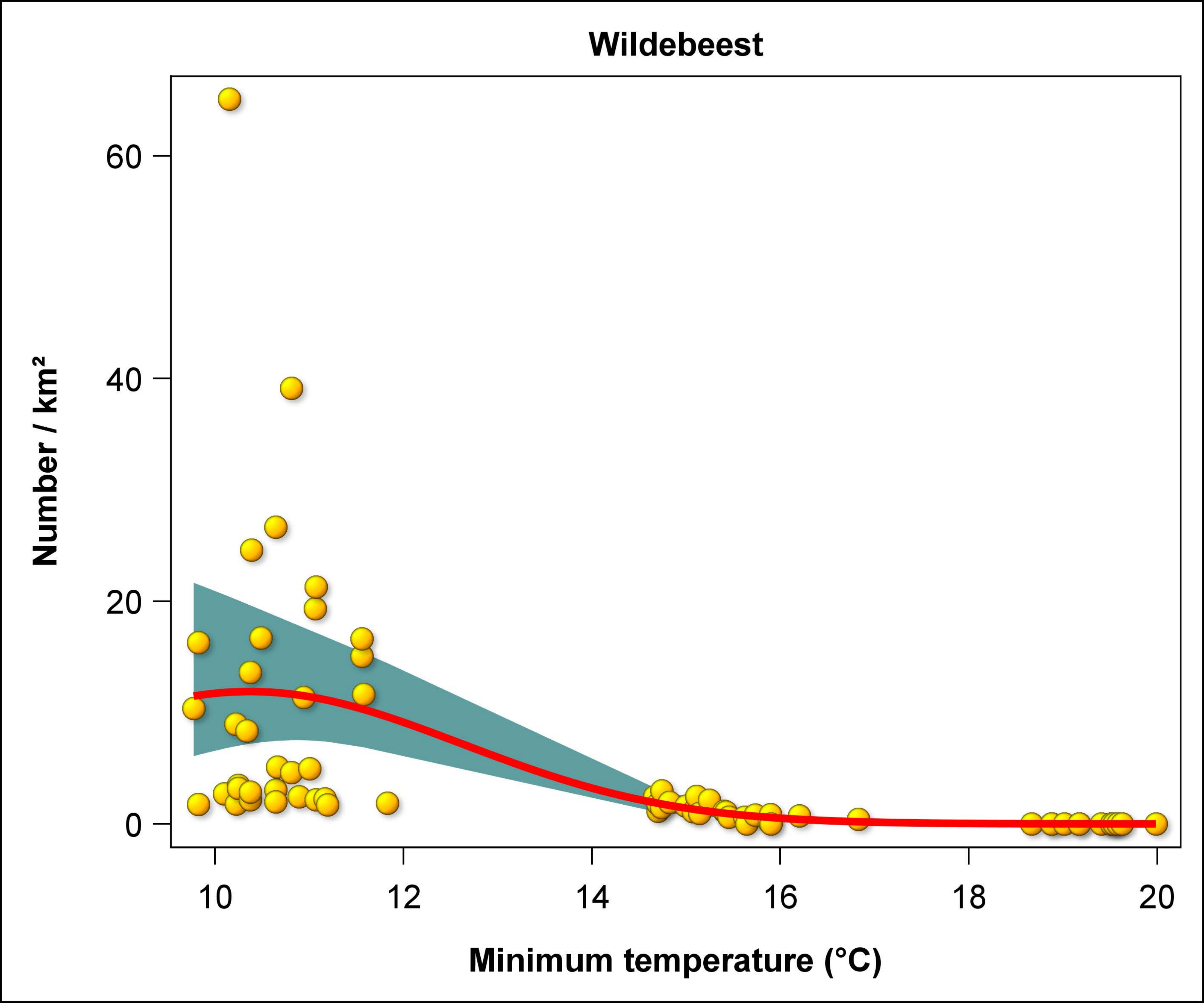

Wildebeest
Number / km²
Minimum temperature (°C)
0
20
40
60
10
12
14
16
18
20

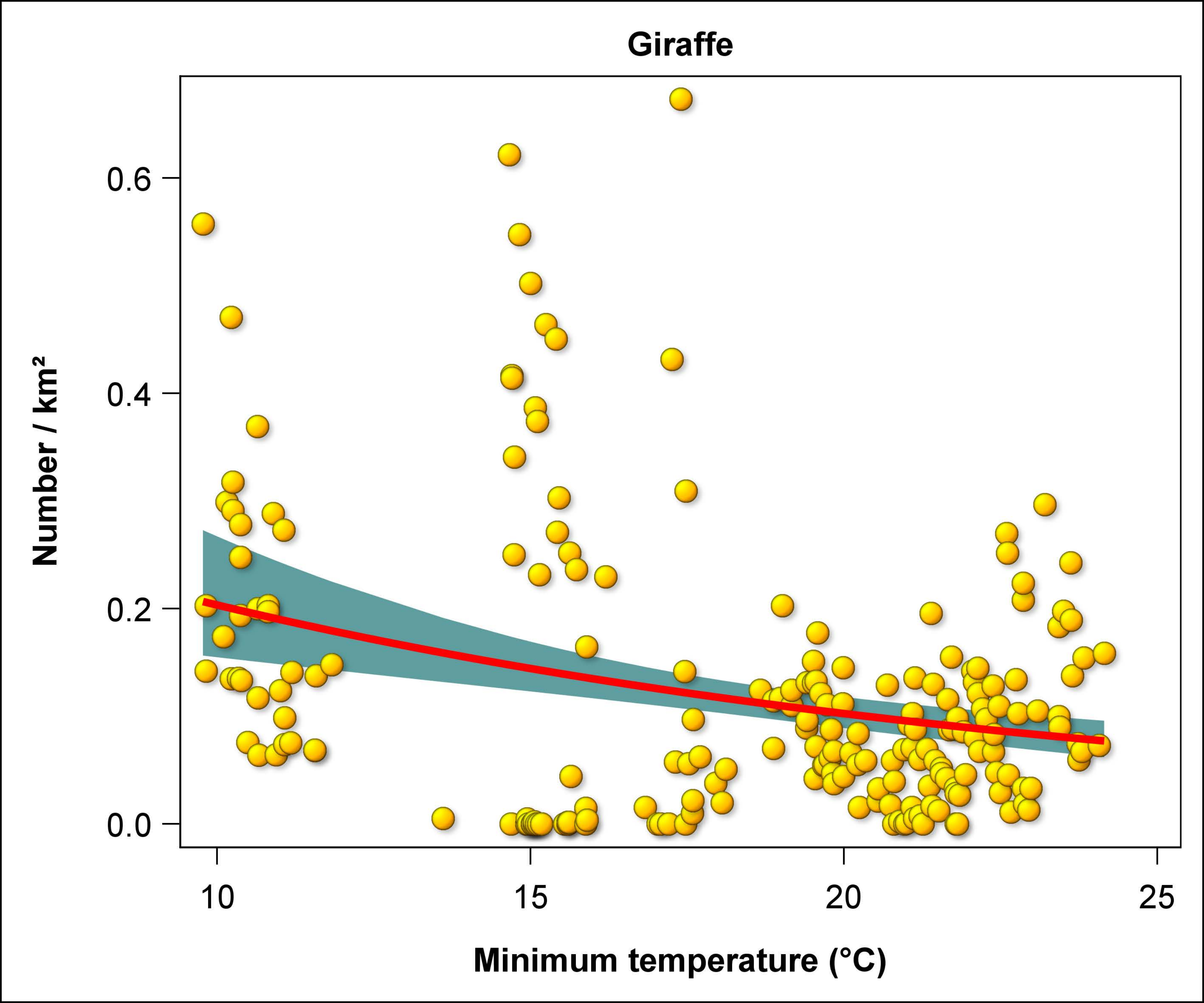
Giraffe
Number / km²
Minimum temperature (°C)
0.0
0.2
0.4
0.6
10
15
20
25

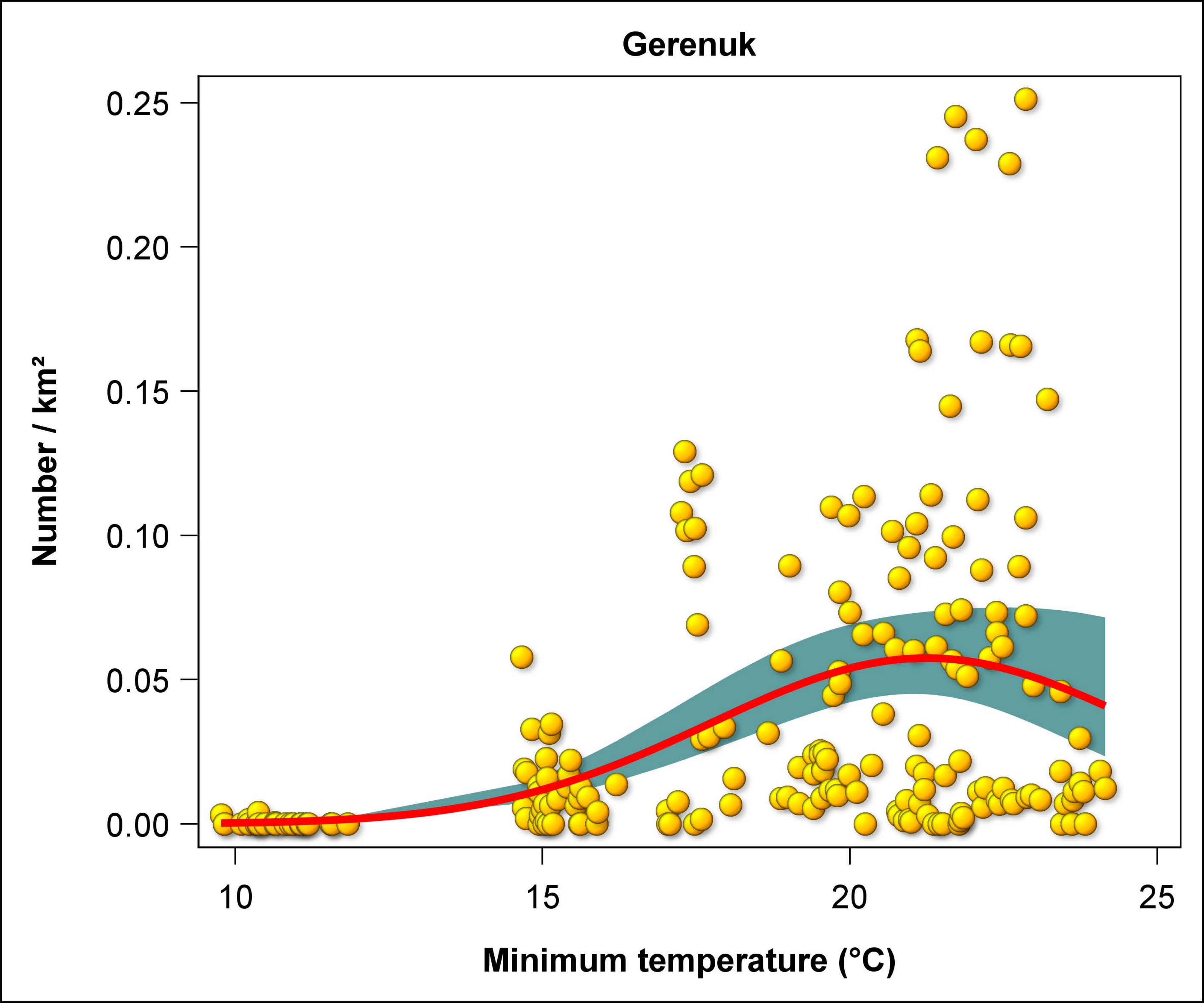

Gerenuk
0.25
0.20
0.15
0.10
0.05
0.00
Number / km²
10
15
20
25
Minimum temperature (°C)

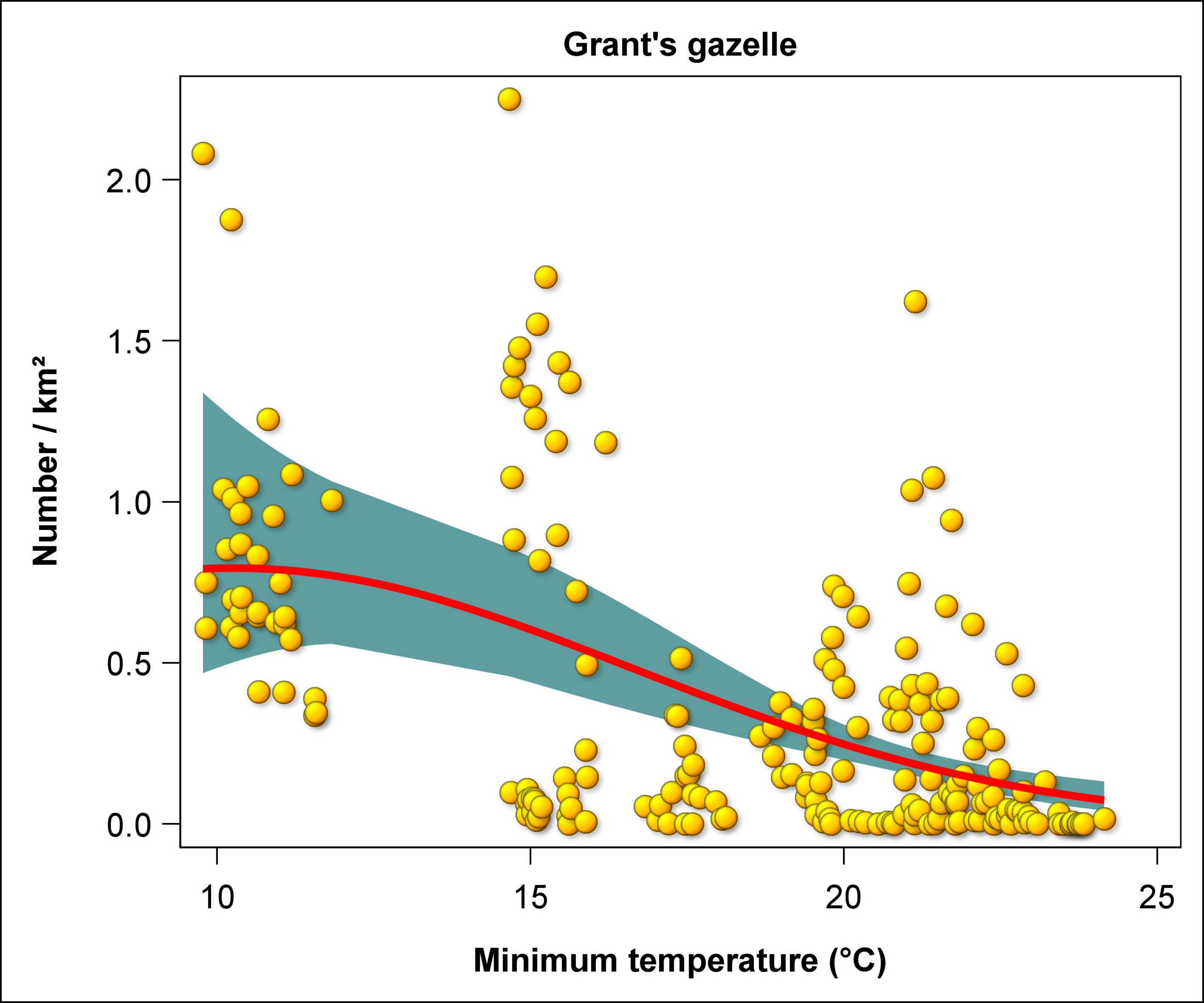

Grant's gazelle
Number / km²
0.0
0.5
1.0
1.5
2.0
10
15
20
25
Minimum temperature (°C)

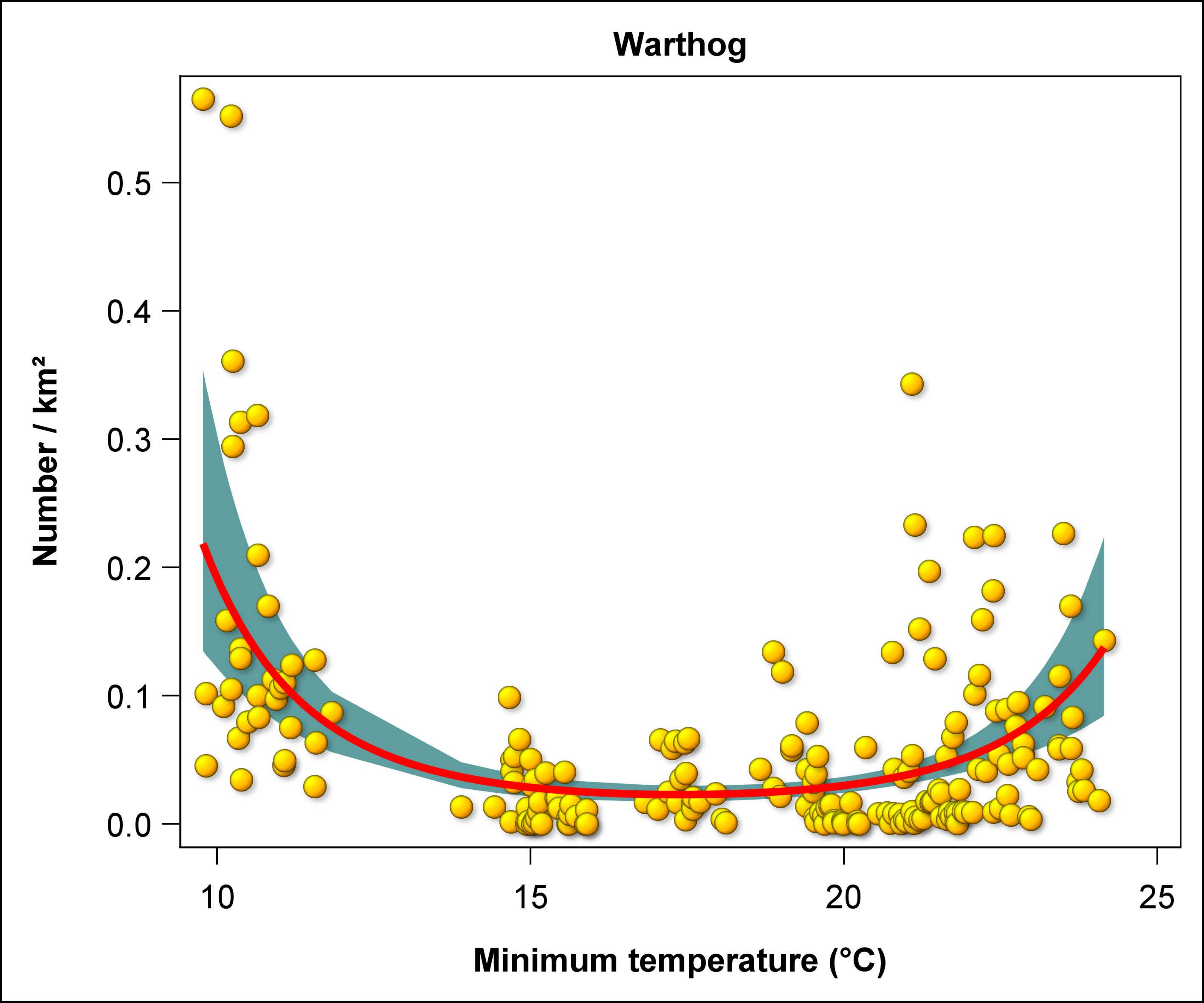

Warthog
Number / km²
0.0
0.1
0.2
0.3
0.4
0.5
10
15
20
25
Minimum temperature (°C)

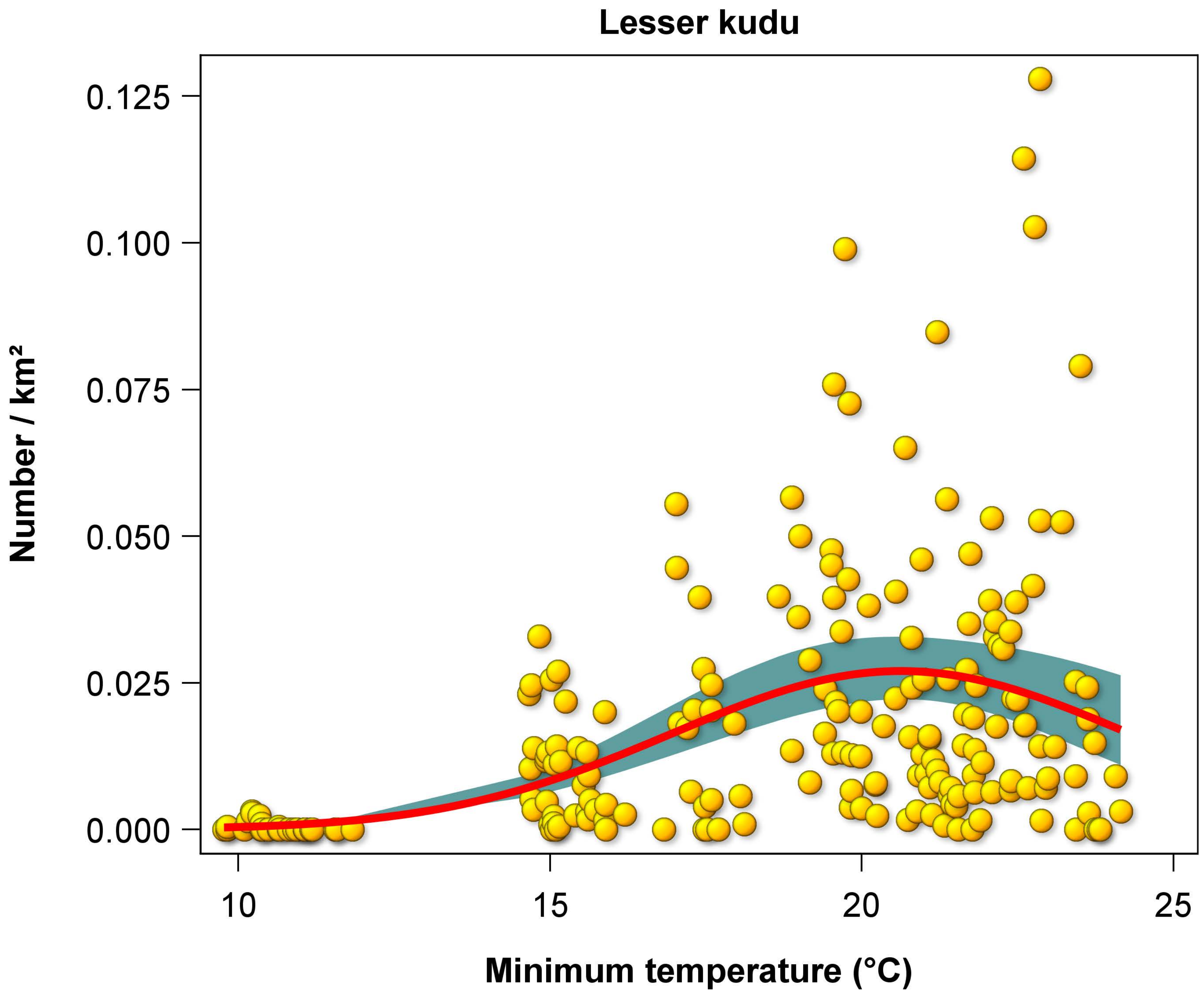

Lesser kudu
Number / km²
0.125
0.100
0.075
0.050
0.025
0.000
10
15
20
25
Minimum temperature (°C)

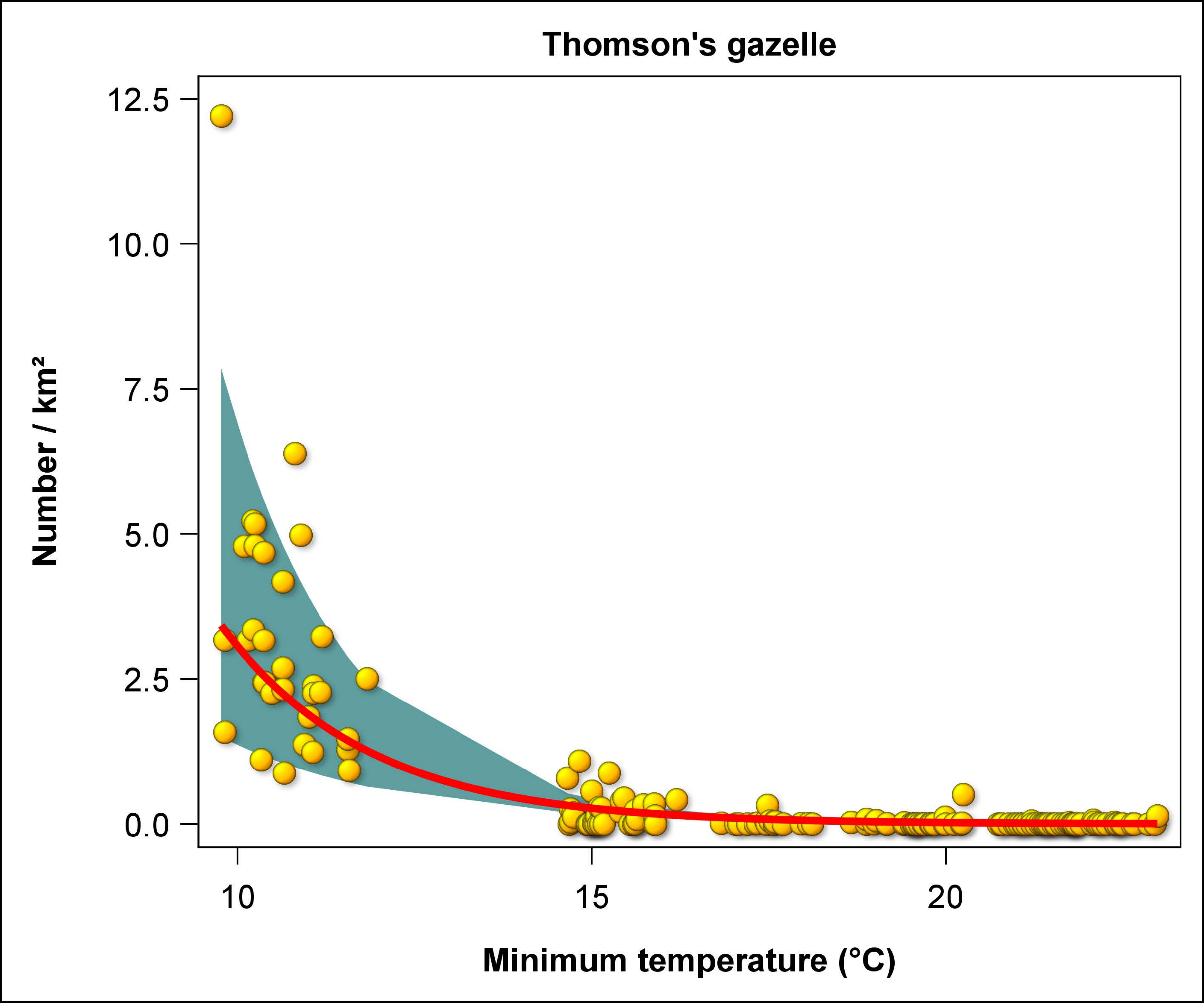

Thomson's gazelle
12.5
10.0
7.5
5.0
2.5
0.0
Number / km²
10
15
20
Minimum temperature (°C)

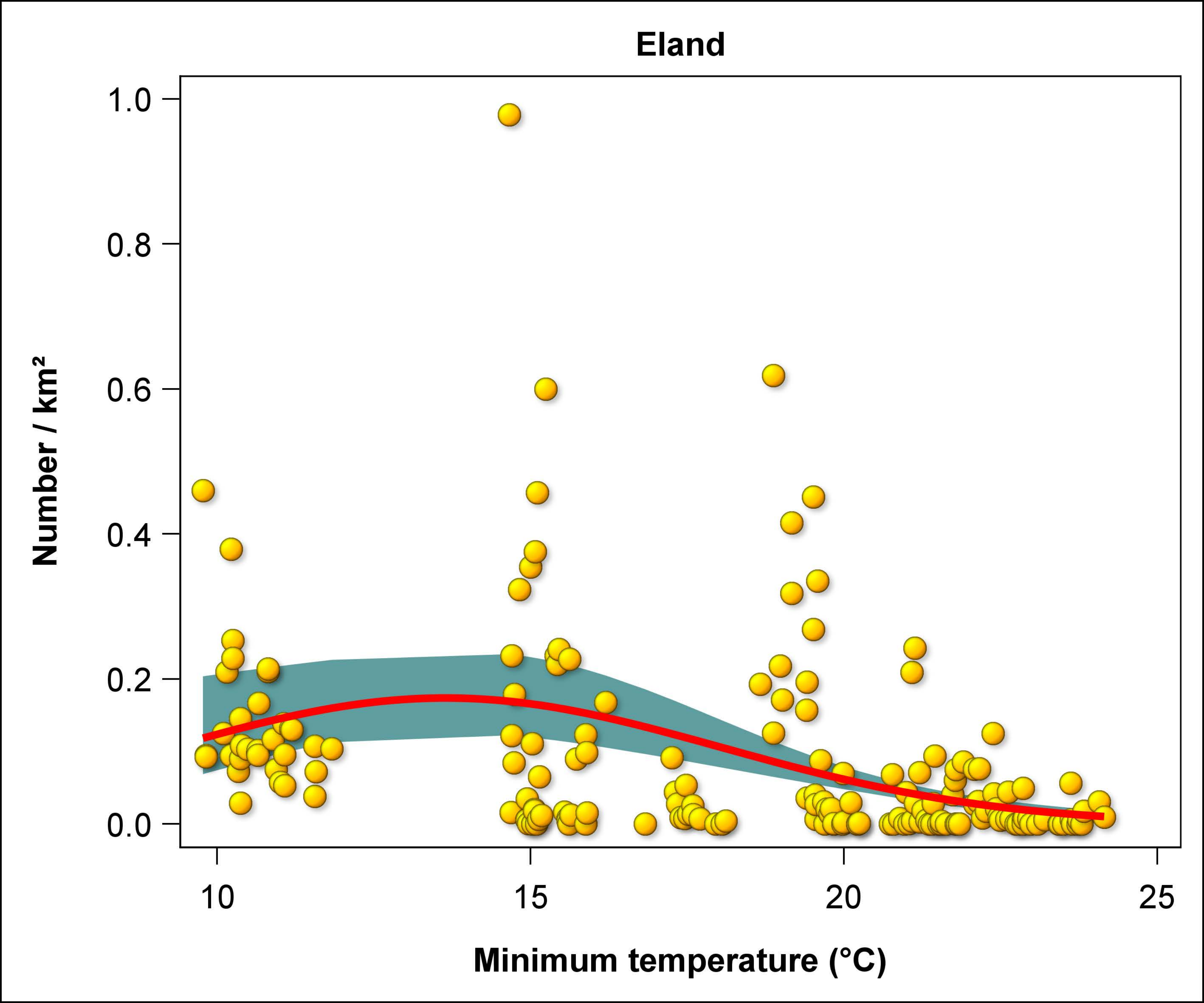

Eland
Number / km²
1.0
0.8
0.6
0.4
0.2
0.0
10
15
20
25
Minimum temperature (°C)

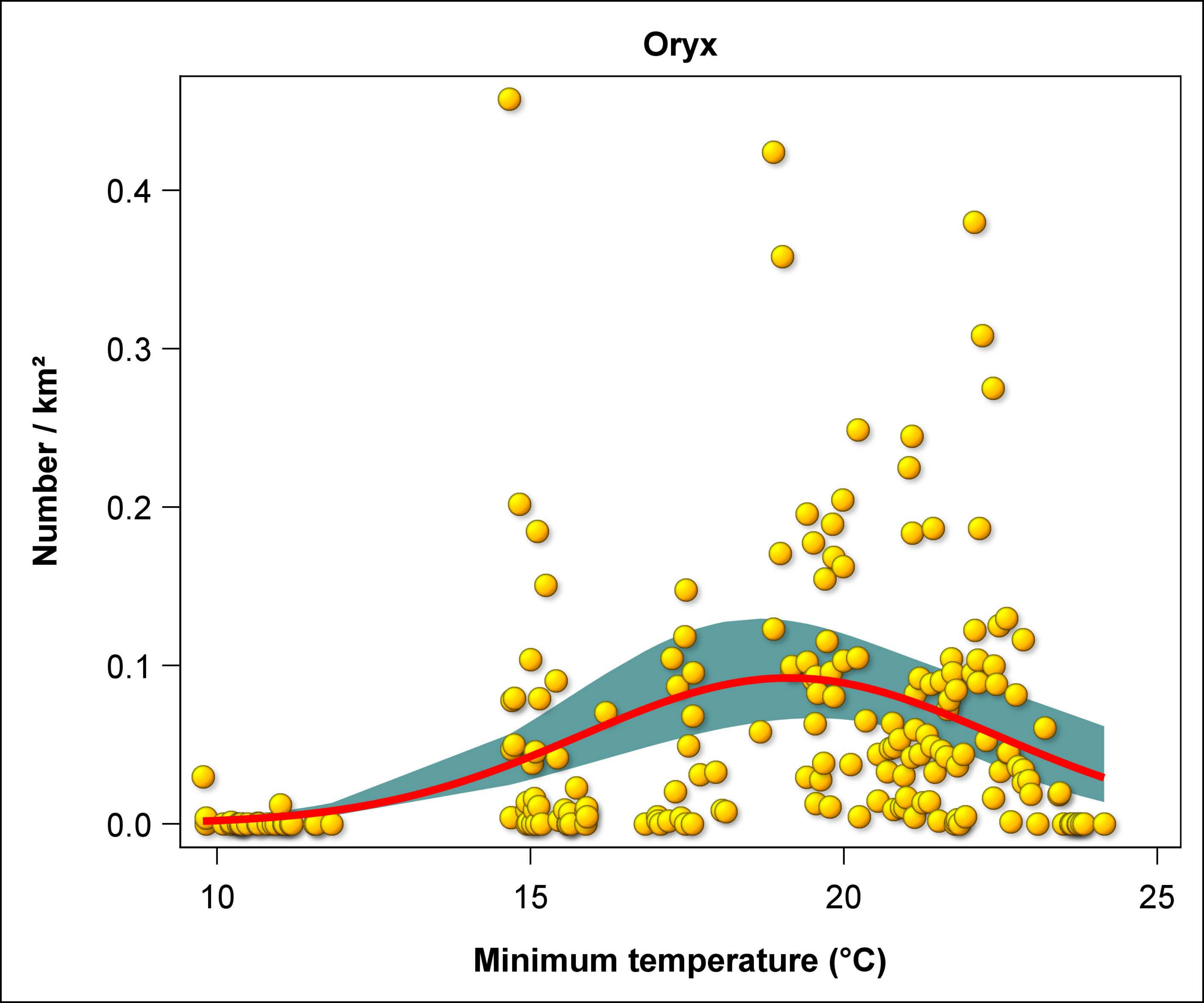
Oryx
Number / km²
0.0
0.1
0.2
0.3
0.4
10
15
20
25
Minimum temperature (°C)

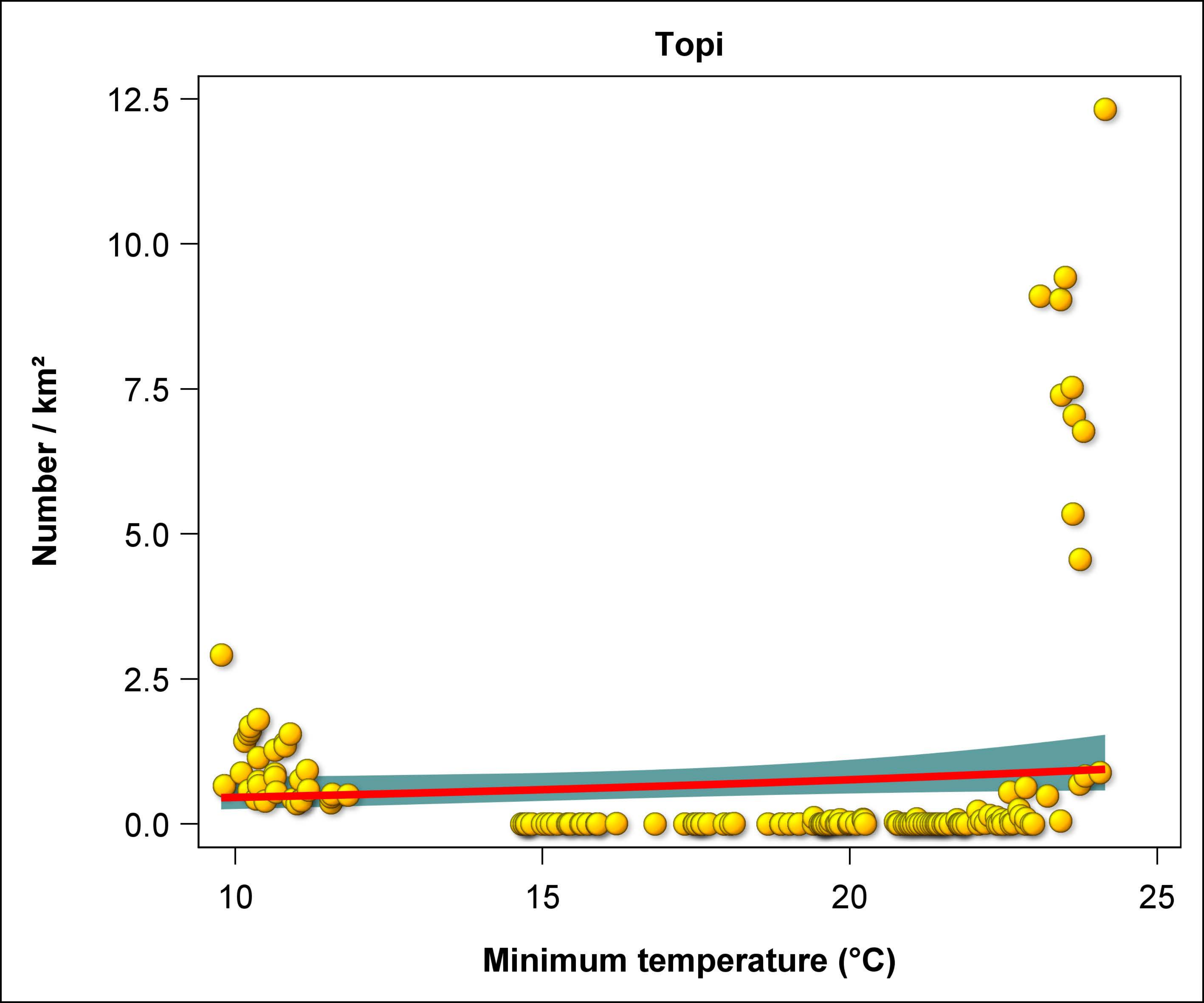
Topi
Number / km²
12.5
10.0
7.5
5.0
2.5
0.0
10
15
20
25
Minimum temperature (°C)

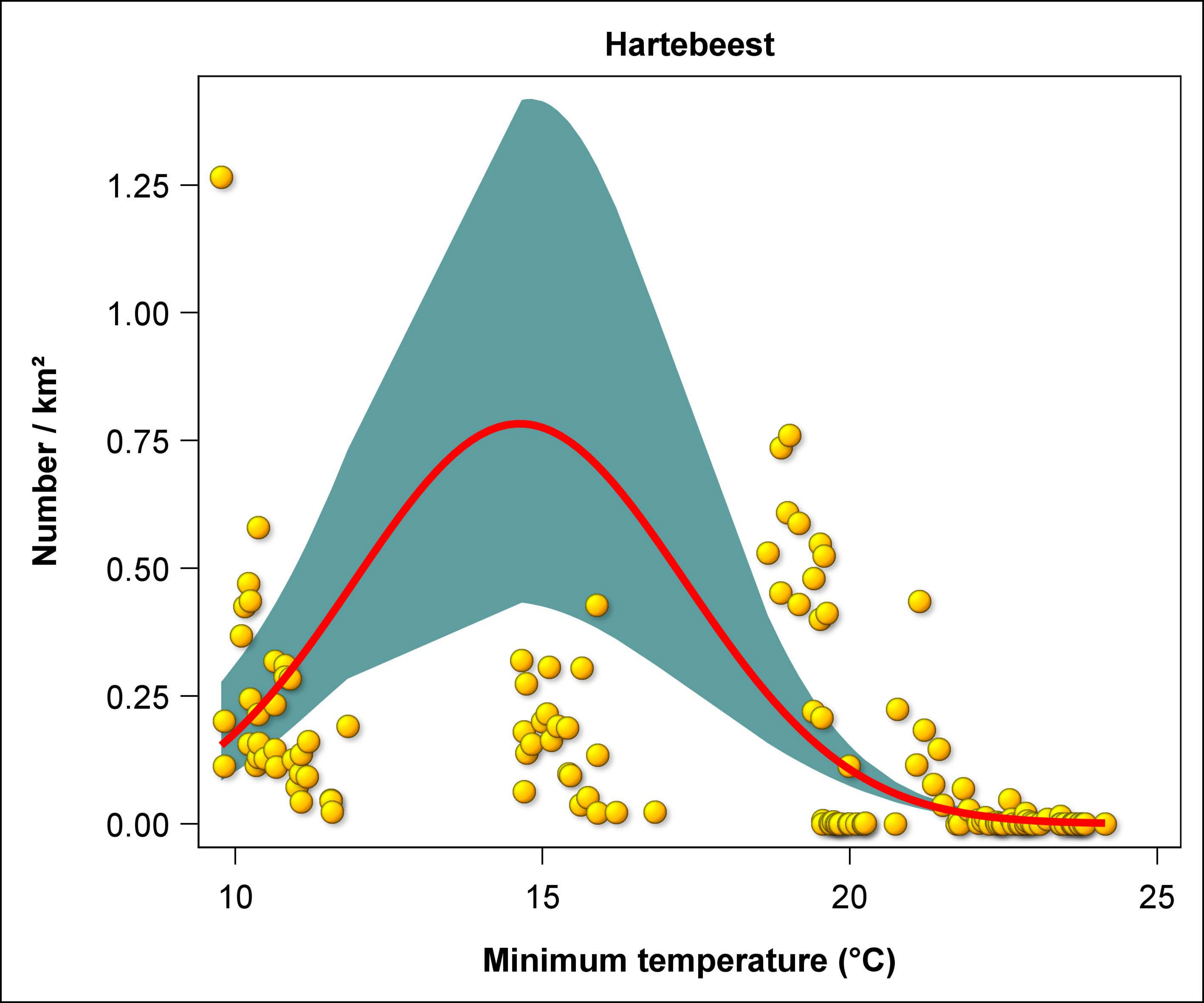

Hartebeest
Number / km²
1.25
1.00
0.75
0.50
0.25
0.00
10
15
20
25
Minimum temperature (°C)

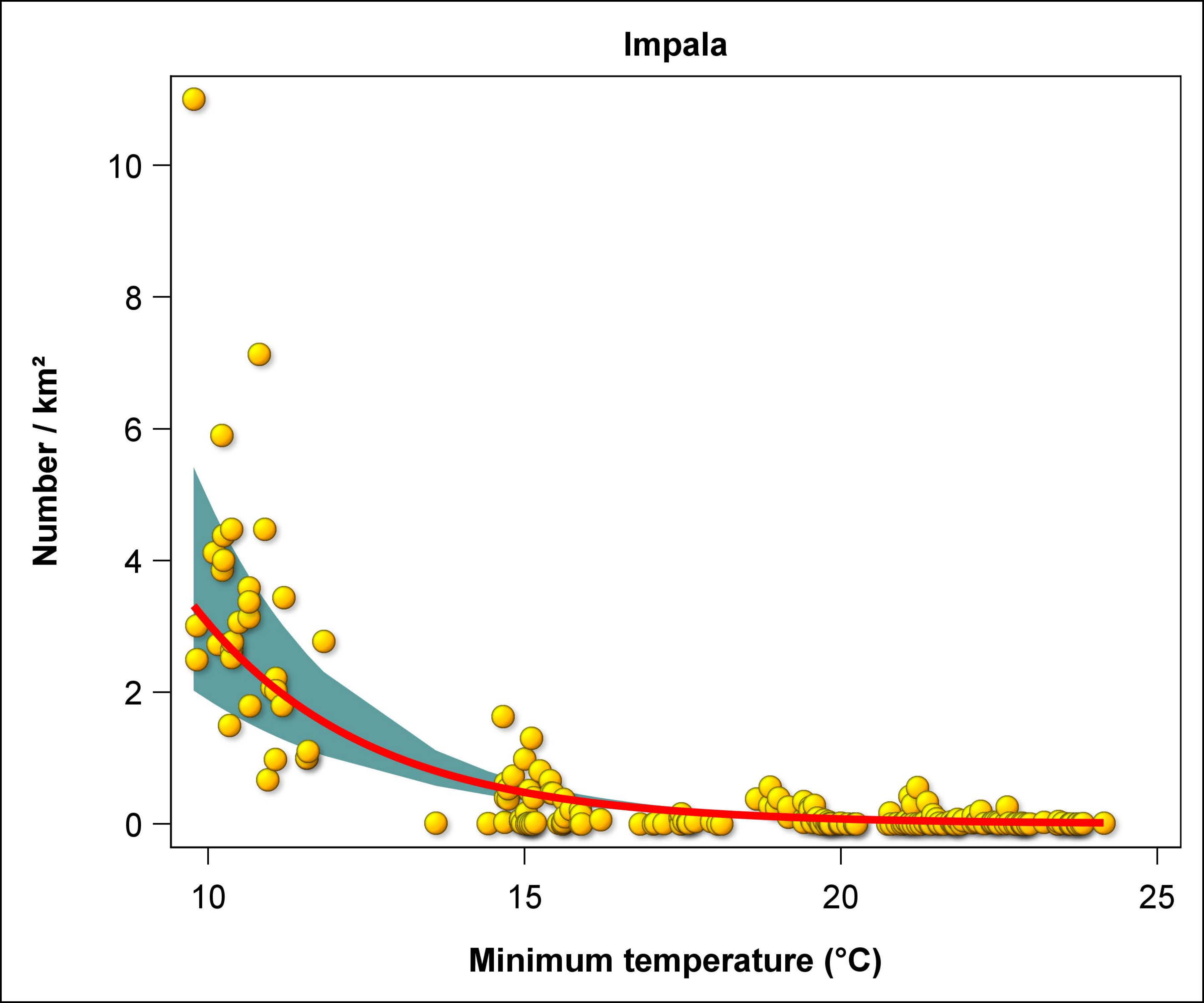

Impala
Number / km²
0
2
4
6
8
10
10
15
20
25
Minimum temperature (°C)

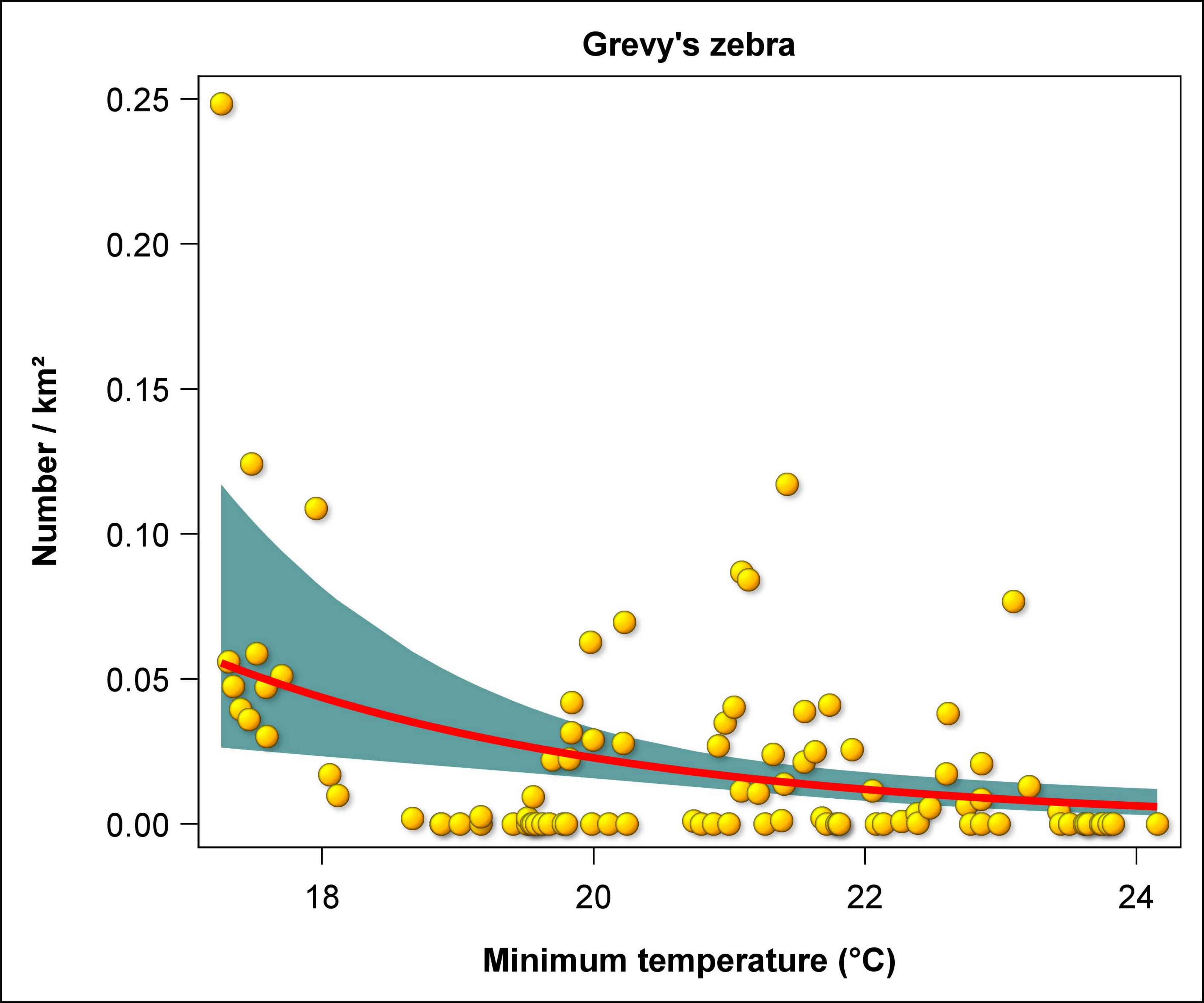

Grevy's zebra
0.25
0.20
0.15
0.10
0.05
0.00
Number / km²
18
20
22
24
Minimum temperature (°C)

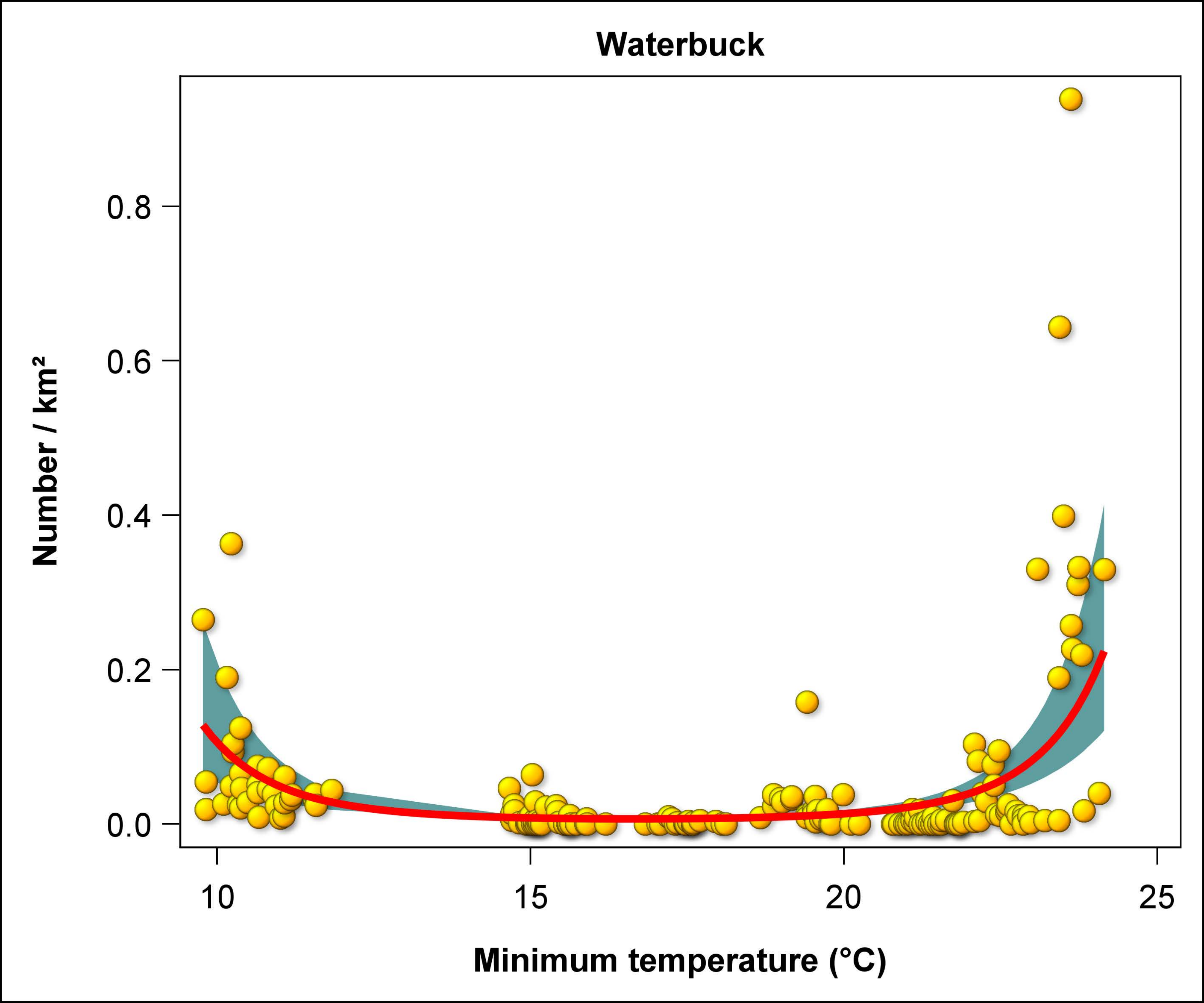
Waterbuck
Number / km²
0.0
0.2
0.4
0.6
0.8
10
15
20
25
Minimum temperature (°C)
