# Supplementary material for: Extreme Wildlife Declines and Concurrent Increase in Livestock Numbers in Kenya: What Are the Causes?
Source: PLoS One. 2016 Sep 27;11(9):e0163249. doi: 10.1371/journal.pone.0163249 (PMC5039022; doi:10.1371/journal.pone.0163249)
Supplement: S32 Fig — (PDF) [file pone.0163249.s042.pdf]

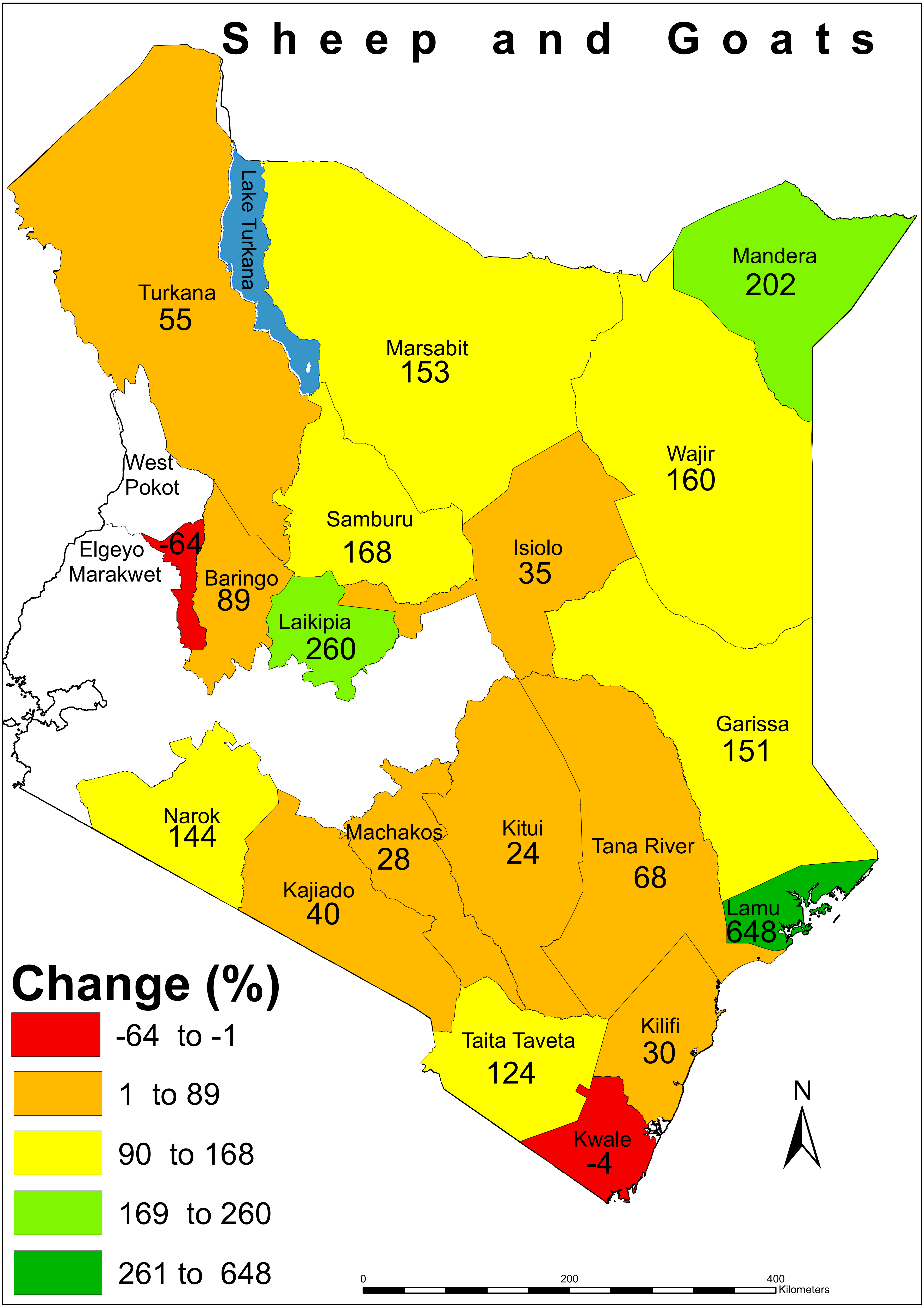
Sheep and Goats
Lake Turkana
Turkana 55
Marsabit 153
Mandera 202
Wajir 160
West Pokot
Samburu 168
Elgeyo Marakwet -64
Baringo 89
Isiolo 35
Laikipia 260
Garissa 151
Narok 144
Machakos 28
Kitui 24
Tana River 68
Kajiado 40
Lamu 648
Kilifi 30
Taita Taveta 124
Kwale -4
Change (%)
-64 to -1
1 to 89
90 to 168
169 to 260
261 to 648
N
0
200
400
Kilometers

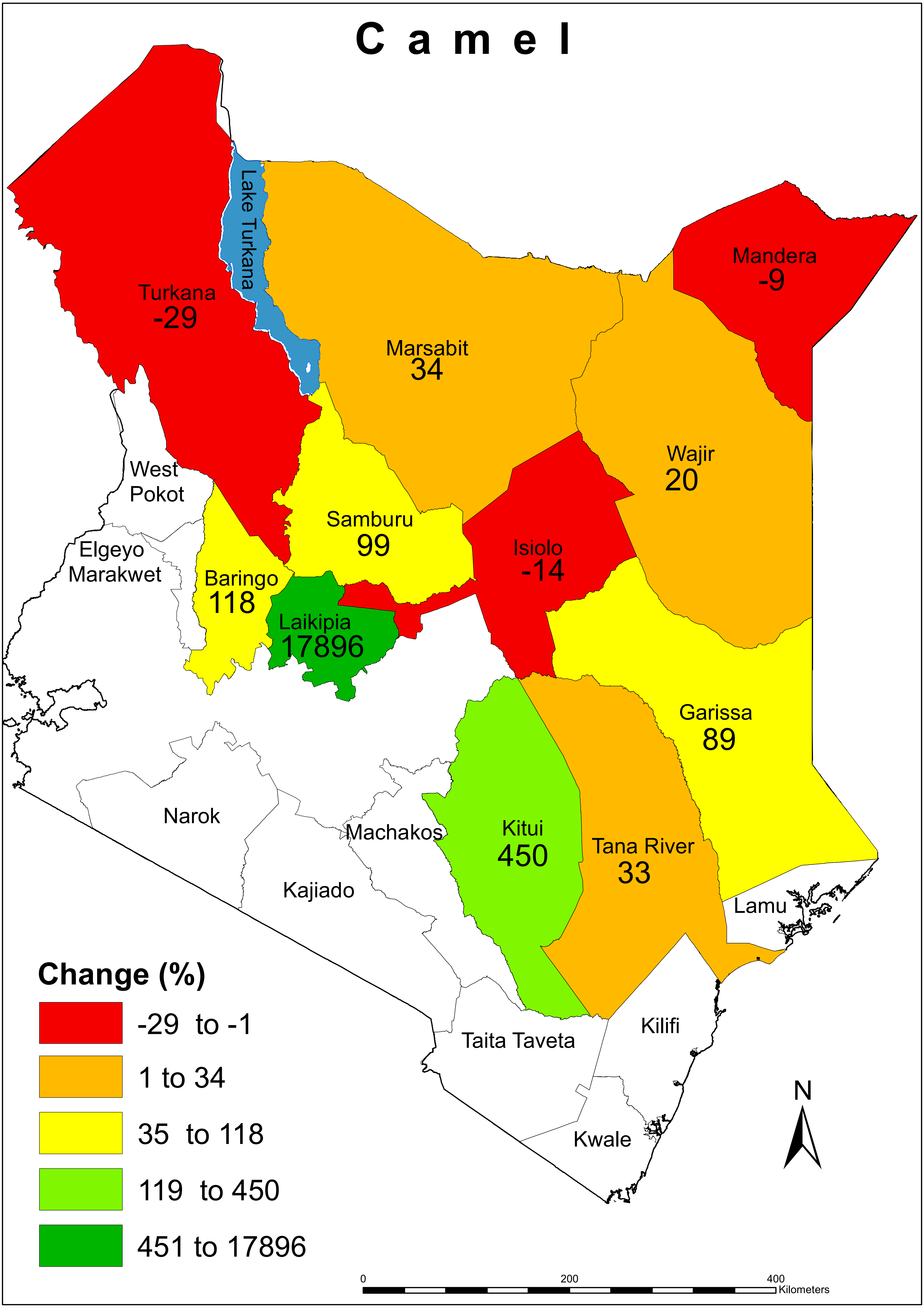
Camel
Lake Turkana
Turkana
-29
Marsabit
34
Mandera
-9
Wajir
20
West
Pokot
Samburu
99
Isiolo
-14
Elgeyo
Marakwet
Baringo
118
Laikipia
17896
Garissa
89
Narok
Machakos
Kitui
450
Tana River
33
Kajiado
Lamu
Change (%)
-29 to -1
1 to 34
35 to 118
119 to 450
451 to 17896
Taita Taveta
Kilifi
Kwale
N
0
200
400
Kilometers

D o n k e y

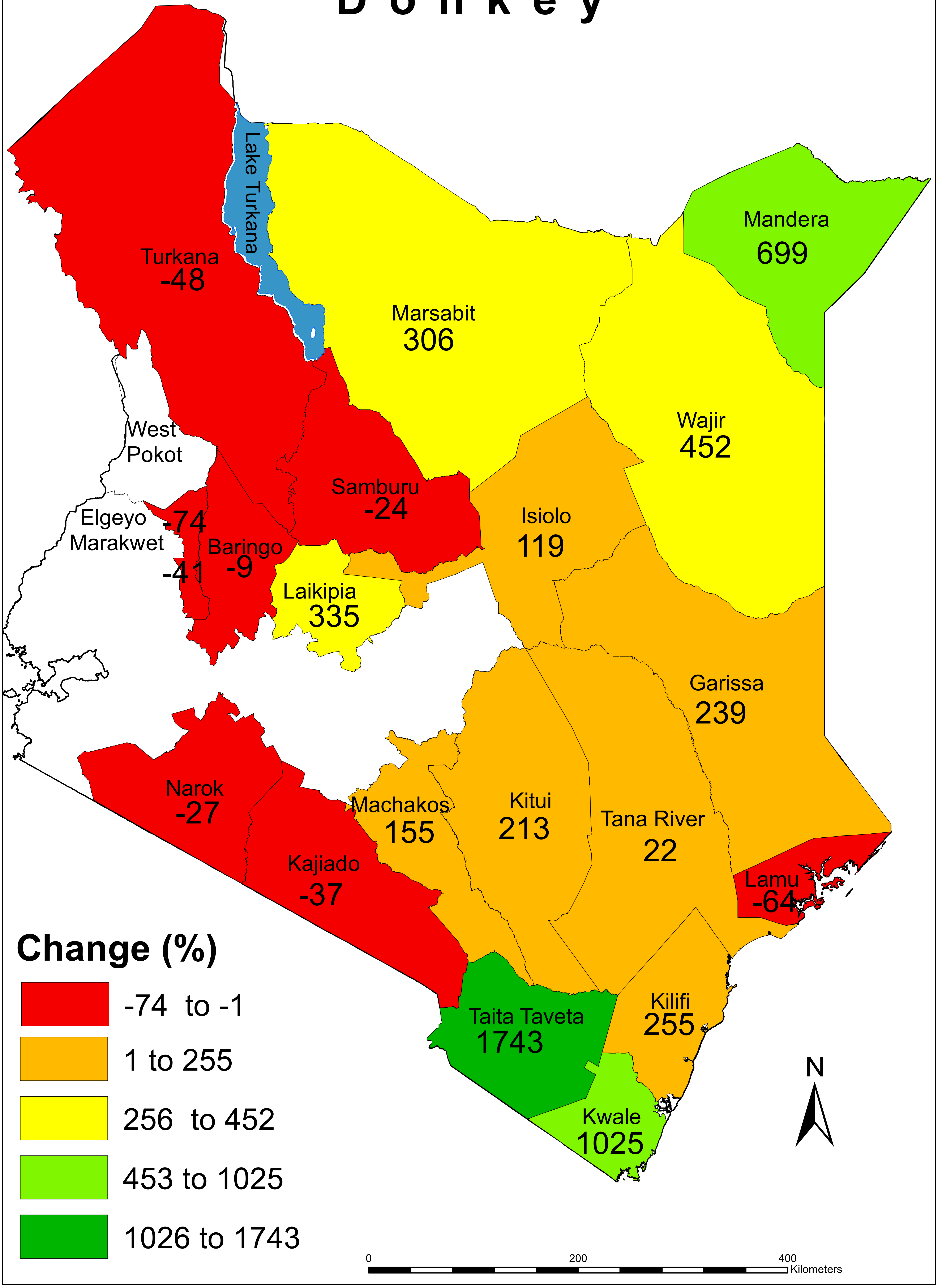
Lake Turkana
Turkana
-48
Marsabit
306
Mandera
699
Wajir
452
West
Pokot
Samburu
-24
Elgeyo
Marakwet
-74
-41
Baringo
-9
Isiolo
119
Laikipia
335
Garissa
239
Narok
-27
Machakos
155
Kitui
213
Tana River
22
Kajiado
-37
Lamu
-64
Taita Taveta
1743
Kilifi
255
Kwale
1025
Change (%)
-74 to -1
1 to 255
256 to 452
453 to 1025
1026 to 1743
N
0
200
400
Kilometers

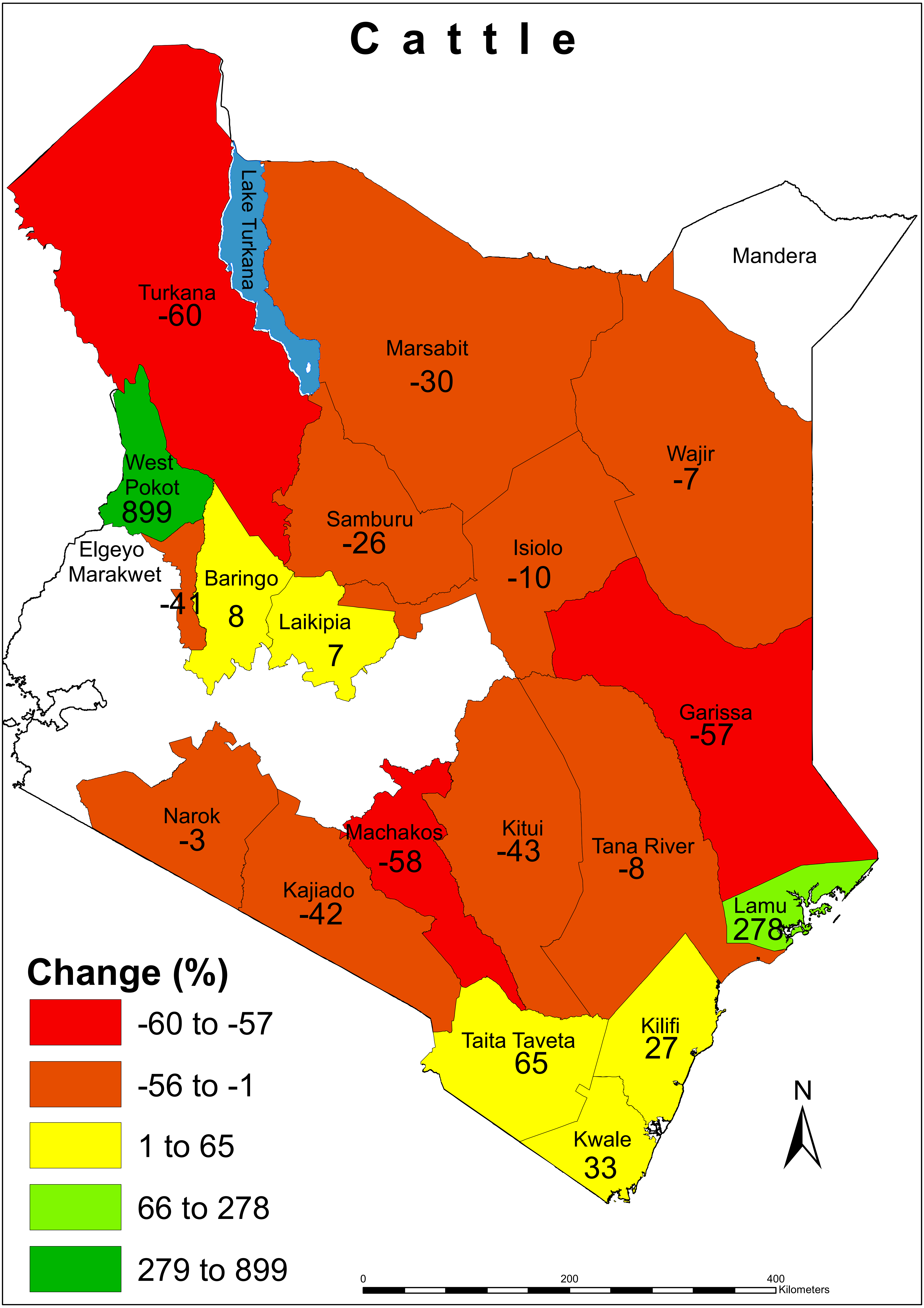

Cattle
Lake Turkana
Turkana
-60
Mandera
Marsabit
-30
Wajir
-7
West
Pokot
899
Samburu
-26
Isiolo
-10
Elgeyo
Marakwet
-41
Baringo
8
Laikipia
7
Garissa
-57
Narok
-3
Machakos
-58
Kitui
-43
Tana River
-8
Kajiado
-42
Lamu
278
Change (%)
-60 to -57
-56 to -1
1 to 65
66 to 278
279 to 899
Taita Taveta
65
Kilifi
27
Kwale
33
N
0
200
400
Kilometers

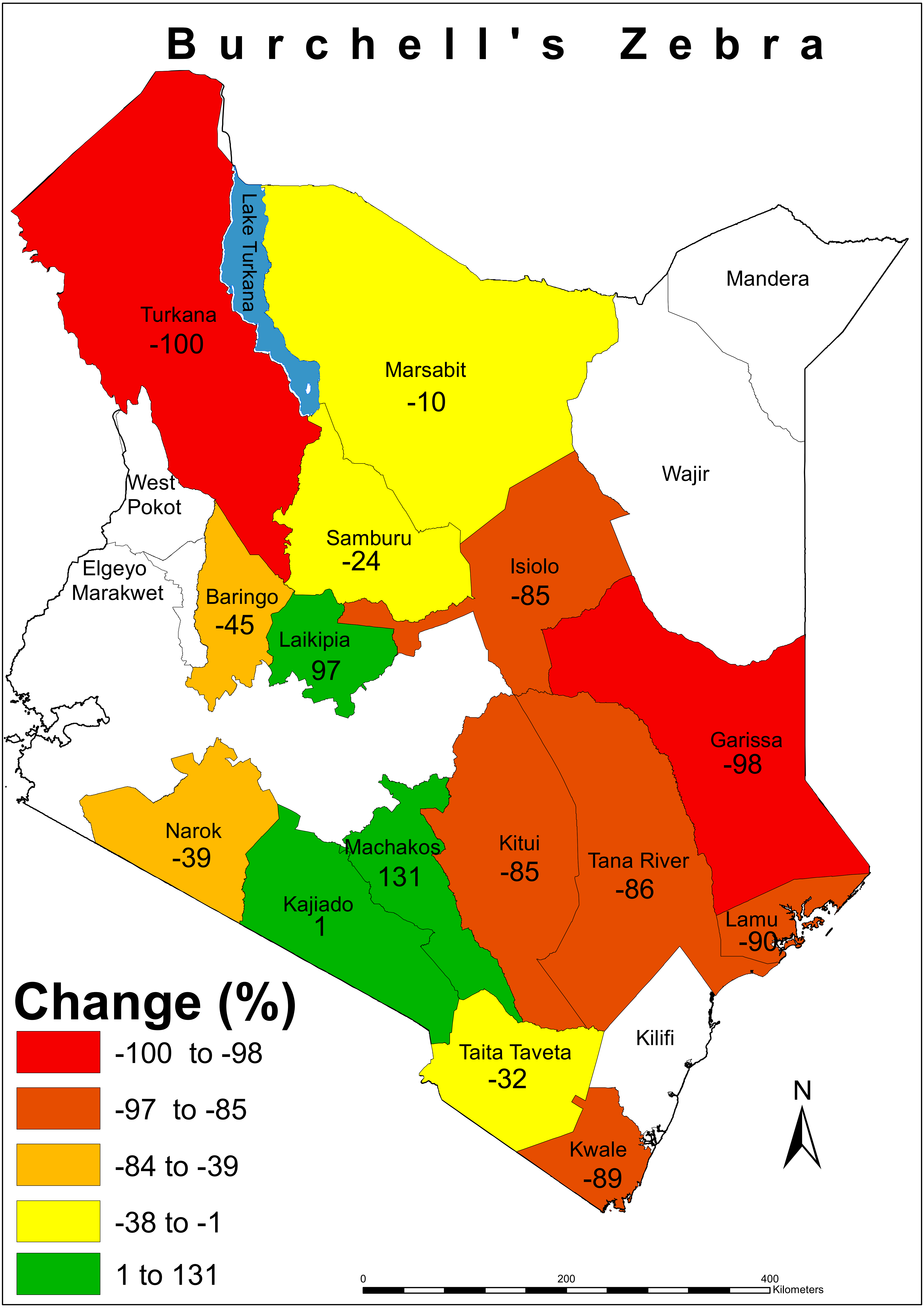
Burchell's Zebra
Lake Turkana
Turkana
-100
Marsabit
-10
Mandera
Wajir
West
Pokot
Samburu
-24
Elgeyo
Marakwet
Baringo
-45
Isiolo
-85
Laikipia
97
Garissa
-98
Narok
-39
Machakos
131
Kitui
-85
Tana River
-86
Kajiado
1
Lamu
-90
Kilifi
Taita Taveta
-32
Kwale
-89
N
Change (%)
-100 to -98
-97 to -85
-84 to -39
-38 to -1
1 to 131
0
200
400
Kilometers

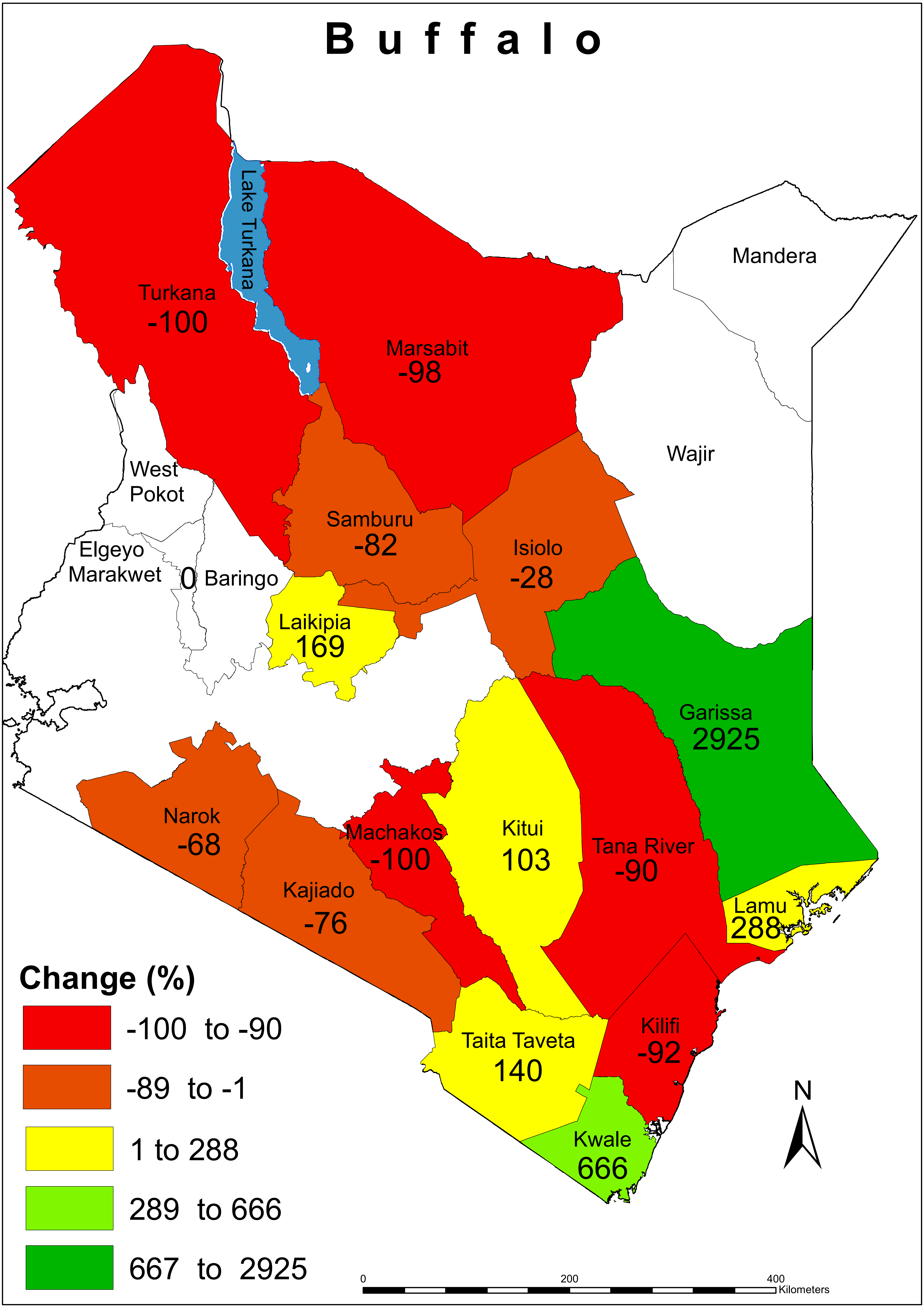
Buffalo
Lake Turkana
Turkana
-100
Marsabit
-98
Mandera
Wajir
West
Pokot
Samburu
-82
Isiolo
-28
Elgeyo
Marakwet
0
Baringo
Laikipia
169
Garissa
2925
Narok
-68
Machakos
-100
Kitui
103
Tana River
-90
Kajiado
-76
Lamu
288
Kilifi
-92
Taita Taveta
140
Kwale
666
N
Change (%)
-100 to -90
-89 to -1
1 to 288
289 to 666
667 to 2925
0
200
400
Kilometers

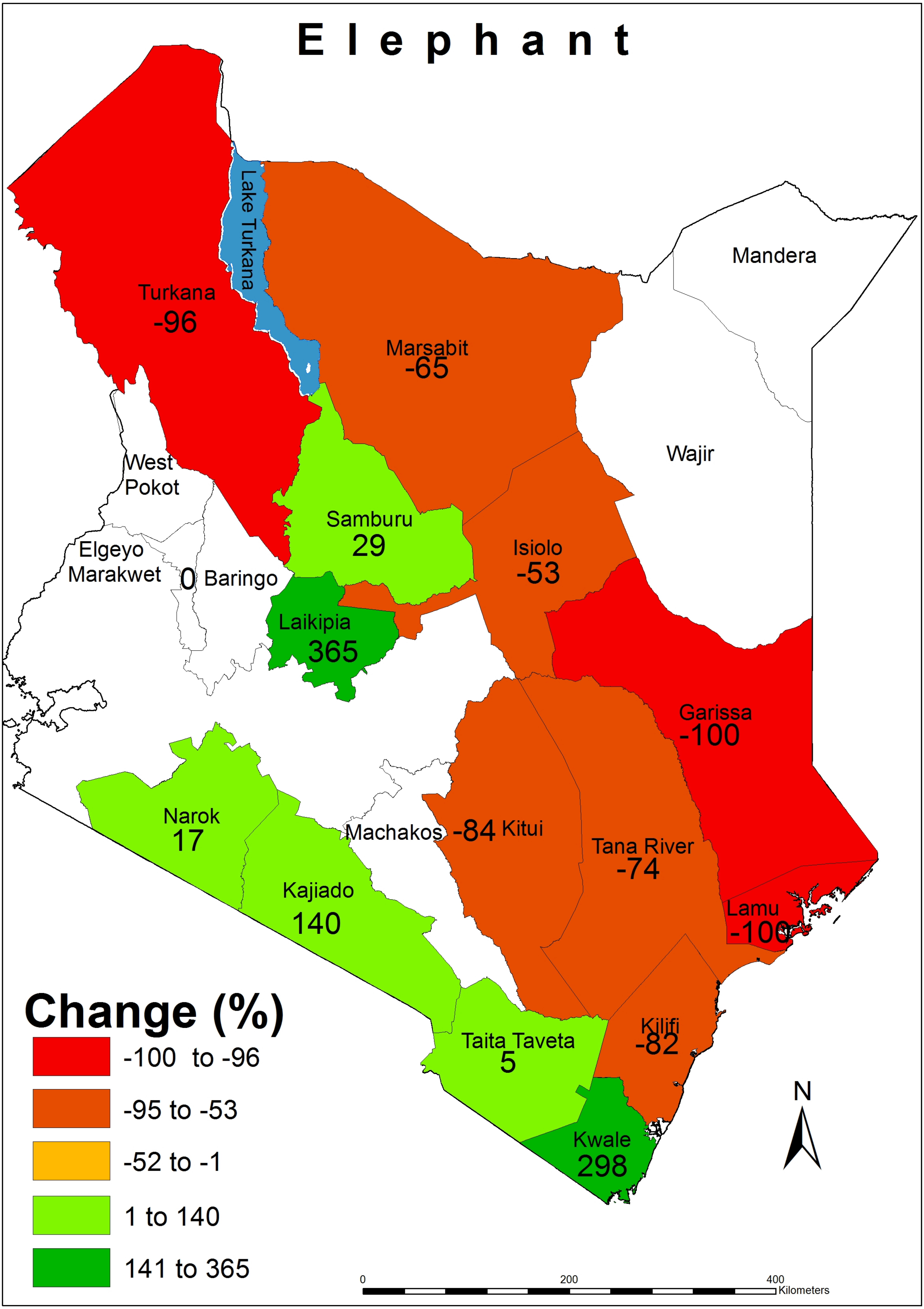

Elephant
Lake Turkana
Turkana
-96
Marsabit
-65
Mandera
Wajir
West
Pokot
Samburu
29
Elgeyo
Marakwet
0
Baringo
Isiolo
-53
Laikipia
365
Garissa
-100
Narok
17
Machakos
-84
Kitui
Tana River
-74
Kajiado
140
Lamu
-100
Taita Taveta
5
Kilifi
-82
Kwale
298
Change (%)
-100 to -96
-95 to -53
-52 to -1
1 to 140
141 to 365
N
0
200
400
Kilometers

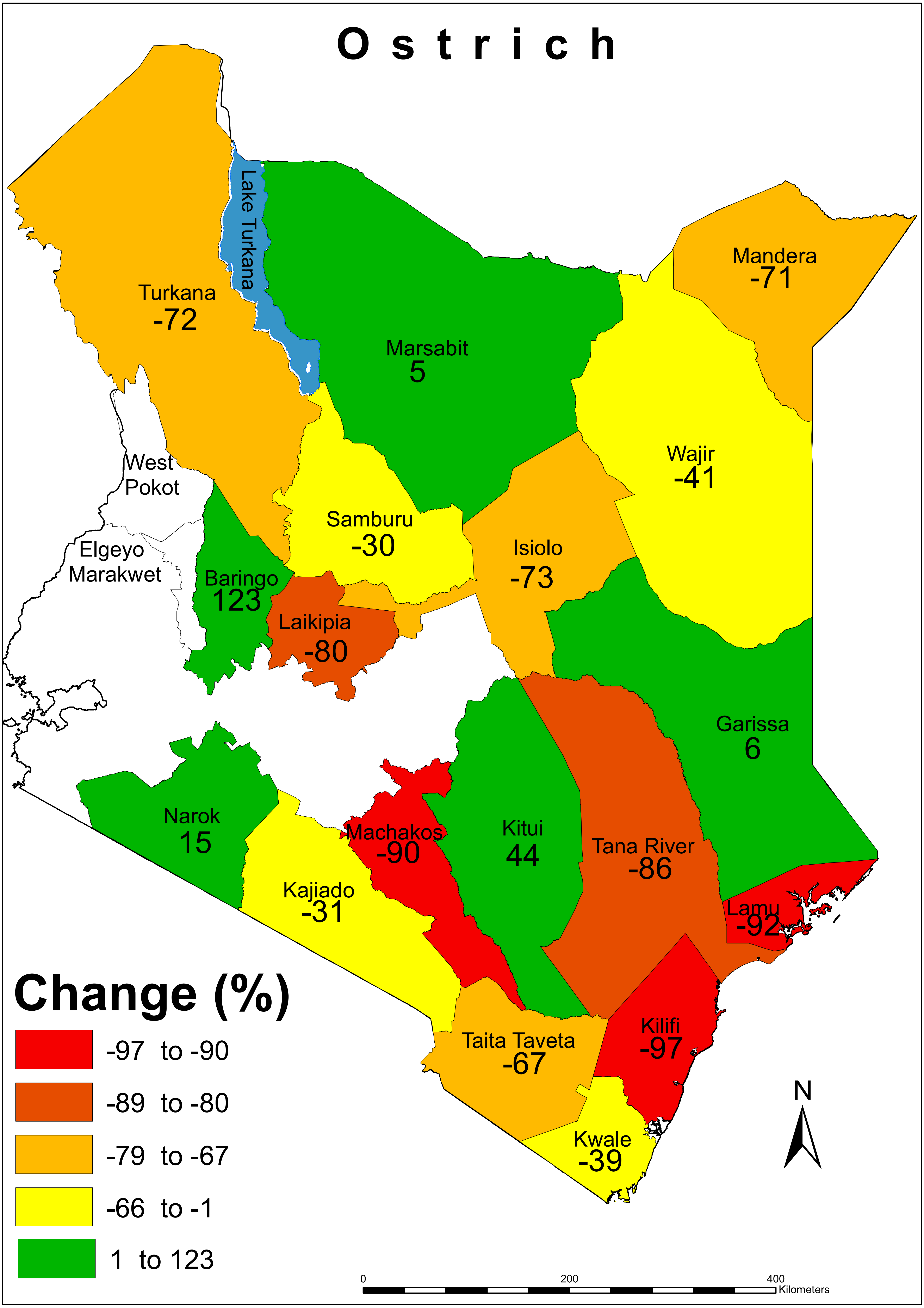
Ostrich
Lake Turkana
Turkana
-72
Marsabit
5
Mandera
-71
Wajir
-41
West
Pokot
Samburu
-30
Isiolo
-73
Elgeyo
Marakwet
Baringo
123
Laikipia
-80
Garissa
6
Narok
15
Machakos
-90
Kitui
44
Tana River
-86
Kajiado
-31
Lamu
-92
Kilifi
-97
Taita Taveta
-67
Kwale
-39
Change (%)
-97 to -90
-89 to -80
-79 to -67
-66 to -1
1 to 123
N
0
200
400
Kilometers

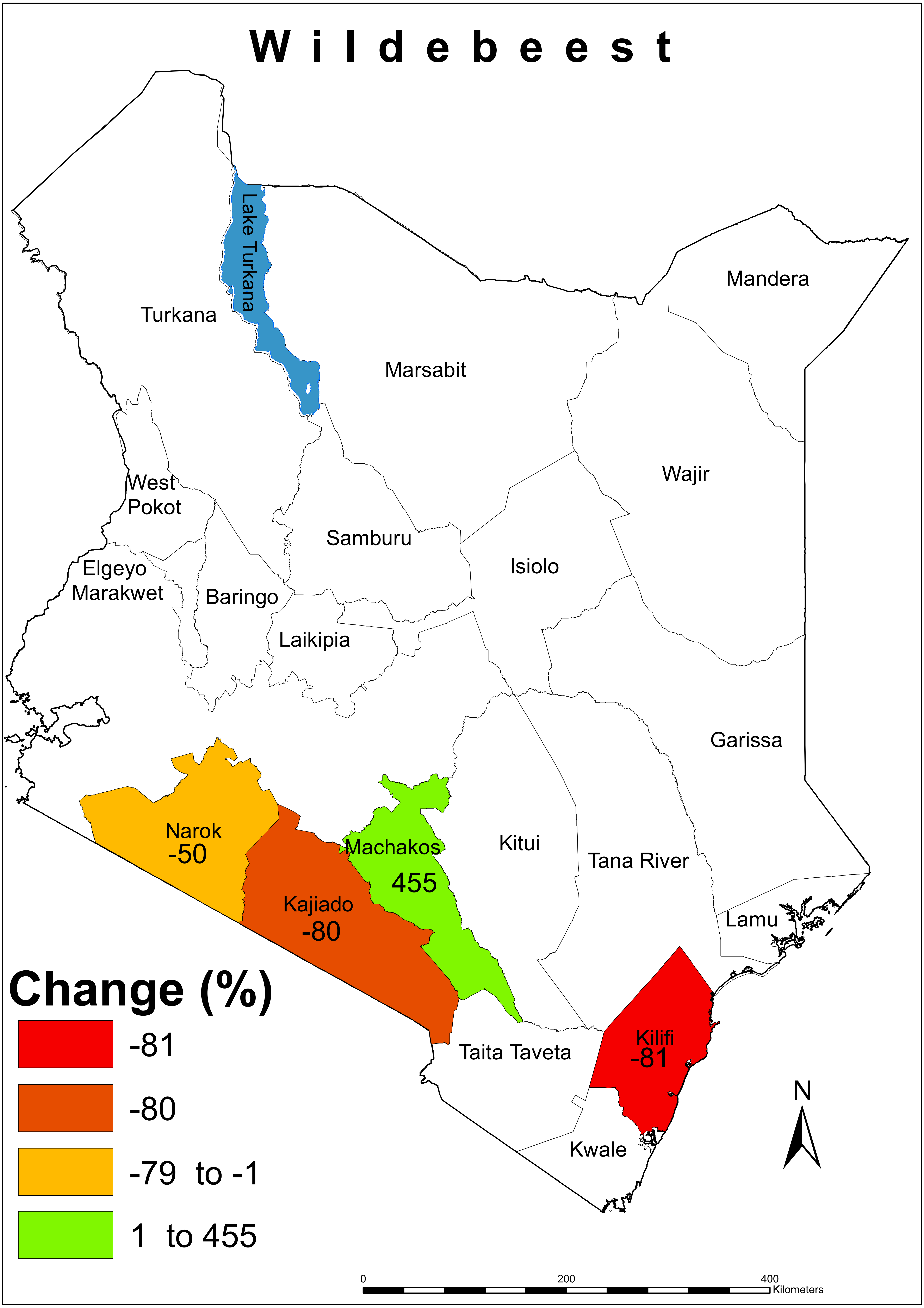

Wildebeest
Lake Turkana
Turkana
Marsabit
Mandera
Wajir
West Pokot
Samburu
Isiolo
Elgeyo Marakwet
Baringo
Laikipia
Garissa
Narok -50
Machakos 455
Kitui
Tana River
Kajiado -80
Lamu
Kilifi -81
Taita Taveta
Kwale
N
Change (%)
-81
-80
-79 to -1
1 to 455
0
200
400
Kilometers

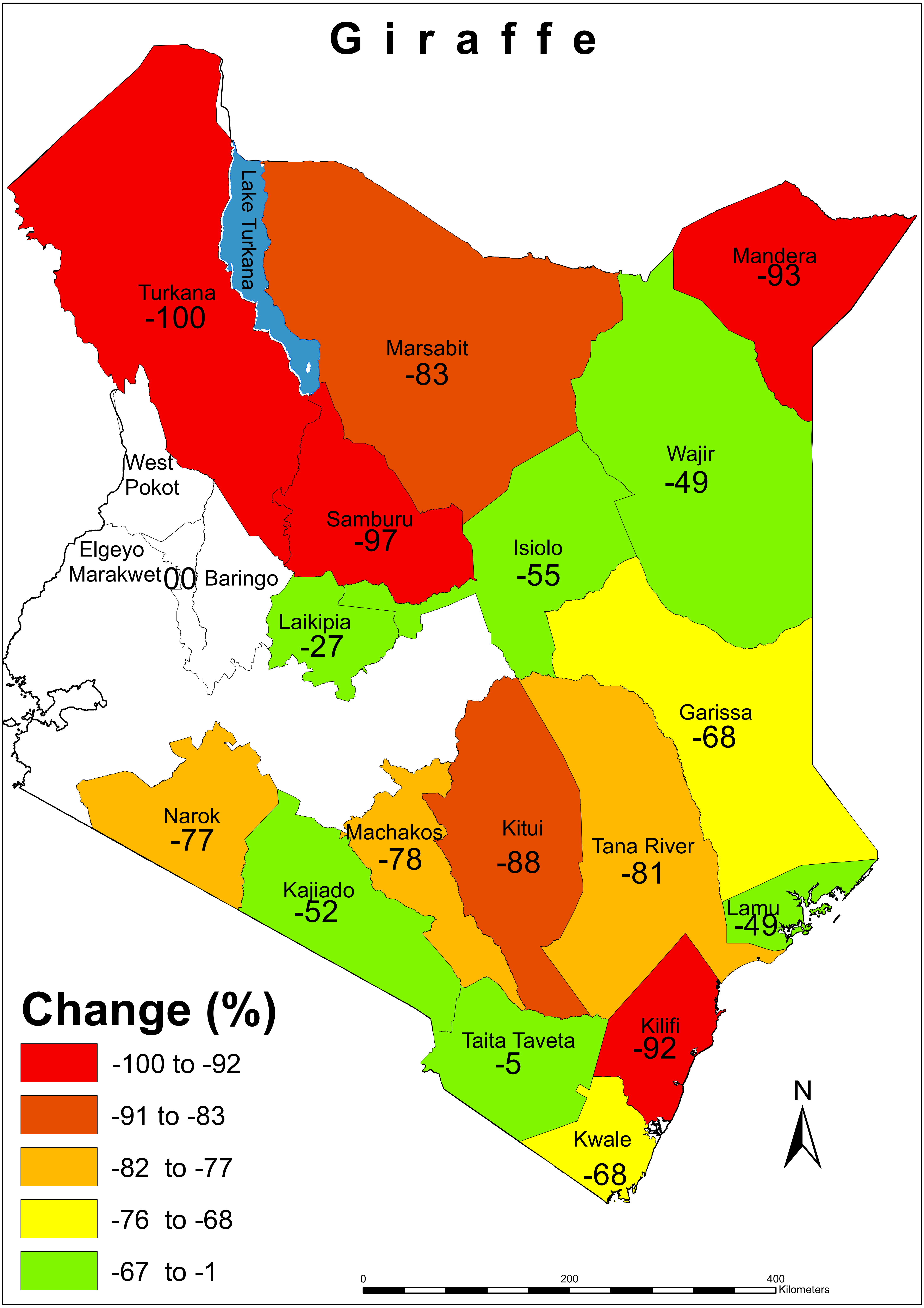
Giraffe
Lake Turkana
Turkana
-100
Marsabit
-83
Mandera
-93
Wajir
-49
West
Pokot
Samburu
-97
Isiolo
-55
Elgeyo
Marakwet
00
Baringo
Laikipia
-27
Garissa
-68
Narok
-77
Machakos
-78
Kitui
-88
Tana River
-81
Kajiado
-52
Lamu
-49
Kilifi
-92
Taita Taveta
-5
Kwale
-68
Change (%)
-100 to -92
-91 to -83
-82 to -77
-76 to -68
-67 to -1
N
0
200
400
Kilometers

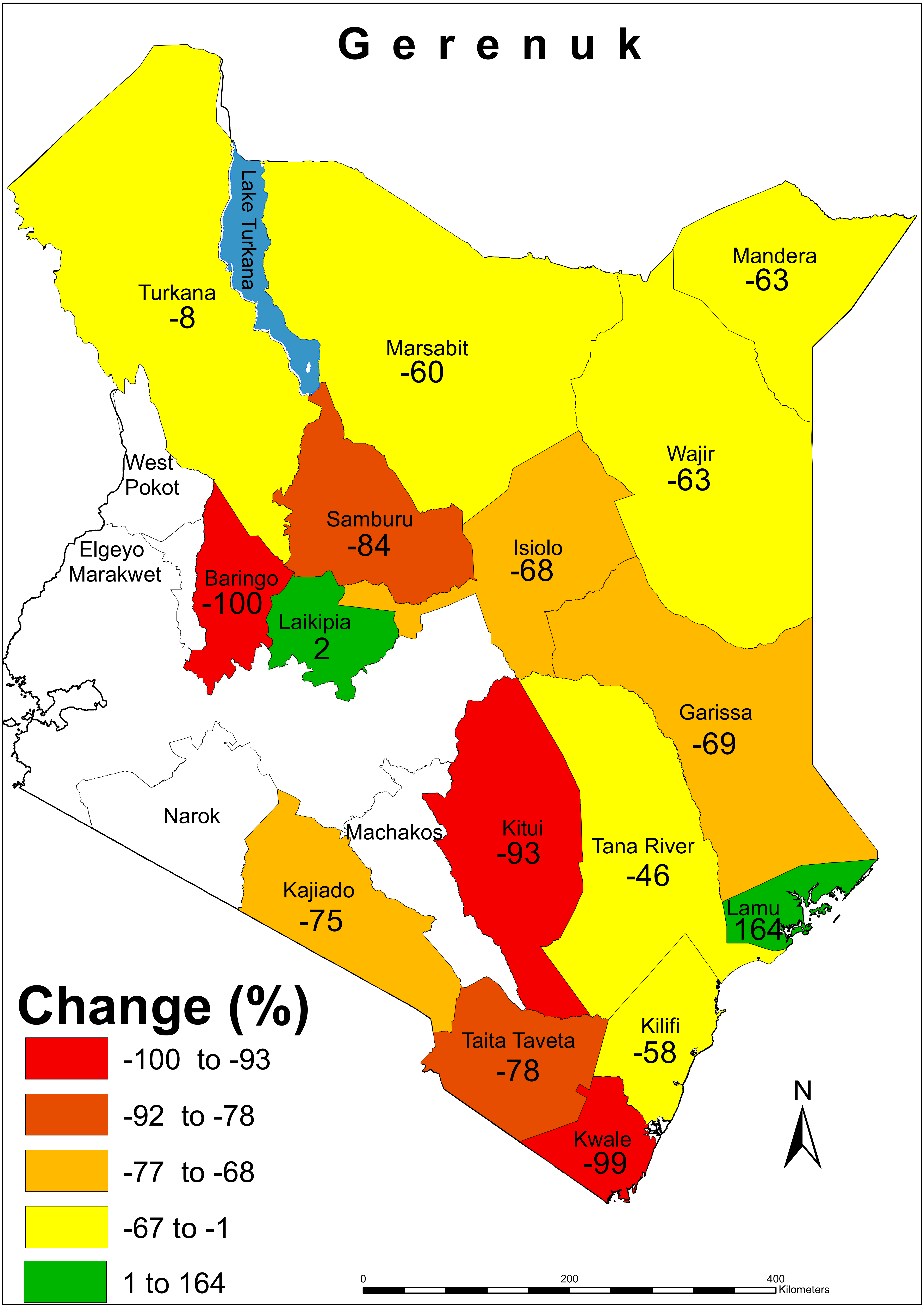
Gerenuk
Lake Turkana
Turkana
-8
Marsabit
-60
Mandera
-63
Wajir
-63
West
Pokot
Samburu
-84
Isiolo
-68
Elgeyo
Marakwet
Baringo
-100
Laikipia
2
Garissa
-69
Narok
Machakos
Kitui
-93
Tana River
-46
Kajiado
-75
Lamu
164
Kilifi
-58
Taita Taveta
-78
Kwale
-99
Change (%)
-100 to -93
-92 to -78
-77 to -68
-67 to -1
1 to 164
N
0
200
400
Kilometers

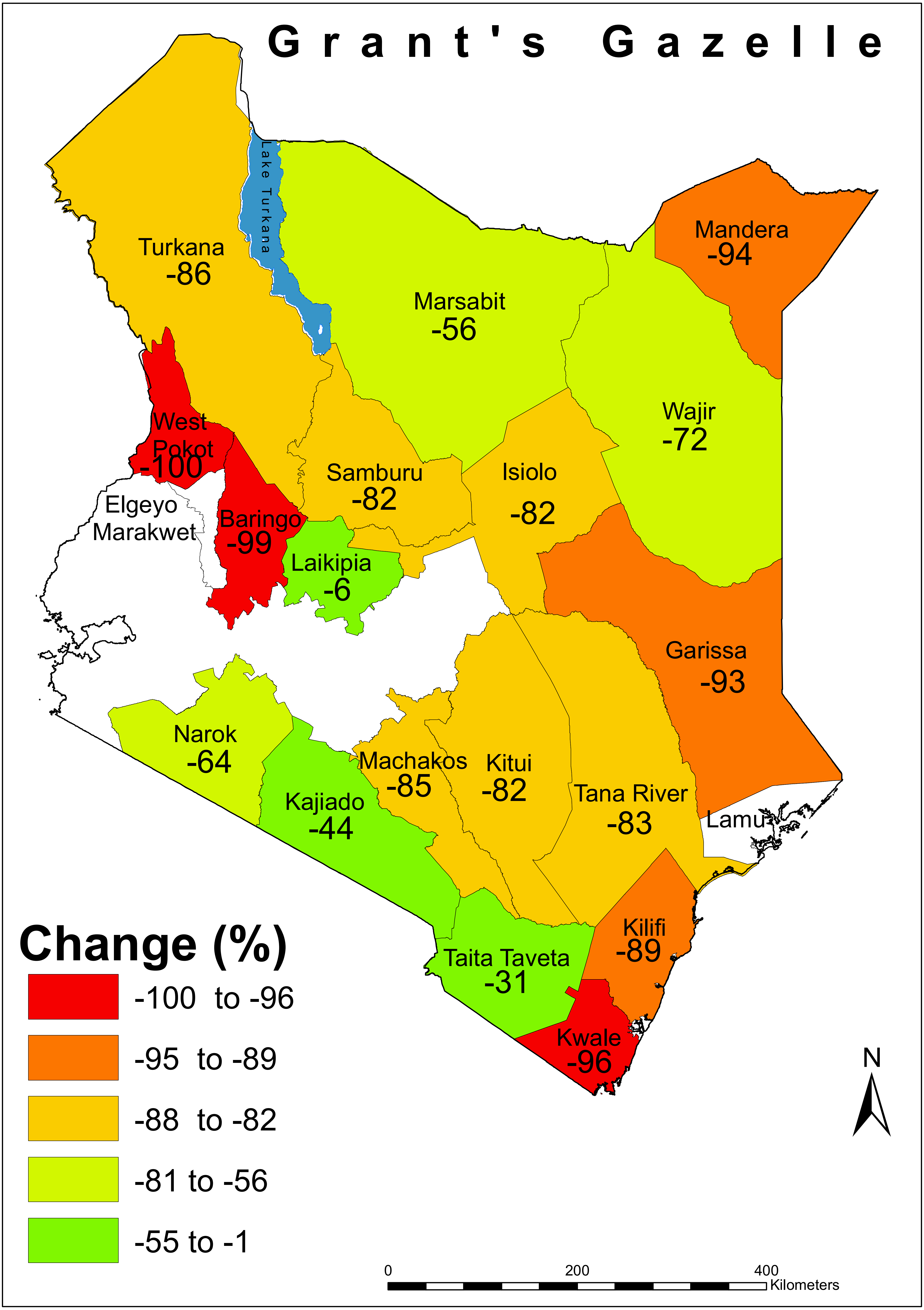
Grant's Gazelle
Lake Turkana
Turkana
-86
Marsabit
-56
Mandera
-94
Wajir
-72
West
Pokot
-100
Samburu
-82
Isiolo
-82
Elgeyo
Marakwet
Baringo
-99
Laikipia
-6
Garissa
-93
Narok
-64
Machakos
-85
Kitui
-82
Tana River
-83
Lamu
Kajiado
-44
Kilifi
-89
Taita Taveta
-31
Kwale
-96
Change (%)
-100 to -96
-95 to -89
-88 to -82
-81 to -56
-55 to -1
N
0
200
400
Kilometers

Warthog

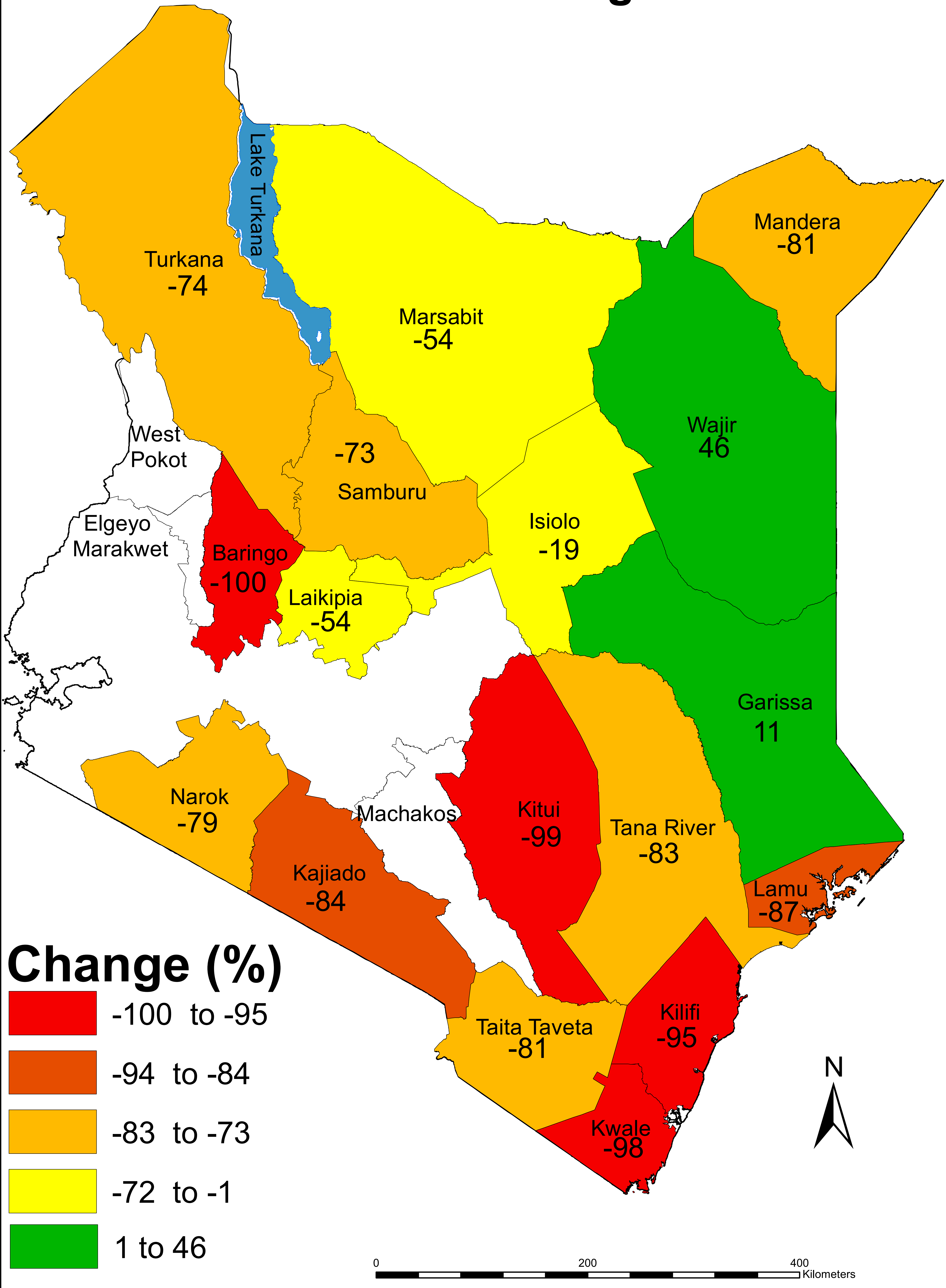
Lake Turkana
Turkana
-74
Marsabit
-54
Mandera
-81
Wajir
46
West
Pokot
-73
Samburu
Isiolo
-19
Elgeyo
Marakwet
Baringo
-100
Laikipia
-54
Garissa
11
Narok
-79
Machakos
Kitui
-99
Tana River
-83
Kajiado
-84
Lamu
-87
Kilifi
-95
Taita Taveta
-81
Kwale
-98
Change (%)
-100 to -95
-94 to -84
-83 to -73
-72 to -1
1 to 46
N
0
200
400
Kilometers

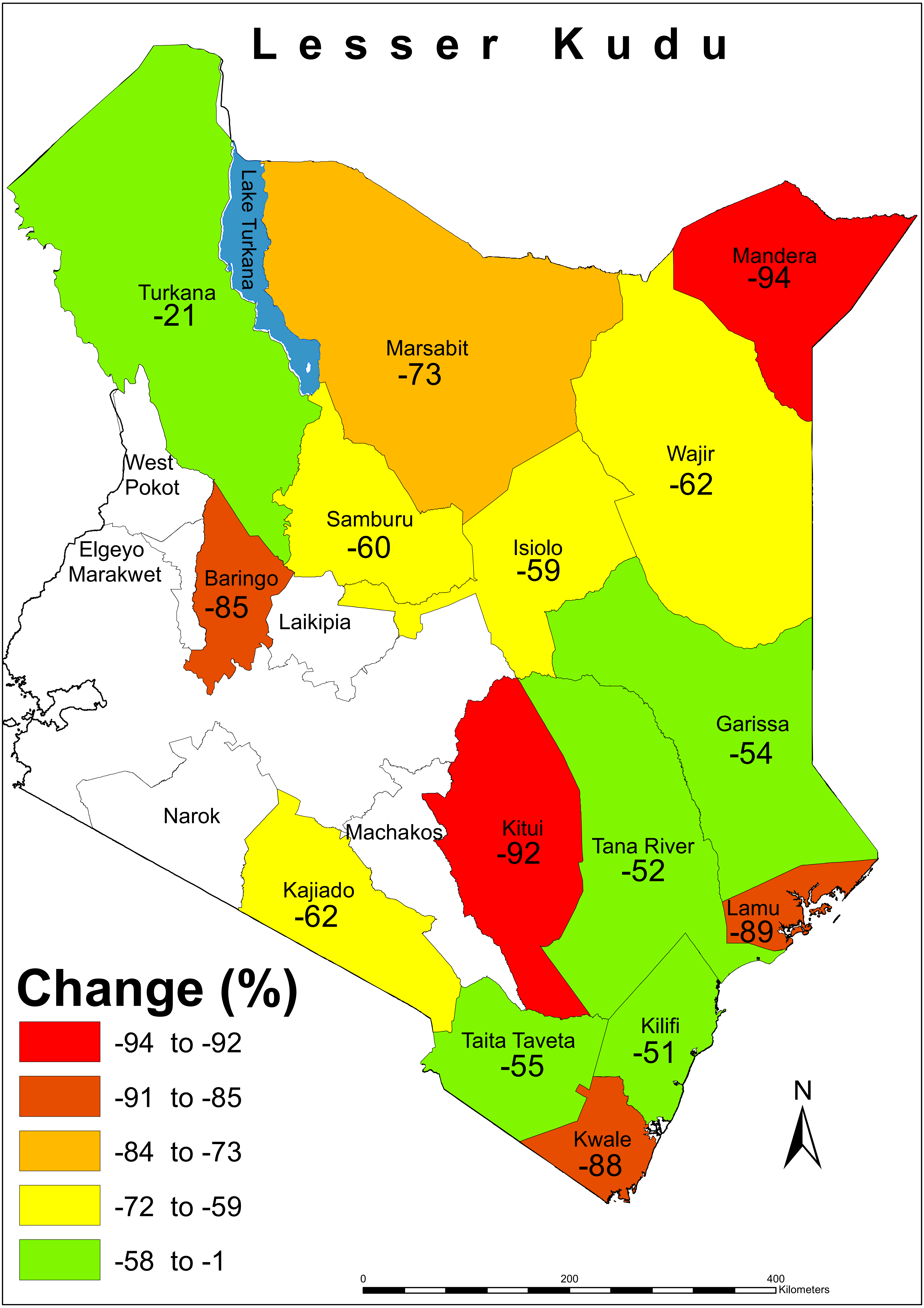
Lesser Kudu
Lake Turkana
Turkana
-21
Marsabit
-73
Mandera
-94
Wajir
-62
West
Pokot
Samburu
-60
Isiolo
-59
Elgeyo
Marakwet
Baringo
-85
Laikipia
Garissa
-54
Narok
Machakos
Kitui
-92
Tana River
-52
Kajiado
-62
Lamu
-89
Change (%)
-94 to -92
-91 to -85
-84 to -73
-72 to -59
-58 to -1
Taita Taveta
-55
Kilifi
-51
Kwale
-88
N
0
200
400
Kilometers

Thomson's Gazelle

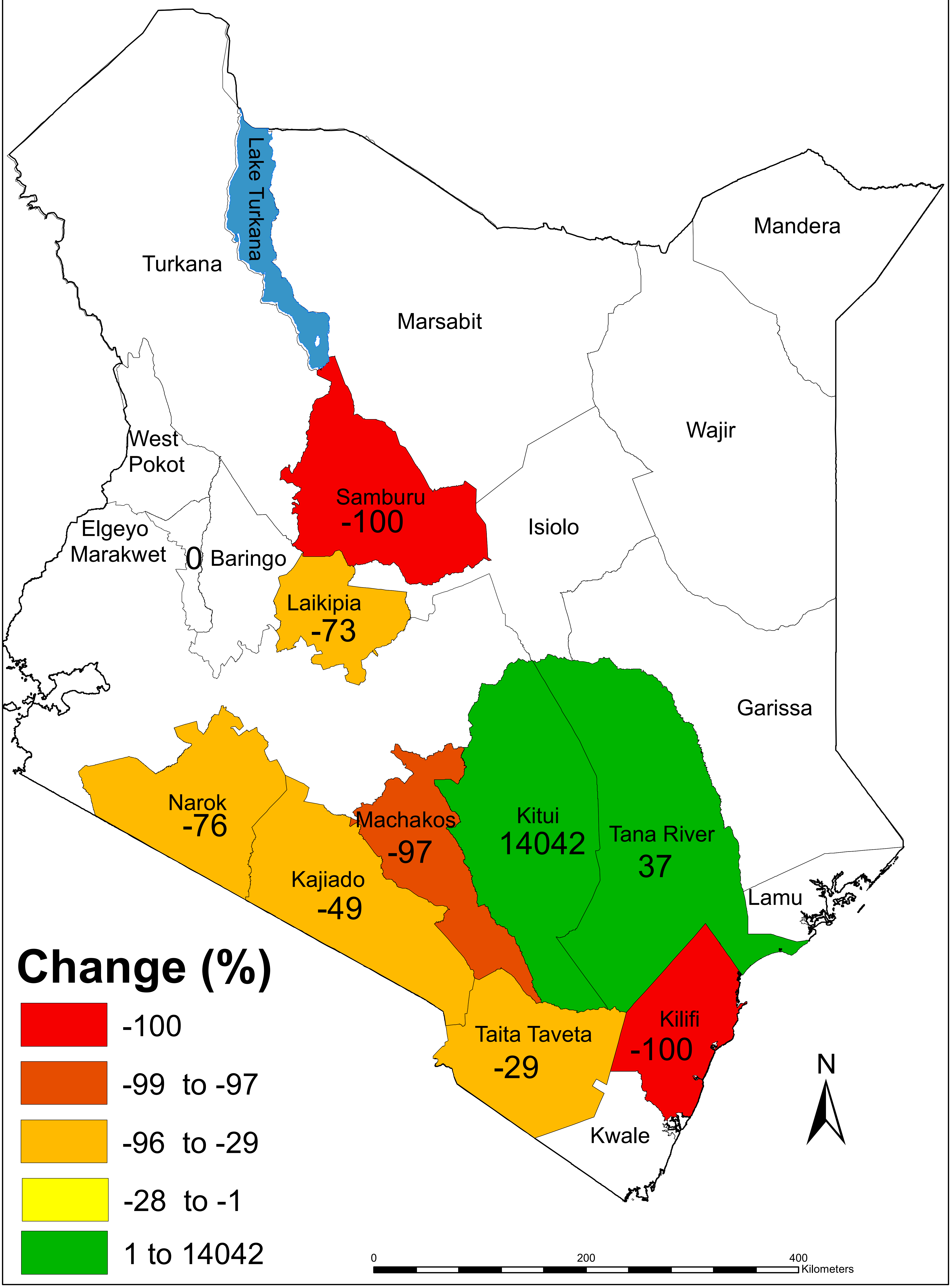

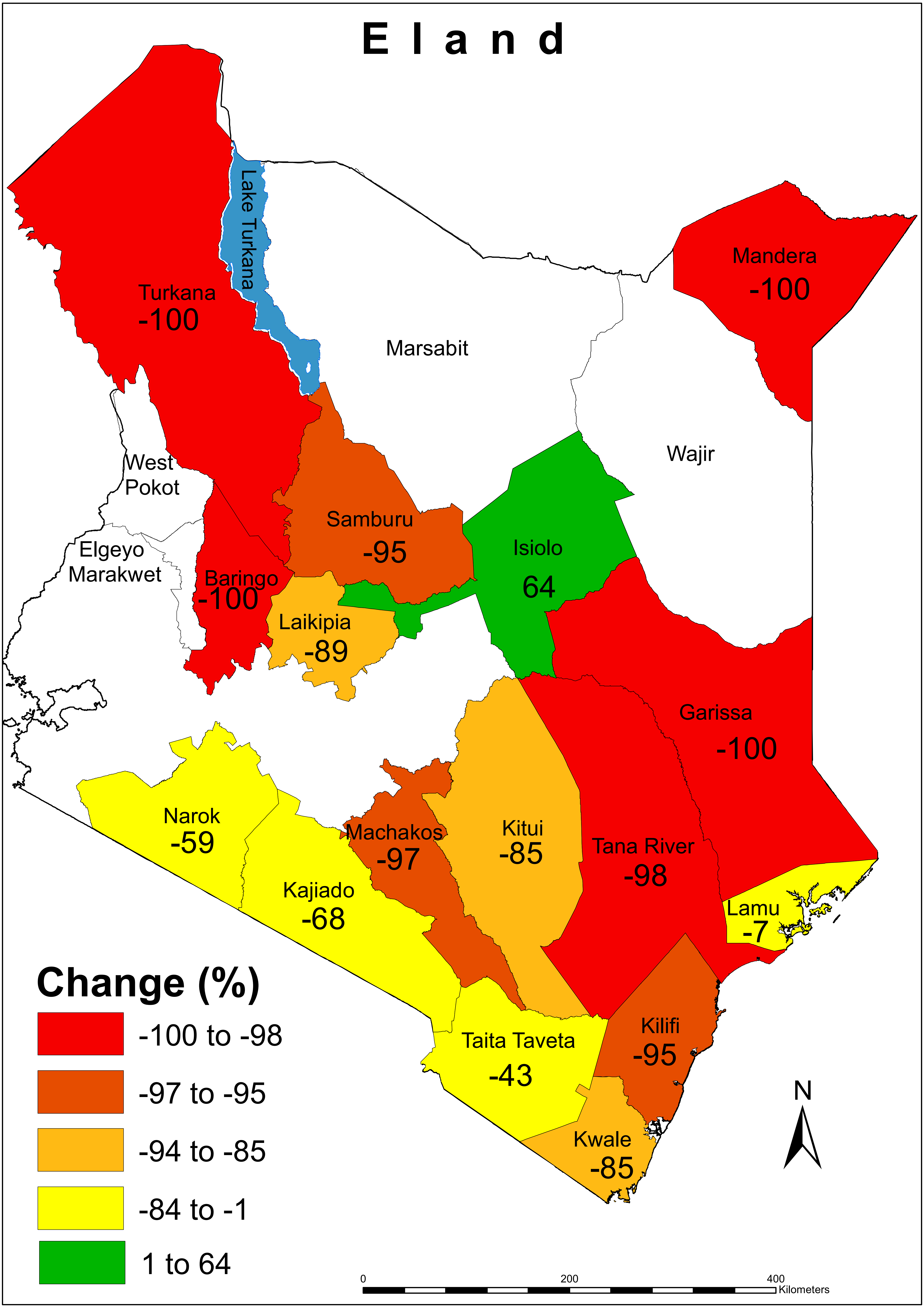
E l a n d
Lake Turkana
Turkana
-100
Mandera
-100
Marsabit
Wajir
West
Pokot
Samburu
-95
Isiolo
64
Elgeyo
Marakwet
Baringo
-100
Laikipia
-89
Garissa
-100
Narok
-59
Machakos
-97
Kitui
-85
Tana River
-98
Kajiado
-68
Lamu
-7
Change (%)
-100 to -98
-97 to -95
-94 to -85
-84 to -1
1 to 64
Kilifi
-95
Taita Taveta
-43
Kwale
-85
N
0
200
400
Kilometers

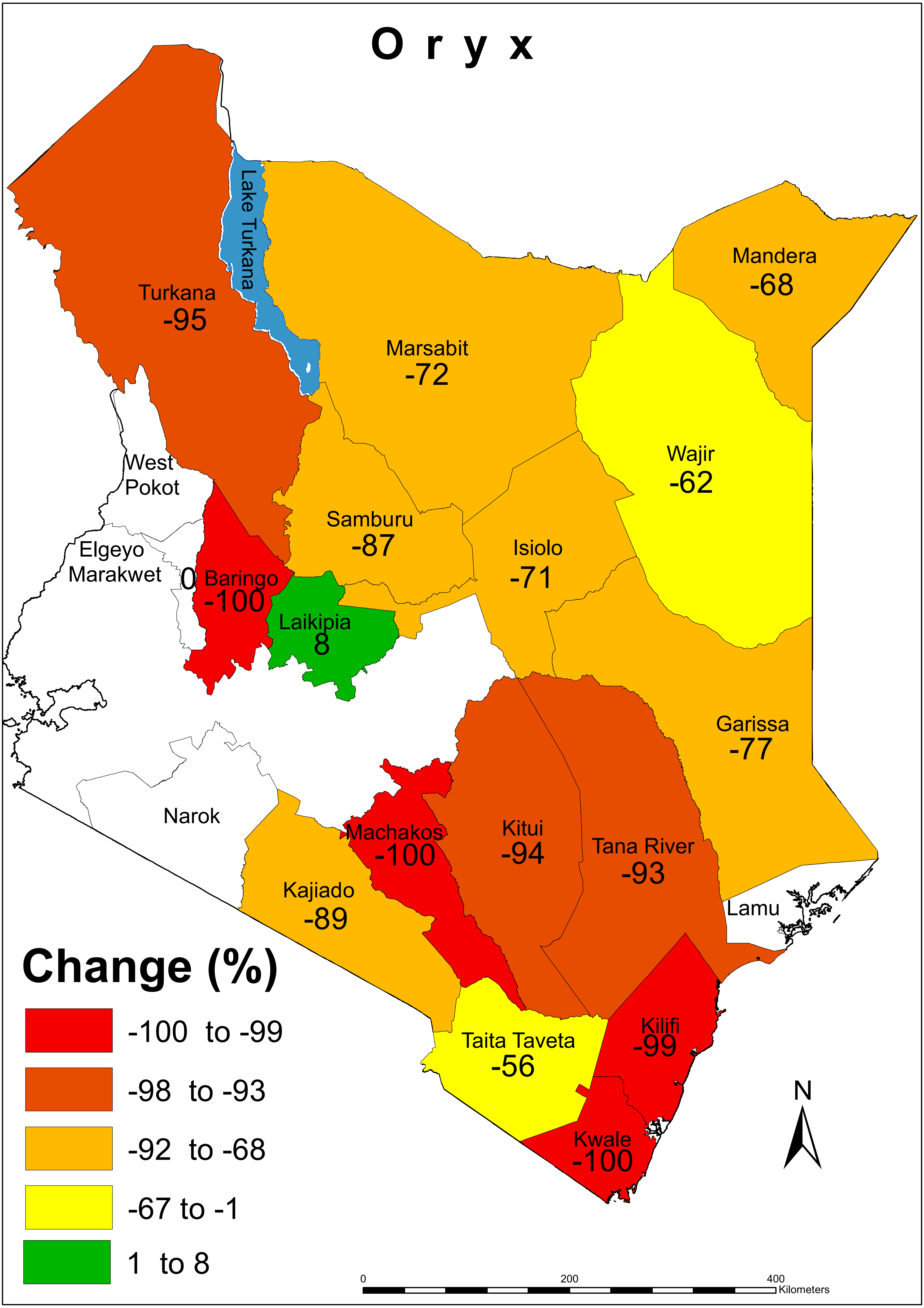

O r y x
Lake Turkana
Turkana
-95
Marsabit
-72
Mandera
-68
Wajir
-62
West
Pokot
Samburu
-87
Isiolo
-71
Elgeyo
Marakwet
0
Baringo
-100
Laikipia
8
Garissa
-77
Narok
Machakos
-100
Kitui
-94
Tana River
-93
Kajiado
-89
Lamu
Change (%)
-100 to -99
-98 to -93
-92 to -68
-67 to -1
1 to 8
Taita Taveta
-56
Kilifi
-99
Kwale
-100
N
0
200
400
Kilometers

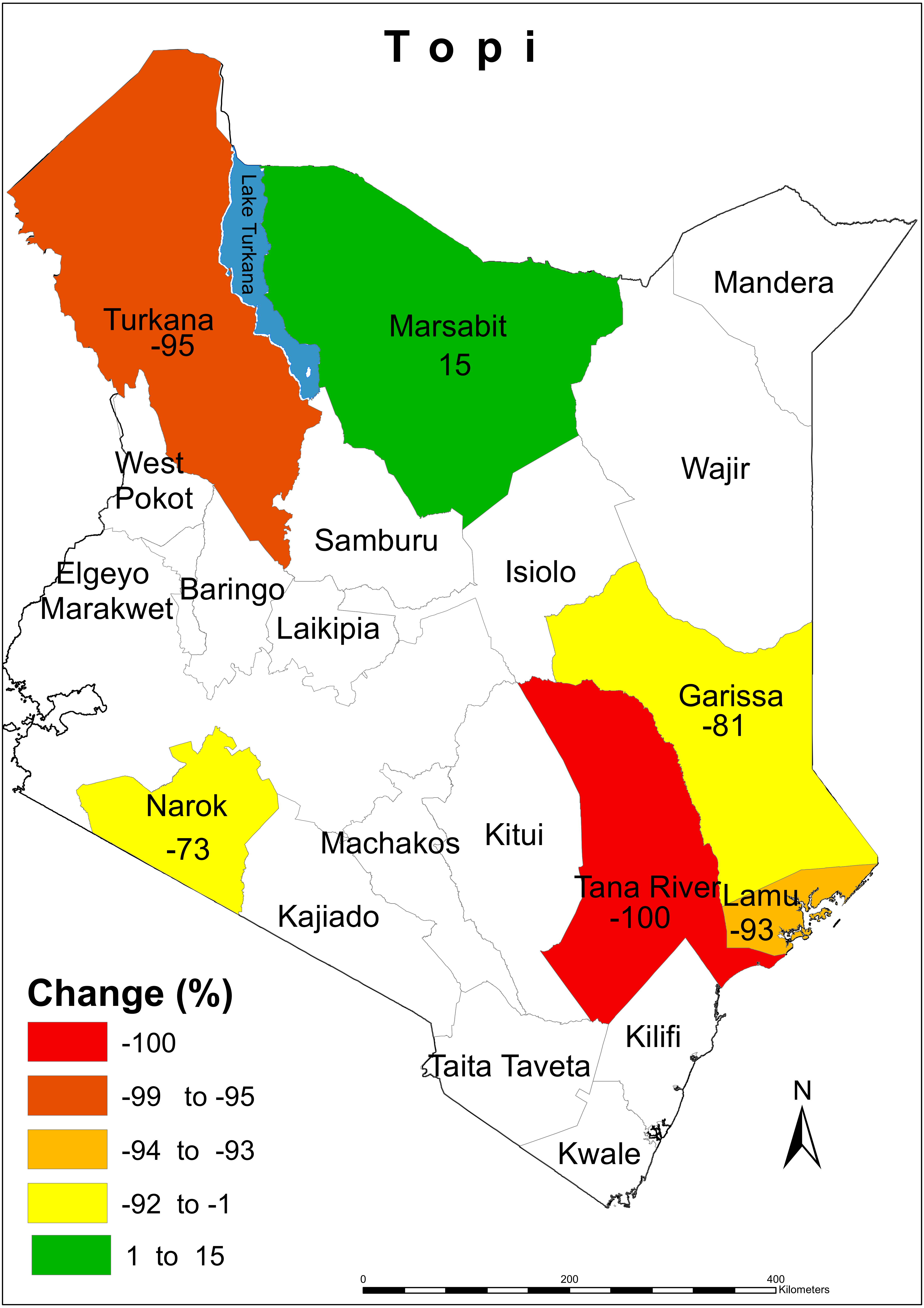
Topi
Lake Turkana
Turkana
-95
Marsabit
15
Mandera
Wajir
West
Pokot
Samburu
Isiolo
Elgeyo
Marakwet
Baringo
Laikipia
Garissa
-81
Narok
-73
Machakos
Kitui
Tana River
-100
Lamu
-93
Kajiado
Kilifi
Taita Taveta
Kwale
Change (%)
-100
-99 to -95
-94 to -93
-92 to -1
1 to 15
N
0
200
400
Kilometers

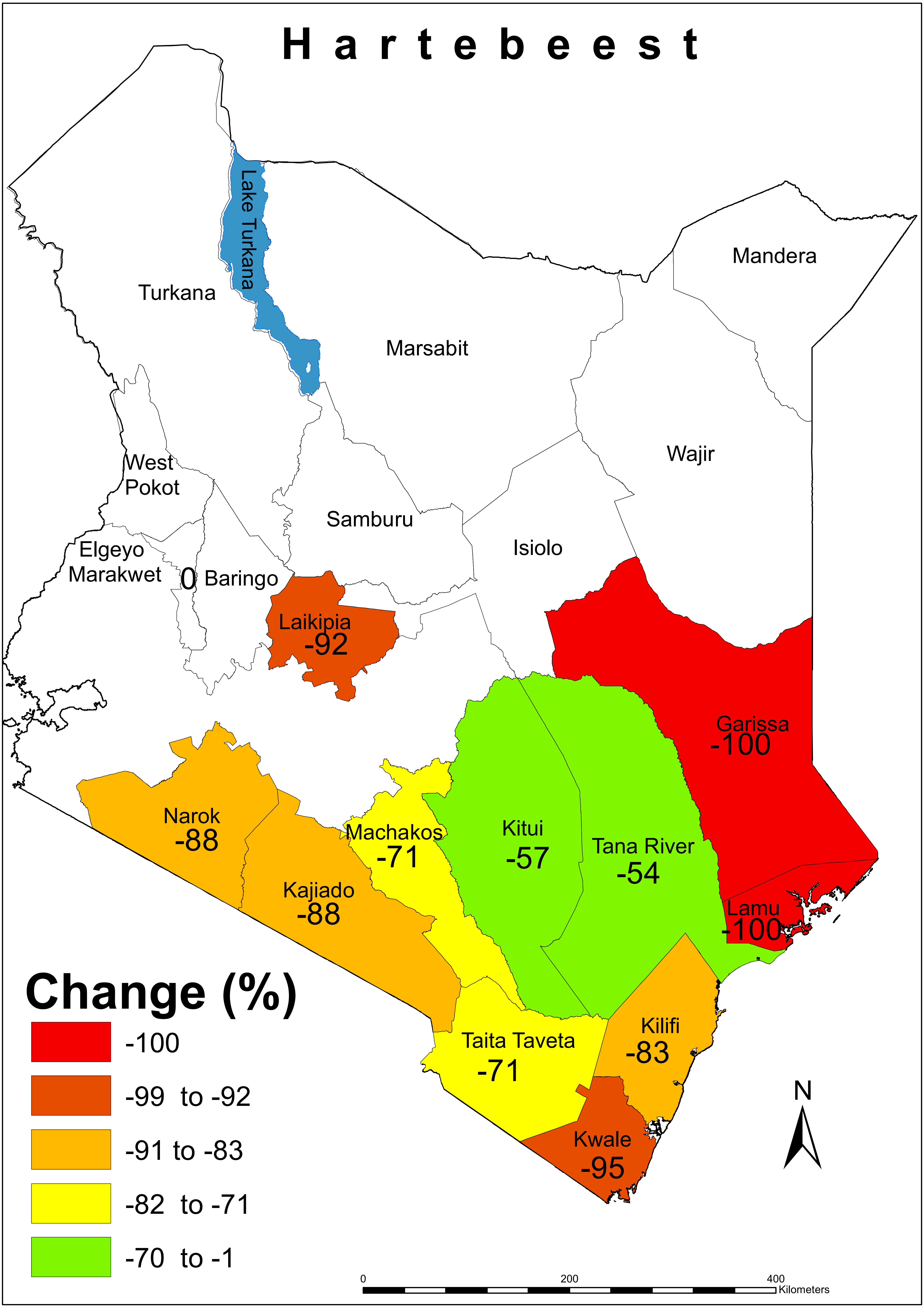

Hartebeest
Lake Turkana
Turkana
Marsabit
Mandera
Wajir
West Pokot
Samburu
Elgeyo Marakwet
0
Baringo
Isiolo
Laikipia
-92
Garissa
-100
Narok
-88
Machakos
-71
Kitui
-57
Tana River
-54
Kajiado
-88
Lamu
-100
Change (%)
-100
-99 to -92
-91 to -83
-82 to -71
-70 to -1
Taita Taveta
-71
Kilifi
-83
Kwale
-95
N
0
200
400
Kilometers

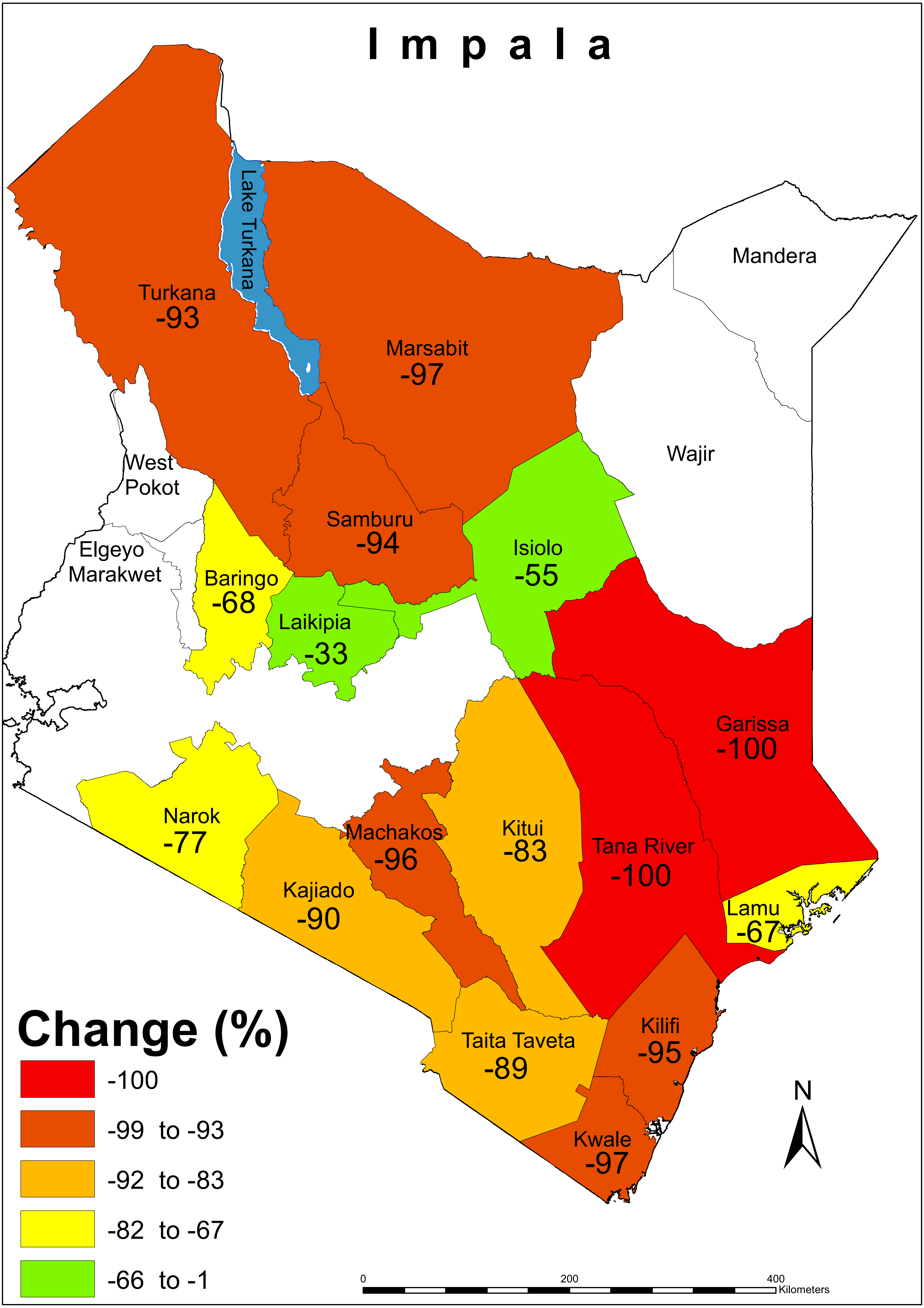
Impala
Lake Turkana
Turkana
-93
Marsabit
-97
Mandera
Wajir
West
Pokot
Samburu
-94
Elgeyo
Marakwet
Baringo
-68
Isiolo
-55
Laikipia
-33
Garissa
-100
Narok
-77
Machakos
-96
Kitui
-83
Tana River
-100
Kajiado
-90
Lamu
-67
Kilifi
-95
Taita Taveta
-89
Kwale
-97
Change (%)
-100
-99 to -93
-92 to -83
-82 to -67
-66 to -1
N
0
200
400
Kilometers

Grevy's Zebra

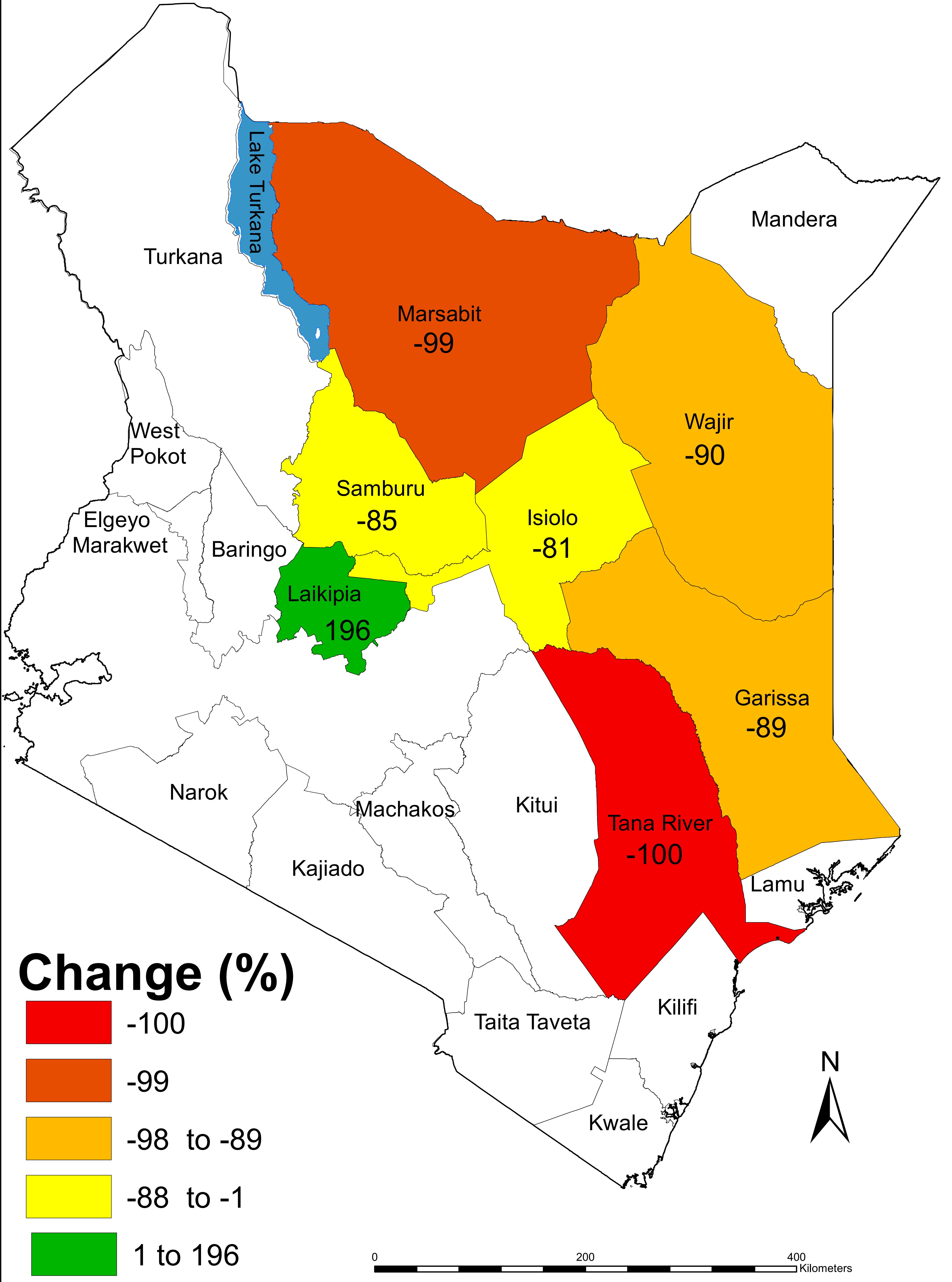

W a t e r b u c k

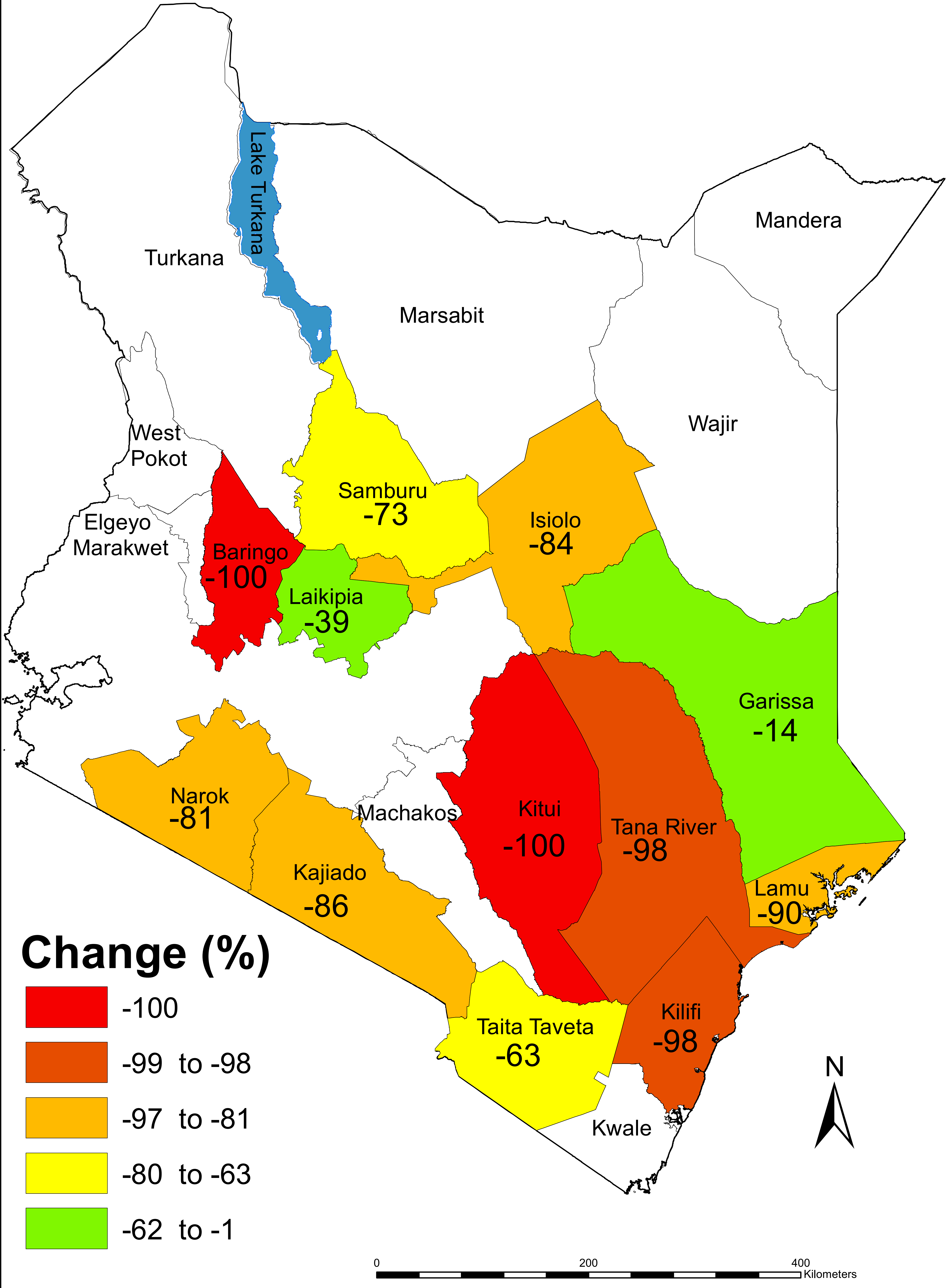
Lake Turkana
Turkana
Marsabit
Mandera
Wajir
West Pokot
Samburu -73
Isiolo -84
Elgeyo Marakwet
Baringo -100
Laikipia -39
Garissa -14
Narok -81
Machakos
Kitui -100
Tana River -98
Kajiado -86
Lamu -90
Kilifi -98
Taita Taveta -63
Kwale
Change (%)
-100
-99 to -98
-97 to -81
-80 to -63
-62 to -1
N
0
200
400
Kilometers
