# Supplementary material for: Extreme Wildlife Declines and Concurrent Increase in Livestock Numbers in Kenya: What Are the Causes?
Source: PLoS One. 2016 Sep 27;11(9):e0163249. doi: 10.1371/journal.pone.0163249 (PMC5039022; doi:10.1371/journal.pone.0163249)
Supplement: S1 File — Separate models were used for wildlife and livestock. The SAS GENSELECT, GLIMMIX and NLIN procedures were used to select and fit models to the covariates most strongly correlated with wildlife population size. (DOCX) [file pone.0163249.s043.docx]

Supplementary Materials SM1.

/*--SAS (Version 9.4, SAS/STAT version 14.1) code used to simultaneously model the population trends for all the wildlife species in each County*/

ods output covparms=covparms parameterestimates=Parmestimates;

**Proc** **glimmix** data=Narok method=RSPL initglm initglm outdesign(names)=XZmat;

class species;

Model count=species species*date /noint dist=negbin link=log ddfm=KR offset=logmean;

Random date /sub=intercept type=pspline knotmethod=equal(**20**);

Random date /sub=species type=pspline knotmethod=equal(**20**);

output out=Pred2 Pred(ilink)=mu lcl(ilink)=lower ucl(ilink)=upper;

nloptions tech=NRRIDG Maxiter=**1000** maxfunc=**5000**;

**run**;

Notes:

1. Species is a categorical or class variable with levels equal to the number of all species, each with sufficiently large counts to model a trend for a given county. For any one County, species is the same as the column labelled species in S4Data. Species models the main effect of species in the model. The “noint option” in the model statement suppresses the general mean so that each species has a separate intercept or average population size.
2. Count is the population size for each species estimated using Jolly’s method 2 and is the same as the column labelled Population estimate with 22 outliers removed in S4Data.
3. .Date is the date of survey as in S4Data.
4. Species*date is the interaction between species, a categorical variable, and date, a continuous variable. Date should have a valid date format. Alternatively. date can be replaced with the running time of surveys.
5. The offset variable, logmean is the same as the logmean column in S4Data.
6. The two random statements do the trend smoothing using penalized cubic basis splines.
7. If the two random statements are replaced with

Random date /sub=intercept type=rsmooth knotmethod=kdtree(bucket=**30**);

Random dtae /sub=Species type=rsmooth knotmethod=kdtree(bucket=**30**);

then the penalized cubic basis spline smoother is replaced with the radial basis smoother. The bucket= option has to be selected appropriately depending on the data set.

1. The spline transformations invoked by the two continuous random statements can be seen in the output data set called XZmat.
2. The other model details can be looked up in SAS user’s guides for the GLIMMIX procedure.

/*--SAS (Version 9.4, SAS/STAT version 14.1) code used to automatically select the supported covariates most strongly correlated with population density of each of the 18 wildlife species */

ods output ParameterEstimates=ParameterEstimates_select_fw SelectedEffects=SelectedEffects_fw SelectionDetails=SelectionDetails_fw SelectionSummary=SelectionSummary_fw SelectionReason=SelectionReason_fw;

**proc** **hpgenselect** data=trends_wildlife4 technique=NRRIDG maxiter=**1000**;

by Species;

model pestimate = Humanden|livestock|perc_protec|Annualrain| maxtemp|mintemp

Humanden*Humanden livestock*livestock perc_protec*perc_protec

annualrain*annualrain maxtemp*maxtemp mintemp*mintemp /dist=nb link=log offset=logarea;

output out=covariate_pred Pred=Pred Lower=Lower Upper=upper;

selection method=forward (choose=AICC) details=all hierarchy=single;

id den_wild ;

**run**;

Notes:

1. Humanden=Human population size per km^2^.
2. Livestock = Total livestock population biomass per km^2^.
3. Annual rainfall = Total annual (Jan-Dec) rainfall in mm.
4. Annual average maximum temperature in °C.
5. Annual average minimum temperature in °C.
6. Perc_Protec=Percentage of area protected in each county.
7. Logarea = Logarithm of the total area of each county in km^2^.
8. | Shorthand notation for interaction between a pair of variables.

/*--SAS (Version 9.4, SAS/STAT version 14.1) code used to relate wildlife population density to total annual rainfall, annual average maximum and minimum temperatures and the percentage area of each county under protection.*/;

ods output covparms=covparms_annualrain tests1=tests1_annualrain_quad fitstatistics=fit_annualrain_quad(where=(descr in ("AICC (smaller is better)")))

parameterestimates=Parameterestimates_rain_quad StandardizedCoefficients=StandardizedCoefficients_rain_quad covb=Cov_annualrain_quad;

**Proc** **glimmix** data=trends_wildlife4 Method=RSPL initglm lognote;

by species;

class County Year;

Model pestimate=annualrain annualrain*annualrain/dist=negbin link=log offset=logarea stdcoef s cl covb htype=**1**;

Random _residual_;

/*Random Year/sub=county type=sp(exp)(time) residual;*/

output out=annualrainred_quad Pred(ilink)=mu LCL(ilink)=lower UCL(Ilink)=Upper ;

nloptions tech=NEWRAP maxiter=**5000** maxfunc=**5000**;

**run**;

Notes

1. Annualrain is the total annual (Jan-Dec) rainfall in mm.
2. Logarea is the logarithm of the total area of each county in km^2^.
3. Random _residual_ fits an overdispersion parameter in addition to the scale parameter of the negative binomial model.
4. /*Random Year/sub=county type=sp(exp)(time) residual;*/
5. The line of code in the preceding note number 4 is intended to fit serial autocorrelation based on spatial generalization of the first-order autoregressive variance-covariance structure. Time is equal to year but is continuous while year is categorical.

/*--SAS (Version 9.4, SAS/STAT version 14.1) code used to relate wildlife population density to human population density or total herbivore biomass. A linear model is obtained by dropping the quadratic term from the model*/

**PROC** **NLIN** data=Trends_wildlife4 NOITPRINT MAXITER=**32000**;

by Species;

parms

a=**0** to **7.0** by **5**

b=**0** to **95.0** by **5**

c=**0** to **7.0** by **5**;

model den_wild=exp(a+b*Humanden+c*(Humanden****2**));

output out=Mav638_Humanden_quad SSE=SSE1 P=Pred L95M=Lower U95M=upper;

ods output ParameterEstimates=ParameterEstimates_Humanden_quad corrb=cov_humanden_quad

EstSummary=EstSummary_Humanden_quad(where=(Label1="Observations Used") rename=(cValue1=n) drop=nValue1);

**RUN**;
