# Supplementary material for: Extreme Wildlife Declines and Concurrent Increase in Livestock Numbers in Kenya: What Are the Causes?
Source: PLoS One. 2016 Sep 27;11(9):e0163249. doi: 10.1371/journal.pone.0163249 (PMC5039022; doi:10.1371/journal.pone.0163249)
Supplement: S2 Table — (DOCX) [file pone.0163249.s045.docx]

**S2 Table.** Wildlife population trends in Kenya reported by earlier studies.

| **Region** | **Period** | **Reported trend** | **Source** |
| --- | --- | --- | --- |
| 18 rangeland Counties of Kenya | 1977-1997 | 40-60% decline in numbers of common wildlife species | [12,13,26] |
| Masai Mara Region | 1958-2010 | 67% average decline in numbers of common wildlife species | [16,17,67,68,70-73] |
| Nakuru Wildlife Conservancy | 1996-2015 | 15 species declined, 29 species increased | [80] |
| Lake Nakuru National Park | 1970-2011 | Decline in warthog and Waterbuck numbers | [18] |
| Nairobi National Park and Athi-Kaputiei Ecosystem | 1949-2011 | Most species declined drastically but a few increased | [19,75] |
| Kajiado County | 1940s-2011 | 67% average decline in numbers of common wildlife species | [19,20,74,75] |
| Kisumu Impala Sanctuary | 1976-2011 | Fluctuating impala population size | [76] |
| Laikipia County | 1985-2005 | Declines in buffalo, hartebeest, waterbuck, eland and Thomson’s gazelle | [77] |
| Ruma National Park | 1976-2008 | Roan population declined from 202 to 43 animals | [75] |
| Amboseli, Tsavo East, Tsavo West and Meru National Parks | 1977-2000 | 41% decline in aggregate number of all the common wildlife species | [21] |
