# Supplementary material for: Extreme Wildlife Declines and Concurrent Increase in Livestock Numbers in Kenya: What Are the Causes?
Source: PLoS One. 2016 Sep 27;11(9):e0163249. doi: 10.1371/journal.pone.0163249 (PMC5039022; doi:10.1371/journal.pone.0163249)
Supplement: S3 Table — (DOCX) [file pone.0163249.s046.docx]

**S3 Table.** Indicators of wildlife population increase and concurrent declines in livestock numbers in Namibia, Zimbabwe, South Africa and Zambia but substantial increase in livestock numbers and concurrent declines in wildlife numbers in Kenya.

| **Country** | **Period** | **Livestock Indicator** | **Wildlife on Private Land Indicator** | **Source** |
| --- | --- | --- | --- | --- |
| Namibia | Early 1970s | 180000 animals | 565000 animals | [138,139] (Also data from WWF-Namibia) |
|  | 2001 | 91800 animals (51% decline) | 1161000 animals |  |
|  | 2009 |  | 1818219 animals |  |
|  | 2012 |  | 2838023 animals (Also big increase in wildlife on Communal Conservancies) |  |
| Zimbabwe | 1992 | 139000t beef |  | [140,141,143] |
|  | 1999 | 67000t beef (48% decline) |  |  |
|  | 1974 |  | 179 cropping permits |  |
|  | 1998 |  | *ca*. 1,000 ranches |  |
|  | 1985 |  | $4 million hunting income |  |
|  | 1998 |  | $24 million hunting income (600% increase relative to 1985). Wildlife decline was reversed/stopped by CAMPFIRE (but data hard to find – refer only to USAID report) |  |
| South Africa | 1964 | 5667000 Goats, 39717000 Sheep  12243000 Cattle |  | [140,144-146] |
|  | 1965 |  | 4 registered fenced farms, 575422 animals |  |
|  | 1974 |  | 3180000 ha |  |
|  | 2007 | 2500000 Goats (56% decline)  28000000 Sheep (30% decline)  8000000 Cattle (35% decline) | 5061 fenced farms, 18591422 animals (+18016000 animals), 20500000 ha |  |
| Zambia | 1997 |  | 30 game ranches (1420 km^2^), 21000 wild animals | [147] |
|  | 2012 |  | 177 game ranches (6000 km^2^), 91000 wild animals.  But serious declines in protected areas and in GMAs |  |
| Botswana | 1999 |  | 17 game ranches | [148,149] |
|  | 2005 |  | 60 game ranches,  Serious declines on communal and state land |  |
| Kenya | 1977-1980 | 6,325,031 Sheep and goats  597,052 Camels  116,487 Donkeys  410,198,5 Cattle | 1,809,605 animals (Buffalo, Eland, Elephant, Giraffe, Grant's gazelle, Gerenuk, Impala, Hartebeest, Lesser kudu, Ostrich, Oryx, Thomson's gazelle, Topi, Waterbuck, Warthog, Wildebeest, Burchell's zebra, Grevy's zebra) | This study |
|  | 2011-2013 | 11,150,690 Sheep and goats (76.3% increase)  675,551 Camels (13.2% increase)  124,274 Donkeys (6.7% increase)  3,068,001 Cattle (25.2% decrease) | 607,233 animals (Buffalo, Eland, Elephant, Giraffe, Grant's gazelle, Gerenuk, Impala, Hartebeest, Lesser kudu, Ostrich, Oryx, Thomson's gazelle, Topi, Waterbuck, Warthog, Wildebeest, Burchell's zebra, Grevy's zebra), 68.1% average decrease |  |

**REFERENCES**

**138**. Barnes J, Jones B (2009) Game ranching in Namibia. In: Suich H, Child B, eds (2009) Evolution and innovation in wildlife conservation: From parks and game ranches to transfrontier conservation areas, p 113-126. London: Earthscan.

**139**. NACSO (2008) Namibia's communal conservancies. A review of progress and challenges in 2007. Windhoek: Nambian Association of CBNRM support organizations.

**140**. Mossman AS, Mossman SL (1976) Wildlife utilization and game ranching: Report on a study of recent progress in this field in southern Africa. Morges, Switzerland: National Union for Conservation of Nature and Natural Resources.

**141**. Booth V (2002) Analysis of wildlife markets (sport hunting and tourism). Harare: WWF Southarn African regional programme office.

**142**. Gambiza J, Nyama C (2006) Country pasture/forage report, Zimbabwe. Rome: FAO.

**143**. Child B, Jones B, Mazambani D, Mlalazi A, Moinuddin H (2003) Final evaluation

report: Zimbabwe Natural Resources Management Program - USAID/Zimbabwe

strategic Objective No. 1. CAMPFIRE: Communal Areas Management Programme for Indigenous Resources. Harare: USAID.

**144**. du Toit JG (2007) Role of the Private Sector in the Wildlife Industry. Pretoria: Wildlife Ranching SA.

**145**. Carruthers J (2008). Wilding the farm or farming the wild? The evolution of scientific game ranching in South Africa from the 1960s to the present. Transactions of the Royal Society of South Africa 63: 160-181.

**146**. Dry G (2011) Commerical wildlife ranching's contribution to a resource efficient, low carbon, pro employment green economy. 7^th^ International Wildlife Ranching Symposium. Kimberly, South Africa.

**147**. Lindsey PA, Barnes J, Nyirenda V, Tambling C, Taylor WA (2012) The Zambian game ranching industry: scale, associated benefits, and limitations affecting its development. Lusaka: A study commissioned by the Wildlife Producers Association of Zambia and funded by Livestock Services Cooperative Society Limited.

**148**. BWPA (2005) The Botswana game ranching handbook. Gabarone: Botswana Wildlife Producers' Association.

**149**. Moswete NN, Dube PO (2011) Wildlife-based tourism and climate: Potential opportunities and challenges for Botswana. University of Botswana: Department of Environmental Sciences. Available online at: [http://www.iipt.org/IIPT%20Book/articles/ updated_articles/](http://www.iipt.org/IIPT%20Book/articles/%20updated_articles/)Naomi%20Moswete.Botswana2.pdf
