# Supplementary material for: Extreme Wildlife Declines and Concurrent Increase in Livestock Numbers in Kenya: What Are the Causes?
Source: PLoS One. 2016 Sep 27;11(9):e0163249. doi: 10.1371/journal.pone.0163249 (PMC5039022; doi:10.1371/journal.pone.0163249)
Supplement: S9 Table — Policy, institutional and market failures that have contributed to catastrophic wildlife population declines in Kenya between 1977 and 2016 are highlighted. (DOCX) [file pone.0163249.s052.docx]

| **Period** | **Policy /Institution** | **Consequences of policy for wildlife conservation, including policy, institutional and market failures** |
| --- | --- | --- |
| Before 1977 | **1) Allowed**: Live capture of wildlife for sale and export; ranching, cropping and culling; tanning, taxidermy and curios; and sport hunting within both long term and short term concession areas and hunting blocks.  **2) Allowed**: compensation for livestock depredation, loss of grazing and crop damage [104,122].  **3) New Institution formed:** The Wildlife Conservation and Management Department (WCMD), formed in 1976 to conserve and manage wildlife | The consumptive industry directly supported landholders and users and provided development funds at the District level [104,122].  **Policy failure**: Over-reliance on Command and Control without the ability to enforce compliance. This is because private land ownership outside protected areas makes command and control ineffective. Providing incentives to landholders outside protected areas to conserve and manage wildlife and their habitats would be more effective [104,122]  **Institutional failure**: Private land owners had no property rights and use rights over wildlife but were free to use the land as they deemed fit, including destroying wildlife habitats through cultivation [104,122]. The ownership and user rights of wildlife were invested entirely in the State. |
| 1977-1992 | **4) Prohibited**: State abruptly banned all hunting in 1977.  **5) Prohibited**: State banned all other consumptive uses of wildlife and use of resources within Protected Areas [104,122]  **6) New institution formed**. The government created the Kenya Wildlife Service (KWS) in 1990 as an autonomous parastatal organization with the mandate to conserve and manage all wildlife [104]. | **Market failure**: Only game viewing allowed from 1978 to 1992. The ban on all consumptive utilization of wildlife severely restricted the opportunities for pastoral landholders to generate revenues from their wildlife resources.  Limited or no financial incentives to landowners to conserve their wildlife resource in effect favoured competing production incentives [104]. Positive net benefits to landowners from wildlife operations are insufficient to guarantee economic incentives to conserve wildlife. Subsidies to agricultural and livestock production reduce the marginal production costs to below social opportunity costs, and hence accelerate the conversion of rangelands to livestock and agricultural production at the expense of conservation objectives and values [104] |
| 1992-2012 | **7) New Institution formed**: KWS established a Community Wildlife Service to encourage neighbouring landholders to form licensed Wildlife Associations and Wildlife Forums to jointly manage their wildlife.  **8) Allowed**: KWS granted wildlife use rights in some areas from 1992 until 2002  9) **Prohibited**: KWS abruptly banns all consumptive uses of wildlife and trades in wildlife products again in 2003.  **10) Prohibited**: KWS abolishes all compensation schemes because they were ineffective and corrupted | KWS reinstated some wildlife related benefits to landowners by permitting some consumptive utilization, limited cropping on game ranches and farms and channelling a proportion of park gate receipts to communities living around the protected areas as Wildlife Development Funds for social investment. KWS licensed some 60 wildlife ranching, cropping and farming operations [104,122].  KWS started reviving and rehabilitating the network of protected areas, improving security and anti-poaching operations.  Lack of benefits from wildlife compounded with the denial of compensation to landholders for the costs of raising state wildlife on their private lands has the perverse impact of creating poverty traps [104].  With little to no benefits from wildlife, wildlife elevates the production costs of livestock and agriculture and the opportunity costs (in terms of foregone benefits of development) of leaving land undeveloped for conservation given the increasing human populations, the price of land, expanding markets and new agricultural technology  KWS acts more as a regulatory and enforcement service than an enabling institution. |
| 2013-2016 | **11)** Wildlife Conservation and Management Act 2013 (**WCM Act 2013**) [53].came into force |  |
|  | **12) WCM Act 2013** [53].is the first to legally recognize and encourage wildlife conservation and management on community and private land. The Act also allows land users to derive benefits from wildlife conservation, regulated and sustainable non-consumptive and limited consumptive utilization of wildlife resources.  The WCM Act 2013 mandates Kenya Wildlife Service (KWS) to conserve and manage all wildlife in Kenya as well as promote, oversee, direct and monitor wildlife conservation on private and communal lands. | This should enable a variety of options for land owners to benefit from wildlife conservation, rather than relying solely on photographic tourism, as is currently the norm, and turn wildlife from a liability to a valued asset in areas endowed with rich wildlife. However, there is a need to manage expectations about the realistic levels of income that everyone can individually hope to derive from the community conservancies.  The ecotourism industry is responding with more diversified tourism products, offering more diverse benefit streams. But nature tourism remains the major source of wildlife revenue and contributor to supporting conservation. Granting land owners’ rights to manage and use wildlife and secure land tenure arrangements, as the Act does, is an important incentive for conservation [122,128]. Regrettably, regulations governing these user rights have yet to be developed and implemented.  Tourism cartels continue to divert most of the wildlife generated revenue away from private landholders, KWS, and County Governments to the service side of the industry. The tourism cartels also maintain barriers that prevent landholders becoming more directly involved in the tourism business. The tourism cartels pass onto the landholders a disproportionate amount of the business risk involved in tourism [104, 122]. There is a need to sideline the tourism cartels and encourage private sector tourism on private land [104]  Investment in conservation is still being hampered by the continuing prohibition of high value activities such as sport hunting, and by over regulation and vacillation [104]. |
|  | **13) WCM Act 2013** [53] devolves wildlife conservation and management rights, opportunities and responsibilities to county governments, land owners and managers of land where wildlife occurs. The Act also recognizes wildlife conservancies and creates mechanisms for setting up rules for regulating the establishment and operation of conservancies with community participation. | Devolution of wildlife conservation is enabling a pluralistic, more inclusive and integrative approaches to conservation that are essential to winning valuable space and place for wildlife and biodiversity conservation in the Kenyan rangelands. About 230 conservancies covering an area of about 43600 km^2^ had been formed by 2015 according to the Kenya Wildlife Conservancies Association, driven largely by the success of the initial conservancies in Masai Mara in Narok County.  The conservancies are quickly emerging as effective vehicles for developing and implementing the pluralistic and locally-adaptive solutions to the regionally varied conservation challenges and contexts. Conservancy ranger networks, for example, have emerged as useful vehicles for combating poachers and cattle rustling and enforcing community rules, among other diverse functions.  But KWS is not adequately funded to support the devolved structures. KWS requires more funding to carry out these tasks.  Moreover, there are absolutely no state funds to support private, community or NGO efforts to protect land and wildlife. County governments created in 2010 also support conservancies, the county structures sometimes make it much harder to implement the Act as local economic imperatives almost always win over wildlife conservation. As well, despite the governance structures being defined in the Act, there is still a general lack of seriousness and commitment to operationalizing these structures. The Act requires effective public participation in conservation through representation in public conservation agencies, community wildlife associations and committees at county and national levels, preparation of management plans and declaration of conservation areas to give effect to devolution of wildlife management and conservation to counties, community and private conservancies. However, public involvement in wildlife conservation is still lacking and there is very little action in this direction. Moreover, there are still very many private initiatives that ignore the Act, like the conservancies, which are not yet managed under the Act, and the worrying privatization of some public game reserves. There is a need for making substantial direct grants to landowners and communities who support wildlife and financially supporting conservation NGOs. |
|  | **14) WCM Act 2013** [53] strengthens and prioritises the protection of endangered species, habitats and ecosystems and provides for a timely review of the listing of species whose numbers have been severely reduced in the Convention on Trade in Endangered Species of Flora and Fauna (CITIES) Red Data Book to enhance their protection status. | The Act requires the identification and publication of a national list of endangered or threatened wildlife habitats and ecosystems, nearly threatened, vulnerable or critically endangered wildlife species from time to time. |
|  | **16) WCM Act 2013** [53] requires the identification and prioritization of the protection and restoration of endangered rangeland ecosystems using multiple instruments, including land eases, conservation easements and negotiated land purchases. | The WCM Act 2013 calls for developing clear policies and legislation on the protection and rehabilitation of degraded wildlife habitats but these have not yet been developed. |
|  | **17) WCM Act 2013** [53] promotes effective deterrence and control of problem animals, effective and timely compensation for human injuries, fatalities or damage to property caused by wildlife to minimize retaliatory killings of problem animals [115]. | The Act is perhaps overly generous in its provisions for compensation as a result of which payments have hardly been made two years later, thus creating huge animosity with communities over compensation. Consequently, perhaps more elephants are being killed in control than by poaching. |
|  | **19) WCM Act 2013** [53] promotes the maintenance of healthy rangelands and wildlife populations by creating a wildlife endowment fund to support the management and restoration of degraded protected areas and conservancies | The actual fund is yet to be created |
|  | **20) WCM Act 2013** [53] promotes landscape-level land use planning and ecosystem management for biodiversity conservation and pastoral livestock production. It also encourages land parcel exchanges, including through purchases and creation of wildlife conservation orders or easements to promote conservation, voluntary land reconsolidation by pastoralists to form conservancies and sanctuaries in the rangelands and payment for ecosystem services. | No such plans have been implemented thus far. |
|  | **18) WCM Act 2013** [53] raises the severity of penalties for wildlife offences upon conviction and promotes robust law enforcement. | This was expected to have two effects - 1) deterrent, and 2) elevate seriousness and raise awareness. However, the new severe penalties are only as effective as their implementation. Although international and domestic wildlife crimes have been taken much more seriously than before, this may have had another unintended effect - more corruption.  The Act pays no attention to new emerging threats such as international wildlife crimes. But, all the domestic efforts will only work if there are diplomatic arrangements on wildlife crimes, migratory species and countries with high demands for wildlife products. |
